# The non-telomeric evolutionary trajectory of TRF2 in zebrafish reveals its specific roles in neurodevelopment and aging

**DOI:** 10.1093/nar/gkac065

**Published:** 2022-02-12

**Authors:** Yilin Ying, Xuefei Hu, Peng Han, Aaron Mendez-Bermudez, Serge Bauwens, Rita Eid, Li Tan, Mélanie Pousse, Marie-Joseph Giraud-Panis, Yiming Lu, Eric Gilson, Jing Ye

**Affiliations:** Department of Geriatrics, Medical center on Aging of Shanghai Ruijin Hospital, Shanghai Jiaotong University school of Medicine; International Laboratory in Hematology and Cancer, Shanghai Jiao Tong University School of Medicine/Ruijin Hospital/CNRS/Inserm/Côte d’Azur University, PR China; Côte d’Azur University, CNRS, INSERM, IRCAN, Faculty of Medicine Nice, France; The State Key Laboratory of Medical Genomics, Pôle Sino-Français de Recherche en Sciences Du Vivant et Génomique, China; Department of Geriatrics, Medical center on Aging of Shanghai Ruijin Hospital, Shanghai Jiaotong University school of Medicine; International Laboratory in Hematology and Cancer, Shanghai Jiao Tong University School of Medicine/Ruijin Hospital/CNRS/Inserm/Côte d’Azur University, PR China; The State Key Laboratory of Medical Genomics, Pôle Sino-Français de Recherche en Sciences Du Vivant et Génomique, China; Department of Geriatrics, Medical center on Aging of Shanghai Ruijin Hospital, Shanghai Jiaotong University school of Medicine; International Laboratory in Hematology and Cancer, Shanghai Jiao Tong University School of Medicine/Ruijin Hospital/CNRS/Inserm/Côte d’Azur University, PR China; The State Key Laboratory of Medical Genomics, Pôle Sino-Français de Recherche en Sciences Du Vivant et Génomique, China; Department of Geriatrics, Medical center on Aging of Shanghai Ruijin Hospital, Shanghai Jiaotong University school of Medicine; International Laboratory in Hematology and Cancer, Shanghai Jiao Tong University School of Medicine/Ruijin Hospital/CNRS/Inserm/Côte d’Azur University, PR China; Côte d’Azur University, CNRS, INSERM, IRCAN, Faculty of Medicine Nice, France; Côte d’Azur University, CNRS, INSERM, IRCAN, Faculty of Medicine Nice, France; Côte d’Azur University, CNRS, INSERM, IRCAN, Faculty of Medicine Nice, France; Shanghai Center for Plant Stress Center, CAS Center for Excellence in Molecular Plant Sciences, PR China; Côte d’Azur University, CNRS, INSERM, IRCAN, Faculty of Medicine Nice, France; Côte d’Azur University, CNRS, INSERM, IRCAN, Faculty of Medicine Nice, France; Department of Geriatrics, Medical center on Aging of Shanghai Ruijin Hospital, Shanghai Jiaotong University school of Medicine; International Laboratory in Hematology and Cancer, Shanghai Jiao Tong University School of Medicine/Ruijin Hospital/CNRS/Inserm/Côte d’Azur University, PR China; The State Key Laboratory of Medical Genomics, Pôle Sino-Français de Recherche en Sciences Du Vivant et Génomique, China; Department of Geriatrics, Medical center on Aging of Shanghai Ruijin Hospital, Shanghai Jiaotong University school of Medicine; International Laboratory in Hematology and Cancer, Shanghai Jiao Tong University School of Medicine/Ruijin Hospital/CNRS/Inserm/Côte d’Azur University, PR China; Côte d’Azur University, CNRS, INSERM, IRCAN, Faculty of Medicine Nice, France; Department of Medical Genetics, CHU; Nice, FHU Oncoage, France; Department of Geriatrics, Medical center on Aging of Shanghai Ruijin Hospital, Shanghai Jiaotong University school of Medicine; International Laboratory in Hematology and Cancer, Shanghai Jiao Tong University School of Medicine/Ruijin Hospital/CNRS/Inserm/Côte d’Azur University, PR China; The State Key Laboratory of Medical Genomics, Pôle Sino-Français de Recherche en Sciences Du Vivant et Génomique, China

## Abstract

The shelterin protein complex is required for telomere protection in various eukaryotic organisms. In mammals, the shelterin subunit TRF2 is specialized in preventing ATM activation at telomeres and chromosome end fusion in somatic cells. Here, we demonstrate that the zebrafish ortholog of TRF2 (encoded by the *terfa* gene) is protecting against unwanted ATM activation genome-wide. The *terfa*-compromised fish develop a prominent and specific embryonic neurodevelopmental failure. The heterozygous fish survive to adulthood but exhibit a premature aging phenotype. The recovery from embryonic neurodevelopmental failure requires both ATM inhibition and transcriptional complementation of neural genes. Furthermore, restoring the expression of TRF2 in glial cells rescues the embryonic neurodevelopment phenotype. These results indicate that the shelterin subunit TRF2 evolved in zebrafish as a general factor of genome maintenance and transcriptional regulation that is required for proper neurodevelopment and normal aging. These findings uncover how TRF2 links development to aging by separate functions in gene expression regulation and genome stability control.

## INTRODUCTION

Linearization of the genome in the eukaryotic lineage made hiding chromosomes ends a necessity to avoid their illicit repair and the resulting chromosomal fusions and genomic instability (1). Moreover, cells had also to be able to compensate for the inevitable loss of terminal sequence due to incomplete replication of ends (2). Facing these problems, eukaryotic ancestors devised common answers: a protein complex capping chromosome termini. In many organisms, this protective cap takes the form of a complex called Shelterin (3,4). On its simplest configuration, the Shelterin complex of ciliates such as *Oxytrichia nova* only contains a heterodimeric protein that protects the single stranded part of telomeres. Humans have evolved a more elaborate system containing 6 proteins (TRF1, TRF2, RAP1, TIN2, TPP1 and POT1) but the ultimate goal remains the same: inhibiting activation of the DNA damage response (DDR) and double strand break (DSB) repair as well as recruiting/regulating the telomerase, an enzyme that elongates telomeres. Despite overall differences in composition, common structural themes can be found: Myb/SANT (Telobox) domains for specific binding to double stranded telomeric DNA repeats (5), oligonucleotide/oligosaccharide binding (OB) folds for binding the single stranded telomeric DNA repeats and TRFH domains for protein and additional DNA interactions (6). Protecting telomeres termini from DNA damage signaling and repair is a major role of TRF2 (7). Indeed, depletion of TRF2 in mouse or human cells causes telomeric recruitment of DDR proteins such as γH2AX and 53BP1, forming foci called TIFs (Telomere dysfunction induced foci), leading to activation of ATM and illicit repair mainly by classical-NHEJ therefore fusing telomeres together. This is achieved by a plethora of telomere protective mechanisms including ATM signaling inhibition (8–10) t-loop formation (11–13), replication elongation facilitation (14) and replication-dependent end-processing (15–17).

Previous studies using conditional mouse models revealed a pivotal role of TRF2 in aging. Whole-body *terf2* deletion in mice led to pro-oxidative and premature-aging vascular phenotypes (18). The deletion of *terf2* in lung leads to type 2 alveolar epithelial cell senescence and inflammation (19). In skeletal muscle cells, the absence of TRF2 did not lead to obvious telomere damage but instead to mitochondrial dysfunction and oxidative stress, indicating that TRF2 may have telomere-independent functions in longevity pathways (20). Indeed, TRF2 can also bind to non-telomeric DNA, where it serves various functions involved in DNA repair, heterochromatin replication and transcriptional regulation (21). Overall, our understanding of the respective contribution of the telomeric and extra-telomeric properties of TRF2 in development, somatic maintenance and aging is still limited. We address this question here by a comparative approach aimed at studying the functions of the zebrafish TRF2 ortholog in telomere protection, development and aging. Strikingly, we unveiled that the zebrafish TRF2 (zfTRF2) has no specific role in telomere protection and is required for a proper neurodevelopment through additive functions in global genome maintenance and transcriptional regulation. We discuss these findings in light of the evolution of the shelterin factors balancing between telomere-specialized and genome-wide functions.

## MATERIALS AND METHODS

### Zebrafish maintenance

These experiments were performed in the Zebrafish Tübingen strain. Zebrafish were raised and maintained under standard conditions as described previously (22). All experiments were performed in accordance with the guidelines of the Animal Care and Use Committee of Shanghai Jiao Tong University. The buffer for embryo maintains were freshly prepared with PBS (MPH 1.08 mg/kg, valproate 300 mg/kg, aripiprazole 0.6 mg/kg, ziprasidone 1.6 mg/kg). The zebrafish were feed per day, and renew the tank every week.

### Cell culture and treatment

293T cell were grown in glutamax-containing DMEM (#11995-065; Gibco) supplemented with 10% FBS (#S11550-500; Biosera) at 37°C and 5% CO_2_. ZF4 cell line (zebrafish embryonic cell) and ZFL cell line (zebrafish liver cell) were grown in glutamax-containing DMEM/F12(1:1) (#11330-032) supplemented with 10% FBS at 28°C and 5% CO_2_.

### Plasmid construction

The *terfa* sequence was synthesis (in Genescript) according to the mRNA sequence in NCBI database, together with tag-Myc on the N-terminal. The overexpression plasmid pWPIR was a gift from Eric Gilson's lab. The neural promotor sequence was synthesis (in Genescript) according to the references (23–25), together with mRNA sequence of terfa and mCherry. The mRNA of *terfa* and mCherry was joined by P2A oligopeptide. The frame of neural promotor meditate plasmid pTol2-mCherry was a gift from Gang Peng's lab.

### Plasmid transductions and microinjection

293T cells were transfected with 4.3 ug pCMV-dR8.91, 1.4 ug pCMV-VSVG and 4.3 ug pWPIR plasmid using ProFection^®^ Mammalian Transfection System (Promega E1200). Supernatants were collected after 48 and 72 h and filtered for cell transduction. The transduction efficiency was determined for the pWPIR vectors by qPCR and western-blot (see in Supplementary Figure S2) analysis of cells 3 days after infection. The plasmid microinjection was done in 1-cell stage embryo. 50 pg plasmid was injected per embryo together with mRNA of tol2-transduction enzyme (100 pg per embryo) which transcript *in vitro* using SP6 mRNA transcriptional kit (Themo). Each step was confirmed by agarose gel electrophoresis. The efficiency of Tol2 transduction was testified by observe mCherry positive embryos every 24 h after injection, the samples were collected at 72hpf for experiments.

### RT-qPCR

Total RNA from cell (ZF4, ZFL, 293T) or zebrafish (embryo and tissues) was extracted using TRIzol reagent (Ambion) and reverse transcribed using the PrimeScript RT reagent kit (TaKaRa) according to the manufacturers’ protocols. RT-qPCR analysis was performed using the QuantStudio Dx Real-Time PCR Instrument (ABI) with SuperRealPreMix Plus (TianGen). The primers used in this study are shown in Supplemental table of primer.

### Western blot

Protein lysates of cell (ZF4, 293T) or zebrafish (embryo and tissues) were prepared by ice-cold RIPA complemented with DMSF (Beyotime Biotechnology), phosphatase and protease inhibitors (Roche). Protein concentration was quantified by PierceTM BCA Protein Assay Kit (Thermo). Protein was loaded onto SurePAGE 4–20% Bis–Tris gradient gels (Genscript). Sample was transferred onto Immobilon-P PVDF 0.45 μm membranes (Millipore) using Trans-Blot Turbo Transfer System (BioRad). Membranes were blocked in 5% NON-Fat Powdered Milk (Sangon) with TBST (0.1% Tween-20 in TBS) for 1 h at room temperature (RT). Hybridization with primary antibodies (Supplemental table of antibody) was performed at 4ºC overnight followed by TBST washing three times. Hybridization with corresponding second antibodies was performed at 37ºC for 2 h followed by TBST washing three times. Imaging was detected using AI600 (GE) and processed using ImageJ software.

### Generation of terfa-knockout zebrafish

terfa-knockout zebrafish were generated using the CRISPR-CAS9 system, in which a guide RNA targeting exon 2 of terfa (sgRNA: 5′-GTGTGGTGGTCAGGCCGG-3′) was designed using ZiFiT Targeter software (http://zifit.partners.org/ZiFiT). The guide RNA was synthesized by cloning annealed oligonucleotides into the sgRNA vector as described previously (26). The founder embryos (F0 generation) were raised to 3 months old and outcrossed with WT zebrafish to obtain potential F1 indel mutations. PCR amplification and sequencing were performed on the genomic DNA isolated from the tails of F1 zebrafish to identify terfa mutants (primers for genotyping: fwd 5′-TTAACCCGCGGTTATCTTCAG-3′; rev 5′-CGTCTCCGACATTCACTCAC-3′).

### RNA-seq analysis

Sequencing was performed by BGI. Illumina HiSeq Xten 2 × 150-bp paired-end was used for sequencing. More than 40 million raw reads were obtained for each sample. The RNA-seq experiment was performed in two biological replicates for vehicle groups, three biological replicates for treated groups. Raw RNA-seq reads were filtrated by SOAPnkue with default setting to remove adapters and low-quality reads and the remaining reads were trimmed into 100 bp for downstream analysis. Reads were aligned using STAR v2.6.1d on zebrafish zv10 release with parameter–quantMode GeneCounts. Differential expression analysis was performed using DESeq2 R package.

### Immunofluorescence of embryo slices

Embryo sections were fixed in 4% PFA for 30 min and then permeabilized and blocked with 0.5% Triton X-100, 2% FBS in 1× PBS for 1 h at room temperature. Hybridization with primary antibodies was performed at 4°C overnight followed by washing three times with 0.1% Tween-20 in PBS. Hybridization with corresponding secondary antibodies (Invitrogen) was performed at 37°C for at least 2 h. Finally, the brain slices were incubated with 1× DAPI for 10 min at room temperature. Imaging was performed using a confocal laser scanning microscope (SP8; Leica). The primary antibodies used in this study are shown in Supplemental table of antibody.

### PNA-FISH

Cells were grown onto glass coverslips and fixed for 15 min with 3.7% formaldehyde. Cells were then permeabilized with 0.5% Triton X-100 for 8min and dehydrated in increasing concentration of ethanol for 5 min (50%, 75%, 100%). Hybridization of PNA probes was performed for at least 2 h at RT after 3 min denaturation in 70% formamide, 10 mM Tris pH7.2 and 1% blocking solution (Roche) at 85°C. After that, the cells were washed in a 70% formamide, 10 mM Tris pH 7.2 solution for 30 min, followed by a solution containing 150 mM NaCl and 50 mM Tris pH 7.5 for 15 min. Next, the cells were incubated with blocking buffer (1% Triton X-100, 1% BSA and 5% serum) and incubated overnight at 4°C with the desired antibody. Cells were then washed with 1× PBS and incubated for 2 h with the corresponding secondary antibody and 10 min with DAPI. Finally, the cells were preserved in a mounting solution (Vector).

### Metaphase analysis

Embryo were arrested at 10 h post-fertilization pf after being treated with colchicine (final concentration 0.4 mg/ml) for 8 h. Embryos were dissociated into single cells, then incubated with 1.1% sodium citrate for 15 min at 28.5°C and followed by methanol/glacial acetic acid (3:1) fixation. Telomeres were visualized with PNA probes hybridized for 2 h in 70% formamide, 10 mM Tris pH 7.2 and 1% blocking solution (Roche) buffer. Images were obtained with fluorescence microscope (ZEISS).

### Whole-mount in situ hybridization

The full-length cDNA of terfa, neurog1 and c-myb was cloned into the pCS2 vector. The EcoRI and XhoI enzymatic sites were incorporated into the end of cDNA sequence to facilitate the directional cloning. The Digoxigenin labeled antisense mRNA probes were transcribed with T3 polymerase from EcoRI linearized plasmid according to the manufacturer's instructions (Roche). The embryos were collected in 24, 48 and 72hpf and fixed with 4% PFA overnight at 4°C. Dehydrate and rehydrate 10 min with Methanal (30%, 70%, 100%). Fix again with 4% PFA 30 min at 4°C. Digest the embryo with proteinase K according to the time of hours post fertilized (24 h: 10 ug/ml; 5 min 48 h: 20 ug/ml; 15 min 72 h: 50 ug/ml; 20 min). Fix again with 4% PFA 2 h at 4°C. Pre-heat the embryos at 68°C.in Hyb buffer (50% formamide, 5× SSC, 0.1% Tween-20, 5 mg/ml yeast RNA, 50 ug/ml heparin) for 30 min. Then add the probe to the Hyb buffer at the concentration at least 50 ng/ml and hybridize at 68°C overnight (gently rock). Wash the embryo with pre-heat SSCT (1× SSC, 0.1% Tween-20) at 68°C for 15 min three times. Then wash the embryo with MABT (100 mM maleic acid, 150 mM NaCl, 0.1% Tween-20, pH 7.5) at RT for 5 min three times. Blocking (MABT, 2% BSA, 1% FBS) the embryos for 1 h at RT, and add anti-Dig antibody (1:5000) gently rock at 4°C overnight. Staining the embryo follow the protocol (Vector, BCIP/NBP kit) and observe the embryo every 10 min until satisfied. The primers for cDNA cloning were used:

Terfa F:ATGAGCGACAAACCCTGCGAA R:GACCATCTTGAGCTTGACCAT;
Neurog1 F:CCCACCAATAAGGTTATCAA R:GCAGACTGTCATTAAGGCAAA;
C-myb F:GGGTTGGACCATTGGAAGAA R:TGTAAAGGCGAGGGTTGATG.

### Whole-mount light sheet fluorescence microscopy and image process

The transgenic zebrafish Tg(huc:GFP) were across with terfa-mutated fish to get terfa+/−Tg(huc:GFP) and terfa−/−Tg(huc:GFP) for the light sheet scan. The embryos were collected at 72hpf and anesthetized with Tricaine (1×, Sigma-Aldrich). Each embryo was transfer into 1.5% low melting agarose with Tricaine while it remains liquid but no more than 35°C. Every embryo one by one was replaced vertically in a transparent tube for light sheet scan and wait 5min for the agarose to solid. The images were taken in ZEISS Z.1 and results were represented in 3D reconstruction using Imaris software (Imaris 8.1). The Tg(huc:GFP) zebrafish was a gift from Du Jiulin's lab.

### Cellular apoptosis assay

The section was fixed in 4% PFA for 20 min, blocking in 3% BSA for 30min. The apoptosis assay was performed with in Situ Cell Death Detection Kit, TMR red (Roche). The assays were performed following the manufacturer's instruction. Then incubate with DAPI for 10 min. The images were taken by Leica SP8 confocal laser scanning microscope.

### Flow cytometry

The embryos for cytometry were collected at 3dpf. They were anesthetized with Tricaine (1×, Sigma-Aldrich) and the brain was selected and digested into single cell by incubating with 0.5% trypsin for 30 min at 30°C. Stop reaction with DMEM media and gently rock 30min at RT till the embryos separate in cells. After washed with PBS 3 times, the embryos of Tg(huc:GFP) and Tg(gfap:GFP) cross with terfa-compromised fish can be cytometry directly. While the embryos for stem cell test, these cells fixed in 0.1% formaldehyde 10 min at RT. Washed with ice-cold PBS 10min 3times, incubate with 0.5% Triton-100 for 10 min. Block with 1% BSA for 30min on ice and incubate with sox2 antibody for 2hr in desired antibody buffer (3% BSA, 0.1% Tween20, antibody 1:100). After washed with ice-cold PBS, fix again with 0.1% formaldehyde 10min at RT. Then incubate with secondary antibody together with blank control (no antibody) and compensation control (no desired antibody). Wash 3 times with PBS with 1% BSA for 10 min and cytometry. The target cell was chosen by comparing the inflorescence signal with blank.

### Whole-mount acridine orange staining

The embryos were collected separately at 4 point (24/48/72/96hpf). They were anesthetized with Tricaine (1×, Sigma-Aldrich). Incubated in a solution of 3 ug/ml acridine orange (Sigma-Aldrich) in dark. Washed 10 min for 4 times and embedded in 1% low melting agarose in with Tricaine. Images were taken with Zeiss A2 microscope.

### Senescence-associated-β-galactosidase (SA-β-gal) assay

SA-β-gal stain was performed using Senescence β-galactosidase Staining Kit (Cell signaling). Zebrafish slices were fixed in 4% PFA for 30 min and washed with PBS 10 min for 3 times. The whole-mount embryos were collected and fixed in 4% PFA at 4°C overnight. Dehydrate and rehydrate 10 min with Methanal (30%, 70%, 100%), then incubate with proteinase K (as described in RNA-FISH). The assays were performed following the manufacturer's instruction. Images were taken with Zeiss A2 microscope.

### EMSA

EMSA experiments were performed in binding buffer 20 mM Tris pH 8, 150 mM KCl, 0,1 mM EDTA, 1 mM DTT, 500 μg/ml acetylated BSA and 5% glycerol. Proteins and DNA were incubated at room temperature for 15 min and loaded under 50 V on a 5% acrylamide: bisacrylamide (19:1) gel containing 0,5× TBE buffer, after adding 2 μl of a 15% ficoll solution. Migration was performed at 120 V for 2 h at room temperature. Gels were dried and exposed on a phosphorimager screen overnight before analysis using a Phosphorimager Typhoon/FLA 9000. The bottom strand oligonucleotides (show below) were purchased purified from Eurogentec. 5’ labeled using T4 Polynucleotide Kinase (Promega) and hybridized with their corresponding top strand in 20 mM Tris pH 8 50 mM NaCl by heating at 85°C in a 5 l water-bath and slow cooling overnight. Protein expressing vectors pET14b containing the coding sequences of both proteins cloned between the NdeI and BamHI sites were purchased from Genscript. For zfTRF2TB the coding sequence was subcloned into pTrcHisB using the NheI and BamHI sites. Proteins were expressed in NiCo21 (DE3) and NEB5a (New England Biolabs) for zfTRF1TB and zfTRF2TB respectively after induction by 0.1 mM IPTG for 16 h at 25°C. Proteins were purified using a Nickel containing column (HisTrap FF crude, Cytiva) and quantified by Bradford assay.

dsT4 top strand: ATCGTCCTAGCAAGGTTAGGGTTAGGGTTAGGGTTAGGGGGCTGCTACCGGCAC
dsT4 bottom strand: GTGCCGGTAGCAGCCCCCTAACCCTAACCCTAACCCTAACCTTGCTAGGACGAT
NSP54 top strand: GGAATCATCGTCCTAGCAAGGTATTGGTGGAGGGGCTGCTACCGGCACTGCGTT
NSP54 bottom strand: AACGCAGTGCCGGTAGCAGCCCCTCCACCAATACCTTGCTAGGACGATGATTCC
zfTERF1TB: MGSSHHHHHHSSGLVPRGSHMHSRKKWTDVEDKKLKAGVKKHGVGKWSKILNDFDFDNRTTVNLKDRWRVLKKQNLVS
zfTERFATB: MGGSHHHHHHGMASVPRGSHMKKYTRKMWSVQESEWLKQGVVRYGVGHWERIRSSFPFAGRTAVNLKDRWRTMVKLKMV

### CT scan and ultrasonic cardiogram

Both CT scanning and ultrasonic cardiogram of zebrafish 6mpf were done in X-med platform of Shanghai Jiaotong University. Adult fish were collected at 6mpf and anesthetized with Tricaine (1×, Sigma-Aldrich) before the scan. They were wrapped with wet cloth and each scan was no more than 8min to make sure the survival of fish. For CT scan was done in Siemens Inveon and UCG in Vevo2100.

## RESULTS

### zfTRF2 downregulation leads to genome-wide DNA damage response with no telomere-specificity

**Figure 1.**
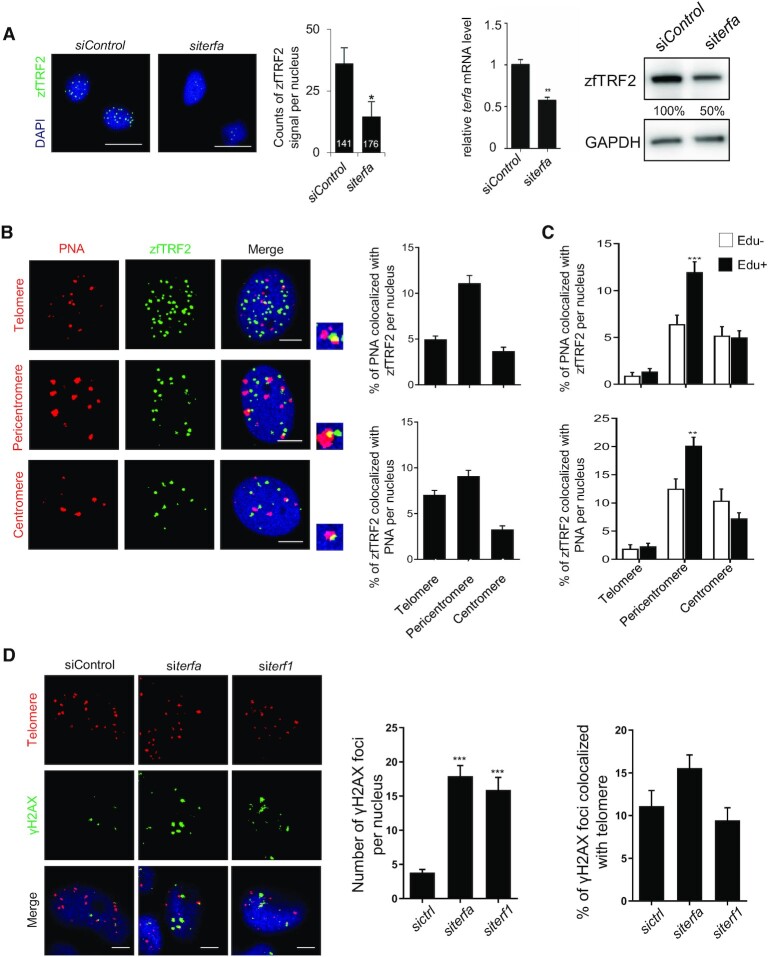
zfTRF2 downregulation leads to genome-wide DNA damage response with no telomere-specificity. (**A**) Representative image and quantification of immunofluorescence (left) and immunoblot (right) of zfTRF2 signal to identify the affinity of the purified anti-zfTRF2 antibodies for zfTRF2 in ZF4 cells following *TERFA* downregulation by siRNA. The number inside the column of the graph indicates the total number of nuclei was counted in three biological replicates. The knockdown efficiency of siRNA was validated by RT-qPCR. Scale bars, 20 μm. (**B**) Representative images and quantification of immunofluorescence of PNA probe(red) for telomeres (top), pericentromeres (middle) and centromeres (bottom) colocalizing with zfTRF2 (green) in ZF4 fish cells. (**C**) The percentage of the signals of PNA probes colocalized with zfTRF2 and percentage of zfTRF2 signals colocalized with PNA were shown in ZF4 cells classified by Edu + and Edu- with immunofluorescence assay. (**D**) Representative images and signal quantification of global and telomere-associated γH2AX foci in ZF4 fish cells upon *terf1* and *terfa* downregulation. Scale bars, 7 μm. Data are shown as the mean ± standard error of the mean (SEM) of at least three biological replicates. For immunofluorescence and confocal section images, at least 30 nuclei were taken and counted in each sample for each biological replicate. Statistical analyses were performed using unpaired two-sided *t* tests (*P < 0.05, **P < 0.01, ***P < 0.001).

In order to investigate the role of TRF2 in zebrafish (zfTRF2 encoded by the *terfa* gene), we use two types of zebrafish cell lines (embryonic ZF4 cells and the liver ZFL cells), zfTRF2 staining by home-made antibodies raised against purified full length zfTRF2 revealed discrete zfTRF2 foci dispersed throughout the nucleus (Figure 1A, Supplementary Figure S1A). Only <10% of them colocalized with telomeres as visualized using a telomere-specific peptide nucleic acid (PNA) probe and concomitantly <10% of telomeres colocalized with zfTRF2 (Figure 1B, Supplementary Figure S1B). We also monitored the association of zfTRF2 to two other highly repeated regions: pericentromeres and centromeres. Roughly 10% (ZF4 cells) and 30% (ZFL cells) of pericentromeric foci as visualized with a PNA probe recognizing SAT-1 satellite repeats were associated with zfTRF2 while <5% of centromeres colocalized with zfTRF2 (Figure 1B, Supplementary Figure S1B). Reminiscent to human cells in which TRF2 binds to pericentromeric heterochromatin during S-phase (27), the association of zfTRF2 increased in dividing cells at pericentromeres but at neither telomeres nor centromeres (Figure 1C, Supplementary Figure S1C−E). Thus, zfTRF2 does not appear to be a specialized telomere binding factor. The fact that a large number of zfTRF2 foci do not colocalize with telomere, pericentromere or centromere can be interpreted by additional zfTRF2 associations to short patches of these repetitive DNA regions that are below our PNA detection, other types of genomic loci or still undefined nuclear substructures. Overall, we conclude that zfTRF2 binds to different types of nuclear structures: a fraction is associated to three major repetitive compartments of the zebrafish genome: telomere, centromere and pericentromere; the localization of the other fraction remains to be determined.

The downregulation by ∼50% of the expression of *terf1* and *terfa* by RNA interference in ZF4 cells was enough to trigger a marked increase in DNA damage response (DDR) throughout the nucleus but not specifically at telomeres (Figure 1A and D, Supplementary Figures S1A and S1F). This indicates that zfTRF1 and zfTRF2 play a genome-wide role in protecting the zebrafish genome against DNA damage response.

### The ability of zfTRF2 to bind telomeres is preserved in human cells

**Figure 2.**
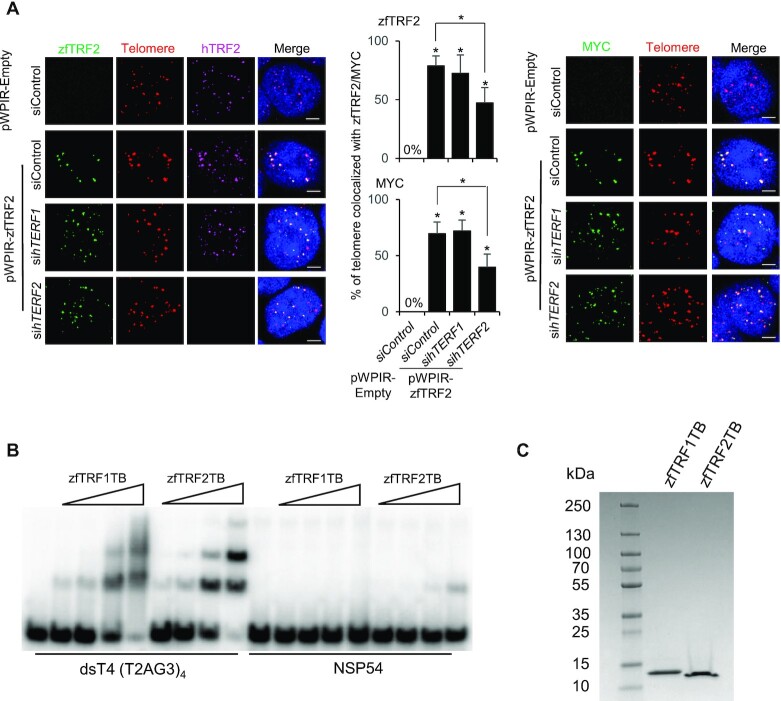
The ability of zfTRF2 binding to telomeres is preserved in human cells. (**A**) Representative confocal section images (left) and signal quantification (right) from a PNA-FISH assay of human 293T cells. The percentages of telomeres (red) that colocalized with zfTRF2 (green, left) or MYC (green, right) in transduced 293T cells overexpressing exogenous zfTRF2-MYC are shown. Human TRF2 (hTRF2) and hTRF1 were knocked down by siRNA for 72 h (scale bars, 7 μm). (**B**) EMSA showing the binding of the Telobox from zebrafish TRF1 (zfTRF1TB) and of the Telobox from zebrafish TRF2 (zfTRF2TB) on a four telomeric repeats containing DNA (dsT4) and a non-specific DNA of the same size (NSP54). Final concentration of the DNA was 5 nM and concentrations of proteins were: 10 nM, 30 nM, 100 nM and 300 nM. (**C**) Coomassie stained SDS PAGE where 5 μg of each protein was loaded. All data are shown as the mean ± SEM of three biological replicates. For immunofluorescence and confocal images, at least 30 nuclei were taken and counted in each sample for each biological replicate.Statistical analyses were performed using unpaired two-sided *t* tests (*P < 0.05, **P < 0.01, ***P < 0.001). Asterisks directly above columns indicate a significant difference between the indicated treatment and the control group (left). Asterisks above two columns indicate a significant difference between the two columns.

Then, we asked whether zfTRF2 maintained an intrinsic property to bind to telomeres in a heterologous cellular system. We expressed a Myc-tagged version of zfTRF2 in human 293T cells and monitored its colocalization with human telomeres. In contrast to observations in zebrafish cells, zfTRF2 predominantly associated with telomeres in 293T cells, as visualized using both anti-zfTRF2 and anti-Myc antibodies (Figure 2A, Supplementary Figure S2A); zfTRF2 colocalized with >70% of the human telomeres. In order to test whether this telomere association resulted from a dimerization between hTRF2 and zfTRF2, we downregulated hTERF2 expression by siRNA (Supplementary Figure S2B). Upon a marked decrease in hTRF2 expression, rendering it undetectable by immunofluorescence, the association of zfTRF2 with telomeres was only partially reduced (Figure 2A, Supplementary Figure S2B), suggesting that the ability of zfTRF2 to dimerize with hTRF2 cannot fully explain its binding to human telomeres. In further agreement with a specific interaction zfTRF2 with telomeric DNA, an electrophoretic mobility shift assay (EMSA) show that purified telobox domain of zfTRF2 specifically binds a telomeric DNA probe (Figure 2B, C). Noteworthy, a parallel experiment with purified telobox of zfTRF1 also exhibits a specific telomeric DNA binding but with a smeary migration of the complexes suggesting an unstable interaction (Figure 2B). This is in agreement with a recent publication showing that zfTRF1 binds telomeric DNA when expressed in mouse cells (28). Overall, we conclude that zfTRF2 in zebrafish cells maintain its intrinsic capacity to bind telomeres. Its partial association to telomeres in zebrafish cells suggests that it is in competition with other telomere-binding factors to specifically bind chromosome ends (29).

### Zebrafish lacking zfTRF2 exhibit premature aging

**Figure 3.**
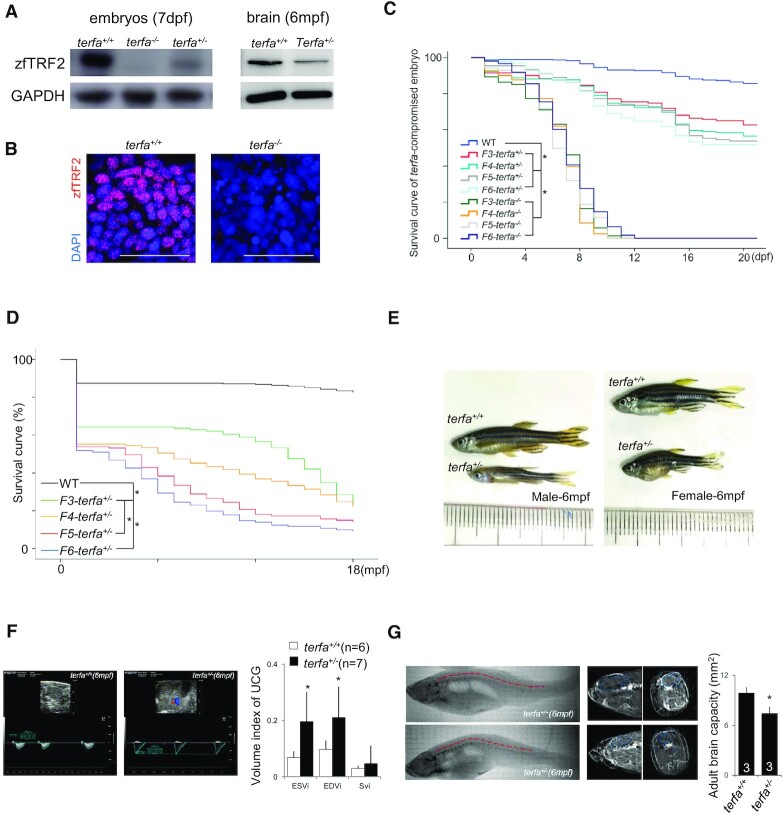
Zebrafish lacking zfTRF2 exhibit premature aging. (**A**) Western blot analysis of zfTRF2 expression in *terfa*-compromised embryos at 7 dpf (left) and fish brains at 6 months post-fertilization (mpf) (right). (**B**) Representative confocal images of zfTRF2 expression in *terfa^+/+^* and *terfa*^−^*^/^*^−^ zebrafish brain 1dpf (scale bar, 20 μm). (C, D) Survival curves of terfa-compromised (*terfa^+/^*^−^ and *terfa*^−^*^/^*^−^) zebrafish during larvae stage (**C**, 0–21dpf) and adult stage (**D**, 0–18mpf) from generations F3 to F6. (**E**) Representative photographs of adult *terfa^+/+^* and *terfa^+/^*^−^ fish at 6 mpf. Left, male; right, female. (**F**) Representative ultrasonic cardiogram (UCG) images (left) and measurements (right) of *terfa*^+/+^ and *terfa*^+/−^ zebrafish at 6 mpf. Volume indices from the UCGs are shown, including the end systolic volume index (ESVi), end diastolic volume index (EDVi), and systolic volume index (SVi). (**G**) Representative computed tomography (CT) images (left) and quantification (right) of zebrafish at 6 mpf. The images show 3D reconstructions of whole fish bodies developed using the CT images. The middle column show extracted two-dimensional (2D) images of the sagittal and coronal sections of the brain collected via CT. The graph (right) shows brain capacity following 3D reconstruction of the brain via CT. Data are shown as the mean ± SEM of three biological replicates. The number inside or below each column indicates the number of embryos detected for each condition. Statistical analyses were performed using unpaired two-sided *t* tests, and survival curves were plotted using a log-rank test (*P < 0.05).

To explore the role of zfTRF2 *in vivo*, we generated a *terfa*-compromised zebrafish model by introducing a frameshift mutation into exon 2 at the center of the TRFH domain (Supplementary Figure S3A). This led to roughly 90% of *terfa* mRNA inhibition probably due nonsense-mediated mRNA decay (Supplementary Figure S3B), which consequently abolished zfTRF2 expression in *terfa*^−/−^ fish and lead to zfTRF2 half expression in *terfa*^+/−^ fish as revealed in embryos by Western blotting and immunofluorescence and in adult heterozygous fish by Western blotting of brain tissues (Figure 3A and B, Supplementary Figure S3C). Homozygous (*terfa*^−/−^) and heterozygous (*terfa*^+/−^) fish, collectively named *terfa*-compromised fish, exhibited different levels of detrimental phenotypes; most homozygous fish died at the larval stage (Figure 3C) and only approximately 50% of heterozygous fish continued to develop to the adult stage (Figure 3D). The adult heterozygous fish exhibited a premature-aging phenotype consisting of spinal curvature (Figure 3E), depigmentation (Supplementary Figure S3D), cardiac dysfunction (Figure 3F) and a marked reduction in brain development (Figure 3G). The fact that the introduced mutation in exon2 leads to a full inhibition of *terfa* expression (Figure 3A, B, Supplementary Figure S3B, C), renders unlikely that the phenotype of the heterozygous fish resulted from a dominant negative effect of an expressed mutated form.

### Zebrafish lacking zfTRF2 exhibit a specific neurodevelopmental failure

**Figure 4.**
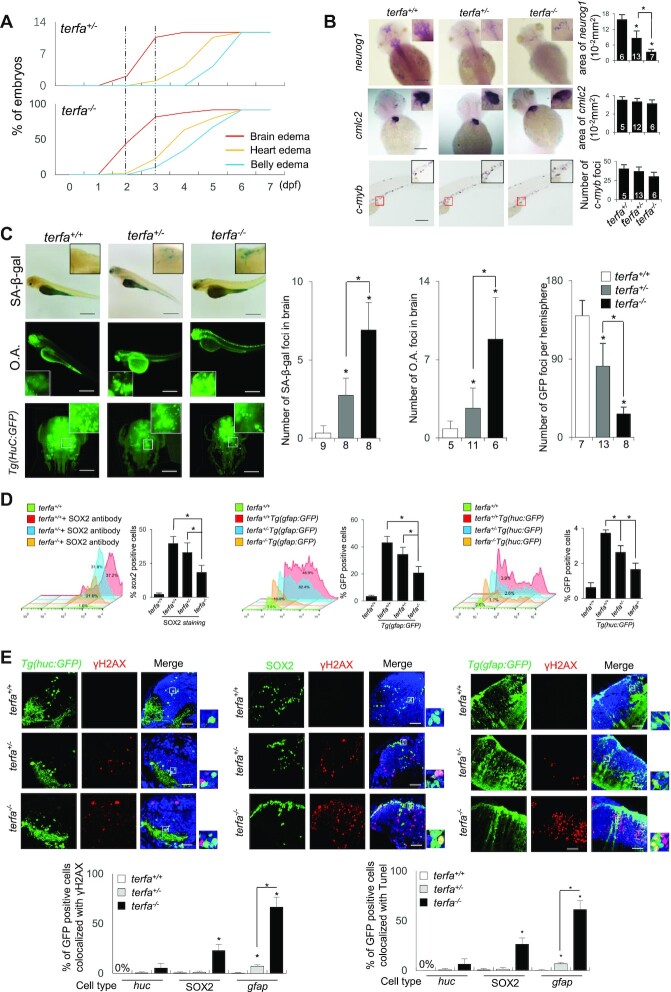
Zebrafish lacking zfTRF2 exhibit a specific neurodevelopmental failure. (**A**) Percentages of *terfa*-compromised embryos with indicated abnormal phenotypes during development (0–7 dpf). The dotted line indicates the dates at which the brain edema phenotype first presented and peaked. (**B**) Representative images (left) and signal quantification (right) from RNA fluorescence *in situ* hybridization (RNA-FISH) using tissue specific markers (neural tissue, *neurog1*; cardiovascular tissue, *cmcl2*; hemopoietic tissue, *c-myb*). Quantification was performed using ImageJ software (NIH). *Neurog1* and *cmcl2* signals were quantified by analyzing positive signals in the same area (10^−2^ mm^2^), and *c-myb* was quantified by counting foci (scale bar, 250 μm). (**C**) Representative images (left) and signal quantification (right) of SA-β-gal staining (up) of *terfa*-compromised embryos, acridine orange staining (middle) of *terfa*-compromised embryos and Light-sheet 3D reconstruction image of *Tg(HuC:GFP) terfa*-compromised brains at 3 dpf. Scale bar, 250 μm and 150 μm. (**D**) The neural cell types (neurons, glial cells, and progenitor cells) presented in *terfa*-compromised embryo brains at 3 dpf were quantified using flow cytometry. Left: the percentage of SOX2-positive (progenitor) cells was calculated by labeling embryos with anti-SOX2 antibody. Middle: percentage of GFP-positive cells (neurons) in *terfa*-compromised *Tg(huc:GFP)* embryos. Right: percentage of GFP-positive (glial) cells in *terfa*-compromised *Tg(gfap:GFP)* embryos. (**E**) Representative confocal section images (top) showing γH2AX foci colocalized with GFP-positive cells (green) in *terfa^+/+^, terfa*^+/−^ or *terfa*^−/−^ embryos at 3 dpf. GFP marked neurons in *Tg(huc:GFP)* brains and glial cells in *Tg(gfap:GFP)* brains. SOX2-positive cells represent progenitor cells (middle). The quantitative results (bottom) showing γH2AX foci (left) and TUNEL signals (right) colocalized with GFP-positive cells (green) in *terfa^+/+^, terfa*^+/−^ or *terfa*^−/−^ embryos at 3 dpf. Scale bars, 20 μm. Data are shown as the mean ± SEM of three biological replicates. The number inside or below each column indicates the number of embryos detected for each condition. Statistical analyses were performed using unpaired two-sided *t* tests, and survival curves were plotted using a log-rank test (*P < 0.05).

In *terfa-*compromised fish, the first prominent phenotype that appeared during development was brain edema, in agreement with the phenotype of an insertional mutant of *terfa* (30), followed by heart and belly edema (Figure 4A, Supplementary Figure S4A). Brain development failure in *terfa-*compromised fish was confirmed by a decreased expression of the pro-neural *neurogenin 1* gene, a neurodevelopment marker, at 2 days post-fertilization (dpf) (Figure 4B). At the same development stage (2 dpf), the expression levels of heart (*cmlc2*) and hematopoiesis (*c-myb*) development markers did not change showing that the developmental defects of these organs appear after the neurodevelopmental failure (Figure 4B). The brain edema phenotype was caused by a loss of function of the *terfa* mutation since it was fully rescued following injection with *terfa* mRNA, also in the heterozygous fish, again indicating an absence of dominant negative effect of the mutant gene (Figure 4B). Of note, injection of a mutant forms of *terfa* lacking the telobox sequence failed to rescue the brain edema phenotype, indicating that this telomeric DNA binding domain of zfTRF2 (Figure 2B) is required for neurodevelopment (Supplementary Figure S4B). The brains of the *terfa*-compromised fish were also strongly stained by senescence-associated beta-galactosidase (SA-β-gal) and acridine orange, indicative of a rapid burst of cellular senescence and apoptosis, respectively (Figure 4C). The partial or complete loss of *terfa* also resulted at 3 dpf in an increased DDR in fish brains, eyes and intestine, but not in muscle (Supplementary Figure S4C). The increased level of DDR was accompanied by a decrease in cell proliferation (Supplementary Figure S4D), as well as a reduced number of glial fibrillary acidic protein (*gfap*)-positive glial cells, *huc*-positive neurons, and *sox2*-positive pluripotent stem cells (Figure. 4D). Notably, the increase in γH2AX-positive and apoptotic cells, as measured by a TUNEL assay, was more pronounced in GFAP-positive glial cells than in HuC-positive neurons or SOX2-positive pluripotent cells (Figure 4E, Supplementary Figure S4E). Similar to observations in fish cell lines (Figure 1), the DDR increase in *terfa*-compromised brain cells was not enriched at telomeres (Supplementary Figure S4F). Moreover, in *terfa*-compromised embryos, we did not detect a significant change in telomere DNA length (Supplementary Figure S4G) or telomere abnormalities in embryonic metaphase spreads (Supplementary Figure S4H). Thus, zfTRF2 appears as a general genome maintenance factor in zebrafish with specific roles in brain development.

### The embryonic neurodevelopmental failure results from both ATM activation and transcriptional dysregulation

**Figure 5.**
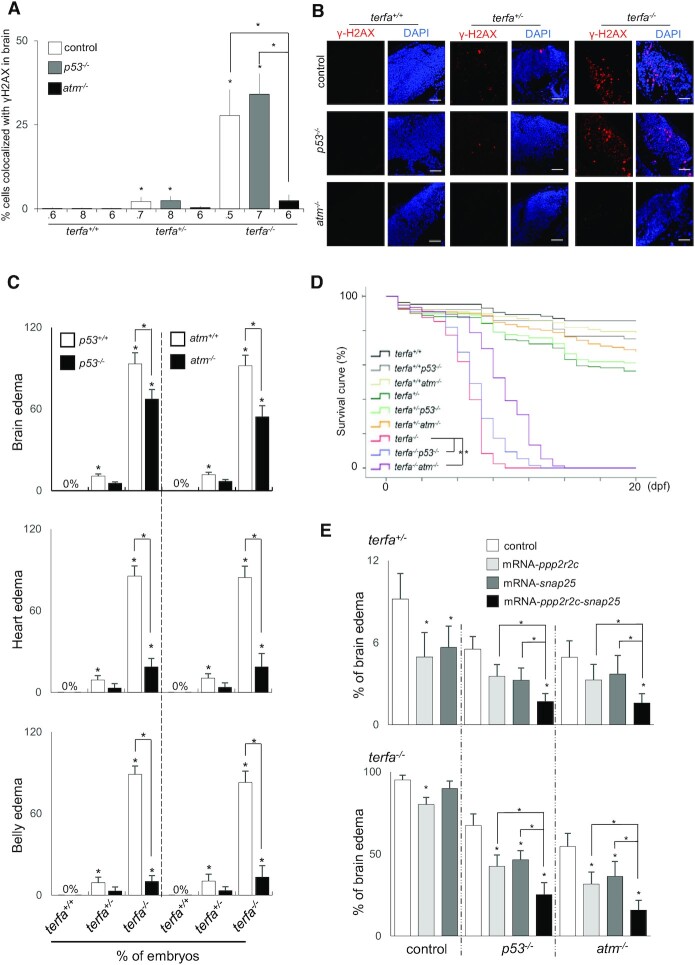
The embryonic neurodevelopmental failure results from both ATM activation and transcriptional dysregulation. (A, B) Representative (**B**) and quantification (**A**) of confocal section images of γH2AX foci (red) in the brains of *terfa*-compromised *p53*^−/−^ or *atm*^−/−^ embryos at 3 dpf (scale bars, 30 μm). (**C**) Percentages of *terfa*-compromised (*terfa*^+/+^*, terfa*^+/−^*, terfa*^−/−^) embryos with brain edema (left), heart edema (middle) and intestine edema (right) rescued by *p53*^−/−^ or *atm*^−/−^. (**D**) Survival curve of *terfa*-compromised *p53*^−/−^ or *atm*^−/−^ embryos during development. (**E**) Percentages of embryos at 3 dpf with brain edema phenotypes after microinjection with the indicated mRNA (*ppp2r2c, snap25* or *ppp2r2c + snap25*) in *terfa*^+/−^*:p53*^−/−^ or *terfa*^+/−^*:atm*^−/−^ (left) and *terfa*^−/−^*:p53*^−/−^ or *terfa*^−/−^*:atm*^−/−^ (right) embryos. All data are shown as the means ± SEM of three biological replicates. Statistical analyses were performed using unpaired two-sided *t* tests; survival curves were plotted using the log-rank test (*P < 0.05). The number below the graph indicates the total number of tested individual embryos. Asterisks directly above columns indicate a significant difference between the indicated treatment and the control group (left). Asterisks above two columns indicate a significant difference between the two columns.

To investigate whether the DDR induced by *terfa* deficiency is responsible for neurodevelopmental abnormalities, we introduced null alleles of *atm*, encoding a key DDR signaling kinase blunted by TRF2 in mammals and of *p53*, encoding a central effector of DDR signaling downstream of DDR activation and involves in senescence initiation (Supplementary Figure S5A, B). In agreement with a major ATM activation in *terfa-*compromised fish, invalidating *atm*, but not *p53*, totally abolished the DDR signaling in the brain of *terfa*-compromised fish, even in homozygous fish (Figure 5A, B). Moreover, pharmacological inhibition of ATM and ATR activities showed that zfTRF2 specifically inhibited *atm*-dependent DDR activation, similar to its ortholog in mammals (Supplementary Figure S5C). Accordingly, *atm*^−/−^ and *p53*^−/−^*terfa*-compromised fish displayed a reduction in the rate of senescence and neuron loss (Supplementary Figure S5D−F). If the inhibition of either *atm* or *p53* almost fully rescued the heart and belly edema, it only partially reduced the brain edema (Figure 5C), neuron loss (Supplementary Figure S5D) and embryonic death rate (Figure 5E). We concluded that *atm*-dependent activation of the DDR contributed only partially to the neurodevelopmental failure of *terfa*-compromised fish while it is the main cause for developmental abnormalities in other organs. This indicates that zfTRF2 performs DDR-independent functions specifically during neurodevelopment. In order to identify the DDR-independent functions of zfTRF2 involved in neurodevelopment, we performed RNA-seq experiments of 72hpf embryos, comparing WT with homozygous and heterozygous fish (Supplementary Figure S5G−I, Supplementary Tables S1−S3). Remarkably, functional analysis of the genes in common between homozygous and heterozygous fish that are differentially expressed as compared to WT, revealed a gene enrichment in KEGG pathways related to cell cycle control, inflammation and neuronal functions (Supplementary Figure S5J). Specifically, these pathways include: (i) cell cycle dysregulation (e.g. senescence, p53, spliceosome, RNA transport, Ribosome, mRNA surveillance and cell cycles), including an increased expression of two p53-targeted genes, *cdkn1a* (p21) and *gadd45*; (ii) inflammation (e.g. Salmonella and Herpes simplex infections, TOL-like receptor signaling and Cell Adhesion), including an increased expression of two pro-inflammatory genes, *il6* and *isg15*; (iii) neuronal dysfunction (e.g. phototransduction, calcium signaling and neuroactive ligand-receptor interaction), including a signature of glial injury (*gfap*, *ctsl*, *metrn*, *gcm2*, *clu* and five members of the *slc1a* family (3–5,7,8)). A set of these differentially expressed genes were validated by RT-qPCR (Supplementary Figure S5K). Generally, these pathways are more markedly altered in homozygous and in heterozygous fish, correlating with the severity of their phenotypes. For instance, in homozygous there is an upregulation of other pro-inflammatory genes (e.g. *il1b*, *cxcl8* and *mmp9*) and a downregulation of other neural genes (e.g. *slc1a2*, *slc1a6*, *ppp2r2c* and *snap25*) (Supplementary Table S2). Noteworthy, the expression of *ppp2r2c* and *snap25* were previously shown to be downregulated by zfTRF2 in morpholino experiments as well as the ortholog of *snap25* in human cells (31). Moreover, both *PPP2R2C* and *SNAP25* genes are bound by TRF2 in human cells (31). Overall, these results indicate that *terfa*-compromised embryos trigger a potent growth arrest, which is likely to result from the DNA damages triggered by *terfa* downregulation, accompanied by transcriptional changes indicative of cell cycle dysregulation, neural dysfunction and inflammation.

To investigate whether the reduced expression of *ppp2r2c* and *snap25* genes contributed to neurodevelopmental failure in *terfa*-compromised fish, we injected their mRNA into *terfa*-compromised zygotes. The restoration of *ppp2r2c* expression decreased the rate of brain edema in both homozygous and heterozygous fish, whereas *snap25* restoration only partially rescued brain edema in heterozygous and homozygous fish (Figure 5E, Supplementary Figure S5L−M). The combined restoration of *ppp2r2c* and *snap25* expression had an additive effect on brain edema reduction in heterozygous fish (Figure 5E), thus indicating that genes regulated by zfTRF2 contribute individually to brain development. In *atm*^−/−^ and *p53*^−/−^*terfa*-compromised fish, the restoration of *ppp2r2c* and *snap25* further decreased the incidence of brain edema (Figure 5E). These transcriptional effects of zfTRF2 could result from the reported interaction, in mammalian cells, of TRF2 with repressor element 1 silencing transcription factor (REST), a master repressor of neuronal gene networks (32–34). Indeed, the *snap25* gene is a known target of REST and TRF2 (33). However, overexpressing *rest* mRNA in *terfa*-compromised fish only slightly rescued the neurodevelopmental phenotype (Supplementary Figure S5L). Overall, these results showed that the loss of *terfa* affected neurodevelopment in a DDR-independent manner through the downregulation of a specific neural gene network, including *ppp2r2c* and *snap25*.

### zfTRF2 plays a specific role in GFAP-positive cells during neurodevelopment

**Figure 6.**
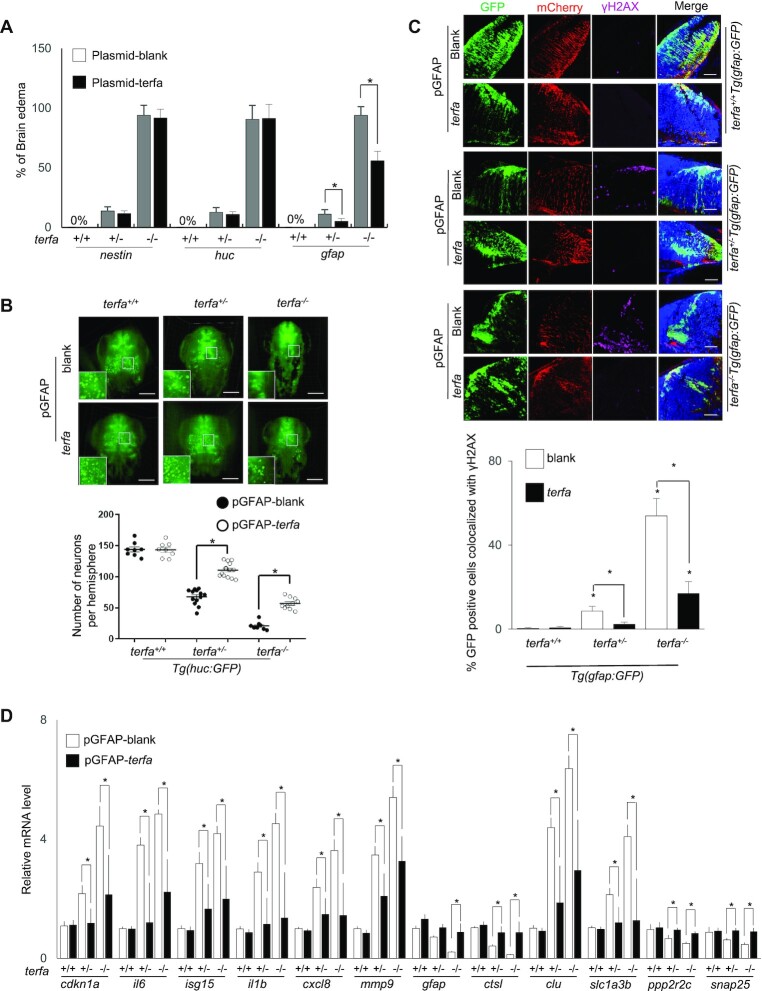
zfTRF2 plays a specific role in GFAP-positive cells during neurodevelopment. (**A**) Percentages of embryos exhibiting brain edema phenotypes at 3 dpf in *terfa*-compromised embryos following microinjection with the plasmids described in (Supplementary Figure S6A). (**B**) Representative three-dimensional (3D) computationally-reconstructed brain images of living embryos. Images were taken via light-sheet microscopy. Green signals indicate neurons of *terfa*-compromised *Tg(huc:GFP)* embryos at 3 dpf after microinjection with the plasmid of expression zfTRF2 meditate by GFAP promotor. GFP-positive cells in whole brains were quantified using Imaris software (Bitplane). scale bars, 150 μm. (**C**) Representative confocal section images (right) and quantification (left) showing γH2AX foci colocalized with GFP-positive or mCherry-positive cells in the brains of *terfa*-compromised *Tg(gfap:GFP)* embryos at 3 dpf, following microinjection plasmid of expression zfTRF2 meditate by GFAP promotor. The quantification (left) shows that percentages of GFP-positive cells that colocalized with γH2AX foci in *terfa*-compromised *Tg(gfap:GFP)* embryos, scale bars, 30 μm. (**D**) Transcript levels of candidate genes from RNA-seq in *terfa*^+/+^, *terfa*^+/−^ and *terfa*^−/−^ embryos at 3 dpf. Embryos were microinjected with the plasmids described in Supplementary Figure S6A. The mRNA transcript levels were measured using RT-qPCR. All data are shown as the mean ± SEM of three biological replicates. The number below each column indicates the number of embryos detected for each condition. Statistical analysis was performed using the unpaired two-sided *t* test, and survival curves were plotted using the log-rank test (*P < 0.05). Asterisks directly above columns indicate a significant difference between the indicated treatment and the control group (left). Asterisks above two columns indicate a significant difference between the two columns.

To identify which neural cells were responsible for neurodevelopmental failure in *terfa*-compromised fish, we selectively restored the expression of zfTRF2 in three neural cell types in *terfa-*compromised fish. This was achieved by injecting a construct containing the *terfa* gene fused to mCherry by a P2A peptide linker. The construct was placed under the control of the *gfap*, *huc*, or *nestin* promoters, which drive gene expression specifically in glial, neuron, or progenitor cells, respectively (Supplementary Figure S6A, B). Recovery from neurodevelopmental failure, which was determined by a reduction in brain edema and an increase in neuron and glial cells, was only observed when zfTRF2 expression was driven by the *gfap* promoter (Figure 6A, B, Supplementary Figure S6C, D). Concomitantly, only the restoration of zfTRF2 expression in *gfap*-positive cells rescued the increased DDR due to *terfa* deficiency in brain cells (Figure 6C, Supplementary Figure S6E, F). The transcriptional dysregulation observed in *terfa-*compromised embryos was also rescued by restoring the expression of zfTRF2 specifically in *gfap*-positive cells, including genes of the glial signature (Figure 6D, Supplementary Figure S6G, H). These results show that zfTRF2 plays a specific role in *gfap*-positive cells for neuronal homeostasis. An interesting possibility is that zfTRF2 controls the fate of *gfap*-positive radial glial cells, which are progenitors for several types of neural cells, including neurons, during development (35).

Noteworthy, the restoration of *terfa* expression upon *gfap* also reduced the rate of heart and belly edema as well as the DDR in eye and intestine cells but not in cartilage cells (Supplementary Figure S6I−K). These results suggest that *terfa* also plays a role in *gfap*-positive cells outside the brain (23). Another non-exclusive explanation is that the restoration of a normal neurodevelopment upon the expression of *terfa* in *gfap*-positive brain cells has systemic effects on peripheral organs.

## DISCUSSION

Overall, this study has revealed that the shelterin subunit zfTRF2 is required for brain development through two separate functions: one preventing ATM activation and the other regulating the transcription of neural genes. This dual role of zfTRF2 in genome maintenance and transcriptional regulation is specifically required for neurodevelopment since the developmental failures of other organs (heart and belly) is fully restored upon *atm* ablation. This specificity can be explained by the fact that zfTRF2 regulates the expression of neural genes, as shown here for *ppp2r2c* and *snap25*. How does zfTRF2 dosage regulate genome-wide stability and the transcription remains to be determined. One could hypothesize that zfTRF2 controls a higher-order chromatin structure, similar to its role in the regulation of the sub-telomeric *SIRT3* gene in human muscle cell through long-range subtelomeric looping (20) or controls pericentromere heterochromatin replication as suggested by its increased association during S-phase (Figure 1) and a similar function in human cells (27). One interesting possibility is that zfTRF2 mediates the formation of large chromatin loops between specific telomeres and internal genes to regulate their expression, in agreement with the binding of zfTRF2 to a subset of telomeres in zebrafish cells and the fact that the neurodevelopmental role of zfTRF2 requires its telobox DNA binding domain. Such structural effects of zfTRF2 on long-range chromatin organization are expected to be dosage dependent, providing an explanation for the detrimental phenotype of *terfa* haploinsufficiency, a situation mimicking the ∼50% reduction of *terfa* expression occurring in aging brain of wild type fish (36).

Our results show that a complete absence of *terfa* gene allows a seemingly normal beginning of organogenesis and therefore somatic differentiation. Such an ability to protect telomeres independently of TRF2 during early development was also reported in mice showing that eukaryotes using TRF2 as a somatic telomere protective factor are also equipped with alternative telomere capping mechanisms (37,38).

TRF1 and TRF2 originated from a common TRF ancestor and duplicated during genomic replication at the root of the chordate lineage circa 500 million years ago (28,39,40). TRF proteins with demonstrated telomere specific function are quite widespread among eukaryotes such as Taz1 in the fission yeast or tbTRF in *Trypanosoma brucei* (41). In contrast to mouse and human cells, our results show that zfTRF2 binds and protect only to a subset of telomeres in somatic fish cells. If zfTRF2 lost part of its telomere-specific functions during fish evolution, why its intrinsic property to bind telomeres was conserved? An explanation could be that, even if partial, the binding and protection of zfTRF2 to telomeres is important to counteract aging, as suggested by the specific role of telomere deprotection in a progeroid mouse model (18). The general tole of zfTRF2 in genome maintenance supports the model in which telomeres evolved from general components of genome maintenance pathways having acquired telomere specific protective functions during their evolution (1). Our results further suggests that telomere factors can subsequently lose part of their telomeric roles while acquiring or reinforcing other non-telomeric properties. These evolutionary trajectories could be driven by developmental demands requiring different facets of the shelterin proteins. Such a back-and-forth evolution could also explain the enigmatic variety of telomere protective factors found throughout the eukaryotic domain, despite the fact that telomere protection is an essential and conserved process.

This work revealed zebrafish as an invaluable model for understanding the evolution and multifunctionality of shelterin proteins, in particular their roles during development. Research in this area could aid the development of drugs with non-telomeric targets to fight against age-related diseases, thus avoiding the potential pro-oncogenic effects of reinforced telomere protection.

## DATA AVAILABILITY

The data that support the findings of this study are available from the corresponding authors upon reasonable request (DOI 10.5281/zenodo.5672266). All raw sequencing reads for RNA-seq are available on NCBI Gene Expression Omnibus under accession number GSE173639.

## Supplementary Material

gkac065_Supplemental_Files
