# Supplementary material for: The non-telomeric evolutionary trajectory of TRF2 in zebrafish reveals its specific roles in neurodevelopment and aging
Source: Nucleic Acids Res. 2022 Feb 12;50(4):2081–95. doi: 10.1093/nar/gkac065 (PMC8887477; doi:10.1093/nar/gkac065)
Supplement: gkac065_Supplemental_Files [file gkac065_supplemental_files.zip › NAR_All_Supplemental Figures and legends 2021-12-13.pdf]

Figure S1

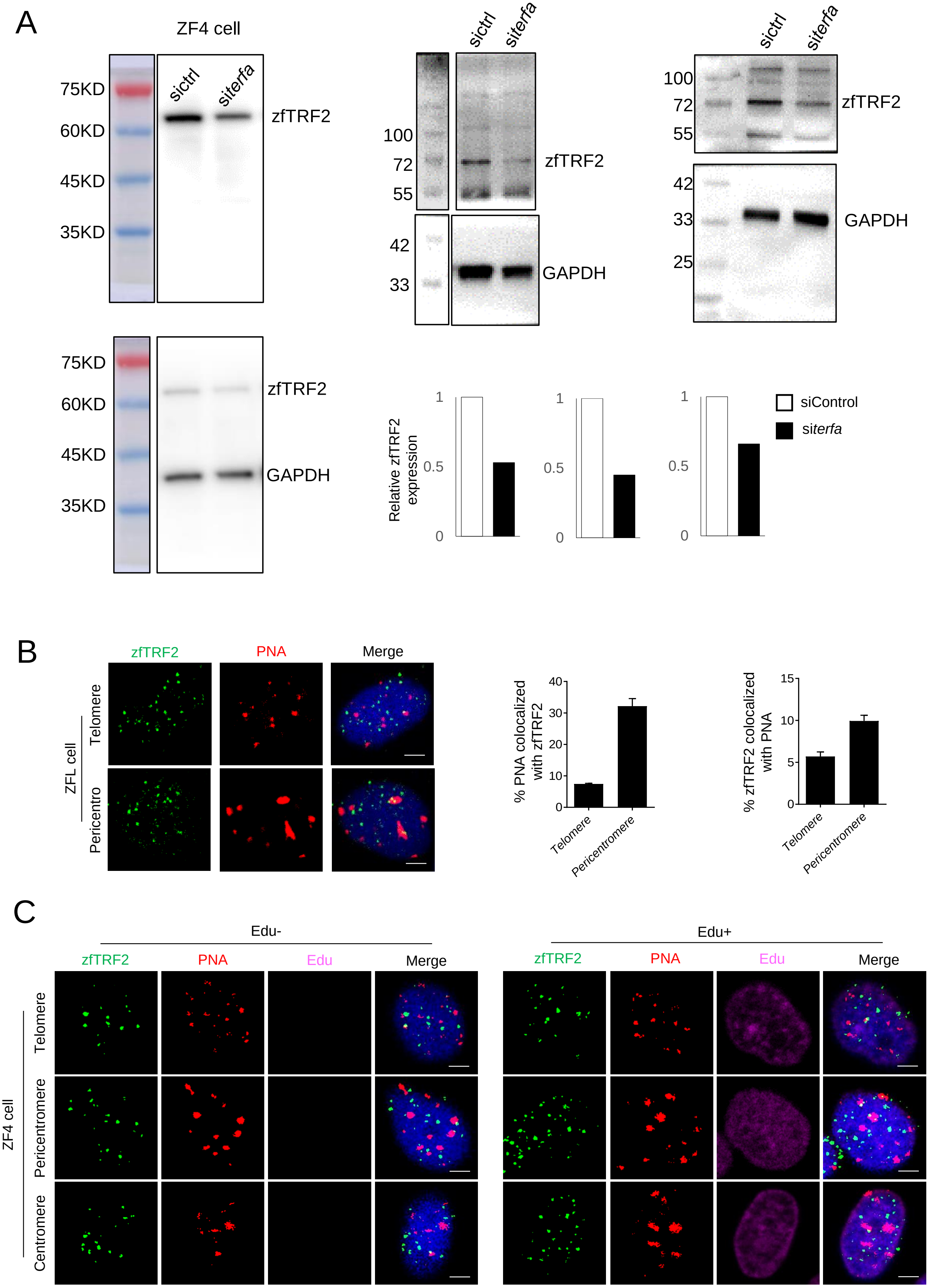

A
ZF4 cell
75KD
60KD
45KD
35KD
sictrl
siterfa
zfTRF2
GAPDH
100
72
55
42
33
25
Relative zfTRF2 expression
1
0.5
0
siControl
siterfa
B
zfTRF2
PNA
Merge
ZFL cell
Telomere
Pericentro
% PNA colocalized with zfTRF2
% zfTRF2 colocalized with PNA
40
30
20
10
15
5
Pericentromere
C
Edu-
Edu+
Edu
ZF4 cell
Centromere

Figure S1

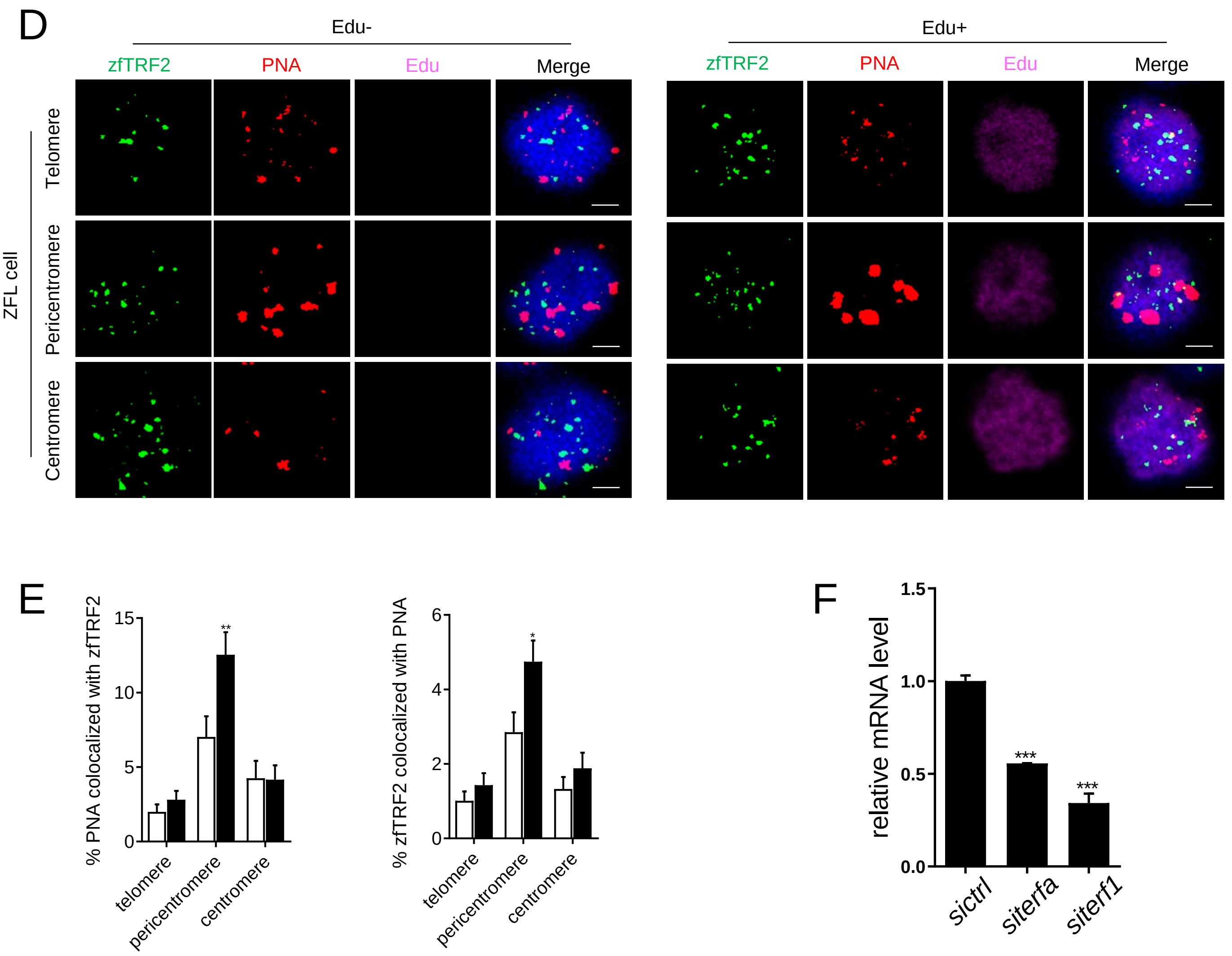
D
Edu-
Edu+
zfTRF2
PNA
Edu
Merge
ZFL cell
Telomere
Pericentromere
Centromere
E
% PNA colocalized with zfTRF2
15
10
5
0
**
telomere
pericentromere
centromere
% zfTRF2 colocalized with PNA
6
4
2
0
*
F
relative mRNA level
1.5
1.0
0.5
0.0
***
***
sictrl
siterfa
siterf1

Figure S2

A

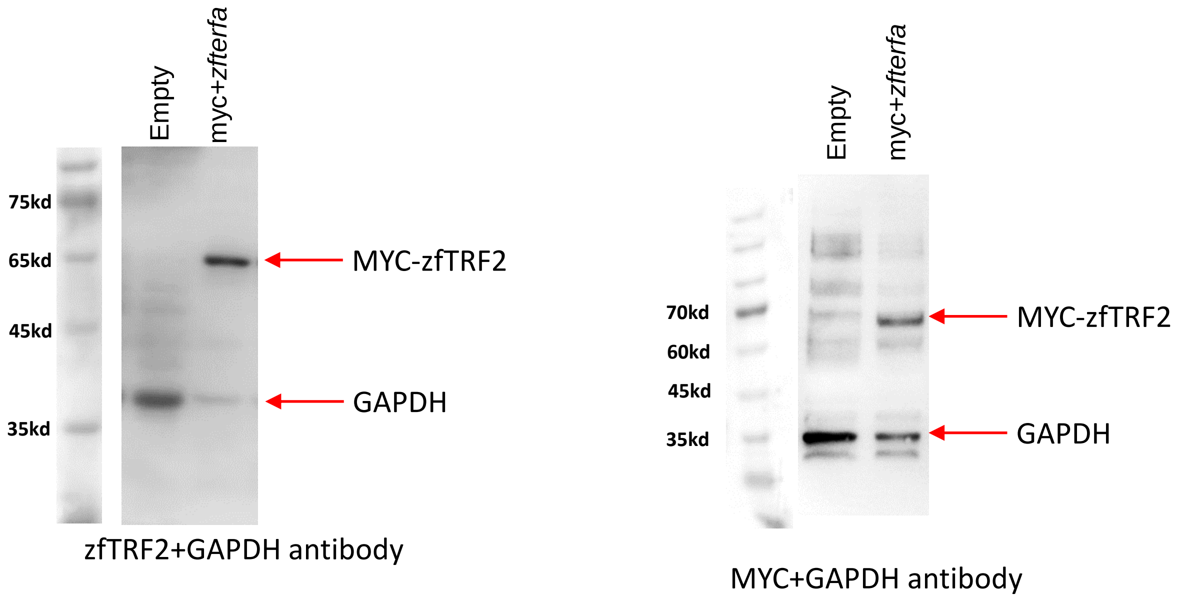

Empty
myc+zfterfa
75kd
65kd
45kd
35kd
MYC-zfTRF2
GAPDH
zfTRF2+GAPDH antibody
Empty
myc+zfterfa
70kd
60kd
45kd
35kd
MYC-zfTRF2
GAPDH
MYC+GAPDH antibody

B

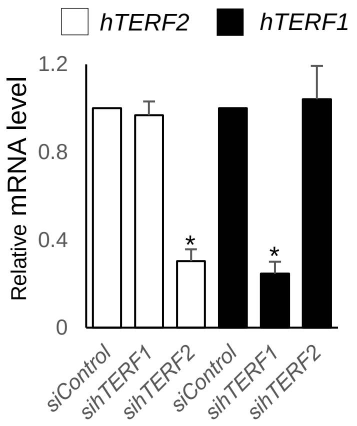

hTERF2
hTERF1
Relative mRNA level
1.2
0.8
0.4
0
*
*
siControl
sihTERF1
sihTERF2
siControl
sihTERF1
sihTERF2

Figure S3

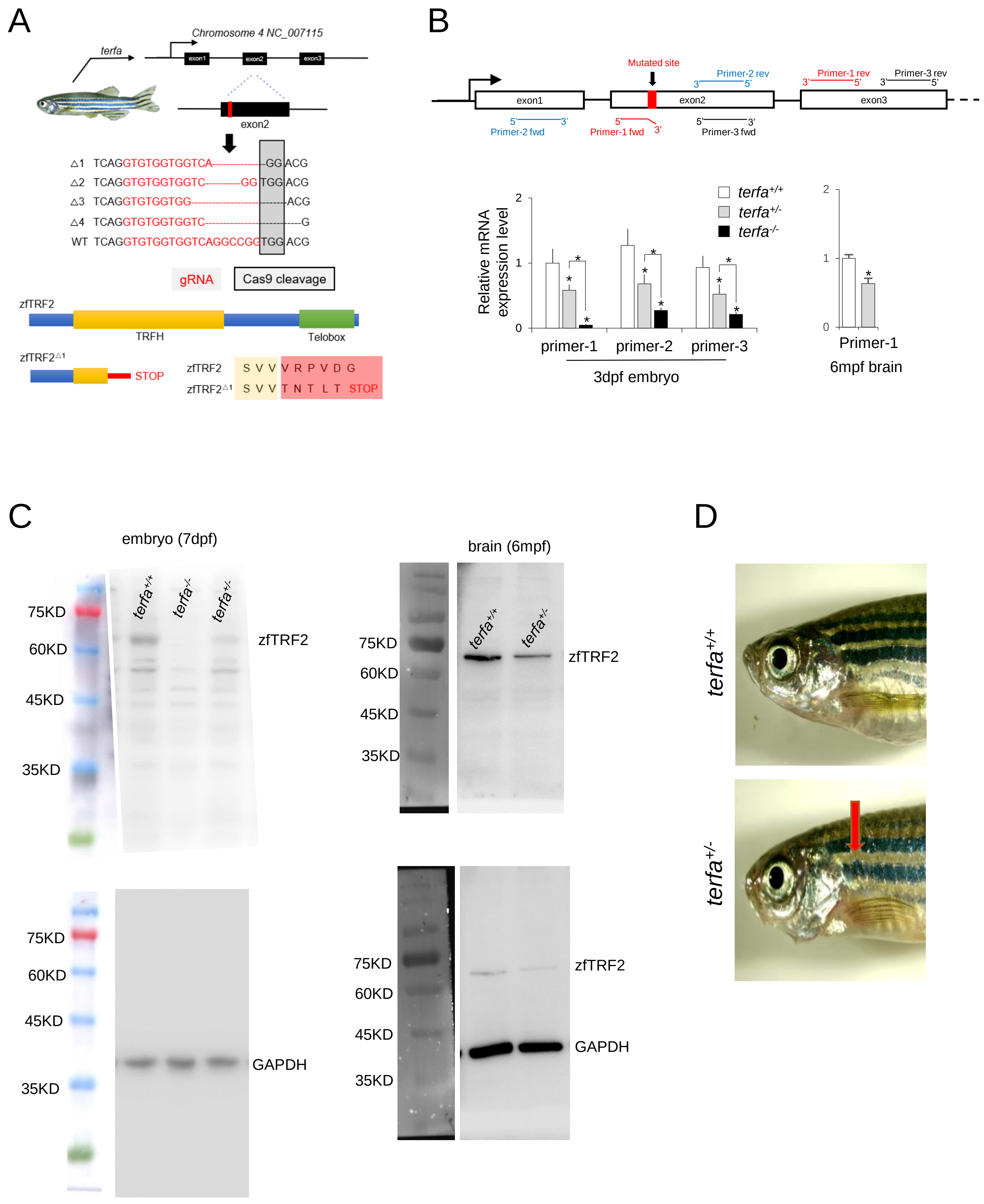

A
terfa
Chromosome 4 NC_007115
exon1
exon2
exon3
exon2
△1 TCAGGTGTGGTGGTCA-------------GG ACG
△2 TCAGGTGTGGTGGTC---------GG TGG ACG
△3 TCAGGTGTGGTGG-------------------ACG
△4 TCAGGTGTGGTGGTC--------------------G
WT TCAGGTGTGGTGGTCAGGCCGGTGG ACG
gRNA
Cas9 cleavage
zfTRF2
TRFH
Telobox
zfTRF2△1
STOP
zfTRF2 S V V V R P V D G
zfTRF2△1 S V V T N T L T STOP
B
Mutated site
Primer-2 rev
Primer-1 rev
Primer-3 rev
exon1
exon2
exon3
Primer-2 fwd
Primer-1 fwd
Primer-3 fwd
Relative mRNA expression level
terfa+/+
terfa+/-
terfa-/-
primer-1
primer-2
primer-3
3dpf embryo
Primer-1
6mpf brain
C
embryo (7dpf)
terfa+/+
terfa-/-
terfa+/-
75KD
60KD
45KD
35KD
zfTRF2
GAPDH
brain (6mpf)
terfa+/+
terfa+/-
D
terfa+/+
terfa+/-

Figure S4

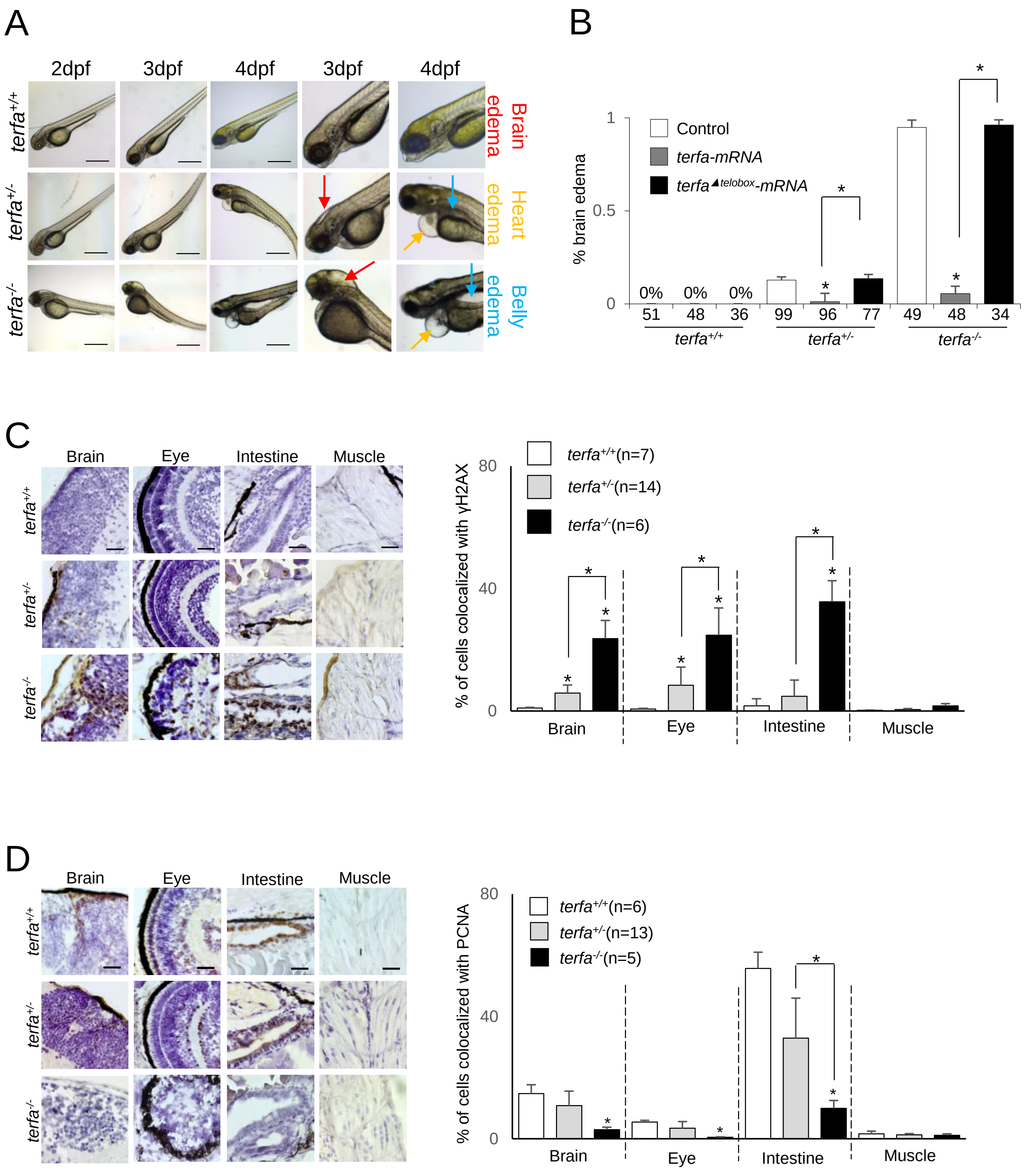
A
2dpf
3dpf
4dpf
3dpf
4dpf
terfa+/+
terfa+/-
terfa-/-
Brain edema
Heart edema
Belly edema
B
% brain edema
Control
terfa-mRNA
terfa▲telobox-mRNA
0%
0%
0%
51
48
36
99
96
77
49
48
34
C
Brain
Eye
Intestine
Muscle
% of cells colocalized with γH2AX
terfa+/+(n=7)
terfa+/-(n=14)
terfa-/-(n=6)
D
% of cells colocalized with PCNA
terfa+/+(n=6)
terfa+/-(n=13)
terfa-/-(n=5)

Figure S4

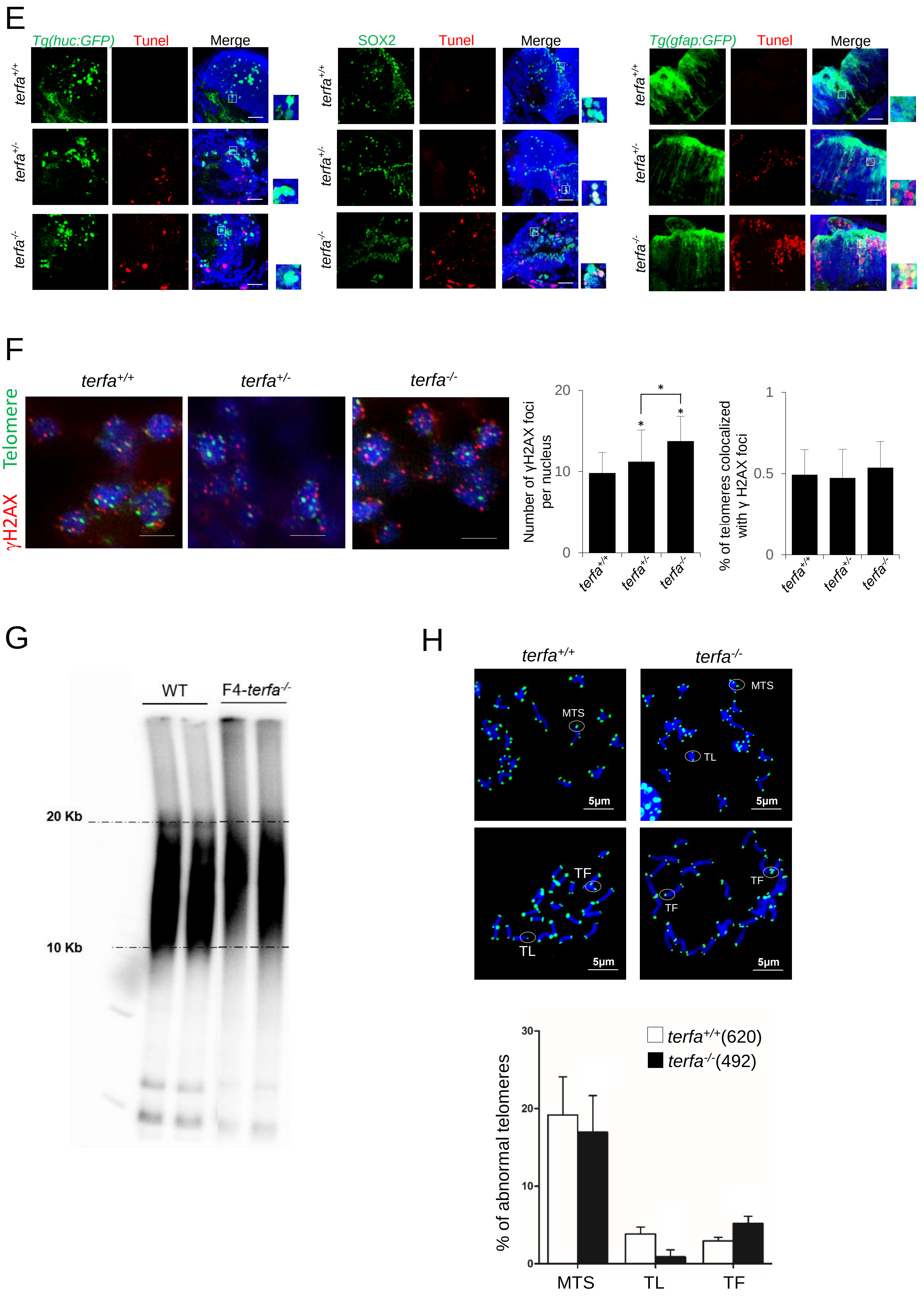
E
Tg(huc:GFP)
Tunel
Merge
SOX2
Tunel
Merge
Tg(gfap:GFP)
Tunel
Merge
terfa+/+
terfa+/-
terfa-/-
F
terfa+/+
terfa+/-
terfa-/-
Telomere
γH2AX
Number of γH2AX foci per nucleus
20
10
0
*
% of telomeres colocalized with γ H2AX foci
1
0.5
0
G
WT
F4-terfa-/-
20 Kb
10 Kb
H
terfa+/+
terfa-/-
MTS
TL
TF
5µm
% of abnormal telomeres
30
20
10
0
terfa+/+(620)
terfa-/-(492)
MTS
TL
TF

Figure S5

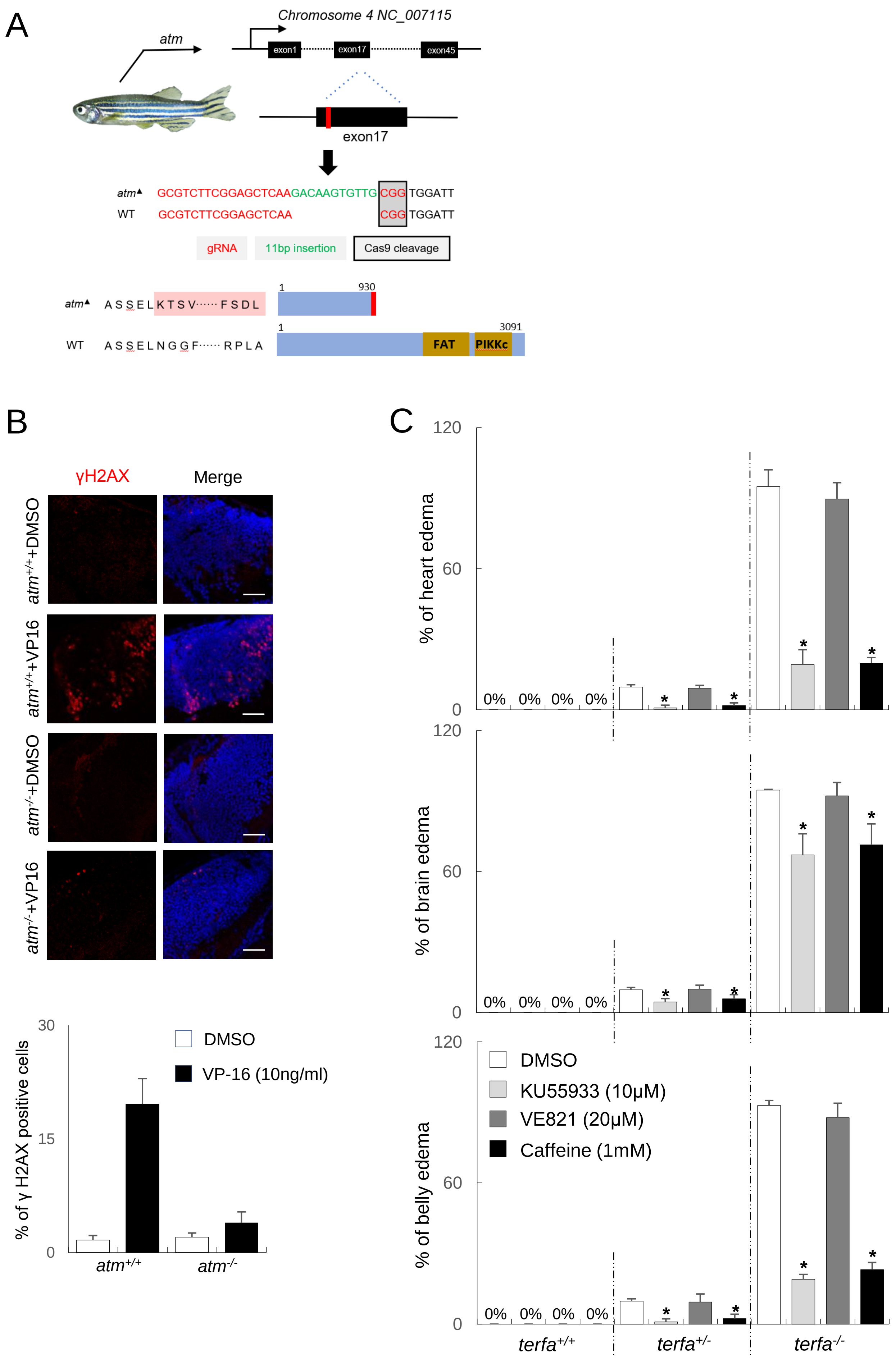
A
atm
Chromosome 4 NC_007115
exon1
exon17
exon45
exon17
atm▲ GCGTCTTCGGAGCTCAAGACAAGTGTTGCGGTGGATT
WT GCGTCTTCGGAGCTCAACGGTGGATT
gRNA
11bp insertion
Cas9 cleavage
atm▲ A S S E L K T S V······ F S D L
1
930
WT A S S E L N G G F······ R P L A
1
3091
FAT
PIKKc
B
γH2AX
Merge
atm+/++DMSO
atm+/++VP16
atm-/-+DMSO
atm-/-+VP16
% of γ H2AX positive cells
30
15
0
DMSO
VP-16 (10ng/ml)
atm+/+
atm-/-
C
% of heart edema
% of brain edema
% of belly edema
120
60
0
0%
DMSO
KU55933 (10μM)
VE821 (20μM)
Caffeine (1mM)
terfa+/+
terfa+/-
terfa-/-

Figure S5

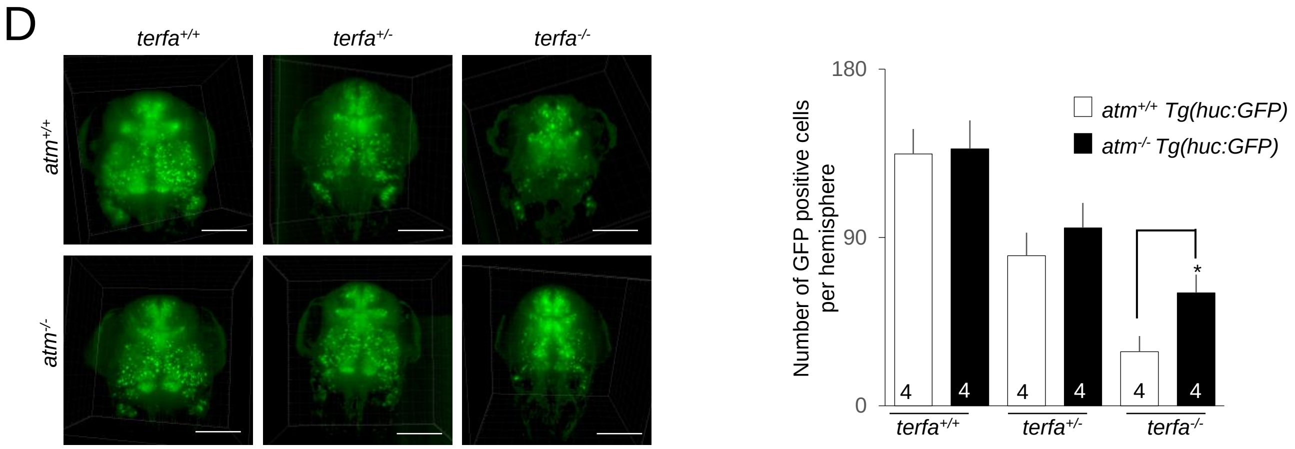
D
terfa+/+
terfa+/-
terfa-/-
atm+/+
atm-/-
180
90
0
Number of GFP positive cells per hemisphere
atm+/+ Tg(huc:GFP)
atm-/- Tg(huc:GFP)
4 4 4 4 4 4
*
terfa+/+
terfa+/-
terfa-/-

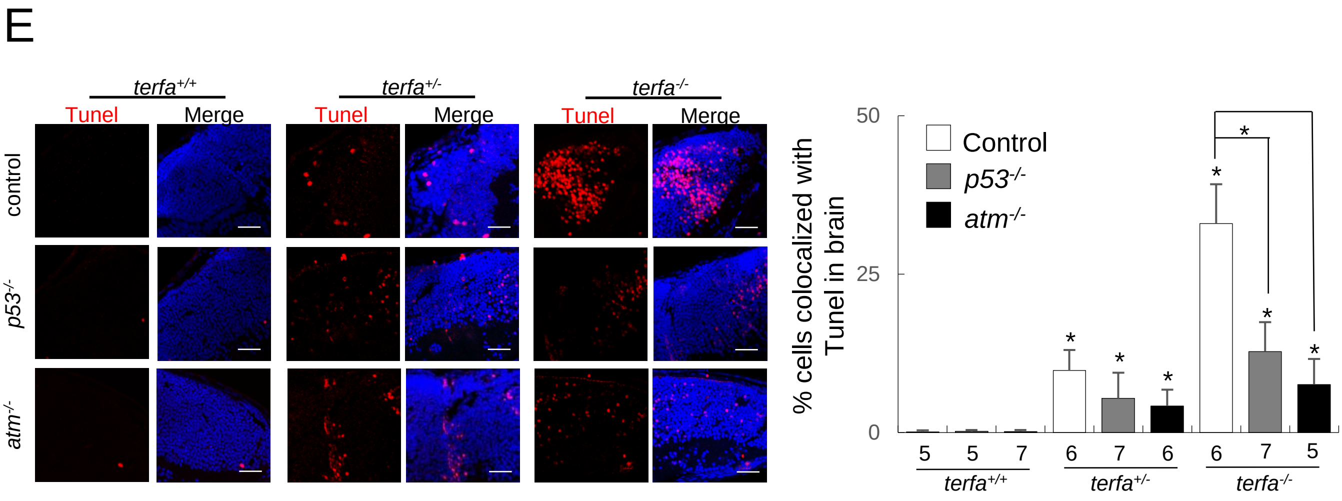
E
terfa+/+
terfa+/-
terfa-/-
Tunel
Merge
control
p53-/-
atm-/-
50
25
0
% cells colocalized with Tunel in brain
Control
p53-/-
atm-/-
*
5 5 7 6 7 6 6 7 5
terfa+/+
terfa+/-
terfa-/-

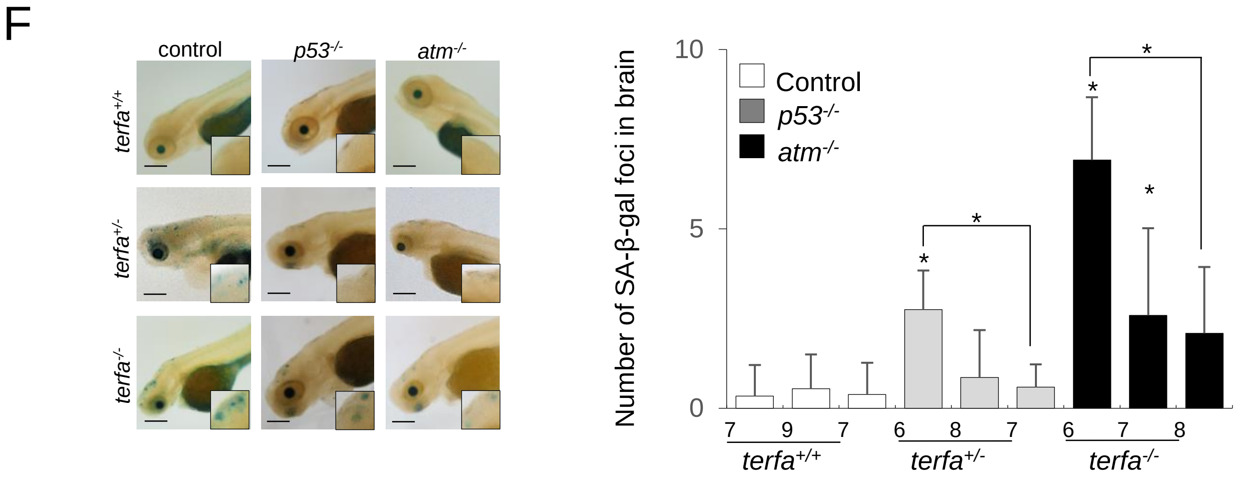
F
control
p53-/-
atm-/-
terfa+/+
terfa+/-
terfa-/-
10
5
0
Number of SA-β-gal foci in brain
Control
p53-/-
atm-/-
*
7 9 7 6 8 7 6 7 8
terfa+/+
terfa+/-
terfa-/-

Figure S5

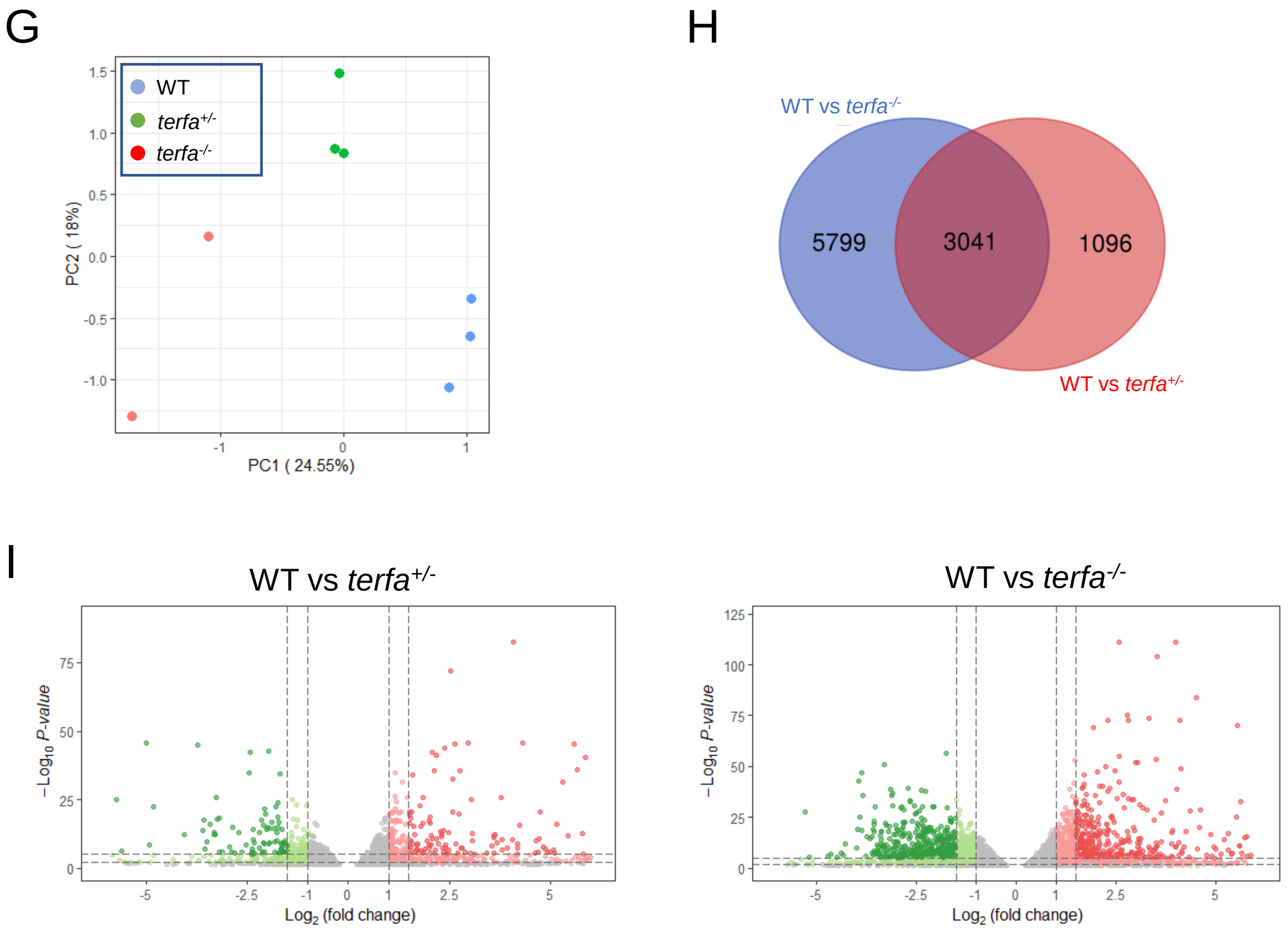

G
WT
terfa+/-
terfa-/-
1.5
1.0
0.5
0.0
-0.5
-1.0
PC2 (18%)
-1
0
1
PC1 (24.55%)
H
WT vs terfa-/-
5799
3041
1096
WT vs terfa+/-
I
WT vs terfa+/-
75
50
25
0
−Log10 P-value
-5
-2.5
-1
0
1
2.5
5
Log2 (fold change)
WT vs terfa-/-
125
100
75
50
25
0
−Log10 P-value
-5
-2.5
-1
0
1
2.5
5
Log2 (fold change)

Figure S5

J

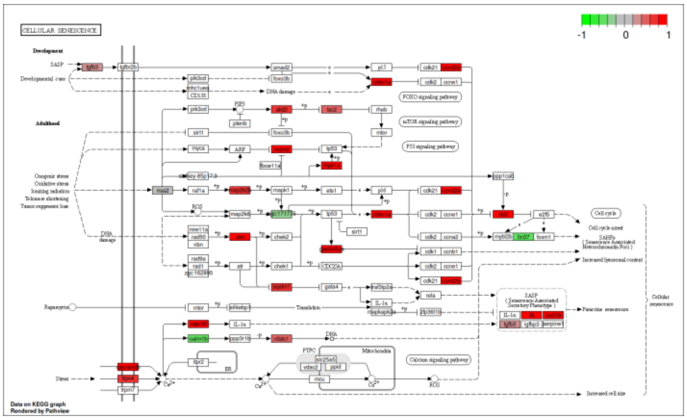
CELLULAR SENESCENCE
-1 0 1
Data on KEGG graph
Rendered by Pathview

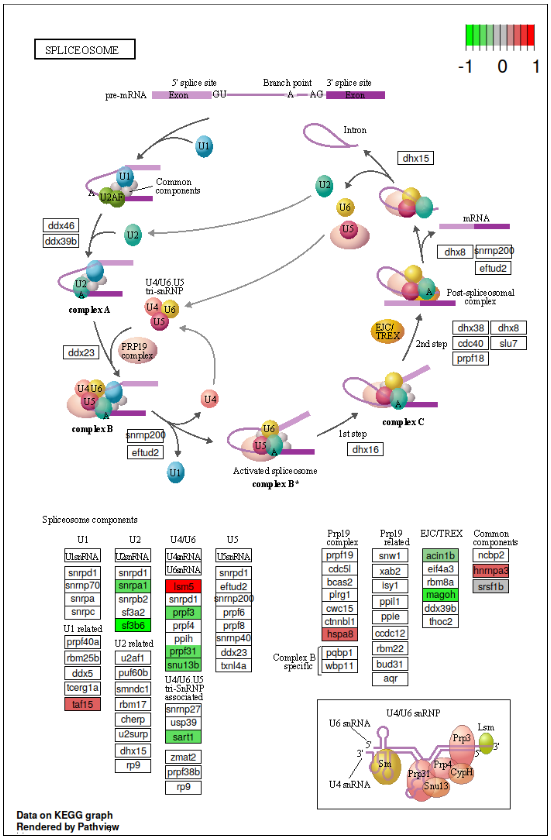
SPLICEOSOME
-1 0 1
Spliceosome components
Data on KEGG graph
Rendered by Pathview

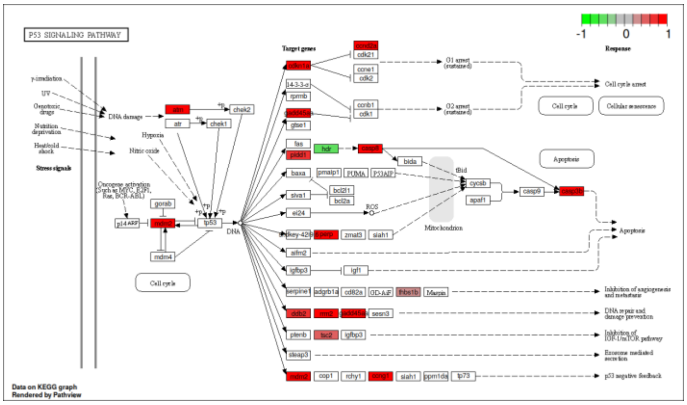
P53 SIGNALING PATHWAY
-1 0 1
Data on KEGG graph
Rendered by Pathview

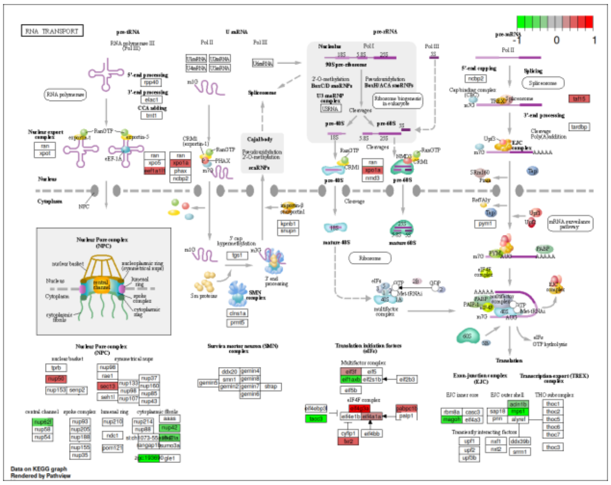
RNA TRANSPORT
-1 0 1
Data on KEGG graph
Rendered by Pathview

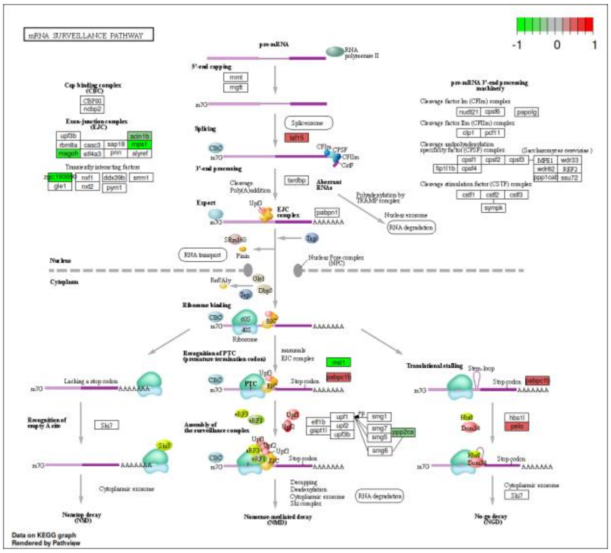
mRNA SURVEILLANCE PATHWAY
-1 0 1
Data on KEGG graph
Rendered by Pathview

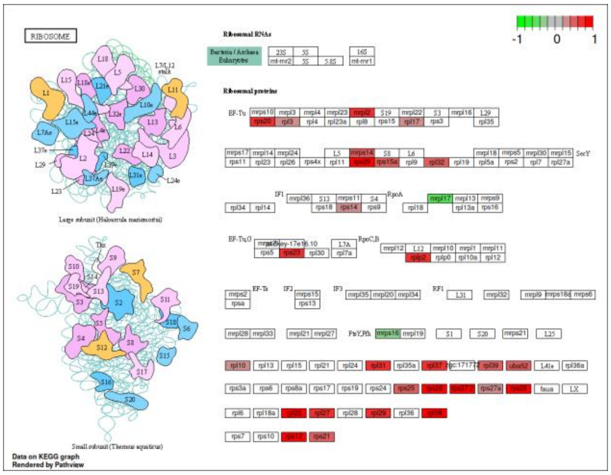
RIBOSOME
-1 0 1
Large subunit (Haloarcula marismortui)
Small subunit (Thermus aquaticus)
Data on KEGG graph
Rendered by Pathview

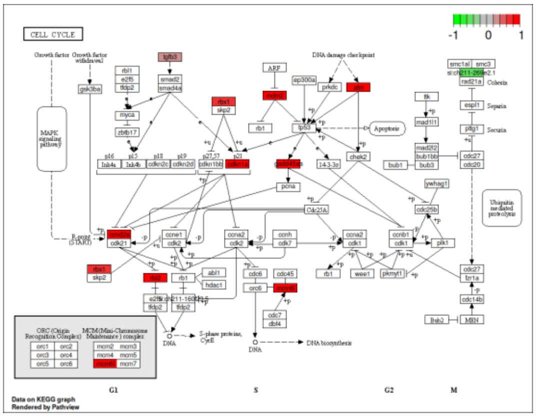
CELL CYCLE
-1 0 1
Data on KEGG graph
Rendered by Pathview

Figure S5

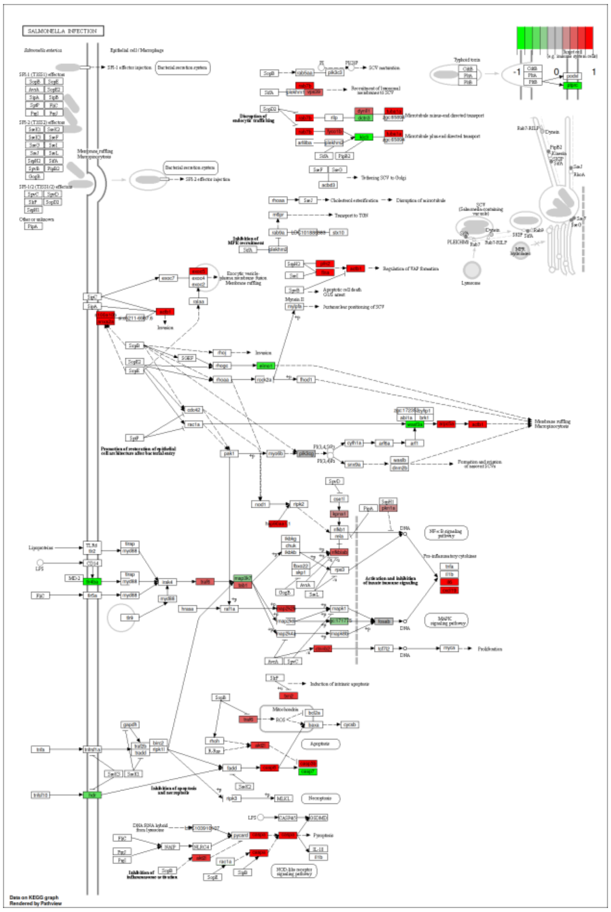

SALMONELLA INFECTION
-1
0
1
Data on KEGG graph
Rendered by Pathview

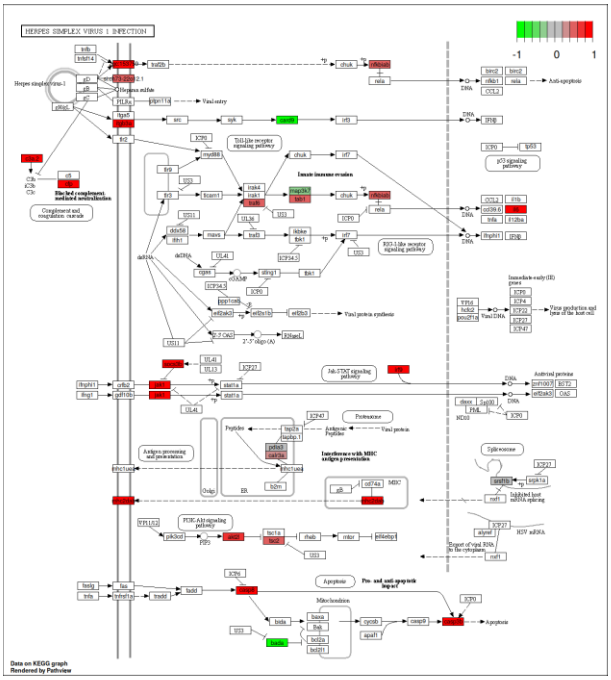

HERPES SIMPLEX VIRUS 1 INFECTION
-1
0
1
Data on KEGG graph
Rendered by Pathview

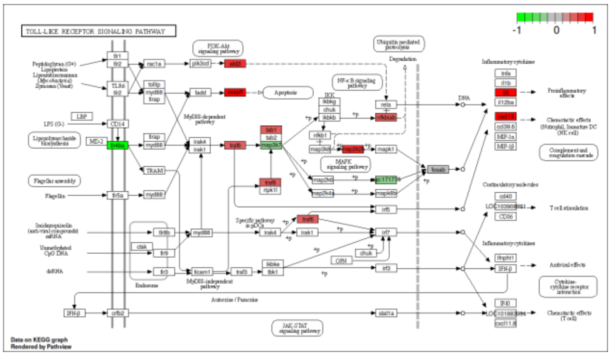

TOLL-LIKE RECEPTOR SIGNALING PATHWAY
-1
0
1
Data on KEGG graph
Rendered by Pathview

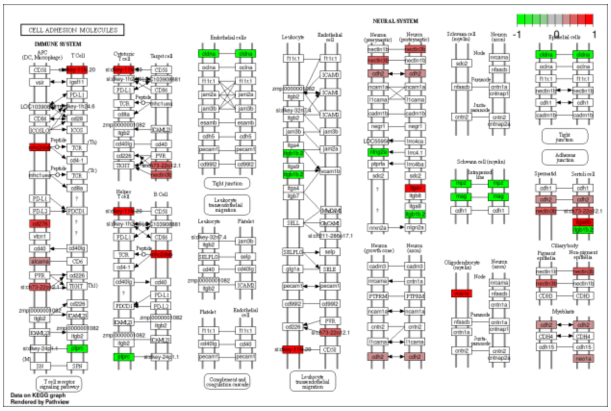

CELL ADHESION MOLECULES
IMMUNE SYSTEM
NEURAL SYSTEM
-1
0
1
Data on KEGG graph
Rendered by Pathview

Figure S5

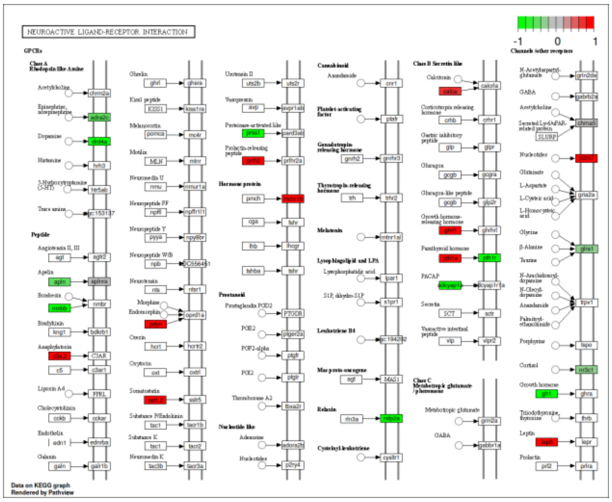
NEUROACTIVE LIGAND-RECEPTOR INTERACTION
-1 0 1
Data on KEGG graph
Rendered by Pathview

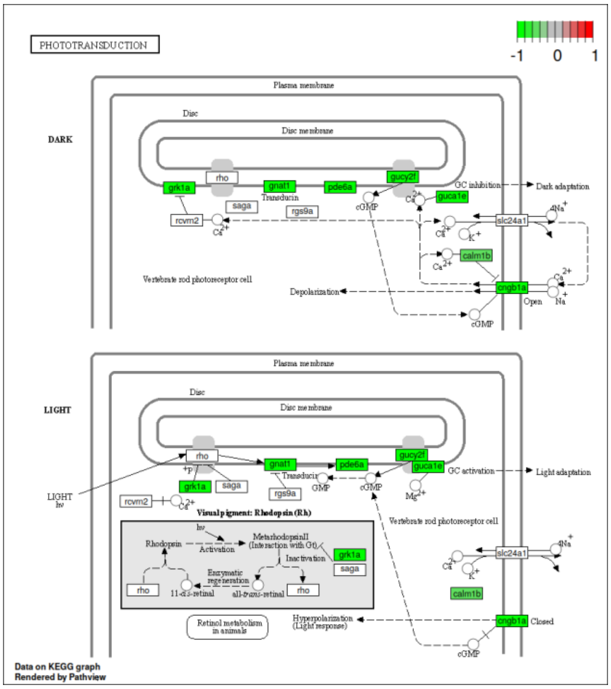
PHOTOTRANSDUCTION
-1 0 1
DARK
LIGHT
Plasma membrane
Disc
Disc membrane
Vertebrate rod photoreceptor cell
Dark adaptation
Light adaptation
Depolarization
Visual pigment: Rhodopsin (Rh)
Retinol metabolism in animals
Data on KEGG graph
Rendered by Pathview

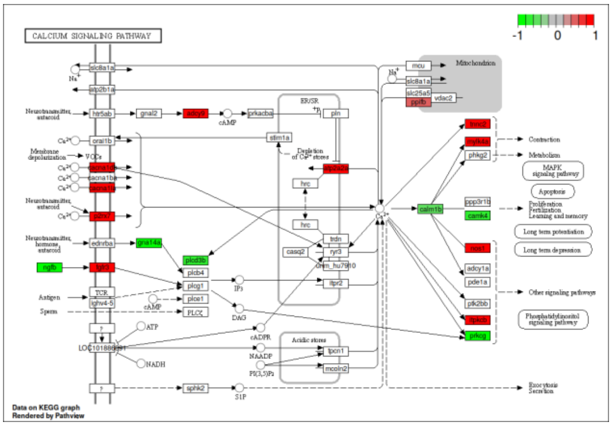
CALCIUM SIGNALING PATHWAY
-1 0 1
Mitochondrion
Data on KEGG graph
Rendered by Pathview

K

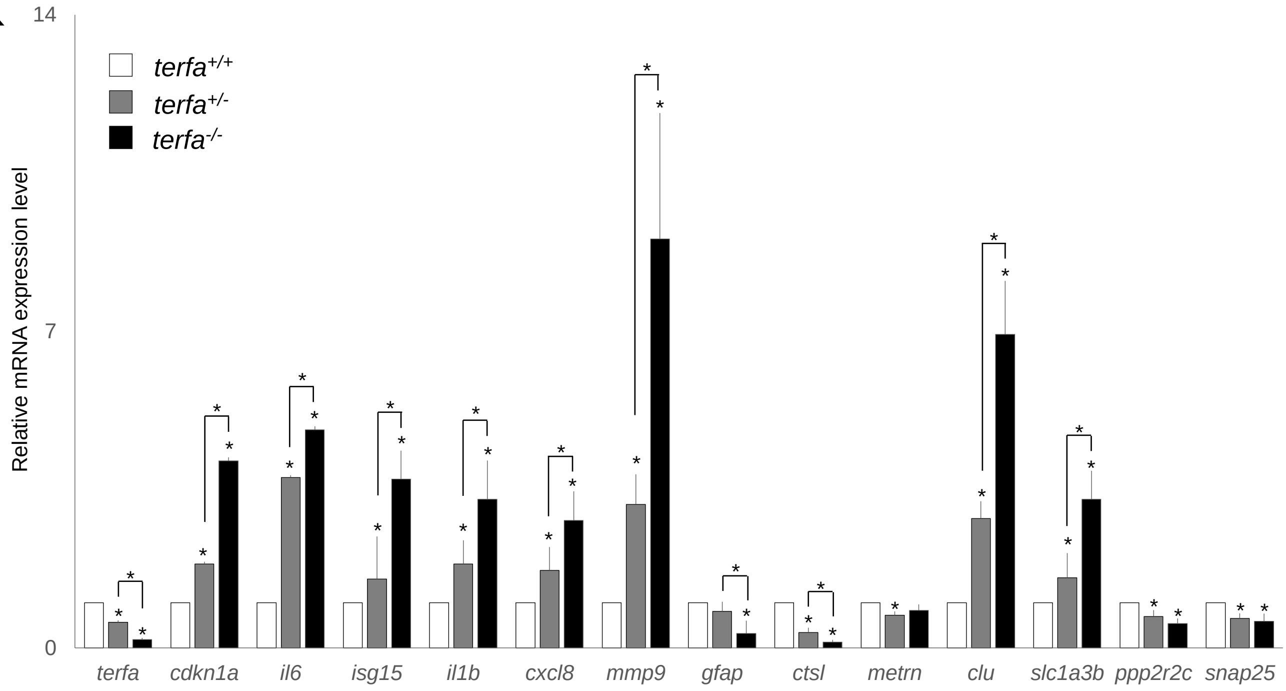
14
7
0
Relative mRNA expression level
terfa+/+
terfa+/-
terfa-/-
terfa
cdkn1a
il6
isg15
il1b
cxcl8
mmp9
gfap
ctsl
metrn
clu
slc1a3b
ppp2r2c
snap25

Figure S5

L

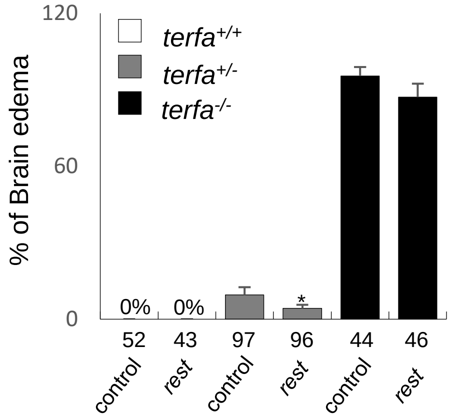

120
60
0
% of Brain edema
terfa+/+
terfa+/-
terfa-/-
0%
0%
*
52
43
97
96
44
46
control
rest
control
rest
control
rest

M

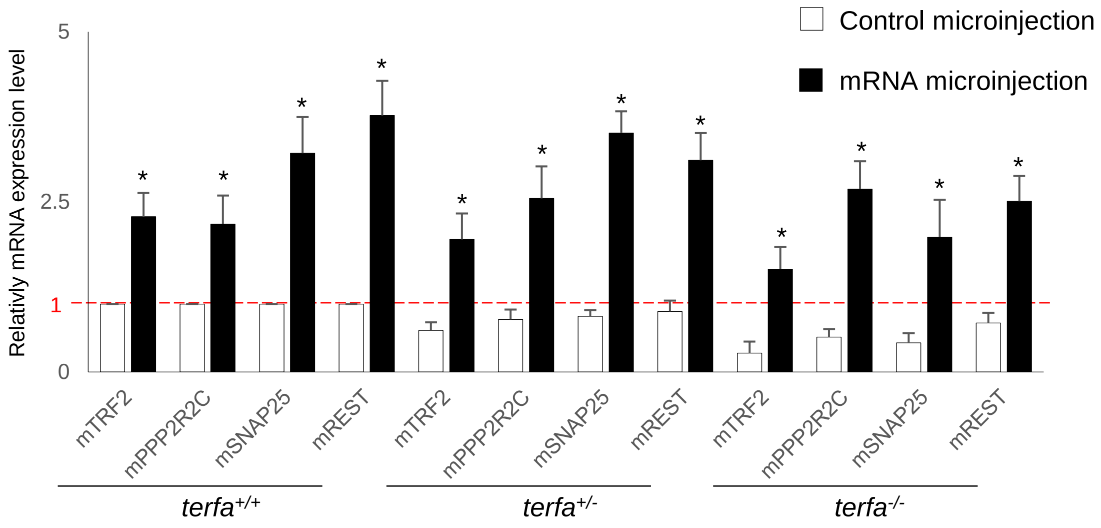

5
2.5
1
0
Relativly mRNA expression level
Control microinjection
mRNA microinjection
mTRF2
mPPP2R2C
mSNAP25
mREST
mTRF2
mPPP2R2C
mSNAP25
mREST
mTRF2
mPPP2R2C
mSNAP25
mREST
terfa+/+
terfa+/-
terfa-/-

Figure S6

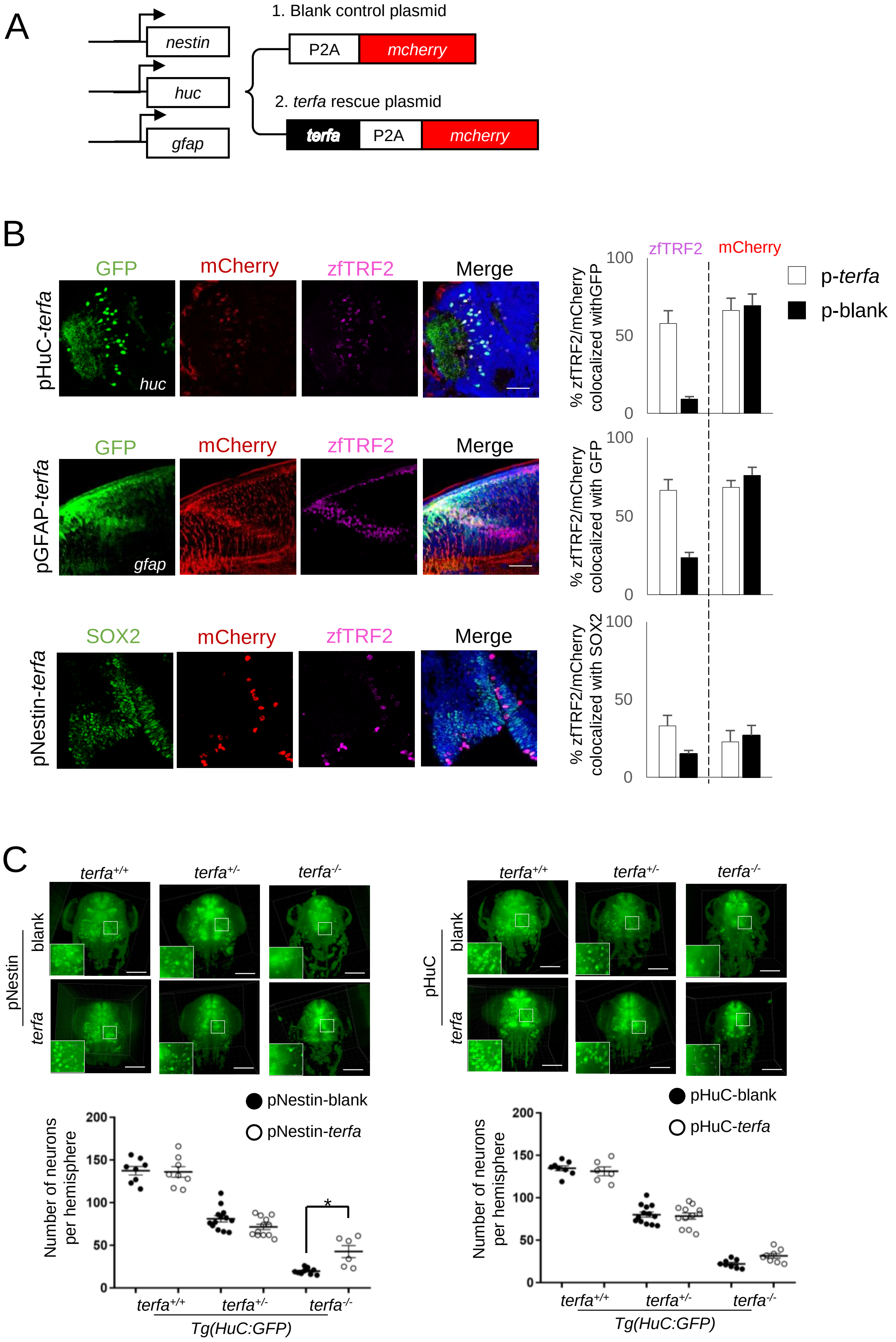
A
nestin
huc
gfap
1. Blank control plasmid
P2A
mcherry
2. terfa rescue plasmid
terfa
P2A
mcherry
B
GFP
mCherry
zfTRF2
Merge
pHuC-terfa
huc
pGFAP-terfa
gfap
SOX2
pNestin-terfa
zfTRF2
mCherry
p-terfa
p-blank
% zfTRF2/mCherry colocalized withGFP
% zfTRF2/mCherry colocalized with GFP
% zfTRF2/mCherry colocalized with SOX2
100
50
0
C
terfa+/+
terfa+/-
terfa-/-
pNestin
blank
terfa
pHuC
pNestin-blank
pNestin-terfa
pHuC-blank
pHuC-terfa
Number of neurons per hemisphere
200
150
100
50
0
*
Tg(HuC:GFP)

Figure S6

D

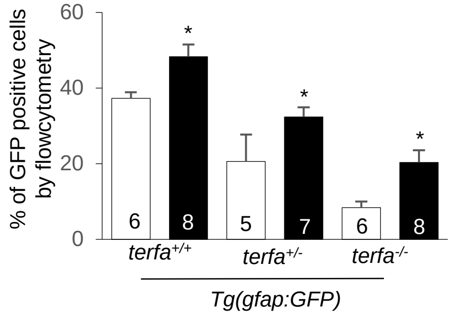

E

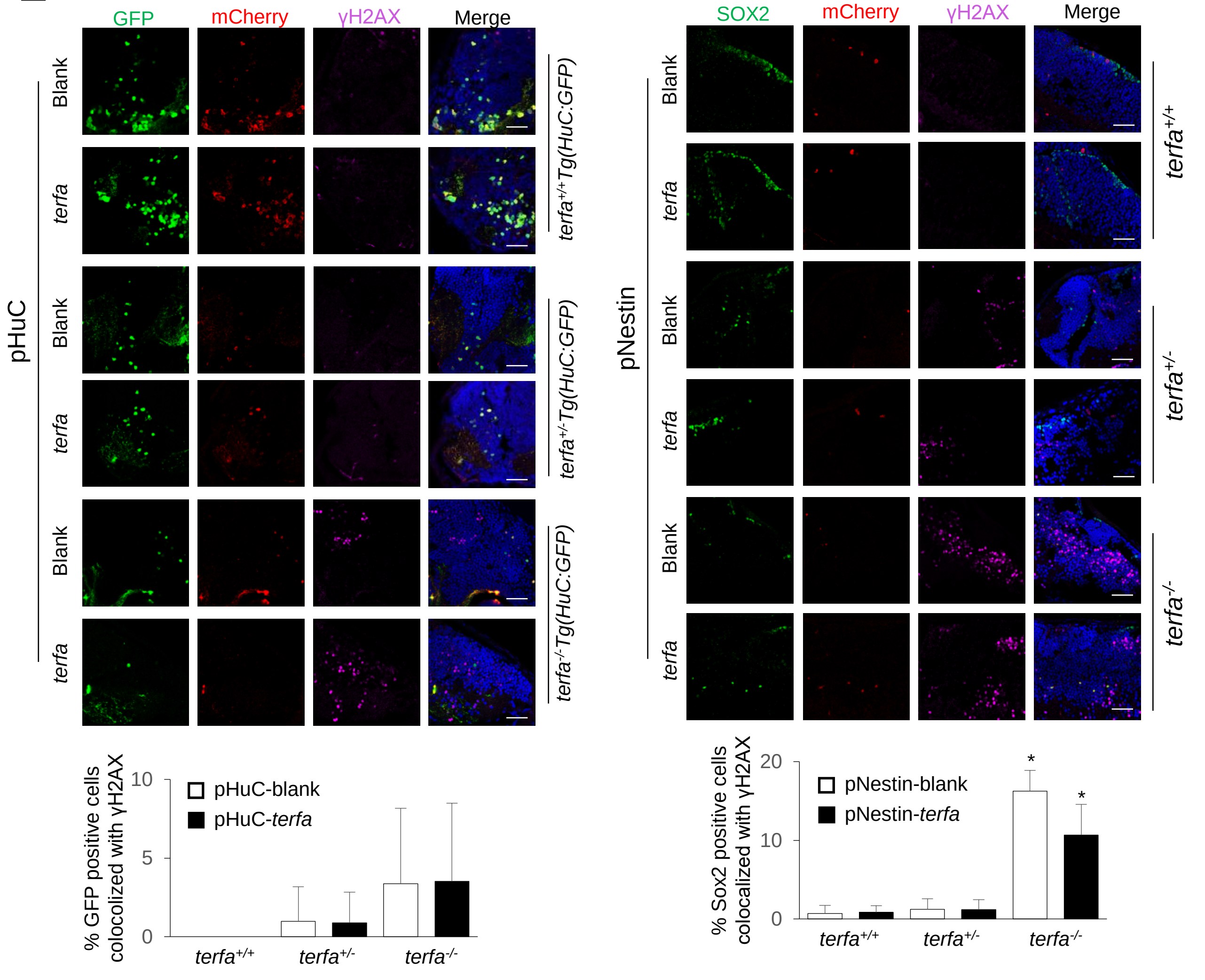

F

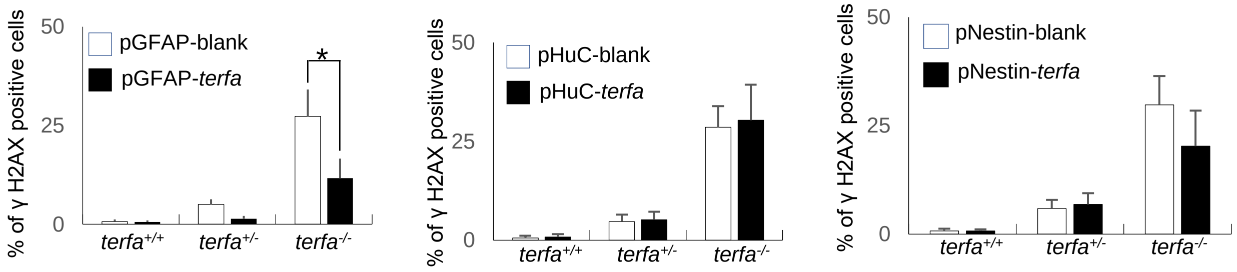

Figure S6

G

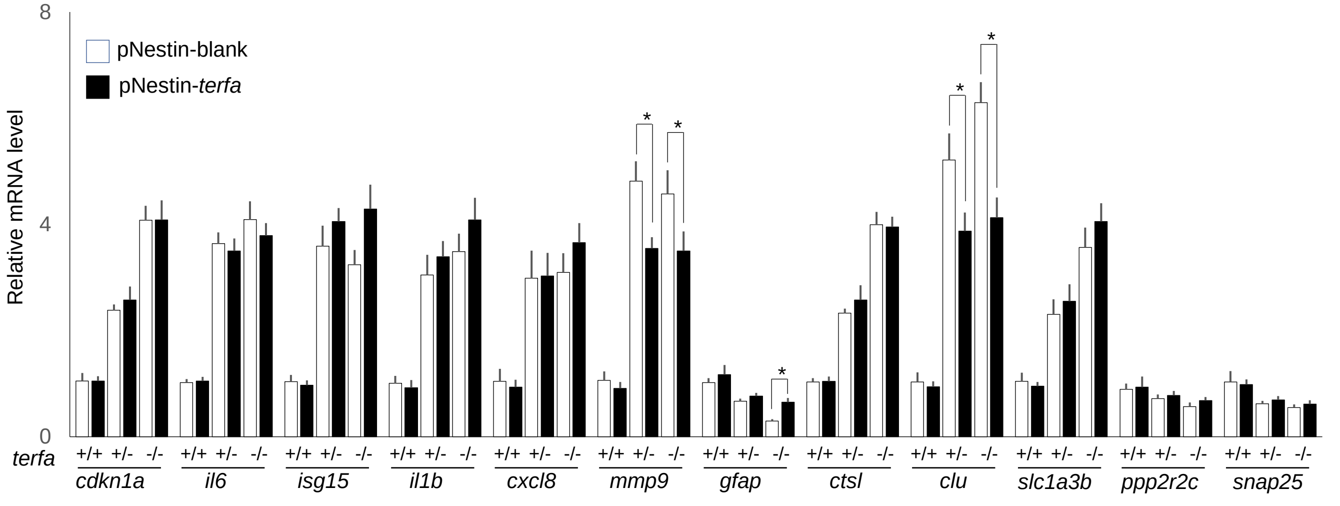

pNestin-blank
pNestin-terfa
Relative mRNA level
8
4
0
terfa
+/+ +/- -/-
cdkn1a
il6
isg15
il1b
cxcl8
mmp9
gfap
ctsl
clu
slc1a3b
ppp2r2c
snap25

H

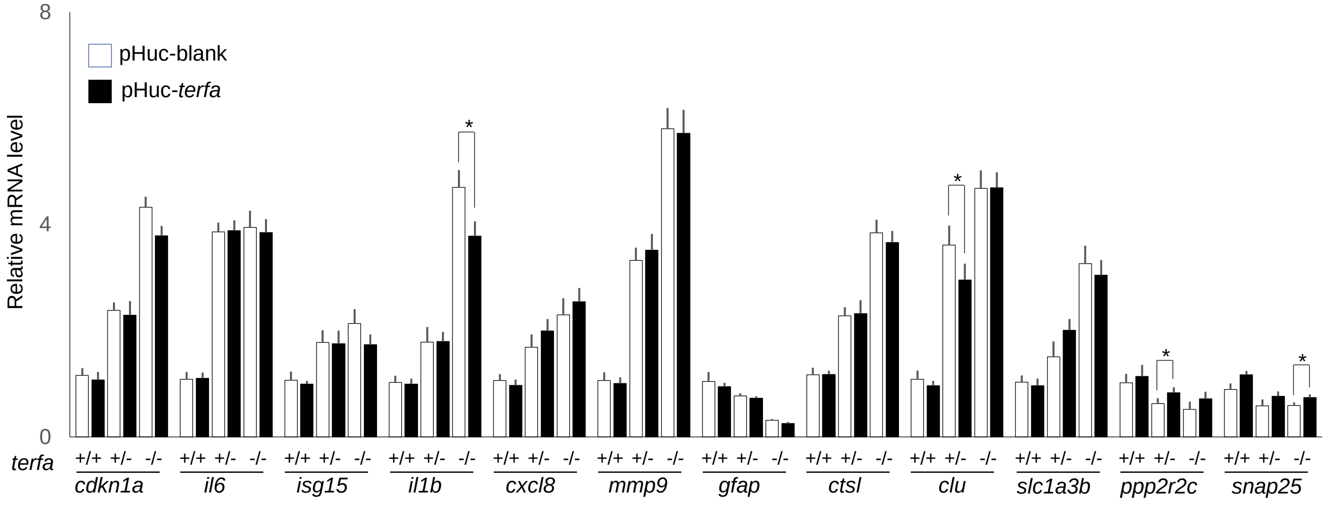

pHuc-blank
pHuc-terfa
Relative mRNA level
8
4
0
terfa
+/+ +/- -/-
cdkn1a
il6
isg15
il1b
cxcl8
mmp9
gfap
ctsl
clu
slc1a3b
ppp2r2c
snap25

Figure S6

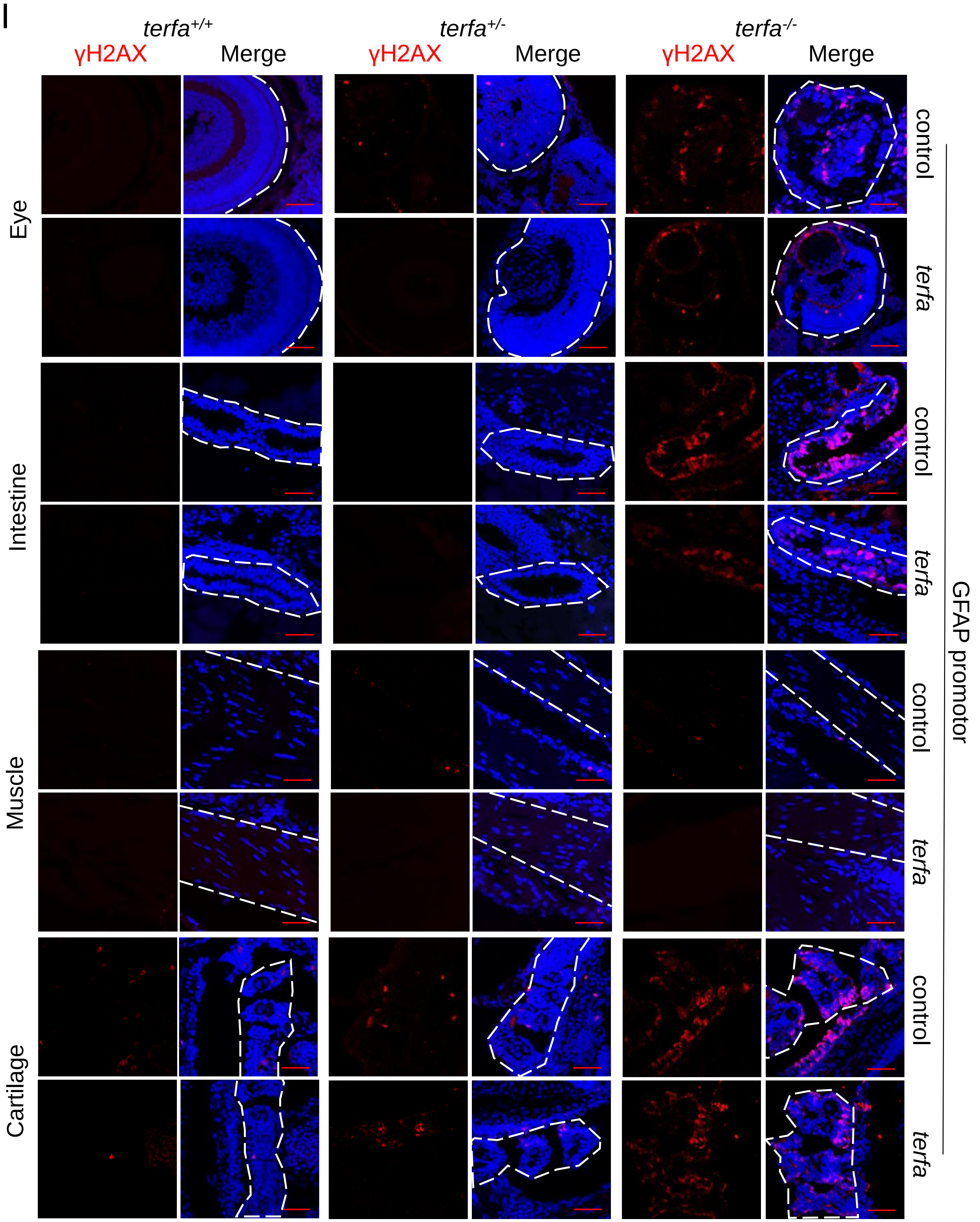
I
terfa+/+
γH2AX
Merge
terfa+/-
γH2AX
Merge
terfa-/-
γH2AX
Merge
Eye
Intestine
Muscle
Cartilage
control
terfa
control
terfa
control
terfa
control
terfa
GFAP promotor

Figure S6

J

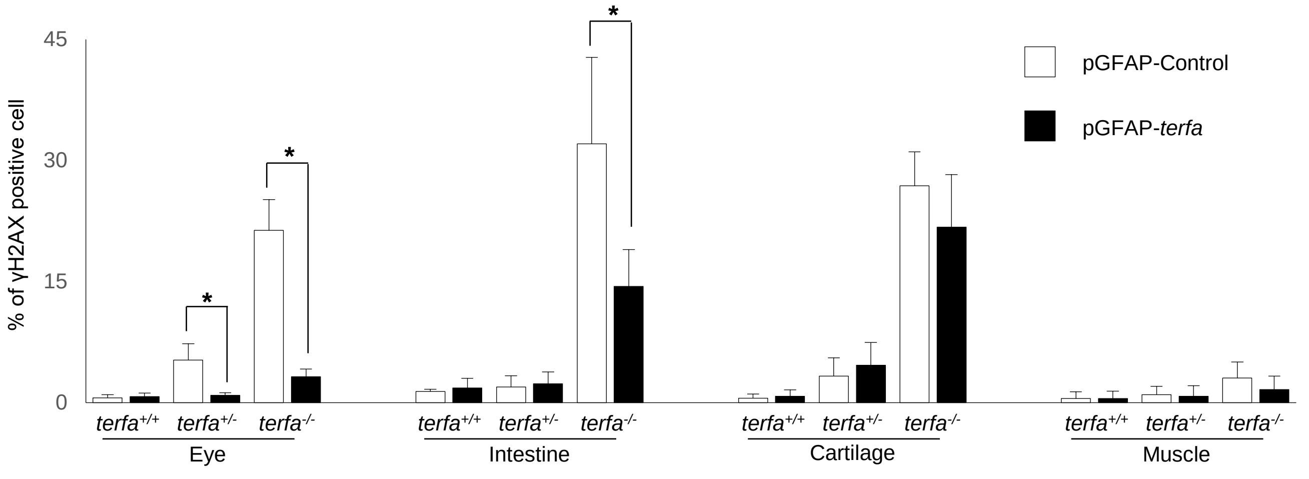
45
30
15
0
% of γH2AX positive cell
pGFAP-Control
pGFAP-terfa
*
*
*
terfa+/+ terfa+/- terfa-/-
Eye
terfa+/+ terfa+/- terfa-/-
Intestine
terfa+/+ terfa+/- terfa-/-
Cartilage
terfa+/+ terfa+/- terfa-/-
Muscle

K

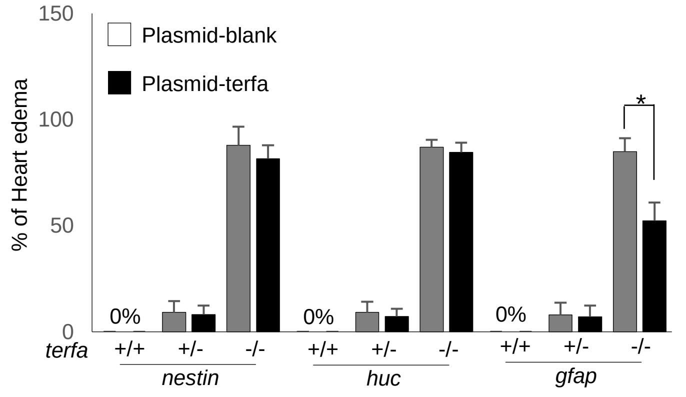
150
100
50
0
% of Heart edema
Plasmid-blank
Plasmid-terfa
*
0%
0%
0%
terfa
+/+ +/- -/-
nestin
+/+ +/- -/-
huc
+/+ +/- -/-
gfap

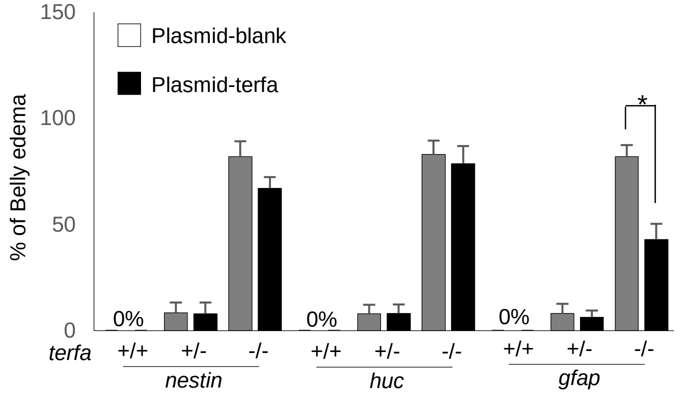
150
100
50
0
% of Belly edema
Plasmid-blank
Plasmid-terfa
*
0%
0%
0%
terfa
+/+ +/- -/-
nestin
+/+ +/- -/-
huc
+/+ +/- -/-
gfap

Figure S1 zfTRF2 downregulation leads to genome-wide DNA damage response with no telomere-specificity in ZF4 and ZFL cell lines

(A) The full western blot image of ZF4 cell extracts shown in **Figure 1A** after *terfa* knockdown with siRNA. A quantification of the zfTRF2/GAPDH ratio for each blot is shown. Noteworthy, different panels of molecular weight markers give slightly different apparent molecular weights for the band we interpret as zfTRF2. The reason of this discrepancy is unknown but the presence of one major band in the 60-75 kDa range together with the fact that the intensity of this band specifically decreases upon *terfa*-knock-down clearly assign this band to zfTRF2. **(B)** Representative images of immunofluorescence of PNA probes(red) for telomeres(top), pericentromeres (bottom) colocalizing with zfTRF2 (green) in ZFL fish cells. The quantification was shown as the percentage of both PNA signals colocalizing with zfTRF2, and the percentages of zfTRF2 signals colocalizing with PNA. Scale bars, 7 μm. **(C)** Representative images of immunofluorescence of PNA probes(red) for telomeres(top), pericentromeres(middle) and centromeres(bottom) colocalizing with zfTRF2(green) in ZF4 fish cells classified by Edu+ and Edu-. Scale bars, 7 μm. The quantification was shown in **(Figure 1C)**. **(D-E)** Representative images of immunofluorescence of PNA probe(red) for telomeres(top), pericentromeres(middle) and centromeres(bottom) colocalizing with zfTRF2(green) in ZFL fish cells. The quantification of both PNA colocalized with zfTRF2, and zfTRF2 colocalized with PNA were shown classified by Edu+ and Edu-cells. Scale bars, 7 μm. **(F)** The efficiencies of shelterin gene siRNA knockdowns in ZF4 cells were calculated using RT-qPCR. All data are shown as the mean ± SEM of three biological replicates. For immunofluorescence and confocal images, at least 30 nuclei were taken and counted in each sample for each biological replicate. Statistical analyses were performed using unpaired two-sided *t* tests (*$P < 0.05$, **$P < 0.01$, ***$P < 0.001$). Asterisks directly above columns indicate a significant difference between the indicated treatment and the control group (left). Asterisks above two columns indicate a significant difference between the two columns.

Figure S2 The validation of overexpression of zfTRF2 and knockdown of hTRF1 and hTRF2 in 293T cells

(A) The immunoblot assays were performed with antibodies against proteins extracted from human 293T cells overexpressing exogeneous zfTRF2. Human 293T cells were subjected to lentiviral transduction with pWPIR vectors containing *terfa-Myc*. The antibodies used are indicated below each blot. **(B)** RT-qPCR of human *TERF1* and *TERF2* in 293T cells downregulated by siRNA. Data are shown as the mean ± SEM of three biological replicates. Statistical analyses were performed using unpaired two-sided *t* tests, and survival curves were plotted using a log-rank test (*$P < 0.05$).

Figure S3 The Schematic diagram of the construction of *terfa*-mutated zebrafishes and its validation

(A) Schematic diagram showing the deletion of the zebrafish *terfa* gene using the CRISPR-Cas9 system. The diagram shows the sgRNA design and construction of *terfa*-mutated zebrafishes. Yellow and red boxes indicate the targeted exon and TRFH domain, respectively. The red dotted line indicates the nucleotide deletion in *terfa*. **(B)** Schematic diagram (top) showing primer design for the amplification of

terfa mRNA. Primer-1 was designed to target the mutation site, and primer-2 and primer-3 were designed to target the regions before and after the mutation site, respectively. The *terfa* mRNA in $terfa^{+/+}$, $terfa^{+/–}$, and $terfa^{–/–}$ embryos at 3 dpf and adult brains at 6 mpf was quantified (bottom) using three primer pairs. **(C)** The full western blot images of **Figure 3A**. **(D)** Representative photos of defective pigmentation on $terfa^{+/-}$ (bottom) compare to $terfa^{+/+}$ (top) at 6mpf. Data are shown as the mean ± SEM of three biological replicates. The number inside or below each column indicates the number of embryos detected for each condition. Statistical analyses were performed using unpaired two-sided *t* tests, and survival curves were plotted using a log-rank test (*$P < 0.05$).

Figure S4 Zebrafishes lacking zfTRF2 exhibit subsequently abnormal phenotype and DDR in multiple organs during development, but no obvious telomere dysfunction

(A) Representative images of the indicated abnormal phenotypes in *terfa*-compromised embryos from 2–4 dpf. Scale bar, 0.6 mm. The quantification was shown in (**Figure 4A**). **(B)** Percentages of embryos exhibiting brain edema phenotypes at 3 dpf in *terfa*-compromised embryos following microinjection with *terfa*-mRNA and $terfa^{▲telobox}$-mRNA. **(C-D)** Representative images (right) and quantitative results (left) from immunohistochemical assay showing the percentages of $terfa^{+/+}$, $terfa^{+/–}$, and $terfa^{–/–}$ embryos exhibiting γH2AX (**C**) and PCNA (**D**) signals in the different tissues (from left to right: brain, eye, intestine, muscle) at 3 dpf. scale bars, 30 μm. **(E)** Representative confocal section images showing TUNEL foci colocalized with GFP-positive cells (green) in $terfa^{+/+}$, $terfa^{+/–}$ or $terfa^{–/–}$ embryos at 3 dpf (as described in **Figure 4E** right). **(F)** Representative delta-vision microscope images and quantitative results from PNA-FISH assays showing the colocalization of telomeric PNA probes and γH2AX foci, and their respective percentages (scale bars, 3 μm). **(G)** Representative images of Southern-bolt of telomere length in *terfa*-compromised 3dpf embryos. **(H)** Representative images and quantification of metaphase spreads visualized using PNA-FISH. Spreads were acquired using fluorescence microscopy of embryos stained with a telomeric PNA probe (TTAGGG, green) at 1 dpf. Strains were generated by intercrossing $terfa^{+/–}$ fish. The number at each symbol indicates the number of chromosomes analyzed for each condition. Solid lines highlight chromosome abnormalities, including multiple telomere signals (MTS), telomere loss (TL), and telomere fusion (TF). Scale bars, 10 μm. For all FISH or IF experiments, approximately 30 nuclei were analyzed per replicate. All data are shown as the mean ± SEM of three biological replicates. Statistical analyses were performed using unpaired two-sided *t* tests, and survival curves were plotted using the log-rank test (*$P < 0.05$). Asterisks directly above columns indicate a significant difference between the indicated treatment and the control group (left). Asterisks above two columns indicate a significant difference between the two columns.

Figure S5 The embryonic neurodevelopmental failure results from both ATM activation and transcriptional dysregulation.

(A) Schematic diagram showing the deletion of the zebrafish *atm* gene using the CRISPR-Cas9 system. The diagram shows the sgRNA design(red) and construction of *atm*-mutated zebrafishes. 11bps(green) were inserted which led to the stop codon at the red box and deletion of *atm* expression. **(B)** Representative confocal section image and quantification of immunofluorescence of γH2AX foci (red) in

the brains of *atm*-compromised embryos treated with DMSO or VP16 (10ug/ml). Scale bars, 30 µm. **(C)** Quantification of *terfa*-compromised embryo phenotypes (brain edema, heart edema and belly edema) treated with *ATM* and *ATR* inhibitors (KU55933, VE821, Caffeine). **(D)** representative images (left) and quantification of three-dimensional (3-D) computationally-reconstructed brain light-sheet of neurons in *terfa*-compromised *Tg(huc:GFP);atm*$^{-/-}$ living embryos at 3 dpf. Green signals indicate neurons of *terfa*-compromised *Tg(huc:GFP)* embryos at 3 dpf. The number of GFP-positive cells in whole brains were calculated using Imaris imaging processing software (Bitplane). Scale bars, 150 µm. **(E)** Representative (left) and quantification (right) of confocal section images of TUNEL foci (red) in the brains of *terfa*-compromised *p53*$^{-/-}$ or *atm*$^{-/-}$ embryos at 3 dpf (scale bars, 30 µm). **(F)** Left: representative light microscopy images showing SA-β-gal staining in brains of *terfa*-compromised *p53*$^{-/-}$ or *atm*$^{-/-}$ embryos at 3 dpf (scale bars, 250 µm). Right: number of foci with SA-β-gal in brains. **(G)** PCA analyses of RNA sequencing samples for 72h *terfa*-compromised embryos (WT, *terfa*$^{+/-}$, *terfa*$^{-/-}$). **(H)** Venn diagram of differentiated genes between WT vs *terfa*$^{+/-}$ and WT vs *terfa*$^{-/-}$ in RNA sequencing. **(I)** Volcano diagram of differentiated genes for WT vs *terfa*$^{+/-}$ and WT vs *terfa*$^{-/-}$ groups in RNA sequencing. **(J)** Selected KEGG pathways affected according to the RNA-seq results in 72h terfa-compromised embryos compared with WT. **(K)** Transcript levels of indicated genes in *terfa*-compromised embryos (*terfa*$^{+/+}$, *terfa*$^{+/-}$ and *terfa*$^{-/-}$) at 3 dpf as measured by RT-qPCR. **(L)** The percentages of *terfa*-compromised embryos exhibiting brain edema phenotypes at 3 dpf. Embryos were microinjected with the indicated mRNAs. **(M)** mRNA levels of indicated genes in *terfa*-compromised embryos microinjected with the indicated mRNAs at 3 dpf, as measured by RT-qPCR. The relative mRNA levels of each gene were calculated based on the level in the corresponding *terfa*$^{+/+}$ control group. All data are shown as the mean ± SEM of three biological replicates. Statistical analyses were performed using unpaired two-sided *t* tests, and survival curves were plotted using the log-rank test (*$P < 0.05$). The number in each column indicates the number of embryos assayed for each condition.

Figure S6 zfTRF2 plays a specific role in GFAP-positive cells during neurodevelopment

(A) Schematic diagram showing plasmid construction for zfTRF2 joint with mCherry by P2A and specifically overexpress zfTRF2 in different neural cells meditated by promotors (*nestin*: progenitor cell, *huc*: neurons, *gfap*: glia cell). **(B)** Representative confocal section images (left) showing the cellular-specific expression of exogeneous zfTRF2 (magenta) foci or mCherry signals (see the scheme of vector construction in **Figure S6A**); zfTRF2 expression was indicated by GFP in *Tg(huc:GFP)/Tg(gfap:GFP)* embryos or by the progenitor cell-specific marker SOX2 (green) after microinjection with vectors for *terfa* expression driven by neural-specific promoters. The quantification of colocalization (right) between zfTRF2 or mCherry with neuro-markers under the condition described above. scale bars, 30µm. **(C)** Top: representative three-dimensional (3-D) computationally-reconstructed brain images of living embryos. Images were taken via light-sheet microscopy. Green signals indicate neurons of *terfa*-compromised *Tg(huc:GFP)* embryos at 3 dpf after microinjection with the plasmids described in **Figure S6A**. Bottom: GFP-positive cells in whole brains were quantified using Imaris software (Bitplane). **(D)** The percentages of GFP-positive cells (glial cells) in *terfa*-compromised *Tg(gfap:GFP)* embryo brains expressing zfTRF2 with the GFAP promoter at 3 dpf, as measured by flow cytometry. **(E)** Representative confocal section

images (top) and quantification (bottom) showing γH2AX foci colocalized with GFP-positive or mCherry-positive cells in the brains of *terfa*-compromised embryos at 3 dpf, following microinjection of the plasmids described in Figure S6A. Left: The percentages of GFP-positive cells colocalizing with γH2AX foci are shown in *terfa*-compromised *Tg(huc:GFP)* embryos after plasmid microinjection. Right: results of the same experiments with *terfa*-compromised embryos after microinjection with plasmids that elicit TRF2 expression driven by the Nestin promoter. The progenitor cells were marked by anti-SOX2 antibodies and the percentages SOX2 positive cells that colocalized with γH2AX foci are also shown. The *terfa*-compromised *Tg(gfap:GFP)* embryos and percentages of GFP-positive cells that colocalized with γH2AX foci are shown in Figure 6D. Scale bars, 30μm. **(F)** Quantifications of the representative confocal section images in Figure 6D. Data are shown as the percentages of cells colocalizing with γH2AX foci in the brain of *terfa*-compromised *Tg(gfap:GFP)* embryos at 3 dpf, following a microinjection of plasmid expressing zfTRF2 meditate by GFAP, HuC and Nestin promotor independently, scale bars, 30μm. **(G-H)** Transcript levels of candidate genes from RNA-seq in $terfa^{+/+}$, $terfa^{+/-}$, and $terfa^{-/-}$ embryos at 3 dpf. Embryos were microinjected with the plasmids described in Supplementary Fig. 6a. The mRNA transcript levels were measured using RT-qPCR. **(I-J)** Representative confocal section images **(I)** and quantification **(J)** showing γH2AX positive cells in eye, intestine, muscle and cartilage tissues of *terfa*-compromised embryos at 3 dpf, following microinjection plasmid of expression zfTRF2 meditate by *gfap* promotor. The quantification **(J)** shows that percentages of γH2AX positive cells in embryos, scale bars, 30μm. **(K)** Percentages of embryos exhibiting heart(top) and belly(bottom) edema phenotypes at 3 dpf in *terfa*-compromised embryos following microinjection with the plasmids described in **(Figure S6A)**. All data are shown as the mean ± SEM of three biological replicates. The number below each column indicates the number of embryos detected for each condition. Statistical analysis was performed using the unpaired two-sided *t* test, and survival curves were plotted using the log-rank test (*$P < 0.05$). Asterisks directly above columns indicate a significant difference between the indicated treatment and the control group (left). Asterisks above two columns indicate a significant difference between the two columns.
