# Supplementary material for: The non-telomeric evolutionary trajectory of TRF2 in zebrafish reveals its specific roles in neurodevelopment and aging
Source: Nucleic Acids Res. 2022 Feb 12;50(4):2081–95. doi: 10.1093/nar/gkac065 (PMC8887477; doi:10.1093/nar/gkac065)
Supplement: gkac065_Supplemental_Files [file gkac065_supplemental_files.zip › sup antibody primer siRNA.pdf]

REAGENT OR RESOURCE	SOURCE	IDENTIFIER
Mouse monoclonal anti-TRF2	Novus Biologicals	NB100-56506; RRID: AB_839054
Rabbit polyclonal anti-TRF2	Novus Biologicals	NB110-57130; RRID: AB_844199
Goat polyclonal anti-SOX2	R＆D	AF2018
Rabbit polyclonal anti-SOX2	GeneTex	GTX124477
Mouse monoclonal Anti-Myc-tag	ProteinTech	60003-2
Rabbit polyclonal anti-γH2AX	GeneTex	GTX127342
Rabbit polyclonal anti-PCNA	GeneTex	GTX124496
Rabbit polyclonal anti-TERFA	Self-made	Self-made
Mouse polyclonal anti-GAPDH	Cell Signaling Technology	CST97166
Anti-mouse Alexa Flour 647	Invitrogen	A31571
Anti-rabbit Alexa Flour 633	Invitrogen	A21070
Anti-goat Alexa Flour 633	Invitrogen	A21082
Anti-mouse Alexa Flour 555	Invitrogen	A21422
Anti-rabbit Alexa Flour 555	Invitrogen	A31572
Anti-mouse Alexa Flour 488	Invitrogen	A21202
Anti-rabbit Alexa Flour 488	Invitrogen	A21206
HRP goat anti-mouse IgG	Abcam	ab6728
HRP goat anti-rabbit IgG	Abcam	ab6721

PRIMER NAME	USAGE	SEQUENCE
RV-M	Plasmid construction	AGCGGATAACAATTTCACACAGGA
T7-cr-fwd	Plasmid construction	GAAATTAATACGACTCACTATA
tracr-rev	Plasmid construction	AAAAAAAGCACCGACTCGGTGCCAC
zf-*terfa*-gRNA-S	Plasmid construction	ataGTGTGGTGGTCAGGCCGGgt
zf-*terfa*-gRNA-AS	Plasmid construction	taaaacCGGCCTGACCACCACAC
zf-*atm*-gRNA-S	Plasmid construction	ataGCGTCTTCGGAGCTCAACGGgt
zf-*atm*-gRNA-AS	Plasmid construction	taaaacCCGTTGAGCTCCGAAGACGC
zf-*terfa*-test	Mutation identify	F: TTAACCCGCGGTTATCTTCAG<br>R: CGTCTCCGACATTCACTCAC
zf-*atm*-test	Mutation identify	F: GTGCGGGATGAGTGATGTTTA<br>R: ACAGATGCCACCGAAATATAGTG
zf-*p53*-test	Mutation identify	F: ATTGCCAGAGTATGTGTCTGT<br>R: CACAAGAGGAGGAATCAAATATGC
zf-ppp2r2c	QPCR	F: CATCTTCCAGAGAGAGACAGAG<br>R: CGTTAGTGGAGAGGAGAAAGT
zf-arf-gap3	QPCR	F: CTGTGTGTGCTGAGTGAAGA<br>R: GTGCAGCGAACGCTAAGTA
zf-pacsin2	QPCR	F: CGCCTACCACAAGCAGATAA<br>R: GGTTGCTGTTGTCCAGTTTG
zf-ehd4	QPCR	F: TGAAGCCATACGAGCTTTCC<br>R: CCCAGAAAGAGCCCAGATAAA
zf-terfa-primer1(hang)	QPCR	F: GAGTGTGGTGGTCAGGCCGGT<br>R: GCTCCTGATACACACCATCAC
zf-terfa-primer2	QPCR	F: TTACACGGCCTTCACACACT<br>R: GACAGAAACTGCATGAGGCG
zf-terfa-primer3	QPCR	F: CAGGATCAACAACGGGGACAA<br>R: CGGATCAGGTTCAGCAGCA
zf-cbx7	QPCR	F: ACGTCTGAGGAAGAGTGGGA<br>R: CCTCTCTCCTGACGTGCTTG
zf-clu	QPCR	F: CACAAGAGGATGCTGTCGGA<br>R: GCAGGGCTTACACTCTTCCC
zf-ctsl	QPCR	F: GCCTCTATGTTCGGACAGGCTA<br>R: CGTGACGAGCAAAGCAAACAT
zf-cxcl8	QPCR	F: TTGAAACAGAAAGCCGACGC<br>R: CCAGTTGTCATCAAGGTGGC
zf-cxcl12	QPCR	F: CATGCACCGATTTCCAACGC<br>R: GTTGGGTGTGTGGAGGAACT
zf-gcm2	QPCR	F: CACCTGTACGGACTCGCATAA<br>R: CAAGGCAAAATTCAGCTCGGTA
zf-gfap	QPCR	F: CAAGATGTGGATGAAGCGGC<br>R: AGCGGTCAAGTCTGGCTTAG

PRIMER NAME	USAGE	SEQUENCE
zf-manf	QPCR	F: GAGTCCTGCAAAGGATGCGT<br>R: CCGCTGTTAGGTGCTCAGTC
zf-metrn	QPCR	F: TCACAGGACGGACATTAGCG<br>R: TCACGAGGACTTTGGATGCG
zf-metrnl	QPCR	F: GAAGCTGTGCCTCAAAGGGA<br>R: GGTTGGCAGGGAGCAGATAA
zf-mmp9	QPCR	F: GTTTCTGGTTCTGGGCACCT<br>R: TAGCATTGGAGATGACCGCC
zf-pcdh8	QPCR	F: GATCACTCGCCGCAATTCAC<br>R: CTCTCCATCCTCACGAGTGC
zf-rpe65	QPCR	F: ATAAGTTCAGGACCTCGGCG<br>R: TCGCCAGCCACAGATAGTTG
zf-slc1a2a	QPCR	F: GTCACGGTCATCGTTGGACT<br>R: GAAGCTGTTCCTAAGGCGGT
zf-slc1a2b	QPCR	F: GTCACGGTCATCGTTGGACT<br>R: GAAGCTGTTCCTAAGGCGGT
zf-slc1a3a	QPCR	F: CAGGAGAGCGAGAGCGACAA<br>R: CTCAGCTCGGACTTCACACATT
zf-slc1a3b	QPCR	F: AGGCCATCATGCGACTTGTT<br>R: CCGTGTATCATGAGGCCGAT
zf-gli2	QPCR	F: TTTGGTGCTTCCCGATGACA<br>R: TGCGTTGCCCATAGAACTGT
zf-gmfb	QPCR	F: CACGGTAGAACTCCTGCACT<br>R: GAAGCGGAAATCACGCAACT
zf-hepacam	QPCR	F: TCACTGGAGAGCGCAACATC<br>R: ATGGCCTTTGTCCCTGTGTC
zf-hepacam	QPCR	F: GACTGTTCAGATCCGCTTGC<br>R: TTCAGGGCGATGATGACGTT
zf-isg15	QPCR	F: ATGATGTGGACGCCAACGAG<br>R: GTCCTGCAACTTCATGCCAG
zf-terfa	QPCR	F: GGGATTCAGGATAACTGGAGTGATGAG<br>R: CCAGTGTCCCACACCATAGC
zf-terf1	QPCR	F: ATGGAGACCATCGACACATTC<br>R: GGGTGGTGTCTCTTCAGTTT
zf-rap1	QPCR	F: AAACCTCCACGACTGATTCC<br>R: ATTGTCACAACCTTGATCTCCT
zf-pot1	QPCR	F: GACCTGGATGAGCATGAAGAA<br>R: CTCACACGTCTGCACTGTATAA
zf-tpp1	QPCR	F: AGTACTTCCTCTGGCTCAAATG<br>R: GGTCTCTGGCTGAGTGTAATG
zf-tin2	QPCR	F: GCTTCAGAGGTGCAGAGAAA<br>R: TGCCGGATGATTGGGTTATC
zf-β-actin	QPCR	F: CCGTATGCAGAAGGAAATCAC<br>R: GGTGGCAACAGTTCTGTTTAG

GENE NAME	SPECIES	SEQUENCE
siControl	human	UUCUCCGAACGUGUCACGUTT
siTRF2	human	CCCAAAGUACCCAAAGGCAdTdT
siTRF1	human	UGAUGAACGAAUUACACCCUUGGAA
si*terfa-1*	zebrafish	GCUGGAGCUUCGAUUUCUA
si*terfa-2*	zebrafish	GCAGUUUAUCAGAGCCGCA
si*terf1-1*	zebrafish	GCCAGAAACUGCAAAGAAA
si*terf1-2*	zebrafish	GGACUGAUGUAGAAGACAA
si*terf1-3*	zebrafish	GGAGAGUCCUAAAGAAACA
si*rap1-1*	zebrafish	CGAAAGAGUUUGAGGAUUC
si*rap1-2*	zebrafish	GGAAGAUCCUGUUGGACAA
si*rap1-3*	zebrafish	GCUCCAGAUUAGAGGAAGA
si*pot1-1*	zebrafish	CUAAAGACAUGGUGGGGAA
si*pot1-2*	zebrafish	GGGAACAGUAUAUGGACGA
si*tpp1-1*	zebrafish	AGAAGAGCCUGGUCUGUUA
si*tpp1-2*	zebrafish	CCAGAGGUAUUGUGUGGAA
si*tpp1-3*	zebrafish	GUGCAGAUAUCAAUGCUGA
si*tin2-1*	zebrafish	GCAGUGAAGUAGAGGAAGA
si*tin2-2*	zebrafish	AGAGAAAGGUAUUCAAUCA
si*tin2-3*	zebrafish	GGUCACAAGAUGAAGGAGA
