# Supplementary material for: The non-telomeric evolutionary trajectory of TRF2 in zebrafish reveals its specific roles in neurodevelopment and aging
Source: Nucleic Acids Res. 2022 Feb 12;50(4):2081–95. doi: 10.1093/nar/gkac065 (PMC8887477; doi:10.1093/nar/gkac065)
Supplement: gkac065_Supplemental_Files [file gkac065_supplemental_files.zip › Table-S1-S3-DEGs-for-RNA-seq-sig.pdf]

Table S1. DEGs of WT vs. *terfa+/-*

	genename	baseMean	log2FoldChange	lfcSE	stat	pvalue	padj
ENSDARG00000104049	crebl2	927.417584	-0.429568168	0.10560843	-4.06755558	4.75E-05	0.00083688
ENSDARG00000102128	eps8a	1322.23808	-0.438729499	0.11624651	-3.77413053	0.00016057	0.00231607
ENSDARG00000100660	si:ch73-252i11.1	152.523377	0.570119548	0.20309365	2.80717558	0.0049978	0.0339864
ENSDARG00000045914	si:ch211-51e12.7	6490.51387	0.453664663	0.13918191	3.25950875	0.00111605	0.01075997
ENSDARG00000093924	BX890562.2	33.0044547	2.373373315	0.42513476	5.58263758	2.37E-08	1.12E-06
ENSDARG00000101047	rtcb	962.088289	0.406054064	0.10913311	3.72072286	0.00019865	0.00275994
ENSDARG00000096118	ost4	605.32554	1.043544088	0.12622212	8.26752141	1.37E-16	2.81E-14
ENSDARG00000045900	agbl5	1311.83545	0.290894763	0.09952534	2.92282114	0.00346876	0.02589846
ENSDARG00000045898	si:ch211-152c2.3	1475.05923	-0.500987237	0.14727208	-3.4017802	0.00066948	0.00725056
ENSDARG00000099313	naga	1319.3782	1.138886441	0.16454666	6.92135864	4.47E-12	4.45E-10
ENSDARG00000063672	zmp:0000000711	658.145438	0.346313132	0.11014465	3.14416659	0.00166561	0.01472765
ENSDARG00000045885	scaf11	3144.04955	0.20039274	0.07552206	2.65343318	0.00796775	0.04802098
ENSDARG00000099129	phlda1	376.871249	0.741012496	0.13890614	5.33462754	9.57E-08	3.93E-06
ENSDARG00000004592	gpr22a	365.760061	-0.383675327	0.13035155	-2.94338917	0.0032464	0.02457043
ENSDARG00000060248	fgd4b	122.247184	-0.87986673	0.24091249	-3.65222548	0.00025998	0.00345178
ENSDARG00000017757	pik3cg	369.17256	-0.549572907	0.13622272	-4.03437046	5.47E-05	0.00094302
ENSDARG00000045863	asb15b	29.80429	-2.129876156	0.42293171	-5.03598126	4.75E-07	1.59E-05
ENSDARG00000090401	CABZ01020840.1	161.583987	1.386060128	0.27630488	5.0164156	5.26E-07	1.74E-05
ENSDARG00000063563	creb3l2	1631.00206	0.369392318	0.10711289	3.4486263	0.00056345	0.00633112
ENSDARG00000039346	ndufa5	1442.78213	-0.417205866	0.10084842	-4.13695996	3.52E-05	0.00064988
ENSDARG00000030694	atp6v1e1b	4222.31236	-0.427140153	0.10288013	-4.15182379	3.30E-05	0.00061271
ENSDARG00000096037	si:ch211-214j24.14	372.053147	-0.534898089	0.13276385	-4.02894385	5.60E-05	0.00096305
ENSDARG00000045850	ccdc167	351.651212	0.42491037	0.14724055	2.88582439	0.0039039	0.02825609
ENSDARG00000097353	BX324164.3	107.097156	0.965506922	0.29894827	3.22967893	0.00123929	0.01166961
ENSDARG00000045835	si:dkey-14d8.6	83.9457117	-3.064499236	0.78010455	-3.92831864	8.55E-05	0.00136466
ENSDARG00000045834	si:dkey-14d8.7	37.2808265	-2.274771835	0.55754501	-4.07997883	4.50E-05	0.00079905
ENSDARG00000054321	ngs	3951.49311	-0.569857247	0.19169044	-2.97279952	0.00295097	0.02291359
ENSDARG00000040610	si:ch73-352p4.8	604.898395	-0.440303116	0.12029494	-3.66019644	0.00025202	0.00335692
ENSDARG00000073858	pex26	283.487722	-0.5313295	0.17689703	-3.00360894	0.00266798	0.02125405
ENSDARG00000090468	ppp1r3aa	272.229773	0.646764505	0.15486124	4.17641302	2.96E-05	0.00055932
ENSDARG00000027799	ucmaa	96.7765317	-1.024809434	0.32217805	-3.18087912	0.00146829	0.01334344
ENSDARG00000078073	nudt5	593.743982	-0.360040052	0.13639675	-2.63965265	0.0082991	0.04948499
ENSDARG00000026052	ccdc3a	131.204152	0.670249088	0.20317088	3.29894273	0.0009705	0.00964482
ENSDARG00000076313	NA	14.3906414	-5.614915208	0.99274431	-5.65595304	1.55E-08	7.83E-07
ENSDARG00000074613	si:ch211-240l19.6	141.905209	-2.705542405	0.38231543	-7.07672822	1.48E-12	1.64E-10
ENSDARG00000039393	si:ch211-240l19.5	248.324007	-1.939793101	0.43731478	-4.43569075	9.18E-06	0.00020775
ENSDARG00000103537	ttll12	298.894612	0.733421019	0.2084229	3.51890797	0.00043333	0.00516563
ENSDARG00000102020	pnpla3	570.09879	0.750277117	0.20835572	3.60094316	0.00031706	0.00403083
ENSDARG00000070230	aldh1l2	1324.33422	0.65250799	0.18869574	3.45799006	0.00054422	0.00615967
ENSDARG00000077926	si:dkey-48p11.3	146.618363	-0.539329313	0.1980805	-2.72277847	0.00647354	0.04119303
ENSDARG00000039185	nfyba	2394.07062	0.335702525	0.12277667	2.73425332	0.00625219	0.04008208
ENSDARG00000058159	ipo8	1302.12985	0.680257034	0.22208547	3.06304155	0.002191	0.0182681
ENSDARG00000016538	zgc:55888	35.6448299	4.186031854	0.66208315	6.32251682	2.57E-10	1.92E-08
ENSDARG00000025859	lmf2b	1598.14759	-0.418398764	0.11006474	-3.80138786	0.00014389	0.00211492
ENSDARG00000045705	meig1	120.416212	0.578506744	0.1984899	2.91453993	0.00356213	0.02647632
ENSDARG00000094732	mical3b	964.010578	-0.508861002	0.13678479	-3.72015773	0.0001991	0.00276327
ENSDARG00000003570	hsp90b1	14389.6937	0.919029228	0.0845353	10.8715437	1.58E-27	8.70E-25
ENSDARG00000070126	si:dkey-22l11.6	26.0842711	-1.491955989	0.48846011	-3.05440699	0.00225506	0.01867872
ENSDARG00000018820	flncb	5050.76499	0.682757831	0.10881161	6.2746784	3.50E-10	2.51E-08
ENSDARG00000045758	cracr2ab	45.7268395	-1.16392931	0.32981981	-3.52898541	0.00041716	0.00500165
ENSDARG00000001781	tspan11	54.3309475	-0.830008254	0.27660044	-3.0007481	0.00269317	0.0213713
ENSDARG00000032765	net1	4814.98083	0.311673618	0.08867198	3.51490519	0.00043991	0.00523157
ENSDARG00000096099	BX465862.3	31.0577137	1.236948513	0.4631132	2.67094203	0.00756387	0.04620592
ENSDARG00000092083	BX899181.1	17.4338497	1.816453178	0.65672318	2.76593431	0.005676	0.03747062
ENSDARG00000045768	cry1a	3392.1309	0.231730392	0.08715625	2.65879249	0.00784212	0.04745707
ENSDARG00000032242	tnnt2c	1233.26113	0.574861161	0.16051617	3.58132876	0.00034185	0.00426542
ENSDARG00000058366	si:dkey-222f8.3	756.668633	-0.449147102	0.12565921	-3.57432697	0.00035113	0.00435062

Table S1. DEGs of WT vs. *terfa+/-*

ENSDARG00000007639	cnot4b	1096.759	0.431755926	0.09417058	4.584828	4.54E-06	0.00011337
ENSDARG00000058244	il17ra1a	183.187689	-0.583282868	0.18437368	-3.16359076	0.00155836	0.01396286
ENSDARG00000110404	AL954848.1	3.85850671	-5.542904504	1.60788656	-3.44732311	0.00056617	0.00635313
ENSDARG00000022165	mgst1.2	802.564994	0.471690435	0.1255216	3.7578428	0.00017138	0.00243833
ENSDARG00000045658	msrb3	922.825927	-0.442364437	0.1221471	-3.6215714	0.00029282	0.00377409
ENSDARG00000017841	cand1	4867.01825	0.217938542	0.07735466	2.81739388	0.00484151	0.03314122
ENSDARG00000045676	calua	2730.1959	0.376754103	0.1048513	3.59322286	0.00032661	0.00411662
ENSDARG00000069780	pus7l	155.62416	-0.718061466	0.20819824	-3.44893143	0.00056281	0.00632684
ENSDARG00000011661	twf1b	1106.03446	-0.282080849	0.09268679	-3.0433772	0.00233939	0.01923668
ENSDARG00000020086	nuak1a	937.096114	0.630004048	0.18416548	3.42085857	0.00062424	0.00688125
ENSDARG00000003811	plxnb2a	3373.21386	0.304059546	0.08981566	3.38537328	0.00071082	0.00760866
ENSDARG00000057334	cmasa	1242.09724	0.433734465	0.092467	4.6906947	2.72E-06	7.28E-05
ENSDARG00000045601	cax1	323.243683	0.625204591	0.22053899	2.83489368	0.00458409	0.03177291
ENSDARG00000094584	BX470240.1	88.5580758	3.614308982	0.4946435	7.3068968	2.73E-13	3.36E-11
ENSDARG00000034823	copg2	5778.04399	0.336348174	0.09391339	3.58147189	0.00034166	0.00426522
ENSDARG00000056938	kera	2242.78337	-0.949963081	0.1790517	-5.30552381	1.12E-07	4.53E-06
ENSDARG00000045580	lum	4305.40033	-0.542144582	0.08464861	-6.40464859	1.51E-10	1.16E-08
ENSDARG00000032653	sinhcaf	600.499538	-0.460267294	0.12517053	-3.67712178	0.00023588	0.00317423
ENSDARG00000027316	tcp11l2	2933.8873	0.351590388	0.09274558	3.79091253	0.00015009	0.00219706
ENSDARG00000029944	parpbp	248.448058	-0.530668579	0.19916886	-2.66441536	0.00771223	0.04691322
ENSDARG00000007175	recql	755.876636	0.328721111	0.1213173	2.709598	0.00673648	0.04242088
ENSDARG00000008867	rap1b	2677.74793	0.235394104	0.08927315	2.63678489	0.00836959	0.04979084
ENSDARG00000045548	lepb	47.7752906	4.642844296	0.87364515	5.31433651	1.07E-07	4.34E-06
ENSDARG00000006766	snd1	7692.78626	0.291573347	0.07750138	3.76216978	0.00016845	0.00241114
ENSDARG00000111326	BX323820.1	152.957151	-1.009204396	0.27225318	-3.70685991	0.00020984	0.00288638
ENSDARG00000104301	zgc:193726	57.5336452	-1.302642879	0.37454998	-3.47788795	0.00050538	0.00582348
ENSDARG00000045543	atp6v1f	1566.66625	-0.382096768	0.09145281	-4.17807554	2.94E-05	0.00055628
ENSDARG00000033443	mdm2	3617.08246	1.119310487	0.1364411	8.20361678	2.33E-16	4.71E-14
ENSDARG00000011769	cpm	807.093299	-0.319868359	0.10233213	-3.1257862	0.0017733	0.0154794
ENSDARG00000022187	usp6nl	1010.44156	-0.383081227	0.10192213	-3.75856776	0.00017089	0.00243406
ENSDARG00000045517	itih5	299.634382	0.642803356	0.19722471	3.25924348	0.0011171	0.01076181
ENSDARG00000045519	sfmbt2	267.328594	-0.583235395	0.16081779	-3.6266845	0.00028708	0.00372609
ENSDARG00000034173	prkcq	233.348981	-1.77845812	0.18205637	-9.76872218	1.53E-22	5.86E-20
ENSDARG00000045522	acot15	486.995106	1.496933644	0.20361181	7.35189988	1.95E-13	2.44E-11
ENSDARG00000069292	acot16	283.36159	-0.67425688	0.20159257	-3.34465139	0.00082386	0.00851129
ENSDARG00000045524	lamb1b	2561.7833	0.509066279	0.10623853	4.79172948	1.65E-06	4.75E-05
ENSDARG00000042469	ndufa12	3643.30115	0.365550242	0.08973375	4.07372088	4.63E-05	0.0008185
ENSDARG00000045528	fgd6	730.635831	0.308105018	0.11634842	2.64812383	0.00809399	0.04855806
ENSDARG00000069261	metap2a	351.104906	0.792342679	0.15660994	5.05933822	4.21E-07	1.45E-05
ENSDARG00000041205	slc6a1l	117.540687	-1.052434347	0.32351387	-3.25313521	0.00114139	0.01094494
ENSDARG00000029071	creld2	480.526284	0.925874823	0.13980657	6.62254135	3.53E-11	3.01E-09
ENSDARG00000014941	tprkb	31.2767054	1.155066012	0.40696531	2.83824197	0.00453628	0.03154671
ENSDARG00000097728	CR388132.1	45.3561497	6.260519939	1.34534137	4.65348058	3.26E-06	8.42E-05
ENSDARG00000096684	CR382372.1	40.0183554	-1.540702757	0.36651034	-4.20370881	2.63E-05	0.00050789
ENSDARG00000091381	si:ch211-214c20.1	30.1856033	1.816582667	0.45692719	3.97565015	7.02E-05	0.00115914
ENSDARG00000092968	znf1128	36.9839672	1.729445106	0.39672789	4.35927281	1.30E-05	0.00028278
ENSDARG00000103471	si:dkey-199m13.4	41.6718843	1.510570049	0.33569274	4.49985916	6.80E-06	0.0001605
ENSDARG00000113016	CU207245.1	40.2511722	4.84194852	0.73986667	6.54435283	5.98E-11	4.97E-09
ENSDARG00000101374	si:dkey-11n14.1	119.279017	-1.041025983	0.29422045	-3.53825162	0.00040279	0.00486219
ENSDARG00000100061	si:dkey-16p6.1	31.9190104	-1.058266545	0.39207886	-2.69911658	0.00695238	0.04346156
ENSDARG00000107177	znf1108	13.6037396	-1.590504457	0.5965096	-2.66635184	0.00766794	0.04668953
ENSDARG00000088245	si:dkey-16p6.1	130.358542	-1.440936388	0.2849132	-5.05745738	4.25E-07	1.46E-05
ENSDARG00000089304	si:dkeyp-4f2.1	91.4207915	-1.333280742	0.27378395	-4.86982797	1.12E-06	3.41E-05
ENSDARG00000094484	si:ch211-162i8.4	17.7508731	-1.525724036	0.52567695	-2.90239861	0.00370317	0.02719122
ENSDARG00000086449	znf1055	20.9397073	1.384169832	0.5188376	2.66782869	0.00763432	0.04651299
ENSDARG00000091013	si:dkey-84h14.2	390.167322	-0.9624171	0.17440241	-5.51837048	3.42E-08	1.58E-06
ENSDARG00000101948	si:ch211-223g7.6	55.5885001	-1.142004637	0.27839199	-4.10214624	4.09E-05	0.00073512
ENSDARG00000102904	si:ch211-246b8.2	22.7156154	1.845379527	0.53427259	3.45400376	0.00055233	0.00623723

Table S1. DEGs of WT vs. *terfa+/-*

ENSDARG00000101459	si:dkey-279j5.1	45.1499693	1.783151788	0.45249416	3.94071779	8.12E-05	0.00130776
ENSDARG00000095275	si:dkey-269p2.1	46.8115489	-0.950746834	0.3230665	-2.94288275	0.00325172	0.02458082
ENSDARG00000101623	znf1109	43.6240034	1.520301529	0.40158224	3.78577879	0.00015323	0.00222848
ENSDARG00000099152	si:dkey-190j3.2	38.9414881	1.244774767	0.38706693	3.21591613	0.00130029	0.01212708
ENSDARG00000098452	si:dkey-151g22.1	14.9993266	1.748976496	0.61747446	2.83246774	0.00461902	0.03197933
ENSDARG00000103473	BX537137.3	9.83284546	2.228109522	0.79525547	2.80175315	0.00508257	0.034478
ENSDARG00000100896	znf973	69.2817462	0.908874571	0.3103811	2.92825355	0.00340872	0.02551919
ENSDARG00000098621	CR759843.1	33.6466938	1.674366969	0.48601832	3.44506966	0.00057091	0.00639767
ENSDARG00000101977	si:ch211-209n20.59	8.06089421	2.872593754	1.07355963	2.67576544	0.00745588	0.04571647
ENSDARG00000099382	si:ch211-209n20.1	12.5334681	3.908805419	1.01201832	3.86238603	0.00011228	0.00172379
ENSDARG00000087070	znf977	120.054497	-1.24195507	0.23140598	-5.36699633	8.01E-08	3.37E-06
ENSDARG00000087788	si:ch211-22k7.9	35.2579608	1.455587801	0.46628681	3.12165766	0.00179836	0.01562875
ENSDARG00000108525	CR749167.2	48.1562969	4.995669821	1.34473654	3.71498034	0.00020322	0.00281035
ENSDARG00000104850	si:ch211-215p11.1	128.277106	-0.849008463	0.21982297	-3.86223722	0.00011235	0.00172379
ENSDARG00000101817	si:dkey-5i16.5	59.9839242	-1.091834743	0.27325276	-3.99569517	6.45E-05	0.00108182
ENSDARG00000095777	si:ch211-218h8.3	6.33252027	3.912150572	1.32954222	2.94247939	0.00325595	0.0245978
ENSDARG00000089831	si:dkey-207m2.4	142.419393	-1.573094718	0.36716196	-4.28447081	1.83E-05	0.00037379
ENSDARG00000090116	znf1136	94.0508098	-0.851559203	0.24517464	-3.47327606	0.00051415	0.0058917
ENSDARG00000110658	zgc:174696	52.2159489	-1.129833163	0.34576019	-3.26767859	0.00108433	0.01049499
ENSDARG00000103441	znf1060	59.5969304	2.143636698	0.3807082	5.63065548	1.80E-08	8.85E-07
ENSDARG00000098390	si:ch211-76m11.8	39.7536779	1.643005571	0.43367301	3.78858154	0.00015151	0.00220737
ENSDARG00000098514	si:ch211-59d8.3	30.432656	2.438397355	0.58266654	4.18489342	2.85E-05	0.00054048
ENSDARG00000113896	si:dkey-29p23.1	117.123444	-3.277083293	0.29009964	-11.296406	1.37E-29	9.71E-27
ENSDARG00000111465	znf1104	129.001728	2.067633909	0.28952403	7.14149319	9.23E-13	1.06E-10
ENSDARG00000092471	si:dkey-7j22.1	41.2479697	1.168730725	0.40375923	2.89462293	0.00379614	0.0276939
ENSDARG00000088861	znf1021	42.2804793	1.304492116	0.39903762	3.26909558	0.00107892	0.01045842
ENSDARG00000109749	si:ch211-162i8.7	53.1073926	2.082956697	0.42753404	4.87202534	1.10E-06	3.37E-05
ENSDARG00000110229	zgc:173702	18.0178915	1.882700183	0.68093061	2.76489286	0.00569415	0.03753968
ENSDARG00000102848	BX927336.3	48.3966932	-1.479718403	0.46232835	-3.20057898	0.00137152	0.01264901
ENSDARG00000099829	BX927336.2	36.22931	2.882451207	0.59276051	4.86275848	1.16E-06	3.50E-05
ENSDARG00000114645	BX942815.11	16.0470314	1.705660913	0.57161428	2.98393683	0.00284566	0.02228102
ENSDARG00000113660	si:ch211-196f19.1	94.2609835	2.1469273	0.34040114	6.30705082	2.84E-10	2.08E-08
ENSDARG00000098928	si:ch211-196h24.1	19.1799969	1.405089363	0.46712989	3.00792008	0.00263042	0.02101707
ENSDARG00000101245	znf1063	14.6614496	2.051165007	0.61444691	3.33822986	0.00084314	0.00867843
ENSDARG00000092000	znf1020	57.9929396	0.91381159	0.318248	2.87138203	0.00408681	0.02930558
ENSDARG00000096065	si:dkey-156k2.7	4.0857592	4.700891809	1.66724144	2.81956272	0.00480891	0.03298166
ENSDARG00000116625	znf1141	176.764839	1.530474435	0.2732359	5.60129332	2.13E-08	1.02E-06
ENSDARG00000100578	si:dkeyp-90h9.1	47.3581121	2.270740267	0.54611161	4.158015	3.21E-05	0.00059948
ENSDARG00000098522	BX649431.1	17.5761686	-1.510760129	0.56452877	-2.67614374	0.00744747	0.0457054
ENSDARG00000102994	znf1034	13.4508456	-2.01682526	0.72888258	-2.76700982	0.00565731	0.03737701
ENSDARG00000105137	zgc:173705	24.9280045	6.560203517	1.26885344	5.17018225	2.34E-07	8.65E-06
ENSDARG00000102595	si:dkey-250k10.1	209.355574	-1.125278933	0.2346193	-4.79619076	1.62E-06	4.65E-05
ENSDARG00000099825	zgc:174944	52.3690032	1.053695036	0.38567003	2.73211543	0.00629291	0.04024968
ENSDARG00000102215	si:ch211-227e10.6	186.472953	-0.903780666	0.31728026	-2.84852472	0.00439224	0.03086213
ENSDARG00000100673	znf974	19.2058655	-1.375272294	0.49765159	-2.76352436	0.00571808	0.03765844
ENSDARG00000101997	si:dkey-191j3.2	25.9272867	-1.42069166	0.50734727	-2.80023515	0.00510654	0.0345651
ENSDARG00000100386	si:dkey-61p9.9	22.4549636	-1.741478271	0.57072299	-3.05135471	0.00227811	0.01884948
ENSDARG00000105304	CR762475.4	30.6416146	-1.67757937	0.44046772	-3.80863177	0.00013974	0.00207354
ENSDARG00000099845	zgc:174275	81.8940294	1.208923584	0.28887225	4.18497651	2.85E-05	0.00054048
ENSDARG00000104476	si:dkey-41c6.3	102.117582	-1.165604724	0.22488961	-5.18300825	2.18E-07	8.14E-06
ENSDARG00000098884	BX649355.1	10.2877394	-1.778414969	0.67208603	-2.64611208	0.00814228	0.04873018
ENSDARG00000103119	si:dkeyp-33c10.7	38.1608455	1.73338016	0.52470647	3.30352349	0.00095478	0.00952289
ENSDARG00000103103	BX957361.2	37.6856352	-1.505070024	0.35022613	-4.29742354	1.73E-05	0.00035612
ENSDARG00000100865	si:dkey-269o24.1	165.718725	-1.669767256	0.24872513	-6.71330345	1.90E-11	1.72E-09
ENSDARG00000112450	si:ch211-238e22.2	15.3185191	-1.945729829	0.67867424	-2.86695695	0.00414439	0.02959176
ENSDARG00000115894	zgc:194906	10.1912335	-1.836579543	0.67775865	-2.709784	0.0067327	0.04241859
ENSDARG00000103597	si:dkey-40j3.3	25.6621658	-1.375196504	0.50451247	-2.72579288	0.00641472	0.04088398
ENSDARG00000087544	BX510934.1	29.26663	2.873816326	0.5415007	5.30713321	1.11E-07	4.51E-06

Table S1. DEGs of WT vs. *terfa+/-*

ENSDARG00000100257	zgc:174314	74.6310923	-0.629448341	0.22705561	-2.77222103	0.00556752	0.0369508
ENSDARG00000104697	si:dkey-57k17.1	106.947383	1.495306792	0.38494978	3.88442043	0.00010257	0.00159442
ENSDARG00000102917	CR388095.1	42.376234	-2.089771861	0.43133099	-4.84493794	1.27E-06	3.79E-05
ENSDARG00000077697	si:dkey-105i14.1	242.426808	-0.529223763	0.16860264	-3.13888184	0.00169594	0.01493712
ENSDARG00000076255	znf1084	56.8246802	1.02655878	0.38252856	2.68361343	0.00728313	0.04497444
ENSDARG00000094653	si:dkey-149m13.5	43.6479458	1.173323633	0.39542229	2.96726733	0.0030046	0.02320672
ENSDARG00000086615	CR846087.1	42.0348515	1.409245288	0.36794708	3.83002164	0.00012813	0.00193013
ENSDARG00000103439	si:dkey-248e17.5	7.42666158	-3.156983339	1.01216949	-3.11902637	0.0018145	0.01573049
ENSDARG00000104416	si:dkey-16p6.1	17.6522019	2.492334162	0.60897369	4.09267955	4.26E-05	0.0007603
ENSDARG00000116007	CABZ01016118.1	10.0904233	-3.033124023	0.94562939	-3.20751877	0.00133885	0.01239836
ENSDARG00000117620	BX000981.15	12.4418537	-6.40672429	1.3287297	-4.8216912	1.42E-06	4.18E-05
ENSDARG00000092940	BX000981.1	3.87673457	-5.387320791	1.59507356	-3.37747482	0.00073155	0.0077762
ENSDARG00000093576	BX000981.2	26.3271649	2.920641433	0.63035281	4.63334406	3.60E-06	9.19E-05
ENSDARG00000102673	si:dkey-26i24.1	27.9438912	2.046963275	0.50421949	4.05966707	4.91E-05	0.00086138
ENSDARG00000098196	znf1077	24.0643881	1.489422215	0.51478485	2.89329071	0.00381228	0.02777203
ENSDARG00000107425	si:dkeyp-35e5.10	16.4846776	1.685892643	0.61345815	2.74817874	0.00599273	0.03888003
ENSDARG00000099346	znf1079	6.97720402	5.884125307	1.40375381	4.19170746	2.77E-05	0.00052934
ENSDARG00000116692	si:ch211-79g12.1	17.812147	2.664548092	0.68767869	3.87469924	0.00010676	0.00165014
ENSDARG00000116367	si:dkey-51d8.1	17.4663051	2.321198636	0.68088201	3.40910557	0.00065176	0.00711181
ENSDARG00000110878	si:dkey-28k24.2	9.81656401	2.529236423	0.79419029	3.18467307	0.00144918	0.01318422
ENSDARG00000112928	CR450780.2	31.1540849	1.843866344	0.49844748	3.6992189	0.00021626	0.00295994
ENSDARG00000105119	si:ch211-258f1.3	99.1378399	-0.929440304	0.30180912	-3.0795634	0.00207304	0.0174979
ENSDARG00000098087	si:dkey-30f3.2	21.9997117	1.627948651	0.57191601	2.84648206	0.00442052	0.03098479
ENSDARG00000100652	znf1147	16.6440032	-1.789461153	0.65486422	-2.73256821	0.00628427	0.04021911
ENSDARG00000098892	si:ch211-223a21.1	66.3975413	1.101479162	0.28314578	3.89014859	0.00010018	0.00156291
ENSDARG00000104887	znf1105	56.5792329	-0.96128277	0.27747598	-3.46438197	0.00053145	0.00605372
ENSDARG00000100370	CT955963.1	27.9037299	-1.262998695	0.47593577	-2.65371669	0.00796106	0.04800394
ENSDARG00000101528	si:ch211-194h1.2	21.9759454	-1.734864183	0.50273796	-3.4508319	0.00055886	0.00629099
ENSDARG00000098991	znf1095	18.9884853	1.645041509	0.57421317	2.86486203	0.00417191	0.02975404
ENSDARG00000103239	si:dkey-14o6.8	28.3833951	-1.273827092	0.43484357	-2.9293916	0.00339626	0.02544893
ENSDARG00000100160	si:dkey-205i10.2	2.78917343	4.71018982	1.73142237	2.72041641	0.00651998	0.04138024
ENSDARG00000104266	si:dkey-29m1.2	40.1912694	2.481348891	0.44229856	5.61012197	2.02E-08	9.82E-07
ENSDARG00000098189	si:dkey-16p6.1	16.734456	3.033023379	0.66575628	4.55575631	5.22E-06	0.00012795
ENSDARG00000113780	zgc:173615	40.2554952	1.742344571	0.43486885	4.00659777	6.16E-05	0.00104068
ENSDARG00000101634	si:ch211-209j12.1	54.7806416	2.915223056	0.48139805	6.05574337	1.40E-09	9.02E-08
ENSDARG00000109319	znf1030	29.3434567	-1.617036473	0.51380035	-3.14720778	0.00164838	0.01463752
ENSDARG00000101807	si:ch211-120c15.3	42.7147283	-4.061089128	0.57557735	-7.0556792	1.72E-12	1.85E-10
ENSDARG00000104561	znf1081	74.605253	1.128199412	0.34711987	3.25017239	0.00115335	0.01103408
ENSDARG00000099090	znf1087	14.4719359	-1.844429798	0.61448247	-3.0015987	0.00268566	0.021347
ENSDARG00000102884	si:ch211-145h19.5	9.57363965	-3.107261786	0.80583419	-3.85595676	0.00011528	0.00176105
ENSDARG00000100743	si:dkey-190j3.4	17.595615	3.373981301	0.75205124	4.48637157	7.24E-06	0.00017003
ENSDARG00000101388	si:ch211-171l17.14	35.8829617	2.709063894	0.49802546	5.43960928	5.34E-08	2.33E-06
ENSDARG00000102616	si:dkey-3h2.3	15.5057949	2.566646999	0.75758858	3.38791671	0.00070426	0.00755472
ENSDARG00000102581	si:dkeyp-44b5.5	9.9964577	2.536190411	0.90568209	2.80030979	0.00510536	0.0345651
ENSDARG00000104897	si:dkey-16p6.1	45.0709063	4.660047336	0.63261501	7.3663243	1.75E-13	2.20E-11
ENSDARG00000103324	si:ch211-127b6.2	26.209213	4.921086646	0.90814272	5.41884724	6.00E-08	2.58E-06
ENSDARG00000101756	im:7141269	137.903756	1.030232172	0.24397519	4.22269234	2.41E-05	0.00047221
ENSDARG00000105279	si:ch211-108c17.2	387.300412	0.967387664	0.15139097	6.38999598	1.66E-10	1.27E-08
ENSDARG00000077794	CU468012.1	24.8743219	2.063360589	0.60055027	3.43578328	0.00059084	0.00656185
ENSDARG00000101728	si:dkeyp-80d11.12	7.74654818	3.150576759	1.00581166	3.13237247	0.001734	0.01522915
ENSDARG00000103254	si:dkeyp-80d11.4	12.056179	3.038499079	0.80511278	3.77400427	0.00016065	0.00231607
ENSDARG00000101071	si:dkeyp-80d11.14	55.4458809	2.668210048	0.37343281	7.1450873	8.99E-13	1.03E-10
ENSDARG00000101421	si:dkeyp-80d11.10	14.9302203	4.207512882	1.19311629	3.52649019	0.00042111	0.00504172
ENSDARG00000099698	BX548059.1	22.235575	4.018740638	0.97313196	4.12969753	3.63E-05	0.00066581
ENSDARG00000116086	si:dkey-193i10.4	9.48002027	2.608861913	0.92164262	2.83066543	0.00464513	0.03211534
ENSDARG00000100240	CT737123.1	22.0262971	4.500115274	0.85629623	5.25532534	1.48E-07	5.77E-06
ENSDARG00000100464	si:dkey-193i10.1	14.7003681	1.695246208	0.61034439	2.77752402	0.00547748	0.03648961
ENSDARG00000102225	si:ch211-205a14.7	14.6938695	2.454692657	0.6938995	3.53753342	0.00040388	0.00487306

Table S1. DEGs of WT vs. *terfa+/-*

ENSDARG00000102713	si:ch211-205a14.1	93.7994393	4.758246652	0.45512525	10.454807	1.39E-25	6.41E-23
ENSDARG00000104890	si:ch211-76m11.3	214.833865	5.603050487	0.37029536	15.1313009	1.01E-51	4.17E-48
ENSDARG00000100338	BX936384.1	8.01148813	4.610471014	1.19809845	3.84815706	0.00011901	0.00181138
ENSDARG00000100006	si:ch211-76m11.5	19.2437078	1.86969648	0.56158891	3.32929734	0.00087065	0.00892008
ENSDARG00000101828	si:dkey-16p6.1	60.2626935	3.817706274	0.4869548	7.83996024	4.51E-15	7.37E-13
ENSDARG00000101757	si:dkey-27n6.4	50.610123	2.828696078	0.43120063	6.56004626	5.38E-11	4.52E-09
ENSDARG00000103306	BX927130.4	150.889511	3.086433595	0.36249376	8.51444612	1.67E-17	4.04E-15
ENSDARG00000114142	si:dkeyp-4f2.1	250.173315	4.327758396	0.28054629	15.4261832	1.09E-53	6.78E-50
ENSDARG00000117400	CU651668.1	296.965524	0.396372759	0.14346477	2.76285783	0.00572977	0.03769595
ENSDARG00000101514	ftr64	42.2851387	-1.498048923	0.37331127	-4.01286816	6.00E-05	0.00101632
ENSDARG00000105731	CR788316.1	384.394345	-0.785966065	0.25496593	-3.08263169	0.00205179	0.01736371
ENSDARG00000105829	CR788316.4	136.076017	-1.119287684	0.34877923	-3.20915811	0.00133124	0.01236012
ENSDARG00000101645	myrfl	408.524376	0.459351732	0.16638698	2.76074329	0.005767	0.03785021
ENSDARG00000102835	CABZ01021435.1	31.4103828	1.358745511	0.4720616	2.87832245	0.00399796	0.02879437
ENSDARG00000105781	zgc:171727	42.8796649	1.818682237	0.38981646	4.66548346	3.08E-06	8.06E-05
ENSDARG00000074298	znf1015	533.483591	-0.322274134	0.10739462	-3.00084067	0.00269235	0.0213713
ENSDARG00000096120	si:ch73-266f23.3	287.142539	0.728833608	0.1604928	4.54122306	5.59E-06	0.00013602
ENSDARG00000103751	si:dkey-262g12.14	328.566529	0.725728043	0.14645535	4.95528533	7.22E-07	2.29E-05
ENSDARG00000080016	si:cabz01032454.3	85.3496774	-1.091004952	0.25269619	-4.31745701	1.58E-05	0.00032915
ENSDARG00000105806	CABZ01069020.1	110.412858	-0.629787644	0.21685015	-2.9042528	0.00368131	0.02709473
ENSDARG00000113971	NA	28.5113296	-9.096455369	1.24409016	-7.31173326	2.64E-13	3.26E-11
ENSDARG00000109502	PHF21B	358.175916	0.674192544	0.19897331	3.38835667	0.00070313	0.00754587
ENSDARG00000017454	nup50	2955.42184	0.460378912	0.11037381	4.17108832	3.03E-05	0.00057136
ENSDARG00000068830	zgc:172139	217.048783	0.788981418	0.18917993	4.17053437	3.04E-05	0.00057177
ENSDARG00000102191	CPNE8	1054.062	-0.524846332	0.13211337	-3.97269646	7.11E-05	0.0011705
ENSDARG00000103844	si:ch211-266c8.1	268.239859	-1.121140929	0.16438853	-6.82006805	9.10E-12	8.67E-10
ENSDARG00000098369	si:dkey-165o8.1	53.9788032	-1.360836536	0.33184374	-4.10083531	4.12E-05	0.00073823
ENSDARG00000090392	si:dkey-71l4.2	81.0759819	-0.878407864	0.27198394	-3.22963132	0.0012395	0.01166961
ENSDARG00000104956	si:dkey-165o8.2	200.363948	-1.037618945	0.26361234	-3.93615463	8.28E-05	0.00132684
ENSDARG00000104166	si:ch211-232d10.1	92.9553774	-1.123103038	0.28469877	-3.94488195	7.98E-05	0.00129025
ENSDARG00000112632	si:ch211-106j21.6	40.0409632	-3.320421597	0.42259902	-7.85714458	3.93E-15	6.51E-13
ENSDARG00000100405	BX957252.3	35.6503474	-2.894596757	0.50250422	-5.7603432	8.39E-09	4.52E-07
ENSDARG00000099327	zgc:174944	23.2678175	-1.792813373	0.47845488	-3.74708972	0.0001789	0.00252786
ENSDARG00000103186	si:ch73-389k6.1	246.154605	-1.546746503	0.31799951	-4.86399016	1.15E-06	3.49E-05
ENSDARG00000102473	zgc:171673	198.067722	-1.191654422	0.20981885	-5.67944415	1.35E-08	6.98E-07
ENSDARG00000054898	ms4a17a.16	14.83915	-1.667839802	0.60353996	-2.76342896	0.00571975	0.03765947
ENSDARG00000043802	ms4a17a.8	45.8264759	-1.224464363	0.38630972	-3.16964419	0.00152626	0.01373466
ENSDARG00000014024	ms4a17a.4	33.9204892	1.71022611	0.52997847	3.22697283	0.00125107	0.01175186
ENSDARG00000093546	si:ch73-56d11.5	457.467876	2.75784471	0.20262451	13.6106175	3.46E-42	7.82E-39
ENSDARG00000106998	si:dkey-240n22.6	11.461329	2.948242351	0.83529368	3.52958778	0.00041621	0.00499509
ENSDARG00000105675	BX005417.3	7.36192716	3.149378629	1.06897775	2.9461592	0.00321747	0.02438858
ENSDARG00000095696	trpm2	124.332476	-0.883823027	0.23737618	-3.72330126	0.00019663	0.00273801
ENSDARG00000104678	CU467646.7	404.693476	0.533709618	0.1355849	3.93635007	8.27E-05	0.00132662
ENSDARG00000099945	CU467646.5	95.4724151	-0.63889169	0.22820378	-2.79965427	0.00511574	0.03459908
ENSDARG00000043781	psmb10	454.61845	0.652688392	0.14720297	4.43393501	9.25E-06	0.00020907
ENSDARG00000104108	slco1d1	1869.40987	0.383046698	0.1366695	2.80272268	0.00506732	0.0344027
ENSDARG00000096762	si:dkey-61p9.11	195.015417	1.705368173	0.24750633	6.89020032	5.57E-12	5.47E-10
ENSDARG00000099384	si:dkey-238k10.1	116.673028	-0.803436507	0.23491156	-3.42016592	0.00062583	0.00688274
ENSDARG00000104380	si:dkey-238k10.2	366.97707	-0.614718558	0.19058278	-3.22546739	0.00125767	0.01180492
ENSDARG00000101992	si:dkey-238k10.3	50.672991	-1.139573085	0.28231518	-4.03652782	5.42E-05	0.00093569
ENSDARG00000100654	si:dkey-207m2.4	106.264795	-1.370398619	0.24366532	-5.62410212	1.86E-08	9.15E-07
ENSDARG00000090778	ttll1	673.136593	0.468898345	0.15656597	2.99489312	0.00274541	0.02170393
ENSDARG00000078014	pacsin2	2392.82951	0.390871567	0.09221901	4.23851408	2.25E-05	0.00044526
ENSDARG00000032405	ckap4	6804.75	0.334393667	0.09898861	3.37810262	0.00072988	0.00776589
ENSDARG00000087206	cct2	3818.00233	0.475741169	0.11961566	3.97724817	6.97E-05	0.00115445
ENSDARG00000113112	NRXN2	1435.34595	0.583326978	0.16295545	3.57967152	0.00034403	0.00428825
ENSDARG00000063150	nrxn2b	1777.17911	0.445044928	0.14613047	3.04553142	0.0023227	0.01914779
ENSDARG00000041203	drap1	4224.07083	0.465141604	0.10785228	4.31276566	1.61E-05	0.00033505

Table S1. DEGs of WT vs. *terfa+/-*

ENSDARG00000102296	zgc:175177	20.3189849	-2.271296878	0.55008409	-4.12899939	3.64E-05	0.00066685
ENSDARG00000105657	si:cabz01080528.1	395.084896	1.365171614	0.15905489	8.58302207	9.24E-18	2.32E-15
ENSDARG00000117751	CABZ01112025.1	80.3597983	0.817360589	0.27576116	2.96401631	0.00303652	0.02338065
ENSDARG00000117818	CR926130.2	336.799889	1.471206378	0.25036545	5.87623552	4.20E-09	2.41E-07
ENSDARG00000117735	CR926130.1	96.5604324	1.093085764	0.37157299	2.94177935	0.00326332	0.02463849
ENSDARG00000076386	epdl1	2259.87571	0.794814724	0.14050223	5.65695461	1.54E-08	7.80E-07
ENSDARG00000105551	si:cabz01101003.1	235.566332	0.490594831	0.17911487	2.73899551	0.00616272	0.03967222
ENSDARG00000039268	emc4	166.322267	-1.490698822	0.22234102	-6.70456058	2.02E-11	1.81E-09
ENSDARG00000035028	lpcat4	385.05465	-1.278830412	0.13321335	-9.59986685	8.00E-22	2.88E-19
ENSDARG00000104227	nop10	627.64114	-0.416245138	0.1084279	-3.83891179	0.00012358	0.00187065
ENSDARG00000079949	supt16h	5072.33227	0.344581495	0.13068138	2.63680639	0.00836906	0.04979084
ENSDARG00000105590	si:cabz01007794.1	3396.28269	1.053132892	0.12140506	8.67453865	4.15E-18	1.08E-15
ENSDARG00000089961	si:cabz01007807.1	269.76916	0.773716624	0.18603754	4.15892749	3.20E-05	0.00059754
ENSDARG00000105625	si:cabz01007812.1	200.049691	0.533729808	0.19140827	2.78843649	0.00529631	0.03556873
ENSDARG00000076146	zgc:172075	166.367676	1.537522758	0.2191383	7.01622106	2.28E-12	2.40E-10
ENSDARG00000097459	NA	14.8057541	-6.254108238	1.84875707	-3.38287184	0.00071732	0.0076618
ENSDARG00000105466	NA	2.43344312	-5.488386344	1.99078025	-2.75690215	0.00583518	0.03820691
ENSDARG00000117704	NA	8.37440739	-4.336947965	1.11292933	-3.89687633	9.74E-05	0.00152606
ENSDARG00000117506	NA	5.97597746	-3.894376494	1.20407872	-3.23432051	0.00121933	0.01153209
ENSDARG00000076257	si:ch211-285c6.1	43.571886	7.343830542	1.22299423	6.00479574	1.92E-09	1.20E-07
ENSDARG00000079234	si:ch211-285c6.2	8.23129035	2.998465237	0.99248966	3.02115515	0.00251812	0.02033334
ENSDARG00000094779	si:dkey-192d15.1	17.5923392	2.066410572	0.61551065	3.35722959	0.00078728	0.0082183
ENSDARG00000043770	si:dkey-192d15.2	388.790213	1.031688029	0.20516423	5.02859598	4.94E-07	1.64E-05
ENSDARG00000011170	ndrg2	9420.84212	-0.343666868	0.09305562	-3.69313402	0.00022151	0.00301344
ENSDARG00000105450	si:ch211-63p21.1	466.820898	-1.035052769	0.20234798	-5.11521177	3.13E-07	1.12E-05
ENSDARG00000086654	cbln11	69.9536645	1.720999405	0.4239926	4.05903171	4.93E-05	0.00086312
ENSDARG00000105443	si:ch211-63p21.8	71.3962988	-1.670316917	0.38155826	-4.37761958	1.20E-05	0.00026344
ENSDARG00000071173	slc12a10.2	1007.3867	0.718495026	0.20411686	3.52001805	0.00043152	0.00515106
ENSDARG00000105470	si:dkey-23a13.2	42.7055989	1.43979508	0.47279614	3.04527669	0.00232466	0.01915766
ENSDARG00000105407	CU459186.6	16.6352983	6.772087409	1.29521897	5.22852703	1.71E-07	6.55E-06
ENSDARG00000100259	zgc:171759	9.22670398	3.335714537	0.97456458	3.42277424	0.00061986	0.00684139
ENSDARG00000105439	CU459186.7	37.5599164	1.115339802	0.36405806	3.06363169	0.00218668	0.01824435
ENSDARG00000015607	si:ch211-220f21.2	22.8323183	7.829934866	1.25772969	6.22545127	4.80E-10	3.35E-08
ENSDARG00000105507	zgc:112234	33.2788355	-1.421813528	0.42253499	-3.36496041	0.00076555	0.00804552
ENSDARG00000078619	pnp5a	4001.68186	1.160109282	0.08084647	14.3495352	1.07E-46	3.33E-43
ENSDARG00000073728	gal3st3	299.658259	0.372816033	0.13204102	2.82348656	0.00475044	0.03267079
ENSDARG00000004658	zgc:101810	2234.86096	0.717198142	0.09712449	7.38431794	1.53E-13	1.94E-11
ENSDARG00000062749	ino80b	869.70299	-0.306252269	0.10124352	-3.02490743	0.00248709	0.02015542
ENSDARG00000102776	cxcl19	5.06582094	4.651980465	1.54408308	3.0127786	0.00258868	0.02076204
ENSDARG00000036893	f13a1b	809.252706	0.401281982	0.14721768	2.72577305	0.00641511	0.04088398
ENSDARG00000039099	aep1	11478.639	0.429742794	0.11668041	3.68307591	0.00023044	0.00311446
ENSDARG00000092521	jac1	43.7082822	2.682310586	0.53099151	5.05151311	4.38E-07	1.49E-05
ENSDARG00000105489	loc564660	7.40024349	3.282910952	1.1822937	2.77673048	0.00549087	0.03656457
ENSDARG00000074983	jac9	96.1159784	4.044784265	1.00600886	4.02062488	5.80E-05	0.00098949
ENSDARG00000076958	jac8	21.3586966	2.719888645	0.97338244	2.79426517	0.00520178	0.03506652
ENSDARG00000104574	pcsk6	477.5134	0.525670619	0.13907729	3.77970123	0.00015702	0.00227331
ENSDARG00000024651	snrpa1	1262.68172	-0.294597936	0.10223533	-2.88156673	0.00395703	0.02854923
ENSDARG00000101762	fah	834.130153	-0.852437546	0.1203384	-7.08367041	1.40E-12	1.58E-10
ENSDARG00000105718	CABZ01068273.1	30.6568567	1.311667585	0.45212252	2.90113308	0.00371816	0.02727766
ENSDARG00000079672	efl1	300.919381	0.530319954	0.15408262	3.44178949	0.00057788	0.00644379
ENSDARG00000058349	polr2l	491.694624	-0.396376166	0.14897001	-2.66077819	0.00779603	0.04724953
ENSDARG00000099257	si:ch73-119p20.1	1566.36974	-0.42586584	0.13136086	-3.24195378	0.00118713	0.01130938
ENSDARG00000036870	arih1l	801.616921	-0.343631456	0.10647401	-3.22737411	0.00124932	0.01174871
ENSDARG00000062909	furina	1521.31099	0.626166464	0.09261025	6.76130857	1.37E-11	1.27E-09
ENSDARG00000003203	rhcga	3279.95773	3.788549837	0.32534103	11.6448573	2.44E-31	1.95E-28
ENSDARG00000036809	ANPEP	1458.23905	-0.44223737	0.13587852	-3.2546525	0.00113531	0.01089505
ENSDARG00000070006	rcn1	1429.67774	0.390389481	0.09172955	4.25587459	2.08E-05	0.00041739
ENSDARG00000105589	CT030144.2	59.1954982	-1.198942196	0.30672526	-3.90884724	9.27E-05	0.00146256

Table S1. DEGs of WT vs. *terfa+/-*

ENSDARG00000060962	btr04	252.43798	-0.512761161	0.15812865	-3.2426835	0.0011841	0.01128479
ENSDARG00000000460	nitr2b	5.65959004	3.405230212	1.1774206	2.89211027	0.00382664	0.02784575
ENSDARG00000055504	si:ch211-212k18.7	2285.84171	0.396285963	0.08616046	4.59939492	4.24E-06	0.00010689
ENSDARG00000086421	si:ch211-212k18.8	407.689544	0.854188651	0.1759593	4.8544671	1.21E-06	3.64E-05
ENSDARG00000069313	oxa1l	1225.41496	-0.616673829	0.12271926	-5.02507785	5.03E-07	1.67E-05
ENSDARG00000104169	si:ch211-212k18.4	5524.38301	-0.298659404	0.07221701	-4.13558263	3.54E-05	0.00065282
ENSDARG00000062085	tkfc	415.930119	-0.487286497	0.12422032	-3.92275993	8.75E-05	0.00139208
ENSDARG00000036414	si:ch73-335l21.1	1719.49635	0.379456314	0.11547203	3.28613178	0.00101573	0.00999237
ENSDARG00000077414	zgc:194312	224.212744	-0.795591785	0.16690546	-4.76672107	1.87E-06	5.27E-05
ENSDARG00000061850	si:dkey-19b23.13	207.074921	-0.63590406	0.20954718	-3.03465813	0.00240808	0.01964324
ENSDARG00000100712	si:dkey-19b23.12	1170.73357	-0.438070698	0.12195436	-3.59208726	0.00032804	0.00412623
ENSDARG00000069376	tnfsf12	286.878463	-0.477331373	0.16584387	-2.87819733	0.00399955	0.02879745
ENSDARG00000098932	gigyf1a	1234.7498	-0.460355129	0.13977192	-3.29361661	0.00098907	0.00979419
ENSDARG00000088087	kdm6ba	8667.06623	0.302224699	0.11379444	2.65588289	0.00791011	0.04778943
ENSDARG00000088589	ponzr3	1379.92309	2.035326779	0.22137069	9.1942018	3.78E-20	1.19E-17
ENSDARG00000087440	ponzr4	614.56782	1.453154428	0.21336619	6.8106125	9.72E-12	9.22E-10
ENSDARG00000016260	fxr2	5489.89764	0.367165574	0.1061107	3.46021238	0.00053975	0.00612579
ENSDARG00000022129	rbm4.3	8471.8954	0.448197858	0.10594868	4.23032981	2.33E-05	0.00045885
ENSDARG00000036386	rbm4.1	1845.65702	0.714939224	0.13140617	5.44068215	5.31E-08	2.32E-06
ENSDARG00000018228	htr2cl1	285.518858	-0.509142893	0.17485379	-2.91182076	0.00359329	0.02660446
ENSDARG00000029170	si:ch211-200p22.4	4031.12173	0.250923866	0.07908956	3.17265467	0.00151052	0.01363253
ENSDARG00000021135	dhrs4	709.323784	-0.522586797	0.17699617	-2.95253162	0.0031518	0.02402267
ENSDARG00000026842	acin1b	5690.89941	-0.215131242	0.06837729	-3.14623808	0.00165385	0.01467508
ENSDARG00000024877	ptgr1	849.605435	1.086635372	0.16143787	6.73098194	1.69E-11	1.55E-09
ENSDARG00000089877	C7H4orf48	2288.43577	0.34723696	0.13144247	2.64174098	0.00824811	0.04923314
ENSDARG00000105583	si:dkeyp-75h12.7	19.9685691	1.355847772	0.45044412	3.01002434	0.00261227	0.02093098
ENSDARG00000054856	CSKMT	133.165844	-0.504692366	0.18268771	-2.76259618	0.00573437	0.03770573
ENSDARG00000054934	zgc:101765	83.6243418	0.889551292	0.23343483	3.81070501	0.00013857	0.00205961
ENSDARG00000054937	badb	266.309172	-0.427213979	0.15633198	-2.73273572	0.00628107	0.04021911
ENSDARG00000056725	hmgb3a	17012.1352	-1.099188748	0.12933602	-8.49870524	1.92E-17	4.50E-15
ENSDARG00000079313	pelp1	3291.26695	0.410367301	0.12336163	3.32653927	0.00087932	0.0089977
ENSDARG00000069463	alox12	1481.66162	0.660342776	0.08809638	7.49568536	6.60E-14	8.91E-12
ENSDARG00000105529	vgf	1043.53824	0.445402606	0.14385092	3.09627915	0.00195966	0.0167379
ENSDARG00000039211	zgc:77439	2087.39292	0.696189373	0.10831236	6.42760782	1.30E-10	1.01E-08
ENSDARG00000016363	her8a	269.108105	0.634953683	0.2274423	2.79171326	0.00524298	0.03524865
ENSDARG00000040265	mpdu1b	908.685895	0.82679417	0.12987421	6.36611497	1.94E-10	1.45E-08
ENSDARG00000092115	eif4a1a	13930.2601	0.349865194	0.11330996	3.08768272	0.00201724	0.01714721
ENSDARG00000036305	phf23b	487.448536	-0.418315349	0.12229257	-3.42061138	0.00062481	0.00688274
ENSDARG00000056590	calca	340.112417	0.556985884	0.12574587	4.42945653	9.45E-06	0.00021269
ENSDARG00000062190	pde3b	951.461556	0.351949754	0.09815179	3.58577024	0.00033608	0.00421038
ENSDARG00000056557	copb1	6686.66776	0.221047668	0.0805511	2.7441918	0.00606601	0.03924275
ENSDARG00000036252	rras2	1398.6595	0.382119785	0.10658191	3.58522184	0.00033679	0.00421711
ENSDARG00000053625	anxa2b	1153.6604	-0.683209403	0.20364269	-3.35494199	0.00079382	0.00827961
ENSDARG00000053568	pstpip1b	14.9018352	-1.960138355	0.55780819	-3.51400068	0.00044141	0.00524164
ENSDARG00000053559	tspan3b	727.334419	-0.455654747	0.12872232	-3.53982703	0.00040039	0.00484031
ENSDARG00000077167	nmbb	38.8484772	-1.403552835	0.36018747	-3.89672866	9.75E-05	0.00152606
ENSDARG00000008491	si:ch211-107o10.3	321.605254	-0.749598658	0.22617073	-3.31430438	0.00091871	0.00927484
ENSDARG00000074340	serf2	4096.64058	0.268284449	0.09375039	2.8616888	0.0042139	0.02998447
ENSDARG00000053480	aqp9b	928.11768	-1.003190004	0.31986629	-3.13627921	0.00171106	0.01505119
ENSDARG00000052728	sltm	5299.79514	-0.588653883	0.09283077	-6.34115019	2.28E-10	1.70E-08
ENSDARG00000010601	mtmr10	815.377712	-0.344201277	0.10772649	-3.19514045	0.00139763	0.01285337
ENSDARG00000012495	mphosph10	892.329008	-0.278797	0.09484308	-2.93956086	0.00328678	0.02477793
ENSDARG00000105548	si:dkey-1h4.4	17.0449765	3.075547681	0.65759209	4.67698403	2.91E-06	7.71E-05
ENSDARG00000053362	cilp	3671.62546	-0.331308439	0.10467164	-3.16521684	0.00154967	0.01390114
ENSDARG00000075158	igdcc3	2349.61013	0.299994851	0.10956989	2.73793137	0.0061827	0.03976992
ENSDARG00000036152	gas2b	117.393106	-0.840856376	0.26854706	-3.13113229	0.00174134	0.01527203
ENSDARG00000036144	tssc4	1037.80263	-0.300832	0.09844568	-3.05581716	0.00224448	0.01860793
ENSDARG00000095082	ITLN1	136.06516	2.242365265	0.33663038	6.6612089	2.72E-11	2.38E-09

Table S1. DEGs of WT vs. *terfa+/-*

ENSDARG00000079440	coro2ba	986.861388	-0.371739765	0.12804108	-2.90328516	0.0036927	0.02714644
ENSDARG00000060238	uacab	5995.44244	-0.480396277	0.08984533	-5.34692517	8.95E-08	3.71E-06
ENSDARG00000036094	pias1a	654.462605	0.334416019	0.12667547	2.63994302	0.008292	0.04945946
ENSDARG00000036140	crybgx	1564.16009	-1.956976896	0.12646266	-15.4747414	5.14E-54	4.26E-50
ENSDARG00000036139	lctla	544.832532	-1.884896104	0.19559384	-9.63678667	5.59E-22	2.04E-19
ENSDARG00000105641	si:ch211-98n17.5	946.146032	0.526864623	0.12381102	4.25539357	2.09E-05	0.00041787
ENSDARG00000039730	zgc:112160	1450.28063	-2.468587641	0.73064231	-3.37865409	0.00072842	0.0077603
ENSDARG00000093844	zgc:136461	278.760791	-3.113767707	0.97601314	-3.1902928	0.00142129	0.01301145
ENSDARG00000090428	ctrb1	3926.74939	-1.546920865	0.57365697	-2.69659561	0.00700523	0.04368162
ENSDARG00000001975	hsd11b2	1689.9627	0.466656863	0.16428843	2.84047315	0.00450467	0.03140631
ENSDARG00000014378	slc12a4	1154.20375	0.329475292	0.120768	2.72816726	0.00636873	0.04066139
ENSDARG00000016141	slc6a2	368.002017	-0.768952724	0.16903102	-4.54918123	5.39E-06	0.00013162
ENSDARG00000103315	CU464124.1	373.488048	0.482559339	0.16359137	2.94978488	0.00317995	0.02420016
ENSDARG00000105651	BX323060.3	513.76088	0.894441344	0.19810506	4.51498477	6.33E-06	0.00015177
ENSDARG00000005315	celf1	3693.38211	-0.203575809	0.06795018	-2.99595681	0.00273585	0.02164214
ENSDARG00000052578	c6ast4	190.815706	-2.703988365	0.85042485	-3.17957355	0.00147492	0.01338901
ENSDARG00000024503	c6ast3	9.77624993	-3.139341164	1.01766984	-3.08483268	0.00203667	0.01726644
ENSDARG00000004447	aplnr2	140.273508	1.095856086	0.25309338	4.32984879	1.49E-05	0.00031483
ENSDARG00000029242	fbxo3	743.373101	0.321706627	0.11214331	2.86870996	0.0041215	0.02945364
ENSDARG00000104129	si:dkey-23n7.10	116.223832	1.200911646	0.28665933	4.18933385	2.80E-05	0.00053371
ENSDARG00000017474	zgc:110699	97.5280272	-0.745341782	0.26496682	-2.81296267	0.00490873	0.03350805
ENSDARG00000015793	creb3l1	781.833733	0.453205914	0.11345783	3.99448762	6.48E-05	0.00108589
ENSDARG00000033042	ptprja	122.469683	-0.858757473	0.23660669	-3.62947248	0.000284	0.00369767
ENSDARG00000052244	zgc:158564	4424.71137	-0.211564111	0.07815503	-2.70698013	0.00678983	0.04267036
ENSDARG00000010658	insig1	801.122039	0.937716951	0.14474091	6.47858943	9.26E-11	7.45E-09
ENSDARG00000008224	vps35	5294.30517	0.224426458	0.0813518	2.75871526	0.00580291	0.03803554
ENSDARG00000012199	gpt2	1156.7107	-0.322013412	0.10018602	-3.21415525	0.00130829	0.01217429
ENSDARG00000075584	itfg1	659.277651	0.354589963	0.09593898	3.69599461	0.00021903	0.00299117
ENSDARG00000098462	CU570782.1	12.9575952	2.32848581	0.68193522	3.41452641	0.00063893	0.00700512
ENSDARG00000099831	BX005396.2	7.05590941	-2.933044086	1.06803021	-2.74621828	0.00602866	0.03906141
ENSDARG00000102706	BX511128.3	146.945041	-1.467304197	0.30588495	-4.79691529	1.61E-06	4.64E-05
ENSDARG00000045139	ca7	107.452121	-0.545374053	0.20062241	-2.7184105	0.00655964	0.04153071
ENSDARG00000052011	rrad	652.37699	1.261687445	0.18398662	6.85749577	7.01E-12	6.82E-10
ENSDARG00000113027	BX005073.3	4.71773371	4.261597665	1.53478012	2.77668287	0.00549167	0.03656457
ENSDARG00000079013	dpy19l3	1710.38645	-0.331917493	0.12048939	-2.7547446	0.0058738	0.03836878
ENSDARG00000111309	AL935126.2	31.9697401	2.5764996	0.57408735	4.48799226	7.19E-06	0.0001689
ENSDARG00000102528	AL935126.1	45.5538341	2.430417166	0.49073183	4.95263815	7.32E-07	2.32E-05
ENSDARG00000078088	si:ch211-186j3.6	1190.99272	0.65553927	0.18934452	3.46215077	0.00053588	0.00609018
ENSDARG00000059792	trpm5	247.637145	-0.48034043	0.17248596	-2.78480891	0.00535593	0.03581425
ENSDARG00000028058	traf6	745.381044	0.363333603	0.12906378	2.81514769	0.00487548	0.03334621
ENSDARG00000035891	acana	3699.24701	0.692551217	0.11928925	5.80564638	6.41E-09	3.55E-07
ENSDARG00000059751	abtb2a	309.644701	1.143296919	0.22968998	4.97756556	6.44E-07	2.07E-05
ENSDARG00000011897	tsg101b	627.808316	-0.392430465	0.13077438	-3.00082065	0.00269253	0.0213713
ENSDARG00000035872	hsd17b12b	1139.26277	-0.308210799	0.10320438	-2.98641203	0.00282272	0.02214606
ENSDARG00000005993	prc1b	468.981024	-0.568099592	0.13324208	-4.26366506	2.01E-05	0.0004044
ENSDARG00000020473	hddc3	518.585663	-0.612545364	0.14435367	-4.24336525	2.20E-05	0.00043748
ENSDARG00000086452	dthd1	36.5180763	-1.594230775	0.38808004	-4.10799477	3.99E-05	0.0007204
ENSDARG00000012499	per1b	4410.69075	1.355934086	0.10844621	12.5032868	7.16E-36	7.74E-33
ENSDARG00000018329	gc2	99.9078301	-0.648579461	0.20875949	-3.10682619	0.00189108	0.01624704
ENSDARG00000003021	hdac8	536.545979	0.522895936	0.12342541	4.23653392	2.27E-05	0.00044849
ENSDARG00000105159	hdac8	1428.21389	1.167394164	0.13874309	8.41407087	3.96E-17	8.95E-15
ENSDARG00000005840	got2b	5212.72237	0.232811753	0.07416734	3.13900644	0.00169522	0.01493606
ENSDARG00000012002	slc38a7	778.447058	0.553284013	0.12786666	4.32703904	1.51E-05	0.00031806
ENSDARG00000006501	cyp2x10.2	35.4265712	-3.548835429	0.56635661	-6.26607933	3.70E-10	2.63E-08
ENSDARG00000091514	CT737190.1	69.9137965	-0.69155271	0.24935923	-2.77331908	0.00554877	0.03685586
ENSDARG00000004714	tcf12	4171.39882	0.248177626	0.07600078	3.26546145	0.00109286	0.01056104
ENSDARG00000077013	znf280d	1369.54484	-0.320693212	0.09581278	-3.34708169	0.00081667	0.00846118
ENSDARG00000035852	cart3	143.554495	-1.028814028	0.31058863	-3.31246519	0.00092478	0.00932267

Table S1. DEGs of WT vs. *terfa+/-*

ENSDARG00000100449	BX322631.1	59.2944568	2.423325834	0.35354943	6.85427734	7.17E-12	6.93E-10
ENSDARG00000104332	cdh28	4.43038158	-5.497172536	1.74705889	-3.14652962	0.00165221	0.01466627
ENSDARG00000104440	cdh30	8.79214353	-2.064233933	0.76319	-2.70474447	0.00683569	0.04291518
ENSDARG00000104530	znf609b	3962.48264	0.373098636	0.10455485	3.56844892	0.0003591	0.0044317
ENSDARG00000103736	gramd2aa	220.806015	-0.482697906	0.17512161	-2.75635831	0.00584489	0.03824028
ENSDARG00000102855	neo1a	4421.87682	0.310440866	0.08944503	3.47074467	0.00051902	0.00593931
ENSDARG00000104342	csnk1g1	1564.80402	0.224838917	0.08169681	2.75211389	0.00592119	0.038577
ENSDARG00000104287	lto1	137.340133	-0.695729319	0.19164511	-3.63030044	0.00028309	0.00368969
ENSDARG00000100420	tpcn2	387.711103	-0.565413211	0.15198725	-3.72013581	0.00019912	0.00276327
ENSDARG00000117533	nadsyn1	103.637094	0.775779983	0.2128495	3.6447348	0.00026767	0.00352751
ENSDARG00000003519	aprt	369.674306	-0.500944518	0.15586405	-3.21398369	0.00130907	0.01217701
ENSDARG00000051853	galns	426.376768	0.710728364	0.20869073	3.40565379	0.00066006	0.00716586
ENSDARG00000079012	cbfa2t3	1402.53496	0.519830534	0.16637146	3.12451741	0.00178097	0.01552104
ENSDARG00000068191	cdh15	770.826359	-0.34947001	0.12603892	-2.77271513	0.00555908	0.03691446
ENSDARG00000078882	slc22a31	1239.19104	0.903629551	0.12474669	7.24371567	4.37E-13	5.22E-11
ENSDARG00000117126	BX664603.1	19.8076398	-1.367554246	0.47964604	-2.85117385	0.00435581	0.03067831
ENSDARG00000077590	arsj	369.697981	-0.984310597	0.17685781	-5.56554777	2.61E-08	1.23E-06
ENSDARG00000036045	penkb	241.64451	-0.48933748	0.16754803	-2.92058032	0.0034938	0.02604632
ENSDARG00000036044	rps20	32918.2752	0.505792388	0.10102697	5.00650848	5.54E-07	1.82E-05
ENSDARG00000104228	lypla1	129.26259	1.603355077	0.21626102	7.4139811	1.23E-13	1.60E-11
ENSDARG00000103744	hacd1	2168.44112	0.327416368	0.10069711	3.25149711	0.00114799	0.01099972
ENSDARG00000099116	nagk	485.270805	0.368548251	0.11689212	3.15289227	0.00161661	0.01440694
ENSDARG00000035544	etnppl	1088.45523	-0.545392844	0.17326445	-3.14774801	0.00164533	0.01461572
ENSDARG00000111788	BX511067.1	26.4884797	8.094042979	1.23729163	6.54174229	6.08E-11	5.04E-09
ENSDARG00000030307	hspa12b	623.539407	-0.412883943	0.12151496	-3.39780334	0.00067929	0.00733081
ENSDARG00000030161	ppp1r14bb	1178.87449	-0.441953904	0.11903721	-3.71273731	0.00020503	0.00282798
ENSDARG00000051955	brms1	448.929099	-0.438883476	0.12275447	-3.57529521	0.00034983	0.00434115
ENSDARG00000051939	pcxb	4661.63949	-0.459141792	0.10515785	-4.36621523	1.26E-05	0.0002749
ENSDARG00000092829	CR589943.1	80.3996638	-2.077784633	0.30999355	-6.70267047	2.05E-11	1.83E-09
ENSDARG00000010925	HTRA3	453.943295	-0.392961383	0.13984931	-2.80989139	0.00495582	0.03376561
ENSDARG00000112670	smim20	280.200516	-0.50863267	0.14592858	-3.4854904	0.00049124	0.00568685
ENSDARG00000052091	rbpjb	401.050982	0.408690438	0.15381677	2.65699536	0.00788405	0.04767838
ENSDARG00000105197	CR318620.1	10.4081345	5.880082818	1.42310746	4.13186144	3.60E-05	0.00066103
ENSDARG00000035751	ipo7	9526.63869	0.199643721	0.06340137	3.14888651	0.00163894	0.01456932
ENSDARG00000104256	bco1	596.685998	-0.517170568	0.16339296	-3.16519495	0.00154979	0.01390114
ENSDARG00000110756	CLEC3A	405.059939	0.575316062	0.1324435	4.34386019	1.40E-05	0.0002992
ENSDARG00000035629	parvab	252.453307	0.511016706	0.15237625	3.35365071	0.00079753	0.00831061
ENSDARG00000035732	arntl1b	883.725458	-0.701569916	0.11448315	-6.12815016	8.89E-10	5.96E-08
ENSDARG00000020252	btbd10b	2243.323	0.336779009	0.0885246	3.804355	0.00014217	0.00209717
ENSDARG00000075833	lyve1a	250.944316	0.576259888	0.20308329	2.83755449	0.00454606	0.03157091
ENSDARG00000059460	sbf2	1783.10073	0.527238273	0.1081544	4.87486671	1.09E-06	3.34E-05
ENSDARG00000102556	nfat5b	235.555826	-0.566238247	0.19823075	-2.85646016	0.00428394	0.0303095
ENSDARG00000099774	cyb5b	1146.33274	0.309886677	0.11191462	2.76895605	0.00562362	0.03720392
ENSDARG00000102850	CU469577.1	134.82469	0.759048819	0.2670526	2.84231952	0.00447866	0.03126883
ENSDARG00000090255	phlpp2	171.741411	-0.86938423	0.23537142	-3.69366944	0.00022104	0.00301205
ENSDARG00000045946	sec24d	2962.40418	0.583850768	0.09305953	6.27394948	3.52E-10	2.51E-08
ENSDARG00000111069	lgi2a	532.291791	-0.382229182	0.13953457	-2.73931522	0.00615673	0.03964393
ENSDARG00000033861	sepsecs	304.838196	-0.422344663	0.142471	-2.96442542	0.00303249	0.02337131
ENSDARG00000026376	aco1	2078.36427	0.592963951	0.12321921	4.81226857	1.49E-06	4.33E-05
ENSDARG00000004015	adcyap1a	128.049513	-0.657605038	0.21761126	-3.02192567	0.00251172	0.02030207
ENSDARG00000088999	mettl4	124.348543	0.787473436	0.23425271	3.36164072	0.00077481	0.00811199
ENSDARG00000006300	psmd9	483.69134	0.651098237	0.15887566	4.09816241	4.16E-05	0.00074626
ENSDARG00000099470	muc5.3	297.088815	1.228492183	0.28138397	4.36589254	1.27E-05	0.00027507
ENSDARG00000088625	si:ch211-142d6.2	5.78611645	-2.891609737	1.02346877	-2.82530334	0.00472359	0.03253593
ENSDARG00000078979	arhgef40	470.140411	0.644758122	0.20233379	3.18660625	0.00143953	0.01313006
ENSDARG00000090371	si:dkey-46i9.6	343.209	-0.401781829	0.13815569	-2.90818164	0.00363537	0.02684407
ENSDARG00000089517	zgc:92594	205.070919	-0.950307908	0.19360326	-4.90853269	9.18E-07	2.83E-05
ENSDARG00000029204	tyrp1a	2429.42042	0.443735128	0.12803789	3.46565489	0.00052894	0.00603066

Table S1. DEGs of WT vs. *terfa+/-*

ENSDARG00000089701	trabd2a	486.775501	0.368479873	0.13337348	2.76276728	0.00573136	0.03769595
ENSDARG00000102788	si:zfos-128g4.1	74.601846	-0.788936806	0.27739555	-2.84408597	0.0044539	0.03117481
ENSDARG00000104647	surf4	1891.77837	0.313480858	0.09441347	3.32029785	0.00089921	0.00914858
ENSDARG00000099226	CABZ01076667.1	2180.87333	0.268629985	0.0881511	3.04738109	0.00230845	0.01905562
ENSDARG00000063126	gusb	1959.98656	0.336527133	0.11163468	3.01453939	0.0025737	0.0207078
ENSDARG00000043589	ca4a	612.421062	-0.759198491	0.19208052	-3.95250132	7.73E-05	0.00125802
ENSDARG00000103846	hspa5	17023.0738	0.899882409	0.08553566	10.520552	6.95E-26	3.39E-23
ENSDARG00000089107	mlxipl	120.004882	-0.917322823	0.23062977	-3.97746923	6.97E-05	0.00115414
ENSDARG00000039887	c1qbp	3024.0627	-0.257372655	0.09352866	-2.75180513	0.00592678	0.03859315
ENSDARG00000017762	srrt	3184.6535	0.311115228	0.10926417	2.84736727	0.00440825	0.03093027
ENSDARG00000104696	apooa	777.275759	0.278477894	0.10068819	2.76574543	0.00567929	0.03748238
ENSDARG00000045979	zgc:153704	1142.01536	-0.390918265	0.12961052	-3.01609978	0.00256049	0.0206158
ENSDARG00000089262	pbx3a	307.635616	-0.420669214	0.1421248	-2.95985794	0.00307781	0.02362537
ENSDARG00000091555	ostf1	827.004558	0.343890281	0.11379308	3.02206678	0.00251055	0.02029921
ENSDARG00000103259	opn4xb	158.699497	-0.817771799	0.26895596	-3.0405416	0.00236153	0.01938436
ENSDARG00000098272	nipbla	3766.08991	0.321341044	0.11173331	2.87596465	0.00402795	0.02896836
ENSDARG00000103351	FP102192.2	8.41786669	4.767002228	1.41162921	3.37695069	0.00073294	0.00778183
ENSDARG00000078214	mybbp1a	2302.21541	-0.32208475	0.09488724	-3.39439458	0.00068781	0.0073942
ENSDARG00000062906	kcnv2b	56.9713518	-1.310375267	0.32579197	-4.02212264	5.77E-05	0.00098457
ENSDARG00000103830	ddr2l	1337.60123	0.528107611	0.15470482	3.41364682	0.000641	0.00702041
ENSDARG00000095464	gstt2	769.477598	-0.402360217	0.12422911	-3.23885611	0.0012001	0.0114067
ENSDARG00000076611	fbxo21	447.569113	-0.509953351	0.11509774	-4.43061146	9.40E-06	0.00021196
ENSDARG00000028346	tesca	268.243763	-0.534697689	0.1978581	-2.70243014	0.00688346	0.04317147
ENSDARG00000005320	nipsnap1	230.285655	-0.625991755	0.17351337	-3.60774367	0.00030887	0.00394501
ENSDARG00000068910	nos1	977.399027	0.842878129	0.19142402	4.40319944	1.07E-05	0.00023799
ENSDARG00000078551	zgc:171242	71.336378	8.842136673	1.25308429	7.05629842	1.71E-12	1.85E-10
ENSDARG00000035631	sdf2l1	1281.25216	0.577097149	0.14357931	4.01936141	5.84E-05	0.00099413
ENSDARG00000101482	hk2	1035.97479	0.550960233	0.0995538	5.53429656	3.12E-08	1.45E-06
ENSDARG00000061994	acacb	3525.76426	-0.314005165	0.11731657	-2.6765627	0.00743816	0.04567085
ENSDARG00000061941	trpv4	527.882536	0.391662348	0.12119435	3.23168813	0.00123061	0.01161674
ENSDARG00000061921	gltpa	392.144836	-0.736771374	0.14033783	-5.24998392	1.52E-07	5.91E-06
ENSDARG00000095754	si:dkey-234h16.7	170.958534	0.821519052	0.23439521	3.50484574	0.00045687	0.00539017
ENSDARG00000056855	gatc	471.552889	-0.492257147	0.14784565	-3.32953425	0.00086991	0.00891617
ENSDARG00000086374	isg15	105.367254	2.333403639	0.38025054	6.13649004	8.44E-10	5.68E-08
ENSDARG00000035603	dao.2	664.056499	-0.987495147	0.18907968	-5.22264011	1.76E-07	6.72E-06
ENSDARG00000035598	coro1ca	4242.42264	0.316940684	0.0860001	3.6853527	0.00022839	0.00309011
ENSDARG00000095509	CR759885.1	63.5832982	-1.088280185	0.29037067	-3.74789986	0.00017832	0.00252258
ENSDARG00000005104	limk2	2029.48898	0.368018211	0.0938028	3.92331776	8.73E-05	0.00138975
ENSDARG00000003281	pik3ip1	1048.14333	-0.490778894	0.1449618	-3.38557387	0.0007103	0.00760638
ENSDARG00000061806	si:dkey-13n15.2	194.541376	0.534464802	0.16974146	3.14869915	0.00163999	0.01457345
ENSDARG00000094417	si:dkey-13n15.2	118.32634	0.664196883	0.23243516	2.85755766	0.00426915	0.03023931
ENSDARG00000035578	hs3st1l2	270.055632	-0.401761733	0.15095784	-2.66141678	0.00778126	0.04719452
ENSDARG00000093945	vma21	1046.78491	-0.396720156	0.14004083	-2.8328892	0.00461294	0.03194819
ENSDARG00000016536	npas2	381.515702	-0.620864968	0.17064042	-3.63844014	0.00027429	0.00359957
ENSDARG00000099624	chmp1b	2591.53356	0.275729985	0.0971688	2.83763912	0.00454485	0.03157091
ENSDARG00000015524	prps1a	4268.36551	0.356431216	0.10463299	3.4064899	0.00065804	0.00715593
ENSDARG00000011929	plp1b	309.332968	-1.008043684	0.22906862	-4.40061893	1.08E-05	0.0002404
ENSDARG00000035555	gbgt1l3	215.82383	-0.766047274	0.2088169	-3.66851195	0.00024397	0.00326889
ENSDARG00000091969	gbgt1l3	24.2776411	-2.07426721	0.53476254	-3.87885661	0.00010495	0.00162828
ENSDARG00000035559	tp53	4381.26849	1.159210993	0.10360548	11.1887036	4.63E-29	3.03E-26
ENSDARG00000028542	si:ch211-114c12.2	3709.05957	-0.377241274	0.07588376	-4.97130449	6.65E-07	2.13E-05
ENSDARG00000030887	phf23a	521.729722	-0.350014782	0.12207517	-2.86720711	0.00414112	0.02957687
ENSDARG00000035563	znf703	2918.65879	0.336177906	0.1152071	2.91803104	0.00352249	0.02623661
ENSDARG00000093639	BX537282.1	10.8665224	5.273869179	1.46845617	3.59143793	0.00032886	0.00413234
ENSDARG00000020465	ewsr1b	10294.2228	-0.469386214	0.12384583	-3.79008488	0.0001506	0.00219921
ENSDARG00000103454	trpm6	659.833982	0.407931915	0.12570352	3.24519081	0.00117372	0.01120307
ENSDARG00000026726	anxa1a	6809.69504	1.515212913	0.15312078	9.89554099	4.35E-23	1.72E-20
ENSDARG00000030311	tmc2b	51.0620271	-1.210330875	0.3008531	-4.02299623	5.75E-05	0.0009816

Table S1. DEGs of WT vs. *terfa+/-*

ENSDARG00000056386	TMC1	37.3046589	-1.172966481	0.35313276	-3.32160203	0.00089502	0.00912835
ENSDARG00000035571	abhd17b	836.367409	0.450094469	0.10751216	4.18645172	2.83E-05	0.00053802
ENSDARG00000061600	cemip2	1477.4997	0.363842731	0.11130102	3.26899725	0.00107929	0.01045842
ENSDARG00000007276	ela3l	792.66516	-3.451471997	0.9000294	-3.83484363	0.00012564	0.00189611
ENSDARG00000027620	bco2l	63.5711758	-0.963209328	0.30257789	-3.18334338	0.00145585	0.01324007
ENSDARG00000004251	dhfr	445.74209	-0.737785291	0.13905885	-5.30556154	1.12E-07	4.53E-06
ENSDARG00000056477	ccdc125	225.413976	0.664454942	0.21310535	3.11796468	0.00182105	0.01574882
ENSDARG00000020952	si:ch211-214j8.1	314.650251	0.761882533	0.26845966	2.83797772	0.00454004	0.03154671
ENSDARG00000004392	hdr	200.582508	-0.51595658	0.17973233	-2.87069426	0.00409571	0.02934317
ENSDARG00000061547	zgc:153409	12.5215722	-2.352363333	0.6633436	-3.54622147	0.0003908	0.00474977
ENSDARG00000035519	histh1l	16293.7978	0.543698642	0.16098214	3.37738495	0.00073179	0.0077762
ENSDARG00000068262	vamp5	764.007691	-0.443630769	0.13702134	-3.23767638	0.00120507	0.01143236
ENSDARG00000097795	zgc:162952	216.18239	-0.821383767	0.19881787	-4.13133766	3.61E-05	0.00066205
ENSDARG00000021948	tnc	17605.0243	0.352425401	0.10613641	3.32049479	0.00089858	0.00914587
ENSDARG00000077960	si:ch211-186e20.7	102.758638	-1.43316212	0.35262444	-4.06427342	4.82E-05	0.00084694
ENSDARG00000091800	zgc:174260	69.1652748	-4.829897319	0.46895741	-10.2992238	7.10E-25	3.04E-22
ENSDARG00000091136	zgc:174259	35.2346021	-2.618851426	0.46260285	-5.66112259	1.50E-08	7.66E-07
ENSDARG00000074712	si:ch211-186e20.2	13.9457762	-2.890401047	0.79542476	-3.63378312	0.0002793	0.00365171
ENSDARG00000056156	npdc1b	1313.89908	-0.306840489	0.11371157	-2.69841055	0.00696715	0.04352059
ENSDARG00000008026	niban2b	970.392117	0.547509552	0.09796436	5.58886484	2.29E-08	1.09E-06
ENSDARG00000076592	man1b1b	599.460075	0.386459821	0.13108641	2.94813038	0.00319702	0.02428545
ENSDARG00000035018	thy1	1054.90264	1.106374756	0.19388913	5.70622377	1.16E-08	6.02E-07
ENSDARG00000061101	snx19a	260.320284	-0.77796666	0.14738879	-5.27832999	1.30E-07	5.19E-06
ENSDARG00000068096	atf5a	1406.39562	-0.31571201	0.09003171	-3.50667583	0.00045374	0.00536379
ENSDARG00000002792	arcn1a	2854.83601	0.349636808	0.08702526	4.01764742	5.88E-05	0.0010007
ENSDARG00000029406	h2ax	902.634701	-0.551581359	0.1614183	-3.4170932	0.00063294	0.00695081
ENSDARG00000091996	tcnbb	6609.71424	0.503805673	0.13244379	3.80392068	0.00014242	0.00209961
ENSDARG00000011841	atp5l	8009.11283	0.58594904	0.10380278	5.64483005	1.65E-08	8.26E-07
ENSDARG00000093921	BX276103.2	43.5120075	-3.606851545	0.44617045	-8.08402156	6.27E-16	1.17E-13
ENSDARG00000035471	si:dkey-220k22.1	892.377057	0.321077783	0.09770844	3.28608024	0.00101592	0.00999237
ENSDARG00000010031	ssh1b	1150.31316	-0.349034247	0.12493441	-2.79373987	0.00521024	0.03510452
ENSDARG00000035438	myhc4	6043.71208	1.423730414	0.16395671	8.68357502	3.84E-18	1.00E-15
ENSDARG00000016319	c9	6476.57822	0.662714927	0.11211542	5.91100605	3.40E-09	1.99E-07
ENSDARG00000067984	gas1b	653.001637	-0.455274495	0.12666736	-3.59425266	0.00032532	0.00410662
ENSDARG00000040248	crata	1690.87608	-0.288299727	0.09329329	-3.09025134	0.00199987	0.01702288
ENSDARG00000083894	CR925757.1	3.82830916	4.666464811	1.56984905	2.97255638	0.00295331	0.02292458
ENSDARG00000093572	lamc3	3953.12512	0.251992961	0.09220043	2.73309955	0.00627414	0.04020203
ENSDARG00000009336	aif1l	1457.39571	-0.302284276	0.10563468	-2.8616006	0.00421508	0.02998447
ENSDARG00000034368	hexb	3461.15203	0.358056157	0.12373918	2.89363613	0.00380809	0.02776478
ENSDARG00000035398	enc1	2825.18634	0.280220178	0.08898242	3.14916354	0.00163739	0.01456593
ENSDARG00000079403	si:dkey-204l11.1	189.549299	3.707554377	0.32680579	11.3448248	7.87E-30	5.75E-27
ENSDARG00000035329	capns1a	3759.6285	0.482546168	0.10017461	4.81705044	1.46E-06	4.24E-05
ENSDARG00000053716	zgc:153990	849.960259	0.390615346	0.14190565	2.75264134	0.00591166	0.03854293
ENSDARG00000035324	hnrnpl	9351.30069	0.379643079	0.13529549	2.80602906	0.00501562	0.03408891
ENSDARG00000100476	si:dkeyp-110c7.4	188.374776	-0.85349521	0.24132492	-3.53670569	0.00040515	0.00488362
ENSDARG00000039436	il13ra2	236.270656	-0.892853233	0.18240612	-4.89486451	9.84E-07	3.03E-05
ENSDARG00000020924	myo1ca	787.938665	0.447245595	0.11320081	3.95090468	7.79E-05	0.00126478
ENSDARG00000035350	ins	645.329928	0.526912819	0.10175601	5.17819857	2.24E-07	8.34E-06
ENSDARG00000092354	si:ch211-284e13.6	1041.51127	-0.826679805	0.11427373	-7.23420684	4.68E-13	5.55E-11
ENSDARG00000060366	slc12a9	668.709487	0.688597599	0.16043064	4.29218262	1.77E-05	0.00036253
ENSDARG00000034424	atp1b2b	1337.96766	-0.578865241	0.16202939	-3.57259413	0.00035346	0.00437515
ENSDARG00000003635	mogat3b	412.015035	-0.432230784	0.15691753	-2.75450916	0.00587802	0.03838631
ENSDARG00000060526	bmp3	621.365736	-0.350749448	0.12819019	-2.73616458	0.00621599	0.03991178
ENSDARG00000033614	rasgef1ba	1712.41726	0.304310792	0.09194983	3.30953066	0.00093453	0.00937354
ENSDARG00000019549	cds1	857.098554	-0.398067671	0.12673795	-3.140872	0.00168446	0.01487288
ENSDARG00000052846	fsta	1244.53588	0.378130405	0.08732336	4.33023179	1.49E-05	0.00031455
ENSDARG00000027109	zfr	8892.01468	0.313282966	0.09448093	3.31583293	0.0009137	0.00923645
ENSDARG00000007720	sub1b	699.440121	-0.382208738	0.1154338	-3.31106426	0.00092942	0.00934116

Table S1. DEGs of WT vs. *terfa+/-*

ENSDARG00000076830	si:dkey-65b12.6	5373.91397	0.8360459	0.12230471	6.83576203	8.16E-12	7.86E-10
ENSDARG00000029692	rufy3	1620.49676	-0.28545065	0.0848883	-3.36266182	0.00077195	0.00808545
ENSDARG00000103167	si:dkey-245n4.2	250.560347	-0.639900394	0.15264982	-4.19194988	2.77E-05	0.00052918
ENSDARG00000117211	CR936462.1	118.455166	0.58940845	0.22306982	2.64225998	0.00823548	0.04918138
ENSDARG00000095369	zgc:112966	8.82902762	6.656928977	1.37662711	4.83568059	1.33E-06	3.95E-05
ENSDARG00000075126	TMEM8B	494.961314	0.466189815	0.14354854	3.24761102	0.00116378	0.01111676
ENSDARG00000007836	ctsla	27813.5047	0.427966463	0.13505939	3.168728	0.00153108	0.01376805
ENSDARG00000021366	fbp1a	724.719233	0.851741198	0.162295	5.24810499	1.54E-07	5.95E-06
ENSDARG00000015072	dmrt2a	628.588704	-0.611999553	0.16130181	-3.79412694	0.00014816	0.00217007
ENSDARG00000008904	smarca2	1510.08547	0.489621932	0.10435601	4.69184239	2.71E-06	7.24E-05
ENSDARG00000060127	adamts3	542.077381	0.501309501	0.13560215	3.69691405	0.00021824	0.002982
ENSDARG00000103515	vcana	141.481666	0.837070563	0.21382945	3.91466446	9.05E-05	0.00143503
ENSDARG00000104537	cox7c	4187.08015	0.703833523	0.103148	6.82353055	8.88E-12	8.49E-10
ENSDARG00000009418	mef2cb	2429.85244	-0.277118084	0.09133035	-3.03423879	0.00241143	0.01965766
ENSDARG00000052690	arrdc3a	3893.78792	0.593109734	0.12005651	4.94025462	7.80E-07	2.45E-05
ENSDARG00000077072	si:ch73-280o22.2	282.462189	0.953542086	0.15297029	6.23351183	4.56E-10	3.21E-08
ENSDARG00000059714	arsk	52.4086965	2.785238978	0.46461069	5.99478028	2.04E-09	1.26E-07
ENSDARG00000067670	pomt1	590.32333	0.583599352	0.15761509	3.70268698	0.00021333	0.00292781
ENSDARG00000067672	card9	58.5253396	-1.744679517	0.39675081	-4.3974189	1.10E-05	0.00024332
ENSDARG00000067673	snapc4	535.384111	-0.513321889	0.11512909	-4.45866369	8.25E-06	0.0001891
ENSDARG00000051914	slc14a2	99.4523869	2.296027018	0.26775009	8.57526153	9.89E-18	2.43E-15
ENSDARG00000098198	NA	4.31455102	-5.204470483	1.50780263	-3.45169214	0.00055708	0.00628235
ENSDARG00000104980	PPEF2	256.75181	-1.058908547	0.19272302	-5.49445797	3.92E-08	1.79E-06
ENSDARG00000102227	CR450749.3	24.2640951	3.012670487	0.61785615	4.87600628	1.08E-06	3.32E-05
ENSDARG00000100293	arhgef15	55.3375623	-2.517648794	0.37877	-6.64690653	2.99E-11	2.59E-09
ENSDARG00000101300	ssna1	135.569289	-0.822928929	0.19630902	-4.19200768	2.76E-05	0.00052918
ENSDARG00000007034	hnrpkl	2839.50165	0.402135455	0.088472	4.54534129	5.48E-06	0.00013378
ENSDARG00000005372	camk4	341.104074	-0.432507755	0.14954918	-2.89207712	0.00382704	0.02784575
ENSDARG00000102200	man2a1	346.547509	0.609407949	0.16377988	3.7208964	0.00019852	0.00275958
ENSDARG00000052462	pisd	1171.82734	-0.356509541	0.10773153	-3.30924046	0.00093549	0.00937947
ENSDARG00000052437	mia	945.229451	-0.428289766	0.14582606	-2.93699061	0.00331414	0.02495185
ENSDARG00000095750	fdxacb1	196.153045	-0.502076208	0.17118553	-2.93293604	0.00335773	0.0252058
ENSDARG00000039626	nrgna	533.747938	-0.813082398	0.18399446	-4.41905903	9.91E-06	0.00022258
ENSDARG00000099091	CU929418.1	18.7904646	1.395526161	0.49735459	2.80589781	0.00501766	0.03409348
ENSDARG00000077107	oafb	99.5350148	-0.583264031	0.2213291	-2.63527952	0.0084068	0.04997634
ENSDARG00000094097	CR936321.5	251.255715	-0.466252608	0.15789876	-2.95285795	0.00314847	0.02400465
ENSDARG00000035152	ap2b1	7444.02689	0.284016138	0.09219666	3.08054693	0.00206621	0.01745602
ENSDARG00000057245	im:7138535	239.965111	-0.647991573	0.15443737	-4.1958212	2.72E-05	0.00052304
ENSDARG00000088069	CR759879.1	112.945823	1.609856411	0.31485149	5.11306587	3.17E-07	1.13E-05
ENSDARG00000059553	sympk	2562.3944	0.357941844	0.13547894	2.64204783	0.00824064	0.04920037
ENSDARG00000035136	selenow1	7073.88666	0.29427949	0.10933541	2.69152961	0.00711252	0.04419541
ENSDARG00000035133	pho	371.225374	-0.542953867	0.17729122	-3.06249728	0.00219498	0.01829522
ENSDARG00000111249	CR394528.1	168.260626	1.238570809	0.23049348	5.37356104	7.72E-08	3.27E-06
ENSDARG00000045262	gsnb	3361.51425	0.503018277	0.15763722	3.19098667	0.00141788	0.01299455
ENSDARG00000035131	surf4l	1039.72261	0.77274292	0.11914925	6.48550383	8.84E-11	7.14E-09
ENSDARG00000103712	plpp7a	691.631042	-0.415660701	0.13102129	-3.17246674	0.0015115	0.01363641
ENSDARG00000052155	lrrc8ab	934.03569	0.546207462	0.10633302	5.13676251	2.80E-07	1.02E-05
ENSDARG00000026979	krt1-c5	646.824763	-0.625788239	0.14820211	-4.22253245	2.42E-05	0.00047221
ENSDARG00000098481	anxa1d	410.717605	0.83247342	0.16939257	4.91446249	8.90E-07	2.76E-05
ENSDARG00000104359	anxa1c	1685.18778	0.997305903	0.15189647	6.56569515	5.18E-11	4.36E-09
ENSDARG00000104555	sh2d3cb	168.240529	-0.769261609	0.17192167	-4.47448884	7.66E-06	0.00017814
ENSDARG00000098673	BX001023.1	90.2527365	-0.780444103	0.29144369	-2.67785553	0.00740952	0.04553995
ENSDARG00000052094	notch1b	3168.87192	0.407770213	0.15115659	2.69766748	0.00698272	0.04357404
ENSDARG00000019752	rom1a	986.734433	-0.942113155	0.22751183	-4.14094131	3.46E-05	0.00064061
ENSDARG00000059472	arhgap31	797.721999	-0.43509895	0.13118585	-3.31666074	0.000911	0.00922682
ENSDARG00000099111	zgc:175280	208.956552	-0.866892184	0.18621947	-4.65521787	3.24E-06	8.38E-05
ENSDARG00000059466	wasf3a	287.105717	-0.626461709	0.17438543	-3.59239705	0.00032765	0.00412341
ENSDARG00000011555	spag7	2569.37796	-0.229472482	0.08333533	-2.75360365	0.00589431	0.03845225

Table S1. DEGs of WT vs. *terfa+/-*

ENSDARG00000059461	mepce	692.120967	-0.382101675	0.09352562	-4.08552947	4.40E-05	0.00078186
ENSDARG00000076888	GANAB	1964.08007	0.51887666	0.10635321	4.87880564	1.07E-06	3.28E-05
ENSDARG00000037121	mat2ab	735.026025	0.659335194	0.13124261	5.02378926	5.07E-07	1.68E-05
ENSDARG00000059442	smtnb	1475.93346	-0.65117578	0.14991417	-4.34365737	1.40E-05	0.00029922
ENSDARG00000102320	mob1a	1268.86778	-0.39139259	0.11657795	-3.35734673	0.00078694	0.00821827
ENSDARG00000098646	mthfd2	1448.51013	0.524871754	0.12097381	4.33872221	1.43E-05	0.00030445
ENSDARG00000105036	arl6ip4	403.134601	-0.425128227	0.12378644	-3.43436828	0.00059394	0.00659031
ENSDARG00000101090	pappaa	462.597773	0.69460024	0.19172001	3.62299295	0.00029121	0.00376397
ENSDARG00000032584	thnsl2	81.3879376	-0.889247094	0.3003463	-2.96073925	0.00306902	0.02357244
ENSDARG00000040912	kdelr3	917.259465	0.77773558	0.14737989	5.27708089	1.31E-07	5.21E-06
ENSDARG00000088820	zgc:174288	338.250394	0.613512021	0.14503575	4.23007453	2.34E-05	0.00045901
ENSDARG00000088022	mhc1zfa	16.8728187	-3.570175462	0.73839751	-4.83503184	1.33E-06	3.95E-05
ENSDARG00000039752	si:dkey-167k11.5	440.211127	0.729057719	0.20520351	3.5528521	0.00038108	0.00465898
ENSDARG00000115199	dicp1.3-4	25.7784091	-2.384818619	0.54826831	-4.34972912	1.36E-05	0.00029256
ENSDARG00000086569	zgc:172051	245.983006	-0.856726397	0.27720634	-3.09057285	0.00199771	0.01701029
ENSDARG00000075173	si:ch1073-322p19.1	961.380286	0.498303037	0.14713484	3.38670999	0.00070736	0.00758476
ENSDARG00000063438	srebf2	5293.8159	0.550419717	0.1160133	4.74445352	2.09E-06	5.83E-05
ENSDARG00000038028	ndufa6	3895.96266	0.42909244	0.10629227	4.03691091	5.42E-05	0.00093482
ENSDARG00000095826	smdt1a	32.6506953	1.719813826	0.46278936	3.71619141	0.00020225	0.00279893
ENSDARG00000044852	wbp2nl	2906.24796	0.268406403	0.09005094	2.98060647	0.00287678	0.02244974
ENSDARG00000035088	si:ch211-254c8.3	14.7286787	6.857360686	1.33159473	5.14973552	2.61E-07	9.55E-06
ENSDARG00000086048	si:ch211-229i14.2	7.76809763	3.303374899	1.07693034	3.06739886	0.00215931	0.01805231
ENSDARG00000074764	zgc:171426	24.776167	-3.122607836	0.66661951	-4.68424306	2.81E-06	7.47E-05
ENSDARG00000038392	si:dkeyp-52c3.7	13.8644516	7.256304499	1.46087168	4.96710602	6.80E-07	2.17E-05
ENSDARG00000093476	si:dkey-175d9.2	18.1308049	-3.881496013	0.77421094	-5.01348638	5.35E-07	1.76E-05
ENSDARG00000095739	si:dkey-73p2.1	6.20502952	-3.989318023	1.31483567	-3.03408108	0.0024127	0.01966149
ENSDARG00000098540	zc3h7ba	477.465555	0.340182215	0.11802204	2.88236179	0.00394706	0.02849382
ENSDARG00000076106	ftr43	30.4745508	2.728747488	0.49709796	5.48935561	4.03E-08	1.83E-06
ENSDARG00000115041	ftr50	4.32939693	-4.898303802	1.45424675	-3.36827557	0.0007564	0.00797299
ENSDARG00000001014	myh9b	17026.2389	0.307950681	0.09362795	3.28908926	0.00100512	0.00990087
ENSDARG00000034777	txn2	2030.47025	0.319930162	0.09980598	3.20552102	0.00134818	0.01246617
ENSDARG00000100246	FP016234.1	257.367904	-1.275138796	0.28666415	-4.44819766	8.66E-06	0.0001971
ENSDARG00000116871	BX284638.2	5592.82349	0.55254861	0.12861491	4.29614752	1.74E-05	0.00035788
ENSDARG00000104099	FP236513.1	225.519698	0.791411297	0.17731557	4.46329277	8.07E-06	0.00018643
ENSDARG00000098397	FP016106.1	65.3160591	4.351407722	1.17529337	3.70240133	0.00021357	0.00292949
ENSDARG00000103413	zgc:109949	668.469045	-0.677916528	0.13075443	-5.1846544	2.16E-07	8.09E-06
ENSDARG00000104773	junbb	2607.1309	-0.699890028	0.15989812	-4.37709988	1.20E-05	0.00026384
ENSDARG00000116743	rnps1	118.875275	-0.940394795	0.27311077	-3.44327248	0.00057472	0.00642827
ENSDARG00000058819	nog1	276.622924	-0.433547464	0.159453	-2.71896716	0.00654861	0.04149012
ENSDARG00000100113	AL935044.2	17.3133987	1.994598474	0.73908271	2.69874867	0.00696007	0.0434885
ENSDARG00000040387	mmd	1074.83079	0.358603223	0.10272103	3.49104013	0.00048114	0.00559798
ENSDARG00000089616	coro7	753.269915	0.586878171	0.12779852	4.59221407	4.39E-06	0.00011
ENSDARG00000100524	abca3b	8777.79889	0.315386286	0.09685204	3.25637229	0.00112846	0.01084182
ENSDARG00000100376	amdhd2	1031.8009	0.761398319	0.18280301	4.1651301	3.11E-05	0.00058284
ENSDARG00000104847	ptger1b	80.6880605	-0.80632344	0.24310606	-3.31675586	0.00091069	0.00922682
ENSDARG00000099695	si:dkey-61n16.5	285.835244	-0.513209845	0.19209537	-2.67164091	0.00754814	0.04615226
ENSDARG00000087188	nfil3-6	391.751266	1.269147779	0.1658056	7.65443252	1.94E-14	2.91E-12
ENSDARG00000097615	si:ch211-108d22.2	734.842813	-0.743110091	0.15704704	-4.73176746	2.23E-06	6.12E-05
ENSDARG00000101534	rab3db	118.373486	0.751042428	0.22326574	3.36389465	0.00076851	0.00806302
ENSDARG00000039338	gadd45gip1	1014.93842	-0.353518122	0.09883689	-3.57678309	0.00034785	0.00432505
ENSDARG00000041217	xpo6	1883.91991	0.276734815	0.08626011	3.20814355	0.00133595	0.01238068
ENSDARG00000044767	tspan10	107.010742	0.560852282	0.20871571	2.68715898	0.00720626	0.0446263
ENSDARG00000070845	si:dkey-56d12.4	54.6307164	3.626765412	0.46340788	7.82629203	5.02E-15	8.11E-13
ENSDARG00000011925	spns1	2991.30713	-0.386906038	0.11907363	-3.24930071	0.00115689	0.0110589
ENSDARG00000094557	nupr1b	1911.69649	-0.839795528	0.14816264	-5.66806542	1.44E-08	7.42E-07
ENSDARG00000026453	zgc:66474	828.609592	-0.411373948	0.09806302	-4.19499553	2.73E-05	0.00052376
ENSDARG00000057707	zgc:66443	322.569613	-0.346250402	0.13128325	-2.63743016	0.00835368	0.04975576
ENSDARG00000021242	mvp	4267.65807	0.439389973	0.11139369	3.94447795	8.00E-05	0.00129159

Table S1. DEGs of WT vs. *terfa+/-*

ENSDARG00000077737	spsb3a	1152.79528	-0.426056088	0.1025639	-4.15405513	3.27E-05	0.00060813
ENSDARG00000057867	lasp1	5234.91361	0.247490493	0.07638036	3.24023723	0.0011943	0.01136028
ENSDARG00000073998	si:ch211-23l10.3	124.103131	-0.887540184	0.20089309	-4.41797272	9.96E-06	0.0002235
ENSDARG00000092120	BX470185.1	45.5032746	1.635683735	0.588199	2.78083391	0.00542195	0.03614874
ENSDARG00000022712	stat3	1992.59983	0.562151379	0.14197366	3.95954696	7.51E-05	0.0012271
ENSDARG00000062565	kcnh4a	90.6677235	0.708575322	0.23018822	3.07824316	0.00208225	0.01754019
ENSDARG00000099079	aclya	5255.83433	0.241413786	0.0785046	3.07515446	0.00210394	0.01769656
ENSDARG00000076490	ankrd40	1017.1144	0.242714139	0.09111014	2.66396408	0.00772258	0.0469647
ENSDARG00000087327	cbx8a	327.962757	-0.372939039	0.13841136	-2.69442517	0.00705102	0.04389006
ENSDARG00000089749	aqp8b	420.70948	0.567560344	0.15145037	3.74750054	0.00017861	0.00252516
ENSDARG00000070780	rln3a	27.9138337	1.391382095	0.44035955	3.15965009	0.00157959	0.01411745
ENSDARG00000056791	pde6ga	37.1102806	1.524048293	0.44069512	3.45828268	0.00054363	0.00615578
ENSDARG00000056767	itgb3a	1632.41289	0.557654455	0.10999631	5.06975583	3.98E-07	1.38E-05
ENSDARG00000035066	ubtf	4779.2146	-0.436632065	0.08392066	-5.20291499	1.96E-07	7.40E-06
ENSDARG00000089936	selenow2b	1558.65175	0.565552321	0.11618303	4.86777058	1.13E-06	3.44E-05
ENSDARG00000038213	slc35b1	1469.36486	0.339802531	0.09172906	3.70441508	0.00021188	0.00290953
ENSDARG00000101826	zwi	562.501997	-0.803316238	0.18166989	-4.42184578	9.79E-06	0.00022012
ENSDARG00000061858	zgc:153968	154.702092	-3.728819987	0.36089243	-10.33222	5.04E-25	2.20E-22
ENSDARG00000007344	tcap	792.784262	1.195748883	0.20317279	5.88537891	3.97E-09	2.29E-07
ENSDARG00000075249	fam171a2a	1958.65713	0.235559047	0.08530739	2.76129697	0.00575723	0.03780867
ENSDARG00000038185	gh1	178.35369	-1.309203284	0.23360813	-5.60427105	2.09E-08	1.01E-06
ENSDARG00000079097	tanc2a	2975.85843	0.330084828	0.10387298	3.1777738	0.0014841	0.01344784
ENSDARG00000075139	hdac5	1182.25774	0.490951586	0.13837514	3.54797535	0.0003882	0.00472982
ENSDARG00000010680	gngt2a	1274.88937	-1.75026119	0.59521583	-2.94054878	0.00327631	0.02470655
ENSDARG00000096994	AL929222.1	11.9210008	-2.073728224	0.60464645	-3.42965417	0.00060435	0.00669989
ENSDARG00000012405	col1a1a	170262.545	0.554988867	0.12067933	4.59887267	4.25E-06	0.00010697
ENSDARG00000055813	si:dkey-225f5.5	84.4879836	1.540479107	0.2840896	5.42251141	5.88E-08	2.53E-06
ENSDARG00000033160	nr1d1	1847.35347	2.597153902	0.20644769	12.5802034	2.71E-36	3.55E-33
ENSDARG00000038025	cbx7a	656.824383	1.247631817	0.23771795	5.24837029	1.53E-07	5.95E-06
ENSDARG00000038030	rbx1	1737.31157	0.732730091	0.12667565	5.78430108	7.28E-09	3.98E-07
ENSDARG00000038059	grb2b	2226.9229	0.260254696	0.08653826	3.00739445	0.00263498	0.02104509
ENSDARG00000022525	mchr1b	123.823147	3.561619953	0.87365729	4.07667859	4.57E-05	0.00080989
ENSDARG00000022531	ntn1b	1835.99551	0.545930147	0.13940311	3.91619782	9.00E-05	0.00142742
ENSDARG00000027529	hmox1a	860.866384	1.204068919	0.18902081	6.37003358	1.89E-10	1.42E-08
ENSDARG00000029688	hsp70.1	49.5235359	-1.178111483	0.32489579	-3.6261211	0.00028771	0.00373125
ENSDARG00000087869	si:ch211-11k18.4	1274.36661	0.370580649	0.13321307	2.7818641	0.00540477	0.03607467
ENSDARG00000055510	ypel3	648.896294	-0.407040389	0.13299866	-3.06048487	0.00220979	0.01838163
ENSDARG00000010160	rps15a	21523.6697	0.401217454	0.09459001	4.24164736	2.22E-05	0.00043979
ENSDARG00000038094	clec16a	1441.83287	-0.244075576	0.08580184	-2.84464264	0.00444613	0.03113792
ENSDARG00000087093	si:ch211-157c3.4	797.496631	0.610920043	0.18381086	3.32363418	0.00088853	0.00906593
ENSDARG00000004034	arhgdig	3692.24263	0.298135798	0.085669	3.48008962	0.00050125	0.00577851
ENSDARG00000070522	cacna1ia	140.348127	1.041389445	0.28066391	3.71045016	0.00020689	0.00285207
ENSDARG00000003599	rpl3	82904.8536	0.24189136	0.08496453	2.84696879	0.00441377	0.03095492
ENSDARG00000083593	BX901883.1	10.7479036	2.832042682	0.90030682	3.14564172	0.00165723	0.0146898
ENSDARG00000015128	rpl27	24302.1551	0.632822555	0.10030755	6.30882255	2.81E-10	2.06E-08
ENSDARG00000054849	bcat2	2355.40305	-0.322848205	0.08658172	-3.7288265	0.00019237	0.00269225
ENSDARG00000070465	si:dkey-13n23.3	38.5064438	-2.278568217	0.48861292	-4.66334005	3.11E-06	8.12E-05
ENSDARG00000054645	si:ch211-51c14.1	1001.25642	-0.350914164	0.0999163	-3.51208115	0.00044461	0.00527486
ENSDARG00000075461	kmt5c	2140.40219	-0.266805675	0.09292139	-2.87130512	0.00408781	0.02930558
ENSDARG00000054616	cldni	9077.57262	0.526351055	0.10225222	5.14757566	2.64E-07	9.63E-06
ENSDARG00000097740	elob	204.992698	-0.546134986	0.18905363	-2.88878344	0.00386735	0.02805867
ENSDARG00000037980	elob	4188.97599	0.306821044	0.08780156	3.49448286	0.00047498	0.00555299
ENSDARG00000054578	arl6ip1	4161.53689	0.207598929	0.07683045	2.70203958	0.00689156	0.04321131
ENSDARG00000090664	moto	8.4008549	-2.445241212	0.89325151	-2.73746104	0.00619155	0.03980622
ENSDARG00000054438	fzd2	3525.50074	-0.286670338	0.08622177	-3.3248021	0.00088481	0.00903535
ENSDARG00000025301	gfap	8898.89206	-0.652673027	0.10241335	-6.37292911	1.85E-10	1.40E-08
ENSDARG00000078092	limd2	1024.51326	0.520240183	0.12390513	4.19869778	2.68E-05	0.00051764
ENSDARG00000016908	zp3e	20.7767433	-1.565554465	0.50305118	-3.11211767	0.0018575	0.01600842

Table S1. DEGs of WT vs. *terfa+/-*

ENSDARG00000030881	baxb	116.372343	-0.71779226	0.25292185	-2.83800023	0.00453972	0.03154671
ENSDARG00000076221	fthl28	697.763581	1.327855224	0.17409846	7.62703604	2.40E-14	3.58E-12
ENSDARG00000077360	fthl30	161.595262	0.903828406	0.31485406	2.87062649	0.00409659	0.02934317
ENSDARG00000113977	fthl29	419.88221	-0.692105863	0.23062035	-3.0010615	0.0026904	0.02137103
ENSDARG00000113150	fthl31	17.2618642	5.933229224	1.32269209	4.48572215	7.27E-06	0.00017039
ENSDARG00000037943	cpt1cb	519.122626	0.555610322	0.14388089	3.86159922	0.00011265	0.00172617
ENSDARG00000096849	si:dkey-16p21.8	7869.85559	0.472201576	0.0922329	5.11966537	3.06E-07	1.09E-05
ENSDARG00000010246	prmt1	6772.58062	-0.311610082	0.08431981	-3.69557365	0.00021939	0.00299284
ENSDARG00000059993	trpm4a	1519.14758	0.645380275	0.11433245	5.64476913	1.65E-08	8.26E-07
ENSDARG00000037961	rcn3	2803.13451	1.118253868	0.13151925	8.50258691	1.85E-17	4.39E-15
ENSDARG00000036966	hsd3b7	831.147945	-0.712364837	0.15148269	-4.70261546	2.57E-06	6.94E-05
ENSDARG00000053260	zgc:113090	103.187342	-0.756225234	0.25126474	-3.00967508	0.00261527	0.02094156
ENSDARG00000070371	kat7a	589.740828	-0.637545414	0.11616885	-5.48809288	4.06E-08	1.84E-06
ENSDARG00000099195	ier2a	1122.61497	-0.601879343	0.14477178	-4.15743566	3.22E-05	0.0006001
ENSDARG00000015538	znf207a	2420.43244	0.466511481	0.10140717	4.60037983	4.22E-06	0.00010652
ENSDARG00000037914	znfl1	11.5130843	-2.00821386	0.75942078	-2.64440204	0.00818354	0.0489182
ENSDARG00000037916	cdk5r1a	314.531142	-0.387627436	0.14383312	-2.69498044	0.00703928	0.04383894
ENSDARG00000037919	rbbp6	3115.91216	-0.341321401	0.08116502	-4.20527731	2.61E-05	0.00050556
ENSDARG00000040072	scpep1	1200.05002	0.641745718	0.14844844	4.32302084	1.54E-05	0.00032254
ENSDARG00000090369	zgc:86896	10737.145	0.323159435	0.08201552	3.94022304	8.14E-05	0.00130961
ENSDARG00000060246	slc16a6b	1613.67421	0.453945855	0.13828596	3.28266045	0.00102832	0.01005864
ENSDARG00000104413	rogdi	1738.67228	-0.329694661	0.1082862	-3.04465986	0.00232944	0.01918395
ENSDARG00000104457	si:dkeyp-72e1.7	298.574203	-0.498457945	0.16879048	-2.95311643	0.00314583	0.02399192
ENSDARG00000094901	abcc6b.2	271.79595	2.584216155	0.24802391	10.4192217	2.03E-25	9.16E-23
ENSDARG00000096233	si:dkey-260c8.6	54.8130475	0.981340892	0.34002812	2.88605805	0.003901	0.02824334
ENSDARG00000097966	BX936420.1	12.5539964	-2.38041261	0.62229734	-3.82520135	0.00013067	0.00196353
ENSDARG00000094994	si:dkey-106c17.2	26.6112067	3.296786106	0.69407423	4.74990423	2.04E-06	5.68E-05
ENSDARG00000004748	zgc:100868	6205.75026	0.485853347	0.08667865	5.60522492	2.08E-08	1.01E-06
ENSDARG00000052905	zgc:165423	1282.12092	1.195857523	0.37701414	3.17191693	0.00151436	0.01363652
ENSDARG00000090340	si:dkey-27o4.1	175.038525	-0.803361952	0.21540163	-3.72960017	0.00019178	0.00268551
ENSDARG00000037870	actb2	190902.734	0.659296527	0.08114278	8.12514112	4.47E-16	8.41E-14
ENSDARG00000037873	cyp3c3	52.7681377	1.955900451	0.43724272	4.47326022	7.70E-06	0.00017878
ENSDARG00000037874	cyp3c3	31.4109738	4.889922196	0.79111029	6.18108785	6.37E-10	4.35E-08
ENSDARG00000097269	si:ch211-222n22.1	36.5259157	1.090904712	0.38752521	2.81505485	0.00487689	0.03334666
ENSDARG00000033382	grifin	311.934342	-1.406298202	0.25931316	-5.42316559	5.86E-08	2.53E-06
ENSDARG00000037879	lfng	2395.87113	-0.313872889	0.11002131	-2.85283725	0.00433308	0.03052685
ENSDARG00000097799	zgc:194207	45.0544677	1.071312831	0.4040516	2.65142581	0.00801527	0.04820638
ENSDARG00000097610	BX005183.3	64.0718494	-1.377331765	0.43421675	-3.17199134	0.00151398	0.01363652
ENSDARG00000062304	pdpk1a	345.323997	-0.54297903	0.13964263	-3.88834709	0.00010093	0.00157279
ENSDARG00000052649	ube2ia	1544.88214	-0.368891185	0.11122797	-3.31653264	0.00091142	0.00922682
ENSDARG00000037838	unkl	2080.82523	-0.444219605	0.08285292	-5.3615446	8.25E-08	3.45E-06
ENSDARG00000020326	tyk2	441.227159	0.394925672	0.14280937	2.76540447	0.00568523	0.03750167
ENSDARG00000090120	si:dkey-248g21.1	106.174219	1.055235578	0.24643097	4.28207375	1.85E-05	0.00037722
ENSDARG00000004470	prkcsh	3772.0818	0.269549587	0.09837685	2.73996977	0.00614448	0.0396061
ENSDARG00000089292	adgrl1a	493.305911	0.63357686	0.21043572	3.01078573	0.00260573	0.02088531
ENSDARG00000099149	hs3st3b1a	155.785457	-0.918891517	0.25688472	-3.57705792	0.00034748	0.00432267
ENSDARG00000101749	zgc:92161	71.7048125	-1.233852807	0.30897348	-3.99339384	6.51E-05	0.00109018
ENSDARG00000099383	dnajb1a	459.900886	0.448364006	0.12811837	3.49960736	0.00046594	0.00546275
ENSDARG00000104436	zgc:153426	11761.0628	0.701422905	0.16058779	4.36784714	1.25E-05	0.00027333
ENSDARG00000038768	mrpl12	2151.54302	-0.245563885	0.08746371	-2.8076087	0.00499108	0.03395518
ENSDARG00000078953	epn3a	1316.70377	0.332322428	0.1000631	3.32112849	0.00089654	0.00913635
ENSDARG00000097976	CR812464.1	53.7889616	-1.23050741	0.38054993	-3.23349793	0.00122284	0.01155656
ENSDARG00000010727	ttyh2l	887.078583	-0.402206896	0.12365972	-3.25252964	0.00114383	0.01096406
ENSDARG00000096946	BX897682.1	32.5447335	4.194948165	0.82879291	5.0615155	4.16E-07	1.43E-05
ENSDARG00000090871	si:dkey-210j14.4	331.536511	-0.420557764	0.14373448	-2.92593503	0.00343423	0.02567921
ENSDARG00000091754	CT573476.1	217.10951	-0.595186178	0.19583122	-3.03928141	0.00237143	0.01944318
ENSDARG00000078904	brd4	3134.14161	0.246867962	0.09071769	2.72127688	0.00650303	0.0413066
ENSDARG00000075015	soul5	865.948368	-0.644829695	0.15460201	-4.17090099	3.03E-05	0.00057136

Table S1. DEGs of WT vs. *terfa+/-*

ENSDARG00000004796	mgrn1b	1854.19131	-0.323396538	0.0926968	-3.48875605	0.00048527	0.00563157
ENSDARG00000079802	aanat2	125.701641	-1.067159877	0.20227747	-5.27572289	1.32E-07	5.23E-06
ENSDARG00000036541	rhbdf1a	2243.49311	0.269451141	0.08048609	3.34779744	0.00081457	0.00845345
ENSDARG00000115405	hbbe1.2	11174.66	0.845233708	0.15808814	5.34659797	8.96E-08	3.71E-06
ENSDARG00000088330	hbae1.3	473.468838	1.463456779	0.2206356	6.63291312	3.29E-11	2.82E-09
ENSDARG00000087390	hbbe1.3	11687.9453	0.865277937	0.26966938	3.20866218	0.00133354	0.01236536
ENSDARG00000089124	hbae1.3	2206.87429	0.586140242	0.16571551	3.53702707	0.00040466	0.00488005
ENSDARG00000079305	hbae3	45969.5921	0.447879062	0.14936632	2.99852787	0.00271287	0.02149457
ENSDARG00000013997	ern1	2167.80856	0.703439272	0.12954092	5.43024778	5.63E-08	2.44E-06
ENSDARG00000076848	zgc:112492	109.564119	-0.790140999	0.24777272	-3.18897495	0.00142778	0.0130515
ENSDARG00000040528	lgals3bpb	361.479457	-0.896337029	0.17195352	-5.21267039	1.86E-07	7.05E-06
ENSDARG00000012192	cant1a	1112.07393	0.346456293	0.10533484	3.28909504	0.0010051	0.00990087
ENSDARG00000089291	cenpx	236.69094	0.503249417	0.1845148	2.72742032	0.00638317	0.04072218
ENSDARG00000053953	myadml2	119.074844	0.886924775	0.24323427	3.64638086	0.00026596	0.00351058
ENSDARG00000078428	si:ch211-256e16.4	15.4307996	2.022250731	0.68402496	2.95639903	0.00311254	0.02382677
ENSDARG00000100690	si:ch211-256e16.11	129.950134	2.74881232	0.24761451	11.1011761	1.24E-28	7.69E-26
ENSDARG00000116806	CU693479.1	1698.95286	0.363250938	0.1303663	2.78638687	0.00532992	0.03570495
ENSDARG00000077732	alyref	8182.94885	0.355764729	0.13458339	2.64345194	0.00820654	0.04903855
ENSDARG00000043795	arhgdia	6866.08494	0.313506353	0.08335653	3.76102954	0.00016922	0.00241847
ENSDARG00000059556	recql5	665.112418	0.528464846	0.15365702	3.43924956	0.00058333	0.00648709
ENSDARG00000059557	xylt2	360.978087	0.735541629	0.17999086	4.0865498	4.38E-05	0.00077898
ENSDARG00000098465	si:ch73-366l1.5	4958.83819	0.733167001	0.12590739	5.82306589	5.78E-09	3.23E-07
ENSDARG00000101919	foxj1a	394.147532	0.840233579	0.18834295	4.46118944	8.15E-06	0.00018775
ENSDARG00000018903	aimp2	3670.36866	0.361953469	0.11230422	3.22297298	0.00126868	0.01189024
ENSDARG00000098021	si:dkey-111k8.2	186.196054	-0.534986843	0.17157659	-3.11806434	0.00182043	0.01574882
ENSDARG00000103947	zgc:113295	147.354113	1.225930379	0.23977477	5.11284139	3.17E-07	1.13E-05
ENSDARG00000074768	zgc:113295	259.518653	-0.47691418	0.1579869	-3.01869454	0.00253866	0.02047175
ENSDARG00000041294	noxo1a	600.672382	1.53350344	0.21885158	7.00704746	2.43E-12	2.54E-10
ENSDARG00000111506	BX470259.1	188.553595	-0.59097198	0.20258619	-2.91713854	0.00353259	0.02629604
ENSDARG00000076831	iqck	109.64828	0.684627975	0.20340184	3.36588877	0.00076297	0.00802188
ENSDARG00000097714	FP017215.1	211.947838	0.933374749	0.23106163	4.03950569	5.36E-05	0.00092751
ENSDARG00000026759	ldlrb	2717.79132	0.957230808	0.10091111	9.48588173	2.40E-21	8.07E-19
ENSDARG00000036613	tab1	1251.47702	0.400695513	0.10370213	3.86390831	0.00011159	0.00171425
ENSDARG00000099309	si:zfos-169g10.3	135.407328	0.689357535	0.21292494	3.23756119	0.00120556	0.01143236
ENSDARG00000038151	zgc:92360	2041.5312	0.319877967	0.09561917	3.34533309	0.00082184	0.00850053
ENSDARG00000100374	txnrd3	4974.04887	0.25576122	0.07399199	3.45660664	0.00054702	0.00618011
ENSDARG00000079884	trim107	130.884134	-0.620374154	0.19440787	-3.19109596	0.00141734	0.01299448
ENSDARG00000074094	tgm2b	3694.99319	0.790865169	0.13985702	5.65481195	1.56E-08	7.87E-07
ENSDARG00000098837	tgm5l	70.2170935	1.22665869	0.32475733	3.77715471	0.00015863	0.0022923
ENSDARG00000077764	faah	268.402954	0.536118258	0.15426873	3.47522322	0.00051043	0.00586261
ENSDARG00000100781	col28a2b	549.779442	-0.436623833	0.15760053	-2.77044641	0.00559795	0.03710322
ENSDARG00000044431	ppig	1503.6596	-0.340480863	0.12166176	-2.7985858	0.00513269	0.03467602
ENSDARG00000029252	ssb	2361.16981	-0.341899413	0.09147636	-3.73757111	0.00018581	0.00261212
ENSDARG00000015126	gorasp2	3018.37633	0.326305158	0.09385797	3.47658436	0.00050784	0.00584103
ENSDARG00000103195	dync1i2b	991.653964	0.423575464	0.12043966	3.51691019	0.0004366	0.00519987
ENSDARG00000020863	trim25l	237.517882	0.42763318	0.14842558	2.88112862	0.00396254	0.02858065
ENSDARG00000088225	STRADB	609.085842	0.282971666	0.10397248	2.72160152	0.00649664	0.04128427
ENSDARG00000032725	rps27a	37475.0909	0.265412788	0.08852665	2.99811166	0.00271658	0.02151709
ENSDARG00000019360	sec23b	4588.94202	0.452725233	0.09793376	4.62276964	3.79E-06	9.65E-05
ENSDARG00000036700	si:ch211-114n24.6	1581.27869	0.991828119	0.30382493	3.26447242	0.00109668	0.01059387
ENSDARG00000058815	ihhb	54.5828447	-0.867779599	0.31061579	-2.7937395	0.00521024	0.03510452
ENSDARG00000063295	myh9a	16303.1749	0.480537902	0.08753172	5.4898717	4.02E-08	1.83E-06
ENSDARG00000094965	nfil3-5	3817.17772	-0.540397816	0.09608912	-5.62392331	1.87E-08	9.15E-07
ENSDARG00000071213	rgl3a	612.6226	0.633779517	0.14211089	4.45975343	8.21E-06	0.00018849
ENSDARG00000037057	gcdha	1981.08584	-0.503736974	0.11176561	-4.50708384	6.57E-06	0.00015617
ENSDARG00000019763	acp5a	235.04831	1.623443	0.57463676	2.82516384	0.00472564	0.03253626
ENSDARG00000071139	zgc:64065	59.3433655	-1.368131195	0.2876075	-4.75693848	1.97E-06	5.50E-05
ENSDARG00000063538	kalrnb	2585.48968	0.31662668	0.11057503	2.86345555	0.00419048	0.02987788

Table S1. DEGs of WT vs. *terfa+/-*

ENSDARG00000073764	si:ch211-113j14.1	592.016824	-0.864142346	0.20523995	-4.21040023	2.55E-05	0.00049577
ENSDARG00000088411	notum2	437.711512	-0.554902247	0.12667067	-4.38066893	1.18E-05	0.00026047
ENSDARG00000096270	BX548046.2	86.0056076	0.950402864	0.33099998	2.87130794	0.00408777	0.02930558
ENSDARG00000044528	slc15a1b	199.882479	-1.005634398	0.32371948	-3.1064995	0.00189317	0.01625376
ENSDARG00000058325	casp8	313.860797	0.751553244	0.18319224	4.10253871	4.09E-05	0.0007344
ENSDARG00000103763	l3hypdh	149.180004	0.738308404	0.24114101	3.06172895	0.00220063	0.01831767
ENSDARG00000071025	dytn	64.1175422	-0.889702742	0.28016184	-3.17567423	0.00149489	0.01351106
ENSDARG00000044521	eef1b2	4919.63964	0.78001658	0.10530768	7.40702469	1.29E-13	1.67E-11
ENSDARG00000023498	gmppab	556.362117	0.49793161	0.12580306	3.95802453	7.56E-05	0.00123332
ENSDARG00000030349	cryba2a	2722.25543	-1.408303934	0.2024444	-6.9564972	3.49E-12	3.52E-10
ENSDARG00000077047	ptprnb	569.921402	0.564776899	0.15798578	3.57485911	0.00035042	0.00434394
ENSDARG00000043361	nck2b	617.16727	0.354876986	0.11026232	3.21847916	0.00128872	0.01204532
ENSDARG00000112756	CU468896.1	9.42650111	-2.283065562	0.84615684	-2.69815885	0.00697242	0.04353163
ENSDARG00000028027	trim63a	1205.75745	-0.629134741	0.10668325	-5.89722118	3.70E-09	2.15E-07
ENSDARG00000020814	faimb	224.683693	-0.767709762	0.23114786	-3.32129297	0.00089601	0.00913472
ENSDARG00000078592	nomo	4526.30024	-0.244900536	0.08245969	-2.96994257	0.00297855	0.0230701
ENSDARG00000100584	ccdc40	316.149585	0.804328661	0.19437269	4.13807453	3.50E-05	0.0006476
ENSDARG00000098924	suz12b	1885.53118	-0.640985459	0.09139395	-7.01343423	2.33E-12	2.44E-10
ENSDARG00000033993	slc19a1	444.349165	-0.393092509	0.13906689	-2.82664348	0.00470387	0.03244023
ENSDARG00000052351	si:dkey-156n14.3	1370.67401	-0.28393994	0.10627326	-2.67179089	0.00754476	0.04614299
ENSDARG00000010873	DDX17	4409.43635	0.339592779	0.07124912	4.7662733	1.88E-06	5.28E-05
ENSDARG00000019808	evpla	2768.03985	0.32180056	0.08761016	3.67309618	0.00023963	0.00321597
ENSDARG00000102415	scinla	7430.08144	-0.63011967	0.12950637	-4.86554952	1.14E-06	3.47E-05
ENSDARG00000098348	znf326	63.1187014	1.093909732	0.33290655	3.28593632	0.00101644	0.00999352
ENSDARG00000061896	slco2a1	432.447373	0.631912453	0.13277639	4.75922312	1.94E-06	5.45E-05
ENSDARG00000061923	amotl2a	3615.78536	-0.293190046	0.08882981	-3.30058183	0.00096485	0.00959633
ENSDARG00000056924	sap130a	1539.11426	0.41410733	0.1348643	3.07054818	0.00213666	0.01790517
ENSDARG00000062030	gpr17	181.469371	-0.598511827	0.16524336	-3.62200234	0.00029233	0.00377058
ENSDARG00000098121	FP101882.1	221.299754	-0.642839322	0.23092949	-2.78370396	0.00537421	0.03591715
ENSDARG00000114184	si:busm1-105l16.2	12.6791712	2.385313708	0.83366523	2.86123689	0.00421992	0.0300103
ENSDARG00000044356	tp63	2452.13224	0.365661591	0.07717602	4.73802097	2.16E-06	5.98E-05
ENSDARG00000044457	gfi1ab	142.524059	-0.525484779	0.18128943	-2.89859587	0.00374838	0.02742594
ENSDARG00000079198	usp13	1515.28438	-0.500374363	0.09882835	-5.06306478	4.13E-07	1.42E-05
ENSDARG00000074892	pde6d	321.89934	-0.551215539	0.16207459	-3.4009991	0.0006714	0.00726446
ENSDARG00000096822	CR847898.1	3.72370497	5.515680817	1.6655533	3.31162072	0.00092757	0.00933474
ENSDARG00000097574	CR847898.3	19.7146063	4.003566208	0.73457474	5.45018224	5.03E-08	2.21E-06
ENSDARG00000097265	CR847898.2	47.8074253	1.104031354	0.38055261	2.90112675	0.00371823	0.02727766
ENSDARG00000017708	lrrc40	434.469182	-0.388779765	0.11444465	-3.39709859	0.00068104	0.00734375
ENSDARG00000020625	jak1	4534.43191	0.326814355	0.11732806	2.78547478	0.00534494	0.03577859
ENSDARG00000020279	efcab7	429.800785	-0.378926721	0.12704829	-2.98254086	0.00285866	0.02235046
ENSDARG00000044365	angptl3	739.280408	0.61109187	0.1511744	4.04229732	5.29E-05	0.00092084
ENSDARG00000044155	mafaa	1039.06247	0.340382628	0.09717455	3.50279612	0.0004604	0.0054131
ENSDARG00000052170	uap1	1008.29559	0.410897413	0.10041401	4.09203252	4.28E-05	0.00076188
ENSDARG00000101275	BX936433.1	39.5650383	-1.427621851	0.40712175	-3.50662141	0.00045383	0.00536379
ENSDARG00000103194	BX936433.2	23.2659914	-1.83591994	0.44202875	-4.15339486	3.28E-05	0.00060943
ENSDARG00000007080	rhcgl1	337.599004	4.09702604	0.30598494	13.3896331	6.95E-41	1.33E-37
ENSDARG00000070792	lrrc15	164.627737	-0.578153766	0.1971775	-2.93214882	0.00336625	0.02525709
ENSDARG00000044332	zranb2	1734.60328	0.327007218	0.1124011	2.90928845	0.00362252	0.02675716
ENSDARG00000032206	cthl	109.326505	-0.801033754	0.25428244	-3.15017325	0.00163174	0.01452088
ENSDARG00000074301	cth	1594.23643	-0.330011466	0.10018249	-3.29410313	0.00098736	0.00978461
ENSDARG00000017590	nit2	561.725186	-0.473327219	0.10942707	-4.32550379	1.52E-05	0.0003192
ENSDARG00000091465	si:dkey-66a8.7	271.997647	0.445665605	0.13872544	3.21257294	0.00131552	0.01222782
ENSDARG00000073841	herc2	7349.16848	0.313431666	0.11418124	2.74503644	0.00605042	0.03917248
ENSDARG00000061039	atp10a	375.205094	0.659227602	0.16338667	4.03476983	5.47E-05	0.00094207
ENSDARG00000097018	CR628341.1	36.0108288	6.072483888	1.06953783	5.67767097	1.37E-08	7.03E-07
ENSDARG00000057497	stk35l	1170.95622	-0.352625016	0.10198294	-3.45768646	0.00054484	0.006161
ENSDARG00000014465	arhgef25b	561.548551	0.577332695	0.16137803	3.57751738	0.00034687	0.00431724
ENSDARG00000017624	krt4	481111.355	0.663642349	0.08515095	7.79371635	6.51E-15	1.04E-12

Table S1. DEGs of WT vs. *terfa+/-*

ENSDARG00000058462	zgc:158846	3918.27005	-0.649722584	0.19947852	-3.25710544	0.00112555	0.01082222
ENSDARG00000028618	krt18b	5929.63117	0.36746655	0.10577445	3.4740578	0.00051265	0.00588201
ENSDARG00000028478	si:ch211-173n18.3	45.7103739	2.482659712	0.38263113	6.48838931	8.68E-11	7.02E-09
ENSDARG00000074979	larp4ab	1650.37803	0.582380929	0.10711161	5.43714116	5.41E-08	2.35E-06
ENSDARG00000060797	pfkmb	2339.76096	0.385910043	0.11651223	3.31218497	0.0009257	0.00932267
ENSDARG00000077946	smarcc2	2660.34458	0.447635839	0.11851638	3.77699546	0.00015873	0.00229243
ENSDARG00000008494	myl6	1458.71009	0.430410853	0.09842751	4.37287155	1.23E-05	0.00026829
ENSDARG00000029075	pfkfb4b	2570.62771	0.702354758	0.10423053	6.73847469	1.60E-11	1.47E-09
ENSDARG00000070394	uroc1	1161.87518	0.503070595	0.15311213	3.28563506	0.00101753	0.01000026
ENSDARG00000019613	ip6k2b	2849.58513	0.362662464	0.11815136	3.06947337	0.00214437	0.01795761
ENSDARG00000021720	col7a1	5342.67619	0.437827158	0.12495771	3.50380257	0.00045867	0.00540034
ENSDARG00000079467	si:ch73-15b2.5	288.986609	0.916839591	0.19698086	4.65446037	3.25E-06	8.40E-05
ENSDARG00000074002	slc6a11a	397.927229	0.60986354	0.13698574	4.45202199	8.51E-06	0.00019433
ENSDARG00000086336	si:ch211-157b11.12	231.883047	-0.451817929	0.15802018	-2.85924192	0.00424655	0.03013337
ENSDARG00000010415	sirt4	207.007893	1.798090834	0.18881505	9.52302695	1.68E-21	5.73E-19
ENSDARG00000053992	sfi1	402.244403	-0.751728951	0.18271993	-4.1141049	3.89E-05	0.00070465
ENSDARG00000016177	eif4enif1	952.533156	-0.388268078	0.115	-3.3762442	0.00073483	0.00779526
ENSDARG00000028396	fkbp5	5927.57648	1.781628005	0.16632252	10.7118868	8.95E-27	4.54E-24
ENSDARG00000003961	parp3	931.459903	0.287975177	0.10772281	2.67329813	0.00751095	0.0459701
ENSDARG00000053574	fancd2	781.828082	0.424906686	0.11479247	3.70152048	0.00021431	0.00293644
ENSDARG00000005675	sec61a1l	1051.84674	0.325607434	0.10740709	3.03152656	0.00243321	0.01978322
ENSDARG00000044199	gnat1	1768.57287	-1.275253984	0.38399953	-3.320978	0.00089703	0.00913753
ENSDARG00000096903	CU062633.1	5.42121027	5.774382533	1.7297623	3.33825205	0.00084307	0.00867843
ENSDARG00000087873	eevs	1957.06513	0.848300012	0.13020834	6.51494397	7.27E-11	5.95E-09
ENSDARG00000014181	foxp1b	1815.1414	0.376200655	0.12851213	2.92735519	0.00341858	0.02558532
ENSDARG00000058522	shq1	297.095932	-0.578959144	0.16275018	-3.55734881	0.00037462	0.00458675
ENSDARG00000017354	epha2a	899.937783	0.372163499	0.0948414	3.92406167	8.71E-05	0.00138723
ENSDARG00000060153	h6pd	1181.94933	0.430913661	0.12797018	3.36729742	0.00075909	0.00799116
ENSDARG00000070486	rbp7b	53.4863905	-1.767676426	0.35021876	-5.04734936	4.48E-07	1.52E-05
ENSDARG00000075540	FAM107A	98.5583184	-0.933154238	0.24141332	-3.86538003	0.00011092	0.00170701
ENSDARG00000044182	stau1	5758.8345	0.318109628	0.06825636	4.66051251	3.15E-06	8.21E-05
ENSDARG00000079897	si:dkey-7k24.5	36.203466	-2.311643797	0.39469251	-5.85682199	4.72E-09	2.67E-07
ENSDARG00000060094	ptgis	294.87536	0.534933657	0.14875762	3.59600848	0.00032314	0.00408732
ENSDARG00000005526	igfn1.1	1974.00246	-0.512054463	0.14337874	-3.57134168	0.00035516	0.00439176
ENSDARG00000086724	im:7151449	235.661874	0.666079546	0.22203816	2.9998427	0.00270119	0.02142249
ENSDARG00000015829	mov10a	941.920707	0.320853375	0.10132075	3.16670937	0.00154174	0.01385896
ENSDARG00000016963	slc16a1a	121.813259	0.596756109	0.20388105	2.92698173	0.00342269	0.02560836
ENSDARG00000020893	slc25a55a	8322.86088	-0.765678273	0.0907705	-8.43532111	3.30E-17	7.60E-15
ENSDARG00000014050	ngfb	205.218232	0.52748006	0.15075445	3.49893528	0.00046712	0.00547396
ENSDARG00000002241	kcna2a	69.995268	-0.720297692	0.26749732	-2.69272864	0.00708699	0.04406985
ENSDARG00000101406	rplp2	11197.4534	0.611952694	0.09568959	6.39518548	1.60E-10	1.23E-08
ENSDARG00000030408	rps26l	18561.0237	0.462916094	0.10174611	4.54971792	5.37E-06	0.00013141
ENSDARG00000043081	ctsz	2052.41907	0.516874241	0.12347154	4.18618122	2.84E-05	0.00053825
ENSDARG00000005191	ahcy	41259.9052	-0.338942506	0.10443121	-3.2456056	0.00117201	0.01119106
ENSDARG00000053047	eif2s2	6955.33342	-0.231458897	0.08173956	-2.83166311	0.00463066	0.03205098
ENSDARG00000052279	osgn1	775.620115	0.452857492	0.11158943	4.05824716	4.94E-05	0.00086481
ENSDARG00000097807	BX530017.2	41.1763523	2.371387875	0.54965246	4.31434053	1.60E-05	0.00033295
ENSDARG00000074781	ptprt	774.742516	0.673208363	0.19215396	3.50348419	0.00045921	0.00540169
ENSDARG00000038577	cox6c	6257.65256	-0.30889373	0.08210541	-3.76216029	0.00016845	0.00241114
ENSDARG00000096319	si:ch211-239j9.1	661.526369	0.438496213	0.15945171	2.75002509	0.00595907	0.03872227
ENSDARG00000074148	rbpjl	37.2894476	-1.236988122	0.45199775	-2.73671302	0.00620564	0.03987621
ENSDARG00000061222	uba7	136.22645	-0.791677713	0.26636535	-2.97214975	0.00295722	0.02294064
ENSDARG00000091061	slc38a3b	1114.69707	-0.334748523	0.1222983	-2.73714775	0.00619745	0.03983385
ENSDARG00000078210	tulp1b	297.559266	-1.075235257	0.1863166	-5.77101166	7.88E-09	4.28E-07
ENSDARG00000003081	mybphb	33778.1403	-0.489350283	0.1460714	-3.35007593	0.00080789	0.00839825
ENSDARG00000014169	SYT2	296.121411	-0.585177764	0.21359734	-2.73963038	0.00615083	0.03963224
ENSDARG00000079972	nfatc2b	33.7142695	2.255823356	0.52801036	4.27230889	1.93E-05	0.00039061
ENSDARG00000101618	ube2c	387.988756	-0.401409428	0.14971212	-2.68120863	0.00733568	0.04520909

Table S1. DEGs of WT vs. *terfa+/-*

ENSDARG00000103277	cyp24a1	746.495431	1.845447447	0.39279229	4.69827817	2.62E-06	7.07E-05
ENSDARG00000018984	eya2	1607.97658	0.329348809	0.10552832	3.1209518	0.00180268	0.01564438
ENSDARG00000040607	rtf2	1048.20182	-0.349762941	0.11167965	-3.13184141	0.00173714	0.01525134
ENSDARG00000100280	znfx1	160.657853	-1.179171535	0.27257095	-4.32610861	1.52E-05	0.00031864
ENSDARG00000088899	ralgapb	2325.40875	0.320469552	0.11342076	2.825493	0.00472079	0.03253593
ENSDARG00000038465	stmn3	939.713874	-0.501107008	0.10014159	-5.0039847	5.62E-07	1.83E-05
ENSDARG00000038467	igsf8	1561.8828	0.243685752	0.09241051	2.63699163	0.00836449	0.04978434
ENSDARG00000059824	soat2	359.711556	-0.655451298	0.15014554	-4.36543954	1.27E-05	0.0002754
ENSDARG00000059836	ddit3	1296.90255	-0.544231118	0.09223082	-5.90075095	3.62E-09	2.11E-07
ENSDARG00000100986	CABZ01117503.1	93.2433853	-1.690612655	0.32310628	-5.2323732	1.67E-07	6.43E-06
ENSDARG00000005774	ddx3xb	18358.0806	-0.417089418	0.10112249	-4.12459614	3.71E-05	0.00067774
ENSDARG00000059794	kdm6al	2557.50473	-0.35874141	0.09701016	-3.69797753	0.00021732	0.00297117
ENSDARG00000045019	aamp	2944.73188	0.341989932	0.08741446	3.91228083	9.14E-05	0.00144558
ENSDARG00000039328	ccndbp1	1251.05819	0.68808385	0.15024041	4.57988525	4.65E-06	0.0001154
ENSDARG00000013522	pck1	9088.84388	1.064679011	0.18278583	5.8247348	5.72E-09	3.21E-07
ENSDARG00000009505	prelid3b	4019.08532	0.58467932	0.10365055	5.64087026	1.69E-08	8.39E-07
ENSDARG00000063518	zgc:153913	1465.95555	0.329955999	0.12456305	2.64890759	0.00807524	0.04850837
ENSDARG00000038667	fggy	727.571382	1.338736641	0.1638202	8.17198753	3.03E-16	5.98E-14
ENSDARG00000101562	znf1014	505.503101	-0.43936634	0.1292534	-3.39926325	0.00067568	0.00729893
ENSDARG00000078694	phlpp1	2511.34808	-0.37904609	0.12445624	-3.0456175	0.00232203	0.01914779
ENSDARG00000097884	CU928013.1	281.786331	-0.524577227	0.15439187	-3.39769987	0.00067955	0.00733081
ENSDARG00000097762	CU693494.3	57.3677542	2.014463593	0.45685866	4.40938038	1.04E-05	0.00023192
ENSDARG00000097118	CU693494.2	4657.53432	1.022153599	0.17412911	5.87009018	4.36E-09	2.49E-07
ENSDARG00000087516	CU693494.1	10.5028106	-2.855921703	0.79620626	-3.58691191	0.00033462	0.00419622
ENSDARG00000091260	mylk4a	866.237663	0.60228651	0.1689227	3.5654563	0.00036322	0.00447595
ENSDARG00000022615	si:ch211-241e1.3	602.10605	0.716709347	0.17117819	4.18691981	2.83E-05	0.00053732
ENSDARG00000053746	CABZ01084566.1	220.904772	-0.684155783	0.24346941	-2.8100277	0.00495372	0.03376057
ENSDARG00000022309	dspa	6684.85811	0.272091046	0.1004518	2.70867275	0.00675529	0.04249628
ENSDARG00000022303	higd1a	1037.33637	0.849623486	0.12777113	6.6495731	2.94E-11	2.55E-09
ENSDARG00000020957	pth1r	102.774088	-0.90954764	0.27035311	-3.36429512	0.00076739	0.00805813
ENSDARG00000042245	myl13	15730.204	0.320647208	0.09685274	3.3106673	0.00093074	0.00934396
ENSDARG00000103038	pik3r3a	1724.63517	0.503744116	0.10204325	4.93657468	7.95E-07	2.49E-05
ENSDARG00000075339	ipp	368.94625	0.510361309	0.14860055	3.43445097	0.00059376	0.00659031
ENSDARG00000079730	fuz	147.127021	0.536850668	0.20202079	2.65740304	0.00787452	0.04763234
ENSDARG00000098344	rab18b	1412.97283	0.32643542	0.11308702	2.88658621	0.00389446	0.0282042
ENSDARG00000045297	phb2a	3488.17764	-0.316310416	0.10863854	-2.91158566	0.00359599	0.02661656
ENSDARG00000039310	parla	340.35022	-0.575863412	0.16502433	-3.48956667	0.0004838	0.00561914
ENSDARG00000014190	sst2	9.13222353	2.746684718	0.92919343	2.9559881	0.00311669	0.02384283
ENSDARG00000034940	slc1a7a	285.832932	-0.625965124	0.22907851	-2.73253535	0.00628489	0.04021911
ENSDARG00000063218	ppm1la	1862.07068	0.262864621	0.08965011	2.93211726	0.0033666	0.02525709
ENSDARG00000114516	CABZ01021592.1	4126.6244	0.744967551	0.11562341	6.44305097	1.17E-10	9.21E-09
ENSDARG00000100981	CCDC39	128.087065	-1.267417283	0.31771021	-3.98922424	6.63E-05	0.00110804
ENSDARG00000031548	ephb3a	2423.6005	0.30259089	0.11088473	2.72887796	0.00635502	0.04058429
ENSDARG00000018562	cope	3475.602	0.297578908	0.11152823	2.66819363	0.00762603	0.04649347
ENSDARG00000002790	ap2m1a	12116.607	0.276229487	0.08055288	3.42916953	0.00060543	0.00670831
ENSDARG00000013613	anxa13l	602.868358	-0.611836497	0.12733315	-4.8050055	1.55E-06	4.47E-05
ENSDARG00000069280	tsen15	319.969452	-0.690799476	0.18723541	-3.68947021	0.00022472	0.00305383
ENSDARG00000019924	cmpk	3621.60004	0.235257282	0.08813267	2.66935378	0.00759974	0.04637649
ENSDARG00000043581	gadd45aa	493.512866	0.467704769	0.12828184	3.64591561	0.00026644	0.00351507
ENSDARG00000009534	wls	3578.0608	0.260929376	0.07879263	3.31159628	0.00092765	0.00933474
ENSDARG00000078822	ccdc18	299.027737	-0.486679037	0.14878889	-3.27093666	0.00107192	0.01041131
ENSDARG00000077666	evi5a	48.5448843	-1.553749941	0.35561706	-4.36916591	1.25E-05	0.00027217
ENSDARG00000062919	tceanc2	85.9114626	-0.719684681	0.23926506	-3.00789717	0.00263062	0.02101707
ENSDARG00000058229	acot11a	297.02335	-0.585958373	0.16162995	-3.62530805	0.00028862	0.00374014
ENSDARG00000058218	fam151a	93.396982	-0.712143448	0.24178552	-2.9453519	0.00322588	0.02443741
ENSDARG00000095190	BX901918.3	6.8832694	5.696725574	1.48102844	3.84646603	0.00011983	0.00182058
ENSDARG00000094331	BX901918.2	155.638321	9.799563729	1.31192283	7.469619	8.04E-14	1.07E-11
ENSDARG00000018809	abhd3	547.726975	0.50409548	0.15423419	3.26837699	0.00108166	0.0104732

Table S1. DEGs of WT vs. *terfa+/-*

ENSDARG00000075560	kmt2cb	4358.15816	0.438726645	0.1497449	2.92982697	0.00339151	0.02542097
ENSDARG00000054980	ebna1bp2	909.286533	-0.345798791	0.13059897	-2.6477911	0.00810196	0.0485815
ENSDARG00000099519	cpdp	919.985248	0.446838255	0.10027603	4.45608228	8.35E-06	0.00019086
ENSDARG00000074233	pde4bb	248.78041	-0.49069538	0.17027997	-2.88169764	0.00395539	0.02854566
ENSDARG00000092949	si:ch73-366i20.1	34.9512186	1.463734241	0.48800931	2.9993982	0.00270514	0.02144693
ENSDARG00000005673	f3b	155.878738	-0.553954237	0.19314252	-2.86811132	0.0041293	0.02950095
ENSDARG00000062661	abca4b	1124.01774	-0.543229974	0.19107239	-2.84305851	0.00446829	0.03124909
ENSDARG00000012325	zgc:110269	375.673639	-0.470219704	0.14142126	-3.32495763	0.00088432	0.00903402
ENSDARG00000092225	si:dkeyp-13a3.10	350.749564	-0.38478597	0.1297732	-2.96506497	0.00302619	0.02334449
ENSDARG00000006200	eif4g1a	11821.8045	0.251304789	0.08227777	3.05434629	0.00225552	0.01867872
ENSDARG00000077022	fam131a	205.914884	-0.542712357	0.15935793	-3.40561879	0.00066014	0.00716586
ENSDARG00000099960	elovl1a	754.153417	0.555669813	0.12695816	4.37679466	1.20E-05	0.00026397
ENSDARG00000099445	cc2d1b	619.047362	0.60656946	0.16005737	3.78970024	0.00015083	0.00220025
ENSDARG00000057253	pimr141	11.7476851	-2.537136405	0.85230066	-2.97680916	0.00291265	0.02267982
ENSDARG00000001913	palmda	526.416326	-0.967039333	0.16222256	-5.96118905	2.50E-09	1.54E-07
ENSDARG00000060622	si:ch73-14h1.2	387.670435	1.104169617	0.24139609	4.57409894	4.78E-06	0.00011828
ENSDARG00000075487	si:ch211-267e7.3	889.968205	0.75334175	0.14680554	5.13156223	2.87E-07	1.04E-05
ENSDARG00000010482	prg4a	74.4086527	1.24875149	0.3100374	4.02774469	5.63E-05	0.00096664
ENSDARG00000011886	pdca	175.465824	-0.735500835	0.23965441	-3.069006	0.00214772	0.01797967
ENSDARG00000039142	arpc5a	2567.88858	0.255840236	0.09178997	2.78723506	0.00531599	0.03564309
ENSDARG00000091879	si:dkey-29d8.3	119.990225	-0.591451993	0.21124912	-2.79978437	0.00511368	0.03459456
ENSDARG00000057751	zbtb47a	123.292295	0.698302955	0.22140207	3.15400374	0.00161047	0.01435908
ENSDARG00000062116	ctdsplb	1803.58957	-0.393802573	0.08828175	-4.46074703	8.17E-06	0.00018779
ENSDARG00000099478	hs2st1a	566.256475	-0.331288805	0.11701814	-2.83108931	0.00463898	0.03209067
ENSDARG00000103370	lrrc8da	427.632909	-0.425026979	0.14473695	-2.93654775	0.00331888	0.02497087
ENSDARG00000104288	hfm1	74.3830249	0.956556046	0.30778705	3.10785021	0.00188454	0.01620205
ENSDARG00000031343	rab6bb	1947.87938	0.359205829	0.10065228	3.56877994	0.00035865	0.00442886
ENSDARG00000056784	aire	9.65341732	-2.032990095	0.73406347	-2.76950179	0.00561421	0.03717133
ENSDARG00000061948	amotl2b	1062.35049	-0.374688601	0.11691887	-3.20468878	0.00135209	0.01249297
ENSDARG00000011373	mknk2a	385.560232	-0.410767596	0.15518805	-2.6469022	0.00812328	0.04865351
ENSDARG00000023053	niban1b	191.150322	-0.63679677	0.20668117	-3.08105851	0.00206266	0.01743577
ENSDARG00000013946	ivns1abpb	2416.9398	0.255128593	0.08271176	3.08455055	0.0020386	0.01727562
ENSDARG00000111218	si:ch211-14p21.3	4.56082332	-3.381331226	1.21984351	-2.77193854	0.00557236	0.03697301
ENSDARG00000030722	xirp1	852.824164	-0.464159965	0.09209737	-5.03988277	4.66E-07	1.56E-05
ENSDARG00000056464	fitm1l	744.766788	0.932880315	0.16423188	5.68026339	1.34E-08	6.96E-07
ENSDARG00000056475	trnau1apb	2376.89158	-0.358340196	0.08253473	-4.34168963	1.41E-05	0.00030114
ENSDARG00000035861	si:rp71-39b20.4	134.878277	-0.933973121	0.28458022	-3.28193269	0.00103098	0.01008067
ENSDARG00000035860	rps28	10722.9626	0.811300437	0.12575625	6.45137294	1.11E-10	8.80E-09
ENSDARG00000035859	angptl4	3781.08941	-0.334636893	0.0930308	-3.5970548	0.00032184	0.00407922
ENSDARG00000056367	mpv17l2	382.390931	-0.427313271	0.14817317	-2.88387744	0.00392812	0.02841482
ENSDARG00000013221	pde4ca	347.666809	0.645818347	0.14892706	4.33647406	1.45E-05	0.00030648
ENSDARG00000056347	rab3aa	1109.38337	-0.319426664	0.1150495	-2.77642811	0.00549598	0.03657364
ENSDARG00000056262	slc35g2a	605.023467	0.566283291	0.1511708	3.74598327	0.00017969	0.00253615
ENSDARG00000078179	fndc3ba	1802.45299	0.323735746	0.11593735	2.79233356	0.00523294	0.03521554
ENSDARG00000056218	tnika	1967.04161	0.286033036	0.10383055	2.75480624	0.00587269	0.03836878
ENSDARG00000056186	eif5a2	10831.5502	-0.375371235	0.09829358	-3.81887838	0.00013406	0.00200726
ENSDARG00000010816	slc7a14a	1482.6654	0.439185257	0.13057698	3.36342024	0.00076983	0.00807007
ENSDARG00000019362	ptbp1a	2693.57249	-0.344855936	0.08534552	-4.04070333	5.33E-05	0.00092497
ENSDARG00000038900	acadm	5071.09395	-0.297497279	0.1061814	-2.80178322	0.0050821	0.034478
ENSDARG00000027584	ttpa	261.038538	-1.243023661	0.20716997	-6.00001841	1.97E-09	1.22E-07
ENSDARG00000038894	tmx3a	548.517652	-1.050409223	0.20803908	-5.04909579	4.44E-07	1.51E-05
ENSDARG00000061450	tesk2	788.795299	-0.468668353	0.10340648	-4.5322917	5.83E-06	0.00014079
ENSDARG00000107511	lyn	465.634781	-0.34751518	0.1244186	-2.79311287	0.00522035	0.03515354
ENSDARG00000014479	ptf1a	86.5650137	-0.778467534	0.26790475	-2.90576232	0.0036636	0.02698835
ENSDARG00000004680	dnajb6a	1917.35289	-0.325037149	0.10467782	-3.10511959	0.00190202	0.01631851
ENSDARG00000038862	kcnb2	474.82165	-0.593484355	0.19185927	-3.09333166	0.00197923	0.01687606
ENSDARG00000061292	pi15b	19.5544884	-1.595328589	0.50409797	-3.16471933	0.00155233	0.01391886
ENSDARG00000061196	emilin2a	693.035898	-0.777153244	0.16406025	-4.73699905	2.17E-06	6.00E-05

Table S1. DEGs of WT vs. *terfa+/-*

ENSDARG00000055565	cacnb2b	334.401107	-0.421658748	0.14872348	-2.8351861	0.0045799	0.03177042
ENSDARG00000077219	si:ch211-106h4.6	49.9057977	-1.038193291	0.38633514	-2.6872867	0.00720351	0.0446263
ENSDARG00000092920	si:ch211-106h4.12	248.784818	-0.493800766	0.17171566	-2.87568866	0.00403147	0.02897693
ENSDARG00000061049	rnf182	80.4208304	-1.474515969	0.26108936	-5.64755286	1.63E-08	8.17E-07
ENSDARG00000014969	ankhb	1056.0492	-0.422599498	0.09713833	-4.35049181	1.36E-05	0.00029205
ENSDARG00000038780	ubtfl	2464.69381	-0.545980361	0.09046661	-6.03515894	1.59E-09	1.01E-07
ENSDARG00000028173	slc4a2a	1098.60625	0.401581522	0.1060024	3.78841918	0.00015161	0.00220752
ENSDARG00000038785	abcf2a	3121.18164	0.316681494	0.09582901	3.30465154	0.00095095	0.00950372
ENSDARG00000093443	BX323559.4	40.7852068	2.210397053	0.43941275	5.0303435	4.90E-07	1.63E-05
ENSDARG00000079589	si:dkeyp-73d8.6	12.659425	5.030888566	1.24048198	4.05559181	5.00E-05	0.00087408
ENSDARG00000076043	si:dkeyp-73d8.9	82.4757727	5.799277888	0.71095821	8.15698838	3.43E-16	6.62E-14
ENSDARG00000011998	bfsp2	1864.1989	-1.743215577	0.18186274	-9.5853367	9.22E-22	3.27E-19
ENSDARG00000055177	pxdc1a	100.449837	-0.837420198	0.22943743	-3.64988489	0.00026236	0.00347782
ENSDARG00000007553	opn4.1	576.387711	-0.632505342	0.1811906	-3.49082875	0.00048152	0.00559798
ENSDARG00000031681	atp6v0b	2047.71892	0.332026115	0.11123485	2.98491097	0.00283661	0.022248
ENSDARG00000014302	brinp2	628.179284	-0.361279956	0.13244403	-2.7277935	0.00637595	0.04068659
ENSDARG00000024829	tnn	16029.0108	-0.342928793	0.1101799	-3.1124442	0.00185545	0.01599626
ENSDARG00000097594	FP236741.1	10.6600205	-1.721639279	0.61862654	-2.78300261	0.00538584	0.03596587
ENSDARG00000068305	mrps14	828.759036	0.527177586	0.12362688	4.26426337	2.01E-05	0.00040397
ENSDARG00000038754	plk3	1514.65258	-0.344360746	0.09748068	-3.53260503	0.00041149	0.00495279
ENSDARG00000011326	ankrd45	25.5226617	1.646876002	0.47487797	3.46799833	0.00052435	0.00598931
ENSDARG00000036257	rasal2	1857.72753	0.332143802	0.112241	2.95920207	0.00308437	0.0236684
ENSDARG00000068288	lamc2	179.637983	1.157131396	0.18152103	6.37464109	1.83E-10	1.39E-08
ENSDARG00000068177	pak2a	2842.02438	-0.195809918	0.07384875	-2.65149952	0.00801352	0.04820638
ENSDARG00000014545	golim4b	337.737923	0.495457309	0.17139788	2.8906852	0.00384403	0.0279307
ENSDARG00000012591	pdcd10b	715.32648	-0.285993418	0.09522227	-3.00342995	0.00266955	0.02125974
ENSDARG00000013576	gadd45bb	264.6339	0.667170151	0.20183025	3.30560033	0.00094773	0.00947541
ENSDARG00000004173	copa	11517.3064	0.265040715	0.08642439	3.06673521	0.0021641	0.01808636
ENSDARG00000060350	apoda.2	1923.29638	-1.047681035	0.35008505	-2.99264718	0.00276569	0.0218267
ENSDARG00000060345	apoda.1	593.055849	-0.56361034	0.14222347	-3.96285036	7.41E-05	0.00121263
ENSDARG00000060362	nadkb	801.69787	0.33316285	0.11173582	2.98170126	0.00286652	0.02239775
ENSDARG00000095191	si:dkey-57k2.7	220.606141	-0.590229756	0.17206586	-3.43025484	0.00060301	0.00668805
ENSDARG00000030448	sppl2	1149.78693	-0.346945152	0.12696707	-2.7325601	0.00628442	0.04021911
ENSDARG00000011157	arhgef18a	1140.47949	-0.525501221	0.12613849	-4.16606562	3.10E-05	0.00058089
ENSDARG00000094681	hbl2	49.4257508	-1.195500783	0.40450888	-2.95543767	0.00312226	0.02387807
ENSDARG00000038713	tep1	242.956694	-0.661676986	0.19988263	-3.31032757	0.00093187	0.00935066
ENSDARG00000117380	LO017954.1	76.8770321	-0.849189077	0.27624204	-3.07407619	0.00211156	0.01774863
ENSDARG00000053792	si:rp71-1g18.1	489.484518	-0.329963191	0.10825226	-3.04809515	0.00230297	0.01901672
ENSDARG00000075543	chd8	4596.92594	0.280571109	0.10401618	2.6973796	0.00698876	0.04360078
ENSDARG00000068240	trim110	72.9957628	-0.816600642	0.28673094	-2.84796839	0.00439993	0.03089272
ENSDARG00000054343	slc7a8b	48.754769	-1.160224039	0.32185667	-3.60478478	0.00031241	0.00397797
ENSDARG00000038743	copb2	7951.3484	0.341616542	0.08434172	4.05038637	5.11E-05	0.0008925
ENSDARG00000038742	rbp1.2	182.738547	-1.34618415	0.26984006	-4.98882245	6.07E-07	1.96E-05
ENSDARG00000094983	ccl34a.3	10.7684537	5.870989186	1.3364163	4.39308408	1.12E-05	0.00024756
ENSDARG00000090873	ccl34a.4	98.0947054	2.009018594	0.30771096	6.52891473	6.62E-11	5.43E-09
ENSDARG00000056490	zgc:110158	4293.73189	0.30392124	0.11479786	2.64744686	0.00811021	0.04859674
ENSDARG00000038668	gbp1	252.465432	0.876579038	0.25438848	3.44582836	0.00056931	0.00638549
ENSDARG00000029596	trim55a	637.539616	0.366686113	0.10872796	3.37250993	0.00074486	0.00788484
ENSDARG00000008858	cyp7b1	379.605475	0.681820973	0.1547858	4.40493242	1.06E-05	0.00023631
ENSDARG00000008191	tmeff1a	191.784127	-0.745336339	0.19417817	-3.83841469	0.00012383	0.00187216
ENSDARG00000079784	si:ch211-235o23.1	821.121636	0.4200959	0.12114435	3.46773001	0.00052487	0.00599254
ENSDARG00000053450	ftr06	25.0722403	4.19142086	0.61121054	6.85757297	7.00E-12	6.82E-10
ENSDARG00000001721	oc90	164.236131	1.785847031	0.62695098	2.8484636	0.00439309	0.03086213
ENSDARG00000074118	ftr12	135.562077	-0.689562342	0.23635623	-2.91747059	0.00352883	0.02627593
ENSDARG00000023217	crema	687.358839	0.468431565	0.16917032	2.76899374	0.00562297	0.03720392
ENSDARG00000022689	itgb1b.2	998.86851	-0.890731715	0.12801185	-6.95819744	3.45E-12	3.50E-10
ENSDARG00000003244	gbp3	433.68558	-0.691337488	0.20987758	-3.29400355	0.00098771	0.00978461
ENSDARG00000068431	si:ch211-195h23.3	553.124365	-0.63449443	0.19830118	-3.19965026	0.00137594	0.01268513

Table S1. DEGs of WT vs. *terfa+/-*

ENSDARG00000038608	sdhc	2824.40044	0.417221091	0.09505813	4.38911507	1.14E-05	0.00025145
ENSDARG00000038609	mpz	242.16649	-1.182839097	0.23290566	-5.07861899	3.80E-07	1.33E-05
ENSDARG00000079777	map6d1	190.923412	-0.597336442	0.21103516	-2.8305068	0.00464743	0.03212234
ENSDARG00000004880	acsm3	373.839535	-0.480188468	0.17040955	-2.81784953	0.00483465	0.03311246
ENSDARG00000053155	alg3	570.332922	0.525904522	0.11294066	4.65646751	3.22E-06	8.34E-05
ENSDARG00000086647	chrng	332.091833	0.530748312	0.17616235	3.01283627	0.00258819	0.02076204
ENSDARG00000091280	si:ch211-66k16.27	335.571191	-1.240055625	0.17741442	-6.98959874	2.76E-12	2.84E-10
ENSDARG00000088423	si:ch211-66k16.28	111.290346	-1.186661252	0.25255938	-4.69854359	2.62E-06	7.06E-05
ENSDARG00000092813	fndc7rs4	469.092017	-0.467826579	0.142431	-3.2845839	0.00102133	0.01002574
ENSDARG00000076757	ephb1	303.44637	0.690002438	0.23205306	2.97346836	0.00294455	0.022878
ENSDARG00000076970	si:ch211-165b10.3	41.7926287	-1.042603609	0.30991935	-3.36411262	0.0007679	0.00806005
ENSDARG00000067985	ftr23	87.4641644	-0.928599135	0.29996779	-3.09566278	0.00196374	0.01675609
ENSDARG00000034503	per2	4653.53771	1.097818136	0.12468948	8.80441657	1.32E-18	3.52E-16
ENSDARG00000104524	CR391991.4	61.3341709	5.432053893	0.692996	7.83850687	4.56E-15	7.41E-13
ENSDARG00000014624	si:dkeyp-94g1.1	184.222783	5.661540716	0.4599647	12.3086417	8.14E-35	8.09E-32
ENSDARG00000017780	rorcb	1334.06796	-1.378935501	0.11409346	-12.0860173	1.25E-33	1.11E-30
ENSDARG00000067958	sh3gl1a	867.306853	0.617159658	0.13066266	4.72330561	2.32E-06	6.35E-05
ENSDARG00000110173	BX323861.3	40.9589222	-1.575738664	0.43124582	-3.6539222	0.00025826	0.00343087
ENSDARG00000052618	ahrrb	62.4425888	1.317580218	0.37821149	3.48371283	0.00049451	0.00571411
ENSDARG00000033231	mcm6l	22.9223464	1.527382053	0.56899361	2.68435712	0.00726694	0.0449415
ENSDARG00000044501	viml	77.4511503	-1.078701578	0.25832407	-4.17576874	2.97E-05	0.00056048
ENSDARG00000078022	fyco1b	980.388442	0.743731692	0.13139453	5.66029403	1.51E-08	7.67E-07
ENSDARG00000103914	THEM6	279.427231	-0.468180085	0.16432468	-2.84911603	0.00438409	0.03083377
ENSDARG00000100260	them6	434.146865	0.609915891	0.14352903	4.24942518	2.14E-05	0.00042752
ENSDARG00000102105	dad1	1729.83792	0.440828385	0.10122508	4.35493248	1.33E-05	0.00028769
ENSDARG00000099839	si:ch211-9d9.1	160.459889	-0.611848341	0.17471046	-3.50207054	0.00046166	0.00541761
ENSDARG00000100184	ly97.2	22.1169073	7.228363295	1.30842438	5.5244792	3.30E-08	1.53E-06
ENSDARG00000102129	crygn1	124.00884	-1.584561385	0.33819743	-4.68531468	2.80E-06	7.44E-05
ENSDARG00000002231	plpp2a	472.572553	0.581789069	0.18005944	3.23109456	0.00123317	0.01163647
ENSDARG00000115610	ccl25a	261.34047	-0.567009384	0.21092069	-2.68825874	0.00718257	0.04453051
ENSDARG00000104244	twsg1a	1291.54428	0.639918443	0.12541918	5.10223739	3.36E-07	1.19E-05
ENSDARG00000040432	klf2b	1459.9338	-0.549614502	0.09111659	-6.0319917	1.62E-09	1.03E-07
ENSDARG00000094889	cplx4c	292.533912	-1.358279774	0.23983486	-5.66339586	1.48E-08	7.59E-07
ENSDARG00000067848	nmrk2	1160.79855	0.700758717	0.22581894	3.10318843	0.00191448	0.01640853
ENSDARG00000097617	si:ch211-178n15.1	400.438168	-0.586861088	0.16077973	-3.65009374	0.00026214	0.00347684
ENSDARG00000067850	jund	1205.22277	0.423719941	0.14026732	3.0208031	0.00252105	0.0203444
ENSDARG00000054666	pgpep1	536.022957	0.583905342	0.12506254	4.66890662	3.03E-06	7.96E-05
ENSDARG00000041435	uba52	22425.0106	0.288952588	0.10337912	2.79507689	0.00518874	0.03499517
ENSDARG00000090086	rab11bb	764.969845	0.453590068	0.16147694	2.8090083	0.00496944	0.03384909
ENSDARG00000068918	map2k2b	653.092769	0.395533843	0.14775109	2.67702826	0.00742784	0.0456187
ENSDARG00000027547	TPGS1	612.181564	0.510667641	0.13273561	3.84725422	0.00011945	0.00181696
ENSDARG00000038476	si:dkeyp-68b7.7	230.966393	-0.467588051	0.14670801	-3.18720195	0.00143656	0.01311749
ENSDARG00000117348	AL954696.2	7.07930255	4.207775891	1.18979651	3.53655088	0.00040539	0.00488411
ENSDARG00000116727	CABZ01081294.2	199.629135	-0.754946338	0.26444659	-2.85481595	0.00430618	0.03041942
ENSDARG00000089172	si:ch1073-391i24.1	238.679313	0.821372907	0.21490259	3.8220708	0.00013234	0.00198384
ENSDARG00000095448	ftr30	30.1883095	2.832440552	0.53092402	5.33492634	9.56E-08	3.93E-06
ENSDARG00000095390	BX537288.2	33.6155938	-2.035313118	0.42500776	-4.78888463	1.68E-06	4.78E-05
ENSDARG00000104071	hikeshi	710.299356	0.346554564	0.10729785	3.22983708	0.00123861	0.01166961
ENSDARG00000102746	tmem39a	1851.44924	0.486116209	0.13259495	3.66617432	0.00024621	0.0032918
ENSDARG00000100223	gpa33a	3112.98386	0.446890728	0.115742	3.86109381	0.00011288	0.00172868
ENSDARG00000088581	f10	1934.20237	1.025884707	0.18547925	5.53099457	3.18E-08	1.48E-06
ENSDARG00000100181	lamp1a	1817.11093	1.075264904	0.17307686	6.21264395	5.21E-10	3.60E-08
ENSDARG00000041589	adprhl1	145.985176	0.627822296	0.21757567	2.88553534	0.00390749	0.02827382
ENSDARG00000058803	grk1a	666.496501	-0.913586855	0.22902515	-3.98902422	6.63E-05	0.00110804
ENSDARG00000104279	appa	15789.1008	0.238263937	0.08781866	2.71313561	0.00666498	0.04204521
ENSDARG00000117321	CZQB01066256.1	200.99717	-0.94268638	0.28472158	-3.31090602	0.00092994	0.00934267
ENSDARG00000007739	atp1a1a.2	4246.62323	-0.546039898	0.13614932	-4.01059596	6.06E-05	0.00102405
ENSDARG00000040245	kpnb3	8475.15307	0.32498054	0.11040445	2.94354573	0.00324476	0.02456915

Table S1. DEGs of WT vs. *terfa+/-*

ENSDARG00000070579	ggact.3	123.996579	0.920957316	0.28221555	3.26331179	0.00110118	0.01062909
ENSDARG00000038248	ggact.2	318.699855	0.813621301	0.20333497	4.00138395	6.30E-05	0.00105951
ENSDARG00000070581	ggact.1	109.041587	1.130603498	0.24946497	4.53211324	5.84E-06	0.00014079
ENSDARG00000037777	stk16	616.519529	0.350743251	0.12698719	2.76203641	0.00574421	0.03776045
ENSDARG00000016706	atic	5886.11364	0.678825776	0.1681212	4.03771663	5.40E-05	0.00093226
ENSDARG00000063297	abcb6a	458.544202	0.53708001	0.14915482	3.60082236	0.00031721	0.00403083
ENSDARG00000063253	hecw2b	512.875518	0.364178855	0.11039722	3.2988046	0.00097097	0.00964571
ENSDARG00000070558	si:ch211-93g23.2	47.4176202	-0.879084351	0.29562383	-2.97365865	0.00294272	0.02287097
ENSDARG00000013741	lancl1	670.189938	-1.271913334	0.10810881	-11.7651215	5.90E-32	4.89E-29
ENSDARG00000074050	efnb2b	470.501826	-0.392313781	0.13080447	-2.99923838	0.00270655	0.02145134
ENSDARG00000018319	arglu1b	2142.91723	-0.447196807	0.0898069	-4.97953742	6.37E-07	2.05E-05
ENSDARG00000092361	si:dkey-79f11.7	31.5902629	-1.606789428	0.39900111	-4.02702999	5.65E-05	0.00096825
ENSDARG00000076176	ptcd1	500.806632	-0.336777328	0.11349689	-2.96728252	0.00300445	0.02320672
ENSDARG00000113858	AL928650.4	53.6349644	-1.694900785	0.43711802	-3.8774443	0.00010556	0.00163673
ENSDARG00000096599	AL928650.3	25.3785675	-3.184010362	0.55598889	-5.72675176	1.02E-08	5.41E-07
ENSDARG00000100771	si:ch211-160d14.15	2.90120452	-5.236782279	1.62917506	-3.21437666	0.00130728	0.01216946
ENSDARG00000037746	actb1	160953.656	0.653655921	0.08333525	7.843691	4.37E-15	7.20E-13
ENSDARG00000037747	fscn1b	938.915468	-0.560553833	0.10727031	-5.22561979	1.74E-07	6.65E-06
ENSDARG00000012458		5109.43455	0.221829174	0.07606224	2.91641665	0.00354077	0.02634119
ENSDARG00000093438	CU467110.1	187.536002	-0.527298278	0.18698389	-2.82001985	0.00480207	0.0329438
ENSDARG00000024740	gng13a	77.8238349	-0.752119471	0.28536679	-2.6356237	0.00839828	0.04993762
ENSDARG00000104203	ugt5b5	3.25440337	-4.32196387	1.61834592	-2.67060571	0.00757145	0.04623795
ENSDARG00000101495	ugt5b2	283.019421	-0.926901543	0.15478315	-5.98838776	2.12E-09	1.30E-07
ENSDARG00000099276	ugt5b2	228.821548	-0.746202847	0.27407391	-2.72263366	0.00647638	0.04120054
ENSDARG00000104995	ugt5b1	98.9203734	-0.839037223	0.31698995	-2.64688907	0.0081236	0.04865351
ENSDARG00000032795	pgap6	633.426984	-0.446474251	0.13520862	-3.30211376	0.00095959	0.00955938
ENSDARG00000008969	fgb	10194.5151	0.382425975	0.08943218	4.2761564	1.90E-05	0.00038518
ENSDARG00000020741	fga	12923.4149	0.355288525	0.0722573	4.91699114	8.79E-07	2.73E-05
ENSDARG00000077533	eif3f	8304.32529	0.30990617	0.10598498	2.92405752	0.00345501	0.02581908
ENSDARG00000062606	si:dkey-26i13.8	217.475858	-0.857445969	0.25184177	-3.40470118	0.00066236	0.00718284
ENSDARG00000002634	b4galt1	579.325322	0.833325016	0.13711201	6.07769517	1.22E-09	7.95E-08
ENSDARG00000018997	cplx2l	3997.26854	-0.278027922	0.09561124	-2.90789991	0.00363865	0.02684434
ENSDARG00000105122	BX323031.2	49.8692413	-1.238579682	0.42554381	-2.91058089	0.00360758	0.02669434
ENSDARG00000117541	FP236197.1	56.2695829	1.565469351	0.50696807	3.08790523	0.00201573	0.01714023
ENSDARG00000056742	crmp1	2077.19515	0.469974911	0.15166792	3.09871009	0.00194365	0.01661832
ENSDARG00000030514	acsl1a	1628.22508	0.465066519	0.09602768	4.84304632	1.28E-06	3.82E-05
ENSDARG00000056369	ufsp2	805.859236	0.451187467	0.1193494	3.78039161	0.00015658	0.0022693
ENSDARG00000062132	cyp4v8	552.89572	-0.540496589	0.1744463	-3.09835511	0.00194598	0.01663252
ENSDARG00000062122	klhl5	412.067259	0.539753556	0.16684347	3.23508943	0.00121605	0.01151422
ENSDARG00000008287	fam114a1	841.518207	0.322457495	0.10542581	3.05861994	0.00222359	0.01846553
ENSDARG00000015495	klf3	2183.02456	0.422003391	0.1478335	2.85458559	0.0043093	0.03042832
ENSDARG00000062055	rnf38	2614.99111	-0.478732589	0.12649274	-3.78466465	0.00015392	0.00223587
ENSDARG00000086957	si:dkeyp-118a3.2	197.821834	-0.816624782	0.25789271	-3.16652911	0.0015427	0.01386254
ENSDARG00000056151	tyrp1b	8068.22592	0.390887034	0.11187264	3.49403609	0.00047578	0.00555706
ENSDARG00000003631	clockb	1876.31401	-0.33875043	0.11289165	-3.00066862	0.00269388	0.0213713
ENSDARG00000070321	clrn2	106.601402	-1.046452647	0.29961872	-3.49261435	0.00047832	0.00558051
ENSDARG00000037487	1-Mar	226.723205	-0.813090107	0.21756782	-3.73717997	0.0001861	0.0026147
ENSDARG00000079250	BX323590.1	366.165317	0.484977964	0.17592783	2.75668707	0.00583902	0.03822198
ENSDARG00000080019	UBA6	829.916341	-0.32778483	0.09872582	-3.32015303	0.00089968	0.00914958
ENSDARG00000055876	msmo1	246.856509	0.784270419	0.18325517	4.27966338	1.87E-05	0.00038071
ENSDARG00000033273	primpol	389.017246	-0.663409552	0.19758745	-3.35754903	0.00078637	0.00821571
ENSDARG00000055439	adamtsl7	519.313662	0.760341561	0.14513801	5.23874855	1.62E-07	6.24E-06
ENSDARG00000000606	dnah6	307.469817	0.685813405	0.18633582	3.68052374	0.00023276	0.00314068
ENSDARG00000034007	prom1b	2829.43744	-0.531424716	0.14305296	-3.71488098	0.0002033	0.00281035
ENSDARG00000061121	adgrl3.1	4068.10106	0.388197363	0.14466027	2.68351056	0.00728537	0.0449771
ENSDARG00000061451	n4bp2	672.991124	-0.533521753	0.11299729	-4.72154456	2.34E-06	6.39E-05
ENSDARG00000092290	si:dkeyp-26a9.2	358.846018	-0.497499589	0.1853294	-2.68440731	0.00726585	0.0449415
ENSDARG00000004246	slit2	3505.01897	0.354748782	0.10745135	3.30148268	0.00096175	0.00957323

Table S1. DEGs of WT vs. *terfa+/-*

ENSDARG00000060994	fbxw7	2439.9046	0.258165955	0.08465417	3.04965445	0.00229105	0.01893085
ENSDARG00000070050	sfrp2	2031.4408	-0.277772051	0.09214854	-3.0143945	0.00257493	0.0207078
ENSDARG00000037281	fgg	6969.63654	0.320281543	0.1086945	2.94662141	0.00321266	0.02435959
ENSDARG00000070039	NA	4.16005236	-6.321833047	1.59554969	-3.9621662	7.43E-05	0.00121451
ENSDARG00000006427	fabp2	4550.16407	-0.567792644	0.15594406	-3.64100213	0.00027158	0.0035677
ENSDARG00000076499	usp53b	223.35074	-0.595762398	0.20059099	-2.97003573	0.00297765	0.0230701
ENSDARG00000037262	cdkn2a/b	32.2589869	1.96979965	0.52887245	3.72452687	0.00019568	0.0027278
ENSDARG00000014790	g3bp2	2267.11082	-0.409804299	0.11178658	-3.66595272	0.00024642	0.00329288
ENSDARG00000031632	chst10	503.761242	-0.353258705	0.11929654	-2.9611814	0.00306461	0.02355682
ENSDARG00000089334	ednrba	243.433355	-0.474091433	0.17627773	-2.68945738	0.00715683	0.04440414
ENSDARG00000053456	GK3P	1096.45415	-0.45548955	0.09032252	-5.04292367	4.58E-07	1.55E-05
ENSDARG00000053466	timmdc1	924.87922	-0.285538893	0.10728814	-2.66142093	0.00778116	0.04719452
ENSDARG00000037109	pwp2h	1371.58187	-0.297777158	0.10186143	-2.92335532	0.00346281	0.0258696
ENSDARG00000053502	cryaa	823.374982	-1.62745948	0.18454288	-8.81886918	1.16E-18	3.12E-16
ENSDARG00000069804	si:ch211-198k9.6	53.0120537	0.883328715	0.31757568	2.78147473	0.00541125	0.0361065
ENSDARG00000096544	CR774189.1	5.37131613	4.944365115	1.48655205	3.32606255	0.00088082	0.00900741
ENSDARG00000093065	ubac2	320.521296	-0.461406225	0.1340741	-3.44142703	0.00057865	0.00644664
ENSDARG00000042688	bora	184.779558	-0.673591752	0.21099799	-3.19240839	0.00141092	0.01294512
ENSDARG00000096527	NA	7.73156052	-6.171441895	1.46718089	-4.20632654	2.60E-05	0.00050361
ENSDARG00000069946	itga6b	2702.65984	0.538720484	0.14603554	3.68896826	0.00022517	0.00305652
ENSDARG00000069953	kcnq5b	180.760387	-0.484620923	0.17665805	-2.74327111	0.00608304	0.03931203
ENSDARG00000053269	slc2a15b	467.3446	0.69471251	0.14869156	4.67217172	2.98E-06	7.86E-05
ENSDARG00000039613	poll	490.985284	-0.81527745	0.11444695	-7.12362741	1.05E-12	1.19E-10
ENSDARG00000058231	nt5c2b	2907.77315	-0.448510105	0.09001327	-4.98271093	6.27E-07	2.02E-05
ENSDARG00000053248	inab	1528.27516	0.320176718	0.11025326	2.90401127	0.00368415	0.02709752
ENSDARG00000033380	gyg2	32.3548402	-1.434533711	0.48235929	-2.97399417	0.00293951	0.02285312
ENSDARG00000079350	znf654	1277.66397	-0.46074392	0.09006783	-5.11552161	3.13E-07	1.12E-05
ENSDARG00000032056	arl6	518.654902	-0.334316852	0.12494409	-2.67573167	0.00745663	0.04571647
ENSDARG00000069910	gtf2f2a	1458.39482	-0.423009188	0.09508967	-4.44852938	8.65E-06	0.00019697
ENSDARG00000037018	gab1	1154.89255	0.270245487	0.10099093	2.67593806	0.00745204	0.04571647
ENSDARG00000094648	si:ch211-194g2.4	17.5542385	1.394240123	0.5093132	2.73749065	0.00619099	0.03980622
ENSDARG00000060383	mmaa	633.041603	0.289985528	0.10993306	2.63783733	0.00834366	0.04970797
ENSDARG00000036995	lsm6	1138.27141	0.46992736	0.16031149	2.93133918	0.00337504	0.0253128
ENSDARG00000074989	sparcl1	814.433752	-0.806952266	0.14049162	-5.74377503	9.26E-09	4.93E-07
ENSDARG00000057303	galnt7	2611.58306	0.596940747	0.07788988	7.66390667	1.80E-14	2.72E-12
ENSDARG00000079183	sod3b	55.817088	-0.898529749	0.33329413	-2.6959063	0.00701974	0.04375017
ENSDARG00000076585	si:ch211-113e8.11	700.85591	-0.353864522	0.13192574	-2.68230082	0.00731177	0.04509528
ENSDARG00000031435	TXN	6988.30025	0.387719707	0.10174782	3.81059475	0.00013863	0.00205961
ENSDARG00000117450	FO834828.2	488.258713	1.180116417	0.2151523	5.48502823	4.13E-08	1.86E-06
ENSDARG00000117485	FO681323.1	831.266605	1.275505696	0.13684161	9.32103672	1.15E-20	3.77E-18
ENSDARG00000117707	CABZ01072532.1	122.465452	1.186815424	0.37964198	3.12614377	0.00177115	0.01547343
ENSDARG00000052895	htra3a	28.2051543	1.417381283	0.48636992	2.91420424	0.00356597	0.02649241
ENSDARG00000069669	adra2c	120.194531	-0.697457929	0.1897742	-3.67519896	0.00023766	0.00319305
ENSDARG00000008706	paip2b	4432.15237	0.329506133	0.10328089	3.19038812	0.00142082	0.01301145
ENSDARG00000078108	dok1a	195.425319	-0.515662188	0.16712065	-3.08556841	0.00203163	0.01723423
ENSDARG00000092551	si:dkey-56e3.2	90.5230092	0.910758259	0.28703025	3.17303931	0.00150852	0.01362787
ENSDARG00000024785	ctnna2	2750.17232	0.377895379	0.13825852	2.73325194	0.00627123	0.04019379
ENSDARG00000087061	si:ch211-71k14.1	6.63284978	-3.11462279	0.95615751	-3.25743693	0.00112423	0.01081378
ENSDARG00000052712	suclg1	10029.2713	0.24102788	0.08943244	2.69508338	0.0070371	0.04383638
ENSDARG00000095949	si:dkey-22i16.9	54.608305	1.568217154	0.51828338	3.02579094	0.00247984	0.02010318
ENSDARG00000059682	slc43a3a	261.237457	-0.973303095	0.16012895	-6.0782455	1.22E-09	7.95E-08
ENSDARG00000078233	ctnnd1	4986.64867	0.277108018	0.06902902	4.01436963	5.96E-05	0.00101214
ENSDARG00000014803	cryba1l2	345.250317	-1.416729723	0.1803037	-7.85746332	3.92E-15	6.51E-13
ENSDARG00000016793	crybb1l2	17908.1113	-1.60890749	0.19085698	-8.42991164	3.46E-17	7.89E-15
ENSDARG00000007786	tmx2b	2367.76328	-0.345618712	0.08504367	-4.06401469	4.82E-05	0.00084728
ENSDARG00000051989	tmem187	139.265098	0.736538815	0.20212918	3.6439014	0.00026854	0.00353708
ENSDARG00000051981	STX3	368.434022	-0.554200913	0.14041912	-3.94676242	7.92E-05	0.00128267
ENSDARG00000016835	tcirg1a	582.106135	-0.337092256	0.12231168	-2.75601044	0.00585111	0.0382709

Table S1. DEGs of WT vs. *terfa+/-*

ENSDARG00000094133	wu:fc21g02	4664.0518	-0.476374721	0.08651929	-5.50599433	3.67E-08	1.69E-06
ENSDARG00000093289	si:dkey-9i23.14	26.3214629	-1.346799571	0.4823297	-2.79227999	0.0052338	0.03521554
ENSDARG00000030972	dnaja1	635.202835	0.513962178	0.12988692	3.9569971	7.59E-05	0.00123701
ENSDARG00000051970	smu1b	312.559187	0.478900795	0.17093965	2.80157819	0.00508533	0.03448272
ENSDARG00000069559	muc13a	1008.90696	-0.445030264	0.16364746	-2.71944505	0.00653916	0.04145982
ENSDARG00000069566	mucms1	24.2364525	4.322402503	0.83878957	5.15314287	2.56E-07	9.39E-06
ENSDARG00000069632	emp1	207.636917	-0.456098819	0.1692912	-2.6941673	0.00705647	0.04390204
ENSDARG00000060184	pkn1a	1740.97022	0.361337025	0.09450234	3.82357762	0.00013153	0.00197532
ENSDARG00000081252	dre-mir-16b	8.04201124	5.537526938	1.33525861	4.14715688	3.37E-05	0.00062451
ENSDARG00000069471	mhc1zca	177.169054	-0.794381859	0.16755512	-4.74101799	2.13E-06	5.91E-05
ENSDARG00000036588	mhc1zba	1001.64753	-0.507440628	0.1852783	-2.73880232	0.00616634	0.03968526
ENSDARG00000015076	gja8b	205.803928	-1.559665758	0.19598371	-7.95813991	1.75E-15	3.10E-13
ENSDARG00000096003	atp5md	1754.10179	1.023489212	0.13856672	7.38625558	1.51E-13	1.92E-11
ENSDARG00000022891	zgc:175214	2286.83806	-0.238657279	0.08740842	-2.73036942	0.00632634	0.04043229
ENSDARG00000069415	col17a1a	23084.1169	0.387136544	0.11573919	3.3449046	0.00082311	0.00851014
ENSDARG00000013667	tbck	888.856856	0.448163879	0.125355	3.57515756	0.00035002	0.00434115
ENSDARG00000016818	abcg2d	94.4860709	1.038133829	0.27320241	3.79987063	0.00014477	0.0021254
ENSDARG00000010655	ppm1k	1284.63235	-0.350181348	0.10147322	-3.45097302	0.00055857	0.00629055
ENSDARG00000075887	herc3	704.888504	0.340078892	0.10928171	3.11194705	0.00185858	0.01601212
ENSDARG00000075043	si:dkeyp-123h10.2	667.675541	-0.48958642	0.13470113	-3.63461266	0.0002784	0.00364381
ENSDARG00000015540	psen2	1108.38532	0.517413373	0.11249969	4.59924252	4.24E-06	0.00010689
ENSDARG00000005544	btbd3a	232.543135	0.790603366	0.24861558	3.18002336	0.00147263	0.01337633
ENSDARG00000008740	esf1	857.243749	0.532164189	0.12240768	4.34747375	1.38E-05	0.00029482
ENSDARG00000052435	spred2a	1576.45841	0.27365944	0.09535737	2.86983008	0.00410692	0.02938331
ENSDARG00000086184	FBXO48	522.363262	-0.513339482	0.11712573	-4.38280702	1.17E-05	0.00025816
ENSDARG00000018891	rnaseh2a	746.314103	-0.366703059	0.12272593	-2.98798351	0.00280825	0.02206036
ENSDARG00000010442	rnf11a	801.265154	-0.286727846	0.10533804	-2.72197827	0.00648924	0.04125066
ENSDARG00000079163	best2	24.7268009	2.684198871	0.62304891	4.30816717	1.65E-05	0.00034152
ENSDARG00000117216	LO017718.1	182.768854	1.579370715	0.48463926	3.25885835	0.00111862	0.01076808
ENSDARG00000093494	si:ch211-217k17.9	1380.87321	0.755014141	0.11366246	6.64259913	3.08E-11	2.66E-09
ENSDARG00000074635	abca1a	8668.55984	-0.457594559	0.108142	-4.23142319	2.32E-05	0.00045703
ENSDARG00000075201	inpp4b	903.157418	0.326272553	0.11764235	2.77342767	0.00554692	0.03685341
ENSDARG00000023151	ucp1	2229.29171	0.923399928	0.15143628	6.09761355	1.08E-09	7.12E-08
ENSDARG00000031203	commd1	668.047647	0.821416143	0.19525818	4.20682066	2.59E-05	0.0005033
ENSDARG00000102252	USP34	2353.10307	0.587160536	0.12171723	4.82397219	1.41E-06	4.15E-05
ENSDARG00000078041	xpo1a	4357.74861	0.47876463	0.09723613	4.92373163	8.49E-07	2.64E-05
ENSDARG00000089742	fam161a	471.130254	-0.844107284	0.16986768	-4.96920484	6.72E-07	2.15E-05
ENSDARG00000013475	cct4	12780.8492	0.407955361	0.11441889	3.56545464	0.00036323	0.00447595
ENSDARG00000012341	capn9	1715.77532	0.474122942	0.1001195	4.73557032	2.18E-06	6.03E-05
ENSDARG00000036371	acta1a	22059.5386	1.206277329	0.13276884	9.08554513	1.03E-19	3.05E-17
ENSDARG00000025325	ccsapa	444.772815	-0.355441973	0.12815923	-2.77344023	0.0055467	0.03685341
ENSDARG00000075870	triobpa	291.56924	0.697377259	0.18112962	3.85015571	0.00011804	0.00179997
ENSDARG00000077790	dcaf15	538.065076	0.36867484	0.11120702	3.31521183	0.00091574	0.00925229
ENSDARG00000117445	CABZ01067151.2	109.875942	-0.772456985	0.27461384	-2.81288435	0.00490993	0.03350805
ENSDARG00000103125	tsc2	2058.34351	0.437232169	0.09933323	4.40167087	1.07E-05	0.00023946
ENSDARG00000091320	nlrc6	113.058019	-1.431697453	0.30972643	-4.62245807	3.79E-06	9.66E-05
ENSDARG00000059835	zfyve27	811.535517	0.330623863	0.11193962	2.95359105	0.003141	0.02396977
ENSDARG00000059826	crtac1a	535.681496	-2.057661552	0.24876368	-8.27155132	1.32E-16	2.74E-14
ENSDARG00000026489	khsrp	4170.96343	0.737463084	0.15986921	4.61291502	3.97E-06	0.0001005
ENSDARG00000031952	mb	1048.12234	-0.698955809	0.12208335	-5.72523436	1.03E-08	5.45E-07
ENSDARG00000019765	zgc:158803	2061.29707	0.324413598	0.08893507	3.64775779	0.00026454	0.0034974
ENSDARG00000096576	si:ch211-286b5.6	7.388963	3.585461133	1.11102174	3.22717459	0.00125019	0.01175186
ENSDARG00000041394	dnajb1b	285.699844	-0.412741653	0.14578025	-2.83125908	0.00463651	0.03208257
ENSDARG00000043719	c3a.6	2099.52699	0.491208326	0.14750217	3.33017702	0.00086791	0.00889928
ENSDARG00000012694	c3a.1	25373.7829	0.406880551	0.10923605	3.72478266	0.00019548	0.00272657
ENSDARG00000087359	c3a.2	1475.28368	1.735526812	0.51867726	3.34606306	0.00081968	0.00848525
ENSDARG00000052207	c3a.3	2223.37449	-0.880292331	0.19287103	-4.56415008	5.02E-06	0.00012354
ENSDARG00000074069	zgc:171452	25.5522638	1.660634679	0.61349472	2.7068443	0.00679261	0.04267703

Table S1. DEGs of WT vs. *terfa+/-*

ENSDARG00000092358	BX469930.1	275.224269	-5.740788163	0.77408705	-7.41620493	1.21E-13	1.59E-11
ENSDARG00000052039	caspb	5125.30121	0.452510044	0.15140693	2.98870099	0.00280166	0.02202255
ENSDARG00000099301	cdc37	4607.13798	0.416344311	0.10422043	3.99484339	6.47E-05	0.00108499
ENSDARG00000099741	mvb12a	361.287576	0.585125101	0.13654155	4.28532631	1.82E-05	0.00037266
ENSDARG00000102091	CABZ01052576.1	659.081567	0.542698498	0.18885759	2.87358591	0.00405841	0.02913683
ENSDARG00000100739	CABZ01052573.1	1260.0859	0.807513654	0.117763	6.85710858	7.03E-12	6.82E-10
ENSDARG00000078042	il10rb	1045.15027	-0.269863579	0.09790137	-2.75648408	0.00584265	0.03823564
ENSDARG00000104528	cldn10b	84.0966789	-1.164257955	0.29310601	-3.97213945	7.12E-05	0.00117156
ENSDARG00000105042	cldn10l2	13.4732048	-1.844828377	0.61666968	-2.99159895	0.00277521	0.02187686
ENSDARG00000099148	bzw1b	3463.44789	-0.378720536	0.12733554	-2.97419342	0.0029376	0.02284543
ENSDARG00000100887	si:dkey-11f4.20	28.2635815	1.426396943	0.46310991	3.08003975	0.00206973	0.01747983
ENSDARG00000059294	marco	371.026678	0.972548229	0.19270919	5.04671442	4.49E-07	1.52E-05
ENSDARG00000100315	slc15a1a	69.8941947	-1.173416056	0.40993978	-2.8624108	0.00420432	0.02994108
ENSDARG00000104727	ino80da	286.052934	0.633759753	0.20186058	3.13959152	0.00169184	0.01491684
ENSDARG00000094004	FP102120.1	226.700858	0.603937504	0.19588327	3.08315005	0.00204822	0.01734531
ENSDARG00000096896	BX004991.1	173.223588	-0.501971845	0.18772469	-2.67397883	0.00749572	0.04591081
ENSDARG00000059351	hnrnpa3	491.120311	1.030171336	0.17164547	6.001739	1.95E-09	1.21E-07
ENSDARG00000059280	hoxd3a	1009.13683	-0.353991738	0.09339018	-3.79046012	0.00015037	0.00219812
ENSDARG00000059255	evx2	426.06983	0.4303826	0.14886593	2.8910752	0.00383926	0.02791833
ENSDARG00000011652	ftr52p	67.7538011	0.716927969	0.263529	2.72048986	0.00651853	0.04138024
ENSDARG00000017359	mettl8	67.934929	-0.81097286	0.26349887	-3.07770903	0.00208598	0.01755745
ENSDARG00000099227	galnt13	582.513331	-0.373343429	0.11370098	-3.28355498	0.00102507	0.01003862
ENSDARG00000114227	CABZ01075242.1	215.929346	0.789427403	0.18643355	4.23436332	2.29E-05	0.00045248
ENSDARG00000063352	inpp4aa	1867.52205	0.330995163	0.10583908	3.12734365	0.00176394	0.01541583
ENSDARG00000075282	irs2b	2335.0896	0.440589049	0.1108282	3.97542381	7.03E-05	0.00115947
ENSDARG00000004771	ankrd10b	809.888405	-0.527791295	0.11552636	-4.56857891	4.91E-06	0.0001212
ENSDARG00000010946	cbsb	2075.83428	0.638411618	0.09735564	6.55752078	5.47E-11	4.58E-09
ENSDARG00000095022	thsd7ba	998.290101	0.358353272	0.13535633	2.64748076	0.0081094	0.04859674
ENSDARG00000109373	tmbim1b	130.217018	0.64346968	0.23895592	2.69283842	0.00708466	0.04406637
ENSDARG00000024112	gmppaa	503.187005	-0.339345751	0.12020592	-2.82303694	0.00475711	0.03268951
ENSDARG00000078392	chpfa	544.217272	0.423172109	0.15001881	2.82079373	0.0047905	0.03287351
ENSDARG00000078671	cdk5r2b	452.44812	-0.442675887	0.1573391	-2.81351478	0.00490031	0.03346999
ENSDARG00000041925	cryba2b	10769.6706	-0.928422937	0.12834196	-7.23397816	4.69E-13	5.55E-11
ENSDARG00000070057	si:dkey-69o16.5	793.658129	-0.444064367	0.11382187	-3.9013975	9.56E-05	0.00150369
ENSDARG00000006314	itgav	9422.46139	0.38968586	0.09000578	4.32956472	1.49E-05	0.00031497
ENSDARG00000015889	zc3h15	2198.12073	0.295640421	0.0881572	3.35355971	0.00079779	0.00831061
ENSDARG00000009839	fam117bb	502.974665	0.387973881	0.12762171	3.04003051	0.00236554	0.01941088
ENSDARG00000019950	carf	747.438408	1.161521454	0.10419449	11.1476287	7.35E-29	4.69E-26
ENSDARG00000058222	mpp4a	282.73075	-0.749599995	0.19433413	-3.85727403	0.00011466	0.00175375
ENSDARG00000019815	fn1a	9626.90893	0.421192698	0.10191154	4.13292446	3.58E-05	0.00065903
ENSDARG00000024694	myo1b	3692.5819	-0.250529474	0.08857831	-2.82833869	0.00467903	0.03229581
ENSDARG00000102261	RSPH1	197.494978	0.579858943	0.17774655	3.2622796	0.0011052	0.01066373
ENSDARG00000103505	fbxl3a	1047.27529	-0.471659105	0.11447322	-4.12025699	3.78E-05	0.00068816
ENSDARG00000041220	ftr53	39.8154751	1.081403238	0.32915834	3.28535875	0.00101853	0.01000611
ENSDARG00000078001	kbtbd7	194.991581	-0.572963618	0.17213214	-3.32862666	0.00087275	0.0089379
ENSDARG00000078593	vwa8	1313.78611	0.37647526	0.10540432	3.57172499	0.00035464	0.00438752
ENSDARG00000038306	tsc22d1	4975.89443	0.287104166	0.09564628	3.00172853	0.00268451	0.02134472
ENSDARG00000093471	CR361551.1	44.8493558	-0.945540871	0.30611456	-3.08884641	0.00200935	0.01709188
ENSDARG00000068681	crfb1	164.004499	-0.484862749	0.18072358	-2.68289699	0.00729875	0.04503733
ENSDARG00000086103	slc37a1	497.201408	0.587271089	0.13525398	4.34198761	1.41E-05	0.00030099
ENSDARG00000093349	si:ch211-141e20.2	84.9958709	-0.68638626	0.26003365	-2.63960548	0.00830026	0.04948499
ENSDARG00000092425	BX677668.2	15.9505797	-1.78533623	0.60416494	-2.95504775	0.00312621	0.02389356
ENSDARG00000077002	igsf3	1557.8178	0.661014383	0.12885458	5.12992534	2.90E-07	1.05E-05
ENSDARG00000093224	CR456628.1	8.75850396	-2.646854562	0.78511469	-3.3712967	0.00074815	0.00790954
ENSDARG00000069934	parp4	1211.86397	0.415654261	0.13903908	2.98947799	0.00279455	0.02200574
ENSDARG00000067751	si:rp71-68n21.9	267.015364	-0.775249745	0.19443788	-3.98713339	6.69E-05	0.00111558
ENSDARG00000093452	BX511129.2	40.8920114	1.091898764	0.34911377	3.12763017	0.00176222	0.01540623
ENSDARG00000102176	pla1a	96.460637	0.848022408	0.28815288	2.94296	0.0032509	0.02458082

Table S1. DEGs of WT vs. *terfa+/-*

ENSDARG00000069920	cox17	1025.39044	-0.307857451	0.09830599	-3.13162445	0.00173842	0.01525436
ENSDARG00000112751	BX511129.3	192.288974	1.072584608	0.16828099	6.37377171	1.84E-10	1.39E-08
ENSDARG00000021889	gja3	114.416437	-2.016553336	0.21777895	-9.25963392	2.05E-20	6.54E-18
ENSDARG00000079575	aff3	598.09278	-0.423964875	0.1358698	-3.12037616	0.0018062	0.01566405
ENSDARG00000069846	zgc:162944	1663.22453	0.402762177	0.10182454	3.95545296	7.64E-05	0.00124421
ENSDARG00000069843	kctd12.1	248.533201	-0.55993653	0.16169725	-3.46286981	0.00053445	0.00607671
ENSDARG00000030177	uchl3	1046.97027	-0.311410515	0.10018302	-3.10841613	0.00188093	0.01617665
ENSDARG00000093318	si:dkey-57a22.15	683.797771	-0.860698271	0.16480559	-5.22250656	1.77E-07	6.72E-06
ENSDARG00000117097	crygm2c	12.9820326	-1.769662623	0.64504175	-2.74348538	0.00607908	0.03930577
ENSDARG00000018797	crygmxl1	89.2124759	-1.233440637	0.31644525	-3.89780107	9.71E-05	0.00152227
ENSDARG00000044875	crygm2e	22.4868929	-1.565200666	0.42452776	-3.6869218	0.00022698	0.00307615
ENSDARG00000069827	crygm2d11	4591.48097	-0.641693912	0.1462793	-4.38677191	1.15E-05	0.00025372
ENSDARG00000069826	crygm2d15	5465.15863	-0.721315766	0.12507448	-5.76709	8.07E-09	4.35E-07
ENSDARG00000116164	crygm2d8	13636.7751	-0.375390506	0.13247246	-2.8337248	0.00460089	0.03188046
ENSDARG00000073874	crygm2d6	2343.16603	-0.763301905	0.14016562	-5.44571419	5.16E-08	2.26E-06
ENSDARG00000091148	crygm2d20	10625.4806	-0.626805204	0.1505301	-4.16398587	3.13E-05	0.00058533
ENSDARG00000115701	crygm2d9	6744.11373	-0.485011779	0.16989529	-2.85476882	0.00430682	0.03041942
ENSDARG00000078134	crygm2f	13.585044	2.599940966	0.64750887	4.01529783	5.94E-05	0.00100934
ENSDARG00000041830	tsn	701.967793	-0.420691658	0.13033037	-3.22788664	0.00124708	0.01173212
ENSDARG00000077643	lypd6b	290.601836	-0.613468546	0.18679303	-3.28421534	0.00102267	0.01002698
ENSDARG00000095527	CR392363.2	9.18990849	3.243608284	0.97524383	3.32594597	0.00088119	0.00900741
ENSDARG00000006848	parp9	220.498158	-0.960999799	0.2159868	-4.449345	8.61E-06	0.00019659
ENSDARG00000006422	esyt3	593.059759	0.804835453	0.15472908	5.20157846	1.98E-07	7.44E-06
ENSDARG00000095915	col6a3	298.397963	0.960139235	0.20905372	4.59278722	4.37E-06	0.00010981
ENSDARG00000062423	zgc:153901	169.690724	0.719926223	0.21660836	3.32363083	0.00088854	0.00906593
ENSDARG00000027611	cavin2a	2634.87202	0.602209624	0.08600371	7.00213528	2.52E-12	2.62E-10
ENSDARG00000005823	slc39a10	1772.56057	0.434876689	0.09334944	4.65858914	3.18E-06	8.27E-05
ENSDARG00000094844	BX323457.3	8.72286763	4.799936652	1.52111195	3.15554464	0.00160199	0.01429196
ENSDARG00000093998	si:ch73-7i4.2	50.0372879	2.286978721	0.53308319	4.2900972	1.79E-05	0.00036565
ENSDARG00000077403	col8a1a	1153.84937	-0.746470877	0.22535581	-3.31241018	0.00092496	0.00932267
ENSDARG00000003641	tfg	3982.68916	0.342419853	0.09386195	3.64812226	0.00026416	0.0034943
ENSDARG00000062156	abi3bpa	128.85839	-0.953229494	0.22115176	-4.31029574	1.63E-05	0.00033854
ENSDARG00000019782	impg2a	205.709059	-1.011079776	0.25868438	-3.90854592	9.29E-05	0.00146346
ENSDARG00000041051	mid1ip1a	1575.3727	0.360355156	0.10680514	3.37394963	0.00074098	0.00785189
ENSDARG00000062147	otc	158.780161	-0.90642497	0.21640635	-4.18853228	2.81E-05	0.00053474
ENSDARG00000010318	srpx	1817.48303	0.424395344	0.08465387	5.01330089	5.35E-07	1.76E-05
ENSDARG00000069451	gja8a	112.536195	-2.099124505	0.23010312	-9.12253815	7.34E-20	2.20E-17
ENSDARG00000095912	si:dkey-229b18.3	665.237282	-0.396572651	0.10374052	-3.82273641	0.00013198	0.00197968
ENSDARG00000056191	si:ch211-168k14.2	59.1965713	-0.691442425	0.25221309	-2.74150092	0.00611592	0.03947317
ENSDARG00000056160	hspd1	10671.7678	0.402059469	0.0919114	4.37442438	1.22E-05	0.00026662
ENSDARG00000031680	ercc1	260.393647	-0.492015777	0.15756393	-3.12264217	0.00179236	0.01558887
ENSDARG00000020618	gatd3a	761.861763	0.511960768	0.12986336	3.94230352	8.07E-05	0.00130167
ENSDARG00000007077	ankrd50l	1498.91578	0.354213522	0.11681378	3.03229227	0.00242704	0.01975249
ENSDARG00000077812	slc5a3b	237.787944	-0.611599921	0.17987874	-3.40006778	0.00067369	0.00728343
ENSDARG00000020573	ddx3xa	6749.54922	-0.516450945	0.08213554	-6.28778871	3.22E-10	2.32E-08
ENSDARG00000023712	mao	1316.8492	-0.416912248	0.09040986	-4.61135802	4.00E-06	0.00010115
ENSDARG00000055705	f5	1364.3219	0.613742667	0.20795779	2.95128482	0.00316455	0.02410324
ENSDARG00000074225	si:ch211-269e2.1	1088.77034	-0.31588935	0.10856564	-2.90966228	0.00361819	0.02673313
ENSDARG00000055415	zp2l1	8.3773318	3.834953489	1.12923385	3.39606672	0.00068362	0.00735554
ENSDARG00000093550	BX005438.1	41.6428656	-1.077584276	0.38381526	-2.80755971	0.00499184	0.03395518
ENSDARG00000025641	gli2a	2244.25277	0.254795186	0.08465586	3.00977609	0.0026144	0.02094135
ENSDARG00000020028	cps1	177.376676	-2.198466261	0.27497086	-7.99526989	1.29E-15	2.33E-13
ENSDARG00000069111	ikzf2	235.395941	0.52529641	0.16078929	3.26698634	0.00108699	0.01051659
ENSDARG00000074749	abca12	3978.97817	0.596032099	0.09566571	6.23036306	4.65E-10	3.27E-08
ENSDARG00000093484	si:dkey-95p16.2	235.514581	-0.912753985	0.18481567	-4.93872629	7.86E-07	2.46E-05
ENSDARG00000045175	pofut2	348.622532	0.422083484	0.13306646	3.17197498	0.00151406	0.01363652
ENSDARG00000028731	stat4	246.420373	-0.476461192	0.16907527	-2.81804186	0.00483175	0.03310176
ENSDARG00000036558	col18a1a	20473.7233	0.430396422	0.08923392	4.82323789	1.41E-06	4.15E-05

Table S1. DEGs of WT vs. *terfa+/-*

ENSDARG00000010962	fkbp7	951.291289	0.620824673	0.12203238	5.08737655	3.63E-07	1.27E-05
ENSDARG00000079397	cerkl	124.190675	-0.807342271	0.20506504	-3.93700595	8.25E-05	0.00132471
ENSDARG00000016939	itgb2	225.555266	-0.584680316	0.14621195	-3.99885452	6.36E-05	0.00106821
ENSDARG00000060682	agr1	2665.23266	0.605424434	0.12457979	4.85973215	1.18E-06	3.55E-05
ENSDARG00000023302	gpbar1	24.3290854	1.555023069	0.43291853	3.59195315	0.00032821	0.00412627
ENSDARG00000041339	zgc:92380	3683.37486	1.021029381	0.1065513	9.58251417	9.47E-22	3.32E-19
ENSDARG00000068893	mettl5	435.596305	0.463977816	0.11631317	3.9890393	6.63E-05	0.00110804
ENSDARG00000076321	col28a2a	3366.02996	2.501035264	0.19988612	12.5123006	6.39E-36	7.22E-33
ENSDARG00000102506	lrp2a	3520.60516	0.46332609	0.12926814	3.58422488	0.00033808	0.00422686
ENSDARG00000019260	dhrs9	285.32017	-0.733407342	0.25838021	-2.83848105	0.00453288	0.03154109
ENSDARG00000091090	xirp2b	2056.6231	-0.685137589	0.21389173	-3.20319808	0.0013591	0.01254849
ENSDARG00000008662	fign	310.871221	0.605278759	0.15664949	3.86390516	0.00011159	0.00171425
ENSDARG00000078468	fap	1838.36979	-0.337292078	0.08783978	-3.83985581	0.00012311	0.00186574
ENSDARG00000099298	xrcc5	1844.3099	0.516522196	0.12530628	4.12207756	3.75E-05	0.00068369
ENSDARG00000100133	cnmd	6517.34961	-0.300701949	0.09158839	-3.2831884	0.0010264	0.01004683
ENSDARG00000068812	tlr7	13.658082	-1.673882359	0.58143554	-2.87887865	0.00399092	0.02875197
ENSDARG00000104832	CU914164.1	20.9975678	-1.945822478	0.51093989	-3.80831972	0.00013991	0.00207492
ENSDARG00000077777	tmsb4x	26888.1258	0.376155698	0.13106427	2.87000941	0.0041046	0.02938331
ENSDARG00000097660	trappc2	849.109639	0.594794557	0.10730579	5.54298661	2.97E-08	1.39E-06
ENSDARG00000098790	sowahca	133.125452	-0.668064059	0.24739705	-2.70037198	0.0069262	0.04337381
ENSDARG00000099236	10-Sep	3952.54279	0.357466932	0.09167599	3.89924279	9.65E-05	0.00151507
ENSDARG00000053365	rpl31	41405.7077	0.473403303	0.11765708	4.02358533	5.73E-05	0.00098113
ENSDARG00000023188	lcp1	665.673266	-0.417962741	0.12726092	-3.28429753	0.00102237	0.01002698
ENSDARG00000091138	actr3	6993.35083	0.337436498	0.09613673	3.50996442	0.00044817	0.00531196
ENSDARG00000098834	sox4b	5282.10892	0.49012827	0.1178459	4.15906072	3.20E-05	0.00059754
ENSDARG00000088689	si:ch211-201o1.1	57.8146501	-1.307225451	0.30876216	-4.23376183	2.30E-05	0.00045334
ENSDARG00000086678	ascc3	1796.2649	0.818394519	0.1077455	7.59562612	3.06E-14	4.45E-12
ENSDARG00000051912	hpxb	362.233996	-1.199259596	0.25035929	-4.79015426	1.67E-06	4.76E-05
ENSDARG00000101602	preb	1200.96486	0.413322902	0.09002199	4.59135507	4.40E-06	0.00011023
ENSDARG00000088764	lyplal1	137.119871	0.808833125	0.22487782	3.59676706	0.0003222	0.00408165
ENSDARG00000045959	csf3r	80.0931434	-0.777670754	0.2595148	-2.99663355	0.00272979	0.02160103
ENSDARG00000041340	mrpl51	1166.01513	-0.376719112	0.10510985	-3.58405154	0.00033831	0.00422754
ENSDARG00000004964	cyp4t8	193.390127	-0.620965746	0.22637313	-2.74310706	0.00608608	0.03932145
ENSDARG00000058964	themis2	27.0637924	-1.387214244	0.47638469	-2.91196228	0.00359166	0.02660091
ENSDARG00000037186	tpbgb	475.750352	0.388993215	0.12236684	3.17891042	0.0014783	0.01341391
ENSDARG00000116442	TENT5A	192.37631	-0.557395716	0.16193665	-3.44206033	0.0005773	0.00644023
ENSDARG00000041363	dctn3	1376.73481	-0.49008821	0.10531341	-4.6536163	3.26E-06	8.42E-05
ENSDARG00000021987	plecb	13593.8891	0.405512368	0.1234832	3.28394771	0.00102364	0.01003005
ENSDARG00000103037	CR848788.1	19.4216376	-1.751922914	0.51860305	-3.37815776	0.00072973	0.00776589
ENSDARG00000041400	ndufa3	1298.84027	0.58707998	0.11635369	5.04564968	4.52E-07	1.53E-05
ENSDARG00000070810	ccka	141.260903	-0.636087179	0.21095721	-3.01524266	0.00256774	0.02066746
ENSDARG00000058732	scgn	96.2895145	-0.659641611	0.24351834	-2.70879643	0.00675278	0.0424912
ENSDARG00000089413	mbpb	2770.12464	-0.291181933	0.10841577	-2.68578952	0.00723587	0.04478273
ENSDARG00000095946	si:ch73-341k19.1	151.82679	-0.5074967	0.18006795	-2.81836224	0.00482693	0.03307786
ENSDARG00000058593	sri	2559.14677	-0.559032862	0.08843934	-6.32108791	2.60E-10	1.92E-08
ENSDARG00000044605	stx12l	1063.16677	-0.362506518	0.09623741	-3.76679423	0.00016536	0.00237432
ENSDARG00000057736	ano10a	1402.49353	0.481711871	0.09488086	5.07701852	3.83E-07	1.34E-05
ENSDARG00000076862	gask1a	403.67096	0.966923904	0.17612861	5.4898742	4.02E-08	1.83E-06
ENSDARG00000035309	entpd3	739.671647	0.525472179	0.18486133	2.84252083	0.00447583	0.03126666
ENSDARG00000035308	grb10b	222.895405	1.234431752	0.23147863	5.33281094	9.67E-08	3.97E-06
ENSDARG00000086425	prpf3	1221.34217	-0.410264214	0.09160303	-4.47871858	7.51E-06	0.00017558
ENSDARG00000102307	taf2	1397.46511	-0.304406957	0.10139144	-3.00229446	0.00267953	0.02131872
ENSDARG00000105239	ecm1b	534.135709	-0.387340112	0.12624	-3.06828358	0.00215292	0.01801713
ENSDARG00000097576	zgc:92912	402.005344	-0.937349749	0.1218399	-7.69329031	1.43E-14	2.19E-12
ENSDARG00000045131	id4	792.442297	-0.396067159	0.14673973	-2.6991133	0.00695245	0.04346156
ENSDARG00000037997	tubb5	19796.8287	0.419390794	0.15577805	2.69223293	0.00709754	0.04411831
ENSDARG00000101892	ino80e	1219.43027	0.817746051	0.11588543	7.05650472	1.71E-12	1.85E-10
ENSDARG00000062831	si:ch73-22o12.1	2125.06831	0.271254126	0.08136544	3.33377571	0.00085676	0.0088031

Table S1. DEGs of WT vs. *terfa+/-*

ENSDARG00000104139	atp1a3b	8036.21504	0.385003926	0.12656656	3.0419088	0.00235083	0.01932206
ENSDARG00000002758	dedd1	2101.44489	0.56775043	0.14293787	3.97200851	7.13E-05	0.00117156
ENSDARG00000057879	p3h3	755.902344	0.564829125	0.11791795	4.79001805	1.67E-06	4.76E-05
ENSDARG00000014496	trpv6	1602.52055	-0.889614245	0.17771283	-5.00590889	5.56E-07	1.82E-05
ENSDARG00000057729	ephb6	645.895285	0.579011243	0.19162102	3.02164782	0.00251403	0.0203141
ENSDARG00000037859	il11a	30.8537631	2.398335573	0.7558909	3.17285945	0.00150946	0.01362787
ENSDARG00000010411	epn1	3576.89161	-0.338988759	0.07341159	-4.6176466	3.88E-06	9.84E-05
ENSDARG00000013371	isoc2	761.222157	0.626594762	0.11686669	5.36161961	8.25E-08	3.45E-06
ENSDARG00000004561	prkcg	78.1910637	-1.040596552	0.3076344	-3.38257537	0.0007181	0.00766678
ENSDARG00000040944	ntd5	2009.62803	0.646385127	0.10882005	5.93994506	2.85E-09	1.70E-07
ENSDARG00000057652	dbpb	816.920757	1.843784343	0.16779004	10.9886399	4.33E-28	2.51E-25
ENSDARG00000062390	cdc42ep5	695.763324	-0.47687612	0.17314976	-2.75412525	0.00588492	0.03841118
ENSDARG00000038052	si:ch211-149k23.9	78.7790901	-0.685990619	0.23991278	-2.85933339	0.00424532	0.03013337
ENSDARG00000095688	zgc:162509	9.18173784	4.180110052	1.2764716	3.27473799	0.0010576	0.01029235
ENSDARG00000005762	col14a1a	1894.88812	0.41251074	0.10821776	3.81185812	0.00013793	0.00205312
ENSDARG00000090447	mtbp	194.7431	-0.708796452	0.17838617	-3.97338233	7.09E-05	0.00116791
ENSDARG00000041729	sntb1	696.160739	0.431801138	0.1367137	3.15843355	0.00158619	0.01416934
ENSDARG00000103486	CT573799.1	18.0365914	-7.392522909	1.33616827	-5.53262871	3.15E-08	1.47E-06
ENSDARG00000105408	BX248082.2	141.393438	0.745218356	0.24335752	3.06223683	0.0021969	0.01829887
ENSDARG00000079717	rbm12b	1157.91616	0.299244734	0.11275016	2.6540516	0.00795317	0.0479796
ENSDARG00000098641	nbeal2	465.86763	-0.503046672	0.13196781	-3.81188915	0.00013791	0.00205312
ENSDARG00000053431	si:ch211-257p13.3	865.532583	-0.323872615	0.09801804	-3.30421422	0.00095243	0.00950709
ENSDARG00000104231	opn9	237.481585	-1.080969784	0.27182556	-3.97670395	6.99E-05	0.00115632
ENSDARG00000043304	nop2	1207.77523	-0.33950077	0.09954855	-3.41040384	0.00064867	0.0070898
ENSDARG00000056914	emg1	425.976461	-0.37296881	0.13568274	-2.74883014	0.00598084	0.03882312
ENSDARG00000079530	si:dkey-17m8.1	727.985412	0.534924263	0.17029781	3.14111073	0.00168308	0.01486604
ENSDARG00000035797	si:dkey-17m8.2	78.1233384	7.027134227	0.88391086	7.9500485	1.86E-15	3.29E-13
ENSDARG00000091144	CR749162.1	11.7208181	5.157876086	1.21895219	4.23140148	2.32E-05	0.00045703
ENSDARG00000056750	si:ch211-254p10.2	49.4188718	-1.798274024	0.35058933	-5.12928912	2.91E-07	1.05E-05
ENSDARG00000021787	abcb5	4804.43562	0.385477374	0.10933992	3.52549522	0.00042269	0.00505826
ENSDARG00000117336	CU855948.1	14.8667258	-1.873684358	0.58114251	-3.22413923	0.00126352	0.01185086
ENSDARG00000040698	plekha8	674.556285	0.29733415	0.10803365	2.75223646	0.00591898	0.03857267
ENSDARG00000095615	si:dkeyp-86h10.3	1204.55952	0.37166314	0.13677242	2.71738358	0.00658003	0.04161492
ENSDARG00000056480	cpvl	1383.21715	0.424589804	0.12897609	3.29200392	0.00099476	0.00983094
ENSDARG00000056339	stk31	32.6720527	-1.803131428	0.36424116	-4.95037802	7.41E-07	2.34E-05
ENSDARG00000099846	si:ch211-154o6.3	492.196892	0.955466608	0.13170514	7.25458861	4.03E-13	4.88E-11
ENSDARG00000022788	cops7a	1400.65003	-0.324918206	0.1018828	-3.18913712	0.00142698	0.01304917
ENSDARG00000056379	si:ch73-86n18.1	482.960105	0.453733459	0.15674189	2.89478106	0.00379423	0.02768808
ENSDARG00000099674	dicp3.1	47.8069301	0.77713656	0.28953096	2.68412244	0.00727205	0.04495069
ENSDARG00000055607	cgna	582.227389	-0.41915729	0.12893361	-3.25095439	0.00115018	0.01101225
ENSDARG00000011824	pbxip1b	1565.15458	-0.411756052	0.11151056	-3.69252961	0.00022203	0.00301896
ENSDARG00000095553	lenep	106.709619	-0.681916704	0.21793737	-3.12895715	0.00175428	0.01536926
ENSDARG00000103199	si:dkey-247k7.2	810.28445	0.698106505	0.14953376	4.66855436	3.03E-06	7.97E-05
ENSDARG00000025254	s100a10b	3710.43027	0.612328141	0.11393513	5.37435769	7.69E-08	3.26E-06
ENSDARG00000101181	s100w	434.29343	0.437453412	0.14512803	3.01425857	0.00257608	0.0207078
ENSDARG00000009978	icn	13809.6325	1.077117247	0.11965746	9.00167209	2.22E-19	6.50E-17
ENSDARG00000055514	icn2	4106.02086	1.090205977	0.1002502	10.8748514	1.52E-27	8.58E-25
ENSDARG00000055475	rps27.2	6625.71598	0.453451206	0.10005312	4.53210465	5.84E-06	0.00014079
ENSDARG00000040300	gtpbp10	530.195732	-0.457355301	0.12912854	-3.54186065	0.00039732	0.00481251
ENSDARG00000040298	apoa4b.1	20718.5088	-0.300791382	0.09930544	-3.0289517	0.00245404	0.01992003
ENSDARG00000094929	apoa4b.2	747.197148	-2.475949179	0.67174801	-3.68583031	0.00022796	0.003086
ENSDARG00000020866	apoa4b.2	4001.69247	-1.030504231	0.15333554	-6.72058325	1.81E-11	1.65E-09
ENSDARG00000090190	bcam	2066.12534	0.522623454	0.10578958	4.94021687	7.80E-07	2.45E-05
ENSDARG00000097110	si:dkey-56f14.4	726.539655	0.740754071	0.18929309	3.91326528	9.11E-05	0.00144241
ENSDARG00000097057	fxyd6l	3506.70902	-0.370342987	0.13286667	-2.78732798	0.00531447	0.03564249
ENSDARG00000032496	pon1	467.099014	0.605115225	0.14637815	4.1339178	3.57E-05	0.00065708
ENSDARG00000097789	pon3.2	291.016732	-0.567022652	0.21321649	-2.65937517	0.00782857	0.04742364
ENSDARG00000016856	pon2	564.012332	0.429005821	0.14434143	2.97215993	0.00295713	0.02294064

Table S1. DEGs of WT vs. *terfa+/-*

ENSDARG00000089123	negaly6	141.271975	-0.738947438	0.20663096	-3.57617005	0.00034866	0.00433303
ENSDARG00000042989	si:dkeyp-84f3.5	813.395276	-0.429207521	0.11844384	-3.62372181	0.00029039	0.00375924
ENSDARG00000042969	znf1035	3729.28117	-0.300865641	0.10469534	-2.8737252	0.00405662	0.0291324
ENSDARG00000086626	im:7147486	1155.84488	-0.342430632	0.1079575	-3.17190218	0.00151444	0.01363652
ENSDARG00000042993	prss1	4048.97545	-1.651850197	0.46767641	-3.53203657	0.00041237	0.00496104
ENSDARG00000074623	tbc1d31	331.627224	-0.473802018	0.17235774	-2.7489454	0.00597873	0.0388196
ENSDARG00000077987	zfat	539.422782	0.408503876	0.14167865	2.88331279	0.00393516	0.02844864
ENSDARG00000055115	dtnbp1a	969.435469	0.338653966	0.11627167	2.91260949	0.00358423	0.02658488
ENSDARG00000055118	mylipb	637.538535	0.780406758	0.1505481	5.18377007	2.17E-07	8.12E-06
ENSDARG00000087832	bcl3	113.954743	1.089971546	0.32288065	3.37577229	0.00073609	0.00780524
ENSDARG00000086847	irgq1	53.592964	-2.342388471	0.51661175	-4.534137	5.78E-06	0.00014012
ENSDARG00000014975	irge1	29.07989	-1.448684326	0.45572971	-3.17882353	0.00147874	0.01341391
ENSDARG00000095944	si:ch73-103b2.1	13.6834317	3.14319972	0.81147123	3.87345801	0.0001073	0.00165754
ENSDARG00000079274	prss59.1	1396.8448	-2.361157071	0.73803522	-3.19924712	0.00137787	0.01269817
ENSDARG00000073742	prss59.2	5629.24259	-1.680397876	0.52875335	-3.1780373	0.00148276	0.01344052
ENSDARG00000075677	mrpl17	550.761196	-0.405003536	0.14769199	-2.74221735	0.00610259	0.03940763
ENSDARG00000011245	esrp1	1968.82263	0.340045461	0.10326369	3.29298196	0.00099131	0.00980768
ENSDARG00000055730	trim35-29	17.3258676	1.974065009	0.51458148	3.83625353	0.00012493	0.00188755
ENSDARG00000061335	galnt1	1867.55235	0.242854079	0.07637031	3.17995404	0.00147298	0.01337633
ENSDARG00000055854	nr4a3	571.768153	0.595356937	0.11798798	5.04591158	4.51E-07	1.53E-05
ENSDARG00000002213	invs	258.403214	0.768823787	0.20203211	3.80545345	0.00014154	0.00208913
ENSDARG00000076752	tmem67	491.374933	0.355412832	0.12912013	2.75257485	0.00591286	0.03854293
ENSDARG00000040352	crot	1362.94943	-0.276315111	0.09974849	-2.7701182	0.0056036	0.03712083
ENSDARG00000070427	s100v1	1520.30079	0.277026581	0.10405916	2.66220283	0.00776311	0.04715348
ENSDARG00000086126	trim33l	389.625603	1.361273674	0.21900149	6.21581916	5.11E-10	3.54E-08
ENSDARG00000088805	nes	802.891958	0.327936199	0.11426913	2.8698582	0.00410656	0.02938331
ENSDARG00000078527	lingo4b	442.43705	-0.435556374	0.12575481	-3.46353656	0.00053312	0.00606611
ENSDARG00000074656	ctss2.1	250.805408	0.585202067	0.17635103	3.31839334	0.00090537	0.00919238
ENSDARG00000061644	ensab	328.967866	-0.734577361	0.16023297	-4.58443324	4.55E-06	0.00011337
ENSDARG00000020890	tmod4	7313.71451	-0.318132356	0.09826055	-3.23764061	0.00120523	0.01143236
ENSDARG00000003027	scnm1	129.397698	-0.626376678	0.19478685	-3.21570315	0.00130125	0.01212911
ENSDARG00000016573	mroh1	2091.85346	0.368323326	0.08555949	4.30487987	1.67E-05	0.00034606
ENSDARG00000073799	zgc:194210	35.0340033	-1.52465875	0.44742257	-3.40764828	0.00065525	0.00713686
ENSDARG00000054814	ptp4a3b	1970.52252	-0.381842997	0.11034509	-3.4604439	0.00053929	0.00612332
ENSDARG00000102094	NA	5.37250587	-5.646687202	1.4755504	-3.82683452	0.0001298	0.00195174
ENSDARG00000021184	rbfox1l	2526.58408	-0.516406286	0.13289585	-3.88579685	0.00010199	0.0015884
ENSDARG00000041947	styk1b	627.336526	0.447678602	0.12781645	3.50251151	0.00046089	0.00541394
ENSDARG00000095904	prpf31	1525.49184	-0.332376616	0.09638552	-3.44840811	0.0005639	0.00633173
ENSDARG00000074947	calhm5.1	75.3946549	-0.945547716	0.27600797	-3.42579859	0.00061299	0.00678361
ENSDARG00000019962	dop1a	1853.69612	0.335015313	0.1094484	3.06094296	0.00220641	0.01835968
ENSDARG00000053291	pnrc2	6527.53025	-0.483174339	0.10113573	-4.77748386	1.78E-06	5.03E-05
ENSDARG00000069815	dnali1	61.4652896	1.115285945	0.2760905	4.03956653	5.36E-05	0.00092751
ENSDARG00000033655	stmn1b	8074.0499	0.292682869	0.10099308	2.89804886	0.00375492	0.02744958
ENSDARG00000090097	CR626886.1	28.7278332	-1.307859879	0.47663741	-2.74393042	0.00607084	0.03926379
ENSDARG00000098223	AL773558.1	138.706895	3.065083176	0.40232148	7.61849247	2.57E-14	3.80E-12
ENSDARG00000101989	thrap3a	620.350886	-0.432529967	0.14982111	-2.88697615	0.00388964	0.02818569
ENSDARG00000102701	eva1ba	625.144031	-0.346877562	0.11603373	-2.98945452	0.00279476	0.02200574
ENSDARG00000102987	capn7	664.2428	-0.50252848	0.14925338	-3.36694881	0.00076005	0.00799788
ENSDARG00000103001	si:ch73-215d9.1	495.080774	0.749292036	0.16489806	4.54397128	5.52E-06	0.00013452
ENSDARG00000075133	adgrb1a	2474.72067	0.430182321	0.15145772	2.84027993	0.0045074	0.03141159
ENSDARG00000087369	cep162	307.794632	-0.674579899	0.14863588	-4.53847278	5.67E-06	0.00013767
ENSDARG00000040118	zgc:113232	4343.33139	-1.000333538	0.32290243	-3.09794363	0.00194868	0.0166499
ENSDARG00000029615	zgc:77056	575.547558	-0.749519591	0.110495	-6.78328966	1.17E-11	1.09E-09
ENSDARG00000097794	CR626944.2	468.723631	0.549420876	0.17616524	3.11878141	0.00181601	0.01573809
ENSDARG00000090181	BX072576.2	103.524669	0.594485631	0.20578746	2.88883313	0.00386674	0.02805867
ENSDARG00000028017	tp53inp1	979.132041	0.656776908	0.11131904	5.89995116	3.64E-09	2.12E-07
ENSDARG00000102445	si:dkey-22o22.2	1780.69091	0.568144235	0.16039963	3.5420545	0.00039702	0.00481132
ENSDARG00000070092	KCNV1	61.1538533	-0.795586753	0.29267396	-2.718338	0.00656108	0.04153071

Table S1. DEGs of WT vs. *terfa+/-*

ENSDARG00000070108	dek	988.632407	-0.437381338	0.12470042	-3.50745678	0.00045241	0.00535462
ENSDARG00000010032	cmtm7	409.510224	-0.438289004	0.12792149	-3.42623435	0.00061201	0.00677575
ENSDARG00000094400	si:dkey-199f5.6	27.0747881	-2.353263875	0.43946033	-5.3548949	8.56E-08	3.58E-06
ENSDARG00000093087	BX649388.3	8.18417853	5.381259864	1.41614807	3.7999274	0.00014474	0.0021254
ENSDARG00000008165	caspa	324.943984	0.469051102	0.14216669	3.2993038	0.00096925	0.00963628
ENSDARG00000069981	cspg5a	7695.1284	0.466988212	0.15980532	2.92223204	0.00347533	0.02593191
ENSDARG00000017397	smarcc1a	7726.35605	0.36088036	0.1218219	2.96236032	0.0030529	0.02347769
ENSDARG00000070000	txnipb	725.025776	-0.872702122	0.17261802	-5.05568377	4.29E-07	1.47E-05
ENSDARG00000030494	hjv	396.96443	-0.490171358	0.14069923	-3.48382394	0.0004943	0.00571411
ENSDARG00000097761	BX005234.1	38.150779	1.7073861	0.41891453	4.07573853	4.59E-05	0.00081259
ENSDARG00000079946	sqlea	140.336804	2.067618517	0.24223757	8.53549897	1.40E-17	3.40E-15
ENSDARG00000095865	oxr1a	2112.43412	-0.391701196	0.1136884	-3.4453929	0.00057023	0.0063929
ENSDARG00000095821	si:dkey-122a22.2	375.597015	-0.445804931	0.14294951	-3.11861805	0.00181701	0.01574133
ENSDARG00000010738	zgc:101716	42.6166521	1.324073918	0.4341574	3.04975547	0.00229028	0.01893078
ENSDARG00000097599	CR925804.1	4.25668971	5.136983818	1.51597836	3.38856012	0.00070261	0.00754353
ENSDARG00000089586	ncam3	134.172177	-1.159096391	0.22513079	-5.14854678	2.63E-07	9.60E-06
ENSDARG00000097746	si:rp71-77l1.1	677.328769	0.851990738	0.16087192	5.29608103	1.18E-07	4.75E-06
ENSDARG00000099117	si:dkey-33i11.4	1591.62845	-0.491745239	0.16691793	-2.94603004	0.00321881	0.02439133
ENSDARG00000033172	nr2f5	1750.98336	0.30515326	0.11228526	2.7176608	0.00657452	0.04159915
ENSDARG00000075718	rpz5	1997.97212	0.539847179	0.12572304	4.29393981	1.76E-05	0.00036026
ENSDARG00000075622	zgc:174938	550.498669	-0.408423311	0.14309882	-2.85413476	0.00431542	0.0304536
ENSDARG00000074390	tmem176l.4	2920.39054	0.722724301	0.11869982	6.08867202	1.14E-09	7.51E-08
ENSDARG00000014340	rab11al	662.24618	0.704288287	0.13326283	5.28495656	1.26E-07	5.03E-06
ENSDARG00000087087	col28a1b	62.2527814	-1.691387035	0.32138038	-5.26288213	1.42E-07	5.59E-06
ENSDARG00000077905	csmd3a	373.364855	0.907592543	0.2116637	4.28789884	1.80E-05	0.00036898
ENSDARG00000088308	notchl	328.306756	-0.71544323	0.20081388	-3.56271799	0.00036703	0.00450839
ENSDARG00000098654	FQ976914.1	79.4717336	0.982808036	0.31383817	3.13157583	0.00173871	0.01525436
ENSDARG00000003820	nr1d2a	4794.04148	0.715914003	0.16046576	4.46147515	8.14E-06	0.00018767
ENSDARG00000041295	lim2.5	361.012981	-0.856738771	0.22451287	-3.81598952	0.00013564	0.00202601
ENSDARG00000099572	hmgn2	21392.844	-0.579447467	0.13832123	-4.18914341	2.80E-05	0.00053371
ENSDARG00000091801	serpinb14	1567.74835	0.49504528	0.15366861	3.22151202	0.00127516	0.01193753
ENSDARG00000067784	SLC9A1	178.10926	0.522379226	0.16522341	3.16165381	0.00156876	0.01404594
ENSDARG00000018192	ubr5	13090.7193	0.426925613	0.12167887	3.50862561	0.00045043	0.00533622
ENSDARG00000012588	ptdss1a	1232.18991	0.446778218	0.10282178	4.34517098	1.39E-05	0.00029768
ENSDARG00000031809	rbm24b	709.031611	-0.469256805	0.1339153	-3.50413151	0.0004581	0.00540034
ENSDARG00000052747	gpatch3	732.406125	-0.45046598	0.12554129	-3.58818982	0.00033298	0.00417993
ENSDARG00000104874	fzd6	1671.97835	0.42617767	0.10424366	4.08828385	4.35E-05	0.00077374
ENSDARG00000102914	dennd4b	2691.10212	0.335724286	0.12317336	2.72562408	0.006418	0.04089195
ENSDARG00000097162	CU861891.1	23.0232996	-1.470663249	0.50074972	-2.93692276	0.00331487	0.02495185
ENSDARG00000095802	pcare2	752.755733	-0.651385786	0.22100688	-2.94735524	0.00320505	0.02432202
ENSDARG00000020984	slc16a10	3028.21267	0.348168414	0.10219207	3.40700029	0.00065681	0.00715069
ENSDARG00000071048	ccn6	627.462025	0.402760721	0.1363453	2.95397575	0.00313709	0.02394726
ENSDARG00000074540	gpr63	529.302847	0.506263794	0.1535938	3.29612126	0.0009803	0.00972278
ENSDARG00000071017	nt5e	415.151048	-0.405880485	0.13370166	-3.0357176	0.00239964	0.01959243
ENSDARG00000015201	pcmt	1434.24757	0.45837039	0.08562675	5.35312114	8.64E-08	3.61E-06
ENSDARG00000095751	C20H6orf58	220.437693	0.724324074	0.23890786	3.03181346	0.00243089	0.01977737
ENSDARG00000090722	C20H6orf58	2364.47232	0.920300278	0.15292055	6.01815951	1.76E-09	1.11E-07
ENSDARG00000063416	ptprk	2954.41947	0.354929281	0.11679724	3.03884998	0.00237483	0.01944318
ENSDARG00000025983	l3mbtl3	373.800315	1.02668511	0.22841786	4.49476714	6.96E-06	0.00016424
ENSDARG00000094978	si:ch73-18b11.2	79.802331	0.771828506	0.28454598	2.71249132	0.00667795	0.04211635
ENSDARG00000043843	akap7	1614.01174	0.401739811	0.13577854	2.95878725	0.00308852	0.02369297
ENSDARG00000029157	med23	2249.62299	0.49542079	0.16179708	3.0619884	0.00219872	0.01830793
ENSDARG00000040180	slc35a1	1125.44826	0.475267471	0.1258102	3.77765449	0.00015831	0.00228904
ENSDARG00000079571	mfsd4b	238.61577	-0.702982468	0.1529756	-4.59538942	4.32E-06	0.00010855
ENSDARG00000009585	papola	4566.50338	0.22257335	0.07577127	2.9374373	0.00330937	0.02493314
ENSDARG00000037064	galca	811.476536	0.493155929	0.16000578	3.08211312	0.00205537	0.01738807
ENSDARG00000092503	arid1b	2737.96814	0.579352744	0.11775719	4.91989255	8.66E-07	2.69E-05
ENSDARG00000037073	si:dkey-60a16.1	280.028659	-0.442624451	0.14690825	-3.01293114	0.00258738	0.02076204

Table S1. DEGs of WT vs. *terfa+/-*

ENSDARG00000004262	cyp46a1.2	76.4879468	-1.04756249	0.2855989	-3.66795004	0.0002445	0.00327255
ENSDARG00000074287	sptlc2b	1769.2093	0.429867307	0.11792478	3.64526692	0.00026711	0.00352208
ENSDARG00000031911	tns3.2	1455.0958	-0.277671643	0.09783969	-2.83802653	0.00453934	0.03154671
ENSDARG00000039682	si:ch211-121a2.2	148.689168	-4.997285195	0.5942828	-8.4089346	4.14E-17	9.26E-15
ENSDARG00000039677	dsc2l	2183.62737	0.764976255	0.10205195	7.49594917	6.58E-14	8.91E-12
ENSDARG00000076945	dsg2.1	2977.15626	0.76386847	0.1140379	6.69837381	2.11E-11	1.88E-09
ENSDARG00000043680	ylpm1	2932.78007	0.765621448	0.09636331	7.94515522	1.94E-15	3.39E-13
ENSDARG00000012390	kcnk5b	214.111599	0.562721067	0.18311691	3.07301536	0.00211908	0.01778178
ENSDARG00000001891	syt14b	215.395403	-0.650018616	0.20463	-3.17655579	0.00149035	0.01348478
ENSDARG00000037042	ddhd1b	1319.6773	0.572072419	0.12679213	4.51189233	6.43E-06	0.00015341
ENSDARG00000057911	ACTC1	4445.08591	2.37921311	0.17497293	13.5976071	4.14E-42	8.57E-39
ENSDARG00000090286	serpina1	12604.5036	-0.283465508	0.09861925	-2.87434267	0.00404869	0.0290839
ENSDARG00000079355	flrt2	734.781699	0.373856279	0.11670196	3.20351336	0.00135762	0.01253942
ENSDARG00000096508	BX322618.1	1865.93157	-1.396985934	0.34876344	-4.00554006	6.19E-05	0.00104337
ENSDARG00000013542	lpgat1	1293.92278	0.409376762	0.08760477	4.67299652	2.97E-06	7.83E-05
ENSDARG00000005619	nek2	183.667883	-0.814941034	0.21227353	-3.83910818	0.00012348	0.00187029
ENSDARG00000031600	rd3	404.747327	-0.667132023	0.12014592	-5.55268168	2.81E-08	1.32E-06
ENSDARG00000096655	BX901920.1	157.634868	2.422817605	0.36181039	6.6963738	2.14E-11	1.90E-09
ENSDARG00000016532	suco	3861.54344	0.319070025	0.09188817	3.47237323	0.00051588	0.00590788
ENSDARG00000057529	itpa	323.266064	0.585956684	0.1769919	3.31064118	0.00093082	0.00934396
ENSDARG00000016357	fmo5	1100.19007	-0.276129262	0.09314212	-2.96460146	0.00303075	0.02337131
ENSDARG00000043511	prdx6	1928.619	-0.269916505	0.09035033	-2.98744354	0.00281321	0.02209238
ENSDARG00000008573	fam20b	1531.21127	0.332411162	0.10760034	3.08931317	0.0020062	0.01707089
ENSDARG00000043555	tmem30ab	1483.51385	-0.292926622	0.08592526	-3.40908636	0.00065181	0.00711181
ENSDARG00000043557	kcnk13b	13.3414818	1.81535294	0.66390712	2.73434774	0.0062504	0.04008093
ENSDARG00000034187	calm1b	4265.8245	-0.344780101	0.1085563	-3.1760488	0.00149296	0.01349854
ENSDARG00000062750	si:ch73-74h11.1	2328.75344	0.438157808	0.10884044	4.02568939	5.68E-05	0.00097311
ENSDARG00000018693	cdh2	12071.3969	0.313296499	0.09961574	3.14505025	0.00166058	0.01470388
ENSDARG00000092257	BX510992.1	71.7738982	-0.890005732	0.27028991	-3.29278193	0.00099201	0.00980768
ENSDARG00000015860	mtmr9	988.276731	0.296374925	0.10615339	2.79194983	0.00523915	0.03524195
ENSDARG00000002745	tdh	6350.43436	0.634802865	0.11822754	5.36933144	7.90E-08	3.34E-06
ENSDARG00000015474	ppp2r5ea	1892.93092	0.293461511	0.08036861	3.65144452	0.00026077	0.00346045
ENSDARG00000034293	hif1ab	6558.40117	0.367982183	0.07178596	5.12610266	2.96E-07	1.06E-05
ENSDARG00000043243	prkchb	282.690931	0.795915545	0.15056323	5.28625433	1.25E-07	5.00E-06
ENSDARG00000092770	si:ch211-253p18.2	23.1830843	3.827155057	0.62217526	6.15124914	7.69E-10	5.19E-08
ENSDARG00000021389	jag2b	1894.78129	0.337451536	0.10291698	3.27887141	0.00104223	0.01016666
ENSDARG00000096539	BX088688.2	3.8286557	4.468292533	1.56265587	2.85942197	0.00424414	0.03013337
ENSDARG00000070494	pdgfra	4295.55327	-0.2564812	0.08195759	-3.12943789	0.00175141	0.01534955
ENSDARG00000043323	lnx1	2468.97266	1.060752527	0.12726801	8.33479333	7.76E-17	1.65E-14
ENSDARG00000040001	fryl	4634.66191	0.272346214	0.10270826	2.65164862	0.00800999	0.04820538
ENSDARG00000020469	map3k7	1896.59463	-0.273467727	0.08677697	-3.15138615	0.00162498	0.01446589
ENSDARG00000004635	epha7	1323.09324	0.353596666	0.09402298	3.76074712	0.00016941	0.0024185
ENSDARG00000042956	cyp2ad6	143.065255	-0.760482398	0.1977996	-3.84471145	0.00012069	0.00183253
ENSDARG00000018485	cyp2v1	699.953742	0.440117895	0.13271099	3.31636364	0.00091197	0.00922682
ENSDARG00000022650	cyp2ad3	1359.10409	-0.605961051	0.14611632	-4.14711416	3.37E-05	0.00062451
ENSDARG00000022631	cyp2p9	105.23592	-6.044294831	1.0866735	-5.56219952	2.66E-08	1.25E-06
ENSDARG00000042990	cyp2p10	106.595439	-1.079644454	0.33790609	-3.19510205	0.00139781	0.01285337
ENSDARG00000004517	ppat	1177.42497	0.291600588	0.10465528	2.78629603	0.00533142	0.03570495
ENSDARG00000043004	si:dkeyp-117h8.4	148.695613	-0.621314391	0.18612252	-3.33820102	0.00084323	0.00867843
ENSDARG00000002991	cep135	726.690305	0.4828443	0.14272308	3.383085	0.00071676	0.00765915
ENSDARG00000010831	churc1	666.22173	0.427859765	0.12911538	3.31377852	0.00092044	0.00928475
ENSDARG00000043035	capn3b	1703.18602	0.332241256	0.09838499	3.37695078	0.00073294	0.00778183
ENSDARG00000042221	mthfd1l	6716.42904	0.32977521	0.08339466	3.95439227	7.67E-05	0.00124893
ENSDARG00000029290	stx11b.1	862.661305	0.377906505	0.12274818	3.0787137	0.00207896	0.0175309
ENSDARG00000070389	foxf2b	228.216015	0.518552708	0.16881042	3.07180514	0.00212769	0.017848
ENSDARG00000026629	gmds	1349.43246	0.353375961	0.11920971	2.96432187	0.00303351	0.02337193
ENSDARG00000070396	serpinb1l2	47.6526522	2.02880791	0.3696186	5.4889227	4.04E-08	1.83E-06
ENSDARG00000091633	si:dkey-177p2.18	92.9076416	-1.030780176	0.2415978	-4.26651308	1.99E-05	0.00040024

Table S1. DEGs of WT vs. *terfa+/-*

ENSDARG00000077377	si:dkey-85n7.6	427.218673	-0.451951882	0.13088301	-3.45309816	0.00055419	0.00625253
ENSDARG00000021345	prph2lb	185.731054	-0.886818391	0.23485351	-3.77604911	0.00015934	0.00229982
ENSDARG00000096703	si:dkey-1h6.8	57.0449613	-0.87754092	0.27787303	-3.15806434	0.00158821	0.01417918
ENSDARG00000027734	srsf5b	7090.8159	-0.3482178	0.09270807	-3.75606796	0.0001726	0.00245147
ENSDARG00000030116	susd6	2174.8023	0.330202185	0.07436102	4.44052767	8.97E-06	0.00020388
ENSDARG00000010785	thbs1b	14007.9383	0.283531245	0.09375393	3.02420649	0.00249286	0.02018244
ENSDARG00000012144	emc7	1737.99555	-0.39381394	0.10214596	-3.85540388	0.00011554	0.00176395
ENSDARG00000029546	grem1a	6.35719905	6.071162995	1.52830881	3.97247137	7.11E-05	0.00117083
ENSDARG00000042535	actc1a	15655.9954	0.733356533	0.11604328	6.31968097	2.62E-10	1.93E-08
ENSDARG00000020711	rrm2	831.137572	6.919188641	0.56099252	12.3338339	5.95E-35	6.17E-32
ENSDARG00000042551	mboat2b	526.846472	0.352541697	0.1281728	2.75051888	0.0059501	0.03869431
ENSDARG00000002847	fndc1	241.855513	-0.717467345	0.23607539	-3.03914496	0.00237251	0.01944318
ENSDARG00000030832	otofa	986.307497	-0.426639759	0.13322367	-3.20243201	0.00136272	0.01257725
ENSDARG00000009001	pdia6	3771.26024	0.648832023	0.11461959	5.66074292	1.51E-08	7.66E-07
ENSDARG00000002656	stxbp5a	1178.01189	0.330301434	0.11799163	2.79936317	0.00512035	0.03461146
ENSDARG00000008678	snx3	1492.64846	0.316600077	0.11206067	2.82525587	0.00472429	0.03253593
ENSDARG00000008593	nbas	1053.66699	0.395548795	0.12523822	3.15837134	0.00158653	0.01416934
ENSDARG00000101924	si:dkey-65b13.13	111.3478	-0.817681859	0.2241908	-3.64725873	0.00026505	0.00350234
ENSDARG00000042130	zp3a.2	37.1860914	1.221542287	0.45657057	2.67547313	0.00746239	0.04574047
ENSDARG00000042033	mettl11b	258.999168	-0.582052072	0.18274067	-3.18512611	0.00144691	0.01317806
ENSDARG00000096810	BX279523.1	86.5889417	1.827081376	0.31869861	5.7329442	9.87E-09	5.23E-07
ENSDARG00000024195	znf395b	1265.58335	0.573481116	0.13237667	4.33219163	1.48E-05	0.00031202
ENSDARG00000030106	stmn4	1804.41886	0.472168844	0.14763979	3.1981137	0.0013833	0.01273402
ENSDARG00000019135	ankef1a	66.3014441	0.784371261	0.25477137	3.07872608	0.00207888	0.0175309
ENSDARG00000033949	fbxo16	94.5835685	-0.625121646	0.22553195	-2.77176536	0.00557532	0.03697634
ENSDARG00000021664	fzd3a	1600.13777	0.387824773	0.13084185	2.9640729	0.00303596	0.02338065
ENSDARG00000006672	fam49a	947.028851	-0.299395691	0.09629487	-3.10915501	0.00187623	0.01614742
ENSDARG00000023228	vsnl1a	2535.21469	0.519145135	0.1193372	4.35023727	1.36E-05	0.00029214
ENSDARG00000009753	sf3b6	1005.53014	-0.752363461	0.13743551	-5.47430194	4.39E-08	1.96E-06
ENSDARG00000003584	pla2g7	842.278316	-0.633675123	0.13153001	-4.81772281	1.45E-06	4.24E-05
ENSDARG00000086445	adgrf7	11.8733423	3.699851775	1.04392092	3.54418779	0.00039382	0.00477488
ENSDARG00000015224	cd2ap	1971.76506	0.307734832	0.09657532	3.18647476	0.00144018	0.01313121
ENSDARG00000073747	si:dkey-1j5.4	320.321723	-0.893577685	0.22925248	-3.8977886	9.71E-05	0.00152227
ENSDARG00000020594	txlnba	1898.69344	-0.435568373	0.13560235	-3.21210048	0.00131768	0.01224337
ENSDARG00000054150	cx23	215.175673	-1.235771368	0.27703154	-4.46076054	8.17E-06	0.00018779
ENSDARG00000007171	aig1	300.663476	0.605005492	0.13943579	4.33895423	1.43E-05	0.00030439
ENSDARG00000054076	tatdn3	50.5861413	0.986703816	0.32816428	3.00673742	0.00264068	0.02108386
ENSDARG00000007869	ehd3	843.761647	-0.459261306	0.15462912	-2.97008282	0.00297719	0.0230701
ENSDARG00000033916	si:ch211-243j20.2	857.681977	0.322068779	0.11929118	2.69985414	0.00693699	0.04340987
ENSDARG00000041870	ift172	1132.22673	0.368470745	0.09056544	4.06855819	4.73E-05	0.00083388
ENSDARG00000029587	msra	290.693209	-0.65099992	0.15807041	-4.11841733	3.81E-05	0.00069311
ENSDARG00000010434	clu	2223.69319	0.687455094	0.18273951	3.76194011	0.0001686	0.00241114
ENSDARG00000013441	hey2	200.996781	-0.473405075	0.16792176	-2.81920031	0.00481435	0.03300071
ENSDARG00000034650	fabp7b	159.287366	-0.820439658	0.22026099	-3.72485236	0.00019543	0.00272657
ENSDARG00000053097	hsf2	1017.05012	0.487357045	0.09277202	5.25327632	1.49E-07	5.82E-06
ENSDARG00000076789	cx32.2	74.6954442	0.979749445	0.2622253	3.73628876	0.00018676	0.00262102
ENSDARG00000013528	mcm9	260.901214	-0.567883666	0.16284081	-3.48735465	0.00048782	0.00565789
ENSDARG00000006094	igf2r	1694.93095	0.582938678	0.15677903	3.71821842	0.00020063	0.00277967
ENSDARG00000023111	plg	4443.63401	0.490392442	0.11022888	4.44885645	8.63E-06	0.00019685
ENSDARG00000069296	moxd1l	192.830277	-0.535392523	0.20164887	-2.65507322	0.00792912	0.04789265
ENSDARG00000069245	matn3a	3461.76884	-0.813867558	0.1700009	-4.7874308	1.69E-06	4.81E-05
ENSDARG00000030263	mfsd2b	170.260641	-0.49285454	0.15805292	-3.1182882	0.00181905	0.01574798
ENSDARG00000016011	gpcpd1	2177.73867	-0.361628075	0.10372497	-3.48641282	0.00048954	0.0056752
ENSDARG00000052652	fermt1	1042.27393	0.339620875	0.11360171	2.98957549	0.00279365	0.02200574
ENSDARG00000041433	si:dkey-7c18.24	64.9154066	1.687829026	0.44067365	3.83011106	0.00012809	0.00193013
ENSDARG00000041414	bmf2	148.606269	-0.994667524	0.18903615	-5.26178459	1.43E-07	5.61E-06
ENSDARG00000068428	si:ch211-153j24.3	156.274012	0.71353587	0.21347188	3.34252858	0.00083019	0.00856907
ENSDARG00000031683	fosab	594.732259	-1.140504403	0.21584593	-5.28388179	1.26E-07	5.05E-06

Table S1. DEGs of WT vs. *terfa+/-*

ENSDARG00000020133	jdp2b	257.738335	-1.344967595	0.20971576	-6.41328803	1.42E-10	1.11E-08
ENSDARG00000030092	tram2	658.231304	0.453503214	0.11736948	3.86389374	0.00011159	0.00171425
ENSDARG00000099804	CABZ01059119.1	513.444419	-0.338657391	0.12257445	-2.76287099	0.00572954	0.03769595
ENSDARG00000102744	mgst3a	1402.30875	0.659082769	0.12932382	5.09637572	3.46E-07	1.22E-05
ENSDARG00000073814	pax1b	153.810438	-0.630485798	0.20633921	-3.05557918	0.00224626	0.01861649
ENSDARG00000101900	xrn2	3115.23563	-0.33782293	0.08874192	-3.8068021	0.00014078	0.00208573
ENSDARG00000019601	col12a1b	15448.1177	0.733903001	0.14767593	4.96968613	6.71E-07	2.15E-05
ENSDARG00000067913	ints9	587.716878	0.393232236	0.14445523	2.72217373	0.0064854	0.04123682
ENSDARG00000100851	gphnb	1737.94127	0.547677491	0.15173093	3.60953102	0.00030675	0.003926
ENSDARG00000007886	slc35b2	829.918891	0.368676428	0.10012604	3.68212348	0.0002313	0.00312273
ENSDARG00000068367	nfkbie	553.297813	0.368230448	0.13793971	2.66950284	0.00759636	0.04636729
ENSDARG00000077387	tcte1	30.1648526	1.758199066	0.47049973	3.73687582	0.00018632	0.00261639
ENSDARG00000091595	LO018154.1	309.777387	-0.501412148	0.13290502	-3.77271028	0.00016148	0.00232408
ENSDARG00000013659	brox	1014.38392	-0.378484428	0.10691538	-3.54003727	0.00040007	0.00483881
ENSDARG00000014532	aida	570.780553	-0.611564646	0.10886228	-5.61778291	1.93E-08	9.43E-07
ENSDARG00000008184	mia3	2529.3292	0.227044254	0.08262223	2.74798023	0.00599636	0.03888327
ENSDARG00000027930	naprt	1364.12163	0.386856736	0.09444988	4.09589424	4.21E-05	0.00075144
ENSDARG00000100007	arhgap39	823.5595	0.534322503	0.13445959	3.97385198	7.07E-05	0.00116638
ENSDARG00000039652	si:dkey-235d18.5	910.091138	0.589774535	0.11819866	4.98968894	6.05E-07	1.96E-05
ENSDARG00000107912	FIG4	516.961672	0.452963239	0.15824973	2.86233181	0.00420536	0.02994108
ENSDARG00000052405	pak6b	404.445885	0.807559596	0.17565634	4.59738373	4.28E-06	0.00010763
ENSDARG00000095767	dio3b	978.528502	0.532607473	0.18750379	2.84051577	0.00450406	0.03140631
ENSDARG00000024746	hsp90aa1.2	4484.31435	1.034636802	0.09236687	11.2013843	4.01E-29	2.70E-26
ENSDARG00000010478	hsp90aa1.1	4632.30807	1.20736446	0.11431113	10.5620904	4.47E-26	2.22E-23
ENSDARG00000041239	wdr20b	321.59739	-0.687458045	0.16263205	-4.227076	2.37E-05	0.00046443
ENSDARG00000052408	mgat2	609.728245	-0.440654076	0.11169881	-3.94502047	7.98E-05	0.00129025
ENSDARG00000041232	rps29	15867.5318	1.048801448	0.11814874	8.87695852	6.87E-19	1.92E-16
ENSDARG00000078095	cipcb	1839.13789	0.727620098	0.10177886	7.14902945	8.74E-13	1.01E-10
ENSDARG00000091326	cep120	953.248083	-0.450005702	0.1165838	-3.85993326	0.00011342	0.00173584
ENSDARG00000113045	CABZ01063543.1	79.4026179	-0.900078564	0.24127773	-3.73046689	0.00019113	0.0026778
ENSDARG00000058553	sprb	276.915176	2.035735229	0.2547726	7.99040104	1.35E-15	2.41E-13
ENSDARG00000058454	dynll1	6061.252	0.578673692	0.09960096	5.8099208	6.25E-09	3.48E-07
ENSDARG00000010572	slc25a25a	1219.35473	0.624387316	0.15334922	4.07166937	4.67E-05	0.0008234
ENSDARG00000058419	gcn1	8908.29815	0.324651347	0.0928231	3.4975277	0.00046959	0.00549257
ENSDARG00000098201	mtmr3	458.307518	0.386445252	0.13525747	2.85710832	0.0042752	0.0302649
ENSDARG00000059028	srrd	87.3720807	0.874843922	0.28420595	3.07820406	0.00208252	0.01754019
ENSDARG00000079745	si:ch211-166a6.5	6924.28038	0.834477588	0.08595382	9.70844066	2.78E-22	1.03E-19
ENSDARG00000010051	ufd1l	1457.10632	-0.284678142	0.09048474	-3.14614519	0.00165438	0.01467508
ENSDARG00000030981	tmem127	761.921065	-0.296909771	0.11078675	-2.68001161	0.00736196	0.04533903
ENSDARG00000038974	FO904844.1	2149.97075	0.622178212	0.17459386	3.56357446	0.00036584	0.00450145
ENSDARG00000001968	dock5	569.499623	0.483490931	0.16047031	3.012962	0.00258711	0.02076204
ENSDARG00000012426	neflb	1470.6846	0.314104507	0.1067255	2.94310648	0.00324937	0.02458082
ENSDARG00000069627	si:ch211-255g12.8	21.0989073	1.788208121	0.62540188	2.85929446	0.00424584	0.03013337
ENSDARG00000094310	si:ch211-255g12.6	3461.29276	-1.685805724	0.12849343	-13.1197812	2.54E-39	4.20E-36
ENSDARG00000043701	gpd1a	821.804393	0.519119001	0.160617	3.23203025	0.00122914	0.01160725
ENSDARG00000034836	si:dkey-222n6.2	246.416354	0.810694162	0.17203999	4.71224245	2.45E-06	6.67E-05
ENSDARG00000063292	ssuh2rs1	5027.08272	-0.23552307	0.07438274	-3.16636724	0.00154356	0.01386524
ENSDARG00000012519	hcfc1b	6514.70104	0.425431066	0.13530829	3.14416102	0.00166564	0.01472765
ENSDARG00000100402	fgd1	953.182178	0.498580208	0.15287659	3.26132473	0.00110893	0.01069556
ENSDARG00000007172	plxna3	3599.99998	0.522087745	0.151634	3.44307831	0.00057513	0.00642827
ENSDARG00000058944	si:dkey-8e10.3	143.422319	-0.951879179	0.27857108	-3.41700649	0.00063314	0.00695081
ENSDARG00000006508	pip5k1bb	455.193454	0.398959608	0.11652334	3.42386009	0.00061738	0.00682307
ENSDARG00000058940	si:ch211-248e11.2	111.502136	1.128273767	0.26896889	4.19481139	2.73E-05	0.00052376
ENSDARG00000029439	atp2a2a	2095.80296	0.463924293	0.1130587	4.10339324	4.07E-05	0.00073275
ENSDARG00000043593	rapgef1a	472.031052	0.454074751	0.15984986	2.84063276	0.00450241	0.03140631
ENSDARG00000077367	ntng2a	574.873898	-0.413761775	0.13153371	-3.14567099	0.00165706	0.0146898
ENSDARG00000058865	endog	575.055884	0.410167228	0.15107671	2.71496005	0.00662838	0.04186742
ENSDARG00000093042	NA	4.74257565	-6.514444983	1.57828112	-4.1275568	3.67E-05	0.0006701

Table S1. DEGs of WT vs. *terfa+/-*

ENSDARG00000043571	KIF2A	1598.75276	-0.345805855	0.09734304	-3.55244573	0.00038167	0.0046616
ENSDARG00000043569	dram2a	29.5288508	2.432927984	0.45754072	5.31740204	1.05E-07	4.27E-06
ENSDARG00000028507	itgb4	4382.96869	1.014762779	0.14364178	7.06453787	1.61E-12	1.76E-10
ENSDARG00000023546	kcnn1b	197.932373	-0.717848287	0.20860599	-3.44116813	0.00057921	0.00644992
ENSDARG00000005392	slc5a5	25.0410351	1.366116542	0.47880727	2.85316581	0.0043286	0.0305126
ENSDARG00000058100	b3gnt3.4	94.1433754	-0.614249886	0.23070703	-2.66246718	0.00775701	0.04715103
ENSDARG00000110845	pimr107	7.12574674	6.278661943	1.40798954	4.45931008	8.22E-06	0.0001887
ENSDARG00000078567	lonrf1l	1343.90767	0.501731671	0.10358311	4.84375964	1.27E-06	3.81E-05
ENSDARG00000008866	cabp4	29.270414	-1.272092062	0.41923216	-3.03433798	0.00241064	0.01965765
ENSDARG00000094088	si:ch211-229n2.7	164.699882	0.725763447	0.19918419	3.64367996	0.00026877	0.00353825
ENSDARG00000039456	acbd6	894.591309	-0.299005657	0.11187676	-2.67263424	0.00752583	0.04603849
ENSDARG00000039459	qsox1	2401.23949	0.419553211	0.09000815	4.6612801	3.14E-06	8.19E-05
ENSDARG00000068050	agbl4	75.2363077	1.355720011	0.28298768	4.79073857	1.66E-06	4.76E-05
ENSDARG00000045639	elavl4	11285.4298	0.312386359	0.10858429	2.87690211	0.004016	0.02889917
ENSDARG00000076290	calr	4907.22087	0.395065761	0.09322147	4.23792674	2.26E-05	0.00044607
ENSDARG00000092337	CU467861.6	1008.34214	1.102210415	0.19012645	5.79724937	6.74E-09	3.70E-07
ENSDARG00000086699	smim15	893.785376	-0.397454372	0.11061805	-3.59303375	0.00032685	0.00411752
ENSDARG00000069283	ercc8	552.952595	-0.54696943	0.13898968	-3.93532393	8.31E-05	0.00132887
ENSDARG00000069279	elovl7a	850.300785	0.41599856	0.14098712	2.95061392	0.00317143	0.0241427
ENSDARG00000086933	tnni3k	113.311622	-0.697897216	0.21887068	-3.18862819	0.0014295	0.01305775
ENSDARG00000057365	elovl8b	163.589173	1.403884447	0.24788926	5.66335331	1.48E-08	7.59E-07
ENSDARG00000062025	cpox	716.776489	0.372281618	0.12659323	2.94077044	0.00327397	0.02469637
ENSDARG00000043281	stap2b	303.009313	-0.456468259	0.16417321	-2.78040653	0.00542909	0.03618664
ENSDARG00000057231	rorca	989.471216	-1.003758073	0.14564372	-6.89187345	5.51E-12	5.43E-10
ENSDARG00000005989	rgl1	2063.65283	-0.294296911	0.09438102	-3.11817897	0.00181972	0.01574833
ENSDARG00000043093	mpeg1.2	495.653934	-0.780506583	0.1403891	-5.55959546	2.70E-08	1.27E-06
ENSDARG00000098592	zgc:92140	968.612679	-0.265809614	0.09849398	-2.69873965	0.00696026	0.0434885
ENSDARG00000009390	npl	631.792188	0.783912595	0.1564015	5.0121809	5.38E-07	1.76E-05
ENSDARG00000104329	si:ch73-281k2.5	282.03608	1.40761763	0.23268286	6.04951158	1.45E-09	9.31E-08
ENSDARG00000043130	notch2	7029.77833	0.404885818	0.10715988	3.77833397	0.00015788	0.00228413
ENSDARG00000062892	foxe3	43.6447963	-1.353238011	0.31280943	-4.32607811	1.52E-05	0.00031864
ENSDARG00000088816	trabd2b	532.822585	0.401067845	0.11594581	3.45909738	0.00054199	0.00614248
ENSDARG00000013575	rfx2	1096.2336	0.379051878	0.0986997	3.84045641	0.00012281	0.00186345
ENSDARG00000093753	BX004774.2	156.024075	-1.401123269	0.19187436	-7.30229555	2.83E-13	3.46E-11
ENSDARG00000003564	dohh	419.195138	0.466549605	0.12739222	3.66230857	0.00024995	0.0033365
ENSDARG00000069102	zgc:112962	622.341197	-0.451106421	0.12270613	-3.67631525	0.00023663	0.00318218
ENSDARG00000038635	magoh	1307.18982	-0.438629991	0.1178572	-3.72170706	0.00019788	0.00275228
ENSDARG00000069093	col2a1a	192141.374	0.36492238	0.10415883	3.50351842	0.00045915	0.00540169
ENSDARG00000069074	cry3a	11904.8559	0.599021056	0.09161804	6.53824324	6.22E-11	5.14E-09
ENSDARG00000038643	alas2	2050.68265	0.891556462	0.17221169	5.17709617	2.25E-07	8.37E-06
ENSDARG00000056744	ela2	1270.09368	-2.579693071	0.84241433	-3.0622616	0.00219671	0.01829887
ENSDARG00000056723	fhad1	92.1671151	2.00840599	0.31818628	6.31204462	2.75E-10	2.02E-08
ENSDARG00000075500	smim1	54.2053573	0.95283786	0.32986232	2.88859259	0.0038697	0.02806578
ENSDARG00000042456	mad2l2	275.784307	0.407491196	0.15042227	2.70898177	0.006749	0.04247823
ENSDARG00000095193	si:dkey-23c22.7	23.0817808	5.078714431	0.85331089	5.951775	2.65E-09	1.61E-07
ENSDARG00000092381	zgc:153759	9.63518181	3.148758569	0.87623011	3.59352928	0.00032623	0.0041139
ENSDARG00000068969	zgc:153759	41.9471106	2.279656566	0.50368921	4.52591898	6.01E-06	0.00014455
ENSDARG00000068966	si:ch211-261n11.7	202.56121	5.184502031	0.40852536	12.6907715	6.65E-37	9.19E-34
ENSDARG00000089901	si:ch211-261n11.8	71.6905762	9.544460627	1.20365951	7.92953536	2.20E-15	3.80E-13
ENSDARG00000093392	CT583651.1	30.3945143	8.442634709	1.26236094	6.68797207	2.26E-11	1.99E-09
ENSDARG00000074758	csde1	19992.6862	0.331441274	0.09081856	3.6494883	0.00026276	0.00348133
ENSDARG00000078052	emilin3a	1150.11429	0.462980441	0.14733667	3.14232999	0.00167609	0.01480953
ENSDARG00000079978	samd10a	270.842971	0.414288336	0.14733873	2.81180871	0.00492638	0.03359263
ENSDARG00000001686	uckl1a	129.268852	-0.701273728	0.22074982	-3.17678056	0.0014892	0.01347924
ENSDARG00000016447	ythdf1	2870.05336	0.31273796	0.07318871	4.27303552	1.93E-05	0.00038966
ENSDARG00000022768	gid8a	489.906586	-0.367125849	0.13013296	-2.8211595	0.00478504	0.03285419
ENSDARG00000027154	tpd52l2a	1442.35988	0.299520232	0.09754329	3.0706389	0.00213601	0.01790517
ENSDARG00000094197	znfl1l	104.663728	4.236096794	0.45454923	9.31933561	1.17E-20	3.78E-18

Table S1. DEGs of WT vs. *terfa+/-*

ENSDARG00000042927	mapre1a	441.740129	-0.390025205	0.13445348	-2.9008189	0.00372189	0.02729642
ENSDARG00000026799	suv39h1a	22.0037475	-1.262514033	0.4201708	-3.00476383	0.00265787	0.02119965
ENSDARG00000094451	cfp	640.446956	-1.379922588	0.18246986	-7.56246881	3.95E-14	5.59E-12
ENSDARG00000078114	si:ch73-237c6.1	938.370553	2.977967215	0.18942331	15.7212289	1.08E-55	1.34E-51
ENSDARG00000068870	fance	189.088381	-0.496336307	0.16949698	-2.92829005	0.00340832	0.02551919
ENSDARG00000078917	zgc:195245	65.6623478	-1.083491039	0.38015919	-2.85009823	0.00437057	0.03076482
ENSDARG00000090194	lamtor5	429.008629	0.656691705	0.11989876	5.47705161	4.32E-08	1.94E-06
ENSDARG00000001621	nfyal	712.7334	0.453768074	0.1303704	3.48060665	0.00050028	0.00577005
ENSDARG00000023861	dnase1l1l	219.579505	-0.871275041	0.29173049	-2.98657518	0.00282121	0.02214123
ENSDARG00000042545	sema3ga	714.827177	0.385172581	0.14605705	2.63713788	0.00836088	0.04977629
ENSDARG00000005015	tspy	1511.48651	-0.274264947	0.09698955	-2.8277784	0.00468722	0.03234341
ENSDARG00000039754	xpc	713.382744	0.611921111	0.12613635	4.85126712	1.23E-06	3.69E-05
ENSDARG00000012848	arih2	2781.03501	-0.289412064	0.07430378	-3.89498446	9.82E-05	0.00153418
ENSDARG00000055825	celsr3	2972.89007	0.550938225	0.19009784	2.89818247	0.00375332	0.02744596
ENSDARG00000097292	slc26a6	82.849984	0.949944294	0.31907976	2.97713746	0.00290954	0.02266264
ENSDARG00000011672	sema3b	1903.38938	0.241877054	0.07541851	3.20713129	0.00134066	0.01241045
ENSDARG00000090721	prdm2a	684.260346	-0.368904714	0.12967477	-2.84484577	0.00444329	0.03112685
ENSDARG00000056090	capza1b	10128.2065	0.212215588	0.07854822	2.70172356	0.00689811	0.04323058
ENSDARG00000003098	kdm5bb	9853.91986	0.310742907	0.10883766	2.85510461	0.00430227	0.0304132
ENSDARG00000112728	KLHL12	350.849401	0.497185494	0.14126756	3.51945986	0.00043243	0.00515737
ENSDARG00000042717	adipor1b	1051.23513	0.367645439	0.09834983	3.73814009	0.00018539	0.00260769
ENSDARG00000101609	si:dkey-109a10.2	4.95113401	4.555651578	1.50298909	3.03106098	0.00243696	0.01980717
ENSDARG00000012403	ercc6l2	326.176956	0.468410858	0.15698054	2.98387854	0.0028462	0.02228102
ENSDARG00000016404	ptch1	3720.17549	0.312534847	0.10387106	3.00887304	0.00262219	0.02099016
ENSDARG00000068438	gucd1	908.941153	-0.260631626	0.08987619	-2.89989629	0.00373286	0.0273285
ENSDARG00000042440	p2rx7	46.1742571	1.565426139	0.52459166	2.98408506	0.00284428	0.02228102
ENSDARG00000026325	mmp11a	576.940542	-0.332040687	0.11486374	-2.89073555	0.00384341	0.0279307
ENSDARG00000020114	slc20a1a	1348.2909	0.691948863	0.16251151	4.25784523	2.06E-05	0.00041406
ENSDARG00000060368	syn1	4002.76261	0.342080764	0.1277696	2.67732506	0.00742126	0.04560084
ENSDARG00000020926	creb3l3l	3190.52165	0.261879854	0.08055228	3.2510547	0.00114978	0.01101225
ENSDARG00000092042	AL935153.1	343.86961	-0.82856156	0.19011467	-4.35821994	1.31E-05	0.00028365
ENSDARG00000074170	fbxo4	187.789099	0.529014008	0.18979085	2.78735254	0.00531406	0.03564249
ENSDARG00000073720	RNF180	145.953248	-2.351551305	0.27637501	-8.50855269	1.76E-17	4.21E-15
ENSDARG00000031044	lipg	662.610756	0.342326647	0.10911649	3.13725848	0.00170536	0.01500944
ENSDARG00000104992	epgn	299.716214	-0.594703733	0.20720974	-2.87005678	0.00410398	0.02938331
ENSDARG00000089418	si:dkey-164f24.2	1548.7886	-0.416814677	0.15735517	-2.64887811	0.00807595	0.04850837
ENSDARG00000079327	hmcn2	5590.85993	0.536321198	0.103267	5.19353924	2.06E-07	7.75E-06
ENSDARG00000092658	CR457445.1	14.6216047	2.699694946	0.88773925	3.04109	0.00235723	0.01936188
ENSDARG00000068365	lmx1bb	688.747604	0.470633891	0.11127969	4.22928836	2.34E-05	0.00046025
ENSDARG00000102759	TMEM132D	268.771817	-0.483877579	0.17181554	-2.81626195	0.0048586	0.03324906
ENSDARG00000116524	zgc:103700	11.7136902	-2.001484681	0.60616978	-3.30185494	0.00096048	0.00956437
ENSDARG00000079105	mhc2dab	9.28335667	6.197941425	1.35307081	4.58064824	4.64E-06	0.00011533
ENSDARG00000006124	kdm5c	8098.16247	0.274801469	0.09663527	2.84369729	0.00445934	0.03120408
ENSDARG00000094937	CR450833.1	5.8454175	5.14562826	1.4111376	3.64643977	0.0002659	0.00351058
ENSDARG00000053644	got1l1	49.4721264	1.117573812	0.37469998	2.98258304	0.00285827	0.02235046
ENSDARG00000060518	pcsk5b	1566.85419	0.376549317	0.09412536	4.00050874	6.32E-05	0.00106148
ENSDARG00000010710	msi1	2121.01484	-0.469709917	0.1098313	-4.27664902	1.90E-05	0.00038464
ENSDARG00000046142	asgrl1	52.1768901	-1.288071017	0.39116947	-3.29287209	0.0009917	0.00980768
ENSDARG00000090945	si:ch211-170d8.8	12.3428499	-4.823122939	0.99964429	-4.82483918	1.40E-06	4.14E-05
ENSDARG00000009470	hnf1a	215.868918	-0.762757422	0.21114648	-3.61245619	0.00030331	0.00388797
ENSDARG00000009629	unc119.1	783.734455	-0.498725924	0.13092195	-3.80933772	0.00013934	0.00206887
ENSDARG00000002172	aplnra	926.575194	-0.597881254	0.11569401	-5.16778071	2.37E-07	8.75E-06
ENSDARG00000098108	dusp2	543.639032	-0.427261106	0.13027791	-3.27961274	0.0010395	0.01014397
ENSDARG00000068217	stx2b	504.787293	0.35793275	0.13357906	2.67955735	0.00737196	0.04538766
ENSDARG00000010933	cacna1fa	346.798629	-0.677753153	0.23257955	-2.91407029	0.0035675	0.02649241
ENSDARG00000060065	ubap2b	7155.82736	0.206410851	0.07451784	2.76995203	0.00560645	0.03712988
ENSDARG00000010070	adam9	1434.51606	0.293854444	0.09542186	3.0795296	0.00207328	0.0174979
ENSDARG00000077114	arhgef16	789.022201	0.460369083	0.14029015	3.28154968	0.00103238	0.01008643

Table S1. DEGs of WT vs. *terfa+/-*

ENSDARG00000007275	si:ch211-251b21.1	12851.1495	-0.295426188	0.10199384	-2.89651017	0.00377338	0.02756023
ENSDARG00000036684	ndufa7	973.522316	0.399255142	0.10052464	3.97171441	7.14E-05	0.00117223
ENSDARG00000059746	plod1a	2165.88934	0.563284307	0.12975945	4.34098861	1.42E-05	0.00030184
ENSDARG00000079504	mfn2	1942.56584	0.426243755	0.08102682	5.26052689	1.44E-07	5.64E-06
ENSDARG00000092738	si:ch211-263k4.2	68.6767705	1.006182519	0.30301424	3.32057828	0.00089831	0.00914587
ENSDARG00000078324	si:ch211-263k4.2	1036.88673	0.536735763	0.15317981	3.50395889	0.0004584	0.00540034
ENSDARG00000068149	tprg1l	262.217002	-0.520573224	0.1923295	-2.70667383	0.0067961	0.04268816
ENSDARG00000025858	zgc:56525	2870.41071	0.578405012	0.11275862	5.12958588	2.90E-07	1.05E-05
ENSDARG00000015293	fam110a	250.267982	0.526867695	0.16115238	3.2693759	0.00107785	0.01045312
ENSDARG00000018914	hnrnpk	4737.10965	0.308134662	0.0903539	3.41030822	0.0006489	0.0070898
ENSDARG00000059897	ntrk2a	1706.13372	0.354661296	0.12409482	2.8579863	0.00426339	0.03021571
ENSDARG00000105214	agtpbp1	923.674871	0.39188592	0.09988472	3.92338212	8.73E-05	0.00138975
ENSDARG00000057465	gfpt1	2406.0467	0.358982722	0.10957178	3.27623329	0.00105202	0.01024603
ENSDARG00000025672	antxr1a	120.488636	-1.012689261	0.24358505	-4.15743601	3.22E-05	0.0006001
ENSDARG00000095807	hp	859.789555	-0.587335265	0.17723617	-3.31385662	0.00092019	0.00928475
ENSDARG00000038422	entpd4	1069.81281	0.294280484	0.10187073	2.888764	0.00386759	0.02805867
ENSDARG00000033594	ido1	88.3510931	-1.689943477	0.38091787	-4.43650352	9.14E-06	0.00020716
ENSDARG00000074828	rhobtb2a	165.688679	0.744654015	0.26460687	2.81418999	0.00489003	0.03340894
ENSDARG00000106579	CABZ01060030.2	78.6616014	1.10559129	0.33329981	3.3171075	0.00090955	0.00922682
ENSDARG00000095922	CR855300.1	193.388964	0.579841482	0.18673199	3.10520697	0.00190146	0.01631851
ENSDARG00000021287	rab7b	544.781664	0.775096194	0.1425659	5.43675711	5.43E-08	2.35E-06
ENSDARG00000063159	si:dkey-32e23.4	664.253124	0.724889028	0.18117199	4.00110971	6.30E-05	0.00105951
ENSDARG00000112101	CR855300.2	46.2666202	1.203478393	0.35315465	3.40779427	0.0006549	0.00713617
ENSDARG00000028071	bmp1a	4999.46917	0.456516527	0.10226511	4.46404945	8.04E-06	0.00018595
ENSDARG00000041358	lgi3	222.09087	-0.594494588	0.17628493	-3.37235052	0.0007453	0.00788604
ENSDARG00000021209	kctd9a	172.471721	-0.67100582	0.18390416	-3.64867126	0.0002636	0.0034887
ENSDARG00000025013	gna14a	45.674978	-1.102799774	0.39992622	-2.75750804	0.00582438	0.03815628
ENSDARG00000039225	vps13a	4774.96884	0.296246901	0.0875866	3.38233138	0.00071873	0.0076703
ENSDARG00000007369	tcf7l1b	1249.50836	0.402215892	0.08982308	4.47786782	7.54E-06	0.00017612
ENSDARG00000015657	zgc:77112	70.7582392	-0.753725069	0.25492898	-2.95660802	0.00311043	0.02382428
ENSDARG00000102599	si:ch1073-392o20.2	1199.66441	0.621891861	0.15914323	3.9077495	9.32E-05	0.00146736
ENSDARG00000086906	stambpb	598.618605	-0.400481468	0.13683513	-2.92674448	0.0034253	0.02562018
ENSDARG00000099294	si:ch73-199g24.2	1613.8238	0.452904082	0.11525793	3.92948321	8.51E-05	0.00135894
ENSDARG00000076218	b3galt4	93.896159	-0.871920886	0.24168149	-3.60772726	0.00030889	0.00394501
ENSDARG00000078215	rbsn	300.406991	-0.473350382	0.14681086	-3.22421906	0.00126317	0.01185086
ENSDARG00000075917	cacna1db	173.764931	0.57514274	0.19728384	2.91530592	0.0035534	0.02642722
ENSDARG00000103609	mib2	803.643552	0.426678255	0.14134887	3.0186181	0.0025393	0.02047175
ENSDARG00000043079	MMP23B	219.337708	-1.009005993	0.15081806	-6.69021975	2.23E-11	1.97E-09
ENSDARG00000102546	cdk11b	3210.34208	0.371352675	0.10477038	3.54444329	0.00039344	0.00477488
ENSDARG00000006990	creld1b	985.850379	0.458293582	0.14574686	3.1444491	0.001664	0.01472363
ENSDARG00000104864	klhdc2	875.22785	0.458940195	0.13877952	3.30697342	0.0009431	0.00944048
ENSDARG00000102257	soul3	1418.47301	0.391421881	0.09066086	4.31742985	1.58E-05	0.00032915
ENSDARG00000098849	SLC25A29	199.909962	-1.100847615	0.2506899	-4.39127227	1.13E-05	0.00024919
ENSDARG00000100954	wars1	584.987197	0.597268573	0.15073454	3.96238702	7.42E-05	0.00121418
ENSDARG00000113183	CABZ01078858.1	955.636693	0.452240499	0.12705279	3.55946923	0.00037161	0.00455717
ENSDARG00000111014	LO018430.2	244.721279	0.443065225	0.15586348	2.84264933	0.00447403	0.0312658
ENSDARG00000099657	akt1	299.655249	0.494033138	0.16759925	2.947705	0.00320142	0.02430403
ENSDARG00000024160	hao1	1997.81126	0.6079708	0.14431143	4.21290814	2.52E-05	0.00049145
ENSDARG00000089458	rp1l1a	409.689038	-0.881250127	0.23506946	-3.74889244	0.00017762	0.00251405
ENSDARG00000040255	ephx2	1079.90874	-0.370106604	0.13952084	-2.65269768	0.00798514	0.04809075
ENSDARG00000006602	chrna2a	112.423291	-0.695251072	0.22662195	-3.06788936	0.00215576	0.01803484
ENSDARG00000032426	coq8ab	147.4903	0.571079299	0.19756015	2.89066036	0.00384433	0.0279307
ENSDARG00000075619	cenpo	115.801605	-0.929541196	0.21164494	-4.39198396	1.12E-05	0.0002486
ENSDARG00000058782	si:ch73-242m19.1	67.3691487	-0.712082418	0.2684563	-2.65250771	0.00798963	0.04810617
ENSDARG00000079664	zgc:172341	182.537055	-0.754415482	0.20911776	-3.60761072	0.00030903	0.00394501
ENSDARG00000075884	shprh	776.092963	0.360997519	0.13342044	2.70571377	0.00681578	0.04280094
ENSDARG00000087784	si:dkeyp-110a12.4	536.026739	0.36316302	0.13550863	2.67999922	0.00736223	0.04533903
ENSDARG00000086256	si:ch211-236p5.2	187.954125	-0.785732219	0.23342178	-3.36614778	0.00076226	0.00801775

Table S1. DEGs of WT vs. *terfa+/-*

ENSDARG00000086418	si:ch211-236p5.3	165.570882	-1.711941919	0.3138302	-5.45499411	4.90E-08	2.17E-06
ENSDARG00000058692	fzd3b	717.972707	0.367916775	0.12390345	2.96938273	0.00298399	0.02309779
ENSDARG00000063190	zranb1b	1937.34664	-0.273239967	0.09762161	-2.7989701	0.00512659	0.0346442
ENSDARG00000109820	NPAS3	1999.12116	0.383601563	0.12750896	3.00842832	0.00262603	0.02101195
ENSDARG00000058444	snx6	206.205334	-1.246137866	0.22185314	-5.61694933	1.94E-08	9.45E-07
ENSDARG00000063233	baz1a	456.139638	-0.30978309	0.11077087	-2.79661153	0.00516416	0.03485071
ENSDARG00000104230	sec23a	2916.75761	0.514481682	0.08546821	6.0195675	1.75E-09	1.11E-07
ENSDARG00000104125	ttc6	196.485699	1.489776165	0.21906455	6.80062641	1.04E-11	9.81E-10
ENSDARG00000102138	foxa1	519.203423	0.419894586	0.1343353	3.12572043	0.0017737	0.0154794
ENSDARG00000097772	si:ch211-185a18.2	252.445783	0.449946209	0.16297066	2.7609031	0.00576418	0.03784169
ENSDARG00000043873	arid4a	2962.65301	-0.241378951	0.07943143	-3.03883423	0.00237495	0.01944318
ENSDARG00000086618	psma3	3786.66245	-0.361736342	0.10524914	-3.43695309	0.0005883	0.00653649
ENSDARG00000013343	kif26ba	2329.96388	0.367253756	0.13116663	2.79990243	0.00511181	0.03459132
ENSDARG00000075766	sccpdha.1	281.292261	-0.654316068	0.24101964	-2.71478322	0.00663192	0.04187914
ENSDARG00000006235	brms1la	837.50516	-0.544619692	0.13697248	-3.97612488	7.00E-05	0.00115837
ENSDARG00000007693	nfkbiab	2703.43408	0.50359519	0.17361513	2.9006412	0.003724	0.02730384
ENSDARG00000091185	FP017295.1	27.7866592	3.21405135	0.58924885	5.45448892	4.91E-08	2.17E-06
ENSDARG00000087443	ptp4a2a	2079.70898	-0.385297603	0.10342031	-3.7255506	0.00019489	0.00272133
ENSDARG00000075707	nid2a	6458.86648	0.528096579	0.10407622	5.074133	3.89E-07	1.36E-05
ENSDARG00000011434	ptger2a	202.170377	-0.606026234	0.17962202	-3.3738973	0.00074112	0.00785189
ENSDARG00000087197	ros1	102.655933	-0.749802976	0.28279551	-2.65139635	0.00801597	0.04820638
ENSDARG00000002635	FAM184A	1211.44522	-0.391390943	0.13202908	-2.96442977	0.00303244	0.02337131
ENSDARG00000003381	si:ch211-266g18.6	422.570278	-0.699449698	0.17427979	-4.0133723	5.99E-05	0.00101484
ENSDARG00000016513	setd3	1930.99957	0.61770926	0.163231	3.78426436	0.00015416	0.00223817
ENSDARG00000097921	BX465857.1	16.7118953	5.987961278	1.84860577	3.23917699	0.00119875	0.01139823
ENSDARG00000018006	vrk1	459.878726	-0.970236	0.12502497	-7.76033799	8.47E-15	1.34E-12
ENSDARG00000071082	p4ha1b	903.680084	0.679359375	0.20709471	3.28042849	0.0010365	0.01012263
ENSDARG00000074663	zgc:162183	264.137912	0.474011089	0.1515244	3.12828232	0.00175831	0.0153775
ENSDARG00000097416	si:ch73-306e8.2	151.316969	-0.61636498	0.20157714	-3.05771269	0.00223033	0.01851534
ENSDARG00000043395	crip3	211.226586	-0.430813876	0.16233391	-2.65387474	0.00795734	0.04799311
ENSDARG00000117493	BX511218.1	41.7634915	-2.066597522	0.36352888	-5.68482344	1.31E-08	6.79E-07
ENSDARG00000056922	ltbp1	2996.51477	0.311308782	0.10554358	2.94957564	0.00318211	0.02420914
ENSDARG00000088493	si:ch211-149k12.3	5.87202217	-3.292406398	1.24659484	-2.64111986	0.00826325	0.04929982
ENSDARG00000075263	ankrd1a	112.586751	-0.60842576	0.20906875	-2.91017079	0.00361231	0.02670554
ENSDARG00000063229	xpo1b	4843.34048	0.253028149	0.06984235	3.62284699	0.00029138	0.00376414
ENSDARG00000097418	si:ch211-63b16.3	26.1996167	-1.447517386	0.42496082	-3.40623729	0.00065865	0.00715816
ENSDARG00000086305	si:ch211-63b16.4	80.7530729	-1.473572979	0.27992353	-5.26419842	1.41E-07	5.56E-06
ENSDARG00000097964	arhgef33	306.192652	0.462529988	0.14512649	3.18708167	0.00143716	0.01311813
ENSDARG00000044102	znf593	451.260806	-0.360754174	0.12995244	-2.77604776	0.00550241	0.03659686
ENSDARG00000033616	selenon	1092.04156	0.331067185	0.10321264	3.20762239	0.00133837	0.01239836
ENSDARG00000002401	gale	908.808881	0.352074543	0.11416035	3.08403512	0.00204213	0.01729968
ENSDARG00000089221	si:ch211-195o20.7	288.515603	-0.71708052	0.1836002	-3.90566297	9.40E-05	0.00147914
ENSDARG00000077788	arhgef10	846.298517	0.358612565	0.12439138	2.88293749	0.00393986	0.02846661
ENSDARG00000104958	kbtbd11	699.905119	-0.308960359	0.11565285	-2.67144604	0.00755252	0.04616771
ENSDARG00000100426	fam167b	320.710521	-1.168879843	0.16654744	-7.01829983	2.25E-12	2.38E-10
ENSDARG00000001463	tdh2	1190.83497	-1.083855653	0.19049009	-5.68982703	1.27E-08	6.61E-07
ENSDARG00000070874	asmt2	245.983141	0.470751121	0.17518068	2.68723201	0.00720469	0.0446263
ENSDARG00000054823	id3	1515.39518	-0.311487003	0.09792063	-3.18101518	0.0014676	0.01334206
ENSDARG00000055270	si:ch1073-358c10.1	73.0562093	1.373335867	0.34304299	4.00339284	6.24E-05	0.00105146
ENSDARG00000042563	mis18bp1	279.317076	-0.538819419	0.18898997	-2.85104771	0.00435754	0.03068179
ENSDARG00000098051	opn6b	228.362736	-1.649779827	0.26206666	-6.29526778	3.07E-10	2.23E-08
ENSDARG00000090708	si:dkey-28g23.6	25.6213861	-1.903210505	0.65353081	-2.91219706	0.00358896	0.02660091
ENSDARG00000028804	ankrd9	788.256856	1.172764472	0.20833669	5.62917867	1.81E-08	8.91E-07
ENSDARG00000060854	kctd3	754.340739	0.376063389	0.14125994	2.66220828	0.00776298	0.04715348
ENSDARG00000054973	itsn2b	2741.27652	0.209238931	0.07607688	2.75036153	0.00595295	0.03870277
ENSDARG00000054537	nhsl1a	1794.38175	-0.473124929	0.0994615	-4.75686507	1.97E-06	5.50E-05
ENSDARG00000074809	zgc:194392	219.128103	-0.593541703	0.21299182	-2.78668779	0.00532498	0.03568408
ENSDARG00000042780	apoba	29631.018	-0.422930334	0.10054241	-4.20648695	2.59E-05	0.00050361

Table S1. DEGs of WT vs. *terfa+/-*

ENSDARG00000042793	tpp1	1522.0535	0.719195586	0.14347272	5.0127688	5.37E-07	1.76E-05
ENSDARG00000042846	disp2	993.021007	-0.318281644	0.11728846	-2.71366541	0.00665433	0.04199936
ENSDARG00000042861	ltk	666.731429	-0.602296417	0.14254857	-4.22520152	2.39E-05	0.00046795
ENSDARG00000090753	si:dkey-13p1.3	39.7790391	1.085035992	0.38416517	2.82439974	0.00473693	0.0326049
ENSDARG00000075444	cgref1	477.833813	0.485362425	0.17630152	2.75302454	0.00590475	0.03851023
ENSDARG00000055133	cenpf	1873.63967	-0.583880037	0.16403719	-3.55943698	0.00037165	0.00455717
ENSDARG00000091471	ccdc28a	84.5467279	0.776444086	0.25782153	3.01155648	0.00259912	0.02083908
ENSDARG00000012874	snap23.1	1483.05908	0.299942378	0.103375	2.90149824	0.00371383	0.02726143
ENSDARG00000015449	fut8a	2766.54734	0.350031169	0.10174176	3.44038841	0.00058088	0.00646274
ENSDARG00000089076	gphna	2144.92105	0.356553381	0.11928939	2.9889782	0.00279912	0.02200954
ENSDARG00000007169	itgb1bp1	807.097737	0.320067468	0.099566	3.21462626	0.00130614	0.012168
ENSDARG00000011141	dpysl5a	4056.92607	0.441245768	0.15200903	2.90276022	0.0036989	0.02716788
ENSDARG00000015566	dnmt3ab	3902.41395	0.297985658	0.10425721	2.858178	0.00426081	0.03020608
ENSDARG00000060594	hadhab	6159.62497	-0.227599454	0.06827061	-3.3337838	0.00085673	0.0088031
ENSDARG00000070673	ptgr2	448.283528	0.508936955	0.11854077	4.29334946	1.76E-05	0.00036093
ENSDARG00000053864	elmsan1b	2051.39872	0.348150565	0.11834104	2.9419258	0.00326178	0.02463432
ENSDARG00000060564	slc25a21	299.687159	-0.400073466	0.13483728	-2.96708342	0.00300639	0.02321339
ENSDARG00000005179	dglucy	956.614394	-0.346249519	0.13063386	-2.65053424	0.00803646	0.04831005
ENSDARG00000029668	crim1	1433.78549	0.455480676	0.15175485	3.0014241	0.0026872	0.02135242
ENSDARG00000038863	babam2	789.950194	-0.36786807	0.10420928	-3.5300892	0.00041542	0.00499046
ENSDARG00000040623	fosl2	938.363067	0.593458712	0.19500236	3.04334112	0.00233967	0.01923668
ENSDARG00000003411	foxa2	468.655375	0.406318081	0.15009638	2.70704779	0.00678845	0.04267036
ENSDARG00000009443	zgc:92137	193.550224	-2.35970262	0.67532206	-3.49418856	0.00047551	0.0055565
ENSDARG00000090912	npc2	2487.74314	0.65351273	0.11994423	5.44847167	5.08E-08	2.23E-06
ENSDARG00000014854	reep1	205.57277	-0.751668395	0.19793313	-3.79758763	0.00014611	0.00214253
ENSDARG00000053558	rtkn2a	274.922055	-0.818756762	0.17859261	-4.58449406	4.55E-06	0.00011337
ENSDARG00000100317	ahsa1b	1393.62258	0.378489308	0.10407819	3.6365863	0.00027628	0.00362174
ENSDARG00000030357	zgc:66313	74.0303033	1.183601051	0.34370373	3.44366656	0.00057388	0.00642516
ENSDARG00000076362	tmem260	293.813015	0.69934404	0.16045057	4.35862607	1.31E-05	0.00028337
ENSDARG00000060323	exoc5	818.265316	0.543944401	0.12307997	4.41943877	9.90E-06	0.00022239
ENSDARG00000075397	cipca	277.219684	0.539017494	0.17704251	3.04456529	0.00233017	0.01918395
ENSDARG00000074100	zgc:163014	12.7956855	-2.342764182	0.61426888	-3.81390667	0.00013679	0.0020395
ENSDARG00000041864	capn3a	2547.16651	-2.445820691	0.18361909	-13.3200788	1.77E-40	3.14E-37
ENSDARG00000016651	znf106a	5657.1627	0.276319172	0.10317167	2.67824659	0.00740087	0.04550931
ENSDARG00000091723	si:ch211-202f3.4	40.9138611	-1.170443391	0.38486046	-3.04121499	0.00235626	0.01936024
ENSDARG00000102288	CABZ01056321.1	364.573703	-0.859240354	0.16885143	-5.08873593	3.60E-07	1.27E-05
ENSDARG00000001937	LO018422.1	150.801084	-1.480087558	0.36432115	-4.06259026	4.85E-05	0.00085126
ENSDARG00000034222	hs1bp3	133.382357	0.828278983	0.26417678	3.13532093	0.00171666	0.01508756
ENSDARG00000040727	tfb1m	143.382151	-0.619747952	0.17189883	-3.6053064	0.00031178	0.00397406
ENSDARG00000016763	znf292a	1720.83399	-0.324855095	0.11740934	-2.76685897	0.00565992	0.03738437
ENSDARG00000100359	AREL1	1323.28229	-0.369384482	0.09826384	-3.7591089	0.00017052	0.0024302
ENSDARG00000044847	zgc:171599	268.521045	-0.46554312	0.16456684	-2.82889991	0.00467083	0.03225715
ENSDARG00000078322	col12a1a	31189.1846	0.669821245	0.09983221	6.70947057	1.95E-11	1.76E-09
ENSDARG00000078419	filip1a	120.975058	-1.432636138	0.24102833	-5.94384962	2.78E-09	1.66E-07
ENSDARG00000074471	vps39	1488.41697	0.423086258	0.09382178	4.50946737	6.50E-06	0.00015487
ENSDARG00000040135	fosaa	262.327327	0.576876499	0.2144166	2.69044698	0.00713564	0.04432798
ENSDARG00000088862	stxbp6	874.452476	0.287099316	0.10860956	2.64340746	0.00820762	0.04903855
ENSDARG00000020448	adi1	364.080261	0.579294279	0.17578972	3.29538196	0.00098288	0.00974451
ENSDARG00000098733	pxdn	2612.86125	0.601147488	0.1858634	3.23435108	0.00121919	0.01153209
ENSDARG00000038969	zgc:113142	146.102185	-1.293543386	0.18158021	-7.12381251	1.05E-12	1.19E-10
ENSDARG00000007377	odc1	5036.71893	0.526654341	0.10179346	5.17375442	2.29E-07	8.51E-06
ENSDARG00000036793	ttll5	211.088408	0.71672514	0.20018915	3.58023962	0.00034328	0.00428109
ENSDARG00000019367	tgfb3	2377.94797	0.296712487	0.09059412	3.27518474	0.00105593	0.01028011
ENSDARG00000041952	prox2	134.438825	-1.641867212	0.21646569	-7.58488414	3.33E-14	4.78E-12
ENSDARG00000041960	si:ch211-173a9.6	214.221214	-1.287796355	0.29978283	-4.29576423	1.74E-05	0.0003579
ENSDARG00000087625	si:ch211-173a9.6	194.24998	-1.825724102	0.31624812	-5.77307497	7.78E-09	4.23E-07
ENSDARG00000042839	dph6	343.017443	0.706552371	0.15235461	4.63755168	3.53E-06	9.02E-05
ENSDARG00000098081	GABRR1	327.74259	0.83693946	0.22347073	3.74518603	0.00018026	0.00254277

Table S1. DEGs of WT vs. *terfa+/-*

ENSDARG00000099534	CABZ01066926.1	385.27152	-0.622823124	0.16339268	-3.81181782	0.00013795	0.00205312
ENSDARG00000034063	unm_sa911	205.858955	0.992412687	0.2491069	3.98388276	6.78E-05	0.00112869
ENSDARG00000097339	slc9a1b	292.473312	0.633327033	0.15461716	4.09609798	4.20E-05	0.00075132
ENSDARG00000101180	mcm7	7444.7891	0.575615497	0.17581504	3.2739833	0.00106043	0.0103118
ENSDARG00000114022	WDR1	9712.29239	0.64421047	0.09864101	6.53085841	6.54E-11	5.38E-09
ENSDARG00000059150	tmem107	413.17983	0.601944499	0.14220532	4.23292522	2.31E-05	0.00045467
ENSDARG00000104789	dele1	649.036558	0.350612007	0.11769835	2.97890322	0.00289282	0.02256074
ENSDARG00000028824	slc34a1a	263.45801	0.599513604	0.17652764	3.39614583	0.00068342	0.00735554
ENSDARG00000099772	CU928046.1	32.7706032	-1.293476769	0.44923172	-2.87930863	0.00398548	0.02872112
ENSDARG00000003854	acsl1b	2966.68197	-0.396658976	0.095197	-4.16671711	3.09E-05	0.00058011
ENSDARG00000071881	arl9	665.863854	-0.390825198	0.14087074	-2.77435321	0.00553116	0.03677821
ENSDARG00000041215	cetn4	326.209229	-0.516356722	0.16046795	-3.21781831	0.0012917	0.01206052
ENSDARG00000101318	pcdh2ab9	239.086946	0.563289641	0.18918866	2.97739644	0.00290708	0.02265771
ENSDARG00000103906	pcdh2ab10	92.074863	0.98966771	0.26030133	3.80200782	0.00014353	0.00211088
ENSDARG00000102095	si:ch73-233f7.5	179.403071	-0.609876096	0.22507195	-2.70969398	0.00673453	0.04241935
ENSDARG00000102198	si:ch73-233f7.3	75.1137544	1.303866309	0.31619643	4.12359593	3.73E-05	0.00067985
ENSDARG00000076557	bicc2	281.872053	0.503571094	0.18324723	2.748042	0.00599523	0.03888327
ENSDARG00000109310	im:7150988	1259.26426	0.793219041	0.14539089	5.45576859	4.88E-08	2.16E-06
ENSDARG00000103019	gstp2	743.684332	0.969417463	0.13369248	7.25109956	4.13E-13	4.99E-11
ENSDARG00000055045	casp3b	556.141174	0.569693569	0.11759971	4.84434502	1.27E-06	3.80E-05
ENSDARG00000098746	dhrs13l1	615.459077	0.721437635	0.13146963	5.48748505	4.08E-08	1.84E-06
ENSDARG00000025912	bscl2l	500.408832	-0.472393387	0.15413466	-3.06480954	0.00217809	0.01819097
ENSDARG00000071304	tbc1d2	262.180336	0.669076484	0.22684768	2.94945268	0.00318337	0.02421136
ENSDARG00000089372	clk4a	3608.61157	-0.243364874	0.08443142	-2.88239718	0.00394662	0.02849382
ENSDARG00000041619	rack1	63297.4006	0.273683105	0.10052854	2.72244191	0.00648014	0.0412139
ENSDARG00000040881	hnrnph1	811.466973	0.828723059	0.11247204	7.36825821	1.73E-13	2.18E-11
ENSDARG00000073845	zgc:110843	933.452696	0.943313155	0.12921578	7.30029384	2.87E-13	3.50E-11
ENSDARG00000029443	zgc:92242	1022.74664	0.58682958	0.13772981	4.26073027	2.04E-05	0.00040941
ENSDARG00000089094	si:dkey-160o24.3	90.8188808	-0.794419653	0.2351994	-3.37764321	0.0007311	0.00777555
ENSDARG00000090986	si:dkeyp-115e12.6	1051.71313	-0.323809499	0.11959726	-2.70749923	0.00677922	0.04262524
ENSDARG00000071880	pimr56	2.67802555	4.586648383	1.72875134	2.65315681	0.00797428	0.04804518
ENSDARG00000037574	rps6kal	2151.15843	-0.314461349	0.09332816	-3.36941555	0.00075328	0.00794345
ENSDARG00000052609	CU468164.1	16.9759558	-6.387971334	1.20050642	-5.32106386	1.03E-07	4.21E-06
ENSDARG00000087636	hmgn6	7704.76929	-0.331121823	0.12166708	-2.72154007	0.00649785	0.04128427
ENSDARG00000020527	nup62l	1703.00996	-0.346495607	0.10021017	-3.45768887	0.00054483	0.006161
ENSDARG00000062788	irg1l	1486.67547	0.962305159	0.16264927	5.91644307	3.29E-09	1.93E-07
ENSDARG00000104919	si:ch211-153b23.3	519.221527	0.727977864	0.21262684	3.42373454	0.00061767	0.00682319
ENSDARG00000077169	si:ch211-153b23.4	1003.69666	0.795381361	0.11092993	7.17012408	7.49E-13	8.74E-11
ENSDARG00000058206	si:ch211-153b23.5	2942.20757	0.735081599	0.08700205	8.4490145	2.94E-17	6.82E-15
ENSDARG00000073820	zgc:174917	83.9819279	1.268939249	0.32235843	3.93642337	8.27E-05	0.00132662
ENSDARG00000037655	pls3	6588.91322	0.251204502	0.09379071	2.67835168	0.00739855	0.0455063
ENSDARG00000103917	znf185	2652.85703	0.431080944	0.09958709	4.32868318	1.50E-05	0.00031596
ENSDARG00000101060	gcna	189.147291	-0.784318642	0.18127885	-4.32658652	1.51E-05	0.00031844
ENSDARG00000100047	ergic1	612.631095	0.627205268	0.11129669	5.635435	1.75E-08	8.63E-07
ENSDARG00000099885	trim105	348.166028	-0.372370826	0.13296715	-2.80047241	0.00510279	0.0345651
ENSDARG00000098350	mxd3	616.535744	-0.54684991	0.16801479	-3.25477253	0.00113483	0.01089465
ENSDARG00000100850	rnf103	738.364241	-0.363600738	0.11049597	-3.29062446	0.00099965	0.00986358
ENSDARG00000076594	pcdh12	497.774074	-0.363308373	0.130531	-2.78331103	0.00538072	0.03594136
ENSDARG00000037488	canx	15044.676	0.473565954	0.07531986	6.28739853	3.23E-10	2.32E-08
ENSDARG00000075014	sqstm1	410.50558	0.534254244	0.1224035	4.36469754	1.27E-05	0.00027609
ENSDARG00000041257	smtnl1	201.474522	0.519599552	0.17452978	2.97713974	0.00290951	0.02266264
ENSDARG00000002732	spon2b	766.306617	-0.348616965	0.13015805	-2.6784126	0.0073972	0.0455063
ENSDARG00000077880	si:ch211-255i20.3	71.7856629	-1.106632426	0.3611065	-3.06455967	0.00217991	0.01820006
ENSDARG00000076332	si:ch211-159i8.4	1421.63837	-0.506149809	0.13453588	-3.76219208	0.00016843	0.00241114
ENSDARG00000079781	slitrk4	663.844023	-0.550485373	0.12051083	-4.56793267	4.93E-06	0.00012145
ENSDARG00000117693	CR318673.1	527.321831	-0.443867918	0.14186964	-3.12870272	0.0017558	0.0153768
ENSDARG00000037421	egr1	2591.17213	-1.353586694	0.13428595	-10.0798831	6.78E-24	2.72E-21
ENSDARG00000076667	ccng1	25526.0463	0.743244265	0.09084227	8.18170087	2.80E-16	5.61E-14

Table S1. DEGs of WT vs. *terfa+/-*

ENSDARG00000037738	fbxl3l	108.037522	-1.512072267	0.25680724	-5.88796587	3.91E-09	2.26E-07
ENSDARG00000102162	BX005043.1	8.2304978	3.304917234	1.12351883	2.94157709	0.00326546	0.0246471
ENSDARG00000013979	ndfip1	3089.28905	-0.272158656	0.09112077	-2.98679046	0.00281923	0.02213264
ENSDARG00000025032	nr3c1	2647.01687	-0.282344184	0.083733	-3.37195833	0.00074636	0.00789392
ENSDARG00000056691	cpeb4a	1478.69925	-0.349635563	0.08816015	-3.96591395	7.31E-05	0.00119794
ENSDARG00000056680	stc2a	557.584452	-0.502727439	0.11501914	-4.37081563	1.24E-05	0.00027059
ENSDARG00000034268	slit3	6727.50264	0.311534361	0.08374646	3.71997047	0.00019925	0.00276354
ENSDARG00000000380	pde6a	396.019807	-1.110328872	0.17321069	-6.41027899	1.45E-10	1.13E-08
ENSDARG00000103788	AL645792.2	46.356197	3.950172778	0.61879504	6.3836529	1.73E-10	1.32E-08
ENSDARG00000012019	glra1	1972.4608	-0.316685308	0.11560302	-2.7394208	0.00615475	0.03964147
ENSDARG00000075618	slc36a1	556.101759	0.502766804	0.16130542	3.11686242	0.00182787	0.01579683
ENSDARG00000088439	gm2a	558.501104	0.387863412	0.13478472	2.87765111	0.00400648	0.028839
ENSDARG00000037238	smad5	1693.59976	0.26116701	0.09818724	2.65988734	0.00781668	0.04736313
ENSDARG00000016143	xiap	840.465036	0.566469904	0.11344416	4.99338099	5.93E-07	1.93E-05
ENSDARG00000061603	sorbs2b	2198.47631	-0.348327066	0.08771553	-3.97109922	7.15E-05	0.00117449
ENSDARG00000073709	fam149a	69.4530928	-1.154018255	0.27508743	-4.19509631	2.73E-05	0.00052376
ENSDARG00000061585	cyp4v7	213.747081	1.261252207	0.24147317	5.22315676	1.76E-07	6.72E-06
ENSDARG00000019063	fat1b	1046.32711	-0.627716115	0.15335655	-4.09318094	4.25E-05	0.0007592
ENSDARG00000034757	zdhhc2	150.436645	-0.55613783	0.17886533	-3.1092545	0.0018756	0.01614742
ENSDARG00000008796	her5	5.73099915	2.738809911	0.95568222	2.86581654	0.00415935	0.02967299
ENSDARG00000094324	efemp2a	2048.48525	0.330516256	0.09881978	3.3446365	0.0008239	0.00851129
ENSDARG00000019636	yif1a	896.98466	0.429446558	0.11329985	3.79035413	0.00015043	0.00219812
ENSDARG00000055786	prss23	972.868789	0.310713957	0.10740955	2.89279629	0.00381829	0.02780649
ENSDARG00000075492	ccdc160	132.107789	-0.603335432	0.19875084	-3.03563718	0.00240028	0.01959243
ENSDARG00000017931	ints6l	554.798183	-0.439492149	0.1337986	-3.2847291	0.0010208	0.01002454
ENSDARG00000102058	si:dkeyp-11e3.1	438.887297	0.487156789	0.13588831	3.58497933	0.0003371	0.0042189
ENSDARG00000006683	arhgef6	1270.21335	-0.281805038	0.09114279	-3.09190704	0.00198875	0.01695144
ENSDARG00000001442	inppl1b	378.533221	-0.661654474	0.14083819	-4.69797623	2.63E-06	7.07E-05
ENSDARG00000105424	si:rp71-46j2.7	570.258122	0.679995459	0.17961003	3.78595477	0.00015312	0.00222821
ENSDARG00000105320	AL590151.2	10.1744461	-3.154765034	0.9076735	-3.47566063	0.0005096	0.00585577
ENSDARG00000053166	chic1	306.014372	0.468351833	0.16377958	2.85964737	0.00424112	0.03013337
ENSDARG00000053194	pdzd11	458.213556	0.527522959	0.12975034	4.06567698	4.79E-05	0.00084305
ENSDARG00000036316	rpl39	14432.3757	0.348735237	0.10683229	3.2643242	0.00109726	0.01059529
ENSDARG00000000489	upf3b	1299.46178	-0.457050418	0.12823685	-3.56411148	0.00036509	0.00449447
ENSDARG00000036329	ndufa1	1505.06239	0.508389665	0.10903221	4.66274749	3.12E-06	8.14E-05
ENSDARG00000053279	apln	2858.30185	-0.232347693	0.07649961	-3.03724029	0.00238755	0.019527
ENSDARG00000074285	si:ch73-335m24.2	181.928085	-1.202720827	0.22378592	-5.37442573	7.68E-08	3.26E-06
ENSDARG00000105392	si:ch73-22o18.1	98.4111125	-1.65114307	0.26255426	-6.28876891	3.20E-10	2.31E-08
ENSDARG00000021352	gria1a	1588.29222	0.372307637	0.13205906	2.81925108	0.00481358	0.03300071
ENSDARG00000088168	ablim3	1123.61772	-0.277242122	0.09533852	-2.90797585	0.00363776	0.02684434
ENSDARG00000089917	sh3tc2	1572.23471	-0.356093619	0.0948835	-3.7529562	0.00017476	0.00247706
ENSDARG00000025535	clint1a	2760.07983	0.283755638	0.07655696	3.70646427	0.00021017	0.0028893
ENSDARG00000060338	trmt12	130.961586	0.694073395	0.23491161	2.95461516	0.00313059	0.02391238
ENSDARG00000079591	si:ch211-203d1.3	546.655212	0.624905904	0.15039828	4.15500694	3.25E-05	0.00060606
ENSDARG00000052739	egf	250.500728	-0.71568533	0.23187967	-3.08645139	0.00202561	0.01720074
ENSDARG00000055463	lrit3a	334.606112	-0.846075496	0.26489144	-3.19404621	0.00140294	0.01289094
ENSDARG00000052855	ctso	342.271667	0.646366505	0.17192022	3.7596887	0.00017012	0.00242736
ENSDARG00000089767	rnf130	1663.64841	0.348274427	0.09009954	3.86544055	0.00011089	0.00170701
ENSDARG00000103996	spdl1	457.036344	-0.475137247	0.14716307	-3.22864463	0.00124378	0.0117055
ENSDARG00000019236	gsr	1106.45582	0.427335305	0.12032367	3.55154824	0.00038297	0.00467293
ENSDARG00000092277	BX663611.2	10.3853422	3.348512333	0.86705322	3.86194554	0.00011249	0.00172479
ENSDARG00000034344	pcdh19	3490.54677	0.521868786	0.12815413	4.07219641	4.66E-05	0.00082329
ENSDARG00000020759	elf1	1888.87571	0.288850738	0.09375246	3.08099366	0.00206311	0.01743577
ENSDARG00000098164	aipl2	359.537582	-0.633287167	0.17978251	-3.52251826	0.00042747	0.00511295
ENSDARG00000087574	nox1	224.581099	0.812225539	0.17909178	4.53524736	5.75E-06	0.00013961
ENSDARG00000018145	mid1ip1l	751.480844	-0.315276915	0.10846762	-2.90664537	0.00365327	0.02693624
ENSDARG00000071384	slc7a11	134.444944	-1.760764006	0.21978141	-8.0114327	1.13E-15	2.07E-13
ENSDARG00000080010	adh5	5186.84833	0.39596185	0.09419118	4.20381025	2.62E-05	0.00050789

Table S1. DEGs of WT vs. *terfa+/-*

ENSDARG00000102753	si:dkey-109l4.3	157.757607	-0.823009182	0.20912445	-3.93549965	8.30E-05	0.00132875
ENSDARG00000068265	tomm5	1208.67901	-0.344971101	0.11409985	-3.02341408	0.0024994	0.0202222
ENSDARG00000026137	sfxn5b	930.14049	0.420182679	0.09831779	4.27371969	1.92E-05	0.00038878
ENSDARG00000060977	zgc:153018	2285.56318	0.440528247	0.1169308	3.76742695	0.00016494	0.00236968
ENSDARG00000007025	ttc9c	211.344362	0.855726812	0.17866056	4.78967955	1.67E-06	4.77E-05
ENSDARG00000075727	map1lc3cl	144.774872	-1.010484604	0.23475021	-4.30450994	1.67E-05	0.00034635
ENSDARG00000076811	ganab	3976.03082	0.278611003	0.09430783	2.95427212	0.00313408	0.02393162
ENSDARG00000079854	scyl1	1962.81278	0.262981128	0.08200419	3.20692304	0.00134163	0.01241481
ENSDARG00000099970	CR383676.2	23473.4206	0.761088491	0.1410639	5.39534571	6.84E-08	2.92E-06
ENSDARG00000026926	rom1b	499.696718	-0.882628217	0.25299441	-3.48872611	0.00048533	0.00563157
ENSDARG00000077726	nocta	786.543391	0.39961332	0.1357613	2.94349956	0.00324524	0.02456915
ENSDARG00000103101	si:ch211-235e9.6	847.25394	0.442807239	0.126566	3.49862711	0.00046766	0.00547771
ENSDARG00000038858	setd7	939.011726	0.53888899	0.11601395	4.64503631	3.40E-06	8.73E-05
ENSDARG00000105411	si:ch211-113d11.5	883.318284	-1.648417673	0.56293203	-2.92827124	0.00340853	0.02551919
ENSDARG00000007576	crybb1l1	9162.17673	-3.227072254	0.29767935	-10.8407662	2.21E-27	1.19E-24
ENSDARG00000032929	cryba1l1	11065.9526	-1.970226629	0.19425081	-10.1426944	3.57E-24	1.46E-21
ENSDARG00000039964	fgfbp2a	149.11618	0.75002211	0.20818107	3.60273919	0.00031488	0.00400531
ENSDARG00000115504	CABZ01080056.1	174.573595	1.13304151	0.20008223	5.66287936	1.49E-08	7.60E-07
ENSDARG00000101072	cnot6b	588.894997	0.405994019	0.12948256	3.1355112	0.00171555	0.01508311
ENSDARG00000014233	sept8b	689.883531	-1.1180061	0.33817469	-3.30600174	0.00094637	0.00946565
ENSDARG00000030224	ppp2ca	1875.95209	-0.280707811	0.08698653	-3.22702631	0.00125084	0.01175186
ENSDARG00000023988	dctn4	2203.93173	-0.332759378	0.10971664	-3.03289789	0.00242218	0.01971935
ENSDARG00000106873	LO018188.1	576.569199	-0.8084785	0.1346724	-6.00329783	1.93E-09	1.21E-07
ENSDARG00000114067	tspan17	527.16057	0.48433576	0.17556765	2.75868455	0.00580345	0.03803554
ENSDARG00000068582	rnf44	907.84984	0.539908789	0.15784553	3.42048836	0.00062509	0.00688274
ENSDARG00000111920	cplx2	3103.64017	-0.322262534	0.12138134	-2.65495952	0.00793179	0.04789716
ENSDARG00000102463	cox18	438.421274	0.418153565	0.13928264	3.00219432	0.00268041	0.02131891
ENSDARG00000104138	igfbp7	1636.41141	-0.353410797	0.11243224	-3.14332269	0.00167042	0.01476465
ENSDARG00000101794	atp6v0e1	577.214298	0.412752328	0.13121831	3.14553909	0.00165781	0.0146898
ENSDARG00000098660	CU469526.1	638.698364	-0.538531608	0.12516602	-4.30253843	1.69E-05	0.00034886
ENSDARG00000063670	gtf2a1l	97.7968906	1.105620484	0.30199972	3.66099842	0.00025123	0.00335002
ENSDARG00000116982	FO904997.1	32.6730067	-1.117786238	0.40096126	-2.78776615	0.00530728	0.03563278
ENSDARG00000117011	ewsr1b	482.192122	-1.048625344	0.23432882	-4.47501647	7.64E-06	0.00017814
ENSDARG00000063626	ddx21	5913.60431	-0.267710556	0.08213487	-3.25940193	0.00111647	0.01075997
ENSDARG00000063631	VIT	5835.69296	0.428835699	0.09055468	4.73565489	2.18E-06	6.03E-05
ENSDARG00000094249	wu:fc17b08	223.806809	-0.620282194	0.1457684	-4.25525823	2.09E-05	0.00041787
ENSDARG00000077306	cutc	574.379295	0.615816083	0.1276024	4.8260542	1.39E-06	4.12E-05
ENSDARG00000039832	gsta.2	256.606882	3.053765203	0.26827009	11.3831744	5.07E-30	3.82E-27
ENSDARG00000113274	AL929536.3	232.270816	-0.775440673	0.18867149	-4.1100046	3.96E-05	0.00071571
ENSDARG00000090228	gsta.1	3376.87483	-1.001411575	0.11336053	-8.83386453	1.01E-18	2.76E-16
ENSDARG00000095157	zgc:163080	226.195112	0.546695698	0.19139965	2.85630463	0.00428604	0.03031572
ENSDARG00000074919	BFSP1	434.989103	-3.287790995	0.26208996	-12.5445135	4.26E-36	5.04E-33
ENSDARG00000037337	cnrip1b	483.043021	-0.603782429	0.17053946	-3.54042654	0.00039948	0.00483403
ENSDARG00000008502	pno1	2263.98097	-0.439419392	0.10059109	-4.36837311	1.25E-05	0.00027292
ENSDARG00000063572	perp	8193.02242	0.470565234	0.11385838	4.13289925	3.58E-05	0.00065903
ENSDARG00000035005	bag2	488.118188	-0.469298552	0.1250007	-3.7543673	0.00017378	0.00246676
ENSDARG00000074745	zmp:0000000760	5684.77911	-1.113460703	0.30886898	-3.6049612	0.0003122	0.0039773
ENSDARG00000025320	col21a1	347.343659	-0.511508987	0.15019706	-3.40558586	0.00066022	0.00716586
ENSDARG00000039066	klhl31	5488.64958	0.254156224	0.08220816	3.09161789	0.00199069	0.01696215
ENSDARG00000075245	wdr11	1674.50989	0.389167131	0.09485717	4.10266457	4.08E-05	0.0007344
ENSDARG00000074653	si:ch211-233m11.1	12.1275938	6.10756067	1.3561334	4.50365775	6.68E-06	0.00015811
ENSDARG00000109443	EML4	3069.39068	0.384928613	0.10247983	3.75614037	0.00017255	0.00245147
ENSDARG00000103960	CU019624.1	10.2506834	-2.764234776	0.74846299	-3.69321506	0.00022144	0.00301344
ENSDARG00000063411	lrrc73	550.331343	-0.444383699	0.12820395	-3.46622462	0.00052782	0.00602342
ENSDARG00000075730	dnph1	188.611359	-0.772622592	0.22693617	-3.40458114	0.00066266	0.00718287
ENSDARG00000039820	mea1	1309.42077	-0.487485601	0.09128093	-5.34049795	9.27E-08	3.83E-06
ENSDARG00000007362	sft2d1	940.637838	0.479111935	0.12297213	3.89610179	9.78E-05	0.00152809
ENSDARG00000098941	CU856520.1	512.645357	-0.720447623	0.20281331	-3.55226993	0.00038192	0.00466242

Table S1. DEGs of WT vs. *terfa+/-*

ENSDARG00000024561	nolc1	2431.29733	0.383614245	0.08879504	4.32022155	1.56E-05	0.00032556
ENSDARG00000058574	abcg2c	777.133498	0.536076727	0.11307062	4.74107869	2.13E-06	5.91E-05
ENSDARG00000075559	ppm1g	3283.25036	0.366690582	0.11766767	3.11632396	0.00183121	0.0158202
ENSDARG00000017422	apmap	1813.70466	-0.43949501	0.09995986	-4.39671497	1.10E-05	0.00024389
ENSDARG00000070069	pitx3	537.11073	-0.385766708	0.11770991	-3.27726622	0.00104817	0.01021262
ENSDARG00000027016	gbf1	2869.18857	0.388557734	0.10695352	3.63295876	0.00028019	0.0036584
ENSDARG00000117431	CABZ01065684.1	199.061472	-0.576035886	0.16609172	-3.4681795	0.000524	0.00598802
ENSDARG00000057671	epas1b	1689.56454	-0.51008725	0.13737486	-3.7131047	0.00020473	0.00282544
ENSDARG00000040534	epcam	9063.65638	0.419655349	0.08682463	4.83336746	1.34E-06	3.98E-05
ENSDARG00000069909	HTRA2	437.293236	0.507886288	0.12884719	3.94177235	8.09E-05	0.00130371
ENSDARG00000057881	si:dkey-33c12.3	1902.07086	0.603476208	0.1960188	3.07866495	0.0020793	0.0175309
ENSDARG00000074779	alms1	1034.66854	-0.316298414	0.09980051	-3.16930649	0.00152803	0.01374565
ENSDARG00000003208	pomk	469.41575	0.439176206	0.12108538	3.6269962	0.00028674	0.00372354
ENSDARG00000073716	cpxm1a	1779.93825	-0.334853334	0.08657805	-3.86764706	0.00010989	0.00169543
ENSDARG00000092855	SRBD1	204.095306	0.465330664	0.17304594	2.68905854	0.00716539	0.04444613
ENSDARG00000058004	six2a	764.417333	-0.268785913	0.10043627	-2.6761837	0.00744658	0.0457054
ENSDARG00000017853	prepl	215.260093	0.562726785	0.19023582	2.95804855	0.00309593	0.0237425
ENSDARG00000017165	slc3a1	285.703863	-0.839147323	0.19631588	-4.27447493	1.92E-05	0.00038778
ENSDARG00000056753	dctn1b	2684.57309	0.476901144	0.1187085	4.01741347	5.88E-05	0.00100101
ENSDARG00000056829	zmp:0000000662	785.547352	0.454189284	0.10811832	4.20085398	2.66E-05	0.00051393
ENSDARG00000028201	commd8	524.901627	0.657464337	0.13536217	4.85707579	1.19E-06	3.59E-05
ENSDARG00000078887	atp10d	971.439322	-0.365091769	0.13250322	-2.75534269	0.00586307	0.03832893
ENSDARG00000024278	adh8b	3484.98941	-0.580372717	0.08366071	-6.9372195	4.00E-12	4.01E-10
ENSDARG00000004782	fgfr3	3956.84677	0.896694052	0.09198154	9.74863029	1.87E-22	7.04E-20
ENSDARG00000039579	cfd	2311.69757	1.477931891	0.12144982	12.1690742	4.54E-34	4.27E-31
ENSDARG00000079056	si:ch211-194c3.5	3060.61509	-0.563541772	0.11651588	-4.83660908	1.32E-06	3.93E-05
ENSDARG00000074036	ky	93.1564899	1.548801902	0.33815523	4.58015065	4.65E-06	0.0001154
ENSDARG00000056640	rpia	1936.09637	0.394394089	0.09136223	4.31681759	1.58E-05	0.00032979
ENSDARG00000003311	pank2	1639.25492	-0.296208378	0.09804763	-3.02106616	0.00251886	0.02033334
ENSDARG00000039563	loxl3b	2022.48453	0.312584545	0.08643675	3.61633855	0.0002988	0.00384005
ENSDARG00000062489	8-Mar	963.982302	0.318096005	0.10184869	3.12322147	0.00178883	0.01557317
ENSDARG00000019742	tlr4ba	14.0074305	-1.596296249	0.59898212	-2.66501485	0.00769849	0.0468526
ENSDARG00000039605	mat1a	9433.85164	-0.376401431	0.11813815	-3.18611247	0.00144199	0.01313803
ENSDARG00000057378	selenou1a	1785.27877	-0.477714432	0.10230231	-4.66963505	3.02E-06	7.94E-05
ENSDARG00000093646	AL954359.1	16.6706576	-1.950365783	0.65562769	-2.97480691	0.00293173	0.02281407
ENSDARG00000045089	si:ch211-270n8.1	445.748929	2.412499457	0.39860854	6.05230243	1.43E-09	9.17E-08
ENSDARG00000039937	ccdc172	15.8715971	-1.520464965	0.52542157	-2.89380005	0.0038061	0.02775843
ENSDARG00000092858	si:ch1073-126c3.2	65.617779	-1.262657329	0.42012933	-3.00540152	0.0026523	0.02116309
ENSDARG00000038639	elovl6l	742.26235	1.099042088	0.15827526	6.94386514	3.82E-12	3.84E-10
ENSDARG00000029100	chchd1	851.991846	-0.300564964	0.10167753	-2.95606095	0.00311596	0.02384283
ENSDARG00000069397	zswim8	4924.96617	0.310316272	0.08903773	3.48522231	0.00049173	0.00568725
ENSDARG00000077293	synpo2la	2966.68323	-0.543699042	0.15125651	-3.59454977	0.00032495	0.00410402
ENSDARG00000019728	bmpr1aa	2483.54533	-0.345005268	0.08816634	-3.91311788	9.11E-05	0.00144241
ENSDARG00000076135	mmrn2a	1115.52302	-0.249303438	0.08759863	-2.84597431	0.00442758	0.03102549
ENSDARG00000093584	zgc:193505	2315.80489	0.863153636	0.14583431	5.91872811	3.24E-09	1.91E-07
ENSDARG00000078102	psda	541.235174	-0.603023798	0.11456832	-5.26344296	1.41E-07	5.58E-06
ENSDARG00000117614	CR550304.1	215.436175	-0.781703046	0.17850452	-4.37917784	1.19E-05	0.0002618
ENSDARG00000077728	supv3l1	492.730923	-0.429020461	0.12873793	-3.33251016	0.00086066	0.00882864
ENSDARG00000027172	phactr2	476.874059	-0.377613042	0.13937596	-2.70931255	0.00674228	0.04244664
ENSDARG00000003829	galnt2	2382.94431	0.258659235	0.07577378	3.41357188	0.00064117	0.00702041
ENSDARG00000055618	acta1b	75140.5502	0.36541777	0.13602553	2.68639099	0.00722285	0.04471332
ENSDARG00000034605	zgc:153169	175.356003	0.507683039	0.18551989	2.73654241	0.00620886	0.03988658
ENSDARG00000000551	slc1a4	2559.95692	0.722040976	0.19351323	3.73122285	0.00019055	0.00267128
ENSDARG00000079011	col17a1b	1662.02477	0.333070811	0.09678093	3.4414922	0.00057852	0.00644664
ENSDARG00000029928	ap3m1	869.168856	-0.327589635	0.10589774	-3.09345251	0.00197842	0.01687498
ENSDARG00000022183	gsto1	57.709833	1.434135892	0.34186958	4.19497956	2.73E-05	0.00052376
ENSDARG00000061413	sec23ip	1497.14207	0.441742891	0.09518633	4.64082257	3.47E-06	8.90E-05
ENSDARG00000031981	pcbd1	157.280072	0.835922966	0.23507784	3.55594119	0.00037663	0.00460911

Table S1. DEGs of WT vs. *terfa+/-*

ENSDARG00000055276	rel	768.864887	0.494813911	0.17315857	2.85757672	0.00426889	0.03023931
ENSDARG00000021547	vrk2	196.318502	0.890720699	0.18517566	4.81013929	1.51E-06	4.37E-05
ENSDARG00000055190	slc17a5	2385.94266	-0.279887414	0.08549285	-3.27381075	0.00106108	0.01031406
ENSDARG00000022614	ddx43	361.583894	-0.840617303	0.25942772	-3.24027561	0.00119414	0.01136028
ENSDARG00000092448	CR762431.1	121.188688	-0.881590421	0.30333703	-2.90630667	0.00365723	0.02695425
ENSDARG00000039399	nt5c2a	2259.61829	-0.320062482	0.10009355	-3.19763353	0.0013856	0.01275052
ENSDARG00000011862	inaa	301.416259	-0.861122196	0.28368138	-3.03552592	0.00240117	0.01959323
ENSDARG00000033327	unc5b	1142.52762	0.381828332	0.12311584	3.10137443	0.00192625	0.01648651
ENSDARG00000017128	myofl	1176.69702	0.508553823	0.14589446	3.48576508	0.00049073	0.00568366
ENSDARG00000015854	chata	1131.80387	-0.419487859	0.12103833	-3.46574397	0.00052877	0.00603066
ENSDARG00000054890	rgra	380.867768	-0.512280675	0.14157196	-3.61851781	0.0002963	0.00381299
ENSDARG00000030626	lrit2	102.185333	-1.023219884	0.3419178	-2.99259029	0.00276621	0.0218267
ENSDARG00000004643	cdhr1a	432.187952	-0.784826919	0.18162479	-4.32114417	1.55E-05	0.00032503
ENSDARG00000030265	scdb	1802.89841	-0.461607308	0.09852256	-4.68529545	2.80E-06	7.44E-05
ENSDARG00000037116	cxcl12a	2049.94803	-0.353497275	0.12138106	-2.91229347	0.00358785	0.02660091
ENSDARG00000054312	slc38a6	387.734477	0.444767793	0.13712824	3.24344429	0.00118094	0.01125901
ENSDARG00000006181	hif1aa	367.138709	0.465430371	0.13847542	3.36110467	0.00077631	0.00812433
ENSDARG00000014091	osr1	638.806549	-0.32329747	0.1189038	-2.71898357	0.00654829	0.04149012
ENSDARG00000006891	kcns3a	272.720779	-0.474070583	0.15826731	-2.99537908	0.00274104	0.02167628
ENSDARG00000069265	matn3b	485.16371	-0.976028173	0.22010785	-4.43431785	9.24E-06	0.00020889
ENSDARG00000002403	nusap1	1297.59736	-0.588092055	0.18514835	-3.17632888	0.00149152	0.01349042
ENSDARG00000014246	jag2a	460.525601	0.530452096	0.16481879	3.21839572	0.0012891	0.01204532
ENSDARG00000013763	rrbp1a	3012.06031	0.230115374	0.067578	3.40518167	0.0006612	0.00717335
ENSDARG00000016656	mgme1	190.612378	-0.636371633	0.16995486	-3.74435684	0.00018086	0.00254829
ENSDARG00000010823	memo1	954.020286	0.402478308	0.1026849	3.91954725	8.87E-05	0.00140896
ENSDARG00000060410	thbs2a	791.06899	0.607627386	0.16621688	3.65562991	0.00025655	0.00341176
ENSDARG00000087911	psme4a	400.7528	-0.69847712	0.12111573	-5.7670225	8.07E-09	4.35E-07
ENSDARG00000086309	erlec1	1545.60745	0.358287163	0.10056137	3.56287089	0.00036682	0.00450839
ENSDARG00000005454	tacc3	1614.8777	-0.433301909	0.16207442	-2.67347501	0.00750699	0.04596299
ENSDARG00000007219	actn1	5521.54914	0.735554419	0.08236523	8.93039992	4.24E-19	1.21E-16
ENSDARG00000017329	cdkl1	441.496646	-0.49746301	0.15917463	-3.12526572	0.00177645	0.01549662
ENSDARG00000002197	pygl	3734.57438	-0.472184798	0.17499402	-2.69829108	0.00696965	0.04352527
ENSDARG00000031483	col9a1b	11922.7875	-1.010978601	0.27704399	-3.64916273	0.0002631	0.00348389
ENSDARG00000014103	dkk1a	145.084021	0.516557648	0.18476458	2.79576128	0.00517776	0.03493303
ENSDARG00000075143	nanp	207.264086	0.558000791	0.18755202	2.97517874	0.00292818	0.02279357
ENSDARG00000052783	cdc42ep3	931.553841	-0.378241452	0.13273259	-2.84965018	0.00437673	0.03079947
ENSDARG00000056986	dact2	352.676061	0.429412704	0.16234425	2.64507484	0.00816729	0.04884453
ENSDARG00000095283	si:dkeyp-2e4.3	91.3440452	0.828929649	0.24280661	3.41395004	0.00064028	0.00701686
ENSDARG00000030440	rsrp1	3691.97269	0.488815891	0.12785076	3.82333179	0.00013166	0.0019761
ENSDARG00000098286	CU695232.1	356.120192	0.715992597	0.14695642	4.87214229	1.10E-06	3.37E-05
ENSDARG00000104541	msh6	1509.8427	0.414537175	0.13550665	3.05916477	0.00221955	0.01845048
ENSDARG00000021853	ntpcr	226.941166	0.958743719	0.20483478	4.68057096	2.86E-06	7.59E-05
ENSDARG00000103163	tomm20a	1184.77607	-0.690423285	0.14227395	-4.8527737	1.22E-06	3.66E-05
ENSDARG00000090656	tomm20a	816.340924	0.585588772	0.12978005	4.5121634	6.42E-06	0.00015336
ENSDARG00000046133	b3galnt2	340.420704	0.503657963	0.14929522	3.37357066	0.000742	0.00785787
ENSDARG00000068710	nid1a	5291.01054	-0.506054607	0.09686573	-5.22428925	1.75E-07	6.69E-06
ENSDARG00000068738	cox5b2	264.163195	-0.568159912	0.15468078	-3.67311261	0.00023961	0.00321597
ENSDARG00000101663	CABZ01078663.1	3.08863541	5.259266726	1.62306411	3.2403321	0.00119391	0.01136028
ENSDARG00000117816	CABZ01078767.1	51.6071377	1.009069986	0.3750866	2.69023206	0.00714023	0.04434546
ENSDARG00000101080	pwwp2b	1597.25747	0.324202141	0.11939867	2.71529113	0.00662175	0.04184685
ENSDARG00000062568	mrpl2	2995.54911	0.547518213	0.09384641	5.83419438	5.41E-09	3.05E-07
ENSDARG00000103320	lclat1	2125.32229	0.367973545	0.12386023	2.9708772	0.0029695	0.02302872
ENSDARG00000100613	CABZ01089777.1	265.075355	-1.178557994	0.25291814	-4.65983977	3.16E-06	8.23E-05
ENSDARG00000059349	homer2	1131.33033	0.448460799	0.14756925	3.03898551	0.00237376	0.01944318
ENSDARG00000087941	pde8a	1018.6762	0.323894037	0.103004	3.14448031	0.00166382	0.01472363
ENSDARG00000036237	slc27a2a	4995.88859	0.630659376	0.15985112	3.94529226	7.97E-05	0.00128972
ENSDARG00000092774	tars3	1288.00789	-0.880367132	0.14292525	-6.15963328	7.29E-10	4.95E-08
ENSDARG00000101085	ss18l2	232.5394	-0.685240336	0.20649402	-3.31845119	0.00090518	0.00919238

Table S1. DEGs of WT vs. *terfa+/-*

ENSDARG00000019081	DHDH	1271.52914	0.623003302	0.15326008	4.06500716	4.80E-05	0.00084488
ENSDARG00000028336	dhdhl	1487.01584	0.473522569	0.10109566	4.68390593	2.81E-06	7.47E-05
ENSDARG00000102249	pepd	1540.06177	0.580488358	0.11241188	5.16394139	2.42E-07	8.90E-06
ENSDARG00000099161	dnaaf4	42.3801411	1.124719013	0.37689872	2.9841412	0.00284376	0.02228102
ENSDARG00000103935	rab27a	787.304551	0.452515878	0.14475965	3.12598065	0.00177213	0.01547658
ENSDARG00000098832	fam168a	1012.33642	0.584741361	0.1652519	3.53848495	0.00040243	0.00486025
ENSDARG00000098597	CU570791.3	74.2168005	-0.8979681	0.33009282	-2.72035029	0.00652128	0.04138024
ENSDARG00000102953	ints4	854.368288	-0.355635442	0.09899252	-3.59254876	0.00032746	0.0041231
ENSDARG00000100555	aqp11	1318.63052	0.913417248	0.11872892	7.69330011	1.43E-14	2.19E-12
ENSDARG00000103959	pak1	1499.98157	0.33767697	0.11862609	2.84656586	0.00441936	0.03098479
ENSDARG00000099871	myo7aa	804.837865	-0.485568177	0.10871777	-4.46631859	7.96E-06	0.00018416
ENSDARG00000099439	serp1	1648.78291	0.903242266	0.11904807	7.5872061	3.27E-14	4.72E-12
ENSDARG00000094185	AL954149.2	30.3070063	1.110082273	0.41718685	2.66087552	0.00779378	0.04724739
ENSDARG00000113393	golim4a	1494.70437	0.500006185	0.09247245	5.40708245	6.41E-08	2.75E-06
ENSDARG00000024771	slc24a5	214.482928	0.620721653	0.16519272	3.75756066	0.00017158	0.00243968
ENSDARG00000059398	myef2	4571.22373	1.06578911	0.13782663	7.73282433	1.05E-14	1.65E-12
ENSDARG00000098096	slc12a1	161.289569	-1.57295451	0.28980785	-5.42757735	5.71E-08	2.47E-06
ENSDARG00000007180	slc30a4	1401.41139	0.538222366	0.1531599	3.51412064	0.00044121	0.00524164
ENSDARG00000061763	spata5l1	172.507827	0.540954984	0.20447859	2.64553368	0.00815622	0.04879008
ENSDARG00000016080	nob1	716.960305	-0.409397352	0.14414179	-2.84024058	0.00450795	0.03141159
ENSDARG00000101210	glg1b	637.015065	0.654621601	0.14637237	4.47230293	7.74E-06	0.00017941
ENSDARG00000088549	si:ch1073-390k14.1	308.005867	0.776736356	0.14593104	5.32262594	1.02E-07	4.18E-06
ENSDARG00000008447	fkbp4	3576.37343	0.341441427	0.07707953	4.42972888	9.44E-06	0.00021261
ENSDARG00000078508	si:dkey-266m15.6	1587.82374	0.484849139	0.13860007	3.49818824	0.00046843	0.00548414
ENSDARG00000095956	CU467655.1	35.5947836	-1.589835036	0.5010687	-3.17288833	0.00150931	0.01362787
ENSDARG00000117755	CU467655.2	100.615359	-1.742871753	0.3844745	-4.53312704	5.81E-06	0.00014051
ENSDARG00000005115	calub	2166.9165	0.375518147	0.10856301	3.45898795	0.00054221	0.00614248
ENSDARG00000070472	arf5	5053.48283	0.295723551	0.09769637	3.02696554	0.00247022	0.0200383
ENSDARG00000113899	zgc:77650	1223.60508	0.255175948	0.09225569	2.76596425	0.00567548	0.03747062
ENSDARG00000093936	si:dkeyp-1h4.6	431.311713	0.640979013	0.15306204	4.18770728	2.82E-05	0.00053587
ENSDARG00000042387	zgc:77752	102.91511	-1.011260181	0.26417121	-3.82804839	0.00012916	0.00194331
ENSDARG00000088343	prrt4	165.437288	-0.82115326	0.18291991	-4.48914097	7.15E-06	0.00016831
ENSDARG00000040100	si:dkey-95h12.1	1130.86532	-0.37879131	0.13487802	-2.80839919	0.00497885	0.03388934
ENSDARG00000017369	sema3d	1757.99197	-0.253536327	0.09446327	-2.68396729	0.00727542	0.04495672
ENSDARG00000042189	tspan33b	43.9848918	-1.72760655	0.38125801	-4.53133176	5.86E-06	0.00014117
ENSDARG00000007461	srgap1a	941.575202	0.42798855	0.12902891	3.31699728	0.00090991	0.00922682
ENSDARG00000026829	cotl1	12583.4828	0.299234929	0.08031137	3.72593472	0.00019459	0.00271872
ENSDARG00000068763	plcg2	221.39179	-0.514505465	0.19519449	-2.63586056	0.00839242	0.04991472
ENSDARG00000092862	si:ch211-260p9.3	13.6644165	-1.816418647	0.57261106	-3.17216828	0.00151305	0.01363652
ENSDARG00000011583	cry1b	620.113767	0.701667824	0.12844256	5.46289189	4.68E-08	2.08E-06
ENSDARG00000030465	tmem263	1299.78362	0.355251266	0.12270884	2.89507475	0.00379068	0.02767031
ENSDARG00000062893	si:dkey-103i16.6	154.763653	0.782860737	0.23045866	3.39696821	0.00068137	0.00734407
ENSDARG00000005972	ric8b	1383.71498	0.275690807	0.08397264	3.28310267	0.00102671	0.01004683
ENSDARG00000100057	ppfibp1b	1945.02468	0.476853879	0.10992179	4.33811978	1.44E-05	0.00030502
ENSDARG00000087659	si:ch211-219a15.3	113.456251	-1.601676665	0.26408858	-6.06492214	1.32E-09	8.59E-08
ENSDARG00000111261	FO834850.1	377.332762	0.562960526	0.13364379	4.21239563	2.53E-05	0.00049218
ENSDARG00000041691	bhlhe41	643.509138	1.029968098	0.15454086	6.66469765	2.65E-11	2.33E-09
ENSDARG00000057698	ctsd	8443.47641	0.473506379	0.13464572	3.51668351	0.00043697	0.00520163
ENSDARG00000062483	lyve1b	127.479163	-1.072518187	0.23207035	-4.62152179	3.81E-06	9.69E-05
ENSDARG00000092364	si:ch211-218c6.8	511.783263	-0.73717093	0.19549911	-3.77071247	0.00016278	0.00234141
ENSDARG00000041572	zfpm1	855.729191	0.443997471	0.12516507	3.54729535	0.00038921	0.00473508
ENSDARG00000013855	slc12a3	237.336891	-1.126519968	0.21453299	-5.2510338	1.51E-07	5.88E-06
ENSDARG00000030872	cetp	356.861008	-0.805777893	0.14342755	-5.61801319	1.93E-08	9.43E-07
ENSDARG00000069122	si:ch211-216l23.2	1096.29194	0.435270253	0.11124468	3.91272878	9.13E-05	0.00144382
ENSDARG00000101438	lonp2	1191.61332	0.664258019	0.10508794	6.32097296	2.60E-10	1.92E-08
ENSDARG00000094021	BX649641.5	3.5854582	-4.16604303	1.56094802	-2.66891849	0.00760959	0.04642523
ENSDARG00000016038	hacd3	875.208786	-0.326388806	0.10949461	-2.98086654	0.00287434	0.02243773
ENSDARG00000069189	si:dkey-242h9.3	88.3404579	3.900373905	1.07677776	3.62226453	0.00029204	0.00376871

Table S1. DEGs of WT vs. *terfa+/-*

ENSDARG00000016337	lctlb	305.584758	-2.429005712	0.16008489	-15.1732352	5.32E-52	2.64E-48
ENSDARG00000015476	iqch	113.216029	-1.75374838	0.23676427	-7.40714972	1.29E-13	1.67E-11
ENSDARG00000041140	ddb2	594.733641	0.649179111	0.13385734	4.8497833	1.24E-06	3.71E-05
ENSDARG00000062370	bcl2l13	1687.78083	0.359796005	0.10749407	3.34712429	0.00081655	0.00846118
ENSDARG00000004823	asz1	32.8929745	2.402130118	0.43900377	5.47177556	4.46E-08	1.99E-06
ENSDARG00000041078	chka	1197.98027	-0.58324665	0.1479807	-3.94136978	8.10E-05	0.00130505
ENSDARG00000041073	si:dkey-12e7.4	47.3909993	2.002502906	0.46558973	4.30100314	1.70E-05	0.000351
ENSDARG00000040942	pnp6	447.083568	-0.554013401	0.16765754	-3.30443472	0.00095168	0.00950709
ENSDARG00000056525	bcar1	2167.06325	0.375444574	0.0896224	4.18918235	2.80E-05	0.00053371
ENSDARG00000013251	hsf4	87.2776624	-2.116951955	0.36198953	-5.84810269	4.97E-09	2.81E-07
ENSDARG00000016676	gnao1a	5874.6092	0.369280879	0.12512019	2.95140912	0.00316328	0.02410277
ENSDARG00000092636	AL935295.1	40.1147672	-1.116890879	0.37638278	-2.9674335	0.00300297	0.02320672
ENSDARG00000095451	si:ch211-196l7.4	1835.28196	-0.469786764	0.11951155	-3.93088993	8.46E-05	0.00135188
ENSDARG00000068709	fam174b	416.239213	-0.447343315	0.15200861	-2.94288144	0.00325173	0.02458082
ENSDARG00000018788	st8sia2	162.318472	0.887220749	0.2479895	3.57765444	0.00034669	0.00431714
ENSDARG00000075881	si:ch211-39k3.2	190.409075	1.239976591	0.24630587	5.03429565	4.80E-07	1.60E-05
ENSDARG00000041108	ctsh	412.425825	0.591224174	0.20802043	2.84214472	0.00448111	0.0312772
ENSDARG00000060885	znf592	654.13222	-0.75528753	0.12600205	-5.99424773	2.04E-09	1.26E-07
ENSDARG00000060841	pik3c2a	2410.7074	0.259899515	0.09124671	2.84831664	0.00439512	0.03086766
ENSDARG00000061977	ppfibp2a	989.590927	-0.396568695	0.11172532	-3.54949693	0.00038597	0.00470487
ENSDARG00000007976	si:ch211-220f16.2	1236.00906	-0.307554164	0.0935035	-3.2892263	0.00100463	0.00990087
ENSDARG00000099336	mvda	305.116651	0.626658071	0.17750075	3.53045306	0.00041485	0.00498601
ENSDARG00000020237	mc1r	102.558045	0.625457076	0.20372741	3.07006837	0.0021401	0.01792792
ENSDARG00000068208	def8	737.185584	0.510542206	0.12160834	4.19824979	2.69E-05	0.00051827
ENSDARG00000101059	dhx36	850.173352	0.272008311	0.10217032	2.66230262	0.00776081	0.04715348
ENSDARG00000092510	CR405715.1	8.60191068	-3.709823089	0.93707019	-3.95895965	7.53E-05	0.00122931
ENSDARG00000093764	si:dkey-145c18.5	9.41764859	3.239521598	0.90398607	3.58359681	0.0003389	0.00423278
ENSDARG00000078738	si:dkey-145c18.3	11.5467819	2.976762961	1.09066673	2.72930572	0.00634678	0.0405421
ENSDARG00000079848	gmps	2987.32145	0.467222768	0.11320986	4.12705022	3.67E-05	0.00067104
ENSDARG00000074996	tbrg1	403.086575	-0.39051451	0.12090644	-3.22989004	0.00123838	0.01166961
ENSDARG00000067741	itpkcb	1666.42487	0.587432373	0.1323599	4.43814469	9.07E-06	0.00020577
ENSDARG00000013777	ppp1r13l	2104.3884	0.398712492	0.09397238	4.24286881	2.21E-05	0.00043775
ENSDARG00000055751	fosb	139.677931	-0.898069256	0.20909084	-4.29511515	1.75E-05	0.00035866
ENSDARG00000068401	yap1	4125.52265	0.307597522	0.09541313	3.22384897	0.0012648	0.0118584
ENSDARG00000036291	nucb2b	546.280596	0.440706607	0.14087015	3.12845991	0.00175725	0.0153768
ENSDARG00000032103	mapk6	2944.04625	-0.365925194	0.12817508	-2.85488557	0.00430523	0.03041942
ENSDARG00000086288	scg3	3055.97975	-0.452238772	0.09721667	-4.65186465	3.29E-06	8.47E-05
ENSDARG00000093591	si:ch211-282k23.2	42.2375551	2.2108993	0.45442529	4.86526469	1.14E-06	3.47E-05
ENSDARG00000006372	ugt5c2	22.9848154	2.693604116	0.52332075	5.14713798	2.64E-07	9.64E-06
ENSDARG00000005176	zgc:101040	383.827732	0.509509782	0.12937418	3.93826495	8.21E-05	0.00131948
ENSDARG00000003420	chrna5	284.770623	-0.449283641	0.14213637	-3.16093365	0.00157264	0.01407057
ENSDARG00000061480	si:ch211-132b12.1	17.9605734	-1.332969712	0.48158346	-2.76788933	0.00564206	0.03729612
ENSDARG00000094946	si:ch211-132b12.2	118.599123	1.505659291	0.24691679	6.09784092	1.08E-09	7.12E-08
ENSDARG00000068374	si:ch211-132b12.7	309.068533	2.306193757	0.29957731	7.69815897	1.38E-14	2.13E-12
ENSDARG00000100607	zbtb38	114.596511	-0.750631151	0.26222414	-2.86255545	0.0042024	0.02994108
ENSDARG00000055124	ccnl1a	3280.22	-0.432779099	0.10503736	-4.12023958	3.78E-05	0.00068816
ENSDARG00000061370	tsen34	375.78692	0.474824431	0.14661828	3.2385078	0.00120157	0.01141464
ENSDARG00000040396	bcl7bb	392.465763	-0.374607759	0.13814686	-2.71166316	0.00669466	0.04221099
ENSDARG00000014673	fzd9b	1033.7074	0.403876679	0.10929449	3.69530695	0.00021962	0.00299434
ENSDARG00000055524	rnf7	1972.17764	0.274135535	0.09836226	2.78699907	0.00531986	0.03565944
ENSDARG00000010654	arhgap42b	134.056256	-0.737324635	0.19371722	-3.80619051	0.00014112	0.00208761
ENSDARG00000011693	ttc36	399.19269	-0.744936747	0.18161285	-4.10178435	4.10E-05	0.00073574
ENSDARG00000086034	nectin1b	1920.24771	0.50380193	0.14097819	3.57361621	0.00035208	0.00436027
ENSDARG00000055054	bcl9l	2008.216	0.305871333	0.09795679	3.12251281	0.00179314	0.01558887
ENSDARG00000061328	cdon	5413.61139	0.260069299	0.07607158	3.41874472	0.00062911	0.00691573
ENSDARG00000054864	aplp2	5983.84457	0.511169945	0.16279642	3.13993359	0.00168986	0.01490472
ENSDARG00000061173	st14a	3597.34399	0.447908083	0.08793236	5.09377984	3.51E-07	1.23E-05
ENSDARG00000061168	dhx34	480.592881	-0.436502091	0.15951609	-2.73641417	0.00621128	0.03989182

Table S1. DEGs of WT vs. *terfa+/-*

ENSDARG00000040362	ehd2b	1691.82626	0.61535711	0.12298137	5.00366132	5.63E-07	1.83E-05
ENSDARG00000061124	srpra	2716.22878	0.243901722	0.08311125	2.93464157	0.00333933	0.025098
ENSDARG00000068242	cngb1a	470.726515	-0.990772259	0.24062668	-4.11746639	3.83E-05	0.00069547
ENSDARG00000007990	wt1b	107.487547	-0.628522156	0.2107617	-2.98214601	0.00286235	0.02237227
ENSDARG00000033899	ap2m1b	7046.05182	0.357462361	0.09839576	3.63290418	0.00028025	0.0036584
ENSDARG00000009825	mmp23bb	449.689673	0.465801939	0.1518484	3.06754594	0.00215824	0.0180495
ENSDARG00000096829	blvrb	947.563272	0.394364715	0.12115914	3.25493159	0.0011342	0.01089276
ENSDARG00000005468	irf2bp1	2373.12446	0.209303861	0.07872332	2.65872746	0.00784364	0.04745707
ENSDARG00000021462	daw1	196.617846	0.72763342	0.21134744	3.44283053	0.00057566	0.00642827
ENSDARG00000041098	barx2	258.465187	0.415292308	0.15488103	2.68136325	0.00733229	0.04519941
ENSDARG00000090866	kcnj1a.2	284.864675	-0.878337868	0.25823353	-3.40133162	0.00067058	0.00725931
ENSDARG00000091232	kcnj1a.1	142.878697	-1.030295887	0.29415989	-3.50250291	0.00046091	0.00541394
ENSDARG00000054584	FO681288.1	117.40804	-0.799554159	0.20390666	-3.9211773	8.81E-05	0.00140036
ENSDARG00000015064	esrrd	56.1052896	-1.091567577	0.32597066	-3.34866819	0.00081201	0.0084375
ENSDARG00000040505	yif1b	477.01334	0.757146178	0.15587366	4.85743496	1.19E-06	3.59E-05
ENSDARG00000040274	scamp5b	1003.82047	-0.631107895	0.22053876	-2.86166426	0.00421423	0.02998447
ENSDARG00000003061	cd276	1048.12692	0.399018444	0.10082363	3.95758852	7.57E-05	0.00123476
ENSDARG00000100991	chrna3	215.867825	-0.544354303	0.15690718	-3.46927598	0.00052186	0.00596638
ENSDARG00000103369	taldo1	4536.68172	0.3643363	0.09397633	3.87689437	0.0001058	0.00163839
ENSDARG00000011777	cttn	4340.51402	0.387139004	0.09388503	4.12354344	3.73E-05	0.00067985
ENSDARG00000099803	pik3c2g	417.70978	-0.366559801	0.13162739	-2.78482931	0.00535559	0.03581425
ENSDARG00000096602	si:ch1073-357b18.4	376.845935	0.353911028	0.13297729	2.66143969	0.00778073	0.04719452
ENSDARG00000103383	dnah9	60.3836487	-0.956007962	0.31108621	-3.0731287	0.00211827	0.01778103
ENSDARG00000076357	CABZ01102039.1	729.323952	-0.580208578	0.11677284	-4.96869471	6.74E-07	2.15E-05
ENSDARG00000026090	adprm	264.999052	1.218954375	0.16016096	7.61080845	2.72E-14	3.98E-12
ENSDARG00000113674	CABZ01058222.1	266.292255	0.946814674	0.17800692	5.31897667	1.04E-07	4.24E-06
ENSDARG00000059903	hs3st3l	243.593941	-0.589193204	0.17296441	-3.40644177	0.00065816	0.00715593
ENSDARG00000071877	dhrs7cb	1516.44629	0.382220318	0.12899474	2.96306908	0.00304588	0.0234382
ENSDARG00000060001	mettl9	666.193485	0.61089637	0.13533779	4.51386382	6.37E-06	0.00015228
ENSDARG00000037358	polr3e	1852.44	-0.293204061	0.07602848	-3.85650288	0.00011502	0.0017582
ENSDARG00000105301	sox8a	109.391396	-0.806036568	0.27554962	-2.92519573	0.0034424	0.02573256
ENSDARG00000103614	pemt	178.963042	0.931256585	0.1814057	5.13355752	2.84E-07	1.03E-05
ENSDARG00000096631	MED9	281.158597	0.446986816	0.14309051	3.12380486	0.00178529	0.01555322
ENSDARG00000096519	irbpl	16.785616	7.316276696	1.27347244	5.74513938	9.18E-09	4.90E-07
ENSDARG00000014005	lrrc45	554.391409	0.351910471	0.13174759	2.67109601	0.0075604	0.04620452
ENSDARG00000079271	dcxr	792.106225	0.913210295	0.13593069	6.7182056	1.84E-11	1.67E-09
ENSDARG00000035809	col1a1b	106648.106	0.382738626	0.09787534	3.91047038	9.21E-05	0.00145461
ENSDARG00000114508	tmem101	502.564157	1.025416176	0.18327064	5.59509238	2.21E-08	1.06E-06
ENSDARG00000109510	lsm12b	2130.26089	0.442394187	0.08878653	4.98267241	6.27E-07	2.02E-05
ENSDARG00000010556	mmp25b	141.73444	-1.148140428	0.20526419	-5.59347666	2.23E-08	1.06E-06
ENSDARG00000063435	trpm4b.2	892.864182	0.885360955	0.13504691	6.55595117	5.53E-11	4.61E-09
ENSDARG00000075022	si:ch211-93e11.8	4.15584433	3.466184384	1.30224532	2.66169847	0.00777475	0.04719452
ENSDARG00000101199	rbp4	35157.2354	-0.682451805	0.09664261	-7.06160339	1.65E-12	1.79E-10
ENSDARG00000031616	g6pca.1	362.979467	0.630458152	0.23124812	2.72632768	0.00640434	0.04084676
ENSDARG00000012829	asah2	106.883752	-1.281755318	0.35281189	-3.63297086	0.00028018	0.0036584
ENSDARG00000045219	dkk1b	181.624294	0.638223291	0.18007429	3.54422217	0.00039377	0.00477488
ENSDARG00000104687	slc16a9b	1264.8492	0.764353731	0.10174993	7.51208125	5.82E-14	7.99E-12
ENSDARG00000079468	rhobtb1	183.295066	0.945456106	0.22629414	4.17799638	2.94E-05	0.00055628
ENSDARG00000099986	si:ch211-207i20.2	129.008295	-0.570332717	0.18659939	-3.05645542	0.00223971	0.01858075
ENSDARG00000031293	dnajc9	701.211743	-0.360966238	0.13288809	-2.71631754	0.00660126	0.04173855
ENSDARG00000033537	p4ha1a	1384.4181	0.545072053	0.10757999	5.06666764	4.05E-07	1.40E-05
ENSDARG00000057429	arg1	37.6542755	-1.5511941	0.36497468	-4.25014163	2.14E-05	0.0004265
ENSDARG00000096505	si:ch73-103l1.2	98.1105124	-0.898371185	0.25927822	-3.46489258	0.00053044	0.00604501
ENSDARG00000057321	tut1	466.783992	-0.387730041	0.1058751	-3.66214553	0.00025011	0.00333684
ENSDARG00000035835	eef2k	3976.81024	0.578502759	0.10844445	5.33455392	9.58E-08	3.93E-06
ENSDARG00000023299	snu13b	1308.03954	-0.270957034	0.09509164	-2.84943079	0.00437975	0.03081199
ENSDARG00000006112	myof	1676.92882	0.460247683	0.1138749	4.04169571	5.31E-05	0.0009217
ENSDARG00000020041	vps35l	1450.37618	0.242364923	0.09133629	2.65354475	0.00796512	0.04801675

Table S1. DEGs of WT vs. *terfa+/-*

ENSDARG00000055527	cmn	3562.49757	-1.075655182	0.20382434	-5.27736382	1.31E-07	5.21E-06
ENSDARG00000074160	paqr4b	78.3025041	-1.085208189	0.37369917	-2.90396199	0.00368473	0.02709752
ENSDARG00000055477	pelo	1221.78005	0.262967579	0.09890224	2.6588638	0.00784047	0.04745707
ENSDARG00000016457	irf9	366.620065	0.614532444	0.17632344	3.48525659	0.00049167	0.00568725
ENSDARG00000071573	lsm5	183.306086	0.784719921	0.22121209	3.54736456	0.00038911	0.00473508
ENSDARG00000045129	fkbp10b	1624.75631	0.399339928	0.10491598	3.80628327	0.00014107	0.00208761
ENSDARG00000055679	mto1	748.162333	0.827391823	0.0963714	8.58544975	9.05E-18	2.29E-15
ENSDARG00000036482	hexim1	1996.16294	-0.313156999	0.09705178	-3.22670016	0.00125227	0.01175862
ENSDARG00000027070	acbd4	105.426456	0.598217352	0.21160935	2.82698926	0.00469879	0.03241422
ENSDARG00000079572	plcd3b	390.681501	-0.42425671	0.13352745	-3.17729965	0.00148653	0.01346326
ENSDARG00000039173	ctslb	21.2113142	-1.472446983	0.51866586	-2.83891248	0.00452676	0.03150729
ENSDARG00000089885	slc16a12b	1724.81473	0.531860226	0.11848847	4.48870854	7.17E-06	0.0001685
ENSDARG00000002298	ankrd22	109.489299	0.737906091	0.2692977	2.74011288	0.00614181	0.03959913
ENSDARG00000056600	papss2b	2900.27097	0.703737457	0.08434544	8.34351484	7.21E-17	1.55E-14
ENSDARG00000022817	pvalb3	292.885942	1.099854307	0.19944583	5.51455156	3.50E-08	1.61E-06
ENSDARG00000075046	rnf151	28.7930488	-2.931163945	0.51950504	-5.64222427	1.68E-08	8.36E-07
ENSDARG00000045159	zdhhc4	447.105203	0.351644971	0.12112972	2.90304453	0.00369554	0.02715928
ENSDARG00000076229	mrtfab	2243.01577	0.323220342	0.11069334	2.91996194	0.00350074	0.02609023
ENSDARG00000075903	chadlb	636.231759	-0.416878241	0.14437302	-2.88750785	0.00388307	0.02814631
ENSDARG00000039117	tefa	3621.16812	0.634396448	0.10546196	6.01540519	1.79E-09	1.13E-07
ENSDARG00000055868	rsl1d1	1097.77835	-0.278937603	0.10571925	-2.63847509	0.00832798	0.04963837
ENSDARG00000100072	mgrn1a	1021.10394	-0.367576188	0.10379971	-3.54120646	0.0003983	0.00482211
ENSDARG00000045144	zgc:163057	58.5188535	1.368664993	0.39260841	3.48608172	0.00049015	0.00567958
ENSDARG00000038147	hbbe3	192.41555	5.633152685	1.21722011	4.62788336	3.69E-06	9.43E-05
ENSDARG00000062902	si:dkey-220f10.4	279.006417	-0.518809492	0.16356775	-3.17183247	0.0015148	0.01363652
ENSDARG00000043816	st6galnac1.2	39.3672561	1.010854925	0.37676554	2.68298139	0.00729691	0.04503714
ENSDARG00000043818	CASKIN2	588.434692	0.486269378	0.18439357	2.63712768	0.00836114	0.04977629
ENSDARG00000061844	si:dkey-38p12.3	1726.54578	0.35665644	0.09537459	3.73953316	0.00018436	0.00259475
ENSDARG00000105255	zbtb4	847.812766	-0.624105841	0.12182345	-5.12303532	3.01E-07	1.08E-05
ENSDARG00000099672	capgb	2486.54137	0.530480389	0.1021914	5.1910471	2.09E-07	7.84E-06
ENSDARG00000089871	jcada	1194.2021	-0.327764405	0.09723534	-3.37083619	0.0007494	0.00791268
ENSDARG00000055075	svila	3217.71622	-0.286026829	0.08103483	-3.52967757	0.00041607	0.00499509
ENSDARG00000018742	psme4b	3439.55554	-0.282110386	0.08306665	-3.39619332	0.0006833	0.00735554
ENSDARG00000021806	zfp36l2	2398.95846	-0.366314707	0.10535334	-3.47701082	0.00050704	0.00583715
ENSDARG00000096712	si:dkey-193p11.2	677.936163	0.796878696	0.24665323	3.23076532	0.00123459	0.01164546
ENSDARG00000109715	sncgb	5752.42846	-0.328833835	0.10089627	-3.25912771	0.00111755	0.01076203
ENSDARG00000073711	mmrn2b	539.328122	1.027713659	0.12634819	8.13398033	4.15E-16	7.88E-14
ENSDARG00000096808	si:dkey-287g12.6	178.469659	-0.841785791	0.19481576	-4.3209327	1.55E-05	0.00032506
ENSDARG00000054030	hoxb5b	1057.77396	0.359889421	0.1301346	2.76551687	0.00568327	0.0374987
ENSDARG00000096637	si:dkey-11c5.11	34.6162082	1.251146798	0.39893256	3.13623639	0.00171131	0.01505119
ENSDARG00000018303	etv4	1039.28349	0.437363586	0.11238875	3.89152477	9.96E-05	0.00155525
ENSDARG00000005134	psmd11b	2010.3098	-0.254201642	0.09229132	-2.75433977	0.00588107	0.0383961
ENSDARG00000045087	cdk5r1b	1696.38199	-0.317386271	0.09784434	-3.24378758	0.00117952	0.01125132
ENSDARG00000096777	si:ch73-81k8.2	21.2344148	1.28054379	0.477973	2.67911324	0.00738174	0.04543495
ENSDARG00000079227	plekhs1	62.8109414	-2.199287088	0.35910138	-6.12441835	9.10E-10	6.06E-08
ENSDARG00000090526	zgc:158404	192.434294	-0.925852751	0.27163363	-3.40846142	0.0006533	0.00712186
ENSDARG00000091836	casp7	583.966835	-1.128656684	0.14231776	-7.93054016	2.18E-15	3.79E-13
ENSDARG00000053862	crygmx	3250.00179	-1.745096227	0.15138385	-11.5276248	9.57E-31	7.44E-28
ENSDARG00000074001	crygmxl2	6731.0178	-0.787961838	0.18424267	-4.27676085	1.90E-05	0.00038464
ENSDARG00000061011	hlfb	316.111895	-0.629316435	0.14100784	-4.46298904	8.08E-06	0.00018653
ENSDARG00000006125	csnk1db	4459.1179	0.291121633	0.0810128	3.59352657	0.00032623	0.0041139
ENSDARG00000026611	socs3b	1867.53237	0.863011361	0.17807653	4.84629481	1.26E-06	3.77E-05
ENSDARG00000076225	tha1	402.066326	0.360983423	0.13506576	2.67264936	0.00752549	0.04603849
ENSDARG00000075621	birc5a	297.046395	-0.850276387	0.1692821	-5.0228369	5.09E-07	1.69E-05
ENSDARG00000100121	si:dkey-21c1.8	92.3368082	0.710152546	0.23542891	3.01642028	0.00255778	0.02060069
ENSDARG00000077081	slc38a10	2098.27386	0.224649639	0.08140931	2.75950804	0.00578885	0.03797355
ENSDARG00000053323	zgc:112285	813.802283	-1.410062716	0.1679763	-8.39441479	4.68E-17	1.03E-14
ENSDARG00000096701	si:dkey-21e13.3	229.846386	-1.038031404	0.15634789	-6.63924137	3.15E-11	2.71E-09

Table S1. DEGs of WT vs. *terfa+/-*

ENSDARG00000060680	si:ch73-127m5.1	1908.91969	0.504241231	0.15714843	3.20869411	0.00133339	0.01236536
ENSDARG00000103516	sec24c	3389.4365	0.44628119	0.08734911	5.10916689	3.24E-07	1.15E-05
ENSDARG00000104442	aatkb	1819.59654	0.413764172	0.10388825	3.98278132	6.81E-05	0.00113317
ENSDARG00000100487	nploc4	1566.29485	-0.430034209	0.10638501	-4.04224442	5.29E-05	0.00092084
ENSDARG00000096736	CR749163.2	7.84572239	-2.432332106	0.78804051	-3.0865572	0.00202489	0.01720049
ENSDARG00000034309	cox10	1391.93233	-0.238936544	0.08955046	-2.66817783	0.00762639	0.04649347
ENSDARG00000086645	hs3st3b1b	1769.89054	-0.233993427	0.08035354	-2.91204879	0.00359067	0.02660091
ENSDARG00000060457	pmp22b	6442.22471	0.36569522	0.12363358	2.95789565	0.00309747	0.02374695
ENSDARG00000060452	sdk2b	1404.55096	0.406019012	0.13009223	3.12100885	0.00180233	0.01564438
ENSDARG00000006413	rpl38	5941.58573	1.177305004	0.12004963	9.80681938	1.05E-22	4.09E-20
ENSDARG00000097080	si:ch73-181m17.1	124.269814	1.703490169	0.27344643	6.22970352	4.67E-10	3.27E-08
ENSDARG00000097826	si:dkey-239b22.2	1784.7565	0.250272566	0.08681843	2.88271239	0.00394267	0.02847868
ENSDARG00000097158	BX294100.1	75.801402	-1.076121983	0.32140366	-3.34819458	0.0008134	0.0084484
ENSDARG00000103490	dpysl4	1921.84874	0.540627818	0.14540771	3.71801336	0.0002008	0.00278038
ENSDARG00000100708	jakmip3	470.642126	-1.251148046	0.12310215	-10.1634946	2.89E-24	1.20E-21
ENSDARG00000099961	bnip3	289.502734	-0.454374558	0.14067356	-3.22999254	0.00123793	0.01166961
ENSDARG00000098785	glrx3	2627.70653	0.288094611	0.09612286	2.99714985	0.00272517	0.02157822
ENSDARG00000103294	si:ch211-221j21.3	364.135301	0.612954221	0.16838842	3.640121	0.00027251	0.00357804
ENSDARG00000117328	FO681472.1	16.2623558	2.193637383	0.65956313	3.32589449	0.00088135	0.00900741
ENSDARG00000104566	foxi1	94.2883439	0.87117508	0.26080167	3.34037392	0.00083666	0.00862867
ENSDARG00000101930	CU914622.1	49.5695239	-4.24613997	1.05850589	-4.01144671	6.03E-05	0.00102107
ENSDARG00000100106	CR385054.1	248.871284	-2.120008254	0.77206515	-2.74589296	0.00603464	0.03908054
ENSDARG00000103650	si:ch73-329n5.1	39.8059066	2.267857725	0.53188851	4.26378401	2.01E-05	0.0004044
ENSDARG00000077461	dhx32a	223.445254	-0.703289489	0.16354387	-4.30031089	1.71E-05	0.0003518
ENSDARG00000005284	fbxl15	283.962591	0.484316015	0.17028565	2.8441387	0.00445317	0.03117481
ENSDARG00000003077	gpx9	162.351961	-1.83018279	0.20393215	-8.97446906	2.85E-19	8.23E-17
ENSDARG00000032578	pax2b	347.932826	0.634079748	0.19025509	3.33278738	0.00085981	0.00882349
ENSDARG00000117474	CABZ01048956.1	31.5752548	2.652144216	0.58825494	4.50849462	6.53E-06	0.00015544
ENSDARG00000113315	zgc:153932	111.28802	-1.023343646	0.25492312	-4.01432257	5.96E-05	0.00101214
ENSDARG00000052779	zgc:153932	189.509459	1.064103593	0.32219469	3.30267263	0.00095768	0.009548
ENSDARG00000092719	AL954655.2	142.102539	-0.537399655	0.18574077	-2.8932778	0.00381244	0.02777203
ENSDARG00000109626	si:ch211-226h7.3	324.715717	-0.580141107	0.21249689	-2.73011573	0.00633121	0.04045301
ENSDARG00000060196	lhpp	404.895921	2.636591822	0.18155479	14.5222925	8.75E-48	3.11E-44
ENSDARG00000104906	mtr	1648.94996	0.365112702	0.1053956	3.46421213	0.00053179	0.00605477
ENSDARG00000087059	selenou1b	43.1795223	0.923955296	0.33711974	2.74073333	0.00613022	0.03954496
ENSDARG00000077760	ascc1	266.575089	1.555948293	0.25116957	6.1948122	5.84E-10	4.02E-08
ENSDARG00000013822	anapc16	731.680952	0.493529172	0.1478882	3.3371776	0.00084634	0.00870324
ENSDARG00000012632	zgc:92749	113.99703	1.115982773	0.25143871	4.43838893	9.06E-06	0.00020573
ENSDARG00000027491	uros	472.777684	-0.425647792	0.13164368	-3.2333326	0.00122355	0.01155885
ENSDARG00000052842	ppifb	1691.83016	0.413745866	0.10872011	3.80560567	0.00014146	0.00208908
ENSDARG00000110727	KCNMA1	152.156364	0.65994425	0.2432376	2.71316703	0.00666435	0.04204521
ENSDARG00000104756	CDHR1	476.090619	-0.518507068	0.15357522	-3.37624167	0.00073483	0.00779526
ENSDARG00000116119	CABZ01097761.1	484.346634	-0.502187719	0.11417607	-4.39836225	1.09E-05	0.00024248
ENSDARG00000096738	si:zfos-911d5.4	11.4427139	2.181991879	0.63795838	3.42027308	0.00062558	0.00688274
ENSDARG00000096716	hmx3b	23.0292771	1.588102934	0.47444479	3.34728709	0.00081607	0.00846118
ENSDARG00000076623	col14a1b	10433.3114	0.420393798	0.13113593	3.20578659	0.00134694	0.01245931
ENSDARG00000059373	dnajc8	2579.32396	0.503390895	0.14002575	3.59498808	0.00032441	0.0040992
ENSDARG00000105003	scn1bb	1731.86824	-0.439179547	0.12059019	-3.64191784	0.00027061	0.00355691
ENSDARG00000002748	sema6d	1130.94831	-0.447958081	0.15178774	-2.95121378	0.00316528	0.02410324
ENSDARG00000042641	cyp51	692.263095	1.324014018	0.12015119	11.0195666	3.08E-28	1.86E-25
ENSDARG00000029043	nup42	303.900894	-0.463021379	0.16172545	-2.86300881	0.00419639	0.02991146
ENSDARG00000018621	slc6a19a.1	284.485769	0.691970265	0.24463277	2.82860817	0.00467509	0.0322776
ENSDARG00000071251	ppp1r18	330.595408	-0.826853099	0.17197885	-4.8078766	1.53E-06	4.42E-05
ENSDARG00000099753	zgc:63863	482.80143	0.658787315	0.15866757	4.15199738	3.30E-05	0.0006127
ENSDARG00000078797	dennd3a	1139.66008	0.385441571	0.10376857	3.71443475	0.00020366	0.00281375
ENSDARG00000079456	si:ch211-149a19.3	221.526291	-0.818088607	0.23153196	-3.53337229	0.00041029	0.00494083
ENSDARG00000074505	mturn	172.914455	-0.572999905	0.17851004	-3.20990292	0.0013278	0.01233274
ENSDARG00000078912	si:ch73-24k9.2	81.2096619	1.528818244	0.32450873	4.7111776	2.46E-06	6.70E-05

Table S1. DEGs of WT vs. *terfa+/-*

ENSDARG00000102318	il6	3.01219017	4.740348393	1.71501854	2.76402166	0.00570937	0.03762103
ENSDARG00000101259	hgh1	316.489804	-0.474747555	0.16701061	-2.84261921	0.00447445	0.0312658
ENSDARG00000008818	hsf1	1034.75481	-0.403028559	0.11415622	-3.53050032	0.00041477	0.00498601
ENSDARG00000035694	stm	6311.60088	-0.57381605	0.13535777	-4.23925472	2.24E-05	0.00044415
ENSDARG00000103618	btr18	52.3105973	-1.332638661	0.38159611	-3.49227525	0.00047892	0.00558304
ENSDARG00000104403	oscp1a	77.1656994	-1.169489624	0.24767463	-4.72187899	2.34E-06	6.39E-05
ENSDARG00000098228	thrap3b	3982.22687	-0.426995605	0.07267268	-5.87559986	4.21E-09	2.42E-07
ENSDARG00000099509	btr23	40.536465	1.009093419	0.37825075	2.66778963	0.0076352	0.04651299
ENSDARG00000099118	TRAPPC9	886.986147	0.890339372	0.11430427	7.78920493	6.74E-15	1.07E-12
ENSDARG00000103106	trappc9	208.763395	0.974191714	0.19469233	5.00374978	5.62E-07	1.83E-05
ENSDARG00000063535	chd4a	11527.2164	0.406144138	0.14934724	2.71946204	0.00653882	0.04145982
ENSDARG00000059109	si:dkeyp-113d7.1	2155.94554	-0.30814913	0.11149296	-2.7638438	0.00571249	0.03763156
ENSDARG00000090268	krtt1c19e	156724.295	0.353333686	0.10458308	3.37849765	0.00072883	0.00776139
ENSDARG00000036832	cyt1l	79323.9532	1.24188781	0.13187526	9.4171405	4.64E-21	1.54E-18
ENSDARG00000036830	krt91	82725.448	0.355568497	0.10986937	3.23628407	0.00121097	0.01147051
ENSDARG00000018259	atp1a3a	20995.6355	0.366889358	0.13099045	2.80088625	0.00509625	0.03453305
ENSDARG00000087674	si:ch1073-127d16.1	84.7043394	-0.77503579	0.2901755	-2.67092082	0.00756435	0.04620592
ENSDARG00000036764	hax1	694.042793	0.488203148	0.13521858	3.61047377	0.00030564	0.00391378
ENSDARG00000007971	cks1b	373.629409	-0.614245628	0.16140128	-3.80570477	0.0001414	0.00208908
ENSDARG00000092878	si:dkeyp-85e10.3	14.4024853	-1.649038977	0.6103135	-2.70195396	0.00689333	0.04321153
ENSDARG00000091951	nuggc.2	21.4400924	-1.459990612	0.48763966	-2.9939948	0.00275351	0.02174719
ENSDARG00000075958	si:dkey-266f7.9	33.2197591	-1.543599637	0.56098537	-2.75158625	0.00593074	0.03859991
ENSDARG00000030326	efna1a	1129.39264	-0.42205134	0.09340955	-4.51828878	6.23E-06	0.00014957
ENSDARG00000058094	ciarta	2642.83299	0.894519846	0.10737131	8.33108808	8.01E-17	1.69E-14
ENSDARG00000062084	clcn1a	200.535371	-0.890788309	0.19904062	-4.47540954	7.63E-06	0.00017799
ENSDARG00000013295	slc2a3a	2116.36659	0.355370536	0.10482824	3.39002663	0.00069886	0.00750654
ENSDARG00000036457	cacng6a	423.956178	-0.825527229	0.16318987	-5.05869173	4.22E-07	1.45E-05
ENSDARG00000063014	dbpa	1187.01544	0.522816286	0.16393321	3.18920294	0.00142666	0.01304917
ENSDARG00000020455	si:ch211-171h4.3	85.547883	-0.874547358	0.28923361	-3.02367132	0.00249728	0.02021159
ENSDARG00000058557	il11b	11.1531782	3.092402006	1.01757358	3.03899597	0.00237368	0.01944318
ENSDARG00000015445	lim2.4	2553.84692	-0.869653207	0.1906681	-4.56108394	5.09E-06	0.00012524
ENSDARG00000096273	si:dkey-3n22.9	71.6835965	1.573763736	0.47035252	3.34592386	0.00082009	0.00848598
ENSDARG00000102004	apoea	14364.632	-0.8347186	0.10224561	-8.16385777	3.24E-16	6.35E-14
ENSDARG00000101029	si:ch73-347e22.4	794.718576	-0.318849271	0.10331507	-3.0861835	0.00202744	0.01720449
ENSDARG00000100396	pip5k1aa	662.19407	-0.605659138	0.13449476	-4.50321738	6.69E-06	0.00015814
ENSDARG00000062820	irgf3	7.08501296	-3.279111197	0.95216877	-3.44383403	0.00057353	0.00642408
ENSDARG00000071116	eepd1	508.892248	-0.444858242	0.12692792	-3.5048099	0.00045693	0.00539017
ENSDARG00000021140	pabpc1b	4771.61004	0.300667322	0.08561541	3.51183631	0.00044502	0.00527721
ENSDARG00000056307	znf706	2740.78523	0.33413071	0.10462556	3.19358579	0.00140518	0.01290674
ENSDARG00000035985	fam210aa	730.149634	-0.318157312	0.09705234	-3.27820346	0.0010447	0.01018276
ENSDARG00000089791	slc25a32a	1080.66019	-0.416545687	0.11853257	-3.51418757	0.0004411	0.00524164
ENSDARG00000102690	rims2b	1335.76349	-0.406145271	0.14761439	-2.75139355	0.00593423	0.03861133
ENSDARG00000104292	ccn2b	301.06066	0.436546873	0.15767734	2.76860884	0.00562962	0.03723368
ENSDARG00000101891	arid1ab	6871.18886	0.388283582	0.11146622	3.48341934	0.00049505	0.00571772
ENSDARG00000070578	edn2	56.0085785	-0.920556743	0.3336088	-2.7593899	0.00579094	0.03797726
ENSDARG00000016999	lin28a	209.076799	0.764638888	0.19599223	3.90137355	9.56E-05	0.00150369
ENSDARG00000062060	gmeb1	1040.1353	0.418684362	0.13769055	3.04076327	0.00235979	0.0193765
ENSDARG00000094436	CR848723.1	31.264619	5.107613972	0.89303807	5.71936871	1.07E-08	5.62E-07
ENSDARG00000004169	stmn1a	1583.40259	-0.449571919	0.15669012	-2.86917856	0.00411539	0.02942615
ENSDARG00000009594	nr1d2b	5301.2565	0.339686074	0.11990888	2.83286829	0.00461324	0.03194819
ENSDARG00000104089	p3h4	588.395099	0.431439263	0.15648831	2.75700635	0.00583332	0.0382048
ENSDARG00000099183	fkbp10a	321.535546	0.492323596	0.15279656	3.22208569	0.00127261	0.01191814
ENSDARG00000116617	spaca4l	4797.07534	0.345167815	0.09621891	3.587318	0.0003341	0.00419181
ENSDARG00000099002	creb5a	90.1448035	0.593255271	0.22019348	2.69424545	0.00705482	0.04390204
ENSDARG00000102501	hoxa5a	249.109749	0.449691219	0.14681173	3.06304685	0.00219096	0.0182681
ENSDARG00000104307	hoxa1a	144.237019	0.6963942	0.24175416	2.88058829	0.00396934	0.02862138
ENSDARG00000036227	npvf	76.9328824	1.421474562	0.39395341	3.60823011	0.00030829	0.00394168
ENSDARG00000033251	osbpl3a	490.98045	0.457886792	0.12106061	3.78229371	0.00015539	0.00225465

Table S1. DEGs of WT vs. *terfa+/-*

ENSDARG00000036168	nfatc1	699.516173	0.534789968	0.11126623	4.80639983	1.54E-06	4.45E-05
ENSDARG00000076820	xkr8.2	214.014553	0.823671435	0.2195451	3.75171854	0.00017563	0.00248729
ENSDARG00000099660	CU915256.1	16.2865052	2.605534029	0.71701276	3.63387398	0.0002792	0.00365171
ENSDARG00000077084	col28a1a	1236.20102	-0.507672774	0.09993057	-5.08025517	3.77E-07	1.32E-05
ENSDARG00000007597	bloc1s4	416.910063	-0.400665725	0.14759581	-2.71461454	0.00663529	0.04188983
ENSDARG00000008859	mylipa	918.798159	-0.419589043	0.10573206	-3.96841828	7.24E-05	0.00118699
ENSDARG00000031751	npr1a	594.123566	0.396259884	0.14963783	2.64812646	0.00809392	0.04855806
ENSDARG00000033599	pdia8	588.571093	0.417524578	0.12339747	3.38357478	0.00071549	0.00764879
ENSDARG00000008832	neu1	865.815829	0.487742387	0.16497928	2.9563858	0.00311267	0.02382677
ENSDARG00000087611	FP085398.1	46.7796207	1.010072314	0.33009465	3.05994754	0.00221376	0.01840848
ENSDARG00000092498	si:dkeyp-46h3.2	7.90985244	2.802594571	0.96548263	2.90279128	0.00369853	0.02716788
ENSDARG00000079276	ube2ql1	1591.9157	0.525412421	0.13590282	3.86608904	0.00011059	0.00170417
ENSDARG00000117259	BX957317.3	11.3447162	-2.37485999	0.67897475	-3.49771475	0.00046926	0.0054913
ENSDARG00000075323	srfbp1	593.196722	-0.293614721	0.1061969	-2.76481438	0.00569552	0.03753968
ENSDARG00000005625	maco1a	830.096926	-0.284285839	0.09767815	-2.91043424	0.00360927	0.02669785
ENSDARG00000035909	mfsd2ab	2024.17705	0.271394828	0.08766969	3.09565166	0.00196381	0.01675609
ENSDARG00000061804	si:ch211-194e15.5	494.237505	-0.36310982	0.12961519	-2.80144496	0.00508743	0.03448272
ENSDARG00000010728	scin	431.288377	-0.467310655	0.13131201	-3.5587807	0.00037258	0.00456632
ENSDARG00000035957	gmnn	311.18393	-0.437206706	0.16209832	-2.69716985	0.00699316	0.04361731
ENSDARG00000070537	stmn2b	4323.10724	0.456592214	0.14011523	3.25869071	0.00111928	0.01077027
ENSDARG00000008920	mrpl53	400.469898	0.544113703	0.12547812	4.33632339	1.45E-05	0.00030648
ENSDARG00000035890	fuca1.1	2788.73331	0.569127109	0.16734604	3.40089975	0.00067164	0.00726446
ENSDARG00000035887	zgc:91944	821.336927	-0.460632995	0.11305314	-4.07448221	4.61E-05	0.00081641
ENSDARG00000035868	spire1a	864.576283	-0.401182497	0.11063877	-3.62605721	0.00028778	0.00373125
ENSDARG00000003320	fam91a1	1534.9031	0.292007589	0.10225254	2.85574907	0.00429354	0.03036017
ENSDARG00000098753	elmo1	3681.14905	-0.243926761	0.08212007	-2.97036723	0.00297444	0.02305979
ENSDARG00000077407	si:dkey-184p18.2	288.022616	-0.771302021	0.21740328	-3.54779378	0.00038847	0.00473076
ENSDARG00000032849	ndrg1a	4641.59429	-0.707512021	0.11489997	-6.15763464	7.38E-10	5.00E-08
ENSDARG00000061446	smap2	144.630093	0.486903953	0.17041378	2.85718652	0.00427415	0.0302649
ENSDARG00000035798	gngt1	1069.46364	-1.346683007	0.43753558	-3.07788226	0.00208477	0.01755319
ENSDARG00000020007	col1a2	102528.191	0.432153639	0.09476393	4.5603176	5.11E-06	0.00012557
ENSDARG00000054848	pdk4	1210.90317	-0.351495388	0.12852542	-2.73483177	0.00624122	0.0400427
ENSDARG00000054804	anp32e	13131.2334	-0.453336509	0.11374514	-3.98554603	6.73E-05	0.00112231
ENSDARG00000099200	zgc:123103	12409.0347	0.291638386	0.10871141	2.68268421	0.00730339	0.0450548
ENSDARG00000018010	crtap	2199.54163	0.455579476	0.12035916	3.78516658	0.00015361	0.00223267
ENSDARG00000020850	eef1a1l1	338101.57	0.376715661	0.07947808	4.73986849	2.14E-06	5.93E-05
ENSDARG00000077572	si:ch211-193k19.2	22.2100528	5.825358202	1.09948944	5.29823932	1.17E-07	4.71E-06
ENSDARG00000016350	cap1	805.43347	0.79242067	0.10501175	7.54601886	4.49E-14	6.20E-12
ENSDARG00000086739	si:ch1073-513e17.1	146.677531	0.945207886	0.28314571	3.33823837	0.00084311	0.00867843
ENSDARG00000027612	gatad1	455.043167	-0.369928424	0.12169589	-3.03977754	0.00236753	0.01942077
ENSDARG00000104082	trps1	883.669761	0.439069727	0.13392542	3.27846444	0.00104374	0.01017734
ENSDARG00000102114	ptdss1b	67.8729312	0.862725883	0.32202765	2.67904286	0.00738329	0.04543495
ENSDARG00000102995	rbm24a	1248.29483	-0.287322586	0.09641655	-2.9800131	0.00288236	0.02248621
ENSDARG00000104478	cap2	1751.46546	0.817721054	0.15184094	5.38537917	7.23E-08	3.08E-06
ENSDARG00000030364	angpt1	449.444422	0.397884339	0.11828801	3.36369126	0.00076908	0.00806556
ENSDARG00000002731	sdc2	3376.93452	-0.419219643	0.08964799	-4.67628604	2.92E-06	7.73E-05
ENSDARG00000078069	rrm2	1756.77368	-0.913944499	0.19164691	-4.76889758	1.85E-06	5.23E-05
ENSDARG00000023472	ctnnb2	17046.0423	0.287523628	0.08735424	3.29146717	0.00099666	0.00984076
ENSDARG00000102618	utp18	192.054329	0.984034712	0.21128512	4.65737824	3.20E-06	8.31E-05
ENSDARG00000098777	CU693379.1	2312.9087	0.314187897	0.09343234	3.36273192	0.00077175	0.00808545
ENSDARG00000101088	si:ch73-359m17.5	73.802852	1.268809036	0.42106847	3.0133081	0.00258416	0.02075263
ENSDARG00000101324	apoa1b	178337.869	-0.352295588	0.08092306	-4.35346346	1.34E-05	0.00028887
ENSDARG00000079795	CABZ01033206.1	329.154816	0.570678731	0.16788965	3.39912994	0.00067601	0.00729893
ENSDARG00000076839	ftr86	581.455135	1.158854448	0.15430999	7.50991187	5.92E-14	8.08E-12
ENSDARG00000090969	cbln18	149.545759	-1.632173275	0.28287871	-5.76986967	7.93E-09	4.30E-07
ENSDARG00000087476	cbln20	393.628402	-1.456758232	0.46089127	-3.16074164	0.00157368	0.01407478
ENSDARG00000069966	alox5b.3	174.781702	-1.886878558	0.6976466	-2.70463377	0.00683797	0.04291864
ENSDARG00000095212	si:ch211-210b2.4	34.2322323	1.455216183	0.52813493	2.75538713	0.00586227	0.03832893

Table S1. DEGs of WT vs. *terfa+/-*

ENSDARG00000069957	zgc:162936	110.261869	0.80250294	0.23250657	3.45152797	0.00055742	0.00628332
ENSDARG00000096856	znf1012	31.8862755	-1.519620593	0.52554594	-2.89150856	0.00383397	0.02788802
ENSDARG00000077301	mlf1	1065.04564	-0.722457706	0.12913686	-5.59451189	2.21E-08	1.06E-06
ENSDARG00000063624	gfm1	2216.68647	0.272611692	0.09933426	2.74438755	0.00606239	0.03922956
ENSDARG00000038879	ift80	822.334625	0.377903629	0.11988047	3.15233679	0.00161969	0.0144292
ENSDARG00000079238	trim59	226.076262	-0.62682939	0.18944575	-3.30875411	0.00093712	0.00938821
ENSDARG00000087489	fam124b	151.836068	0.625236408	0.1944353	3.21565271	0.00130148	0.01212911
ENSDARG00000070019	taf15	10025.575	0.617373591	0.12687198	4.86611438	1.14E-06	3.46E-05
ENSDARG00000059035	porb	2802.00113	0.836332511	0.14385219	5.81383245	6.11E-09	3.41E-07
ENSDARG00000025285	panx1a	803.007907	-0.533434678	0.14244942	-3.74473051	0.00018059	0.00254595
ENSDARG00000057677	neu4	25.2772491	-1.798384482	0.50241721	-3.57946435	0.0003443	0.0042895
ENSDARG00000069888	cldna	227.993062	0.528108408	0.18506702	2.85360626	0.00432261	0.03048763
ENSDARG00000099645	zgc:153184	383.96057	-0.440385179	0.13129164	-3.35425155	0.0007958	0.00829681
ENSDARG00000105503	fndc3a	2330.8472	0.265522656	0.08050092	3.29838043	0.00097244	0.00965644
ENSDARG00000078683	RNF14	1410.56683	1.545285335	0.16198712	9.53955675	1.43E-21	4.95E-19
ENSDARG00000112337	mindy4b	153.191645	-0.595492085	0.18579715	-3.20506571	0.00135032	0.01248126
ENSDARG00000117109	CR626884.6	307.591558	0.894400422	0.27170443	3.29181397	0.00099543	0.00983367
ENSDARG00000104834	ANO7	108.833583	-0.715500234	0.22972919	-3.1145378	0.00184233	0.01589972
ENSDARG00000042823	spcs2	1464.94324	0.426608816	0.0864166	4.93665339	7.95E-07	2.49E-05
ENSDARG00000063475	abcg1	211.516948	-1.000508673	0.1689267	-5.92273869	3.17E-09	1.87E-07
ENSDARG00000043241	arrb1	374.671757	-0.415198997	0.14855002	-2.79501143	0.00518979	0.03499517
ENSDARG00000070038	rbp2a	4021.92004	-0.560979672	0.1993229	-2.81442655	0.00488643	0.03339355
ENSDARG00000097102	si:ch73-281n10.2	11798.8806	-0.365350114	0.10903584	-3.35073418	0.00080598	0.00838212
ENSDARG00000002385	atm	752.221731	0.61579607	0.1419844	4.33706859	1.44E-05	0.00030596
ENSDARG00000100452	nop53	3368.87252	-0.314959684	0.11374026	-2.76911353	0.0056209	0.03720392
ENSDARG00000011488	sirt2	1328.11735	0.299315241	0.10958097	2.73145277	0.00630558	0.04032033
ENSDARG00000018073	mrps22	853.863224	-0.325373977	0.09704169	-3.35292975	0.00079961	0.00832606
ENSDARG00000069675	her8.2	114.700822	-0.731673216	0.22490593	-3.2532411	0.00114097	0.01094494
ENSDARG00000055344	mipep	793.763815	0.444026543	0.10748211	4.13116705	3.61E-05	0.00066205
ENSDARG00000087835	si:ch211-113p18.3	230.475048	-0.797697592	0.14500648	-5.50111672	3.77E-08	1.73E-06
ENSDARG00000062562	egln2	397.819999	0.559785853	0.16744671	3.3430686	0.00082857	0.00855597
ENSDARG00000062577	arhgap35a	2543.25872	0.33007961	0.08748387	3.77303394	0.00016127	0.00232241
ENSDARG00000057504	si:dkey-202g17.3	324.76813	-0.887383797	0.20705456	-4.28574873	1.82E-05	0.00037226
ENSDARG00000038106	slc37a4a	953.936439	0.418920738	0.15035363	2.78623631	0.0053324	0.03570495
ENSDARG00000091042	sacs	970.93018	-0.609099607	0.15203502	-4.00631122	6.17E-05	0.00104068
ENSDARG00000099186	slc1a5	2886.2557	0.522124007	0.08765535	5.95655579	2.58E-09	1.57E-07
ENSDARG00000087018	FO704748.1	173.668504	0.557404097	0.20023626	2.78373207	0.00537374	0.03591715
ENSDARG00000102364	si:dkey-202l22.6	4160.42855	1.093213335	0.14358118	7.61390417	2.66E-14	3.91E-12
ENSDARG00000096950	BX927244.1	20.4651905	-4.930356704	0.74414964	-6.62549097	3.46E-11	2.96E-09
ENSDARG00000097692	si:ch211-116o3.5	612.637716	-0.580377882	0.09990271	-5.80943088	6.27E-09	3.49E-07
ENSDARG00000100455	pnkp	452.989422	-0.362920624	0.11992803	-3.02615337	0.00247687	0.02008565
ENSDARG00000098171		1646.50662	1.199431836	0.10701857	11.2076977	3.74E-29	2.58E-26
ENSDARG00000104960	si:ch211-11n16.3	17.5629263	-2.686564638	0.74721432	-3.59544053	0.00032384	0.00409416
ENSDARG00000101130	coq8b	732.018397	-0.504855501	0.09956282	-5.07072337	3.96E-07	1.38E-05
ENSDARG00000101748	NA	6.03141389	-6.857821146	1.47774997	-4.64071818	3.47E-06	8.90E-05
ENSDARG00000074137	c2cd3	489.352976	-0.33100519	0.11942477	-2.77166267	0.00557708	0.03697634
ENSDARG00000003963	traf4a	1831.98224	0.264684283	0.09225354	2.86909625	0.00411647	0.02942615
ENSDARG00000031562	tada2a	648.936061	-0.355891743	0.12610474	-2.82219161	0.00476967	0.03275769
ENSDARG00000073926	synrg	2048.57511	0.409963947	0.09977367	4.10893909	3.97E-05	0.00071798
ENSDARG00000097404	BX511308.1	7.72958371	2.893419877	0.93139623	3.10654025	0.00189291	0.01625376
ENSDARG00000057805	mrpl28	1373.25092	-0.27695356	0.10253825	-2.70097811	0.00691359	0.04331668
ENSDARG00000098012	itgae.1	161.894075	-1.454253248	0.2138763	-6.79950641	1.05E-11	9.85E-10
ENSDARG00000104200	BX901923.2	2306.4004	0.483338434	0.09987148	4.83960422	1.30E-06	3.88E-05
ENSDARG00000108515	CR388163.2	3.30613675	-4.479184405	1.55336352	-2.88353908	0.00393234	0.02843709
ENSDARG00000014849	pih1d2	97.4252117	-0.81290266	0.23286837	-3.49082469	0.00048153	0.00559798
ENSDARG00000097663	BX324206.3	321.948344	-0.80076941	0.15902548	-5.03547857	4.77E-07	1.60E-05
ENSDARG00000042659	thyn1	109.857579	0.882062628	0.31767796	2.77659372	0.00549318	0.0365648
ENSDARG00000042658	acad8	807.739679	0.760713112	0.11131516	6.83386829	8.27E-12	7.93E-10

Table S1. DEGs of WT vs. *terfa+/-*

ENSDARG00000037402	lim2.3	653.632852	-0.981582871	0.18415461	-5.33021064	9.81E-08	4.02E-06
ENSDARG00000086272	si:dkey-4p15.5	822.57955	-0.555847788	0.14337207	-3.87696017	0.00010577	0.00163839
ENSDARG00000079119	si:ch211-229d2.5	1400.29251	-0.56538575	0.13300884	-4.25073802	2.13E-05	0.00042571
ENSDARG00000023814	dyrk1ab	1960.22843	-0.271487174	0.09594503	-2.82961168	0.00466045	0.03220038
ENSDARG00000042724	supt5h	3857.16775	0.353743262	0.09789691	3.6134264	0.00030218	0.00387745
ENSDARG00000097205	ulk2	2706.72646	0.565714412	0.12370472	4.57310295	4.81E-06	0.00011873
ENSDARG00000056314	a2ml	10603.2614	-0.318836433	0.11420508	-2.79178846	0.00524176	0.03524865
ENSDARG00000041645	sb:cb37	1973.62703	0.408442782	0.09391339	4.34914341	1.37E-05	0.00029309
ENSDARG00000077872	CR626907.1	1144.0637	-0.582769332	0.16067373	-3.62703549	0.00028669	0.00372354
ENSDARG00000097504	CR626907.3	852.624723	1.265137469	0.20964585	6.03464121	1.59E-09	1.02E-07
ENSDARG00000013892	sorl1	3493.68354	0.426448718	0.08476636	5.030872	4.88E-07	1.63E-05
ENSDARG00000062315	sik2b	1858.49813	0.388369023	0.10322169	3.762475	0.00016824	0.00241114
ENSDARG00000056921	ankk1	204.10972	0.51115504	0.18665962	2.73843395	0.00617326	0.03971946
ENSDARG00000069361	spa17	366.419618	-0.484600854	0.16687687	-2.90394253	0.00368496	0.02709752
ENSDARG00000056134	c1qtnf5	357.02182	0.621901172	0.1483726	4.1914825	2.77E-05	0.00052946
ENSDARG00000040565	ckmb	238680.343	0.332086794	0.11401435	2.91267545	0.00358347	0.02658488
ENSDARG00000055965	klc3	102.193823	-0.651705402	0.22469795	-2.90036206	0.00372732	0.02731965
ENSDARG00000069282	bbc3	514.790204	0.826035395	0.14345075	5.75832071	8.50E-09	4.55E-07
ENSDARG00000097770	si:ch211-167j9.4	162.050273	-0.798336692	0.21992406	-3.63005616	0.00028336	0.00369125
ENSDARG00000097871	si:ch1073-145m9.1	73.3782716	1.337606287	0.25431929	5.25955503	1.44E-07	5.66E-06
ENSDARG00000055591	pipox	334.414938	-0.61997161	0.21728272	-2.8532946	0.00432685	0.03050889
ENSDARG00000031814	dhrs13b.1	247.366659	0.748084003	0.16743874	4.46780719	7.90E-06	0.00018305
ENSDARG00000055632	smtnl	849.421107	0.427344644	0.15827629	2.69999156	0.00693412	0.04340987
ENSDARG00000076834	LRRC75A	473.014509	-0.309757163	0.11202121	-2.7651654	0.00568939	0.03751921
ENSDARG00000090481	trarg1a	1348.11065	-0.371308814	0.12747685	-2.91275475	0.00358256	0.02658488
ENSDARG00000075067	aipl1	135.385557	-0.802309735	0.23888834	-3.35851358	0.00078363	0.00819053
ENSDARG00000042727	exo5	238.418254	0.618382187	0.15502563	3.98890286	6.64E-05	0.00110804
ENSDARG00000101859	mnta	1075.14806	0.470011304	0.1135859	4.13793687	3.50E-05	0.0006476
ENSDARG00000055838	mettl16	289.888206	-0.566884508	0.14863606	-3.81390964	0.00013679	0.0020395
ENSDARG00000030913	cluha	4571.04439	0.260043827	0.08898635	2.92228907	0.00347469	0.02593191
ENSDARG00000100826	hif1al	11792.2528	0.405874857	0.07894917	5.14096429	2.73E-07	9.95E-06
ENSDARG00000061383	serpinf2b	2222.83431	-0.35992496	0.11711666	-3.07321747	0.00211764	0.01778103
ENSDARG00000034227	si:dkey-243i1.1	366.044978	-0.942411023	0.16476769	-5.71963482	1.07E-08	5.62E-07
ENSDARG00000090764	bcas3	983.456485	0.573934639	0.09470961	6.05994084	1.36E-09	8.81E-08
ENSDARG00000041141	cryba1a	42.3900823	-2.37949145	0.37771041	-6.2997772	2.98E-10	2.17E-08
ENSDARG00000007655	crybb1l3	76.7556172	-1.392506609	0.32553542	-4.27758859	1.89E-05	0.00038365
ENSDARG00000034453	unc119a	303.554954	-1.216228798	0.19334258	-6.29053781	3.16E-10	2.29E-08
ENSDARG00000026149	slc46a1	368.968142	0.61466068	0.1543923	3.98116159	6.86E-05	0.0011394
ENSDARG00000088440	ssh2a	1491.86431	0.362344422	0.09262577	3.91191787	9.16E-05	0.00144684
ENSDARG00000074967	zgc:162698	1311.57967	0.273849829	0.09448979	2.89819489	0.00375317	0.02744596
ENSDARG00000013804	capns1b	3580.52517	0.309813108	0.10528097	2.94272651	0.00325336	0.02458564
ENSDARG00000025504	gucy2f	159.540984	-1.033874176	0.27490123	-3.76089322	0.00016931	0.00241847
ENSDARG00000019949	serpinh1b	4542.83123	2.532681664	0.13474275	18.7964227	8.08E-79	2.01E-74
ENSDARG00000054543	samsn1a	659.778338	-0.445941736	0.13568772	-3.28652969	0.0010143	0.00998434
ENSDARG00000097157	si:ch211-207n23.2	716.745171	0.612585155	0.18171265	3.371175	0.00074848	0.00790967
ENSDARG00000068965	nrip1a	1427.5422	-0.358498595	0.12540345	-2.85876187	0.00425298	0.03016775
ENSDARG00000097247	BX322555.3	273.775359	1.2955121	0.24207078	5.3517904	8.71E-08	3.63E-06
ENSDARG00000025903	lgals9l1	210.299695	0.727174628	0.21772565	3.33986659	0.00083819	0.00864086
ENSDARG00000054748	cuedc1b	800.434109	-0.466171863	0.11049457	-4.21895726	2.45E-05	0.00047921
ENSDARG00000054744	si:ch211-244e12.7	21.0367843	-1.979442753	0.49023065	-4.03777846	5.40E-05	0.00093226
ENSDARG00000070025	dchs1a	1097.37003	0.374965873	0.13686971	2.7395825	0.00615173	0.03963224
ENSDARG00000086261	fhdc4	376.171745	-0.647860728	0.12917371	-5.01542231	5.29E-07	1.74E-05
ENSDARG00000102626	frem2b	5266.04246	0.56615203	0.11345929	4.98991336	6.04E-07	1.96E-05
ENSDARG00000104489	CR855389.2	40.5934031	3.002007465	0.98824377	3.03771959	0.00238376	0.01950239
ENSDARG00000099764	CR855389.1	90.2609053	1.762119176	0.37755103	4.6672345	3.05E-06	8.00E-05
ENSDARG00000104023	mag	795.949027	-0.545723171	0.18946256	-2.8803747	0.00397203	0.02863247
ENSDARG00000099291	lsr	1568.74584	0.263751831	0.09375467	2.81321267	0.00490492	0.03349225
ENSDARG00000099918	arl4aa	300.364802	-0.683253504	0.13143586	-5.19837961	2.01E-07	7.56E-06

Table S1. DEGs of WT vs. *terfa+/-*

ENSDARG00000075892	bag6	3736.69016	0.19664881	0.07254144	2.71084786	0.00671114	0.04230419
ENSDARG00000068840	zgc:66024	27.2583878	1.405900247	0.44763113	3.14075622	0.00168512	0.01487348
ENSDARG00000033161	sst1.2	197.519166	0.484171008	0.16928579	2.86008069	0.00423533	0.03010271
ENSDARG00000097533	BX571880.1	504.812523	1.089725545	0.23933881	4.55306656	5.29E-06	0.00012947
ENSDARG00000011581	si:dkey-23k10.5	95.7996452	-0.952512345	0.29315243	-3.24920502	0.00115728	0.0110589
ENSDARG00000097653	si:dkey-23k10.3	57.8869379	2.361885614	0.38208416	6.18158467	6.35E-10	4.35E-08
ENSDARG00000089838	si:dkey-262k9.4	136.14766	-0.832124218	0.23640485	-3.51991173	0.00043169	0.00515106
ENSDARG00000100088	zmp:0000001114	2383.42388	0.432084262	0.1093925	3.94985266	7.82E-05	0.00126953
ENSDARG00000077236	hspb6	768.460311	0.933421708	0.21435557	4.35454846	1.33E-05	0.00028794
ENSDARG00000097591	proser3	225.578778	-0.484616727	0.16170135	-2.99698628	0.00272663	0.02158292
ENSDARG00000053950	lin37	410.958009	-0.466758292	0.15868829	-2.94135302	0.00326782	0.02465745
ENSDARG00000097872	si:ch211-137j23.6	3.1051526	-5.279137172	1.59211356	-3.31580441	0.0009138	0.00923645
ENSDARG00000097816	si:dkey-117a8.1	41.463404	-1.519323953	0.48070439	-3.16062012	0.00157434	0.01407559
ENSDARG00000076710	si:dkey-42l23.7	95.7968062	-1.560186133	0.30207871	-5.16483308	2.41E-07	8.87E-06
ENSDARG00000068680	ctrl	1009.62666	-1.996122728	0.59621006	-3.3480192	0.00081391	0.00845022
ENSDARG00000075697	si:ch73-380l3.2	261.371318	-1.419449078	0.21940738	-6.46946833	9.83E-11	7.86E-09
ENSDARG00000096863	si:ch73-380l3.4	11.3133113	3.006524997	0.82979872	3.62319794	0.00029098	0.00376397
ENSDARG00000026784	robo1	4083.548	0.405858313	0.11728007	3.46059051	0.00053899	0.00612278
ENSDARG00000043448	itm2ca	2664.88148	0.357235303	0.12339061	2.89515799	0.00378968	0.02767031
ENSDARG00000043446	efhd1	976.170882	-0.448539103	0.13271558	-3.37970181	0.00072565	0.00773409
ENSDARG00000097824	smco4	585.390852	0.372769437	0.13596318	2.74169397	0.00611233	0.03946022
ENSDARG00000092025	nlrc8	39.6683289	3.913790375	0.98277006	3.98240701	6.82E-05	0.0011342
ENSDARG00000094067	si:dkey-121n8.7	39.591012	1.350343495	0.51086844	2.6432314	0.00821189	0.04905227
ENSDARG00000026704	ftr72	13.9395984	2.290285355	0.70006653	3.27152529	0.00106969	0.01039373
ENSDARG00000016864	farsb	1768.86658	0.361910043	0.11739706	3.08278625	0.00205072	0.01736061
ENSDARG00000025920	tiam1b	951.951256	0.490446731	0.17890911	2.74131776	0.00611933	0.03948493
ENSDARG00000091847	si:ch211-181d7.1	451.772723	-0.796264115	0.20222632	-3.93749007	8.23E-05	0.0013229
ENSDARG00000068621	si:ch211-181d7.3	410.688373	-0.610409131	0.22318836	-2.73495051	0.00623897	0.0400386
ENSDARG00000005425	wdfy1	910.789565	0.312510948	0.11718246	2.66687477	0.00765602	0.04662837
ENSDARG00000101334	ctsc	1340.15984	0.566799197	0.15336548	3.69574157	0.00021925	0.00299251
ENSDARG00000039077	tyr	1040.8317	0.799021851	0.15808788	5.05428923	4.32E-07	1.47E-05
ENSDARG00000003973	chordc1a	231.329308	1.022484536	0.16578481	6.16754054	6.94E-10	4.72E-08
ENSDARG00000040668	lrrc51	26.4295395	1.161724002	0.42069119	2.76146498	0.00575427	0.03780659
ENSDARG00000114883	CU655961.4	10.6919113	5.804241647	1.39161431	4.17086948	3.03E-05	0.00057136
ENSDARG00000091760	cwf19l2	655.96115	0.764140153	0.15374532	4.9701685	6.69E-07	2.14E-05
ENSDARG00000037501	aasdhppt	181.646356	-0.582886491	0.20784799	-2.80438845	0.00504121	0.03423479
ENSDARG00000038378	sagb	3537.95138	-0.998819125	0.29500945	-3.38571905	0.00070992	0.00760563
ENSDARG00000117241	CABZ01067657.1	60.9716142	1.582303838	0.40468315	3.90998202	9.23E-05	0.00145663
ENSDARG00000097973	si:ch1073-190k2.1	727.396804	11.58481372	2.02966172	5.70775593	1.14E-08	5.99E-07
ENSDARG00000088885	si:ch1073-340i21.3	989.051697	2.340050891	0.71659551	3.26551152	0.00109267	0.01056104
ENSDARG00000060439	clcn2c	802.970152	1.494118741	0.18888779	7.91008642	2.57E-15	4.41E-13
ENSDARG00000088463	CLPB	281.356096	0.649662377	0.1723121	3.77026564	0.00016307	0.00234425
ENSDARG00000090232	clpb	646.348514	0.375504104	0.11684629	3.21365886	0.00131055	0.01218623
ENSDARG00000045976	sidt2	3210.92711	0.454734187	0.095129	4.78018465	1.75E-06	4.97E-05
ENSDARG00000097718	stard10	780.190412	-0.352288823	0.10523825	-3.34753578	0.00081533	0.00845791
ENSDARG00000086735	SAMD4B	2332.61076	-0.288679725	0.10100037	-2.85820465	0.00426046	0.03020608
ENSDARG00000077011	uhrf1bp1	601.263158	0.58407251	0.17523733	3.33303699	0.00085904	0.00881922
ENSDARG00000037837	ogfr	1296.34864	-0.27840438	0.09869476	-2.82086276	0.00478947	0.03287351
ENSDARG00000027658	irf10	31.3025216	1.288213233	0.48675352	2.64654119	0.00813196	0.04868012
ENSDARG00000077400	rbm10	922.288226	-0.603774195	0.15006644	-4.02337933	5.74E-05	0.00098113
ENSDARG00000087641	cdh26.2	695.601554	-0.387048454	0.11485041	-3.3700223	0.00075162	0.00792935
ENSDARG00000078404	cdh26.1	753.57747	-0.478566084	0.14780905	-3.23773196	0.00120484	0.01143236
ENSDARG00000117260	CABZ01079870.1	36.3274473	4.736715434	0.62652285	7.56032354	4.02E-14	5.65E-12
ENSDARG00000117249	CABZ01079872.1	5.95479483	3.866087707	1.45077814	2.66483731	0.00770256	0.04686587
ENSDARG00000117527	CABZ01079873.1	18.7382552	7.538781034	1.26520254	5.95855668	2.54E-09	1.56E-07
ENSDARG00000100222	utrn	6250.03229	0.456590192	0.09223017	4.95055135	7.40E-07	2.34E-05
ENSDARG00000061024	rabggta	1081.08354	0.367815345	0.10660752	3.45018199	0.00056021	0.0063033
ENSDARG00000006288	prdm5	346.872763	0.672435499	0.13599582	4.94453068	7.63E-07	2.40E-05

Table S1. DEGs of WT vs. *terfa+/-*

ENSDARG00000019774	zhx3	896.286312	0.386261658	0.09043155	4.27131526	1.94E-05	0.00039204
ENSDARG00000074790	rab5if	2506.18437	0.435588921	0.10093389	4.31558653	1.59E-05	0.00033136
ENSDARG00000017121	mafba	1530.58005	-0.311952554	0.09776376	-3.19088144	0.00141839	0.01299455
ENSDARG00000105341	si:dkey-9l20.3	4265.25371	1.1005781	0.14531664	7.57365517	3.63E-14	5.15E-12
ENSDARG00000020699	slc9a8	542.191235	0.327896133	0.11940259	2.74613909	0.00603012	0.03906141
ENSDARG00000013729	srsf6a	5420.70146	0.47285238	0.1167828	4.04898986	5.14E-05	0.00089721
ENSDARG00000002483	wnt7ab	159.306174	-0.695899749	0.1993851	-3.49022948	0.00048261	0.00560784
ENSDARG00000045776	cnbpa	5874.4644	-0.372848784	0.09186355	-4.058724	4.93E-05	0.00086365
ENSDARG00000011400	tnnc1a	237.139999	0.446493902	0.16904443	2.64128128	0.00825931	0.04928816
ENSDARG00000117500	FO904870.1	24.2937978	-2.01321071	0.53628656	-3.75398312	0.00017405	0.00246914
ENSDARG00000117613	CABZ01030033.1	24.9437053	-1.780109848	0.47936597	-3.71346725	0.00020444	0.00282296
ENSDARG00000098308	CR936540.1	505.725265	0.562695582	0.1491341	3.77308476	0.00016124	0.00232241
ENSDARG00000075295	tulp1a	724.584872	-1.362396329	0.37671827	-3.61648599	0.00029863	0.00383985
ENSDARG00000040523	smpd2a	518.16386	-0.363038922	0.13449791	-2.69921616	0.0069503	0.04346156
ENSDARG00000037804	phlda3	785.300931	1.025566129	0.13309261	7.70565788	1.30E-14	2.02E-12
ENSDARG00000063230	bmp7b	445.827775	-0.353421802	0.12661882	-2.79122652	0.00525087	0.03529216
ENSDARG00000013963	mipb	1466.89749	-2.859029446	0.20912992	-13.6710682	1.51E-42	3.76E-39
ENSDARG00000089626	ptges3b	2745.87894	-0.304311433	0.10715365	-2.83995404	0.004512	0.03143101
ENSDARG00000110967	FP102169.1	21.3899593	2.753362983	0.57207542	4.81293707	1.49E-06	4.32E-05
ENSDARG00000090179	si:ch211-117c9.1	40.0975336	-1.272683242	0.37889891	-3.35889922	0.00078254	0.00818353
ENSDARG00000075948	slco4a1	261.988286	-0.481286799	0.17720858	-2.715934	0.00660891	0.04177631
ENSDARG00000101120	kif3b	954.303685	0.27568484	0.10409571	2.64837858	0.00808789	0.04854492
ENSDARG00000025850	rps21	19451.2118	0.600517329	0.09595329	6.2584338	3.89E-10	2.75E-08
ENSDARG00000058543	lama5	10410.9085	0.516605716	0.11251111	4.59159744	4.40E-06	0.00011021
ENSDARG00000058381	zgc:171775	1819.73092	-0.37888671	0.09649358	-3.92654846	8.62E-05	0.00137386
ENSDARG00000058371	krt5	146410.295	0.474013935	0.07623853	6.21751133	5.05E-10	3.52E-08
ENSDARG00000058358	krt8	40898.5191	0.467172573	0.10200052	4.58010006	4.65E-06	0.0001154
ENSDARG00000018404	krt18a.1	11346.0546	0.357106954	0.09705197	3.67954358	0.00023365	0.00314936
ENSDARG00000093320	BX323028.1	13.450547	4.117839782	0.93357581	4.41082526	1.03E-05	0.00023059
ENSDARG00000075842	pigt	160.051042	-0.597850442	0.19216499	-3.11113087	0.00186372	0.01605088
ENSDARG00000061445	adcy6a	1187.35518	0.433813914	0.13411004	3.23476095	0.00121745	0.01152309
ENSDARG00000058082	birc7	215.455886	-1.507163007	0.2159943	-6.97779081	3.00E-12	3.08E-10
ENSDARG00000095139	CR388373.2	75.0314298	-1.55204713	0.26272329	-5.90753544	3.47E-09	2.03E-07
ENSDARG00000077762	si:rp71-79p20.2	177.644743	0.609478869	0.19087986	3.1929973	0.00140804	0.0129283
ENSDARG00000013838	sulf2b	6045.76564	0.282691601	0.10510037	2.68972994	0.00715099	0.04437897
ENSDARG00000075539	snphb	481.143105	-0.569814716	0.16254641	-3.50555093	0.00045566	0.00538285
ENSDARG00000012513	sdcbp2	5339.47465	0.266038421	0.08570765	3.10402203	0.00190909	0.01637352
ENSDARG00000115843	si:rp71-1k13.6	193.134032	0.673930149	0.17957608	3.75289476	0.0001748	0.00247706
ENSDARG00000092166	si:dkey-147f3.8	405.737334	0.703036873	0.18546152	3.79074245	0.0001502	0.00219727
ENSDARG00000004836	dnajc5ab	3836.10206	-0.27701673	0.08788899	-3.15189356	0.00162215	0.01444595
ENSDARG00000011049	slc17a9b	417.35369	0.572598539	0.12024722	4.76184442	1.92E-06	5.39E-05
ENSDARG00000008263	mfsd4ab	160.480001	-1.012579156	0.18867505	-5.36678894	8.02E-08	3.37E-06
ENSDARG00000017659	sephs2	2708.59744	0.285176736	0.09719941	2.9339349	0.00334694	0.02514001
ENSDARG00000025581	rpl10	45720.5557	0.321308722	0.09422827	3.40989713	0.00064987	0.00709693
ENSDARG00000074201	flna	17437.5412	0.791432036	0.13004595	6.08578754	1.16E-09	7.60E-08
ENSDARG00000037432	haus7	302.017106	-0.566183827	0.15789183	-3.58589685	0.00033592	0.00421038
ENSDARG00000070857	si:dkey-32e6.6	157.263058	-0.546925208	0.20592324	-2.65596635	0.00790815	0.04778922
ENSDARG00000037539	tnnc1b	6507.13524	0.460060529	0.0974485	4.72106302	2.35E-06	6.40E-05
ENSDARG00000006019	tktb	11874.3095	0.35812076	0.07085004	5.05463006	4.31E-07	1.47E-05
ENSDARG00000070835	tnnc2	125241.91	0.4419197	0.0975718	4.52917429	5.92E-06	0.00014248
ENSDARG00000070834	taf13	904.915495	-0.336115459	0.11186393	-3.00468133	0.00265859	0.02119965
ENSDARG00000037517	slc35c2	3364.7792	0.363689044	0.08651459	4.20378844	2.62E-05	0.00050789
ENSDARG00000070770	her4.3	53.7055981	2.028115826	0.42891494	4.7284803	2.26E-06	6.21E-05
ENSDARG00000056729	her4.2	137.067406	1.348539976	0.24743382	5.45010379	5.03E-08	2.21E-06
ENSDARG00000044982	dhrs3a	572.789778	0.815106114	0.24694548	3.30075339	0.00096426	0.00959431
ENSDARG00000074331	eif4g3b	2320.02183	0.325484653	0.11153096	2.91833465	0.00351906	0.02621893
ENSDARG00000001437	slc2a1a	195.988795	-0.802649965	0.23522095	-3.41232341	0.00064412	0.00704954
ENSDARG00000077875	slc2a5	54.2039259	2.217800224	0.47850122	4.63488938	3.57E-06	9.13E-05

Table S1. DEGs of WT vs. *terfa+/-*

ENSDARG00000056499	ca6	3616.75837	-0.209842886	0.07917084	-2.65050736	0.0080371	0.04831005
ENSDARG00000079388	agrn	7183.77459	0.430129826	0.09312582	4.61880332	3.86E-06	9.80E-05
ENSDARG00000094508	CR925709.2	36.4246391	7.294636381	1.25770587	5.79995415	6.63E-09	3.66E-07
ENSDARG00000022560	clcnk	129.543049	-0.793601921	0.2232802	-3.55428697	0.00037901	0.00463592
ENSDARG00000003475	sult1st5	63.7084738	1.337891494	0.36791512	3.63641342	0.00027646	0.00362226
ENSDARG00000061890	atp13a2	561.556315	0.93889168	0.11306916	8.30369362	1.01E-16	2.11E-14
ENSDARG00000023648	idh3g	2544.25026	0.259291658	0.08327698	3.11360539	0.00184816	0.01594451
ENSDARG00000092807	si:dkey-151g10.6	44184.6931	0.668490717	0.0967501	6.90945752	4.87E-12	4.82E-10
ENSDARG00000029146	lrp1ab	9168.00625	0.818052248	0.133519	6.12686028	8.96E-10	5.99E-08
ENSDARG00000030650	r3hdml	62.7423483	-0.929145324	0.28051953	-3.31223047	0.00092555	0.00932267
ENSDARG00000077882	ccn5	66.2173882	0.91524527	0.28693472	3.18973345	0.00142404	0.01303186
ENSDARG00000104197	ptpn22	267.234568	0.545422193	0.17502954	3.1161722	0.00183215	0.01582285
ENSDARG00000089429	si:dkey-205h13.2	2053.00806	-0.616608813	0.13572564	-4.54305336	5.54E-06	0.00013497
ENSDARG00000014386	galnt6	1095.03959	0.738928481	0.09235568	8.00089903	1.24E-15	2.24E-13
ENSDARG00000037284	ptges3a	2295.82882	0.244699676	0.08891895	2.75194054	0.00592433	0.03858731
ENSDARG00000037285	mipa	1988.89942	-1.807061955	0.28827253	-6.26858873	3.64E-10	2.59E-08
ENSDARG00000094977	si:ch211-156j22.4	488.87815	-0.635573229	0.2235627	-2.84293052	0.00447008	0.03125285
ENSDARG00000037071	rps26	8762.50515	0.49060443	0.11043842	4.44233456	8.90E-06	0.00020236
ENSDARG00000007988	masp2	628.115072	0.768402582	0.17647298	4.35422235	1.34E-05	0.00028812
ENSDARG00000037017	ube4b	3145.15344	0.248777934	0.09297216	2.67583256	0.00745439	0.04571647
ENSDARG00000039931	slc25a33	1343.6699	0.614534156	0.13613562	4.51413206	6.36E-06	0.00015223
ENSDARG00000074985	aurkaip1	1424.82198	0.274655982	0.10381542	2.64561845	0.00815417	0.0487896
ENSDARG00000053820	pcmtd2a	255.033163	-0.516117162	0.18709182	-2.75863024	0.00580442	0.03803554
ENSDARG00000078244	si:ch211-197l9.2	493.330127	-0.610960352	0.13553474	-4.50777675	6.55E-06	0.00015581
ENSDARG00000060484	selenoi	598.476574	0.3813014	0.11957247	3.18887276	0.00142829	0.0130515
ENSDARG00000036754	hmgn3	2475.50183	-0.665349557	0.13809582	-4.81802816	1.45E-06	4.23E-05
ENSDARG00000036876	zgc:153284	404.439998	0.623551737	0.16767603	3.71878872	0.00020018	0.00277495
ENSDARG00000036875	rps12	3178.11626	1.006623357	0.28908385	3.48211546	0.00049747	0.0057403
ENSDARG00000025147	cd63	2583.31813	0.358656571	0.12493285	2.87079471	0.00409441	0.02934317
ENSDARG00000000796	nr4a1	1920.70971	0.5497951	0.13975409	3.9340181	8.35E-05	0.00133526
ENSDARG00000060626	dgkaa	2035.34022	0.296261092	0.08134144	3.64219144	0.00027033	0.00355501
ENSDARG00000036831	rnf41	1363.28842	0.529376908	0.11097679	4.77015868	1.84E-06	5.20E-05
ENSDARG00000079525	slc39a5	493.786621	0.943300287	0.18115561	5.20712699	1.92E-07	7.24E-06
ENSDARG00000095268	si:dkey-261h17.1	3102.1003	-0.256647843	0.09013688	-2.84731223	0.00440901	0.03093027
ENSDARG00000011570	pou6f1	1096.31023	-0.374026648	0.13513613	-2.76777684	0.00564401	0.03729908
ENSDARG00000007867	dazap2	3349.85351	-0.357887298	0.08944569	-4.00116887	6.30E-05	0.00105951
ENSDARG00000092856	si:ch211-210c8.7	433.911221	0.384921384	0.1393999	2.76127443	0.00575763	0.03780867
ENSDARG00000089004	si:ch211-207e14.4	341.079848	-0.506015395	0.18417731	-2.74743614	0.00600632	0.03893769
ENSDARG00000105452	plxna1a	2050.08125	0.636340223	0.14990756	4.24488414	2.19E-05	0.00043488
ENSDARG00000061272	BX294434.1	2075.7499	0.35974177	0.09906041	3.6315394	0.00028174	0.00367394
ENSDARG00000017525	ccnt1	688.984764	0.404397517	0.12068959	3.35072414	0.00080601	0.00838212
ENSDARG00000001889	tuba1a	16875.2867	1.030287973	0.1433531	7.18706473	6.62E-13	7.76E-11
ENSDARG00000036695	calcoco1a	2115.68415	-0.314533612	0.08085637	-3.8900289	0.00010023	0.00156291
ENSDARG00000070340	hoxc5a	465.167457	0.405257301	0.14897098	2.72037749	0.00652074	0.04138024
ENSDARG00000070338	hoxc4a	545.528949	0.483404353	0.11943517	4.04742046	5.18E-05	0.00090198
ENSDARG00000094560	BX005254.2	33.1296357	3.568078212	0.50040152	7.13043047	1.00E-12	1.14E-10
ENSDARG00000036675	hnrnpa1b	10036.1422	0.566039768	0.14341466	3.94687535	7.92E-05	0.00128267
ENSDARG00000063594	hipk1a	309.436404	1.130342121	0.28721297	3.93555389	8.30E-05	0.00132875
ENSDARG00000044325	zgc:193690	100.523237	-1.330090306	0.26239635	-5.06901224	4.00E-07	1.39E-05
ENSDARG00000111188	cdaa	129.994853	-0.993904258	0.25650462	-3.87480054	0.00010671	0.00165014
ENSDARG00000061240	cbln4	74.4165807	1.0062131	0.315789	3.18634625	0.00144082	0.01313223
ENSDARG00000095280	CU682811.3	134.572106	1.80270062	0.27386015	6.58255898	4.62E-11	3.91E-09
ENSDARG00000088123	znf217	1146.31854	-0.410417245	0.11713161	-3.50389834	0.0004585	0.00540034
ENSDARG00000044485	sall4	760.311522	0.369039034	0.11847854	3.11481746	0.00184059	0.01589017
ENSDARG00000115869	ATP9A	335.657378	0.498835151	0.15099726	3.30360403	0.00095451	0.00952289
ENSDARG00000099038	kcng1	199.221107	0.711992679	0.18560426	3.8360795	0.00012501	0.00188774
ENSDARG00000104435	si:ch73-217b7.1	750.86598	-0.306969528	0.10167842	-3.01902332	0.00253591	0.02045766
ENSDARG00000054641	tent5ab	349.41324	-0.993784103	0.23853669	-4.16616882	3.10E-05	0.00058089

Table S1. DEGs of WT vs. *terfa+/-*

ENSDARG00000027495	elovl4b	2374.39837	-1.000231139	0.2836133	-3.52674266	0.00042071	0.00503934
ENSDARG00000086856	stk35	852.191367	0.631714021	0.14019685	4.50590737	6.61E-06	0.00015663
ENSDARG00000087798	pdyn	16.4002766	2.295433741	0.61655382	3.72300625	0.00019686	0.00273968
ENSDARG00000102356	scp2b	1081.31884	-0.370245474	0.08726302	-4.24286806	2.21E-05	0.00043775
ENSDARG00000102805	cyp2aa12	49.3771436	-0.952949075	0.35989166	-2.64787766	0.00809988	0.0485815
ENSDARG00000103590	cyp2aa6	463.589128	-0.695237669	0.20721375	-3.35517156	0.00079316	0.00827621
ENSDARG00000104540	cyp2aa8	1634.88143	0.504992817	0.1406564	3.59025843	0.00033035	0.00414899
ENSDARG00000103456	cyp2aa11	58.831761	1.985200881	0.41388854	4.7964626	1.61E-06	4.65E-05
ENSDARG00000098890	cyp2aa9	591.252084	1.110267149	0.15513521	7.15677069	8.26E-13	9.59E-11
ENSDARG00000104811	cyp2aa2	148.178407	1.218371604	0.19688159	6.18834698	6.08E-10	4.17E-08
ENSDARG00000103347	cyp2aa3	777.236917	-0.269837791	0.10002107	-2.69780958	0.00697974	0.04356638
ENSDARG00000077571	zgc:174862	38.3161412	3.434075572	0.58549511	5.86525068	4.48E-09	2.55E-07
ENSDARG00000103725	znf341	411.527193	0.609320609	0.13617818	4.47443658	7.66E-06	0.00017814
ENSDARG00000088882	si:ch211-149b19.2	103.013791	-0.725032697	0.23738264	-3.05427848	0.00225603	0.01867872
ENSDARG00000012125	cnga1b	158.320561	-0.74299216	0.22588342	-3.28927271	0.00100447	0.00990087
ENSDARG00000070127	nfxl1	1112.11253	0.688319503	0.11899243	5.78456546	7.27E-09	3.98E-07
ENSDARG00000088091	pfn1	41554.0515	0.591151089	0.09441656	6.26109547	3.82E-10	2.71E-08
ENSDARG00000116713	si:ch73-265d7.2	500.922359	0.756056835	0.18880082	4.00452088	6.21E-05	0.00104717
ENSDARG00000095832	CU682777.2	503.957563	-0.711489475	0.17290502	-4.11491511	3.87E-05	0.00070269
ENSDARG00000054211	st8sia7.1	149.232982	-1.15038453	0.18896224	-6.08790705	1.14E-09	7.52E-08
ENSDARG00000054207	CU682777.1	42.7548719	-1.241619904	0.41938139	-2.9605985	0.00307042	0.02357592
ENSDARG00000076317	plod3	4433.24785	0.530123013	0.09133203	5.80434961	6.46E-09	3.57E-07
ENSDARG00000095861	si:ch1073-441p17.1	6.79117224	4.066058867	1.26723495	3.20860694	0.0013338	0.01236536
ENSDARG00000039901	sh3gl2b	265.628412	0.709165351	0.22198423	3.19466547	0.00139993	0.01286807
ENSDARG00000039900	si:ch73-168d20.1	7.62267487	5.90096884	1.4352481	4.11146256	3.93E-05	0.00071173
ENSDARG00000099425	CABZ01073265.1	3587.7991	0.429777875	0.11291893	3.80607449	0.00014119	0.00208761
ENSDARG00000036897	sgms2b	221.397964	0.553224145	0.15907616	3.47773137	0.00050568	0.00582418
ENSDARG00000100797	CABZ01076996.1	560.729304	0.463406334	0.14024627	3.3042328	0.00095237	0.00950709
ENSDARG00000077799	egr4	375.812785	-1.117095297	0.19777275	-5.64837829	1.62E-08	8.15E-07
ENSDARG00000100075	abcg2a	1312.97647	0.519434832	0.15266881	3.40236373	0.00066806	0.00723825
ENSDARG00000101407	tgm1l4	124.903063	2.173771596	0.27514594	7.9004313	2.78E-15	4.67E-13
ENSDARG00000100353	si:ch1073-398f15.1	551.989912	-0.492710946	0.14189788	-3.47229259	0.00051603	0.00590788
ENSDARG00000100789	plgrkt	769.07707	-0.309837617	0.10903849	-2.84154354	0.00448957	0.03132743
ENSDARG00000011921	txnl1	2518.02321	-0.257840034	0.09663841	-2.66809049	0.00762837	0.04649415
ENSDARG00000089844	scarb2c	720.435534	0.469544308	0.13839367	3.39281635	0.00069178	0.00743372
ENSDARG00000003462	fech	167.026194	0.931760132	0.24211531	3.84841473	0.00011888	0.00181059
ENSDARG00000100274	WDR7	2096.54446	0.440653002	0.13687787	3.21931517	0.00128497	0.01202031
ENSDARG00000098176	si:rp71-1h20.5	137.19277	-0.577365036	0.21302119	-2.71036435	0.00672093	0.04235517
ENSDARG00000102321	si:ch73-299h12.1	158.034424	-0.547677346	0.17658052	-3.10157293	0.00192495	0.01648114
ENSDARG00000101790	si:ch73-299h12.3	156.845838	-0.604651927	0.19290231	-3.13449807	0.00172148	0.0151246
ENSDARG00000103795	zgc:113348	430.13034	-0.403172072	0.14341858	-2.81115644	0.00493638	0.03365158
ENSDARG00000107858	CABZ01073424.1	245.099214	0.570520956	0.20283567	2.81272497	0.00491236	0.03351546
ENSDARG00000098909	SHC3	283.086262	0.666300181	0.19618884	3.39621856	0.00068324	0.00735554
ENSDARG00000022218	uck1	237.584049	-0.69024745	0.14497259	-4.76122728	1.92E-06	5.40E-05
ENSDARG00000029905	phyhd1	864.431683	-0.362264765	0.1252017	-2.89344933	0.00381036	0.02777203
ENSDARG00000004187	zgc:122979	610.019079	0.993602529	0.18514573	5.36659693	8.02E-08	3.37E-06
ENSDARG00000034291	rpl37	15417.9052	0.540603087	0.09861101	5.48217791	4.20E-08	1.89E-06
ENSDARG00000099416	ly6m3	23.7689155	5.706442016	1.18804497	4.80322054	1.56E-06	4.51E-05
ENSDARG00000017602	ccng2	1365.84871	-0.301570549	0.09969982	-3.02478524	0.0024881	0.02015699
ENSDARG00000096310	wu:fj64h06	407.593234	0.615787975	0.12176156	5.0573266	4.25E-07	1.46E-05
ENSDARG00000086746	prodha	1254.94741	0.548000769	0.13385764	4.09390731	4.24E-05	0.00075737
ENSDARG00000090818	CACFD1	415.763752	0.535887188	0.15281479	3.50677576	0.00045357	0.00536379
ENSDARG00000044808	slc4a4b	1375.56977	0.346079224	0.12854908	2.69219535	0.00709834	0.04411831
ENSDARG00000093173	si:dkey-93m18.6	20.3851897	-5.84519942	1.1533788	-5.06789218	4.02E-07	1.39E-05
ENSDARG00000092664	si:dkey-93m18.3	9.07687634	-1.873214933	0.70833301	-2.64453994	0.00818021	0.04891003
ENSDARG00000093667	BX005283.1	18.6100236	-2.643695847	0.53850574	-4.90931789	9.14E-07	2.83E-05
ENSDARG00000023362	nr5a1b	30.7905011	1.302502493	0.40468057	3.21859406	0.00128821	0.01204532
ENSDARG00000060106	crb2a	446.08044	0.338296149	0.12636047	2.67723089	0.00742335	0.04560239

Table S1. DEGs of WT vs. *terfa+/-*

ENSDARG00000069980	lman1	1168.40427	0.463544071	0.10104787	4.58737097	4.49E-06	0.0001122
ENSDARG00000059978	cplx4a	1433.24018	-0.991568015	0.21476952	-4.61689352	3.90E-06	9.87E-05
ENSDARG00000075191	oacyl	230.534627	0.526876597	0.15517833	3.39529743	0.00068554	0.00737305
ENSDARG00000100709	agxt2	727.683431	1.997774077	0.22561075	8.85495947	8.37E-19	2.31E-16
ENSDARG00000017490	cel.1	899.234142	-1.496959983	0.53456961	-2.80030878	0.00510537	0.0345651
ENSDARG00000034048	zgc:92275	195.152016	-1.059429463	0.18899562	-5.60557673	2.08E-08	1.01E-06
ENSDARG00000058740	ube2r2	694.324172	-0.302598287	0.11073179	-2.73271382	0.00628149	0.04021911
ENSDARG00000088318	ubap2a	8557.8684	0.389669563	0.12309281	3.16565664	0.00154733	0.01388912
ENSDARG00000026787	aqp7	258.032602	-0.947260091	0.19795682	-4.78518551	1.71E-06	4.86E-05
ENSDARG00000070045	celf4	1804.66197	0.571277935	0.16742377	3.41216746	0.00064449	0.00705047
ENSDARG00000117235	CR293531.4	6.57178375	6.551318156	1.39571992	4.69386304	2.68E-06	7.20E-05
ENSDARG00000094455	CR293531.1	5.06309364	3.951286875	1.4859429	2.65911084	0.00783472	0.04743774
ENSDARG00000113932	LO018011.1	11.5932286	2.037394634	0.73145661	2.78539371	0.00534628	0.03577859
ENSDARG00000074894	myorg	2240.63717	-0.286402174	0.10165492	-2.81739601	0.00484148	0.03314122
ENSDARG00000012126	zgc:109965	710.741805	-0.613715928	0.22583068	-2.71759324	0.00657586	0.04159915
ENSDARG00000028900	odf2a	308.864272	-0.406259176	0.1420877	-2.85921417	0.00424692	0.03013337
ENSDARG00000044776	clic3	79.3279672	1.37444619	0.2621835	5.24230615	1.59E-07	6.13E-06
ENSDARG00000062970	niban2a	1348.54241	0.404135209	0.09691955	4.16980086	3.05E-05	0.00057318
ENSDARG00000095592	si:dkey-11o15.5	26.954057	2.184239148	0.44064682	4.95689305	7.16E-07	2.28E-05
ENSDARG00000044751	ddt	1253.28972	-0.35186495	0.11341757	-3.10238474	0.00191968	0.01644733
ENSDARG00000044755	GCNT4	176.88348	-0.570981481	0.17602521	-3.24374828	0.00117968	0.01125132
ENSDARG00000057936	lhx5	902.648652	0.50087663	0.16940349	2.95670786	0.00310943	0.02382428
ENSDARG00000005122	atp2a2b	7067.90048	0.450984682	0.07831463	5.75862658	8.48E-09	4.55E-07
ENSDARG00000079286	bcr	2853.81376	0.282272665	0.10494079	2.68982782	0.00714889	0.04437703
ENSDARG00000057556	rpl17	37054.7691	0.32989778	0.07992599	4.12754094	3.67E-05	0.0006701
ENSDARG00000043339	abraxas1	208.664208	-0.526369654	0.17293481	-3.0437461	0.00233652	0.01922989
ENSDARG00000063634	hpse	247.061508	-0.524191659	0.15499812	-3.38192264	0.0007198	0.00767843
ENSDARG00000079104	mfhas1	802.015753	-0.382600057	0.10432596	-3.66735236	0.00024507	0.00327844
ENSDARG00000029832	slc26a1	444.207912	-0.681353304	0.13893755	-4.9040256	9.39E-07	2.90E-05
ENSDARG00000011671	pde6b	476.021608	-0.727353594	0.21125927	-3.44294285	0.00057542	0.00642827
ENSDARG00000078113	atp5mea	988.544376	-0.365959701	0.12809699	-2.85689537	0.00427807	0.03027659
ENSDARG00000007671	ghrb	440.767548	-0.592126488	0.16696116	-3.54649252	0.0003904	0.00474721
ENSDARG00000093052	c6	166.264772	1.059673599	0.30138518	3.51601093	0.00043808	0.00521233
ENSDARG00000057121	c7b	261.741988	1.057937727	0.2253782	4.69405529	2.68E-06	7.20E-05
ENSDARG00000062307	ccdc61	434.129335	-0.507128145	0.14402907	-3.5210124	0.0004299	0.00513713
ENSDARG00000057039	si:ch211-191j22.8	32.8340276	-1.211823623	0.42459771	-2.85405124	0.00431656	0.0304536
ENSDARG00000006604	nectin3b	587.85731	0.421248826	0.11091866	3.79781746	0.00014598	0.00214181
ENSDARG00000075161	defbl1	1542.15996	-0.448688631	0.15092305	-2.97296283	0.0029494	0.02290856
ENSDARG00000076169	pold3	849.348598	0.341803972	0.12712606	2.6887011	0.00717306	0.04448265
ENSDARG00000010047	neu3.2	255.261075	-0.790637552	0.20636072	-3.83133734	0.00012745	0.00192217
ENSDARG00000044632	myo7ab	462.7481	-0.440273816	0.1223472	-3.59856056	0.00031998	0.00406189
ENSDARG00000079293	gdpd4b	189.214631	-0.572697181	0.17372396	-3.29659291	0.00097865	0.00971034
ENSDARG00000044619	birc2	3334.57997	0.225838928	0.08084303	2.79354857	0.00521332	0.03511574
ENSDARG00000103687	sycn.2	188.470999	-2.282959363	0.6333613	-3.60451351	0.00031274	0.00398009
ENSDARG00000042970	gng8	98.2668653	-1.076102639	0.25269267	-4.25854316	2.06E-05	0.00041311
ENSDARG00000056239	tmem45b	1179.00992	0.634843791	0.12828423	4.9487281	7.47E-07	2.36E-05
ENSDARG00000073707	cnksr2b	839.575105	0.472797068	0.17393074	2.71830649	0.0065617	0.04153071
ENSDARG00000020811	efemp2b	383.518347	0.374880179	0.1399895	2.67791643	0.00740817	0.04553995
ENSDARG00000094217	si:dkey-17e16.15	259.912662	-1.171259061	0.202608	-5.78091216	7.43E-09	4.05E-07
ENSDARG00000069497	sumf2	196.792141	0.511463438	0.16737166	3.05585447	0.0022442	0.01860793
ENSDARG00000091669	bicdl2l	847.054463	0.960513594	0.1373527	6.99304486	2.69E-12	2.79E-10
ENSDARG00000009215	zgc:112437	265.738729	-0.911021309	0.18907585	-4.818285	1.45E-06	4.23E-05
ENSDARG00000069766	caln2	134.797121	-0.650187751	0.21990814	-2.95663335	0.00311018	0.02382428
ENSDARG00000002391	tlcd1	309.836077	0.451932593	0.15145226	2.98399376	0.00284513	0.02228102
ENSDARG00000077860	ankhd1	8351.14573	0.414808178	0.11402101	3.63799769	0.00027477	0.00360386
ENSDARG00000037012	slc3a2b	5968.05263	0.441447571	0.13620656	3.24101549	0.00119105	0.01134233
ENSDARG00000087993	bada	393.814301	-0.461948075	0.12098563	-3.8182061	0.00013443	0.00201153
ENSDARG00000005945	sart1	3210.57396	-0.332272843	0.09510335	-3.49380808	0.00047618	0.0055592

Table S1. DEGs of WT vs. *terfa+/-*

ENSDARG00000055278	cfb	3350.27918	0.807713044	0.13749982	5.87428457	4.25E-09	2.43E-07
ENSDARG00000061454	nrxn2a	3015.53075	0.350304958	0.12733619	2.75102424	0.00594092	0.03864477
ENSDARG00000055518	pygma	7377.24082	-0.447701221	0.12080719	-3.70591543	0.00021063	0.00289396
ENSDARG00000077257	timd4	74.5269658	-0.9155268	0.26698974	-3.42907114	0.00060565	0.00670831
ENSDARG00000032263	zgc:110224	139.483984	-0.721309475	0.2430405	-2.96785707	0.00299884	0.02318386
ENSDARG00000030006	slc6a7	594.285549	0.383577539	0.13708602	2.79807917	0.00514075	0.03472102
ENSDARG00000073961	si:ch211-160j14.2	123.212635	-1.282751439	0.28030005	-4.5763511	4.73E-06	0.00011713
ENSDARG00000005908	clk4b	1255.96955	-0.397148931	0.11343834	-3.50101147	0.0004635	0.00543662
ENSDARG00000104924	BX072577.1	80.1542491	0.826673652	0.31147291	2.65407879	0.00795253	0.0479796
ENSDARG00000103606	ndst1b	2127.64727	-0.236270669	0.08215787	-2.87581314	0.00402988	0.02897388
ENSDARG00000036629	rps14	25862.5101	0.307767956	0.10296015	2.98919494	0.00279714	0.02200574
ENSDARG00000032373	rnf145b	1797.72929	-0.253922462	0.0797271	-3.1848951	0.00144806	0.01317912
ENSDARG00000033444	map4k6	509.242	-0.804988791	0.11441885	-7.03545587	1.99E-12	2.11E-10
ENSDARG00000006408	hmgb3b	1638.6602	-0.63855549	0.1266945	-5.04011996	4.65E-07	1.56E-05
ENSDARG00000007108	lipia	421.9583	-0.366819255	0.12983523	-2.82526747	0.00472412	0.03253593
ENSDARG00000054530	rars1	1836.45736	0.253539512	0.09108374	2.78358697	0.00537614	0.03592045
ENSDARG00000117130	BX936439.1	143.064841	-0.836943058	0.18780185	-4.45652197	8.33E-06	0.00019074
ENSDARG00000060695	znf346	2246.88815	0.787143565	0.13423011	5.86413559	4.51E-09	2.56E-07
ENSDARG00000115557	cox7b	5354.81665	0.62676249	0.10758741	5.82561186	5.69E-09	3.20E-07
ENSDARG00000054191	pgk1	14947.526	0.277345862	0.09898176	2.80198958	0.00507885	0.03447156
ENSDARG00000054103	ripply1	100.310782	-0.686099532	0.23116438	-2.96801584	0.00299729	0.0231791
ENSDARG00000054060	pof1b	2603.28422	0.430038312	0.09147283	4.70126801	2.59E-06	6.98E-05
ENSDARG00000053831	vtnb	3466.48794	0.693498254	0.09150166	7.57907848	3.48E-14	4.97E-12
ENSDARG00000053853	slc13a2	3491.02481	1.282112175	0.13962916	9.18226694	4.22E-20	1.30E-17
ENSDARG00000053875	cryba1b	8475.43081	-2.339791487	0.28679448	-8.15842581	3.39E-16	6.59E-14
ENSDARG00000095185	si:ch211-274p24.2	53.5531774	-2.313414163	0.32676762	-7.07969212	1.44E-12	1.62E-10
ENSDARG00000075551	aifm5	109.353621	-1.500463192	0.28325094	-5.29729294	1.18E-07	4.73E-06
ENSDARG00000087088	si:ch211-274p24.4	55.7656165	-0.819705045	0.28192807	-2.90749712	0.00364334	0.02687096
ENSDARG00000010385	sept4a	462.593907	-0.537893258	0.18497321	-2.90795227	0.00363804	0.02684434
ENSDARG00000017843	srsf1b	2910.17232	0.508265142	0.08324	6.10602008	1.02E-09	6.79E-08
ENSDARG00000046012	slc47a1	309.342892	-0.562637842	0.16114023	-3.49160382	0.00048013	0.00558952
ENSDARG00000069048	serpinf1	2762.10373	0.311620005	0.10187661	3.0587983	0.00222227	0.01846071
ENSDARG00000080188	CR847523.1	7.79039159	2.363039757	0.82828761	2.85292176	0.00433193	0.03052685
ENSDARG00000104280	bnip1b	459.449205	-0.340489337	0.12598302	-2.70266064	0.00687869	0.04315243
ENSDARG00000020122	endou2	416.67469	-0.701554795	0.25076926	-2.7976108	0.00514821	0.03476196
ENSDARG00000096879	CU462980.1	82.1417332	-0.692425659	0.24360475	-2.84241442	0.00447733	0.03126831
ENSDARG00000010085	p4ha2	1154.16731	1.003538749	0.11972117	8.38229996	5.19E-17	1.13E-14
ENSDARG00000004754	hspa4a	3671.41759	0.684269977	0.09511212	7.19435108	6.28E-13	7.39E-11
ENSDARG00000077861	sowahaa	106.025412	-0.607646056	0.20383412	-2.98108112	0.00287233	0.02242906
ENSDARG00000044328	ankrd46a	1334.23232	-0.395354262	0.08985205	-4.40005839	1.08E-05	0.00024081
ENSDARG00000092985	CU499330.1	169.755448	-0.60401619	0.22372261	-2.69984415	0.0069372	0.04340987
ENSDARG00000053624	csf1rb	95.3927472	-0.968388471	0.23399648	-4.13847457	3.50E-05	0.00064705
ENSDARG00000004161	ik	3608.27659	-0.213623453	0.07426659	-2.87644098	0.00402187	0.02893305
ENSDARG00000087492	FO704810.1	459.049904	0.672814041	0.16523302	4.07191036	4.66E-05	0.00082339
ENSDARG00000019713	oatx	602.988465	0.510320242	0.17841974	2.86022292	0.00423343	0.03009782
ENSDARG00000042811	fgf1b	125.584013	-1.121885884	0.29471047	-3.80673911	0.00014081	0.00208573
ENSDARG00000100774	cmc4	31.6441487	1.55832393	0.50477007	3.08719559	0.00202055	0.01716946
ENSDARG00000103740	fundc2	1180.3549	-0.427357708	0.11239517	-3.80227837	0.00014337	0.00211036
ENSDARG00000068732	spry4	1649.27613	0.303322351	0.1074035	2.82413834	0.00474079	0.03262247
ENSDARG00000060282	rnf121	1067.06481	0.377263672	0.1300807	2.90022796	0.00372891	0.02731965
ENSDARG00000044319	fstl4	645.646803	0.346925696	0.11768062	2.94802738	0.00319809	0.02428612
ENSDARG00000105104	si:dkey-223p19.1	36.6760518	-1.387548221	0.44750733	-3.10061562	0.00193119	0.01651744
ENSDARG00000104375	sec24a	4686.11075	0.246671634	0.07438105	3.31632354	0.0009121	0.00922682
ENSDARG00000099329	macroh2a1	697.384597	-0.33007987	0.119864	-2.75378644	0.00589102	0.03844087
ENSDARG00000110357	txndc15	803.633279	0.489647323	0.11692236	4.18779872	2.82E-05	0.00053587
ENSDARG00000088347	sp1	674.582431	-0.724998246	0.13559037	-5.34697428	8.94E-08	3.71E-06
ENSDARG00000011407	col2a1b	48668.6835	0.254140038	0.08700852	2.92086383	0.00349062	0.02603044
ENSDARG00000117448	CABZ01072245.1	926.526455	0.820110512	0.159686	5.13576952	2.81E-07	1.02E-05

Table S1. DEGs of WT vs. *terfa+/-*

ENSDARG00000109859	CABZ01072242.1	326.92667	0.598674875	0.1859549	3.21946267	0.00128431	0.01201865
ENSDARG00000059342	avil	176.941835	0.744262246	0.24289757	3.06409922	0.00218326	0.01822198
ENSDARG00000021059	alas1	5215.08046	0.433786526	0.11774559	3.68410004	0.00022951	0.00310366
ENSDARG00000024295	slc11a2	1535.58625	0.674076382	0.11326717	5.95120688	2.66E-09	1.61E-07
ENSDARG00000097003	CR855375.2	8.50968884	-2.562711084	0.80270945	-3.1925762	0.0014101	0.01294238
ENSDARG00000034624	nuf2	562.669605	-0.473702821	0.17074983	-2.77425057	0.0055329	0.03677998
ENSDARG00000070922	cnbpb	7559.52365	-0.2960898	0.0783091	-3.78103962	0.00015617	0.00226472
ENSDARG00000100157	tamm41	426.48007	0.46220477	0.11620474	3.97750346	6.96E-05	0.00115414
ENSDARG00000007955	iars1	4486.57496	0.538104105	0.11274685	4.77267509	1.82E-06	5.15E-05
ENSDARG00000077717	rpl29	7479.93039	0.407207332	0.09240614	4.40671273	1.05E-05	0.00023458
ENSDARG00000016783	srsf6b	5100.93767	0.363367983	0.07116166	5.10623232	3.29E-07	1.17E-05
ENSDARG00000097827	lrp1aa	9642.77974	0.372659596	0.11401422	3.268536	0.00108105	0.0104714
ENSDARG00000044179	rbms2a	1832.24129	0.322984337	0.08730162	3.69963746	0.00021591	0.00295669
ENSDARG00000103133	hoxc12b	128.125321	-0.792063658	0.21588139	-3.66897607	0.00024352	0.00326472
ENSDARG00000101954	hoxc6b	203.506906	-1.16208234	0.19194544	-6.05423253	1.41E-09	9.08E-08
ENSDARG00000101308	smug1	354.184372	-0.611255825	0.15924137	-3.8385492	0.00012376	0.00187216
ENSDARG00000011020	hnrnpa1a	13343.9112	0.650718466	0.15319942	4.24752552	2.16E-05	0.00043047
ENSDARG00000007207	cnpy2	609.463582	0.938748499	0.13262422	7.07825831	1.46E-12	1.63E-10
ENSDARG00000101048	FQ790208.1	13.9673135	-2.922931899	0.66235474	-4.41294024	1.02E-05	0.00022855
ENSDARG00000002897	si:ch211-160o17.2	549.145476	-0.493819429	0.13848691	-3.56582013	0.00036272	0.00447415
ENSDARG00000077095	si:ch211-160o17.4	4927.3036	0.571201659	0.08915607	6.40676154	1.49E-10	1.15E-08
ENSDARG00000009733	kif21b	686.66572	0.60557434	0.21432484	2.82549763	0.00472072	0.03253593
ENSDARG00000091298	pmela	5975.77604	0.450389778	0.10784033	4.17645029	2.96E-05	0.00055932
ENSDARG00000059357	sarnp	1293.74422	-0.376585893	0.10227148	-3.68221824	0.00023121	0.00312273
ENSDARG00000020840	mcrs1	1416.01822	-0.283041958	0.10664448	-2.65407052	0.00795272	0.0479796
ENSDARG00000055014	si:dkey-33m11.8	214.324926	-0.640143838	0.19693144	-3.25059239	0.00115165	0.01102203
ENSDARG00000059323	rpn1	8233.71156	0.328030612	0.09731056	3.37096634	0.00074905	0.0079123
ENSDARG00000003877	pbrm1	1177.89255	0.543633038	0.12945749	4.19931706	2.68E-05	0.00051663
ENSDARG00000006219	gnl3	1921.14395	-0.389394287	0.10639359	-3.65994122	0.00025227	0.00335846
ENSDARG00000040157	glt8d1	691.442923	0.373519609	0.10338765	3.61280673	0.0003029	0.00388472
ENSDARG00000074221	ABCA7	1071.41612	-0.841909235	0.13058734	-6.44709667	1.14E-10	9.02E-09
ENSDARG00000063254	r3hdm4	1085.26443	0.455873951	0.12366307	3.68641955	0.00022743	0.00308055
ENSDARG00000070846	dazap1	374.512757	-0.656185595	0.14397497	-4.55763652	5.17E-06	0.00012693
ENSDARG00000003165	nr2f6b	1644.9865	-0.276222787	0.09072093	-3.04475275	0.00232872	0.01918395
ENSDARG00000059177	tax1bp3	1094.32968	0.352617701	0.13063541	2.69925051	0.00694958	0.04346156
ENSDARG00000042777	ndufa11	1594.00926	0.637456149	0.13419018	4.75039348	2.03E-06	5.67E-05
ENSDARG00000097452	CU929070.5	129.852782	-0.484157705	0.17652142	-2.74277032	0.00609233	0.03935156
ENSDARG00000087349	reep6	140.087533	-0.669564096	0.20176703	-3.31850109	0.00090502	0.00919238
ENSDARG00000001782	prkacba	214.590539	-0.522010487	0.18046262	-2.89262391	0.00382038	0.02781361
ENSDARG00000007024	uox	788.245496	-0.611361144	0.22307603	-2.74059543	0.0061328	0.03955129
ENSDARG00000053113	ly75	736.959157	-0.461655553	0.15443868	-2.98924823	0.00279665	0.02200574
ENSDARG00000007715	lgsn	611.099407	-2.047096723	0.19994167	-10.2384695	1.33E-24	5.62E-22
ENSDARG00000006242	ptp4a1	7384.66768	-0.379777988	0.10298306	-3.68777156	0.00022623	0.00306925
ENSDARG00000074508	si:dkey-28e7.3	849.340081	-0.486810109	0.12083854	-4.02859961	5.61E-05	0.0009638
ENSDARG00000045298	wipf2a	2086.95454	0.307972609	0.08192091	3.75938956	0.00017033	0.00242887
ENSDARG00000044942	mrpl45	699.091704	0.313648818	0.10259797	3.05706666	0.00223515	0.0185491
ENSDARG00000058448	zrsr2	328.937595	-0.533351259	0.12282803	-4.34226027	1.41E-05	0.00030087
ENSDARG00000089681	btf3l4	1457.99886	-0.290639035	0.09596927	-3.02845936	0.00245804	0.01994601
ENSDARG00000103735	elovl1b	2100.28157	0.321600995	0.07993523	4.02326992	5.74E-05	0.00098113
ENSDARG00000070083	atp5f1b	81540.7095	0.309376872	0.09324428	3.31791786	0.00090691	0.00920428
ENSDARG00000040779	med16	721.530774	0.412484538	0.12051195	3.42276886	0.00061987	0.00684139
ENSDARG00000104047	si:ch211-262i1.3	300.685893	-1.13968466	0.16351066	-6.97009386	3.17E-12	3.24E-10
ENSDARG00000104204	slc1a8a	438.068033	-0.612556532	0.16274461	-3.76391285	0.00016728	0.00240048
ENSDARG00000069481	ghrh	23.07711	2.189164336	0.69216961	3.1627571	0.00156283	0.01399786
ENSDARG00000044925	cdk5rap1	250.870036	0.466749467	0.13662616	3.41625254	0.00063489	0.00696701
ENSDARG00000058225	arpc4l	2156.56991	-0.255337629	0.08908061	-2.86636593	0.00415214	0.02963855
ENSDARG00000105445	CR769769.2	669.551778	-0.900250834	0.11012535	-8.17478295	2.96E-16	5.89E-14
ENSDARG00000016773	cishb	109.255616	-0.656757998	0.21064807	-3.11779738	0.00182208	0.01575228

Table S1. DEGs of WT vs. *terfa+/-*

ENSDARG00000019797	qrich1	1086.05491	0.380379057	0.12266895	3.1008584	0.00192961	0.01650958
ENSDARG00000060954	tmcc1b	1535.23235	-0.292789695	0.08352889	-3.5052505	0.00045618	0.00538637
ENSDARG00000070651	prkcdb	2733.20945	0.608810778	0.11876347	5.12624602	2.96E-07	1.06E-05
ENSDARG00000077404	ncoa3	3136.92824	0.437492156	0.10361103	4.22244774	2.42E-05	0.00047221
ENSDARG00000040764	id1	341.292838	1.062832708	0.23727108	4.4794026	7.49E-06	0.00017518
ENSDARG00000037587	SYNPR	434.812836	-0.867091696	0.15769125	-5.49866726	3.83E-08	1.75E-06
ENSDARG00000044894	zgc:113307	502.49798	-0.424109111	0.13748305	-3.08481009	0.00203682	0.01726644
ENSDARG00000076120	foxp4	4125.23711	0.328446682	0.11184976	2.9364987	0.0033194	0.02497087
ENSDARG00000014358	optc	60.4503664	1.105374866	0.29963933	3.68901793	0.00022512	0.00305652
ENSDARG00000086927	pik3c2b	2244.92666	0.474687258	0.10818902	4.38757344	1.15E-05	0.00025301
ENSDARG00000011613	rbm39a	5688.6318	-0.201005988	0.07281439	-2.76052556	0.00577084	0.03786545
ENSDARG00000002912	adipor1a	1903.49045	-0.256059612	0.08434217	-3.03596179	0.0023977	0.01959065
ENSDARG00000013087	ndrg3a	5984.22322	0.233573764	0.08438496	2.76795479	0.00564093	0.03729612
ENSDARG00000056995	snai1a	697.127546	-0.420273261	0.15061794	-2.79032672	0.00526549	0.03538083
ENSDARG00000089724	cyldb	537.337403	1.450979493	0.20590774	7.04674559	1.83E-12	1.96E-10
ENSDARG00000070545	top1l	3981.36069	0.503375292	0.09962947	5.05247363	4.36E-07	1.49E-05
ENSDARG00000077883	fam83d	336.346189	-0.724865	0.20016391	-3.62135719	0.00029306	0.00377412
ENSDARG00000102967	CT573282.1	138.745456	-1.091419234	0.25999922	-4.19777889	2.70E-05	0.00051894
ENSDARG00000044862	opn1lw1	27.6348697	-2.815002615	0.49902763	-5.64097545	1.69E-08	8.39E-07
ENSDARG00000025679	comtb	506.678188	-0.522013633	0.16319999	-3.19861306	0.0013809	0.0127167
ENSDARG00000077434	mon1bb	655.094074	-0.333426872	0.10927206	-3.05134595	0.00227818	0.01884948
ENSDARG00000070513	brpf3a	1188.86405	-0.329993219	0.10737722	-3.07321442	0.00211766	0.01778103
ENSDARG00000007494	st3gal8	638.871104	-0.422432582	0.12347606	-3.42116998	0.00062352	0.00687868
ENSDARG00000055620	acad9	1107.16216	0.290417625	0.08971786	3.23701009	0.00120789	0.01145009
ENSDARG00000044827	wnt7aa	1017.90137	-0.392310213	0.10992939	-3.56874716	0.00035869	0.00442886
ENSDARG00000077215	eif4g3a	2803.50717	0.35431127	0.11614543	3.05058289	0.00228398	0.01888497
ENSDARG00000104418	BX248318.1	205.883774	-0.653476601	0.20823992	-3.13809472	0.0017005	0.01497198
ENSDARG00000070437	rpl22	11898.6355	0.533855555	0.09525987	5.60420175	2.09E-08	1.01E-06
ENSDARG00000056541	nr0b1	182.902441	-0.538954259	0.16386545	-3.28900485	0.00100542	0.00990087
ENSDARG00000103205	si:ch211-161f7.2	4.59654111	3.749502678	1.30443973	2.8744162	0.00404775	0.0290839
ENSDARG00000097491	ugt1b1	961.409062	0.354471161	0.11846116	2.99229861	0.00276885	0.02183371
ENSDARG00000055976	pecr	519.730668	-0.414888401	0.11944117	-3.47357947	0.00051357	0.00588776
ENSDARG00000043442	zgc:153665	732.63565	0.693425683	0.132931	5.21643319	1.82E-07	6.93E-06
ENSDARG00000078959	tpcn3	373.808903	0.350456061	0.12198106	2.87303667	0.00406547	0.0291791
ENSDARG00000086881	ier2b	660.337362	-0.475648957	0.14124619	-3.367517	0.00075848	0.00799057
ENSDARG00000001897	man2b1	1768.97747	0.389144068	0.12875206	3.02242973	0.00250754	0.02028148
ENSDARG00000004632	egln1b	457.67509	0.423371475	0.11681598	3.62426001	0.00028979	0.00375338
ENSDARG00000068193	tmem44	40.7969364	0.951199025	0.33538682	2.83612523	0.00456645	0.03169484
ENSDARG00000061260	si:dkey-202e22.2	603.187568	0.407289885	0.12036101	3.38390206	0.00071463	0.00764296
ENSDARG00000078585	mon1a	312.881452	0.464102046	0.15800682	2.93722789	0.00331161	0.02494242
ENSDARG00000041974	brk1	3933.68225	0.281583048	0.10573264	2.66316111	0.00774103	0.04706541
ENSDARG00000003526	psma5	2823.42901	-0.244684419	0.08804117	-2.77920444	0.00544922	0.0363111
ENSDARG00000090297	ldlrad2	224.525389	0.684718428	0.22219724	3.08157942	0.00205906	0.01741335
ENSDARG00000105511	BX248521.2	44.1400313	1.566427035	0.40514775	3.86631057	0.00011049	0.00170368
ENSDARG00000105454	AL928854.1	71.7239785	-0.998555243	0.28011404	-3.56481684	0.00036411	0.00448462
ENSDARG00000044569	cldn19	640.826371	-0.510395529	0.16447469	-3.10318586	0.00191449	0.01640853
ENSDARG00000071212	p3h1	718.711004	0.373316538	0.12465851	2.99471368	0.00274703	0.02170815
ENSDARG00000078805	ap5b1	302.025231	-0.478945037	0.1784655	-2.68368419	0.00728159	0.04497444
ENSDARG00000010519	per3	3926.03969	0.538049007	0.12775557	4.21155036	2.54E-05	0.00049364
ENSDARG00000060900	znf362a	1593.93721	-0.448630222	0.08233789	-5.44864838	5.08E-08	2.23E-06
ENSDARG00000098988	si:ch211-193i15.1	13.1950813	-3.390016203	0.69004148	-4.91277164	8.98E-07	2.78E-05
ENSDARG00000095536	trim62.2	65.5080898	-3.432325725	1.25111715	-2.74340875	0.00608049	0.03930577
ENSDARG00000101201	si:dkey-207b20.2	16.7837304	6.606355982	1.33837208	4.93611313	7.97E-07	2.49E-05
ENSDARG00000060901	trim62.1	273.36345	1.304962938	0.18752741	6.95878514	3.43E-12	3.50E-10
ENSDARG00000102305	svbp	477.036207	-0.502670788	0.16594602	-3.02912223	0.00245265	0.0199153
ENSDARG00000099306	zgc:175088	3457.27991	0.630141033	0.13381852	4.70892254	2.49E-06	6.76E-05
ENSDARG00000098972	mrps16	532.356712	-0.420458028	0.11916302	-3.528427	0.00041804	0.0050098
ENSDARG00000108098	CR759952.1	2.81658384	5.82760901	1.96803304	2.96113372	0.00306509	0.02355682

Table S1. DEGs of WT vs. *terfa+/-*

ENSDARG00000116835	park7	1276.62603	0.468290231	0.11468596	4.08323929	4.44E-05	0.00078904
ENSDARG00000074808	megf6b	1044.96705	0.418810759	0.12761864	3.28173654	0.0010317	0.01008372
ENSDARG00000038205	her2	146.593803	-1.076894171	0.20383432	-5.28318389	1.27E-07	5.07E-06
ENSDARG00000054560	her15.2	212.028837	0.622713747	0.2285406	2.7247401	0.00643521	0.04099108
ENSDARG00000112527	FP325130.1	680.013262	0.625951856	0.13645519	4.58723381	4.49E-06	0.0001122
ENSDARG00000098374	FLNB	3844.36007	0.552070345	0.11771756	4.6897875	2.73E-06	7.30E-05
ENSDARG00000100339	arf4b	168.451686	2.302877544	0.30749167	7.48923554	6.93E-14	9.31E-12
ENSDARG00000074210	zgc:110286	34.3009288	3.03453333	0.5310897	5.71378686	1.10E-08	5.79E-07
ENSDARG00000045989	arf4a	1255.63636	0.290890924	0.10342407	2.81260384	0.00491422	0.03351889
ENSDARG00000021838	rps23	27515.3593	0.665578902	0.08976185	7.4149423	1.22E-13	1.60E-11
ENSDARG00000098359	il17rd	1177.69995	0.474142262	0.08482487	5.58966066	2.28E-08	1.08E-06
ENSDARG00000104758	zgc:194981	127.468469	0.594534633	0.20463867	2.9052897	0.00366913	0.02702113
ENSDARG00000102755	EML6	1452.94006	0.492420103	0.16368379	3.00836215	0.0026266	0.02101195
ENSDARG00000112439	CABZ01092982.2	396.812848	0.644784356	0.19300779	3.34071667	0.00083562	0.00862161
ENSDARG00000105456	FO834831.1	7.63717456	6.012815955	1.38634164	4.33718196	1.44E-05	0.00030596
ENSDARG00000086826	sult6b1	1486.20467	0.653645246	0.11917874	5.48457913	4.14E-08	1.87E-06
ENSDARG00000076371	SHROOM2	453.823652	-0.451466259	0.16088083	-2.80621535	0.00501272	0.03407853
ENSDARG00000105442	LOXL4	422.518031	0.659800085	0.12978172	5.08392143	3.70E-07	1.29E-05
ENSDARG00000101057	srsf7b	253.304683	-0.544149092	0.18326838	-2.96913794	0.00298637	0.023109
ENSDARG00000099755	pfklb	2539.33531	0.501048779	0.10766805	4.65364388	3.26E-06	8.42E-05
ENSDARG00000101050	tmc8	67.5502593	1.164788529	0.3889578	2.99463988	0.00274769	0.02170815
ENSDARG00000098639	pycr1b	3201.48769	0.625035265	0.10765966	5.80565887	6.41E-09	3.55E-07
ENSDARG00000098118	trappc10	2895.33373	0.299517131	0.08857096	3.38166303	0.00072048	0.00768239
ENSDARG00000098082	CU459093.1	190.28366	-0.807625408	0.26468694	-3.05124766	0.00227893	0.01884948
ENSDARG00000100503	sos1	1667.99548	0.359773432	0.08946124	4.0215567	5.78E-05	0.00098626
ENSDARG00000105394	gemin6	355.004476	0.409483384	0.15195715	2.69472924	0.00704458	0.04386101
ENSDARG00000063570	dyrk1aa	4351.67307	-0.460736467	0.10706585	-4.30330014	1.68E-05	0.00034795
ENSDARG00000075650	vps26c	1696.12084	0.554609115	0.11021906	5.0318802	4.86E-07	1.62E-05
ENSDARG00000058988	KCNJ15	319.040744	0.506109553	0.14209923	3.56166277	0.00036851	0.00452316
ENSDARG00000102127	NHLRC3	1068.10916	0.390075809	0.12183021	3.20179862	0.00136572	0.01260025
ENSDARG00000103612	akt2l	2188.66631	0.35413488	0.13306101	2.66144751	0.00778055	0.04719452
ENSDARG00000103006	si:ch1073-303d10.1	95.0994278	-1.020194989	0.35176923	-2.90018256	0.00372945	0.02731965
ENSDARG00000014550	rfx3	1128.19407	0.38644665	0.12865782	3.00367783	0.00266738	0.02125405
ENSDARG00000069726	glis3	84.2789606	-0.977088716	0.33721201	-2.89755014	0.0037609	0.02747709
ENSDARG00000027600	pdlim5b	1840.56202	0.355113748	0.08807457	4.03196701	5.53E-05	0.00095206
ENSDARG00000109022	WDR70	744.747657	0.349510678	0.11529057	3.03156336	0.00243291	0.01978322
ENSDARG00000092467	si:ch73-46j18.5	10862.3295	0.317949289	0.11990627	2.65164854	0.00800999	0.04820538
ENSDARG00000040087	epb41l4a	761.728808	0.619455145	0.13064761	4.74141953	2.12E-06	5.91E-05
ENSDARG00000013024	erap1a	266.13517	-0.594603164	0.19359631	-3.07135581	0.00213089	0.01786885
ENSDARG00000075446	naa25	2150.63793	-0.282951434	0.07913248	-3.57566755	0.00034934	0.0043392
ENSDARG00000114920	TTC28	3039.54544	0.329788056	0.11105246	2.9696602	0.00298129	0.02308413
ENSDARG00000075802	grin3a	196.064706	-0.427802716	0.15737699	-2.71833071	0.00656122	0.04153071
ENSDARG00000063665	mat2al	822.179959	0.532188382	0.15012788	3.54490051	0.00039276	0.0047713
ENSDARG00000003429	hnrnpdl	3714.83974	-0.322851978	0.10460872	-3.08628169	0.00202677	0.01720449
ENSDARG00000021082	sec31a	2852.52778	0.639330206	0.09504096	6.72689101	1.73E-11	1.58E-09
ENSDARG00000100729	tln1	9648.43062	0.298202931	0.09543069	3.12481169	0.00177919	0.01551096
ENSDARG00000040334	mat2aa	10002.3444	0.737256223	0.09323412	7.90757976	2.62E-15	4.45E-13
ENSDARG00000053487	osbp2	673.620295	0.424342353	0.14461179	2.93435511	0.00334242	0.02511359
ENSDARG00000036670	aplnrb	807.963154	0.42442449	0.15032427	2.823393	0.00475183	0.03267128
ENSDARG00000057992	fstb	1147.05341	0.374206083	0.13599699	2.75157621	0.00593092	0.03859991
ENSDARG00000075600	si:dkeyp-41f9.3	281.211961	-1.714675452	0.22700723	-7.55339561	4.24E-14	5.89E-12
ENSDARG00000044254	anxa3b	1133.80339	-0.369801192	0.12140517	-3.04600848	0.00231901	0.01913648
ENSDARG00000079049	cercam	1031.14837	0.632917298	0.095863	6.60231027	4.05E-11	3.43E-09
ENSDARG00000058102	sardh	3927.14612	0.411356154	0.11685998	3.5200772	0.00043142	0.00515106
ENSDARG00000093881	NA	4.34015842	-6.321998929	1.54337406	-4.09621952	4.20E-05	0.00075132
ENSDARG00000043729	plac8.1	486.137697	-0.830043608	0.22473932	-3.69336172	0.00022131	0.00301344
ENSDARG00000000804	rassf6	398.466185	-0.538680201	0.13640977	-3.94898556	7.85E-05	0.00127247
ENSDARG00000085561	CR759894.1	64.534535	-0.903947741	0.28947157	-3.12275139	0.00179169	0.01558887

Table S1. DEGs of WT vs. *terfa+/-*

ENSDARG00000026736	il11ra	401.972674	-0.93348435	0.17990399	-5.18879174	2.12E-07	7.92E-06
ENSDARG00000087779	pum3	1298.14556	-0.676750806	0.10172692	-6.65262249	2.88E-11	2.51E-09
ENSDARG00000073695	mamdc2b	159.790875	0.837218004	0.21058167	3.97574012	7.02E-05	0.00115914
ENSDARG00000063309	tjp2a	2408.50277	0.357719665	0.09751684	3.668286	0.00024418	0.00327001
ENSDARG00000076560	apbb1	1499.12223	-0.37438268	0.13146848	-2.84769916	0.00440365	0.03091014
ENSDARG00000095594	si:dkey-184a18.5	102.093188	-1.037451266	0.28051057	-3.69843916	0.00021693	0.00296741
ENSDARG00000017229	megf10	731.92074	0.320824257	0.10818066	2.96563406	0.0030206	0.02331582
ENSDARG00000044304	prrc1	1305.70119	0.302886659	0.08182161	3.70179295	0.00021408	0.00293491
ENSDARG00000114472	ctxn3	104.000186	1.717644162	0.26529002	6.47459026	9.51E-11	7.62E-09
ENSDARG00000051896	fbn2a	708.198294	1.590619535	0.13072849	12.1673521	4.64E-34	4.27E-31
ENSDARG00000046053	slc27a6	281.928244	-0.755293596	0.13985878	-5.40040172	6.65E-08	2.84E-06
ENSDARG00000069472	chsy3	664.751119	0.576415746	0.11527671	5.00027919	5.72E-07	1.86E-05
ENSDARG00000093444	si:dkey-106l3.7	73.4601376	-1.061511084	0.26273026	-4.04030759	5.34E-05	0.00092588
ENSDARG00000078105	depdc5	2002.91018	0.331643617	0.09453043	3.50832646	0.00045094	0.00533968
ENSDARG00000025220	sppl3	2286.38346	0.242300038	0.08882086	2.72796334	0.00637267	0.04067609
ENSDARG00000010641	slc20a1b	7707.2646	0.264869196	0.08047341	3.29138774	0.00099694	0.00984076
ENSDARG00000011743	pigo	1102.22513	-0.388799877	0.10031285	-3.87587321	0.00010624	0.00164425
ENSDARG00000039351	ccl19b	69.1961532	1.064328387	0.34795521	3.05880863	0.00222219	0.01846071
ENSDARG00000044183	prkab1a	736.611075	-0.356849868	0.09997912	-3.56924407	0.00035801	0.00442487
ENSDARG00000032083	dpysl2b	6146.18972	0.65540395	0.13964809	4.693254	2.69E-06	7.21E-05
ENSDARG00000027687	tmem230a	104.212229	-0.722168477	0.23069212	-3.13044276	0.00174543	0.01530253
ENSDARG00000075864	igsf9a	1052.24999	-0.35430349	0.09956514	-3.55850956	0.00037297	0.00456878
ENSDARG00000013110	dmtn	582.199661	-0.35154369	0.11014201	-3.19173119	0.00141423	0.01297072
ENSDARG00000044010	loxl2a	1790.36538	0.772377647	0.10058117	7.67914744	1.60E-14	2.43E-12
ENSDARG00000057665	sprn2	279.301561	-0.963960789	0.16036575	-6.01101428	1.84E-09	1.16E-07
ENSDARG00000103013	pcdh1g22	437.787745	0.983223452	0.21028735	4.67561853	2.93E-06	7.75E-05
ENSDARG00000099447	pcdh1g18	124.008097	-0.853140921	0.24359375	-3.50231046	0.00046124	0.00541529
ENSDARG00000099035	pcdh1g33	612.855904	-0.698948419	0.20115984	-3.4745923	0.00051163	0.0058737
ENSDARG00000112454	FO744833.2	118.270886	0.894881981	0.25990188	3.4431532	0.00057497	0.00642827
ENSDARG00000079251	nlgn2b	939.446382	0.509344948	0.14891842	3.42029509	0.00062553	0.00688274
ENSDARG00000056885	per1a	851.286311	2.193592195	0.15544051	14.1121007	3.20E-45	8.83E-42
ENSDARG00000056873	and3	6882.75472	0.470786051	0.10091752	4.66505769	3.09E-06	8.07E-05
ENSDARG00000039215	arrb2a	752.53633	-0.440035484	0.11094096	-3.96639315	7.30E-05	0.00119633
ENSDARG00000094899	si:ch1073-143l10.2	96.9346588	1.114187531	0.27126522	4.1073734	4.00E-05	0.00072179
ENSDARG00000099273	zgc:103508	432.9341	-0.481139504	0.15540713	-3.09599385	0.00196154	0.01674826
ENSDARG00000086665	si:dkey-175g6.2	542.699812	0.652445311	0.1493227	4.36936446	1.25E-05	0.00027216
ENSDARG00000044091	pitpnab	1190.75787	0.34590278	0.10886928	3.17723025	0.00148689	0.01346326
ENSDARG00000061120	slc43a2b	7477.90641	0.501102292	0.14563004	3.44092672	0.00057973	0.00645279
ENSDARG00000062361	paxbp1	1857.34044	-0.305089964	0.11486271	-2.65612716	0.00790438	0.04777806
ENSDARG00000016439	slc7a1a	541.697058	-0.500253173	0.17191752	-2.90984405	0.00361609	0.02672553
ENSDARG00000076121	smpd1	848.799028	0.451863251	0.1499078	3.01427442	0.00257595	0.0207078
ENSDARG00000095144	AL954816.1	8.04283977	-5.148611344	1.51038414	-3.40880919	0.00065247	0.00711591
ENSDARG00000078430	tiam1a	3047.43183	0.427450248	0.11615215	3.68008885	0.00023315	0.00314434
ENSDARG00000076856	frem2a	2357.80835	0.425821041	0.13610622	3.12859365	0.00175645	0.0153768
ENSDARG00000095400	si:ch73-52f15.5	648.881571	0.630147145	0.10979715	5.73919377	9.51E-09	5.05E-07
ENSDARG00000090629	tmtops3b	28.3905498	1.292317131	0.42460374	3.04358393	0.00233778	0.01923389
ENSDARG00000079895	ehbp1l1b	818.386422	-0.308971933	0.1157955	-2.66825508	0.00762463	0.04649347
ENSDARG00000032816	tm7sf2	236.03649	0.58129977	0.19683761	2.95319459	0.00314504	0.02399192
ENSDARG00000043663	faub	260.352775	-0.547815447	0.16116061	-3.39918938	0.00067586	0.00729893
ENSDARG00000043493	cltca	13520.8534	0.347522809	0.08976864	3.8713166	0.00010825	0.00167114
ENSDARG00000015199	cblb	901.26628	0.428026673	0.10421343	4.1072123	4.00E-05	0.00072179
ENSDARG00000026531	alcama	3206.73536	0.262451683	0.07695873	3.41029112	0.00064894	0.0070898
ENSDARG00000012866	picalma	2354.44289	0.279111167	0.07419318	3.76195181	0.00016859	0.00241114
ENSDARG00000013670	hyou1	3934.29129	0.606347251	0.08579342	7.06752605	1.58E-12	1.73E-10
ENSDARG00000068992	hspa8	159328.045	0.340792194	0.0753861	4.52062393	6.17E-06	0.00014807
ENSDARG00000068974	lim2.1	754.396505	-0.477358533	0.15969827	-2.98912767	0.00279775	0.02200574
ENSDARG00000005645	robo3	3460.21851	0.398169769	0.1366333	2.91414881	0.0035666	0.02649241
ENSDARG00000068919	rad51c	70.667036	-0.726222262	0.25941426	-2.79946937	0.00511867	0.03460949

Table S1. DEGs of WT vs. *terfa+/-*

ENSDARG00000026499	ppm1e	790.779511	0.426280644	0.14397405	2.96081572	0.00306825	0.02357244
ENSDARG00000098867	PRDM15	257.870153	0.605911888	0.21405259	2.83066838	0.00464509	0.03211534
ENSDARG00000090814	si:dkey-18a10.3	150.739823	1.583832764	0.26604259	5.95330539	2.63E-09	1.60E-07
ENSDARG00000032820	rxfp2a	130.434786	-2.089998892	0.30152125	-6.93151436	4.16E-12	4.16E-10
ENSDARG00000056001	fryb	4984.95629	0.312801456	0.11326736	2.76162049	0.00575153	0.03779859
ENSDARG00000098865	BX548044.7	5.84352507	-3.92406981	1.31093291	-2.99334144	0.00275941	0.02178688
ENSDARG00000079542	stard13a	1083.40487	0.362778937	0.13696353	2.64872645	0.00807957	0.04851841
ENSDARG00000068755	exosc8	368.038896	-0.585855013	0.15958247	-3.6711741	0.00024144	0.00323851
ENSDARG00000077224	ypel2a	206.334713	-0.411986934	0.14982561	-2.74977649	0.00596359	0.03874153
ENSDARG00000055092	pora	2762.13216	0.260869419	0.08656279	3.01364394	0.00258131	0.02074308
ENSDARG00000093822	si:dkey-259j3.5	42.7809717	1.018229335	0.36307521	2.80445842	0.00504012	0.03423479
ENSDARG00000088937	si:dkey-259j3.5	128.774307	0.804919475	0.24540113	3.28001531	0.00103801	0.01013348
ENSDARG00000004763	hhla2a.2	137.283401	-1.942489847	0.26227926	-7.40618918	1.30E-13	1.67E-11
ENSDARG00000085822	dre-mir-2184	6.70354034	-2.859723382	0.98054538	-2.91646206	0.00354026	0.02634119
ENSDARG00000099175	hmgb1a	11980.299	-1.013271324	0.09639048	-10.5121512	7.59E-26	3.63E-23
ENSDARG00000060983	smyd4	202.231676	-0.77477825	0.16399788	-4.72431856	2.31E-06	6.33E-05
ENSDARG00000043148	slc1a3b	3486.28749	0.637029217	0.16809597	3.78967578	0.00015084	0.00220025
ENSDARG00000043154	ucp2	7007.33956	0.542352754	0.13519183	4.01172727	6.03E-05	0.00102055
ENSDARG00000063519	PLEKHB1	101.220161	-0.737106111	0.2749298	-2.68107022	0.00733871	0.04521658
ENSDARG00000111240	dhrs13a.2	1858.65455	1.563587527	0.14526359	10.763795	5.10E-27	2.64E-24
ENSDARG00000068716	cuedc1a	1050.21728	-0.341123737	0.12419585	-2.74665973	0.00602056	0.0390198
ENSDARG00000103981	bhlha9	314.911586	0.949989048	0.23437941	4.05321037	5.05E-05	0.00088241
ENSDARG00000075954	serpinh1a	1272.69113	0.728817505	0.12572163	5.79707316	6.75E-09	3.70E-07
ENSDARG00000111843	BX000451.4	121.237564	-0.679634713	0.23515787	-2.89012103	0.00385094	0.02797048
ENSDARG00000108401	LO018336.1	56.8252567	1.110353162	0.27714109	4.0064545	6.16E-05	0.00104068
ENSDARG00000012395	mmp13a	130.970498	1.297311553	0.3285163	3.94900204	7.85E-05	0.00127247
ENSDARG00000045887	mmp30	966.511279	-0.497958337	0.1225557	-4.06311866	4.84E-05	0.00084994
ENSDARG00000103919	si:ch73-1a9.3	21540.371	-0.45438736	0.12188805	-3.72790743	0.00019308	0.00270057
ENSDARG00000041811	rps25	19605.6301	0.283950569	0.0899444	3.15695671	0.00159425	0.01422804
ENSDARG00000104953	stt3a	8782.54875	0.404929306	0.08634826	4.68948998	2.74E-06	7.30E-05
ENSDARG00000019498	cry5	388.347698	1.076039172	0.1281241	8.39841353	4.53E-17	1.00E-14
ENSDARG00000002666	kcnj1b	88.6859399	-0.743364914	0.26221579	-2.83493576	0.00458349	0.03177291
ENSDARG00000017251	smtlb	76.9970636	-1.301743626	0.35873736	-3.62868151	0.00028487	0.00370319
ENSDARG00000093230	si:ch211-139n6.3	128.694823	-0.834137921	0.22987068	-3.62872689	0.00028482	0.00370319
ENSDARG00000036456	anxa4	3958.60802	0.338944565	0.09138836	3.70883731	0.00020821	0.00286711
ENSDARG00000019897	gpat4	1422.88525	0.3266502	0.1221107	2.67503333	0.00747218	0.04578922
ENSDARG00000078258	CABZ01049847.1	314.388782	-0.772812582	0.18301541	-4.2226641	2.41E-05	0.00047221
ENSDARG00000018342	ggt1b	180.218217	-0.591510186	0.18161247	-3.25699097	0.001126	0.0108224
ENSDARG00000093535	BX005309.1	779.830658	-0.587170719	0.11445665	-5.130071	2.90E-07	1.05E-05
ENSDARG00000112575	CU467905.1	202.441561	1.669312381	0.33403678	4.99739099	5.81E-07	1.89E-05
ENSDARG00000089340	rhobtb2b	639.083995	1.109398231	0.17851768	6.21450068	5.15E-10	3.56E-08
ENSDARG00000116344	CABZ01063556.1	294.849854	0.570369124	0.19759003	2.88662905	0.00389393	0.0282042
ENSDARG00000104846	atg10	155.661767	0.705036737	0.21271206	3.31451227	0.00091803	0.00927171
ENSDARG00000035914	tmem167a	814.453736	0.336642374	0.10786172	3.12105518	0.00180204	0.01564438
ENSDARG00000009401	vcanb	11404.684	0.598846613	0.10075243	5.94374357	2.79E-09	1.66E-07
ENSDARG00000068516	hapln1b	1167.97931	-0.446585797	0.14874369	-3.00238489	0.00267873	0.02131872
ENSDARG00000036028	arrdc3b	4693.58522	0.455695214	0.11352898	4.01391081	5.97E-05	0.00101322
ENSDARG00000036031	sept5b	1007.62431	0.612437772	0.22390419	2.73526714	0.00623297	0.04001043
ENSDARG00000068507	crybb1	3625.14152	-3.580817933	0.3411344	-10.4967952	8.94E-26	4.19E-23
ENSDARG00000024548	cryba4	14181.1209	-1.123505343	0.18889484	-5.94778204	2.72E-09	1.63E-07
ENSDARG00000037229	denr	1282.29684	-0.376897818	0.10468983	-3.60013787	0.00031805	0.00403939
ENSDARG00000060627	hip1ra	173.761718	0.626683647	0.16899773	3.70823715	0.00020871	0.00287232
ENSDARG00000117504	CABZ01072084.1	222.936112	0.745132108	0.14811095	5.03090492	4.88E-07	1.63E-05
ENSDARG00000114742	CABZ01072083.2	221.456144	-0.476890265	0.17074951	-2.79292317	0.00522341	0.03516464
ENSDARG00000102632	ubc	35006.2064	0.345050003	0.09569157	3.60585584	0.00031113	0.00396769
ENSDARG00000098683	zmiz2	2021.804	0.378147951	0.12107177	3.1233372	0.00178813	0.0155725
ENSDARG00000076005	piezo2a.2	1995.18834	0.346224251	0.12449329	2.78106757	0.00541805	0.03613242
ENSDARG00000095432	BX323074.2	313.388119	-0.885898196	0.18372747	-4.82180578	1.42E-06	4.18E-05

Table S1. DEGs of WT vs. *terfa+/-*

ENSDARG00000063701	rreb1a	2022.55791	0.524073873	0.09356186	5.60136213	2.13E-08	1.02E-06
ENSDARG00000071860	nrn1a	864.909228	-0.332523256	0.09709921	-3.42457211	0.00061577	0.00681127
ENSDARG00000045447	slc35g2b	827.948853	0.984949284	0.166374	5.92009141	3.22E-09	1.90E-07
ENSDARG00000019976	idi1	1820.51066	-0.271772313	0.09626098	-2.82328643	0.00475341	0.03267311
ENSDARG00000028000	pfkpa	4590.55667	0.343446188	0.0884429	3.88325323	0.00010307	0.0016011
ENSDARG00000045442	cpb1	1733.88975	-2.003236234	0.52686028	-3.80221531	0.00014341	0.00211036
ENSDARG00000011821	plod2	1585.88314	0.32504777	0.11209426	2.89977163	0.00373435	0.02733132
ENSDARG00000034883	acbd5a	696.406967	-0.461297236	0.13293812	-3.47001479	0.00052043	0.00595273
ENSDARG00000011640	syt5b	3532.15768	-0.539559897	0.16295657	-3.31106569	0.00092941	0.00934116
ENSDARG00000008164	btr33	55.0776038	-0.854907646	0.29008392	-2.94710457	0.00320765	0.02432898
ENSDARG00000104791	CABZ01077956.1	25.7431398	1.99574949	0.50507592	3.95138515	7.77E-05	0.00126307
ENSDARG00000045414	elovl2	165.032714	-0.841684327	0.27980762	-3.00808217	0.00262902	0.02101707
ENSDARG00000078261	nphp3	601.640205	0.663674911	0.1402969	4.7305032	2.24E-06	6.15E-05
ENSDARG00000090230	tmem108	648.49659	-0.345478017	0.12633578	-2.73460149	0.00624558	0.04006038
ENSDARG00000063573	vps41	1299.79024	0.379911568	0.1002631	3.78914647	0.00015117	0.00220365
ENSDARG00000071735	prlh2	77.3202333	1.078280437	0.29761824	3.62303214	0.00029117	0.00376397
ENSDARG00000094422	si:dkey-12l12.1	410.443031	-0.819375717	0.14251649	-5.74933963	8.96E-09	4.79E-07
ENSDARG00000005220	pdcd6	1111.06313	0.284553744	0.10022429	2.8391694	0.00452311	0.03149075
ENSDARG00000041828	si:dkeyp-28d2.4	16.4229983	-2.717336033	0.58252832	-4.66472778	3.09E-06	8.08E-05
ENSDARG00000026801	stau2	10369.7637	0.385687995	0.08559711	4.50585321	6.61E-06	0.00015663
ENSDARG00000058730	rdh10a	2605.76541	-0.226940138	0.06842134	-3.31680356	0.00091054	0.00922682
ENSDARG00000019137	tram1	1344.72756	0.397979665	0.12876049	3.09085248	0.00199583	0.0170001
ENSDARG00000058601	gdap1	395.128751	-0.374302347	0.1342823	-2.78742887	0.00531281	0.03564249
ENSDARG00000032098	c8g	497.036822	-0.53265677	0.19332868	-2.75518756	0.00586585	0.03833702
ENSDARG00000077318	CR376737.1	544.38544	-0.505577344	0.15461029	-3.27001088	0.00107543	0.01044137
ENSDARG00000045402	commd3	372.605653	0.451283178	0.14458892	3.12114623	0.00180149	0.01564438
ENSDARG00000097929	BX005392.3	132.79578	5.019557133	0.81806941	6.13585726	8.47E-10	5.69E-08
ENSDARG00000018491	pdia4	3972.25214	0.962648762	0.09304231	10.3463548	4.35E-25	1.93E-22
ENSDARG00000018637	sec61g	398.550278	1.270234598	0.26623531	4.77109738	1.83E-06	5.18E-05
ENSDARG00000058966	zgc:112332	236.963831	1.096584261	0.2715569	4.03813805	5.39E-05	0.00093188
ENSDARG00000063474	arfgef1	2543.73994	0.30628242	0.08698301	3.52117508	0.00042964	0.00513645
ENSDARG00000038428	sulf1	2585.9511	0.255217834	0.08784947	2.90517231	0.00367051	0.02702326
ENSDARG00000038429	csrnp1b	1825.51988	0.337054409	0.09368131	3.59788306	0.00032082	0.00406833
ENSDARG00000091249	CR381686.1	22.5024323	1.466399145	0.5536553	2.64857782	0.00808312	0.04852803
ENSDARG00000002396	cry-dash	977.398484	0.683845726	0.12577646	5.43699297	5.42E-08	2.35E-06
ENSDARG00000059097	nktr	2741.82317	-0.484919928	0.09505641	-5.10139113	3.37E-07	1.19E-05
ENSDARG00000059058	vipr1b	86.5422359	-0.78679474	0.22631022	-3.47662041	0.00050778	0.00584103
ENSDARG00000016360	kpna1	2525.7211	0.275672123	0.08431307	3.26962489	0.0010769	0.01045154
ENSDARG00000076318	atp6v1ab	2915.82281	0.369476396	0.10399953	3.55267373	0.00038134	0.00465985
ENSDARG00000071579	urad	100.980976	-1.028938451	0.28881258	-3.5626511	0.00036713	0.00450839
ENSDARG00000058318	c1qtnf9	321.64291	-0.565220432	0.1916646	-2.949008	0.00318796	0.02423465
ENSDARG00000032272	sat1b	221.367188	-0.429411796	0.16223829	-2.64679689	0.00812581	0.04865505
ENSDARG00000026444	apoob	283.546894	0.47160595	0.16753941	2.81489563	0.00487931	0.03335401
ENSDARG00000058357	ankrd33ba	330.267316	-0.986625546	0.17721375	-5.56743238	2.59E-08	1.22E-06
ENSDARG00000062415	ctnnd2a	1041.87395	0.511339789	0.15453441	3.30890565	0.00093661	0.00938691
ENSDARG00000095252	si:dkey-40h20.1	58.0882363	2.332773994	0.48385829	4.8211926	1.43E-06	4.18E-05
ENSDARG00000075542	zfhx4	8672.00692	0.46408754	0.14679448	3.16147824	0.00156971	0.01404934
ENSDARG00000062460	SGK3	691.710974	0.410251204	0.12623896	3.24979863	0.00115487	0.01104434
ENSDARG00000001829	zgc:112982	2722.68763	-0.246427199	0.08356392	-2.94896658	0.00318838	0.02423465
ENSDARG00000057912	eif1axb	3965.16707	-0.314702998	0.07899342	-3.98391421	6.78E-05	0.00112869
ENSDARG00000058158	trim55b	292.356726	0.850177536	0.15540517	5.47071584	4.48E-08	2.00E-06
ENSDARG00000086254	hhla2b.2	519.10085	-0.526251606	0.14448435	-3.64227407	0.00027024	0.00355501
ENSDARG00000088514	and1	23024.2152	0.420015317	0.15810832	2.65650355	0.00789556	0.04773636
ENSDARG00000010244	rpl22l1	4944.9705	0.520391361	0.09781977	5.3198998	1.04E-07	4.23E-06
ENSDARG00000062087	nceh1b.1	115.177609	-0.635106273	0.23354841	-2.71937744	0.00654049	0.04145982
ENSDARG00000062146	larp4b	1320.24408	-0.300820074	0.08886676	-3.38506836	0.00071161	0.00761384
ENSDARG00000057249	zmynd11	3963.15947	-0.378288388	0.08035526	-4.70769917	2.51E-06	6.80E-05
ENSDARG00000093608	ccl34b.1	173.699429	-0.80051477	0.23442739	-3.41476644	0.00063837	0.00700204

Table S1. DEGs of WT vs. *terfa+/-*

ENSDARG00000057169	abca4a	440.689044	-0.80056733	0.17556378	-4.55998006	5.12E-06	0.00012565
ENSDARG00000100352	aglb	2599.96907	-1.003011079	0.12213284	-8.21246024	2.17E-16	4.41E-14
ENSDARG00000106630	LO018627.1	539.135087	-1.077031559	0.21723877	-4.9578239	7.13E-07	2.27E-05
ENSDARG00000109869	zgc:174719	14.0120437	2.020321431	0.70407327	2.86947613	0.00411152	0.02940775
ENSDARG00000099358	hccsb	1158.99664	0.306678704	0.09813132	3.1251869	0.00177692	0.01549662
ENSDARG00000102899	cremb	39.2682103	1.087984309	0.39379629	2.76280992	0.00573061	0.03769595
ENSDARG00000029445	eif1b	2596.56707	-0.401765108	0.08340599	-4.81698142	1.46E-06	4.24E-05
ENSDARG00000075192	yme1l1a	1213.69448	0.540000283	0.10314672	5.23526362	1.65E-07	6.34E-06
ENSDARG00000089645	si:ch1073-406l10.2	231.758766	0.498086382	0.18572264	2.68188293	0.00732091	0.04514045
ENSDARG00000052330	slc4a2b	1157.94975	0.311129069	0.10012092	3.10753307	0.00188656	0.01621384
ENSDARG00000056683	cdk5	512.249389	0.528711369	0.13500967	3.9161	9.00E-05	0.00142742
ENSDARG00000087822	agap3	1201.54606	0.448984304	0.1530479	2.93361954	0.00335035	0.02515796
ENSDARG00000102441	ctnna1	7054.96692	0.268865373	0.07444116	3.61178395	0.0003041	0.00389606
ENSDARG00000076559	rb1cc1	2695.50375	-0.289586049	0.07875211	-3.67718456	0.00023582	0.00317423
ENSDARG00000031015	dtna	656.337708	-0.422768437	0.13557093	-3.11842994	0.00181817	0.01574589
ENSDARG00000114991	tmem14cb	110.679551	-0.827111403	0.26297997	-3.14514983	0.00166002	0.01470388
ENSDARG00000077057	esco1	688.827294	-0.327366829	0.09417248	-3.4762474	0.00050848	0.00584567
ENSDARG00000042751	riok3	1886.23684	-0.380940295	0.10176809	-3.74321942	0.00018168	0.00255841
ENSDARG00000097264	si:ch211-40k21.5	242.564646	-0.411524516	0.1509496	-2.7262379	0.00640608	0.04084739
ENSDARG00000030156	naglu	431.351152	0.39468834	0.14445909	2.73218072	0.00629166	0.04024968
ENSDARG00000056502	si:ch73-334d15.4	392.909914	-0.815009131	0.22953715	-3.55066322	0.00038426	0.00468637
ENSDARG00000092662	dnaaf3	12.8664877	6.49259087	1.37119817	4.73497631	2.19E-06	6.04E-05
ENSDARG00000117271	CR954298.1	22.9291284	7.377609357	1.38261149	5.33599597	9.50E-08	3.92E-06
ENSDARG00000095214	CU469584.1	29.6856932	1.198591435	0.36661332	3.26936141	0.00107791	0.01045312
ENSDARG00000025507	wdr24	770.841462	0.5011759	0.14065824	3.5630753	0.00036654	0.00450779
ENSDARG00000030367	metrn	1004.18863	-0.370485988	0.12393945	-2.9892499	0.00279663	0.02200574
ENSDARG00000071454	crp1	47.1082071	-1.314653985	0.32545742	-4.03940393	5.36E-05	0.00092751
ENSDARG00000042613	crp3	240.56135	2.247021024	0.53579637	4.19379662	2.74E-05	0.0005257
ENSDARG00000093671	BX001030.1	739.442033	1.10937982	0.22502971	4.92992597	8.23E-07	2.57E-05
ENSDARG00000021265	mybpc2b	5362.80226	0.452173693	0.10941691	4.13257583	3.59E-05	0.00065947
ENSDARG00000071463	zgc:154125	16.4808996	-1.936598548	0.62438607	-3.10160434	0.00192475	0.01648114
ENSDARG00000087102	si:ch1073-164k15.3	52.6456069	-1.613255971	0.46664775	-3.45711722	0.00054599	0.00617122
ENSDARG00000077487	spsb3b	29.0965774	-1.441156628	0.45693443	-3.15396814	0.00161067	0.01435908
ENSDARG00000070957	im:7160594	492.047652	0.648226339	0.18024864	3.59628969	0.00032279	0.00408498
ENSDARG00000026281	lyrm1	111.360409	0.815153731	0.27644781	2.94867132	0.00319143	0.0242504
ENSDARG00000067859	scospondin	579.679448	1.486161187	0.24979905	5.94942694	2.69E-09	1.62E-07
ENSDARG00000101687	CU929259.1	857.591006	0.663641405	0.17083625	3.88466377	0.00010247	0.00159382
ENSDARG00000114031	smyhc3	612.804885	1.615208561	0.17571793	9.19205305	3.85E-20	1.20E-17
ENSDARG00000114818	CU633479.5	342.97243	1.637297325	0.19080846	8.58084244	9.42E-18	2.34E-15
ENSDARG00000103837	CU633479.2	175.634662	1.191324327	0.24989588	4.76728275	1.87E-06	5.26E-05
ENSDARG00000061417	nub1	162.773997	0.791529409	0.21792692	3.63208646	0.00028114	0.00366809
ENSDARG00000030411	crygn2	15500.8408	-0.843302765	0.15389367	-5.47977554	4.26E-08	1.91E-06
ENSDARG00000029710	lrrc30a	101.445831	0.780067209	0.22621421	3.44835631	0.00056401	0.00633173
ENSDARG00000101316	ppp4r1	1273.38791	0.450686159	0.09521227	4.73348838	2.21E-06	6.07E-05
ENSDARG00000101627	ssr1	5489.16275	0.27510295	0.07773282	3.53908359	0.00040152	0.0048516
ENSDARG00000086848	atad3	1179.62155	-0.543072926	0.13650351	-3.97845396	6.94E-05	0.00115091
ENSDARG00000075768	sdhb	6092.77311	0.409495899	0.11982446	3.41746508	0.00063207	0.00694525
ENSDARG00000102687	CABZ01085139.1	316.277554	-0.45529654	0.16012616	-2.84336141	0.00446404	0.03122819
ENSDARG00000115428	CABZ01101813.1	567.116822	0.773932086	0.1145616	6.75559787	1.42E-11	1.31E-09
ENSDARG00000112931	ndufaf3	1553.26553	0.385040262	0.11487685	3.35176561	0.00080298	0.00835765
ENSDARG00000040401	slc25a20	3466.01432	0.295494005	0.0902537	3.27403758	0.00106023	0.0103118
ENSDARG00000071558	fblim1	558.434188	0.847185064	0.14845509	5.70667592	1.15E-08	6.02E-07
ENSDARG00000020298	btg2	2409.97396	-0.692523876	0.17183041	-4.03027535	5.57E-05	0.00095828
ENSDARG00000046002	necap2	1880.50539	0.367804883	0.10116171	3.63581108	0.00027711	0.00362882
ENSDARG00000090145	tmem240b	295.510073	1.031339229	0.20238055	5.09603934	3.47E-07	1.22E-05
ENSDARG00000063661	nuak2	648.581176	0.460979022	0.14254089	3.23401261	0.00122064	0.01154014
ENSDARG00000076554	cdkn1a	1017.96069	2.085119699	0.17719141	11.7676117	5.73E-32	4.89E-29
ENSDARG00000077982	elf3	668.299729	-0.590381016	0.14974107	-3.94267919	8.06E-05	0.00130047

Table S1. DEGs of WT vs. *terfa+/-*

ENSDARG00000091131	cry3b	1867.38591	-0.883522456	0.11021597	-8.01628356	1.09E-15	2.01E-13
ENSDARG00000101567	guca1e	54.7241885	-1.250785509	0.38819103	-3.22208765	0.0012726	0.01191814
ENSDARG00000104894	si:ch73-138e16.2	163.097232	-0.578735911	0.18830774	-3.07335177	0.00211669	0.01778103
ENSDARG00000103078	zgc:174222	31.7669032	-1.126527649	0.35774458	-3.14897199	0.00163846	0.01456932
ENSDARG00000104065	znf1154	182.251846	0.452863547	0.16246029	2.7875338	0.00531109	0.03564249
ENSDARG00000104613	znf1179	1925.03693	1.855482895	0.28959436	6.40717908	1.48E-10	1.15E-08
ENSDARG00000099428	si:dkey-15h8.17	155.1516	2.350493528	0.28823932	8.15465956	3.50E-16	6.69E-14
ENSDARG00000102606	znf1158	109.973133	-0.652545112	0.2245316	-2.90625064	0.00365788	0.02695425
ENSDARG00000086744	znf1152	74.7384888	0.695639511	0.25882077	2.68772679	0.00719402	0.04459038
ENSDARG00000094563	znf1144	135.236576	-0.73108686	0.19972262	-3.66051107	0.00025171	0.00335459
ENSDARG00000111899	zgc:171435	90.6473344	2.071629927	0.24797296	8.35425743	6.58E-17	1.42E-14
ENSDARG00000073821	znf1177	49.5392418	8.529706103	1.21214359	7.03687762	1.97E-12	2.10E-10
ENSDARG00000106808	CU856622.1	84.873967	-0.87970333	0.26497461	-3.31995334	0.00090032	0.00915238
ENSDARG00000039687	zgc:194221	219.477857	-0.8413285	0.25445872	-3.30634567	0.00094521	0.00945785
ENSDARG00000102765	lonp1	2110.73091	0.500480499	0.08337679	6.00263573	1.94E-09	1.21E-07
ENSDARG00000039164	mhc1uma	77.835592	-1.190785397	0.34102328	-3.49180091	0.00047978	0.00558802
ENSDARG00000059039	FO904903.1	117.039664	-1.330423525	0.31676903	-4.19997985	2.67E-05	0.00051552
ENSDARG00000099675	kdm4b	6325.01228	0.350692171	0.10679115	3.28390677	0.00102379	0.01003005
ENSDARG00000098237	fbn2b	17621.2582	0.474939337	0.15739797	3.01744265	0.00254917	0.02053799
ENSDARG00000058984	rad23ab	555.687449	-0.424981056	0.14148857	-3.0036424	0.00266769	0.02125405
ENSDARG00000103979	calr3a	6278.12516	0.266696177	0.08525005	3.12839902	0.00175761	0.0153768
ENSDARG00000076264	zgc:195170	56.9105762	-3.32344029	0.44435094	-7.47931416	7.47E-14	9.98E-12
ENSDARG00000086457	tnfaip8l1	510.406658	-0.288587032	0.10179173	-2.83507344	0.00458152	0.03177276
ENSDARG00000063412	cers1	612.971942	-0.502881841	0.11934473	-4.21369137	2.51E-05	0.00049014
ENSDARG00000043242	si:dkey-222f2.1	1613.83559	-0.338497488	0.10518703	-3.21805352	0.00129064	0.01205516
ENSDARG00000092124	cox14	615.331627	0.498398469	0.12826942	3.88555962	0.00010209	0.00158895
ENSDARG00000071662	si:rp71-36a1.3	41.0935339	-1.684334752	0.48775443	-3.4532434	0.00055389	0.006252
ENSDARG00000058794	si:dkey-19a16.2	138.536681	-1.332469452	0.26785041	-4.97467763	6.54E-07	2.10E-05
ENSDARG00000093862	si:ch211-274k16.2	47.1425212	1.613169246	0.35982892	4.48315623	7.35E-06	0.00017229
ENSDARG00000095907	AL928685.2	288.423098	-0.626806972	0.18097697	-3.46346263	0.00053327	0.00606611
ENSDARG00000071648	zgc:113298	101.841045	0.684882661	0.23927245	2.86235482	0.00420506	0.02994108
ENSDARG00000071644	si:dkey-19a16.7	425.34931	0.697820919	0.15595762	4.47442662	7.66E-06	0.00017814
ENSDARG00000100000	CR352226.5	477.683689	0.923759538	0.1305916	7.07365199	1.51E-12	1.67E-10
ENSDARG00000071643	zgc:171490	154.303401	0.826784156	0.29726659	2.78128848	0.00541436	0.03611753
ENSDARG00000090847	si:ch211-209l18.4	623.128624	1.011807612	0.15695804	6.44635742	1.15E-10	9.04E-09
ENSDARG00000110288	CT583625.5	24.3472258	2.202979085	0.56537524	3.89649023	9.76E-05	0.0015266
ENSDARG00000074085	si:ch1073-188e1.1	44.096039	2.208220354	0.46940433	4.70430332	2.55E-06	6.90E-05
ENSDARG00000117742	FO904898.6	4.08364602	4.694103032	1.69312519	2.77244888	0.00556363	0.03693481
ENSDARG00000090167	FO904898.1	4.09178448	4.561328667	1.51156089	3.01762813	0.00254761	0.02053208
ENSDARG00000071554	CU929149.1	7.33795821	3.793683603	1.12569305	3.37008707	0.00075144	0.00792935
ENSDARG00000058753	si:ch73-213k20.5	35.0700072	1.724607122	0.45173389	3.81775015	0.00013467	0.00201282
ENSDARG00000075027	fgfr1bl	249.439404	0.988966696	0.21274486	4.64860436	3.34E-06	8.59E-05
ENSDARG00000043168	cela1.5	10.1806029	-2.650647297	0.80705891	-3.28432941	0.00102225	0.01002698
ENSDARG00000099364	BX511034.5	66.0629828	2.241899084	0.4764036	4.70588192	2.53E-06	6.85E-05
ENSDARG00000071629	BX511034.1	76.4818073	1.480029683	0.46772122	3.16434155	0.00155434	0.01393191
ENSDARG00000013022	si:ch211-59h6.1	61.9781625	1.179915397	0.42010611	2.80861281	0.00497554	0.03388141
ENSDARG00000077115	si:ch73-44m9.1	27.8468766	1.76685777	0.51223348	3.44932111	0.000562	0.00632057
ENSDARG00000094821	si:ch73-44m9.2	29.9660011	1.33834447	0.44175141	3.02963261	0.00244851	0.01988817
ENSDARG00000079645	sc:d217	205.302811	1.88911608	0.32036346	5.89679017	3.71E-09	2.15E-07
ENSDARG00000099511	CABZ01034698.2	158.878538	1.948884731	0.26359243	7.39355362	1.43E-13	1.83E-11
ENSDARG00000099023	CABZ01034698.1	16.5872894	2.520385763	0.83095973	3.03310218	0.00242054	0.01971246
ENSDARG00000077415	zmp:0000001175	30.336616	2.212185047	0.62415055	3.54431322	0.00039364	0.00477488
ENSDARG00000093024	si:ch211-213a13.2	95.6303932	1.349626237	0.28207492	4.78463747	1.71E-06	4.87E-05
ENSDARG00000074546	si:ch211-213a13.2	244.609417	1.149736961	0.2044789	5.62276564	1.88E-08	9.19E-07
ENSDARG00000071604	si:ch211-156p11.1	12.4248463	2.138016755	0.8040291	2.65912857	0.00783431	0.04743774
ENSDARG00000071618	si:ch211-213a13.5	8.38787047	2.917320778	0.93426883	3.12257103	0.00179279	0.01558887
ENSDARG00000068749	si:ch211-250k18.5	20.3857974	1.724164804	0.57617625	2.99242602	0.0027677	0.02183152
ENSDARG00000093964	si:ch211-250k18.6	15.1953598	2.07904745	0.68533429	3.03362531	0.00241634	0.01968476

Table S1. DEGs of WT vs. *terfa+/-*

ENSDARG00000093761	si:ch211-250k18.7	15.8153048	2.373171068	0.67957037	3.49216382	0.00047912	0.00558304
ENSDARG00000077068	si:ch211-11p18.6	28.9303142	4.701655754	0.87052856	5.40092074	6.63E-08	2.84E-06
ENSDARG00000093402	si:dkey-286j17.4	234.09002	-1.982081468	0.47542023	-4.16911471	3.06E-05	0.00057447
ENSDARG00000043131	BX664625.1	16.4438037	-3.115622712	0.69644847	-4.47358685	7.69E-06	0.00017867
ENSDARG00000103309	BX664625.5	14.1035736	4.3958847	1.03484512	4.24786727	2.16E-05	0.00043016
ENSDARG00000071590	si:ch211-236g6.1	130.295271	-2.146044061	0.26688996	-8.040932	8.92E-16	1.65E-13
ENSDARG00000071589	si:dkey-253d23.2	221.768136	-0.521210251	0.17843189	-2.92105996	0.00348843	0.02602186
ENSDARG00000095643	si:dkey-253d23.3	211.392806	-1.142377412	0.18966823	-6.02302973	1.71E-09	1.09E-07
ENSDARG00000043126	blf	87.8119059	-0.714324703	0.239602	-2.98129691	0.0028703	0.0224203
ENSDARG00000116843	BX324216.4	8.20259367	-2.948830834	1.06392663	-2.77164867	0.00557732	0.03697634
ENSDARG00000109202	BX324216.3	125.948055	-0.696958794	0.23784158	-2.93034879	0.00338582	0.02538597
ENSDARG00000071578	si:ch211-222k6.3	255.470756	-0.578615187	0.15821174	-3.65722021	0.00025497	0.00339249
ENSDARG00000058537	si:dkey-102c8.2	55.0177692	1.952144294	0.44060584	4.43059107	9.40E-06	0.00021196
ENSDARG00000002917	gls2b	87.86075	-1.194618886	0.27902624	-4.28138545	1.86E-05	0.00037808
ENSDARG00000008306	rdh5	196.308676	-0.981847913	0.21867866	-4.48991183	7.13E-06	0.00016787
ENSDARG00000021346	pdhb	8505.89925	0.395490516	0.08460066	4.67479256	2.94E-06	7.77E-05
ENSDARG00000043095	kctd6a	606.917326	-0.386950597	0.12569987	-3.078369	0.00208137	0.01754019
ENSDARG00000043077	nisch	1258.16079	-0.380328928	0.09193958	-4.13672676	3.52E-05	0.00065006
ENSDARG00000044132	ogna	2009.64147	-0.393411501	0.13078856	-3.00799629	0.00262976	0.02101707
ENSDARG00000071549	ecm2	976.309451	0.508025925	0.10424838	4.87322608	1.10E-06	3.36E-05
ENSDARG00000008049	si:dkey-42i9.4	1208.89608	-0.40813071	0.13000084	-3.13944681	0.00169267	0.01491892
ENSDARG00000004840	rassf1	872.877022	-0.495130003	0.11110493	-4.45641806	8.33E-06	0.00019074
ENSDARG00000029841	tmprss9	540.084519	-0.583175072	0.19552163	-2.98266265	0.00285753	0.02235046
ENSDARG00000071518	krt222	519.121176	-0.492745581	0.16788624	-2.9349968	0.00333551	0.02507687
ENSDARG00000062865	mras	1159.13597	-0.400456503	0.10409649	-3.84697424	0.00011959	0.00181792
ENSDARG00000041602	zgc:92335	680.429793	0.323783069	0.12111136	2.67343262	0.00750794	0.04596299
ENSDARG00000057975	plcd4a	148.037657	-0.59615578	0.19633955	-3.03635098	0.0023946	0.01957182
ENSDARG00000071475	aox5	1520.46571	0.667806533	0.1270064	5.258054	1.46E-07	5.69E-06
ENSDARG00000010481	bzw1a	5060.32827	-0.223123436	0.0819372	-2.72310296	0.00646719	0.04116313
ENSDARG00000042900	gtpbp1l	898.794883	0.441353448	0.12820038	3.44268427	0.00057597	0.00642827
ENSDARG00000024588	gpc5b	477.365995	0.370941758	0.12353117	3.00281902	0.00267491	0.02129564
ENSDARG00000087697	lpl	2473.11748	-0.403275633	0.15231076	-2.64771598	0.00810376	0.0485815
ENSDARG00000020467	safb	6274.4269	0.363201278	0.10133116	3.58430003	0.00033798	0.00422686
ENSDARG00000042667	klf2a	2799.26817	0.964311801	0.3311561	2.91195543	0.00359174	0.02660091
ENSDARG00000071462	lama3	400.150338	-0.56441034	0.20563836	-2.74467444	0.0060571	0.0392055
ENSDARG00000057159	ankrd29	152.297789	-0.542405109	0.18216665	-2.97752153	0.00290589	0.02265557
ENSDARG00000013928	plekhb2	705.739348	0.593270861	0.11508684	5.15498457	2.54E-07	9.31E-06
ENSDARG00000024964	patj	1656.73755	0.308433132	0.11406156	2.70409346	0.0068491	0.04297764
ENSDARG00000002293	si:ch211-197g15.6	186.241178	1.037074704	0.24422382	4.24641091	2.17E-05	0.00043227
ENSDARG00000061735	hnrnpm	12209.6022	0.603817788	0.1405585	4.2958467	1.74E-05	0.0003579
ENSDARG00000016188	SBNO2	1591.40974	0.596468684	0.16534513	3.60741614	0.00030926	0.00394594
ENSDARG00000005783	ncanb	1390.16152	0.808308743	0.18395661	4.39401846	1.11E-05	0.00024672
ENSDARG00000013351	cirbpb	63432.0271	-0.70880294	0.11110437	-6.3796134	1.78E-10	1.35E-08
ENSDARG00000092788	si:dkey-21e2.15	48.1178256	-1.243279301	0.46217831	-2.69004251	0.00714429	0.04435957
ENSDARG00000042065	eef2a.2	71.7423952	-0.884393943	0.29270153	-3.02148724	0.00251536	0.02031828
ENSDARG00000101128	si:dkey-211f22.5	579.862855	-0.403980655	0.14094751	-2.86617797	0.00415461	0.02964764
ENSDARG00000056483	ssbp4	5105.57985	0.369963086	0.12135929	3.04849409	0.00229991	0.0189978
ENSDARG00000021432	odf3l2b	7.74071961	-2.525893407	0.91901857	-2.74846831	0.00598744	0.03885584
ENSDARG00000071410	cks2	135.990421	-0.897623754	0.21995493	-4.08094402	4.49E-05	0.0007963
ENSDARG00000008767	tle2b	772.9487	0.46466446	0.140322	3.31141562	0.00092825	0.00933699
ENSDARG00000071424	ap3d1	3965.59636	0.281138974	0.09295594	3.02443254	0.002491	0.02017394
ENSDARG00000100940	nr5a2	687.612046	0.308305806	0.09917659	3.10865499	0.00187941	0.01616918
ENSDARG00000071437	ptprc	104.714476	-1.319898594	0.26166309	-5.04426744	4.55E-07	1.54E-05
ENSDARG00000103754	aspm	2928.9429	-0.649583035	0.18698604	-3.47396541	0.00051283	0.00588201
ENSDARG00000100442	cfh	4680.94845	0.468745033	0.10700897	4.38042731	1.18E-05	0.00026053
ENSDARG00000103760	cfhl2	1027.33964	1.525251606	0.14122557	10.8001098	3.44E-27	1.82E-24
ENSDARG00000103026	p3h2	630.843991	-0.493210451	0.11416072	-4.32031664	1.56E-05	0.00032556
ENSDARG00000041703	rrbp1b	4080.36061	0.440419951	0.10751958	4.0961838	4.20E-05	0.00075132

Table S1. DEGs of WT vs. *terfa+/-*

ENSDARG00000097229	si:ch211-226h8.15	3.29229945	3.812255976	1.43690653	2.65309948	0.00797564	0.04804518
ENSDARG00000088923	si:ch211-12h2.8	11.9350929	3.081815925	0.87628755	3.51690026	0.00043662	0.00519987
ENSDARG00000099266	vasna	1777.91265	-0.260076991	0.08968386	-2.89993088	0.00373245	0.0273285
ENSDARG00000110069	pdgfaa	175.230826	-0.685868576	0.2281339	-3.00642988	0.00264335	0.02109842
ENSDARG00000109371	pdgfaa	119.706142	1.734494549	0.22952448	7.55690434	4.13E-14	5.76E-12
ENSDARG00000001818	c3b.2	2029.43369	-0.607078917	0.13947131	-4.35271553	1.34E-05	0.00028961
ENSDARG00000093068	c3b.1	3231.44213	0.453266883	0.12538896	3.61488678	0.00030048	0.00385765
ENSDARG00000055592	capn2b	1776.21924	0.248679797	0.09183296	2.70795804	0.00676986	0.04257713
ENSDARG00000034211	capn2l	1293.19841	0.861253939	0.15525244	5.54744225	2.90E-08	1.35E-06
ENSDARG00000117646	CABZ01072309.2	2457.09894	-0.374708551	0.10829639	-3.46002811	0.00054012	0.00612718
ENSDARG00000100869	adcy9	1006.79027	0.315467228	0.11796929	2.67414707	0.00749196	0.0458991
ENSDARG00000055100	cxcl12b	1297.52645	-0.469359195	0.09351426	-5.01911919	5.19E-07	1.72E-05
ENSDARG00000071143	si:dkey-208m12.2	162.387075	1.294180321	0.2395796	5.4018803	6.59E-08	2.83E-06
ENSDARG00000091937	BX927308.1	406.402152	-0.567225951	0.16844453	-3.36743464	0.00075871	0.00799057
ENSDARG00000103860	wu:fu71h07	88.6540483	-0.668662034	0.22188608	-3.01353757	0.00258221	0.02074365
ENSDARG00000071103	si:dkey-222p3.1	28.9508349	-3.501964874	0.47835559	-7.32084032	2.46E-13	3.06E-11
ENSDARG00000071095	abi3bpb	3907.95551	-0.330442748	0.10118649	-3.26568041	0.00109201	0.01056104
ENSDARG00000054858	tp53bp2b	1397.44266	0.301226133	0.09514122	3.16609488	0.001545	0.01387322
ENSDARG00000060933	baiap2l2b	82.472901	-0.886989421	0.25396718	-3.49253558	0.00047846	0.00558051
ENSDARG00000040853	shoc2	1669.4925	-0.288925346	0.10062157	-2.87140569	0.00408651	0.02930558
ENSDARG00000019307	dusp5	357.228242	-0.561518974	0.18492285	-3.03650396	0.00239339	0.01956832
ENSDARG00000054818	rpl32	21449.1165	0.329579044	0.10008683	3.29293128	0.00099149	0.00980768
ENSDARG00000054807	sec13	3225.47204	0.404036429	0.11735985	3.44271432	0.00057591	0.00642827
ENSDARG00000063177	manf	1048.31036	0.566680158	0.11590524	4.88916769	1.01E-06	3.11E-05
ENSDARG00000099446	slit1b	2078.65131	0.381930836	0.12540643	3.04554437	0.0023226	0.01914779
ENSDARG00000075846	kcnip2	34.3871803	-0.950086699	0.35794983	-2.65424546	0.0079486	0.0479796
ENSDARG00000003952	pfn2	13558.838	0.299816268	0.08293054	3.61526978	0.00030004	0.00385394
ENSDARG00000041839	tsc22d2	1846.05142	0.285876333	0.08566023	3.33732861	0.00084588	0.00870212
ENSDARG00000002710	ncl	20825.4218	-0.295265903	0.08160039	-3.6184374	0.00029639	0.00381299
ENSDARG00000101094	ATP11B	1875.76673	0.272073659	0.08962047	3.03584295	0.00239864	0.01959194
ENSDARG00000044420	dnajc19	341.84982	0.773537035	0.16444542	4.70391359	2.55E-06	6.90E-05
ENSDARG00000013845	ttc14	1241.34576	-0.762472695	0.13503855	-5.64633339	1.64E-08	8.21E-07
ENSDARG00000115925	htr2b	46.9122998	-0.942135606	0.35547653	-2.65034543	0.00804095	0.04832153
ENSDARG00000100625	si:ch73-197b13.1	233.980766	-1.742958908	0.19582475	-8.90060582	5.55E-19	1.57E-16
ENSDARG00000010312	cp	4582.5662	-1.037298858	0.17432294	-5.95044365	2.67E-09	1.62E-07
ENSDARG00000101008	soul4	1625.55726	-0.339756338	0.12659	-2.68391132	0.00727664	0.04495672
ENSDARG00000068126	nppc	157.434289	-0.754778602	0.19716399	-3.82817669	0.0001291	0.00194331
ENSDARG00000015273	alpi.1	800.450926	0.551943423	0.10543022	5.23515381	1.65E-07	6.34E-06
ENSDARG00000053774	alpi.2	68.787705	2.0703353	0.7372041	2.80836108	0.00497944	0.03388934
ENSDARG00000004932	anos1b	1095.70278	0.471298862	0.09761888	4.82794775	1.38E-06	4.09E-05
ENSDARG00000089893	tasora	1904.03474	-0.376029144	0.11974898	-3.14014486	0.00168864	0.01489926
ENSDARG00000017658	gmppb	2236.99165	0.533252465	0.09459745	5.63707031	1.73E-08	8.56E-07
ENSDARG00000046157	RPS17	26497.7752	0.606803825	0.1109564	5.46884944	4.53E-08	2.01E-06
ENSDARG00000031774	pus7	998.96234	-0.327417549	0.10280338	-3.18489095	0.00144809	0.01317912
ENSDARG00000073905	vwa5a	1738.51583	0.361108279	0.11736108	3.07689981	0.00209166	0.01759922
ENSDARG00000040649	prickle1a	514.472059	0.327715299	0.12315775	2.66093941	0.0077923	0.04724739
ENSDARG00000088356	tmem117	91.2369708	0.667066195	0.22633225	2.94728737	0.00320575	0.02432202
ENSDARG00000103388	znf609a	1913.31254	0.325589882	0.11690069	2.78518358	0.00534974	0.03579215
ENSDARG00000045968	spg11	1739.90195	0.467618524	0.1324972	3.52927089	0.00041671	0.00499867
ENSDARG00000099798	mon2	3168.11896	0.238169531	0.08037379	2.96327351	0.00304386	0.0234299
ENSDARG00000010279	scamp2	1153.28841	-0.514560607	0.0906204	-5.67819854	1.36E-08	7.02E-07
ENSDARG00000007711	rab3il1	589.795025	0.447684605	0.13557404	3.30214118	0.0009595	0.00955938
ENSDARG00000071062	hps5	757.937038	0.440568433	0.13729894	3.20882608	0.00133278	0.01236536
ENSDARG00000071076	ldhbb	643.702793	-0.949776808	0.18415634	-5.15744822	2.50E-07	9.20E-06
ENSDARG00000098911	zgc:153293	179.529342	-0.692813225	0.17760336	-3.90090158	9.58E-05	0.00150568
ENSDARG00000069673	chid1	644.846389	0.615470801	0.13117338	4.69204032	2.70E-06	7.24E-05
ENSDARG00000053609	gatd1	571.8936	-0.369818134	0.12375309	-2.98835483	0.00280484	0.02204054
ENSDARG00000078785	tmem258	184.869065	1.214261092	0.25173192	4.82362786	1.41E-06	4.15E-05

Table S1. DEGs of WT vs. *terfa+/-*

ENSDARG00000074626	pidd1	228.922385	0.500261173	0.17618051	2.83948086	0.0045187	0.03146884
ENSDARG00000088844	deaf1	351.789932	0.519427582	0.1575605	3.29668657	0.00097833	0.00971034
ENSDARG00000038363	drd4a	182.048559	-1.107737451	0.30132614	-3.67620761	0.00023673	0.00318218
ENSDARG00000103684	ap4e1	486.051788	-0.377599722	0.12862466	-2.93567122	0.00332827	0.02502999
ENSDARG00000098360	cyp19a1b	34.5829591	-2.396170332	0.42982205	-5.57479616	2.48E-08	1.17E-06
ENSDARG00000097889	si:ch73-265h17.2	37.4364201	2.604824447	0.52971733	4.9173857	8.77E-07	2.72E-05
ENSDARG00000097639	si:ch73-265h17.4	46.2746051	1.604244674	0.35602585	4.50597805	6.61E-06	0.00015663
ENSDARG00000010183	si:ch73-265h17.1	69.2742572	0.957003966	0.33733282	2.83697261	0.00455435	0.03161967
ENSDARG00000086458	hdac10	400.888867	-0.783887399	0.15049935	-5.20857668	1.90E-07	7.20E-06
ENSDARG00000067711	parvg	90.5114857	0.791592579	0.24604964	3.21720688	0.00129445	0.01208172
ENSDARG00000101164	nansb	395.314798	0.74435837	0.13481772	5.52122072	3.37E-08	1.56E-06
ENSDARG00000092798	ppib	14608.4063	0.312693215	0.09838863	3.17814376	0.00148221	0.01344049
ENSDARG00000045928	psma4	3257.54872	-0.273804695	0.09407952	-2.9103538	0.0036102	0.02669785
ENSDARG00000104702	cat	4387.95574	-0.433718629	0.10730758	-4.04182675	5.30E-05	0.0009217
ENSDARG00000032650	fuk	591.844179	0.552818565	0.16145122	3.42405948	0.00061693	0.0068211
ENSDARG00000029232	FO704779.1	67.8055427	1.153285039	0.3282078	3.51388673	0.0004416	0.00524164
ENSDARG00000074468	prdm11	598.073911	-0.396135206	0.10915693	-3.62904298	0.00028447	0.00370188
ENSDARG00000021378	phf21ab	1009.27199	-0.327670232	0.11696181	-2.80151474	0.00508633	0.03448272
ENSDARG00000058556	muc5.2	2419.66638	0.897348116	0.16717938	5.36757638	7.98E-08	3.37E-06
ENSDARG00000060951	polg	483.185446	0.450693137	0.15181564	2.96868707	0.00299075	0.02313573
ENSDARG00000070971	furinb	1414.8313	0.510921288	0.15972537	3.19874844	0.00138026	0.01271544
ENSDARG00000046030	zgc:110339	2584.68212	0.513340541	0.12393216	4.14210914	3.44E-05	0.00063783
ENSDARG00000097573	si:ch211-107e6.5	5.03784935	4.439204147	1.46096786	3.03853648	0.0023773	0.019456
ENSDARG00000097285	ANPEP	422.089287	-0.434899412	0.15007432	-2.89789364	0.00375678	0.02745509
ENSDARG00000097352	si:ch211-147g22.7	10.1590077	6.614017468	1.33467062	4.95554286	7.21E-07	2.29E-05
ENSDARG00000104384	agbl1	506.680017	0.432936048	0.16057991	2.69607853	0.00701611	0.04373852
ENSDARG00000077228	ntrk3a	1250.98742	0.529100218	0.13631332	3.88150061	0.00010381	0.00161168
ENSDARG00000103814	CR450808.4	33.8176403	2.894423455	0.62008745	4.66776657	3.04E-06	7.99E-05
ENSDARG00000101336	CR450808.3	3.60735756	5.025287623	1.5074466	3.33364221	0.00085717	0.00880369
ENSDARG00000111882	si:ch211-194m7.4	3.82072743	5.220159679	1.55161264	3.36434464	0.00076726	0.00805813
ENSDARG00000103271	pla2g15	7823.61889	-0.417824979	0.10321113	-4.04825512	5.16E-05	0.0008994
ENSDARG00000099424	lcat	561.137612	-0.660070958	0.19295654	-3.42082707	0.00062431	0.00688125
ENSDARG00000102403	cry2	1339.81837	0.854812823	0.11077687	7.71652826	1.20E-14	1.87E-12
ENSDARG00000029168	ppfibp2b	1176.03747	-0.360754197	0.12512164	-2.88322795	0.00393622	0.02844864
ENSDARG00000043722	cpa4	563.225324	-2.382475922	0.47265849	-5.04058632	4.64E-07	1.56E-05
ENSDARG00000010146	cpa2	57.409444	0.985148111	0.33224139	2.96515769	0.00302528	0.02334449
ENSDARG00000070734	dyrk4	877.897503	-0.57984264	0.14130349	-4.10352662	4.07E-05	0.00073275
ENSDARG00000013784	zgc:77158	347.658796	0.548954111	0.15868945	3.45929815	0.00054159	0.00614101
ENSDARG00000051748	ccnd2a	1243.17235	0.322950305	0.11470509	2.81548368	0.00487039	0.03332053
ENSDARG00000070775	cyp2x9	244.508537	-0.698458567	0.24609048	-2.83821854	0.00453661	0.03154671
ENSDARG00000042107	cngb1b	517.610843	-0.903197873	0.18720232	-4.82471525	1.40E-06	4.14E-05
ENSDARG00000051962	mmp15a	1246.02394	-0.302477339	0.09491124	-3.18694945	0.00143782	0.01311931
ENSDARG00000045773	PYURF	84.927993	0.758348863	0.28397114	2.67051385	0.00757352	0.04623924
ENSDARG00000093738	pth1a	23.2636441	4.16453347	0.88224996	4.72035551	2.35E-06	6.42E-05
ENSDARG00000006791	arntl1a	1103.82432	-0.465501601	0.1219217	-3.81803739	0.00013452	0.00201169
ENSDARG00000070914	dusp6	5419.75324	0.269133473	0.09446708	2.84896569	0.00438616	0.03083961
ENSDARG00000030915	cpa1	109.078488	-3.187440174	0.36963583	-8.62319038	6.51E-18	1.67E-15
ENSDARG00000021339	cpa5	3353.05223	-2.236206751	0.54414398	-4.10958649	3.96E-05	0.00071649
ENSDARG00000051857	tes	1002.24351	0.583785862	0.12872593	4.5351071	5.76E-06	0.00013961
ENSDARG00000062960	armc10	415.151721	-0.314615131	0.11926652	-2.63791657	0.00834171	0.04970797
ENSDARG00000015732	cax2	337.306673	0.517632568	0.13610825	3.80309467	0.0001429	0.00210538
ENSDARG00000101725	parietopsin	218.231595	0.634218846	0.20630395	3.0741964	0.0021107	0.01774748
ENSDARG00000079783	isg20	904.699708	0.445038037	0.09882467	4.5033089	6.69E-06	0.00015814
ENSDARG00000007302	sh3gl3b	431.665401	-0.388144266	0.14582534	-2.6617066	0.00777456	0.04719452
ENSDARG00000045827	lyrm5b	27.1095059	1.440237606	0.53656477	2.68418222	0.00727075	0.04495069
ENSDARG00000045824	zgc:101783	637.25886	0.34250525	0.12133561	2.82279244	0.00476074	0.0327054
ENSDARG00000036671	tnni4b.2	248.269474	0.507998741	0.17427834	2.91487026	0.00355836	0.02645622
ENSDARG00000070826	bpgm	754.392884	-0.395593122	0.1150974	-3.43702904	0.00058813	0.00653649

Table S1. DEGs of WT vs. *terfa+/-*

ENSDARG00000077569	lrrn3a	493.008524	-0.422969705	0.1214313	-3.48320155	0.00049546	0.00571971
ENSDARG00000062168	tmem168b	209.48142	-0.49015329	0.18183045	-2.69566117	0.00702491	0.0437714
ENSDARG00000051861	pkp3a	4836.69575	0.465676751	0.09138343	5.09585529	3.47E-07	1.22E-05
ENSDARG00000117711	CR354549.11	23.5015309	-1.479296102	0.50786165	-2.91279347	0.00358211	0.02658488
ENSDARG00000070560	nap1l4a	2514.39754	-0.303980776	0.10521522	-2.8891331	0.00386306	0.02805032
ENSDARG00000062054	cpt1ab	4562.21863	-0.357322778	0.09270938	-3.85422464	0.0001161	0.00177139
ENSDARG00000088171	ciartb	432.499316	0.933989375	0.1570103	5.9485866	2.70E-09	1.63E-07
ENSDARG00000045633	asb15a	713.723963	-0.504229873	0.13538737	-3.72434951	0.00019582	0.00272819
ENSDARG00000045638	slc13a1	153.750604	-0.864595972	0.32206634	-2.68452762	0.00726324	0.04494095
ENSDARG00000051800	fnbp4	5252.16919	0.25721894	0.09572273	2.68712493	0.007207	0.0446263
ENSDARG00000061802	cnot2	2143.57377	0.27107071	0.09925398	2.73108143	0.00631269	0.04035541
ENSDARG00000045611	nfybb	771.877099	0.508169116	0.15700544	3.23663378	0.00120949	0.01146083
ENSDARG00000103002	nptna	2654.37528	-0.332759606	0.10352088	-3.21442017	0.00130708	0.01216946
ENSDARG00000013990	ube2q2	2545.10845	-0.25511475	0.08727286	-2.92318528	0.0034647	0.02587596
ENSDARG00000015709	hsd17b12a	1670.93996	1.080762878	0.09826282	10.9986963	3.88E-28	2.29E-25
ENSDARG00000102640	pdia3	7570.82642	0.347052508	0.07977192	4.35055981	1.36E-05	0.00029205
ENSDARG00000017562	banp	594.649797	0.407000961	0.14965942	2.71951448	0.00653778	0.04145982
ENSDARG00000101209	lamb1a	10220.6211	0.218814869	0.08207187	2.66613733	0.00767283	0.04670788
ENSDARG00000039133	lamb4	2742.58358	0.36678903	0.10198836	3.59638124	0.00032267	0.00408498
ENSDARG00000018593	parp16	706.642973	0.301129023	0.1012351	2.97455166	0.00293417	0.02282592
ENSDARG00000062632	duox	167.758645	1.546510733	0.36366539	4.25256511	2.11E-05	0.00042259
ENSDARG00000061587	ctdspl2a	1805.42318	-0.291438427	0.08810105	-3.30800173	0.00093964	0.00940967
ENSDARG00000023104	scaper	1492.07019	0.321166193	0.11327222	2.83534824	0.00457758	0.03176316
ENSDARG00000003216	anxa2a	5627.90652	1.064970394	0.20424942	5.21406814	1.85E-07	7.01E-06
ENSDARG00000100513	rps27l	1923.48099	1.047636439	0.14450542	7.24980735	4.17E-13	5.01E-11
ENSDARG00000051762	CABZ01080568.1	1128.61258	1.034348075	0.11278475	9.17099216	4.69E-20	1.42E-17
ENSDARG00000061357	chst6	399.093397	0.542868324	0.14232274	3.81434708	0.00013654	0.00203831
ENSDARG00000003084	spire2	258.626416	-0.477532467	0.16116852	-2.96293888	0.00304717	0.02344086
ENSDARG00000075513	ccdc136b	357.438447	-0.619956261	0.22957339	-2.70047085	0.00692414	0.04337185
ENSDARG00000076320	ano9a	259.78262	0.420101157	0.14544436	2.88839779	0.0038721	0.02807497
ENSDARG00000006169	lrrk2	1465.21363	1.083059981	0.15914591	6.80545261	1.01E-11	9.52E-10
ENSDARG00000045636	rbl2	1855.39646	0.630466251	0.10396348	6.06430507	1.33E-09	8.60E-08
ENSDARG00000051731	faap24	38.4244665	-1.316835954	0.35716057	-3.68695779	0.00022695	0.00307615
ENSDARG00000096603	bmb	667.445067	2.132414263	0.16968341	12.5670169	3.21E-36	3.98E-33
ENSDARG00000096626	CU914144.1	36.9182732	-1.19791485	0.36003506	-3.32721721	0.00087718	0.00897953
ENSDARG00000090108	si:ch1073-174d20.1	516.402963	0.777319697	0.20950295	3.71030424	0.00020701	0.00285213
ENSDARG00000079651	si:ch1073-174d20.2	2158.85356	-0.305098701	0.08169845	-3.73444909	0.00018813	0.00263876
ENSDARG00000070387	nudt7	139.334988	0.879980594	0.23281995	3.77966151	0.00015704	0.00227331
ENSDARG00000088079	tmed6	83.8625015	-1.316908766	0.28961689	-4.54707177	5.44E-06	0.00013281
ENSDARG00000102453	slc1a2b	22311.0238	0.442430302	0.14177102	3.12073868	0.00180398	0.01565024
ENSDARG00000089892	cd44b	42.153055	-1.199703604	0.39581588	-3.03096381	0.00243774	0.01980717
ENSDARG00000103220	slc10a3	1548.27805	0.373593461	0.09387356	3.97975157	6.90E-05	0.00114541
ENSDARG00000110103	FO834799.1	324.929828	0.836715844	0.18549413	4.5107404	6.46E-06	0.0001541
ENSDARG00000099362	CABZ01088346.1	346.839791	0.557845585	0.14494907	3.84856264	0.00011881	0.00181059
ENSDARG00000063895	mt-nd1	47735.592	-0.543648535	0.10997411	-4.94342287	7.68E-07	2.42E-05
ENSDARG00000063899	mt-nd2	42326.1411	-0.362030147	0.09290251	-3.8968822	9.74E-05	0.00152606
ENSDARG00000063911	mt-atp6	56641.0699	-0.405522087	0.10614312	-3.82052166	0.00013317	0.00199514
ENSDARG00000063916	mt-nd4l	5138.67437	-0.342538031	0.09458475	-3.62149306	0.00029291	0.00377409
ENSDARG00000063917	mt-nd4	41333.932	-0.381256934	0.10362865	-3.67906869	0.00023409	0.00315352
ENSDARG00000063921	mt-nd5	38631.3631	-0.5921942	0.09977289	-5.93542211	2.93E-09	1.74E-07
ENSDARG00000063922	mt-nd6	5509.07907	-0.486470911	0.17693473	-2.7494371	0.00596977	0.03877154
ENSDARG00000098527	CABZ01060491.1	12.5333313	3.065642034	0.80781955	3.79495897	0.00014767	0.00216408
ENSDARG00000101938	CABZ01060492.1	7.03824956	3.150123773	1.06606418	2.95491008	0.0031276	0.02389688
ENSDARG00000098332	zgc:112970	56.1132183	3.623053299	0.41583102	8.71280196	2.96E-18	7.84E-16
ENSDARG00000109381	CU457778.2	12.5076289	2.506336223	0.88577123	2.82955251	0.00466131	0.03220038
ENSDARG00000114529	CU457778.9	205.261184	1.473608609	0.26492239	5.56241621	2.66E-08	1.25E-06
ENSDARG00000099583	CABZ01034082.1	20.4275188	6.778907735	1.28484168	5.27606464	1.32E-07	5.23E-06
ENSDARG00000105095	AL929028.8	45.38144	3.621741864	0.6102866	5.93449352	2.95E-09	1.75E-07

Table S1. DEGs of WT vs. *terfa+/-*

ENSDARG00000101754	BX664622.1	93.0855287	1.929181371	0.36680986	5.25934976	1.45E-07	5.66E-06
ENSDARG00000113941	CU182823.3	11.7423889	3.598793978	0.96586723	3.7259717	0.00019456	0.00271872
ENSDARG00000105274	CABZ01074130.1	16566.7903	0.693764722	0.19281435	3.59809694	0.00032055	0.00406706
ENSDARG00000101592	col22a1	2418.11538	-0.441052939	0.12175609	-3.62243015	0.00029185	0.00376825
ENSDARG00000104424	CABZ01009512.2	12.6334813	1.885705117	0.67786054	2.7818482	0.00540503	0.03607467
ENSDARG00000034598	dicp1.16	5.66279104	-5.395067602	1.59312562	-3.38646716	0.00070799	0.0075882
ENSDARG00000103285	CCDC134	87.6938839	-3.065376782	1.09585712	-2.79724128	0.0051541	0.03479229
ENSDARG00000100188	rnps1	163.902703	0.914684207	0.33689548	2.71503852	0.00662681	0.04186742
ENSDARG00000102440	CABZ01070852.1	360.917653	1.554378225	0.22903685	6.78658588	1.15E-11	1.07E-09
ENSDARG00000114730	CABZ01113192.1	1225.26319	-0.284443164	0.10442908	-2.72379264	0.0064537	0.04108779
ENSDARG00000100332	CABZ01084942.1	46.9703414	1.589771612	0.4446574	3.57527305	0.00034986	0.00434115
ENSDARG00000103044	ASS1	718.290943	-0.463768775	0.14320749	-3.23843942	0.00120186	0.01141464
ENSDARG00000099006	CABZ01029938.1	158.151798	1.105508923	0.32031182	3.45135226	0.00055779	0.00628456
ENSDARG00000102421	acp7	248.118668	0.915278041	0.21048959	4.34832927	1.37E-05	0.00029393
ENSDARG00000104937	PTPRD	8671.72319	-0.455249588	0.14154925	-3.21619209	0.00129904	0.01211997
ENSDARG00000104672	CABZ01074397.1	200.430789	0.687449299	0.23045059	2.98306585	0.00285377	0.02233323
ENSDARG00000099419	CABZ01025780.1	26.2792735	-3.256395441	0.88910393	-3.66255882	0.00024971	0.00333504
ENSDARG00000101084	CABZ01069184.1	66.4946753	1.738784064	0.36214968	4.80128566	1.58E-06	4.55E-05
ENSDARG00000103775	thbs1a	2757.31451	0.472265339	0.17048318	2.7701579	0.00560291	0.03712083
ENSDARG00000098258	SLC16A7	522.141402	-0.57872392	0.19583972	-2.95508962	0.00312578	0.02389356
ENSDARG00000100562	CABZ01080702.1	2767.90894	0.508759269	0.13919009	3.65513997	0.00025704	0.00341646
ENSDARG00000099827	CABZ01000633.1	14.9642591	6.001519327	1.29022728	4.65152102	3.29E-06	8.48E-05
ENSDARG00000099574	CABZ01030017.2	15.0194841	-2.503290993	0.66818898	-3.74638175	0.0001794	0.00253356
ENSDARG00000100614	zgc:171497	120.870536	-1.034886383	0.21464636	-4.82135547	1.43E-06	4.18E-05
ENSDARG00000103230	CABZ01079302.1	18.7465385	1.613471406	0.52619525	3.0662979	0.00216727	0.01810674
ENSDARG00000102395	HAPLN1	167.041289	5.892186294	0.45691336	12.8956313	4.76E-38	7.40E-35
ENSDARG00000104040	CABZ01046088.1	57.374317	1.281420868	0.29339734	4.36752727	1.26E-05	0.00027349
ENSDARG00000102877	bphl	515.972808	-0.84529559	0.13077552	-6.46371402	1.02E-10	8.14E-09
ENSDARG00000101800	CABZ01088271.1	301.903063	-0.475585522	0.16694913	-2.84868527	0.00439003	0.03085808
ENSDARG00000117379	CABZ01088275.1	21.0682505	-1.732641624	0.58049161	-2.98478323	0.00283779	0.02225027
ENSDARG00000117513	CABZ01024844.1	4.16922516	4.72714159	1.69446896	2.78974812	0.00527491	0.03543454
ENSDARG00000102550	CABZ01080371.1	799.532852	-0.36382141	0.12746825	-2.85421195	0.00431437	0.0304536
ENSDARG00000100651	PDCL3	1373.49406	0.361857698	0.10621969	3.40669132	0.00065755	0.00715566
ENSDARG00000117659	CABZ01079280.1	80.9930052	-0.744788872	0.2637519	-2.82382377	0.00474545	0.03264547
ENSDARG00000117609	CABZ01079281.1	125.218613	-0.737652446	0.2657039	-2.77621989	0.0054995	0.03658728
ENSDARG00000117160	CABZ01034596.1	98.8697847	9.497518337	1.20110616	7.90730969	2.63E-15	4.45E-13
ENSDARG00000117813	CABZ01092942.1	759.30687	0.927344625	0.19354723	4.79130911	1.66E-06	4.75E-05
ENSDARG00000098214	CABZ01072487.1	150.2106	-1.037857937	0.2192248	-4.73421776	2.20E-06	6.06E-05
ENSDARG00000102116	CABZ01072487.2	20.6003099	2.407870293	0.52104093	4.62126895	3.81E-06	9.69E-05
ENSDARG00000116978	CABZ01010830.1	25.485856	-1.90165623	0.46702658	-4.07183722	4.66E-05	0.00082339
ENSDARG00000101462	setdb2	623.027535	0.40539573	0.14740798	2.7501613	0.00595659	0.0387163
ENSDARG00000102733	EMC4	128.586322	1.288522623	0.28423795	4.53325323	5.81E-06	0.00014051
ENSDARG00000101621	CABZ01040054.1	69.8678904	-1.88099798	0.45746053	-4.11182576	3.93E-05	0.00071113
ENSDARG00000099154	CABZ01032488.1	735.366803	1.068116145	0.18210242	5.8654693	4.48E-09	2.55E-07
ENSDARG00000099719	cdkn1d	1986.87682	-0.318759228	0.09490084	-3.35886638	0.00078263	0.00818353
ENSDARG00000100478	TULP1	263.71609	1.764025519	0.45935857	3.84019286	0.00012294	0.00186432
ENSDARG00000100185	elovl7b	1142.3283	0.637379527	0.13925052	4.57721475	4.71E-06	0.00011677
ENSDARG00000101913	CABZ01092156.1	455.315744	0.485289554	0.16518265	2.93789664	0.00330447	0.02490377
ENSDARG00000100540	CABZ01114105.1	222.315174	-0.672369181	0.17642798	-3.81101228	0.0001384	0.00205859
ENSDARG00000117313	CABZ01005857.2	25.8236305	2.488820075	0.57798686	4.306015	1.66E-05	0.00034457
ENSDARG00000100552	taco1	405.394672	0.882462315	0.20621858	4.27925713	1.88E-05	0.00038109
ENSDARG00000090119	tlr8a	7.55060476	6.142806959	1.43398179	4.28374125	1.84E-05	0.00037471
ENSDARG00000101736	CABZ01113816.1	29.9186252	4.894245173	0.94600189	5.17361035	2.30E-07	8.51E-06
ENSDARG00000104246	exosc3	233.62114	1.898395808	0.29255718	6.48897359	8.64E-11	7.02E-09
ENSDARG00000098584	CABZ01102240.1	2366.55327	0.315010289	0.11562821	2.72433761	0.00644306	0.04103056
ENSDARG00000101369	CABZ01090890.1	2193.14171	0.727984859	0.11178811	6.51218513	7.41E-11	6.04E-09
ENSDARG00000111606	smyhc2	52.8421801	4.102524968	1.22606233	3.3460982	0.00081957	0.00848525
ENSDARG00000113433	FAM126B	78.8856382	1.044193273	0.33545163	3.11279832	0.00185323	0.01598263

Table S1. DEGs of WT vs. *terfa+/-*

ENSDARG00000102859	CABZ01092170.1	243.35083	0.744308341	0.18024255	4.12948187	3.64E-05	0.00066594
ENSDARG00000110510	ybey	301.517631	0.719780467	0.19152123	3.75822815	0.00017112	0.00243597
ENSDARG00000104316	CABZ01078055.1	1202.38029	0.523370193	0.14504668	3.60828789	0.00030822	0.00394168
ENSDARG00000113284	CABZ01090361.1	125.178118	5.32198265	0.41756608	12.7452465	3.31E-37	4.84E-34
ENSDARG00000102207	zgc:86738	678.945478	0.593671443	0.14740406	4.0275109	5.64E-05	0.00096693
ENSDARG00000116774	PCNP	25.9935336	8.144713664	1.26579694	6.43445515	1.24E-10	9.72E-09

Table S2. DEGs of WT vs. *terfa-/-*

	genename	baseMean	log2FoldChange	lfcSE	stat	pvalue	padj
ENSDARG00000035559	tp53	4381.26849	2.584357944	0.11494024	22.4843614	5.90E-112	1.47E-107
ENSDARG00000096603	bmb	667.445067	4.017101422	0.18380727	21.8549649	6.97E-106	8.67E-102
ENSDARG00000093546	si:ch73-56d11.5	457.467876	4.514885356	0.21807044	20.7037933	3.20E-95	2.65E-91
ENSDARG00000076667	ccng1	25526.0463	1.952177183	0.1014216	19.2481408	1.46E-82	9.09E-79
ENSDARG00000114142	si:dkeyp-4f2.1	250.173315	5.552547285	0.29196846	19.017627	1.22E-80	6.06E-77
ENSDARG00000019949	serpinh1b	4542.83123	2.81581519	0.15013031	18.7558081	1.74E-78	7.19E-75
ENSDARG00000078683	RNF14	1410.56683	3.340990673	0.17874105	18.6917926	5.77E-78	2.05E-74
ENSDARG00000033443	mdm2	3617.08246	2.793154892	0.15160681	18.423677	8.48E-76	2.64E-72
ENSDARG00000020741	fga	12923.4149	1.464005097	0.08045888	18.1956936	5.58E-74	1.54E-70
ENSDARG00000076554	cdkn1a	1017.96069	3.535902774	0.19514185	18.1196535	2.23E-73	5.54E-70
ENSDARG00000078114	si:ch73-237c6.1	938.370553	3.517817522	0.20898619	16.8327751	1.40E-63	3.17E-60
ENSDARG00000039579	cfd	2311.69758	2.241647585	0.13472888	16.6382114	3.68E-62	7.63E-59
ENSDARG00000104204	slc1a8a	438.068034	-3.859237522	0.23493301	-16.4269702	1.23E-60	2.34E-57
ENSDARG00000104890	si:ch211-76m11.3	214.833865	6.23407682	0.38043397	16.3867513	2.38E-60	4.22E-57
ENSDARG00000043323	lnx1	2468.97266	2.310278345	0.14114665	16.3679293	3.24E-60	5.37E-57
ENSDARG00000016337	lctlb	305.584758	-3.299933118	0.20194107	-16.3410697	5.04E-60	7.82E-57
ENSDARG00000099175	hmgb1a	11980.299	-1.76382185	0.10818152	-16.3042801	9.20E-60	1.35E-56
ENSDARG00000055278	cfb	3350.27918	2.475932707	0.15276815	16.2071259	4.49E-59	6.20E-56
ENSDARG00000100513	rps27l	1923.48099	2.573696072	0.15999317	16.0862873	3.18E-58	4.16E-55
ENSDARG00000096762	si:dkey-61p9.11	195.015417	4.121420071	0.25971495	15.8690135	1.04E-56	1.29E-53
ENSDARG00000060196	lhpp	404.895921	3.037637146	0.19736639	15.3908533	1.89E-53	2.23E-50
ENSDARG00000079403	si:dkey-204l11.1	189.549299	5.330030533	0.34733217	15.3456288	3.79E-53	4.28E-50
ENSDARG00000078619	pnp5a	4001.68186	1.356809066	0.09005666	15.0661713	2.70E-51	2.92E-48
ENSDARG00000079745	si:ch211-166a6.5	6924.28038	1.44079855	0.09577157	15.0441161	3.77E-51	3.91E-48
ENSDARG00000038894	tmx3a	548.517652	-3.953154683	0.26319584	-15.0198223	5.45E-51	5.21E-48
ENSDARG00000013963	mipb	1466.89749	-3.5706052	0.23769429	-15.0218381	5.28E-51	5.21E-48
ENSDARG00000037804	phlda3	785.300931	2.184071081	0.14556006	15.0046038	6.85E-51	6.31E-48
ENSDARG00000038424	c4b	2424.55447	2.1035843	0.14131627	14.8856482	4.09E-50	3.63E-47
ENSDARG00000010434	clu	2223.69319	3.002780604	0.20275109	14.8101826	1.26E-49	1.08E-46
ENSDARG00000045636	rbl2	1855.39646	1.69274704	0.11477256	14.748708	3.14E-49	2.60E-46
ENSDARG00000003961	parp3	931.459903	1.71876914	0.11673019	14.7242904	4.50E-49	3.61E-46
ENSDARG00000103760	cfhl2	1027.33964	2.288493126	0.15585235	14.6837258	8.20E-49	6.37E-46
ENSDARG00000073711	mmrn2b	539.328122	1.998276791	0.13701241	14.5846404	3.52E-48	2.65E-45
ENSDARG00000034007	prom1b	2829.43744	-2.372669594	0.16314428	-14.543382	6.43E-48	4.70E-45
ENSDARG00000051981	STX3	368.434022	-2.717398788	0.18756921	-14.4874462	1.45E-47	1.03E-44
ENSDARG00000061101	snx19a	260.320285	-3.0844315	0.21410568	-14.4061172	4.74E-47	3.27E-44
ENSDARG00000041339	zgc:92380	3683.37486	1.7050266	0.11852211	14.385726	6.36E-47	4.27E-44
ENSDARG00000102288	CABZ01056321.1	364.573703	-3.149265413	0.22039222	-14.2893677	2.55E-46	1.67E-43
ENSDARG00000051896	fbn2a	708.198294	2.040868492	0.14387328	14.1851809	1.13E-45	7.21E-43
ENSDARG00000098171		1646.50662	1.666150259	0.11856037	14.0531799	7.37E-45	4.58E-42
ENSDARG00000039832	gsta.2	256.606882	4.042043001	0.28829556	14.0204831	1.17E-44	7.08E-42
ENSDARG00000104980	PPEF2	256.75181	-3.832586997	0.27483009	-13.9452962	3.36E-44	1.99E-41
ENSDARG00000012694	c3a.1	25373.7829	1.698490113	0.1220101	13.9208974	4.73E-44	2.73E-41
ENSDARG00000004658	zgc:101810	2234.86096	1.495395259	0.10753796	13.905743	5.85E-44	3.30E-41
ENSDARG00000010478	hsp90aa1.1	4632.30807	1.756491963	0.12740525	13.786653	3.07E-43	1.69E-40
ENSDARG00000096310	wu:fj64h06	407.593234	1.785834806	0.12981777	13.7564738	4.66E-43	2.52E-40
ENSDARG00000056885	per1a	851.286311	2.352349042	0.17207143	13.6707702	1.52E-42	8.03E-40
ENSDARG00000007219	actn1	5521.54914	1.253315704	0.09171779	13.6649135	1.64E-42	8.52E-40
ENSDARG00000026611	socs3b	1867.53237	2.686561279	0.1976235	13.5943414	4.33E-42	2.19E-39
ENSDARG00000059294	marco	371.026678	2.828182734	0.20815182	13.5871149	4.78E-42	2.37E-39
ENSDARG00000055514	icn2	4106.02086	1.514008887	0.11165516	13.5596863	6.94E-42	3.38E-39
ENSDARG00000086272	si:dkey-4p15.5	822.57955	-2.278282911	0.16975058	-13.4213558	4.53E-41	2.17E-38
ENSDARG00000102395	HAPLN1	167.041289	6.369879828	0.47486971	13.4139528	5.01E-41	2.35E-38
ENSDARG00000103413	zgc:109949	668.469045	-2.07747735	0.15584067	-13.3307779	1.53E-40	7.06E-38
ENSDARG00000051762	CABZ01080568.1	1128.61258	1.657818419	0.12443958	13.3222762	1.72E-40	7.76E-38
ENSDARG00000057911	ACTC1	4445.08591	2.595076173	0.19526581	13.2899669	2.65E-40	1.17E-37
ENSDARG00000079783	isg20	904.699708	1.415313863	0.10752427	13.1627386	1.44E-39	6.27E-37

Table S2. DEGs of WT vs. *terfa-/-*

ENSDARG00000097080	si:ch73-181m17.1	124.269814	3.757049587	0.28664856	13.1068149	3.01E-39	1.29E-36
ENSDARG00000078210	tulp1b	297.559266	-3.116157385	0.23821873	-13.0810763	4.22E-39	1.78E-36
ENSDARG00000102713	si:ch211-205a14.1	93.7994393	5.998738564	0.46268502	12.9650591	1.93E-38	8.00E-36
ENSDARG00000041864	capn3a	2547.16651	-2.660809208	0.20613574	-12.9080439	4.05E-38	1.65E-35
ENSDARG00000008969	fgb	10194.5151	1.283989307	0.09971271	12.8768872	6.07E-38	2.40E-35
ENSDARG00000062902	si:dkey-220f10.4	279.006417	-2.859244462	0.22203927	-12.8772014	6.05E-38	2.40E-35
ENSDARG00000074390	tmem176l.4	2920.39054	1.698307378	0.13192016	12.8737519	6.33E-38	2.46E-35
ENSDARG00000075027	fgfr1bl	249.439405	2.901020427	0.22806707	12.7200321	4.58E-37	1.75E-34
ENSDARG00000011640	syt5b	3532.15768	-2.336080483	0.18439508	-12.668887	8.80E-37	3.31E-34
ENSDARG00000018491	pdia4	3972.25214	1.312680789	0.10363911	12.6658831	9.14E-37	3.39E-34
ENSDARG00000059978	cplx4a	1433.24018	-3.103723047	0.2461616	-12.6084775	1.90E-36	6.93E-34
ENSDARG00000017624	krt4	481111.355	1.197008309	0.09519752	12.5739442	2.94E-36	1.06E-33
ENSDARG00000093753	BX004774.2	156.024075	-3.542259874	0.28313607	-12.5108041	6.52E-36	2.31E-33
ENSDARG00000016319	c9	6476.57822	1.562717944	0.12500506	12.5012372	7.35E-36	2.57E-33
ENSDARG00000052011	rrad	652.37699	2.523001313	0.20260343	12.4529055	1.35E-35	4.65E-33
ENSDARG00000098164	aipl2	359.537582	-2.816420494	0.22667777	-12.4247758	1.92E-35	6.53E-33
ENSDARG00000058206	si:ch211-153b23.5	2942.20757	1.199821092	0.09666886	12.4116613	2.26E-35	7.59E-33
ENSDARG00000089742	fam161a	471.130254	-2.54123345	0.20531139	-12.3774599	3.46E-35	1.15E-32
ENSDARG00000025301	gfap	8898.89206	-1.417273232	0.11498065	-12.3261894	6.55E-35	2.14E-32
ENSDARG00000096701	si:dkey-21e13.3	229.846386	-2.495472641	0.20251468	-12.3224283	6.86E-35	2.21E-32
ENSDARG00000026726	anxa1a	6809.69504	2.093904115	0.17095806	12.2480576	1.72E-34	5.48E-32
ENSDARG00000007080	rhcgl1	337.599004	4.101602054	0.33592928	12.2097188	2.76E-34	8.68E-32
ENSDARG00000076321	col28a2a	3366.02996	2.719490266	0.22302256	12.1937901	3.35E-34	1.04E-31
ENSDARG00000007344	tcap	792.784262	2.72678376	0.2243971	12.1515998	5.63E-34	1.73E-31
ENSDARG00000074094	tgm2b	3694.99319	1.890538148	0.15573983	12.1390792	6.56E-34	1.99E-31
ENSDARG00000012504	rlbp1a	1015.53788	-2.875857682	0.23780269	-12.0934615	1.14E-33	3.42E-31
ENSDARG00000025254	s100a10b	3710.43027	1.531815072	0.12674648	12.0856616	1.26E-33	3.72E-31
ENSDARG00000094901	abcc6b.2	271.79595	3.272268222	0.27136779	12.0584252	1.75E-33	5.12E-31
ENSDARG00000078322	col12a1a	31189.1846	1.340196281	0.11155233	12.0140591	3.00E-33	8.66E-31
ENSDARG00000003203	rhcga	3279.95773	4.354994318	0.3629809	11.9978609	3.65E-33	1.04E-30
ENSDARG00000056725	hmgb3a	17012.1352	-1.736309337	0.14479272	-11.9916895	3.93E-33	1.11E-30
ENSDARG00000099227	galnt13	582.513331	-1.636825378	0.13723043	-11.9275684	8.50E-33	2.37E-30
ENSDARG00000068966	si:ch211-261n11.7	202.56121	5.23412756	0.43934635	11.9134426	1.01E-32	2.78E-30
ENSDARG00000021242	mvp	4267.65807	1.46842712	0.12395724	11.8462395	2.25E-32	6.15E-30
ENSDARG00000101892	ino80e	1219.43027	1.513653001	0.12801741	11.823806	2.94E-32	7.94E-30
ENSDARG00000054804	anp32e	13131.2334	-1.507849912	0.12756073	-11.8206439	3.05E-32	8.16E-30
ENSDARG00000038025	cbx7a	656.824384	3.096485162	0.26244128	11.7987732	3.96E-32	1.05E-29
ENSDARG00000101919	foxj1a	394.147532	2.409015192	0.2049507	11.7541206	6.73E-32	1.76E-29
ENSDARG00000111240	dhrs13a.2	1858.65455	1.892767818	0.16174197	11.7023913	1.24E-31	3.21E-29
ENSDARG00000015709	hsd17b12a	1670.93997	1.274563826	0.10924785	11.6667182	1.89E-31	4.83E-29
ENSDARG00000074919	BFSP1	434.989103	-3.468491267	0.29752675	-11.6577462	2.10E-31	5.31E-29
ENSDARG00000000380	pde6a	396.019807	-2.41767745	0.20742327	-11.6557675	2.14E-31	5.38E-29
ENSDARG00000003216	anxa2a	5627.90652	2.643242753	0.22796013	11.5951974	4.36E-31	1.08E-28
ENSDARG00000040159	wnt4b	1034.70576	1.62005637	0.13991411	11.5789352	5.27E-31	1.30E-28
ENSDARG00000076055	rpgrip1	506.579439	-3.165507887	0.27366224	-11.5672073	6.04E-31	1.47E-28
ENSDARG00000067859	scospondin	579.679448	3.187708308	0.27569119	11.5626048	6.37E-31	1.54E-28
ENSDARG00000014624	si:dkeyp-94g1.1	184.222783	5.621222991	0.48632539	11.5585637	6.68E-31	1.60E-28
ENSDARG00000039677	dsc2l	2183.62737	1.309364506	0.11329954	11.5566623	6.83E-31	1.62E-28
ENSDARG00000086374	isg15	105.367254	4.633037169	0.40114575	11.5495108	7.42E-31	1.74E-28
ENSDARG00000116713	si:ch73-265d7.2	500.922359	2.378407772	0.20629021	11.5294261	9.38E-31	2.18E-28
ENSDARG00000013741	lancl1	670.189938	-1.416184974	0.12293159	-11.5201065	1.04E-30	2.40E-28
ENSDARG00000004643	cdhr1a	432.187952	-2.514884729	0.21856174	-11.5065186	1.22E-30	2.79E-28
ENSDARG00000017780	rorcb	1334.06796	-1.46917058	0.12840302	-11.4418692	2.58E-30	5.84E-28
ENSDARG00000104380	si:dkey-238k10.2	366.97707	-2.661988311	0.2332487	-11.4126607	3.61E-30	8.09E-28
ENSDARG00000103844	si:ch211-266c8.1	268.239859	-2.286330751	0.20044768	-11.4061226	3.90E-30	8.65E-28
ENSDARG00000043719	c3a.6	2099.52699	1.865726487	0.16375702	11.3932609	4.52E-30	9.94E-28
ENSDARG00000044010	loxl2a	1790.36538	1.268021675	0.11152428	11.3699161	5.90E-30	1.29E-27
ENSDARG00000071251	ppp1r18	330.595408	-2.380357802	0.21042851	-11.311955	1.15E-29	2.47E-27

Table S2. DEGs of WT vs. *terfa-/-*

ENSDARG00000019752	rom1a	986.734433	-2.960849899	0.26203746	-11.2993384	1.32E-29	2.83E-27
ENSDARG00000006413	rpl38	5941.58573	1.511494476	0.13399706	11.2800568	1.65E-29	3.50E-27
ENSDARG00000010415	sirt4	207.007893	2.286617818	0.20432419	11.1911265	4.51E-29	9.49E-27
ENSDARG00000090873	ccl34a.4	98.0947054	3.638387231	0.32552134	11.1771081	5.28E-29	1.10E-26
ENSDARG00000019782	impg2a	205.709059	-3.921245809	0.35142945	-11.1579886	6.55E-29	1.36E-26
ENSDARG00000042900	gtpbp1l	898.794883	1.568593189	0.14066273	11.1514483	7.05E-29	1.45E-26
ENSDARG00000059903	hs3st3l	243.593941	-2.479138693	0.22257796	-11.1382938	8.17E-29	1.65E-26
ENSDARG00000016188	SBNO2	1591.40974	2.042894604	0.18341312	11.138214	8.17E-29	1.65E-26
ENSDARG00000028396	fkbp5	5927.57648	2.064035041	0.18574519	11.1121857	1.09E-28	2.19E-26
ENSDARG00000053853	slc13a2	3491.02481	1.730240218	0.15572322	11.1109972	1.11E-28	2.21E-26
ENSDARG00000007077	ankrd50l	1498.91578	1.431549523	0.12896841	11.1000014	1.25E-28	2.47E-26
ENSDARG00000100690	si:ch211-256e16.1	129.950135	2.937309717	0.26498213	11.0849353	1.48E-28	2.91E-26
ENSDARG00000069282	bbc3	514.790204	1.738099667	0.15687202	11.0797304	1.57E-28	3.06E-26
ENSDARG00000016350	cap1	805.43347	1.277563605	0.11546695	11.0643226	1.87E-28	3.60E-26
ENSDARG00000103199	si:dkey-247k7.2	810.28445	1.81756106	0.16461593	11.0412222	2.42E-28	4.62E-26
ENSDARG00000063295	myh9a	16303.1749	1.078792266	0.09774115	11.0372369	2.53E-28	4.76E-26
ENSDARG00000037587	SYNPR	434.812837	-2.061005514	0.18673073	-11.0373132	2.52E-28	4.76E-26
ENSDARG00000037746	actb1	160953.656	1.023273776	0.09316221	10.9837861	4.57E-28	8.55E-26
ENSDARG00000028507	itgb4	4382.96869	1.75861341	0.16022762	10.9757196	5.00E-28	9.28E-26
ENSDARG00000017676	mmp2	11412.006	1.3848445	0.12651597	10.9460051	6.94E-28	1.28E-25
ENSDARG00000041703	rrbp1b	4080.36061	1.307061665	0.11965858	10.9232591	8.92E-28	1.63E-25
ENSDARG00000098051	opn6b	228.362736	-3.560632973	0.32624048	-10.9141361	9.87E-28	1.79E-25
ENSDARG00000059682	slc43a3a	261.237457	-2.144287678	0.19671241	-10.9006224	1.14E-27	2.05E-25
ENSDARG00000086288	scg3	3055.97975	-1.198890583	0.10998108	-10.9008803	1.14E-27	2.05E-25
ENSDARG00000103919	si:ch73-1a9.3	21540.371	-1.485971626	0.13648864	-10.8871448	1.33E-27	2.36E-25
ENSDARG00000039211	zgc:77439	2087.39292	1.306287862	0.12023637	10.8643323	1.70E-27	3.00E-25
ENSDARG00000074201	flna	17437.5412	1.57564367	0.14529231	10.8446464	2.11E-27	3.70E-25
ENSDARG00000009978	icn	13809.6325	1.449290763	0.13368345	10.8412129	2.20E-27	3.82E-25
ENSDARG00000043781	psmb10	454.61845	1.729122096	0.15973013	10.8252723	2.61E-27	4.48E-25
ENSDARG00000039652	si:dkey-235d18.5	910.091138	1.406834448	0.12995726	10.8253626	2.61E-27	4.48E-25
ENSDARG00000056767	itgb3a	1632.41289	1.316494448	0.12178932	10.8096051	3.10E-27	5.27E-25
ENSDARG00000075718	rpz5	1997.97212	1.507807637	0.13949379	10.8091378	3.12E-27	5.27E-25
ENSDARG00000095802	pcare2	752.755733	-2.783701802	0.25764082	-10.8045838	3.27E-27	5.46E-25
ENSDARG00000036832	cyt1l	79323.9532	1.592926524	0.14742462	10.8050236	3.26E-27	5.46E-25
ENSDARG00000054543	samsn1a	659.778339	-1.721588508	0.15960384	-10.7866356	3.98E-27	6.60E-25
ENSDARG00000073845	zgc:110843	933.452696	1.537923424	0.14276657	10.7722937	4.65E-27	7.66E-25
ENSDARG00000062661	abca4b	1124.01774	-2.362200894	0.21963455	-10.7551427	5.60E-27	9.16E-25
ENSDARG00000007709	adamts8a	423.023431	2.271844117	0.21144498	10.7443749	6.30E-27	1.02E-24
ENSDARG00000076872	mpp4l	230.431817	-2.829521471	0.26358306	-10.7348381	6.98E-27	1.13E-24
ENSDARG00000054797	zgc:153441	383.337072	-2.44009939	0.22750454	-10.7254976	7.73E-27	1.24E-24
ENSDARG00000114031	smyhc3	612.804885	2.081757765	0.19443358	10.706781	9.46E-27	1.51E-24
ENSDARG00000098890	cyp2aa9	591.252084	1.826182809	0.17072514	10.6966252	1.06E-26	1.67E-24
ENSDARG00000111106	arl3l1	781.724161	-2.600803972	0.24426743	-10.6473631	1.79E-26	2.82E-24
ENSDARG00000007576	crybb1l1	9162.17673	-3.545227126	0.33309689	-10.643231	1.88E-26	2.93E-24
ENSDARG00000058803	grk1a	666.496501	-2.831266372	0.2662211	-10.6350186	2.05E-26	3.18E-24
ENSDARG00000092354	si:ch211-284e13.6	1041.51127	-1.386228967	0.13049412	-10.6229231	2.33E-26	3.60E-24
ENSDARG00000010933	cacna1fa	346.79863	-3.015074184	0.28428402	-10.6058518	2.80E-26	4.29E-24
ENSDARG00000105590	si:cabz01007794.1	3396.28269	1.435748993	0.13538611	10.6048473	2.83E-26	4.31E-24
ENSDARG00000099672	capgb	2486.54137	1.201969257	0.11345836	10.5939239	3.18E-26	4.82E-24
ENSDARG00000040274	scamp5b	1003.82047	-2.686944985	0.25388062	-10.5834977	3.55E-26	5.35E-24
ENSDARG00000099572	hmgn2	21392.844	-1.638474976	0.15485592	-10.5806416	3.66E-26	5.45E-24
ENSDARG00000102364	si:dkey-202l22.6	4160.42855	1.694982364	0.16018664	10.5812966	3.64E-26	5.45E-24
ENSDARG00000022712	stat3	1992.59983	1.666260074	0.15768685	10.5668928	4.24E-26	6.28E-24
ENSDARG00000031588	si:dkey-239b22.1	907.347081	2.433386741	0.23036033	10.5633933	4.40E-26	6.48E-24
ENSDARG00000104200	BX901923.2	2306.4004	1.170363747	0.11080008	10.5628421	4.43E-26	6.48E-24
ENSDARG00000003570	hsp90b1	14389.6937	0.993314815	0.09446457	10.51521	7.35E-26	1.07E-23
ENSDARG00000103846	hspa5	17023.0738	1.004781671	0.09558566	10.5118456	7.62E-26	1.10E-23
ENSDARG00000094584	BX470240.1	88.5580758	5.516280115	0.52533789	10.5004421	8.60E-26	1.24E-23

Table S2. DEGs of WT vs. *terfa-/-*

ENSDARG00000044632	myo7ab	462.7481	-1.545830497	0.14727167	-10.4964554	8.97E-26	1.28E-23
ENSDARG00000020814	faimb	224.683693	-3.175241382	0.30266493	-10.4909458	9.51E-26	1.35E-23
ENSDARG00000089429	si:dkey-205h13.2	2053.00806	-1.612758038	0.15382755	-10.4841948	1.02E-25	1.44E-23
ENSDARG00000078882	slc22a31	1239.19104	1.445576806	0.13829092	10.4531579	1.42E-25	1.99E-23
ENSDARG00000036371	acta1a	22059.5386	1.548153195	0.14838519	10.4333408	1.75E-25	2.44E-23
ENSDARG00000101757	si:dkey-27n6.4	50.610123	4.589119121	0.44069041	10.4134762	2.15E-25	2.99E-23
ENSDARG00000058357	ankrd33ba	330.267316	-2.200299756	0.21162064	-10.3973778	2.55E-25	3.52E-23
ENSDARG00000103775	thbs1a	2757.31451	1.968198259	0.18976725	10.3716436	3.34E-25	4.58E-23
ENSDARG00000038742	rbp1.2	182.738547	-3.556819565	0.3430979	-10.3667775	3.51E-25	4.80E-23
ENSDARG00000040944	ntd5	2009.62803	1.251258638	0.1207825	10.3596019	3.79E-25	5.14E-23
ENSDARG00000100339	arf4b	168.451686	3.451945155	0.33329953	10.3568859	3.89E-25	5.26E-23
ENSDARG00000012125	cnga1b	158.320561	-3.246505539	0.31360754	-10.3521286	4.09E-25	5.50E-23
ENSDARG00000004836	dnajc5ab	3836.10206	-1.023123916	0.09931027	-10.3022976	6.88E-25	9.19E-23
ENSDARG00000099425	CABZ01073265.1	3587.7991	1.291472039	0.12564537	10.2787079	8.79E-25	1.17E-22
ENSDARG00000037281	fgg	6969.63654	1.244578191	0.12118416	10.2701393	9.61E-25	1.27E-22
ENSDARG00000013134	prph2la	297.687698	-2.306177717	0.2247451	-10.2613039	1.05E-24	1.38E-22
ENSDARG00000037870	actb2	190902.734	0.929937279	0.09071401	10.2513078	1.17E-24	1.53E-22
ENSDARG00000035163	tlcd5b	146.239887	-2.993876388	0.29239472	-10.2391604	1.32E-24	1.72E-22
ENSDARG00000020711	rrm2	831.137572	6.254113225	0.61455146	10.176712	2.52E-24	3.26E-22
ENSDARG00000117614	CR550304.1	215.436175	-2.281058856	0.22424793	-10.1720398	2.64E-24	3.40E-22
ENSDARG00000023886	cacna2d4b	755.992235	-2.178836962	0.21422019	-10.1710157	2.67E-24	3.42E-22
ENSDARG00000098465	si:ch73-366l1.5	4958.83819	1.425082326	0.14042729	10.1481867	3.38E-24	4.30E-22
ENSDARG00000034424	atp1b2b	1337.96766	-1.873092766	0.18475701	-10.1381421	3.74E-24	4.74E-22
ENSDARG00000034453	unc119a	303.554954	-2.325006967	0.22944593	-10.1331367	3.94E-24	4.97E-22
ENSDARG00000101071	si:dkeyp-80d11.14	55.4458809	3.919856497	0.38689862	10.1314824	4.01E-24	5.03E-22
ENSDARG00000058371	krt5	146410.295	0.863023168	0.08522617	10.1262692	4.22E-24	5.28E-22
ENSDARG00000094217	si:dkey-17e16.15	259.912662	-2.476794334	0.245029	-10.1081681	5.08E-24	6.32E-22
ENSDARG00000069934	parp4	1211.86397	1.542986557	0.15369098	10.0395386	1.02E-23	1.26E-21
ENSDARG00000043035	capn3b	1703.18603	1.088215626	0.10872908	10.0085061	1.40E-23	1.72E-21
ENSDARG00000060439	clcn2c	802.970152	2.096088583	0.20950616	10.0049019	1.45E-23	1.78E-21
ENSDARG00000009401	vcanb	11404.6841	1.12310929	0.11250638	9.98262746	1.82E-23	2.21E-21
ENSDARG00000088589	ponzr3	1379.92309	2.461342034	0.24659266	9.98140849	1.84E-23	2.23E-21
ENSDARG00000052700	SLC9B2	1372.29964	-2.432291421	0.24389933	-9.97252186	2.01E-23	2.43E-21
ENSDARG00000040334	mat2aa	10002.3444	1.037235374	0.10412058	9.96186691	2.24E-23	2.69E-21
ENSDARG00000042107	cngb1b	517.610843	-2.166823529	0.21755905	-9.95970297	2.29E-23	2.73E-21
ENSDARG00000010385	sept4a	462.593907	-2.186502497	0.21963155	-9.95532065	2.39E-23	2.84E-21
ENSDARG00000035438	myhc4	6043.71208	1.821466076	0.18310577	9.9476169	2.58E-23	3.06E-21
ENSDARG00000026829	cotl1	12583.4828	0.890998408	0.08962062	9.94189031	2.74E-23	3.22E-21
ENSDARG00000103306	BX927130.4	150.889511	3.889139503	0.39162756	9.93070953	3.06E-23	3.59E-21
ENSDARG00000096118	ost4	605.32554	1.38145207	0.13913976	9.92852101	3.13E-23	3.65E-21
ENSDARG00000052575	arl13a	302.221285	-2.971894789	0.29941243	-9.92575637	3.22E-23	3.74E-21
ENSDARG00000058158	trim55b	292.356726	1.661460339	0.16819883	9.877954	5.19E-23	6.00E-21
ENSDARG00000027495	elovl4b	2374.39837	-3.158677875	0.32014425	-9.8664208	5.82E-23	6.70E-21
ENSDARG00000100442	cfh	4680.94845	1.175857932	0.11923923	9.86133459	6.12E-23	7.01E-21
ENSDARG00000105159	hdac8	1428.21389	1.517748572	0.15433837	9.8339033	8.04E-23	9.17E-21
ENSDARG00000036558	col18a1a	20473.7233	0.978212763	0.09967942	9.81358802	9.84E-23	1.12E-20
ENSDARG00000007553	opn4.1	576.387711	-2.0686911	0.2108819	-9.80971373	1.02E-22	1.16E-20
ENSDARG00000099695	si:dkey-61n16.5	285.835244	-2.316222605	0.23622022	-9.80535304	1.07E-22	1.20E-20
ENSDARG00000040362	ehd2b	1691.82626	1.337454451	0.13648659	9.79916383	1.14E-22	1.27E-20
ENSDARG00000099384	si:dkey-238k10.1	116.673029	-3.279151315	0.3347566	-9.79562855	1.18E-22	1.31E-20
ENSDARG00000102440	CABZ01070852.1	360.917653	2.465302917	0.25171589	9.79399005	1.19E-22	1.33E-20
ENSDARG00000004782	fgfr3	3956.84677	1.005111254	0.10266524	9.79018083	1.24E-22	1.37E-20
ENSDARG00000012126	zgc:109965	710.741805	-2.559965889	0.26152164	-9.7887343	1.26E-22	1.38E-20
ENSDARG00000086678	ascc3	1796.26491	1.172720689	0.11981356	9.78787985	1.27E-22	1.39E-20
ENSDARG00000068507	crybb1	3625.14152	-3.734810719	0.38190517	-9.77941912	1.38E-22	1.50E-20
ENSDARG00000052279	osgn1	775.620115	1.195972037	0.12236282	9.77398226	1.46E-22	1.58E-20
ENSDARG00000041735	tmem237a	410.795863	-2.859660206	0.2934008	-9.74659979	1.91E-22	2.06E-20
ENSDARG00000075993	clic5a	676.873162	1.753910845	0.18002278	9.74271615	1.98E-22	2.13E-20

Table S2. DEGs of WT vs. *terfa-/-*

ENSDARG00000012499	per1b	4410.69075	1.180700665	0.12120807	9.74110622	2.01E-22	2.15E-20
ENSDARG00000076945	dsg2.1	2977.15626	1.23744755	0.12702685	9.74162223	2.00E-22	2.15E-20
ENSDARG00000015273	alpi.1	800.450926	1.126696889	0.1157948	9.73011671	2.24E-22	2.38E-20
ENSDARG00000076221	fthl28	697.763581	1.876337669	0.19287455	9.72828007	2.28E-22	2.42E-20
ENSDARG00000037337	cnrip1b	483.043021	-1.947604829	0.20029852	-9.72351073	2.39E-22	2.52E-20
ENSDARG00000113284	CABZ01090361.1	125.178118	4.258754497	0.43937729	9.69270521	3.24E-22	3.40E-20
ENSDARG00000102967	CT573282.1	138.745457	-3.369618917	0.34833146	-9.67359919	3.90E-22	4.08E-20
ENSDARG00000117393	CABZ01064941.1	913.295493	2.049487802	0.21194675	9.66982423	4.05E-22	4.21E-20
ENSDARG00000037421	egr1	2591.17213	-1.454623575	0.15050528	-9.6649339	4.25E-22	4.40E-20
ENSDARG00000051914	slc14a2	99.4523869	2.779867952	0.28816563	9.6467714	5.07E-22	5.23E-20
ENSDARG00000021345	prph2lb	185.731054	-2.911619419	0.30197918	-9.64178866	5.33E-22	5.47E-20
ENSDARG00000101828	si:dkey-16p6.1	60.2626935	4.891147168	0.50758735	9.63606978	5.63E-22	5.76E-20
ENSDARG00000115528	FO082780.1	216.932525	-3.010653397	0.31245738	-9.63540511	5.67E-22	5.77E-20
ENSDARG00000095831	si:ch211-175f12.2	221.037151	-3.590008896	0.37276661	-9.63071485	5.93E-22	6.02E-20
ENSDARG00000098359	il17rd	1177.69995	0.897911702	0.09353649	9.59958689	8.03E-22	8.11E-20
ENSDARG00000105392	si:ch73-22o18.1	98.4111125	-3.381128017	0.35228266	-9.59777031	8.17E-22	8.22E-20
ENSDARG00000055133	cenpf	1873.63967	-1.780438961	0.1855687	-9.59450025	8.43E-22	8.45E-20
ENSDARG00000088091	pfn1	41554.0515	1.011499517	0.10552491	9.58540996	9.21E-22	9.19E-20
ENSDARG00000074749	abca12	3978.97817	1.021671012	0.10661011	9.58324669	9.40E-22	9.35E-20
ENSDARG00000104231	opn9	237.481585	-3.198637832	0.33385095	-9.58103563	9.61E-22	9.51E-20
ENSDARG00000071684	rx1	984.501329	-1.900189132	0.19836994	-9.57901776	9.80E-22	9.66E-20
ENSDARG00000023537	ahr1b	758.003685	-1.621939188	0.16936719	-9.57646661	1.00E-21	9.87E-20
ENSDARG00000031600	rd3	404.747327	-1.358028151	0.14195669	-9.56649614	1.11E-21	1.08E-19
ENSDARG00000100255	si:ch73-29l19.1	293.786058	-2.442690624	0.25601155	-9.54132981	1.41E-21	1.37E-19
ENSDARG00000090297	ldlrad2	224.525389	2.285971979	0.23956891	9.54202273	1.40E-21	1.37E-19
ENSDARG00000010710	msi1	2121.01484	-1.186400228	0.12441893	-9.5355283	1.49E-21	1.44E-19
ENSDARG00000068716	cuedc1a	1050.21728	-1.362358124	0.14290431	-9.53335927	1.52E-21	1.47E-19
ENSDARG00000001463	tdh2	1190.83497	-2.058345608	0.21606491	-9.52651494	1.63E-21	1.56E-19
ENSDARG00000038862	kcnb2	474.821651	-2.147628078	0.22547322	-9.52498057	1.65E-21	1.58E-19
ENSDARG00000036942	gpd1c	1602.87843	1.028966114	0.10803466	9.5244074	1.66E-21	1.58E-19
ENSDARG00000095400	si:ch73-52f15.5	648.881571	1.145227417	0.12040867	9.51117044	1.89E-21	1.79E-19
ENSDARG00000029146	lrp1ab	9168.00625	1.415642741	0.14912762	9.49282761	2.25E-21	2.12E-19
ENSDARG00000044362	unc119b	1680.39495	-1.211070204	0.12785634	-9.47211692	2.74E-21	2.58E-19
ENSDARG00000045087	cdk5r1b	1696.38199	-1.055147694	0.11144779	-9.46764107	2.86E-21	2.68E-19
ENSDARG00000037539	tnnc1b	6507.13524	1.02602227	0.10867427	9.44126259	3.68E-21	3.44E-19
ENSDARG00000079327	hmcn2	5590.85993	1.081196333	0.11518232	9.38682514	6.18E-21	5.76E-19
ENSDARG00000023151	ucp1	2229.29171	1.581084538	0.16865158	9.37485734	6.93E-21	6.42E-19
ENSDARG00000055679	mto1	748.162333	0.99803525	0.10662835	9.35994262	7.98E-21	7.37E-19
ENSDARG00000087873	eevs	1957.06513	1.353261903	0.14486193	9.34173589	9.48E-21	8.72E-19
ENSDARG00000044521	eef1b2	4919.63964	1.09668512	0.11750347	9.3332148	1.03E-20	9.42E-19
ENSDARG00000038205	her2	146.593803	-2.410035874	0.2582454	-9.33234778	1.04E-20	9.46E-19
ENSDARG00000089296	si:dkey-240n22.6	231.165704	-2.321561272	0.24921371	-9.31554416	1.21E-20	1.10E-18
ENSDARG00000016854	neurod2	225.811474	-2.376652341	0.25512169	-9.31575974	1.21E-20	1.10E-18
ENSDARG00000097102	si:ch73-281n10.2	11798.8806	-1.138054472	0.12220647	-9.31255508	1.25E-20	1.13E-18
ENSDARG00000056464	fitm1l	744.766788	1.691071117	0.18161803	9.31114123	1.26E-20	1.14E-18
ENSDARG00000079119	si:ch211-229d2.5	1400.29251	-1.40568363	0.15112908	-9.3012124	1.39E-20	1.25E-18
ENSDARG00000090369	zgc:86896	10737.145	0.850122476	0.09152427	9.28849188	1.56E-20	1.40E-18
ENSDARG00000074396	fscn2b	673.58548	-1.700334437	0.18307656	-9.28755963	1.58E-20	1.41E-18
ENSDARG00000099099	CABZ01015815.1	141.007902	-3.071531308	0.33081939	-9.28461698	1.62E-20	1.44E-18
ENSDARG00000059466	wasf3a	287.105717	-1.957777579	0.21090332	-9.28282003	1.65E-20	1.46E-18
ENSDARG00000039459	qsox1	2401.23949	0.925888881	0.0999271	9.26564321	1.94E-20	1.71E-18
ENSDARG00000089724	cyldb	537.337403	2.108117558	0.22784186	9.25254716	2.19E-20	1.93E-18
ENSDARG00000054616	cldni	9077.57262	1.055834259	0.11414994	9.24953858	2.25E-20	1.97E-18
ENSDARG00000006314	itgav	9422.46139	0.929063419	0.10045047	9.24897062	2.27E-20	1.98E-18
ENSDARG00000100613	CABZ01089777.1	265.075355	-2.78588746	0.30146162	-9.24126751	2.44E-20	2.12E-18
ENSDARG00000021547	vrk2	196.318502	1.840718862	0.19935237	9.23349381	2.62E-20	2.27E-18
ENSDARG00000057121	c7b	261.741988	2.272410461	0.24612022	9.23292868	2.63E-20	2.27E-18
ENSDARG00000101090	pappaa	462.597773	1.943021117	0.21066665	9.22320242	2.88E-20	2.48E-18

Table S2. DEGs of WT vs. *terfa-/-*

ENSDARG00000011886	pdca	175.465825	-2.83036981	0.30726841	-9.21139205	3.22E-20	2.76E-18
ENSDARG00000100709	agxt2	727.683432	2.308921364	0.25081982	9.20549791	3.40E-20	2.90E-18
ENSDARG00000040008	neurod6a	404.320171	-1.664596513	0.18107659	-9.19277587	3.83E-20	3.26E-18
ENSDARG00000089458	rp1l1a	409.689039	-2.52680096	0.27492281	-9.19094682	3.89E-20	3.30E-18
ENSDARG00000018404	krt18a.1	11346.0546	0.994205538	0.10834093	9.17663819	4.45E-20	3.76E-18
ENSDARG00000038770	zgc:103625	231.022196	-2.854879799	0.31132436	-9.17011377	4.73E-20	3.98E-18
ENSDARG00000010946	cbsb	2075.83428	0.992365149	0.10825951	9.1665403	4.88E-20	4.10E-18
ENSDARG00000097746	si:rp71-77l1.1	677.328769	1.627062161	0.17765722	9.15843531	5.27E-20	4.41E-18
ENSDARG00000018820	flncb	5050.76499	1.111483182	0.12140129	9.15544778	5.41E-20	4.50E-18
ENSDARG00000056036	stxbp1b	2211.99767	-1.875004286	0.20479533	-9.15550313	5.41E-20	4.50E-18
ENSDARG00000070835	tnnc2	125241.91	0.998451388	0.10907448	9.15384949	5.49E-20	4.55E-18
ENSDARG00000031768	roraa	2223.87546	-1.205491178	0.13181911	-9.14504111	5.96E-20	4.92E-18
ENSDARG00000068474	si:dkey-94e7.2	143.973281	-3.602476815	0.39503744	-9.11933005	7.56E-20	6.22E-18
ENSDARG00000014386	galnt6	1095.03959	0.934234827	0.10246404	9.11768521	7.67E-20	6.30E-18
ENSDARG00000117485	FO681323.1	831.266605	1.386640642	0.15216676	9.11263842	8.04E-20	6.57E-18
ENSDARG00000032653	sinhcaf	600.499538	-1.326403629	0.14568107	-9.10484565	8.64E-20	7.04E-18
ENSDARG00000063665	mat2al	822.179959	1.507357712	0.16562031	9.10128527	8.93E-20	7.25E-18
ENSDARG00000026926	rom1b	499.696718	-2.679100611	0.29470452	-9.09080271	9.83E-20	7.96E-18
ENSDARG00000038378	sagb	3537.95138	-3.011325891	0.33151737	-9.08346351	1.05E-19	8.49E-18
ENSDARG00000099775	CABZ01074307.1	472.025629	1.873589852	0.20630822	9.08150867	1.07E-19	8.61E-18
ENSDARG00000093052	c6	166.264773	2.964428993	0.32645207	9.08074817	1.08E-19	8.65E-18
ENSDARG00000103788	AL645792.2	46.356197	5.77024014	0.63594949	9.07342522	1.15E-19	9.22E-18
ENSDARG00000011671	pde6b	476.021608	-2.233322745	0.24635827	-9.06534527	1.24E-19	9.90E-18
ENSDARG00000071384	slc7a11	134.444944	-2.381539245	0.2630222	-9.05451805	1.37E-19	1.09E-17
ENSDARG00000058325	casp8	313.860797	1.808809036	0.19992227	9.04756149	1.46E-19	1.16E-17
ENSDARG00000026820	gucy2d	1648.85752	-2.268451818	0.2509702	-9.03872978	1.59E-19	1.25E-17
ENSDARG00000104756	CDHR1	476.090619	-1.626128558	0.18012355	-9.02785073	1.75E-19	1.38E-17
ENSDARG00000114022	WDR1	9712.29239	0.993726288	0.11015586	9.02109358	1.86E-19	1.46E-17
ENSDARG00000035861	si:rp71-39b20.4	134.878277	-3.407828268	0.37789258	-9.01798144	1.92E-19	1.50E-17
ENSDARG00000035350	ins	645.329928	1.004887442	0.11146679	9.01512849	1.97E-19	1.53E-17
ENSDARG00000078014	pacsin2	2392.82952	0.92145679	0.10238381	9.00002428	2.26E-19	1.75E-17
ENSDARG00000094310	si:ch211-255g12.6	3461.29276	-1.289917386	0.14342878	-8.99343511	2.40E-19	1.86E-17
ENSDARG00000056347	rab3aa	1109.38337	-1.187698675	0.13207807	-8.99239871	2.42E-19	1.87E-17
ENSDARG00000028625	jph2	7546.52236	1.351356722	0.15030034	8.99104238	2.45E-19	1.88E-17
ENSDARG00000074221	ABCA7	1071.41612	-1.329576116	0.14803666	-8.98139744	2.67E-19	2.05E-17
ENSDARG00000060626	dgkaa	2035.34022	0.808433741	0.09003449	8.97915582	2.73E-19	2.09E-17
ENSDARG00000028173	slc4a2a	1098.60625	1.048515862	0.11696774	8.96414571	3.13E-19	2.38E-17
ENSDARG00000056600	papss2b	2900.27097	0.841598507	0.09405036	8.94838168	3.61E-19	2.74E-17
ENSDARG00000078102	psda	541.235174	-1.189525445	0.13299541	-8.94410894	3.75E-19	2.84E-17
ENSDARG00000104478	cap2	1751.46546	1.510469889	0.16892034	8.9419066	3.83E-19	2.89E-17
ENSDARG00000035835	eef2k	3976.81024	1.080589196	0.12088097	8.93928281	3.92E-19	2.95E-17
ENSDARG00000035066	ubtf	4779.2146	-0.843080389	0.09432699	-8.93784933	3.97E-19	2.98E-17
ENSDARG00000059035	porb	2802.00113	1.432745616	0.16036358	8.93435772	4.10E-19	3.07E-17
ENSDARG00000058656	desma	11214.3733	1.174145912	0.1314893	8.92959278	4.28E-19	3.19E-17
ENSDARG00000030265	scdb	1802.89841	-0.995175272	0.11160431	-8.91699697	4.79E-19	3.57E-17
ENSDARG00000103744	hacd1	2168.44112	0.995930114	0.11170925	8.91537713	4.86E-19	3.61E-17
ENSDARG00000101584	CU467822.2	1411.77593	-1.518956737	0.17053832	-8.90683519	5.25E-19	3.88E-17
ENSDARG00000036541	rhbdf1a	2243.49311	0.79358802	0.08914922	8.9017947	5.50E-19	4.05E-17
ENSDARG00000019063	fat1b	1046.32711	-1.552166516	0.17447674	-8.8961231	5.78E-19	4.25E-17
ENSDARG00000068242	cngb1a	470.726515	-2.475212566	0.27825849	-8.89537135	5.82E-19	4.27E-17
ENSDARG00000102473	zgc:171673	198.067722	-2.245446569	0.25246072	-8.89424129	5.88E-19	4.30E-17
ENSDARG00000006978	zak	254.576611	1.980673285	0.2227656	8.89128879	6.04E-19	4.40E-17
ENSDARG00000096003	atp5md	1754.10179	1.371847873	0.15431411	8.88997069	6.11E-19	4.44E-17
ENSDARG00000033444	map4k6	509.242001	-1.169584105	0.13164543	-8.88435018	6.43E-19	4.66E-17
ENSDARG00000001910	rorab	3273.9684	-1.052643071	0.11849728	-8.88326785	6.49E-19	4.69E-17
ENSDARG00000044815	serinc4	385.779193	-1.786644182	0.20128384	-8.87624239	6.92E-19	4.98E-17
ENSDARG00000028071	bmp1a	4999.46917	1.011635984	0.11401551	8.8727931	7.13E-19	5.12E-17
ENSDARG00000023181	pcp4l1	341.129767	-1.917093997	0.2163563	-8.86081907	7.94E-19	5.67E-17

Table S2. DEGs of WT vs. *terfa-/-*

ENSDARG00000013351	cirbpb	63432.0271	-1.100967136	0.12425074	-8.86084967	7.94E-19	5.67E-17
ENSDARG00000036754	hmgn3	2475.50183	-1.378284748	0.15556113	-8.86008459	8.00E-19	5.68E-17
ENSDARG00000030915	cpa1	109.078488	-3.971203074	0.44820766	-8.86018562	7.99E-19	5.68E-17
ENSDARG00000052223	rcvrna	1432.54447	-3.397638191	0.38352942	-8.85887237	8.08E-19	5.72E-17
ENSDARG00000038822	mrc1b	545.77548	1.634809245	0.18463406	8.85432097	8.42E-19	5.95E-17
ENSDARG00000104897	si:dkey-16p6.1	45.0709063	5.729980968	0.6471817	8.85374381	8.46E-19	5.96E-17
ENSDARG00000091131	cry3b	1867.38591	-1.097274066	0.12398242	-8.85023905	8.73E-19	6.13E-17
ENSDARG00000028017	tp53inp1	979.132041	1.089322935	0.12314915	8.84555765	9.11E-19	6.38E-17
ENSDARG00000091260	mylk4a	866.237663	1.650858111	0.18688825	8.83339707	1.02E-18	7.09E-17
ENSDARG00000029615	zgc:77056	575.547559	-1.12014334	0.12695262	-8.82331825	1.11E-18	7.74E-17
ENSDARG00000017532	kif25	140.699306	-2.359968717	0.26762246	-8.8182759	1.16E-18	8.07E-17
ENSDARG00000113896	si:dkey-29p23.1	117.123444	-2.80435194	0.31806387	-8.81694582	1.18E-18	8.14E-17
ENSDARG00000012395	mmp13a	130.970498	3.119399745	0.35401337	8.81153083	1.23E-18	8.52E-17
ENSDARG00000101407	tgm1l4	124.903063	2.626481096	0.2982337	8.80678854	1.29E-18	8.87E-17
ENSDARG00000020625	jak1	4534.43191	1.151423058	0.13075568	8.80591244	1.30E-18	8.91E-17
ENSDARG00000061890	atp13a2	561.556315	1.100186266	0.12505771	8.79742857	1.40E-18	9.59E-17
ENSDARG00000036140	crybgx	1564.16009	-1.231029999	0.1399722	-8.79481763	1.43E-18	9.78E-17
ENSDARG00000033160	nr1d1	1847.35347	2.026888811	0.23066815	8.78703376	1.54E-18	1.05E-16
ENSDARG00000099793	cspg5b	1724.75419	-1.226490236	0.13973635	-8.77717415	1.68E-18	1.14E-16
ENSDARG00000100555	aqp11	1318.63052	1.15869862	0.13207334	8.77314568	1.74E-18	1.18E-16
ENSDARG00000074656	ctss2.1	250.805408	1.664692457	0.19027305	8.74896598	2.15E-18	1.45E-16
ENSDARG00000102626	frem2b	5266.04246	1.107475973	0.12658568	8.74882488	2.16E-18	1.45E-16
ENSDARG00000087349	reep6	140.087533	-2.307836493	0.26400067	-8.74178274	2.29E-18	1.54E-16
ENSDARG00000021287	rab7b	544.781664	1.370310571	0.15690658	8.73328956	2.47E-18	1.66E-16
ENSDARG00000042727	exo5	238.418254	1.455161235	0.16672592	8.72786441	2.60E-18	1.73E-16
ENSDARG00000010085	p4ha2	1154.16731	1.162284223	0.13320964	8.7252258	2.66E-18	1.77E-16
ENSDARG00000026979	krt1-c5	646.824763	-1.488789002	0.17076755	-8.71821982	2.83E-18	1.88E-16
ENSDARG00000019013	barhl1b	531.094002	-1.688526551	0.19372532	-8.71608624	2.88E-18	1.91E-16
ENSDARG00000076146	zgc:172075	166.367676	2.080506714	0.23873154	8.71483811	2.91E-18	1.92E-16
ENSDARG00000039997	ptp4a3a	2234.85542	1.016878573	0.11668351	8.71484379	2.91E-18	1.92E-16
ENSDARG00000051857	tes	1002.24351	1.240648338	0.14236633	8.71447877	2.92E-18	1.92E-16
ENSDARG00000100506	crb1	439.170215	-1.46630991	0.16829988	-8.71248347	2.97E-18	1.95E-16
ENSDARG00000074892	pde6d	321.899341	-1.684740414	0.19343695	-8.70950656	3.05E-18	1.99E-16
ENSDARG00000104693	il6st	3607.64271	1.594998661	0.18313247	8.70953504	3.05E-18	1.99E-16
ENSDARG00000111465	znf1104	129.001728	2.726102064	0.31319103	8.70427885	3.20E-18	2.08E-16
ENSDARG00000032705	foxg1b	2212.0375	-1.819081978	0.20934417	-8.6894322	3.64E-18	2.36E-16
ENSDARG00000053502	cryaa	823.374982	-1.805388538	0.20790181	-8.68385214	3.83E-18	2.48E-16
ENSDARG00000086746	prodha	1254.94741	1.288059037	0.14840381	8.67942027	3.98E-18	2.57E-16
ENSDARG00000101482	hk2	1035.97479	0.954785174	0.1100435	8.6764338	4.08E-18	2.63E-16
ENSDARG00000059792	trpm5	247.637145	-1.826620234	0.21073831	-8.66771812	4.41E-18	2.83E-16
ENSDARG00000100654	si:dkey-207m2.4	106.264795	-2.657737083	0.30708292	-8.6547863	4.94E-18	3.16E-16
ENSDARG00000006422	esyt3	593.059759	1.477383726	0.17071792	8.65394615	4.97E-18	3.17E-16
ENSDARG00000021082	sec31a	2852.52778	0.916456837	0.10589737	8.65419791	4.96E-18	3.17E-16
ENSDARG00000077341	ppp1r14c	6526.39828	-1.44929482	0.1678786	-8.63299334	5.98E-18	3.80E-16
ENSDARG00000069451	gja8a	112.536195	-2.318190869	0.26884141	-8.62289349	6.53E-18	4.14E-16
ENSDARG00000104403	oscp1a	77.1656994	-3.080049286	0.35729477	-8.62047115	6.67E-18	4.22E-16
ENSDARG00000104359	anxa1c	1685.18778	1.45592864	0.16914234	8.60771232	7.45E-18	4.70E-16
ENSDARG00000002634	b4galt1	579.325322	1.300913735	0.1512935	8.59860933	8.07E-18	5.08E-16
ENSDARG00000055463	lrit3a	334.606112	-2.681331759	0.31244165	-8.58186394	9.33E-18	5.86E-16
ENSDARG00000100826	hif1al	11792.2528	0.75463106	0.08815235	8.5605323	1.12E-17	7.03E-16
ENSDARG00000042172	c7a	1128.89457	1.463196033	0.17096143	8.55863254	1.14E-17	7.13E-16
ENSDARG00000012381	hsc70	6309.14625	1.404023215	0.16451331	8.53440489	1.41E-17	8.78E-16
ENSDARG00000095280	CU682811.3	134.572106	2.518652637	0.2952363	8.53097213	1.45E-17	9.02E-16
ENSDARG00000090228	gsta.1	3376.87483	-1.0828146	0.12699074	-8.5267209	1.51E-17	9.33E-16
ENSDARG00000078522	si:ch211-80h18.1	3893.11214	0.909127143	0.10677339	8.51454789	1.67E-17	1.03E-15
ENSDARG00000029493	f9b	326.35413	1.317971869	0.15478554	8.51482584	1.67E-17	1.03E-15
ENSDARG00000051861	pkp3a	4836.69575	0.867084873	0.10189217	8.50982824	1.74E-17	1.07E-15
ENSDARG00000058222	mpp4a	282.73075	-1.957453968	0.23015638	-8.50488682	1.82E-17	1.12E-15

Table S2. DEGs of WT vs. *terfa-/-*

ENSDARG00000053831	vtnb	3466.48794	0.867285865	0.10207189	8.49681431	1.95E-17	1.19E-15
ENSDARG00000053862	crygmx	3250.00179	-1.43695366	0.16914054	-8.49561958	1.97E-17	1.20E-15
ENSDARG00000075261	timp2b	1048.55446	2.152469421	0.25337935	8.49504687	1.98E-17	1.21E-15
ENSDARG00000075295	tulp1a	724.584872	-3.657143258	0.43054433	-8.49423158	1.99E-17	1.21E-15
ENSDARG00000023111	plg	4443.63401	1.043687968	0.12288577	8.49315543	2.01E-17	1.22E-15
ENSDARG00000041232	rps29	15867.5318	1.120264341	0.13205631	8.48323213	2.19E-17	1.32E-15
ENSDARG00000068374	si:ch211-132b12.7	309.068533	2.811050006	0.33148202	8.48024876	2.25E-17	1.36E-15
ENSDARG00000040623	fosl2	938.363067	1.833184845	0.2162826	8.47587765	2.33E-17	1.40E-15
ENSDARG00000027611	cavin2a	2634.87202	0.811856556	0.09581053	8.47356308	2.38E-17	1.43E-15
ENSDARG00000062727	cep290	1956.71117	-0.897466824	0.10594076	-8.47140278	2.42E-17	1.45E-15
ENSDARG00000020086	nuak1a	937.096115	1.728834735	0.20419466	8.46660096	2.53E-17	1.51E-15
ENSDARG00000063435	trpm4b.2	892.864182	1.268676075	0.14985869	8.46581583	2.54E-17	1.52E-15
ENSDARG00000061918	cplx2	2635.00402	-0.951451505	0.11247352	-8.45933771	2.69E-17	1.60E-15
ENSDARG00000013670	hyou1	3934.2913	0.809495055	0.0956977	8.45887655	2.70E-17	1.60E-15
ENSDARG00000042658	acad8	807.739679	1.042333619	0.12324289	8.45755567	2.73E-17	1.61E-15
ENSDARG00000075948	slco4a1	261.988286	-1.811955072	0.21422292	-8.45826883	2.71E-17	1.61E-15
ENSDARG00000045946	sec24d	2962.40418	0.87670502	0.10368856	8.45517638	2.79E-17	1.64E-15
ENSDARG00000043446	efhd1	976.170882	-1.280815847	0.15153851	-8.45208161	2.86E-17	1.68E-15
ENSDARG00000062750	si:ch73-74h11.1	2328.75344	1.022394314	0.12104256	8.44656911	3.00E-17	1.76E-15
ENSDARG00000099351	igfbp1a	1077.54816	2.077924509	0.24609986	8.44342014	3.08E-17	1.80E-15
ENSDARG00000021462	daw1	196.617846	1.919423619	0.2280403	8.41703692	3.86E-17	2.25E-15
ENSDARG00000045548	lepb	47.7752906	7.504740626	0.89189718	8.41435632	3.95E-17	2.30E-15
ENSDARG00000113660	si:ch211-196f19.1	94.2609835	3.088517598	0.36709842	8.41332315	3.99E-17	2.31E-15
ENSDARG00000098924	suz12b	1885.53118	-0.866512425	0.1030076	-8.41212117	4.03E-17	2.33E-15
ENSDARG00000045958	egfl6	4982.03866	0.753844881	0.08967456	8.40645224	4.23E-17	2.43E-15
ENSDARG00000098392	ly6pge	1371.51826	-3.329307228	0.39603323	-8.40663602	4.22E-17	2.43E-15
ENSDARG00000096700	FP565451.1	69.2309371	2.845533386	0.33849286	8.40647992	4.22E-17	2.43E-15
ENSDARG00000042387	zgc:77752	102.91511	-2.97117585	0.35354317	-8.40399736	4.32E-17	2.48E-15
ENSDARG00000090401	CABZ01020840.1	161.583987	2.528380782	0.30104773	8.39860446	4.52E-17	2.59E-15
ENSDARG00000087636	hmgn6	7704.76929	-1.145645583	0.13646142	-8.39538073	4.64E-17	2.65E-15
ENSDARG00000076557	bicc2	281.872053	1.667744355	0.19867865	8.39418019	4.69E-17	2.67E-15
ENSDARG00000057169	abca4a	440.689044	-1.706987746	0.2033575	-8.39402419	4.70E-17	2.67E-15
ENSDARG00000094965	nfil3-5	3817.17772	-0.90535279	0.10796076	-8.38594291	5.03E-17	2.86E-15
ENSDARG00000045811	lrtm2b	160.834282	-2.210410603	0.26363417	-8.38438585	5.10E-17	2.89E-15
ENSDARG00000025903	lgals9l1	210.299696	1.976664538	0.23589761	8.37933279	5.32E-17	3.01E-15
ENSDARG00000042988	slc24a2	1206.11999	-1.869560196	0.22313751	-8.37851157	5.36E-17	3.02E-15
ENSDARG00000042221	mthfd1l	6716.42904	0.778886172	0.09300677	8.37451051	5.55E-17	3.11E-15
ENSDARG00000054060	pof1b	2603.28422	0.851937875	0.10172886	8.37459392	5.54E-17	3.11E-15
ENSDARG00000019950	carf	747.438408	0.975597986	0.11651337	8.37327103	5.60E-17	3.14E-15
ENSDARG00000052779	zgc:153932	189.509459	2.943318305	0.3518387	8.36553309	5.98E-17	3.34E-15
ENSDARG00000091669	bicdl2l	847.054463	1.27315727	0.1524633	8.35058182	6.79E-17	3.79E-15
ENSDARG00000053010	lpcat2	571.459199	1.315259121	0.15753857	8.34880696	6.90E-17	3.83E-15
ENSDARG00000036139	lctla	544.832532	-1.832161698	0.21965249	-8.34118348	7.36E-17	4.07E-15
ENSDARG00000060682	agr1	2665.23266	1.157507048	0.13876658	8.34139636	7.34E-17	4.07E-15
ENSDARG00000098223	AL773558.1	138.706895	3.648478695	0.43744179	8.34048963	7.40E-17	4.09E-15
ENSDARG00000100625	si:ch73-197b13.1	233.980766	-1.860070772	0.22306403	-8.33873035	7.51E-17	4.14E-15
ENSDARG00000100708	jakmip3	470.642126	-1.152541879	0.13824046	-8.3372254	7.61E-17	4.18E-15
ENSDARG00000071497	zic3	1094.52644	-1.075391164	0.129049	-8.3332002	7.87E-17	4.32E-15
ENSDARG00000101363	ano2b	642.722978	-1.219596977	0.14637097	-8.33223267	7.93E-17	4.34E-15
ENSDARG00000009021	chrna1	2634.16119	0.988404	0.11899761	8.30608278	9.89E-17	5.40E-15
ENSDARG00000117160	CABZ01034596.1	98.8697847	10.0147585	1.20632845	8.30185054	1.03E-16	5.59E-15
ENSDARG00000022615	si:ch211-241e1.3	602.10605	1.568099849	0.18903355	8.29535193	1.08E-16	5.89E-15
ENSDARG00000097576	zgc:92912	402.005344	-1.156140201	0.13939833	-8.29378832	1.10E-16	5.94E-15
ENSDARG00000055014	si:dkey-33m11.8	214.324926	-1.994997098	0.24054268	-8.29373435	1.10E-16	5.94E-15
ENSDARG00000104537	cox7c	4187.08015	0.953958315	0.11509494	8.28844724	1.15E-16	6.20E-15
ENSDARG00000035860	rps28	10722.9627	1.164427593	0.14049874	8.28781505	1.15E-16	6.22E-15
ENSDARG00000058553	sprb	276.915176	2.330048005	0.28120989	8.28579687	1.17E-16	6.31E-15
ENSDARG00000058358	krt8	40898.5192	0.944543066	0.11400485	8.28511269	1.18E-16	6.33E-15

Table S2. DEGs of WT vs. *terfa-/-*

ENSDARG00000104244	twsg1a	1291.54428	1.152955287	0.13923715	8.28051516	1.23E-16	6.57E-15
ENSDARG00000078244	si:ch211-197l9.2	493.330127	-1.298629016	0.1568701	-8.27837193	1.25E-16	6.67E-15
ENSDARG00000045976	sidt2	3210.92712	0.876506506	0.10592296	8.27494313	1.29E-16	6.85E-15
ENSDARG00000037838	unkl	2080.82523	-0.774362048	0.09361316	-8.27193594	1.32E-16	7.01E-15
ENSDARG00000111840	cxcl18a.1	71.6138522	3.067399571	0.37083733	8.27155013	1.32E-16	7.02E-15
ENSDARG00000086647	chrng	332.091833	1.588757052	0.19209336	8.27075476	1.33E-16	7.05E-15
ENSDARG00000052728	sltm	5299.79514	-0.860809004	0.10411262	-8.26805644	1.36E-16	7.20E-15
ENSDARG00000071558	fblim1	558.434188	1.35358042	0.16388006	8.25957959	1.46E-16	7.71E-15
ENSDARG00000030626	lrit2	102.185333	-3.884130937	0.47059222	-8.25370828	1.54E-16	8.09E-15
ENSDARG00000038151	zgc:92360	2041.53121	0.874976836	0.10609079	8.24743483	1.62E-16	8.50E-15
ENSDARG00000092499	si:ch211-183d21.1	1357.02927	1.604767598	0.19462586	8.24539747	1.65E-16	8.63E-15
ENSDARG00000103854	pdlim7	2754.25961	0.870593055	0.10559175	8.24489692	1.65E-16	8.65E-15
ENSDARG00000000606	dnah6	307.469817	1.677255309	0.20348622	8.24259882	1.69E-16	8.80E-15
ENSDARG00000102207	zgc:86738	678.945478	1.33945442	0.1625386	8.240839	1.71E-16	8.91E-15
ENSDARG00000057113	c6	1024.02992	1.818527271	0.22073214	8.23861585	1.74E-16	9.06E-15
ENSDARG00000089340	rhobtb2b	639.083995	1.630744029	0.19802515	8.23503502	1.80E-16	9.29E-15
ENSDARG00000090847	si:ch211-209l18.4	623.128624	1.430782734	0.17374083	8.23515547	1.79E-16	9.29E-15
ENSDARG00000102299	cxcl8b.1	73.5920854	3.049113197	0.37030648	8.23402614	1.81E-16	9.35E-15
ENSDARG00000014907	htra1b	814.321541	2.120137107	0.25770615	8.22695574	1.92E-16	9.90E-15
ENSDARG00000054537	nhsl1a	1794.38175	-0.924859574	0.11246535	-8.22350684	1.98E-16	1.02E-14
ENSDARG00000100311	si:dkey-165e24.1	291.148537	1.661081467	0.20202981	8.22196224	2.00E-16	1.03E-14
ENSDARG00000022817	pvalb3	292.885942	1.796303923	0.21880647	8.20955569	2.22E-16	1.14E-14
ENSDARG00000114818	CU633479.5	342.97243	1.733493442	0.21123423	8.20649895	2.28E-16	1.16E-14
ENSDARG00000104375	sec24a	4686.11075	0.679462349	0.08280832	8.20524304	2.30E-16	1.17E-14
ENSDARG00000092807	si:dkey-151g10.6	44184.6932	0.886861638	0.10814923	8.20035108	2.40E-16	1.22E-14
ENSDARG00000057590	si:ch1073-396h14.	736.752575	1.028589887	0.12555662	8.19223944	2.56E-16	1.30E-14
ENSDARG00000057527	mef2ab	260.017575	-1.986185084	0.24277888	-8.18104553	2.81E-16	1.43E-14
ENSDARG00000005776	guk1b	948.084757	-3.447828967	0.42206449	-8.16896251	3.11E-16	1.57E-14
ENSDARG00000060153	h6pd	1181.94933	1.157456723	0.14170906	8.16783837	3.14E-16	1.58E-14
ENSDARG00000055400	si:dkey-56f14.7	666.145822	-1.2923183	0.15820784	-8.16848453	3.12E-16	1.58E-14
ENSDARG00000043148	slc1a3b	3486.28749	1.531143154	0.1875208	8.165191	3.21E-16	1.61E-14
ENSDARG00000036830	krt91	82725.448	1.001756488	0.12281713	8.15648857	3.45E-16	1.73E-14
ENSDARG00000103454	trpm6	659.833982	1.123444341	0.13816607	8.13111581	4.25E-16	2.13E-14
ENSDARG00000037961	rcn3	2803.13451	1.192984978	0.14683735	8.12453363	4.49E-16	2.25E-14
ENSDARG00000022303	higd1a	1037.33637	1.153018153	0.14198529	8.12068717	4.64E-16	2.31E-14
ENSDARG00000105279	si:ch211-108c17.2	387.300412	1.352739667	0.16675219	8.11227543	4.97E-16	2.47E-14
ENSDARG00000103019	gstp2	743.684332	1.20273827	0.14838121	8.10573157	5.24E-16	2.61E-14
ENSDARG00000038780	ubtfl	2464.69381	-0.824604677	0.10184879	-8.09636203	5.66E-16	2.81E-14
ENSDARG00000031316	six6b	670.199077	-1.658415593	0.20514668	-8.08404796	6.27E-16	3.10E-14
ENSDARG00000074285	si:ch73-335m24.2	181.928085	-2.152669559	0.26670764	-8.07127082	6.96E-16	3.44E-14
ENSDARG00000098753	elmo1	3681.14905	-0.746070537	0.09258195	-8.05848797	7.72E-16	3.81E-14
ENSDARG00000042245	myl13	15730.204	0.871632707	0.10817934	8.05729401	7.80E-16	3.84E-14
ENSDARG00000036876	zgc:153284	404.439998	1.480158976	0.18370945	8.05706495	7.81E-16	3.84E-14
ENSDARG00000020544	rem1	348.60569	-1.32276354	0.16420983	-8.05532475	7.93E-16	3.89E-14
ENSDARG00000075837	si:dkey-1d7.3	708.374326	-1.837148248	0.22809375	-8.05435606	7.99E-16	3.91E-14
ENSDARG00000089901	si:ch211-261n11.8	71.6905762	9.744489907	1.21017322	8.05214473	8.14E-16	3.97E-14
ENSDARG00000087440	ponzr4	614.56782	1.907897784	0.23697604	8.05101561	8.21E-16	4.00E-14
ENSDARG00000006467	pcdh8	534.138687	-2.315399745	0.28802333	-8.0389312	9.06E-16	4.41E-14
ENSDARG00000034940	slc1a7a	285.832932	-2.175925332	0.27089475	-8.03236424	9.56E-16	4.64E-14
ENSDARG00000018006	vrk1	459.878727	-1.140825774	0.14223489	-8.02071662	1.05E-15	5.09E-14
ENSDARG00000057303	galnt7	2611.58306	0.696422732	0.08686335	8.01745201	1.08E-15	5.22E-14
ENSDARG00000070651	prkcdb	2733.20946	1.060335272	0.1323505	8.01156964	1.13E-15	5.47E-14
ENSDARG00000098374	FLNB	3844.36007	1.050685731	0.13128935	8.00282555	1.22E-15	5.86E-14
ENSDARG00000077306	cutc	574.379295	1.123334708	0.14041967	7.99983853	1.25E-15	5.99E-14
ENSDARG00000055504	si:ch211-212k18.7	2285.84171	0.765705492	0.09576339	7.99580632	1.29E-15	6.17E-14
ENSDARG00000103311	fbln1	2825.09001	0.910654992	0.11389218	7.99576368	1.29E-15	6.17E-14
ENSDARG00000060127	adamts3	542.077381	1.190392309	0.14899246	7.98961469	1.35E-15	6.47E-14
ENSDARG00000070666	rhol	145.18849	-3.370555409	0.42200871	-7.98693325	1.38E-15	6.60E-14

Table S2. DEGs of WT vs. *terfa-/-*

ENSDARG00000071877	dhrs7cb	1516.44629	1.142175644	0.14308241	7.98264189	1.43E-15	6.81E-14
ENSDARG00000111612	mapkapk3	391.140117	1.170176136	0.14659161	7.98255875	1.43E-15	6.81E-14
ENSDARG00000057992	fstb	1147.05341	1.201362856	0.15056866	7.97883746	1.48E-15	7.01E-14
ENSDARG00000002771	SLC4A5	459.226814	-2.101032105	0.2633831	-7.9770953	1.50E-15	7.09E-14
ENSDARG00000039066	klhl31	5488.64958	0.730639391	0.09161202	7.97536636	1.52E-15	7.18E-14
ENSDARG00000077760	ascc1	266.575089	2.207228463	0.27687325	7.97198178	1.56E-15	7.36E-14
ENSDARG00000093381	tgm2l	489.795526	1.595285667	0.20016752	7.96975291	1.59E-15	7.48E-14
ENSDARG00000104125	ttc6	196.485699	1.917430307	0.24072616	7.96519295	1.65E-15	7.75E-14
ENSDARG00000013335	anxa6	3106.69533	1.148703198	0.14424888	7.96334219	1.67E-15	7.85E-14
ENSDARG00000019815	fn1a	9626.90893	0.90539079	0.11379872	7.95607175	1.78E-15	8.31E-14
ENSDARG00000096849	si:dkey-16p21.8	7869.85559	0.818638297	0.10296285	7.95081235	1.85E-15	8.66E-14
ENSDARG00000053480	aqp9b	928.11768	-2.882794079	0.36288901	-7.94401046	1.96E-15	9.13E-14
ENSDARG00000057403	CR847953.1	821.759455	-0.871742311	0.10978904	-7.94015767	2.02E-15	9.40E-14
ENSDARG00000092204	ms4a17a.5	147.114798	2.310404958	0.29119584	7.93419643	2.12E-15	9.84E-14
ENSDARG00000094488	BX510923.1	559.725111	-1.352423021	0.17047496	-7.9332651	2.13E-15	9.90E-14
ENSDARG00000018637	sec61g	398.550278	2.332814705	0.2941346	7.93111291	2.17E-15	1.00E-13
ENSDARG00000030270	tnnt3a	18952.4885	0.88243195	0.11125847	7.93136874	2.17E-15	1.00E-13
ENSDARG00000094331	BX901918.2	155.638321	10.63475503	1.34257198	7.92118055	2.35E-15	1.08E-13
ENSDARG00000037487	1-Mar	226.723205	-2.04425541	0.25820234	-7.91726134	2.43E-15	1.12E-13
ENSDARG00000004932	anos1b	1095.70278	0.855016281	0.10801813	7.91548885	2.46E-15	1.13E-13
ENSDARG00000102441	ctnna1	7054.96692	0.656853902	0.08301995	7.91200027	2.53E-15	1.16E-13
ENSDARG00000034227	si:dkey-243i1.1	366.044978	-1.500784713	0.18974066	-7.90966319	2.58E-15	1.18E-13
ENSDARG00000112772	CR381647.5	323.097179	-1.242761847	0.15719788	-7.90571654	2.66E-15	1.22E-13
ENSDARG00000101634	si:ch211-209j12.1	54.7806416	4.015775837	0.50820533	7.90187675	2.75E-15	1.25E-13
ENSDARG00000026376	aco1	2078.36427	1.083651519	0.13716971	7.90007905	2.79E-15	1.27E-13
ENSDARG00000059442	smtnb	1475.93346	-1.337165387	0.16928492	-7.89890427	2.81E-15	1.27E-13
ENSDARG00000095767	dio3b	978.528503	1.643264456	0.20803147	7.8991146	2.81E-15	1.27E-13
ENSDARG00000101959	etv1	482.394067	-1.239477986	0.15689231	-7.90018322	2.78E-15	1.27E-13
ENSDARG00000045089	si:ch211-270n8.1	445.748929	3.49288388	0.44292798	7.88589569	3.12E-15	1.41E-13
ENSDARG00000060900	znf362a	1593.93721	-0.734845185	0.09320966	-7.88378769	3.18E-15	1.43E-13
ENSDARG00000035797	si:dkey-17m8.2	78.1233384	7.062045933	0.89665968	7.87594901	3.38E-15	1.52E-13
ENSDARG00000092337	CU467861.6	1008.34214	1.664613571	0.21148892	7.87092556	3.52E-15	1.58E-13
ENSDARG00000102759	TMEM132D	268.771817	-1.614892096	0.20526526	-7.86734251	3.62E-15	1.63E-13
ENSDARG00000105095	AL929028.8	45.38144	4.978977325	0.63341531	7.86052576	3.83E-15	1.71E-13
ENSDARG00000095252	si:dkey-40h20.1	58.0882363	4.070401853	0.51834309	7.85271746	4.07E-15	1.82E-13
ENSDARG00000017354	epha2a	899.937783	0.820411238	0.10450908	7.85014283	4.16E-15	1.85E-13
ENSDARG00000019601	col12a1b	15448.1177	1.295491978	0.16503331	7.84988168	4.16E-15	1.85E-13
ENSDARG00000071549	ecm2	976.309451	0.905081921	0.11529344	7.8502462	4.15E-15	1.85E-13
ENSDARG00000041382	si:dkey-283b15.2	834.74129	-1.239185356	0.1580909	-7.83843591	4.56E-15	2.02E-13
ENSDARG00000062909	furina	1521.31099	0.807546146	0.10306342	7.83542903	4.67E-15	2.07E-13
ENSDARG00000019566	neurod1	6561.12182	-2.472214958	0.31594219	-7.82489668	5.08E-15	2.25E-13
ENSDARG00000006112	myof	1676.92882	0.989595269	0.12650531	7.82255926	5.18E-15	2.29E-13
ENSDARG00000055377	gnb5b	1354.23164	-1.712063158	0.21888717	-7.82166977	5.21E-15	2.30E-13
ENSDARG00000105674	ms4a17a.2	73.4428752	3.515434693	0.44955496	7.81981067	5.29E-15	2.33E-13
ENSDARG00000077367	ntng2a	574.873898	-1.18970992	0.15214504	-7.81957761	5.30E-15	2.33E-13
ENSDARG00000060345	apoda.1	593.055849	-1.277846876	0.16355694	-7.81285629	5.59E-15	2.45E-13
ENSDARG00000045880	gas2l3	487.988358	-1.329348288	0.17022085	-7.80955029	5.74E-15	2.51E-13
ENSDARG00000103515	vcana	141.481666	1.794151264	0.22984863	7.80579494	5.91E-15	2.58E-13
ENSDARG00000028228	zbtb18	3894.91684	-1.420995784	0.18203513	-7.80616239	5.90E-15	2.58E-13
ENSDARG00000059280	hoxd3a	1009.13683	-0.834157601	0.10699484	-7.79624109	6.38E-15	2.78E-13
ENSDARG00000007836	ctsla	27813.5047	1.176603708	0.15094562	7.79488499	6.45E-15	2.80E-13
ENSDARG00000099511	CABZ01034698.2	158.878538	2.247647115	0.28910061	7.77461903	7.57E-15	3.28E-13
ENSDARG00000100660	si:ch73-252i11.1	152.523378	1.682468755	0.21680464	7.76029863	8.47E-15	3.67E-13
ENSDARG00000056938	kera	2242.78337	-1.559233424	0.20111794	-7.75283101	8.99E-15	3.88E-13
ENSDARG00000009336	aif1l	1457.39571	-0.929941295	0.12000895	-7.748933	9.27E-15	4.00E-13
ENSDARG00000067741	itpkcb	1666.42488	1.139609505	0.14720414	7.74169453	9.81E-15	4.23E-13
ENSDARG00000008026	niban2b	970.392117	0.838947702	0.10851355	7.7312711	1.06E-14	4.58E-13
ENSDARG00000089232	prr14	1376.20919	-0.926040258	0.11982133	-7.72850952	1.09E-14	4.67E-13

Table S2. DEGs of WT vs. *terfa-/-*

ENSDARG00000102430	opn6a	678.865126	-3.050745357	0.39487812	-7.72578976	1.11E-14	4.76E-13
ENSDARG00000105341	si:dkey-9l20.3	4265.25371	1.253340768	0.16232429	7.72121535	1.15E-14	4.93E-13
ENSDARG00000032242	tnnt2c	1233.26113	1.374562126	0.1783034	7.70911884	1.27E-14	5.41E-13
ENSDARG00000061896	slco2a1	432.447373	1.122137151	0.14570159	7.70161202	1.34E-14	5.73E-13
ENSDARG00000051748	ccnd2a	1243.17235	0.977678383	0.12696224	7.70054462	1.35E-14	5.77E-13
ENSDARG00000101388	si:ch211-171l17.1	35.8829617	3.994692629	0.51924575	7.69326014	1.43E-14	6.09E-13
ENSDARG00000070108	dek	988.632407	-1.092253863	0.14202936	-7.69033833	1.47E-14	6.22E-13
ENSDARG00000010727	ttyh2l	887.078583	-1.086383146	0.14133288	-7.686698	1.51E-14	6.39E-13
ENSDARG00000055439	adamtsl7	519.313662	1.231326569	0.1602134	7.68554047	1.52E-14	6.44E-13
ENSDARG00000032929	cryba1l1	11065.9526	-1.66832157	0.21715452	-7.68264735	1.56E-14	6.57E-13
ENSDARG00000101754	BX664622.1	93.0855287	3.042353348	0.39600552	7.68260339	1.56E-14	6.57E-13
ENSDARG00000007370	dysf	1901.57516	1.115549296	0.14533768	7.67556817	1.65E-14	6.93E-13
ENSDARG00000016457	irf9	366.620065	1.483141449	0.19330124	7.67269488	1.68E-14	7.07E-13
ENSDARG00000059993	trpm4a	1519.14758	0.975683065	0.12717527	7.67195609	1.69E-14	7.10E-13
ENSDARG00000104672	CABZ01074397.1	200.430789	1.915758409	0.24981622	7.6686712	1.74E-14	7.27E-13
ENSDARG00000039077	tyr	1040.8317	1.346405014	0.17558011	7.66832334	1.74E-14	7.28E-13
ENSDARG00000018530	mapkapk2b	113.437639	-3.02333892	0.39476447	-7.65858925	1.88E-14	7.84E-13
ENSDARG00000101438	lonp2	1191.61333	0.894188391	0.11679756	7.65588252	1.92E-14	7.99E-13
ENSDARG00000117448	CABZ01072245.1	926.526455	1.357009365	0.17730163	7.65367691	1.95E-14	8.12E-13
ENSDARG00000104361	barhl2	879.925542	-1.559383707	0.20377361	-7.65253017	1.97E-14	8.18E-13
ENSDARG00000022372	kng1	1412.11532	0.987284525	0.1291319	7.64555084	2.08E-14	8.62E-13
ENSDARG00000019902	rcvrn2	4155.5619	-3.398913433	0.44479698	-7.64149393	2.15E-14	8.88E-13
ENSDARG00000098332	zgc:112970	56.1132183	3.372211674	0.44132233	7.64115358	2.15E-14	8.89E-13
ENSDARG00000044199	gnat1	1768.57287	-3.301743833	0.4324061	-7.63574754	2.25E-14	9.25E-13
ENSDARG00000103186	si:ch73-389k6.1	246.154605	-2.820748057	0.36968173	-7.63020675	2.34E-14	9.64E-13
ENSDARG00000089326	arhgap20	180.081717	-1.827172253	0.23957475	-7.62673128	2.41E-14	9.89E-13
ENSDARG00000074210	zgc:110286	34.3009288	4.218867296	0.55339321	7.62363403	2.47E-14	1.01E-12
ENSDARG00000071076	ldhbb	643.702793	-1.594589446	0.2092585	-7.62018958	2.53E-14	1.04E-12
ENSDARG00000105731	CR788316.1	384.394345	2.101077234	0.27579564	7.61823942	2.57E-14	1.05E-12
ENSDARG00000010146	cpa2	57.409444	2.657189681	0.34889437	7.61602915	2.62E-14	1.07E-12
ENSDARG00000098746	dhrs13l1	615.459077	1.105632932	0.14539271	7.60445898	2.86E-14	1.17E-12
ENSDARG00000095139	CR388373.2	75.0314298	-2.489785688	0.32745594	-7.60342197	2.88E-14	1.17E-12
ENSDARG00000037655	pls3	6588.91322	0.795283142	0.10461243	7.60218562	2.91E-14	1.18E-12
ENSDARG00000021838	rps23	27515.3593	0.762769222	0.10033635	7.60212285	2.91E-14	1.18E-12
ENSDARG00000101826	zwi	562.501997	-1.57697715	0.20747772	-7.60070592	2.95E-14	1.19E-12
ENSDARG00000013371	isoc2	761.222157	0.981642411	0.12919573	7.59810278	3.01E-14	1.21E-12
ENSDARG00000079467	si:ch73-15b2.5	288.98661	1.636702056	0.21567129	7.5888731	3.23E-14	1.30E-12
ENSDARG00000059826	crtac1a	535.681496	-2.120146887	0.27948414	-7.58592912	3.30E-14	1.33E-12
ENSDARG00000043483	otx5	1765.24985	-1.655116725	0.21822123	-7.58458162	3.34E-14	1.34E-12
ENSDARG00000037517	slc35c2	3364.7792	0.730737965	0.09634784	7.58437293	3.34E-14	1.34E-12
ENSDARG00000097653	si:dkey-23k10.3	57.8869379	3.083344558	0.40667981	7.58174973	3.41E-14	1.37E-12
ENSDARG00000005015	tspy	1511.48651	-0.834651636	0.11022977	-7.57192595	3.68E-14	1.47E-12
ENSDARG00000101924	si:dkey-65b13.13	111.3478	-2.180989379	0.28818029	-7.56814222	3.79E-14	1.51E-12
ENSDARG00000039328	ccndbp1	1251.05819	1.263622515	0.16702706	7.56537627	3.87E-14	1.54E-12
ENSDARG00000023217	crema	687.358839	1.414717905	0.18702485	7.56433101	3.90E-14	1.55E-12
ENSDARG00000013777	ppp1r13l	2104.3884	0.790161616	0.10447373	7.56325628	3.93E-14	1.56E-12
ENSDARG00000007804	gas6	1662.49785	0.714257158	0.09444792	7.56244433	3.96E-14	1.57E-12
ENSDARG00000093921	BX276103.2	43.5120075	-4.288296491	0.56713532	-7.56132853	3.99E-14	1.58E-12
ENSDARG00000034268	slit3	6727.50264	0.706433693	0.09342575	7.56144495	3.99E-14	1.58E-12
ENSDARG00000117818	CR926130.2	336.799889	2.097526852	0.27745671	7.55983461	4.04E-14	1.59E-12
ENSDARG00000102058	si:dkeyp-11e3.1	438.887297	1.124062525	0.1486904	7.55975181	4.04E-14	1.59E-12
ENSDARG00000042130	zp3a.2	37.1860914	3.53102403	0.4673166	7.5559568	4.16E-14	1.64E-12
ENSDARG00000030694	atp6v1e1b	4222.31236	-0.872056331	0.11554	-7.54765716	4.43E-14	1.74E-12
ENSDARG00000054748	cuedc1b	800.434109	-0.951855669	0.12612043	-7.54719632	4.45E-14	1.75E-12
ENSDARG00000062788	irg1l	1486.67547	1.364816932	0.18126147	7.52954781	5.09E-14	1.99E-12
ENSDARG00000055618	acta1b	75140.5502	1.144979659	0.15205918	7.52982904	5.08E-14	1.99E-12
ENSDARG00000056239	tmem45b	1179.00992	1.072506393	0.14245706	7.52862941	5.13E-14	2.00E-12
ENSDARG00000008165	caspa	324.943984	1.164822953	0.15473831	7.52769582	5.16E-14	2.02E-12

Table S2. DEGs of WT vs. *terfa-/-*

ENSDARG00000115557	cox7b	5354.81665	0.903916183	0.12010819	7.52584978	5.24E-14	2.04E-12
ENSDARG00000019236	gsr	1106.45582	1.002266517	0.13326232	7.52100435	5.44E-14	2.11E-12
ENSDARG00000058102	sardh	3927.14612	0.979662758	0.13027813	7.51977919	5.49E-14	2.13E-12
ENSDARG00000027529	hmox1a	860.866384	1.580178086	0.21039772	7.51043338	5.89E-14	2.29E-12
ENSDARG00000094041	krt17	17346.7288	0.971914335	0.12948362	7.50607916	6.09E-14	2.36E-12
ENSDARG00000060927	si:ch211-136a13.1	851.146327	1.540219817	0.20521321	7.50546136	6.12E-14	2.37E-12
ENSDARG00000021889	gja3	114.416437	-1.833560827	0.24433223	-7.50437564	6.17E-14	2.38E-12
ENSDARG00000100698	eva1bb	392.32256	1.333368439	0.17769592	7.50365266	6.21E-14	2.39E-12
ENSDARG00000020114	slc20a1a	1348.2909	1.355985261	0.18076513	7.50136524	6.32E-14	2.42E-12
ENSDARG00000062688	gpnmb	3639.71643	-0.868029616	0.11570639	-7.50200215	6.29E-14	2.42E-12
ENSDARG00000006019	tktb	11874.3095	0.593589039	0.07912817	7.5016151	6.30E-14	2.42E-12
ENSDARG00000019125	klhl40b	412.225073	1.1297724	0.15063219	7.50020581	6.37E-14	2.44E-12
ENSDARG00000008127	pcdh15b	708.916813	-1.529628352	0.2041661	-7.49207822	6.78E-14	2.59E-12
ENSDARG00000001968	dock5	569.499623	1.324783642	0.17690685	7.48859427	6.96E-14	2.66E-12
ENSDARG00000013522	pck1	9088.84388	1.527654922	0.20426355	7.47884249	7.50E-14	2.86E-12
ENSDARG00000057736	ano10a	1402.49353	0.787373705	0.105335	7.4749484	7.72E-14	2.94E-12
ENSDARG00000076043	si:dkeyp-73d8.9	82.4757727	5.591295454	0.74806174	7.47437701	7.76E-14	2.95E-12
ENSDARG00000099447	pcdh1g18	124.008097	-2.241991272	0.2999679	-7.474104	7.77E-14	2.95E-12
ENSDARG00000076144	icam3	464.213715	1.587793929	0.21248741	7.47241429	7.87E-14	2.98E-12
ENSDARG00000014465	arhgef25b	561.548551	1.330309268	0.17807255	7.47060251	7.98E-14	3.02E-12
ENSDARG00000039626	nrgna	533.747938	-1.570672947	0.21026433	-7.46999248	8.02E-14	3.03E-12
ENSDARG00000010962	fkbp7	951.291289	1.010517877	0.13529044	7.46924825	8.07E-14	3.04E-12
ENSDARG00000100260	them6	434.146865	1.175612345	0.15757407	7.46069696	8.61E-14	3.24E-12
ENSDARG00000103754	aspm	2928.9429	-1.566535301	0.20997377	-7.46062379	8.61E-14	3.24E-12
ENSDARG00000053559	tspan3b	727.334419	-1.098701565	0.14728644	-7.45962464	8.68E-14	3.26E-12
ENSDARG00000016160	gmpr	3018.2042	0.975843916	0.13082943	7.45890194	8.72E-14	3.27E-12
ENSDARG00000029596	trim55a	637.539616	0.889655024	0.11931375	7.45643368	8.89E-14	3.33E-12
ENSDARG00000091471	ccdc28a	84.5467279	2.025724613	0.27177722	7.45362182	9.08E-14	3.39E-12
ENSDARG00000079802	aanat2	125.701641	-1.824257176	0.24483214	-7.45105277	9.26E-14	3.46E-12
ENSDARG00000078069	rrm2	1756.77368	-1.605812453	0.21552055	-7.45085533	9.27E-14	3.46E-12
ENSDARG00000026840	arhgap23b	213.50905	-2.365735823	0.31754796	-7.4500111	9.33E-14	3.47E-12
ENSDARG00000042535	actc1a	15655.9954	0.966075388	0.12969004	7.44911012	9.40E-14	3.49E-12
ENSDARG00000104687	slc16a9b	1264.8492	0.844140398	0.11336153	7.44644522	9.59E-14	3.56E-12
ENSDARG00000078671	cdk5r2b	452.44812	-1.356724824	0.18247624	-7.43507666	1.05E-13	3.87E-12
ENSDARG00000053953	myadml2	119.074844	1.940484186	0.26118069	7.42966167	1.09E-13	4.03E-12
ENSDARG00000055565	cacnb2b	334.401108	-1.302353543	0.1753116	-7.42879265	1.10E-13	4.05E-12
ENSDARG00000032405	ckap4	6804.75	0.820332726	0.11045635	7.42675932	1.11E-13	4.10E-12
ENSDARG00000062326	hmgn7	9913.0004	-0.856761047	0.11540651	-7.42385404	1.14E-13	4.19E-12
ENSDARG00000038667	fggy	727.571383	1.354462212	0.18250888	7.42134949	1.16E-13	4.26E-12
ENSDARG00000093584	zgc:193505	2315.80489	1.206627165	0.16266644	7.41780027	1.19E-13	4.37E-12
ENSDARG00000103038	pik3r3a	1724.63517	0.841593842	0.11346265	7.41736486	1.19E-13	4.38E-12
ENSDARG00000086826	sult6b1	1486.20467	0.983058109	0.13260321	7.41353206	1.23E-13	4.50E-12
ENSDARG00000004034	arhgdig	3692.24263	0.706692282	0.09541358	7.40662175	1.30E-13	4.74E-12
ENSDARG00000100876	sez6a	350.224059	-1.342410488	0.18126645	-7.40573073	1.30E-13	4.76E-12
ENSDARG00000104949	slc27a1b	995.20486	1.154081644	0.15585861	7.40467043	1.31E-13	4.79E-12
ENSDARG00000027600	pdlim5b	1840.56202	0.723610005	0.0977977	7.39904953	1.37E-13	4.99E-12
ENSDARG00000071304	tbc1d2	262.180336	1.836156337	0.24817193	7.39872701	1.37E-13	5.00E-12
ENSDARG00000090969	cbln18	149.545759	-2.458089163	0.33241417	-7.39465812	1.42E-13	5.14E-12
ENSDARG00000101225	cngb3.1	84.6742184	-2.386607744	0.32276658	-7.39422204	1.42E-13	5.15E-12
ENSDARG00000105445	CR769769.2	669.551778	-0.916935363	0.12405949	-7.39109428	1.46E-13	5.27E-12
ENSDARG00000030326	efna1a	1129.39265	-0.783536332	0.10615127	-7.38131824	1.57E-13	5.66E-12
ENSDARG00000103950	pcdh1gc6	490.73963	-1.71886641	0.2330446	-7.37569718	1.63E-13	5.90E-12
ENSDARG00000054191	pgk1	14947.526	0.815259556	0.11055945	7.37394765	1.66E-13	5.97E-12
ENSDARG00000069737	pou4f2	1701.02329	-1.582890546	0.21467204	-7.37352926	1.66E-13	5.98E-12
ENSDARG00000057074	rpgrb	534.580401	-1.124914591	0.15262363	-7.37051406	1.70E-13	6.10E-12
ENSDARG00000074119	frmpd2	229.725348	-2.649744453	0.35970304	-7.36647781	1.75E-13	6.28E-12
ENSDARG00000061173	st14a	3597.34399	0.722108796	0.09804051	7.36541268	1.77E-13	6.32E-12
ENSDARG00000016651	znf106a	5657.1627	0.847477513	0.11508353	7.36402109	1.78E-13	6.38E-12

Table S2. DEGs of WT vs. *terfa-/-*

ENSDARG00000018569	tnfrsf1a	794.808262	1.581245324	0.21474352	7.36341353	1.79E-13	6.40E-12
ENSDARG00000014233	sept8b	689.883531	-2.830299507	0.38439174	-7.36306021	1.80E-13	6.40E-12
ENSDARG00000015567	zic1	2049.08033	-0.850583348	0.1155181	-7.36320425	1.80E-13	6.40E-12
ENSDARG00000094508	CR925709.2	36.4246391	9.326934625	1.26690085	7.36200832	1.81E-13	6.44E-12
ENSDARG00000061836	nfixb	611.786946	-1.571666594	0.21349841	-7.36149079	1.82E-13	6.46E-12
ENSDARG00000059960	plch2a	2048.01039	-0.986228854	0.13402339	-7.35863245	1.86E-13	6.59E-12
ENSDARG00000109310	im:7150988	1259.26426	1.189834085	0.16177289	7.35496577	1.91E-13	6.76E-12
ENSDARG00000025703	dmgdh	5263.7424	0.699110708	0.09506881	7.35373353	1.93E-13	6.81E-12
ENSDARG00000102690	rims2b	1335.76349	-1.229184706	0.16715881	-7.35339481	1.93E-13	6.82E-12
ENSDARG00000034503	per2	4653.53771	1.024271118	0.13936004	7.34981925	1.98E-13	7.00E-12
ENSDARG00000058140	ttc25	113.929011	1.726694584	0.23496046	7.34887301	2.00E-13	7.04E-12
ENSDARG00000093494	si:ch211-217k17.9	1380.87321	0.930094274	0.12660656	7.34633582	2.04E-13	7.16E-12
ENSDARG00000105551	si:cabz01101003.1	235.566332	1.422116836	0.19363959	7.34414286	2.07E-13	7.27E-12
ENSDARG00000077236	hspb6	768.460311	1.748540307	0.23821282	7.34024452	2.13E-13	7.46E-12
ENSDARG00000073821	znf1177	49.5392418	8.95479775	1.21992969	7.34042117	2.13E-13	7.46E-12
ENSDARG00000113674	CABZ01058222.1	266.292255	1.431684608	0.19518226	7.33511664	2.22E-13	7.74E-12
ENSDARG00000075833	lyve1a	250.944316	1.622965006	0.22129855	7.33382587	2.24E-13	7.81E-12
ENSDARG00000101199	rbp4	35157.2354	-0.792174856	0.1080784	-7.32963146	2.31E-13	8.05E-12
ENSDARG00000025891	shox	977.240416	-1.130884528	0.15430644	-7.32882286	2.32E-13	8.08E-12
ENSDARG00000045522	acot15	486.995106	1.658481024	0.2263035	7.32856979	2.33E-13	8.09E-12
ENSDARG00000103917	znf185	2652.85703	0.811865271	0.11090446	7.32040229	2.47E-13	8.58E-12
ENSDARG00000052330	slc4a2b	1157.94975	0.810546074	0.11074667	7.31892049	2.50E-13	8.67E-12
ENSDARG00000087832	bcl3	113.954743	2.564083975	0.35040753	7.31743411	2.53E-13	8.75E-12
ENSDARG00000104228	lypla1	129.26259	1.734558421	0.23709854	7.31577029	2.56E-13	8.85E-12
ENSDARG00000055118	mylipb	637.538535	1.219600006	0.16671483	7.31548617	2.56E-13	8.85E-12
ENSDARG00000030479	hmgb1b	7950.5417	-1.310312111	0.17920966	-7.31161542	2.64E-13	9.10E-12
ENSDARG00000056134	c1qtnf5	357.02182	1.187441493	0.16252629	7.30615047	2.75E-13	9.46E-12
ENSDARG00000099719	cdkn1d	1986.87682	-0.783479443	0.10727035	-7.30378381	2.80E-13	9.62E-12
ENSDARG00000111899	zgc:171435	90.6473344	1.979863465	0.27117691	7.30100311	2.86E-13	9.81E-12
ENSDARG00000002403	nusap1	1297.59736	-1.527204238	0.20922079	-7.29948611	2.89E-13	9.90E-12
ENSDARG00000020028	cps1	177.376676	-2.276897261	0.31206384	-7.2962547	2.96E-13	1.01E-11
ENSDARG00000035329	capns1a	3759.6285	0.814938412	0.11171679	7.29468141	2.99E-13	1.02E-11
ENSDARG00000092358	BX469930.1	275.224269	-6.540677385	0.89668352	-7.29429866	3.00E-13	1.02E-11
ENSDARG00000014246	jag2a	460.525601	1.323669358	0.18146657	7.29428781	3.00E-13	1.02E-11
ENSDARG00000091801	serpinb14	1567.74835	1.246281478	0.17091464	7.29183567	3.06E-13	1.04E-11
ENSDARG00000019845	pdlim1	1286.52694	0.914142027	0.12541442	7.28897069	3.12E-13	1.06E-11
ENSDARG00000114704	KCTD20	300.508013	-1.9516935	0.26813256	-7.27883818	3.37E-13	1.14E-11
ENSDARG00000078551	zgc:171242	71.336378	9.248854495	1.27075997	7.2782073	3.38E-13	1.15E-11
ENSDARG00000075676	fndc7b	268.18232	-1.727302591	0.23732564	-7.27819617	3.38E-13	1.15E-11
ENSDARG00000103837	CU633479.2	175.634662	1.990163476	0.27357923	7.27454147	3.48E-13	1.18E-11
ENSDARG00000104956	si:dkey-165o8.2	200.363948	-2.260226864	0.31116948	-7.26365209	3.77E-13	1.27E-11
ENSDARG00000090120	si:dkey-248g21.1	106.174219	1.922793533	0.26468598	7.26443293	3.75E-13	1.27E-11
ENSDARG00000102888	gpr39	474.547013	1.902715748	0.26195121	7.26362664	3.77E-13	1.27E-11
ENSDARG00000057652	dbpb	816.920757	1.363976688	0.18778959	7.26332447	3.78E-13	1.27E-11
ENSDARG00000109371	pdgfaa	119.706142	1.821927075	0.25126769	7.25094045	4.14E-13	1.39E-11
ENSDARG00000099154	CABZ01032488.1	735.366804	1.467326127	0.20237584	7.2505004	4.15E-13	1.39E-11
ENSDARG00000062030	gpr17	181.469371	-1.446113286	0.1995125	-7.24823416	4.22E-13	1.41E-11
ENSDARG00000051874	stra6	1446.77484	0.679529943	0.09375385	7.24802186	4.23E-13	1.41E-11
ENSDARG00000003754	tspan2a	1115.63541	-0.983226515	0.13576633	-7.24205001	4.42E-13	1.48E-11
ENSDARG00000007715	lgsn	611.099407	-1.609566151	0.22241667	-7.23671535	4.60E-13	1.53E-11
ENSDARG00000029069	tnni2a.4	79931.8183	0.791727587	0.10941818	7.23579556	4.63E-13	1.54E-11
ENSDARG00000033537	p4ha1a	1384.4181	0.864964032	0.11955067	7.23512506	4.65E-13	1.55E-11
ENSDARG00000094730	acbd7	803.058452	-1.07195575	0.14818903	-7.23370521	4.70E-13	1.56E-11
ENSDARG00000035732	arntl1b	883.725458	-0.935949203	0.12942031	-7.2318572	4.76E-13	1.58E-11
ENSDARG00000091996	tcnbb	6609.71424	1.069001899	0.14787843	7.22892387	4.87E-13	1.61E-11
ENSDARG00000100739	CABZ01052573.1	1260.0859	0.948276851	0.13121067	7.22713214	4.93E-13	1.63E-11
ENSDARG00000037588	bhlhe23	457.320403	-1.882612975	0.26056774	-7.22504247	5.01E-13	1.66E-11
ENSDARG00000102744	mgst3a	1402.30875	1.039314725	0.14387719	7.22362391	5.06E-13	1.67E-11

Table S2. DEGs of WT vs. *terfa-/-*

ENSDARG00000099985	ccn1l2	407.322031	1.045352896	0.14472647	7.22295592	5.09E-13	1.68E-11
ENSDARG00000102599	si:ch1073-392o20.	1199.66441	1.277649571	0.17693503	7.22100986	5.16E-13	1.70E-11
ENSDARG00000028336	dhdhl	1487.01584	0.810379753	0.11230445	7.21591868	5.36E-13	1.76E-11
ENSDARG00000101084	CABZ01069184.1	66.4946753	2.815084972	0.39011641	7.21601263	5.35E-13	1.76E-11
ENSDARG00000097929	BX005392.3	132.79578	6.455747552	0.89489103	7.21400413	5.43E-13	1.78E-11
ENSDARG00000044125	txn	1187.28502	1.544919328	0.21434572	7.20760508	5.69E-13	1.86E-11
ENSDARG00000002745	tdh	6350.43436	0.951684275	0.13203954	7.20757034	5.70E-13	1.86E-11
ENSDARG00000037859	il11a	30.8537631	5.613507662	0.77890568	7.20691579	5.72E-13	1.87E-11
ENSDARG00000045737	guca1g	154.599875	-2.796407031	0.38806155	-7.20609149	5.76E-13	1.88E-11
ENSDARG00000059362	cavin1b	2682.42814	0.756735009	0.10505189	7.20344009	5.87E-13	1.91E-11
ENSDARG00000091800	zgc:174260	69.1652748	-3.250771449	0.45166895	-7.19724363	6.14E-13	2.00E-11
ENSDARG00000102456	cfhl4	4371.4259	1.130457159	0.15712256	7.19474761	6.26E-13	2.03E-11
ENSDARG00000078440	ccdc88aa	850.662758	-1.643946349	0.22857051	-7.19229429	6.37E-13	2.07E-11
ENSDARG00000090268	krtt1c19e	156724.295	0.840329262	0.11691851	7.18730752	6.61E-13	2.14E-11
ENSDARG00000055784	ptpn3	1299.20844	0.953147463	0.13269526	7.18298061	6.82E-13	2.21E-11
ENSDARG00000038363	drd4a	182.048559	-2.560410501	0.35659091	-7.18024612	6.96E-13	2.25E-11
ENSDARG00000102249	pepd	1540.06177	0.897673943	0.12504858	7.17860142	7.04E-13	2.27E-11
ENSDARG00000056690	mtmr1a	1319.94141	-1.037081501	0.14448206	-7.17792573	7.08E-13	2.28E-11
ENSDARG00000062315	sik2b	1858.49813	0.823437799	0.11473026	7.17716349	7.12E-13	2.29E-11
ENSDARG00000011841	atp5l	8009.11283	0.832038827	0.11594606	7.17608513	7.17E-13	2.31E-11
ENSDARG00000070951	hmga1b	2583.22678	-0.97763822	0.13627889	-7.17380556	7.29E-13	2.34E-11
ENSDARG00000060711	sv2bb	2500.11201	-0.79187159	0.11043656	-7.17037532	7.48E-13	2.40E-11
ENSDARG00000057427	sv2ba	2555.48221	-2.555873997	0.35651506	-7.16904927	7.55E-13	2.42E-11
ENSDARG00000035018	thy1	1054.90264	1.547427816	0.21586553	7.16848053	7.58E-13	2.42E-11
ENSDARG00000020504	h3f3b.1	9453.71944	-1.025696406	0.14306948	-7.16921867	7.54E-13	2.42E-11
ENSDARG00000026090	adprm	264.999053	1.271184338	0.17732765	7.16856268	7.58E-13	2.42E-11
ENSDARG00000034504	lmo1	1319.08902	-1.09581741	0.15301548	-7.16148084	7.98E-13	2.54E-11
ENSDARG00000035508	barhl1a	362.436505	-1.710512055	0.2388802	-7.16054361	8.04E-13	2.56E-11
ENSDARG00000076830	si:dkey-65b12.6	5373.91397	0.978009259	0.13663638	7.1577513	8.20E-13	2.61E-11
ENSDARG00000020158	spag6	112.223781	1.710434402	0.23898416	7.15710357	8.24E-13	2.62E-11
ENSDARG00000007697	fabp7a	18906.5611	-0.833761829	0.11655662	-7.15327727	8.47E-13	2.68E-11
ENSDARG00000103220	slc10a3	1548.27805	0.745165271	0.10417258	7.15318032	8.48E-13	2.68E-11
ENSDARG00000100088	zmp:0000001114	2383.42388	0.870869671	0.12180365	7.14978323	8.69E-13	2.75E-11
ENSDARG00000061603	sorbs2b	2198.47632	-0.70768006	0.09898887	-7.14908714	8.74E-13	2.76E-11
ENSDARG00000054562	her15.1	496.858938	-1.572132131	0.22033727	-7.13511656	9.67E-13	3.05E-11
ENSDARG00000053666	myb	937.720697	-0.836643736	0.11742966	-7.1246374	1.04E-12	3.29E-11
ENSDARG00000100293	arhgef15	55.3375623	-3.334073209	0.46806156	-7.12315114	1.05E-12	3.32E-11
ENSDARG00000078917	zgc:195245	65.6623478	-4.007992247	0.56277593	-7.12182597	1.07E-12	3.35E-11
ENSDARG00000002748	sema6d	1130.94831	-1.224527321	0.17200863	-7.11898778	1.09E-12	3.41E-11
ENSDARG00000099959	smyhc1	48501.8549	0.919789852	0.12920753	7.11870175	1.09E-12	3.41E-11
ENSDARG00000079271	dcxr	792.106225	1.075606568	0.1511261	7.11727852	1.10E-12	3.45E-11
ENSDARG00000099298	xrcc5	1844.3099	0.99159746	0.13944661	7.11094717	1.15E-12	3.60E-11
ENSDARG00000039827	bbs5	273.881408	-1.319971754	0.18564154	-7.11032548	1.16E-12	3.61E-11
ENSDARG00000009386	mpp4l	205.722108	-2.13780457	0.30077906	-7.1075579	1.18E-12	3.68E-11
ENSDARG00000029898	cnga1a	146.419799	-2.144306776	0.30180654	-7.10490484	1.20E-12	3.75E-11
ENSDARG00000011998	bfsp2	1864.1989	-1.443180009	0.20317595	-7.10310457	1.22E-12	3.79E-11
ENSDARG00000031048	gspt1	1530.48549	0.651733449	0.09175198	7.10320851	1.22E-12	3.79E-11
ENSDARG00000078797	dennd3a	1139.66008	0.815947558	0.11494472	7.09860875	1.26E-12	3.91E-11
ENSDARG00000103277	cyp24a1	746.495431	3.105154582	0.43760808	7.09574334	1.29E-12	3.99E-11
ENSDARG00000057408	CABZ01067232.1	351.960518	-1.781168796	0.25114145	-7.0922933	1.32E-12	4.08E-11
ENSDARG00000117755	CU467655.2	100.615359	-3.355348981	0.47373379	-7.08277322	1.41E-12	4.37E-11
ENSDARG00000104874	fzd6	1671.97835	0.820440813	0.1158445	7.08225947	1.42E-12	4.38E-11
ENSDARG00000055045	casp3b	556.141174	0.91813839	0.12969428	7.07925143	1.45E-12	4.47E-11
ENSDARG00000070412	mtmr7b	1066.11466	-0.816729775	0.11546061	-7.073666	1.51E-12	4.65E-11
ENSDARG00000067958	sh3gl1a	867.306853	1.024840649	0.14491848	7.07184222	1.53E-12	4.70E-11
ENSDARG00000031307	zic4	542.703917	-0.924529982	0.13073206	-7.0719456	1.53E-12	4.70E-11
ENSDARG00000040565	ckmb	238680.343	0.901279123	0.12746571	7.07075734	1.54E-12	4.73E-11
ENSDARG00000004256	wnt11f2	200.099848	1.315387483	0.18624449	7.06269219	1.63E-12	5.00E-11

Table S2. DEGs of WT vs. *terfa-/-*

ENSDARG00000014024	ms4a17a.4	33.9204892	3.906656729	0.55320178	7.06190193	1.64E-12	5.03E-11
ENSDARG00000088882	si:ch211-149b19.2	103.013791	-2.10049599	0.29746323	-7.06136345	1.65E-12	5.04E-11
ENSDARG00000068874	PTCHD3	32.5880274	3.512646369	0.49753902	7.06004196	1.66E-12	5.08E-11
ENSDARG00000103379	pax6a	2591.90138	-1.334112705	0.18898324	-7.05942324	1.67E-12	5.10E-11
ENSDARG00000104396	trim25	423.994671	1.287445614	0.18261467	7.05006682	1.79E-12	5.45E-11
ENSDARG00000103830	ddr2l	1337.60123	1.211674914	0.17204537	7.04276402	1.88E-12	5.73E-11
ENSDARG00000024295	slc11a2	1535.58625	0.888440607	0.12616989	7.0416216	1.90E-12	5.77E-11
ENSDARG00000075067	aipl1	135.385557	-2.041486001	0.28996561	-7.04044171	1.92E-12	5.82E-11
ENSDARG00000111326	BX323820.1	152.957152	-2.2840848	0.32445216	-7.03981991	1.92E-12	5.83E-11
ENSDARG00000101406	rplp2	11197.4535	0.752510695	0.10692848	7.03751398	1.96E-12	5.92E-11
ENSDARG00000063475	abcg1	211.516948	-1.37866387	0.19590029	-7.03757942	1.96E-12	5.92E-11
ENSDARG00000090552	si:dkey-7j14.6	1629.76716	1.256853081	0.17861265	7.03675284	1.97E-12	5.94E-11
ENSDARG00000070272	casp10	149.76181	1.437332594	0.20428653	7.03586576	1.98E-12	5.97E-11
ENSDARG00000056723	fhad1	92.1671151	2.445608443	0.34773713	7.03292286	2.02E-12	6.09E-11
ENSDARG00000002172	aplnra	926.575194	-0.920436881	0.13106452	-7.02277702	2.18E-12	6.54E-11
ENSDARG00000101992	si:dkey-238k10.3	50.672991	-2.759913556	0.3931591	-7.01983895	2.22E-12	6.65E-11
ENSDARG00000037943	cpt1cb	519.122626	1.113993596	0.15868781	7.02003275	2.22E-12	6.65E-11
ENSDARG00000035798	gngt1	1069.46364	-3.468806249	0.49414563	-7.01980557	2.22E-12	6.65E-11
ENSDARG00000011049	slc17a9b	417.35369	0.927528827	0.13212905	7.01987055	2.22E-12	6.65E-11
ENSDARG00000045979	zgc:153704	1142.01536	-1.03256023	0.14714847	-7.01713196	2.26E-12	6.77E-11
ENSDARG00000035028	lpcat4	385.05465	-1.041714837	0.1484671	-7.01646911	2.28E-12	6.80E-11
ENSDARG00000045139	ca7	107.452121	-1.800663878	0.25668284	-7.01513149	2.30E-12	6.85E-11
ENSDARG00000093738	pth1a	23.2636441	6.310242101	0.90071033	7.0058507	2.45E-12	7.32E-11
ENSDARG00000077249	gmip	256.967086	-1.307015118	0.18657117	-7.0054507	2.46E-12	7.33E-11
ENSDARG00000105218	ompa	251.153765	-2.179524468	0.31114053	-7.00495198	2.47E-12	7.35E-11
ENSDARG00000038788	dnai1.2	115.466093	1.514230598	0.21627056	7.00155668	2.53E-12	7.52E-11
ENSDARG00000088330	hbae1.3	473.468838	1.715520154	0.24507664	6.99993329	2.56E-12	7.60E-11
ENSDARG00000061191	plekho1a	306.585507	-1.357088935	0.19393438	-6.99767067	2.60E-12	7.71E-11
ENSDARG00000027236	rs1a	3687.82586	-2.445120646	0.34949853	-6.99608284	2.63E-12	7.79E-11
ENSDARG00000013430	bhmt	128328.14	0.78328143	0.11200092	6.99352694	2.68E-12	7.92E-11
ENSDARG00000016480	slc17a7a	532.778611	-1.880641483	0.26903171	-6.99040827	2.74E-12	8.09E-11
ENSDARG00000109749	si:ch211-162i8.7	53.1073926	3.178783713	0.45501075	6.98617281	2.82E-12	8.33E-11
ENSDARG00000099871	myo7aa	804.837865	-0.864423249	0.12373982	-6.98581328	2.83E-12	8.34E-11
ENSDARG00000070961	lepr	1196.7546	0.830775668	0.11894977	6.98425609	2.86E-12	8.42E-11
ENSDARG00000058082	birc7	215.455886	-1.719640974	0.2462183	-6.98421271	2.86E-12	8.42E-11
ENSDARG00000086458	hdac10	400.888867	-1.202373658	0.17234365	-6.97660558	3.02E-12	8.87E-11
ENSDARG00000006526	fn1b	40422.0999	2.632535775	0.37745009	6.97452676	3.07E-12	9.00E-11
ENSDARG00000073905	vwa5a	1738.51583	0.909508883	0.13042194	6.97358835	3.09E-12	9.04E-11
ENSDARG00000005993	prc1b	468.981024	-1.067556083	0.15326062	-6.96562565	3.27E-12	9.55E-11
ENSDARG00000018621	slc6a19a.1	284.48577	1.87292398	0.26887866	6.96568489	3.27E-12	9.55E-11
ENSDARG00000116625	znf1141	176.764839	2.090108778	0.30011677	6.96431849	3.30E-12	9.63E-11
ENSDARG00000076317	plod3	4433.24785	0.709954324	0.10194952	6.96378287	3.31E-12	9.65E-11
ENSDARG00000117450	FO834828.2	488.258713	1.663463249	0.2388902	6.96329642	3.32E-12	9.67E-11
ENSDARG00000035578	hs3st1l2	270.055632	-1.245738467	0.17898652	-6.9599568	3.40E-12	9.89E-11
ENSDARG00000068992	hspa8	159328.045	0.586192156	0.08427791	6.9554662	3.51E-12	1.02E-10
ENSDARG00000096946	BX897682.1	32.5447335	6.042336616	0.86896178	6.95351253	3.56E-12	1.03E-10
ENSDARG00000039613	poll	490.985284	-0.901651618	0.12965532	-6.95421981	3.55E-12	1.03E-10
ENSDARG00000069295	bcl6ab	673.747919	1.464225044	0.21094902	6.9411324	3.89E-12	1.13E-10
ENSDARG00000075161	defbl1	1542.15996	-1.181791037	0.17040753	-6.93508715	4.06E-12	1.17E-10
ENSDARG00000002295	si:dkey-21p1.3	187.73115	1.909689905	0.27544754	6.93304409	4.12E-12	1.19E-10
ENSDARG00000032820	rxfp2a	130.434787	-2.395383886	0.34559198	-6.93124841	4.17E-12	1.20E-10
ENSDARG00000117260	CABZ01079870.1	36.3274473	4.552970639	0.65714377	6.92842396	4.26E-12	1.23E-10
ENSDARG00000017634	pdcb	2221.88794	-3.257850427	0.47033488	-6.92666137	4.31E-12	1.24E-10
ENSDARG00000077799	egr4	375.812785	-1.558012577	0.22492714	-6.92674352	4.31E-12	1.24E-10
ENSDARG00000103006	si:ch1073-303d10.	95.0994278	-3.142095597	0.45444517	-6.91413581	4.71E-12	1.35E-10
ENSDARG00000035598	coro1ca	4242.42264	0.662544918	0.09587528	6.91048782	4.83E-12	1.38E-10
ENSDARG00000031751	npr1a	594.123566	1.140317516	0.16504208	6.90925312	4.87E-12	1.40E-10
ENSDARG00000113016	CU207245.1	40.2511722	5.328921549	0.7717567	6.90492422	5.02E-12	1.44E-10

Table S2. DEGs of WT vs. *terfa-/-*

ENSDARG00000100222	utrn	6250.03229	0.71093227	0.10297667	6.90381883	5.06E-12	1.45E-10
ENSDARG00000053875	cryba1b	8475.43081	-2.211570883	0.32067981	-6.89650805	5.33E-12	1.52E-10
ENSDARG00000057790	ankrd6a	230.996464	-1.661689719	0.24102943	-6.89413617	5.42E-12	1.54E-10
ENSDARG00000006640	eomesa	758.943587	-1.181270441	0.17138906	-6.89233274	5.49E-12	1.56E-10
ENSDARG00000010680	gngt2a	1274.88937	-4.629277103	0.67208605	-6.88792319	5.66E-12	1.61E-10
ENSDARG00000104524	CR391991.4	61.3341709	5.021031629	0.72900229	6.88753887	5.68E-12	1.61E-10
ENSDARG00000017128	myofl	1176.69702	1.116309108	0.16207976	6.88740616	5.68E-12	1.61E-10
ENSDARG00000060797	pfkmb	2339.76096	0.893057619	0.12967952	6.88665141	5.71E-12	1.62E-10
ENSDARG00000060106	crb2a	446.08044	0.952323444	0.13828447	6.88669839	5.71E-12	1.62E-10
ENSDARG00000068275	ptx3a	2163.88116	0.854943574	0.1241654	6.88552173	5.76E-12	1.63E-10
ENSDARG00000117109	CR626884.6	307.591558	2.061743633	0.29962943	6.88097849	5.94E-12	1.68E-10
ENSDARG00000104811	cyp2aa2	148.178407	1.480910262	0.21526623	6.87943606	6.01E-12	1.70E-10
ENSDARG00000112527	FP325130.1	680.013262	1.039157671	0.15108261	6.87807601	6.07E-12	1.71E-10
ENSDARG00000058543	lama5	10410.9085	0.864176153	0.1256991	6.87495877	6.20E-12	1.75E-10
ENSDARG00000063509	lrrc58b	1410.93588	0.922993511	0.13428872	6.87320203	6.28E-12	1.77E-10
ENSDARG00000037932	lin7b	1593.66198	-0.83908176	0.12211022	-6.87151112	6.35E-12	1.78E-10
ENSDARG00000022730	aasdh	266.256432	1.531768037	0.22306933	6.86678001	6.57E-12	1.84E-10
ENSDARG00000098475	arr3b	3599.78176	-3.417684889	0.49781328	-6.86539522	6.63E-12	1.86E-10
ENSDARG00000016793	crybb1l2	17908.1113	-1.464822273	0.21339434	-6.86439151	6.68E-12	1.87E-10
ENSDARG00000104329	si:ch73-281k2.5	282.03608	1.767649087	0.25751967	6.86413239	6.69E-12	1.87E-10
ENSDARG00000062592	myl10	9541.1251	0.804390498	0.11731345	6.85676289	7.04E-12	1.97E-10
ENSDARG00000098481	anxa1d	410.717605	1.280988543	0.18706432	6.84785074	7.50E-12	2.09E-10
ENSDARG00000012513	sdcbp2	5339.47465	0.654400782	0.09557819	6.8467585	7.55E-12	2.10E-10
ENSDARG00000039577	ptk2bb	1546.42086	0.849917956	0.12417698	6.84440837	7.68E-12	2.14E-10
ENSDARG00000076371	SHROOM2	453.823652	-1.269339448	0.18553132	-6.84164527	7.83E-12	2.18E-10
ENSDARG00000041621	bbs2	904.208125	-1.079708729	0.15794435	-6.83600724	8.14E-12	2.26E-10
ENSDARG00000009830	hdlbpa	52815.5631	0.514371977	0.07527019	6.83367435	8.28E-12	2.30E-10
ENSDARG00000104230	sec23a	2916.75762	0.651516788	0.0953565	6.83243204	8.35E-12	2.31E-10
ENSDARG00000071394	foxq2	79.9097854	-2.986705691	0.43748397	-6.82700605	8.67E-12	2.40E-10
ENSDARG00000031136	moxd1	361.060384	1.876524745	0.27523289	6.8179524	9.23E-12	2.55E-10
ENSDARG00000067984	gas1b	653.001637	-0.987980342	0.14493803	-6.81657072	9.32E-12	2.57E-10
ENSDARG00000043141	si:ch211-132f19.7	504.682973	-2.792629281	0.40998737	-6.81150078	9.66E-12	2.66E-10
ENSDARG00000104555	sh2d3cb	168.240529	-1.391229806	0.20441064	-6.80605394	1.00E-11	2.76E-10
ENSDARG00000043847	tmem244	152.127869	-2.125935838	0.31233937	-6.80649325	1.00E-11	2.76E-10
ENSDARG00000098639	pycr1b	3201.48769	0.81771956	0.12014859	6.8059022	1.00E-11	2.76E-10
ENSDARG00000053746	CABZ01084566.1	220.904772	-1.949340127	0.28683269	-6.79608769	1.07E-11	2.95E-10
ENSDARG00000074319	sall1a	2470.20829	-0.747580329	0.11004603	-6.79334244	1.10E-11	3.01E-10
ENSDARG00000077169	si:ch211-153b23.4	1003.69666	0.83996868	0.12369328	6.79073843	1.12E-11	3.06E-10
ENSDARG00000043581	gadd45aa	493.512866	0.957383694	0.14100086	6.78991381	1.12E-11	3.07E-10
ENSDARG00000062568	mrpl2	2995.54911	0.710436864	0.10470593	6.78506841	1.16E-11	3.17E-10
ENSDARG00000057141	zgc:110045	638.017173	-1.935847232	0.2854297	-6.7822208	1.18E-11	3.23E-10
ENSDARG00000071860	nrn1a	864.909228	-0.752564189	0.11096865	-6.78177273	1.19E-11	3.24E-10
ENSDARG00000094854	ms4a17a.9	864.266446	1.15179473	0.16988082	6.78001625	1.20E-11	3.27E-10
ENSDARG00000045580	lum	4305.40033	-0.643150545	0.09487793	-6.77871591	1.21E-11	3.30E-10
ENSDARG00000099306	zgc:175088	3457.27991	1.012368073	0.14934205	6.77885476	1.21E-11	3.30E-10
ENSDARG00000044415	tlr5a	70.4418834	1.924669224	0.28398381	6.77739067	1.22E-11	3.32E-10
ENSDARG00000101209	lamb1a	10220.6211	0.620879678	0.09162469	6.77633617	1.23E-11	3.34E-10
ENSDARG00000036344	calb2b	3581.40033	-1.455942062	0.21503079	-6.77085404	1.28E-11	3.47E-10
ENSDARG00000075621	birc5a	297.046395	-1.319567566	0.19491755	-6.76987564	1.29E-11	3.49E-10
ENSDARG00000031814	dhrs13b.1	247.366659	1.237892687	0.18302361	6.76356834	1.35E-11	3.64E-10
ENSDARG00000077013	znf280d	1369.54484	-0.734339592	0.10863638	-6.75961062	1.38E-11	3.73E-10
ENSDARG00000061231	tinagl1	540.084401	0.966888188	0.14303623	6.75974343	1.38E-11	3.73E-10
ENSDARG00000021959	zgc:101100	97.323945	-2.087849664	0.30889425	-6.75910814	1.39E-11	3.74E-10
ENSDARG00000058410	CR956623.1	95.5817782	-2.622268203	0.38800316	-6.75836812	1.40E-11	3.76E-10
ENSDARG00000091253	smyd1b	2755.67606	0.741733506	0.10978752	6.75608212	1.42E-11	3.81E-10
ENSDARG00000006235	brms1la	837.50516	-1.04998738	0.15543624	-6.7551002	1.43E-11	3.84E-10
ENSDARG00000096667	hemgn	566.71897	-0.933794698	0.13836946	-6.7485605	1.49E-11	4.01E-10
ENSDARG00000035131	surf4l	1039.72261	0.894562625	0.13272284	6.74008063	1.58E-11	4.24E-10

Table S2. DEGs of WT vs. *terfa-/-*

ENSDARG00000042041	tal2	471.785975	-1.351638958	0.20055214	-6.73958868	1.59E-11	4.25E-10
ENSDARG00000100476	si:dkeyp-110c7.4	188.374776	-1.914053219	0.28405919	-6.73821964	1.60E-11	4.29E-10
ENSDARG00000076225	tha1	402.066326	0.993209455	0.14747837	6.7346112	1.64E-11	4.39E-10
ENSDARG00000004771	ankrd10b	809.888405	-0.883362435	0.13121447	-6.7322027	1.67E-11	4.46E-10
ENSDARG00000056805	plch2b	222.289969	-2.102386885	0.31229584	-6.73203617	1.67E-11	4.46E-10
ENSDARG00000003077	gpx9	162.351961	-1.524235399	0.22644426	-6.73117255	1.68E-11	4.48E-10
ENSDARG00000027424	slc25a3a	1011.94849	-2.918611215	0.43369629	-6.72961991	1.70E-11	4.53E-10
ENSDARG00000039051	hhatla	8290.77673	0.818500859	0.12163289	6.72927254	1.71E-11	4.53E-10
ENSDARG00000010770	sox19a	1606.91274	-1.166424879	0.17335849	-6.72839786	1.72E-11	4.54E-10
ENSDARG00000089643	mcama	2343.0479	-1.015723997	0.15096382	-6.72826106	1.72E-11	4.54E-10
ENSDARG00000117011	ewsr1b	482.192122	-1.789340012	0.26591893	-6.72889306	1.71E-11	4.54E-10
ENSDARG00000032849	ndrg1a	4641.59429	-0.865911483	0.12868768	-6.72878315	1.71E-11	4.54E-10
ENSDARG00000103735	elovl1b	2100.28157	0.597885778	0.08887644	6.72715734	1.73E-11	4.57E-10
ENSDARG00000094300	nupr1a	1955.60035	1.04230382	0.1550017	6.72446712	1.76E-11	4.65E-10
ENSDARG00000040321	rx2	269.291277	-2.227715183	0.33137929	-6.72255395	1.79E-11	4.71E-10
ENSDARG00000093572	lamc3	3953.12512	0.690254397	0.1027445	6.71816412	1.84E-11	4.84E-10
ENSDARG00000070394	uroc1	1161.87518	1.142833731	0.17010219	6.71851276	1.84E-11	4.84E-10
ENSDARG00000052035	nxnl1	76.1654378	-2.627247883	0.3910618	-6.7182422	1.84E-11	4.84E-10
ENSDARG00000098330	spef2	210.848955	1.383414628	0.20594223	6.71748871	1.85E-11	4.86E-10
ENSDARG00000088440	ssh2a	1491.86431	0.690571977	0.10283424	6.71538936	1.88E-11	4.92E-10
ENSDARG00000100584	ccdc40	316.149585	1.4367348	0.21395954	6.71498364	1.88E-11	4.93E-10
ENSDARG00000025325	ccsapa	444.772815	-0.996403991	0.1484369	-6.71264363	1.91E-11	5.01E-10
ENSDARG00000090903	creb3l3b	205.43412	1.266479338	0.18868669	6.71207582	1.92E-11	5.02E-10
ENSDARG00000040123	zfpm2a	893.438098	-1.327737522	0.19792815	-6.70817936	1.97E-11	5.15E-10
ENSDARG00000099829	BX927336.2	36.22931	4.156394547	0.61995575	6.70434071	2.02E-11	5.28E-10
ENSDARG00000015829	mov10a	941.920707	0.750013319	0.11195134	6.69945846	2.09E-11	5.46E-10
ENSDARG00000053493	aldh1a2	2908.66369	0.61724377	0.09227824	6.68894144	2.25E-11	5.86E-10
ENSDARG00000105657	si:cabz01080528.1	395.084896	1.187551264	0.17755386	6.68840002	2.26E-11	5.87E-10
ENSDARG00000100729	tln1	9648.43062	0.7118548	0.1065659	6.67994897	2.39E-11	6.21E-10
ENSDARG00000089255	kcnj11l	434.232358	-1.057451148	0.15833403	-6.67860954	2.41E-11	6.26E-10
ENSDARG00000099321	btbd17a	566.360966	-1.363761814	0.2042356	-6.6773951	2.43E-11	6.31E-10
ENSDARG00000056640	rpia	1936.09637	0.67829265	0.10164713	6.67301308	2.51E-11	6.49E-10
ENSDARG00000041141	cryba1a	42.3900823	-3.283887137	0.49211453	-6.67301393	2.51E-11	6.49E-10
ENSDARG00000045131	id4	792.442297	-1.114632439	0.16713496	-6.66905617	2.57E-11	6.66E-10
ENSDARG00000057231	rorca	989.471216	-1.090480281	0.16354241	-6.66787477	2.60E-11	6.71E-10
ENSDARG00000031616	g6pca.1	362.979467	1.698606159	0.25487361	6.66450383	2.66E-11	6.85E-10
ENSDARG00000075733	zyx	1673.34872	0.678290662	0.10178001	6.66428161	2.66E-11	6.86E-10
ENSDARG00000035327	ckma	174629.159	0.940286769	0.14110909	6.66354482	2.67E-11	6.88E-10
ENSDARG00000059461	mepce	692.120967	-0.7134577	0.10707961	-6.66287154	2.69E-11	6.91E-10
ENSDARG00000008861	tfap2e	985.825067	-1.705436922	0.25599203	-6.66207037	2.70E-11	6.94E-10
ENSDARG00000042469	ndufa12	3643.30115	0.665917178	0.10002927	6.65722355	2.79E-11	7.17E-10
ENSDARG00000015524	prps1a	4268.36551	0.776597205	0.11669945	6.65467736	2.84E-11	7.28E-10
ENSDARG00000009637	rcvrn3	6963.07957	-3.067139665	0.46093149	-6.65422032	2.85E-11	7.30E-10
ENSDARG00000061120	slc43a2b	7477.90641	1.082356289	0.16266281	6.65398728	2.85E-11	7.30E-10
ENSDARG00000093392	CT583651.1	30.3945143	8.535059319	1.28346464	6.65001517	2.93E-11	7.49E-10
ENSDARG00000074085	si:ch1073-188e1.1	44.096039	3.342783046	0.50266841	6.65007582	2.93E-11	7.49E-10
ENSDARG00000069030	skor1a	530.70811	-1.228510539	0.18476255	-6.64913166	2.95E-11	7.52E-10
ENSDARG00000100986	CABZ01117503.1	93.2433853	-2.552624167	0.3843274	-6.64179595	3.10E-11	7.90E-10
ENSDARG00000099766	myl12.1	14225.1872	0.611703861	0.09213843	6.63896568	3.16E-11	8.04E-10
ENSDARG00000006607	gpr27	511.936896	-1.072938614	0.16162946	-6.63826166	3.17E-11	8.07E-10
ENSDARG00000086645	hs3st3b1b	1769.89054	-0.604205372	0.09102381	-6.63788308	3.18E-11	8.09E-10
ENSDARG00000059746	plod1a	2165.88934	0.959647256	0.14462025	6.63563547	3.23E-11	8.20E-10
ENSDARG00000098286	CU695232.1	356.120192	1.074511628	0.16194026	6.63523454	3.24E-11	8.22E-10
ENSDARG00000093244	CU681836.1	648.431986	1.06884845	0.16110497	6.63448475	3.26E-11	8.25E-10
ENSDARG00000104906	mtr	1648.94996	0.776830362	0.11711444	6.63308788	3.29E-11	8.32E-10
ENSDARG00000002231	plpp2a	472.572553	1.319403927	0.19893322	6.63239627	3.30E-11	8.35E-10
ENSDARG00000001014	myh9b	17026.2389	0.693650183	0.10461014	6.63081205	3.34E-11	8.43E-10
ENSDARG00000015076	gja8b	205.803928	-1.461531948	0.22044026	-6.63005922	3.36E-11	8.47E-10

Table S2. DEGs of WT vs. *terfa-/-*

ENSDARG00000052846	fsta	1244.53588	0.642043679	0.09689013	6.62651253	3.44E-11	8.66E-10
ENSDARG00000079591	si:ch211-203d1.3	546.655212	1.101414121	0.16625742	6.62475166	3.48E-11	8.76E-10
ENSDARG00000104484	itgb1b	3396.6553	0.652769474	0.09857299	6.62219401	3.54E-11	8.90E-10
ENSDARG00000104566	foxi1	94.2883439	1.85403157	0.28008794	6.61946236	3.61E-11	9.06E-10
ENSDARG00000037921	gng13b	2692.32	-1.286799587	0.19442599	-6.61845451	3.63E-11	9.11E-10
ENSDARG00000078092	limd2	1024.51326	0.909772616	0.13758797	6.61229764	3.78E-11	9.48E-10
ENSDARG00000033539	paics	8135.05125	1.110446977	0.16793535	6.61234793	3.78E-11	9.48E-10
ENSDARG00000075881	si:ch211-39k3.2	190.409075	1.786057797	0.27013114	6.61181748	3.80E-11	9.50E-10
ENSDARG00000096655	BX901920.1	157.634868	2.649853735	0.40080405	6.61134473	3.81E-11	9.51E-10
ENSDARG00000002230	sypb	5020.82794	-0.916245979	0.13858633	-6.61137342	3.81E-11	9.51E-10
ENSDARG00000037285	mipa	1988.89942	-2.134731502	0.32294975	-6.61010419	3.84E-11	9.58E-10
ENSDARG00000094560	BX005254.2	33.1296357	3.504456639	0.53035555	6.60774958	3.90E-11	9.73E-10
ENSDARG00000063126	gusb	1959.98656	0.820344056	0.12419909	6.60507299	3.97E-11	9.90E-10
ENSDARG00000055276	rel	768.864887	1.267360752	0.19192391	6.60345427	4.02E-11	9.99E-10
ENSDARG00000004748	zgc:100868	6205.75026	0.639096289	0.09681356	6.6013098	4.08E-11	1.01E-09
ENSDARG00000053939	tgfa	178.30755	-1.662924527	0.25186699	-6.60239178	4.05E-11	1.01E-09
ENSDARG00000100797	CABZ01076996.1	560.729304	1.021627886	0.15475473	6.60159378	4.07E-11	1.01E-09
ENSDARG00000058103	glra4b	153.491896	-2.615849487	0.39645696	-6.5980668	4.17E-11	1.03E-09
ENSDARG00000076585	si:ch211-113e8.11	700.85591	-0.994296103	0.15074442	-6.59590666	4.23E-11	1.05E-09
ENSDARG00000101688	si:dkey-207l24.2	109.331507	-2.897854479	0.43987708	-6.58787337	4.46E-11	1.10E-09
ENSDARG00000098350	mxd3	616.535744	-1.261212631	0.19144536	-6.5878466	4.46E-11	1.10E-09
ENSDARG00000095915	col6a3	298.397963	1.520495893	0.23083563	6.58692026	4.49E-11	1.11E-09
ENSDARG00000103067	adgrd2	622.874002	1.303289325	0.19793052	6.58458008	4.56E-11	1.12E-09
ENSDARG00000039007	eno3	29688.8763	0.679720089	0.10330703	6.5796111	4.72E-11	1.16E-09
ENSDARG00000087198	cthrc1a	1411.62812	0.88258923	0.1341765	6.57782289	4.77E-11	1.17E-09
ENSDARG00000034757	zdhhc2	150.436645	-1.42684384	0.21696023	-6.57652263	4.82E-11	1.18E-09
ENSDARG00000069752	ckba	1301.00981	0.952271245	0.14479701	6.57659488	4.81E-11	1.18E-09
ENSDARG00000043571	KIF2A	1598.75276	-0.723351111	0.10999956	-6.57594553	4.83E-11	1.19E-09
ENSDARG00000040432	klf2b	1459.9338	-0.67384202	0.10254123	-6.57142517	4.98E-11	1.22E-09
ENSDARG00000055632	smtnl	849.421107	1.152377342	0.17547244	6.56728403	5.12E-11	1.25E-09
ENSDARG00000062906	kcnv2b	56.9713518	-2.906012514	0.44254093	-6.56665256	5.15E-11	1.26E-09
ENSDARG00000008788	camk1gb	437.883159	-1.343255618	0.20461542	-6.56478185	5.21E-11	1.27E-09
ENSDARG00000052207	c3a.3	2223.37449	1.405186509	0.21413586	6.56212614	5.30E-11	1.29E-09
ENSDARG00000036684	ndufa7	973.522316	0.730399717	0.11130582	6.56209796	5.31E-11	1.29E-09
ENSDARG00000060885	znf592	654.13222	-0.934841843	0.14246707	-6.56181022	5.32E-11	1.30E-09
ENSDARG00000046133	b3galnt2	340.420704	1.071558438	0.16334842	6.55995603	5.38E-11	1.31E-09
ENSDARG00000036295	golga7ba	1484.17822	-0.9815554	0.14966697	-6.55826337	5.44E-11	1.32E-09
ENSDARG00000079397	cerkl	124.190675	-1.629006644	0.24836269	-6.55898307	5.42E-11	1.32E-09
ENSDARG00000041400	ndufa3	1298.84027	0.848429954	0.12946624	6.55329111	5.63E-11	1.37E-09
ENSDARG00000113393	golim4a	1494.70437	0.674499451	0.102945	6.55203723	5.68E-11	1.38E-09
ENSDARG00000108372	LO017648.1	531.073281	-1.733805671	0.26502532	-6.54203779	6.07E-11	1.47E-09
ENSDARG00000088181	mpped1	428.723051	-1.279873806	0.19567948	-6.54066442	6.12E-11	1.48E-09
ENSDARG00000014790	g3bp2	2267.11082	-0.82279902	0.12580466	-6.54029046	6.14E-11	1.48E-09
ENSDARG00000010572	slc25a25a	1219.35473	1.116208703	0.17065691	6.54065914	6.12E-11	1.48E-09
ENSDARG00000026137	sfxn5b	930.14049	0.712250863	0.10891764	6.53935283	6.18E-11	1.49E-09
ENSDARG00000070670	crip2	1488.59767	-0.800089874	0.12238586	-6.53743734	6.26E-11	1.51E-09
ENSDARG00000029692	rufy3	1620.49676	-0.628156728	0.0961102	-6.53579684	6.33E-11	1.52E-09
ENSDARG00000079645	sc:d217	205.302811	2.312662528	0.35385905	6.53554724	6.34E-11	1.52E-09
ENSDARG00000076106	ftr43	30.4745508	3.44479304	0.52731138	6.53274931	6.46E-11	1.55E-09
ENSDARG00000012972	cfl1l	9252.21365	0.67858049	0.10388255	6.53218957	6.48E-11	1.56E-09
ENSDARG00000116052	il4r.2	165.377212	1.387354115	0.21243034	6.530866	6.54E-11	1.57E-09
ENSDARG00000029406	h2ax	902.634701	-1.193747996	0.18280627	-6.53012605	6.57E-11	1.58E-09
ENSDARG00000055190	slc17a5	2385.94266	-0.629231485	0.096401	-6.52722982	6.70E-11	1.60E-09
ENSDARG00000075917	cacna1db	173.764931	1.395603555	0.21389513	6.52470935	6.81E-11	1.63E-09
ENSDARG00000075707	nid2a	6458.86648	0.757679772	0.11624373	6.5180273	7.12E-11	1.70E-09
ENSDARG00000061205	stag1a	1958.90001	-0.867249709	0.13308724	-6.5164001	7.20E-11	1.72E-09
ENSDARG00000104574	pcsk6	477.5134	0.998546805	0.1533475	6.51166018	7.43E-11	1.77E-09
ENSDARG00000016143	xiap	840.465036	0.819740727	0.12589888	6.5111043	7.46E-11	1.78E-09

Table S2. DEGs of WT vs. *terfa-/-*

ENSDARG00000102702	lrrn3b	1220.70044	-0.743731171	0.11427026	-6.50852794	7.59E-11	1.81E-09
ENSDARG00000100956	si:ch211-276i12.4	314.970604	-2.177374795	0.33461707	-6.50706431	7.66E-11	1.82E-09
ENSDARG00000061450	tesk2	788.795299	-0.764750424	0.11754894	-6.50580448	7.73E-11	1.84E-09
ENSDARG00000038465	stmn3	939.713874	-0.737197974	0.11335535	-6.50342437	7.85E-11	1.86E-09
ENSDARG00000075540	FAM107A	98.5583184	-1.907921875	0.29341093	-6.50255896	7.90E-11	1.87E-09
ENSDARG00000037646	rgs11	327.664568	-1.545252743	0.23763985	-6.50249832	7.90E-11	1.87E-09
ENSDARG00000074216	wu:fc38h03	645.33137	0.840380463	0.12931625	6.4986455	8.10E-11	1.92E-09
ENSDARG00000070053	foxg1d	234.614812	-1.696674991	0.2612233	-6.49511344	8.30E-11	1.96E-09
ENSDARG00000012968	rhoub	1721.91563	0.915890265	0.14102774	6.49439808	8.34E-11	1.97E-09
ENSDARG00000035136	selenow1	7073.88667	0.792094877	0.12204833	6.49000974	8.58E-11	2.03E-09
ENSDARG00000056092	si:dkey-12h9.6	1003.2684	-1.120232828	0.17263581	-6.48899473	8.64E-11	2.04E-09
ENSDARG00000100105	impg2b	167.993682	-1.91130825	0.29455303	-6.48884259	8.65E-11	2.04E-09
ENSDARG00000041414	bmf2	148.606269	-1.432168831	0.22078595	-6.48668466	8.77E-11	2.07E-09
ENSDARG00000092774	tars3	1288.00789	-1.040851269	0.16050594	-6.48481463	8.88E-11	2.09E-09
ENSDARG00000099974	ldb3b	10825.87	0.696961417	0.10749103	6.48390304	8.94E-11	2.10E-09
ENSDARG00000061049	rnf182	80.4208304	-2.029608592	0.31309117	-6.48248432	9.02E-11	2.12E-09
ENSDARG00000100741	cdc20	1051.59563	-1.220313323	0.1883345	-6.47949952	9.20E-11	2.16E-09
ENSDARG00000098012	itgae.1	161.894075	-1.57906167	0.24384844	-6.47558648	9.44E-11	2.21E-09
ENSDARG00000103519	adamts1	1485.93422	1.292376572	0.19958784	6.47522714	9.47E-11	2.22E-09
ENSDARG00000062341	wfs1a	600.912728	1.009137863	0.15586928	6.47425749	9.53E-11	2.23E-09
ENSDARG00000095268	si:dkey-261h17.1	3102.1003	-0.656428598	0.10143232	-6.47159181	9.70E-11	2.27E-09
ENSDARG00000070511	si:dkey-183j2.10	509.207059	-2.801167949	0.43285505	-6.47137641	9.71E-11	2.27E-09
ENSDARG00000055344	mipep	793.763816	0.769517329	0.11892603	6.47055434	9.76E-11	2.28E-09
ENSDARG00000069261	metap2a	351.104906	1.117696452	0.17276083	6.46961744	9.83E-11	2.29E-09
ENSDARG00000013763	rrbp1a	3012.06031	0.486554779	0.07520576	6.46964826	9.82E-11	2.29E-09
ENSDARG00000067673	snapc4	535.384111	-0.851001356	0.13155726	-6.4686763	9.89E-11	2.30E-09
ENSDARG00000011370	fyna	2176.03711	-0.786301121	0.12156541	-6.46813203	9.92E-11	2.30E-09
ENSDARG00000044861	opn1lw2	24604.6301	-2.849623969	0.44054559	-6.4683975	9.90E-11	2.30E-09
ENSDARG00000086756	RGS9BP	418.179395	-1.965532155	0.30434102	-6.45832163	1.06E-10	2.45E-09
ENSDARG00000074613	si:ch211-240l19.6	141.905209	-2.788891551	0.43224146	-6.45216115	1.10E-10	2.55E-09
ENSDARG00000091138	actr3	6993.35083	0.692261035	0.10732369	6.45021653	1.12E-10	2.58E-09
ENSDARG00000040085	ccsapb	703.511648	-0.921753716	0.14304306	-6.44389002	1.16E-10	2.69E-09
ENSDARG00000014803	cryba1l2	345.250317	-1.303013499	0.20221977	-6.4435515	1.17E-10	2.70E-09
ENSDARG00000096756	si:ch211-196c10.1	100.314549	-2.12417835	0.3301229	-6.43450771	1.24E-10	2.86E-09
ENSDARG00000111102	camk2n2	710.909048	-0.998195887	0.15513913	-6.43419788	1.24E-10	2.86E-09
ENSDARG00000097157	si:ch211-207n23.2	716.745171	1.296976035	0.20158727	6.43381926	1.24E-10	2.87E-09
ENSDARG00000099439	serp1	1648.78291	0.855134895	0.1329895	6.4300932	1.28E-10	2.93E-09
ENSDARG00000063701	rreb1a	2022.55791	0.670766683	0.10432429	6.42963071	1.28E-10	2.94E-09
ENSDARG00000054031	mxd4	695.82382	-0.72754867	0.11318308	-6.42806941	1.29E-10	2.97E-09
ENSDARG00000104266	si:dkey-29m1.2	40.1912695	3.020490028	0.47013675	6.42470525	1.32E-10	3.03E-09
ENSDARG00000052712	suclg1	10029.2713	0.641435306	0.09985584	6.42361322	1.33E-10	3.05E-09
ENSDARG00000045298	wipf2a	2086.95454	0.58524032	0.09111177	6.42332318	1.33E-10	3.05E-09
ENSDARG00000073720	RNF180	145.953248	-1.967240737	0.30639158	-6.42067487	1.36E-10	3.10E-09
ENSDARG00000077151	cbln2b	708.944269	-1.299721849	0.2024218	-6.42085907	1.36E-10	3.10E-09
ENSDARG00000015201	pcmt	1434.24757	0.611807382	0.09529449	6.42017593	1.36E-10	3.11E-09
ENSDARG00000100139	si:ch211-113d22.2	537.011482	-2.838477944	0.44212525	-6.42007656	1.36E-10	3.11E-09
ENSDARG00000026519	ralgps2	943.050884	-0.674045692	0.1050156	-6.41852938	1.38E-10	3.14E-09
ENSDARG00000004169	stmn1a	1583.40259	-1.132833113	0.17661792	-6.41403279	1.42E-10	3.23E-09
ENSDARG00000091280	si:ch211-66k16.27	335.571192	-1.28264754	0.2000357	-6.41209306	1.44E-10	3.27E-09
ENSDARG00000058597	nt5c3a	2903.48162	0.823550517	0.12844632	6.41163192	1.44E-10	3.27E-09
ENSDARG00000109859	CABZ01072242.1	326.926671	1.310861613	0.20447104	6.41098911	1.45E-10	3.28E-09
ENSDARG00000052775	opn3	107.318472	-1.745406762	0.27230251	-6.40980779	1.46E-10	3.31E-09
ENSDARG00000095603	grid2ipb	529.778097	-1.007043724	0.15723101	-6.40486715	1.51E-10	3.41E-09
ENSDARG00000098376	si:ch211-207l14.1	396.02944	-2.889347128	0.45122196	-6.40338328	1.52E-10	3.44E-09
ENSDARG00000060248	fgd4b	122.247184	-1.843383104	0.28797512	-6.40118867	1.54E-10	3.49E-09
ENSDARG00000025522	sgk1	2883.96343	1.224981172	0.19144127	6.39873086	1.57E-10	3.54E-09
ENSDARG00000104340	rspo1	156.907982	1.495402502	0.23373105	6.39796245	1.57E-10	3.56E-09
ENSDARG00000036329	ndufa1	1505.06239	0.775825067	0.12133964	6.39383042	1.62E-10	3.65E-09

Table S2. DEGs of WT vs. *terfa-/-*

ENSDARG00000092829	CR589943.1	80.3996638	-2.279217222	0.35655187	-6.39238612	1.63E-10	3.68E-09
ENSDARG00000021113	ptmaa	14362.2985	-0.73109064	0.1143883	-6.3913062	1.64E-10	3.71E-09
ENSDARG00000032056	arl6	518.654902	-0.919921254	0.14394206	-6.39091346	1.65E-10	3.71E-09
ENSDARG00000115868	sp5l	136.278799	-2.39644452	0.37512719	-6.38835199	1.68E-10	3.77E-09
ENSDARG00000001913	palmda	526.416326	-1.170226211	0.18321983	-6.38700625	1.69E-10	3.80E-09
ENSDARG00000044938	cbx2	657.644754	-0.973789558	0.15251341	-6.38494402	1.71E-10	3.85E-09
ENSDARG00000079578	rbpms2b	1328.54453	-0.978082178	0.15332188	-6.37927337	1.78E-10	3.99E-09
ENSDARG00000053323	zgc:112285	813.802283	-1.197355679	0.18771257	-6.37866535	1.79E-10	4.00E-09
ENSDARG00000101984	pde6gb	949.953639	-1.314140862	0.20606517	-6.37730706	1.80E-10	4.04E-09
ENSDARG00000098369	si:dkey-165o8.1	53.9788032	-2.715827774	0.42613489	-6.3731645	1.85E-10	4.14E-09
ENSDARG00000063572	perp	8193.02242	0.810074438	0.12718459	6.36928163	1.90E-10	4.24E-09
ENSDARG00000105136	ccdc50	237.594033	-1.291457109	0.20284659	-6.3666692	1.93E-10	4.31E-09
ENSDARG00000079163	best2	24.7268009	4.163235012	0.65406787	6.36514219	1.95E-10	4.35E-09
ENSDARG00000070769	foxg1a	813.668355	-0.887580413	0.13948746	-6.36315582	1.98E-10	4.41E-09
ENSDARG00000019498	cry5	388.347698	0.911564498	0.14328517	6.36188995	1.99E-10	4.44E-09
ENSDARG00000023472	ctnnb2	17046.0423	0.620851057	0.09760023	6.36116388	2.00E-10	4.45E-09
ENSDARG00000019302	mid1ip1b	1287.59493	-0.767432075	0.12064335	-6.36116347	2.00E-10	4.45E-09
ENSDARG00000076257	si:ch211-285c6.1	43.571886	7.842631439	1.2329662	6.36078383	2.01E-10	4.46E-09
ENSDARG00000038608	sdhc	2824.40044	0.674009249	0.1059641	6.36073181	2.01E-10	4.46E-09
ENSDARG00000099491	tmem176l.1	837.581923	1.124004642	0.17677653	6.35833656	2.04E-10	4.52E-09
ENSDARG00000092124	cox14	615.331627	0.900886835	0.14169311	6.35801426	2.04E-10	4.53E-09
ENSDARG00000095082	ITLN1	136.06516	2.352356619	0.37019427	6.35438426	2.09E-10	4.63E-09
ENSDARG00000101368	cngb3.2	541.76328	-3.268485884	0.51451961	-6.35250011	2.12E-10	4.68E-09
ENSDARG00000094324	efemp2a	2048.48525	0.698448465	0.10996251	6.35169619	2.13E-10	4.70E-09
ENSDARG00000057481	RNF157	1537.70182	-0.803536281	0.12651764	-6.35118006	2.14E-10	4.72E-09
ENSDARG00000100887	si:dkey-11f4.20	28.2635815	3.047817795	0.48017142	6.34735367	2.19E-10	4.83E-09
ENSDARG00000018319	arglu1b	2142.91723	-0.640966585	0.10100416	-6.34594234	2.21E-10	4.87E-09
ENSDARG00000114516	CABZ01021592.1	4126.6244	0.819358145	0.1291724	6.34313618	2.25E-10	4.96E-09
ENSDARG00000054666	pgpep1	536.022957	0.87760442	0.13835892	6.34295513	2.25E-10	4.96E-09
ENSDARG00000007788	atp2b1b	2211.31088	-2.228349698	0.35147917	-6.33991958	2.30E-10	5.05E-09
ENSDARG00000074989	sparcl1	814.433752	-1.003020756	0.15833752	-6.33470029	2.38E-10	5.22E-09
ENSDARG00000038166	hyal6	55.347887	-3.473869683	0.54891286	-6.32863598	2.47E-10	5.43E-09
ENSDARG00000099698	BX548059.1	22.235575	6.210217823	0.98148368	6.3273776	2.49E-10	5.47E-09
ENSDARG00000053835	plekhh1	697.632208	1.322016372	0.20894386	6.32713682	2.50E-10	5.47E-09
ENSDARG00000053254	mylpfa	222007.963	0.773405989	0.12227041	6.32537317	2.53E-10	5.53E-09
ENSDARG00000007655	crybb1l3	76.7556172	-2.531457979	0.40031072	-6.32373265	2.55E-10	5.58E-09
ENSDARG00000104919	si:ch211-153b23.3	519.221527	1.49089271	0.23580103	6.32267248	2.57E-10	5.61E-09
ENSDARG00000092166	si:dkey-147f3.8	405.737334	1.296593645	0.20506352	6.32288777	2.57E-10	5.61E-09
ENSDARG00000094426	her4.2	311.030986	-1.697454493	0.26855603	-6.32067162	2.60E-10	5.68E-09
ENSDARG00000092099	si:ch211-133n4.6	684.262408	1.11743968	0.17684845	6.31862876	2.64E-10	5.75E-09
ENSDARG00000056511	arr3a	9681.91454	-2.660204532	0.42104223	-6.31814181	2.65E-10	5.76E-09
ENSDARG00000099741	mvb12a	361.287576	0.948263919	0.15019653	6.31348736	2.73E-10	5.93E-09
ENSDARG00000099534	CABZ01066926.1	385.27152	-1.18417897	0.18759767	-6.31233292	2.75E-10	5.97E-09
ENSDARG00000007247	ric8a	881.865127	-1.109210805	0.17573464	-6.31185071	2.76E-10	5.99E-09
ENSDARG00000088584	si:ch73-352p18.4	170.629146	-2.224010835	0.35243034	-6.31049764	2.78E-10	6.03E-09
ENSDARG00000036671	tnni4b.2	248.269474	1.199131459	0.19007874	6.30860377	2.82E-10	6.10E-09
ENSDARG00000024160	hao1	1997.81126	1.014649454	0.16086999	6.30726359	2.84E-10	6.15E-09
ENSDARG00000074983	jac9	96.1159784	6.922893311	1.0978652	6.30577719	2.87E-10	6.20E-09
ENSDARG00000038639	elovl6l	742.26235	1.112568728	0.17649548	6.30366682	2.91E-10	6.28E-09
ENSDARG00000095236	CT990618.1	407.060853	1.288680439	0.20446962	6.30255218	2.93E-10	6.32E-09
ENSDARG00000003311	pank2	1639.25493	-0.698008688	0.11078435	-6.30060732	2.96E-10	6.40E-09
ENSDARG00000020574	atp2a1	269619.713	0.809130292	0.12844297	6.29952964	2.99E-10	6.43E-09
ENSDARG00000045219	dkk1b	181.624294	1.2329907	0.195735	6.29928591	2.99E-10	6.43E-09
ENSDARG00000076005	piezo2a.2	1995.18835	0.87305563	0.13859356	6.29939522	2.99E-10	6.43E-09
ENSDARG00000069946	itga6b	2702.65984	1.02573423	0.16289651	6.29684593	3.04E-10	6.53E-09
ENSDARG00000009215	zgc:112437	265.738729	-1.366460361	0.21712074	-6.2935507	3.10E-10	6.67E-09
ENSDARG00000009505	prelid3b	4019.08532	0.728060054	0.11574889	6.28999612	3.17E-10	6.81E-09
ENSDARG00000092660	cyp27c1	434.503895	1.438342317	0.22880479	6.28632966	3.25E-10	6.97E-09

Table S2. DEGs of WT vs. *terfa-/-*

ENSDARG00000087413	bean1	461.378231	-0.965126319	0.15356899	-6.28464349	3.29E-10	7.04E-09
ENSDARG00000090352	CR855311.1	979.625545	2.582668599	0.41098292	6.28412636	3.30E-10	7.06E-09
ENSDARG00000071410	cks2	135.990421	-1.642363151	0.2614551	-6.28162594	3.35E-10	7.17E-09
ENSDARG00000002385	atm	752.221731	0.989382843	0.15755927	6.27943274	3.40E-10	7.26E-09
ENSDARG00000036044	rps20	32918.2752	0.709020246	0.11292945	6.27843533	3.42E-10	7.30E-09
ENSDARG00000071590	si:ch211-236g6.1	130.295271	-1.867364018	0.29750938	-6.27665595	3.46E-10	7.38E-09
ENSDARG00000053917	alg10	268.774824	-1.167957542	0.18613717	-6.27471412	3.50E-10	7.47E-09
ENSDARG00000007362	sft2d1	940.637838	0.856135093	0.1364805	6.27294813	3.54E-10	7.54E-09
ENSDARG00000103980	ets2	1603.47406	0.954802612	0.15220745	6.27303454	3.54E-10	7.54E-09
ENSDARG00000023272	tfap2d	454.072734	-1.804377632	0.28771153	-6.27148176	3.58E-10	7.60E-09
ENSDARG00000070371	kat7a	589.740828	-0.825203412	0.131668	-6.26730414	3.67E-10	7.80E-09
ENSDARG00000079049	cercam	1031.14837	0.669872075	0.10689044	6.26690378	3.68E-10	7.82E-09
ENSDARG00000014340	rab11al	662.24618	0.927565992	0.14802646	6.26621747	3.70E-10	7.85E-09
ENSDARG00000056084	igsf21b	583.064091	-1.429122726	0.22810154	-6.26529181	3.72E-10	7.89E-09
ENSDARG00000001975	hsd11b2	1689.9627	1.146216474	0.18297575	6.26430809	3.74E-10	7.93E-09
ENSDARG00000087993	bada	393.814301	-0.874843038	0.13972465	-6.26119336	3.82E-10	8.08E-09
ENSDARG00000113572	si:dkey-102c8.3	1117.4589	0.900993234	0.14390285	6.26112162	3.82E-10	8.08E-09
ENSDARG00000015128	rpl27	24302.1551	0.70185428	0.11213092	6.25923934	3.87E-10	8.17E-09
ENSDARG00000099217	RDH8	72.969454	-2.331628438	0.37253451	-6.25882538	3.88E-10	8.18E-09
ENSDARG00000045677	opn1sw1	29929.0988	-3.590161475	0.57379562	-6.25686457	3.93E-10	8.28E-09
ENSDARG00000034211	capn2l	1293.19841	1.082971491	0.17310073	6.25630796	3.94E-10	8.30E-09
ENSDARG00000037262	cdkn2a/b	32.2589869	3.490618838	0.55815792	6.25381937	4.01E-10	8.43E-09
ENSDARG00000078748	si:ch211-137a8.4	10047.0303	-1.176385678	0.1881787	-6.25142843	4.07E-10	8.55E-09
ENSDARG00000054641	tent5ab	349.413241	-1.69891306	0.27177309	-6.25121892	4.07E-10	8.55E-09
ENSDARG00000060668	tm4sf18	267.174319	1.228756141	0.19655648	6.25141499	4.07E-10	8.55E-09
ENSDARG00000077882	ccn5	66.2173882	1.914643416	0.30633031	6.25025793	4.10E-10	8.59E-09
ENSDARG00000116617	spaca4l	4797.07534	0.670953304	0.10736254	6.24941713	4.12E-10	8.63E-09
ENSDARG00000045936	pax6b	1856.23277	-1.393118033	0.22295984	-6.24829122	4.15E-10	8.69E-09
ENSDARG00000028804	ankrd9	788.256856	1.449799816	0.23206823	6.24729989	4.18E-10	8.72E-09
ENSDARG00000100000	CR352226.5	477.683689	0.90893517	0.14549163	6.24733637	4.18E-10	8.72E-09
ENSDARG00000046157	RPS17	26497.7752	0.77485434	0.1240299	6.24731918	4.18E-10	8.72E-09
ENSDARG00000043796	ms4a17a.7	274.3038	1.52948159	0.24484225	6.24680431	4.19E-10	8.74E-09
ENSDARG00000090190	bcam	2066.12534	0.736788064	0.11795894	6.2461401	4.21E-10	8.77E-09
ENSDARG00000074508	si:dkey-28e7.3	849.340081	-0.855707274	0.13701509	-6.24535069	4.23E-10	8.79E-09
ENSDARG00000040764	id1	341.292838	1.640952406	0.26273043	6.24576457	4.22E-10	8.79E-09
ENSDARG00000063519	PLEKHB1	101.220161	-2.117494873	0.33904938	-6.24538793	4.23E-10	8.79E-09
ENSDARG00000027930	naprt	1364.12163	0.654615449	0.10493918	6.23804646	4.43E-10	9.21E-09
ENSDARG00000045074	arl4d	1205.79424	-0.750959708	0.12039779	-6.23732131	4.45E-10	9.24E-09
ENSDARG00000062606	si:dkey-26i13.8	217.475858	-1.819496184	0.29176989	-6.2360656	4.49E-10	9.31E-09
ENSDARG00000078574	si:dkey-21c19.3	1105.49821	-0.777643206	0.1247069	-6.23576729	4.50E-10	9.32E-09
ENSDARG00000020645	slc7a3a	2780.3116	-0.599592574	0.09617291	-6.23452695	4.53E-10	9.39E-09
ENSDARG00000074002	slc6a11a	397.927229	0.94187859	0.15111062	6.23304018	4.57E-10	9.47E-09
ENSDARG00000010031	ssh1b	1150.31316	-0.881223449	0.14139803	-6.23221874	4.60E-10	9.51E-09
ENSDARG00000078078	lrfn4b	759.261881	-0.822118549	0.13204092	-6.22624083	4.78E-10	9.87E-09
ENSDARG00000005739	gpm6ba	2128.65152	-0.804838667	0.12927896	-6.22559681	4.80E-10	9.90E-09
ENSDARG00000097187	si:ch211-153l6.6	364.616326	-1.488378209	0.23916918	-6.22311888	4.87E-10	1.01E-08
ENSDARG00000058794	si:dkey-19a16.2	138.536681	-1.925700172	0.30947771	-6.22241962	4.90E-10	1.01E-08
ENSDARG00000009922	dmbx1a	1273.60768	-1.122825128	0.18053389	-6.21946997	4.99E-10	1.03E-08
ENSDARG00000010472	atp1a2a	4911.93624	0.937965665	0.15081011	6.21951436	4.99E-10	1.03E-08
ENSDARG00000008867	rap1b	2677.74794	0.617516355	0.09934679	6.21576549	5.11E-10	1.05E-08
ENSDARG00000057249	zmynd11	3963.15947	-0.560478681	0.09017213	-6.21565348	5.11E-10	1.05E-08
ENSDARG00000053609	gatd1	571.8936	-0.882052529	0.1419618	-6.21330884	5.19E-10	1.06E-08
ENSDARG00000099008	EGFLAM	354.4245	-1.981952376	0.31911779	-6.21072358	5.27E-10	1.08E-08
ENSDARG00000093924	BX890562.2	33.0044547	2.813025456	0.45298645	6.20995502	5.30E-10	1.09E-08
ENSDARG00000010602	zgc:112294	90.1801276	-2.612624759	0.4207201	-6.20988809	5.30E-10	1.09E-08
ENSDARG00000087359	c3a.2	1475.28368	3.596282377	0.57918541	6.20920749	5.33E-10	1.09E-08
ENSDARG00000097369	FP017274.1	119.2292	-1.790143044	0.28834945	-6.20824162	5.36E-10	1.10E-08
ENSDARG00000045147	tmem184ba	1455.17374	-0.690839814	0.11136259	-6.20351787	5.52E-10	1.13E-08

Table S2. DEGs of WT vs. *terfa-/-*

ENSDARG00000087784	si:dkeyp-110a12.4	536.026739	0.92467268	0.14911455	6.20108939	5.61E-10	1.14E-08
ENSDARG00000098817	CABZ01055522.1	523.573381	-1.332600045	0.21486231	-6.20211164	5.57E-10	1.14E-08
ENSDARG00000025504	gucy2f	159.540984	-1.995056049	0.32187697	-6.19819437	5.71E-10	1.16E-08
ENSDARG00000037675	npm1b	828.567749	-0.674313519	0.10884454	-6.19519833	5.82E-10	1.18E-08
ENSDARG00000091298	pmela	5975.77604	0.745788755	0.12042331	6.19305982	5.90E-10	1.20E-08
ENSDARG00000043243	prkchb	282.690931	1.028881603	0.16623807	6.1892058	6.05E-10	1.23E-08
ENSDARG00000008858	cyp7b1	379.605475	1.057479703	0.17090464	6.18754238	6.11E-10	1.24E-08
ENSDARG00000099470	muc5.3	297.088815	1.92997532	0.31199773	6.1858633	6.18E-10	1.25E-08
ENSDARG00000068995	h2ax1	11658.9679	-0.588289226	0.09510698	-6.18555263	6.19E-10	1.25E-08
ENSDARG00000034624	nuf2	562.669605	-1.204907504	0.19486545	-6.18327944	6.28E-10	1.27E-08
ENSDARG00000054942	lgals2a	919.497385	0.973655341	0.1575905	6.17838871	6.48E-10	1.31E-08
ENSDARG00000102765	lonp1	2110.73091	0.574655018	0.09303517	6.17675024	6.54E-10	1.32E-08
ENSDARG00000001437	slc2a1a	195.988795	-1.685404387	0.27289464	-6.17602594	6.57E-10	1.33E-08
ENSDARG00000045886	slc38a2	3778.6529	-0.629447478	0.10195116	-6.17400975	6.66E-10	1.34E-08
ENSDARG00000009001	pdia6	3771.26024	0.790039097	0.12800371	6.17200138	6.74E-10	1.36E-08
ENSDARG00000088549	si:ch1073-390k14.	308.005867	0.994438563	0.16111501	6.17222798	6.73E-10	1.36E-08
ENSDARG00000095185	si:ch211-274p24.2	53.5531774	-2.306042547	0.37363394	-6.17193013	6.75E-10	1.36E-08
ENSDARG00000015404	arl3l2	276.748821	-3.08613194	0.50035458	-6.16788993	6.92E-10	1.39E-08
ENSDARG00000058556	muc5.2	2419.66639	1.150840386	0.1866556	6.16558182	7.02E-10	1.41E-08
ENSDARG00000037873	cyp3c3	52.7681377	2.907145662	0.47155574	6.16500958	7.05E-10	1.42E-08
ENSDARG00000003811	plxnb2a	3373.21386	0.616980822	0.1001207	6.16237058	7.17E-10	1.44E-08
ENSDARG00000040534	epcam	9063.65638	0.597669012	0.09699841	6.16163726	7.20E-10	1.44E-08
ENSDARG00000061081	arpp21	851.663864	1.051740001	0.17067811	6.16212591	7.18E-10	1.44E-08
ENSDARG00000056979	lhx9	1666.02527	-1.171653518	0.19011818	-6.16276432	7.15E-10	1.44E-08
ENSDARG00000017180	npc1	5551.97595	0.632906745	0.10274318	6.16008516	7.27E-10	1.45E-08
ENSDARG00000020794	neurod6b	1343.711	-1.281683375	0.20809929	-6.15899925	7.32E-10	1.46E-08
ENSDARG00000013734	polr3glb	427.929066	-1.084149711	0.17608434	-6.15699126	7.41E-10	1.48E-08
ENSDARG00000058160	tnfaip2b	238.820493	1.304216588	0.21193124	6.1539609	7.56E-10	1.51E-08
ENSDARG00000039133	lamb4	2742.58358	0.699496403	0.11367763	6.15333383	7.59E-10	1.51E-08
ENSDARG00000093608	ccl34b.1	173.699429	-1.688081747	0.27449003	-6.14988373	7.75E-10	1.55E-08
ENSDARG00000079009	abca1b	7374.05136	0.876592626	0.14271317	6.14233888	8.13E-10	1.62E-08
ENSDARG00000088634	adcy1b	433.64087	-1.592352401	0.25942144	-6.13809091	8.35E-10	1.66E-08
ENSDARG00000089961	si:cabz01007807.1	269.76916	1.257717751	0.20493287	6.13721815	8.40E-10	1.67E-08
ENSDARG00000038609	mpz	242.16649	-1.633186829	0.26611559	-6.13713314	8.40E-10	1.67E-08
ENSDARG00000045156	rgs9b	392.097226	-1.007410188	0.16414194	-6.13743319	8.39E-10	1.67E-08
ENSDARG00000070726	cnga3a	561.458897	-2.60747233	0.42503135	-6.13477649	8.53E-10	1.69E-08
ENSDARG00000055592	capn2b	1776.21925	0.625669373	0.10198567	6.13487544	8.52E-10	1.69E-08
ENSDARG00000041205	slc6a1l	117.540687	-2.363676709	0.38536394	-6.13362188	8.59E-10	1.70E-08
ENSDARG00000028048	rdh8a	957.375425	-1.105020239	0.18015225	-6.133813	8.58E-10	1.70E-08
ENSDARG00000099583	CABZ01034082.1	20.4275188	7.979767763	1.30087806	6.13413971	8.56E-10	1.70E-08
ENSDARG00000091116	pkhd1l1	3793.4726	1.022367617	0.16672559	6.13203782	8.68E-10	1.71E-08
ENSDARG00000070107	six7	1106.33054	-2.925433951	0.47729944	-6.12913758	8.84E-10	1.74E-08
ENSDARG00000008803	marcksb	10322.2405	-0.944934228	0.15428524	-6.12459269	9.09E-10	1.79E-08
ENSDARG00000038969	zgc:113142	146.102185	-1.257596703	0.20534336	-6.12436035	9.10E-10	1.79E-08
ENSDARG00000100869	adcy9	1006.79027	0.80075668	0.13074398	6.1246163	9.09E-10	1.79E-08
ENSDARG00000100240	CT737123.1	22.0262971	5.384699134	0.87952466	6.12228332	9.22E-10	1.82E-08
ENSDARG00000075045	cxcl18b	102.865026	2.354728038	0.38472482	6.12055141	9.33E-10	1.83E-08
ENSDARG00000071103	si:dkey-222p3.1	28.9508349	-3.300063979	0.5391291	-6.12110155	9.29E-10	1.83E-08
ENSDARG00000105561	BX072554.2	94.1650199	2.995053257	0.48959027	6.11746891	9.51E-10	1.87E-08
ENSDARG00000045893	kctd15a	1401.90213	-0.850895337	0.13913068	-6.1157996	9.61E-10	1.88E-08
ENSDARG00000034293	hif1ab	6558.40117	0.490293814	0.08017728	6.11512191	9.65E-10	1.89E-08
ENSDARG00000006983	celf3b	1529.79815	-1.243575534	0.20338563	-6.11437257	9.69E-10	1.90E-08
ENSDARG00000011613	rbm39a	5688.6318	-0.499676793	0.08174971	-6.11227604	9.82E-10	1.92E-08
ENSDARG00000044526	camk1ga	242.818081	-2.279653053	0.37329676	-6.10681186	1.02E-09	1.99E-08
ENSDARG00000075123	trafd1	777.301522	-0.660362387	0.10815978	-6.10543405	1.03E-09	2.00E-08
ENSDARG00000087070	znf977	120.054497	-1.632839465	0.26750497	-6.10395933	1.03E-09	2.02E-08
ENSDARG00000104130	rbm26	1473.70724	-0.61625555	0.10095791	-6.10408399	1.03E-09	2.02E-08
ENSDARG00000027734	srsf5b	7090.8159	-0.633916675	0.10387307	-6.10280098	1.04E-09	2.03E-08

Table S2. DEGs of WT vs. *terfa-/-*

ENSDARG00000056873	and3	6882.75472	0.687922646	0.11272524	6.10264959	1.04E-09	2.03E-08
ENSDARG00000009123	sele	138.248011	1.432405683	0.23476008	6.10157263	1.05E-09	2.04E-08
ENSDARG00000098777	CU693379.1	2312.9087	0.634708504	0.1040234	6.10159377	1.05E-09	2.04E-08
ENSDARG00000023648	idh3g	2544.25026	0.565575607	0.09268323	6.10224317	1.05E-09	2.04E-08
ENSDARG00000101736	CABZ01113816.1	29.9186252	5.895061815	0.96614219	6.10165032	1.05E-09	2.04E-08
ENSDARG00000101434	slc39a3	208.237719	1.33915931	0.21949069	6.10121251	1.05E-09	2.04E-08
ENSDARG00000059231	hephl1a	399.271628	0.93865113	0.15386901	6.10032604	1.06E-09	2.05E-08
ENSDARG00000004754	hspa4a	3671.41759	0.648296591	0.10631265	6.0980191	1.07E-09	2.08E-08
ENSDARG00000079672	efl1	300.919382	1.029480809	0.16885179	6.09694929	1.08E-09	2.09E-08
ENSDARG00000087100	ntng2b	333.559602	-1.357516347	0.22266655	-6.09663353	1.08E-09	2.10E-08
ENSDARG00000057465	gfpt1	2406.0467	0.743176041	0.1220642	6.08840278	1.14E-09	2.20E-08
ENSDARG00000028824	slc34a1a	263.45801	1.178964347	0.19364096	6.08840369	1.14E-09	2.20E-08
ENSDARG00000038508	chrnb3b	76.9282077	-1.885726362	0.30979533	-6.08700698	1.15E-09	2.22E-08
ENSDARG00000045904	nr2e3	412.203265	-1.727469414	0.28385383	-6.08577102	1.16E-09	2.24E-08
ENSDARG00000014969	ankhb	1056.0492	-0.66861641	0.10990178	-6.08376301	1.17E-09	2.26E-08
ENSDARG00000054683	prdm8b	1210.6532	-1.221390905	0.2007836	-6.08312096	1.18E-09	2.27E-08
ENSDARG00000030176	zgc:92429	564.38474	0.757856119	0.12463937	6.08039128	1.20E-09	2.31E-08
ENSDARG00000055559	chrna6	357.434922	-1.116452954	0.18363739	-6.07966025	1.20E-09	2.32E-08
ENSDARG00000073820	zgc:174917	83.9819279	2.130845903	0.35051344	6.07921306	1.21E-09	2.32E-08
ENSDARG00000097158	BX294100.1	75.801402	-2.381810971	0.3918723	-6.07802835	1.22E-09	2.33E-08
ENSDARG00000013687	cilp2	650.259811	1.236905715	0.20350023	6.07815398	1.22E-09	2.33E-08
ENSDARG00000100332	CABZ01084942.1	46.9703414	2.890865142	0.475758	6.07633535	1.23E-09	2.36E-08
ENSDARG00000110288	CT583625.5	24.3472258	3.608197616	0.59426267	6.07172183	1.27E-09	2.42E-08
ENSDARG00000040884	mxi1	2790.46568	-0.74936027	0.12347586	-6.06888065	1.29E-09	2.46E-08
ENSDARG00000056090	capza1b	10128.2065	0.531988125	0.08770302	6.06579006	1.31E-09	2.51E-08
ENSDARG00000098764	musk	314.447017	1.272927717	0.20988762	6.06480625	1.32E-09	2.52E-08
ENSDARG00000062621	satb1b	646.017135	-1.042423781	0.17189194	-6.0644134	1.32E-09	2.53E-08
ENSDARG00000017168	nr2f1b	673.439017	-0.894045015	0.14742887	-6.06424654	1.33E-09	2.53E-08
ENSDARG00000103504	CR855311.4	44.7439265	-3.432697132	0.56630454	-6.06157446	1.35E-09	2.57E-08
ENSDARG00000103483	litaf	245.899119	1.075977202	0.17753927	6.06050267	1.36E-09	2.58E-08
ENSDARG00000069615	ckmt2a	4306.47027	-2.68366604	0.44282351	-6.06035136	1.36E-09	2.58E-08
ENSDARG00000056477	ccdc125	225.413976	1.41682999	0.2338044	6.0598945	1.36E-09	2.59E-08
ENSDARG00000043249	irf1b	139.768867	1.731348407	0.2856918	6.06019641	1.36E-09	2.59E-08
ENSDARG00000036058	gnao1b	3459.85427	-1.081962696	0.17856113	-6.05934052	1.37E-09	2.60E-08
ENSDARG00000013422	slc8a1a	296.056586	0.931881636	0.15386553	6.05646775	1.39E-09	2.64E-08
ENSDARG00000076789	cx32.2	74.6954442	1.707473161	0.28201762	6.05449112	1.41E-09	2.67E-08
ENSDARG00000061260	si:dkey-202e22.2	603.187568	0.804400838	0.13285331	6.05480477	1.41E-09	2.67E-08
ENSDARG00000093440	tnfaip6	304.001705	1.334784145	0.22061403	6.05031396	1.45E-09	2.73E-08
ENSDARG00000115428	CABZ01101813.1	567.116822	0.772511325	0.12767567	6.05057578	1.44E-09	2.73E-08
ENSDARG00000053864	elmsan1b	2051.39873	0.797186265	0.13180202	6.04836145	1.46E-09	2.77E-08
ENSDARG00000105261	nfkb1	588.901409	0.837457781	0.13849439	6.04687143	1.48E-09	2.79E-08
ENSDARG00000096717	AL732488.2	403.231489	1.196845112	0.19793029	6.04680117	1.48E-09	2.79E-08
ENSDARG00000067831	tbata	95.2534565	2.040634488	0.33753068	6.04577492	1.49E-09	2.80E-08
ENSDARG00000027612	gatad1	455.043167	-0.848117994	0.14029197	-6.04537802	1.49E-09	2.81E-08
ENSDARG00000093357	nyap2b	874.222706	-1.480209878	0.24493414	-6.04329751	1.51E-09	2.84E-08
ENSDARG00000003564	dohh	419.195138	0.846275166	0.14002869	6.04358423	1.51E-09	2.84E-08
ENSDARG00000041691	bhlhe41	643.509138	1.040719025	0.17229668	6.04027338	1.54E-09	2.89E-08
ENSDARG00000055792	foxo4	2734.19797	-0.668860439	0.11075129	-6.03930164	1.55E-09	2.91E-08
ENSDARG00000002084	lamb2	6922.24365	0.727858292	0.1205362	6.03850388	1.56E-09	2.92E-08
ENSDARG00000079013	dpy19l3	1710.38645	-0.819768615	0.13577578	-6.03766461	1.56E-09	2.93E-08
ENSDARG00000103996	spdl1	457.036344	-1.018120543	0.16869252	-6.0353626	1.59E-09	2.97E-08
ENSDARG00000105749	CR788316.2	57.0888768	2.432138981	0.4031413	6.03296905	1.61E-09	3.01E-08
ENSDARG00000012610	saga	1241.55343	-2.278276445	0.37767753	-6.03233247	1.62E-09	3.01E-08
ENSDARG00000034836	si:dkey-222n6.2	246.416354	1.141915346	0.18927838	6.03299404	1.61E-09	3.01E-08
ENSDARG00000007382	ubtd1a	292.640294	-1.439837382	0.23868953	-6.03226042	1.62E-09	3.01E-08
ENSDARG00000103032	rap2b	1816.69318	0.683532961	0.11330382	6.03274412	1.61E-09	3.01E-08
ENSDARG00000059163	rbp3	2681.62799	-3.130975691	0.51902141	-6.03245963	1.61E-09	3.01E-08
ENSDARG00000044862	opn1lw1	27.6348697	-4.373851909	0.7250054	-6.03285425	1.61E-09	3.01E-08

Table S2. DEGs of WT vs. *terfa-/-*

ENSDARG00000076888	GANAB	1964.08007	0.715041463	0.11857052	6.03051661	1.63E-09	3.04E-08
ENSDARG00000079350	znf654	1277.66397	-0.612331249	0.10153636	-6.03065982	1.63E-09	3.04E-08
ENSDARG00000099149	hs3st3b1a	155.785457	-1.814608257	0.30120262	-6.02454346	1.70E-09	3.15E-08
ENSDARG00000075192	yme1l1a	1213.69448	0.692101726	0.11487487	6.0248316	1.69E-09	3.15E-08
ENSDARG00000097018	CR628341.1	36.0108288	6.508707182	1.08068724	6.02274825	1.71E-09	3.18E-08
ENSDARG00000016706	atic	5886.11365	1.131230934	0.18781003	6.02327232	1.71E-09	3.18E-08
ENSDARG00000002217	scocb	1574.1586	-0.744111433	0.12356739	-6.02190778	1.72E-09	3.20E-08
ENSDARG00000019713	oatx	602.988465	1.190011911	0.19768163	6.01984054	1.75E-09	3.24E-08
ENSDARG00000060586	ctdspl2b	1720.22396	-0.71369832	0.11859751	-6.01781899	1.77E-09	3.27E-08
ENSDARG00000113971	NA	28.5113296	-9.085516217	1.51000599	-6.01687429	1.78E-09	3.29E-08
ENSDARG00000053279	apln	2858.30185	-0.518376969	0.08615121	-6.01705934	1.78E-09	3.29E-08
ENSDARG00000095451	si:ch211-196l7.4	1835.28196	-0.808926702	0.13447232	-6.01556278	1.79E-09	3.31E-08
ENSDARG00000078888	iqgap1	4532.56479	0.618182561	0.10281719	6.01244392	1.83E-09	3.37E-08
ENSDARG00000077717	rpl29	7479.93039	0.620560176	0.10320868	6.01267446	1.82E-09	3.37E-08
ENSDARG00000099035	pcdh1g33	612.855905	-1.370389689	0.2279512	-6.01176795	1.84E-09	3.38E-08
ENSDARG00000039483	si:dkey-51a16.9	776.548322	-1.006954247	0.16751118	-6.01126585	1.84E-09	3.39E-08
ENSDARG00000087443	ptp4a2a	2079.70898	-0.699573178	0.11638933	-6.01062983	1.85E-09	3.40E-08
ENSDARG00000063910	mt-atp8	318.47722	6.789372686	1.13016041	6.0074416	1.88E-09	3.47E-08
ENSDARG00000004251	dhfr	445.74209	-0.947778078	0.15779818	-6.00626744	1.90E-09	3.49E-08
ENSDARG00000086112	si:ch211-266i6.3	813.502895	-1.109924103	0.18482437	-6.00529077	1.91E-09	3.51E-08
ENSDARG00000011459	gsna	3256.26659	0.971835893	0.1619084	6.00238083	1.94E-09	3.57E-08
ENSDARG00000009743	efhc1	171.598862	1.381217077	0.230162	6.00106474	1.96E-09	3.60E-08
ENSDARG00000007169	itgb1bp1	807.097737	0.659917055	0.11000597	5.99892026	1.99E-09	3.64E-08
ENSDARG00000011570	pou6f1	1096.31023	-0.916972081	0.15288353	-5.99784724	2.00E-09	3.66E-08
ENSDARG00000010445	trabd	829.500485	0.681968612	0.11377429	5.99404852	2.05E-09	3.75E-08
ENSDARG00000039099	aep1	11478.639	0.781095234	0.1303715	5.99130369	2.08E-09	3.80E-08
ENSDARG00000005619	nek2	183.667883	-1.48175787	0.24731258	-5.99143741	2.08E-09	3.80E-08
ENSDARG00000060901	trim62.1	273.36345	1.249679665	0.20869213	5.98814948	2.12E-09	3.88E-08
ENSDARG00000005122	atp2a2b	7067.90048	0.523550379	0.0875075	5.98291993	2.19E-09	4.00E-08
ENSDARG00000039142	arpc5a	2567.88858	0.611342996	0.10219419	5.9821701	2.20E-09	4.02E-08
ENSDARG00000035178	gna14	324.037406	1.016525779	0.16996003	5.98096971	2.22E-09	4.04E-08
ENSDARG00000062370	bcl2l13	1687.78083	0.715310025	0.1196023	5.98073782	2.22E-09	4.04E-08
ENSDARG00000020289	pif1	201.972129	-1.843113398	0.30821671	-5.97992685	2.23E-09	4.06E-08
ENSDARG00000078145	si:ch211-218g4.2	727.234945	0.883046075	0.14769116	5.97900441	2.25E-09	4.08E-08
ENSDARG00000033683	tpma	283236.082	0.712603514	0.11927954	5.97423107	2.31E-09	4.20E-08
ENSDARG00000101589	cacna1db	134.51365	-1.443408496	0.24169274	-5.97208057	2.34E-09	4.25E-08
ENSDARG00000075058	gabra6a	298.034277	-3.17714856	0.53202753	-5.97177474	2.35E-09	4.26E-08
ENSDARG00000094197	znfl1l	104.663728	2.999749105	0.50249573	5.96970068	2.38E-09	4.31E-08
ENSDARG00000042641	cyp51	692.263096	0.808339551	0.13548587	5.96622769	2.43E-09	4.40E-08
ENSDARG00000097416	si:ch73-306e8.2	151.316969	-1.433055107	0.24028201	-5.96405491	2.46E-09	4.45E-08
ENSDARG00000114529	CU457778.9	205.261184	1.748489843	0.29335082	5.96040552	2.52E-09	4.55E-08
ENSDARG00000028618	krt18b	5929.63117	0.703725604	0.11809649	5.95890388	2.54E-09	4.59E-08
ENSDARG00000015224	cd2ap	1971.76506	0.640391399	0.10746823	5.95889027	2.54E-09	4.59E-08
ENSDARG00000060518	pcsk5b	1566.85419	0.623957947	0.1047192	5.95839084	2.55E-09	4.60E-08
ENSDARG00000102403	cry2	1339.81837	0.738015239	0.12388098	5.95745419	2.56E-09	4.62E-08
ENSDARG00000097008	opn1mw1	13205.4726	-3.776566051	0.63395625	-5.95713992	2.57E-09	4.63E-08
ENSDARG00000012588	ptdss1a	1232.18991	0.680787737	0.11436458	5.95278482	2.64E-09	4.75E-08
ENSDARG00000035957	gmnn	311.18393	-1.118345507	0.18788951	-5.95214459	2.65E-09	4.76E-08
ENSDARG00000098263	CU929294.1	152.215299	-2.144691408	0.36033968	-5.95186015	2.65E-09	4.77E-08
ENSDARG00000043081	ctsz	2052.41907	0.819154176	0.13764843	5.95106098	2.66E-09	4.79E-08
ENSDARG00000013251	hsf4	87.2776624	-2.493475024	0.41906536	-5.9500862	2.68E-09	4.81E-08
ENSDARG00000096721	si:rp71-1c10.11	387.965824	-1.164712292	0.19573933	-5.95032339	2.68E-09	4.81E-08
ENSDARG00000025535	clint1a	2760.07983	0.507406495	0.08528409	5.94960313	2.69E-09	4.82E-08
ENSDARG00000060120	glyctk	569.842218	0.798595177	0.13425772	5.94822518	2.71E-09	4.86E-08
ENSDARG00000025147	cd63	2583.31813	0.828233126	0.13926196	5.94730347	2.73E-09	4.88E-08
ENSDARG00000068629	cd151l	993.627094	0.679863863	0.1143276	5.94662939	2.74E-09	4.90E-08
ENSDARG00000079738	znf219	1822.2225	-1.022651092	0.1720274	-5.94469886	2.77E-09	4.95E-08
ENSDARG00000005139	hhatlb	1966.29362	0.749494791	0.12607436	5.94486299	2.77E-09	4.95E-08

Table S2. DEGs of WT vs. *terfa-/-*

ENSDARG00000070494	pdgfra	4295.55327	-0.546980072	0.09203243	-5.94334074	2.79E-09	4.99E-08
ENSDARG00000089586	ncam3	134.172177	-1.549338319	0.2607918	-5.94090112	2.83E-09	5.06E-08
ENSDARG00000076952	fer1l4	310.236135	0.85875814	0.1445728	5.93997048	2.85E-09	5.08E-08
ENSDARG00000060323	exoc5	818.265317	0.811798099	0.13667226	5.93974303	2.85E-09	5.09E-08
ENSDARG00000006242	ptp4a1	7384.66768	-0.685059763	0.1153449	-5.93922878	2.86E-09	5.10E-08
ENSDARG00000051730	slc7a10b	218.34316	-1.314227886	0.22133266	-5.93779472	2.89E-09	5.14E-08
ENSDARG00000033029	pkd1b	853.435468	1.276560085	0.21506139	5.93579385	2.92E-09	5.20E-08
ENSDARG00000089162	afap1l1a	736.563123	0.82464972	0.13894107	5.93524794	2.93E-09	5.21E-08
ENSDARG00000099448	sh3d21	1626.65745	0.685362892	0.11548234	5.93478534	2.94E-09	5.22E-08
ENSDARG00000097547	AL954861.1	39.0688649	2.150976033	0.36278928	5.9289955	3.05E-09	5.41E-08
ENSDARG00000016818	abcg2d	94.486071	1.756455055	0.29626511	5.92865977	3.05E-09	5.41E-08
ENSDARG00000063297	abcb6a	458.544202	0.976860235	0.16477767	5.92835325	3.06E-09	5.42E-08
ENSDARG00000090106	slc17a7b	575.817577	-1.453945168	0.24528739	-5.92751697	3.08E-09	5.44E-08
ENSDARG00000034291	rpl37	15417.9052	0.653239907	0.11021897	5.92674651	3.09E-09	5.46E-08
ENSDARG00000001767	slc29a2	511.408212	-1.349672689	0.22771817	-5.92694339	3.09E-09	5.46E-08
ENSDARG00000055705	f5	1364.3219	1.373366484	0.23177506	5.92542835	3.11E-09	5.50E-08
ENSDARG00000070006	rcn1	1429.67774	0.604167365	0.10203114	5.92140151	3.19E-09	5.63E-08
ENSDARG00000100184	ly97.2	22.1169073	7.904575993	1.33565434	5.91813	3.26E-09	5.74E-08
ENSDARG00000041062	calb2a	3514.38558	-0.942062294	0.15919948	-5.91749616	3.27E-09	5.76E-08
ENSDARG00000042846	disp2	993.021007	-0.786609676	0.13299062	-5.91477542	3.32E-09	5.85E-08
ENSDARG00000041776	ndst3	903.533581	-0.722525036	0.12218308	-5.91346237	3.35E-09	5.90E-08
ENSDARG00000036279	lamc1	15809.9468	0.561685319	0.09500573	5.9121202	3.38E-09	5.94E-08
ENSDARG00000038018	prph2a	2395.45981	-2.960996767	0.5008782	-5.91161035	3.39E-09	5.95E-08
ENSDARG00000076673	dspb	5010.5365	0.775983665	0.13132558	5.9088537	3.44E-09	6.05E-08
ENSDARG00000011222	ccdc28b	328.949775	-0.950557959	0.16097636	-5.90495381	3.53E-09	6.19E-08
ENSDARG00000075054	rasgrf2a	353.00503	-1.608213915	0.27240758	-5.90370466	3.55E-09	6.23E-08
ENSDARG00000037738	fbxl3l	108.037522	-1.74003741	0.29480847	-5.90226396	3.59E-09	6.28E-08
ENSDARG00000099118	TRAPPC9	886.986147	0.754460856	0.12786902	5.90026287	3.63E-09	6.36E-08
ENSDARG00000102753	si:dkey-109l4.3	157.757607	-1.441403117	0.24430865	-5.89992668	3.64E-09	6.37E-08
ENSDARG00000058940	si:ch211-248e11.2	111.502136	1.731594999	0.29352687	5.89927259	3.65E-09	6.38E-08
ENSDARG00000070972	si:ch211-81a5.8	449.10176	-1.3582002	0.23022587	-5.89942469	3.65E-09	6.38E-08
ENSDARG00000091465	si:dkey-66a8.7	271.997647	0.893624479	0.15149912	5.89854575	3.67E-09	6.40E-08
ENSDARG00000093535	BX005309.1	779.830658	-0.762368566	0.12924338	-5.89870517	3.66E-09	6.40E-08
ENSDARG00000052895	htra3a	28.2051543	2.994182332	0.50764793	5.89814743	3.68E-09	6.41E-08
ENSDARG00000103743	si:dkeyp-72e1.9	352.863705	-1.347259064	0.22855982	-5.89455788	3.76E-09	6.54E-08
ENSDARG00000007988	masp2	628.115072	1.154515702	0.19585649	5.89470229	3.75E-09	6.54E-08
ENSDARG00000030357	zgc:66313	74.0303033	2.182247828	0.37030983	5.89303239	3.79E-09	6.60E-08
ENSDARG00000089441	si:ch211-105c13.3	2308.58221	0.775488648	0.13162158	5.89180479	3.82E-09	6.64E-08
ENSDARG00000056379	si:ch73-86n18.1	482.960105	1.019496625	0.17305292	5.89124211	3.83E-09	6.66E-08
ENSDARG00000098228	thrap3b	3982.22687	-0.479767872	0.08145291	-5.89012549	3.86E-09	6.70E-08
ENSDARG00000059836	ddit3	1296.90255	-0.610805064	0.10370348	-5.8899188	3.86E-09	6.71E-08
ENSDARG00000104246	exosc3	233.62114	1.912990005	0.32491363	5.88768773	3.92E-09	6.79E-08
ENSDARG00000039351	ccl19b	69.1961533	2.20823963	0.37508154	5.88735894	3.92E-09	6.80E-08
ENSDARG00000095432	BX323074.2	313.388119	-1.229655018	0.20889193	-5.88656081	3.94E-09	6.82E-08
ENSDARG00000010555	pdha1b	166.238765	-2.126460662	0.36122333	-5.88683095	3.94E-09	6.82E-08
ENSDARG00000002193	rho	34631.572	-2.54873553	0.43321579	-5.8832932	4.02E-09	6.96E-08
ENSDARG00000068910	nos1	977.399027	1.254643331	0.21329294	5.88225453	4.05E-09	7.00E-08
ENSDARG00000006288	prdm5	346.872763	0.883874676	0.15027489	5.88171904	4.06E-09	7.01E-08
ENSDARG00000040198	fstl5	3314.61918	-0.709516905	0.12074716	-5.87605466	4.20E-09	7.25E-08
ENSDARG00000103614	pemt	178.963042	1.172896873	0.19961139	5.87590145	4.21E-09	7.25E-08
ENSDARG00000030367	metrn	1004.18864	-0.824652749	0.14033933	-5.87613417	4.20E-09	7.25E-08
ENSDARG00000090722	C20H6orf58	2364.47232	1.003335222	0.17077145	5.87531014	4.22E-09	7.26E-08
ENSDARG00000021346	pdhb	8505.89925	0.555308273	0.09451153	5.87556145	4.21E-09	7.26E-08
ENSDARG00000101789	cyp1c2	311.765856	1.399265518	0.23817639	5.87491283	4.23E-09	7.28E-08
ENSDARG00000061413	sec23ip	1497.14207	0.622163885	0.10599289	5.86986441	4.36E-09	7.50E-08
ENSDARG00000057365	elovl8b	163.589173	1.607364589	0.27390701	5.86828575	4.40E-09	7.56E-08
ENSDARG00000094977	si:ch211-156j22.4	488.87815	-1.491785253	0.25423234	-5.86780279	4.42E-09	7.57E-08
ENSDARG00000060627	hip1ra	173.761718	1.079264456	0.18392088	5.86809109	4.41E-09	7.57E-08

Table S2. DEGs of WT vs. *terfa-/-*

ENSDARG00000070127	nfxl1	1112.11253	0.77804025	0.13267612	5.86420705	4.51E-09	7.74E-08
ENSDARG00000077596	phf24	1210.51842	-0.670543482	0.11435614	-5.86364222	4.53E-09	7.76E-08
ENSDARG00000088343	prrt4	165.437288	-1.247132636	0.21274289	-5.86215895	4.57E-09	7.82E-08
ENSDARG00000091699	capn2a	5328.16316	0.718540449	0.12259578	5.86105363	4.60E-09	7.87E-08
ENSDARG00000101645	myrfl	408.524377	1.074614646	0.18337557	5.86018424	4.62E-09	7.90E-08
ENSDARG00000091836	casp7	583.966835	-0.931342295	0.15898259	-5.85814007	4.68E-09	8.00E-08
ENSDARG00000097663	BX324206.3	321.948344	-1.060343045	0.18100843	-5.85797614	4.69E-09	8.00E-08
ENSDARG00000055226	slc7a7	606.070101	0.768797434	0.13125982	5.85706622	4.71E-09	8.03E-08
ENSDARG00000077308	gpr84	57.9002275	2.673433468	0.45646052	5.85687773	4.72E-09	8.03E-08
ENSDARG00000097247	BX322555.3	273.775359	1.572004696	0.26839822	5.85698634	4.71E-09	8.03E-08
ENSDARG00000104696	apooa	777.275759	0.650727011	0.11111837	5.85616074	4.74E-09	8.06E-08
ENSDARG00000101181	s100w	434.293431	0.936171393	0.15988222	5.85538137	4.76E-09	8.09E-08
ENSDARG00000074546	si:ch211-213a13.2	244.609417	1.325967552	0.22645448	5.85533811	4.76E-09	8.09E-08
ENSDARG00000074248	tmem237b	352.818203	-1.05396655	0.18004769	-5.85381889	4.80E-09	8.16E-08
ENSDARG00000087492	FO704810.1	459.049904	1.071437555	0.18307123	5.8525719	4.84E-09	8.22E-08
ENSDARG00000074519	BX248501.1	167.00674	-1.922343876	0.3284696	-5.85242559	4.84E-09	8.22E-08
ENSDARG00000011066	glra3	376.283219	-1.363145373	0.23295747	-5.85147744	4.87E-09	8.26E-08
ENSDARG00000015540	psen2	1108.38532	0.732312565	0.12518993	5.84961255	4.93E-09	8.35E-08
ENSDARG00000041623	mt2	934.919354	-1.103626717	0.18871946	-5.84797512	4.98E-09	8.42E-08
ENSDARG00000111788	BX511067.1	26.4884797	7.366434524	1.25996894	5.84652075	5.02E-09	8.49E-08
ENSDARG00000002696	gnb3b	9018.13563	-2.996801389	0.51258975	-5.84639348	5.02E-09	8.49E-08
ENSDARG00000025983	l3mbtl3	373.800315	1.480857368	0.25338158	5.84437659	5.08E-09	8.59E-08
ENSDARG00000055064	prdx5	3336.69792	0.639127613	0.10940108	5.84205936	5.16E-09	8.71E-08
ENSDARG00000026801	stau2	10369.7637	0.558507655	0.09563862	5.83977102	5.23E-09	8.82E-08
ENSDARG00000036875	rps12	3178.11626	1.88538643	0.32290444	5.83883705	5.26E-09	8.86E-08
ENSDARG00000111795	CR855311.6	53.4246503	-2.463273659	0.42189951	-5.83853163	5.27E-09	8.87E-08
ENSDARG00000044972	bcap31	2307.24588	0.575125045	0.09852712	5.83722603	5.31E-09	8.94E-08
ENSDARG00000015607	si:ch211-220f21.2	22.8323183	7.482228096	1.28215738	5.835655	5.36E-09	9.02E-08
ENSDARG00000100828	CU929150.2	29.6725985	2.696118401	0.4621031	5.8344521	5.40E-09	9.07E-08
ENSDARG00000017251	smtlb	76.9970636	-2.536064588	0.4346773	-5.83436165	5.40E-09	9.07E-08
ENSDARG00000094451	cfp	640.446956	-1.19018888	0.20400195	-5.83420355	5.40E-09	9.08E-08
ENSDARG00000105287	mpp1	3943.77246	0.741815136	0.12717501	5.83302618	5.44E-09	9.13E-08
ENSDARG00000004979	elovl5	571.60067	0.914033187	0.1567507	5.83112659	5.51E-09	9.23E-08
ENSDARG00000094983	ccl34a.3	10.7684537	7.862746489	1.34849455	5.83075883	5.52E-09	9.25E-08
ENSDARG00000069463	alox12	1481.66162	0.574606762	0.09855585	5.83026533	5.53E-09	9.27E-08
ENSDARG00000109686	si:ch211-59p23.1	42.8563572	2.909095705	0.49908798	5.82882341	5.58E-09	9.34E-08
ENSDARG00000020241	icmt	604.244869	-0.810912059	0.13920437	-5.82533487	5.70E-09	9.53E-08
ENSDARG00000043873	arid4a	2962.65301	-0.520423212	0.08937486	-5.82292611	5.78E-09	9.67E-08
ENSDARG00000093443	BX323559.4	40.7852068	2.741770016	0.47098256	5.82138331	5.84E-09	9.75E-08
ENSDARG00000074758	csde1	19992.6862	0.590706555	0.10149603	5.81999693	5.88E-09	9.82E-08
ENSDARG00000079056	si:ch211-194c3.5	3060.61509	-0.760214515	0.13061935	-5.82007588	5.88E-09	9.82E-08
ENSDARG00000111261	FO834850.1	377.332762	0.858083804	0.14744907	5.81952688	5.90E-09	9.84E-08
ENSDARG00000086222	nat16	2227.81076	-0.749768191	0.12884464	-5.81916465	5.91E-09	9.85E-08
ENSDARG00000104435	si:ch73-217b7.1	750.86598	-0.674716491	0.11594872	-5.8190941	5.92E-09	9.85E-08
ENSDARG00000104945	sncb	5651.55564	-0.857680024	0.14743845	-5.81720733	5.98E-09	9.96E-08
ENSDARG00000022689	itgb1b.2	998.86851	-0.834255341	0.14342347	-5.81672822	6.00E-09	9.98E-08
ENSDARG00000012341	capn9	1715.77532	0.64894493	0.11157141	5.81640856	6.01E-09	9.98E-08
ENSDARG00000010186	myo3a	436.300728	-0.997953468	0.17157359	-5.81647486	6.01E-09	9.98E-08
ENSDARG00000005762	col14a1a	1894.88813	0.701106372	0.1205832	5.81429579	6.09E-09	1.01E-07
ENSDARG00000095193	si:dkey-23c22.7	23.0817808	5.142428163	0.88536682	5.80824586	6.31E-09	1.05E-07
ENSDARG00000086881	ier2b	660.337362	-0.93094143	0.16028775	-5.80793879	6.32E-09	1.05E-07
ENSDARG00000031495	seta	7809.13495	-0.797017653	0.1372755	-5.80597155	6.40E-09	1.06E-07
ENSDARG00000006603	csrp1a	1326.87614	0.786224741	0.13545569	5.80429482	6.46E-09	1.07E-07
ENSDARG00000070846	dazap1	374.512757	-0.952996587	0.16420572	-5.80367471	6.49E-09	1.07E-07
ENSDARG00000095893	si:dkey-85n7.7	33.7566771	3.361829589	0.57947931	5.8014661	6.57E-09	1.09E-07
ENSDARG00000097973	si:ch1073-190k2.1	727.396804	12.71176784	2.19126607	5.80110649	6.59E-09	1.09E-07
ENSDARG00000011929	plp1b	309.332968	-1.511821631	0.26071952	-5.79865143	6.69E-09	1.10E-07
ENSDARG00000069767	nectin1a	327.81708	-1.407888752	0.24276022	-5.79950364	6.65E-09	1.10E-07

Table S2. DEGs of WT vs. *terfa-/-*

ENSDARG00000075314	zgc:174906	577.119903	-0.841567135	0.1451755	-5.79689489	6.76E-09	1.11E-07
ENSDARG00000071062	hps5	757.937038	0.882023796	0.15221135	5.79473088	6.84E-09	1.13E-07
ENSDARG00000069111	ikzf2	235.395942	1.01752277	0.17563792	5.79329788	6.90E-09	1.14E-07
ENSDARG00000004840	rassf1	872.877022	-0.727668056	0.12561865	-5.79267522	6.93E-09	1.14E-07
ENSDARG00000052746	grinab	2291.85713	0.668970232	0.11550974	5.79146172	6.98E-09	1.15E-07
ENSDARG00000040162	fgfbp3	1692.17363	-1.179773041	0.20370244	-5.79164911	6.97E-09	1.15E-07
ENSDARG00000017658	gmppb	2236.99165	0.611372276	0.10559642	5.78970672	7.05E-09	1.16E-07
ENSDARG00000042961	zgc:100920	643.541412	-0.968524465	0.16746246	-5.78353174	7.31E-09	1.20E-07
ENSDARG00000070781	gjd2b	270.770935	-1.366583625	0.2363736	-5.78145626	7.41E-09	1.21E-07
ENSDARG00000089372	clk4a	3608.61157	-0.548691011	0.09488497	-5.78269671	7.35E-09	1.21E-07
ENSDARG00000099236	10-Sep	3952.54279	0.591310776	0.10229392	5.78050758	7.45E-09	1.22E-07
ENSDARG00000058548	bves	994.84284	0.79462146	0.13750918	5.77867954	7.53E-09	1.23E-07
ENSDARG00000052405	pak6b	404.445885	1.124939804	0.19468531	5.77824714	7.55E-09	1.23E-07
ENSDARG00000070437	rpl22	11898.6355	0.615168057	0.10647077	5.77781151	7.57E-09	1.24E-07
ENSDARG00000074476	odam	118.630232	-2.519269805	0.43613336	-5.77637496	7.63E-09	1.25E-07
ENSDARG00000069254	chrm4a	521.362237	-0.852545167	0.14767039	-5.77329792	7.77E-09	1.27E-07
ENSDARG00000023814	dyrk1ab	1960.22843	-0.624160785	0.10812831	-5.77240861	7.81E-09	1.27E-07
ENSDARG00000013393	guca1b	44.0380388	-3.541017866	0.61334046	-5.77333162	7.77E-09	1.27E-07
ENSDARG00000092269	si:dkeyp-41f9.4	346.197178	-1.869802432	0.32384601	-5.77373926	7.75E-09	1.27E-07
ENSDARG00000014378	slc12a4	1154.20376	0.773956153	0.13410153	5.77141915	7.86E-09	1.28E-07
ENSDARG00000017441	mylz3	190303.636	0.732380915	0.12690342	5.77116749	7.87E-09	1.28E-07
ENSDARG00000087373	dnah5	469.917675	1.151953208	0.19962455	5.7705988	7.90E-09	1.28E-07
ENSDARG00000059363	tgfbr2a	225.723647	1.402542808	0.24301341	5.77146272	7.86E-09	1.28E-07
ENSDARG00000045626	nek8	202.720798	0.949453077	0.16450272	5.77165598	7.85E-09	1.28E-07
ENSDARG00000094889	cplx4c	292.533912	-1.561514893	0.27078192	-5.76668817	8.08E-09	1.31E-07
ENSDARG00000015554	zic2a	1943.32426	-0.735914617	0.12762305	-5.7663142	8.10E-09	1.31E-07
ENSDARG00000102245	tk1	934.827032	-0.982472595	0.17038518	-5.76618571	8.11E-09	1.31E-07
ENSDARG00000088069	CR759879.1	112.945823	1.99691936	0.3464238	5.76438279	8.20E-09	1.33E-07
ENSDARG00000044375	zgc:158291	1311.20688	-1.245232898	0.21605223	-5.76357344	8.24E-09	1.33E-07
ENSDARG00000098344	rab18b	1412.97284	0.724209206	0.12569463	5.76165573	8.33E-09	1.34E-07
ENSDARG00000076710	si:dkey-42l23.7	95.7968062	-2.012826407	0.34933394	-5.76189771	8.32E-09	1.34E-07
ENSDARG00000005908	clk4b	1255.96955	-0.737819563	0.12805695	-5.76165186	8.33E-09	1.34E-07
ENSDARG00000095557	BX664610.1	415.233068	-1.283097266	0.22265688	-5.76266628	8.28E-09	1.34E-07
ENSDARG00000105625	si:cabz01007812.1	200.049691	1.204932646	0.20918472	5.76013694	8.40E-09	1.36E-07
ENSDARG00000086654	cbln11	69.9536646	2.64268192	0.45889329	5.75881577	8.47E-09	1.36E-07
ENSDARG00000102047	mab21l1	4243.32026	-0.892365515	0.15495708	-5.75879135	8.47E-09	1.36E-07
ENSDARG00000088514	and1	23024.2152	1.017711913	0.17672253	5.75881258	8.47E-09	1.36E-07
ENSDARG00000077906	rnf165a	1387.4307	-0.668069763	0.11603978	-5.7572479	8.55E-09	1.38E-07
ENSDARG00000102585	fam160a1b	356.111069	-1.142115165	0.19845232	-5.75511121	8.66E-09	1.39E-07
ENSDARG00000029689	tkta	3718.46404	-1.098766583	0.1909012	-5.75568206	8.63E-09	1.39E-07
ENSDARG00000021265	mybpc2b	5362.80226	0.703063187	0.12218983	5.75386023	8.72E-09	1.40E-07
ENSDARG00000099764	CR855389.1	90.2609053	2.371088245	0.41225238	5.75154527	8.84E-09	1.42E-07
ENSDARG00000037925	rgs9a	421.966125	-3.40858235	0.59280513	-5.74992042	8.93E-09	1.43E-07
ENSDARG00000009753	sf3b6	1005.53014	-0.888069998	0.15447176	-5.7490769	8.97E-09	1.44E-07
ENSDARG00000005454	tacc3	1614.8777	-1.048570891	0.18240194	-5.74868267	8.99E-09	1.44E-07
ENSDARG00000034862	f7	916.058625	0.780409172	0.1357811	5.74755386	9.05E-09	1.45E-07
ENSDARG00000103543	gngt2b	9642.03129	-3.167785985	0.55120604	-5.74700883	9.08E-09	1.45E-07
ENSDARG00000015557	zgc:100829	1460.86767	0.601041445	0.10463116	5.74438273	9.23E-09	1.47E-07
ENSDARG00000102750	cdh1	3811.25027	0.865006137	0.15064176	5.74214047	9.35E-09	1.49E-07
ENSDARG00000005098	zgc:86764	548.910827	-1.121561636	0.19529839	-5.74281042	9.31E-09	1.49E-07
ENSDARG00000103324	si:ch211-127b6.2	26.209213	5.436527008	0.94710742	5.74013769	9.46E-09	1.51E-07
ENSDARG00000117241	CABZ01067657.1	60.9716142	2.521436424	0.43928763	5.73983026	9.48E-09	1.51E-07
ENSDARG00000024278	adh8b	3484.98941	-0.537449576	0.0936464	-5.73913764	9.52E-09	1.52E-07
ENSDARG00000104413	rogdi	1738.67228	-0.700333547	0.12205163	-5.73801073	9.58E-09	1.53E-07
ENSDARG00000038028	ndufa6	3895.96266	0.6804253	0.11863872	5.73527166	9.74E-09	1.55E-07
ENSDARG00000019081	DHDH	1271.52914	0.979093138	0.17070636	5.73554003	9.72E-09	1.55E-07
ENSDARG00000100374	txnrd3	4974.04887	0.47307526	0.08255518	5.73041275	1.00E-08	1.59E-07
ENSDARG00000114508	tmem101	502.564157	1.168376774	0.20388631	5.73053087	1.00E-08	1.59E-07

Table S2. DEGs of WT vs. *terfa-/-*

ENSDARG00000098652	pcdh11	1502.76133	-0.693940451	0.12114134	-5.72835376	1.01E-08	1.61E-07
ENSDARG00000004763	hhla2a.2	137.283401	-1.674438793	0.2924817	-5.72493526	1.03E-08	1.64E-07
ENSDARG00000103505	fbxl3a	1047.27529	-0.73926703	0.12918703	-5.72245565	1.05E-08	1.66E-07
ENSDARG00000004204	efhc2	97.12373	1.439992588	0.2516969	5.72113763	1.06E-08	1.67E-07
ENSDARG00000042545	sema3ga	714.827177	0.925815107	0.16179938	5.72199404	1.05E-08	1.67E-07
ENSDARG00000044328	ankrd46a	1334.23232	-0.58001317	0.10136635	-5.72195006	1.05E-08	1.67E-07
ENSDARG00000070621	FAM72B	519.834169	-0.96098096	0.16796722	-5.72124126	1.06E-08	1.67E-07
ENSDARG00000062865	mras	1159.13597	-0.672509567	0.11757825	-5.71967644	1.07E-08	1.69E-07
ENSDARG00000074563	gpr37a	328.704718	-1.349314862	0.23591873	-5.71940553	1.07E-08	1.69E-07
ENSDARG00000022309	dspa	6684.85811	0.640774748	0.11215214	5.71344206	1.11E-08	1.74E-07
ENSDARG00000053262	atp1b4	4093.89913	-0.671022601	0.11742998	-5.71423594	1.10E-08	1.74E-07
ENSDARG00000002494	itgb6	835.865058	0.592236859	0.10364835	5.71390523	1.10E-08	1.74E-07
ENSDARG00000101362	mibp	1062.74786	-0.860083853	0.15052706	-5.71381555	1.10E-08	1.74E-07
ENSDARG00000104497	pcdh1g29	396.818353	-1.476333682	0.25840909	-5.71316462	1.11E-08	1.75E-07
ENSDARG00000102415	scinla	7430.08144	-0.82784884	0.14493291	-5.71194536	1.12E-08	1.76E-07
ENSDARG00000063411	lrrc73	550.331343	-0.834394758	0.14609096	-5.71147426	1.12E-08	1.76E-07
ENSDARG00000007818	palm1b	2436.20515	-0.660916762	0.11580895	-5.70695764	1.15E-08	1.81E-07
ENSDARG00000052419	ankrd12	3978.21914	-0.565097641	0.09902897	-5.70638708	1.15E-08	1.81E-07
ENSDARG00000026348	csad	996.184762	1.061994516	0.18617139	5.70439174	1.17E-08	1.83E-07
ENSDARG00000037874	cyp3c3	31.4109738	4.775121162	0.83722854	5.7034859	1.17E-08	1.84E-07
ENSDARG00000075931	acsl5	753.591355	1.098550511	0.19264531	5.70245151	1.18E-08	1.85E-07
ENSDARG00000056151	tyrp1b	8068.22592	0.712437573	0.12496466	5.70111223	1.19E-08	1.86E-07
ENSDARG00000006598	sgk2b	816.572812	1.180038636	0.20697786	5.70127943	1.19E-08	1.86E-07
ENSDARG00000094176	cdkn2d	158.740831	-1.415172422	0.24826479	-5.70025416	1.20E-08	1.87E-07
ENSDARG00000089190	tanc1b	1453.24614	0.8523729	0.14959594	5.69783458	1.21E-08	1.90E-07
ENSDARG00000019969	smarcd3b	2311.52608	-0.595195124	0.10447697	-5.69690278	1.22E-08	1.91E-07
ENSDARG00000074056	kctd1	391.727949	-1.073412819	0.18843	-5.69661313	1.22E-08	1.91E-07
ENSDARG00000103981	bhlha9	314.911586	1.478460506	0.25956739	5.69586392	1.23E-08	1.92E-07
ENSDARG00000060354	samd7	566.969327	-2.333176686	0.4097002	-5.69483905	1.23E-08	1.93E-07
ENSDARG00000068969	zgc:153759	41.9471106	3.10420657	0.54522548	5.69343639	1.25E-08	1.94E-07
ENSDARG00000059097	nktr	2741.82317	-0.606978956	0.10661258	-5.69331486	1.25E-08	1.94E-07
ENSDARG00000094077	si:dkey-21e2.16	45.5081446	-3.498000374	0.61447524	-5.69266288	1.25E-08	1.95E-07
ENSDARG00000070261	crlf3	1439.02621	0.702489976	0.12343322	5.69125558	1.26E-08	1.96E-07
ENSDARG00000053315	tmprss3a	290.232517	2.78729024	0.48993307	5.68912453	1.28E-08	1.98E-07
ENSDARG00000022767	apobb.1	157462.635	0.81205254	0.14273416	5.68926569	1.28E-08	1.98E-07
ENSDARG00000077081	slc38a10	2098.27386	0.515136808	0.09054281	5.68942834	1.27E-08	1.98E-07
ENSDARG00000059357	sarnp	1293.74422	-0.656874537	0.11546811	-5.68879621	1.28E-08	1.98E-07
ENSDARG00000089486	cplx3b	398.88867	-1.338553467	0.23533569	-5.68784728	1.29E-08	1.99E-07
ENSDARG00000101201	si:dkey-207b20.2	16.7837304	7.766278966	1.36587026	5.68595657	1.30E-08	2.02E-07
ENSDARG00000087352	dnah2	334.747161	1.043478945	0.18357984	5.68406068	1.32E-08	2.04E-07
ENSDARG00000035308	grb10b	222.895405	1.458809817	0.2566531	5.68397503	1.32E-08	2.04E-07
ENSDARG00000013892	sorl1	3493.68354	0.537898569	0.09464364	5.68340982	1.32E-08	2.04E-07
ENSDARG00000075954	serpinh1a	1272.69113	0.797344002	0.14029416	5.68337255	1.32E-08	2.04E-07
ENSDARG00000089042	foxb1a	455.599214	-0.80665949	0.14194561	-5.68287726	1.32E-08	2.05E-07
ENSDARG00000096257	si:ch73-367p23.2	75773.5294	0.792294134	0.13949129	5.67988249	1.35E-08	2.08E-07
ENSDARG00000042970	gng8	98.2668653	-1.702724294	0.29989043	-5.67782147	1.36E-08	2.10E-07
ENSDARG00000021140	pabpc1b	4771.61004	0.542437579	0.09554146	5.67751	1.37E-08	2.11E-07
ENSDARG00000075043	si:dkeyp-123h10.2	667.675541	-0.866995489	0.15276303	-5.67542743	1.38E-08	2.13E-07
ENSDARG00000086615	CR846087.1	42.0348515	2.219170534	0.39112778	5.67377375	1.40E-08	2.15E-07
ENSDARG00000004712	tbr1b	762.030398	-0.915222224	0.16134804	-5.67234793	1.41E-08	2.17E-07
ENSDARG00000077176	zgc:174888	333.794222	1.122083271	0.19785431	5.67126024	1.42E-08	2.18E-07
ENSDARG00000088178	tp53inp2	935.505519	-1.125236083	0.19844801	-5.67018081	1.43E-08	2.19E-07
ENSDARG00000016607	echdc2	472.196108	0.822379475	0.14504945	5.66964903	1.43E-08	2.20E-07
ENSDARG00000039931	slc25a33	1343.6699	0.859967135	0.15172168	5.66805688	1.44E-08	2.22E-07
ENSDARG00000076264	zgc:195170	56.9105762	-2.746897524	0.48465568	-5.66772998	1.45E-08	2.22E-07
ENSDARG00000012405	col1a1a	170262.545	0.764608092	0.1349199	5.66712619	1.45E-08	2.23E-07
ENSDARG00000104457	si:dkeyp-72e1.7	298.574203	-1.104482051	0.19490073	-5.66689548	1.45E-08	2.23E-07
ENSDARG00000115504	CABZ01080056.1	174.573595	1.254004314	0.22132813	5.66581547	1.46E-08	2.24E-07

Table S2. DEGs of WT vs. *terfa-/-*

ENSDARG00000009133	myo1eb	1872.79055	0.573326668	0.10120164	5.6651913	1.47E-08	2.25E-07
ENSDARG00000058693	cast	8416.76796	0.595983464	0.10526435	5.66177878	1.50E-08	2.29E-07
ENSDARG00000058039	bhlhe22	1544.01473	-1.322424971	0.2336054	-5.66093487	1.51E-08	2.30E-07
ENSDARG00000045164	nptx2b	84.7368229	-3.045438476	0.53810173	-5.65959614	1.52E-08	2.32E-07
ENSDARG00000117808	CABZ01068366.1	154.26918	1.968391371	0.34788108	5.65823061	1.53E-08	2.33E-07
ENSDARG00000042816	mmp9	772.593481	3.63556681	0.64285161	5.65537487	1.56E-08	2.37E-07
ENSDARG00000117527	CABZ01079873.1	18.7382552	7.301690219	1.29113965	5.65522885	1.56E-08	2.37E-07
ENSDARG00000091349	plekhd1	107.46606	-1.865281286	0.32994638	-5.65328615	1.57E-08	2.40E-07
ENSDARG00000039430	msl2b	1447.19195	-0.650293446	0.11511027	-5.64930881	1.61E-08	2.45E-07
ENSDARG00000030961	ak8	73.9416016	1.855858956	0.32856967	5.64829668	1.62E-08	2.46E-07
ENSDARG00000034777	txn2	2030.47026	0.627659872	0.11111637	5.64867148	1.62E-08	2.46E-07
ENSDARG00000020473	hddc3	518.585663	-0.924617047	0.16378317	-5.64537274	1.65E-08	2.51E-07
ENSDARG00000101954	hoxc6b	203.506906	-1.22599566	0.21737342	-5.64004394	1.70E-08	2.58E-07
ENSDARG00000057519	pcdh1g9	569.451514	-1.157028653	0.20515022	-5.63990946	1.70E-08	2.58E-07
ENSDARG00000105549		126.179514	-1.830928222	0.32466247	-5.63948218	1.71E-08	2.59E-07
ENSDARG00000054578	arl6ip1	4161.53689	0.482959985	0.08565045	5.63873265	1.71E-08	2.60E-07
ENSDARG00000035634	rfc5	1000.73602	-0.641167845	0.11376146	-5.63607239	1.74E-08	2.63E-07
ENSDARG00000043795	arhgdia	6866.08494	0.524689995	0.09308516	5.6366664	1.73E-08	2.63E-07
ENSDARG00000090764	bcas3	983.456485	0.595655971	0.10567647	5.63659965	1.73E-08	2.63E-07
ENSDARG00000092947	cyt1	148986.178	0.659717444	0.11707053	5.63521362	1.75E-08	2.64E-07
ENSDARG00000012199	gpt2	1156.7107	-0.638629296	0.11335057	-5.63410735	1.76E-08	2.66E-07
ENSDARG00000021149	cbr1l	1546.25415	0.711246256	0.12630724	5.63108082	1.79E-08	2.71E-07
ENSDARG00000059177	tax1bp3	1094.32968	0.817003001	0.14512314	5.62972242	1.80E-08	2.73E-07
ENSDARG00000018025	tbx2a	1305.3341	-0.705561588	0.12534784	-5.62882928	1.81E-08	2.74E-07
ENSDARG00000056525	bcar1	2167.06325	0.562365284	0.09990754	5.62885719	1.81E-08	2.74E-07
ENSDARG00000032631	ltb4r	59.5421719	1.894608534	0.33666831	5.6275227	1.83E-08	2.76E-07
ENSDARG00000062147	otc	158.780161	-1.413352411	0.25117988	-5.62685362	1.84E-08	2.76E-07
ENSDARG00000057767	nudt4a	1036.42418	0.842641603	0.14984966	5.6232467	1.87E-08	2.82E-07
ENSDARG00000011989	crx	2688.53353	-2.156529121	0.38355758	-5.62243903	1.88E-08	2.83E-07
ENSDARG00000070427	s100v1	1520.30079	0.650307512	0.11565584	5.62278142	1.88E-08	2.83E-07
ENSDARG00000098853	ehd1a	2409.47353	0.718502294	0.12780627	5.62180774	1.89E-08	2.84E-07
ENSDARG00000032885	si:ch211-226m7.4	83.5286737	1.804451219	0.32098405	5.62162264	1.89E-08	2.84E-07
ENSDARG00000089616	coro7	753.269915	0.79845298	0.14210042	5.61893486	1.92E-08	2.88E-07
ENSDARG00000087188	nfil3-6	391.751266	1.042104923	0.18545603	5.61914811	1.92E-08	2.88E-07
ENSDARG00000053624	csf1rb	95.3927472	-1.555743747	0.27690759	-5.6182778	1.93E-08	2.89E-07
ENSDARG00000028478	si:ch211-173n18.3	45.7103739	2.343421207	0.41719991	5.61702232	1.94E-08	2.91E-07
ENSDARG00000018891	rnaseh2a	746.314103	-0.783809454	0.13953741	-5.6171996	1.94E-08	2.91E-07
ENSDARG00000070408	ccnd2b	357.921986	0.932831489	0.16608654	5.61653881	1.95E-08	2.92E-07
ENSDARG00000070656	si:ch211-69g19.2	1953.0049	-1.116417771	0.19879196	-5.61601072	1.95E-08	2.92E-07
ENSDARG00000021163	thrb	1293.63698	-0.674777546	0.12014324	-5.6164421	1.95E-08	2.92E-07
ENSDARG00000052960	nppa	685.746431	0.98532496	0.17547414	5.61521451	1.96E-08	2.93E-07
ENSDARG00000003429	hnrnpdl	3714.83974	-0.659128087	0.11738535	-5.61507986	1.96E-08	2.93E-07
ENSDARG00000100288	impg2b	64.2105574	-2.230195716	0.39731923	-5.6131079	1.99E-08	2.96E-07
ENSDARG00000076241	txlnbb	1651.03049	0.740496971	0.13196266	5.61141278	2.01E-08	2.99E-07
ENSDARG00000002768	pvalb2	208036.285	0.810746611	0.14449049	5.61107246	2.01E-08	3.00E-07
ENSDARG00000027322	lin7c	2802.60932	-0.572695849	0.10220771	-5.60325504	2.10E-08	3.13E-07
ENSDARG00000089706	si:ch211-276a23.5	91.2447082	1.554706016	0.27757895	5.60095075	2.13E-08	3.17E-07
ENSDARG00000095930	myha	794.524682	1.203516687	0.21488825	5.60066304	2.14E-08	3.17E-07
ENSDARG00000004023	isl1	2612.95067	-1.071949043	0.19139487	-5.6007198	2.13E-08	3.17E-07
ENSDARG00000030408	rps26l	18561.0238	0.63696253	0.11372331	5.60098485	2.13E-08	3.17E-07
ENSDARG00000079780	hnrnpul1l	10677.9544	-0.734199052	0.13118184	-5.59680421	2.18E-08	3.24E-07
ENSDARG00000029124	adamts15a	1025.30998	0.864952386	0.1546022	5.59469645	2.21E-08	3.28E-07
ENSDARG00000062510	bcl11ba	2918.561	-0.974898581	0.17424841	-5.59487793	2.21E-08	3.28E-07
ENSDARG00000078654	tpx2	2449.16839	-0.990618309	0.17709201	-5.59380583	2.22E-08	3.29E-07
ENSDARG00000101766	ptmab	36266.6771	-0.874758929	0.15639087	-5.59341421	2.23E-08	3.30E-07
ENSDARG00000100743	si:dkey-190j3.4	17.595615	4.329915665	0.77434729	5.59169729	2.25E-08	3.33E-07
ENSDARG00000114472	ctxn3	104.000186	1.641299673	0.2935305	5.59158131	2.25E-08	3.33E-07
ENSDARG00000014840	prph2b	3029.21038	-3.400545828	0.6082332	-5.59085865	2.26E-08	3.34E-07

Table S2. DEGs of WT vs. *terfa-/-*

ENSDARG00000098695	vclb	2645.66351	0.599235073	0.10720713	5.58950749	2.28E-08	3.36E-07
ENSDARG00000104023	mag	795.949027	-1.195981263	0.21407576	-5.58671974	2.31E-08	3.42E-07
ENSDARG00000028066	diras1a	2069.39559	-0.725838909	0.12992372	-5.58665417	2.31E-08	3.42E-07
ENSDARG00000008034	skib	1437.98189	-0.514737491	0.0921383	-5.58657465	2.32E-08	3.42E-07
ENSDARG00000074839	impg1b	219.47009	-1.901619807	0.34042645	-5.58599309	2.32E-08	3.43E-07
ENSDARG00000099846	si:ch211-154o6.3	492.196892	0.822413893	0.14727751	5.58411054	2.35E-08	3.45E-07
ENSDARG00000100093	ppp2r2ca	2062.59276	-0.799735679	0.14319841	-5.58480834	2.34E-08	3.45E-07
ENSDARG00000103011	CABZ01090373.1	989.388462	0.723146044	0.12948906	5.5846111	2.34E-08	3.45E-07
ENSDARG00000060841	pik3c2a	2410.7074	0.567500595	0.10162171	5.5844424	2.34E-08	3.45E-07
ENSDARG00000096950	BX927244.1	20.4651905	-4.332740709	0.77587885	-5.58430059	2.35E-08	3.45E-07
ENSDARG00000006487	anp32a	9199.83791	-0.658369003	0.11790886	-5.58371084	2.35E-08	3.46E-07
ENSDARG00000055874	cpe	13001.9356	-0.599176747	0.10731954	-5.58310951	2.36E-08	3.47E-07
ENSDARG00000100223	gpa33a	3112.98387	0.7210687	0.12917135	5.58226508	2.37E-08	3.48E-07
ENSDARG00000005840	got2b	5212.72237	0.461800329	0.08274957	5.58069743	2.40E-08	3.51E-07
ENSDARG00000014479	ptf1a	86.5650137	-1.823908353	0.32681952	-5.58078152	2.39E-08	3.51E-07
ENSDARG00000020901	gabrp	658.835934	-0.705906895	0.12648331	-5.58102792	2.39E-08	3.51E-07
ENSDARG00000100466	nrl	86.7962968	-2.545148826	0.45629631	-5.57784228	2.44E-08	3.56E-07
ENSDARG00000098345	mamdc2a	410.031525	-0.947108184	0.16984051	-5.57645628	2.45E-08	3.59E-07
ENSDARG00000087084	hcar1-4	57.8325303	2.215070992	0.39724165	5.57612978	2.46E-08	3.60E-07
ENSDARG00000075500	smim1	54.2053573	1.957752634	0.35115644	5.57515792	2.47E-08	3.61E-07
ENSDARG00000079985	nrip2	822.680408	-0.931662793	0.16711713	-5.57490888	2.48E-08	3.62E-07
ENSDARG00000076958	jac8	21.3586966	5.655468517	1.01454343	5.57439766	2.48E-08	3.62E-07
ENSDARG00000031981	pcbd1	157.280072	1.432953405	0.25706052	5.5743814	2.48E-08	3.62E-07
ENSDARG00000088638	BX927260.1	108.71082	-2.33599718	0.4190395	-5.57464677	2.48E-08	3.62E-07
ENSDARG00000100131	cdh29	87.0813264	2.011495809	0.36087071	5.57400692	2.49E-08	3.63E-07
ENSDARG00000095533	si:ch211-198c19.3	141.056284	2.192370944	0.39332114	5.57399725	2.49E-08	3.63E-07
ENSDARG00000103971	si:ch73-173p19.2	81.7681573	-2.053825983	0.36849599	-5.57353692	2.50E-08	3.63E-07
ENSDARG00000071475	aox5	1520.46571	0.789870832	0.14171136	5.57380029	2.49E-08	3.63E-07
ENSDARG00000088202	sh3d19	809.690204	0.684221978	0.12278656	5.57244996	2.51E-08	3.65E-07
ENSDARG00000102128	eps8a	1322.23808	-0.729334199	0.13097955	-5.56830592	2.57E-08	3.73E-07
ENSDARG00000032261	rngtt	1035.4334	-0.681359148	0.12235462	-5.56872429	2.57E-08	3.73E-07
ENSDARG00000071570	si:dkeyp-87e7.4	1155.84808	-0.685496941	0.12315547	-5.56611025	2.60E-08	3.78E-07
ENSDARG00000105829	CR788316.4	136.076017	2.039083946	0.36640867	5.56505383	2.62E-08	3.80E-07
ENSDARG00000033382	grifin	311.934342	-1.628563568	0.29264333	-5.56501174	2.62E-08	3.80E-07
ENSDARG00000008740	esf1	857.243749	0.757276679	0.1360776	5.5650355	2.62E-08	3.80E-07
ENSDARG00000113107	CABZ01085275.1	45.0253495	2.059128394	0.37005282	5.56441755	2.63E-08	3.81E-07
ENSDARG00000030107	si:ch211-207i1.2	2188.32695	-0.736760291	0.13241134	-5.56417831	2.63E-08	3.81E-07
ENSDARG00000042872	zdhhc8a	599.380221	-1.476769594	0.26544901	-5.56328918	2.65E-08	3.83E-07
ENSDARG00000103119	si:dkeyp-33c10.7	38.1608455	3.108630391	0.55894604	5.56159307	2.67E-08	3.86E-07
ENSDARG00000038428	sulf1	2585.9511	0.544018199	0.09785891	5.5592098	2.71E-08	3.91E-07
ENSDARG00000071213	rgl3a	612.6226	0.877343693	0.15786709	5.55748328	2.74E-08	3.95E-07
ENSDARG00000023771	gabrb3	1003.99365	-1.1217306	0.20184212	-5.55746541	2.74E-08	3.95E-07
ENSDARG00000099186	slc1a5	2886.2557	0.544138409	0.09793442	5.55615064	2.76E-08	3.98E-07
ENSDARG00000097824	smco4	585.390852	0.834257231	0.150192	5.55460489	2.78E-08	4.01E-07
ENSDARG00000021997	cfap206	60.1593524	1.892937795	0.34086543	5.55332879	2.80E-08	4.03E-07
ENSDARG00000042552	cacna1sb	2633.24541	0.944397011	0.17005845	5.55336709	2.80E-08	4.03E-07
ENSDARG00000093639	BX537282.1	10.8665224	8.279374549	1.49145102	5.55122121	2.84E-08	4.08E-07
ENSDARG00000092521	jac1	43.7082822	3.204061332	0.57725483	5.55051454	2.85E-08	4.09E-07
ENSDARG00000039563	loxl3b	2022.48453	0.534028628	0.09625549	5.54803311	2.89E-08	4.15E-07
ENSDARG00000105450	si:ch211-63p21.1	466.820898	-1.264552018	0.22798386	-5.54667355	2.91E-08	4.18E-07
ENSDARG00000024546	pla2g4aa	452.923752	1.069521795	0.19285625	5.54569437	2.93E-08	4.20E-07
ENSDARG00000057661	aldoca	267.965897	-1.635584471	0.29510759	-5.54233283	2.98E-08	4.28E-07
ENSDARG00000104678	CU467646.7	404.693476	0.830322791	0.14984735	5.54112415	3.01E-08	4.31E-07
ENSDARG00000025187	six6a	116.505559	-1.35064984	0.24374844	-5.54116305	3.00E-08	4.31E-07
ENSDARG00000044776	clic3	79.3279672	1.58907262	0.28685604	5.5396171	3.03E-08	4.34E-07
ENSDARG00000099406	lrit1b	1177.14943	-2.251232582	0.40671918	-5.53510301	3.11E-08	4.45E-07
ENSDARG00000061099	nfasca	2446.26864	-0.567874247	0.10262029	-5.53374231	3.13E-08	4.48E-07
ENSDARG00000075859	mcf2la	2125.80215	-0.544888774	0.09848304	-5.53281846	3.15E-08	4.50E-07

Table S2. DEGs of WT vs. *terfa-/-*

ENSDARG00000038891	AL954146.1	69.7469113	-2.48374135	0.44890032	-5.53294631	3.15E-08	4.50E-07
ENSDARG00000060072	abi3a	276.610593	-1.06178284	0.1920348	-5.52911687	3.22E-08	4.59E-07
ENSDARG00000036057	tradd	310.156676	0.928612281	0.16802113	5.52675904	3.26E-08	4.65E-07
ENSDARG00000062338	zeb2a	3988.72099	-0.726338847	0.13144574	-5.52576923	3.28E-08	4.68E-07
ENSDARG00000101060	gcna	189.147291	-1.155612675	0.20919389	-5.5241224	3.31E-08	4.72E-07
ENSDARG00000034855	ccnb3	427.596991	-1.311438195	0.2374953	-5.52195441	3.35E-08	4.77E-07
ENSDARG00000021366	fbp1a	724.719233	0.99857908	0.18084094	5.52186391	3.35E-08	4.77E-07
ENSDARG00000055813	si:dkey-225f5.5	84.4879836	1.723559898	0.31212809	5.5219635	3.35E-08	4.77E-07
ENSDARG00000040942	pnp6	447.083568	-1.054928129	0.19105165	-5.52169077	3.36E-08	4.77E-07
ENSDARG00000062137	stag1b	2795.58492	-0.733152004	0.13277314	-5.52183978	3.35E-08	4.77E-07
ENSDARG00000102227	CR450749.3	24.2640951	3.646420243	0.66047084	5.52094056	3.37E-08	4.78E-07
ENSDARG00000063223	arl14	141.450289	1.829650611	0.33137922	5.52131977	3.36E-08	4.78E-07
ENSDARG00000098360	cyp19a1b	34.5829591	-2.86665701	0.51922036	-5.52107973	3.37E-08	4.78E-07
ENSDARG00000042529	gnat2	8633.22159	-2.907288942	0.52664476	-5.52039847	3.38E-08	4.80E-07
ENSDARG00000090890	cmklr1	102.794249	1.762570157	0.31932381	5.51969544	3.40E-08	4.81E-07
ENSDARG00000026281	lyrm1	111.36041	1.659811962	0.300736	5.51916617	3.41E-08	4.82E-07
ENSDARG00000079795	CABZ01033206.1	329.154816	1.021071462	0.18502329	5.51861055	3.42E-08	4.84E-07
ENSDARG00000015790	atp1b3a	4406.15909	-0.429206353	0.0777995	-5.51682654	3.45E-08	4.88E-07
ENSDARG00000003061	cd276	1048.12692	0.618207052	0.11207704	5.51591189	3.47E-08	4.91E-07
ENSDARG00000031775	ube2s	1302.08742	-0.813997826	0.14761928	-5.51417008	3.50E-08	4.95E-07
ENSDARG00000035253	npr3	1038.55576	0.941666683	0.17080836	5.51300117	3.53E-08	4.98E-07
ENSDARG00000074137	c2cd3	489.352976	-0.754886782	0.13694151	-5.51247584	3.54E-08	4.99E-07
ENSDARG00000075608	mical2a	298.01314	0.959418233	0.17406303	5.51190133	3.55E-08	5.01E-07
ENSDARG00000097692	si:ch211-116o3.5	612.637716	-0.620765309	0.11268625	-5.50879356	3.61E-08	5.09E-07
ENSDARG00000104416	si:dkey-16p6.1	17.6522019	3.507311819	0.63688327	5.50699317	3.65E-08	5.14E-07
ENSDARG00000043509	rpl11	24869.5467	0.584640743	0.1062244	5.5038272	3.72E-08	5.23E-07
ENSDARG00000013845	ttc14	1241.34576	-0.833573024	0.15145557	-5.50374622	3.72E-08	5.23E-07
ENSDARG00000061044	si:dkey-72l14.3	188.647517	-1.192981148	0.21679617	-5.50277776	3.74E-08	5.26E-07
ENSDARG00000087663	si:ch211-248l17.3	198.599739	-2.146456852	0.39020929	-5.50078364	3.78E-08	5.32E-07
ENSDARG00000013415	lmna	1460.04776	0.695137034	0.12639224	5.4998395	3.80E-08	5.34E-07
ENSDARG00000077788	arhgef10	846.298518	0.758696979	0.13794998	5.49979763	3.80E-08	5.34E-07
ENSDARG00000053563	ms4a17a.12	35.8016055	3.051946227	0.55512031	5.49781046	3.85E-08	5.40E-07
ENSDARG00000094282	dnah11	211.533692	1.078669867	0.19630646	5.49482615	3.91E-08	5.49E-07
ENSDARG00000071150	cica	3481.11316	0.908296036	0.16530974	5.49451025	3.92E-08	5.49E-07
ENSDARG00000071454	crp1	47.1082071	-2.214783968	0.40308294	-5.49461105	3.92E-08	5.49E-07
ENSDARG00000037747	fscn1b	938.915468	-0.663374763	0.12074112	-5.49419095	3.93E-08	5.50E-07
ENSDARG00000058094	ciarta	2642.83299	0.660298717	0.12018438	5.49404794	3.93E-08	5.50E-07
ENSDARG00000104713	zgc:165653	635.624316	1.138947384	0.20734799	5.49292709	3.95E-08	5.53E-07
ENSDARG00000076928	tet2	3477.20527	-0.608308429	0.11075963	-5.49214957	3.97E-08	5.55E-07
ENSDARG00000022466	clcn5b	443.536997	0.797189747	0.14522974	5.48916337	4.04E-08	5.64E-07
ENSDARG00000021987	plecb	13593.8891	0.757406194	0.13799801	5.48852977	4.05E-08	5.66E-07
ENSDARG00000053232	itgb1b.1	3412.30415	0.747767805	0.13624913	5.48823895	4.06E-08	5.67E-07
ENSDARG00000077764	faah	268.402954	0.930011571	0.16948661	5.48722754	4.08E-08	5.69E-07
ENSDARG00000055177	pxdc1a	100.449837	-1.489719403	0.27155038	-5.48597793	4.11E-08	5.73E-07
ENSDARG00000013804	capns1b	3580.52517	0.644244835	0.11744508	5.48549893	4.12E-08	5.74E-07
ENSDARG00000105137	zgc:173705	24.9280045	7.068969042	1.28881174	5.48487327	4.14E-08	5.76E-07
ENSDARG00000043154	ucp2	7007.33956	0.82844617	0.15104454	5.48478062	4.14E-08	5.76E-07
ENSDARG00000026875	tlcd3ba	851.897004	-0.789633427	0.14397623	-5.48447097	4.15E-08	5.77E-07
ENSDARG00000078001	kbtbd7	194.991581	-1.099435792	0.20048095	-5.48399127	4.16E-08	5.78E-07
ENSDARG00000039517	c8b	1491.09868	0.663405567	0.12099857	5.48275524	4.19E-08	5.82E-07
ENSDARG00000061143	tapt1b	750.211376	-0.656719453	0.11980124	-5.48174183	4.21E-08	5.85E-07
ENSDARG00000101094	ATP11B	1875.76673	0.546571378	0.09973491	5.48024164	4.25E-08	5.89E-07
ENSDARG00000007955	iars1	4486.57496	0.690146881	0.12594064	5.47993786	4.25E-08	5.90E-07
ENSDARG00000069296	moxd1l	192.830277	-1.292243701	0.2358844	-5.47829232	4.29E-08	5.95E-07
ENSDARG00000061941	trpv4	527.882536	0.733381956	0.13388979	5.47750473	4.31E-08	5.97E-07
ENSDARG00000030490	sptb	4760.94584	0.629361599	0.11490015	5.47746558	4.31E-08	5.97E-07
ENSDARG00000100578	si:dkeyp-90h9.1	47.3581121	3.235292266	0.59067582	5.47727221	4.32E-08	5.98E-07
ENSDARG00000008732	zgc:66479	1887.10511	0.740033741	0.13511869	5.47691617	4.33E-08	5.99E-07

Table S2. DEGs of WT vs. *terfa-/-*

ENSDARG00000107353	BX957322.3	354.772379	-1.779962539	0.32507875	-5.47548112	4.36E-08	6.03E-07
ENSDARG00000045574	lrmp	554.082687	-2.325269551	0.42479968	-5.47380251	4.40E-08	6.09E-07
ENSDARG00000053820	pcmtd2a	255.033163	-1.182836546	0.21614664	-5.47237998	4.44E-08	6.13E-07
ENSDARG00000077569	lrrn3a	493.008524	-0.758676974	0.13864874	-5.47193549	4.45E-08	6.14E-07
ENSDARG00000079201	tshz2	1511.38982	-0.531321438	0.09710827	-5.47143359	4.46E-08	6.16E-07
ENSDARG00000020656	prkg1a	481.112183	1.009295882	0.18452621	5.46966124	4.51E-08	6.22E-07
ENSDARG00000027582	angptl7	2224.88432	0.920595823	0.16834839	5.46839709	4.54E-08	6.25E-07
ENSDARG00000056307	znf706	2740.78523	0.63788887	0.1166504	5.46838132	4.54E-08	6.25E-07
ENSDARG00000087956	she	395.55526	0.925722105	0.16929751	5.46801958	4.55E-08	6.26E-07
ENSDARG00000103935	rab27a	787.304551	0.878867275	0.16072347	5.46819513	4.55E-08	6.26E-07
ENSDARG00000087779	pum3	1298.14556	-0.623165731	0.11396835	-5.46788428	4.55E-08	6.26E-07
ENSDARG00000086103	slc37a1	497.201408	0.820013519	0.15003992	5.4653024	4.62E-08	6.35E-07
ENSDARG00000062959	zgc:158785	135.124667	-1.971511145	0.36079145	-5.4644065	4.64E-08	6.37E-07
ENSDARG00000061587	ctdspl2a	1805.42318	-0.5426712	0.09930563	-5.46465699	4.64E-08	6.37E-07
ENSDARG00000011672	sema3b	1903.38938	0.458430935	0.08389888	5.46408895	4.65E-08	6.38E-07
ENSDARG00000068261	pros1	919.584398	0.74745345	0.13685152	5.46178419	4.71E-08	6.46E-07
ENSDARG00000017107	nr2e1	310.744645	-1.253108753	0.22942659	-5.46191596	4.71E-08	6.46E-07
ENSDARG00000011533	sema6dl	2110.56464	-0.595073522	0.10895284	-5.46175316	4.71E-08	6.46E-07
ENSDARG00000104647	surf4	1891.77837	0.573848513	0.10508793	5.46065098	4.74E-08	6.49E-07
ENSDARG00000061576	gldn	240.539568	-1.104435162	0.20224552	-5.46086343	4.74E-08	6.49E-07
ENSDARG00000090543	tmem138	162.494202	-1.372071618	0.25127306	-5.46048051	4.75E-08	6.49E-07
ENSDARG00000016538	zgc:55888	35.6448299	3.888044959	0.71218247	5.45933818	4.78E-08	6.53E-07
ENSDARG00000053364	pax10	462.950346	-1.648563032	0.30199623	-5.4588861	4.79E-08	6.54E-07
ENSDARG00000074747	brwd1	2250.10012	-0.511516825	0.09370209	-5.45896914	4.79E-08	6.54E-07
ENSDARG00000094946	si:ch211-132b12.2	118.599123	1.491210541	0.27321565	5.45799828	4.82E-08	6.57E-07
ENSDARG00000030733	b3gat2	627.66866	-0.708149172	0.1297624	-5.45727567	4.83E-08	6.59E-07
ENSDARG00000116743	rnps1	118.875275	-1.757807452	0.32216626	-5.45621219	4.86E-08	6.62E-07
ENSDARG00000001686	uckl1a	129.268852	-1.423272201	0.26084834	-5.45632064	4.86E-08	6.62E-07
ENSDARG00000022727	epha2b	1710.1316	0.702197245	0.12876395	5.4533684	4.94E-08	6.72E-07
ENSDARG00000099428	si:dkey-15h8.17	155.1516	1.756737615	0.32213465	5.45342643	4.94E-08	6.72E-07
ENSDARG00000117504	CABZ01072084.1	222.936112	0.891739487	0.16353384	5.45293543	4.95E-08	6.74E-07
ENSDARG00000010420	ndrg1b	1045.1632	-2.549582909	0.46759009	-5.45260251	4.96E-08	6.75E-07
ENSDARG00000055455	gpm6aa	22333.0526	-0.768388379	0.1409416	-5.45182121	4.99E-08	6.77E-07
ENSDARG00000076320	ano9a	259.78262	0.867217331	0.15909524	5.45093199	5.01E-08	6.80E-07
ENSDARG00000094400	si:dkey-199f5.6	27.0747881	-2.9777595	0.54634545	-5.45032364	5.03E-08	6.82E-07
ENSDARG00000098398	CABZ01079480.1	172.55133	1.159243477	0.21273556	5.44922284	5.06E-08	6.86E-07
ENSDARG00000101900	xrn2	3115.23563	-0.542733272	0.09962042	-5.44801225	5.09E-08	6.90E-07
ENSDARG00000063631	VIT	5835.69296	0.551086495	0.10116105	5.44761524	5.10E-08	6.91E-07
ENSDARG00000060094	ptgis	294.87536	0.891688267	0.16371408	5.44661935	5.13E-08	6.95E-07
ENSDARG00000017880	kcnip3b	117.232945	-1.410387117	0.25897432	-5.44605014	5.15E-08	6.97E-07
ENSDARG00000046002	necap2	1880.50539	0.613484941	0.11269363	5.44382966	5.21E-08	7.05E-07
ENSDARG00000073918	rnf41l	162.207798	-1.402859086	0.25776347	-5.44242793	5.26E-08	7.10E-07
ENSDARG00000030933	ksr1b	736.41375	0.621325473	0.11419444	5.44094355	5.30E-08	7.16E-07
ENSDARG00000021794	hmmr	1087.89071	-1.214728412	0.22333793	-5.43897043	5.36E-08	7.23E-07
ENSDARG00000070174	PLEKHH3	1533.9918	0.637552861	0.11728789	5.43579439	5.46E-08	7.36E-07
ENSDARG00000011909	itpr2	978.252124	0.791601036	0.1456269	5.43581614	5.45E-08	7.36E-07
ENSDARG00000052494	pcdh18b	3329.89514	-0.525658939	0.09671368	-5.43520777	5.47E-08	7.38E-07
ENSDARG00000018165	depdc1a	497.445256	-1.223040553	0.22513978	-5.43236087	5.56E-08	7.49E-07
ENSDARG00000041072	mier1a	607.77937	-0.903997689	0.16644066	-5.43135131	5.59E-08	7.53E-07
ENSDARG00000003902	ctsl.1	312.404182	1.265925704	0.23307415	5.43142893	5.59E-08	7.53E-07
ENSDARG00000089749	aqp8b	420.70948	0.909577957	0.16751159	5.42994042	5.64E-08	7.58E-07
ENSDARG00000032865	pacsin1a	3565.6154	-0.705339385	0.12990174	-5.4297916	5.64E-08	7.58E-07
ENSDARG00000097504	CR626907.3	852.624723	1.270160228	0.23403443	5.42723672	5.72E-08	7.69E-07
ENSDARG00000099645	zgc:153184	383.96057	-0.817109818	0.15056363	-5.4270068	5.73E-08	7.70E-07
ENSDARG00000069473	frem1a	1943.61825	0.83509338	0.15391311	5.42574555	5.77E-08	7.75E-07
ENSDARG00000003757	cnpy1	469.221267	0.769180737	0.1418301	5.42325456	5.85E-08	7.85E-07
ENSDARG00000116806	CU693479.1	1698.95286	0.78708296	0.14516112	5.42213345	5.89E-08	7.89E-07
ENSDARG00000097592	si:dkey-40c23.1	48.0508678	2.607670093	0.480915	5.4223098	5.88E-08	7.89E-07

Table S2. DEGs of WT vs. *terfa-/-*

ENSDARG00000089936	selenow2b	1558.65175	0.702508122	0.1296024	5.42048719	5.94E-08	7.96E-07
ENSDARG00000044485	sall4	760.311522	0.711874948	0.13135623	5.41942282	5.98E-08	8.00E-07
ENSDARG00000035907	fam49al	3147.61013	-0.767895259	0.14171919	-5.41842839	6.01E-08	8.04E-07
ENSDARG00000093313	ubb	5295.48758	1.164945042	0.21500654	5.41818418	6.02E-08	8.05E-07
ENSDARG00000100865	si:dkey-269o24.1	165.718725	-1.50919272	0.27855344	-5.41796467	6.03E-08	8.06E-07
ENSDARG00000089646	ctdspl3	660.182623	-0.756823007	0.13969187	-5.4178027	6.03E-08	8.06E-07
ENSDARG00000038030	rbx1	1737.31157	0.766358038	0.14146338	5.41736004	6.05E-08	8.07E-07
ENSDARG00000078155	inavaa	462.412928	-1.078566625	0.19920448	-5.41436936	6.15E-08	8.21E-07
ENSDARG00000057334	cmasa	1242.09725	0.557496498	0.1029928	5.41296602	6.20E-08	8.26E-07
ENSDARG00000036897	sgms2b	221.397964	0.943682951	0.17433761	5.41296257	6.20E-08	8.26E-07
ENSDARG00000021004	c5	2881.55053	0.617422054	0.11407509	5.41241765	6.22E-08	8.28E-07
ENSDARG00000092290	si:dkeyp-26a9.2	358.846018	-1.148154168	0.21213441	-5.41239004	6.22E-08	8.28E-07
ENSDARG00000021059	alas1	5215.08046	0.711571049	0.13149315	5.41146867	6.25E-08	8.32E-07
ENSDARG00000097804	lypc	479.618983	-0.988141637	0.18276007	-5.40676989	6.42E-08	8.53E-07
ENSDARG00000116367	si:dkey-51d8.1	17.4663051	3.794194551	0.70212355	5.40388445	6.52E-08	8.67E-07
ENSDARG00000086735	SAMD4B	2332.61076	-0.613757314	0.11358447	-5.40353193	6.53E-08	8.68E-07
ENSDARG00000086453	cx52.9	110.708949	-1.823643706	0.33751443	-5.40315767	6.55E-08	8.69E-07
ENSDARG00000040157	glt8d1	691.442923	0.618720718	0.11451644	5.40289858	6.56E-08	8.70E-07
ENSDARG00000101679	cfap52	88.0191773	1.632381617	0.30218299	5.40196393	6.59E-08	8.74E-07
ENSDARG00000076015	PLBD1	44.831155	-2.842734157	0.52623202	-5.40205474	6.59E-08	8.74E-07
ENSDARG00000045299	vmo1b	414.362891	1.13358885	0.20988016	5.40112449	6.62E-08	8.77E-07
ENSDARG00000037291	dpf2l	1612.78051	-0.747427095	0.13839048	-5.40085651	6.63E-08	8.78E-07
ENSDARG00000054539	lrp10	1867.27917	0.581565617	0.10768865	5.40043569	6.65E-08	8.80E-07
ENSDARG00000062998	pglyrp2	44.7302462	2.339187077	0.43317295	5.4001227	6.66E-08	8.81E-07
ENSDARG00000069675	her8.2	114.700822	-1.441602745	0.26701651	-5.39892733	6.70E-08	8.86E-07
ENSDARG00000036894	aimp1b	816.595205	0.680565747	0.12606693	5.3984477	6.72E-08	8.88E-07
ENSDARG00000104267	postnb	50001.6376	0.537061344	0.09949047	5.39811867	6.73E-08	8.89E-07
ENSDARG00000100636	rbm12	1911.3924	-0.651537124	0.12069332	-5.39828657	6.73E-08	8.89E-07
ENSDARG00000117461	CR932999.1	560.032054	1.028061481	0.19045825	5.39783107	6.75E-08	8.90E-07
ENSDARG00000101975	si:dkey-225f5.4	392.707153	-0.796830375	0.14763952	-5.39713482	6.77E-08	8.93E-07
ENSDARG00000079166	ace	1546.63669	0.826011851	0.1530668	5.3964142	6.80E-08	8.96E-07
ENSDARG00000078979	arhgef40	470.140411	1.211077063	0.22454085	5.39357113	6.91E-08	9.10E-07
ENSDARG00000022531	ntn1b	1835.99551	0.838625093	0.15550709	5.39284143	6.94E-08	9.13E-07
ENSDARG00000097560	BX511311.3	217.430572	-1.36549949	0.25323849	-5.39214822	6.96E-08	9.16E-07
ENSDARG00000001558	kifc1	1919.15687	-1.215903563	0.22552631	-5.39140442	6.99E-08	9.19E-07
ENSDARG00000056400	helt	88.4033295	-1.817306046	0.33708672	-5.39121223	7.00E-08	9.20E-07
ENSDARG00000033971	prrx1a	1834.69326	0.560023738	0.10389652	5.39020671	7.04E-08	9.25E-07
ENSDARG00000092788	si:dkey-21e2.15	48.1178256	-3.31112536	0.61439629	-5.38923395	7.08E-08	9.29E-07
ENSDARG00000040401	slc25a20	3466.01432	0.542465207	0.10066936	5.38858289	7.10E-08	9.32E-07
ENSDARG00000061858	zgc:153968	154.702092	-2.033812425	0.37751086	-5.38742754	7.15E-08	9.37E-07
ENSDARG00000069192	zgc:163030	606.924423	0.917605358	0.17037576	5.38577409	7.21E-08	9.46E-07
ENSDARG00000054530	rars1	1836.45736	0.545556254	0.10132573	5.3841827	7.28E-08	9.54E-07
ENSDARG00000017034	sqor	1192.33348	0.613665656	0.11400592	5.38275238	7.34E-08	9.61E-07
ENSDARG00000100449	BX322631.1	59.2944568	2.100185305	0.39021847	5.38207559	7.36E-08	9.64E-07
ENSDARG00000101602	preb	1200.96486	0.539472373	0.10023806	5.38191173	7.37E-08	9.64E-07
ENSDARG00000092813	fndc7rs4	469.092017	-0.873535328	0.16234714	-5.38066346	7.42E-08	9.70E-07
ENSDARG00000093576	BX000981.2	26.3271649	3.599175411	0.66902832	5.37970561	7.46E-08	9.75E-07
ENSDARG00000079946	sqlea	140.336804	1.462968199	0.2719875	5.37880667	7.50E-08	9.79E-07
ENSDARG00000020465	ewsr1b	10294.2228	-0.745286393	0.13858454	-5.37784651	7.54E-08	9.84E-07
ENSDARG00000042732	tmem189	1872.47601	0.606780902	0.11294722	5.37225182	7.78E-08	1.01E-06
ENSDARG00000094512	gabrz	172.235734	-1.391509941	0.25895167	-5.37362795	7.72E-08	1.01E-06
ENSDARG00000003462	fech	167.026194	1.429607076	0.26612641	5.37190976	7.79E-08	1.02E-06
ENSDARG00000076302	dtx4a	1809.79959	-0.757700554	0.14111798	-5.36927022	7.91E-08	1.03E-06
ENSDARG00000078912	si:ch73-24k9.2	81.2096619	1.911789544	0.35612414	5.36832332	7.95E-08	1.03E-06
ENSDARG00000110658	zgc:174696	52.2159489	-2.284103767	0.42561635	-5.36657901	8.02E-08	1.04E-06
ENSDARG00000009418	mef2cb	2429.85244	-0.551205435	0.10271628	-5.36629102	8.04E-08	1.04E-06
ENSDARG00000104404	b4galt1l	2010.85452	0.58927138	0.10981028	5.3662678	8.04E-08	1.04E-06
ENSDARG00000030547	rnd1a	1212.41423	-0.81223938	0.15136188	-5.3662083	8.04E-08	1.04E-06

Table S2. DEGs of WT vs. *terfa-/-*

ENSDARG00000040907	gcgb	219.881102	1.277683959	0.23816225	5.36476271	8.11E-08	1.05E-06
ENSDARG00000060115	lrrn1	3020.46003	-0.936786602	0.17468911	-5.36259319	8.20E-08	1.06E-06
ENSDARG00000023546	kcnn1b	197.932373	-1.289439106	0.24049328	-5.36164293	8.25E-08	1.07E-06
ENSDARG00000086626	im:7147486	1155.84488	-0.653619567	0.12194652	-5.359887	8.33E-08	1.08E-06
ENSDARG00000100742	reep5	3435.21741	0.495479691	0.09243179	5.36049028	8.30E-08	1.08E-06
ENSDARG00000002396	cry-dash	977.398484	0.751845261	0.1402814	5.35955046	8.34E-08	1.08E-06
ENSDARG00000116774	PCNP	25.9935336	6.968469797	1.30024059	5.35936951	8.35E-08	1.08E-06
ENSDARG00000117298	AL929048.1	40.7933526	-2.752801816	0.51387557	-5.35694235	8.46E-08	1.09E-06
ENSDARG00000103133	hoxc12b	128.125321	-1.364101681	0.25457311	-5.35838879	8.40E-08	1.09E-06
ENSDARG00000097323	si:ch211-14i3.2	44.0904576	-2.916838274	0.54450025	-5.356909	8.47E-08	1.09E-06
ENSDARG00000022858	wnt7ba	44.0890409	-2.205639855	0.41188547	-5.35498344	8.56E-08	1.10E-06
ENSDARG00000005559	pou4f1	795.693203	-0.881134808	0.16453436	-5.35532392	8.54E-08	1.10E-06
ENSDARG00000024276	pcbp4	2373.55096	-0.588888024	0.10995339	-5.35579691	8.52E-08	1.10E-06
ENSDARG00000079712	gal3st4	1644.99401	-0.970423243	0.18128024	-5.35316628	8.64E-08	1.11E-06
ENSDARG00000103259	opn4xb	158.699497	-1.66668449	0.31135602	-5.35298624	8.65E-08	1.11E-06
ENSDARG00000098540	zc3h7ba	477.465555	0.695928337	0.12996499	5.35473697	8.57E-08	1.11E-06
ENSDARG00000019579	ldb2a	1264.44349	-0.68107576	0.12723027	-5.35309532	8.65E-08	1.11E-06
ENSDARG00000045153	slc16a8	215.679869	1.756301408	0.32806056	5.35358897	8.62E-08	1.11E-06
ENSDARG00000091140	pik3r6b	17.7653988	4.078095458	0.76197019	5.35204066	8.70E-08	1.12E-06
ENSDARG00000105060	rdh8b	371.232913	-1.158025133	0.2164455	-5.35019272	8.79E-08	1.13E-06
ENSDARG00000040295	apoeb	39780.4325	0.761780477	0.14238372	5.35019381	8.79E-08	1.13E-06
ENSDARG00000040505	yif1b	477.01334	0.927150258	0.17329905	5.35000195	8.80E-08	1.13E-06
ENSDARG00000098365	lrrc74b	59.2383784	1.606374389	0.30034556	5.34842071	8.87E-08	1.14E-06
ENSDARG00000099412	bcan	1219.98833	-0.581006674	0.10863916	-5.34804078	8.89E-08	1.14E-06
ENSDARG00000039225	vps13a	4774.96884	0.522864868	0.09776957	5.34793029	8.90E-08	1.14E-06
ENSDARG00000018903	aimp2	3670.36866	0.670042238	0.12532522	5.346428	8.97E-08	1.15E-06
ENSDARG00000032575	ywhaz	12855.5585	0.546951701	0.10235199	5.34383077	9.10E-08	1.16E-06
ENSDARG00000007207	cnpy2	609.463582	0.793089075	0.14836611	5.34548666	9.02E-08	1.16E-06
ENSDARG00000088593	chst1	541.663944	-1.032340641	0.19318143	-5.34389185	9.10E-08	1.16E-06
ENSDARG00000104197	ptpn22	267.234568	1.027928637	0.19240447	5.34254021	9.17E-08	1.17E-06
ENSDARG00000062263	arhgap17b	1071.49008	0.653614009	0.12237365	5.3411334	9.24E-08	1.18E-06
ENSDARG00000091555	ostf1	827.004558	0.673604697	0.12616561	5.33905177	9.34E-08	1.19E-06
ENSDARG00000088073	fam110b	1222.1555	-0.681369063	0.1276683	-5.3370263	9.45E-08	1.21E-06
ENSDARG00000011652	ftr52p	67.7538011	1.507659392	0.28254394	5.33601744	9.50E-08	1.21E-06
ENSDARG00000098733	pxdn	2612.86125	1.10712362	0.20749765	5.33559605	9.52E-08	1.21E-06
ENSDARG00000093667	BX005283.1	18.6100236	-4.718358447	0.88430705	-5.33565628	9.52E-08	1.21E-06
ENSDARG00000044356	tp63	2452.13224	0.459432236	0.08612822	5.3342824	9.59E-08	1.22E-06
ENSDARG00000020984	slc16a10	3028.21267	0.608189526	0.11399118	5.33540888	9.53E-08	1.22E-06
ENSDARG00000105214	agtpbp1	923.674871	0.592023091	0.1109843	5.33429611	9.59E-08	1.22E-06
ENSDARG00000055092	pora	2762.13216	0.51466572	0.09647042	5.33495913	9.56E-08	1.22E-06
ENSDARG00000030364	angpt1	449.444422	0.69609187	0.13052493	5.33301842	9.66E-08	1.23E-06
ENSDARG00000076230	atp10b	293.682748	1.119178003	0.20994068	5.33092497	9.77E-08	1.24E-06
ENSDARG00000094436	CR848723.1	31.264619	4.972530074	0.93315329	5.32873874	9.89E-08	1.26E-06
ENSDARG00000033251	osbpl3a	490.98045	0.713832805	0.1339568	5.3288283	9.88E-08	1.26E-06
ENSDARG00000059279	tfap2a	5375.34975	-0.726029815	0.13625598	-5.32842535	9.91E-08	1.26E-06
ENSDARG00000074036	ky	93.1564899	1.978449282	0.37141883	5.32673393	1.00E-07	1.27E-06
ENSDARG00000087369	cep162	307.794632	-0.901527861	0.16931376	-5.32459896	1.01E-07	1.28E-06
ENSDARG00000099510	cnn3a	2903.70029	0.516034221	0.0969122	5.32476019	1.01E-07	1.28E-06
ENSDARG00000045543	atp6v1f	1566.66625	-0.548065433	0.10295191	-5.32350896	1.02E-07	1.29E-06
ENSDARG00000104685	grk1b	981.02399	-3.688968581	0.69300312	-5.32316299	1.02E-07	1.29E-06
ENSDARG00000033273	primpol	389.017246	-1.196505256	0.22485518	-5.32122604	1.03E-07	1.30E-06
ENSDARG00000088764	lyplal1	137.119871	1.308828759	0.24593174	5.32191885	1.03E-07	1.30E-06
ENSDARG00000013414	lin7a	1603.86928	-0.689702948	0.12962443	-5.32077887	1.03E-07	1.31E-06
ENSDARG00000097118	CU693494.2	4657.53432	1.035416023	0.19462638	5.32001901	1.04E-07	1.31E-06
ENSDARG00000035715	marcksl1b	19192.9029	-0.758575612	0.14256427	-5.32093787	1.03E-07	1.31E-06
ENSDARG00000071458	si:dkey-121a11.3	560.113981	-0.925544734	0.17401339	-5.3188132	1.04E-07	1.32E-06
ENSDARG00000045748	stab2	1183.36195	0.977269909	0.18383358	5.31605772	1.06E-07	1.34E-06
ENSDARG00000038868	en2b	218.467042	-1.018446208	0.19161873	-5.31496171	1.07E-07	1.34E-06

Table S2. DEGs of WT vs. *terfa-/-*

ENSDARG00000100747	GUCA1A	124.523045	-1.805021343	0.33954322	-5.31602831	1.06E-07	1.34E-06
ENSDARG00000010531	scamp4	1787.46175	0.934458485	0.17579229	5.31569659	1.06E-07	1.34E-06
ENSDARG00000011326	ankrd45	25.5226617	2.645115067	0.49776717	5.31396049	1.07E-07	1.35E-06
ENSDARG00000000018	nrf1	2007.03693	-0.840016034	0.15814828	-5.31157246	1.09E-07	1.37E-06
ENSDARG00000071173	slc12a10.2	1007.3867	1.208326055	0.22747388	5.31193332	1.08E-07	1.37E-06
ENSDARG00000002510	dmbx1b	133.243621	-1.193271433	0.22467803	-5.31102859	1.09E-07	1.37E-06
ENSDARG00000016022	zic5	852.22299	-0.738728051	0.13911619	-5.31015172	1.10E-07	1.38E-06
ENSDARG00000043457	gapdh	66832.7659	0.765629435	0.14421455	5.30896109	1.10E-07	1.38E-06
ENSDARG00000105258	cep72	97.3692644	-1.67016916	0.31457491	-5.30928909	1.10E-07	1.38E-06
ENSDARG00000044431	ppig	1503.6596	-0.727389635	0.13705545	-5.30726522	1.11E-07	1.40E-06
ENSDARG00000058203	smc1al	6269.81344	-0.509594359	0.09605318	-5.30533571	1.12E-07	1.41E-06
ENSDARG00000103129	nid1b	6380.97645	0.631289292	0.11899364	5.30523568	1.13E-07	1.41E-06
ENSDARG00000045887	mmp30	966.511279	0.712843896	0.13436951	5.30510154	1.13E-07	1.41E-06
ENSDARG00000062023	fndc3bb	1509.81754	0.768080861	0.14475646	5.30602141	1.12E-07	1.41E-06
ENSDARG00000020795	rac3b	3448.23282	-0.56738473	0.10697041	-5.30412771	1.13E-07	1.42E-06
ENSDARG00000013307	rpl19	40247.0015	0.498797678	0.0941044	5.30047124	1.16E-07	1.44E-06
ENSDARG00000038687	nfkb2	1331.22677	0.686039908	0.12942686	5.3005994	1.15E-07	1.44E-06
ENSDARG00000095826	smdt1a	32.6506953	2.599344253	0.49046462	5.29975898	1.16E-07	1.45E-06
ENSDARG00000090963	atp6ap1lb	682.978394	-0.668185785	0.12608047	-5.29967699	1.16E-07	1.45E-06
ENSDARG00000019304	phactr3b	833.21219	-0.899135882	0.16969936	-5.29840459	1.17E-07	1.46E-06
ENSDARG00000102638	si:rp71-7l19.2	19.3587201	3.921594151	0.74033554	5.29704974	1.18E-07	1.47E-06
ENSDARG00000025858	zgc:56525	2870.41071	0.667072669	0.12594028	5.29673797	1.18E-07	1.47E-06
ENSDARG00000035870	laptm4b	1719.91012	-0.570268106	0.10768863	-5.29552772	1.19E-07	1.48E-06
ENSDARG00000103612	akt2l	2188.66631	0.785320506	0.14831388	5.29498984	1.19E-07	1.48E-06
ENSDARG00000057110	slc25a1a	606.787802	-1.004879086	0.18980429	-5.29429078	1.19E-07	1.49E-06
ENSDARG00000117659	CABZ01079280.1	80.9930052	-1.682418824	0.31780207	-5.29392025	1.20E-07	1.49E-06
ENSDARG00000041433	si:dkey-7c18.24	64.9154066	2.538619604	0.47964921	5.2926588	1.21E-07	1.50E-06
ENSDARG00000105274	CABZ01074130.1	16566.7903	1.140664156	0.21552582	5.29247095	1.21E-07	1.50E-06
ENSDARG00000018797	crygmxl1	89.2124759	-1.970841584	0.37253277	-5.29038451	1.22E-07	1.52E-06
ENSDARG00000006672	fam49a	947.028851	-0.577590893	0.10920607	-5.28899987	1.23E-07	1.53E-06
ENSDARG00000007523	kmt2e	3776.93953	-0.498040976	0.09425068	-5.2842164	1.26E-07	1.56E-06
ENSDARG00000018241	mfap1	1632.77413	-0.578813616	0.1095221	-5.28490258	1.26E-07	1.56E-06
ENSDARG00000028275	sult1st1	1852.69242	0.655816753	0.12410506	5.28436771	1.26E-07	1.56E-06
ENSDARG00000040668	lrrc51	26.4295395	2.303868838	0.43596193	5.28456427	1.26E-07	1.56E-06
ENSDARG00000089838	si:dkey-262k9.4	136.14766	-1.45359714	0.2750977	-5.28393063	1.26E-07	1.57E-06
ENSDARG00000074727	dnajc10	1164.65007	0.491026221	0.0929591	5.28217507	1.28E-07	1.58E-06
ENSDARG00000036291	nucb2b	546.280596	0.823572251	0.15594824	5.28106144	1.28E-07	1.59E-06
ENSDARG00000092283	cxl34b.11	417.628022	0.73597201	0.13937286	5.28059764	1.29E-07	1.59E-06
ENSDARG00000089920	mlip	617.61374	0.992315165	0.18796219	5.2793338	1.30E-07	1.60E-06
ENSDARG00000020618	gatd3a	761.861764	0.761624001	0.14429774	5.27814237	1.31E-07	1.61E-06
ENSDARG00000095809	dlg4b	47.6627298	-2.637006655	0.49956879	-5.27856568	1.30E-07	1.61E-06
ENSDARG00000036144	tssc4	1037.80263	-0.588176183	0.11146832	-5.2766218	1.32E-07	1.62E-06
ENSDARG00000053906	angpt2b	139.569569	1.150803425	0.21805672	5.27754175	1.31E-07	1.62E-06
ENSDARG00000101507	ATG2A	2060.09402	0.623989521	0.11828518	5.27529759	1.33E-07	1.63E-06
ENSDARG00000029832	slc26a1	444.207912	-0.828837957	0.15712016	-5.27518526	1.33E-07	1.64E-06
ENSDARG00000037837	ogfr	1296.34864	-0.587982258	0.11152795	-5.2720621	1.35E-07	1.66E-06
ENSDARG00000007461	srgap1a	941.575202	0.755965218	0.14344851	5.26994113	1.36E-07	1.68E-06
ENSDARG00000101317	grinaa	817.719192	-0.661725128	0.12556567	-5.26995246	1.36E-07	1.68E-06
ENSDARG00000013928	plekhb2	705.739349	0.675467674	0.12819074	5.26923909	1.37E-07	1.69E-06
ENSDARG00000033355	zgc:101699	16.9129885	3.312629479	0.62909455	5.26571007	1.40E-07	1.72E-06
ENSDARG00000061585	cyp4v7	213.747081	1.409430333	0.26780088	5.26297883	1.42E-07	1.74E-06
ENSDARG00000097527	ms4a17a.5	20.4268047	3.37656716	0.64161674	5.26259208	1.42E-07	1.75E-06
ENSDARG00000045269	pts	448.387624	0.8410726	0.15986109	5.26127148	1.43E-07	1.76E-06
ENSDARG00000104418	BX248318.1	205.883774	-1.266085202	0.2407155	-5.25967456	1.44E-07	1.77E-06
ENSDARG00000014966	ccdc82	777.895732	-0.706192906	0.13429257	-5.25861471	1.45E-07	1.78E-06
ENSDARG00000101562	znf1014	505.503101	-0.773487279	0.14713886	-5.25685249	1.47E-07	1.80E-06
ENSDARG00000109510	lsm12b	2130.26089	0.520967378	0.09910507	5.25671767	1.47E-07	1.80E-06
ENSDARG00000100359	AREL1	1323.28229	-0.582157175	0.11076534	-5.25577025	1.47E-07	1.81E-06

Table S2. DEGs of WT vs. *terfa-/-*

ENSDARG00000061600	cemip2	1477.4997	0.651067423	0.12391702	5.25405965	1.49E-07	1.82E-06
ENSDARG00000014953	cnih3	282.345862	-0.941057063	0.17913461	-5.25335136	1.49E-07	1.83E-06
ENSDARG00000077068	si:ch211-11p18.6	28.9303142	4.911098185	0.93489351	5.25310975	1.50E-07	1.83E-06
ENSDARG00000073808	WDR31	150.660402	-1.165313616	0.22189552	-5.2516321	1.51E-07	1.84E-06
ENSDARG00000078738	si:dkey-145c18.3	11.5467819	5.774265099	1.09968774	5.25082248	1.51E-07	1.85E-06
ENSDARG00000103295	cyp3a65	323.184041	1.561030469	0.29734754	5.24985156	1.52E-07	1.86E-06
ENSDARG00000104540	cyp2aa8	1634.88143	0.823186157	0.15681655	5.24935782	1.53E-07	1.86E-06
ENSDARG00000101627	ssr1	5489.16275	0.455593549	0.08678487	5.24968856	1.52E-07	1.86E-06
ENSDARG00000102550	CABZ01080371.1	799.532852	-0.75819457	0.1444339	-5.24942254	1.53E-07	1.86E-06
ENSDARG00000098584	CABZ01102240.1	2366.55327	0.675700772	0.12886683	5.2434033	1.58E-07	1.92E-06
ENSDARG00000055172	si:ch211-256m1.8	2332.12059	-0.904613022	0.17259024	-5.24139158	1.59E-07	1.94E-06
ENSDARG00000038635	magoh	1307.18982	-0.695674396	0.13271801	-5.24174832	1.59E-07	1.94E-06
ENSDARG00000069537	si:dkey-238c7.16	726.27128	0.612459468	0.11690209	5.23908064	1.61E-07	1.96E-06
ENSDARG00000074082	gpc5c	215.397428	1.080606733	0.20627596	5.23864601	1.62E-07	1.97E-06
ENSDARG00000110186	CDCP1	1148.70593	0.605005851	0.11552064	5.23721012	1.63E-07	1.98E-06
ENSDARG00000031317	ppdpfb	10945.5851	0.670990445	0.12810412	5.23785222	1.62E-07	1.98E-06
ENSDARG00000097224	prr33	4237.37648	0.90650017	0.17312694	5.23604345	1.64E-07	1.99E-06
ENSDARG00000054609	slco2b1	866.662836	1.185118841	0.22636453	5.23544411	1.65E-07	2.00E-06
ENSDARG00000105001	zgc:91999	1747.40647	0.723003717	0.13812913	5.23425973	1.66E-07	2.01E-06
ENSDARG00000095283	si:dkeyp-2e4.3	91.3440452	1.37260992	0.26227347	5.23350651	1.66E-07	2.02E-06
ENSDARG00000005945	sart1	3210.57396	-0.558004511	0.10671563	-5.22889206	1.71E-07	2.07E-06
ENSDARG00000096616	si:ch211-255p10.3	3393.77397	0.733195016	0.14024986	5.22777719	1.72E-07	2.08E-06
ENSDARG00000031770	kat7b	1243.31307	-0.5256703	0.10055567	-5.22765438	1.72E-07	2.08E-06
ENSDARG00000008433	unc45b	1717.97522	0.55959047	0.10715101	5.22244721	1.77E-07	2.14E-06
ENSDARG00000012667	tfap2b	2510.74528	-1.248055763	0.2391447	-5.21883104	1.80E-07	2.18E-06
ENSDARG00000074653	si:ch211-233m11.1	12.1275938	7.2224283	1.38414725	5.21796239	1.81E-07	2.19E-06
ENSDARG00000105882	CABZ01041495.1	384.244471	-2.597344343	0.4977614	-5.21805095	1.81E-07	2.19E-06
ENSDARG00000020527	nup62l	1703.00996	-0.588448605	0.11279962	-5.21676056	1.82E-07	2.20E-06
ENSDARG00000010137	ldb1a	4747.72762	-0.653101326	0.12519173	-5.21680868	1.82E-07	2.20E-06
ENSDARG00000035088	si:ch211-254c8.3	14.7286787	7.130359624	1.36736578	5.21466876	1.84E-07	2.22E-06
ENSDARG00000089886	si:dkey-19e4.5	2133.6012	-0.583728322	0.1119899	-5.21233021	1.86E-07	2.25E-06
ENSDARG00000034541	tgfbr2b	1608.03921	0.664887718	0.12756501	5.21214812	1.87E-07	2.25E-06
ENSDARG00000060601	rgs7bpa	496.12061	-0.670126187	0.12858434	-5.21156904	1.87E-07	2.26E-06
ENSDARG00000058421	lgi1b	549.469157	-1.09579652	0.21028281	-5.21106086	1.88E-07	2.26E-06
ENSDARG00000022768	gid8a	489.906586	-0.774567607	0.14865087	-5.21064984	1.88E-07	2.27E-06
ENSDARG00000075725	cep152	630.268379	0.787199748	0.15110641	5.20957233	1.89E-07	2.28E-06
ENSDARG00000004497	tspan33a	453.37667	0.728348267	0.13984338	5.20831415	1.91E-07	2.29E-06
ENSDARG00000034048	zgc:92275	195.152016	-1.114121505	0.21394983	-5.2073961	1.92E-07	2.30E-06
ENSDARG00000037507	kctd12b	87.95724	-1.83901964	0.35324703	-5.20604418	1.93E-07	2.32E-06
ENSDARG00000035326	nccrp1	1157.3422	0.703234801	0.13512212	5.20443867	1.95E-07	2.33E-06
ENSDARG00000053291	pnrc2	6527.53026	-0.589196018	0.11319807	-5.20500064	1.94E-07	2.33E-06
ENSDARG00000017602	ccng2	1365.84871	-0.585728528	0.11253327	-5.20493644	1.94E-07	2.33E-06
ENSDARG00000039215	arrb2a	752.536331	-0.653170072	0.12549321	-5.20482395	1.94E-07	2.33E-06
ENSDARG00000026482	arhgap12b	1644.67032	-0.622506709	0.11965557	-5.2024884	1.97E-07	2.36E-06
ENSDARG00000052567	tmem35	2852.95443	-0.730155648	0.14037446	-5.20148491	1.98E-07	2.37E-06
ENSDARG00000102793	slc16a6a	572.677566	-2.370565034	0.45588869	-5.19987685	1.99E-07	2.39E-06
ENSDARG00000070037	rgs8	511.113883	-1.10617854	0.21282565	-5.19758095	2.02E-07	2.42E-06
ENSDARG00000099416	ly6m3	23.7689155	6.346040767	1.22146038	5.19545362	2.04E-07	2.44E-06
ENSDARG00000063144	shisa7b	3100.36945	-0.744712547	0.14336373	-5.19456716	2.05E-07	2.45E-06
ENSDARG00000090223	msl1b	1256.81079	-0.574993462	0.11072293	-5.19308395	2.07E-07	2.47E-06
ENSDARG00000074160	paqr4b	78.3025041	-2.326184671	0.44798038	-5.19260389	2.07E-07	2.48E-06
ENSDARG00000039490	pitpnaa	1546.45855	0.525734804	0.10124878	5.19250516	2.07E-07	2.48E-06
ENSDARG00000114719	CABZ01068246.1	151.892707	0.99038215	0.19075395	5.19193531	2.08E-07	2.48E-06
ENSDARG00000042861	ltk	666.731429	-0.835038669	0.16086508	-5.19092554	2.09E-07	2.50E-06
ENSDARG00000061764	ahnak	20391.6849	0.917045818	0.17669467	5.19000273	2.10E-07	2.51E-06
ENSDARG00000007603	stxbp2	1469.87302	0.551922592	0.10638775	5.18783948	2.13E-07	2.54E-06
ENSDARG00000101026	CABZ01038709.1	208.347621	-3.196971076	0.61624452	-5.18782878	2.13E-07	2.54E-06
ENSDARG00000094809	ms4a17a.11	1121.89129	1.083225669	0.20884917	5.18664093	2.14E-07	2.55E-06

Table S2. DEGs of WT vs. *terfa-/-*

ENSDARG00000026796	grm1a	926.918848	-0.958779771	0.18484712	-5.1868796	2.14E-07	2.55E-06
ENSDARG00000103826	gpib	2985.62608	0.505842314	0.09752557	5.18676611	2.14E-07	2.55E-06
ENSDARG00000062307	ccdc61	434.129335	-0.850576976	0.16407244	-5.18415496	2.17E-07	2.58E-06
ENSDARG00000101348	plppr5b	240.801046	-1.430953809	0.27600974	-5.18443238	2.17E-07	2.58E-06
ENSDARG00000097855	BX323586.3	295.566398	-0.884131163	0.17056586	-5.18351766	2.18E-07	2.59E-06
ENSDARG00000006169	lrrk2	1465.21363	0.922274321	0.17793264	5.18327788	2.18E-07	2.59E-06
ENSDARG00000045524	lamb1b	2561.78331	0.61463705	0.11862281	5.18144057	2.20E-07	2.61E-06
ENSDARG00000028098	fut9d	1985.219	0.722295583	0.1393884	5.1818917	2.20E-07	2.61E-06
ENSDARG00000094666	CU571324.1	44.5840164	2.305395188	0.44502371	5.18038731	2.21E-07	2.62E-06
ENSDARG00000021697	morn3	98.3793125	1.388009119	0.26793081	5.1804759	2.21E-07	2.62E-06
ENSDARG00000071648	zgc:113298	101.841045	1.341873821	0.25902536	5.18047286	2.21E-07	2.62E-06
ENSDARG00000056921	ankk1	204.10972	1.059895715	0.20461737	5.17989123	2.22E-07	2.63E-06
ENSDARG00000099519	cpdp	919.985248	0.577984317	0.11162081	5.17810533	2.24E-07	2.65E-06
ENSDARG00000045129	fkbp10b	1624.75631	0.605275811	0.11690667	5.17742745	2.25E-07	2.66E-06
ENSDARG00000098752	csf3b	37.1544181	3.975677288	0.76782145	5.17786689	2.24E-07	2.66E-06
ENSDARG00000091650	igflr1	105.158146	-1.335165667	0.25790876	-5.1768916	2.26E-07	2.67E-06
ENSDARG00000078973	uckl1b	734.247864	0.775946137	0.14990794	5.17615108	2.27E-07	2.68E-06
ENSDARG00000018459	msrb2	2062.5882	1.005565736	0.19433438	5.17440991	2.29E-07	2.70E-06
ENSDARG00000101606	rims2a	986.411992	-0.712543002	0.13772781	-5.17355941	2.30E-07	2.71E-06
ENSDARG00000019930	tal1	740.147025	-1.066778613	0.20619145	-5.17372858	2.29E-07	2.71E-06
ENSDARG00000101567	guca1e	54.7241885	-2.471981596	0.4779509	-5.17204094	2.32E-07	2.73E-06
ENSDARG00000002635	FAM184A	1211.44522	-0.769154658	0.14876492	-5.17026894	2.34E-07	2.75E-06
ENSDARG00000114883	CU655961.4	10.6919113	7.339391852	1.41949479	5.17042534	2.34E-07	2.75E-06
ENSDARG00000087979	CT573433.1	392.135064	-0.851903312	0.16483977	-5.16806914	2.37E-07	2.78E-06
ENSDARG00000053431	si:ch211-257p13.3	865.532583	-0.574037416	0.1110918	-5.16723504	2.38E-07	2.80E-06
ENSDARG00000054288	zgc:63972	143.634165	-1.298904517	0.25142615	-5.16614732	2.39E-07	2.81E-06
ENSDARG00000101514	ftr64	42.2851387	-2.344214556	0.45384028	-5.16528541	2.40E-07	2.82E-06
ENSDARG00000012593	col5a1	32620.9882	0.512078452	0.09913948	5.16523231	2.40E-07	2.82E-06
ENSDARG00000098874	exoc6b	1374.79296	0.630874384	0.12217915	5.16351931	2.42E-07	2.85E-06
ENSDARG00000092770	si:ch211-253p18.2	23.1830843	3.44296227	0.66690593	5.1625906	2.44E-07	2.86E-06
ENSDARG00000044001	lgals3b	3993.71437	0.726198666	0.14067467	5.16225597	2.44E-07	2.86E-06
ENSDARG00000017105	vaspb	4872.55392	0.439305175	0.08511092	5.16156045	2.45E-07	2.87E-06
ENSDARG00000090615	cd59	266.106026	-1.269226825	0.24592075	-5.16112136	2.45E-07	2.88E-06
ENSDARG00000061909	si:ch73-233f7.1	1708.27892	-1.065561728	0.20652036	-5.15959663	2.47E-07	2.90E-06
ENSDARG00000103456	cyp2aa11	58.831761	2.323928019	0.45043663	5.15927847	2.48E-07	2.90E-06
ENSDARG00000086425	prpf3	1221.34217	-0.532230843	0.10316435	-5.15905798	2.48E-07	2.91E-06
ENSDARG00000097728	CR388132.1	45.3561497	7.293167332	1.41454205	5.15585051	2.52E-07	2.95E-06
ENSDARG00000019808	evpla	2768.03985	0.503854283	0.09772884	5.15563559	2.53E-07	2.96E-06
ENSDARG00000006791	arntl1a	1103.82432	-0.708035882	0.13733383	-5.15558239	2.53E-07	2.96E-06
ENSDARG00000041952	prox2	134.438825	-1.227869515	0.23823387	-5.15405094	2.55E-07	2.98E-06
ENSDARG00000028335	hmga1a	42820.2068	-0.867943278	0.16841979	-5.15345193	2.56E-07	2.99E-06
ENSDARG00000058256	draxin	627.87917	-1.064214016	0.20657554	-5.15169428	2.58E-07	3.01E-06
ENSDARG00000101164	nansb	395.314798	0.773043976	0.15009421	5.15039189	2.60E-07	3.03E-06
ENSDARG00000091951	nuggc.2	21.4400924	-4.479497992	0.86978745	-5.15010648	2.60E-07	3.04E-06
ENSDARG00000025850	rps21	19451.2118	0.55245498	0.10727914	5.149696	2.61E-07	3.04E-06
ENSDARG00000095906	CR848047.1	1332.32499	-1.287448322	0.25008148	-5.14811538	2.63E-07	3.06E-06
ENSDARG00000055270	si:ch1073-358c10.	73.0562093	1.924143763	0.37374054	5.14834109	2.63E-07	3.06E-06
ENSDARG00000076313	NA	14.3906414	-8.044704169	1.56281538	-5.14757168	2.64E-07	3.07E-06
ENSDARG00000086184	FBXO48	522.363262	-0.68340724	0.13276495	-5.14749759	2.64E-07	3.07E-06
ENSDARG00000036878	sh3bgrl2	490.946519	-0.851704075	0.16547773	-5.14694068	2.65E-07	3.08E-06
ENSDARG00000010752	acsl4b	672.58432	1.006533129	0.19557928	5.14642012	2.66E-07	3.09E-06
ENSDARG00000052148	ptgs1	519.461996	0.959612924	0.18648589	5.14576692	2.66E-07	3.09E-06
ENSDARG00000058753	si:ch73-213k20.5	35.0700072	2.511850594	0.4880808	5.1463827	2.66E-07	3.09E-06
ENSDARG00000041294	noxo1a	600.672382	1.258774604	0.24464995	5.14520688	2.67E-07	3.10E-06
ENSDARG00000070098	lcorl	1783.17568	-0.555609485	0.10800276	-5.14440088	2.68E-07	3.11E-06
ENSDARG00000071143	si:dkey-208m12.2	162.387075	1.366134483	0.26558013	5.14396355	2.69E-07	3.12E-06
ENSDARG00000091029	phox2bb	743.269363	-0.714789028	0.13901492	-5.14181514	2.72E-07	3.15E-06
ENSDARG00000069909	HTRA2	437.293236	0.733506628	0.14266436	5.14148472	2.73E-07	3.16E-06

Table S2. DEGs of WT vs. *terfa-/-*

ENSDARG00000074697	npdc1a	647.356602	-0.833786901	0.1621771	-5.14121227	2.73E-07	3.16E-06
ENSDARG00000083189	arhgap19	531.096551	-0.940658978	0.18300826	-5.13998101	2.75E-07	3.18E-06
ENSDARG00000103732	plp1a	460.899082	-1.100129657	0.21412191	-5.13786579	2.78E-07	3.22E-06
ENSDARG00000043012	napgb	1425.52989	-0.633180968	0.12329604	-5.1354528	2.81E-07	3.26E-06
ENSDARG00000038107	sgcg	1568.1964	1.025574906	0.19972414	5.13495712	2.82E-07	3.26E-06
ENSDARG00000043770	si:dkey-192d15.2	388.790213	1.172380462	0.228347	5.13420559	2.83E-07	3.27E-06
ENSDARG00000059987	dnah12	296.229232	0.996881942	0.19418918	5.13356074	2.84E-07	3.28E-06
ENSDARG00000097639	si:ch73-265h17.4	46.2746051	1.979411886	0.38573556	5.13152562	2.87E-07	3.32E-06
ENSDARG00000100006	si:ch211-76m11.5	19.2437078	3.010525759	0.58678167	5.13057224	2.89E-07	3.33E-06
ENSDARG00000044501	viml	77.4511503	-1.559838124	0.30406442	-5.12995937	2.90E-07	3.34E-06
ENSDARG00000069910	gtf2f2a	1458.39482	-0.548604889	0.10694897	-5.12959485	2.90E-07	3.35E-06
ENSDARG00000037476	sorbs3	1116.38266	0.87932489	0.17143915	5.12907862	2.91E-07	3.36E-06
ENSDARG00000097533	BX571880.1	504.812523	1.36680617	0.26657572	5.12727185	2.94E-07	3.39E-06
ENSDARG00000091702	csnk2a4	82.5780696	1.527796414	0.29806583	5.12570123	2.96E-07	3.41E-06
ENSDARG00000070673	ptgr2	448.283529	0.672966838	0.13142876	5.12039261	3.05E-07	3.51E-06
ENSDARG00000059423	prune2	1286.2567	-0.58367275	0.1140297	-5.11860274	3.08E-07	3.54E-06
ENSDARG00000070083	atp5f1b	81540.7095	0.533588646	0.10424125	5.11878603	3.08E-07	3.54E-06
ENSDARG00000007377	odc1	5036.71893	0.581854494	0.11375417	5.11501705	3.14E-07	3.60E-06
ENSDARG00000079977	nhsb	1666.76368	-0.825879978	0.16148951	-5.11414019	3.15E-07	3.62E-06
ENSDARG00000002589	mylpfb	40518.2537	0.65548226	0.12818219	5.11367654	3.16E-07	3.63E-06
ENSDARG00000094709	arl8	1592.73966	-0.64974324	0.12709122	-5.11241647	3.18E-07	3.65E-06
ENSDARG00000075650	vps26c	1696.12084	0.629003661	0.12303629	5.11234273	3.18E-07	3.65E-06
ENSDARG00000005626		158.793275	-1.052570562	0.20595875	-5.11058914	3.21E-07	3.68E-06
ENSDARG00000095715	BX465834.1	391.801844	-0.89021732	0.1742154	-5.10986586	3.22E-07	3.69E-06
ENSDARG00000061039	atp10a	375.205094	0.925898351	0.18122746	5.10903995	3.24E-07	3.71E-06
ENSDARG00000070826	bpgm	754.392884	-0.666072616	0.13037813	-5.10877563	3.24E-07	3.71E-06
ENSDARG00000024940	rnf144b	689.068403	0.580146121	0.11356645	5.10842879	3.25E-07	3.72E-06
ENSDARG00000099197	actc1b	699020.914	0.770237188	0.15082637	5.10678064	3.28E-07	3.75E-06
ENSDARG00000058946	CU856539.2	1392.23947	0.54327286	0.10641839	5.10506576	3.31E-07	3.78E-06
ENSDARG00000070683	dkk3b	366.253614	0.915740949	0.17939169	5.10470114	3.31E-07	3.79E-06
ENSDARG00000014420	elavl3	10774.398	-0.786095146	0.15402064	-5.10382982	3.33E-07	3.80E-06
ENSDARG00000033161	sst1.2	197.519166	0.943132209	0.18478202	5.10402598	3.33E-07	3.80E-06
ENSDARG00000062374	leng9	206.733244	1.00438826	0.19681976	5.10308651	3.34E-07	3.81E-06
ENSDARG00000014013	lbr	2396.57174	-1.069535671	0.20959757	-5.10280571	3.35E-07	3.81E-06
ENSDARG00000011196	dnajc11a	2365.16844	0.492252003	0.09645823	5.10326596	3.34E-07	3.81E-06
ENSDARG00000029710	lrrc30a	101.445831	1.2546292	0.2458675	5.10286726	3.35E-07	3.81E-06
ENSDARG00000053644	got1l1	49.4721265	2.061097076	0.40396736	5.10213759	3.36E-07	3.83E-06
ENSDARG00000043242	si:dkey-222f2.1	1613.83559	-0.604062953	0.11843053	-5.10056807	3.39E-07	3.86E-06
ENSDARG00000002213	invs	258.403214	1.139450238	0.22346522	5.09900482	3.41E-07	3.89E-06
ENSDARG00000044182	stau1	5758.8345	0.38864247	0.07626087	5.09622375	3.46E-07	3.94E-06
ENSDARG00000036171	rnasel3	1314.73181	0.642877304	0.12616592	5.09549107	3.48E-07	3.95E-06
ENSDARG00000102956	mmp17b	245.198255	-1.087540577	0.21343819	-5.09534212	3.48E-07	3.96E-06
ENSDARG00000053558	rtkn2a	274.922055	-1.032504764	0.20266916	-5.09453309	3.50E-07	3.97E-06
ENSDARG00000101495	ugt5b2	283.019421	-0.88670525	0.17406703	-5.09404471	3.51E-07	3.98E-06
ENSDARG00000017143	brd9	1030.92215	-0.571477548	0.11224143	-5.09150264	3.55E-07	4.03E-06
ENSDARG00000102332	spint1a	3760.92334	0.639044779	0.12555798	5.08963907	3.59E-07	4.07E-06
ENSDARG00000091771	lysmd2	1011.3624	-0.763931412	0.15009263	-5.08973307	3.59E-07	4.07E-06
ENSDARG00000091631	si:ch211-197h24.6	1363.44601	-0.624452422	0.12271225	-5.0887537	3.60E-07	4.08E-06
ENSDARG00000104291	GPR151	267.391716	-1.036450579	0.20369485	-5.08825116	3.61E-07	4.09E-06
ENSDARG00000097819	znf576.1	1344.18569	-0.615874588	0.12105205	-5.087684	3.62E-07	4.10E-06
ENSDARG00000012874	snap23.1	1483.05908	0.585244668	0.11502239	5.08809365	3.62E-07	4.10E-06
ENSDARG00000020079	neil3	279.809369	-0.948963069	0.18652299	-5.08764667	3.63E-07	4.10E-06
ENSDARG00000095448	ftr30	30.1883095	2.907236486	0.57156662	5.08643503	3.65E-07	4.13E-06
ENSDARG00000029071	creld2	480.526284	0.795178246	0.15635698	5.08565881	3.66E-07	4.14E-06
ENSDARG00000042793	tpp1	1522.0535	0.814599971	0.16017448	5.08570399	3.66E-07	4.14E-06
ENSDARG00000032263	zgc:110224	139.483985	-1.437070137	0.28266715	-5.08396578	3.70E-07	4.17E-06
ENSDARG00000093862	si:ch211-274k16.2	47.1425212	1.988100518	0.39108816	5.08350991	3.71E-07	4.18E-06
ENSDARG00000010556	mmp25b	141.73444	-1.181828219	0.23250185	-5.08309178	3.71E-07	4.19E-06

Table S2. DEGs of WT vs. *terfa-/-*

ENSDARG00000110967	FP102169.1	21.3899593	3.093311246	0.6085868	5.08277742	3.72E-07	4.19E-06
ENSDARG00000078095	cipcb	1839.13789	0.578931134	0.11391565	5.08210379	3.73E-07	4.21E-06
ENSDARG00000007383	kcnk6	705.273189	0.705366262	0.13879885	5.08193148	3.74E-07	4.21E-06
ENSDARG00000062618	kcnj12b	575.789244	1.027111729	0.20215351	5.08085041	3.76E-07	4.23E-06
ENSDARG00000019564	asap2b	1970.06153	0.669967392	0.13188632	5.07988559	3.78E-07	4.25E-06
ENSDARG00000056395	onecut3a	215.683595	1.077992135	0.21219984	5.08007999	3.77E-07	4.25E-06
ENSDARG00000007886	slc35b2	829.918891	0.564820329	0.111203	5.07918252	3.79E-07	4.26E-06
ENSDARG00000044752	p2rx4a	128.228215	1.188590219	0.23403556	5.07867361	3.80E-07	4.27E-06
ENSDARG00000006501	cyp2x10.2	35.4265712	-3.180865035	0.62650727	-5.07713984	3.83E-07	4.31E-06
ENSDARG00000089767	rnf130	1663.64841	0.509682253	0.10039446	5.0767967	3.84E-07	4.31E-06
ENSDARG00000104893	si:ch211-207e19.2	20.9022389	3.155873165	0.62169208	5.07626406	3.85E-07	4.32E-06
ENSDARG00000062055	rnf38	2614.99111	-0.719949697	0.14182723	-5.0762446	3.85E-07	4.32E-06
ENSDARG00000093471	CR361551.1	44.8493558	-1.977372563	0.3895604	-5.07590749	3.86E-07	4.32E-06
ENSDARG00000059398	myef2	4571.22373	0.78248767	0.15416166	5.07576057	3.86E-07	4.32E-06
ENSDARG00000097770	si:ch211-167j9.4	162.050273	-1.285026086	0.25316145	-5.07591525	3.86E-07	4.32E-06
ENSDARG00000043226	nfixa	215.963117	-1.297951742	0.25580138	-5.0740608	3.89E-07	4.36E-06
ENSDARG00000015657	zgc:77112	70.7582392	-1.572367347	0.30993942	-5.07314407	3.91E-07	4.38E-06
ENSDARG00000099961	bnip3	289.502734	-0.820385171	0.16170345	-5.07339319	3.91E-07	4.38E-06
ENSDARG00000020326	tyk2	441.227159	0.800464489	0.15785257	5.07096274	3.96E-07	4.43E-06
ENSDARG00000077768	malt1	238.033946	1.21272113	0.23922347	5.06940697	3.99E-07	4.46E-06
ENSDARG00000052170	uap1	1008.29559	0.566295103	0.11174713	5.06764791	4.03E-07	4.50E-06
ENSDARG00000024047	tmem38a	10370.2869	0.631741134	0.12467818	5.06697424	4.04E-07	4.52E-06
ENSDARG00000111902	FO704772.1	35.8486937	2.956147884	0.58360176	5.06535125	4.08E-07	4.55E-06
ENSDARG00000079777	map6d1	190.923412	-1.238041177	0.24449518	-5.06366288	4.11E-07	4.59E-06
ENSDARG00000069922	pla1a	362.762049	0.696586424	0.1375701	5.06350166	4.12E-07	4.59E-06
ENSDARG00000074808	megf6b	1044.96705	0.718604022	0.14199054	5.06092903	4.17E-07	4.65E-06
ENSDARG00000052462	pisd	1171.82734	-0.614901178	0.12151273	-5.06038467	4.18E-07	4.66E-06
ENSDARG00000037916	cdk5r1a	314.531142	-0.837495668	0.16552845	-5.05952702	4.20E-07	4.68E-06
ENSDARG00000099069	bsk146	869.604747	-0.801589441	0.15849696	-5.05744367	4.25E-07	4.73E-06
ENSDARG00000017446	camk1db	627.783071	-0.904262653	0.17886066	-5.0556823	4.29E-07	4.77E-06
ENSDARG00000078261	nphp3	601.640205	0.789534736	0.15622181	5.05393399	4.33E-07	4.82E-06
ENSDARG00000024030	angptl2a	695.234716	0.863695604	0.17091935	5.05323474	4.34E-07	4.83E-06
ENSDARG00000020952	si:ch211-214j8.1	314.650251	1.503425723	0.29770276	5.05009	4.42E-07	4.91E-06
ENSDARG00000101334	ctsc	1340.15984	0.86361255	0.17101178	5.05001804	4.42E-07	4.91E-06
ENSDARG00000117427	AL772373.1	99.4702469	1.300664746	0.25764884	5.04820722	4.46E-07	4.95E-06
ENSDARG00000079839	arrdc1b	253.041145	0.956719645	0.18951456	5.04826459	4.46E-07	4.95E-06
ENSDARG00000029204	tyrp1a	2429.42042	0.721072914	0.14286292	5.04730619	4.48E-07	4.97E-06
ENSDARG00000104836	myom1b	12331.2995	0.679978097	0.13473824	5.0466602	4.50E-07	4.98E-06
ENSDARG00000070266	spock3	3499.57286	-0.600973886	0.11908794	-5.04647129	4.50E-07	4.98E-06
ENSDARG00000043102	lxn	980.011207	1.010900554	0.20030909	5.04670347	4.49E-07	4.98E-06
ENSDARG00000062319	si:dkey-103g5.3	1024.35998	-0.854367623	0.16927963	-5.04707864	4.49E-07	4.98E-06
ENSDARG00000078508	si:dkey-266m15.6	1587.82375	0.779698664	0.15451702	5.04603753	4.51E-07	4.99E-06
ENSDARG00000077054	gask1b	270.065702	1.013903529	0.20101136	5.0440111	4.56E-07	5.04E-06
ENSDARG00000052331	abcf2b	340.70418	0.716957586	0.14215917	5.04334383	4.57E-07	5.06E-06
ENSDARG00000037889	wnt9b	1022.30295	0.817033552	0.1620023	5.04334525	4.57E-07	5.06E-06
ENSDARG00000022438	cox6a1	4081.04555	0.576251947	0.11425833	5.04341297	4.57E-07	5.06E-06
ENSDARG00000015822	sesn3	1967.78342	0.610466176	0.12105905	5.04271409	4.59E-07	5.07E-06
ENSDARG00000053155	alg3	570.332922	0.633293248	0.12561535	5.04152741	4.62E-07	5.10E-06
ENSDARG00000095949	si:dkey-22i16.9	54.608305	2.83189532	0.56175581	5.04115002	4.63E-07	5.10E-06
ENSDARG00000087238	si:ch211-161h7.4	1040.02392	-0.880367897	0.17462948	-5.04134758	4.62E-07	5.10E-06
ENSDARG00000042627	nhsl1b	1702.11098	-0.504730749	0.10015519	-5.03948652	4.67E-07	5.15E-06
ENSDARG00000074779	alms1	1034.66854	-0.568461054	0.11281384	-5.03893025	4.68E-07	5.16E-06
ENSDARG00000057833	gulp1b	77.1525845	-1.464559236	0.29066904	-5.03858001	4.69E-07	5.17E-06
ENSDARG00000089399	tmem176l.2	266.191555	1.629539786	0.32347895	5.03754508	4.72E-07	5.19E-06
ENSDARG00000075169	bbs1	363.601179	-0.904653199	0.17962023	-5.0364772	4.74E-07	5.22E-06
ENSDARG00000078593	vwa8	1313.78611	0.591039369	0.11736786	5.03578544	4.76E-07	5.24E-06
ENSDARG00000029761	mpp6b	1880.62695	-1.210586269	0.24044247	-5.03482704	4.78E-07	5.26E-06
ENSDARG00000101421	si:dkeyp-80d11.10	14.9302203	6.094696127	1.21057995	5.0345259	4.79E-07	5.27E-06

Table S2. DEGs of WT vs. *terfa-/-*

ENSDARG00000017953	tp73	221.671452	0.910361759	0.1808548	5.03366107	4.81E-07	5.29E-06
ENSDARG00000006566	nlk1	2949.26914	-0.69637762	0.13846063	-5.02942705	4.92E-07	5.40E-06
ENSDARG00000038792	klf17	280.9829	-0.869001961	0.1727972	-5.02902812	4.93E-07	5.41E-06
ENSDARG00000075551	aifm5	109.353621	-1.619558494	0.32204452	-5.02898951	4.93E-07	5.41E-06
ENSDARG00000093024	si:ch211-213a13.2	95.6303932	1.560587858	0.3103561	5.02837831	4.95E-07	5.43E-06
ENSDARG00000077572	si:ch211-193k19.2	22.2100528	5.642866142	1.1227257	5.02604167	5.01E-07	5.48E-06
ENSDARG00000099555	foxo1a	1125.20437	0.818414425	0.16282305	5.02640412	5.00E-07	5.48E-06
ENSDARG00000023174	fez1	4677.63168	-0.685921428	0.13646842	-5.02622811	5.00E-07	5.48E-06
ENSDARG00000061697	ca14	73.0589856	-1.562262564	0.31089428	-5.0250605	5.03E-07	5.51E-06
ENSDARG00000039393	si:ch211-240l19.5	248.324007	-2.480314281	0.49370972	-5.02383118	5.07E-07	5.54E-06
ENSDARG00000098646	mthfd2	1448.51013	0.677663958	0.13492176	5.02264384	5.10E-07	5.58E-06
ENSDARG00000100651	PDCL3	1373.49406	0.593963125	0.11826711	5.02221713	5.11E-07	5.59E-06
ENSDARG00000030263	mfsd2b	170.260641	-0.928168955	0.18483717	-5.02154932	5.13E-07	5.60E-06
ENSDARG00000088168	ablim3	1123.61772	-0.541314976	0.10781909	-5.02058589	5.15E-07	5.63E-06
ENSDARG00000032318	mfsd6a	287.938292	0.949723444	0.18921255	5.01934711	5.18E-07	5.66E-06
ENSDARG00000056590	calca	340.112418	0.69925335	0.13932032	5.0190334	5.19E-07	5.67E-06
ENSDARG00000004402	elovl6	1305.20416	-0.63230345	0.12602292	-5.01736857	5.24E-07	5.71E-06
ENSDARG00000075713	shox2	642.08182	-0.823048539	0.16403142	-5.01762745	5.23E-07	5.71E-06
ENSDARG00000040009	palld	3824.51878	0.441377798	0.08802836	5.01404072	5.33E-07	5.81E-06
ENSDARG00000021309	rhoca	2133.3407	0.539869854	0.10768406	5.01346131	5.35E-07	5.83E-06
ENSDARG00000109648	si:ch211-147m6.1	182.651807	1.442191058	0.28778538	5.01134241	5.41E-07	5.89E-06
ENSDARG00000024827	rnf150a	103.632545	1.220479269	0.24364887	5.00917267	5.47E-07	5.95E-06
ENSDARG00000041217	xpo6	1883.91991	0.48124092	0.0960806	5.00872128	5.48E-07	5.96E-06
ENSDARG00000058644	dnajb2	359.908206	0.770837357	0.15393567	5.00752931	5.51E-07	6.00E-06
ENSDARG00000054619	fras1	7014.50999	0.733498733	0.14652148	5.00608326	5.55E-07	6.04E-06
ENSDARG00000101205	mbd1a	1158.82733	-0.555410707	0.11095042	-5.005936	5.56E-07	6.04E-06
ENSDARG00000075501	cdnf	450.762228	0.860705343	0.17198802	5.00444943	5.60E-07	6.09E-06
ENSDARG00000105036	arl6ip4	403.134601	-0.707540094	0.14140732	-5.0035606	5.63E-07	6.11E-06
ENSDARG00000024740	gng13a	77.8238349	-1.739441796	0.34764248	-5.00353637	5.63E-07	6.11E-06
ENSDARG00000103969	smyhc2	9775.95336	0.65917094	0.13177031	5.00242376	5.66E-07	6.14E-06
ENSDARG00000089885	slc16a12b	1724.81473	0.661419268	0.13222516	5.00221948	5.67E-07	6.15E-06
ENSDARG00000092662	dnaaf3	12.8664877	7.058950784	1.41136289	5.00151366	5.69E-07	6.17E-06
ENSDARG00000102997	rrn3	901.022108	0.588421308	0.11767171	5.00053337	5.72E-07	6.19E-06
ENSDARG00000101047	rtcb	962.088289	0.606729149	0.12137371	4.99885155	5.77E-07	6.25E-06
ENSDARG00000015312	klf12a	607.519285	0.797686982	0.15958443	4.99852642	5.78E-07	6.25E-06
ENSDARG00000087517	btbd3b	523.280597	-0.86213223	0.17257488	-4.99569951	5.86E-07	6.34E-06
ENSDARG00000077095	si:ch211-160o17.4	4927.3036	0.498082971	0.09970119	4.99575769	5.86E-07	6.34E-06
ENSDARG00000103441	znf1060	59.5969304	2.091552359	0.41871519	4.99516717	5.88E-07	6.36E-06
ENSDARG00000056209	myoz1a	3550.92641	0.666817686	0.13351599	4.99429102	5.91E-07	6.38E-06
ENSDARG00000079781	slitrk4	663.844023	-0.678514408	0.1359125	-4.99228865	5.97E-07	6.45E-06
ENSDARG00000088492	cntrl	890.561556	0.520412428	0.10428666	4.99021107	6.03E-07	6.51E-06
ENSDARG00000092225	si:dkeyp-13a3.10	350.749564	-0.743925288	0.14911123	-4.98906271	6.07E-07	6.55E-06
ENSDARG00000060622	si:ch73-14h1.2	387.670435	1.340543542	0.2687118	4.98877804	6.08E-07	6.55E-06
ENSDARG00000008720	lmo3	2364.17461	-0.853477805	0.17109648	-4.9882839	6.09E-07	6.57E-06
ENSDARG00000059714	arsk	52.4086965	2.548389118	0.51113983	4.9856986	6.17E-07	6.65E-06
ENSDARG00000017773	slc16a12a	375.678424	0.927350204	0.18599424	4.98590832	6.17E-07	6.65E-06
ENSDARG00000071496	zic6	248.288576	-1.09780356	0.22023539	-4.98468292	6.21E-07	6.68E-06
ENSDARG00000060149	ablim1a	629.466373	-0.692123177	0.1388693	-4.98398992	6.23E-07	6.70E-06
ENSDARG00000101485	si:ch1073-469d17.	518.737088	-2.665590302	0.53491112	-4.98323967	6.25E-07	6.73E-06
ENSDARG00000101316	ppp4r1	1273.38791	0.529125517	0.10620084	4.98231018	6.28E-07	6.76E-06
ENSDARG00000063661	nuak2	648.581176	0.78835006	0.15827056	4.98102775	6.32E-07	6.80E-06
ENSDARG00000100697	FQ323156.1	3317.54046	0.931265968	0.18700777	4.9798249	6.36E-07	6.84E-06
ENSDARG00000011777	cttn	4340.51402	0.52212979	0.10485911	4.97934598	6.38E-07	6.85E-06
ENSDARG00000025847	sox12	1895.66286	-1.695890241	0.34064522	-4.97846482	6.41E-07	6.88E-06
ENSDARG00000076895	flrt3	2253.2403	-0.498799925	0.10020155	-4.97796626	6.43E-07	6.90E-06
ENSDARG00000017931	ints6l	554.798183	-0.755620815	0.15180362	-4.97762064	6.44E-07	6.91E-06
ENSDARG00000087574	nox1	224.581099	0.987146741	0.19832417	4.9774405	6.44E-07	6.91E-06
ENSDARG00000091320	nlrc6	113.058019	-1.760788018	0.35382091	-4.97649503	6.47E-07	6.94E-06

Table S2. DEGs of WT vs. *terfa-/-*

ENSDARG00000058351	tapbpl	400.282553	-1.164048824	0.23391895	-4.97629135	6.48E-07	6.94E-06
ENSDARG00000077588	pdgfc	749.413656	0.594475098	0.11947015	4.97593006	6.49E-07	6.95E-06
ENSDARG00000096045	si:ch73-263o4.4	400.105245	0.736620892	0.14810292	4.97370934	6.57E-07	7.03E-06
ENSDARG00000068232	cbln12	61.4342084	-2.311724704	0.46491123	-4.97240027	6.61E-07	7.08E-06
ENSDARG00000104953	stt3a	8782.54875	0.479781927	0.09650471	4.97159076	6.64E-07	7.10E-06
ENSDARG00000103125	tsc2	2058.34351	0.551046646	0.11086003	4.97065195	6.67E-07	7.13E-06
ENSDARG00000025046	AL935186.1	73.2599131	1.860010514	0.37429252	4.96940341	6.72E-07	7.17E-06
ENSDARG00000015552	phactr4a	1450.97469	0.624726255	0.12571904	4.96922537	6.72E-07	7.17E-06
ENSDARG00000105362	faim2b	266.120451	-1.435592096	0.2888903	-4.96933299	6.72E-07	7.17E-06
ENSDARG00000070787	jupa	10183.8496	0.378220399	0.07611323	4.96918098	6.72E-07	7.17E-06
ENSDARG00000018881	apobec2a	1261.598	0.639465788	0.12867788	4.96950817	6.71E-07	7.17E-06
ENSDARG00000099309	si:zfos-169g10.3	135.407329	1.154213165	0.23230472	4.96853091	6.75E-07	7.19E-06
ENSDARG00000020759	elf1	1888.87571	0.518828595	0.10444279	4.96758674	6.78E-07	7.23E-06
ENSDARG00000077737	spsb3a	1152.79528	-0.573514473	0.11546498	-4.96699921	6.80E-07	7.24E-06
ENSDARG00000117493	BX511218.1	41.7634915	-2.05862858	0.41449027	-4.96665114	6.81E-07	7.25E-06
ENSDARG00000025404	ube2g2	1119.96113	0.545496515	0.10984149	4.96621559	6.83E-07	7.27E-06
ENSDARG00000097211	CR381647.3	70.0038579	-1.997547551	0.40229846	-4.96533728	6.86E-07	7.30E-06
ENSDARG00000004386	ELAPOR2	2123.11868	-0.741588697	0.14936993	-4.9647791	6.88E-07	7.31E-06
ENSDARG00000045947	hrc	5685.3072	0.601256462	0.12110264	4.96485036	6.88E-07	7.31E-06
ENSDARG00000039268	emc4	166.322267	-1.230000131	0.24775514	-4.96457968	6.89E-07	7.32E-06
ENSDARG00000069481	ghrh	23.07711	3.687638832	0.74289987	4.96384367	6.91E-07	7.34E-06
ENSDARG00000103002	nptna	2654.37528	-0.576830972	0.11621215	-4.96360299	6.92E-07	7.35E-06
ENSDARG00000088245	si:dkey-16p6.1	130.358542	-1.601489785	0.32274139	-4.96214571	6.97E-07	7.40E-06
ENSDARG00000058448	zrsr2	328.937595	-0.694267586	0.13991813	-4.961956	6.98E-07	7.40E-06
ENSDARG00000103810	cacna2d4a	103.439863	-1.622700731	0.32708469	-4.96110266	7.01E-07	7.43E-06
ENSDARG00000063079	ago3b	594.122514	0.724043881	0.14593875	4.96128602	7.00E-07	7.43E-06
ENSDARG00000029936	grtp1a	850.440235	-0.794818716	0.16024737	-4.95994853	7.05E-07	7.47E-06
ENSDARG00000018329	gc2	99.9078301	-1.228247108	0.24766183	-4.9593719	7.07E-07	7.49E-06
ENSDARG00000100582	si:ch211-195b11.3	5367.52016	0.625297158	0.12609179	4.9590634	7.08E-07	7.50E-06
ENSDARG00000077090	si:ch211-127b11.1	108.402252	1.604270078	0.32369278	4.95615034	7.19E-07	7.61E-06
ENSDARG00000104099	FP236513.1	225.519698	0.972460962	0.19626489	4.95483906	7.24E-07	7.66E-06
ENSDARG00000025549	ndufaf1	1141.47681	0.491926361	0.09928712	4.95458368	7.25E-07	7.66E-06
ENSDARG00000104384	agbl1	506.680018	0.881358321	0.17788247	4.95472285	7.24E-07	7.66E-06
ENSDARG00000054423	slc7a6	847.601706	0.833003644	0.16818962	4.95276491	7.32E-07	7.73E-06
ENSDARG00000040072	scpep1	1200.05002	0.820076916	0.16558024	4.95274617	7.32E-07	7.73E-06
ENSDARG00000062962	kmt2ba	3168.47679	-0.603909991	0.12198018	-4.9508863	7.39E-07	7.80E-06
ENSDARG00000089271	nexmifa	2389.25841	-0.721954607	0.14585685	-4.94974765	7.43E-07	7.84E-06
ENSDARG00000062562	egln2	397.819999	0.918273141	0.18556164	4.94861519	7.47E-07	7.88E-06
ENSDARG00000011400	tnnc1a	237.139999	0.91678896	0.18529083	4.94783761	7.50E-07	7.91E-06
ENSDARG00000057568	nefla	519.154781	0.771529661	0.15594251	4.9475264	7.52E-07	7.92E-06
ENSDARG00000053204	snx22	171.086213	1.242475455	0.25126457	4.94488916	7.62E-07	8.02E-06
ENSDARG00000008049	si:dkey-42i9.4	1208.89608	-0.723916318	0.14641868	-4.94415289	7.65E-07	8.05E-06
ENSDARG00000043821	klf7b	2305.34145	-0.508941804	0.1029514	-4.94351517	7.67E-07	8.07E-06
ENSDARG00000099002	creb5a	90.1448035	1.175967537	0.23787738	4.94358699	7.67E-07	8.07E-06
ENSDARG00000071331	ryr3	11024.8561	0.731772839	0.14809324	4.9412981	7.76E-07	8.16E-06
ENSDARG00000098037	CU927934.3	38.756218	2.294854236	0.46447686	4.94072892	7.78E-07	8.18E-06
ENSDARG00000029112	gem	330.311228	-1.159115725	0.23469173	-4.93888608	7.86E-07	8.25E-06
ENSDARG00000087110	akna	634.929292	-0.762725807	0.15442862	-4.93901854	7.85E-07	8.25E-06
ENSDARG00000023963	tpm4a	4235.48431	0.514020895	0.10408758	4.93835003	7.88E-07	8.27E-06
ENSDARG00000113045	CABZ01063543.1	79.4026179	-1.404453653	0.2845386	-4.93589849	7.98E-07	8.37E-06
ENSDARG00000102917	CR388095.1	42.376234	-2.483352278	0.50328198	-4.93431595	8.04E-07	8.43E-06
ENSDARG00000012192	cant1a	1112.07393	0.578060547	0.11715006	4.93435992	8.04E-07	8.43E-06
ENSDARG00000002564	syngr1a	1284.60733	-0.553590907	0.11223535	-4.93241121	8.12E-07	8.51E-06
ENSDARG00000016048	gpat3	1512.32374	0.644354914	0.13064712	4.93202541	8.14E-07	8.52E-06
ENSDARG00000069600	zgc:109889	1810.44886	-0.666388764	0.13520306	-4.92879951	8.27E-07	8.66E-06
ENSDARG00000054597	cnot6l	1320.18338	0.659353573	0.13380917	4.92756637	8.33E-07	8.71E-06
ENSDARG00000102225	si:ch211-205a14.7	14.6938695	3.571505103	0.72484375	4.92727584	8.34E-07	8.72E-06
ENSDARG00000074322	si:ch211-194m7.3	516.588581	0.685548135	0.13914782	4.92676153	8.36E-07	8.74E-06

Table S2. DEGs of WT vs. *terfa-/-*

ENSDARG00000055475	rps27.2	6625.71598	0.550701186	0.1118063	4.92549326	8.41E-07	8.80E-06
ENSDARG00000053301	insm1b	1130.15316	-1.012432475	0.20557902	-4.92478502	8.45E-07	8.82E-06
ENSDARG00000001859	dbx1b	160.842972	-1.451877413	0.29492755	-4.92282743	8.53E-07	8.91E-06
ENSDARG00000055128	armc4	188.309232	1.071696707	0.21771347	4.92250991	8.54E-07	8.92E-06
ENSDARG00000090912	npc2	2487.74314	0.659687048	0.13403173	4.92187217	8.57E-07	8.94E-06
ENSDARG00000060410	thbs2a	791.06899	0.910601754	0.18508916	4.9198007	8.66E-07	9.03E-06
ENSDARG00000101579	epc1a	1753.65052	-0.460722485	0.09364664	-4.91979743	8.66E-07	9.03E-06
ENSDARG00000020850	eef1a1l1	338101.57	0.437173291	0.08885842	4.91988622	8.66E-07	9.03E-06
ENSDARG00000092257	BX510992.1	71.7738982	-1.595055107	0.32424663	-4.91926498	8.69E-07	9.05E-06
ENSDARG00000022165	mgst1.2	802.564994	0.686915663	0.13964388	4.91905301	8.70E-07	9.06E-06
ENSDARG00000089831	si:dkey-207m2.4	142.419393	-2.051917234	0.41720818	-4.91820954	8.73E-07	9.09E-06
ENSDARG00000098769	slitrk6	943.668716	-0.57147974	0.11619982	-4.9180777	8.74E-07	9.09E-06
ENSDARG00000088810	prox3	514.036934	-1.063932395	0.21637522	-4.91707137	8.78E-07	9.14E-06
ENSDARG00000069313	oxa1l	1225.41496	-0.676908443	0.13768987	-4.91618177	8.82E-07	9.17E-06
ENSDARG00000058574	abcg2c	777.133498	0.619213813	0.12599459	4.9146064	8.90E-07	9.24E-06
ENSDARG00000016999	lin28a	209.076799	1.063834546	0.21652904	4.91312642	8.96E-07	9.31E-06
ENSDARG00000076611	fbxo21	447.569113	-0.640865055	0.1304496	-4.91274073	8.98E-07	9.32E-06
ENSDARG00000043531	jun	4492.55824	0.463349212	0.0943141	4.91283076	8.98E-07	9.32E-06
ENSDARG00000071871	glod5	447.83114	0.89768395	0.18274453	4.91223419	9.00E-07	9.34E-06
ENSDARG00000060980	atp8b1	1917.72719	0.547000675	0.1113605	4.91198096	9.02E-07	9.35E-06
ENSDARG00000104631	RNF14	35.4983037	2.021866402	0.41166912	4.91138706	9.04E-07	9.37E-06
ENSDARG00000058008	six3a	707.584401	-1.040041914	0.21178395	-4.91086286	9.07E-07	9.39E-06
ENSDARG00000054251	slc66a3	211.975347	0.918516104	0.1870324	4.91100002	9.06E-07	9.39E-06
ENSDARG00000003290	dab1b	315.072335	-1.348013405	0.27451088	-4.91060106	9.08E-07	9.40E-06
ENSDARG00000030913	cluha	4571.04439	0.487448023	0.09932985	4.90736684	9.23E-07	9.55E-06
ENSDARG00000034307	chrne	1270.9146	0.787359267	0.16045099	4.9071636	9.24E-07	9.56E-06
ENSDARG00000097660	trappc2	849.109639	0.587903717	0.11982964	4.90616267	9.29E-07	9.60E-06
ENSDARG00000098724	rgrb	1145.83031	0.753860899	0.15367237	4.90563737	9.31E-07	9.62E-06
ENSDARG00000104242	dcp2	1194.40651	-0.526041769	0.10725628	-4.90453126	9.37E-07	9.67E-06
ENSDARG00000019208	camsap1a	1416.4183	-0.677478221	0.13814334	-4.90416849	9.38E-07	9.69E-06
ENSDARG00000033466	tagln2	2916.4505	0.68746098	0.14020653	4.9032022	9.43E-07	9.73E-06
ENSDARG00000117474	CABZ01048956.1	31.5752548	3.146235677	0.64185122	4.90181461	9.50E-07	9.79E-06
ENSDARG00000028096	cldn23a	1169.99243	0.572165138	0.11672513	4.90181607	9.50E-07	9.79E-06
ENSDARG00000032317	tox	5164.28089	-0.757557623	0.15462903	-4.89919394	9.62E-07	9.92E-06
ENSDARG00000010108	bri3bp	973.024672	-0.726196437	0.1482643	-4.89798572	9.68E-07	9.97E-06
ENSDARG00000078598	si:ch1073-184j22.2	331.930532	0.945646476	0.19306338	4.89811428	9.68E-07	9.97E-06
ENSDARG00000086450	tdg.2	708.921108	-0.965074964	0.19704425	-4.89775765	9.69E-07	9.98E-06
ENSDARG00000102261	RSPH1	197.494978	0.95592191	0.1952443	4.89602969	9.78E-07	1.01E-05
ENSDARG00000042014	cyp11c1	239.641603	-1.11198871	0.22719304	-4.89446636	9.86E-07	1.01E-05
ENSDARG00000090337	pprc1	1065.30055	0.540071819	0.11034277	4.89449214	9.86E-07	1.01E-05
ENSDARG00000036840	krt15	6769.34766	0.908704912	0.18567076	4.89417341	9.87E-07	1.01E-05
ENSDARG00000117271	CR954298.1	22.9291284	7.028010995	1.43567392	4.89526966	9.82E-07	1.01E-05
ENSDARG00000099313	naga	1319.3782	0.900714948	0.18406031	4.89358586	9.90E-07	1.02E-05
ENSDARG00000008660	coro1b	3490.59315	-0.568049977	0.11609111	-4.89313918	9.92E-07	1.02E-05
ENSDARG00000078755	plppr4b	440.236198	-1.094640084	0.22371804	-4.89294499	9.93E-07	1.02E-05
ENSDARG00000090392	si:dkey-71l4.2	81.0759819	-1.570650979	0.32117286	-4.89036017	1.01E-06	1.03E-05
ENSDARG00000100308	SEMA4F	555.075352	-0.863816054	0.17658851	-4.89168885	1.00E-06	1.03E-05
ENSDARG00000017119	vps37a	849.948589	-0.497055135	0.10165008	-4.88986477	1.01E-06	1.03E-05
ENSDARG00000075709	ndufb3	2653.11295	0.58954206	0.12051734	4.8917614	9.99E-07	1.03E-05
ENSDARG00000008982	casq2	6068.67891	0.517638355	0.10583798	4.89085614	1.00E-06	1.03E-05
ENSDARG00000044142	acss1	616.859176	0.625025218	0.12779898	4.89069011	1.00E-06	1.03E-05
ENSDARG00000063612	antxr1c	1614.95174	0.588284882	0.12028899	4.89059617	1.01E-06	1.03E-05
ENSDARG00000103529	snx1b	611.867406	-0.662180127	0.13541534	-4.8899934	1.01E-06	1.03E-05
ENSDARG00000038476	si:dkeyp-68b7.7	230.966393	-0.827622375	0.16933818	-4.88739389	1.02E-06	1.04E-05
ENSDARG00000093354	si:ch211-57i17.2	39.4484291	2.839261366	0.58080369	4.88850439	1.02E-06	1.04E-05
ENSDARG00000099221	ppp2r2bb	1299.91854	-0.940975379	0.19254277	-4.88709807	1.02E-06	1.04E-05
ENSDARG00000063133	slc4a10a	5365.61654	-0.635623636	0.13005663	-4.8872836	1.02E-06	1.04E-05
ENSDARG00000042054	zgc:171971	306.222942	-0.82741488	0.16931792	-4.8867531	1.03E-06	1.05E-05

Table S2. DEGs of WT vs. *terfa-/-*

ENSDARG00000093768	prr18	1533.2415	-1.005872053	0.20597603	-4.88344238	1.04E-06	1.06E-05
ENSDARG00000058220	tada3l	1171.78761	-0.55755084	0.11414113	-4.88474943	1.04E-06	1.06E-05
ENSDARG00000100782	f7l	312.818042	1.056486897	0.21651076	4.8796045	1.06E-06	1.08E-05
ENSDARG00000011521	upb1	537.888037	0.873742858	0.17902523	4.88055699	1.06E-06	1.08E-05
ENSDARG00000011821	plod2	1585.88314	0.609310512	0.12485037	4.88032623	1.06E-06	1.08E-05
ENSDARG00000070473	parp6b	866.779131	-0.606679677	0.12434177	-4.87913018	1.07E-06	1.08E-05
ENSDARG00000015947	matn4	30938.4323	0.420345665	0.08615567	4.87890883	1.07E-06	1.09E-05
ENSDARG00000090814	si:dkey-18a10.3	150.739823	1.444804577	0.29621318	4.87758375	1.07E-06	1.09E-05
ENSDARG00000103937	ndrg4	9956.70438	-0.752897112	0.15432183	-4.87874655	1.07E-06	1.09E-05
ENSDARG00000012581	AL590149.1	278.297889	-0.937608686	0.19229309	-4.87593538	1.08E-06	1.10E-05
ENSDARG00000100560	zfpm2b	655.526488	-1.28117665	0.26271104	-4.87675216	1.08E-06	1.10E-05
ENSDARG00000063634	hpse	247.061508	-0.869435696	0.17828521	-4.87665632	1.08E-06	1.10E-05
ENSDARG00000005891	cyb5r3	1257.77209	0.559951702	0.11489387	4.87364314	1.10E-06	1.11E-05
ENSDARG00000063518	zgc:153913	1465.95555	0.676006677	0.13869377	4.87409535	1.09E-06	1.11E-05
ENSDARG00000113899	zgc:77650	1223.60508	0.499647444	0.10250924	4.87417001	1.09E-06	1.11E-05
ENSDARG00000089517	zgc:92594	205.070919	-1.069849191	0.21957864	-4.87228259	1.10E-06	1.12E-05
ENSDARG00000058658	si:dkey-178e17.3	248.686188	1.125173232	0.23099314	4.87102437	1.11E-06	1.12E-05
ENSDARG00000010948	kif11	3103.14596	-1.063526156	0.21830365	-4.8717746	1.11E-06	1.12E-05
ENSDARG00000094677	si:dkey-92j12.5	368.126439	-0.907758202	0.18645021	-4.86863595	1.12E-06	1.14E-05
ENSDARG00000060001	mettl9	666.193485	0.733721185	0.15071413	4.86829715	1.13E-06	1.14E-05
ENSDARG00000059399	si:ch211-160o17.6	68.6782343	-1.96364307	0.40344358	-4.86720619	1.13E-06	1.14E-05
ENSDARG00000093068	c3b.1	3231.44213	0.68158451	0.14000422	4.86831413	1.13E-06	1.14E-05
ENSDARG00000062132	cyp4v8	552.89572	-0.959673646	0.19721986	-4.86600926	1.14E-06	1.15E-05
ENSDARG00000090447	mtbp	194.7431	-0.995520811	0.20456378	-4.86655467	1.14E-06	1.15E-05
ENSDARG00000102528	AL935126.1	45.5538341	2.607451958	0.53602856	4.86438995	1.15E-06	1.16E-05
ENSDARG00000105190	rhbdl3	436.067858	-0.689094673	0.14165991	-4.86442988	1.15E-06	1.16E-05
ENSDARG00000058090	cfap57	83.2965015	1.503298825	0.30904771	4.86429367	1.15E-06	1.16E-05
ENSDARG00000091683	cnrip1a	1614.54522	-0.776555461	0.15962836	-4.86477136	1.15E-06	1.16E-05
ENSDARG00000044062	ctbp2a	4275.50876	-0.668942449	0.13750131	-4.86498951	1.14E-06	1.16E-05
ENSDARG00000053803	klhl43	2840.97458	0.706053249	0.14512365	4.86518387	1.14E-06	1.16E-05
ENSDARG00000102899	cremb	39.2682103	2.044701284	0.42045023	4.86312323	1.16E-06	1.16E-05
ENSDARG00000008287	fam114a1	841.518207	0.569117681	0.1170368	4.86272431	1.16E-06	1.17E-05
ENSDARG00000052652	fermt1	1042.27393	0.614204167	0.12632253	4.86219005	1.16E-06	1.17E-05
ENSDARG00000097574	CR847898.3	19.7146063	3.769728568	0.77561854	4.86028679	1.17E-06	1.18E-05
ENSDARG00000070239	knl1	1084.89856	-0.997720082	0.20531723	-4.85940738	1.18E-06	1.18E-05
ENSDARG00000096851	znf1143	251.001054	1.029693467	0.21194708	4.85825749	1.18E-06	1.19E-05
ENSDARG00000043279	aqp12	1719.38841	0.522304914	0.10749883	4.8587033	1.18E-06	1.19E-05
ENSDARG00000044365	angptl3	739.280408	0.817630337	0.16833984	4.85702232	1.19E-06	1.20E-05
ENSDARG00000097620	si:ch211-210b2.1	596.955591	0.847863918	0.17458395	4.85648264	1.19E-06	1.20E-05
ENSDARG00000070914	dusp6	5419.75324	0.512123862	0.10548224	4.85507176	1.20E-06	1.21E-05
ENSDARG00000094422	si:dkey-12l12.1	410.443031	-0.776611708	0.16003109	-4.85288032	1.22E-06	1.22E-05
ENSDARG00000099827	CABZ01000633.1	14.9642591	6.384199933	1.31558683	4.85273931	1.22E-06	1.22E-05
ENSDARG00000007976	si:ch211-220f16.2	1236.00906	-0.511425074	0.10545276	-4.84980276	1.24E-06	1.24E-05
ENSDARG00000074806	afap1l2	993.055168	0.693430771	0.14297118	4.85014379	1.23E-06	1.24E-05
ENSDARG00000078363	mcoln3a	143.690759	1.097990072	0.22650771	4.84747339	1.25E-06	1.25E-05
ENSDARG00000088900	si:dkey-21o19.2	110.14801	-1.994870801	0.41154765	-4.84724144	1.25E-06	1.25E-05
ENSDARG00000096519	irbpl	16.785616	6.369553097	1.31437281	4.84607796	1.26E-06	1.26E-05
ENSDARG00000087921	plce1	1024.91908	0.598239157	0.12347053	4.8451979	1.26E-06	1.27E-05
ENSDARG00000075846	kcnip2	34.3871803	-2.26258648	0.4671135	-4.84376168	1.27E-06	1.27E-05
ENSDARG00000026855	cacna2d4a	153.832535	-1.377079687	0.28435675	-4.84278887	1.28E-06	1.28E-05
ENSDARG00000112928	CR450780.2	31.1540849	2.580291927	0.53272974	4.843529	1.28E-06	1.28E-05
ENSDARG00000078572	stox2b	2381.39805	-0.497434452	0.10274001	-4.84168209	1.29E-06	1.29E-05
ENSDARG00000105641	si:ch211-98n17.5	946.146032	0.667679794	0.13797745	4.83905	1.30E-06	1.30E-05
ENSDARG00000055283	id2a	4255.57988	-0.566404354	0.11712806	-4.83577001	1.33E-06	1.32E-05
ENSDARG00000068401	yap1	4125.52265	0.515196934	0.10652395	4.83644214	1.32E-06	1.32E-05
ENSDARG00000036168	nfatc1	699.516173	0.599665085	0.12398748	4.83649716	1.32E-06	1.32E-05
ENSDARG00000079323	rgmb	2341.70008	-0.547036193	0.11313424	-4.83528403	1.33E-06	1.33E-05
ENSDARG00000019062	arpc5b	1545.51843	0.589265478	0.12187752	4.83489879	1.33E-06	1.33E-05

Table S2. DEGs of WT vs. *terfa-/-*

ENSDARG00000068515	chs1	321.599908	2.74728534	0.56816403	4.83537361	1.33E-06	1.33E-05
ENSDARG00000088347	sp1	674.582431	-0.736004677	0.15221454	-4.83531139	1.33E-06	1.33E-05
ENSDARG00000105689	znf1009	228.299046	-0.828227825	0.17135859	-4.83330198	1.34E-06	1.34E-05
ENSDARG00000059556	recql5	665.112418	0.825523879	0.17084337	4.83205112	1.35E-06	1.34E-05
ENSDARG00000021938	smad9	492.265534	0.83090126	0.17193183	4.83273678	1.35E-06	1.34E-05
ENSDARG00000093484	si:dkey-95p16.2	235.514581	-1.010402629	0.20911317	-4.83184605	1.35E-06	1.35E-05
ENSDARG00000062707	plat	292.663996	0.842060614	0.174327	4.83035121	1.36E-06	1.35E-05
ENSDARG00000046030	zgc:110339	2584.68213	0.668577411	0.13838453	4.83130152	1.36E-06	1.35E-05
ENSDARG00000033616	selenon	1092.04156	0.55444567	0.11479357	4.82993678	1.37E-06	1.36E-05
ENSDARG00000016815	casc1	60.4378033	1.492986639	0.30926015	4.82760762	1.38E-06	1.37E-05
ENSDARG00000104832	CU914164.1	20.9975678	-3.242022483	0.67181413	-4.82577302	1.39E-06	1.38E-05
ENSDARG00000043820	nme2a	367.379492	-2.212322446	0.45847496	-4.82539425	1.40E-06	1.39E-05
ENSDARG00000069280	tsen15	319.969452	-1.025523654	0.2126009	-4.82370319	1.41E-06	1.40E-05
ENSDARG00000043624	slc66a1	664.101148	0.774436159	0.16055796	4.82340544	1.41E-06	1.40E-05
ENSDARG00000057879	p3h3	755.902344	0.63388853	0.13145559	4.8220737	1.42E-06	1.41E-05
ENSDARG00000037121	mat2ab	735.026025	0.705606024	0.14640874	4.81942558	1.44E-06	1.42E-05
ENSDARG00000077777	tmsb4x	26888.1258	0.706069028	0.14650591	4.81938942	1.44E-06	1.42E-05
ENSDARG00000041155	morf4l1	1611.62053	-0.561777616	0.11652822	-4.8209576	1.43E-06	1.42E-05
ENSDARG00000079372	si:ch211-264f5.6	1990.94274	0.67145546	0.13928002	4.82090296	1.43E-06	1.42E-05
ENSDARG00000011170	ndrg2	9420.84212	-0.501627663	0.10414518	-4.81661905	1.46E-06	1.44E-05
ENSDARG00000096031	si:ch73-6k14.2	127.410332	-2.062953136	0.42823346	-4.81735624	1.45E-06	1.44E-05
ENSDARG00000013528	mcm9	260.901214	-0.897376109	0.18630868	-4.81660931	1.46E-06	1.44E-05
ENSDARG00000091598	si:ch73-91k6.2	369.903847	0.978269248	0.20305291	4.81780458	1.45E-06	1.44E-05
ENSDARG00000101910	pcdh20	600.88416	0.783097776	0.16254662	4.81768121	1.45E-06	1.44E-05
ENSDARG00000020573	ddx3xa	6749.54922	-0.442371853	0.09185902	-4.81576921	1.47E-06	1.45E-05
ENSDARG00000073766	tnni1d	502.222429	-1.23924947	0.257413	-4.81424593	1.48E-06	1.46E-05
ENSDARG00000045019	aamp	2944.73189	0.469695315	0.09757214	4.81382614	1.48E-06	1.46E-05
ENSDARG00000021787	abcb5	4804.43562	0.587949244	0.12212776	4.81421449	1.48E-06	1.46E-05
ENSDARG00000067701	myoz3a	482.855994	1.276411839	0.26512369	4.81440124	1.48E-06	1.46E-05
ENSDARG00000058537	si:dkey-102c8.2	55.0177692	2.313450911	0.48054045	4.81426883	1.48E-06	1.46E-05
ENSDARG00000013441	hey2	200.996781	-0.9378319	0.19487937	-4.81237148	1.49E-06	1.47E-05
ENSDARG00000074415	zgc:171482	225.228342	-0.936224731	0.19454803	-4.81230642	1.49E-06	1.47E-05
ENSDARG00000061328	cdon	5413.61139	0.408824609	0.08495054	4.81250157	1.49E-06	1.47E-05
ENSDARG00000079530	si:dkey-17m8.1	727.985412	0.911173928	0.18944236	4.80976867	1.51E-06	1.49E-05
ENSDARG00000053479	gdf6a	362.186781	0.718296028	0.14935717	4.80925055	1.51E-06	1.49E-05
ENSDARG00000099203	atp1b2a	9466.91035	-0.572543627	0.11904921	-4.80930207	1.51E-06	1.49E-05
ENSDARG00000011488	sirt2	1328.11735	0.586205506	0.12192024	4.8081064	1.52E-06	1.50E-05
ENSDARG00000091377	galn	190.089886	1.382633045	0.28762608	4.80705043	1.53E-06	1.50E-05
ENSDARG00000044074	loxl2b	2490.03085	0.52852874	0.10998619	4.80540987	1.54E-06	1.51E-05
ENSDARG00000044852	wbp2nl	2906.24796	0.482632438	0.10043935	4.80521287	1.55E-06	1.51E-05
ENSDARG00000087927	nudt9	866.896316	0.592398765	0.12325973	4.80610153	1.54E-06	1.51E-05
ENSDARG00000059073	gsc	357.626678	-0.708760324	0.14745019	-4.80677789	1.53E-06	1.51E-05
ENSDARG00000032802	ktn1	7550.62561	0.367473956	0.07646832	4.80557119	1.54E-06	1.51E-05
ENSDARG00000058367	abhd14a	299.570147	-0.846343606	0.17609118	-4.8062804	1.54E-06	1.51E-05
ENSDARG00000069279	elovl7a	850.300785	0.753214813	0.15675497	4.80504598	1.55E-06	1.52E-05
ENSDARG00000012204	CDK18	404.860564	-0.805567923	0.16766468	-4.80463708	1.55E-06	1.52E-05
ENSDARG00000035471	si:dkey-220k22.1	892.377057	0.521232631	0.1085352	4.80242947	1.57E-06	1.53E-05
ENSDARG00000013575	rfx2	1096.2336	0.52775212	0.10990911	4.80171414	1.57E-06	1.54E-05
ENSDARG00000041145	paqr5a	88.3951811	-1.32414312	0.27580618	-4.80099141	1.58E-06	1.54E-05
ENSDARG00000057027	rtn2b	1333.91508	0.665004003	0.13848262	4.80207564	1.57E-06	1.54E-05
ENSDARG00000020840	mcrs1	1416.01822	-0.577050719	0.12018071	-4.80152519	1.57E-06	1.54E-05
ENSDARG00000042777	ndufa11	1594.00926	0.719272176	0.14982261	4.80082517	1.58E-06	1.54E-05
ENSDARG00000014804	cacna2d1a	1637.93552	0.631245664	0.13153095	4.7992179	1.59E-06	1.56E-05
ENSDARG00000098592	zgc:92140	968.612679	-0.535150126	0.11153391	-4.79809369	1.60E-06	1.56E-05
ENSDARG00000057698	ctsd	8443.47641	0.722032696	0.15046781	4.79858582	1.60E-06	1.56E-05
ENSDARG00000068168	hes2.2	88.5530544	-2.012690385	0.41954981	-4.79726206	1.61E-06	1.57E-05
ENSDARG00000013976	anxa13	3388.2995	0.465695309	0.09709074	4.79649566	1.61E-06	1.57E-05
ENSDARG00000053365	rpl31	41405.7077	0.630797722	0.13153267	4.79574942	1.62E-06	1.58E-05

Table S2. DEGs of WT vs. *terfa-/-*

ENSDARG00000036944	ensaa	212.221286	-0.895877875	0.18681144	-4.79562629	1.62E-06	1.58E-05
ENSDARG00000035872	hsd17b12b	1139.26277	-0.558324037	0.11647928	-4.79333341	1.64E-06	1.59E-05
ENSDARG00000036764	hax1	694.042793	0.720757989	0.15033489	4.79434929	1.63E-06	1.59E-05
ENSDARG00000099927	dpf1	534.336896	-0.804747292	0.1678405	-4.79471453	1.63E-06	1.59E-05
ENSDARG00000102251	pdzph1	71.2671065	-1.963458707	0.40960874	-4.7934981	1.64E-06	1.59E-05
ENSDARG00000075768	sdhb	6092.77311	0.641799179	0.13386059	4.79453413	1.63E-06	1.59E-05
ENSDARG00000117216	LO017718.1	182.768854	2.58119205	0.53865536	4.79191752	1.65E-06	1.60E-05
ENSDARG00000069815	dnali1	61.4652896	1.443216577	0.3011247	4.79275387	1.65E-06	1.60E-05
ENSDARG00000043569	dram2a	29.5288508	2.382695347	0.49710828	4.79311135	1.64E-06	1.60E-05
ENSDARG00000018303	etv4	1039.28349	0.599897818	0.12517487	4.79247807	1.65E-06	1.60E-05
ENSDARG00000068516	hapln1b	1167.97931	-0.802171152	0.16738521	-4.79236567	1.65E-06	1.60E-05
ENSDARG00000035810	rgcc	1527.32528	0.728205713	0.15201525	4.7903464	1.66E-06	1.61E-05
ENSDARG00000075046	rnf151	28.7930488	-2.819677687	0.58864271	-4.79013438	1.67E-06	1.62E-05
ENSDARG00000015611	rasl11b	493.619415	0.917796683	0.19166888	4.78844912	1.68E-06	1.63E-05
ENSDARG00000016528	wdcp	277.115123	-1.065581466	0.22252769	-4.7885343	1.68E-06	1.63E-05
ENSDARG00000068830	zgc:172139	217.048783	1.00234377	0.20939035	4.78696259	1.69E-06	1.64E-05
ENSDARG00000015732	cax2	337.306673	0.720911487	0.15064032	4.78564747	1.70E-06	1.65E-05
ENSDARG00000101441	lima1a	2574.60973	0.592235745	0.12377257	4.78487085	1.71E-06	1.66E-05
ENSDARG00000095170	lrrfip1b	934.867809	-0.721553646	0.15088012	-4.78229776	1.73E-06	1.67E-05
ENSDARG00000042826	egr2b	204.509213	-0.949783171	0.19856745	-4.78317663	1.73E-06	1.67E-05
ENSDARG00000078785	tmem258	184.869065	1.336178897	0.27936427	4.78292694	1.73E-06	1.67E-05
ENSDARG00000097006	CR854839.1	421.060781	0.767472201	0.16052249	4.78108841	1.74E-06	1.68E-05
ENSDARG00000043077	nisch	1258.16079	-0.494565476	0.10346671	-4.77994772	1.75E-06	1.69E-05
ENSDARG00000022904	skilb	659.909075	-0.609033792	0.12743537	-4.77915828	1.76E-06	1.70E-05
ENSDARG00000090408	ERBB4	225.900419	1.064233229	0.22275803	4.77753036	1.77E-06	1.71E-05
ENSDARG00000098837	tgm5l	70.2170936	1.694671425	0.35480277	4.77637599	1.78E-06	1.72E-05
ENSDARG00000101089	gart	3171.01342	0.749631427	0.15696668	4.77573603	1.79E-06	1.72E-05
ENSDARG00000077114	arhgef16	789.022202	0.744898462	0.15596755	4.77598373	1.79E-06	1.72E-05
ENSDARG00000055158	prox1a	2133.55992	-0.893370384	0.18704767	-4.77616409	1.79E-06	1.72E-05
ENSDARG00000090126	si:ch211-105f12.2	38.9136788	-2.914131236	0.61003819	-4.77696523	1.78E-06	1.72E-05
ENSDARG00000117445	CABZ01067151.2	109.875942	-1.535235818	0.32153743	-4.77467223	1.80E-06	1.73E-05
ENSDARG00000115199	dicp1.3-4	25.7784091	-3.217425396	0.67402641	-4.77344115	1.81E-06	1.74E-05
ENSDARG00000071196	cavin2b	3776.55333	0.416296003	0.08721621	4.77314926	1.81E-06	1.74E-05
ENSDARG00000077022	fam131a	205.914884	-0.875150093	0.18335406	-4.77300648	1.81E-06	1.74E-05
ENSDARG00000103309	BX664625.5	14.1035736	5.118143268	1.07216893	4.77363513	1.81E-06	1.74E-05
ENSDARG00000076639	esamb	206.23575	-1.083426059	0.22704606	-4.77183371	1.83E-06	1.75E-05
ENSDARG00000101170	rab42b	102.462904	1.400299148	0.29352037	4.77070524	1.84E-06	1.76E-05
ENSDARG00000056175	scrt2	1117.97307	-0.939081238	0.19689093	-4.76955059	1.85E-06	1.77E-05
ENSDARG00000052609	CU468164.1	16.9759558	-5.170418085	1.08386491	-4.77035289	1.84E-06	1.77E-05
ENSDARG00000100614	zgc:171497	120.870536	-1.165590545	0.24434997	-4.77016857	1.84E-06	1.77E-05
ENSDARG00000099152	si:dkey-190j3.2	38.9414881	1.979645888	0.41514788	4.76853186	1.86E-06	1.78E-05
ENSDARG00000020863	trim25l	237.517882	0.776826666	0.16289928	4.7687544	1.85E-06	1.78E-05
ENSDARG00000010425	scara5	626.288197	0.880554303	0.18482268	4.76431958	1.89E-06	1.81E-05
ENSDARG00000077018	socs7	841.785347	-0.774209802	0.16246166	-4.76549234	1.88E-06	1.81E-05
ENSDARG00000002710	ncl	20825.4218	-0.434960979	0.091282	-4.76502479	1.89E-06	1.81E-05
ENSDARG00000078416	zeb2b	2425.54324	-0.803704939	0.16871919	-4.7635656	1.90E-06	1.82E-05
ENSDARG00000043680	ylpm1	2932.78007	0.514163771	0.10792659	4.76401372	1.90E-06	1.82E-05
ENSDARG00000016936	hmcn1	2497.00727	0.810933162	0.17023396	4.76363914	1.90E-06	1.82E-05
ENSDARG00000099825	zgc:174944	52.3690032	1.979514342	0.4156476	4.76248229	1.91E-06	1.83E-05
ENSDARG00000054749	lmo4b	1764.64307	-0.552536592	0.11603969	-4.7616172	1.92E-06	1.83E-05
ENSDARG00000059794	kdm6al	2557.50473	-0.518324607	0.1088475	-4.76193387	1.92E-06	1.83E-05
ENSDARG00000002299	arsh	346.076286	1.168063741	0.24530135	4.76175009	1.92E-06	1.83E-05
ENSDARG00000112454	FO744833.2	118.270886	1.3570823	0.28500631	4.76158678	1.92E-06	1.83E-05
ENSDARG00000104613	znf1179	1925.03693	1.541574954	0.32372698	4.76196007	1.92E-06	1.83E-05
ENSDARG00000024433	pvalb4	9956.9203	0.839954702	0.17650958	4.75869197	1.95E-06	1.86E-05
ENSDARG00000086856	stk35	852.191367	0.743885679	0.15632284	4.75864989	1.95E-06	1.86E-05
ENSDARG00000092471	si:dkey-7j22.1	41.2479697	2.052200715	0.4313925	4.75715437	1.96E-06	1.87E-05
ENSDARG00000012848	arih2	2781.03501	-0.396778889	0.08342525	-4.75610066	1.97E-06	1.88E-05

Table S2. DEGs of WT vs. *terfa-/-*

ENSDARG00000055751	fosb	139.677931	-1.135537043	0.23874344	-4.75630677	1.97E-06	1.88E-05
ENSDARG00000075842	pigt	160.051042	-1.059979184	0.22289084	-4.75559782	1.98E-06	1.88E-05
ENSDARG00000032919	chchd3a	1645.67825	0.503150502	0.10581091	4.75518552	1.98E-06	1.89E-05
ENSDARG00000099169	cxcl8b.3	51.6017648	1.759124649	0.37001899	4.75414701	1.99E-06	1.89E-05
ENSDARG00000035629	parvab	252.453307	0.798447769	0.16792135	4.75489141	1.99E-06	1.89E-05
ENSDARG00000079029	dhx32b	609.743677	-1.207136777	0.25385058	-4.75530431	1.98E-06	1.89E-05
ENSDARG00000078989	alpk3a	565.97033	0.872379608	0.18349072	4.75435282	1.99E-06	1.89E-05
ENSDARG00000037867	atp5mf	4588.34162	0.542761969	0.11418914	4.75318391	2.00E-06	1.90E-05
ENSDARG00000017398	slc44a5a	89.2489154	-1.321331793	0.27795086	-4.75383246	2.00E-06	1.90E-05
ENSDARG00000105631	CABP4	67.7616362	-2.542516671	0.53503999	-4.75201245	2.01E-06	1.91E-05
ENSDARG00000002463	arhgef37	384.975358	0.675117113	0.14204974	4.75268125	2.01E-06	1.91E-05
ENSDARG00000099006	CABZ01029938.1	158.151798	1.678521557	0.35319052	4.75245356	2.01E-06	1.91E-05
ENSDARG00000102835	CABZ01021435.1	31.4103828	2.388885043	0.50290441	4.75017719	2.03E-06	1.92E-05
ENSDARG00000030896	foxq1a	1158.77438	-0.782468381	0.16472466	-4.75015947	2.03E-06	1.92E-05
ENSDARG00000060065	ubap2b	7155.82736	0.39533845	0.08321725	4.75067888	2.03E-06	1.92E-05
ENSDARG00000101010	hpdl	85.88223	-1.504210231	0.31665218	-4.75035486	2.03E-06	1.92E-05
ENSDARG00000104561	znf1081	74.605253	1.795700975	0.37823324	4.74760227	2.06E-06	1.95E-05
ENSDARG00000086393	dbx1a	154.29194	-1.622188138	0.34169216	-4.74751349	2.06E-06	1.95E-05
ENSDARG00000017391	unc13ba	392.662444	-1.072545085	0.22591349	-4.74759202	2.06E-06	1.95E-05
ENSDARG00000093659	entpd5b	1561.97065	0.560355211	0.11807459	4.74577329	2.08E-06	1.96E-05
ENSDARG00000013615	pbx3b	4410.03228	-0.570348164	0.12017178	-4.74610744	2.07E-06	1.96E-05
ENSDARG00000038863	babam2	789.950194	-0.559393378	0.11785182	-4.7465825	2.07E-06	1.96E-05
ENSDARG00000052565	cenpi	458.715032	-0.825532988	0.1739421	-4.74602169	2.07E-06	1.96E-05
ENSDARG00000041747	sspn	516.801005	0.739870676	0.15590249	4.74572705	2.08E-06	1.96E-05
ENSDARG00000053241	sts	191.733652	1.222805671	0.25785124	4.74229114	2.11E-06	1.99E-05
ENSDARG00000075222	amer2	1654.25047	-0.833975726	0.17582637	-4.74317771	2.10E-06	1.99E-05
ENSDARG00000020084	tg	757.17223	0.914488028	0.19285402	4.74186649	2.12E-06	2.00E-05
ENSDARG00000026229	prnpa	140.013835	-1.677931352	0.3539197	-4.7409945	2.13E-06	2.00E-05
ENSDARG00000060830	fam83hb	1195.13711	0.489811289	0.1033165	4.74088166	2.13E-06	2.00E-05
ENSDARG00000099839	si:ch211-9d9.1	160.459889	-0.957969553	0.20207747	-4.74060548	2.13E-06	2.01E-05
ENSDARG00000093936	si:dkeyp-1h4.6	431.311714	0.806044656	0.17005585	4.73988203	2.14E-06	2.01E-05
ENSDARG00000040705	glsb	1418.27317	0.672957197	0.1420132	4.73869456	2.15E-06	2.02E-05
ENSDARG00000040214	scrt1b	468.803484	-0.801547781	0.16917052	-4.7381057	2.16E-06	2.03E-05
ENSDARG00000103403	sar1b	4260.09409	0.475447811	0.10035125	4.73783651	2.16E-06	2.03E-05
ENSDARG00000115405	hbbe1.2	11174.66	0.837216506	0.17673105	4.73723489	2.17E-06	2.04E-05
ENSDARG00000095901	col18a1b	597.37921	-0.932920414	0.19694629	-4.7369281	2.17E-06	2.04E-05
ENSDARG00000012144	emc7	1737.99555	-0.543434872	0.1147361	-4.73638974	2.18E-06	2.04E-05
ENSDARG00000026052	ccdc3a	131.204152	1.052279085	0.22226093	4.73443132	2.20E-06	2.06E-05
ENSDARG00000068397	tns2b	1375.46229	0.529226678	0.11179038	4.73409858	2.20E-06	2.06E-05
ENSDARG00000099273	zgc:103508	432.934101	-0.835558094	0.17646909	-4.73486949	2.19E-06	2.06E-05
ENSDARG00000088315	si:ch211-103f14.3	362.58071	-0.776548521	0.16403008	-4.73418376	2.20E-06	2.06E-05
ENSDARG00000043722	cpa4	563.225324	-2.506137307	0.52939979	-4.73392201	2.20E-06	2.06E-05
ENSDARG00000077096	fndc7a	421.795519	-0.838547528	0.17721443	-4.7318243	2.23E-06	2.08E-05
ENSDARG00000062790	CU639469.1	3911.54693	-0.521389336	0.11018778	-4.73182559	2.23E-06	2.08E-05
ENSDARG00000054332	trim66	199.456663	-0.965214098	0.20399289	-4.73160664	2.23E-06	2.09E-05
ENSDARG00000021948	tnc	17605.0243	0.561182836	0.11863033	4.73051736	2.24E-06	2.09E-05
ENSDARG00000067889	gltpd2	88.0502246	1.426867477	0.30161656	4.73073322	2.24E-06	2.09E-05
ENSDARG00000057648	dnttip2	1292.39094	0.503882238	0.10650973	4.73085664	2.24E-06	2.09E-05
ENSDARG00000075014	sqstm1	410.505581	0.643147382	0.13595226	4.73068543	2.24E-06	2.09E-05
ENSDARG00000074303	phf12a	1323.24992	-0.595169156	0.12580216	-4.73099303	2.23E-06	2.09E-05
ENSDARG00000111812	CABZ01073083.1	132.321537	1.058163171	0.22369507	4.7303821	2.24E-06	2.09E-05
ENSDARG00000018109	mafga	125.256251	-1.470726592	0.31101432	-4.72880671	2.26E-06	2.11E-05
ENSDARG00000069297	upf3a	1073.28661	-0.655815634	0.13868035	-4.72897298	2.26E-06	2.11E-05
ENSDARG00000035556	rps6ka3a	2014.78048	0.450694094	0.09533979	4.72724009	2.28E-06	2.12E-05
ENSDARG00000053483	zgc:113054	1853.25379	0.697707343	0.14757945	4.72767275	2.27E-06	2.12E-05
ENSDARG00000090721	prdm2a	684.260346	-0.693774397	0.1467651	-4.72710752	2.28E-06	2.12E-05
ENSDARG00000055100	cxcl12b	1297.52645	-0.496097149	0.10497468	-4.72587431	2.29E-06	2.14E-05
ENSDARG00000003695	vdac3	17356.0534	0.35888057	0.07596901	4.72403921	2.31E-06	2.15E-05

Table S2. DEGs of WT vs. *terfa-/-*

ENSDARG00000058032	tpk2	416.857993	0.812090147	0.17189666	4.72429277	2.31E-06	2.15E-05
ENSDARG00000002300	p2rx2	88.4232787	-1.52069671	0.32206842	-4.72165735	2.34E-06	2.18E-05
ENSDARG00000056247	bricd5	121.758507	1.071063382	0.22686775	4.72109148	2.35E-06	2.18E-05
ENSDARG00000014674	acsl3b	3875.4507	-0.495523355	0.10496733	-4.72073906	2.35E-06	2.19E-05
ENSDARG00000112632	si:ch211-106j21.6	40.0409632	-1.971265931	0.41778085	-4.71842096	2.38E-06	2.21E-05
ENSDARG00000110642	cacna1db	111.95644	-1.436508668	0.3044297	-4.7186877	2.37E-06	2.21E-05
ENSDARG00000059029	mmp28	574.472335	0.672827697	0.14260808	4.71801943	2.38E-06	2.21E-05
ENSDARG00000061140	colec12	3967.84105	0.562958814	0.11933076	4.71763384	2.39E-06	2.22E-05
ENSDARG00000009472	med19b	363.352721	-0.715159132	0.15158847	-4.71776722	2.38E-06	2.22E-05
ENSDARG00000045014	tuba2	9158.2537	-0.553429444	0.11734271	-4.71635115	2.40E-06	2.23E-05
ENSDARG00000076564	hspg2	7589.59683	0.754294852	0.15999513	4.71448648	2.42E-06	2.25E-05
ENSDARG00000029239	syt9b	694.027402	-0.93361378	0.19802292	-4.71467548	2.42E-06	2.25E-05
ENSDARG00000087544	BX510934.1	29.26663	2.757405158	0.58507993	4.7128692	2.44E-06	2.26E-05
ENSDARG00000013847	egfra	1253.34694	0.798310756	0.16936042	4.71367965	2.43E-06	2.26E-05
ENSDARG00000003973	chordc1a	231.329308	0.873709962	0.18538518	4.712944	2.44E-06	2.26E-05
ENSDARG00000109373	tmbim1b	130.217018	1.2317997	0.26143196	4.71174107	2.46E-06	2.27E-05
ENSDARG00000021853	ntpcr	226.941166	1.071772898	0.22745862	4.7119467	2.45E-06	2.27E-05
ENSDARG00000074715	stk17a	1195.63085	-0.613202947	0.1301406	-4.71184975	2.45E-06	2.27E-05
ENSDARG00000104040	CABZ01046088.1	57.374317	1.509099198	0.32025115	4.71223661	2.45E-06	2.27E-05
ENSDARG00000103369	taldo1	4536.68172	0.494535057	0.10497246	4.71109353	2.46E-06	2.28E-05
ENSDARG00000091561	si:dkey-156k2.4	71.3188794	2.270879602	0.48209676	4.71042287	2.47E-06	2.29E-05
ENSDARG00000004307	lypd6	423.069161	-0.657147041	0.13951686	-4.71016207	2.48E-06	2.29E-05
ENSDARG00000025495	nhlh2	1284.4686	-0.890187987	0.18901512	-4.70961258	2.48E-06	2.29E-05
ENSDARG00000022650	cyp2ad3	1359.10409	-0.772111146	0.16393195	-4.70994902	2.48E-06	2.29E-05
ENSDARG00000030630	mfsd2aa	243.890266	-1.065085205	0.22614497	-4.70974532	2.48E-06	2.29E-05
ENSDARG00000045601	cax1	323.243683	1.150217819	0.24425125	4.70915837	2.49E-06	2.30E-05
ENSDARG00000007025	ttc9c	211.344362	0.932890338	0.1981507	4.707984	2.50E-06	2.31E-05
ENSDARG00000007971	cks1b	373.629409	-0.861582358	0.18301679	-4.70766834	2.51E-06	2.31E-05
ENSDARG00000098355	atp5pd	8386.31413	0.523507253	0.11122242	4.70685018	2.52E-06	2.32E-05
ENSDARG00000041505	itm2bb	4830.65929	0.654376157	0.1390329	4.70662816	2.52E-06	2.32E-05
ENSDARG00000078105	depdc5	2002.91018	0.49624119	0.10542455	4.70707444	2.51E-06	2.32E-05
ENSDARG00000024365	crlf1a	572.513187	0.639025674	0.13579828	4.70569772	2.53E-06	2.33E-05
ENSDARG00000038473	ube2d3	3466.35884	0.456901952	0.09709421	4.70575881	2.53E-06	2.33E-05
ENSDARG00000020924	myo1ca	787.938665	0.592808068	0.12599625	4.70496609	2.54E-06	2.34E-05
ENSDARG00000034473	ttyh3a	2307.12547	-0.541056787	0.11500342	-4.70470153	2.54E-06	2.34E-05
ENSDARG00000069844	acod1	61.8677354	2.056574518	0.43718972	4.7040779	2.55E-06	2.34E-05
ENSDARG00000104641	si:ch211-261d7.6	548.917424	-0.613888118	0.13050787	-4.70383991	2.55E-06	2.35E-05
ENSDARG00000096554	si:dkey-25o16.4	1219.56204	-1.050297953	0.22334822	-4.70251315	2.57E-06	2.36E-05
ENSDARG00000069846	zgc:162944	1663.22453	0.534077614	0.11357856	4.70227509	2.57E-06	2.36E-05
ENSDARG00000016923	si:dkey-94f20.4	60.6219784	-2.106503288	0.44833834	-4.69846791	2.62E-06	2.40E-05
ENSDARG00000046053	slc27a6	281.928244	-0.740367211	0.15754714	-4.6993378	2.61E-06	2.40E-05
ENSDARG00000026322	dhrs13a.1	656.796305	0.610750461	0.12997825	4.69886657	2.62E-06	2.40E-05
ENSDARG00000016153	dag1	5866.48869	0.472572283	0.10060523	4.69729325	2.64E-06	2.42E-05
ENSDARG00000039365	cuedc2	280.325681	-0.790754831	0.1683998	-4.6956993	2.66E-06	2.44E-05
ENSDARG00000091136	zgc:174259	35.2346021	-2.441134696	0.52008174	-4.69375198	2.68E-06	2.46E-05
ENSDARG00000020956	pck2	4367.46426	0.687260457	0.14641287	4.6939893	2.68E-06	2.46E-05
ENSDARG00000099757	wdr47b	311.082276	-0.767329747	0.16347496	-4.69386729	2.68E-06	2.46E-05
ENSDARG00000116076	FO704772.3	334.375447	4.49672035	0.9583834	4.69198479	2.71E-06	2.48E-05
ENSDARG00000095956	CU467655.1	35.5947836	-2.940428725	0.62706595	-4.68918577	2.74E-06	2.51E-05
ENSDARG00000088204	shisa8b	83.6445686	-1.316576462	0.28075998	-4.6893309	2.74E-06	2.51E-05
ENSDARG00000004415	tcf7l2	3953.93502	-0.786525581	0.16774159	-4.68891206	2.75E-06	2.51E-05
ENSDARG00000051853	galns	426.376768	1.086850537	0.23183927	4.68794841	2.76E-06	2.52E-05
ENSDARG00000012297	cnga3b	186.351307	-1.627598896	0.3471575	-4.68835874	2.75E-06	2.52E-05
ENSDARG00000105527	SMIM36	157.411727	-1.807894876	0.38569499	-4.68736934	2.77E-06	2.53E-05
ENSDARG00000095294	si:dkey-200l5.4	76.2652511	-1.97687158	0.42178333	-4.68693625	2.77E-06	2.53E-05
ENSDARG00000070845	si:dkey-56d12.4	54.6307164	2.418404553	0.51610327	4.68589274	2.79E-06	2.54E-05
ENSDARG00000057867	lasp1	5234.91361	0.399607204	0.08529086	4.68522892	2.80E-06	2.55E-05
ENSDARG00000013926	slc16a9a	348.434659	1.26677556	0.27039349	4.68493365	2.80E-06	2.55E-05

Table S2. DEGs of WT vs. *terfa-/-*

ENSDARG00000061486	DNAH10	275.472858	1.08936956	0.2325746	4.68395756	2.81E-06	2.56E-05
ENSDARG00000037789	pvalb1	219121.163	0.640260152	0.13667184	4.68465295	2.80E-06	2.56E-05
ENSDARG00000095390	BX537288.2	33.6155938	-2.328409343	0.49719466	-4.68309401	2.83E-06	2.57E-05
ENSDARG00000003420	chrna5	284.770623	-0.762959724	0.16289767	-4.68367495	2.82E-06	2.57E-05
ENSDARG00000087844	plekhn1	326.530982	0.926237157	0.19778448	4.68306301	2.83E-06	2.57E-05
ENSDARG00000042717	adipor1b	1051.23513	0.512550033	0.10950391	4.68065515	2.86E-06	2.60E-05
ENSDARG00000058117	snap25b	5676.65904	-0.766621947	0.1638342	-4.67925468	2.88E-06	2.62E-05
ENSDARG00000016825	vtg6	9.36672254	4.434832543	0.94769825	4.67958292	2.87E-06	2.62E-05
ENSDARG00000005574	vsx2	537.745658	-0.880347018	0.1882619	-4.67618266	2.92E-06	2.66E-05
ENSDARG00000057321	tut1	466.783992	-0.563631919	0.12053231	-4.67618936	2.92E-06	2.66E-05
ENSDARG00000103218	BX927130.3	17.1617339	3.605253926	0.77123311	4.67466176	2.94E-06	2.67E-05
ENSDARG00000104071	hikeshi	710.299356	0.556663228	0.11907168	4.67502614	2.94E-06	2.67E-05
ENSDARG00000074170	fbxo4	187.789099	0.974310146	0.20846391	4.67375944	2.96E-06	2.68E-05
ENSDARG00000086169	rskrb	241.500339	-0.962687934	0.20597291	-4.67385711	2.96E-06	2.68E-05
ENSDARG00000101361	znf644a	941.127905	-0.59066473	0.12645867	-4.67081239	3.00E-06	2.72E-05
ENSDARG00000045006	ptprga	347.871396	-0.855991698	0.18325329	-4.67108503	3.00E-06	2.72E-05
ENSDARG00000011615	mybpc3	5972.14308	0.504624078	0.10805112	4.67023447	3.01E-06	2.73E-05
ENSDARG00000056836	si:ch211-125o16.4	4891.18605	0.648317125	0.13887283	4.66842326	3.04E-06	2.75E-05
ENSDARG00000070387	nudt7	139.334989	1.196185581	0.256316	4.66683926	3.06E-06	2.77E-05
ENSDARG00000040178	havcr1	180.006245	0.922816747	0.19779423	4.66553918	3.08E-06	2.79E-05
ENSDARG00000105479	si:ch211-126c2.4	450.190874	-0.754428038	0.16174416	-4.66432934	3.10E-06	2.80E-05
ENSDARG00000086903	gig2o	50.7332751	1.68514853	0.36141837	4.66259789	3.12E-06	2.82E-05
ENSDARG00000024746	hsp90aa1.2	4484.31435	0.483127441	0.10361514	4.66271094	3.12E-06	2.82E-05
ENSDARG00000055791	zgc:158423	663.720284	-0.609412622	0.13070666	-4.66244523	3.12E-06	2.83E-05
ENSDARG00000038392	si:dkeyp-52c3.7	13.8644516	7.120991475	1.5274395	4.66204486	3.13E-06	2.83E-05
ENSDARG00000044491	kif20a	1098.45164	-0.845516803	0.18135056	-4.66233364	3.13E-06	2.83E-05
ENSDARG00000099362	CABZ01088346.1	346.839791	0.748966392	0.16066313	4.66171917	3.14E-06	2.83E-05
ENSDARG00000109774	FH	846.293307	2.163703015	0.46410345	4.66211365	3.13E-06	2.83E-05
ENSDARG00000037100	ankrd10a	383.503988	-1.093167201	0.23454636	-4.66077243	3.15E-06	2.84E-05
ENSDARG00000052625	fkbp1b	1083.16872	0.634436345	0.13611492	4.66103465	3.15E-06	2.84E-05
ENSDARG00000010958	glmnb	329.133552	-2.459816085	0.52804907	-4.6583097	3.19E-06	2.87E-05
ENSDARG00000009480	pdcl	3577.62637	-0.589509835	0.12653858	-4.65873598	3.18E-06	2.87E-05
ENSDARG00000059574	fscn2a	169.940665	-1.634108354	0.35085768	-4.65746778	3.20E-06	2.88E-05
ENSDARG00000027689	pold1	1745.93833	-0.778883181	0.16725418	-4.65688317	3.21E-06	2.89E-05
ENSDARG00000069093	col2a1a	192141.374	0.542322683	0.11645039	4.65711369	3.21E-06	2.89E-05
ENSDARG00000022203	gtf2ird1	1835.81005	-0.673952939	0.14474049	-4.6562848	3.22E-06	2.90E-05
ENSDARG00000040076	pycard	1887.58936	0.504202367	0.10828549	4.65623193	3.22E-06	2.90E-05
ENSDARG00000031293	dnajc9	701.211743	-0.700365834	0.15041367	-4.65626454	3.22E-06	2.90E-05
ENSDARG00000068288	lamc2	179.637983	0.946110738	0.20323423	4.6552725	3.24E-06	2.91E-05
ENSDARG00000075556	luzp1	2333.74532	0.525688882	0.1129253	4.65519126	3.24E-06	2.91E-05
ENSDARG00000016763	znf292a	1720.83399	-0.614182723	0.13196168	-4.65425062	3.25E-06	2.92E-05
ENSDARG00000056877	vamp2	2108.71542	-0.783310293	0.16832873	-4.6534557	3.26E-06	2.93E-05
ENSDARG00000059360	srsf3b	6116.99379	-0.508965713	0.10938651	-4.65291101	3.27E-06	2.94E-05
ENSDARG00000055416	serpinb1	1171.41866	0.591509975	0.12720996	4.64987149	3.32E-06	2.98E-05
ENSDARG00000009196	anxa3a	525.308204	0.776456689	0.16702658	4.6487013	3.34E-06	3.00E-05
ENSDARG00000021896	asap3	1034.14369	0.845033303	0.18178516	4.64852752	3.34E-06	3.00E-05
ENSDARG00000117283	CABZ01085594.1	894.114878	-0.768180147	0.16530388	-4.64707888	3.37E-06	3.02E-05
ENSDARG00000053026	kif19	647.076992	-0.734760631	0.15810472	-4.64730354	3.36E-06	3.02E-05
ENSDARG00000036252	rras2	1398.6595	0.552042326	0.11880886	4.64647454	3.38E-06	3.03E-05
ENSDARG00000038147	hbbe3	192.41555	6.272414884	1.35016559	4.64566343	3.39E-06	3.04E-05
ENSDARG00000051731	faap24	38.4244665	-2.023862011	0.43564304	-4.64568881	3.39E-06	3.04E-05
ENSDARG00000059982	poc5	154.539277	-0.915688784	0.19713845	-4.64490197	3.40E-06	3.05E-05
ENSDARG00000001829	zgc:112982	2722.68763	-0.435909874	0.09385933	-4.64428909	3.41E-06	3.05E-05
ENSDARG00000032737	gria3a	2916.37013	-0.693747475	0.14938409	-4.64405211	3.42E-06	3.06E-05
ENSDARG00000027799	ucmaa	96.7765317	-1.742730798	0.37536097	-4.64281301	3.44E-06	3.07E-05
ENSDARG00000079847	zgc:194578	1548.8919	-0.619449053	0.13342314	-4.64274085	3.44E-06	3.07E-05
ENSDARG00000031463	syt6b	210.505862	-0.926249549	0.19952885	-4.64218355	3.45E-06	3.08E-05
ENSDARG00000104129	si:dkey-23n7.10	116.223832	1.468411188	0.31643517	4.64048036	3.48E-06	3.10E-05

Table S2. DEGs of WT vs. *terfa-/-*

ENSDARG00000100917	slc30a8	548.274982	-0.752558881	0.1622197	-4.6391336	3.50E-06	3.12E-05
ENSDARG00000024964	patj	1656.73756	0.589697211	0.12710094	4.63959767	3.49E-06	3.12E-05
ENSDARG00000055510	ypel3	648.896294	-0.698091661	0.15053128	-4.63751884	3.53E-06	3.15E-05
ENSDARG00000100540	CABZ01114105.1	222.315174	-0.931731865	0.20098077	-4.63592549	3.55E-06	3.17E-05
ENSDARG00000032765	net1	4814.98083	0.459070784	0.09903682	4.63535459	3.56E-06	3.18E-05
ENSDARG00000031222	lhx2b	983.590943	-0.884335347	0.19078493	-4.63524745	3.57E-06	3.18E-05
ENSDARG00000041689	usf1	1227.06685	-0.624486312	0.13473031	-4.63508423	3.57E-06	3.18E-05
ENSDARG00000045676	calua	2730.1959	0.54238471	0.11704516	4.63397817	3.59E-06	3.19E-05
ENSDARG00000068973	pdlim4	1111.42777	0.626609988	0.13524767	4.63305576	3.60E-06	3.21E-05
ENSDARG00000116978	CABZ01010830.1	25.485856	-2.652083826	0.57251672	-4.63232552	3.62E-06	3.22E-05
ENSDARG00000087224	si:ch73-380n15.2	219.372996	-1.148484784	0.24798218	-4.63131981	3.63E-06	3.23E-05
ENSDARG00000043701	gpd1a	821.804393	0.828226041	0.17883652	4.63119073	3.64E-06	3.23E-05
ENSDARG00000114495	ATP8A1	265.737747	-0.773536632	0.16701935	-4.63141918	3.63E-06	3.23E-05
ENSDARG00000031248	enkur	100.278058	1.325009446	0.28608546	4.63151625	3.63E-06	3.23E-05
ENSDARG00000092759	si:dkey-61p9.9	133.962017	0.96568586	0.20856367	4.63017284	3.65E-06	3.24E-05
ENSDARG00000101036	traf2b	273.111362	0.720298406	0.15556111	4.63032431	3.65E-06	3.24E-05
ENSDARG00000060041	lig1	1835.98191	-0.703315911	0.1519	-4.6301246	3.65E-06	3.24E-05
ENSDARG00000038213	slc35b1	1469.36486	0.473182328	0.10223953	4.6281741	3.69E-06	3.27E-05
ENSDARG00000078233	ctnnd1	4986.64867	0.356860109	0.07711538	4.62761296	3.70E-06	3.28E-05
ENSDARG00000099900	twnk	510.452236	-0.661098532	0.14286909	-4.62730273	3.70E-06	3.28E-05
ENSDARG00000098766	pcxa	5720.03783	0.406353496	0.0878208	4.62707583	3.71E-06	3.29E-05
ENSDARG00000098145	BX572628.1	19.1032395	-2.910120437	0.62901291	-4.6264876	3.72E-06	3.30E-05
ENSDARG00000105197	CR318620.1	10.4081345	6.78312347	1.46658418	4.62511704	3.74E-06	3.31E-05
ENSDARG00000028131	hccsa.1	523.409441	0.709604118	0.1534064	4.62564864	3.73E-06	3.31E-05
ENSDARG00000043130	notch2	7029.77833	0.553814516	0.11974609	4.62490685	3.75E-06	3.31E-05
ENSDARG00000074156	sgca	1183.51243	0.634123044	0.13710166	4.62520314	3.74E-06	3.31E-05
ENSDARG00000089063	lactbl1a	341.034368	-2.545436237	0.55031612	-4.62540737	3.74E-06	3.31E-05
ENSDARG00000036500	fam122b	868.103353	-0.533238623	0.11532303	-4.62386926	3.77E-06	3.33E-05
ENSDARG00000035899	lingo1b	549.44649	-0.707471039	0.15301881	-4.6234253	3.77E-06	3.34E-05
ENSDARG00000042825	rnf169	821.230448	0.82837235	0.17926636	4.62090235	3.82E-06	3.38E-05
ENSDARG00000018342	ggt1b	180.218217	0.87979633	0.1905179	4.61791961	3.88E-06	3.42E-05
ENSDARG00000035665	si:dkey-267n13.1	62.5756704	1.689282006	0.3658386	4.61756084	3.88E-06	3.43E-05
ENSDARG00000056617	rpgra	93.5586796	-1.82356978	0.39504781	-4.61607358	3.91E-06	3.45E-05
ENSDARG00000022315	atp6v1g1	6000.19554	-0.538910519	0.11675913	-4.61557494	3.92E-06	3.46E-05
ENSDARG00000113474	CABZ01067151.1	90.4375183	-1.522419993	0.32988188	-4.61504587	3.93E-06	3.46E-05
ENSDARG00000104181	tmsb1	5047.28741	0.823646421	0.17846536	4.6151613	3.93E-06	3.46E-05
ENSDARG00000024229	ubl7a	412.008733	-0.722320073	0.15648958	-4.61577102	3.92E-06	3.46E-05
ENSDARG00000036136	tipin	320.696008	-0.7160855	0.15518206	-4.61448637	3.94E-06	3.47E-05
ENSDARG00000043128	cldne	4459.11894	0.459776468	0.09963501	4.61460776	3.94E-06	3.47E-05
ENSDARG00000054442	cbx1b	1280.99445	-0.591255588	0.12814975	-4.6137867	3.95E-06	3.48E-05
ENSDARG00000094994	si:dkey-106c17.2	26.6112067	3.469243186	0.75198776	4.61343041	3.96E-06	3.49E-05
ENSDARG00000074379	knstrn	304.600736	-0.828770298	0.17965454	-4.61313295	3.97E-06	3.49E-05
ENSDARG00000056732	her4.1	95.2480398	-1.520665238	0.32964715	-4.61300888	3.97E-06	3.49E-05
ENSDARG00000088816	trabd2b	532.822585	0.593284867	0.12862566	4.61249247	3.98E-06	3.50E-05
ENSDARG00000067781	urp2	66.7532343	1.769227054	0.38364372	4.61164085	4.00E-06	3.51E-05
ENSDARG00000104697	si:dkey-57k17.1	106.947383	1.958264298	0.42469146	4.61102816	4.01E-06	3.52E-05
ENSDARG00000018105	casq1b	3570.31635	0.71458601	0.15496627	4.61123573	4.00E-06	3.52E-05
ENSDARG00000012426	neflb	1470.6846	0.548154025	0.11887222	4.61128801	4.00E-06	3.52E-05
ENSDARG00000042839	dph6	343.017443	0.781462297	0.16948308	4.61085724	4.01E-06	3.52E-05
ENSDARG00000026453	zgc:66474	828.609592	-0.509659773	0.11055384	-4.61005949	4.03E-06	3.53E-05
ENSDARG00000027547	TPGS1	612.181564	0.680485824	0.14761306	4.60992957	4.03E-06	3.53E-05
ENSDARG00000042969	znf1035	3729.28117	-0.540798788	0.11735823	-4.60810273	4.06E-06	3.56E-05
ENSDARG00000010246	prmt1	6772.58062	-0.434980785	0.09441151	-4.60728575	4.08E-06	3.58E-05
ENSDARG00000089123	negaly6	141.271975	-1.09799458	0.23832211	-4.60718721	4.08E-06	3.58E-05
ENSDARG00000101307	pcdh1gb2	96.4679161	-1.135509083	0.24648349	-4.60683625	4.09E-06	3.58E-05
ENSDARG00000056623	ptenb	2917.94885	-0.457864471	0.09940477	-4.60606155	4.10E-06	3.59E-05
ENSDARG00000067670	pomt1	590.32333	0.807553713	0.17537036	4.60484725	4.13E-06	3.61E-05
ENSDARG00000092120	BX470185.1	45.5032746	2.928844696	0.63598174	4.60523395	4.12E-06	3.61E-05

Table S2. DEGs of WT vs. *terfa-/-*

ENSDARG00000028800		468.708833	-0.738161925	0.16033192	-4.60396121	4.15E-06	3.62E-05
ENSDARG00000002391	tlcd1	309.836077	0.769701579	0.16718179	4.60397989	4.14E-06	3.62E-05
ENSDARG00000105465	BX005228.2	972.874894	-0.598723012	0.13012573	-4.60111164	4.20E-06	3.67E-05
ENSDARG00000105046	ccnf	1347.1694	-0.437872816	0.09517668	-4.60063146	4.21E-06	3.68E-05
ENSDARG00000034187	calm1b	4265.8245	-0.559550218	0.12162493	-4.60062123	4.21E-06	3.68E-05
ENSDARG00000098845	lrp12	2091.10515	-0.511011997	0.11108014	-4.60039032	4.22E-06	3.68E-05
ENSDARG00000043843	akap7	1614.01174	0.696389248	0.15139831	4.59971625	4.23E-06	3.69E-05
ENSDARG00000100564	sil1	593.022483	0.665499851	0.1447404	4.59788582	4.27E-06	3.72E-05
ENSDARG00000069472	chsy3	664.751119	0.591199199	0.1286424	4.5956793	4.31E-06	3.76E-05
ENSDARG00000038859	rgs20	709.415529	-0.828308502	0.18031943	-4.59356203	4.36E-06	3.80E-05
ENSDARG00000027131	tmem169a	366.156172	-0.782859853	0.1704348	-4.5933099	4.36E-06	3.80E-05
ENSDARG00000102472	btbd17b	290.141291	-1.059474046	0.23071576	-4.59211824	4.39E-06	3.82E-05
ENSDARG00000007720	sub1b	699.440121	-0.599326858	0.13056548	-4.59023996	4.43E-06	3.86E-05
ENSDARG00000003632	zgc:172270	137.986512	0.997868696	0.21740233	4.58996313	4.43E-06	3.86E-05
ENSDARG00000114901	FP236318.5	165.648056	-0.893319674	0.19463146	-4.58980101	4.44E-06	3.86E-05
ENSDARG00000075536	cpne1	3378.23768	0.436755587	0.09516857	4.5892837	4.45E-06	3.87E-05
ENSDARG00000045989	arf4a	1255.63636	0.528102413	0.11510769	4.58789852	4.48E-06	3.89E-05
ENSDARG00000074287	sptlc2b	1769.2093	0.603552623	0.13157584	4.58710814	4.49E-06	3.90E-05
ENSDARG00000088040	si:dkeyp-27c8.2	224.639225	1.047806541	0.22841527	4.58728763	4.49E-06	3.90E-05
ENSDARG00000070677	fezf2	505.12509	-1.229722652	0.26807732	-4.58719399	4.49E-06	3.90E-05
ENSDARG00000045568	bcat1	525.737527	-0.883875371	0.19269704	-4.58686539	4.50E-06	3.91E-05
ENSDARG00000109563	LSM10	81.6698759	-2.002308948	0.43661185	-4.58601609	4.52E-06	3.92E-05
ENSDARG00000095651	hmx1	294.147994	-1.177582975	0.25679637	-4.5856683	4.53E-06	3.93E-05
ENSDARG00000060662	prf1.9	26.7284595	3.274303317	0.71462012	4.58187955	4.61E-06	4.00E-05
ENSDARG00000034896	ldb2b	1529.81671	-0.705904495	0.15408094	-4.58138754	4.62E-06	4.00E-05
ENSDARG00000010823	memo1	954.020286	0.524145298	0.11439312	4.58196513	4.61E-06	4.00E-05
ENSDARG00000013110	dmtn	582.199661	-0.57251787	0.12497963	-4.58088943	4.63E-06	4.01E-05
ENSDARG00000028542	si:ch211-114c12.2	3709.05957	-0.389188212	0.08498619	-4.57942904	4.66E-06	4.04E-05
ENSDARG00000099382	si:ch211-209n20.1	12.5334681	4.76536209	1.04084384	4.57836411	4.69E-06	4.05E-05
ENSDARG00000045951	kat5b	774.150069	-0.534388646	0.116719	-4.5784205	4.69E-06	4.05E-05
ENSDARG00000040465	irf2	404.711523	0.672176778	0.14681326	4.57844729	4.68E-06	4.05E-05
ENSDARG00000041098	barx2	258.465187	0.780477949	0.1704654	4.57851239	4.68E-06	4.05E-05
ENSDARG00000111701	lbh	279.950095	0.705416215	0.1541039	4.57753654	4.70E-06	4.07E-05
ENSDARG00000007739	atp1a1a.2	4246.62323	-0.697492158	0.15240238	-4.57664865	4.72E-06	4.08E-05
ENSDARG00000052764	chrnb3a	488.201806	-0.784727391	0.17145984	-4.57674159	4.72E-06	4.08E-05
ENSDARG00000079067	mms19	1225.88749	0.526178691	0.11496792	4.57674348	4.72E-06	4.08E-05
ENSDARG00000101906	zgc:113442	133.684497	0.976256808	0.21333273	4.57621681	4.73E-06	4.09E-05
ENSDARG00000032369	btbd6b	1459.23637	-0.456240171	0.09970677	-4.57581939	4.74E-06	4.09E-05
ENSDARG00000105018	lrrc20	556.747818	0.72399281	0.15821534	4.5759962	4.74E-06	4.09E-05
ENSDARG00000024209	wasf2	2413.47632	0.44029255	0.0962228	4.57576107	4.74E-06	4.09E-05
ENSDARG00000039820	mea1	1309.42077	-0.468309578	0.10235128	-4.57551263	4.75E-06	4.10E-05
ENSDARG00000114236	znf974	34.4486134	2.051738765	0.44847317	4.57494205	4.76E-06	4.11E-05
ENSDARG00000039392	wnk1b	4850.05137	0.55026689	0.12030368	4.57398221	4.79E-06	4.12E-05
ENSDARG00000096496	BX571705.1	22.4428935	2.529400479	0.55298514	4.57408401	4.78E-06	4.12E-05
ENSDARG00000013784	zgc:77158	347.658796	0.804051988	0.17579023	4.57392872	4.79E-06	4.12E-05
ENSDARG00000005104	limk2	2029.48898	0.478819658	0.10469038	4.5736741	4.79E-06	4.13E-05
ENSDARG00000041081	kmt5b	2935.10485	-0.480465117	0.1050635	-4.57309274	4.81E-06	4.13E-05
ENSDARG00000102538	mccc1	3386.61029	0.42312024	0.09251527	4.57351775	4.80E-06	4.13E-05
ENSDARG00000105630	alpk3b	129.107178	1.233754236	0.26982337	4.57245143	4.82E-06	4.15E-05
ENSDARG00000100003	glulb	9150.07019	0.648167275	0.14179566	4.57113612	4.85E-06	4.17E-05
ENSDARG00000029865	rassf2a	1092.12693	-0.511480786	0.11189696	-4.57099811	4.85E-06	4.17E-05
ENSDARG00000036316	rpl39	14432.3757	0.545542504	0.11940104	4.56899307	4.90E-06	4.21E-05
ENSDARG00000077115	si:ch73-44m9.1	27.8468766	2.510364545	0.54944982	4.56886952	4.90E-06	4.21E-05
ENSDARG00000008540	sox21b	444.168777	-0.775729506	0.1698626	-4.56680586	4.95E-06	4.25E-05
ENSDARG00000030750	gabrr3b	152.756508	-1.352705258	0.29620533	-4.56678231	4.95E-06	4.25E-05
ENSDARG00000061357	chst6	399.093397	0.721710027	0.15803078	4.56689529	4.95E-06	4.25E-05
ENSDARG00000062997	hyal2b	1137.22624	0.575422431	0.12602773	4.56584002	4.97E-06	4.27E-05
ENSDARG00000102859	CABZ01092170.1	243.35083	0.912251941	0.19980409	4.56573206	4.98E-06	4.27E-05

Table S2. DEGs of WT vs. *terfa-/-*

ENSDARG00000092142	BX663503.1	323.894023	-1.525547603	0.33428532	-4.5636093	5.03E-06	4.31E-05
ENSDARG00000007867	dazap2	3349.85352	-0.457439649	0.10023909	-4.56348582	5.03E-06	4.31E-05
ENSDARG00000056691	cpeb4a	1478.69925	-0.452016381	0.09911265	-4.56063255	5.10E-06	4.37E-05
ENSDARG00000075619	cenpo	115.801605	-1.10462914	0.24227888	-4.55932912	5.13E-06	4.39E-05
ENSDARG00000010411	epn1	3576.89161	-0.374946086	0.08225879	-4.55812769	5.16E-06	4.41E-05
ENSDARG00000029544	id2b	373.746688	-0.797023094	0.17484225	-4.55852683	5.15E-06	4.41E-05
ENSDARG00000057792	cx52.7	34.0406589	-2.456538296	0.53890961	-4.5583494	5.16E-06	4.41E-05
ENSDARG00000021378	phf21ab	1009.27199	-0.601376263	0.13192247	-4.55855814	5.15E-06	4.41E-05
ENSDARG00000062821	slc6a15	972.640081	-0.865933074	0.19002643	-4.5569085	5.19E-06	4.43E-05
ENSDARG00000035458	atp2a1l	22343.3895	0.756433251	0.16598742	4.55717225	5.18E-06	4.43E-05
ENSDARG00000055490	galnt12	422.923885	0.652823517	0.14324555	4.55737392	5.18E-06	4.43E-05
ENSDARG00000039900	si:ch73-168d20.1	7.62267487	6.754305554	1.48218914	4.55697952	5.19E-06	4.43E-05
ENSDARG00000097615	si:ch211-108d22.2	734.842813	-0.802794637	0.17621513	-4.55576448	5.22E-06	4.46E-05
ENSDARG00000036994	tbc1d16	608.197869	0.562031654	0.12336983	4.55566525	5.22E-06	4.46E-05
ENSDARG00000088377	rp1l1b	81.4383865	-2.519799116	0.55312298	-4.55558563	5.22E-06	4.46E-05
ENSDARG00000006397	laptm4a	3690.2615	0.396653563	0.08707643	4.55523446	5.23E-06	4.46E-05
ENSDARG00000099183	fkbp10a	321.535546	0.769612664	0.16894985	4.55527277	5.23E-06	4.46E-05
ENSDARG00000037475	foxb2	64.3152953	-1.704528058	0.374297	-4.55394529	5.26E-06	4.49E-05
ENSDARG00000098527	CABZ01060491.1	12.5333313	3.841134582	0.84355943	4.55348427	5.28E-06	4.49E-05
ENSDARG00000101232	pcdh1g2	113.415239	-1.153817288	0.25347012	-4.55208407	5.31E-06	4.52E-05
ENSDARG00000061066	c2cd2l	969.486876	-0.612919646	0.13466016	-4.55160358	5.32E-06	4.53E-05
ENSDARG00000020699	slc9a8	542.191235	0.601494472	0.13216615	4.55104782	5.34E-06	4.54E-05
ENSDARG00000100430	CU634008.1	111.12673	-1.514098361	0.33276159	-4.55009959	5.36E-06	4.56E-05
ENSDARG00000011113	chrna10a	80.9022325	-1.570988691	0.34533187	-4.54921429	5.38E-06	4.58E-05
ENSDARG00000018423	sulf2a	1962.21941	-0.717024652	0.15761159	-4.5493143	5.38E-06	4.58E-05
ENSDARG00000087556	pacrg	71.6744215	1.257831573	0.27654477	4.54838324	5.41E-06	4.59E-05
ENSDARG00000013755	actn3a	55617.1809	0.564725982	0.1241833	4.54751971	5.43E-06	4.61E-05
ENSDARG00000099038	kcng1	199.221107	0.933135484	0.20521928	4.54701658	5.44E-06	4.62E-05
ENSDARG00000069402	lrrc4.1	1652.21291	-0.680145926	0.14962277	-4.54573798	5.47E-06	4.65E-05
ENSDARG00000061213	rabep2	675.500246	0.539883353	0.1187738	4.54547521	5.48E-06	4.65E-05
ENSDARG00000099902	il17rc	1091.36385	0.677954262	0.14914555	4.54558815	5.48E-06	4.65E-05
ENSDARG00000019160	sh3bp5la	504.527731	-0.659476162	0.14511259	-4.54458265	5.50E-06	4.67E-05
ENSDARG00000056050	kctd17	994.719325	-0.533500055	0.11741168	-4.54384153	5.52E-06	4.68E-05
ENSDARG00000101423	cyp2y3	3245.19487	0.627965549	0.13819729	4.54397882	5.52E-06	4.68E-05
ENSDARG00000013245	wbp1la	344.264983	0.673630372	0.14827545	4.54310105	5.54E-06	4.69E-05
ENSDARG00000002912	adipor1a	1903.49045	-0.431132603	0.09489152	-4.5434259	5.53E-06	4.69E-05
ENSDARG00000101459	si:dkey-279j5.1	45.1499693	2.24370815	0.49389444	4.54289011	5.55E-06	4.70E-05
ENSDARG00000063412	cers1	612.971942	-0.611112105	0.134522	-4.5428414	5.55E-06	4.70E-05
ENSDARG00000053091	DAB2	2361.06652	0.667482616	0.14697041	4.54161225	5.58E-06	4.72E-05
ENSDARG00000013968	psap	15699.2063	0.480523805	0.10580602	4.5415545	5.58E-06	4.72E-05
ENSDARG00000024651	snrpa1	1262.68172	-0.523253236	0.11523048	-4.5409274	5.60E-06	4.73E-05
ENSDARG00000106529	dut	1459.21061	-0.645909641	0.14225316	-4.54056457	5.61E-06	4.74E-05
ENSDARG00000039335	st6gal2a	3161.30555	-0.485633935	0.10702134	-4.53772976	5.69E-06	4.80E-05
ENSDARG00000098189	si:dkey-16p6.1	16.734456	3.206771791	0.70685491	4.53667615	5.71E-06	4.82E-05
ENSDARG00000012896	anos1a	1854.84909	-0.70870133	0.15620727	-4.53692924	5.71E-06	4.82E-05
ENSDARG00000069360	ppp3r1b	4253.93845	-0.405072552	0.08928295	-4.53695284	5.71E-06	4.82E-05
ENSDARG00000001313	g2e3	609.402809	-1.108746275	0.24439116	-4.53676909	5.71E-06	4.82E-05
ENSDARG00000079388	agrn	7183.77459	0.472173329	0.10409243	4.53609675	5.73E-06	4.83E-05
ENSDARG00000007786	tmx2b	2367.76328	-0.432770923	0.0954131	-4.53576018	5.74E-06	4.84E-05
ENSDARG00000060435	bnipl	597.030734	0.727761115	0.16044619	4.53585792	5.74E-06	4.84E-05
ENSDARG00000062831	si:ch73-22o12.1	2125.06831	0.411519934	0.09074219	4.53504512	5.76E-06	4.85E-05
ENSDARG00000004517	ppat	1177.42497	0.527867899	0.1164213	4.53411801	5.78E-06	4.87E-05
ENSDARG00000062577	arhgap35a	2543.25872	0.442791465	0.09766144	4.53394346	5.79E-06	4.87E-05
ENSDARG00000105822	CABZ01117780.1	66.7939155	-1.442496963	0.31829989	-4.53188023	5.85E-06	4.92E-05
ENSDARG00000021200	nebl	33.8879482	-2.292979659	0.50598407	-4.53172299	5.85E-06	4.92E-05
ENSDARG00000062974	itga2.2	554.200658	1.051602084	0.23205342	4.53172412	5.85E-06	4.92E-05
ENSDARG00000015901	ptbp2b	3126.70144	-0.663962849	0.14655747	-4.5303925	5.89E-06	4.95E-05
ENSDARG00000044605	stx12l	1063.16677	-0.49115343	0.10841223	-4.53042474	5.89E-06	4.95E-05

Table S2. DEGs of WT vs. *terfa-/-*

ENSDARG00000026329	arhgap29a	757.051375	0.592378468	0.13075144	4.53056954	5.88E-06	4.95E-05
ENSDARG00000037159	oprd1b	55.7909954	-1.644806941	0.3631095	-4.5297822	5.90E-06	4.96E-05
ENSDARG00000017311	gpsm2	1433.95846	-0.433585001	0.0957508	-4.52826498	5.95E-06	4.99E-05
ENSDARG00000015902	stat6	1158.42717	0.72291615	0.15964571	4.52825283	5.95E-06	4.99E-05
ENSDARG00000090871	si:dkey-210j14.4	331.536511	-0.743989751	0.16433118	-4.5273804	5.97E-06	5.01E-05
ENSDARG00000113274	AL929536.3	232.270816	-0.96734537	0.21366251	-4.52744555	5.97E-06	5.01E-05
ENSDARG00000079443	vipb	217.339518	-1.176217674	0.25981922	-4.52706188	5.98E-06	5.02E-05
ENSDARG00000058953	abcc4	1665.13819	0.61779058	0.13649599	4.52607136	6.01E-06	5.04E-05
ENSDARG00000076870	piezo1	1215.08246	0.759457352	0.16784396	4.52478205	6.05E-06	5.07E-05
ENSDARG00000022891	zgc:175214	2286.83806	-0.444486471	0.09824419	-4.52430274	6.06E-06	5.08E-05
ENSDARG00000097889	si:ch73-265h17.2	37.4364201	2.622248811	0.57960088	4.52423197	6.06E-06	5.08E-05
ENSDARG00000061548	si:ch211-145o7.3	110.758214	1.269465092	0.28074547	4.52176523	6.13E-06	5.13E-05
ENSDARG00000063636	galnt11	1633.97057	0.448831822	0.09927182	4.52124094	6.15E-06	5.14E-05
ENSDARG00000001953	pfkfb3	740.911864	1.232751001	0.2727331	4.51999045	6.18E-06	5.17E-05
ENSDARG00000053450	ftr06	25.0722403	3.01353941	0.66685515	4.51903151	6.21E-06	5.19E-05
ENSDARG00000030716	ing4	3064.28883	-0.526438089	0.11649319	-4.51904616	6.21E-06	5.19E-05
ENSDARG00000086309	erlec1	1545.60745	0.50669506	0.11212893	4.51886123	6.22E-06	5.20E-05
ENSDARG00000094557	nupr1b	1911.69649	-0.748831634	0.1657341	-4.51827141	6.23E-06	5.21E-05
ENSDARG00000006202	erbb3a	2510.53239	0.517839201	0.11461328	4.5181433	6.24E-06	5.21E-05
ENSDARG00000045199	CAPN1	1706.22348	0.45518028	0.10077773	4.51667536	6.28E-06	5.24E-05
ENSDARG00000098988	si:ch211-193i15.1	13.1950813	-5.403635804	1.19672976	-4.51533502	6.32E-06	5.28E-05
ENSDARG00000062187	kif14	578.87047	-1.092670861	0.24201283	-4.51492958	6.33E-06	5.28E-05
ENSDARG00000062430	gpd2	784.291186	0.74108957	0.16421061	4.5130433	6.39E-06	5.33E-05
ENSDARG00000077167	nmbb	38.8484772	-1.944372935	0.43088384	-4.51252227	6.41E-06	5.34E-05
ENSDARG00000074245	spen	3566.67204	-0.589858396	0.13072581	-4.51218022	6.42E-06	5.35E-05
ENSDARG00000094844	BX323457.3	8.72286763	6.982351582	1.54807089	4.5103565	6.47E-06	5.39E-05
ENSDARG00000003210	ano2a	423.088136	-0.771186913	0.17098892	-4.51015736	6.48E-06	5.39E-05
ENSDARG00000075864	igsf9a	1052.24999	-0.505987017	0.1121765	-4.510633	6.46E-06	5.39E-05
ENSDARG00000078825	bub1ba	416.958216	-0.949637343	0.21065022	-4.50812422	6.54E-06	5.44E-05
ENSDARG00000004680	dnajb6a	1917.35289	-0.53005991	0.11758416	-4.50791922	6.55E-06	5.45E-05
ENSDARG00000028878	vipr1a	243.422495	0.882202531	0.19572942	4.50725563	6.57E-06	5.46E-05
ENSDARG00000106671	CABZ01078261.1	47.2262666	-1.788453087	0.39688666	-4.50620616	6.60E-06	5.49E-05
ENSDARG00000087554	cdk1	1305.19367	-0.732259431	0.16250997	-4.50593533	6.61E-06	5.49E-05
ENSDARG00000057575	pnp4a	1799.47515	-0.967671062	0.21475492	-4.50593201	6.61E-06	5.49E-05
ENSDARG00000042370	ptafr	15.8257314	2.904931735	0.64472602	4.50568406	6.62E-06	5.50E-05
ENSDARG00000012340	ptpn11b	519.40281	-0.651273867	0.14456057	-4.50519703	6.63E-06	5.51E-05
ENSDARG00000100913	arhgef12b	2685.59274	0.432364314	0.09599511	4.50402437	6.67E-06	5.53E-05
ENSDARG00000088862	stxbp6	874.452476	0.543362957	0.12063269	4.50427616	6.66E-06	5.53E-05
ENSDARG00000091756	insm1a	1669.9182	-1.097910791	0.24376932	-4.50389245	6.67E-06	5.54E-05
ENSDARG00000091381	si:ch211-214c20.1	30.1856033	2.224737524	0.49402008	4.50333421	6.69E-06	5.55E-05
ENSDARG00000102349	cep55l	256.726186	-0.875886293	0.19451708	-4.50287597	6.70E-06	5.56E-05
ENSDARG00000074663	zgc:162183	264.137912	0.752274509	0.16711004	4.5016715	6.74E-06	5.59E-05
ENSDARG00000105086	CABZ01079005.1	30.798563	-2.410896924	0.53560982	-4.50121869	6.76E-06	5.60E-05
ENSDARG00000039750	ldlrap1b	249.151949	0.827841072	0.18395597	4.50021322	6.79E-06	5.62E-05
ENSDARG00000105424	si:rp71-46j2.7	570.258122	0.899913086	0.20000543	4.49944324	6.81E-06	5.64E-05
ENSDARG00000078118	sinhcafl	1211.30945	-1.596950744	0.35515884	-4.49644095	6.91E-06	5.72E-05
ENSDARG00000014680	znf710a	761.762987	-0.879215512	0.19553049	-4.49656483	6.91E-06	5.72E-05
ENSDARG00000080010	adh5	5186.84833	0.473201741	0.1052509	4.49593995	6.93E-06	5.73E-05
ENSDARG00000052413	crfb12	289.574399	1.032307903	0.22968082	4.49453246	6.97E-06	5.77E-05
ENSDARG00000032129	gtf2f1	1538.77119	-0.673801359	0.14992089	-4.49437942	6.98E-06	5.77E-05
ENSDARG00000024540	tspan36	2270.55641	0.737231502	0.16404079	4.49419635	6.98E-06	5.77E-05
ENSDARG00000116725	CABZ01114053.1	225.86275	-1.376382241	0.30625231	-4.49427548	6.98E-06	5.77E-05
ENSDARG00000030844	klf11a	2324.1429	-0.942731649	0.20981921	-4.49306636	7.02E-06	5.80E-05
ENSDARG00000057419	slc44a5b	682.170883	-0.606358043	0.13495664	-4.49298425	7.02E-06	5.80E-05
ENSDARG00000062171	olfml3b	1626.94907	0.479058763	0.10663375	4.49256251	7.04E-06	5.81E-05
ENSDARG00000023495	ift74	2530.79379	-0.495055383	0.11021913	-4.49155607	7.07E-06	5.83E-05
ENSDARG00000038075	cyc1	11071.4071	0.422027692	0.09397262	4.49096425	7.09E-06	5.84E-05
ENSDARG00000101130	coq8b	732.018397	-0.502481654	0.11188068	-4.49122801	7.08E-06	5.84E-05

Table S2. DEGs of WT vs. *terfa-/-*

ENSDARG00000005980	sfswap	3354.233	-0.404347859	0.09003009	-4.49125232	7.08E-06	5.84E-05
ENSDARG00000104894	si:ch73-138e16.2	163.097232	-0.974767932	0.21706534	-4.49066591	7.10E-06	5.85E-05
ENSDARG00000005749	cand2	738.170878	0.858057924	0.191088	4.49038092	7.11E-06	5.86E-05
ENSDARG00000090116	znf1136	94.0508098	-1.27703579	0.28452627	-4.48828776	7.18E-06	5.91E-05
ENSDARG00000003920	setb	8104.22899	-0.497298122	0.11081022	-4.48783609	7.20E-06	5.92E-05
ENSDARG00000052182	rbfox2	6015.47806	-0.430883467	0.09602381	-4.48725657	7.21E-06	5.94E-05
ENSDARG00000055620	acad9	1107.16216	0.447909477	0.09983236	4.48661613	7.24E-06	5.95E-05
ENSDARG00000056743	cavin4b	5585.95765	0.496177906	0.1105914	4.4865868	7.24E-06	5.95E-05
ENSDARG00000114670	ube2c	1103.9736	-0.89133303	0.19870026	-4.48581705	7.26E-06	5.97E-05
ENSDARG00000011422	ryr1a	4275.66211	0.64308045	0.1433751	4.48530084	7.28E-06	5.98E-05
ENSDARG00000098390	si:ch211-76m11.8	39.7536779	2.11479612	0.47153169	4.48495013	7.29E-06	5.99E-05
ENSDARG00000095402	BX897741.1	47.0484049	-2.375731936	0.52969622	-4.48508382	7.29E-06	5.99E-05
ENSDARG00000015589	ankrd33bb	38.101436	-2.780501951	0.62017162	-4.48343949	7.34E-06	6.03E-05
ENSDARG00000054150	cx23	215.175673	-1.400831238	0.31257131	-4.48163736	7.41E-06	6.08E-05
ENSDARG00000052739	egf	250.500728	-1.181912627	0.26375639	-4.48107673	7.43E-06	6.09E-05
ENSDARG00000116835	park7	1276.62604	0.573196812	0.12794642	4.47997553	7.47E-06	6.12E-05
ENSDARG00000068246	plcb3	1817.01539	0.610431303	0.13628675	4.47902156	7.50E-06	6.14E-05
ENSDARG00000039516	c8a	1440.98874	0.537865502	0.12008144	4.47917271	7.49E-06	6.14E-05
ENSDARG00000002311	fabp11b	73.4821083	-1.45939497	0.32581935	-4.47915378	7.49E-06	6.14E-05
ENSDARG00000117313	CABZ01005857.2	25.8236305	2.791605867	0.62323015	4.47925353	7.49E-06	6.14E-05
ENSDARG00000078527	lingo4b	442.43705	-0.639283549	0.1427561	-4.47815218	7.53E-06	6.16E-05
ENSDARG00000040607	rtf2	1048.20182	-0.563555046	0.1258608	-4.47760588	7.55E-06	6.18E-05
ENSDARG00000093699	si:ch73-27e22.4	162.325801	0.943438108	0.21071888	4.47723577	7.56E-06	6.19E-05
ENSDARG00000101831	irx1a	1095.67066	-0.778718149	0.17396549	-4.47627939	7.60E-06	6.21E-05
ENSDARG00000098747	CU633479.1	519.36895	1.03786407	0.23195251	4.47446788	7.66E-06	6.26E-05
ENSDARG00000008880	usp28	1691.115	0.556888182	0.1244718	4.47401084	7.68E-06	6.27E-05
ENSDARG00000110900	CABZ01078606.1	20.0652741	-5.100221185	1.14010447	-4.47346827	7.70E-06	6.29E-05
ENSDARG00000089805	pcdh18a	1652.2022	-0.582436428	0.13023137	-4.47232065	7.74E-06	6.32E-05
ENSDARG00000045930	rbpms2a	2459.07399	-0.634660586	0.14191322	-4.47217376	7.74E-06	6.32E-05
ENSDARG00000093120	si:dkey-117m1.4	1036.51093	-0.720696211	0.16117896	-4.47140369	7.77E-06	6.34E-05
ENSDARG00000102321	si:ch73-299h12.1	158.034425	-0.913389187	0.2043092	-4.47062188	7.80E-06	6.36E-05
ENSDARG00000095897	atp5f1e	4329.51741	0.646539468	0.14469256	4.46836707	7.88E-06	6.43E-05
ENSDARG00000063570	dyrk1aa	4351.67307	-0.535441348	0.11985029	-4.4675848	7.91E-06	6.45E-05
ENSDARG00000033307	igf2b	1572.84545	0.647836829	0.14501894	4.46725682	7.92E-06	6.46E-05
ENSDARG00000035887	zgc:91944	821.336927	-0.568569354	0.12728379	-4.46694247	7.93E-06	6.47E-05
ENSDARG00000087897	macroh2a2	6978.80833	-0.503638767	0.11279175	-4.46520915	8.00E-06	6.51E-05
ENSDARG00000061580	kctd7	338.161758	-0.872475922	0.19538642	-4.46538669	7.99E-06	6.51E-05
ENSDARG00000097285	ANPEP	422.089287	-0.761312227	0.17048691	-4.46551714	7.99E-06	6.51E-05
ENSDARG00000077883	fam83d	336.346189	-1.011132649	0.22647823	-4.46459099	8.02E-06	6.53E-05
ENSDARG00000070416	zgc:162816	494.833301	0.551694689	0.12357237	4.46454736	8.02E-06	6.53E-05
ENSDARG00000044925	cdk5rap1	250.870037	0.672519837	0.15073852	4.46149942	8.14E-06	6.62E-05
ENSDARG00000101210	glg1b	637.015065	0.728133936	0.16321425	4.4612155	8.15E-06	6.63E-05
ENSDARG00000077323	thap4	401.723743	-0.711903616	0.15960999	-4.46026986	8.19E-06	6.65E-05
ENSDARG00000008413	atp11a	2630.72449	-0.589653539	0.13221892	-4.45967585	8.21E-06	6.66E-05
ENSDARG00000074927	bub1bb	546.432034	-0.860611235	0.19296789	-4.45986748	8.20E-06	6.66E-05
ENSDARG00000097530	xkr5b	210.104668	-1.070740257	0.24007034	-4.46011054	8.19E-06	6.66E-05
ENSDARG00000044808	slc4a4b	1375.56977	0.638817969	0.14324136	4.45973126	8.21E-06	6.66E-05
ENSDARG00000076121	smpd1	848.799028	0.744111086	0.16688308	4.45887681	8.24E-06	6.69E-05
ENSDARG00000091746	si:dkey-16p6.1	19.7117239	2.831121904	0.63501511	4.45835359	8.26E-06	6.70E-05
ENSDARG00000020857	ccdc149b	330.667306	-0.617449263	0.13849814	-4.45817732	8.27E-06	6.70E-05
ENSDARG00000003829	galnt2	2382.94431	0.37692864	0.08454461	4.45834049	8.26E-06	6.70E-05
ENSDARG00000030440	rsrp1	3691.97269	0.63669398	0.14282678	4.45780528	8.28E-06	6.70E-05
ENSDARG00000057586	sall3b	989.005235	0.507397642	0.11380531	4.45847084	8.25E-06	6.70E-05
ENSDARG00000042509	glod4	271.20862	0.81802872	0.18350826	4.45772146	8.28E-06	6.70E-05
ENSDARG00000077257	timd4	74.5269658	-1.392682868	0.31240447	-4.45794794	8.27E-06	6.70E-05
ENSDARG00000009477	prkar2ab	310.81454	-1.381686006	0.30994277	-4.45787464	8.28E-06	6.70E-05
ENSDARG00000014058	rab36	72.1098502	1.378433835	0.30928915	4.45678041	8.32E-06	6.73E-05
ENSDARG00000092798	ppib	14608.4063	0.489938711	0.10996398	4.45544727	8.37E-06	6.77E-05

Table S2. DEGs of WT vs. *terfa-/-*

ENSDARG00000105301	sox8a	109.391396	-1.425772621	0.32021808	-4.45250511	8.49E-06	6.86E-05
ENSDARG00000034717	def6c	292.678407	0.683234452	0.15346182	4.45214617	8.50E-06	6.87E-05
ENSDARG00000104047	si:ch211-262i1.3	300.685893	-0.807516083	0.18141499	-4.45120929	8.54E-06	6.90E-05
ENSDARG00000098892	si:ch211-223a21.1	66.3975413	1.375981722	0.30923883	4.44957611	8.60E-06	6.95E-05
ENSDARG00000117274	CABZ01076744.1	48.3355335	-1.905527808	0.42836556	-4.44836835	8.65E-06	6.98E-05
ENSDARG00000068759	si:ch211-244o22.2	921.133372	-0.837287467	0.18821349	-4.4486051	8.64E-06	6.98E-05
ENSDARG00000034409	pik3r3b	3077.10914	-0.502666836	0.11301083	-4.44795283	8.67E-06	7.00E-05
ENSDARG00000078703	unm_hu7910	888.636829	0.495785656	0.11148282	4.44719353	8.70E-06	7.02E-05
ENSDARG00000056235	smndc1	1810.03417	-0.564576711	0.12695975	-4.44689528	8.71E-06	7.02E-05
ENSDARG00000105442	LOXL4	422.518031	0.644063137	0.14483435	4.44689484	8.71E-06	7.02E-05
ENSDARG00000068227	tmem150aa	366.396064	0.669368418	0.15054644	4.44625877	8.74E-06	7.04E-05
ENSDARG00000103486	CT573799.1	18.0365915	-3.827983471	0.86154919	-4.44313978	8.87E-06	7.14E-05
ENSDARG00000056750	si:ch211-254p10.2	49.4188718	-1.766458454	0.39763913	-4.44236575	8.90E-06	7.16E-05
ENSDARG00000016584	rgs7a	598.144689	-0.64828289	0.14592219	-4.44266153	8.89E-06	7.16E-05
ENSDARG00000037071	rps26	8762.50515	0.548200929	0.12344518	4.44084507	8.96E-06	7.21E-05
ENSDARG00000102056	svild	3060.05414	0.475437324	0.10706921	4.44046729	8.98E-06	7.22E-05
ENSDARG00000027807	frk	463.684924	0.728367879	0.16404697	4.43999591	9.00E-06	7.24E-05
ENSDARG00000087394	tshz3a	618.760014	-0.548078826	0.12345233	-4.43959893	9.01E-06	7.25E-05
ENSDARG00000053446	sub1a	1944.12135	-0.562470291	0.12670083	-4.43935767	9.02E-06	7.25E-05
ENSDARG00000060609	cd109	420.914984	-0.76916678	0.17327381	-4.4390251	9.04E-06	7.26E-05
ENSDARG00000099943	mapre2	3222.73774	-0.506299481	0.11405892	-4.43892949	9.04E-06	7.26E-05
ENSDARG00000010437	tent5c	393.828492	0.794631358	0.17902126	4.4387541	9.05E-06	7.27E-05
ENSDARG00000102033	zgc:193790	73.9994977	-1.309709101	0.29507009	-4.43863732	9.05E-06	7.27E-05
ENSDARG00000012325	zgc:110269	375.673639	-0.71296148	0.1606878	-4.436936	9.12E-06	7.32E-05
ENSDARG00000056292	vsx1	2744.87724	-1.725715741	0.38899527	-4.43634115	9.15E-06	7.34E-05
ENSDARG00000042989	si:dkeyp-84f3.5	813.395276	-0.59204285	0.13347108	-4.43573894	9.18E-06	7.35E-05
ENSDARG00000103101	si:ch211-235e9.6	847.25394	0.625118005	0.14092651	4.435773	9.17E-06	7.35E-05
ENSDARG00000061233	abcc5	9784.99089	0.499849566	0.1126805	4.43599009	9.17E-06	7.35E-05
ENSDARG00000026531	alcama	3206.73536	0.381104273	0.08590901	4.43613869	9.16E-06	7.35E-05
ENSDARG00000032005	ccdc65	187.954832	1.107689639	0.24982702	4.43382646	9.26E-06	7.41E-05
ENSDARG00000098785	glrx3	2627.70653	0.475535149	0.10724822	4.43396774	9.25E-06	7.41E-05
ENSDARG00000012763	arl13b	645.487337	-0.662964294	0.14953401	-4.4335351	9.27E-06	7.42E-05
ENSDARG00000105470	si:dkey-23a13.2	42.7055989	2.274578903	0.51311856	4.43285251	9.30E-06	7.44E-05
ENSDARG00000076763	sp2	973.664051	-0.546371766	0.12326101	-4.43264072	9.31E-06	7.45E-05
ENSDARG00000070555	ldlrad4a	1195.76112	-0.642395048	0.14499587	-4.43043693	9.40E-06	7.52E-05
ENSDARG00000016065	tlr3	58.9051271	1.3144165	0.29677429	4.42901069	9.47E-06	7.57E-05
ENSDARG00000043816	st6galnac1.2	39.3672561	1.786369081	0.40335392	4.42878822	9.48E-06	7.57E-05
ENSDARG00000004632	egln1b	457.67509	0.574029696	0.12964196	4.42780784	9.52E-06	7.61E-05
ENSDARG00000075706	si:ch211-106h4.9	650.19685	-0.696701598	0.15736707	-4.42723871	9.54E-06	7.62E-05
ENSDARG00000041925	cryba2b	10769.6706	-0.635020318	0.14344977	-4.42677815	9.57E-06	7.64E-05
ENSDARG00000058943	cdcp1a	216.229934	1.040070306	0.23496422	4.42650507	9.58E-06	7.64E-05
ENSDARG00000011487	gnaq	364.131398	-0.677326193	0.15303316	-4.4260094	9.60E-06	7.65E-05
ENSDARG00000110877	FO834855.1	39.764507	-2.051249218	0.46344519	-4.42608804	9.60E-06	7.65E-05
ENSDARG00000070786	znf770	1098.85554	-0.535114972	0.12089383	-4.4263216	9.59E-06	7.65E-05
ENSDARG00000087659	si:ch211-219a15.3	113.456251	-1.299261971	0.29355409	-4.42597122	9.60E-06	7.65E-05
ENSDARG00000093591	si:ch211-282k23.2	42.2375551	2.201815808	0.49764679	4.42445501	9.67E-06	7.70E-05
ENSDARG00000113599	hbbe1.1	27934.0559	0.721353016	0.16312497	4.4220883	9.78E-06	7.78E-05
ENSDARG00000067832	tle2c	529.999609	-0.536973305	0.12142091	-4.42241212	9.76E-06	7.78E-05
ENSDARG00000005774	ddx3xb	18358.0806	-0.500061131	0.11309615	-4.42155768	9.80E-06	7.80E-05
ENSDARG00000105127	si:ch211-155e24.3	1017.63027	-0.738054364	0.16691929	-4.42162426	9.80E-06	7.80E-05
ENSDARG00000069044	agpat4	558.554594	0.570131609	0.12895279	4.42124305	9.81E-06	7.81E-05
ENSDARG00000044183	prkab1a	736.611075	-0.499760623	0.11303655	-4.42123024	9.81E-06	7.81E-05
ENSDARG00000069407	zgc:194990	209.347262	-1.155298837	0.26133107	-4.42082465	9.83E-06	7.82E-05
ENSDARG00000098153	lhfpl2b	739.28317	0.485332993	0.10979562	4.42033103	9.85E-06	7.83E-05
ENSDARG00000111638	CABZ01040998.1	197.118996	0.94625881	0.21407678	4.4201842	9.86E-06	7.84E-05
ENSDARG00000022631	cyp2p9	105.23592	-5.278181834	1.19418938	-4.41988675	9.88E-06	7.84E-05
ENSDARG00000062892	foxe3	43.6447963	-1.620406344	0.36668067	-4.4191213	9.91E-06	7.86E-05
ENSDARG00000071657	si:dkey-19a16.4	36.6182138	1.848492258	0.41829207	4.41914254	9.91E-06	7.86E-05

Table S2. DEGs of WT vs. *terfa-/-*

ENSDARG00000098644	si:ch211-126j24.1	2691.80638	-0.544119672	0.12312084	-4.41939554	9.90E-06	7.86E-05
ENSDARG00000033009	h3f3c	30161.5538	-0.493506764	0.11168672	-4.41867023	9.93E-06	7.88E-05
ENSDARG00000031562	tada2a	648.936061	-0.630630597	0.14273554	-4.4181751	9.95E-06	7.89E-05
ENSDARG00000098963	pcdh1g13	133.165158	-0.999463007	0.22620586	-4.41837808	9.94E-06	7.89E-05
ENSDARG00000079636	fam177a1	1215.0723	-0.554768576	0.12561631	-4.4163738	1.00E-05	7.96E-05
ENSDARG00000074050	efnb2b	470.501826	-0.65558937	0.14847303	-4.41554523	1.01E-05	7.98E-05
ENSDARG00000101561	dbt	2570.98914	0.403106268	0.09129462	4.41544388	1.01E-05	7.98E-05
ENSDARG00000104587	srl	6169.57397	0.575733306	0.13042239	4.41437467	1.01E-05	8.02E-05
ENSDARG00000103799	kars1	8452.41539	0.442215289	0.1001839	4.41403569	1.01E-05	8.03E-05
ENSDARG00000011862	inaa	301.416259	-1.417282305	0.32107701	-4.41415063	1.01E-05	8.03E-05
ENSDARG00000036577	atp6v0cb	9571.86274	-0.576588267	0.13062977	-4.41391179	1.02E-05	8.03E-05
ENSDARG00000075933	cox15	932.899985	0.57202901	0.12960673	4.41357492	1.02E-05	8.04E-05
ENSDARG00000070092	KCNV1	61.1538533	-1.553125301	0.35191238	-4.41338642	1.02E-05	8.05E-05
ENSDARG00000005629	smyd2b	1557.6689	0.422280186	0.09568985	4.41300904	1.02E-05	8.05E-05
ENSDARG00000076839	ftr86	581.455135	0.764427356	0.1732229	4.4129693	1.02E-05	8.05E-05
ENSDARG00000098308	CR936540.1	505.725265	0.732185631	0.16591175	4.4131029	1.02E-05	8.05E-05
ENSDARG00000005595	adgb	162.682559	0.883424483	0.20020048	4.41269913	1.02E-05	8.06E-05
ENSDARG00000103618	btr18	52.3105973	-1.979870856	0.44869726	-4.41248709	1.02E-05	8.07E-05
ENSDARG00000020521	exoc6	1235.34381	-0.474673597	0.1075875	-4.41197705	1.02E-05	8.08E-05
ENSDARG00000025641	gli2a	2244.25277	0.416474475	0.09441659	4.4110307	1.03E-05	8.11E-05
ENSDARG00000087087	col28a1b	62.2527814	-1.598800277	0.36244013	-4.41121205	1.03E-05	8.11E-05
ENSDARG00000010160	rps15a	21523.6697	0.466310908	0.10574269	4.40986445	1.03E-05	8.15E-05
ENSDARG00000010231	ppm1na	217.77593	-1.609113791	0.36488873	-4.40987526	1.03E-05	8.15E-05
ENSDARG00000110804	PARD3	146.501793	1.050582041	0.2382689	4.40922865	1.04E-05	8.17E-05
ENSDARG00000074081	dnai2b	75.6034929	1.368053827	0.31028169	4.40907038	1.04E-05	8.18E-05
ENSDARG00000087788	si:ch211-22k7.9	35.2579608	2.221804707	0.50404334	4.40796362	1.04E-05	8.22E-05
ENSDARG00000040306	otomp	2013.28277	0.488887169	0.11091516	4.40775804	1.04E-05	8.22E-05
ENSDARG00000051975	cd99	1587.6099	0.697649109	0.15831428	4.4067353	1.05E-05	8.25E-05
ENSDARG00000061664	C1H3orf70	310.527827	-0.886620582	0.20118986	-4.40688501	1.05E-05	8.25E-05
ENSDARG00000077139	col6a3	10620.6798	0.46078669	0.10456881	4.40654039	1.05E-05	8.25E-05
ENSDARG00000038422	entpd4	1069.81281	0.499474327	0.11334944	4.40650035	1.05E-05	8.25E-05
ENSDARG00000044975	krt94	2163.35358	0.550735409	0.12497373	4.4068093	1.05E-05	8.25E-05
ENSDARG00000004296	ambp	4580.80327	0.502551756	0.11404559	4.40658647	1.05E-05	8.25E-05
ENSDARG00000036394	pop7	826.179514	-0.708626948	0.16082583	-4.40617633	1.05E-05	8.26E-05
ENSDARG00000103747	cav1	4372.0327	0.519349286	0.11786488	4.40631092	1.05E-05	8.26E-05
ENSDARG00000041140	ddb2	594.733641	0.658100632	0.14938121	4.40551161	1.06E-05	8.28E-05
ENSDARG00000077224	ypel2a	206.334713	-0.764018233	0.17342444	-4.4054818	1.06E-05	8.28E-05
ENSDARG00000079684	taf6l	771.706142	-0.528760292	0.12003267	-4.4051366	1.06E-05	8.29E-05
ENSDARG00000017673	nova2	11178.3409	-0.696314559	0.15807484	-4.40496779	1.06E-05	8.30E-05
ENSDARG00000100047	ergic1	612.631095	0.548515792	0.12454664	4.40409955	1.06E-05	8.33E-05
ENSDARG00000102277	lama1	1900.4614	0.898294731	0.20399289	4.40355913	1.06E-05	8.34E-05
ENSDARG00000076306	loxl3a	156.061398	-1.406410035	0.31939297	-4.4033844	1.07E-05	8.35E-05
ENSDARG00000086665	si:dkey-175g6.2	542.699813	0.732923849	0.16645994	4.40300439	1.07E-05	8.36E-05
ENSDARG00000011094	ccna2	1694.66317	-1.012364624	0.22994461	-4.40264558	1.07E-05	8.37E-05
ENSDARG00000028982	pcca	2634.62653	0.425754155	0.09672699	4.40160675	1.07E-05	8.41E-05
ENSDARG00000038858	setd7	939.011726	0.570054214	0.12951224	4.40154699	1.07E-05	8.41E-05
ENSDARG00000074897	hes2.1	29.584112	-2.664854737	0.60561008	-4.40028133	1.08E-05	8.45E-05
ENSDARG00000058444	snx6	206.205334	-1.09248503	0.24826628	-4.40045676	1.08E-05	8.45E-05
ENSDARG00000074985	aurkaip1	1424.82198	0.50858567	0.11558707	4.40002217	1.08E-05	8.46E-05
ENSDARG00000087741	asb14b	385.977906	0.678168082	0.15415138	4.39936443	1.09E-05	8.48E-05
ENSDARG00000011407	col2a1b	48668.6835	0.427923514	0.09726697	4.39947396	1.09E-05	8.48E-05
ENSDARG00000103684	ap4e1	486.051788	-0.642249768	0.14600238	-4.39889918	1.09E-05	8.50E-05
ENSDARG00000043313	ank2b	11132.5866	-0.536188171	0.1219012	-4.39854728	1.09E-05	8.51E-05
ENSDARG00000034270	ogdha	10365.4317	0.451441899	0.10263949	4.39832547	1.09E-05	8.51E-05
ENSDARG00000103261	CABZ01038699.1	57.6636862	-2.195504654	0.49914986	-4.39848796	1.09E-05	8.51E-05
ENSDARG00000086327	srrm4	1429.58128	-0.584411947	0.13289322	-4.39760552	1.09E-05	8.53E-05
ENSDARG00000021483	cdc14b	1971.40543	-0.513402507	0.11674459	-4.3976557	1.09E-05	8.53E-05
ENSDARG00000093303	ifitm1	333.476143	1.433554075	0.32605006	4.39672997	1.10E-05	8.56E-05

Table S2. DEGs of WT vs. *terfa-/-*

ENSDARG00000086445	adgrf7	11.8733423	4.735203646	1.07698214	4.39673367	1.10E-05	8.56E-05
ENSDARG00000036482	hexim1	1996.16294	-0.479258618	0.10901101	-4.39642386	1.10E-05	8.57E-05
ENSDARG00000071224	si:ch211-133n4.4	3103.04958	-0.457646313	0.10409763	-4.3963181	1.10E-05	8.57E-05
ENSDARG00000004270	atl3	1671.47091	0.492058115	0.11193159	4.39606126	1.10E-05	8.58E-05
ENSDARG00000029331	atxn7l3	519.902747	-0.689388835	0.15684499	-4.39535121	1.11E-05	8.60E-05
ENSDARG00000076804	ttyh1	1995.52425	-0.85506586	0.19452935	-4.39556222	1.10E-05	8.60E-05
ENSDARG00000016864	farsb	1768.86658	0.575534623	0.13093369	4.39561893	1.10E-05	8.60E-05
ENSDARG00000061810	mbd3b	2217.61011	-0.710949044	0.16179244	-4.39420448	1.11E-05	8.64E-05
ENSDARG00000104260	MYORG	38.9872787	-2.567680754	0.58455123	-4.39256753	1.12E-05	8.71E-05
ENSDARG00000052016	cabp2a	145.484649	-1.545945082	0.35195813	-4.39241186	1.12E-05	8.71E-05
ENSDARG00000020235	sept9a	2579.69594	0.4227347	0.0962627	4.39146949	1.13E-05	8.74E-05
ENSDARG00000008904	smarca2	1510.08547	0.511890959	0.11657902	4.39093545	1.13E-05	8.76E-05
ENSDARG00000040087	epb41l4a	761.728809	0.640453369	0.14585187	4.39112214	1.13E-05	8.76E-05
ENSDARG00000038666	igfbp1b	162.185235	-1.241539869	0.28276872	-4.39065493	1.13E-05	8.77E-05
ENSDARG00000086266	CR925773.1	106.845268	1.380965388	0.3145891	4.38974331	1.13E-05	8.80E-05
ENSDARG00000037919	rbbp6	3115.91216	-0.399172178	0.09095966	-4.38845297	1.14E-05	8.85E-05
ENSDARG00000011235	otx2b	1792.01995	-1.677945486	0.38250889	-4.3866836	1.15E-05	8.92E-05
ENSDARG00000089245	dusp23b	473.142967	0.755364633	0.17227759	4.38457873	1.16E-05	9.01E-05
ENSDARG00000040326	dhx29	893.440674	0.465542884	0.10618304	4.3843432	1.16E-05	9.01E-05
ENSDARG00000061272	BX294434.1	2075.7499	0.484593779	0.11056013	4.38307893	1.17E-05	9.06E-05
ENSDARG00000097352	si:ch211-147g22.7	10.1590077	6.077902017	1.38688333	4.38241768	1.17E-05	9.09E-05
ENSDARG00000006757	klhl41b	4492.10403	0.551809328	0.12592631	4.38200175	1.18E-05	9.10E-05
ENSDARG00000103103	BX957361.2	37.6856352	-1.789480609	0.40838772	-4.38181783	1.18E-05	9.11E-05
ENSDARG00000024092	lmbr1	543.416648	-0.621370333	0.14181551	-4.38154017	1.18E-05	9.12E-05
ENSDARG00000101652	haus6	460.903018	-0.743356073	0.16968117	-4.38089907	1.18E-05	9.14E-05
ENSDARG00000027063	arpc1b	1977.54661	0.454140788	0.10368745	4.37990125	1.19E-05	9.17E-05
ENSDARG00000056829	zmp:0000000662	785.547352	0.528008916	0.12054558	4.38015988	1.19E-05	9.17E-05
ENSDARG00000104629	SLC16A6	244.490874	1.48167663	0.33829125	4.37988454	1.19E-05	9.17E-05
ENSDARG00000079997	si:zfos-223e1.2	206.515023	0.838659145	0.19148884	4.37967632	1.19E-05	9.18E-05
ENSDARG00000087388	si:dkey-29b11.3	23.4175873	-2.723758766	0.62206831	-4.3785525	1.19E-05	9.22E-05
ENSDARG00000091579	si:ch211-66e2.5	599.965003	0.78646556	0.17962107	4.37847057	1.20E-05	9.23E-05
ENSDARG00000076411	cavin1a	504.283035	0.709961912	0.16218067	4.37759889	1.20E-05	9.26E-05
ENSDARG00000002369	UBB	5011.40177	0.743910097	0.16995541	4.37708978	1.20E-05	9.28E-05
ENSDARG00000098099	zgc:64065	76.2303758	1.349200552	0.30826479	4.37675857	1.20E-05	9.29E-05
ENSDARG00000111249	CR394528.1	168.260626	1.124509503	0.25694636	4.37643672	1.21E-05	9.30E-05
ENSDARG00000062217	kcnj3b	445.009059	-0.730000921	0.16681595	-4.37608583	1.21E-05	9.30E-05
ENSDARG00000104823	znf648	1042.2922	0.876249309	0.20023328	4.3761423	1.21E-05	9.30E-05
ENSDARG00000016477	eif4a2	1397.83338	0.528291111	0.12071302	4.37642183	1.21E-05	9.30E-05
ENSDARG00000057556	rpl17	37054.7691	0.391051007	0.0893536	4.37644368	1.21E-05	9.30E-05
ENSDARG00000070931	si:ch211-232m10.(	134.673909	-1.326215793	0.30312413	-4.37515745	1.21E-05	9.34E-05
ENSDARG00000076386	epdl1	2259.87571	0.687293902	0.15710401	4.37476995	1.22E-05	9.35E-05
ENSDARG00000008723	prkcba	1911.24929	0.46043844	0.10527464	4.3736881	1.22E-05	9.39E-05
ENSDARG00000095643	si:dkey-253d23.3	211.392806	-0.925281328	0.21155156	-4.3737863	1.22E-05	9.39E-05
ENSDARG00000016038	hacd3	875.208786	-0.540587519	0.12360392	-4.37354664	1.22E-05	9.40E-05
ENSDARG00000059287	mak	1007.68242	-0.514655164	0.11768958	-4.37298845	1.23E-05	9.42E-05
ENSDARG00000061763	spata5l1	172.507827	0.982156809	0.22467789	4.37139952	1.23E-05	9.49E-05
ENSDARG00000014806	hacd2	2335.96918	-0.400961041	0.0917355	-4.37083854	1.24E-05	9.50E-05
ENSDARG00000095904	prpf31	1525.49184	-0.473659726	0.10836473	-4.37097676	1.24E-05	9.50E-05
ENSDARG00000110019	CABZ01079267.1	162.196591	0.978114553	0.22381131	4.37026417	1.24E-05	9.53E-05
ENSDARG00000004059	galk2	292.637526	1.100278014	0.25183357	4.36906802	1.25E-05	9.58E-05
ENSDARG00000099108	clic6	192.36912	-1.02002096	0.23356077	-4.36726153	1.26E-05	9.65E-05
ENSDARG00000063672	zmp:0000000711	658.145438	0.53421965	0.1223487	4.36636959	1.26E-05	9.69E-05
ENSDARG00000059858	pappab	271.666528	1.14901623	0.26315919	4.36624013	1.26E-05	9.69E-05
ENSDARG00000001676	gpm6bb	2420.14244	-0.614534525	0.14075565	-4.36596696	1.27E-05	9.70E-05
ENSDARG00000056322	ldb3a	5422.16199	0.603090114	0.13814506	4.36562921	1.27E-05	9.71E-05
ENSDARG00000098237	fbn2b	17621.2582	0.768092779	0.17594383	4.36555683	1.27E-05	9.71E-05
ENSDARG00000059801	pdzd2	1596.04583	-0.802422065	0.18386071	-4.36429325	1.28E-05	9.77E-05
ENSDARG00000102953	ints4	854.368288	-0.48724723	0.11166305	-4.3635495	1.28E-05	9.80E-05

Table S2. DEGs of WT vs. *terfa-/-*

ENSDARG00000006094	igf2r	1694.93095	0.76365118	0.17504319	4.36264439	1.28E-05	9.83E-05
ENSDARG00000074968	polr2m	193.883537	-0.874157521	0.20039403	-4.36219345	1.29E-05	9.85E-05
ENSDARG00000015678	si:dkeyp-75b4.9	14.9533849	3.587507294	0.82241002	4.36218822	1.29E-05	9.85E-05
ENSDARG00000087835	si:ch211-113p18.3	230.475048	-0.709940924	0.16275194	-4.36210427	1.29E-05	9.85E-05
ENSDARG00000058471	plk1	1921.31492	-0.82140998	0.18832731	-4.36160841	1.29E-05	9.87E-05
ENSDARG00000045273	tmem204	943.677385	0.580196566	0.13304175	4.36101111	1.29E-05	9.89E-05
ENSDARG00000078468	fap	1838.36979	-0.430150149	0.09864501	-4.3605872	1.30E-05	9.91E-05
ENSDARG00000016875	gys1	3287.15685	0.417128617	0.09566406	4.36034812	1.30E-05	9.92E-05
ENSDARG00000060954	tmcc1b	1535.23235	-0.409768671	0.09397947	-4.36019343	1.30E-05	9.92E-05
ENSDARG00000003531	tcea1	2075.13981	-0.590238012	0.13538836	-4.35959198	1.30E-05	9.94E-05
ENSDARG00000110637	si:ch211-212k5.1	105.164271	-1.321182318	0.3030764	-4.35923852	1.31E-05	9.96E-05
ENSDARG00000101913	CABZ01092156.1	455.315745	0.798915518	0.1832744	4.3591222	1.31E-05	9.96E-05
ENSDARG00000101049	CABZ01075274.1	43.4462658	1.857999483	0.42628387	4.35859677	1.31E-05	9.98E-05
ENSDARG00000112870	CABZ01021599.1	146.177733	0.884919736	0.20302521	4.35866927	1.31E-05	9.98E-05
ENSDARG00000019000	smc3	7618.39587	-0.541227169	0.12417689	-4.35851784	1.31E-05	9.98E-05
ENSDARG00000094088	si:ch211-229n2.7	164.699882	0.958282833	0.2199201	4.35741366	1.32E-05	0.00010025
ENSDARG00000102307	taf2	1397.46511	-0.497128379	0.11409284	-4.35722699	1.32E-05	0.0001003
ENSDARG00000006766	snd1	7692.78626	0.377352795	0.08660912	4.35696353	1.32E-05	0.00010039
ENSDARG00000037018	gab1	1154.89255	0.489536378	0.11237978	4.35608957	1.32E-05	0.00010076
ENSDARG00000022218	uck1	237.584049	-0.713071357	0.16373104	-4.3551385	1.33E-05	0.00010117
ENSDARG00000058486	caps2	57.453612	1.257865688	0.288886	4.35419403	1.34E-05	0.00010158
ENSDARG00000074979	larp4ab	1650.37803	0.521422766	0.11978391	4.35302857	1.34E-05	0.00010209
ENSDARG00000078022	fyco1b	980.388442	0.639532636	0.14696629	4.35156006	1.35E-05	0.00010274
ENSDARG00000074581	add2	2113.54221	-0.468909635	0.10783987	-4.34820291	1.37E-05	0.0001043
ENSDARG00000108788	ARSB	434.265085	0.728488731	0.16758574	4.34696137	1.38E-05	0.00010486
ENSDARG00000030465	tmem263	1299.78362	0.594127101	0.13671646	4.34568825	1.39E-05	0.00010543
ENSDARG00000075600	si:dkeyp-41f9.3	281.211961	-1.089912543	0.25082616	-4.34529049	1.39E-05	0.00010559
ENSDARG00000069673	chid1	644.846389	0.636013477	0.14637868	4.34498714	1.39E-05	0.00010571
ENSDARG00000052304	uqcrc1	13017.4873	0.387008156	0.08907719	4.34463804	1.40E-05	0.00010581
ENSDARG00000074796	dbf4	328.306655	-0.818818296	0.18846536	-4.34466199	1.39E-05	0.00010581
ENSDARG00000001880	stx3a	708.310735	-0.670532783	0.15441601	-4.34237864	1.41E-05	0.00010687
ENSDARG00000051965	btr01	246.974701	0.679530603	0.15650931	4.34179033	1.41E-05	0.00010713
ENSDARG00000039616	cenpk	158.53429	-1.019668945	0.23486871	-4.34144219	1.42E-05	0.00010726
ENSDARG00000088171	ciartb	432.499316	0.762954526	0.17576275	4.34082038	1.42E-05	0.00010754
ENSDARG00000070513	brpf3a	1188.86405	-0.524675676	0.12087456	-4.34066254	1.42E-05	0.00010758
ENSDARG00000094466	si:ch73-199e17.1	341.004236	-0.865291213	0.19934855	-4.34059451	1.42E-05	0.00010758
ENSDARG00000101308	smug1	354.184372	-0.781653237	0.18014126	-4.33911266	1.43E-05	0.00010828
ENSDARG00000087059	selenou1b	43.1795223	1.570928222	0.36220374	4.3371397	1.44E-05	0.00010922
ENSDARG00000076229	mrtfab	2243.01577	0.535556127	0.12350658	4.33625599	1.45E-05	0.00010962
ENSDARG00000010183	si:ch73-265h17.1	69.2742572	1.59676372	0.36825857	4.33598528	1.45E-05	0.00010973
ENSDARG00000053453	mpp2a	668.438863	-0.705657896	0.16275366	-4.33574217	1.45E-05	0.00010981
ENSDARG00000095369	zgc:112966	8.82902762	6.219585973	1.43453806	4.33560194	1.45E-05	0.00010985
ENSDARG00000100857	FP101914.1	373.264144	-0.900051166	0.20759914	-4.3355246	1.45E-05	0.00010986
ENSDARG00000059227	fabp1b.1	7349.53826	0.790244584	0.18228238	4.33527697	1.46E-05	0.00010995
ENSDARG00000056475	trnau1apb	2376.89158	-0.401160833	0.09253997	-4.33500058	1.46E-05	0.00011005
ENSDARG00000015314	zc4h2	4150.3862	-0.741489479	0.17111221	-4.33335233	1.47E-05	0.00011084
ENSDARG00000070809	znf516	2065.19202	-0.771443721	0.178037	-4.33305276	1.47E-05	0.00011096
ENSDARG00000016691	cd9b	2618.0446	0.559852145	0.12921393	4.33275396	1.47E-05	0.00011108
ENSDARG00000042642	wtap	2713.15276	-0.501132209	0.11566521	-4.3326096	1.47E-05	0.00011112
ENSDARG00000008920	mrpl53	400.469898	0.604998269	0.13965173	4.33219317	1.48E-05	0.00011129
ENSDARG00000104089	p3h4	588.395099	0.753269553	0.17388297	4.332049	1.48E-05	0.00011133
ENSDARG00000100642	plppr1	662.877657	-0.668550984	0.15435284	-4.33131638	1.48E-05	0.00011167
ENSDARG00000096637	si:dkey-11c5.11	34.6162082	1.865296403	0.43070286	4.33081964	1.49E-05	0.00011189
ENSDARG00000051956	isca1	1483.3064	0.639930952	0.14788029	4.32735809	1.51E-05	0.00011359
ENSDARG00000057484	srsf2a	6360.12952	-0.477566814	0.11035907	-4.32739082	1.51E-05	0.00011359
ENSDARG00000086996	si:ch73-234b20.5	442.302902	0.884713097	0.20445749	4.32712489	1.51E-05	0.00011368
ENSDARG00000040252	atp1a1a.5	359.564349	0.855809092	0.19779987	4.32664139	1.51E-05	0.00011389
ENSDARG00000070173	gria2a	3859.52228	-0.606812825	0.14026296	-4.32625143	1.52E-05	0.00011406

Table S2. DEGs of WT vs. *terfa-/-*

ENSDARG00000034693	mysm1	543.416439	-0.55613181	0.12856099	-4.32582099	1.52E-05	0.00011425
ENSDARG00000102904	si:ch211-246b8.2	22.7156154	2.469911499	0.57102193	4.32542319	1.52E-05	0.00011442
ENSDARG00000021151	ptpreb	924.140024	-0.584320071	0.13510892	-4.32480737	1.53E-05	0.00011471
ENSDARG00000044420	dnajc19	341.84982	0.792531164	0.18327237	4.32433529	1.53E-05	0.00011492
ENSDARG00000042091	btbd2a	1641.42877	-0.516403118	0.11946267	-4.32271544	1.54E-05	0.00011573
ENSDARG00000016858	smad7	563.495126	-0.67538953	0.15631704	-4.32063913	1.56E-05	0.00011679
ENSDARG00000100960	AL928901.1	10.9554989	3.55446216	0.82279862	4.31996611	1.56E-05	0.00011711
ENSDARG00000039453	dhrs7ca	146.029403	1.003392004	0.23227227	4.31989586	1.56E-05	0.00011711
ENSDARG00000088885	si:ch1073-340i21.3	989.051697	3.456361417	0.80017616	4.31950061	1.56E-05	0.00011729
ENSDARG00000019976	idi1	1820.51066	-0.467399395	0.10821213	-4.31928854	1.57E-05	0.00011736
ENSDARG00000063177	manf	1048.31036	0.559303725	0.12949596	4.3190823	1.57E-05	0.00011744
ENSDARG00000029232	FO704779.1	67.8055427	1.554067615	0.35983651	4.31881586	1.57E-05	0.00011755
ENSDARG00000045595	si:dkey-14k9.3	201.693486	-0.779960584	0.18060304	-4.31864586	1.57E-05	0.0001176
ENSDARG00000004211	ccdc106a	1056.13032	-0.536661603	0.1242684	-4.3185687	1.57E-05	0.00011761
ENSDARG00000004161	ik	3608.27659	-0.359886717	0.0833423	-4.31817595	1.57E-05	0.00011778
ENSDARG00000096829	blvrb	947.563272	0.58259612	0.13492683	4.31786709	1.58E-05	0.00011791
ENSDARG00000059057	atpv0e2	862.487088	-0.78759247	0.18240597	-4.31779993	1.58E-05	0.00011791
ENSDARG00000091760	cwf19l2	655.96115	0.741270925	0.17170026	4.31723823	1.58E-05	0.00011817
ENSDARG00000061923	amotl2a	3615.78536	-0.42979003	0.09957201	-4.31637391	1.59E-05	0.0001186
ENSDARG00000056922	ltbp1	2996.51477	0.508463981	0.1178138	4.3158269	1.59E-05	0.00011886
ENSDARG00000060298	nin	668.829432	-0.706317627	0.16367859	-4.31527184	1.59E-05	0.0001191
ENSDARG00000079572	plcd3b	390.681501	-0.654860743	0.15175523	-4.31524327	1.59E-05	0.0001191
ENSDARG00000098790	sowahca	133.125452	-1.23044089	0.28520171	-4.31428308	1.60E-05	0.00011959
ENSDARG00000019529	parp1	2863.13718	-0.532622823	0.12346549	-4.31394085	1.60E-05	0.00011974
ENSDARG00000089159	acot14	509.575787	0.713666071	0.16544534	4.31360635	1.61E-05	0.00011978
ENSDARG00000077690	mb21d2	914.745783	-0.668249804	0.15491728	-4.31359103	1.61E-05	0.00011978
ENSDARG00000022261	pdzk1	847.793242	1.01967391	0.23638549	4.31360619	1.61E-05	0.00011978
ENSDARG00000068263	csf1b	152.46587	0.867844381	0.20118702	4.3136202	1.61E-05	0.00011978
ENSDARG00000079884	trim107	130.884134	-0.969366654	0.22473474	-4.31338147	1.61E-05	0.00011986
ENSDARG00000077388	obsl1b	1737.88228	0.493705393	0.11450755	4.31155315	1.62E-05	0.00012082
ENSDARG00000103802	si:ch73-389b16.2	2180.78056	-0.524082395	0.12156306	-4.3111977	1.62E-05	0.00012098
ENSDARG00000092067	atn1	2604.28665	-0.586712526	0.13612557	-4.31008332	1.63E-05	0.00012155
ENSDARG00000089903	CABZ01039863.1	100.994241	1.116149874	0.25903231	4.3089215	1.64E-05	0.00012216
ENSDARG00000009881	ier5	3365.23958	-0.393341874	0.09129459	-4.30849043	1.64E-05	0.00012236
ENSDARG00000063451	ccdc13	44.9930398	1.748838083	0.40598628	4.30762849	1.65E-05	0.0001228
ENSDARG00000093044	si:ch211-161h7.5	170.051459	1.347252009	0.31283278	4.30662036	1.66E-05	0.00012332
ENSDARG00000042021	mapk12a	1244.71566	0.511423054	0.11876426	4.30620354	1.66E-05	0.00012352
ENSDARG00000090815	kcnj10a	253.535595	-1.020259567	0.23695037	-4.30579437	1.66E-05	0.00012371
ENSDARG00000069888	cldna	227.993062	0.879331099	0.20425586	4.30504709	1.67E-05	0.00012409
ENSDARG00000068820	h2az2a	3405.10284	-0.684206163	0.15893751	-4.30487542	1.67E-05	0.00012415
ENSDARG00000069375	zgc:162608	1057.23712	0.715005848	0.16610048	4.30465854	1.67E-05	0.00012423
ENSDARG00000010255	bin2b	251.036616	0.812601724	0.18878779	4.30431294	1.68E-05	0.00012439
ENSDARG00000016375	asns	1349.51864	0.836879203	0.19444505	4.30393672	1.68E-05	0.00012456
ENSDARG00000077047	ptprnb	569.921402	0.757077362	0.17591197	4.30372848	1.68E-05	0.00012464
ENSDARG00000090156	pttg1ipa	610.555212	0.688721136	0.16006579	4.3027378	1.69E-05	0.00012517
ENSDARG00000017835	brf1a	237.940979	0.736208229	0.17121175	4.29998668	1.71E-05	0.00012669
ENSDARG00000096411	si:dkey-261j15.2	293.802013	-0.706691904	0.1644023	-4.2985524	1.72E-05	0.00012748
ENSDARG00000041779	trdn	1956.53029	0.665393019	0.15480053	4.2983897	1.72E-05	0.00012753
ENSDARG00000040513	zgc:92313	858.1947	0.510806723	0.11885386	4.29777128	1.73E-05	0.00012785
ENSDARG00000060697	kmt2bb	4274.66848	-0.597575989	0.13906161	-4.29720316	1.73E-05	0.00012814
ENSDARG00000029439	atp2a2a	2095.80296	0.542395808	0.12627538	4.2953411	1.74E-05	0.00012918
ENSDARG00000100397	pde6c	4807.6166	-2.210683629	0.51468424	-4.2952231	1.75E-05	0.00012921
ENSDARG00000100596	si:ch73-43g23.1	3107.27266	0.786080847	0.18302488	4.29493985	1.75E-05	0.00012934
ENSDARG00000112100	neto2b	649.925797	-0.778537275	0.18129913	-4.29421409	1.75E-05	0.00012972
ENSDARG00000068367	nfkbie	553.297813	0.657210035	0.15307025	4.29351896	1.76E-05	0.00013009
ENSDARG00000010641	slc20a1b	7707.2646	0.386032096	0.08991798	4.29315786	1.76E-05	0.00013027
ENSDARG00000078172	CU984600.1	1572.86769	0.593107836	0.13820796	4.29141598	1.78E-05	0.00013125
ENSDARG00000045268	tectb	272.696079	-0.884005304	0.20600122	-4.29126243	1.78E-05	0.0001313

Table S2. DEGs of WT vs. *terfa-/-*

ENSDARG00000104487	mthfs	216.006102	0.881193343	0.20535721	4.29102713	1.78E-05	0.0001314
ENSDARG00000103371	si:dkey-190j3.6	9.41710377	4.127236487	0.96208454	4.28988964	1.79E-05	0.00013196
ENSDARG00000010400	lrrfip2	1462.56885	0.487593388	0.11365891	4.28997063	1.79E-05	0.00013196
ENSDARG00000034883	acbd5a	696.406968	-0.642759601	0.14983115	-4.28989299	1.79E-05	0.00013196
ENSDARG00000041895	cad	6506.53114	0.398590861	0.09294216	4.28859027	1.80E-05	0.0001327
ENSDARG00000017274	opn1sw2	8736.06323	-3.310495965	0.77202742	-4.28805491	1.80E-05	0.00013298
ENSDARG00000060862	atxn1b	2253.38546	0.452285256	0.10551012	4.28665282	1.81E-05	0.00013378
ENSDARG00000037884	rab30	515.431134	-0.561838212	0.13108257	-4.28613972	1.82E-05	0.00013405
ENSDARG00000032976	cmlc1	540.080895	0.534943415	0.12481663	4.28583465	1.82E-05	0.00013415
ENSDARG00000105456	FO834831.1	7.63717457	6.157238564	1.43664923	4.28583292	1.82E-05	0.00013415
ENSDARG00000104576		385.14874	-0.678529823	0.15835253	-4.28493212	1.83E-05	0.00013466
ENSDARG00000042613	crp3	240.56135	2.558165979	0.59705121	4.28466763	1.83E-05	0.00013478
ENSDARG00000071018	ptena	4548.50845	-0.439938591	0.10269455	-4.28395269	1.84E-05	0.00013517
ENSDARG00000009390	npl	631.792188	0.748366626	0.17469847	4.28376185	1.84E-05	0.00013525
ENSDARG00000016177	eif4enif1	952.533156	-0.554637622	0.12948742	-4.28333227	1.84E-05	0.00013547
ENSDARG00000053460	si:dkey-283b1.7	25.6611407	-2.576642766	0.60158337	-4.28310175	1.84E-05	0.00013557
ENSDARG00000057714	cmah	1276.97958	0.668657662	0.15614046	4.28241133	1.85E-05	0.00013595
ENSDARG00000105584	slc8b1	406.443111	0.723713324	0.16902418	4.28171484	1.85E-05	0.00013634
ENSDARG00000102176	pla1a	96.460637	1.354711436	0.3164664	4.28074337	1.86E-05	0.00013689
ENSDARG00000033201	crip2l	861.803081	-0.6425844	0.1501312	-4.28015241	1.87E-05	0.00013722
ENSDARG00000102200	man2a1	346.547509	0.779033229	0.18202702	4.279767	1.87E-05	0.00013741
ENSDARG00000027867	paplna	2238.11971	0.829076548	0.19379116	4.27819597	1.88E-05	0.00013835
ENSDARG00000019001	ak7a	239.757872	1.03036522	0.24088875	4.27734887	1.89E-05	0.00013883
ENSDARG00000104887	znf1105	56.5792329	-1.394542744	0.32608988	-4.27655943	1.90E-05	0.00013929
ENSDARG00000067784	SLC9A1	178.10926	0.778021433	0.18193653	4.27633427	1.90E-05	0.00013939
ENSDARG00000075459	ptpn20	347.709877	-0.908395264	0.21249417	-4.27491847	1.91E-05	0.00014023
ENSDARG00000079227	plekhs1	62.8109414	-1.687361788	0.39471706	-4.27486406	1.91E-05	0.00014023
ENSDARG00000012141	papolg	3074.11472	-0.464184341	0.1086042	-4.274092	1.92E-05	0.00014067
ENSDARG00000009466	rgs9bp	78.7688715	-1.277326298	0.29899627	-4.27204757	1.94E-05	0.00014192
ENSDARG00000079688	tnrc6a	8080.58733	-0.422769825	0.09898286	-4.27114187	1.94E-05	0.00014246
ENSDARG00000055589	s100t	1721.43192	-0.621098163	0.14542229	-4.27099705	1.95E-05	0.00014251
ENSDARG00000100552	taco1	405.394672	0.981185872	0.2297613	4.27045762	1.95E-05	0.00014281
ENSDARG00000100244	ebf3a	5522.30122	-0.662082692	0.1550502	-4.27011827	1.95E-05	0.00014299
ENSDARG00000077782	acer2	204.658238	0.868317331	0.20335677	4.26992101	1.96E-05	0.00014307
ENSDARG00000059557	xylt2	360.978087	0.855432049	0.20035397	4.26960364	1.96E-05	0.00014324
ENSDARG00000097196	CR847531.1	777.180813	-0.883100451	0.20684067	-4.26947203	1.96E-05	0.00014328
ENSDARG00000116863	CU984579.1	156.956788	1.118500597	0.26198065	4.26940164	1.96E-05	0.00014328
ENSDARG00000088411	notum2	437.711512	-0.609634979	0.14280357	-4.26904565	1.96E-05	0.00014347
ENSDARG00000074372	elfn1b	263.307211	-0.864954139	0.20262421	-4.26876014	1.97E-05	0.00014361
ENSDARG00000055786	prss23	972.868789	0.509968431	0.11950537	4.26732643	1.98E-05	0.00014449
ENSDARG00000036616	gpr182	353.552003	0.740084756	0.17343886	4.26712192	1.98E-05	0.00014458
ENSDARG00000078258	CABZ01049847.1	314.388782	-0.879664655	0.20623054	-4.2654432	2.00E-05	0.00014563
ENSDARG00000030514	acsl1a	1628.22508	0.457791451	0.10733373	4.26512209	2.00E-05	0.00014575
ENSDARG00000076135	mmrn2a	1115.52302	-0.421780897	0.09888954	-4.26517213	2.00E-05	0.00014575
ENSDARG00000092968	znf1128	36.9839672	1.850800143	0.43405024	4.26402288	2.01E-05	0.00014643
ENSDARG00000103184	PAMR1	329.921261	0.809873069	0.18997401	4.26307299	2.02E-05	0.00014694
ENSDARG00000013076	bmi1b	595.628769	-0.549211831	0.12883056	-4.26305567	2.02E-05	0.00014694
ENSDARG00000093320	BX323028.1	13.450547	4.240485455	0.99467972	4.2631667	2.02E-05	0.00014694
ENSDARG00000035891	acana	3699.24701	0.568715741	0.13340899	4.26294927	2.02E-05	0.00014696
ENSDARG00000031435	TXN	6988.30025	0.484591448	0.11371151	4.26158645	2.03E-05	0.00014782
ENSDARG00000004173	copa	11517.3064	0.411576826	0.09658319	4.26137143	2.03E-05	0.00014792
ENSDARG00000075898	spata7	407.700561	-0.769409266	0.18057727	-4.26083125	2.04E-05	0.00014823
ENSDARG00000099970	CR383676.2	23473.4206	0.671974165	0.157715	4.26068639	2.04E-05	0.00014829
ENSDARG00000105562	si:dkey-23i12.7	27.7406629	3.450184537	0.80995347	4.25973177	2.05E-05	0.00014888
ENSDARG00000008551	pole3	877.143532	-0.626993987	0.14725255	-4.25795004	2.06E-05	0.00015002
ENSDARG00000006527	brd3a	2342.9455	-0.515331022	0.12103263	-4.2577858	2.06E-05	0.00015009
ENSDARG00000009823	pou3f1	2434.46762	-0.727617867	0.17093779	-4.25662377	2.08E-05	0.00015083
ENSDARG00000044514	st6gal1	365.730719	-0.757347111	0.177933	-4.25636106	2.08E-05	0.00015096

Table S2. DEGs of WT vs. *terfa-/-*

ENSDARG00000070165	tnfrsf1b	348.107398	0.803391068	0.18876915	4.25594468	2.08E-05	0.0001512
ENSDARG00000019179	lrit1a	267.398072	-1.24040644	0.2914587	-4.2558566	2.08E-05	0.00015121
ENSDARG00000018283	cyba	318.039975	0.638995326	0.15017349	4.2550474	2.09E-05	0.00015172
ENSDARG00000027183	namptb	681.789834	-0.539364695	0.12676839	-4.25472562	2.09E-05	0.00015189
ENSDARG00000097292	slc26a6	82.849984	1.489539625	0.35015867	4.25389905	2.10E-05	0.00015241
ENSDARG00000093549	selenop	26578.1258	0.619878039	0.14574564	4.25314989	2.11E-05	0.00015288
ENSDARG00000045011	tapbp.2	60.4066962	1.711281074	0.4024621	4.25203037	2.12E-05	0.0001536
ENSDARG00000069435	cfap58	90.0955488	1.421481516	0.33435393	4.25142758	2.12E-05	0.00015397
ENSDARG00000033965	nup58	1467.00471	-0.513907083	0.12088184	-4.25131737	2.13E-05	0.000154
ENSDARG00000042874	phlda2	685.286061	0.611733587	0.14390686	4.25089948	2.13E-05	0.00015424
ENSDARG00000061562	tmem121aa	176.458024	-0.860084675	0.20235091	-4.25046107	2.13E-05	0.0001545
ENSDARG00000105408	BX248082.2	141.393438	1.140048991	0.26822318	4.2503746	2.13E-05	0.00015451
ENSDARG00000030155	ssbp3b	2521.26071	-0.514845846	0.12114911	-4.24968756	2.14E-05	0.00015494
ENSDARG00000057882	arpc3	3047.5309	0.413655727	0.09736863	4.24834717	2.15E-05	0.00015583
ENSDARG00000026834	cdr2l	1713.26421	-0.51703912	0.12170881	-4.24816509	2.16E-05	0.00015586
ENSDARG00000057610	cdkn2c	100.292788	-1.128805767	0.2657158	-4.24816959	2.16E-05	0.00015586
ENSDARG00000094899	si:ch1073-143l10.2	96.9346588	1.272610226	0.29958872	4.24785759	2.16E-05	0.00015603
ENSDARG00000104166	si:ch211-232d10.1	92.9553774	-1.382862982	0.32562924	-4.24674084	2.17E-05	0.00015676
ENSDARG00000054378	pcbp3	1825.96681	-0.946811883	0.22295572	-4.24663651	2.17E-05	0.00015679
ENSDARG00000004697	rxrgb	370.526583	-0.727350291	0.17128297	-4.24648339	2.17E-05	0.00015681
ENSDARG00000052856	khdrbs1a	28694.1379	-0.607355341	0.1430248	-4.24650385	2.17E-05	0.00015681
ENSDARG00000105411	si:ch211-113d11.5	883.318284	-2.680682201	0.63130491	-4.24625594	2.17E-05	0.00015692
ENSDARG00000010052	ndrg3b	2406.51491	-0.410846458	0.09677092	-4.24555723	2.18E-05	0.00015737
ENSDARG00000105142	tcirg1b	1236.80382	0.81305045	0.19153061	4.24501568	2.19E-05	0.0001577
ENSDARG00000037574	rps6kal	2151.15843	-0.444682365	0.1047562	-4.24492644	2.19E-05	0.00015772
ENSDARG00000009315	clgn	2311.23703	-0.531546717	0.12522711	-4.24466178	2.19E-05	0.00015786
ENSDARG00000006604	nectin3b	587.85731	0.523984117	0.12346204	4.24409078	2.19E-05	0.00015821
ENSDARG00000074899	taok2a	3154.20281	-0.474813277	0.11189664	-4.24332011	2.20E-05	0.00015871
ENSDARG00000032630	neb	41974.9447	0.812594687	0.19150944	4.24310508	2.20E-05	0.00015882
ENSDARG00000077069	srgn	446.638133	0.812526868	0.19153034	4.24228806	2.21E-05	0.00015935
ENSDARG00000113780	zgc:173615	40.2554952	2.013943554	0.47475316	4.24208564	2.21E-05	0.00015945
ENSDARG00000022101	obscnb	4584.91615	0.973690792	0.22958719	4.24105023	2.22E-05	0.00016014
ENSDARG00000099346	znf1079	6.97720402	6.168380392	1.45457449	4.24067687	2.23E-05	0.00016036
ENSDARG00000076831	iqck	109.64828	0.944192616	0.22270416	4.23967203	2.24E-05	0.00016102
ENSDARG00000019636	yif1a	896.98466	0.53548398	0.12630452	4.23962643	2.24E-05	0.00016102
ENSDARG00000077489	lrfn2b	580.213579	-0.813141851	0.19180726	-4.23936947	2.24E-05	0.00016116
ENSDARG00000116915	si:ch211-133h13.1	50.7751183	1.305321999	0.30798091	4.23832117	2.25E-05	0.00016187
ENSDARG00000038106	slc37a4a	953.936439	0.709774621	0.16747201	4.23816856	2.25E-05	0.00016193
ENSDARG00000042824	nfe2l2a	3865.00835	0.481056699	0.11350995	4.23801369	2.26E-05	0.00016199
ENSDARG00000076013	mab21l3	46.0566338	-1.90587395	0.44972292	-4.23788481	2.26E-05	0.00016204
ENSDARG00000061468	ncaph	1019.89908	-0.829742958	0.19579946	-4.23771828	2.26E-05	0.00016207
ENSDARG00000102105	dad1	1729.83792	0.479165663	0.11307036	4.23776536	2.26E-05	0.00016207
ENSDARG00000044807	dck	240.733409	-0.965621515	0.22791519	-4.23675797	2.27E-05	0.00016271
ENSDARG00000104596	gtf2a1l	847.355875	-0.489079474	0.1154429	-4.23654871	2.27E-05	0.00016282
ENSDARG00000051955	brms1	448.929099	-0.588941765	0.13905038	-4.23545603	2.28E-05	0.00016356
ENSDARG00000015015	tspan18b	1551.54472	-0.54128644	0.12782617	-4.23455096	2.29E-05	0.00016418
ENSDARG00000114451	mmp13a	248.135392	4.105672711	0.96967234	4.23408251	2.29E-05	0.00016447
ENSDARG00000021374	cab39l1	1545.23075	0.631571775	0.14916739	4.23398029	2.30E-05	0.0001645
ENSDARG00000117751	CABZ01112025.1	80.3597983	1.274974478	0.30115767	4.23357795	2.30E-05	0.00016475
ENSDARG00000018010	crtap	2199.54163	0.568803691	0.13440755	4.23193261	2.32E-05	0.00016591
ENSDARG00000069495	tmem179b	127.581652	-0.900371263	0.21279856	-4.23109664	2.33E-05	0.00016648
ENSDARG00000062156	abi3bpa	128.85839	-1.061490672	0.25090102	-4.23071488	2.33E-05	0.00016671
ENSDARG00000010244	rpl22l1	4944.9705	0.462654495	0.10938407	4.22963332	2.34E-05	0.00016747
ENSDARG00000093597	oip5	55.6010176	-1.548319066	0.36609336	-4.22930118	2.34E-05	0.00016767
ENSDARG00000068166	whrnb	197.028425	-0.868179333	0.20535354	-4.22773015	2.36E-05	0.00016879
ENSDARG00000102616	si:dkey-3h2.3	15.5057949	3.412617063	0.80726885	4.22736125	2.36E-05	0.00016902
ENSDARG00000105045	bmpr1ab	1768.68664	0.397491687	0.09402994	4.22728859	2.37E-05	0.00016903
ENSDARG00000013524	cyp2ae1	148.458678	1.056981977	0.25005393	4.2270161	2.37E-05	0.00016919

Table S2. DEGs of WT vs. *terfa-/-*

ENSDARG00000034982	zcchc8	897.28104	-0.569106509	0.13466694	-4.22602982	2.38E-05	0.00016988
ENSDARG00000115248	CDK2AP1	343.688599	-0.803113543	0.19004396	-4.22593558	2.38E-05	0.0001699
ENSDARG00000101184	BX510922.1	50.9569492	-1.630952909	0.38599889	-4.22527871	2.39E-05	0.00017035
ENSDARG00000031632	chst10	503.761242	-0.571846607	0.13536232	-4.22456284	2.39E-05	0.00017084
ENSDARG00000100719	uraha	194.461322	1.35336991	0.32036355	4.22448158	2.39E-05	0.00017085
ENSDARG00000033170	sult2st1	1617.50497	0.544805997	0.12896864	4.22432932	2.40E-05	0.00017092
ENSDARG00000024844	max	4326.12094	-0.395029331	0.09352173	-4.22393088	2.40E-05	0.00017117
ENSDARG00000074996	tbrg1	403.086575	-0.580077705	0.13734067	-4.22364115	2.40E-05	0.00017135
ENSDARG00000106248	LRFN2	461.150789	-0.718126594	0.17007098	-4.22251095	2.42E-05	0.00017216
ENSDARG00000075980	tmem125b	136.420307	-1.113818292	0.26380555	-4.22211851	2.42E-05	0.00017241
ENSDARG00000022978	tmpob	2314.26627	-0.725943516	0.17194761	-4.22188771	2.42E-05	0.00017254
ENSDARG00000038743	copb2	7951.3484	0.397985571	0.09427121	4.22170856	2.42E-05	0.00017262
ENSDARG00000028664	ahsa1a	318.560689	0.762629855	0.18066775	4.22117325	2.43E-05	0.00017298
ENSDARG00000091326	cep120	953.248083	-0.553150381	0.13106661	-4.22037591	2.44E-05	0.00017355
ENSDARG00000098802	map7d1a	1215.36739	0.446786128	0.10586832	4.22020599	2.44E-05	0.00017363
ENSDARG00000101458	r3hdm1	5992.98436	-0.481035274	0.11398937	-4.22000108	2.44E-05	0.00017374
ENSDARG00000076644	kcnv2a	63.6935398	-1.570405002	0.37216661	-4.21962901	2.45E-05	0.00017397
ENSDARG00000079074	frmpd1a	141.782632	-1.275291756	0.30224562	-4.21938871	2.45E-05	0.00017411
ENSDARG00000058280	si:dkey-208b23.5	65.0426771	1.23161133	0.2919048	4.21922267	2.45E-05	0.00017419
ENSDARG00000101865	pcdh1g30	650.384461	-0.825962381	0.19580183	-4.21835875	2.46E-05	0.00017481
ENSDARG00000096804	si:dkey-219e21.4	187.218516	0.766651649	0.18177152	4.21766654	2.47E-05	0.00017529
ENSDARG00000096688	CU660013.1	14.4580564	-4.536462963	1.07570407	-4.21720348	2.47E-05	0.0001756
ENSDARG00000039943	tent5ba	1670.31323	0.537396798	0.12744966	4.2165418	2.48E-05	0.00017607
ENSDARG00000006174	lrrc23	64.3352686	1.291512737	0.30636874	4.21555001	2.49E-05	0.0001768
ENSDARG00000056450	MANEAL	612.642367	-0.519982301	0.12336155	-4.21510847	2.50E-05	0.00017709
ENSDARG00000100573	ckap2l	507.605128	-0.878833355	0.20850999	-4.21482617	2.50E-05	0.00017726
ENSDARG00000100892	cfap53	92.8281467	1.407683657	0.33400008	4.21462069	2.50E-05	0.00017737
ENSDARG00000043211	ripk4	561.528815	0.549862136	0.13046874	4.21451251	2.50E-05	0.00017741
ENSDARG00000032838	si:dkey-206f10.1	443.465415	-2.070900913	0.49140686	-4.21422875	2.51E-05	0.00017758
ENSDARG00000068217	stx2b	504.787293	0.624339231	0.14817132	4.21363071	2.51E-05	0.000178
ENSDARG00000043403	paox1	280.499073	0.714115399	0.16948877	4.21334933	2.52E-05	0.00017817
ENSDARG00000029072	klf6a	4574.87456	0.517648481	0.12286943	4.21299639	2.52E-05	0.0001784
ENSDARG00000091150	mki67	7265.21004	-0.732531703	0.17388029	-4.21285074	2.52E-05	0.00017846
ENSDARG00000010070	adam9	1434.51606	0.448020688	0.10636419	4.21213829	2.53E-05	0.00017893
ENSDARG00000087417	zdhhc5b	869.277981	-0.436536898	0.10363639	-4.21219697	2.53E-05	0.00017893
ENSDARG00000014103	dkk1a	145.084021	0.853443684	0.2026323	4.21178503	2.53E-05	0.00017915
ENSDARG00000098082	CU459093.1	190.28366	-1.267093547	0.30089472	-4.21108608	2.54E-05	0.00017966
ENSDARG00000100231	ccdc135	164.730368	1.112431426	0.26421429	4.21033793	2.55E-05	0.0001802
ENSDARG00000039964	fgfbp2a	149.11618	0.967286549	0.22982519	4.20879264	2.57E-05	0.00018139
ENSDARG00000063563	creb3l2	1631.00207	0.503017125	0.11951925	4.20867055	2.57E-05	0.00018144
ENSDARG00000068572	slc16a1b	2898.86795	0.498798939	0.11854465	4.20768824	2.58E-05	0.00018217
ENSDARG00000057248	sclt1	506.816172	-0.604170935	0.14359287	-4.20752752	2.58E-05	0.00018225
ENSDARG00000101848	dydc2	53.7664906	1.508558111	0.35855648	4.20730957	2.58E-05	0.00018238
ENSDARG00000062056	elmod1	2750.05766	-0.566305383	0.13462082	-4.20667009	2.59E-05	0.00018284
ENSDARG00000077687	rp1	21.56831	-3.204759855	0.76185896	-4.2065002	2.59E-05	0.00018287
ENSDARG00000001710	flot1a	4044.19001	-0.40866629	0.09715103	-4.20650503	2.59E-05	0.00018287
ENSDARG00000018264	trim101	1061.62767	0.728679808	0.17324409	4.20608747	2.60E-05	0.00018316
ENSDARG00000030153	gnptab	1559.20693	0.398107342	0.09467205	4.20512026	2.61E-05	0.00018389
ENSDARG00000079823	mrps36	1744.56883	0.46537639	0.1106716	4.20502076	2.61E-05	0.00018392
ENSDARG00000003200	foxm1	382.682823	-0.818464115	0.19466028	-4.20457694	2.62E-05	0.00018423
ENSDARG00000112575	CU467905.1	202.441561	1.565916769	0.37250667	4.2037282	2.63E-05	0.00018487
ENSDARG00000062420	nfia	1373.97072	-0.733923176	0.17459304	-4.20362229	2.63E-05	0.0001849
ENSDARG00000100114	sf3a3	2263.55621	-0.502046596	0.11944198	-4.20326741	2.63E-05	0.00018514
ENSDARG00000004721	mpp5b	141.449871	-1.291201413	0.30720293	-4.20308953	2.63E-05	0.00018523
ENSDARG00000038991	nsfb	1258.35845	0.43619747	0.10379155	4.20262998	2.64E-05	0.00018556
ENSDARG00000024771	slc24a5	214.482928	0.769195132	0.18304106	4.20230919	2.64E-05	0.00018576
ENSDARG00000091726	aebp1	1842.20222	0.389182758	0.09261278	4.20225768	2.64E-05	0.00018576
ENSDARG00000068657	irgq2	41.5261186	-1.894989369	0.4510062	-4.20169247	2.65E-05	0.00018617

Table S2. DEGs of WT vs. *terfa-/-*

ENSDARG00000070844	gamt	18651.1509	0.557182222	0.13262805	4.2010888	2.66E-05	0.00018661
ENSDARG00000075783	hmgxb4a	229.831676	-0.810095111	0.19284801	-4.20069214	2.66E-05	0.00018689
ENSDARG00000105212	cfhl1	107.137353	1.345855474	0.32041299	4.20037742	2.66E-05	0.00018709
ENSDARG00000077762	si:rp71-79p20.2	177.644743	0.884656682	0.21064152	4.19982097	2.67E-05	0.0001875
ENSDARG00000104387	SLC4A5	500.890936	-0.776774832	0.18503269	-4.19804115	2.69E-05	0.00018893
ENSDARG00000071694	ndc80	1010.84526	-0.754146478	0.17966903	-4.19742062	2.70E-05	0.00018939
ENSDARG00000067976	ar	243.917172	0.795705722	0.18957373	4.19734174	2.70E-05	0.00018941
ENSDARG00000057055	ndufs8b	32.7842923	-2.222049293	0.52946706	-4.19676589	2.71E-05	0.00018983
ENSDARG00000096489	BX248324.1	141.948708	-1.310048404	0.31217281	-4.1965487	2.71E-05	0.00018991
ENSDARG00000074328	apba1b	1804.50186	-0.659913607	0.15725038	-4.19657866	2.71E-05	0.00018991
ENSDARG00000068732	spry4	1649.27613	0.502538839	0.11976115	4.19617592	2.71E-05	0.00019017
ENSDARG00000052057	pcolceb	989.562088	-0.824520828	0.19649798	-4.19607796	2.72E-05	0.0001902
ENSDARG00000101085	ss18l2	232.5394	-0.984763918	0.23469216	-4.19598141	2.72E-05	0.00019022
ENSDARG00000092488	khnyn	855.137907	0.514806193	0.12273802	4.1943499	2.74E-05	0.00019154
ENSDARG00000056888	dnah5l	320.426821	1.162293675	0.27729448	4.19155	2.77E-05	0.00019387
ENSDARG00000036041	f2	3680.3646	0.415028491	0.09902568	4.19111976	2.78E-05	0.00019418
ENSDARG00000029290	stx11b.1	862.661306	0.572692863	0.13666544	4.19047311	2.78E-05	0.00019468
ENSDARG00000052787	zdhhc12b	247.074235	-0.901798788	0.2152751	-4.18905294	2.80E-05	0.00019585
ENSDARG00000042688	bora	184.779558	-1.01034693	0.24119798	-4.18886977	2.80E-05	0.00019595
ENSDARG00000110845	pimr107	7.12574674	6.148957432	1.4679529	4.1887975	2.80E-05	0.00019596
ENSDARG00000053528	nog3	566.409524	-0.667302158	0.15934189	-4.18786393	2.82E-05	0.00019671
ENSDARG00000078953	epn3a	1316.70378	0.467254782	0.11157818	4.18768975	2.82E-05	0.00019681
ENSDARG00000052649	ube2ia	1544.88214	-0.523028157	0.1249191	-4.18693497	2.83E-05	0.00019741
ENSDARG00000099195	ier2a	1122.61497	-0.679555619	0.16231043	-4.18676502	2.83E-05	0.0001975
ENSDARG00000075249	fam171a2a	1958.65713	0.398182254	0.09510847	4.18661192	2.83E-05	0.00019758
ENSDARG00000116556	im:7142702	1002.24974	-0.631350632	0.15080462	-4.18654693	2.83E-05	0.00019758
ENSDARG00000100426	fam167b	320.710521	-0.77220121	0.18452868	-4.18472201	2.86E-05	0.00019912
ENSDARG00000117457	FP236157.5	21.0131373	2.131381853	0.5093797	4.18426934	2.86E-05	0.00019946
ENSDARG00000009629	unc119.1	783.734455	-0.616003952	0.14722522	-4.18409256	2.86E-05	0.00019956
ENSDARG00000041811	rps25	19605.6301	0.420651163	0.10053766	4.18401585	2.86E-05	0.00019957
ENSDARG00000038905	hectd3	2194.61215	0.37238762	0.08900941	4.18368845	2.87E-05	0.0001998
ENSDARG00000059870	clk2b	748.122691	-0.614127385	0.14681042	-4.18313218	2.88E-05	0.00020019
ENSDARG00000045802	hapln3	891.457987	0.526663665	0.12590202	4.18312326	2.88E-05	0.00020019
ENSDARG00000037373	ephb2a	609.523782	-0.988939282	0.23643974	-4.18262716	2.88E-05	0.00020057
ENSDARG00000012002	slc38a7	778.447058	0.596728871	0.14270249	4.18162899	2.89E-05	0.00020139
ENSDARG00000036767	urgcp	279.326576	1.395582005	0.33386351	4.18009749	2.91E-05	0.0002027
ENSDARG00000099918	arl4aa	300.364802	-0.616868207	0.14762356	-4.17865677	2.93E-05	0.00020393
ENSDARG00000097372	si:ch211-139d20.3	35.5164893	-1.884292683	0.4509504	-4.17849211	2.93E-05	0.00020402
ENSDARG00000077399	ubash3bb	152.160133	-0.852880346	0.2041455	-4.17780629	2.94E-05	0.00020458
ENSDARG00000061759	kdm6a	2521.94005	-0.388439498	0.09298147	-4.17760106	2.95E-05	0.0002047
ENSDARG00000077794	CU468012.1	24.8743219	2.716272757	0.65024809	4.17728677	2.95E-05	0.00020493
ENSDARG00000020607	emc3	2153.14625	-0.459932172	0.1101223	-4.17655799	2.96E-05	0.00020553
ENSDARG00000115540	cacng4a	41.7237939	-1.66312958	0.39828406	-4.17573722	2.97E-05	0.00020622
ENSDARG00000101200	zgc:112964	1039.41354	-0.454056634	0.10875543	-4.17502498	2.98E-05	0.0002068
ENSDARG00000042829	si:dkey-30j22.1	1135.0225	1.062810769	0.25463909	4.17379273	3.00E-05	0.00020787
ENSDARG00000053130	pcp4a	1045.06285	-0.643449916	0.15417917	-4.17339072	3.00E-05	0.00020818
ENSDARG00000015065	c4	54.4272875	1.738910647	0.41678409	4.17220981	3.02E-05	0.0002092
ENSDARG00000018069	rdh12	480.647813	-0.644519468	0.15450931	-4.1713956	3.03E-05	0.00020989
ENSDARG00000008866	cabp4	29.270414	-2.19161959	0.5254437	-4.17098844	3.03E-05	0.00021021
ENSDARG00000020007	col1a2	102528.191	0.441799529	0.10594848	4.1699468	3.05E-05	0.00021111
ENSDARG00000068993	zgc:153631	339.709975	-0.986161115	0.23649684	-4.16987015	3.05E-05	0.00021112
ENSDARG00000057159	ankrd29	152.297789	-0.876618481	0.21023074	-4.16979213	3.05E-05	0.00021114
ENSDARG00000016260	fxr2	5489.89764	0.494326609	0.11856427	4.16927134	3.06E-05	0.00021155
ENSDARG00000100252	capzb	11011.7312	0.369682818	0.08866955	4.16921945	3.06E-05	0.00021155
ENSDARG00000039486	bag3	609.550309	0.677192703	0.16244216	4.16882345	3.06E-05	0.00021186
ENSDARG00000038976	pdcd7	367.940092	-0.630672561	0.15129822	-4.16840713	3.07E-05	0.00021219
ENSDARG00000097047	BX629346.1	62.524443	-1.424205537	0.3417116	-4.16785837	3.07E-05	0.00021264
ENSDARG00000100918	prc1a	902.698947	-0.866374379	0.20789678	-4.16732948	3.08E-05	0.00021308

Table S2. DEGs of WT vs. *terfa-/-*

ENSDARG00000043137	cdca8	687.743016	-0.874602394	0.2098926	-4.16690441	3.09E-05	0.00021341
ENSDARG00000100954	wars1	584.987197	0.69974301	0.16798839	4.16542482	3.11E-05	0.00021474
ENSDARG00000060340	si:dkey-157l19.2	191.837584	0.986516489	0.2368389	4.16534823	3.11E-05	0.00021475
ENSDARG00000069101	napbb	776.038321	-0.782897566	0.18798602	-4.16465854	3.12E-05	0.00021526
ENSDARG00000076296	triqk	697.954284	-0.465693081	0.11182041	-4.16465202	3.12E-05	0.00021526
ENSDARG00000079296	gcga	42.6789609	1.670859123	0.40120276	4.16462516	3.12E-05	0.00021526
ENSDARG00000024325	cert1a	812.6982	0.483463212	0.11610706	4.16394336	3.13E-05	0.00021584
ENSDARG00000023797	ryr1b	9430.9182	0.708810357	0.17023213	4.16378718	3.13E-05	0.00021593
ENSDARG00000031336	hsd20b2	499.939218	0.890166193	0.21386472	4.16228633	3.15E-05	0.00021729
ENSDARG00000044400	wdr78	62.9950016	1.487757505	0.35745088	4.16213133	3.15E-05	0.00021738
ENSDARG00000052842	ppifb	1691.83016	0.505150924	0.12137263	4.16198398	3.15E-05	0.00021746
ENSDARG00000040224	krcp	1001.80131	-0.749913883	0.18019267	-4.16173348	3.16E-05	0.00021757
ENSDARG00000018787	efna1b	831.549056	-0.632035132	0.15187008	-4.16168303	3.16E-05	0.00021757
ENSDARG00000095351	CU914776.1	551.210815	1.052596067	0.25292006	4.16177368	3.16E-05	0.00021757
ENSDARG00000062964	si:dkey-246g23.2	88.5310077	1.300801642	0.31258344	4.16145414	3.16E-05	0.00021772
ENSDARG00000036848	slc43a2a	3531.00788	0.620131958	0.14904572	4.1606829	3.17E-05	0.0002184
ENSDARG00000032188	lrrc8aa	1312.85472	0.629208037	0.15125989	4.15978113	3.19E-05	0.0002192
ENSDARG00000100352	aglb	2599.96907	-0.566637597	0.13622351	-4.15961665	3.19E-05	0.0002193
ENSDARG00000035532	zgc:110329	597.700504	-0.516696162	0.12423441	-4.15904227	3.20E-05	0.00021973
ENSDARG00000030832	otofa	986.307498	-0.62297853	0.14978817	-4.15906361	3.20E-05	0.00021973
ENSDARG00000002764	miox	772.610281	0.595398951	0.14317554	4.15852424	3.20E-05	0.00022017
ENSDARG00000098771	cbx8b	370.661088	-0.645185797	0.15516651	-4.1580221	3.21E-05	0.00022053
ENSDARG00000101704	lrrc24	387.47842	-1.006549778	0.24207312	-4.15804032	3.21E-05	0.00022053
ENSDARG00000004358	gnb3a	3659.8299	-1.686456712	0.4056442	-4.15747771	3.22E-05	0.00022094
ENSDARG00000017843	srsf1b	2910.17232	0.387433003	0.09318805	4.15753968	3.22E-05	0.00022094
ENSDARG00000103523	nudt6	257.872782	0.715640631	0.17214967	4.15708392	3.22E-05	0.00022126
ENSDARG00000045442	cpb1	1733.88976	-2.450763908	0.58955375	-4.15698128	3.22E-05	0.00022129
ENSDARG00000040988	tpi1b	13729.1518	0.35298958	0.08494329	4.15559096	3.24E-05	0.00022258
ENSDARG00000074759	ccar1	2084.52561	-0.531951661	0.12803165	-4.15484497	3.26E-05	0.00022325
ENSDARG00000062350	synm	843.860426	0.866783599	0.20865286	4.15418987	3.26E-05	0.00022383
ENSDARG00000001447	mier1b	546.88475	-0.55362519	0.13329642	-4.15333891	3.28E-05	0.0002246
ENSDARG00000074471	vps39	1488.41697	0.435257764	0.10483876	4.15168735	3.30E-05	0.00022616
ENSDARG00000011884	MFN1	114.167064	-1.479393189	0.35636002	-4.1514006	3.30E-05	0.00022639
ENSDARG00000099385	lsp1b	1291.15686	0.627709389	0.15121033	4.15123347	3.31E-05	0.00022649
ENSDARG00000077836	ttc22	207.702503	0.797110963	0.19202904	4.15099172	3.31E-05	0.00022667
ENSDARG00000041078	chka	1197.98027	-0.688462922	0.16593374	-4.14902308	3.34E-05	0.00022856
ENSDARG00000061819	wscd2	316.190698	-0.856547769	0.20645892	-4.14875636	3.34E-05	0.00022876
ENSDARG00000088524	acot17	295.007399	0.688651308	0.16600961	4.14826172	3.35E-05	0.0002292
ENSDARG00000060917	anln	1634.36482	-0.853339286	0.20572408	-4.14797963	3.35E-05	0.00022941
ENSDARG00000034174	mrrf	484.376687	0.593018238	0.14302541	4.14624395	3.38E-05	0.0002311
ENSDARG00000061472	gba2	1112.18255	0.418956213	0.10105476	4.14583347	3.39E-05	0.00023145
ENSDARG00000011298	atf7a	725.341363	0.548333327	0.13227582	4.14537848	3.39E-05	0.00023184
ENSDARG00000070626	cacng8b	869.513196	-0.578960527	0.13968412	-4.14478411	3.40E-05	0.00023238
ENSDARG00000101023	msx2b	469.806811	0.729332244	0.1759784	4.14444192	3.41E-05	0.00023267
ENSDARG00000098528	rtn4rl1b	249.421918	-0.760425395	0.18352166	-4.14351842	3.42E-05	0.00023354
ENSDARG00000062148	SENP7	884.572894	-0.508495773	0.12277325	-4.14174739	3.45E-05	0.00023529
ENSDARG00000099870	tnni4a	119.775642	-0.879905751	0.21247229	-4.14127296	3.45E-05	0.00023571
ENSDARG00000013711	zgc:77486	500.62688	0.717866648	0.17335639	4.14098743	3.46E-05	0.00023594
ENSDARG00000098980	si:ch211-153f2.3	87.6163974	-1.20801759	0.29181255	-4.13970404	3.48E-05	0.0002372
ENSDARG00000099453	elof1	2305.83792	-0.555732838	0.13427056	-4.13890317	3.49E-05	0.00023796
ENSDARG00000037261	mtap	791.900764	0.522135562	0.12621332	4.13692912	3.52E-05	0.00023995
ENSDARG00000021984	ndufa2	1503.2599	0.48481917	0.11720075	4.13665601	3.52E-05	0.00024017
ENSDARG00000104372	gnb1b	10186.1524	-0.354375005	0.08567927	-4.13606482	3.53E-05	0.00024072
ENSDARG00000101300	ssna1	135.569289	-0.923861464	0.22338183	-4.13579506	3.54E-05	0.00024094
ENSDARG00000018691	phf2	1714.69732	-0.511340243	0.12364693	-4.13548671	3.54E-05	0.0002412
ENSDARG00000074708	armc3	114.598588	1.302146115	0.31508207	4.1327204	3.58E-05	0.00024405
ENSDARG00000011701	ctsll	43.3983659	2.183491558	0.52843301	4.13201203	3.60E-05	0.00024474
ENSDARG00000020887	armc1l	462.324615	-0.590092767	0.14281892	-4.13175488	3.60E-05	0.00024495

Table S2. DEGs of WT vs. *terfa-/-*

ENSDARG00000008494	myl6	1458.71009	0.45416097	0.10995827	4.13030314	3.62E-05	0.00024636
ENSDARG00000007329	tbx16	36.5076996	-1.638691623	0.39674441	-4.13034586	3.62E-05	0.00024636
ENSDARG00000043818	CASKIN2	588.434692	0.847045809	0.20510089	4.12989834	3.63E-05	0.00024673
ENSDARG00000077928	ccdc173	98.5923406	1.043648914	0.25275032	4.12916955	3.64E-05	0.00024745
ENSDARG00000016364	gna15.1	630.468689	0.620040893	0.15020552	4.12795006	3.66E-05	0.00024869
ENSDARG00000032373	rnf145b	1797.72929	-0.37006261	0.08965333	-4.12770608	3.66E-05	0.00024889
ENSDARG00000070122	stk11ip	986.133847	-0.51296373	0.12428264	-4.12739634	3.67E-05	0.00024912
ENSDARG00000075892	bag6	3736.69016	0.334211257	0.0809744	4.12736947	3.67E-05	0.00024912
ENSDARG00000078696	synpo2lb	1281.29032	0.451391968	0.10940261	4.12597093	3.69E-05	0.00025057
ENSDARG00000070465	si:dkey-13n23.3	38.5064438	-2.289112088	0.55489085	-4.12533764	3.70E-05	0.00025105
ENSDARG00000038643	alas2	2050.68265	0.794232812	0.19252511	4.12534672	3.70E-05	0.00025105
ENSDARG00000058416	si:dkey-11m19.5	39.5901522	1.871120265	0.4535654	4.1253594	3.70E-05	0.00025105
ENSDARG00000056745	necab2	3459.75091	-0.432939924	0.10495236	-4.12510892	3.71E-05	0.00025123
ENSDARG00000058528	cnot10	742.229815	-0.659489046	0.15988599	-4.12474578	3.71E-05	0.00025149
ENSDARG00000058557	il11b	11.1531782	4.434153228	1.07500171	4.12478715	3.71E-05	0.00025149
ENSDARG00000062019	rufy2	2122.8678	-0.386618993	0.09374174	-4.12429918	3.72E-05	0.00025191
ENSDARG00000000002	ccdc80	733.645222	0.513234317	0.12444657	4.12413378	3.72E-05	0.00025196
ENSDARG00000087822	agap3	1201.54606	0.703918759	0.1706812	4.12417277	3.72E-05	0.00025196
ENSDARG00000006878	phf21aa	1551.90356	-0.514905993	0.12485541	-4.12401821	3.72E-05	0.00025202
ENSDARG00000015385	ndufs3	2734.90559	0.42076805	0.1020323	4.12387107	3.73E-05	0.00025206
ENSDARG00000098911	zgc:153293	179.529342	-0.833181847	0.20203982	-4.12384966	3.73E-05	0.00025206
ENSDARG00000054690	si:dkey-166k12.1	235.971812	-0.741307592	0.1797895	-4.12319736	3.74E-05	0.00025271
ENSDARG00000017882	rdh1	312.066049	0.865471834	0.20992622	4.12274299	3.74E-05	0.00025308
ENSDARG00000090194	lamtor5	429.008629	0.55373255	0.13431204	4.1227321	3.74E-05	0.00025308
ENSDARG00000032103	mapk6	2944.04625	-0.59212924	0.14362888	-4.12263371	3.75E-05	0.00025312
ENSDARG00000070467	lactbl1b	226.146374	-1.337155499	0.32438532	-4.12212087	3.75E-05	0.00025362
ENSDARG00000071084	wipf1b	715.169846	0.532506673	0.12919492	4.12173067	3.76E-05	0.00025398
ENSDARG00000070894	abhd6b	80.9072797	-1.46575006	0.35568253	-4.12095041	3.77E-05	0.00025477
ENSDARG00000088929	ypel5	1107.09119	0.509918016	0.12376088	4.12018741	3.79E-05	0.00025555
ENSDARG00000113150	fthl31	17.2618642	5.719455117	1.38853629	4.11905339	3.80E-05	0.00025674
ENSDARG00000056633	fgf13b	808.731971	-0.523173972	0.12703204	-4.11844108	3.81E-05	0.00025735
ENSDARG00000004082	cx39.9	327.53204	0.823733299	0.20005334	4.11756825	3.83E-05	0.00025826
ENSDARG00000010130	smpdl3b	477.729381	0.663738316	0.16130793	4.11472849	3.88E-05	0.00026139
ENSDARG00000103163	tomm20a	1184.77607	-0.655379676	0.15929743	-4.11418871	3.89E-05	0.00026193
ENSDARG00000063309	tjp2a	2408.50277	0.448053529	0.10890727	4.11408294	3.89E-05	0.00026198
ENSDARG00000089795	sertad4	736.296896	-0.537809025	0.1307419	-4.11351695	3.90E-05	0.00026255
ENSDARG00000099257	si:ch73-119p20.1	1566.36974	-0.606213893	0.14737877	-4.11330557	3.90E-05	0.00026272
ENSDARG00000014675	efna3a	523.754488	-0.576900696	0.14026715	-4.11287091	3.91E-05	0.00026314
ENSDARG00000015978	COX5B	11873.6072	0.396786516	0.09649242	4.11210041	3.92E-05	0.00026395
ENSDARG00000041220	ftr53	39.8154751	1.464459581	0.3561624	4.1117748	3.93E-05	0.00026425
ENSDARG00000005115	calub	2166.9165	0.498277264	0.12120565	4.11100695	3.94E-05	0.00026506
ENSDARG00000094388	celsr2	90.6761395	-1.524193948	0.37089969	-4.10945059	3.97E-05	0.00026673
ENSDARG00000104235	myo5c	344.143307	0.750147235	0.18254297	4.10942823	3.97E-05	0.00026673
ENSDARG00000017708	lrrc40	434.469182	-0.533393075	0.12980146	-4.10929957	3.97E-05	0.00026681
ENSDARG00000101735	chn1	2004.90058	-0.823387066	0.20038373	-4.1090515	3.97E-05	0.00026703
ENSDARG00000106808	CU856622.1	84.873967	-1.259499554	0.30663082	-4.10754393	4.00E-05	0.0002687
ENSDARG00000004588	sox4a	7965.27115	-0.653228098	0.15909039	-4.10601861	4.03E-05	0.00027033
ENSDARG00000058200	plcxd2	22.7328724	-6.147621502	1.49720383	-4.1060685	4.02E-05	0.00027033
ENSDARG00000108939	FQ378016.1	127.399081	-1.495134546	0.36418371	-4.10544044	4.04E-05	0.00027094
ENSDARG00000074790	rab5if	2506.18437	0.463036874	0.11279001	4.10530057	4.04E-05	0.00027103
ENSDARG00000007769	sult5a1	36.4293181	1.75209914	0.42682111	4.10499645	4.04E-05	0.00027131
ENSDARG00000099283	epb41a	2230.2757	-0.775415082	0.18891868	-4.10449142	4.05E-05	0.00027183
ENSDARG00000093550	BX005438.1	41.6428656	-1.872058502	0.45620144	-4.10357871	4.07E-05	0.00027283
ENSDARG00000088581	f10	1934.20237	0.850519358	0.20739599	4.10094407	4.11E-05	0.00027588
ENSDARG00000074033	adamtsl2	199.391758	-0.91575641	0.22340598	-4.09906846	4.15E-05	0.00027805
ENSDARG00000067601	arfgap2	2903.14457	0.391980462	0.09562879	4.0989795	4.15E-05	0.00027809
ENSDARG00000092042	AL935153.1	343.86961	-0.875773764	0.21366859	-4.09874825	4.15E-05	0.00027829
ENSDARG00000111315	CT583642.2	32.1330405	1.778059412	0.43386325	4.09820236	4.16E-05	0.00027887

Table S2. DEGs of WT vs. *terfa-/-*

ENSDARG00000000729	daxx	945.660543	-0.553758705	0.13512484	-4.09812661	4.17E-05	0.00027889
ENSDARG00000075639	si:ch211-89o9.6	492.250883	-0.564161972	0.13767255	-4.0978537	4.17E-05	0.00027914
ENSDARG00000053468	adam10a	5324.64049	0.428372006	0.10455422	4.09712795	4.18E-05	0.00027985
ENSDARG00000114554	cdx1a	166.303751	0.942580916	0.23006158	4.097081	4.18E-05	0.00027985
ENSDARG00000075024	si:ch73-375g18.1	1833.98542	0.456172767	0.11134057	4.09709386	4.18E-05	0.00027985
ENSDARG00000040912	kdelr3	917.259465	0.675220279	0.1648156	4.09682274	4.19E-05	0.00028009
ENSDARG00000117211	CR936462.1	118.455166	1.001589925	0.24450905	4.09633073	4.20E-05	0.00028061
ENSDARG00000101369	CABZ01090890.1	2193.14171	0.512608897	0.12516655	4.09541446	4.21E-05	0.00028164
ENSDARG00000076899	slc2a13b	414.655296	0.617821416	0.1509163	4.09380183	4.24E-05	0.00028353
ENSDARG00000007764	klhdc10	937.56044	-0.498398143	0.1217542	-4.0934782	4.25E-05	0.00028378
ENSDARG00000013079	tubgcp2	855.062856	-0.486117704	0.11875287	-4.09352399	4.25E-05	0.00028378
ENSDARG00000078164	znf576.2	1415.06123	-0.467811784	0.11429106	-4.09316153	4.26E-05	0.00028403
ENSDARG00000114742	CABZ01072083.2	221.456144	-0.799922978	0.19542995	-4.09314425	4.26E-05	0.00028403
ENSDARG00000100166	si:dkeyp-9d4.3	2934.54681	-0.710378418	0.17358079	-4.09249444	4.27E-05	0.00028475
ENSDARG00000075914	kcnj14	48.1065637	-1.949987681	0.47662949	-4.0912023	4.29E-05	0.00028627
ENSDARG00000070109	ncapg	1879.09729	-0.750394391	0.18344653	-4.09053475	4.30E-05	0.00028702
ENSDARG00000096527	NA	7.73156053	-7.122290033	1.74126088	-4.09030612	4.31E-05	0.00028722
ENSDARG00000078647	samsn1b	132.619995	-1.560858202	0.3816434	-4.08983409	4.32E-05	0.00028773
ENSDARG00000029668	crim1	1433.78549	0.6923767	0.16932183	4.08911658	4.33E-05	0.00028854
ENSDARG00000100562	CABZ01080702.1	2767.90894	0.635823009	0.1555028	4.08882022	4.34E-05	0.00028884
ENSDARG00000036613	tab1	1251.47702	0.473223555	0.11573796	4.08874967	4.34E-05	0.00028885
ENSDARG00000060165	dnah7	186.53762	0.923508298	0.22587156	4.08864349	4.34E-05	0.0002889
ENSDARG00000108781	C17H14orf132	72.5540523	-1.593286214	0.38973874	-4.08808783	4.35E-05	0.00028952
ENSDARG00000011703	clocka	809.570837	-0.598274778	0.14635008	-4.08797019	4.35E-05	0.00028959
ENSDARG00000075975	slc45a3	58.7234186	1.455338265	0.35614157	4.08640383	4.38E-05	0.00029147
ENSDARG00000041830	tsn	701.967793	-0.600281882	0.14691345	-4.08595583	4.39E-05	0.00029195
ENSDARG00000055965	klc3	102.193823	-1.069251554	0.26170933	-4.08564555	4.40E-05	0.00029227
ENSDARG00000029305	baiap2l1a	456.740451	0.603634966	0.14777416	4.08484781	4.41E-05	0.00029319
ENSDARG00000105548	si:dkey-1h4.4	17.0449765	2.882680539	0.70604818	4.08283826	4.45E-05	0.00029566
ENSDARG00000105037	snrpf	1502.73407	-0.520804789	0.12756531	-4.08265222	4.45E-05	0.00029574
ENSDARG00000103991	sec14l1	595.409121	0.857126083	0.20994024	4.08271463	4.45E-05	0.00029574
ENSDARG00000094732	mical3b	964.010578	-0.626850267	0.15359238	-4.08125898	4.48E-05	0.00029744
ENSDARG00000077545	si:dkey-16p21.7	1734.30025	-0.561965229	0.13770647	-4.08089195	4.49E-05	0.00029783
ENSDARG00000089564	MDFI	733.632527	0.632889439	0.1551178	4.08005693	4.50E-05	0.00029882
ENSDARG00000109715	sncgb	5752.42846	-0.460682773	0.11295064	-4.07862025	4.53E-05	0.00030059
ENSDARG00000013576	gadd45bb	264.6339	0.913594172	0.22403168	4.07796859	4.54E-05	0.00030136
ENSDARG00000008811	cmtm3	171.596855	0.810734769	0.19882685	4.07759197	4.55E-05	0.00030177
ENSDARG00000015184	mpp3a	1020.24954	-0.453257873	0.11116174	-4.07746308	4.55E-05	0.0003018
ENSDARG00000071573	lsm5	183.306086	0.998164422	0.24480151	4.07744395	4.55E-05	0.0003018
ENSDARG00000055731	si:dkeyp-69e1.8	452.421135	0.512603905	0.12575559	4.07619183	4.58E-05	0.00030334
ENSDARG00000014962	ppp3cca	1533.48384	-0.414412102	0.10167402	-4.07588993	4.58E-05	0.0003035
ENSDARG00000098529	ccne2	688.48857	-0.907097434	0.22254772	-4.07596821	4.58E-05	0.0003035
ENSDARG00000045773	PYURF	84.927993	1.265072134	0.31037503	4.07594695	4.58E-05	0.0003035
ENSDARG00000052747	gpatch3	732.406125	-0.575900779	0.14129812	-4.07578517	4.59E-05	0.00030355
ENSDARG00000100181	lamp1a	1817.11093	0.789091188	0.19363504	4.07514675	4.60E-05	0.00030422
ENSDARG00000097827	lrp1aa	9642.77974	0.519304038	0.12743124	4.07517071	4.60E-05	0.00030422
ENSDARG00000026704	ftr72	13.9395984	3.047458557	0.74786209	4.07489374	4.60E-05	0.00030447
ENSDARG00000070084	phf10	903.381076	-0.556255847	0.13654223	-4.07387402	4.62E-05	0.00030573
ENSDARG00000100952	wu:fj16a03	1730.26073	0.618292068	0.15177486	4.07374505	4.63E-05	0.00030582
ENSDARG00000074776	tmem154	245.758012	0.993913424	0.24407865	4.07210317	4.66E-05	0.0003079
ENSDARG00000037794	nptx2a	77.5972635	-1.34388067	0.33005046	-4.0717431	4.67E-05	0.00030821
ENSDARG00000033234	stmn2a	6182.66049	-0.439471499	0.10793136	-4.07176858	4.67E-05	0.00030821
ENSDARG00000038789	tmem53	343.171768	-0.668049785	0.16408004	-4.07148714	4.67E-05	0.00030847
ENSDARG00000033327	unc5b	1142.52762	0.558689305	0.13726499	4.07015146	4.70E-05	0.00031016
ENSDARG00000026599	en2a	416.655026	-0.835081339	0.20520673	-4.06946372	4.71E-05	0.00031099
ENSDARG00000096994	AL929222.1	11.9210009	-4.097446913	1.00693608	-4.06922244	4.72E-05	0.00031123
ENSDARG00000076312	myot	1776.72935	0.587701402	0.14444201	4.06877072	4.73E-05	0.00031176
ENSDARG00000020798	necap1	1538.14236	-0.546891391	0.13445295	-4.0675298	4.75E-05	0.00031334

Table S2. DEGs of WT vs. *terfa-/-*

ENSDARG00000054458	slmapa	2946.46631	0.366559855	0.09012933	4.06704287	4.76E-05	0.00031391
ENSDARG00000099446	slit1b	2078.65131	0.569211376	0.13998122	4.06634117	4.78E-05	0.00031477
ENSDARG00000055253	slc12a10.3	110.661196	1.027775632	0.25277893	4.06590698	4.78E-05	0.00031527
ENSDARG00000057513	mdm4	2407.50785	-0.391372909	0.09627845	-4.06501037	4.80E-05	0.0003164
ENSDARG00000095863	afp4	12949.2094	0.500132052	0.12304636	4.06458224	4.81E-05	0.0003169
ENSDARG00000096508	BX322618.1	1865.93157	-1.585928817	0.39020141	-4.06438511	4.82E-05	0.00031706
ENSDARG00000102640	pdia3	7570.82642	0.362456576	0.08917968	4.06434038	4.82E-05	0.00031706
ENSDARG00000034930	cx52.6	29.2977624	-1.986543849	0.48886038	-4.06362209	4.83E-05	0.00031796
ENSDARG00000025348	igfbp5b	3478.85006	0.7495515	0.18449943	4.06262238	4.85E-05	0.00031915
ENSDARG00000077217	zgc:162184	163.212781	1.175923846	0.28944533	4.0626803	4.85E-05	0.00031915
ENSDARG00000015866	apoa2	128868.108	0.41453342	0.10203805	4.06253767	4.85E-05	0.00031918
ENSDARG00000032571	il20ra	302.034524	0.790269986	0.19453783	4.06229459	4.86E-05	0.00031943
ENSDARG00000068840	zgc:66024	27.2583878	1.952994594	0.48083231	4.06169586	4.87E-05	0.00032017
ENSDARG00000086100	cd302	548.386265	0.628497577	0.15475849	4.06115095	4.88E-05	0.00032083
ENSDARG00000069090	atp6v0d1	3814.85655	-0.388275939	0.09566767	-4.0585911	4.94E-05	0.00032428
ENSDARG00000095896	pou3f3b	2518.13595	-0.600274934	0.14791089	-4.05835521	4.94E-05	0.00032452
ENSDARG00000021208	serpind1	326.222252	0.789670576	0.19460905	4.05772801	4.96E-05	0.00032531
ENSDARG00000075564	fam13a	1276.65672	0.654524462	0.16131258	4.05749171	4.96E-05	0.00032555
ENSDARG00000041108	ctsh	412.425825	0.937896104	0.23122522	4.05620154	4.99E-05	0.00032727
ENSDARG00000017454	nup50	2955.42184	0.500271073	0.12334306	4.05593226	4.99E-05	0.00032756
ENSDARG00000018984	eya2	1607.97658	0.477419549	0.11772665	4.05532254	5.01E-05	0.00032833
ENSDARG00000026634	ehmt1b	3178.72029	-0.45818318	0.11299227	-4.05499582	5.01E-05	0.0003287
ENSDARG00000101437	fsbp	41.2698999	-1.754115487	0.43263705	-4.05447353	5.02E-05	0.00032935
ENSDARG00000076462	ndnfl	755.876656	-0.528522596	0.13040133	-4.05304595	5.06E-05	0.00033128
ENSDARG00000095328	si:ch211-191i18.4	14.0027222	-3.562013549	0.87893466	-4.05264885	5.06E-05	0.00033176
ENSDARG00000077364	mapk9	501.566992	0.666642591	0.16453517	4.0516723	5.09E-05	0.00033306
ENSDARG00000102129	crygn1	124.00884	-1.539219209	0.37990885	-4.05154866	5.09E-05	0.00033308
ENSDARG00000070589	mrpl35	646.288899	0.640758899	0.15815473	4.05146835	5.09E-05	0.00033308
ENSDARG00000077161	ccdc153	20.9411226	-2.837042502	0.70024108	-4.05152252	5.09E-05	0.00033308
ENSDARG00000074601	cbln2a	60.7248569	-1.638823421	0.40451345	-4.0513447	5.09E-05	0.00033317
ENSDARG00000093764	si:dkey-145c18.5	9.41764859	3.855407491	0.95173563	4.05092273	5.10E-05	0.00033369
ENSDARG00000070000	txnipb	725.025776	-0.782505151	0.19318141	-4.05062337	5.11E-05	0.00033403
ENSDARG00000077487	spsb3b	29.0965774	-2.235405486	0.55192507	-4.05019743	5.12E-05	0.00033455
ENSDARG00000039265	arhgap4a	83.0974286	1.135944501	0.2805391	4.04914863	5.14E-05	0.00033596
ENSDARG00000071872	zdhhc15b	621.441482	-0.56881759	0.14049809	-4.04857874	5.15E-05	0.00033669
ENSDARG00000088444	selenol	207.749528	1.00607144	0.2485656	4.0475088	5.18E-05	0.00033814
ENSDARG00000002732	spon2b	766.306617	-0.594317256	0.14686369	-4.04672711	5.19E-05	0.00033919
ENSDARG00000101377	ccdc142	761.482538	-0.501952873	0.12404238	-4.04662417	5.20E-05	0.00033925
ENSDARG00000094286	si:ch211-117m20.4	28.2863494	-3.046758014	0.7530189	-4.04605784	5.21E-05	0.00033994
ENSDARG00000068812	tlr7	13.658082	-3.334892773	0.8242404	-4.04601957	5.21E-05	0.00033994
ENSDARG00000013221	pde4ca	347.666809	0.671524439	0.16600952	4.04509589	5.23E-05	0.0003412
ENSDARG00000056653	fhl1b	450.272812	0.613475167	0.1516771	4.04461286	5.24E-05	0.00034181
ENSDARG00000077468	si:ch73-206p6.1	415.35126	-0.588460404	0.14550276	-4.04432479	5.25E-05	0.00034214
ENSDARG00000087863	cd44a	90.1123231	1.488194961	0.36799853	4.04402419	5.25E-05	0.00034249
ENSDARG00000027938	rad18	301.61856	-0.691175799	0.17095086	-4.0431255	5.27E-05	0.0003437
ENSDARG00000020133	jdp2b	257.738335	-0.941410084	0.23284496	-4.0430769	5.28E-05	0.0003437
ENSDARG00000011825	cetn3	526.303201	-0.605415889	0.14976627	-4.04240469	5.29E-05	0.0003446
ENSDARG00000111309	AL935126.2	31.9697401	2.548829262	0.63054635	4.04225524	5.29E-05	0.00034472
ENSDARG00000014943	kif23	1463.24772	-0.699148084	0.17300114	-4.04129181	5.32E-05	0.00034605
ENSDARG00000091144	CR749162.1	11.7208182	5.115022467	1.26573674	4.04114245	5.32E-05	0.00034618
ENSDARG00000043716	cldn5a	598.28718	0.641848439	0.15885124	4.04056312	5.33E-05	0.00034686
ENSDARG00000022509	cox4i2	726.649404	-0.652422655	0.16146745	-4.04058324	5.33E-05	0.00034686
ENSDARG00000086685	trim63b	1149.79403	0.840369853	0.2080531	4.03920861	5.36E-05	0.00034878
ENSDARG00000076218	b3galt4	93.896159	-1.120248158	0.27735706	-4.03901074	5.37E-05	0.00034898
ENSDARG00000041904	ankzf1	469.637189	0.531930672	0.13170655	4.03875637	5.37E-05	0.00034927
ENSDARG00000038185	gh1	178.35369	-1.051493714	0.26041498	-4.03776195	5.40E-05	0.00035062
ENSDARG00000102185	PCDH8	634.10153	-0.766150591	0.18974795	-4.03772784	5.40E-05	0.00035062
ENSDARG00000090035	rtn4r	436.186712	-0.74778654	0.18520379	-4.03764173	5.40E-05	0.00035065

Table S2. DEGs of WT vs. *terfa-/-*

ENSDARG00000074121	dnmbp	1806.99134	-0.444349759	0.11006989	-4.03697821	5.41E-05	0.00035156
ENSDARG00000101578	e2f4	1119.63778	-0.586972485	0.14541688	-4.03648108	5.43E-05	0.00035221
ENSDARG00000098679	nup85	670.913395	-0.592544819	0.14681293	-4.03605329	5.44E-05	0.00035276
ENSDARG00000023583	coq9	1779.39586	0.492895341	0.12215457	4.03501369	5.46E-05	0.00035423
ENSDARG00000070571	inavab	414.796484	0.664427292	0.16472823	4.03347557	5.50E-05	0.00035647
ENSDARG00000018159	ano10b	657.315578	0.575937157	0.14280215	4.03311264	5.50E-05	0.00035692
ENSDARG00000110810	si:dkey-3h2.2	21.1730048	2.751839221	0.68233018	4.03300237	5.51E-05	0.000357
ENSDARG00000075851	oafa	555.496103	0.534378817	0.13251974	4.03244691	5.52E-05	0.00035775
ENSDARG00000117653	CABZ01018269.1	52.2254687	1.660631147	0.41196769	4.03097421	5.55E-05	0.00035991
ENSDARG00000055093	cdh27	769.370653	0.693291557	0.17213445	4.02761659	5.63E-05	0.00036499
ENSDARG00000020279	efcab7	429.800785	-0.580252012	0.14407274	-4.02749353	5.64E-05	0.00036508
ENSDARG00000092878	si:dkeyp-85e10.3	14.4024853	-3.553773167	0.88248445	-4.02700939	5.65E-05	0.00036574
ENSDARG00000009839	fam117bb	502.974665	0.570942024	0.14178936	4.02669147	5.66E-05	0.00036605
ENSDARG00000042157	ppil6	20.4706423	2.092822803	0.51973024	4.02674818	5.66E-05	0.00036605
ENSDARG00000027355	slc25a4	28229.2203	0.564934404	0.1403041	4.0264996	5.66E-05	0.00036625
ENSDARG00000006206	pou4f3	193.216788	-1.076995267	0.26750637	-4.02605463	5.67E-05	0.00036685
ENSDARG00000036546	pdxkb	416.757371	0.778273558	0.19340705	4.02401847	5.72E-05	0.00036994
ENSDARG00000016154	zfp36l1a	4382.93684	0.417560429	0.10378198	4.02343858	5.74E-05	0.00037072
ENSDARG00000057000	camkvl	510.43333	-0.934661504	0.23230634	-4.02340073	5.74E-05	0.00037072
ENSDARG00000056985	tpte	1115.10553	0.629661315	0.15652198	4.02283002	5.75E-05	0.00037152
ENSDARG00000077384	dnah11	42.1668283	1.799038131	0.44727159	4.02224994	5.76E-05	0.00037234
ENSDARG00000026759	ldlrb	2717.79132	0.455605621	0.1132784	4.02199924	5.77E-05	0.00037264
ENSDARG00000036066	si:dkey-78a14.4	66.8309591	1.150557435	0.28608736	4.02169962	5.78E-05	0.00037302
ENSDARG00000027345	mpzl2b	836.705425	0.47634715	0.11844954	4.02151962	5.78E-05	0.00037314
ENSDARG00000070600	dyrk3	3064.92576	-0.322685101	0.08024002	-4.02149818	5.78E-05	0.00037314
ENSDARG00000069415	col17a1a	23084.1169	0.520284764	0.12938419	4.021239	5.79E-05	0.00037346
ENSDARG00000041839	tsc22d2	1846.05142	0.384309265	0.09559904	4.02001174	5.82E-05	0.00037531
ENSDARG00000053136	b2m	92.799858	1.104634289	0.27479835	4.01979959	5.82E-05	0.00037555
ENSDARG00000007560	sema3bl	1911.5434	0.503060664	0.12514871	4.01970316	5.83E-05	0.00037561
ENSDARG00000041051	mid1ip1a	1575.37271	0.479078082	0.11918754	4.0195317	5.83E-05	0.00037578
ENSDARG00000077818	nrg2a	336.579344	0.557529346	0.13871061	4.01937068	5.84E-05	0.00037594
ENSDARG00000109337	nanos1	3605.03336	-0.321365595	0.07995724	-4.01921805	5.84E-05	0.00037609
ENSDARG00000093964	si:ch211-250k18.6	15.1953598	2.90840587	0.72466447	4.01345172	5.98E-05	0.0003853
ENSDARG00000111290	ERG28	124.670062	-1.110944654	0.27683287	-4.01305187	5.99E-05	0.00038585
ENSDARG00000036943	golph3l	998.759554	0.485313257	0.12095872	4.01222207	6.01E-05	0.00038711
ENSDARG00000007412	slc2a1b	1485.79317	0.528386603	0.13170571	4.01187309	6.02E-05	0.00038758
ENSDARG00000075441	vwa2	1239.45248	0.546660436	0.13626673	4.0116941	6.03E-05	0.00038778
ENSDARG00000096784	CU929359.2	14.7698264	-3.944063813	0.98323896	-4.01129734	6.04E-05	0.00038833
ENSDARG00000098221	st8sia1	999.785079	-0.527737848	0.13160497	-4.01001449	6.07E-05	0.00039035
ENSDARG00000105489	loc564660	7.40024349	4.906986183	1.22392732	4.00921372	6.09E-05	0.00039147
ENSDARG00000077187	impg1a	314.914839	-2.425983881	0.60510005	-4.00922767	6.09E-05	0.00039147
ENSDARG00000014941	tprkb	31.2767054	1.7455982	0.43543484	4.00886208	6.10E-05	0.00039195
ENSDARG00000015343	pgd	2647.04777	0.434513288	0.10840161	4.0083658	6.11E-05	0.00039267
ENSDARG00000096233	si:dkey-260c8.6	54.8130475	1.475132422	0.36801993	4.00829494	6.12E-05	0.00039269
ENSDARG00000079468	rhobtb1	183.295067	1.008695901	0.25169448	4.00762032	6.13E-05	0.00039371
ENSDARG00000094653	si:dkey-149m13.5	43.6479458	1.716643142	0.42844929	4.00664251	6.16E-05	0.00039524
ENSDARG00000075487	si:ch211-267e7.3	889.968205	0.65774791	0.16417084	4.00648447	6.16E-05	0.0003954
ENSDARG00000003803	LHX3	367.384718	-0.831527492	0.20759527	-4.0055224	6.19E-05	0.00039691
ENSDARG00000070148	cygb2	312.411262	-0.665770039	0.16624665	-4.00471256	6.21E-05	0.00039817
ENSDARG00000024561	nolc1	2431.29733	0.397426388	0.09924659	4.00443375	6.22E-05	0.00039854
ENSDARG00000071009	kif20ba	715.004163	-0.714368684	0.17840418	-4.00421494	6.22E-05	0.00039881
ENSDARG00000079994	akap13	2270.99427	0.516302826	0.12896676	4.00337908	6.24E-05	0.00040012
ENSDARG00000099291	lsr	1568.74584	0.418415613	0.10451941	4.00323374	6.25E-05	0.00040026
ENSDARG00000076484	stab1	1383.93948	0.692635511	0.17302897	4.00300315	6.25E-05	0.00040055
ENSDARG00000092083	BX899181.1	17.4338497	2.76961686	0.69192981	4.00274252	6.26E-05	0.00040088
ENSDARG00000104758	zgc:194981	127.468469	0.89950062	0.22475124	4.00220545	6.28E-05	0.00040169
ENSDARG00000051923	ccnb1	1599.82725	-0.771356212	0.19274674	-4.00191583	6.28E-05	0.00040208
ENSDARG00000102746	tmem39a	1851.44924	0.592527052	0.14807358	4.00157162	6.29E-05	0.00040256

Table S2. DEGs of WT vs. *terfa-/-*

ENSDARG00000105304	CR762475.4	30.6416146	-2.069302026	0.517267	-4.00045245	6.32E-05	0.00040437
ENSDARG00000077559	LO017815.1	248.19066	0.695128797	0.17377838	4.00008788	6.33E-05	0.00040489
ENSDARG00000071463	zgc:154125	16.4808996	-3.321526285	0.83052344	-3.99931674	6.35E-05	0.0004061
ENSDARG00000058988	KCNJ15	319.040744	0.631051095	0.15780539	3.99891971	6.36E-05	0.00040668
ENSDARG00000045683	ccdc87	30.6429143	2.039807511	0.51013038	3.99860038	6.37E-05	0.00040713
ENSDARG00000023858	ccdc174	612.802769	-0.603066998	0.15082623	-3.99842255	6.38E-05	0.00040733
ENSDARG00000079274	prss59.1	1396.8448	-3.301938589	0.82623133	-3.99638513	6.43E-05	0.00041074
ENSDARG00000090637	myh6	566.285362	0.692707405	0.17335477	3.99589465	6.45E-05	0.00041148
ENSDARG00000097731	CR936482.1	62.1599205	-1.38194971	0.34584724	-3.99583848	6.45E-05	0.00041148
ENSDARG00000097752	si:rp71-77l1.2	15.2130315	2.665708325	0.66724827	3.99507717	6.47E-05	0.0004127
ENSDARG00000093622	spc24	367.195915	-0.840855366	0.21048685	-3.99481185	6.47E-05	0.00041305
ENSDARG00000058354	selenot1a	2197.17394	-0.387617052	0.09711549	-3.99130008	6.57E-05	0.00041911
ENSDARG00000099546	kynu	507.998749	0.658522623	0.16504711	3.98990696	6.61E-05	0.00042147
ENSDARG00000073952	slc4a7	866.76039	0.462356445	0.11589238	3.98953282	6.62E-05	0.00042203
ENSDARG00000102204	jph3	1074.40787	-0.612386827	0.15354481	-3.98832641	6.65E-05	0.00042407
ENSDARG00000018976	sptlc2a	742.583994	0.509441469	0.12774036	3.98810113	6.66E-05	0.00042429
ENSDARG00000052769	glrbb	1587.38994	-0.563754115	0.14135978	-3.98808004	6.66E-05	0.00042429
ENSDARG00000063341	czib	688.793085	0.497945813	0.12488298	3.98729916	6.68E-05	0.00042558
ENSDARG00000010758	capn12	2050.83514	0.644764369	0.161707	3.98723858	6.68E-05	0.00042558
ENSDARG00000059298	spcs1	17.94396	2.927720583	0.73433162	3.98691886	6.69E-05	0.00042605
ENSDARG00000023157	cbln10	282.710777	0.803692011	0.20167123	3.98515944	6.74E-05	0.00042911
ENSDARG00000115549	NCEH1	77.7244025	1.069718474	0.26844193	3.98491575	6.75E-05	0.00042944
ENSDARG00000056541	nr0b1	182.902441	-0.746976496	0.18749183	-3.98404827	6.78E-05	0.0004309
ENSDARG00000003684	obsl1a	978.048572	0.535470719	0.13440974	3.9838685	6.78E-05	0.00043097
ENSDARG00000011824	pbxip1b	1565.15458	-0.498295797	0.12507966	-3.98382757	6.78E-05	0.00043097
ENSDARG00000091574	suox	613.164232	0.480575709	0.12063071	3.98385879	6.78E-05	0.00043097
ENSDARG00000078489	si:ch211-284d12.3	10.4099053	3.461272255	0.86898907	3.98310218	6.80E-05	0.00043218
ENSDARG00000035398	enc1	2825.18634	0.395731461	0.0993596	3.98282049	6.81E-05	0.0004325
ENSDARG00000011740	u2af2b	4762.89226	-0.448153605	0.11252221	-3.98280145	6.81E-05	0.0004325
ENSDARG00000033440	metap1	3400.33605	0.364671491	0.09157356	3.98227912	6.83E-05	0.00043334
ENSDARG00000012432	fam76b	1928.69323	-0.507732668	0.12752104	-3.98155987	6.85E-05	0.00043455
ENSDARG00000091799	si:ch211-266k22.6	279.814004	-0.798489807	0.2006204	-3.98010282	6.89E-05	0.00043711
ENSDARG00000103740	fundc2	1180.3549	-0.502089244	0.12616605	-3.97959084	6.90E-05	0.00043794
ENSDARG00000061451	n4bp2	672.991124	-0.504398128	0.12675834	-3.97921073	6.91E-05	0.00043853
ENSDARG00000044655	st14b	1126.81229	0.482575558	0.12130581	3.97817341	6.94E-05	0.00044033
ENSDARG00000095190	BX901918.3	6.8832694	6.14013257	1.54371843	3.97749516	6.96E-05	0.00044148
ENSDARG00000108006	CU693481.1	204.03533	-0.949127859	0.23868619	-3.9764675	6.99E-05	0.00044327
ENSDARG00000078901	anks1ab	510.519599	-0.826683387	0.20792156	-3.97593882	7.01E-05	0.00044415
ENSDARG00000033046	ccni2	135.741483	-1.060177387	0.26672023	-3.97486684	7.04E-05	0.00044604
ENSDARG00000070597	prelp	542.745332	-0.772526483	0.19438026	-3.97430521	7.06E-05	0.00044698
ENSDARG00000014244	rbmx	3683.5283	-0.48752213	0.12267321	-3.97415322	7.06E-05	0.00044715
ENSDARG00000044769	kctd13	813.373148	-0.536461962	0.13503642	-3.97272068	7.11E-05	0.00044973
ENSDARG00000036180	ccnb2	190.39757	-0.931407053	0.23449309	-3.97200208	7.13E-05	0.00045098
ENSDARG00000052624	spryd7a	722.936365	-0.501191235	0.12618784	-3.97178719	7.13E-05	0.00045127
ENSDARG00000015355	fosl1a	372.107273	1.233076272	0.31050527	3.97119271	7.15E-05	0.00045228
ENSDARG00000093065	ubac2	320.521296	-0.60392623	0.15208317	-3.97102603	7.16E-05	0.00045249
ENSDARG00000044345	cyfip1	5717.80421	0.345518231	0.08701817	3.97064484	7.17E-05	0.00045309
ENSDARG00000099594	CU468012.3	16.2845693	3.20657951	0.80762771	3.97036838	7.18E-05	0.00045351
ENSDARG00000097485	cfap99	97.6035443	1.062658315	0.26771076	3.96942701	7.20E-05	0.00045518
ENSDARG00000076542	pogzb	2173.54823	-0.49982197	0.12593618	-3.96885125	7.22E-05	0.00045617
ENSDARG00000056909	LO018309.1	261.300799	0.842733707	0.21240379	3.96760208	7.26E-05	0.00045845
ENSDARG00000014588	adcy2b	759.62699	0.504713544	0.12722996	3.96693956	7.28E-05	0.00045951
ENSDARG00000041586	dhx40	696.934915	-0.608152181	0.15330544	-3.96693146	7.28E-05	0.00045951
ENSDARG00000053499	isl2b	792.581613	-0.65144879	0.16424962	-3.96621195	7.30E-05	0.00046066
ENSDARG00000040002	pnpla7b	1719.39734	0.429030752	0.10817063	3.96624083	7.30E-05	0.00046066
ENSDARG00000100718	me2	2488.21915	0.412687943	0.10405435	3.96608063	7.31E-05	0.0004608
ENSDARG00000077387	tcte1	30.1648526	2.039590267	0.51437994	3.96514348	7.34E-05	0.0004625
ENSDARG00000102252	USP34	2353.10308	0.539591248	0.13608673	3.96505404	7.34E-05	0.00046255

Table S2. DEGs of WT vs. *terfa-/-*

ENSDARG00000045305	pde7a	1814.58766	-0.422160948	0.10648632	-3.96446164	7.36E-05	0.00046347
ENSDARG00000097721	CU207301.3	34.0446998	2.130423478	0.53737839	3.96447553	7.36E-05	0.00046347
ENSDARG00000053990	hmgb2b	12410.8921	-0.845206038	0.21320937	-3.96420674	7.36E-05	0.00046385
ENSDARG00000101245	znf1063	14.6614496	2.596446	0.65498895	3.96410657	7.37E-05	0.00046392
ENSDARG00000101452	SUSD2	292.935707	0.844252902	0.21301632	3.96332504	7.39E-05	0.00046533
ENSDARG00000043640	cenpn	225.363789	-0.707589374	0.178553	-3.96290955	7.40E-05	0.00046602
ENSDARG00000027500	oxsr1b	1491.01263	0.446929642	0.11280548	3.96194979	7.43E-05	0.00046778
ENSDARG00000018426	aldh7a1	6159.4417	0.52173016	0.13169699	3.96159528	7.45E-05	0.00046836
ENSDARG00000033175	snrpe	1279.73463	-0.526192606	0.13283877	-3.96113741	7.46E-05	0.00046914
ENSDARG00000036593	kdm2ba	3981.86883	-0.48872334	0.1233976	-3.96055803	7.48E-05	0.00047016
ENSDARG00000090895	kansl1a	3352.41469	-0.409479381	0.1033931	-3.96041324	7.48E-05	0.00047032
ENSDARG00000034823	copg2	5778.04399	0.415537305	0.10495318	3.95926353	7.52E-05	0.00047247
ENSDARG00000031894	lef1	1297.08938	-0.551869079	0.13941111	-3.95857314	7.54E-05	0.00047372
ENSDARG00000037593	prickle2b	1364.50491	-0.385356125	0.09735712	-3.95817078	7.55E-05	0.0004744
ENSDARG00000040445	si:ch211-219a4.3	195.054959	0.945069824	0.23879673	3.95763296	7.57E-05	0.00047535
ENSDARG00000058208	slc25a26	648.339725	0.518750469	0.13111238	3.95653297	7.60E-05	0.00047742
ENSDARG00000020785	lama4	6694.07033	0.409489161	0.1035535	3.95437293	7.67E-05	0.00048163
ENSDARG00000062304	pdpk1a	345.323997	-0.623147318	0.15763242	-3.95316717	7.71E-05	0.00048395
ENSDARG00000102624	kif22	1316.52016	-0.666912199	0.16870675	-3.95308548	7.71E-05	0.00048399
ENSDARG00000035558	gps2	1172.05624	-0.442173307	0.11187031	-3.95255289	7.73E-05	0.00048494
ENSDARG00000078419	filip1a	120.975058	-1.053278286	0.26654028	-3.95166651	7.76E-05	0.00048662
ENSDARG00000098430	znf1078	29.9615246	-1.868890251	0.47294776	-3.95157864	7.76E-05	0.00048668
ENSDARG00000116400	CR388008.1	50.2574662	-1.751718047	0.44333002	-3.95127325	7.77E-05	0.00048718
ENSDARG00000044569	cldn19	640.826371	-0.731533674	0.18514351	-3.95117101	7.78E-05	0.00048726
ENSDARG00000096863	si:ch73-380l3.4	11.3133113	3.481680192	0.88140133	3.95016447	7.81E-05	0.00048919
ENSDARG00000056795	serpine1	300.976366	0.942574926	0.23867589	3.94918367	7.84E-05	0.00049095
ENSDARG00000068457	tnnt3b	136535.287	0.493675588	0.12500659	3.94919649	7.84E-05	0.00049095
ENSDARG00000075325	mrpl16	2305.99135	0.420371475	0.10644755	3.94909473	7.84E-05	0.00049101
ENSDARG00000091994	znf1048	44.4147136	-1.616405344	0.40934625	-3.94874842	7.86E-05	0.0004916
ENSDARG00000042993	prss1	4048.97545	-2.065226159	0.52305963	-3.948357	7.87E-05	0.00049228
ENSDARG00000060526	bmp3	621.365736	-0.571810846	0.14483309	-3.94806776	7.88E-05	0.00049275
ENSDARG00000054323	pparab	188.817642	1.127626766	0.28564228	3.94768865	7.89E-05	0.00049341
ENSDARG00000037390	gsg1l	98.293227	-1.153372527	0.29220628	-3.94711752	7.91E-05	0.00049446
ENSDARG00000016536	npas2	381.515702	-0.759029111	0.19234081	-3.94627189	7.94E-05	0.00049608
ENSDARG00000104826	pcdh1gc5	745.939886	-0.66040724	0.16739702	-3.94515526	7.97E-05	0.00049828
ENSDARG00000071017	nt5e	415.151048	-0.597456486	0.15146442	-3.94453343	8.00E-05	0.00049939
ENSDARG00000100482	pmt	443.186381	0.86708377	0.21982102	3.94449896	8.00E-05	0.00049939
ENSDARG00000033533	ccdc115	294.889205	-0.72123052	0.1828565	-3.94424331	8.01E-05	0.0004998
ENSDARG00000068050	agbl4	75.2363077	1.240759918	0.31466505	3.94311317	8.04E-05	0.00050204
ENSDARG00000099265	slc7a5	1819.74317	-0.571733588	0.14500819	-3.94276755	8.05E-05	0.00050263
ENSDARG00000009170	ctr9	2523.51197	-0.407420211	0.10335429	-3.94197671	8.08E-05	0.00050417
ENSDARG00000097794	CR626944.2	468.723631	0.772557491	0.19605455	3.94052318	8.13E-05	0.00050711
ENSDARG00000042840	eml1	1596.73259	-0.458438805	0.11638804	-3.93888251	8.19E-05	0.00051046
ENSDARG00000029612	gpkow	958.149416	-0.51489357	0.13076281	-3.93761486	8.23E-05	0.00051303
ENSDARG00000058259	celsr1b	1584.87528	-0.669657885	0.17007382	-3.93745437	8.24E-05	0.00051325
ENSDARG00000061904	fhod3b	486.313	-0.572112291	0.14534357	-3.9362751	8.28E-05	0.00051564
ENSDARG00000071353		8520.821	0.415298015	0.10550966	3.93611366	8.28E-05	0.00051573
ENSDARG00000056831	gng2	1653.08864	-0.621652147	0.15793345	-3.93616521	8.28E-05	0.00051573
ENSDARG00000024588	gpc5b	477.365995	0.540075979	0.13721511	3.93598027	8.29E-05	0.00051589
ENSDARG00000034933	chchd3b	834.469381	0.54871525	0.13946562	3.93441227	8.34E-05	0.00051914
ENSDARG00000091624	ugt2a7	102.630639	1.272929794	0.32355896	3.93415097	8.35E-05	0.00051957
ENSDARG00000061057	cyhr1	1556.67634	0.39697104	0.10093073	3.93310398	8.39E-05	0.00052171
ENSDARG00000069595	si:ch211-214c7.4	1569.04721	-0.525895621	0.13372121	-3.93277649	8.40E-05	0.00052229
ENSDARG00000098660	CU469526.1	638.698364	-0.552603251	0.14053141	-3.93224022	8.42E-05	0.00052333
ENSDARG00000097767	BX000438.1	14.3734212	-3.663307968	0.93183333	-3.93129098	8.45E-05	0.00052514
ENSDARG00000031698	mylk2	149.044331	0.971537255	0.24712641	3.93133727	8.45E-05	0.00052514
ENSDARG00000007387	irf2a	585.773586	-0.62620485	0.1593014	-3.93094386	8.46E-05	0.00052576
ENSDARG00000099679	tent2	791.765005	-0.539056875	0.13715256	-3.93034495	8.48E-05	0.00052694

Table S2. DEGs of WT vs. *terfa-/-*

ENSDARG00000107989	FAM163B	85.6633297	-1.520133749	0.38680597	-3.92996459	8.50E-05	0.00052765
ENSDARG00000070688	ncalda	1498.95673	-0.492704582	0.12538077	-3.92966613	8.51E-05	0.00052817
ENSDARG00000079570	rspo2	420.121985	0.538894599	0.13714482	3.92938367	8.52E-05	0.00052866
ENSDARG00000097265	CR847898.2	47.8074253	1.625697267	0.41378538	3.92884177	8.54E-05	0.00052972
ENSDARG00000034043	irx5a	1547.65478	-0.589857181	0.15015024	-3.92844647	8.55E-05	0.00053046
ENSDARG00000030038	C20H12orf57	1115.1879	-0.457386846	0.11643463	-3.92827172	8.56E-05	0.00053071
ENSDARG00000010878	cdkn1ca	827.554926	-0.787157327	0.20040389	-3.92785457	8.57E-05	0.0005315
ENSDARG00000026053	hltf	442.302678	-0.62982101	0.16035111	-3.92776211	8.57E-05	0.00053157
ENSDARG00000005315	celf1	3693.38211	-0.29929568	0.07621721	-3.92687787	8.61E-05	0.0005334
ENSDARG00000005372	camk4	341.104074	-0.666398538	0.16973303	-3.92615713	8.63E-05	0.00053486
ENSDARG00000073961	si:ch211-160j14.2	123.212635	-1.237578836	0.3152653	-3.92551559	8.65E-05	0.00053616
ENSDARG00000010279	scamp2	1153.28841	-0.397622118	0.10131915	-3.92445172	8.69E-05	0.0005384
ENSDARG00000062190	pde3b	951.461556	0.429584838	0.10947728	3.9239634	8.71E-05	0.00053936
ENSDARG00000003934	nxnl2	227.000327	-0.980589961	0.24992437	-3.9235468	8.73E-05	0.00054015
ENSDARG00000015654	ca15a	1229.57031	0.646025024	0.16466227	3.92333374	8.73E-05	0.0005405
ENSDARG00000060316	cish	1148.72243	-0.752404839	0.19182814	-3.922286	8.77E-05	0.00054272
ENSDARG00000109481	CABZ01092745.1	190.726375	-0.859358044	0.21910952	-3.922048	8.78E-05	0.00054299
ENSDARG00000035538	atp6v0a2a	856.861407	-0.746652586	0.19037196	-3.92207223	8.78E-05	0.00054299
ENSDARG00000017049	adsl	4077.7075	0.478959881	0.12212891	3.92175689	8.79E-05	0.00054351
ENSDARG00000103167	si:dkey-245n4.2	250.560348	-0.676017711	0.17238829	-3.92148276	8.80E-05	0.00054399
ENSDARG00000029114	si:dkey-40m6.8	3677.13982	-0.591313736	0.15083139	-3.92036262	8.84E-05	0.00054639
ENSDARG00000020602	grk7a	2320.97782	-2.548656823	0.65022025	-3.91968234	8.87E-05	0.0005478
ENSDARG00000076844	plin6	357.448274	0.872835805	0.2227008	3.91932045	8.88E-05	0.00054848
ENSDARG00000063145	tfam	839.145716	-0.521303293	0.13301784	-3.91904806	8.89E-05	0.00054897
ENSDARG00000036069	ddx39b	1218.01083	-0.403275515	0.10294233	-3.91748974	8.95E-05	0.00055225
ENSDARG00000061692	yjefn3	366.870014	-0.684442959	0.17471371	-3.91751146	8.95E-05	0.00055225
ENSDARG00000038153	lgals2b	4172.02752	0.768387779	0.19622737	3.91580329	9.01E-05	0.00055586
ENSDARG00000052522	snx11	696.023963	0.561350137	0.14335508	3.91580214	9.01E-05	0.00055586
ENSDARG00000105154	creg1	1218.76518	-0.432725968	0.11053486	-3.91483694	9.05E-05	0.00055794
ENSDARG00000040712	adprh	485.524715	0.717099605	0.18320785	3.91413134	9.07E-05	0.00055944
ENSDARG00000012866	picalma	2354.44289	0.324392569	0.08288934	3.91356181	9.09E-05	0.00056062
ENSDARG00000103361	tshz3b	2366.02408	-0.392019505	0.10021138	-3.91192611	9.16E-05	0.00056429
ENSDARG00000091633	si:dkey-177p2.18	92.9076416	-1.072989169	0.27430941	-3.91160175	9.17E-05	0.00056491
ENSDARG00000061149	nrn1lb	73.7171643	-1.439420194	0.3680289	-3.91116081	9.19E-05	0.0005658
ENSDARG00000060729	trim8b	2884.66518	-0.428620445	0.1095923	-3.91104538	9.19E-05	0.00056594
ENSDARG00000054818	rpl32	21449.1165	0.437469512	0.11188458	3.91000718	9.23E-05	0.00056823
ENSDARG00000096398	si:ch211-276a17.5	104.591658	-1.687947076	0.43180462	-3.90905282	9.27E-05	0.0005702
ENSDARG00000079123	ikbkb	464.708209	0.683733865	0.17490899	3.90908358	9.26E-05	0.0005702
ENSDARG00000059534	JPH3	1060.2888	-0.471243631	0.12056371	-3.90866887	9.28E-05	0.00057082
ENSDARG00000060246	slc16a6b	1613.67421	0.603417351	0.15437793	3.90870219	9.28E-05	0.00057082
ENSDARG00000009950	edf1	3615.93212	0.40274291	0.1030501	3.90822418	9.30E-05	0.00057173
ENSDARG00000101748	NA	6.03141389	-6.846884777	1.75205933	-3.90790693	9.31E-05	0.00057234
ENSDARG00000102421	acp7	248.118668	0.916898484	0.2346299	3.90785014	9.31E-05	0.00057234
ENSDARG00000099023	CABZ01034698.1	16.5872894	3.463212872	0.8862947	3.90751842	9.32E-05	0.00057298
ENSDARG00000074794	necab3	812.868425	-0.479077782	0.12261998	-3.90701256	9.34E-05	0.0005739
ENSDARG00000012458		5109.43455	0.331969201	0.08496699	3.90703723	9.34E-05	0.0005739
ENSDARG00000098074	trip4	327.347598	0.591787136	0.15148483	3.90657694	9.36E-05	0.00057479
ENSDARG00000058323	tmbim1a	673.436468	0.601918306	0.15409451	3.9061631	9.38E-05	0.00057545
ENSDARG00000006848	parp9	220.498158	-0.948338658	0.24278243	-3.90612554	9.38E-05	0.00057545
ENSDARG00000092039	si:dkey-70p6.1	1063.63309	-0.717688912	0.18373446	-3.90612037	9.38E-05	0.00057545
ENSDARG00000042440	p2rx7	46.1742571	2.234642032	0.57212795	3.90584312	9.39E-05	0.00057597
ENSDARG00000057644	adam8b	324.79521	1.116809455	0.2859614	3.90545532	9.40E-05	0.00057647
ENSDARG00000005220	pdcd6	1111.06314	0.436056991	0.11165306	3.90546398	9.40E-05	0.00057647
ENSDARG00000019137	tram1	1344.72756	0.561023603	0.14364797	3.90554505	9.40E-05	0.00057647
ENSDARG00000014569	hnrnpr	6915.96134	-0.704284137	0.18033919	-3.90533057	9.41E-05	0.00057662
ENSDARG00000008784	prpf4ba	1460.74119	-0.450057005	0.11533896	-3.90203788	9.54E-05	0.00058438
ENSDARG00000111785	trappc2l	371.922655	0.585701359	0.15012645	3.90138693	9.56E-05	0.00058581
ENSDARG00000071097	si:ch73-367f21.4	370.615769	-0.670237935	0.17180602	-3.90113184	9.57E-05	0.00058628

Table S2. DEGs of WT vs. *terfa-/-*

ENSDARG00000036722	slc9a3.2	139.441307	1.162114926	0.29790228	3.90099373	9.58E-05	0.00058633
ENSDARG00000100075	abcg2a	1312.97647	0.664830032	0.17042417	3.90103131	9.58E-05	0.00058633
ENSDARG00000052351	si:dkey-156n14.3	1370.67401	-0.4661629	0.11950061	-3.90092487	9.58E-05	0.00058635
ENSDARG00000027491	uros	472.777684	-0.580272383	0.14875712	-3.90080416	9.59E-05	0.0005865
ENSDARG00000000887	zgc:56699	187.509936	0.918494266	0.23550117	3.90016864	9.61E-05	0.00058775
ENSDARG00000038207	aldh4a1	2233.46084	0.450519316	0.11551185	3.90019993	9.61E-05	0.00058775
ENSDARG00000038141	atf4b	6725.32786	-0.389643902	0.09994339	-3.89864604	9.67E-05	0.00059131
ENSDARG00000003631	clockb	1876.31401	-0.493712648	0.12664859	-3.89828771	9.69E-05	0.00059204
ENSDARG00000016447	ythdf1	2870.05336	0.318978726	0.08182668	3.89822409	9.69E-05	0.00059205
ENSDARG00000004621	gpm6ab	9253.36609	-0.478604149	0.12278762	-3.89782086	9.71E-05	0.00059289
ENSDARG00000056964	ilk	3363.98465	0.356930718	0.09163949	3.89494438	9.82E-05	0.00059983
ENSDARG00000104301	zgc:193726	57.5336452	-1.675928277	0.43042272	-3.89367985	9.87E-05	0.00060267
ENSDARG00000015460	racgap1	987.003118	-0.758392136	0.19477232	-3.89373677	9.87E-05	0.00060267
ENSDARG00000024026	sdf2	718.879392	0.49717656	0.12769381	3.89350555	9.88E-05	0.00060295
ENSDARG00000044325	zgc:193690	100.523237	-1.141763587	0.29328706	-3.89299003	9.90E-05	0.00060409
ENSDARG00000097494	CR382327.2	85.1425202	0.986086182	0.25331249	3.89276575	9.91E-05	0.0006045
ENSDARG00000063313	plbd1	485.294741	0.608924054	0.15644	3.89238089	9.93E-05	0.00060531
ENSDARG00000086216	prr13	1372.63256	0.543317296	0.13958777	3.89229866	9.93E-05	0.00060537
ENSDARG00000035852	cart3	143.554495	1.29204077	0.33198093	3.89191263	9.95E-05	0.00060618
ENSDARG00000004954	grna	177.702836	1.193401716	0.30678765	3.88999272	0.00010025	0.00061085
ENSDARG00000117432	BX927219.1	25.5215067	1.804008294	0.46387317	3.88901195	0.00010065	0.00061317
ENSDARG00000104937	PTPRD	8671.72319	-0.615555717	0.15833744	-3.8876196	0.00010123	0.00061655
ENSDARG00000035868	spire1a	864.576283	-0.483437567	0.12439126	-3.88642724	0.00010173	0.00061943
ENSDARG00000074447	dapk3	1141.41142	0.44543304	0.11463457	3.88567807	0.00010205	0.00062089
ENSDARG00000030656	3-Sep	1696.15921	-0.539280326	0.13878504	-3.88572376	0.00010203	0.00062089
ENSDARG00000074626	pidd1	228.922385	0.757513218	0.19494446	3.88578992	0.000102	0.00062089
ENSDARG00000112889	rab38b	93.5521958	-1.253563526	0.3226331	-3.88541514	0.00010216	0.00062141
ENSDARG00000011583	cry1b	620.113767	0.559045533	0.14390652	3.88478249	0.00010242	0.00062287
ENSDARG00000069552	atoh7	85.8403385	-1.809813001	0.46589837	-3.88456605	0.00010251	0.00062328
ENSDARG00000004447	aplnr2	140.273508	1.093681593	0.28157314	3.88418291	0.00010268	0.0006238
ENSDARG00000012632	zgc:92749	113.99703	1.086665793	0.27976673	3.88418522	0.00010267	0.0006238
ENSDARG00000075513	ccdc136b	357.438447	-1.006522247	0.259126	-3.88429656	0.00010263	0.0006238
ENSDARG00000046019	snai1b	514.374669	0.640050726	0.16478776	3.88409151	0.00010271	0.00062389
ENSDARG00000059128	uqcrh	4239.86799	0.40506943	0.10430815	3.88339207	0.00010301	0.0006255
ENSDARG00000005141	camkvb	740.042591	-0.638484952	0.16441621	-3.88334561	0.00010303	0.0006255
ENSDARG00000044982	dhrs3a	572.789778	1.069176138	0.275374	3.88263279	0.00010333	0.00062718
ENSDARG00000009262	FAR2	567.426774	0.674192655	0.17364905	3.88250122	0.00010339	0.00062737
ENSDARG00000002847	fndc1	241.855513	-1.037963175	0.26738372	-3.88192366	0.00010363	0.00062871
ENSDARG00000035577	cds2	2219.42035	-0.377119469	0.09715803	-3.88150584	0.00010381	0.00062948
ENSDARG00000069843	kctd12.1	248.533201	-0.712058192	0.18344665	-3.88155453	0.00010379	0.00062948
ENSDARG00000075964	plekhg4	4883.27642	0.390659909	0.10066306	3.88086661	0.00010409	0.00063098
ENSDARG00000024619	foxo6b	776.90957	-0.777162365	0.20026622	-3.88064629	0.00010418	0.0006314
ENSDARG00000018485	cyp2v1	699.953742	0.573818198	0.14786963	3.88056829	0.00010421	0.0006314
ENSDARG00000022183	gsto1	57.709833	1.467082645	0.37806268	3.88052752	0.00010423	0.0006314
ENSDARG00000040946	olig2	412.254223	-1.153271521	0.29720695	-3.88036525	0.0001043	0.00063167
ENSDARG00000096544	CR774189.1	5.37131613	5.939029021	1.5308276	3.87961976	0.00010462	0.00063345
ENSDARG00000105367	si:dkey-88n24.11	8.39255193	-7.068320487	1.82224973	-3.8788978	0.00010493	0.00063518
ENSDARG00000076571	sp5a	355.361034	-0.943084872	0.24317765	-3.87817243	0.00010524	0.00063692
ENSDARG00000040314	psph	2374.17552	0.524462213	0.13526897	3.87718047	0.00010567	0.00063937
ENSDARG00000062590	pleca	12430.6972	0.587796081	0.15160831	3.87707039	0.00010572	0.0006395
ENSDARG00000068096	atf5a	1406.39562	-0.392046042	0.10113567	-3.87643708	0.000106	0.00064101
ENSDARG00000104069	rnf170	1707.19722	0.487325667	0.12572598	3.87609377	0.00010615	0.00064176
ENSDARG00000068918	map2k2b	653.092769	0.637269135	0.16442422	3.87576203	0.00010629	0.00064248
ENSDARG00000014196	myl1	39725.847	0.39060581	0.10079178	3.87537385	0.00010646	0.00064335
ENSDARG00000032496	pon1	467.099014	0.632879436	0.16331835	3.87512765	0.00010657	0.00064384
ENSDARG00000091962	RUNDC1	249.278854	-0.682876808	0.17624178	-3.87465905	0.00010677	0.00064493
ENSDARG00000063624	gfm1	2216.68647	0.429353104	0.11085124	3.87323666	0.0001074	0.00064855
ENSDARG00000074850	il12rb2l	365.584873	0.781668736	0.20181985	3.87310137	0.00010746	0.00064859

Table S2. DEGs of WT vs. *terfa-/-*

ENSDARG00000100875	GAN	405.389788	0.61512926	0.15881887	3.87314976	0.00010744	0.00064859
ENSDARG00000103200	gpr107	1122.86441	-0.491324341	0.1268897	-3.87205863	0.00010792	0.00065106
ENSDARG00000041602	zgc:92335	680.429793	0.521396035	0.13465414	3.87211286	0.0001079	0.00065106
ENSDARG00000038569	fzd8b	134.547345	-1.017779511	0.26292378	-3.87100601	0.00010839	0.00065372
ENSDARG00000095184	si:ch73-52p7.1	46.4665627	1.445741722	0.37351879	3.87059971	0.00010857	0.00065465
ENSDARG00000054418	ssuh2.2	21.290628	2.204048454	0.56945588	3.87044638	0.00010864	0.0006549
ENSDARG00000029445	eif1b	2596.56707	-0.361292925	0.09336055	-3.869867	0.0001089	0.0006563
ENSDARG00000054823	id3	1515.39518	-0.425603185	0.10998386	-3.86968755	0.00010898	0.00065662
ENSDARG00000103860	wu:fu71h07	88.6540483	-0.994306434	0.25696247	-3.86946164	0.00010908	0.00065707
ENSDARG00000101431	gas8	330.271965	-0.69287944	0.17912069	-3.86822669	0.00010963	0.00066025
ENSDARG00000003749	polb	776.22151	-0.593653705	0.1534779	-3.86800764	0.00010973	0.00066068
ENSDARG00000042835	tfdp2	1425.66636	-0.458000955	0.11841314	-3.86782216	0.00010981	0.00066102
ENSDARG00000074761	bicdl1	267.77133	-1.087034097	0.28107574	-3.86740628	0.00011	0.00066199
ENSDARG00000055252	snap23.2	122.61736	1.791237589	0.46317891	3.86726932	0.00011006	0.0006622
ENSDARG00000056059	rangap1b	942.810745	-0.622112355	0.16089649	-3.86653778	0.00011039	0.00066403
ENSDARG00000010905	mettl13	425.229202	0.545339971	0.1410589	3.8660444	0.00011062	0.00066521
ENSDARG00000008020	med31	480.095064	-0.580176672	0.15011151	-3.86497123	0.0001111	0.00066798
ENSDARG00000105296	AP3B2	2021.66166	-0.622740336	0.16115006	-3.86435063	0.00011139	0.00066952
ENSDARG00000086838	itga2.3	70.3129188	1.639718986	0.42446334	3.86304033	0.00011199	0.00067296
ENSDARG00000043646	slc6a8	1841.57944	0.938963608	0.24307032	3.86292995	0.00011204	0.00067311
ENSDARG00000116692	si:ch211-79g12.1	17.812147	2.867213413	0.74228309	3.8626953	0.00011214	0.00067354
ENSDARG00000034420	irx6a	188.653037	-1.030786697	0.26686382	-3.86259439	0.00011219	0.00067354
ENSDARG00000059925	usp24	2905.31645	0.521589127	0.13503558	3.86260528	0.00011218	0.00067354
ENSDARG00000029952	ampd2b	1084.5844	-0.469563856	0.12157161	-3.86244673	0.00011226	0.00067379
ENSDARG00000020708	mdkb	6808.81995	-0.577271617	0.14946186	-3.86233407	0.00011231	0.00067393
ENSDARG00000062221	arap1a	510.074104	0.700870302	0.18146896	3.86220497	0.00011237	0.00067413
ENSDARG00000033738	zgc:153867	9058.76384	0.336833171	0.08722917	3.86147408	0.00011271	0.00067586
ENSDARG00000104550	clcn2b	817.158253	0.702256595	0.18186286	3.86146231	0.00011271	0.00067586
ENSDARG00000086484	fbxw10	15.476757	2.431721966	0.62991254	3.86041203	0.0001132	0.0006786
ENSDARG00000061989	mpnd	726.085856	-0.679400545	0.17604649	-3.85921091	0.00011375	0.00068178
ENSDARG00000079438	pkp3b	1202.32802	0.569592557	0.14761667	3.85859251	0.00011404	0.00068334
ENSDARG00000103736	gramd2aa	220.806015	-0.771115786	0.19985968	-3.85828582	0.00011419	0.00068387
ENSDARG00000022614	ddx43	361.583894	-1.127166648	0.29214138	-3.85829165	0.00011418	0.00068387
ENSDARG00000032098	c8g	497.036822	-0.840485079	0.21786878	-3.85775824	0.00011443	0.00068519
ENSDARG00000079651	si:ch1073-174d20.	2158.85356	-0.353445296	0.09163069	-3.85728085	0.00011466	0.00068636
ENSDARG00000071107	wnt7bb	241.36053	0.77371572	0.20061313	3.85675514	0.0001149	0.00068767
ENSDARG00000010296	kcnh6b	32.5205158	-2.089301018	0.54178955	-3.85629628	0.00011512	0.0006888
ENSDARG00000070625	enpp5	739.137808	-0.58221487	0.15098445	-3.85612464	0.0001152	0.00068911
ENSDARG00000028201	commd8	524.901627	0.583782497	0.15139987	3.8558983	0.00011531	0.00068959
ENSDARG00000055255	pitpnm3	750.097438	0.54385246	0.14107258	3.85512536	0.00011567	0.0006916
ENSDARG00000098049	BX248121.1	463.856433	-0.74764069	0.19395235	-3.85476477	0.00011584	0.00069246
ENSDARG00000097573	si:ch211-107e6.5	5.03784935	5.706300922	1.48043533	3.85447496	0.00011598	0.00069311
ENSDARG00000004823	asz1	32.8929745	1.880403445	0.487882	3.85421769	0.0001161	0.00069367
ENSDARG00000054833	nucb1	1944.41852	0.315599613	0.08189305	3.85380206	0.0001163	0.00069468
ENSDARG00000054352	lrrc9	46.4220713	1.29349232	0.33568467	3.85329581	0.00011654	0.00069596
ENSDARG00000075380	kri1	806.99104	-0.574465945	0.14912074	-3.8523544	0.00011699	0.00069847
ENSDARG00000102018	btr25	11.7278983	2.709826315	0.7034625	3.85212619	0.0001171	0.00069895
ENSDARG00000040290	pon3.2	429.570058	0.590588121	0.15333503	3.85161893	0.00011734	0.00069997
ENSDARG00000093745	htr1aa	181.474699	-0.820914507	0.21313627	-3.85159464	0.00011735	0.00069997
ENSDARG00000086173	relb	643.205771	0.628778589	0.16324798	3.85167753	0.00011731	0.00069997
ENSDARG00000052697	fam172a	643.742397	0.554789128	0.14404832	3.85140997	0.00011744	0.00070033
ENSDARG00000088820	zgc:174288	338.250394	0.623066389	0.16178752	3.85114006	0.00011757	0.00070093
ENSDARG00000090797	fam222a	848.918577	-0.492842514	0.12799127	-3.85059467	0.00011783	0.00070233
ENSDARG00000076988	znf839	1460.8695	-0.435196762	0.11303186	-3.85021335	0.00011802	0.00070325
ENSDARG00000099084	pcif1	663.330387	-0.599772911	0.15578205	-3.85007722	0.00011808	0.00070347
ENSDARG00000010149	atp5fa1	72549.111	0.420070087	0.10911879	3.84965854	0.00011828	0.00070451
ENSDARG00000113254	COX5B	172.339025	-0.855805467	0.22233392	-3.84918988	0.00011851	0.00070552
ENSDARG00000113941	CU182823.3	11.742389	3.908967859	1.01551532	3.84924558	0.00011848	0.00070552

Table S2. DEGs of WT vs. *terfa-/-*

ENSDARG00000101756	im:7141269	137.903756	1.044551616	0.27140533	3.84867766	0.00011876	0.00070683
ENSDARG00000086551	irs4a	904.753301	-0.553127569	0.14373026	-3.84837233	0.00011891	0.00070754
ENSDARG00000005343	ccdc85al	520.409042	-0.576577179	0.14987162	-3.84714061	0.0001195	0.00071093
ENSDARG00000060796	slc20a2	2879.09252	0.442476013	0.11501766	3.84702673	0.00011956	0.00071109
ENSDARG00000029944	parpbp	248.448058	-0.871418393	0.2265643	-3.84622994	0.00011995	0.00071324
ENSDARG00000104217	gpc2	758.251407	-0.773171048	0.20103756	-3.84590339	0.00012011	0.00071402
ENSDARG00000102583	il4r.1	228.023741	1.079573934	0.28073915	3.84546985	0.00012032	0.00071494
ENSDARG00000026236	zgc:56585	167.783859	1.015111684	0.26397313	3.84551144	0.0001203	0.00071494
ENSDARG00000040184	syncrip	13826.2296	-0.493390106	0.12832261	-3.84491947	0.00012059	0.0007162
ENSDARG00000098959	CABZ01113771.1	574.850769	0.480546291	0.12498058	3.84496772	0.00012057	0.0007162
ENSDARG00000088891	slc23a3	139.454248	1.111890105	0.28920624	3.84462697	0.00012074	0.00071689
ENSDARG00000015686	bmp6	255.459135	0.611134058	0.15896224	3.84452345	0.00012079	0.00071702
ENSDARG00000021595	lhfpl3	1424.37467	-0.494969837	0.12874901	-3.84445543	0.00012082	0.00071705
ENSDARG00000020250	rhoj	377.203533	0.673625882	0.17522705	3.84430305	0.0001209	0.00071732
ENSDARG00000035650	evla	1547.42259	-0.486544731	0.12659048	-3.84345425	0.00012132	0.00071964
ENSDARG00000073957	tox3	1304.93103	-0.494664576	0.12870931	-3.84326969	0.00012141	0.00072001
ENSDARG00000074995	eif2b5	1721.65447	0.413237539	0.10753808	3.84270887	0.00012168	0.00072148
ENSDARG00000087393	prr11	153.464336	-1.194519191	0.31087683	-3.8424195	0.00012183	0.00072216
ENSDARG00000036239	gatm	37883.9888	0.473194772	0.12319878	3.84090472	0.00012258	0.00072646
ENSDARG00000052512	tsga10	74.530935	-1.240896861	0.32308775	-3.8407425	0.00012266	0.00072677
ENSDARG00000070929	sox14	667.926395	-0.815450576	0.21236346	-3.83988185	0.00012309	0.00072903
ENSDARG00000098395	CU929451.2	55.5122787	-1.616860202	0.42107242	-3.83986252	0.0001231	0.00072903
ENSDARG00000026842	acin1b	5690.89941	-0.294084629	0.07659311	-3.83957012	0.00012325	0.00072931
ENSDARG00000101446	si:dkey-46i9.1	7096.41374	-0.417968916	0.10885834	-3.83956737	0.00012325	0.00072931
ENSDARG00000023578	lpp	1133.84875	0.477155364	0.12427366	3.83955337	0.00012326	0.00072931
ENSDARG00000098158	cpxm1b	539.14603	0.607264594	0.15816105	3.83953314	0.00012327	0.00072931
ENSDARG00000007863	tra2a	2860.98629	-0.367280369	0.09567126	-3.83898315	0.00012355	0.00073077
ENSDARG00000060656	si:ch211-10a23.2	610.802749	-0.603162359	0.15711793	-3.83891486	0.00012358	0.0007308
ENSDARG00000026500	xkr9	54.7378764	1.594904014	0.41553614	3.8381836	0.00012395	0.00073281
ENSDARG00000016460	fut9a	714.797153	-0.556816577	0.14508397	-3.83789167	0.0001241	0.0007335
ENSDARG00000037488	canx	15044.676	0.323282904	0.08424674	3.83733418	0.00012438	0.000735
ENSDARG00000029859	armh1	53.4612888	1.491304981	0.38864701	3.83717085	0.00012446	0.00073531
ENSDARG00000076532	si:ch211-222l21.1	30001.3253	-0.605765214	0.15788532	-3.83674183	0.00012468	0.00073642
ENSDARG00000032970	cox4i1	9526.31288	0.396721242	0.10341744	3.83611533	0.000125	0.00073813
ENSDARG00000015472	gpc4	5046.74632	0.344561405	0.08983322	3.83556798	0.00012527	0.0007396
ENSDARG00000071024	zgc:171679	559.032886	0.852194332	0.22226782	3.83408782	0.00012603	0.00074389
ENSDARG00000041869	tnfrsf19	1275.18253	0.469118276	0.12240989	3.83235599	0.00012692	0.00074897
ENSDARG00000086204	gsg1l2a	36.8523551	-1.966015394	0.51306432	-3.83190826	0.00012715	0.00075015
ENSDARG00000104593	cyp2k18	89.5481951	-1.195300476	0.31194121	-3.83181325	0.0001272	0.00075026
ENSDARG00000037968	fus	6318.03816	-0.501324784	0.13085962	-3.83101218	0.00012762	0.00075253
ENSDARG00000079104	mfhas1	802.015753	-0.449607398	0.11737253	-3.83060166	0.00012783	0.00075361
ENSDARG00000093844	zgc:136461	278.760792	-4.204895153	1.0977609	-3.83042899	0.00012792	0.00075396
ENSDARG00000070717	slc25a18	722.595197	-0.470131652	0.12274089	-3.83027724	0.000128	0.00075425
ENSDARG00000095509	CR759885.1	63.5832982	-1.276007421	0.33314242	-3.83021594	0.00012803	0.00075426
ENSDARG00000037064	galca	811.476536	0.683142358	0.17838438	3.82960866	0.00012835	0.00075594
ENSDARG00000056374	noxo1b	67.1329486	1.476210795	0.38551046	3.82923673	0.00012854	0.00075691
ENSDARG00000090064	CABZ01084273.1	439.832879	-0.693180037	0.18104023	-3.82887303	0.00012873	0.00075749
ENSDARG00000088130	si:dkey-30c15.10	832.884659	-0.504622932	0.13179154	-3.82894796	0.00012869	0.00075749
ENSDARG00000090039	reck	6212.50457	0.375159215	0.09798114	3.82889198	0.00012872	0.00075749
ENSDARG00000030349	cryba2a	2722.25543	-0.865079332	0.22602811	-3.82730868	0.00012955	0.00076213
ENSDARG00000041947	styk1b	627.336526	0.545168348	0.14246302	3.82673579	0.00012985	0.00076373
ENSDARG00000077300	usp42	1151.53761	-0.472137483	0.12339267	-3.82630089	0.00013008	0.0007649
ENSDARG00000075539	snphb	481.143105	-0.699884657	0.18292633	-3.8260466	0.00013022	0.00076551
ENSDARG00000097807	BX530017.2	41.1763523	2.317851245	0.60582668	3.82593129	0.00013028	0.00076568
ENSDARG00000061741	itpr3	1503.59492	0.708347606	0.18516527	3.82548857	0.00013051	0.00076688
ENSDARG00000016968	tmem214	2814.55939	0.343217809	0.08972171	3.82535955	0.00013058	0.0007671
ENSDARG00000076620	abhd8b	130.174357	-0.97702399	0.25544907	-3.82473108	0.00013092	0.00076888
ENSDARG00000023299	snu13b	1308.03954	-0.409214891	0.10700405	-3.82429342	0.00013115	0.00077006

Table S2. DEGs of WT vs. *terfa-/-*

ENSDARG00000111474	cfap100	78.1238989	1.346209254	0.35209722	3.82340211	0.00013162	0.00077259
ENSDARG00000061124	srpra	2716.22878	0.354762889	0.092788	3.82337057	0.00013164	0.00077259
ENSDARG00000078592	nomo	4526.30024	-0.353145092	0.09238116	-3.82269591	0.000132	0.00077452
ENSDARG00000077499	plekho1b	790.571103	-0.461555594	0.12074417	-3.8225913	0.00013206	0.00077467
ENSDARG00000068562	rbm33a	3704.96516	-0.4045442	0.10584151	-3.82216944	0.00013228	0.00077549
ENSDARG00000077881	chl1a	1075.58867	-0.462001377	0.12087459	-3.82215469	0.00013229	0.00077549
ENSDARG00000043976	etf1b	8185.51454	0.322487797	0.08437087	3.8222646	0.00013223	0.00077549
ENSDARG00000073988	mntb	1018.46967	-0.509345721	0.13331785	-3.82053655	0.00013316	0.00078041
ENSDARG00000030092	tram2	658.231304	0.500301473	0.13095481	3.82041317	0.00013323	0.00078062
ENSDARG00000074696	zgc:194621	55.8770678	-1.346196523	0.35238667	-3.8202255	0.00013333	0.00078103
ENSDARG00000086490	si:dkey-92f12.2	393.220635	-0.623901517	0.16332978	-3.81988836	0.00013351	0.00078191
ENSDARG00000029482	ush2a	546.699312	-1.024791257	0.26832258	-3.81925095	0.00013386	0.00078375
ENSDARG00000080174	dre-mir-124-5	100.44203	-1.331313937	0.34859575	-3.81907681	0.00013395	0.00078412
ENSDARG00000077960	si:ch211-186e20.7	102.758638	-1.519062933	0.39776686	-3.81897811	0.00013401	0.00078425
ENSDARG00000116393	CABZ01067581.1	194.589507	-0.83064443	0.21752295	-3.81865196	0.00013418	0.0007851
ENSDARG00000078057	snx8b	268.565127	-1.209307411	0.31674069	-3.81797302	0.00013455	0.0007869
ENSDARG00000033789	ndufb7	3309.39642	0.432009942	0.1131509	3.8179984	0.00013454	0.0007869
ENSDARG00000027915	zc2hc1a	833.278338	-0.528933146	0.13854772	-3.8176964	0.0001347	0.00078759
ENSDARG00000015161	zgc:92664	495.200804	-0.571050265	0.14960416	-3.81707474	0.00013504	0.00078939
ENSDARG00000102824	pcdh10b	3549.41916	-0.536767208	0.14064586	-3.81644504	0.00013539	0.00079122
ENSDARG00000015805	cgnl1	2776.87136	0.33001944	0.0864746	3.81637421	0.00013543	0.00079127
ENSDARG00000087798	pdyn	16.4002766	2.536374204	0.66467438	3.81596507	0.00013565	0.00079239
ENSDARG00000010318	srpx	1817.48303	0.361548118	0.09474782	3.81589914	0.00013569	0.00079242
ENSDARG00000117816	CABZ01078767.1	51.6071377	1.561689278	0.40926965	3.81579552	0.00013575	0.00079256
ENSDARG00000102977	cant1b	1491.32156	0.540997364	0.14182091	3.81465168	0.00013638	0.00079606
ENSDARG00000077872	CR626907.1	1144.0637	-0.686921526	0.18010085	-3.81409375	0.00013668	0.00079767
ENSDARG00000031372	efna2a	568.1975	-0.650361447	0.1705328	-3.81370298	0.0001369	0.00079874
ENSDARG00000098044	BX663503.2	163.774929	-1.307895892	0.34295797	-3.81357489	0.00013697	0.00079897
ENSDARG00000093198	c3a.4	45.6506351	1.488968259	0.39054625	3.81252732	0.00013755	0.00080218
ENSDARG00000098883	stac3	2429.57731	0.483604208	0.12685122	3.81237336	0.00013764	0.00080237
ENSDARG00000011785	tbx6	156.710377	0.857744067	0.22499075	3.81235259	0.00013765	0.00080237
ENSDARG00000021086	znf367	453.167123	-0.835187474	0.21908398	-3.81217956	0.00013775	0.00080268
ENSDARG00000022139	ocstamp	11.2219295	3.518892451	0.9230751	3.81214103	0.00013777	0.00080268
ENSDARG00000002792	arcn1a	2854.83601	0.370796283	0.09726927	3.81205998	0.00013781	0.00080275
ENSDARG00000007663	amph	3632.85143	-0.575068787	0.15086697	-3.81176064	0.00013798	0.00080354
ENSDARG00000034262	pld2	1011.27791	0.463951649	0.12172441	3.81149215	0.00013813	0.00080422
ENSDARG00000061201	acsf2	3586.89944	0.479708818	0.12586269	3.81136627	0.0001382	0.00080445
ENSDARG00000043095	kctd6a	606.917326	-0.540299343	0.14179635	-3.81038966	0.00013875	0.00080744
ENSDARG00000105407	CU459186.6	16.6352983	5.144319507	1.35015758	3.81016231	0.00013888	0.00080792
ENSDARG00000075827	f7i	732.95325	0.671363995	0.17620514	3.81012722	0.0001389	0.00080792
ENSDARG00000114227	CABZ01075242.1	215.929346	0.791327407	0.20773934	3.80923229	0.0001394	0.00081066
ENSDARG00000067713	snx18a	1020.65852	0.38500513	0.10109181	3.80846991	0.00013983	0.00081297
ENSDARG00000018936	zcchc17	563.845813	-0.575768584	0.15119027	-3.80823844	0.00013996	0.00081354
ENSDARG00000054973	itsn2b	2741.27652	0.323344753	0.08492052	3.8076164	0.00014031	0.0008154
ENSDARG00000038938	itprid2	946.582785	0.461481528	0.12122825	3.80671593	0.00014082	0.00081818
ENSDARG00000031702	prkg1b	1049.17276	0.775147214	0.20371255	3.805103	0.00014175	0.00082334
ENSDARG00000002293	si:ch211-197g15.6	186.241178	1.035439282	0.27215091	3.80465113	0.000142	0.00082465
ENSDARG00000103606	ndst1b	2127.64727	-0.350937691	0.09225139	-3.8041453	0.0001423	0.00082615
ENSDARG00000058454	dynll1	6061.252	0.423821863	0.11141947	3.80384016	0.00014247	0.00082662
ENSDARG00000058419	gcn1	8908.29815	0.394673486	0.10375467	3.80391076	0.00014243	0.00082662
ENSDARG00000008947	rtf1	2954.57988	-0.416860166	0.10958954	-3.80383157	0.00014248	0.00082662
ENSDARG00000012741	maco1b	1789.62206	-0.429412517	0.11290114	-3.80343822	0.0001427	0.00082774
ENSDARG00000017821	gata5	577.13644	0.604735285	0.15899981	3.80337116	0.00014274	0.00082777
ENSDARG00000092260	si:ch73-290k24.6	484.921896	-0.640290634	0.168351	-3.80330751	0.00014278	0.00082779
ENSDARG00000090468	ppp1r3aa	272.229773	0.656310604	0.17261516	3.80216094	0.00014344	0.00083143
ENSDARG00000076490	ankrd40	1017.1144	0.385563803	0.10143438	3.80111548	0.00014405	0.00083476
ENSDARG00000114454	CR762483.1	24.5777921	2.251262059	0.59231607	3.80077829	0.00014424	0.0008357
ENSDARG00000000212	krt97	9358.01017	1.202261233	0.31647074	3.79896496	0.0001453	0.00084164

Table S2. DEGs of WT vs. *terfa-/-*

ENSDARG00000111877	bcl7ba	358.166067	0.568487149	0.14968598	3.79786499	0.00014595	0.00084519
ENSDARG00000095855	si:ch211-163l21.1	16.58025	-4.039101526	1.06371202	-3.79717578	0.00014635	0.00084734
ENSDARG00000037773	rybpa	1481.7352	-0.512296899	0.13493315	-3.79667203	0.00014665	0.00084867
ENSDARG00000044795	entpd2b	17.8957188	2.687727267	0.70791364	3.79668807	0.00014664	0.00084867
ENSDARG00000053122	clvs2	667.570903	-0.743692129	0.19589187	-3.79644214	0.00014679	0.00084906
ENSDARG00000057881	si:dkey-33c12.3	1902.07086	0.831212487	0.21894439	3.79645489	0.00014678	0.00084906
ENSDARG00000033655	stmn1b	8074.04991	-0.429961001	0.11326427	-3.79608684	0.000147	0.00085008
ENSDARG00000095263	si:dkey-145c18.4	3.58716687	6.831882822	1.79987704	3.79574976	0.0001472	0.00085104
ENSDARG00000114908	znf1089	16.1634683	2.85158336	0.75128766	3.79559457	0.00014729	0.00085137
ENSDARG00000011404	fen1	1242.99573	-0.586089041	0.15442561	-3.79528409	0.00014747	0.00085224
ENSDARG00000058232	fbrsl1	3346.49648	-0.472950469	0.12465065	-3.79420783	0.00014812	0.00085574
ENSDARG00000004049	marcksa	1165.59526	-0.650573274	0.17148336	-3.79379836	0.00014836	0.00085696
ENSDARG00000033901	msi2a	915.442068	-0.491827413	0.12964808	-3.79355715	0.0001485	0.00085759
ENSDARG00000011490	scube3	628.058974	0.611493533	0.1612274	3.79273952	0.00014899	0.00086022
ENSDARG00000097871	si:ch1073-145m9.1	73.3782716	1.079929992	0.2847911	3.7920075	0.00014943	0.00086256
ENSDARG00000077817	cxxc4	482.535908	-0.557433098	0.1470045	-3.79194585	0.00014947	0.00086258
ENSDARG00000099720	evlb	2509.6919	-0.61239734	0.16152411	-3.79136792	0.00014982	0.00086439
ENSDARG00000079608	SGPP2	200.706996	0.729038947	0.192328	3.79060228	0.00015028	0.00086685
ENSDARG00000038386	ascl1a	415.616206	-0.857373974	0.22619575	-3.79040707	0.0001504	0.00086733
ENSDARG00000102874	immt	5946.58644	0.34641168	0.09139598	3.79022879	0.00015051	0.00086776
ENSDARG00000075021	fam120a	2027.45314	0.340588883	0.08989321	3.78881661	0.00015137	0.0008725
ENSDARG00000060330	si:ch211-284e13.4	382.411106	-0.676645528	0.17859701	-3.78867224	0.00015146	0.0008728
ENSDARG00000039117	tefa	3621.16812	0.447126618	0.11802131	3.78852452	0.00015155	0.00087312
ENSDARG00000007639	cnot4b	1096.759	0.399041944	0.105336	3.788277	0.0001517	0.00087379
ENSDARG00000090945	si:ch211-170d8.8	12.3428499	-3.697040418	0.97598614	-3.78800504	0.00015186	0.00087454
ENSDARG00000097897	sgip1a	1980.84909	-0.492468589	0.1300259	-3.78746539	0.00015219	0.00087624
ENSDARG00000063224	nxph2a	273.285138	-0.620396765	0.16380532	-3.78740314	0.00015223	0.00087626
ENSDARG00000105806	CABZ01069020.1	110.412858	-0.947119539	0.25009282	-3.78707203	0.00015243	0.00087722
ENSDARG00000078891	map11	915.739843	-0.513027942	0.13547416	-3.78690623	0.00015254	0.00087724
ENSDARG00000092651	si:dkey-57a22.11	200.021936	-0.823489715	0.21745776	-3.78689506	0.00015254	0.00087724
ENSDARG00000074293	adnpb	1294.03819	-0.516193215	0.13630976	-3.78691312	0.00015253	0.00087724
ENSDARG00000016532	suco	3861.54344	0.388760462	0.10267925	3.78616378	0.00015299	0.00087962
ENSDARG00000098397	FP016106.1	65.3160591	4.917026714	1.29880324	3.78581341	0.00015321	0.00088066
ENSDARG00000039034	marcksl1a	6775.2278	-0.565558451	0.14944676	-3.78434747	0.00015411	0.00088566
ENSDARG00000098621	CR759843.1	33.6466938	2.001732683	0.52918204	3.782692	0.00015514	0.00089136
ENSDARG00000095030	si:ch73-120g24.4	159.714268	-0.901433859	0.2383562	-3.78187718	0.00015565	0.00089408
ENSDARG00000040180	slc35a1	1125.44826	0.530975302	0.14047941	3.77973756	0.00015699	0.00090159
ENSDARG00000109443	EML4	3069.39068	0.432824639	0.11452186	3.77940645	0.0001572	0.00090258
ENSDARG00000045155	phf5a	517.029221	-0.594281746	0.15726112	-3.77894892	0.00015749	0.00090403
ENSDARG00000057531	pgap3	318.948445	0.85034015	0.22503784	3.77865403	0.00015768	0.00090489
ENSDARG00000007711	rab3il1	589.795025	0.570705913	0.15104081	3.77848816	0.00015778	0.00090529
ENSDARG00000109202	BX324216.3	125.948055	-1.027414286	0.27195008	-3.77795182	0.00015812	0.00090703
ENSDARG00000089892	cd44b	42.153055	-1.76714801	0.46779191	-3.77763699	0.00015832	0.00090797
ENSDARG00000026577	cdk2	574.141006	-0.637719706	0.16882224	-3.77746255	0.00015843	0.00090839
ENSDARG00000041431	slc24a1	136.927445	-1.358057482	0.35952259	-3.77739122	0.00015848	0.00090844
ENSDARG00000113364	CABZ01074307.2	1284.67554	0.46723912	0.12370133	3.77715506	0.00015863	0.0009091
ENSDARG00000037713	pcnp	871.945108	-0.489064796	0.12951186	-3.77621629	0.00015923	0.00091232
ENSDARG00000098991	znf1095	18.9884853	2.320700024	0.61459645	3.77597371	0.00015938	0.00091297
ENSDARG00000042652	rreb1b	1778.10273	0.51213835	0.13563259	3.77592402	0.00015942	0.00091297
ENSDARG00000102059	si:ch211-117l17.6	35.6254458	1.622870254	0.42986801	3.77527575	0.00015983	0.00091513
ENSDARG00000078585	mon1a	312.881452	0.66183993	0.17535023	3.77438865	0.0001604	0.00091818
ENSDARG00000102116	CABZ01072487.2	20.6003099	2.153370101	0.57054325	3.77424517	0.00016049	0.0009185
ENSDARG00000103923	tfap4	805.39368	-0.6043407	0.16012863	-3.77409514	0.00016059	0.00091884
ENSDARG00000077264	wdr43	2963.08298	0.339645916	0.08999922	3.77387604	0.00016073	0.00091944
ENSDARG00000114329	HOOK3	1480.05461	0.485975766	0.12879445	3.77326641	0.00016112	0.00092148
ENSDARG00000053792	si:rp71-1g18.1	489.484518	-0.462576414	0.12261003	-3.77274528	0.00016146	0.00092319
ENSDARG00000055238	kif17	40.901949	1.574614141	0.41740687	3.77237238	0.0001617	0.00092436
ENSDARG00000075461	kmt5c	2140.40219	-0.393317048	0.10426822	-3.77216632	0.00016184	0.00092491

Table S2. DEGs of WT vs. *terfa-/-*

ENSDARG00000098776	BX640547.2	27.0875002	2.544169851	0.67452508	3.77179429	0.00016208	0.00092608
ENSDARG00000039512	ap1m3	495.853934	0.581272702	0.15418642	3.76993455	0.00016329	0.00093279
ENSDARG00000007180	slc30a4	1401.41139	0.644773232	0.17103743	3.76977859	0.00016339	0.00093316
ENSDARG00000008170	dbnla	667.356139	0.476285092	0.12634935	3.76958885	0.00016352	0.00093366
ENSDARG00000056160	hspd1	10671.7678	0.387345764	0.10276179	3.76935589	0.00016367	0.00093431
ENSDARG00000007315	tmpoa	1179.20516	-0.659277219	0.17490975	-3.7692423	0.00016374	0.00093452
ENSDARG00000039697	shtn1	181.778707	0.84783101	0.22496225	3.76877008	0.00016405	0.00093608
ENSDARG00000019342	chrnd	906.046146	0.502343487	0.13329749	3.76858939	0.00016417	0.00093654
ENSDARG00000007129	slc6a16a	645.980131	0.474952693	0.12603468	3.76842871	0.00016428	0.00093693
ENSDARG00000094627		25.3578966	2.029627415	0.53864331	3.76803603	0.00016454	0.00093819
ENSDARG00000071691	uqcrc2b	9220.72513	0.39186842	0.104007	3.76771215	0.00016475	0.00093919
ENSDARG00000021443	zfp36l1b	2461.85073	0.448352481	0.11901327	3.76724773	0.00016506	0.00094072
ENSDARG00000027394	si:ch211-150g13.3	987.9052	-0.539824955	0.14329821	-3.7671437	0.00016513	0.0009409
ENSDARG00000077130	bcl10	259.371604	0.609820971	0.16188413	3.76702141	0.00016521	0.00094115
ENSDARG00000062146	larp4b	1320.24408	-0.376155527	0.0998629	-3.7667196	0.00016541	0.00094207
ENSDARG00000097685	si:ch211-235i11.3	191.977454	-0.827204936	0.21964452	-3.76610775	0.00016581	0.00094416
ENSDARG00000098734	ccdc14	152.678729	-0.797841644	0.2119177	-3.76486551	0.00016664	0.00094865
ENSDARG00000097322	si:dkey-81l17.6	306.200101	-0.744058407	0.19768118	-3.76393146	0.00016726	0.00095198
ENSDARG00000059247	tmem54a	747.678561	0.530994078	0.14110996	3.76298081	0.0001679	0.00095527
ENSDARG00000017489	ifi45	131.259275	0.986662047	0.26220406	3.76295486	0.00016792	0.00095527
ENSDARG00000034956	myclb	984.573309	-0.406603635	0.1080634	-3.76263959	0.00016813	0.00095626
ENSDARG00000074765	mhc1zja	41.1529805	1.728266902	0.45936341	3.76230858	0.00016835	0.00095731
ENSDARG00000042796	yy1a	2243.1902	-0.442449882	0.11763099	-3.76133767	0.00016901	0.00096081
ENSDARG00000110922	CR847936.6	254.916588	-0.91663795	0.24374982	-3.76056879	0.00016953	0.00096355
ENSDARG00000101029	si:ch73-347e22.4	794.718576	-0.43776659	0.11642342	-3.76012494	0.00016983	0.00096504
ENSDARG00000101193	BX855614.3	63.2560068	-1.323704909	0.35208106	-3.75965952	0.00017015	0.00096662
ENSDARG00000097591	proser3	225.578778	-0.692135259	0.18415216	-3.75849648	0.00017094	0.0009709
ENSDARG00000052154	lix1	826.746691	-0.566433098	0.15071483	-3.75831031	0.00017107	0.0009714
ENSDARG00000041572	zfpm1	855.729191	0.524783733	0.13965889	3.75761069	0.00017154	0.0009739
ENSDARG00000001825	cfap43	87.3146035	1.100022255	0.29275391	3.75749811	0.00017162	0.00097411
ENSDARG00000026629	gmds	1349.43246	0.499592295	0.13299675	3.75642497	0.00017236	0.00097807
ENSDARG00000093087	BX649388.3	8.18417853	5.528719393	1.47191201	3.75614802	0.00017255	0.00097893
ENSDARG00000036569	bach2a	174.784358	-0.775159698	0.20643405	-3.75499928	0.00017334	0.00098321
ENSDARG00000095527	CR392363.2	9.1899085	3.839807105	1.02261527	3.75488928	0.00017342	0.00098342
ENSDARG00000091560	slc6a19a.2	582.035014	0.971974579	0.25886955	3.75468878	0.00017356	0.00098398
ENSDARG00000092856	si:ch211-210c8.7	433.911221	0.581298794	0.15482648	3.75451794	0.00017368	0.00098436
ENSDARG00000089893	tasora	1904.03474	-0.504034375	0.13424886	-3.75447791	0.0001737	0.00098436
ENSDARG00000102221	HACD4	88.4575008	0.946390926	0.25212157	3.75370871	0.00017424	0.00098671
ENSDARG00000030224	ppp2ca	1875.95209	-0.366466649	0.09762613	-3.75377642	0.00017419	0.00098671
ENSDARG00000103691	rfx7b	2909.32636	-0.48391173	0.12891423	-3.75374951	0.00017421	0.00098671
ENSDARG00000005176	zgc:101040	383.827732	0.541436544	0.14424945	3.75347379	0.0001744	0.00098741
ENSDARG00000055106	znf148	1055.94936	-0.401981385	0.10710727	-3.75307302	0.00017468	0.00098877
ENSDARG00000110961	zgc:110045	600.86148	-1.798423759	0.47929162	-3.7522537	0.00017525	0.00099178
ENSDARG00000039682	si:ch211-121a2.2	148.689168	-2.343444318	0.62470258	-3.75129607	0.00017592	0.00099535
ENSDARG00000069627	si:ch211-255g12.8	21.0989073	2.54507439	0.67860505	3.75045013	0.00017652	0.00099848
ENSDARG00000013475	cct4	12780.8492	0.479693895	0.12790607	3.75036071	0.00017658	0.00099861
ENSDARG00000092419	vtg7	340.064819	2.473772142	0.65964849	3.75013689	0.00017674	0.00099928
ENSDARG00000056369	ufsp2	805.859236	0.499538952	0.13321432	3.74988929	0.00017691	0.00100004
ENSDARG00000040137	jdp2a	186.694686	0.687034439	0.1832291	3.74959236	0.00017712	0.00100099
ENSDARG00000019532	fads2	250.773129	-1.130290093	0.30148637	-3.74905864	0.0001775	0.0010029
ENSDARG00000094708	sftpba	283.291606	2.414320341	0.64412456	3.74821965	0.00017809	0.00100603
ENSDARG00000027187	kat8	1064.9119	-0.466791964	0.1245413	-3.74808986	0.00017819	0.00100632
ENSDARG00000110160	SBSPON	82.3444734	-1.512623128	0.40385859	-3.74542761	0.00018009	0.00101682
ENSDARG00000054864	aplp2	5983.84457	0.681477486	0.18195429	3.74532241	0.00018016	0.00101702
ENSDARG00000032932	cnksr1	536.764987	0.639412377	0.17073928	3.74496347	0.00018042	0.00101824
ENSDARG00000089922	dsn1	378.292393	-0.747413594	0.19967844	-3.74308608	0.00018177	0.00102565
ENSDARG00000028119	sumo3a	6036.87192	-0.475975898	0.12716798	-3.74289103	0.00018192	0.00102621
ENSDARG00000102160	si:dkeyp-61b2.1	26.5892538	2.33420886	0.6237306	3.74233502	0.00018232	0.00102826

Table S2. DEGs of WT vs. *terfa-/-*

ENSDARG00000042515	gpr176	159.784959	-0.900548537	0.24070151	-3.74134983	0.00018304	0.00103206
ENSDARG00000099093	dock1	2052.09169	0.341204577	0.09122707	3.74016793	0.0001839	0.00103646
ENSDARG00000058873	ptpdc1b	446.522974	-1.040902223	0.27830257	-3.74018181	0.00018389	0.00103646
ENSDARG00000054201	rnf40	3337.6496	-0.359527109	0.09613011	-3.74000503	0.00018402	0.00103689
ENSDARG00000019614	jade3	1076.26645	-0.408896413	0.10933546	-3.73983345	0.00018414	0.00103737
ENSDARG00000016775	aqr	2344.93512	-0.400770281	0.10716528	-3.73973995	0.00018421	0.00103752
ENSDARG00000086342	TMEM263	1008.9707	-0.427779759	0.11441098	-3.73897472	0.00018477	0.00104044
ENSDARG00000116442	TENT5A	192.37631	-0.687886595	0.18398938	-3.73872986	0.00018495	0.00104122
ENSDARG00000099774	cyb5b	1146.33274	0.466426514	0.1247668	3.73838659	0.00018521	0.00104241
ENSDARG00000003326	cacng5a	110.220911	-1.139371886	0.3047995	-3.73810293	0.00018541	0.00104335
ENSDARG00000059267	hoxd11a	207.903683	-0.68366114	0.1829695	-3.73647602	0.00018662	0.00104988
ENSDARG00000117126	BX664603.1	19.8076398	-2.244023856	0.6006749	-3.73583757	0.00018709	0.00105205
ENSDARG00000056855	gatc	471.552889	-0.622709958	0.16668785	-3.73578489	0.00018713	0.00105205
ENSDARG00000075929	si:dkey-219c10.4	43.7434513	-1.469495848	0.39335639	-3.73578738	0.00018713	0.00105205
ENSDARG00000103871	si:ch73-109d9.2	476.821687	-0.521304692	0.13956674	-3.73516414	0.00018759	0.00105441
ENSDARG00000029100	chchd1	851.991846	-0.428083212	0.11462251	-3.73472203	0.00018792	0.00105603
ENSDARG00000104057	pcdh1g14	111.465605	-1.175019611	0.31468482	-3.73395711	0.0001885	0.001059
ENSDARG00000042563	mis18bp1	279.317076	-0.799388464	0.21412702	-3.7332442	0.00018903	0.00106177
ENSDARG00000097491	ugt1b1	961.409062	0.492957939	0.13205464	3.7329845	0.00018922	0.00106262
ENSDARG00000005162	tpm3	18004.2023	0.445722148	0.11940524	3.73285262	0.00018932	0.0010627
ENSDARG00000020054	aox6	505.539501	0.663357703	0.17770692	3.73287493	0.00018931	0.0010627
ENSDARG00000103862	hoxa4a	1508.35995	-0.392045474	0.10503162	-3.73264239	0.00018948	0.00106335
ENSDARG00000093062	FP016056.1	104.337465	-1.077625294	0.2888072	-3.73129651	0.0001905	0.00106814
ENSDARG00000013822	anapc16	731.680952	0.615333408	0.16490849	3.73136285	0.00019045	0.00106814
ENSDARG00000015003	arhgap4b	1562.63394	-0.465101658	0.12464792	-3.73132316	0.00019048	0.00106814
ENSDARG00000002994	itpkca	1323.22825	-0.468814497	0.12564437	-3.73128139	0.00019051	0.00106814
ENSDARG00000077084	col28a1a	1236.20102	-0.417149948	0.1118031	-3.73111271	0.00019064	0.00106862
ENSDARG00000005972	ric8b	1383.71498	0.349626407	0.09371102	3.73089978	0.0001908	0.00106928
ENSDARG00000059906	sdc4	4434.18008	0.460429096	0.12342431	3.73045704	0.00019113	0.00107092
ENSDARG00000039269	arg2	718.209294	0.755831835	0.2026307	3.73009544	0.00019141	0.00107222
ENSDARG00000068602	dalrd3	1282.96284	-0.457625378	0.12270855	-3.72936823	0.00019196	0.00107483
ENSDARG00000025421	tdrd3	1125.98009	-0.441855561	0.11847822	-3.72942426	0.00019192	0.00107483
ENSDARG00000090170	rab11fip4a	1379.8119	-0.587036391	0.1574136	-3.72926097	0.00019204	0.00107505
ENSDARG00000061375	sgpl1	2844.65334	0.475224093	0.12745401	3.72859266	0.00019255	0.00107766
ENSDARG00000079399	F2RL3	110.186557	0.996444388	0.26725512	3.7284389	0.00019267	0.00107807
ENSDARG00000112439	CABZ01092982.2	396.812848	0.801375073	0.21500377	3.72726051	0.00019357	0.00108288
ENSDARG00000023536	nnt	30050.319	0.319788836	0.08579927	3.72717448	0.00019364	0.00108301
ENSDARG00000060362	nadkb	801.69787	0.463877595	0.12447059	3.72680477	0.00019392	0.00108435
ENSDARG00000034321	palmdb	60.7240974	-1.173351242	0.31487258	-3.72643192	0.00019421	0.00108571
ENSDARG00000104739	AL935294.2	15.3388606	-4.013180039	1.07702641	-3.72616678	0.00019441	0.00108661
ENSDARG00000023498	gmppab	556.362117	0.52315883	0.14040968	3.72594557	0.00019458	0.00108732
ENSDARG00000088116	gstm.3	740.569761	0.831400363	0.2231487	3.72576835	0.00019472	0.00108784
ENSDARG00000088207	rce1b	12.4555283	-4.027957117	1.08129789	-3.72511329	0.00019523	0.0010903
ENSDARG00000068484	CABZ01100185.1	526.255295	-0.682144838	0.18312194	-3.72508533	0.00019525	0.0010903
ENSDARG00000100524	abca3b	8777.79889	0.403220891	0.10825359	3.72478083	0.00019549	0.00109137
ENSDARG00000073963	si:ch211-258f14.2	173.483794	-0.828673514	0.22251478	-3.72412798	0.00019599	0.00109395
ENSDARG00000074712	si:ch211-186e20.2	13.9457762	-3.733041572	1.00253416	-3.72360537	0.0001964	0.00109597
ENSDARG00000095921	si:ch1073-325m22	4525.94412	0.401190582	0.10774587	3.72348917	0.00019649	0.00109623
ENSDARG00000002607	unm_sa1614	680.972418	-0.622137215	0.16709	-3.72336602	0.00019658	0.00109652
ENSDARG00000005481	nfkbiaa	577.111433	0.94219901	0.25307178	3.72305047	0.00019683	0.00109765
ENSDARG00000105118	GGH	1014.61405	0.465534882	0.12504917	3.72281475	0.00019701	0.00109843
ENSDARG00000052713	lrrtm1	1207.02871	-0.623310028	0.16747486	-3.72181262	0.0001978	0.00110255
ENSDARG00000105052	cfhl5	75.6404216	1.38058676	0.37110769	3.72017829	0.00019908	0.00110946
ENSDARG00000086744	znf1152	74.7384888	1.051296155	0.28263562	3.71961666	0.00019953	0.00111168
ENSDARG00000009252	napepld	230.407231	0.670595333	0.18029373	3.71946016	0.00019965	0.00111212
ENSDARG00000011245	esrp1	1968.82263	0.428760834	0.11531885	3.71804648	0.00020077	0.00111811
ENSDARG00000091628	cspp1b	547.112996	-0.48736209	0.13109092	-3.71774099	0.00020101	0.00111886
ENSDARG00000078041	xpo1a	4357.74861	0.404320721	0.1087523	3.71781304	0.00020096	0.00111886

Table S2. DEGs of WT vs. *terfa-/-*

ENSDARG00000057429	arg1	37.6542755	-1.540430019	0.41434935	-3.7177083	0.00020104	0.00111886
ENSDARG00000079854	scyl1	1962.81278	0.340287331	0.09156096	3.71651116	0.00020199	0.00112392
ENSDARG00000069283	ercc8	552.952595	-0.580340959	0.15616836	-3.71612382	0.0002023	0.00112514
ENSDARG00000074100	zgc:163014	12.7956855	-2.8624298	0.77026164	-3.71617857	0.00020226	0.00112514
ENSDARG00000045776	cnbpa	5874.4644	-0.381813058	0.10278189	-3.71478916	0.00020337	0.00113084
ENSDARG00000101630	si:dkey-8l13.5	40.3055873	1.393295658	0.37510604	3.71440471	0.00020368	0.0011323
ENSDARG00000095907	AL928685.2	288.423098	-0.758390104	0.20418289	-3.71426862	0.00020379	0.00113266
ENSDARG00000071644	si:dkey-19a16.7	425.34931	0.647306803	0.17428308	3.71411167	0.00020392	0.00113311
ENSDARG00000023058	foxo3a	4378.05412	-0.483878587	0.13028477	-3.71400733	0.000204	0.00113332
ENSDARG00000094280	usp21	158.245471	-2.347197332	0.63200556	-3.71388717	0.0002041	0.00113361
ENSDARG00000061249	myom1a	16893.7305	0.486165773	0.13091927	3.71347769	0.00020443	0.00113519
ENSDARG00000100897	pdgfrb	1523.42548	0.444499703	0.1197059	3.71326485	0.0002046	0.00113589
ENSDARG00000069980	lman1	1168.40427	0.419637026	0.11303694	3.71238856	0.00020531	0.00113958
ENSDARG00000090930	si:ch211-120g10.1	60.9783953	1.504950408	0.40541606	3.71211348	0.00020554	0.00114057
ENSDARG00000093289	si:dkey-9i23.14	26.3214629	-2.154957601	0.58059139	-3.71165962	0.00020591	0.00114236
ENSDARG00000100302	si:dkey-237g15.2	44.4274702	-1.643789546	0.44287891	-3.7116004	0.00020595	0.00114237
ENSDARG00000090160	zgc:173709	175.913799	1.013486569	0.27306787	3.71148236	0.00020605	0.00114265
ENSDARG00000091135	si:ch211-253p18.5	27.7852971	-2.066329073	0.55686982	-3.71061425	0.00020676	0.00114632
ENSDARG00000039093	got1	8354.06396	0.461259361	0.1243205	3.71024373	0.00020706	0.00114774
ENSDARG00000043004	si:dkeyp-117h8.4	148.695614	-0.787505273	0.21226008	-3.71009594	0.00020718	0.00114816
ENSDARG00000007221	pbk	628.093466	-0.57588009	0.15522778	-3.70990349	0.00020734	0.00114877
ENSDARG00000062960	armc10	415.151721	-0.502459173	0.13544196	-3.7097747	0.00020744	0.0011491
ENSDARG00000094242	znf1171	121.388704	-0.894841659	0.24122482	-3.70957547	0.00020761	0.00114975
ENSDARG00000012035	zgc:100832	387.256977	0.557135977	0.15021408	3.70894633	0.00020812	0.00115235
ENSDARG00000089428	rab12	791.487243	0.480797959	0.12963455	3.70887199	0.00020819	0.00115243
ENSDARG00000045835	si:dkey-14d8.6	83.9457118	-3.261581484	0.87942599	-3.70876178	0.00020828	0.00115268
ENSDARG00000004196	tnfsf10l	1960.6106	0.445497683	0.12014094	3.7081256	0.0002088	0.00115532
ENSDARG00000102618	utp18	192.054329	0.875007714	0.2360176	3.70738332	0.00020941	0.00115845
ENSDARG00000078186	tex2	2043.14698	0.405568165	0.10941232	3.70678696	0.00020991	0.00116093
ENSDARG00000070278	mettl14	379.839333	-0.711021162	0.19184358	-3.70625467	0.00021035	0.00116297
ENSDARG00000056896	ttc12	76.7616712	1.033023235	0.27872629	3.70622822	0.00021037	0.00116297
ENSDARG00000088079	tmed6	83.8625015	-1.204860008	0.32512605	-3.7058243	0.0002107	0.00116457
ENSDARG00000079784	si:ch211-235o23.1	821.121636	0.500839705	0.13515202	3.70575083	0.00021077	0.00116465
ENSDARG00000041435	uba52	22425.0106	0.428220108	0.11556333	3.70550165	0.00021097	0.00116553
ENSDARG00000033450	nkap	1455.79537	-0.456110492	0.12309296	-3.70541488	0.00021105	0.00116567
ENSDARG00000078093	zgc:172065	17.9726374	2.243812993	0.60561877	3.70499246	0.0002114	0.00116736
ENSDARG00000098952	gata4	219.728841	0.969183237	0.26165476	3.70405348	0.00021218	0.00117143
ENSDARG00000014532	aida	570.780553	-0.450037925	0.12150949	-3.70372672	0.00021246	0.00117268
ENSDARG00000101618	ube2c	387.988756	-0.628070817	0.16961707	-3.70287499	0.00021317	0.00117636
ENSDARG00000075098	wdr17	678.618593	-0.602579713	0.16274646	-3.70256733	0.00021343	0.00117753
ENSDARG00000098408	stbd1	451.687113	-0.608500889	0.16438361	-3.70171266	0.00021415	0.00118124
ENSDARG00000104399	tppp	10.4463666	2.611471506	0.70550261	3.70157595	0.00021427	0.00118162
ENSDARG00000104011	rps17	8717.70373	0.389800256	0.10533153	3.70069858	0.00021501	0.00118518
ENSDARG00000089156	egr3	307.802317	-0.813828203	0.21990985	-3.7007355	0.00021498	0.00118518
ENSDARG00000096826	BX323575.1	9.03903341	3.819986163	1.03267959	3.69910107	0.00021636	0.0011924
ENSDARG00000004592	gpr22a	365.760061	-0.546639966	0.14777998	-3.6990124	0.00021644	0.00119256
ENSDARG00000039752	si:dkey-167k11.5	440.211127	0.846154227	0.22877759	3.69858879	0.0002168	0.00119428
ENSDARG00000069808	ostm1	441.020175	-0.574623298	0.15537381	-3.69832793	0.00021702	0.00119525
ENSDARG00000094840	si:dkey-21e2.13	19.4901448	-3.314704727	0.8962868	-3.69826346	0.00021708	0.00119528
ENSDARG00000043684	bpnt1	615.289844	0.675443338	0.18265476	3.69792348	0.00021737	0.00119662
ENSDARG00000001162	matr3l1.1	2350.91476	-0.335738407	0.09080131	-3.69750613	0.00021773	0.00119832
ENSDARG00000030449	crabp2b	2101.85786	0.562349418	0.15209307	3.69740326	0.00021782	0.00119854
ENSDARG00000052019	si:ch73-109d9.1	275.585103	-0.575397247	0.1556247	-3.69733873	0.00021787	0.00119858
ENSDARG00000076856	frem2a	2357.80835	0.562052878	0.15203704	3.69681541	0.00021832	0.00120079
ENSDARG00000009014	col11a1b	20037.1593	0.386968044	0.1046798	3.69668304	0.00021844	0.00120115
ENSDARG00000097264	si:ch211-40k21.5	242.564646	-0.636124458	0.17208933	-3.69647829	0.00021861	0.00120159
ENSDARG00000098214	CABZ01072487.1	150.2106	-0.907437397	0.2454838	-3.69652662	0.00021857	0.00120159
ENSDARG00000110105	CABZ01044235.1	164.260212	0.768009755	0.20778156	3.69623631	0.00021882	0.00120247

Table S2. DEGs of WT vs. *terfa-/-*

ENSDARG00000098671	prelid1a	2067.48024	-0.481716449	0.13034863	-3.69560042	0.00021937	0.00120521
ENSDARG00000011609	FOXK2	2809.1401	-0.41052796	0.11110124	-3.69508008	0.00021982	0.00120742
ENSDARG00000093449	CR388143.1	41.566495	1.349872297	0.36532334	3.69500704	0.00021988	0.0012075
ENSDARG00000099293	BX914214.2	18.4799894	-2.807611418	0.75987283	-3.6948438	0.00022002	0.00120801
ENSDARG00000117646	CABZ01072309.2	2457.09894	0.444315165	0.120271	3.69428355	0.00022051	0.00121041
ENSDARG00000041378	sh2d4a	174.042761	0.764398788	0.20692055	3.69416575	0.00022061	0.0012107
ENSDARG00000103926	inpp5e	618.56842	-0.44509956	0.12052831	-3.69290474	0.00022171	0.00121645
ENSDARG00000025319	fynb	713.314872	-0.679184894	0.18396163	-3.6919922	0.0002225	0.00122056
ENSDARG00000095157	zgc:163080	226.195112	0.782794055	0.21203881	3.691749	0.00022272	0.00122146
ENSDARG00000039020	fbxo5	352.401911	-1.102768962	0.29873329	-3.69148332	0.00022295	0.00122246
ENSDARG00000069619	atf7ip	6770.46514	-0.476092616	0.12898117	-3.69117925	0.00022322	0.00122366
ENSDARG00000079290	si:ch211-162k9.5	14.0636621	2.317141799	0.62779758	3.69090591	0.00022346	0.0012247
ENSDARG00000101385	f8	520.61345	0.701173825	0.19006594	3.68910818	0.00022504	0.00123311
ENSDARG00000104886	CABZ01085365.1	158.941382	0.740115271	0.20063888	3.68879296	0.00022532	0.00123437
ENSDARG00000057025	chrna2b	194.984065	-0.729095993	0.19768256	-3.68821616	0.00022583	0.0012369
ENSDARG00000002336	dlc	1407.74505	-0.517715592	0.14041049	-3.68715741	0.00022677	0.00124178
ENSDARG00000033597	api5	4147.96052	-0.424266034	0.11514596	-3.68459345	0.00022907	0.00125407
ENSDARG00000104656	si:dkeyp-97a10.3	268.667208	0.55844237	0.15157231	3.68432974	0.00022931	0.00125509
ENSDARG00000020764	slmapb	636.950363	-0.56511506	0.15343177	-3.68316853	0.00023035	0.00126055
ENSDARG00000062122	klhl5	412.067259	0.683994469	0.18578796	3.68158657	0.00023179	0.00126812
ENSDARG00000016623	si:ch211-195b13.1	1732.13993	-0.719216807	0.19540866	-3.68057796	0.00023271	0.00127287
ENSDARG00000075569	gpbp1l1	2858.74913	-0.308065302	0.08371381	-3.67998182	0.00023325	0.00127557
ENSDARG00000038658	cavin4a	3530.59058	0.509453037	0.13846238	3.67936082	0.00023382	0.00127812
ENSDARG00000056519	si:dkey-280e21.3	823.718905	-0.610391289	0.16589607	-3.67935953	0.00023382	0.00127812
ENSDARG00000101009	cited4b	2043.10731	-0.483495012	0.13143072	-3.67870616	0.00023442	0.00128112
ENSDARG00000016141	slc6a2	368.002017	-0.696788558	0.18941681	-3.67859945	0.00023452	0.00128137
ENSDARG00000032603	st7l	329.669819	-0.646450158	0.17582846	-3.67659573	0.00023637	0.00129119
ENSDARG00000017474	zgc:110699	97.5280272	-1.123016152	0.3055096	-3.67587848	0.00023703	0.00129454
ENSDARG00000004131	rttn	326.891435	-0.679338334	0.18483327	-3.67541159	0.00023747	0.00129662
ENSDARG00000071492	fbxo45	769.914179	-0.436455137	0.11875213	-3.6753457	0.00023753	0.00129667
ENSDARG00000099786	actn4	16708.2105	0.259915949	0.07072265	3.6751446	0.00023772	0.00129741
ENSDARG00000052138	slc1a2a	693.895385	-2.144097377	0.58345761	-3.67481261	0.00023802	0.00129881
ENSDARG00000025089	loxl4	823.213743	0.449512362	0.12233038	3.67457666	0.00023824	0.00129973
ENSDARG00000009685	ppa2	282.717543	-0.561589857	0.15283604	-3.67445954	0.00023835	0.00130004
ENSDARG00000009610	rgs12a	966.638834	-0.474785001	0.12923866	-3.67370717	0.00023906	0.00130359
ENSDARG00000043322	gsx2	137.571993	-1.12565259	0.30642587	-3.67349076	0.00023926	0.00130441
ENSDARG00000039929	ckmt2b	9220.41476	0.574293396	0.15645047	3.67076814	0.00024182	0.00131809
ENSDARG00000104030	taf12	2703.5078	-0.422850265	0.11520194	-3.67051335	0.00024206	0.00131836
ENSDARG00000117337	CABZ01071874.1	34.4925988	1.614169317	0.43976909	3.67049289	0.00024208	0.00131836
ENSDARG00000069601	snx30	516.43696	-0.575744311	0.15685745	-3.67049386	0.00024208	0.00131836
ENSDARG00000109460	CR762420.1	21.6751361	2.306463923	0.62836175	3.67059887	0.00024198	0.00131836
ENSDARG00000100338	BX936384.1	8.01148813	4.574253037	1.24624732	3.67042158	0.00024215	0.00131844
ENSDARG00000102266	isg20l2	249.84243	-0.749130469	0.20410588	-3.67030321	0.00024226	0.00131876
ENSDARG00000014685	esco2	807.491126	-0.654531214	0.1783893	-3.66911694	0.00024339	0.0013246
ENSDARG00000003328	slf2	465.071655	-0.545085711	0.14858287	-3.66856371	0.00024392	0.00132718
ENSDARG00000098452	si:dkey-151g22.1	14.9993266	2.421058157	0.66000855	3.66822243	0.00024424	0.00132866
ENSDARG00000030722	xirp1	852.824165	-0.378000389	0.10308091	-3.66702597	0.00024539	0.0013346
ENSDARG00000017388	gstt1b	646.168858	-0.713531792	0.19459417	-3.66676859	0.00024564	0.00133565
ENSDARG00000074387	alkal1	21.2042025	-2.419079034	0.65974782	-3.66667228	0.00024573	0.00133586
ENSDARG00000038918	drd1b	186.65132	-0.981903118	0.26779817	-3.66657884	0.00024582	0.00133606
ENSDARG00000092240	si:ch211-243a20.3	416.646835	-0.743378382	0.20276133	-3.66627306	0.00024611	0.00133736
ENSDARG00000016837	glipr2l	189.779372	0.833587127	0.22740651	3.6656256	0.00024674	0.00134046
ENSDARG00000103320	lclat1	2125.32229	0.506923694	0.13831002	3.66512621	0.00024722	0.00134278
ENSDARG00000117707	CABZ01072532.1	122.465452	1.546089196	0.42188289	3.66473553	0.00024759	0.00134425
ENSDARG00000074828	rhobtb2a	165.688679	1.074858023	0.29329403	3.6647798	0.00024755	0.00134425
ENSDARG00000097920	tex36	8.08463439	3.382795336	0.92318538	3.66426444	0.00024805	0.00134643
ENSDARG00000096217	si:dkey-51d8.9	34.4216602	-1.702673783	0.46467938	-3.66419054	0.00024812	0.00134652
ENSDARG00000101399	CR788226.1	25.9458852	1.810813276	0.49420962	3.66405916	0.00024825	0.00134692

Table S2. DEGs of WT vs. *terfa-/-*

ENSDARG00000117643	CU896641.1	11.9019574	-4.674594967	1.27587023	-3.66384829	0.00024845	0.00134774
ENSDARG00000054076	tatdn3	50.5861413	1.31464922	0.3588344	3.66366554	0.00024863	0.0013484
ENSDARG00000009494	xrcc1	1112.83828	-0.550812538	0.15034769	-3.6635917	0.0002487	0.0013485
ENSDARG00000043673	si:dkey-174m14.3	630.541955	-0.540816849	0.1476258	-3.6634304	0.00024886	0.00134905
ENSDARG00000008966	tbl1xr1b	1540.86954	-0.324836144	0.08868842	-3.6626669	0.0002496	0.00135249
ENSDARG00000104493	TBC1D8	519.937612	0.696280416	0.19010107	3.66268535	0.00024959	0.00135249
ENSDARG00000076857	her3	18.1626107	-5.640600688	1.54040194	-3.661772	0.00025048	0.00135693
ENSDARG00000060951	polg	483.185446	0.618691673	0.16896735	3.66160497	0.00025064	0.00135752
ENSDARG00000027584	ttpa	261.038538	-0.842071664	0.22997834	-3.66152592	0.00025072	0.00135764
ENSDARG00000098944	nme8	114.519257	1.064907574	0.29085743	3.66127001	0.00025097	0.0013587
ENSDARG00000070012	sesn2	392.053368	0.890230331	0.24316183	3.66106111	0.00025117	0.00135952
ENSDARG00000101628	ascl1b	549.901391	-0.787825323	0.21522676	-3.66044324	0.00025178	0.0013625
ENSDARG00000053456	GK3P	1096.45415	-0.369893359	0.10107455	-3.65960918	0.0002526	0.00136665
ENSDARG00000038489	b3gnt7	602.63366	0.698352133	0.19083204	3.65951199	0.0002527	0.00136687
ENSDARG00000023703	kctd6b	741.955414	-0.46299566	0.12653468	-3.65904164	0.00025316	0.00136908
ENSDARG00000090537	ifit11	52.4577702	1.43000213	0.39084683	3.65872772	0.00025347	0.00137046
ENSDARG00000016343	zgc:92518	322.069627	0.721914973	0.19736957	3.65768117	0.00025451	0.00137577
ENSDARG00000079756	peak1	1249.61257	0.685762725	0.18750201	3.65736191	0.00025482	0.00137718
ENSDARG00000042467	tlk1a	1648.96363	-0.323184518	0.08837329	-3.65703853	0.00025515	0.00137862
ENSDARG00000036335	c1galt1c1	872.565655	0.493585445	0.13498301	3.65664864	0.00025553	0.00138039
ENSDARG00000089223	ngfrb	395.150555	0.649614041	0.17765533	3.65659759	0.00025559	0.00138039
ENSDARG00000117400	CU651668.1	296.965524	0.581349292	0.15899496	3.65640067	0.00025578	0.00138115
ENSDARG00000070723	ndufa9a	6004.56878	0.337390114	0.09230579	3.65513507	0.00025705	0.00138768
ENSDARG00000074804	atxn7	626.438008	-0.476822024	0.13046513	-3.6547852	0.0002574	0.00138897
ENSDARG00000014793	ehd1b	3197.3418	0.393301537	0.10761222	3.65480378	0.00025738	0.00138897
ENSDARG00000040110	kif20bb	717.702654	-0.671766569	0.18382512	-3.65437844	0.00025781	0.00139088
ENSDARG00000017193	cnn1a	19.9196878	-2.956890035	0.80926737	-3.65378631	0.0002584	0.00139379
ENSDARG00000117484	CABZ01052570.1	308.005539	1.044464349	0.28587092	3.65362223	0.00025857	0.00139438
ENSDARG00000054777	C8H5orf51	793.791119	-0.483871656	0.13245453	-3.6531153	0.00025908	0.00139683
ENSDARG00000069440	dachd	2198.16816	-0.42368412	0.11598624	-3.65288277	0.00025931	0.00139779
ENSDARG00000045141	aqp8a.1	893.150071	0.636614445	0.17428666	3.65268593	0.00025951	0.00139856
ENSDARG00000006754	mllt3	547.523451	-0.429586926	0.11761242	-3.65256434	0.00025964	0.00139862
ENSDARG00000037402	lim2.3	653.632852	-0.751128606	0.2056427	-3.65259076	0.00025961	0.00139862
ENSDARG00000052693	si:dkey-172m14.1	606.187062	0.504770005	0.13821092	3.65217155	0.00026003	0.00140045
ENSDARG00000020623	baxa	1254.9352	0.449483941	0.12310694	3.65116646	0.00026105	0.00140529
ENSDARG00000034086	zgc:65895	1277.26444	-0.543155274	0.14876402	-3.65111999	0.0002611	0.00140529
ENSDARG00000054794	plcxd3	542.018987	-0.616312113	0.16879698	-3.65120354	0.00026101	0.00140529
ENSDARG00000029039	zdhhc23a	112.872778	1.168661649	0.32010966	3.65081656	0.00026141	0.00140665
ENSDARG00000102673	si:dkey-26i24.1	27.9438912	2.017352116	0.55265248	3.65030862	0.00026193	0.00140913
ENSDARG00000059109	si:dkeyp-113d7.1	2155.94554	-0.45629891	0.12502024	-3.64980037	0.00026244	0.00141161
ENSDARG00000059349	homer2	1131.33033	0.600961634	0.164667	3.64955728	0.00026269	0.00141264
ENSDARG00000052765	gria2b	2257.30501	-0.425028802	0.11649055	-3.64861178	0.00026366	0.00141754
ENSDARG00000107130	CU927890.1	341.991305	0.67946908	0.18624851	3.64818525	0.0002641	0.00141959
ENSDARG00000007276	ela3l	792.66516	-3.673804671	1.00706803	-3.64802035	0.00026427	0.00142019
ENSDARG00000091234	si:ch73-335l21.4	325.433769	1.035872464	0.2839762	3.64774391	0.00026455	0.00142142
ENSDARG00000020219	dld	751.444307	-0.877565992	0.24059987	-3.64740841	0.0002649	0.00142297
ENSDARG00000102558	pde6ha	1929.4415	-2.073994793	0.56866692	-3.64711701	0.0002652	0.00142427
ENSDARG00000076861	tmem79b	563.137015	0.554961476	0.15217876	3.64677348	0.00026555	0.00142587
ENSDARG00000052063	col4a5	30141.9488	0.322489827	0.08845886	3.64564759	0.00026672	0.00143151
ENSDARG00000057912	eif1axb	3965.16707	-0.322413182	0.08843665	-3.64569661	0.00026667	0.00143151
ENSDARG00000001015	znf384l	1756.56161	-0.477704759	0.13104983	-3.64521466	0.00026717	0.00143361
ENSDARG00000077023	pcdhb	294.224375	-0.698341704	0.19159141	-3.64495311	0.00026744	0.00143476
ENSDARG00000086539	CABZ01115881.1	671.584777	0.471618666	0.12939524	3.64479149	0.00026761	0.00143535
ENSDARG00000104110	col4a2	3400.33019	0.459625402	0.12610979	3.64464479	0.00026776	0.00143586
ENSDARG00000059916	hyal1	128.508656	0.897503733	0.24629397	3.64403461	0.0002684	0.00143896
ENSDARG00000017591	fat1a	11788.6383	0.513583863	0.14094522	3.64385436	0.00026859	0.0014394
ENSDARG00000089791	slc25a32a	1080.66019	-0.484675407	0.13301205	-3.64384576	0.00026859	0.0014394
ENSDARG00000018145	mid1ip1l	751.480844	-0.445604111	0.12229453	-3.64369629	0.00026875	0.00143992

Table S2. DEGs of WT vs. *terfa-/-*

ENSDARG00000043864	nptnb	1577.02879	-0.520746738	0.14294011	-3.64311137	0.00026936	0.00144289
ENSDARG00000069696	si:ch211-261n11.5	891.275692	-0.393120891	0.10792703	-3.64246943	0.00027004	0.00144618
ENSDARG00000104458	rnasekb	1457.42804	-0.475217701	0.13046957	-3.64236423	0.00027015	0.00144646
ENSDARG00000092895	si:dkey-188i13.11	435.981935	0.765507177	0.21020579	3.64170364	0.00027084	0.00144987
ENSDARG00000088082	si:ch211-214c20.1	23.2758581	2.261057708	0.62097834	3.6411217	0.00027145	0.00145283
ENSDARG00000105144	znf1130	67.9842885	1.451789343	0.39878068	3.64057091	0.00027203	0.00145563
ENSDARG00000035544	etnppl	1088.45523	-0.707193824	0.19427323	-3.6402022	0.00027242	0.0014574
ENSDARG00000042182	drc1	105.865321	1.006391446	0.27647842	3.64003617	0.0002726	0.00145803
ENSDARG00000079382	hdx	599.369191	-0.840299388	0.23090591	-3.6391419	0.00027355	0.00146279
ENSDARG00000087596	si:ch73-95l15.5	1152.15008	0.372080952	0.10225471	3.63876607	0.00027395	0.00146461
ENSDARG00000015293	fam110a	250.267982	0.651321584	0.17903078	3.63804249	0.00027472	0.00146841
ENSDARG00000004796	mgrn1b	1854.19131	-0.378121659	0.10394483	-3.63771483	0.00027507	0.00146997
ENSDARG00000104169	si:ch211-212k18.4	5524.38301	-0.293928997	0.08081773	-3.63693722	0.0002759	0.00147409
ENSDARG00000056127	polr2gl	1122.91405	-0.499489214	0.13734936	-3.63663291	0.00027623	0.00147552
ENSDARG00000057665	sprn2	279.301561	-0.646888824	0.17790069	-3.63623563	0.00027665	0.00147716
ENSDARG00000034165	lingo1a	678.239698	-0.576941669	0.1586623	-3.63628703	0.0002766	0.00147716
ENSDARG00000038052	si:ch211-149k23.9	78.7790901	-1.011606551	0.2782213	-3.63597815	0.00027693	0.00147832
ENSDARG00000007624	gins1	283.704204	-0.629401774	0.17314138	-3.63518972	0.00027778	0.00148253
ENSDARG00000086449	znf1055	20.9397073	2.02108689	0.55622099	3.63360415	0.00027949	0.00149135
ENSDARG00000075748	NCKAP1L	308.483345	0.627033955	0.17258523	3.63318433	0.00027995	0.00149346
ENSDARG00000068432	si:dkey-56m19.5	8733.59554	-0.496307374	0.13664445	-3.63210777	0.00028112	0.00149902
ENSDARG00000099260	PITPNC1	201.766981	0.763411641	0.21018439	3.63210431	0.00028112	0.00149902
ENSDARG00000078504	si:ch73-60h1.1	400.768832	-0.592796342	0.16321215	-3.63206023	0.00028117	0.00149902
ENSDARG00000026149	slc46a1	368.968142	0.625669412	0.17226706	3.63197357	0.00028126	0.0014992
ENSDARG00000117249	CABZ01079872.1	5.95479483	5.351490926	1.47347393	3.63188707	0.00028136	0.00149938
ENSDARG00000055477	pelo	1221.78005	0.400374668	0.1102522	3.63144376	0.00028184	0.00150099
ENSDARG00000009194	col16a1	7605.74482	0.330695712	0.09106239	3.63152895	0.00028175	0.00150099
ENSDARG00000019772	si:ch1073-280e3.1	142.484998	1.546008647	0.42572193	3.63149872	0.00028178	0.00150099
ENSDARG00000070214	slc41a1	323.320554	0.690289925	0.19009815	3.63122906	0.00028208	0.00150192
ENSDARG00000077057	esco1	688.827294	-0.38538217	0.10613488	-3.63106036	0.00028226	0.00150258
ENSDARG00000077726	nocta	786.543391	0.549464691	0.15135265	3.63036065	0.00028303	0.00150634
ENSDARG00000055989	tmem161b	673.93518	-0.428782604	0.11814309	-3.62934987	0.00028414	0.00151192
ENSDARG00000038615	pip4p1a	455.504101	-0.516608577	0.14239054	-3.62811034	0.0002855	0.00151824
ENSDARG00000061405	eva1a	268.010237	-0.595327746	0.16409011	-3.62805371	0.00028557	0.00151824
ENSDARG00000086027	si:ch211-168d23.3	309.97681	-0.798475547	0.22007434	-3.6282083	0.0002854	0.00151824
ENSDARG00000060983	smyd4	202.231676	-0.666596755	0.18373405	-3.62805245	0.00028557	0.00151824
ENSDARG00000014190	sst2	9.13222353	3.523898471	0.97150329	3.62726353	0.00028644	0.00152256
ENSDARG00000069196	ebf1b	216.845415	-0.721821075	0.19909178	-3.62556937	0.00028833	0.00153225
ENSDARG00000074070	irx5b	270.591598	-0.737063879	0.20332513	-3.62505061	0.00028891	0.001535
ENSDARG00000068709	fam174b	416.239213	-0.62203715	0.17159781	-3.62497146	0.00028899	0.00153514
ENSDARG00000069469	khdrbs2	904.624112	-0.550547212	0.15189152	-3.62460806	0.0002894	0.00153697
ENSDARG00000037238	smad5	1693.59976	0.397042992	0.10954697	3.62440886	0.00028962	0.00153783
ENSDARG00000039987	hivep2a	2161.66551	0.46792913	0.12911333	3.62417365	0.00028989	0.0015389
ENSDARG00000095865	oxr1a	2112.43412	-0.461480344	0.12736638	-3.62325091	0.00029092	0.00154375
ENSDARG00000013990	ube2q2	2545.10845	-0.354622709	0.09787332	-3.62328273	0.00029089	0.00154375
ENSDARG00000013730	slc4a4a	4522.55096	-0.463457408	0.12793972	-3.62246695	0.00029181	0.00154778
ENSDARG00000019033	tmem59	2100.9201	0.381144303	0.10521684	3.62246497	0.00029181	0.00154778
ENSDARG00000079897	si:dkey-7k24.5	36.203466	-1.497897853	0.41353248	-3.6222012	0.00029211	0.00154903
ENSDARG00000101728	si:dkeyp-80d11.12	7.74654818	3.819985872	1.05466913	3.62197563	0.00029236	0.00155005
ENSDARG00000093997	si:dkey-9i23.15	237.355777	0.898256599	0.24801032	3.62185174	0.0002925	0.00155047
ENSDARG00000039513	C18H19orf54	115.076933	0.863124186	0.238316	3.62176355	0.0002926	0.00155066
ENSDARG00000013843	sept5a	893.999287	-0.472820089	0.13055281	-3.62167674	0.0002927	0.00155084
ENSDARG00000096808	si:dkey-287g12.6	178.469659	-0.793021777	0.21896852	-3.62162464	0.00029276	0.00155084
ENSDARG00000093105	BX004774.1	18.2486304	-2.692117667	0.74342613	-3.62123089	0.00029321	0.00155287
ENSDARG00000061096	dapk2a	210.419947	0.996235609	0.27512356	3.62104796	0.00029341	0.00155363
ENSDARG00000040265	mpdu1b	908.685895	0.527807984	0.14577433	3.62071968	0.00029379	0.00155494
ENSDARG00000110545	CABZ01113373.1	1086.42731	-0.686700714	0.18965829	-3.62072615	0.00029378	0.00155494
ENSDARG00000086703	si:dkey-126g1.7	54.662446	1.357880547	0.37518288	3.61924977	0.00029546	0.00156347

Table S2. DEGs of WT vs. *terfa-/-*

ENSDARG00000058287	gpalpp1	555.276839	-0.626252032	0.17310602	-3.61773681	0.00029719	0.0015723
ENSDARG00000078179	fndc3ba	1802.45299	0.468098199	0.12942269	3.61681709	0.00029825	0.00157722
ENSDARG00000040649	prickle1a	514.472059	0.495229365	0.13692341	3.61683502	0.00029823	0.00157722
ENSDARG00000074060	map3k14a	585.278862	0.496515046	0.13728508	3.61667159	0.00029842	0.00157778
ENSDARG00000117069	CABZ01060373.1	252.222942	0.977267155	0.27023989	3.61629502	0.00029885	0.00157973
ENSDARG00000019362	ptbp1a	2693.57249	-0.345569041	0.09557061	-3.61585056	0.00029936	0.00158211
ENSDARG00000019418	kcnj2a	502.594578	0.552024191	0.15269635	3.615176	0.00030014	0.0015859
ENSDARG00000041530	ppih	574.956415	-0.47672888	0.13188456	-3.61474366	0.00030065	0.00158821
ENSDARG00000117533	nadsyn1	103.637094	0.852018701	0.23578168	3.61359159	0.00030199	0.00159431
ENSDARG00000025789	chd4b	12267.2499	-0.395909054	0.10956126	-3.61358607	0.00030199	0.00159431
ENSDARG00000007923	ptpn1	1214.89851	0.398676025	0.11032486	3.61365557	0.00030191	0.00159431
ENSDARG00000012729	hcls1	1290.93759	0.434601638	0.12028673	3.61304721	0.00030262	0.00159728
ENSDARG00000008573	fam20b	1531.21127	0.433892893	0.12010482	3.61261847	0.00030312	0.00159959
ENSDARG00000076442	zgc:174310	503.73584	0.566606397	0.15687216	3.61189901	0.00030396	0.00160369
ENSDARG00000052695	nr2f1a	4683.63875	-0.504263228	0.13961847	-3.61172292	0.00030417	0.00160379
ENSDARG00000116928	CABZ01059408.1	80.8686489	0.985635087	0.27289351	3.61179379	0.00030409	0.00160379
ENSDARG00000095019	lmo2	280.195584	-0.659970199	0.18273021	-3.6117192	0.00030417	0.00160379
ENSDARG00000055395	foxq1b	514.692643	-0.640514742	0.17736327	-3.61131556	0.00030465	0.00160594
ENSDARG00000075629	si:dkey-197i20.6	226.633661	-0.680374286	0.18842866	-3.61077923	0.00030528	0.00160893
ENSDARG00000086906	stambpb	598.618605	-0.556627874	0.1541636	-3.61063105	0.00030545	0.0016095
ENSDARG00000017315	larp7	698.952124	-0.485873919	0.13457566	-3.61041449	0.00030571	0.00161051
ENSDARG00000038785	abcf2a	3121.18164	0.38655966	0.10707219	3.61027139	0.00030588	0.00161106
ENSDARG00000056141	coq10b	1731.03715	0.45867627	0.12706804	3.6096904	0.00030656	0.00161433
ENSDARG00000078760	cacna2d3	1018.79325	-0.438708168	0.1215735	-3.60858377	0.00030787	0.00162088
ENSDARG00000089626	ptges3b	2745.87894	-0.433308945	0.12008277	-3.60841907	0.00030807	0.00162157
ENSDARG00000017036	svilc	1931.37081	0.487187279	0.13507262	3.60685444	0.00030993	0.00163103
ENSDARG00000086985	klc4	2164.64175	0.35568425	0.09863063	3.60622512	0.00031068	0.00163464
ENSDARG00000017023	ak7b	96.5469153	1.136663771	0.31527226	3.60534024	0.00031174	0.00163987
ENSDARG00000042368	kif26aa	847.530817	-0.498183475	0.13818582	-3.60517065	0.00031195	0.0016406
ENSDARG00000055566	mastl	427.004129	-0.689818129	0.19138323	-3.60438136	0.0003129	0.00164524
ENSDARG00000061656	ptpdc1a	426.462462	-0.491682475	0.1364235	-3.60408934	0.00031325	0.00164675
ENSDARG00000091185	FP017295.1	27.7866592	2.352439778	0.65291203	3.60299651	0.00031457	0.00165334
ENSDARG00000057857	arfrp1	690.295605	-0.454179908	0.12606007	-3.60288491	0.00031471	0.0016537
ENSDARG00000032199	gpc3	199.327717	-0.77274002	0.21449503	-3.60260099	0.00031505	0.00165516
ENSDARG00000091584	si:ch73-217n20.1	493.406073	0.593557216	0.16486614	3.60023734	0.00031793	0.00166992
ENSDARG00000052438	actr2a	4180.16608	0.299096552	0.08309371	3.59950899	0.00031882	0.00167425
ENSDARG00000077722	ppp2r3a	2080.49189	0.579674041	0.16109215	3.59840034	0.00032018	0.00168105
ENSDARG00000103795	zgc:113348	430.13034	-0.582902955	0.1620027	-3.59810654	0.00032054	0.00168224
ENSDARG00000097972	CT033842.1	4.69571282	5.566492329	1.54703843	3.59816034	0.00032048	0.00168224
ENSDARG00000099387	jpt1a	288.30703	-0.63613	0.17684016	-3.59720333	0.00032166	0.00168773
ENSDARG00000104584	si:dkeyp-4f2.3	43.4461975	-1.454799601	0.40457609	-3.59586159	0.00032332	0.00169588
ENSDARG00000039456	acbd6	894.591309	-0.452946913	0.12596412	-3.59584078	0.00032335	0.00169588
ENSDARG00000043589	ca4a	612.421062	-0.773958236	0.21524234	-3.5957528	0.00032346	0.00169609
ENSDARG00000105816	BX855614.4	149.377077	-0.706311212	0.19650018	-3.59445581	0.00032507	0.0017042
ENSDARG00000042956	cyp2ad6	143.065255	-0.803829536	0.22364427	-3.59423267	0.00032535	0.0017053
ENSDARG00000020252	btbd10b	2243.323	0.355531565	0.09894552	3.59320517	0.00032664	0.00171168
ENSDARG00000006200	eif4g1a	11821.8045	0.330420489	0.09196922	3.59272892	0.00032723	0.00171446
ENSDARG00000076735	si:dkey-193c22.2	1229.71248	0.499923761	0.13915793	3.59249209	0.00032753	0.00171565
ENSDARG00000068436	si:ch1073-429i10.3	13172.2727	-0.531485352	0.14796111	-3.59206124	0.00032807	0.00171813
ENSDARG00000004561	prkcg	78.1910637	-1.259714412	0.3507091	-3.59190679	0.00032827	0.00171879
ENSDARG00000045692	pphln1	1267.23319	-0.389101452	0.10832998	-3.59181703	0.00032838	0.00171902
ENSDARG00000027609	hpn	157.697822	0.85670608	0.23853421	3.59154384	0.00032873	0.00172046
ENSDARG00000005713	ethe1	404.896446	0.60682003	0.16896306	3.59143601	0.00032886	0.00172081
ENSDARG00000059911	bbs7	611.255273	-0.438697171	0.12215417	-3.59134014	0.00032898	0.00172108
ENSDARG00000077227	fbxl6	221.929693	-0.817787186	0.22775573	-3.59063277	0.00032988	0.00172539
ENSDARG00000117076	BX005085.7	262.691268	-0.609841633	0.16988333	-3.58976729	0.00033097	0.00173073
ENSDARG00000056806	pde5aa	43.6354458	-1.661172083	0.46275827	-3.58971888	0.00033104	0.00173073
ENSDARG00000076798	C9H3orf38	156.812893	0.767460481	0.21379875	3.58963974	0.00033114	0.00173089

Table S2. DEGs of WT vs. *terfa-/-*

ENSDARG00000060394	mtfr2	222.455551	-0.779137851	0.21708278	-3.58912778	0.00033179	0.00173393
ENSDARG00000059244	arfip2a	1228.10653	-0.485510721	0.13528957	-3.58867812	0.00033236	0.00173655
ENSDARG00000091079	si:zfos-1192g2.3	1011.66327	0.501693843	0.13980312	3.58857394	0.00033249	0.00173688
ENSDARG00000099596	btd	230.467277	0.811939106	0.22626885	3.58838212	0.00033274	0.00173779
ENSDARG00000039863	lifrb	220.972995	0.949912148	0.26472735	3.58826595	0.00033289	0.0017382
ENSDARG00000062711	gal3st1a	300.119637	-0.726094048	0.20236024	-3.58812597	0.00033306	0.00173877
ENSDARG00000069048	serpinf1	2762.10373	0.408276304	0.11379036	3.58796929	0.00033326	0.00173945
ENSDARG00000104489	CR855389.2	40.5934031	3.894169893	1.08542459	3.58769273	0.00033362	0.00174093
ENSDARG00000060101	trappc12	1179.13375	-0.488353542	0.1361525	-3.58681302	0.00033474	0.00174645
ENSDARG00000014273	camk2d2	3181.51537	-0.525814351	0.14660538	-3.58659664	0.00033502	0.00174753
ENSDARG00000077403	col8a1a	1153.84937	-0.905224604	0.25243762	-3.58593388	0.00033587	0.00175124
ENSDARG00000036107	txnipa	12134.6803	0.60221053	0.16793524	3.58596886	0.00033583	0.00175124
ENSDARG00000068176	uqcc1	1422.63564	0.37180559	0.10370801	3.58511937	0.00033692	0.00175606
ENSDARG00000040319	CABZ01041494.1	29.7606745	1.540532539	0.42970331	3.58510749	0.00033694	0.00175606
ENSDARG00000020031	cldn11a	1037.98789	0.490612406	0.13686204	3.58472231	0.00033744	0.00175828
ENSDARG00000093899	BX510657.1	1036.2128	0.42288218	0.11799162	3.5840018	0.00033837	0.00176277
ENSDARG00000037941	syt5a	529.223029	-1.895724845	0.52897244	-3.58378756	0.00033865	0.0017637
ENSDARG00000042876	abracl	2139.24041	0.493605356	0.13773412	3.58375516	0.00033869	0.0017637
ENSDARG00000090389	ndufv3	1786.39895	0.516065168	0.14401179	3.58349252	0.00033903	0.0017651
ENSDARG00000056913	pcolcea	1680.63929	0.468683548	0.13079887	3.58323865	0.00033936	0.00176645
ENSDARG00000099466	si:dkey-190j3.2	4.99306758	5.536099736	1.5451099	3.58298122	0.0003397	0.00176782
ENSDARG00000094857	dio2	48.3249635	-1.388110565	0.38745922	-3.58259789	0.00034019	0.00177005
ENSDARG00000093150	fndc7rs3	136.08968	-0.925596973	0.25849311	-3.58074132	0.00034262	0.0017823
ENSDARG00000045822	tnnt2e	2960.83482	0.602962742	0.16841788	3.58015864	0.00034339	0.00178591
ENSDARG00000071724	ankha	124.176154	1.164352173	0.32524481	3.57992545	0.00034369	0.00178713
ENSDARG00000009607	ccnc	1238.14047	-0.390713286	0.10914364	-3.57980811	0.00034385	0.00178756
ENSDARG00000098700	il1b	91.544699	3.819425793	1.06714868	3.57909434	0.00034479	0.00179207
ENSDARG00000102986	csf1ra	882.32306	0.721902876	0.2017234	3.57867685	0.00034534	0.00179456
ENSDARG00000018765	pknox1.1	909.211037	-0.497328365	0.13897988	-3.57841993	0.00034568	0.00179595
ENSDARG00000074225	si:ch211-269e2.1	1088.77034	-0.436688479	0.12204182	-3.57818736	0.00034599	0.00179717
ENSDARG00000073859	kif13bb	941.508047	0.483428519	0.13512058	3.57775635	0.00034656	0.00179976
ENSDARG00000075017	myzap	806.514097	0.614618574	0.17179189	3.57769265	0.00034664	0.00179982
ENSDARG00000004964	cyp4t8	193.390127	-0.918520122	0.25674145	-3.57760738	0.00034675	0.00180004
ENSDARG00000007320	rpl7	46801.3263	0.375611059	0.10500552	3.57706001	0.00034748	0.00180343
ENSDARG00000014488	ca2	2188.58148	0.56407655	0.15769637	3.57697876	0.00034759	0.00180361
ENSDARG00000067805	ggcx	1245.14795	0.412371724	0.11529173	3.57676765	0.00034787	0.00180469
ENSDARG00000089124	hbae1.3	2206.87429	0.66227669	0.18517107	3.57656683	0.00034814	0.0018057
ENSDARG00000030756	dnmt1	4078.57638	-0.574397979	0.16070124	-3.57432211	0.00035114	0.00182088
ENSDARG00000102891	ppp1r26	827.106161	-0.449885561	0.1258735	-3.5741087	0.00035142	0.00182199
ENSDARG00000037186	tpbgb	475.750352	0.487023723	0.13626765	3.57402317	0.00035154	0.00182221
ENSDARG00000000906	skap2	99.6605514	0.859243118	0.24041978	3.5739286	0.00035167	0.00182224
ENSDARG00000102300	ca9	191.868431	0.648758961	0.18152642	3.57390931	0.00035169	0.00182224
ENSDARG00000101977	si:ch211-209n20.5	8.06089421	4.022601471	1.12611243	3.57211356	0.00035411	0.0018344
ENSDARG00000023967	atp6v1c1a	2750.60573	-0.336422886	0.09420276	-3.57126364	0.00035526	0.00183991
ENSDARG00000076270	adamts13	438.944769	0.701479149	0.1964257	3.57121884	0.00035532	0.00183991
ENSDARG00000111716	si:ch211-162i8.5	44.154537	1.372486853	0.38437418	3.57070514	0.00035602	0.00184314
ENSDARG00000010083	rbfox3a	2072.92082	-0.331732244	0.09291309	-3.57034998	0.00035651	0.00184526
ENSDARG00000117734	CABZ01044023.1	5.87188109	5.872509197	1.64506343	3.56977677	0.00035729	0.00184853
ENSDARG00000051816	aass	1805.57599	0.55459544	0.1553574	3.56980393	0.00035725	0.00184853
ENSDARG00000011555	spag7	2569.37796	-0.333656276	0.09348717	-3.5690061	0.00035834	0.00185357
ENSDARG00000075803	slc41a2a	682.939713	0.638297215	0.17884715	3.56895385	0.00035841	0.00185357
ENSDARG00000012073	kif15	1302.10457	-0.654821547	0.18350804	-3.56835346	0.00035923	0.00185744
ENSDARG00000013397	bnip2	879.401285	0.468231525	0.13122258	3.56822373	0.00035941	0.00185797
ENSDARG00000114067	tspan17	527.16057	0.697802438	0.19556855	3.56807077	0.00035962	0.00185867
ENSDARG00000077383	anxa11a	4579.75322	0.367185819	0.10291506	3.56785304	0.00035992	0.00185983
ENSDARG00000001721	oc90	164.236131	2.491490231	0.69834903	3.56768625	0.00036015	0.00186062
ENSDARG00000021399	yipf2	1015.08414	-0.377806267	0.10590099	-3.56754243	0.00036035	0.00186126
ENSDARG00000075825	dlec1	142.488781	0.709883566	0.19902397	3.56682452	0.00036133	0.00186598

Table S2. DEGs of WT vs. *terfa-/-*

ENSDARG00000019530	hdgfl2	1719.00179	-0.393613439	0.11038535	-3.56581242	0.00036273	0.0018728
ENSDARG00000062231	znf362b	576.521428	-0.589094049	0.16527318	-3.56436561	0.00036474	0.00188277
ENSDARG00000017983	rsph14	35.1585271	1.451012372	0.40710618	3.56421109	0.00036495	0.00188349
ENSDARG00000087911	psme4a	400.7528	-0.480300634	0.13478525	-3.56345092	0.00036601	0.00188856
ENSDARG00000033172	nr2f5	1750.98336	0.446575831	0.12532357	3.5633825	0.00036611	0.00188866
ENSDARG00000079361	abcg2b	55.4682986	1.362649352	0.38242931	3.56314047	0.00036644	0.00189001
ENSDARG00000095013	si:dkey-240n22.3	114.666041	1.008653321	0.28310497	3.56282449	0.00036689	0.0018919
ENSDARG00000062058	slc12a7b	5554.46067	0.361143358	0.10137677	3.5623876	0.0003675	0.00189466
ENSDARG00000055588	stat5b	859.641285	0.574997262	0.16141998	3.56211947	0.00036787	0.00189547
ENSDARG00000019521	mpx	623.517871	0.527950376	0.14821273	3.5621123	0.00036788	0.00189547
ENSDARG00000071554	CU929149.1	7.33795821	4.213987597	1.18300103	3.56211661	0.00036788	0.00189547
ENSDARG00000031848	pparg	323.990122	0.727549653	0.20431399	3.56093903	0.00036953	0.00190356
ENSDARG00000078736	doc2a	147.475518	-1.190753613	0.33444265	-3.56041195	0.00037027	0.00190699
ENSDARG00000103814	CR450808.4	33.8176403	2.431077122	0.6828286	3.56030359	0.00037043	0.00190739
ENSDARG00000052118	adamtsl5	478.823148	0.628598262	0.17661481	3.55914808	0.00037206	0.0019154
ENSDARG00000042747	coasy	973.880158	0.361522959	0.10157838	3.55905412	0.00037219	0.00191569
ENSDARG00000097170	pcdh1a4	110.533749	-0.911176583	0.25602731	-3.55890388	0.00037241	0.00191639
ENSDARG00000044669	hp1bp3	9087.74953	-0.483347094	0.13585344	-3.55785676	0.00037389	0.00192364
ENSDARG00000076659	cdca7b	918.134621	-0.575552681	0.16180999	-3.55696634	0.00037516	0.00192977
ENSDARG00000058593	sri	2559.14677	-0.35119826	0.0987433	-3.55667948	0.00037557	0.00193068
ENSDARG00000079571	mfsd4b	238.61577	-0.609857747	0.1714681	-3.55668342	0.00037557	0.00193068
ENSDARG00000071579	urad	100.980976	-1.164251535	0.32733835	-3.55672213	0.00037551	0.00193068
ENSDARG00000056801	sufu	1469.39615	0.336634395	0.09466	3.55624751	0.00037619	0.00193346
ENSDARG00000056781	zanl	1296.26548	1.841526296	0.51787276	3.55594355	0.00037663	0.00193529
ENSDARG00000060948	dync1i1	1095.34124	-0.543781807	0.15293095	-3.55573427	0.00037693	0.00193643
ENSDARG00000013224	phc2b	1667.75821	-0.576669473	0.16219613	-3.55538365	0.00037743	0.00193862
ENSDARG00000015889	zc3h15	2198.12073	0.350129344	0.09848796	3.5550473	0.00037791	0.0019407
ENSDARG00000061899	iqub	75.1363129	1.069314591	0.30084081	3.55442001	0.00037881	0.00194493
ENSDARG00000020041	vps35l	1450.37618	0.362072405	0.10187162	3.55420303	0.00037913	0.00194614
ENSDARG00000056080	si:dkey-191g9.5	658.193059	-0.801051415	0.22539987	-3.55391254	0.00037955	0.00194788
ENSDARG00000062174	cacnb4b	3367.36441	-0.457124828	0.12865994	-3.55296952	0.00038091	0.00195448
ENSDARG00000089009	frmpd1b	2589.11548	-0.502065858	0.14131372	-3.55284585	0.00038109	0.00195499
ENSDARG00000097832	CR382327.3	119.231828	0.826775392	0.23273564	3.55242283	0.0003817	0.00195758
ENSDARG00000018971	b3gnt5a	1261.68673	0.446897678	0.12580202	3.55238885	0.00038175	0.00195758
ENSDARG00000008363	mcl1b	1991.68135	0.752577852	0.2118834	3.55184908	0.00038253	0.0019612
ENSDARG00000073998	si:ch211-23l10.3	124.103131	-0.801328045	0.22566271	-3.55099902	0.00038377	0.00196714
ENSDARG00000076586	csf2rb	38.552358	1.510107543	0.42527962	3.55085799	0.00038398	0.00196779
ENSDARG00000071709	ppp1r9bb	1404.54527	-0.555404315	0.1564185	-3.55075849	0.00038412	0.00196812
ENSDARG00000098477	tec	69.3992893	0.973949537	0.27431684	3.55045481	0.00038457	0.00196999
ENSDARG00000061629	ndufaf5	848.446098	0.453920905	0.12787725	3.54966112	0.00038573	0.00197553
ENSDARG00000030914	tmem120a	209.248177	-0.735453676	0.20720833	-3.54934424	0.00038619	0.0019775
ENSDARG00000032426	coq8ab	147.4903	0.774499742	0.21824572	3.54875109	0.00038706	0.00198155
ENSDARG00000089304	si:dkeyp-4f2.1	91.4207915	-1.082493614	0.30505503	-3.54851915	0.0003874	0.0019827
ENSDARG00000057138	zgc:174164	335.130222	0.602053862	0.16966479	3.54849035	0.00038745	0.0019827
ENSDARG00000097762	CU693494.3	57.3677542	1.802169448	0.50794986	3.54792784	0.00038828	0.00198653
ENSDARG00000093629	si:ch211-106h4.4	31.0128239	1.66842482	0.47031646	3.54745145	0.00038898	0.00198972
ENSDARG00000010785	thbs1b	14007.9384	0.371755593	0.10480214	3.54721367	0.00038933	0.0019911
ENSDARG00000004094	acsbg2	3891.59079	-0.362737028	0.10226515	-3.54702469	0.00038961	0.00199212
ENSDARG00000078983	fam118b	478.871935	0.459420403	0.12953396	3.54671771	0.00039006	0.00199404
ENSDARG00000056314	a2ml	10603.2614	-0.453065667	0.12775251	-3.54643268	0.00039048	0.00199578
ENSDARG00000016830	rimkla	8736.18637	-0.381976837	0.10771515	-3.54617569	0.00039087	0.00199732
ENSDARG00000102115	ndufc2	1919.65464	0.415230512	0.1171326	3.544961	0.00039267	0.00200613
ENSDARG00000023683	cacna1fb	797.863559	-1.28846306	0.36348575	-3.54474165	0.000393	0.00200739
ENSDARG00000036375	cyfip2	6311.4018	-0.4575239	0.12908143	-3.54445963	0.00039342	0.00200913
ENSDARG00000041363	dctn3	1376.73481	-0.41764102	0.117853	-3.54374544	0.00039449	0.00201416
ENSDARG00000093671	BX001030.1	739.442033	0.891819793	0.25168448	3.54340398	0.000395	0.00201636
ENSDARG00000035682	FP102018.1	1606.67037	0.356702265	0.1006734	3.54316307	0.00039536	0.00201779
ENSDARG00000014496	trpv6	1602.52055	-0.703516373	0.19864461	-3.54158293	0.00039773	0.0020295

Table S2. DEGs of WT vs. *terfa-/-*

ENSDARG00000068305	mrps14	828.759036	0.489544826	0.13823152	3.54148481	0.00039788	0.00202964
ENSDARG00000070043	dars1	5683.79031	0.344947747	0.09740282	3.54145538	0.00039793	0.00202964
ENSDARG00000099646	pcdh1g3	94.9592502	-0.990394683	0.27967348	-3.54125346	0.00039823	0.00203078
ENSDARG00000061409	bivm	388.36505	-0.563268044	0.15909142	-3.54053055	0.00039932	0.00203552
ENSDARG00000070145	magi1a	2985.6101	-0.626895289	0.17706026	-3.5405759	0.00039926	0.00203552
ENSDARG00000043493	cltca	13520.8534	0.355279644	0.10036247	3.53996527	0.00040018	0.00203946
ENSDARG00000057529	itpa	323.266064	0.697231009	0.19697951	3.5396118	0.00040072	0.00204178
ENSDARG00000073974	colgalt1	459.988158	0.559949938	0.15821052	3.5392713	0.00040123	0.002044
ENSDARG00000041295	lim2.5	361.012981	-0.891707699	0.25200486	-3.5384544	0.00040248	0.00204991
ENSDARG00000062476	snapc5	594.114079	-0.478446051	0.13522246	-3.53821428	0.00040284	0.00205136
ENSDARG00000020258	ewsr1a	14623.8248	-0.569051257	0.16085517	-3.53766223	0.00040369	0.00205523
ENSDARG00000115174	im:6904045	114.537857	0.745368424	0.21071669	3.53730137	0.00040424	0.00205762
ENSDARG00000067646	tmem232	11.0201999	2.582565859	0.73018216	3.53687888	0.00040489	0.00206049
ENSDARG00000052644	ca10a	696.229386	-0.448652592	0.12685642	-3.53669611	0.00040517	0.00206149
ENSDARG00000020976	ubald1b	1088.73372	0.490490386	0.13868899	3.53662107	0.00040528	0.00206166
ENSDARG00000096777	si:ch73-81k8.2	21.2344148	1.817395422	0.51390312	3.53645536	0.00040554	0.00206253
ENSDARG00000055036	itih3a	626.075109	0.643499137	0.18204984	3.53474154	0.00040817	0.00207553
ENSDARG00000078050	phf19	319.424463	-0.593763152	0.16798591	-3.53460085	0.00040839	0.00207621
ENSDARG00000035560	capga	21.1902396	1.761878008	0.49849451	3.53439801	0.00040871	0.00207708
ENSDARG00000003469	neurod4	1767.69711	-1.708934695	0.48351739	-3.53438101	0.00040873	0.00207708
ENSDARG00000099189	CU856516.1	146.814251	0.815411436	0.2307157	3.53426942	0.0004089	0.00207754
ENSDARG00000111506	BX470259.1	188.553595	-0.81265374	0.22994741	-3.53408515	0.00040919	0.00207856
ENSDARG00000098994	CR352263.1	30.1714318	1.699498266	0.48089608	3.53402393	0.00040928	0.00207862
ENSDARG00000008818	hsf1	1034.75481	-0.452691454	0.12809885	-3.53392277	0.00040944	0.00207899
ENSDARG00000042236	atrx	11943.1712	-0.382908191	0.1083564	-3.53378459	0.00040966	0.00207965
ENSDARG00000016494	ddc	314.493548	0.54415195	0.15400964	3.53323306	0.00041051	0.00208357
ENSDARG00000020371	g6pc3	232.391194	0.646951671	0.18318639	3.53165789	0.00041296	0.00209559
ENSDARG00000060695	znf346	2246.88815	0.530608056	0.15024864	3.53153312	0.00041316	0.00209615
ENSDARG00000094043	si:ch73-376l24.3	257.387061	-0.770504913	0.21819455	-3.5312748	0.00041356	0.00209777
ENSDARG00000088475	pcdh1gb9	131.287833	-0.947933268	0.26846191	-3.53097863	0.00041403	0.00209969
ENSDARG00000095922	CR855300.1	193.388964	0.731461937	0.20717629	3.53062565	0.00041458	0.00210207
ENSDARG00000099880	sp6	206.072176	0.674948873	0.19118538	3.53033726	0.00041503	0.00210393
ENSDARG00000076765	ddhd2	622.854303	-0.477311793	0.13521091	-3.53012774	0.00041536	0.00210517
ENSDARG00000070581	ggact.1	109.041587	0.983406669	0.27858201	3.5300437	0.00041549	0.00210541
ENSDARG00000028900	odf2a	308.864272	-0.568698836	0.16115199	-3.52895953	0.0004172	0.00211362
ENSDARG00000019335	hes6	957.792131	-0.665977093	0.18875306	-3.52829836	0.00041824	0.00211848
ENSDARG00000101389	zgc:174180	10.6672883	2.818454649	0.79889114	3.52795832	0.00041878	0.00212077
ENSDARG00000036442	atp11c	1282.83156	0.48396975	0.13718399	3.52788796	0.00041889	0.0021209
ENSDARG00000043907	fgf11b	529.069741	-0.609394195	0.17275935	-3.52741666	0.00041964	0.00212425
ENSDARG00000071082	p4ha1b	903.680084	0.815584685	0.23121841	3.52733461	0.00041977	0.00212447
ENSDARG00000053318	lrrc6	72.8472953	0.936046805	0.26541756	3.52669506	0.00042078	0.00212918
ENSDARG00000098537	spata22	362.957894	-0.777244034	0.22040117	-3.52649685	0.0004211	0.00213034
ENSDARG00000076623	col14a1b	10433.3114	0.516917755	0.14658853	3.52631784	0.00042138	0.00213099
ENSDARG00000077116	ccnjl	945.459133	-0.543091473	0.15401135	-3.52630815	0.0004214	0.00213099
ENSDARG00000112931	ndufaf3	1553.26553	0.452388127	0.12830454	3.52589338	0.00042206	0.00213389
ENSDARG00000006299	arhgef7a	1683.30836	-0.428105955	0.12142305	-3.52573891	0.0004223	0.00213471
ENSDARG00000043973	znf292b	2504.64062	-0.458477316	0.13004041	-3.52565253	0.00042244	0.00213497
ENSDARG00000041417	atp6ap1a	3681.39133	-0.470971507	0.13359276	-3.52542701	0.0004228	0.00213635
ENSDARG00000008765	tmed5	664.689084	0.517291501	0.14675297	3.52491338	0.00042362	0.00214002
ENSDARG00000040966	zgc:113149	692.493825	-0.571513643	0.16213778	-3.52486424	0.0004237	0.00214002
ENSDARG00000070734	dyrk4	877.897503	-0.557796465	0.15828473	-3.52400689	0.00042507	0.00214653
ENSDARG00000089549	baalcb	179.501685	-0.788338353	0.22375099	-3.52328435	0.00042623	0.00215195
ENSDARG00000088301	si:dkey-254e13.6	18.7957427	2.132111473	0.60518498	3.52307399	0.00042657	0.00215305
ENSDARG00000095688	zgc:162509	9.18173784	4.787481358	1.35890577	3.52304145	0.00042663	0.00215305
ENSDARG00000098175	dgkab	102.304072	-1.086512491	0.30848673	-3.52207207	0.00042819	0.0021605
ENSDARG00000002945	bgnb	560.874857	0.794398407	0.2255653	3.52181129	0.00042861	0.00216218
ENSDARG00000009372	diras1b	98.3968702	-1.258907074	0.35755957	-3.52083175	0.0004302	0.00216975
ENSDARG00000077791	brsk2b	1732.4891	-0.544239375	0.15460433	-3.52020779	0.00043121	0.00217442

Table S2. DEGs of WT vs. *terfa-/-*

ENSDARG00000117295	AL929210.2	90.3982844	1.078002605	0.30629299	3.51951441	0.00043234	0.00217967
ENSDARG00000113380	BX510922.3	76.9870206	-0.962960467	0.27362431	-3.51927968	0.00043272	0.00218115
ENSDARG00000008772	cacng1a	919.520076	0.601380727	0.17094188	3.51804214	0.00043474	0.00219091
ENSDARG00000006092	rad21a	11591.1828	-0.497107664	0.14133866	-3.5171386	0.00043623	0.00219753
ENSDARG00000008502	pno1	2263.98097	-0.395917286	0.1125699	-3.51707962	0.00043632	0.00219753
ENSDARG00000101536	rpp25a	71.0987426	-1.150645895	0.32715767	-3.51709896	0.00043629	0.00219753
ENSDARG00000075041	fmnl2b	2520.94379	-0.589480544	0.16762642	-3.51663258	0.00043706	0.00220079
ENSDARG00000017355	rsph9	64.1679368	1.111151788	0.31598707	3.51644702	0.00043736	0.00220169
ENSDARG00000020946	auts2b	837.859542	-0.475393109	0.13519252	-3.51641586	0.00043742	0.00220169
ENSDARG00000108005	CABZ01053213.1	18.2163595	-2.479392622	0.70514986	-3.51612157	0.0004379	0.00220369
ENSDARG00000025034	syngr3b	957.380201	-0.47581365	0.1353646	-3.51505221	0.00043967	0.00221213
ENSDARG00000044011	xkrx	1171.58351	0.402859168	0.11462248	3.5146612	0.00044032	0.00221494
ENSDARG00000007406	phox2a	1046.318	-0.44185801	0.1257294	-3.5143571	0.00044082	0.00221703
ENSDARG00000086305	si:ch211-63b16.4	80.7530729	-1.086226339	0.30909543	-3.51421024	0.00044106	0.00221781
ENSDARG00000052703	cisd2	268.439903	-0.598567215	0.17033813	-3.51399425	0.00044142	0.00221917
ENSDARG00000008447	fkbp4	3576.37343	0.302909592	0.08622481	3.51302137	0.00044304	0.00222686
ENSDARG00000093761	si:ch211-250k18.7	15.8153048	2.585226542	0.7359195	3.51292029	0.00044321	0.00222725
ENSDARG00000056339	stk31	32.6720527	-1.401577263	0.39903739	-3.51239583	0.00044409	0.0022312
ENSDARG00000035809	col1a1b	106648.106	0.384303422	0.10942772	3.5119384	0.00044485	0.0022346
ENSDARG00000078077	dph2	350.395957	0.481167281	0.13702519	3.51152416	0.00044555	0.00223763
ENSDARG00000074852	myo15b	614.546639	0.698717597	0.19904874	3.51028398	0.00044763	0.00224764
ENSDARG00000045486	pawr	955.560622	0.373671356	0.106453	3.51020024	0.00044777	0.00224789
ENSDARG00000020292	TAFA3	332.234224	-0.555837345	0.15835569	-3.51005605	0.00044801	0.00224866
ENSDARG00000060054	epc1b	2496.52353	-0.364631274	0.10388577	-3.50992499	0.00044823	0.00224931
ENSDARG00000042948	dnajc5aa	1246.82115	-0.444932563	0.12684298	-3.50774303	0.00045193	0.00226738
ENSDARG00000022187	usp6nl	1010.44156	-0.401125514	0.11436093	-3.50753977	0.00045227	0.00226866
ENSDARG00000003084	spire2	258.626416	-0.640571156	0.18263822	-3.50732252	0.00045264	0.00227005
ENSDARG00000005397	trim3b	1890.04634	-0.431512915	0.12304354	-3.50699375	0.0004532	0.0022724
ENSDARG00000090714	gaa	46.6439562	1.24184974	0.3541577	3.50648801	0.00045406	0.00227626
ENSDARG00000101611	HUS1B	269.403491	-0.662442516	0.18896006	-3.50572762	0.00045536	0.00228231
ENSDARG00000061177	mov10b.1	257.719337	0.662767407	0.18908044	3.50521401	0.00045624	0.00228626
ENSDARG00000088247	si:ch1073-303k11.	704.050337	0.587299772	0.16755799	3.50505379	0.00045652	0.00228718
ENSDARG00000045589	kcnj8	111.393347	0.835753489	0.23848712	3.50439672	0.00045764	0.00229237
ENSDARG00000074308	lrrc75ba	354.438884	-0.669397969	0.19105067	-3.50377196	0.00045872	0.00229729
ENSDARG00000078854	tusc3	1382.47065	-0.470902842	0.13441602	-3.50332393	0.00045949	0.00230069
ENSDARG00000002546	rundc3ab	1485.38996	-0.466934762	0.13328716	-3.50322395	0.00045966	0.00230109
ENSDARG00000016088	rtn2a	1133.31283	0.507084572	0.14475241	3.50311674	0.00045985	0.00230155
ENSDARG00000101979	eps8l3a	563.13369	0.671828643	0.19183417	3.50213228	0.00046155	0.00230961
ENSDARG00000005154	aspa	132.675365	0.876245672	0.25021878	3.50191816	0.00046192	0.00231054
ENSDARG00000036161	hnrnpa0l	20781.6255	-0.473576109	0.13523219	-3.50194806	0.00046187	0.00231054
ENSDARG00000068365	lmx1bb	688.747604	0.435771426	0.12446255	3.50122527	0.00046312	0.00231609
ENSDARG00000055561	c1galt1b	868.730242	0.524124165	0.14972054	3.50068318	0.00046407	0.00232034
ENSDARG00000093042	NA	4.74257565	-6.50350753	1.85792984	-3.50040534	0.00046455	0.00232229
ENSDARG00000002377	mief2	559.431352	0.451401943	0.12896205	3.50026973	0.00046479	0.002323
ENSDARG00000074902	nol4la	452.583695	-0.737553695	0.21071887	-3.50017861	0.00046495	0.00232318
ENSDARG00000004931	slc8a3	401.213546	0.696047106	0.19886249	3.50014284	0.00046501	0.00232318
ENSDARG00000088463	CLPB	281.356096	0.672537204	0.19214966	3.50006971	0.00046514	0.00232335
ENSDARG00000099144	igfbp3	1287.44387	0.507040909	0.14490393	3.49915217	0.00046674	0.00233089
ENSDARG00000077414	zgc:194312	224.212744	-0.6529568	0.18661809	-3.49889332	0.00046719	0.00233268
ENSDARG00000092510	CR405715.1	8.60191068	-4.410774703	1.26078359	-3.49843917	0.00046799	0.00233619
ENSDARG00000025094	edem1	1372.76743	0.522074719	0.14923825	3.49826353	0.0004683	0.00233711
ENSDARG00000054584	FO681288.1	117.40804	-0.805837314	0.23035591	-3.49822717	0.00046836	0.00233711
ENSDARG00000056206	camk2g2	5274.19486	-0.333747945	0.09541066	-3.49801539	0.00046873	0.0023385
ENSDARG00000105317	si:ch1073-365p7.2	14.2517335	-2.539080257	0.72594666	-3.4976127	0.00046944	0.00234156
ENSDARG00000040352	crot	1362.94943	-0.391841431	0.11205912	-3.49673838	0.00047098	0.00234878
ENSDARG00000053404	PPP2CA	4311.0287	0.369825611	0.10578129	3.49613442	0.00047205	0.00235363
ENSDARG00000080484	dre-mir-21-2	2.95676206	6.142857491	1.75726687	3.49568844	0.00047284	0.00235709
ENSDARG00000062082	hipk3b	1919.92697	0.653460378	0.18694908	3.49539228	0.00047337	0.00235923

Table S2. DEGs of WT vs. *terfa-/-*

ENSDARG00000105507	zgc:112234	33.2788355	-1.718512332	0.49184606	-3.49400444	0.00047583	0.00237106
ENSDARG00000053190	galnt16	683.813416	-0.474453259	0.13579524	-3.49388718	0.00047604	0.00237162
ENSDARG00000026799	suv39h1a	22.0037475	-1.757460007	0.5030759	-3.49342916	0.00047686	0.00237522
ENSDARG00000042357	cldnk	313.601753	-0.710354124	0.20335967	-3.49309242	0.00047746	0.00237774
ENSDARG00000044601	rtn4a	9585.16512	0.400838978	0.11476511	3.49269034	0.00047818	0.00238037
ENSDARG00000090833	igfbp6b	242.643518	1.112107142	0.31840989	3.49269036	0.00047818	0.00238037
ENSDARG00000104297	si:ch73-380l10.2	67.1605527	1.086727701	0.31116463	3.49245249	0.00047861	0.00238201
ENSDARG00000073799	zgc:194210	35.0340033	-1.808267664	0.51782663	-3.49203298	0.00047936	0.00238528
ENSDARG00000038695	elavl1a	7045.23626	-0.368861093	0.10565113	-3.49131232	0.00048065	0.00239124
ENSDARG00000099112	si:dkey-71l4.3	41.7561656	1.610891829	0.46141191	3.49122288	0.00048082	0.00239141
ENSDARG00000000142	tmem267	416.272688	-0.558199521	0.15988819	-3.49118666	0.00048088	0.00239141
ENSDARG00000087301	crygm2d14	7133.063	0.550779037	0.15779209	3.49053645	0.00048205	0.00239676
ENSDARG00000054911	tmsb	85.1690441	1.572808648	0.45059973	3.49047846	0.00048216	0.0023968
ENSDARG00000089871	jcada	1194.2021	-0.380910342	0.10916576	-3.48928392	0.00048432	0.00240706
ENSDARG00000038133	zgc:113411	134.65146	0.802056191	0.22988046	3.48901415	0.00048481	0.002409
ENSDARG00000111987	CABZ01068235.1	31.5709657	-1.658783288	0.47560424	-3.48773868	0.00048712	0.00242004
ENSDARG00000117742	FO904898.6	4.08364602	6.154524871	1.76470949	3.48755696	0.00048746	0.0024212
ENSDARG00000052708	tnni1b	619.073756	0.675436196	0.19367433	3.48748438	0.00048759	0.00242138
ENSDARG00000043898	timm10b	267.512532	-0.603605291	0.17308753	-3.48728351	0.00048795	0.00242271
ENSDARG00000100115	dnah1	250.983173	0.673604303	0.19318622	3.48681333	0.00048881	0.00242649
ENSDARG00000062837	spata13	2826.27799	0.363382464	0.10421844	3.48673858	0.00048895	0.00242668
ENSDARG00000106579	CABZ01060030.2	78.6616014	1.287278417	0.36921327	3.48654427	0.0004893	0.00242796
ENSDARG00000031952	mb	1048.12234	-0.474951586	0.13623554	-3.48625329	0.00048984	0.00243012
ENSDARG00000055575	pcolce2b	580.370862	0.614247913	0.17621361	3.48581417	0.00049064	0.00243363
ENSDARG00000104207	pcyt1bb	376.099095	0.614877212	0.17639871	3.48572392	0.00049081	0.00243396
ENSDARG00000015325	u2af1	2825.45832	-0.526926742	0.15118392	-3.48533593	0.00049152	0.00243701
ENSDARG00000070230	aldh1l2	1324.33422	0.734536243	0.21080116	3.48449817	0.00049306	0.00244393
ENSDARG00000101364	wfikkn1	8.81376379	3.527730301	1.01241499	3.48447064	0.00049311	0.00244393
ENSDARG00000019185	slain1a	390.099878	-0.684314923	0.19640298	-3.48423908	0.00049354	0.00244555
ENSDARG00000100534	irak4	278.587751	0.616690672	0.17700922	3.48394655	0.00049408	0.00244774
ENSDARG00000056797	ppp2r2ca	1317.59498	-0.507334281	0.14562311	-3.48388584	0.00049419	0.00244781
ENSDARG00000077523	tdrd7b	284.388841	-0.63326928	0.18177881	-3.48373546	0.00049447	0.0024485
ENSDARG00000116723	BX470259.3	69.3822088	-1.253047581	0.35968838	-3.48370323	0.00049453	0.0024485
ENSDARG00000011553	atp5pb	15691.5572	0.353619345	0.1015105	3.48357415	0.00049477	0.0024492
ENSDARG00000003475	sult1st5	63.7084739	1.419711139	0.40759925	3.48310536	0.00049563	0.002453
ENSDARG00000103485	card14	208.106696	0.760370388	0.21831291	3.48293825	0.00049594	0.00245404
ENSDARG00000068219	b3gnt2l	846.700841	0.49890385	0.14324468	3.48287869	0.00049605	0.0024541
ENSDARG00000044093	rpl13a	15966.9515	0.386349522	0.11093321	3.48272179	0.00049634	0.00245505
ENSDARG00000077570	cdh16	43.0583851	1.24480118	0.35749807	3.48198011	0.00049772	0.00246137
ENSDARG00000058835	si:ch73-212j7.3	309.982524	-0.625520831	0.17965747	-3.48174122	0.00049817	0.00246308
ENSDARG00000078073	nudt5	593.743982	-0.535244837	0.15378027	-3.48058204	0.00050033	0.00247327
ENSDARG00000005823	slc39a10	1772.56057	0.363593752	0.10447219	3.48029221	0.00050087	0.00247545
ENSDARG00000090948	si:ch73-29c22.1	63.2843725	1.192779365	0.34282159	3.47930061	0.00050272	0.00248414
ENSDARG00000117451	CR759887.4	5.26338691	5.755233341	1.65435704	3.47883389	0.0005036	0.00248797
ENSDARG00000073926	synrg	2048.57511	0.38806604	0.11156637	3.47834232	0.00050453	0.00249205
ENSDARG00000111386	CABZ01079178.1	45.2161156	1.379912916	0.3967278	3.47823602	0.00050473	0.00249254
ENSDARG00000076790	crygm2d16	3171.03652	0.563174121	0.16192446	3.47800524	0.00050516	0.00249419
ENSDARG00000017988	dse	1255.26134	0.391428616	0.11257145	3.47715712	0.00050676	0.0025016
ENSDARG00000056210	hspa1b	100.064335	1.117153695	0.32131066	3.47686475	0.00050731	0.00250383
ENSDARG00000056535	pla2g4f.1	232.116649	0.804560522	0.23140921	3.47678698	0.00050746	0.00250406
ENSDARG00000041359	zgc:161969	909.831097	-0.351243639	0.10103304	-3.4765225	0.00050796	0.00250603
ENSDARG00000077198	chst12b	20.8122887	-2.00945313	0.57802923	-3.47638669	0.00050822	0.0025068
ENSDARG00000075593	trim71	2596.99702	0.425672718	0.12246905	3.47575761	0.00050941	0.00251219
ENSDARG00000018997	cplx2l	3997.26854	-0.372089525	0.10707281	-3.4751076	0.00051065	0.00251779
ENSDARG00000099481	ttc8	544.734746	-0.576744248	0.16596854	-3.47502144	0.00051081	0.0025181
ENSDARG00000005392	slc5a5	25.0410351	1.809015734	0.52066226	3.47445141	0.0005119	0.00252295
ENSDARG00000028816	tmed3	1239.79625	0.402298751	0.11579584	3.47420721	0.00051237	0.00252475
ENSDARG00000079105	mhc2dab	9.28335667	5.007072534	1.4412864	3.47403024	0.0005127	0.00252592

Table S2. DEGs of WT vs. *terfa-/-*

ENSDARG00000110229	zgc:173702	18.0178915	2.544040725	0.73257515	3.47273687	0.00051518	0.00253698
ENSDARG00000044875	crygm2e	22.4868929	-1.721212076	0.49564881	-3.47264443	0.00051536	0.00253698
ENSDARG00000022841	ptk2ba	248.85835	0.980662548	0.28239083	3.47271387	0.00051522	0.00253698
ENSDARG00000099161	dnaaf4	42.3801411	1.433903555	0.41290967	3.47268095	0.00051529	0.00253698
ENSDARG00000075034	raver2	748.62578	-0.398867319	0.11487693	-3.47212739	0.00051635	0.00254137
ENSDARG00000061566	slc7a3b	437.150412	-0.515411182	0.14848265	-3.47118784	0.00051816	0.00254967
ENSDARG00000044387	cldn2	216.52159	-0.793014941	0.22845914	-3.47114563	0.00051824	0.00254967
ENSDARG00000086623	vav3a	190.053206	0.852133549	0.24551616	3.47078401	0.00051894	0.0025526
ENSDARG00000090255	phlpp2	171.741411	-0.919845189	0.26503286	-3.47068357	0.00051914	0.00255305
ENSDARG00000012485	aurka	533.252825	-0.754145318	0.21730716	-3.47041167	0.00051966	0.00255462
ENSDARG00000003526	psma5	2823.42901	-0.342559684	0.09870763	-3.47044774	0.00051959	0.00255462
ENSDARG00000094978	si:ch73-18b11.2	79.802331	1.084299227	0.3124662	3.47013286	0.0005202	0.00255617
ENSDARG00000007807	cyth3a	548.154623	-0.507510263	0.14625275	-3.47009031	0.00052028	0.00255617
ENSDARG00000071095	abi3bpb	3907.95551	-0.393082721	0.11327542	-3.47015013	0.00052017	0.00255617
ENSDARG00000037073	si:dkey-60a16.1	280.028659	-0.577831628	0.16652103	-3.47002194	0.00052042	0.00255631
ENSDARG00000061480	si:ch211-132b12.1	17.9605734	-2.073533515	0.59758133	-3.46987667	0.0005207	0.00255719
ENSDARG00000091595	LO018154.1	309.777387	-0.519818167	0.14981996	-3.46961895	0.0005212	0.00255914
ENSDARG00000074507	rmdn1	1099.72871	0.557893272	0.16085306	3.46834109	0.00052368	0.00257083
ENSDARG00000078324	si:ch211-263k4.2	1036.88673	0.593335555	0.17108916	3.46799043	0.00052437	0.00257329
ENSDARG00000101501	si:ch73-305o9.3	159.780093	-0.840154643	0.24226064	-3.46797826	0.00052439	0.00257329
ENSDARG00000103578	gtf2b	1338.98613	-0.422302023	0.12178404	-3.46763039	0.00052507	0.00257611
ENSDARG00000104348	fgf2	150.94381	0.90606738	0.2613818	3.46645165	0.00052738	0.00258692
ENSDARG00000099674	dicp3.1	47.8069301	1.096253131	0.31625674	3.46633922	0.0005276	0.0025875
ENSDARG00000043798	ms4a17a.1	18.3691963	2.208550393	0.63718292	3.46611677	0.00052803	0.00258862
ENSDARG00000002192	aspn	1076.86451	0.578902559	0.16701564	3.46615775	0.00052795	0.00258862
ENSDARG00000060036	aimp1a	2104.17699	0.387763845	0.11189	3.46558084	0.00052909	0.00259327
ENSDARG00000017757	pik3cg	369.17256	-0.530307275	0.1530349	-3.46527013	0.0005297	0.00259576
ENSDARG00000004015	adcyap1a	128.049513	-0.860182526	0.24823852	-3.4651453	0.00052995	0.00259609
ENSDARG00000103106	trappc9	208.763395	0.756625673	0.21835422	3.46512956	0.00052998	0.00259609
ENSDARG00000070019	taf15	10025.575	0.49156861	0.14186647	3.46500907	0.00053021	0.00259623
ENSDARG00000109164	CABZ01045617.1	14.6052126	2.119575202	0.61170037	3.46505461	0.00053012	0.00259623
ENSDARG00000035514	adam28	434.147114	0.493327471	0.14241169	3.46409397	0.00053202	0.00260405
ENSDARG00000056638	pir	453.784121	0.562277067	0.16231561	3.46409726	0.00053201	0.00260405
ENSDARG00000096905	si:ch73-23l24.1	599.671509	0.869067352	0.25091099	3.46364802	0.0005329	0.00260786
ENSDARG00000076255	znf1084	56.8246802	1.451267484	0.41903841	3.46332804	0.00053354	0.00261045
ENSDARG00000057884	zgc:114174	136.079813	0.876861883	0.25319491	3.46318925	0.00053381	0.00261128
ENSDARG00000058561	fbxo30a	449.74135	0.559634787	0.16162148	3.46262637	0.00053493	0.00261623
ENSDARG00000075111	als2a	317.224485	0.79844278	0.23063037	3.46200185	0.00053617	0.0026218
ENSDARG00000001785	irx2a	762.49336	-0.571168534	0.16499383	-3.46175698	0.00053666	0.00262367
ENSDARG00000099776	glula	9344.68374	-0.59524704	0.17196781	-3.46138651	0.0005374	0.00262642
ENSDARG00000027016	gbf1	2869.18858	0.413790854	0.11954542	3.46136935	0.00053744	0.00262642
ENSDARG00000086421	si:ch211-212k18.8	407.689544	0.681891214	0.19703928	3.46068672	0.0005388	0.00263134
ENSDARG00000087093	si:ch211-157c3.4	797.496631	0.710125946	0.20519997	3.46065336	0.00053887	0.00263134
ENSDARG00000038683	MFAP4	29.3152348	2.072550692	0.59887708	3.460728	0.00053872	0.00263134
ENSDARG00000079858	tmem163a	233.860977	-0.740779021	0.21405757	-3.46065331	0.00053887	0.00263134
ENSDARG00000052855	ctso	342.271668	0.663689993	0.19180757	3.46018673	0.0005398	0.00263497
ENSDARG00000102571	metap2b	5826.87392	0.296572567	0.08571023	3.46017684	0.00053982	0.00263497
ENSDARG00000053269	slc2a15b	467.3446	0.575958348	0.16647748	3.45967734	0.00054082	0.00263934
ENSDARG00000074030	myt1a	3628.1168	-0.672466191	0.19444972	-3.45830378	0.00054359	0.00265231
ENSDARG00000051989	tmem187	139.265098	0.777226386	0.2247564	3.45808347	0.00054403	0.00265396
ENSDARG00000073978	crabp2a	1827.63011	0.437807428	0.12662479	3.45751745	0.00054518	0.00265902
ENSDARG00000040177	rgs16	257.2289	-1.024975665	0.2964908	-3.45702348	0.00054618	0.00266338
ENSDARG00000059835	zfyve27	811.535517	0.431440456	0.12482117	3.45646871	0.0005473	0.00266834
ENSDARG00000075952	mbd2	308.463932	0.614454103	0.17777327	3.45639206	0.00054746	0.00266858
ENSDARG00000039497	ccdc85a	329.205603	-0.656739454	0.19002208	-3.45612188	0.00054801	0.00267073
ENSDARG00000058853	sash1b	672.410638	-0.44049999	0.12746113	-3.45595543	0.00054835	0.00267186
ENSDARG00000099493	CR847906.1	15.3580632	2.37957349	0.68858487	3.45574465	0.00054878	0.00267342
ENSDARG00000104768	akap17a	690.301647	-0.511136749	0.14794094	-3.45500537	0.00055028	0.00267971

Table S2. DEGs of WT vs. *terfa-/-*

ENSDARG00000100398	pax7a	586.462792	-0.632696855	0.18312365	-3.45502537	0.00055024	0.00267971
ENSDARG00000007195	grm2b	1316.32883	-0.593837777	0.17188622	-3.45483059	0.00055064	0.00268093
ENSDARG00000087717	tmprss5	126.73243	-0.827053024	0.23943272	-3.45421882	0.00055189	0.00268649
ENSDARG00000018399	rps6kb1a	781.089529	0.501214161	0.14512804	3.45359979	0.00055316	0.00269214
ENSDARG00000117378	CABZ01078989.1	25.7622552	1.559155842	0.45147043	3.45350598	0.00055335	0.00269254
ENSDARG00000034063	unm_sa911	205.858955	0.959620974	0.27791072	3.45298293	0.00055442	0.00269724
ENSDARG00000106245	CR931802.3	53.294878	-1.293361948	0.37461545	-3.45250566	0.00055541	0.00270149
ENSDARG00000061579	myo1cb	3945.02653	0.398330375	0.11538452	3.45219947	0.00055604	0.00270403
ENSDARG00000040838	dcun1d2a	1531.18881	0.354666243	0.10275416	3.45159998	0.00055727	0.00270951
ENSDARG00000037958	nosip	547.240292	-0.548452701	0.1589232	-3.45105505	0.0005584	0.00271446
ENSDARG00000099200	zgc:123103	12409.0347	0.419247583	0.121516	3.45014311	0.00056029	0.00272311
ENSDARG00000116586	fthl27	118.121918	1.012642535	0.29354047	3.4497544	0.0005611	0.0027265
ENSDARG00000010408	igsf9b	1284.6855	-0.737099124	0.21368292	-3.44949949	0.00056163	0.00272855
ENSDARG00000098334	exosc3	160.852057	-0.862632002	0.25015379	-3.44840669	0.0005639	0.00273907
ENSDARG00000104501	FP236812.1	59.0476443	-1.304381767	0.3782762	-3.44822588	0.00056428	0.0027393
ENSDARG00000101534	rab3db	118.373486	0.853316606	0.24746452	3.44823818	0.00056426	0.0027393
ENSDARG00000016263	zdhhc5a	2068.13582	-0.346485507	0.10048025	-3.44829459	0.00056414	0.0027393
ENSDARG00000003931	cndp2	1753.11326	0.362655248	0.10520434	3.44715117	0.00056653	0.00274937
ENSDARG00000033447	nxph1	1202.13827	-0.53459181	0.15508316	-3.4471299	0.00056658	0.00274937
ENSDARG00000074843	phldb2b	1654.9662	0.391467639	0.11357946	3.44664116	0.0005676	0.00275381
ENSDARG00000100558	slbp	888.920286	-0.748493131	0.21717541	-3.44649111	0.00056792	0.0027548
ENSDARG00000007824	camta1b	3745.58873	-0.457214768	0.13267358	-3.44616293	0.00056861	0.00275761
ENSDARG00000053517	EML5	1636.71708	-0.581909005	0.16886401	-3.4460214	0.00056891	0.00275851
ENSDARG00000092716	VASH1	425.146479	-0.51043082	0.14815922	-3.4451505	0.00057074	0.00276688
ENSDARG00000056744	ela2	1270.09368	-3.245613122	0.94259196	-3.44328539	0.00057469	0.00278549
ENSDARG00000089320	ccdc175	68.7942567	1.33402599	0.38744529	3.44313382	0.00057502	0.00278651
ENSDARG00000069441	lpar6b	191.551514	0.70029785	0.20341867	3.44264302	0.00057606	0.00279102
ENSDARG00000033757	ncaph2	526.952188	-0.587937854	0.17082102	-3.44183559	0.00057778	0.00279882
ENSDARG00000088145	atg4db	97.8150089	0.910423531	0.26452916	3.44167558	0.00057812	0.00279993
ENSDARG00000031203	commd1	668.047647	0.750983557	0.21824555	3.44100278	0.00057956	0.00280636
ENSDARG00000045705	meig1	120.416212	0.753118217	0.21895359	3.43962492	0.00058252	0.00281958
ENSDARG00000102308	ttc39c	1190.96208	-0.415828085	0.12089319	-3.43963193	0.00058251	0.00281958
ENSDARG00000071467	zbtb33	803.419213	-0.396307636	0.11523164	-3.43922579	0.00058338	0.00282319
ENSDARG00000038909	msl2a	873.797819	-0.409024506	0.1189322	-3.43914014	0.00058357	0.00282354
ENSDARG00000044053	vsnl1b	5305.16954	-0.410418805	0.11935046	-3.43877024	0.00058436	0.00282653
ENSDARG00000086848	atad3	1179.62155	-0.525638781	0.15285762	-3.43874768	0.00058441	0.00282653
ENSDARG00000109802	BX324003.2	9.57069123	-3.885837247	1.13005431	-3.43862877	0.00058467	0.00282722
ENSDARG00000091768	dclre1a	422.426236	-0.506093723	0.14723755	-3.4372598	0.00058763	0.002841
ENSDARG00000069583	zgc:114181	4796.76336	0.403702868	0.11745683	3.43703177	0.00058813	0.00284284
ENSDARG00000030340	guk1a	1875.94699	-0.473160975	0.13768473	-3.43655376	0.00058917	0.00284731
ENSDARG00000058327	neu3.3	51.3149988	1.516389909	0.44128901	3.43627387	0.00058977	0.0028497
ENSDARG00000055638	ankrd33aa	124.24813	-1.329405427	0.38692156	-3.43585259	0.00059069	0.00285358
ENSDARG00000098108	dusp2	543.639032	-0.503677676	0.14661484	-3.43538004	0.00059172	0.002858
ENSDARG00000062177	dcbld2	984.431104	0.521028341	0.15169178	3.43478293	0.00059303	0.0028632
ENSDARG00000091833	sv2	397.834164	0.638008855	0.18574855	3.43479868	0.00059299	0.0028632
ENSDARG00000055305	ret	1294.88666	-0.370826528	0.10796809	-3.43459368	0.00059344	0.00286464
ENSDARG00000105781	zgc:171727	42.8796649	1.491387019	0.43431581	3.43387686	0.00059501	0.00287167
ENSDARG00000007477	fbxo8	592.638239	0.500967668	0.14589674	3.433714	0.00059537	0.00287284
ENSDARG00000100405	BX957252.3	35.6503474	-1.81975924	0.52998588	-3.43359948	0.00059562	0.00287349
ENSDARG00000033707	jade1	1241.52435	-0.345355045	0.10060913	-3.43264121	0.00059773	0.00288311
ENSDARG00000090418	wdsub1	573.666199	0.494379431	0.14402611	3.43256818	0.00059789	0.00288332
ENSDARG00000032079	acsl3a	368.654926	0.681266673	0.1984919	3.43221402	0.00059868	0.00288653
ENSDARG00000040741	kcnab1b	255.121105	-0.913043962	0.26606388	-3.43167198	0.00059987	0.00289118
ENSDARG00000007597	bloc1s4	416.910063	-0.572121964	0.16671654	-3.43170499	0.0005998	0.00289118
ENSDARG00000001976	fkbp16	336.678641	-0.915616274	0.26682578	-3.43151347	0.00060022	0.00289231
ENSDARG00000117342	CU570781.3	136.132746	-0.70319064	0.20493255	-3.43132728	0.00060064	0.00289374
ENSDARG00000074967	zgc:162698	1311.57967	0.361695899	0.10545161	3.42997035	0.00060365	0.00290765
ENSDARG00000068749	si:ch211-250k18.5	20.3857974	2.126539313	0.61999663	3.42992078	0.00060376	0.00290765

Table S2. DEGs of WT vs. *terfa-/-*

ENSDARG00000045038	tekt3	89.584514	-1.148353933	0.3348369	-3.42959193	0.00060449	0.00291061
ENSDARG00000093224	CR456628.1	8.75850396	-4.246498292	1.23826203	-3.429402	0.00060491	0.00291209
ENSDARG00000077850	lgals3a	3707.95346	0.36477244	0.10637292	3.42918532	0.0006054	0.00291385
ENSDARG00000062361	paxbp1	1857.34044	-0.441585014	0.12881813	-3.42797253	0.00060811	0.00292633
ENSDARG00000002406	zmynd10	78.4134261	1.064925885	0.31078311	3.42658864	0.00061121	0.00294071
ENSDARG00000024306	zbed4	1187.78713	-0.433623867	0.12656498	-3.42609676	0.00061232	0.00294548
ENSDARG00000094249	wu:fc17b08	223.806809	-0.560792863	0.16370699	-3.42558897	0.00061347	0.00295042
ENSDARG00000078518	kazald2	43.0982671	1.320717823	0.38564103	3.42473367	0.0006154	0.00295915
ENSDARG00000042940	nab1a	217.627751	-0.68714819	0.20064811	-3.42464329	0.00061561	0.00295956
ENSDARG00000052741	card11	260.530162	0.778877136	0.22754362	3.42297951	0.00061939	0.00297658
ENSDARG00000041300	drc3	134.461178	0.703622778	0.20555555	3.42302978	0.00061927	0.00297658
ENSDARG00000060238	uacab	5995.44244	-0.343741732	0.1004362	-3.42248857	0.00062051	0.00298139
ENSDARG00000102810	samd1a	457.38685	-0.53559282	0.15655205	-3.42118057	0.0006235	0.0029946
ENSDARG00000020442	snx5	1520.15705	0.39062591	0.1141775	3.42121617	0.00062342	0.0029946
ENSDARG00000045167	dlgap5	1587.33788	-0.749096305	0.21897149	-3.42097641	0.00062397	0.00299627
ENSDARG00000028106	glrx	118.35618	1.063145223	0.31078634	3.42082354	0.00062432	0.00299738
ENSDARG00000090230	tmem108	648.49659	-0.486845491	0.14233473	-3.42042658	0.00062523	0.00300118
ENSDARG00000014239	esyt1b	1003.48432	0.445292176	0.13018885	3.42035562	0.00062539	0.00300138
ENSDARG00000004472	atat1	686.684812	-0.510049559	0.1491413	-3.4199083	0.00062642	0.00300574
ENSDARG00000110954	CABZ01043952.1	319.614791	0.604877987	0.17689743	3.41937119	0.00062766	0.0030111
ENSDARG00000101637	ccnd1	4655.71205	0.371044787	0.10851577	3.41927055	0.00062789	0.00301163
ENSDARG00000086336	si:ch211-157b11.1	231.883047	-0.613234381	0.17935442	-3.41912063	0.00062824	0.00301271
ENSDARG00000043257	ckbb	26100.943	-0.506600731	0.14818769	-3.41864246	0.00062934	0.00301743
ENSDARG00000006052	malt2	227.086094	0.706957418	0.20680526	3.41846927	0.00062974	0.00301877
ENSDARG00000078970	il7r	14.8245368	-2.891490803	0.8459761	-3.41793441	0.00063098	0.00302412
ENSDARG00000021480	olfml2bb	318.370047	0.760935308	0.22264155	3.41775968	0.00063139	0.00302546
ENSDARG00000004576	plk4	604.412779	-0.718758129	0.21030657	-3.41766839	0.0006316	0.00302546
ENSDARG00000029443	zgc:92242	1022.74664	0.526388243	0.15402024	3.41765631	0.00063163	0.00302546
ENSDARG00000081937	CR936321.3	4.52600372	5.426225204	1.58784607	3.41734965	0.00063234	0.00302814
ENSDARG00000092658	CR457445.1	14.6216047	3.260006131	0.95396843	3.41731028	0.00063243	0.00302814
ENSDARG00000053724	adcyap1r1a	668.501247	-0.467635736	0.13685423	-3.41703522	0.00063307	0.00303054
ENSDARG00000087666	fibpb	63.9172707	1.103050673	0.32281354	3.41699009	0.00063318	0.00303054
ENSDARG00000058731	slc2a6	50.0242435	1.509586082	0.44194427	3.41578385	0.00063599	0.00304341
ENSDARG00000043361	nck2b	617.16727	0.419979755	0.12296032	3.4155713	0.00063648	0.0030452
ENSDARG00000104097	tmem222b	112.500794	0.836557419	0.2449304	3.41549039	0.00063667	0.00304552
ENSDARG00000062830	tent5d	62.0110749	1.244409694	0.36437512	3.41518843	0.00063738	0.0030478
ENSDARG00000030723	cldn11b	482.182115	-0.474228333	0.13885889	-3.41518155	0.0006374	0.0030478
ENSDARG00000070843	arid3a	915.817101	0.593326322	0.17373679	3.41508738	0.00063762	0.00304827
ENSDARG00000054290	acin1a	10509.9819	-0.300394831	0.08796559	-3.41491301	0.00063802	0.00304963
ENSDARG00000098462	CU570782.1	12.9575952	2.501997243	0.73280499	3.41427432	0.00063952	0.00305586
ENSDARG00000091001	mycbp	101.293874	1.051272165	0.30790697	3.41425261	0.00063957	0.00305586
ENSDARG00000104280	bnip1b	459.449205	-0.486032954	0.14236388	-3.41401877	0.00064012	0.00305789
ENSDARG00000060868	mbtd1	1171.97192	-0.422114557	0.12364817	-3.41383574	0.00064055	0.00305936
ENSDARG00000117321	CZQB01066256.1	200.99717	-1.094034639	0.32054023	-3.41309616	0.00064229	0.00306709
ENSDARG00000053463	poglut1	280.325064	-0.576988296	0.16906566	-3.41280592	0.00064298	0.00306977
ENSDARG00000007916	xpnpep3	539.698744	0.494813819	0.14499435	3.41264215	0.00064336	0.00307102
ENSDARG00000018149	slc38a4	4194.81441	0.428746352	0.12564152	3.41245757	0.0006438	0.00307251
ENSDARG00000051880	kcnj11	530.453861	0.608024194	0.17818065	3.41240297	0.00064393	0.00307254
ENSDARG00000098696	rela	523.104139	0.585472096	0.17159541	3.41193341	0.00064504	0.00307724
ENSDARG00000102212	si:dkey-16p6.1	15.7097925	2.041916231	0.59853587	3.41151859	0.00064602	0.00308134
ENSDARG00000098849	SLC25A29	199.909962	-0.95671796	0.28048126	-3.41098708	0.00064728	0.00308676
ENSDARG00000002241	kcna2a	69.995268	-1.05439618	0.30914861	-3.41064502	0.00064809	0.00309004
ENSDARG00000014313	atp5pf	5134.48893	0.328909589	0.09646365	3.40967379	0.00065041	0.00310047
ENSDARG00000096654	si:dkey-119m7.8	214.565039	0.733646285	0.21517791	3.40948697	0.00065085	0.003102
ENSDARG00000025667	adgrb2	441.474151	-0.678954993	0.19914658	-3.40932283	0.00065124	0.00310327
ENSDARG00000077400	rbm10	922.288226	-0.572751225	0.1680271	-3.40868371	0.00065277	0.00310995
ENSDARG00000103239	si:dkey-14o6.8	28.3833951	-1.736782215	0.50953057	-3.40859279	0.00065299	0.00311039
ENSDARG00000093269	si:ch73-62b13.1	64.214804	-1.239726298	0.36378495	-3.40785479	0.00065476	0.00311822

Table S2. DEGs of WT vs. *terfa-/-*

ENSDARG00000075720	il2rb	28.2224724	1.55150601	0.45532138	3.40749652	0.00065562	0.00312097
ENSDARG00000104864	klhdc2	875.227851	0.527896387	0.15492383	3.40745756	0.00065571	0.00312097
ENSDARG00000104516	polr3d	430.078835	0.490174483	0.14385001	3.40753886	0.00065552	0.00312097
ENSDARG00000113721	HPDL	127.240207	1.079420839	0.316798	3.40728431	0.00065613	0.00312235
ENSDARG00000062801	erfl3	1747.89004	0.379225952	0.11130523	3.40708127	0.00065662	0.00312326
ENSDARG00000003153	nudt21	1440.48784	-0.435095456	0.12770198	-3.40711588	0.00065653	0.00312326
ENSDARG00000020218	amfra	2329.29631	0.289759961	0.08504721	3.40704836	0.0006567	0.00312326
ENSDARG00000105091	zgc:174944	23.3439625	1.999801874	0.58700002	3.40681738	0.00065725	0.00312531
ENSDARG00000010945	rapgef6	1568.57695	0.367226882	0.10780192	3.40649671	0.00065802	0.00312838
ENSDARG00000004806	grwd1	753.991273	0.462581421	0.13579677	3.40642424	0.0006582	0.00312842
ENSDARG00000024548	cryba4	14181.1209	-0.719260022	0.21115031	-3.40638874	0.00065828	0.00312842
ENSDARG00000039535	dapp1	51.5825464	1.530154754	0.44924589	3.40605179	0.0006591	0.00313169
ENSDARG00000076590	atad2	1659.91875	-0.545445144	0.16014908	-3.40585876	0.00065956	0.0031333
ENSDARG00000101239	hsd17b4	2618.65506	0.453100809	0.13304209	3.40569512	0.00065996	0.00313458
ENSDARG00000105420	CR361564.1	14.033744	-3.126938178	0.91817594	-3.40559804	0.00066019	0.0031351
ENSDARG00000038669	gbp2	135.865893	0.879722692	0.25836495	3.40496149	0.00066173	0.00314181
ENSDARG00000060397	hhip	2272.45044	0.354401209	0.10409491	3.40459691	0.00066262	0.00314541
ENSDARG00000115425	CABZ01038524.1	77.6888972	1.094338251	0.32144288	3.40445634	0.00066296	0.00314583
ENSDARG00000057571	pgam2	3693.22778	0.49821012	0.14634003	3.40446912	0.00066293	0.00314583
ENSDARG00000018814	esrp2	2241.97853	0.316426015	0.0929525	3.40416898	0.00066366	0.00314854
ENSDARG00000069397	zswim8	4924.96617	0.338807296	0.09953162	3.40401666	0.00066403	0.00314969
ENSDARG00000003952	pfn2	13558.838	0.315576793	0.09271715	3.40365072	0.00066492	0.00315331
ENSDARG00000034668	celf3a	4661.30018	-0.646368203	0.18993668	-3.40307205	0.00066633	0.0031594
ENSDARG00000036135	bbox1	587.068548	0.510372562	0.1499973	3.4025451	0.00066761	0.00316489
ENSDARG00000038668	gbp1	252.465432	0.964569169	0.28349927	3.4023691	0.00066804	0.00316572
ENSDARG00000096716	hmx3b	23.0292771	1.764939368	0.51873265	3.40240653	0.00066795	0.00316572
ENSDARG00000086815	MYADM	1270.89354	0.460326115	0.13531039	3.40200137	0.00066894	0.00316938
ENSDARG00000102802	brdt	1061.35903	-0.38872143	0.1142839	-3.40136639	0.0006705	0.00317565
ENSDARG00000100700	EPS15L1	125.161691	0.77124634	0.22674664	3.40135727	0.00067052	0.00317565
ENSDARG00000005500	dgcr6	366.377461	-0.489844877	0.14403024	-3.40098632	0.00067143	0.00317936
ENSDARG00000033231	mcm6l	22.9223464	2.09915519	0.61726597	3.40073049	0.00067206	0.00318173
ENSDARG00000056764	hydin	355.000013	0.791875094	0.23285811	3.40067653	0.00067219	0.00318175
ENSDARG00000019945	ptprdb	2239.69907	-0.660098	0.19412241	-3.40042148	0.00067282	0.00318237
ENSDARG00000095615	si:dkeyp-86h10.3	1204.55952	0.518996381	0.15262393	3.40049151	0.00067265	0.00318237
ENSDARG00000018073	mrps22	853.863224	-0.371046362	0.10911797	-3.40041469	0.00067284	0.00318237
ENSDARG00000110357	txndc15	803.633279	0.444740693	0.13078674	3.40050301	0.00067262	0.00318237
ENSDARG00000034893	rarab	1821.10857	-0.358139371	0.10532401	-3.40035826	0.00067298	0.00318242
ENSDARG00000001452	adam8a	268.076345	0.746322138	0.21952107	3.39977456	0.00067441	0.00318862
ENSDARG00000021789	myoc	2622.65398	0.605274245	0.17806188	3.39923532	0.00067575	0.00319431
ENSDARG00000009402	si:ch211-210b2.2	244.075647	0.756555464	0.22258199	3.39899681	0.00067634	0.00319648
ENSDARG00000075339	ipp	368.94625	0.563009432	0.16566674	3.39844585	0.0006777	0.00320232
ENSDARG00000086553	nlrc3l1	880.731264	0.450705018	0.13262648	3.39830349	0.00067805	0.00320338
ENSDARG00000002831	col4a4	376.395963	0.661238095	0.19460682	3.39781565	0.00067926	0.00320849
ENSDARG00000103165	AL929057.1	8.74139264	3.372818341	0.99293101	3.3968305	0.00068171	0.00321945
ENSDARG00000010316	qars1	2163.95225	0.40902597	0.12043116	3.39634663	0.00068292	0.00322453
ENSDARG00000117699	CABZ01074708.1	206.237414	0.661041711	0.19464901	3.3960703	0.00068361	0.00322718
ENSDARG00000115271	zgc:171772	28033.5464	0.325905137	0.09597287	3.39580471	0.00068427	0.0032297
ENSDARG00000014248	pdlim3b	395.150386	0.631664283	0.18609357	3.39433693	0.00068795	0.00324644
ENSDARG00000068989	gabra1	1352.65893	-0.535856484	0.15788135	-3.39404555	0.00068868	0.00324928
ENSDARG00000098825	farsa	2063.71075	0.401187496	0.11821439	3.39372807	0.00068948	0.00325243
ENSDARG00000087290	si:ch211-202h22.1	73.3367179	-1.057773073	0.31169748	-3.39358881	0.00068983	0.00325347
ENSDARG00000044327	tmem185	846.208743	-0.446037057	0.13146205	-3.3928959	0.00069158	0.00326109
ENSDARG00000057114	hpf1	698.743028	-0.602426854	0.17755897	-3.39282684	0.00069175	0.00326129
ENSDARG00000116727	CABZ01081294.2	199.629135	-1.012322714	0.29839215	-3.39259161	0.00069235	0.00326348
ENSDARG00000006832	galnt9	1109.59136	-0.436783609	0.12876172	-3.39218526	0.00069338	0.00326714
ENSDARG00000109646	si:ch211-153a8.4	277.756174	-0.724073959	0.21345386	-3.39218025	0.00069339	0.00326714
ENSDARG00000040045	cldn1	2206.92642	0.400893597	0.11821276	3.39128882	0.00069565	0.00327716
ENSDARG00000079752	col6a4a	10339.2857	0.391307468	0.11541411	3.39046464	0.00069774	0.00328641

Table S2. DEGs of WT vs. *terfa-/-*

ENSDARG00000076272	znf1041	187.399737	-0.667961218	0.19711673	-3.3886581	0.00070236	0.00330751
ENSDARG00000017111	fam8a1a	67.7218805	-1.053634929	0.3109906	-3.38799609	0.00070405	0.00331488
ENSDARG00000042045	hmg20b	1140.63325	-0.378621825	0.1117641	-3.38768722	0.00070485	0.00331798
ENSDARG00000088967	wnk4b	906.162812	0.417834855	0.12335194	3.38733929	0.00070574	0.00332156
ENSDARG00000068640	rsf1b.1	2334.98339	-0.402561054	0.11885438	-3.38701081	0.00070659	0.00332491
ENSDARG00000071374	lrrtm2	744.286744	-0.597561019	0.17643858	-3.38679335	0.00070715	0.00332692
ENSDARG00000013031	mta2	6040.06093	-0.519392254	0.15340561	-3.38574481	0.00070985	0.00333839
ENSDARG00000068456	tmem91	69.566466	-1.034687566	0.30559746	-3.38578586	0.00070975	0.00333839
ENSDARG00000077155	si:ch211-157p22.1	158.946422	-0.673553697	0.19894553	-3.38561861	0.00071018	0.00333929
ENSDARG00000056414	usp1	1281.97015	-0.512749202	0.15145149	-3.38556727	0.00071031	0.00333929
ENSDARG00000102463	cox18	438.421274	0.525036291	0.15509038	3.38535702	0.00071086	0.00334122
ENSDARG00000077239	si:ch211-165i18.2	10.9105808	2.316169252	0.68419581	3.38524325	0.00071115	0.00334134
ENSDARG00000099476	zgc:174863	368.457374	0.683505934	0.20190675	3.38525542	0.00071112	0.00334134
ENSDARG00000010792	cdc25b	698.664911	-0.617474966	0.1824241	-3.38483217	0.00071222	0.0033454
ENSDARG00000026766	bcl2l10	1729.86527	-0.42470505	0.12547456	-3.3847902	0.00071233	0.0033454
ENSDARG00000058830	zdhhc3b	912.679126	0.403317833	0.11915719	3.3847546	0.00071242	0.0033454
ENSDARG00000098662	si:ch73-359m17.9	254.905949	0.658817322	0.19465282	3.38457634	0.00071288	0.00334694
ENSDARG00000007869	ehd3	843.761647	-0.587294924	0.17355385	-3.38393482	0.00071455	0.00335387
ENSDARG00000097320	CR848791.3	5.23088579	4.819674163	1.42429374	3.38390463	0.00071463	0.00335387
ENSDARG00000067997	myhz1.3	108053.75	0.450525517	0.13316735	3.38315306	0.00071659	0.00336179
ENSDARG00000102310	si:ch211-156l18.8	237.644228	-1.198719271	0.35431929	-3.38316119	0.00071657	0.00336179
ENSDARG00000077288	si:ch211-188c16.1	31.8608751	1.492184861	0.44109457	3.38291371	0.00071721	0.00336401
ENSDARG00000036186	mbpa	800.245669	-1.307585603	0.38653168	-3.38286791	0.00071733	0.00336401
ENSDARG00000100157	tamm41	426.48007	0.439488802	0.12992361	3.38267078	0.00071785	0.00336579
ENSDARG00000061335	galnt1	1867.55235	0.288576438	0.08532055	3.38226185	0.00071892	0.00337017
ENSDARG00000058389	ccl19a.1	78.8168173	1.085952784	0.32111249	3.38184542	0.00072001	0.00337338
ENSDARG00000052027	tspan1	28.9273349	-2.036528041	0.60218576	-3.38189342	0.00071988	0.00337338
ENSDARG00000092810	stap2a	777.736314	-0.628967205	0.18597983	-3.38191079	0.00071984	0.00337338
ENSDARG00000000796	nr4a1	1920.70971	0.528224473	0.15623136	3.38103994	0.00072212	0.00338264
ENSDARG00000034396	mars1	2188.30475	0.39131579	0.11574561	3.38082625	0.00072268	0.00338464
ENSDARG00000092281	flnb	884.635284	0.540187471	0.15979694	3.38046202	0.00072364	0.00338849
ENSDARG00000037855	taf11	872.159943	-0.415691997	0.1229851	-3.38001908	0.00072481	0.00339331
ENSDARG00000077860	ankhd1	8351.14573	0.43072972	0.1274705	3.37905426	0.00072736	0.00340445
ENSDARG00000036424	pcdh20	438.004763	-0.605712388	0.17925709	-3.3790149	0.00072746	0.00340445
ENSDARG00000074675	pan2	1040.25588	-0.398929889	0.11806663	-3.37885382	0.00072789	0.0034058
ENSDARG00000097964	arhgef33	306.192652	0.545738262	0.16154636	3.37821455	0.00072958	0.00341309
ENSDARG00000079779	radil	342.31852	-0.639682996	0.18936132	-3.37810805	0.00072986	0.00341313
ENSDARG00000057173	ifit8	5.2358458	4.137199114	1.22470088	3.37813028	0.00072981	0.00341313
ENSDARG00000067549	adamts12	848.342209	0.536678921	0.15891964	3.37704602	0.00073269	0.00342569
ENSDARG00000033134	ndufaf7	338.500297	0.545391071	0.16150333	3.37696497	0.0007329	0.00342605
ENSDARG00000113332	CABZ01084501.2	379.899332	0.554748277	0.16429395	3.37655932	0.00073399	0.00343046
ENSDARG00000058366	si:dkey-222f8.3	756.668634	-0.475911739	0.14101219	-3.37496874	0.00073824	0.0034497
ENSDARG00000093618	si:dkey-45h7.1	13.9272057	-2.31460408	0.68588808	-3.37460899	0.00073921	0.00345333
ENSDARG00000014358	optc	60.4503664	1.120821744	0.33213708	3.37457577	0.0007393	0.00345333
ENSDARG00000042857	si:ch211-246m6.4	177.325728	0.600130125	0.17785042	3.37435312	0.00073989	0.00345548
ENSDARG00000044894	zgc:113307	502.49798	-0.522428289	0.15485666	-3.37362484	0.00074185	0.00346398
ENSDARG00000102483	numa1	1763.45477	-0.614328979	0.18210595	-3.37347012	0.00074227	0.00346528
ENSDARG00000076996	IPO13	371.677127	0.510511338	0.15134146	3.37324187	0.00074289	0.00346685
ENSDARG00000104834	ANO7	108.833583	-0.88247397	0.26160975	-3.37324573	0.00074288	0.00346685
ENSDARG00000060029	fam160b2	617.389258	0.470025621	0.13934437	3.37312247	0.00074321	0.0034677
ENSDARG00000007678	ttyh3b	6711.53465	-0.404325591	0.11987214	-3.37297395	0.00074361	0.00346892
ENSDARG00000053547	jakmip2	2597.0816	-0.515006362	0.15271363	-3.37236669	0.00074525	0.00347593
ENSDARG00000038910	pccb	3216.86964	0.382020641	0.11328714	3.37214495	0.00074585	0.00347808
ENSDARG00000016227	mhc1lba	4.07830063	5.758404814	1.70769966	3.37202434	0.00074618	0.00347895
ENSDARG00000011605	dennd6b	531.711256	-0.519737936	0.1541523	-3.3715874	0.00074736	0.00348382
ENSDARG00000015471	chd1l	566.256888	-0.528538793	0.1567749	-3.37132275	0.00074808	0.00348651
ENSDARG00000075758	ywhabb	9026.60327	-0.296902296	0.08808321	-3.37070236	0.00074977	0.00349372
ENSDARG00000006863	si:ch1073-349o24.	100.723595	0.832366529	0.24694887	3.37060267	0.00075004	0.00349433

Table S2. DEGs of WT vs. *terfa-/-*

ENSDARG00000103952	CABZ01054391.1	113.833615	0.917592703	0.27227253	3.37012584	0.00075134	0.00349972
ENSDARG00000015254	fzr1a	1953.30905	-0.359247033	0.10661299	-3.36963674	0.00075267	0.00350528
ENSDARG00000028793	nlk2	1605.931	-0.431075832	0.12794764	-3.3691582	0.00075398	0.00351072
ENSDARG00000014083	wdr82	1670.70617	-0.388073421	0.11518735	-3.36906287	0.00075424	0.00351127
ENSDARG00000113609	TMEM164	332.525239	0.713401145	0.21177075	3.36874255	0.00075512	0.0035147
ENSDARG00000035952	cdr2a	360.49533	-0.795830145	0.23626349	-3.36840088	0.00075606	0.0035184
ENSDARG00000011519	myl4	1492.13236	0.825798569	0.24518357	3.36808277	0.00075693	0.00352138
ENSDARG00000095204	AL935117.1	12.684127	-2.93160649	0.87041415	-3.36805931	0.00075699	0.00352138
ENSDARG00000007891	meox1	571.354983	0.460405698	0.13669952	3.3680125	0.00075712	0.00352138
ENSDARG00000036281	CC2D1A	960.091751	0.467037663	0.13869692	3.36732549	0.00075901	0.00352951
ENSDARG00000044541	ppp1r14ba	2147.8872	-0.669084961	0.19874675	-3.3665203	0.00076123	0.00353916
ENSDARG00000040251	ctsk	2233.25407	0.775970475	0.23058898	3.36516719	0.00076497	0.0035559
ENSDARG00000070039	NA	4.16005236	-6.310895824	1.87594695	-3.3641121	0.0007679	0.00356886
ENSDARG00000075555	adamts17	575.145452	0.54752359	0.16277795	3.36362253	0.00076927	0.00357452
ENSDARG00000098294	col5a3a	273.777563	0.650546074	0.1934209	3.36337007	0.00076997	0.00357683
ENSDARG00000032340	serhl	406.667197	0.571121127	0.16980764	3.36334169	0.00077005	0.00357683
ENSDARG00000096626	CU914144.1	36.9182732	-1.395788612	0.41502356	-3.36315517	0.00077057	0.00357857
ENSDARG00000103492	si:ch73-111k22.3	7.7890655	3.439908505	1.02304626	3.36241737	0.00077263	0.00358707
ENSDARG00000025218	myo5ab	1085.4891	0.429451439	0.12772179	3.36239771	0.00077269	0.00358707
ENSDARG00000020493	lgi1a	1742.37078	-0.496611621	0.14769959	-3.36230885	0.00077294	0.00358755
ENSDARG00000014946	angpt2a	46.8181495	1.152092096	0.34265572	3.36224385	0.00077312	0.00358772
ENSDARG00000095592	si:dkey-11o15.5	26.954057	1.655560013	0.4924441	3.36192475	0.00077401	0.0035912
ENSDARG00000113801	LO017872.1	614.79464	0.436059044	0.12970808	3.3618494	0.00077422	0.00359151
ENSDARG00000091792	akap12a	726.583632	-0.6489689	0.193052	-3.36162743	0.00077485	0.00359373
ENSDARG00000023220	selenot2	2578.75417	0.348746455	0.10374539	3.3615609	0.00077503	0.00359393
ENSDARG00000062758	CR352265.1	130.650454	0.771660405	0.22958654	3.36108727	0.00077636	0.00359942
ENSDARG00000102191	CPNE8	1054.062	-0.497219214	0.14794156	-3.36091645	0.00077684	0.00360098
ENSDARG00000012574	slkb	1567.91517	-0.42095724	0.1252714	-3.36036188	0.0007784	0.00360754
ENSDARG00000017744	smc2	2893.09889	-0.615982893	0.18331909	-3.36016781	0.00077895	0.00360924
ENSDARG00000103907	sgo1	713.759782	-0.781152761	0.232477	-3.36012918	0.00077906	0.00360924
ENSDARG00000074451	mnd1	274.315033	-0.565110687	0.16819392	-3.35987594	0.00077978	0.00361187
ENSDARG00000109869	zgc:174719	14.0120437	2.538865573	0.75567291	3.35974143	0.00078016	0.00361296
ENSDARG00000012422	col11a2	37965.5837	0.39144973	0.11653268	3.35914134	0.00078185	0.00362014
ENSDARG00000029751	wbp1lb	502.910958	0.614332264	0.18290539	3.35874331	0.00078298	0.00362468
ENSDARG00000070538	hey1	1095.87523	-0.390760936	0.11637278	-3.35783806	0.00078555	0.00363589
ENSDARG00000043442	zgc:153665	732.63565	0.500264814	0.14904386	3.3564939	0.00078938	0.00365293
ENSDARG00000061544	ano6	418.563462	0.595791221	0.17752608	3.35607709	0.00079057	0.00365776
ENSDARG00000052994	slc25a28	2025.89648	0.369339446	0.11007739	3.35527078	0.00079287	0.00366776
ENSDARG00000103351	FP102192.2	8.41786669	5.009783601	1.49330812	3.35482246	0.00079416	0.00367249
ENSDARG00000099294	si:ch73-199g24.2	1613.8238	0.432316432	0.12886461	3.35481111	0.00079419	0.00367249
ENSDARG00000098096	slc12a1	161.289569	-1.076435373	0.32088479	-3.35458521	0.00079484	0.00367481
ENSDARG00000103251	sh3pxd2b	1994.21445	0.446319348	0.13305046	3.354512	0.00079505	0.0036751
ENSDARG00000007654	nsfa	5821.51875	-0.476321656	0.14203785	-3.35348395	0.00079801	0.00368809
ENSDARG00000043470	rsph3	67.9586471	1.253222465	0.37371889	3.35338271	0.0007983	0.00368847
ENSDARG00000007753	cpne2	653.817258	-0.48789805	0.14549559	-3.35335282	0.00079839	0.00368847
ENSDARG00000092680	si:dkey-58f10.12	96.6619034	1.137739331	0.33929693	3.35322617	0.00079875	0.00368947
ENSDARG00000012030	dnaaf1	46.1081653	1.274447707	0.38009269	3.35299189	0.00079943	0.00369122
ENSDARG00000070644	ttpal	432.300098	-0.55774654	0.16634282	-3.35299433	0.00079942	0.00369122
ENSDARG00000005894	slc7a9	93.0738271	0.909901469	0.27137481	3.3529327	0.0007996	0.00369133
ENSDARG00000018022	msh2	1293.22689	-0.481472083	0.14360046	-3.35285896	0.00079982	0.00369162
ENSDARG00000054978	kifc3	3608.07308	-0.35128741	0.10481533	-3.35148889	0.00080378	0.00370872
ENSDARG00000079219	mfn1b	1918.28471	0.369387136	0.11021622	3.35147723	0.00080382	0.00370872
ENSDARG00000069866	sox1a	756.10604	-0.616497419	0.18398112	-3.35087331	0.00080557	0.00371613
ENSDARG00000068240	trim110	72.9957628	-1.103699409	0.32940854	-3.35054888	0.00080652	0.00371979
ENSDARG00000031737	pthlha	152.25432	0.638819605	0.19066886	3.35041401	0.00080691	0.00372091
ENSDARG00000043010	camk2d1	1332.54654	0.38466165	0.11483567	3.34967049	0.00080908	0.00373022
ENSDARG00000101695	si:dkey-26m3.3	108.809825	0.855823957	0.25553592	3.34913372	0.00081065	0.00373676
ENSDARG00000013794	klf11b	1463.94006	-0.391954861	0.11703701	-3.34898228	0.00081109	0.00373811

Table S2. DEGs of WT vs. *terfa-/-*

ENSDARG00000003860	cbx3a	2493.05202	-0.469521233	0.14022926	-3.34824002	0.00081327	0.00374744
ENSDARG00000058992	cers2b	1906.38015	0.455141952	0.13594654	3.34794804	0.00081412	0.0037507
ENSDARG00000025106	ppp3cb	3071.34363	-0.484349377	0.14467605	-3.34782003	0.0008145	0.00375174
ENSDARG00000009285	rpl15	36044.1417	0.368854205	0.11020226	3.34706565	0.00081672	0.00376126
ENSDARG00000110510	ybey	301.517631	0.715235356	0.21378279	3.34561712	0.000821	0.00378027
ENSDARG00000096631	MED9	281.158597	0.532369053	0.15915405	3.34499214	0.00082285	0.00378809
ENSDARG00000092369	si:dkeyp-77c8.3	134.183476	0.979293138	0.29277346	3.34488361	0.00082317	0.00378887
ENSDARG00000052907	bcl7a	1657.59206	-0.51874375	0.15509609	-3.34466031	0.00082383	0.00379122
ENSDARG00000095678	ftr38	20.4202124	1.855128309	0.55481003	3.34371805	0.00082664	0.00380342
ENSDARG00000112572	CR293507.1	226.898377	0.738529752	0.22088444	3.34351197	0.00082725	0.00380554
ENSDARG00000069100	aldh9a1a.1	16359.7173	0.467076949	0.13970824	3.34323124	0.00082809	0.00380869
ENSDARG00000090119	tlr8a	7.55060476	5.114801853	1.53005894	3.34287897	0.00082914	0.00381282
ENSDARG00000103457	spartb	565.545493	0.457462919	0.13685043	3.34279482	0.00082939	0.00381327
ENSDARG00000105131	cog1	791.697319	0.376772072	0.1127152	3.34269084	0.0008297	0.00381399
ENSDARG00000077193	nags	36.1014755	-1.635503329	0.4893434	-3.34224051	0.00083105	0.00381948
ENSDARG00000069109	znrd2	845.673294	0.454382481	0.13596056	3.34201676	0.00083172	0.00382185
ENSDARG00000057707	zgc:66443	322.569613	-0.497666725	0.14891507	-3.34195005	0.00083192	0.00382207
ENSDARG00000079233	si:ch211-160f23.5	331.645087	-0.650914944	0.19477494	-3.34188234	0.00083212	0.00382229
ENSDARG00000068855	si:ch211-163l21.1(	43.7127762	1.351254345	0.40434771	3.34181277	0.00083233	0.00382254
ENSDARG00000055760	srm	1184.24566	0.365837256	0.10948793	3.34134784	0.00083373	0.00382824
ENSDARG00000041493	ipo4	1529.70639	0.306929999	0.09186624	3.34105337	0.00083461	0.0038316
ENSDARG00000058865	endog	575.055884	0.562334314	0.16832234	3.34081814	0.00083532	0.00383414
ENSDARG00000068641	taf10	1470.43579	-0.385952631	0.11553956	-3.34043706	0.00083647	0.0038387
ENSDARG00000054304	homeza	586.266989	0.532900224	0.15953573	3.34031895	0.00083682	0.00383962
ENSDARG00000042780	apoba	29631.018	-0.375405946	0.11241679	-3.33941174	0.00083956	0.00385147
ENSDARG00000074868	AL929017.1	386.387825	-0.545190495	0.16327303	-3.3391339	0.0008404	0.00385461
ENSDARG00000088290	foxj1b	149.906338	0.722679478	0.21643805	3.33896688	0.00084091	0.00385622
ENSDARG00000079234	si:ch211-285c6.2	8.23129035	3.523878102	1.05547968	3.33865083	0.00084186	0.0038599
ENSDARG00000014910	panx1b	335.918142	-0.526481231	0.15772395	-3.33799167	0.00084386	0.00386835
ENSDARG00000017425	crocc2	247.829219	0.718995751	0.2154103	3.33779653	0.00084446	0.00387036
ENSDARG00000078959	tpcn3	373.808903	0.452827638	0.13567523	3.33758529	0.0008451	0.00387259
ENSDARG00000005643	gcat	1418.38855	0.415279541	0.12444892	3.33694776	0.00084704	0.00388076
ENSDARG00000103960	CU019624.1	10.2506834	-2.936268092	0.87997571	-3.33676038	0.00084761	0.00388267
ENSDARG00000101750	si:ch211-196p9.1	608.610531	-0.381288905	0.11427723	-3.33652561	0.00084833	0.00388452
ENSDARG00000103052	pcyox1	665.142602	0.600834806	0.18007708	3.33654234	0.00084828	0.00388452
ENSDARG00000060012	f2r	180.706681	0.830395605	0.24890417	3.33620615	0.0008493	0.00388827
ENSDARG00000035700	zgc:101664	348.167757	-0.542355529	0.16257631	-3.33600592	0.00084991	0.00389035
ENSDARG00000042608	ica1	234.933807	-0.598453611	0.17941546	-3.33557434	0.00085123	0.00389568
ENSDARG00000045837	si:dkey-14d8.20	227.218265	0.659660669	0.19777863	3.33534865	0.00085193	0.00389812
ENSDARG00000074023	rbms1a	2882.7032	0.330495026	0.09911148	3.3345788	0.00085429	0.00390821
ENSDARG00000110069	pdgfaa	175.230826	-0.859748815	0.25786013	-3.33416737	0.00085555	0.00391328
ENSDARG00000114922	CU855895.3	353.287657	-0.552456344	0.16570646	-3.33394567	0.00085623	0.00391568
ENSDARG00000069114	unc93b1	197.345059	0.584396278	0.17533298	3.33306544	0.00085895	0.00392736
ENSDARG00000067672	card9	58.5253396	-1.473365242	0.44218559	-3.33200646	0.00086222	0.00394161
ENSDARG00000028058	traf6	745.381044	0.479536057	0.14392325	3.33188747	0.00086259	0.00394257
ENSDARG00000108649	GALNT3	187.043006	1.001532334	0.30062951	3.33145057	0.00086395	0.00394804
ENSDARG00000073876	si:ch1073-165f9.2	52.3909165	1.032246688	0.30986063	3.33132568	0.00086433	0.00394909
ENSDARG00000013659	brox	1014.38393	-0.399394265	0.11992036	-3.3304959	0.00086691	0.00396015
ENSDARG00000035333	mis18a	144.104731	-0.7426489	0.22298905	-3.33042767	0.00086713	0.00396039
ENSDARG00000099946	fam189a2	411.915547	0.507273379	0.15232113	3.33028897	0.00086756	0.00396164
ENSDARG00000094380	zzef1	2375.87939	0.383548073	0.11518468	3.32985315	0.00086892	0.00396711
ENSDARG00000036924	tatdn1	158.956899	0.73957319	0.22211787	3.32964291	0.00086957	0.00396938
ENSDARG00000026489	khsrp	4170.96343	0.595162143	0.17877087	3.32918964	0.00087099	0.00397512
ENSDARG00000097003	CR855375.2	8.50968884	-4.142250983	1.24467439	-3.32797962	0.00087478	0.00399169
ENSDARG00000081252	dre-mir-16b	8.04201124	4.657063493	1.39947767	3.32771547	0.00087561	0.00399474
ENSDARG00000098415	CT956093.1	111.534394	1.062705112	0.31938627	3.32733495	0.00087681	0.00399947
ENSDARG00000012949	scml2	1929.25987	-0.546916164	0.16438019	-3.32714152	0.00087742	0.00400151
ENSDARG00000098368	aplp1	9324.23305	-0.353601188	0.10629839	-3.32649608	0.00087945	0.00401005

Table S2. DEGs of WT vs. *terfa-/-*

ENSDARG00000008263	mfsd4ab	160.480001	-0.695286668	0.20905635	-3.32583371	0.00088155	0.00401886
ENSDARG00000029107	ube2d1a	606.996408	-0.471726199	0.14186745	-3.32511937	0.00088381	0.00402843
ENSDARG00000016594	haus4	266.250953	-1.09832279	0.33031723	-3.32505446	0.00088401	0.00402863
ENSDARG00000111796	CABZ01050238.1	4.40689058	-5.639600234	1.69613906	-3.32496337	0.0008843	0.00402921
ENSDARG00000063414	rxylt1	412.873072	-0.557533254	0.16768883	-3.32480849	0.00088479	0.00403071
ENSDARG00000042644	sod2	2371.0294	0.392126027	0.11795629	3.32433349	0.0008863	0.00403684
ENSDARG00000035883	nt5c1aa	248.164611	0.768082119	0.23111582	3.32336452	0.00088939	0.00405015
ENSDARG00000027501	usp20	1038.61418	0.327842302	0.09865056	3.32326865	0.00088969	0.0040504
ENSDARG00000030177	uchl3	1046.97027	-0.374041291	0.11255303	-3.32324502	0.00088977	0.0040504
ENSDARG00000054744	si:ch211-244e12.7	21.0367843	-1.83557741	0.55238266	-3.32301781	0.00089049	0.00405296
ENSDARG00000092775	BX640547.1	15.3402738	-2.62609032	0.79038399	-3.32255001	0.00089199	0.00405901
ENSDARG00000056277	or132-5	15.9281255	2.113414604	0.63617895	3.32204422	0.00089361	0.00406563
ENSDARG00000093240	GMEB2	583.052811	-0.41194765	0.1240349	-3.32122362	0.00089624	0.00407686
ENSDARG00000001442	inppl1b	378.533221	-0.522796028	0.15741544	-3.32112297	0.00089656	0.00407759
ENSDARG00000071735	prlh2	77.3202333	1.09758456	0.33061028	3.3198743	0.00090058	0.00409452
ENSDARG00000071643	zgc:171490	154.303401	1.096609556	0.33031759	3.31986422	0.00090061	0.00409452
ENSDARG00000074702	arfgef2	2788.41063	0.355532653	0.10709594	3.31975845	0.00090095	0.00409532
ENSDARG00000069326	mus81	320.546487	-0.601892883	0.18133261	-3.31927543	0.00090251	0.00410166
ENSDARG00000076547	si:ch211-221f10.2	1302.32086	0.526742659	0.15871374	3.31882214	0.00090398	0.00410607
ENSDARG00000051891	rsph10b	82.640565	0.845623115	0.2547913	3.31888537	0.00090378	0.00410607
ENSDARG00000058381	zgc:171775	1819.73092	-0.358591519	0.10804695	-3.31884911	0.00090389	0.00410607
ENSDARG00000086853	dpyda.3	185.226936	0.667602954	0.20116049	3.31875781	0.00090419	0.00410627
ENSDARG00000003991	fhl2b	424.875858	-0.619450404	0.18668541	-3.3181511	0.00090616	0.00411445
ENSDARG00000042522	katnbl1	462.966241	-0.504930994	0.15218515	-3.31787292	0.00090706	0.00411779
ENSDARG00000016345	ncoa5	3378.39004	-0.402711409	0.12138893	-3.31752994	0.00090817	0.0041221
ENSDARG00000014053	olfm1b	4337.38401	-0.440212561	0.13269817	-3.31739745	0.0009086	0.0041233
ENSDARG00000102924	zgc:112334	90.7399245	-1.207819157	0.36412854	-3.31701316	0.00090985	0.00412822
ENSDARG00000079979	fam89a	578.632935	0.561286858	0.16923422	3.3166274	0.00091111	0.00413317
ENSDARG00000029063	clpxa	3016.15672	0.333815362	0.10065296	3.31649815	0.00091153	0.00413358
ENSDARG00000086057	plxnd1	484.726676	0.614531941	0.1852934	3.31653441	0.00091141	0.00413358
ENSDARG00000029296	nexmifb	1133.35741	-0.450731463	0.13594485	-3.31554655	0.00091464	0.00414616
ENSDARG00000097513	CT573383.1	149.915687	-1.133771462	0.34195523	-3.31555529	0.00091461	0.00414616
ENSDARG00000100262	BX936382.1	82.9767099	0.91870687	0.27710138	3.31541788	0.00091506	0.00414731
ENSDARG00000045850	ccdc167	351.651212	0.543083564	0.16381369	3.31525141	0.00091561	0.00414903
ENSDARG00000055903	luc7l	4233.46109	-0.419577759	0.12657304	-3.31490616	0.00091674	0.0041534
ENSDARG00000060023	ncapg2	667.591189	-0.584612694	0.17636943	-3.31470542	0.0009174	0.00415498
ENSDARG00000096686	CT573003.1	16.6875601	-2.192810598	0.66154159	-3.31469801	0.00091742	0.00415498
ENSDARG00000096037	si:ch211-214j24.14	372.053147	0.473049792	0.14279579	3.31277131	0.00092377	0.00418294
ENSDARG00000042189	tspan33b	43.9848918	-1.399063142	0.42234202	-3.31263071	0.00092423	0.00418428
ENSDARG00000092347	si:dkey-161j23.6	395.774396	-0.515396216	0.15559569	-3.3124068	0.00092497	0.00418687
ENSDARG00000005108	oclna	2085.05421	0.395519533	0.11941365	3.31218024	0.00092572	0.0041895
ENSDARG00000002536	tm9sf4	2734.3406	0.330341765	0.0997397	3.31203887	0.00092619	0.00419086
ENSDARG00000077291	rps2	88333.0402	0.375256413	0.1133115	3.31172391	0.00092723	0.00419481
ENSDARG00000059348	rxfp3.3b	129.893593	-0.801385831	0.24202114	-3.31122239	0.00092889	0.00420157
ENSDARG00000031138	irx3b	285.556103	-0.775432897	0.23419419	-3.3110681	0.00092941	0.00420313
ENSDARG00000036721	tomm40l	1519.11122	-0.440148172	0.13293829	-3.31092108	0.00092989	0.00420457
ENSDARG00000054362	ccdc47	4265.57008	-0.341394658	0.10312541	-3.31048042	0.00093136	0.00421043
ENSDARG00000090708	si:dkey-28g23.6	25.6213861	-2.51997974	0.76123209	-3.3103961	0.00093164	0.00421093
ENSDARG00000062646	tet3	8198.20163	-0.507771367	0.15339967	-3.31012031	0.00093256	0.00421432
ENSDARG00000057678	sfrp1b	219.981991	-0.666262599	0.20129174	-3.30993513	0.00093318	0.00421634
ENSDARG00000089302	hnrnpa0a	4890.63154	-0.498350969	0.15056611	-3.30984831	0.00093347	0.00421688
ENSDARG00000052688	paqr5b	141.45837	0.890225743	0.26896902	3.30977056	0.00093373	0.00421729
ENSDARG00000032482	si:dkey-40c11.2	1695.91352	0.435402488	0.13155999	3.30953579	0.00093451	0.00422006
ENSDARG00000104287	lto1	137.340133	-0.716931829	0.21664504	-3.30924645	0.00093548	0.00422366
ENSDARG00000061352	bcl11aa	3356.70399	-0.454232212	0.13727064	-3.30902669	0.00093621	0.0042262
ENSDARG00000070834	taf13	904.915495	-0.416077714	0.12574414	-3.30892328	0.00093656	0.004227
ENSDARG00000035735	gsx1	241.386773	-1.643619181	0.49677164	-3.30860106	0.00093763	0.0042311
ENSDARG00000078694	phlpp1	2511.34808	-0.461028972	0.13934901	-3.30844821	0.00093815	0.00423264

Table S2. DEGs of WT vs. *terfa-/-*

ENSDARG00000104114	kmt5ab	559.458013	-0.781601039	0.23625989	-3.3082257	0.00093889	0.00423523
ENSDARG00000097189	FP101861.1	88.7293214	-0.991159354	0.29967836	-3.30741053	0.00094163	0.00424681
ENSDARG00000104899	CR931813.2	132.558448	-2.510189191	0.75903277	-3.30708937	0.00094271	0.00425091
ENSDARG00000046010	kdm2bb	1563.09787	-0.382030693	0.11559786	-3.30482498	0.00095036	0.00428462
ENSDARG00000074773	si:dkey-110g7.8	144.525921	1.090663367	0.33003706	3.30466934	0.00095089	0.00428623
ENSDARG00000035390	tnpo1	2461.23629	-0.323722517	0.09796303	-3.30453746	0.00095133	0.00428747
ENSDARG00000006196	sav1	1528.90946	0.326464347	0.09880566	3.30410588	0.0009528	0.00429329
ENSDARG00000093822	si:dkey-259j3.5	42.7809717	1.316122855	0.39835669	3.3038804	0.00095357	0.00429597
ENSDARG00000041896	dnajc5ga	1117.75793	-0.37791166	0.11442103	-3.30281639	0.00095719	0.00431152
ENSDARG00000110902	CABZ01086835.1	15.9424157	-2.034523638	0.61601465	-3.30271959	0.00095752	0.00431223
ENSDARG00000079504	mfn2	1942.56584	0.299930731	0.09081634	3.30260774	0.0009579	0.00431317
ENSDARG00000098898	si:ch211-255f4.2	131.640081	-0.888109355	0.2689167	-3.30254448	0.00095812	0.00431336
ENSDARG00000055070	borcs7	104.242405	-0.765927317	0.23196092	-3.30196706	0.00096009	0.00432045
ENSDARG00000010096	slc1a6	465.121804	-0.531114232	0.16084958	-3.30193117	0.00096022	0.00432045
ENSDARG00000103044	ASS1	718.290943	0.520321828	0.15757927	3.30196868	0.00096009	0.00432045
ENSDARG00000027933	glis1b	168.996519	0.642889815	0.19472964	3.30144823	0.00096187	0.00432711
ENSDARG00000098720	LPL	99.9790523	-1.019021109	0.3086739	-3.3012869	0.00096242	0.00432882
ENSDARG00000105511	BX248521.2	44.1400313	1.480139041	0.44839366	3.30098122	0.00096347	0.00433275
ENSDARG00000086337	si:dkey-102g19.3	23.6883914	1.703968574	0.51621719	3.3008753	0.00096384	0.00433361
ENSDARG00000039374	apool	1085.83556	0.358412108	0.10859283	3.30051369	0.00096508	0.00433771
ENSDARG00000077442	tubgcp5	792.626484	-0.427942805	0.12965968	-3.30050797	0.0009651	0.00433771
ENSDARG00000097160	si:ch211-121j5.4	334.3274	-1.060398271	0.32131431	-3.30019005	0.00096619	0.00434185
ENSDARG00000010519	per3	3926.03969	0.471411198	0.14285388	3.29995377	0.00096701	0.00434472
ENSDARG00000090872	si:dkey-276j7.1	4990.15301	-0.488934017	0.14818029	-3.2995888	0.00096827	0.00434958
ENSDARG00000111606	smyhc2	52.8421801	4.413462678	1.33783724	3.29895338	0.00097046	0.00435865
ENSDARG00000086228	si:dkey-120c6.5	61.4248964	1.099543348	0.33333634	3.29860031	0.00097168	0.00436335
ENSDARG00000003382	cwc25	868.781923	-0.420190064	0.12738758	-3.29851672	0.00097197	0.00436386
ENSDARG00000098273	zbtb14	1105.19784	-0.491274797	0.14895663	-3.2981062	0.00097339	0.00436946
ENSDARG00000004018	CHST13	249.540676	0.608300729	0.18446392	3.29766783	0.00097491	0.00437549
ENSDARG00000086980	mbd6	1401.23162	-0.623696701	0.18916022	-3.29718751	0.00097658	0.00438219
ENSDARG00000105651	BX323060.3	513.76088	0.730832404	0.22167928	3.29680069	0.00097793	0.00438744
ENSDARG00000083588	dre-mir-181b-3	34.3530483	-2.148234856	0.65173988	-3.29615374	0.00098018	0.00439676
ENSDARG00000059303	hnrnpl2	5789.52977	-0.390374767	0.11844017	-3.29596607	0.00098084	0.00439891
ENSDARG00000098897	pds5b	4760.26543	-0.392472607	0.11909205	-3.29553998	0.00098233	0.00440479
ENSDARG00000068705	tmem168a	667.444228	0.380271147	0.11540276	3.29516514	0.00098364	0.00440987
ENSDARG00000029248	fubp1	12271.9636	-0.342288269	0.10388266	-3.29495084	0.00098439	0.00441217
ENSDARG00000025581	rpl10	45720.5557	0.347115553	0.10534879	3.2949173	0.00098451	0.00441217
ENSDARG00000002339	sgf29	384.562161	-0.598406823	0.1816193	-3.29484158	0.00098477	0.00441257
ENSDARG00000029250	poli	212.784277	0.61266082	0.18597848	3.29425662	0.00098682	0.00442096
ENSDARG00000015422	ppil1	852.67258	0.479199867	0.14547143	3.29411677	0.00098732	0.00442234
ENSDARG00000102569	polr3a	836.578498	0.450150927	0.13665503	3.29406783	0.00098749	0.00442234
ENSDARG00000062457	las1l	986.274244	-0.411221709	0.12483963	-3.29399963	0.00098773	0.00442262
ENSDARG00000095899	BX088696.2	2.98342194	6.008955679	1.82446764	3.2935392	0.00098935	0.00442907
ENSDARG00000089331	urahb	34.1914773	-1.650273396	0.50108367	-3.29340883	0.0009898	0.00443032
ENSDARG00000017748	arhgap29b	1349.07925	0.363444117	0.11035902	3.29328887	0.00099023	0.00443142
ENSDARG00000092738	si:ch211-263k4.2	68.6767705	1.105217312	0.33562559	3.29300674	0.00099122	0.00443507
ENSDARG00000101613	si:dkey-238i5.2	248.672647	0.678825719	0.20615631	3.29277193	0.00099205	0.00443797
ENSDARG00000042032	pou3f3a	1045.38271	-0.511548725	0.15535875	-3.29269337	0.00099233	0.00443842
ENSDARG00000009771	ppme1	1720.38346	-0.420471503	0.12771913	-3.2921576	0.00099422	0.00444559
ENSDARG00000086302	proser1	2169.01626	-0.500743454	0.15210282	-3.29213784	0.00099429	0.00444559
ENSDARG00000078167	si:dkeyp-100h4.1	43.3035722	1.550919629	0.47114561	3.2918053	0.00099547	0.00445005
ENSDARG00000099949	FP236551.1	6.66631797	3.644754358	1.10753421	3.29087294	0.00099877	0.00446247
ENSDARG00000060235	mst1ra	379.509508	0.56385908	0.17134045	3.29086962	0.00099878	0.00446247
ENSDARG00000035676	ptp4a2b	3215.03086	0.293542085	0.08919886	3.29087247	0.00099877	0.00446247
ENSDARG00000035910	znf281b	1488.37747	-0.586575963	0.17826241	-3.2905197	0.00100003	0.00446722
ENSDARG00000079366	ppp1r9ba	2782.91203	-0.409830955	0.12455756	-3.29029385	0.00100083	0.00446941
ENSDARG00000075389	si:ch211-281g13.4	10.0634311	2.459158342	0.7474007	3.29028101	0.00100087	0.00446941
ENSDARG00000004537	kmt2a	4309.47596	-0.56784841	0.1726148	-3.28968545	0.00100299	0.00447807

Table S2. DEGs of WT vs. *terfa-/-*

ENSDARG00000070903	met	714.972651	0.479881905	0.1459148	3.28878148	0.00100622	0.00449167
ENSDARG00000052594	nkiras2	881.856703	-0.428900583	0.130432	-3.2883079	0.00100792	0.00449843
ENSDARG00000079525	slc39a5	493.786621	0.667818586	0.20309536	3.28820213	0.00100829	0.00449931
ENSDARG00000001414	mctp2a	282.675365	0.558997475	0.17002373	3.28776143	0.00100987	0.00450555
ENSDARG00000015134	camkk1b	165.344241	-1.132620736	0.34455044	-3.28724218	0.00101174	0.00451306
ENSDARG00000051875	islr2	8286.47489	-0.442419024	0.13459332	-3.28708014	0.00101232	0.00451484
ENSDARG00000093406	C19H6orf62	5618.88354	-0.329419241	0.10022094	-3.2869302	0.00101286	0.00451644
ENSDARG00000078251	TESK1	1022.22072	-0.430818574	0.13107781	-3.28673924	0.00101355	0.00451869
ENSDARG00000038281	natd1	1289.08661	0.334435057	0.10179363	3.28542216	0.0010183	0.00453824
ENSDARG00000070828	actl6a	2330.01103	-0.496722973	0.15118923	-3.28543879	0.00101824	0.00453824
ENSDARG00000103556	si:ch211-218g23.6	32.5613399	1.413960102	0.43038258	3.28535626	0.00101854	0.00453849
ENSDARG00000087013	cubn	915.734985	0.62174803	0.18926521	3.28506237	0.0010196	0.00454241
ENSDARG00000044136	rab3gap2	1637.81553	0.344372672	0.1048364	3.284858	0.00102034	0.00454489
ENSDARG00000010248	wdr54	413.999185	-0.559301181	0.17027556	-3.28468262	0.00102097	0.00454691
ENSDARG00000101799	si:ch1073-82l19.1	126.777549	-0.761680277	0.23189647	-3.28457041	0.00102138	0.0045479
ENSDARG00000005374	tubgcp4	826.29427	-0.409573935	0.12473926	-3.28344053	0.00102548	0.00456454
ENSDARG00000012932	mat2b	749.864744	-0.480670003	0.14639107	-3.28346536	0.00102539	0.00456454
ENSDARG00000023713	aqp1a.1	3620.43747	0.303813629	0.09258859	3.28132898	0.00103319	0.00459803
ENSDARG00000035459	spns3	108.961015	-0.84750451	0.25828554	-3.28126974	0.00103341	0.00459817
ENSDARG00000021556	ubl3b	2104.48915	-0.454084628	0.13839558	-3.281063	0.00103417	0.00460072
ENSDARG00000092003	ccs	202.771135	-0.845484628	0.25771071	-3.28075078	0.00103531	0.00460416
ENSDARG00000053222	asb5b	559.195124	0.490174882	0.14940888	3.28076137	0.00103527	0.00460416
ENSDARG00000013582	csnk2a2b	1871.14116	-0.338345342	0.10314013	-3.28044312	0.00103644	0.00460836
ENSDARG00000032465	slc1a8b	58.7960465	-1.961934772	0.59809546	-3.28030377	0.00103695	0.00460887
ENSDARG00000054220	ap4s1	327.156754	0.517528418	0.15777052	3.28026064	0.00103711	0.00460887
ENSDARG00000102114	ptdss1b	67.8729312	1.165208912	0.35521218	3.28031804	0.0010369	0.00460887
ENSDARG00000002642	sh3gl1b	1197.21766	0.364678232	0.11122348	3.27878814	0.00104254	0.00463216
ENSDARG00000091754	CT573476.1	217.10951	-0.725796184	0.22139071	-3.27834976	0.00104416	0.00463853
ENSDARG00000045123	oplah	1451.54911	0.355442848	0.10842872	3.27812456	0.00104499	0.0046414
ENSDARG00000056004	sprn	577.848918	-0.546832831	0.16681534	-3.27807284	0.00104518	0.00464143
ENSDARG00000042213	ELAPOR2	137.062969	0.691991229	0.21110831	3.27789666	0.00104584	0.00464349
ENSDARG00000089645	si:ch1073-406l10.2	231.758766	0.675913749	0.20621204	3.2777608	0.00104634	0.0046449
ENSDARG00000096009	BX901962.1	2.86160429	5.786885314	1.76593101	3.27696002	0.00104931	0.00465541
ENSDARG00000006677	ripk1l	316.405128	0.525761788	0.16044384	3.27692096	0.00104946	0.00465541
ENSDARG00000074271	get1	649.350946	-0.465122102	0.14193475	-3.27701363	0.00104911	0.00465541
ENSDARG00000007097	her13	254.107207	-0.690828179	0.21080718	-3.27706192	0.00104893	0.00465541
ENSDARG00000055415	zp2l1	8.3773318	3.924661039	1.19793563	3.27618692	0.00105219	0.00466586
ENSDARG00000116524	zgc:103700	11.7136902	-2.406685455	0.73459851	-3.27619158	0.00105217	0.00466586
ENSDARG00000022939	ino80e	348.653105	-0.667677089	0.20382154	-3.27579258	0.00105366	0.00467155
ENSDARG00000075281	tbc1d30	796.966673	-0.484530983	0.14792306	-3.27556091	0.00105452	0.00467455
ENSDARG00000063009	catip	64.3308273	0.933969971	0.28515681	3.27528558	0.00105555	0.00467786
ENSDARG00000093170	mef2b	66.9312986	-1.050275442	0.32066934	-3.27525997	0.00105565	0.00467786
ENSDARG00000078781	si:ch211-161c3.6	358.790363	-0.530094381	0.16185083	-3.27520341	0.00105586	0.00467796
ENSDARG00000036345	cmtr2	364.550382	-0.467921047	0.14287829	-3.27496251	0.00105676	0.00468112
ENSDARG00000057184	C2H7orf25	534.583676	0.414007254	0.12641812	3.27490437	0.00105698	0.00468125
ENSDARG00000008886	msx1b	560.603972	0.567036018	0.17316194	3.27459967	0.00105812	0.00468547
ENSDARG00000025017	prdm8	187.473241	-0.788729831	0.24089283	-3.27419391	0.00105964	0.00469136
ENSDARG00000045262	gsnb	3361.51425	0.576819444	0.1761769	3.27409234	0.00106002	0.00469138
ENSDARG00000041085	arpin	270.460665	0.709184218	0.21660208	3.27413391	0.00105986	0.00469138
ENSDARG00000097599	CR925804.1	4.25668971	5.225458615	1.59632164	3.27343719	0.00106248	0.00470143
ENSDARG00000100453	GALNT10	791.230247	0.369583238	0.11290766	3.27332311	0.00106291	0.00470249
ENSDARG00000057245	im:7138535	239.965111	-0.566810966	0.17317128	-3.27312337	0.00106366	0.00470498
ENSDARG00000054456	clip3	1557.60112	-0.423238364	0.12931039	-3.27304228	0.00106397	0.00470549
ENSDARG00000062579	kremen1	460.421137	0.541893254	0.16557763	3.27274442	0.00106509	0.00470962
ENSDARG00000070957	im:7160594	492.047652	0.658645366	0.20127356	3.27238896	0.00106643	0.0047147
ENSDARG00000068407	six9	43.7104935	-1.345956761	0.41135736	-3.271989	0.00106794	0.00472054
ENSDARG00000013596	brd1a	1364.8511	-0.387660379	0.11848535	-3.27180021	0.00106865	0.00472285
ENSDARG00000060366	slc12a9	668.709487	0.587161311	0.17946938	3.27165174	0.00106921	0.00472449

Table S2. DEGs of WT vs. *terfa-/-*

ENSDARG00000090167	FO904898.1	4.09178448	5.15672394	1.57632748	3.27135319	0.00107034	0.00472864
ENSDARG00000004151	rab23	502.68953	0.531509834	0.16249743	3.27088149	0.00107213	0.00473569
ENSDARG00000011334	ncaldb	494.648742	-0.937977877	0.28680355	-3.27045421	0.00107375	0.00474201
ENSDARG00000056502	si:ch73-334d15.4	392.909914	-0.841544318	0.25734237	-3.27013516	0.00107496	0.00474652
ENSDARG00000042548	tpd52l1	424.942237	-0.446022897	0.13642374	-3.26939364	0.00107778	0.00475813
ENSDARG00000060504	pfkla	1700.94539	0.516657646	0.15803973	3.26916307	0.00107866	0.00476117
ENSDARG00000070546	msgn1	16.3563167	1.866555463	0.57103533	3.2687215	0.00108035	0.00476691
ENSDARG00000021339	cpa5	3353.05223	-1.988598141	0.60836494	-3.26875864	0.0010802	0.00476691
ENSDARG00000030573	nudt1	107.087468	-0.926139751	0.28336369	-3.26837838	0.00108166	0.00477185
ENSDARG00000033594	ido1	88.3510931	-1.387442853	0.42453381	-3.26815631	0.00108251	0.00477474
ENSDARG00000105355	zgc:154164	49.7206289	-1.575119281	0.48203281	-3.26765992	0.00108441	0.00478228
ENSDARG00000036629	rps14	25862.5101	0.376040134	0.11510524	3.26692466	0.00108723	0.00479386
ENSDARG00000040440	snrpd2	1748.86111	-0.456071303	0.13961267	-3.2666899	0.00108813	0.00479699
ENSDARG00000039458	lhx4	906.571304	-0.60463059	0.18512316	-3.26609919	0.0010904	0.00480615
ENSDARG00000078134	crygm2f	13.585044	2.308235146	0.70676907	3.26589721	0.00109118	0.00480873
ENSDARG00000006003	mycla	797.383016	-0.749098346	0.22938071	-3.2657425	0.00109178	0.00481051
ENSDARG00000013841	abl2	1370.55948	0.319540406	0.09785075	3.26558991	0.00109236	0.00481225
ENSDARG00000003599	rpl3	82904.8536	0.310183879	0.09499092	3.26540568	0.00109307	0.00481452
ENSDARG00000018918	rad17	378.034659	-0.505506121	0.15481139	-3.26530321	0.00109347	0.00481541
ENSDARG00000112811	CZQB01141835.1	1596.24284	-0.472106284	0.14462006	-3.26445914	0.00109673	0.00482893
ENSDARG00000008757	entpd6	1127.96484	0.374578716	0.11475345	3.26420454	0.00109772	0.00483241
ENSDARG00000020298	btg2	2409.97396	-0.627271196	0.19217442	-3.26407235	0.00109823	0.00483381
ENSDARG00000053201	zgc:172323	81.0015089	-1.387738386	0.42529871	-3.26297342	0.0011025	0.00485174
ENSDARG00000042489	thap12a	741.33274	-0.496372509	0.15213923	-3.26262005	0.00110387	0.00485693
ENSDARG00000019311	fbxo30b	344.832412	0.481566502	0.14761382	3.26234031	0.00110496	0.00486086
ENSDARG00000040881	hnrnph1	811.466973	0.414077031	0.12693844	3.26203038	0.00110617	0.00486532
ENSDARG00000024789	mxc	105.909191	0.884173765	0.27106241	3.26188264	0.00110675	0.00486614
ENSDARG00000104891	si:dkey-109j17.5	316.678044	-0.592714297	0.18170828	-3.2619004	0.00110668	0.00486614
ENSDARG00000019135	ankef1a	66.3014441	0.917054641	0.28119353	3.26129358	0.00110905	0.00487539
ENSDARG00000059972	raly	629.226028	-0.503908354	0.15451789	-3.26116524	0.00110955	0.00487674
ENSDARG00000018809	abhd3	547.726975	0.561266552	0.17213522	3.26061423	0.00111171	0.00488536
ENSDARG00000035056	fgf13a	1195.69598	-0.389388159	0.1194249	-3.26052751	0.00111205	0.00488599
ENSDARG00000037363	dennd2b	1672.03643	0.26735668	0.08200661	3.26018434	0.0011134	0.00489104
ENSDARG00000043126	blf	87.8119059	-0.892393927	0.27373232	-3.26009701	0.00111374	0.00489168
ENSDARG00000006396	nrcama	1047.04777	-0.456654985	0.14010149	-3.25945835	0.00111625	0.00490184
ENSDARG00000058243	phactr3a	685.169404	-0.612084221	0.18780213	-3.2591974	0.00111728	0.00490549
ENSDARG00000057987	uba3	1141.56683	-0.37297309	0.11444893	-3.25886038	0.00111861	0.00491045
ENSDARG00000089806	si:dkey-239j18.3	318.849192	-1.789353746	0.54910428	-3.25867746	0.00111933	0.00491275
ENSDARG00000074921	fam117ba	1375.31742	-0.530560343	0.16282004	-3.25856914	0.00111976	0.00491376
ENSDARG00000074676	rprd2b	938.464428	-0.450334665	0.1382055	-3.25844249	0.00112026	0.00491509
ENSDARG00000042336	impdh1a	626.142461	-0.542070631	0.16637502	-3.25812517	0.00112151	0.00491798
ENSDARG00000102879	myt1b	2490.2767	-0.579968356	0.17800678	-3.2581251	0.00112151	0.00491798
ENSDARG00000019998	slc30a7	1447.15654	0.345203905	0.10595102	3.25814626	0.00112143	0.00491798
ENSDARG00000012467	spint1b	1385.70366	0.418064258	0.12836724	3.25678302	0.00112683	0.00494042
ENSDARG00000062165	tub	926.351058	-0.46496085	0.14277701	-3.25655273	0.00112774	0.0049429
ENSDARG00000013607	sema3gb	1730.59574	0.430089987	0.1320696	3.25654041	0.00112779	0.0049429
ENSDARG00000005284	fbxl15	283.962591	0.616767461	0.18940038	3.25642154	0.00112826	0.0049441
ENSDARG00000043079	MMP23B	219.337708	-0.537497597	0.16507146	-3.25615101	0.00112934	0.00494794
ENSDARG00000024488	top2a	6631.92582	-0.613550399	0.18845891	-3.25561896	0.00113146	0.00495635
ENSDARG00000109844	BX901962.2	74.2527587	-0.954774657	0.29328933	-3.25540195	0.00113232	0.00495926
ENSDARG00000053019	rnft2	463.898283	-0.622735636	0.19130893	-3.25513113	0.0011334	0.00496225
ENSDARG00000006392	exosc9	457.615379	-0.533540553	0.16390665	-3.25514893	0.00113333	0.00496225
ENSDARG00000044155	mafaa	1039.06247	0.353494807	0.10860375	3.25490423	0.00113431	0.00496534
ENSDARG00000055652	aclyb	304.711778	0.772301958	0.23728303	3.25477122	0.00113484	0.00496679
ENSDARG00000078328	sec22a	565.06448	-0.558836109	0.17170058	-3.25471303	0.00113507	0.00496693
ENSDARG00000040727	tfb1m	143.382151	-0.632549646	0.19436079	-3.25451267	0.00113587	0.00496956
ENSDARG00000075444	cgref1	477.833813	0.639337715	0.19646286	3.25424211	0.00113695	0.00497342
ENSDARG00000017444	hal	1043.49998	0.679828408	0.20891493	3.25409202	0.00113755	0.00497517

Table S2. DEGs of WT vs. *terfa-/-*

ENSDARG00000074827	si:ch73-184c24.1	99.8169043	0.889010211	0.27324753	3.25349772	0.00113994	0.00498472
ENSDARG00000070568	atad5a	1144.35603	-0.584541027	0.17968861	-3.2530778	0.00114162	0.00499121
ENSDARG00000033320	sar1ab	998.8498	0.471165677	0.14484383	3.25292189	0.00114225	0.00499307
ENSDARG00000034042	dnal1	52.141261	-1.2557962	0.38606463	-3.25281337	0.00114269	0.0049941
ENSDARG00000013681	si:dkey-42i9.6	2361.80379	0.471103203	0.14483258	3.25274325	0.00114297	0.00499445
ENSDARG00000101216	meaf6	1030.24339	-0.494385202	0.15200524	-3.25242215	0.00114426	0.00499922
ENSDARG00000074004	stac	102.559312	-0.929072938	0.28566154	-3.25235576	0.00114453	0.00499951
ENSDARG00000044781	bace2	469.010737	0.495499951	0.15238017	3.25173509	0.00114703	0.00500956
ENSDARG00000063649	tead3b	988.527699	0.495214635	0.15232026	3.2511409	0.00114943	0.00501916
ENSDARG00000005540	xpo7	2500.04469	-0.437102265	0.13445302	-3.25096647	0.00115013	0.00502136
ENSDARG00000071259	fbxo34	2292.51802	0.391659968	0.12048868	3.25059546	0.00115164	0.00502703
ENSDARG00000091027	aqp1a.2	4.98417417	-5.714335896	1.75809507	-3.25029971	0.00115283	0.00503138
ENSDARG00000056649	htatsf1	1200.76475	-0.400372796	0.12319728	-3.24985089	0.00115466	0.00503844
ENSDARG00000104225	ndel1b	1097.24738	0.378942425	0.11661067	3.24963775	0.00115552	0.00504133
ENSDARG00000070486	rbp7b	53.4863905	-1.244767574	0.38308706	-3.24930731	0.00115686	0.00504617
ENSDARG00000055412	mylk4b	447.911875	0.669221696	0.20596093	3.24926528	0.00115704	0.00504617
ENSDARG00000033259	dis3l2	810.513439	-0.352028474	0.108346	-3.24911358	0.00115765	0.00504797
ENSDARG00000093413	edil3a	1962.45489	-0.419420564	0.12910271	-3.24873566	0.00115919	0.00505291
ENSDARG00000089883	si:dkey-210j14.3	158.853537	-0.719029067	0.22132404	-3.24876168	0.00115909	0.00505291
ENSDARG00000101616	arfgap1	2369.1264	0.374378752	0.11528866	3.24731634	0.00116499	0.00507729
ENSDARG00000093374	EPB41L2	6801.17044	-0.40176137	0.12372766	-3.24714274	0.0011657	0.0050795
ENSDARG00000012818	csnk2a2a	1594.83664	-0.361164971	0.11122947	-3.24702589	0.00116618	0.00508069
ENSDARG00000044304	prrc1	1305.70119	0.297139036	0.09151644	3.24683779	0.00116695	0.00508316
ENSDARG00000037892	psmc3ip	136.137389	-0.879203024	0.27083347	-3.24628648	0.00116921	0.00509212
ENSDARG00000115830	BX465228.2	6.22939834	5.226031255	1.60989293	3.24619802	0.00116958	0.00509258
ENSDARG00000070396	serpinb1l2	47.6526522	1.355283361	0.41750344	3.24616094	0.00116973	0.00509258
ENSDARG00000061100	nars1	4706.42426	0.433626493	0.13360002	3.24570689	0.00117159	0.00509982
ENSDARG00000079805	tagln3a	319.318426	-0.786176289	0.24222515	-3.24564264	0.00117186	0.00510005
ENSDARG00000091933	BX649388.1	22.2407374	1.561001588	0.4809602	3.24559409	0.00117206	0.00510005
ENSDARG00000076283	snrpd3l	970.381572	-0.527636878	0.16257622	-3.24547385	0.00117255	0.00510132
ENSDARG00000099677	bambib	450.524066	0.39767337	0.12255156	3.24494744	0.00117472	0.00510897
ENSDARG00000060390	stk26	1212.66474	0.355859964	0.10966496	3.24497432	0.00117461	0.00510897
ENSDARG00000055875	pag1	1620.96277	-0.310586114	0.09571689	-3.24484125	0.00117516	0.00510998
ENSDARG00000058319	cep76	207.32416	-0.650524693	0.20050103	-3.24449552	0.00117659	0.00511529
ENSDARG00000054934	zgc:101765	83.6243418	0.843371028	0.25996197	3.24420923	0.00117777	0.00511954
ENSDARG00000046054	CT027815.1	403.017891	0.496816031	0.15314629	3.24406173	0.00117838	0.0051213
ENSDARG00000038794	zgc:113531	904.508042	-0.519548837	0.16017311	-3.24367086	0.00118	0.00512653
ENSDARG00000068965	nrip1a	1427.5422	-0.456070388	0.14060187	-3.24370064	0.00117988	0.00512653
ENSDARG00000103525	YTHDC2	289.749375	-0.734723027	0.22652457	-3.24345841	0.00118088	0.00512892
ENSDARG00000041361	ttk	1083.56352	-0.709268613	0.21867796	-3.24343902	0.00118096	0.00512892
ENSDARG00000098257	pcdh15a	277.86394	0.611125192	0.18843297	3.2431967	0.00118197	0.00513238
ENSDARG00000060813	plekha7a	875.975166	0.46048975	0.14199689	3.24295652	0.00118296	0.00513581
ENSDARG00000077577	ntsr1	45.5782347	1.231916102	0.37990962	3.24265575	0.00118421	0.00514034
ENSDARG00000004322	ush1ga	169.822495	-0.871177033	0.26867217	-3.242528	0.00118474	0.00514175
ENSDARG00000087528	snip1	839.971815	-0.432184444	0.13331659	-3.2417904	0.00118781	0.00515417
ENSDARG00000105266	si:dkey-16p6.1	33.0018777	1.352287038	0.41714963	3.24173136	0.00118806	0.00515434
ENSDARG00000096299	CR786578.2	5.04092881	-6.110914139	1.88510777	-3.24167894	0.00118828	0.00515439
ENSDARG00000078805	ap5b1	302.025231	-0.653417393	0.20158497	-3.24139944	0.00118944	0.00515855
ENSDARG00000039910	rbm42	994.589976	-0.418066573	0.12898265	-3.24126206	0.00119002	0.00516013
ENSDARG00000099499	ndufa4l	6779.61421	0.40788257	0.12584726	3.24109224	0.00119073	0.00516231
ENSDARG00000055539	epdl2	521.529026	0.813428436	0.25100766	3.24065188	0.00119257	0.00516939
ENSDARG00000100259	zgc:171759	9.22670398	3.353535772	1.03500201	3.2401249	0.00119477	0.00517805
ENSDARG00000078733	cnnm2b	196.754596	0.588287596	0.18157351	3.23994179	0.00119554	0.00518047
ENSDARG00000055524	rnf7	1972.17764	0.35589783	0.10985343	3.23975179	0.00119634	0.00518302
ENSDARG00000021647	gnai1	3060.89969	0.263046155	0.08121727	3.23879573	0.00120036	0.00519941
ENSDARG00000078981	faxcb	485.098303	-0.495573811	0.15301383	-3.23875171	0.00120054	0.00519941
ENSDARG00000036462	rab11fip1b	278.320247	0.504923488	0.15593433	3.23805213	0.00120349	0.00521127
ENSDARG00000102506	lrp2a	3520.60516	0.467905612	0.1445092	3.23789501	0.00120415	0.00521323

Table S2. DEGs of WT vs. *terfa-/-*

ENSDARG00000062632	duox	167.758645	1.314852685	0.40623756	3.23665957	0.00120938	0.00523494
ENSDARG00000077926	si:dkey-48p11.3	146.618363	-0.730289034	0.22564805	-3.2364074	0.00121045	0.00523866
ENSDARG00000024598	homezb	1553.52247	-0.497333221	0.15367898	-3.23618245	0.0012114	0.00524188
ENSDARG00000029795	prxl2b	696.548098	-0.502849614	0.15538777	-3.23609514	0.00121177	0.00524257
ENSDARG00000058990	slc35a2	582.23546	0.455767123	0.14084587	3.23592816	0.00121248	0.00524435
ENSDARG00000036189	spata4	59.5490159	1.121400255	0.34654988	3.2358986	0.0012126	0.00524435
ENSDARG00000100553	lsm14ab	3801.44067	-0.372619365	0.11517386	-3.23527721	0.00121525	0.00525486
ENSDARG00000006544	c1qtnf6b	167.299499	0.815082248	0.25194985	3.23509714	0.00121601	0.00525635
ENSDARG00000059983	zufsp	247.667731	-0.517998374	0.16011751	-3.23511385	0.00121594	0.00525635
ENSDARG00000074844	CABZ01061495.1	513.730941	0.665048722	0.20558254	3.2349475	0.00121665	0.00525819
ENSDARG00000079175	si:ch211-79k12.1	998.376273	0.459811675	0.14214319	3.23484845	0.00121707	0.0052591
ENSDARG00000101008	soul4	1625.55726	-0.459000714	0.14190696	-3.23451876	0.00121848	0.00526426
ENSDARG00000027590	2-Sep	5219.56894	0.29295586	0.09057387	3.23444109	0.00121881	0.00526478
ENSDARG00000074905	camk1da	595.842669	-0.586363797	0.18130984	-3.23404292	0.00122051	0.00527121
ENSDARG00000089538	rnf166	269.628201	-0.784208438	0.24249326	-3.23393907	0.00122096	0.00527221
ENSDARG00000070606	ikbke	372.15909	0.499683768	0.15453225	3.23352421	0.00122273	0.00527896
ENSDARG00000091348	rtn3	3553.97485	0.371923585	0.11502974	3.23328211	0.00122377	0.00528225
ENSDARG00000103914	THEM6	279.427231	-0.600669701	0.18577911	-3.2332467	0.00122392	0.00528225
ENSDARG00000056468	asb16	142.065452	1.017727658	0.31478457	3.23309258	0.00122458	0.00528419
ENSDARG00000098342	CABZ01065679.1	47.2105033	-1.120571881	0.3466678	-3.23240831	0.00122752	0.00529502
ENSDARG00000036237	slc27a2a	4995.88859	0.577697194	0.17871869	3.23243871	0.00122739	0.00529502
ENSDARG00000000690	sypl2b	488.364785	0.455004673	0.14076602	3.23234743	0.00122778	0.00529523
ENSDARG00000043241	arrb1	374.671757	-0.541570393	0.16756456	-3.2320103	0.00122923	0.00530056
ENSDARG00000017429	sphkap	1050.20422	-0.536418378	0.16598587	-3.23171101	0.00123051	0.00530519
ENSDARG00000099812	si:dkey-31f5.11	714.475487	-0.512979504	0.15874731	-3.23142175	0.00123176	0.00530872
ENSDARG00000005754	ptprfb	9427.5187	0.35910071	0.11112737	3.23143369	0.00123171	0.00530872
ENSDARG00000104290	ripk2	248.246793	0.658897872	0.20391372	3.23125821	0.00123247	0.00531084
ENSDARG00000094760	si:dkey-125i10.3	620.915788	0.523619153	0.1621513	3.22920104	0.00124137	0.00534827
ENSDARG00000060253	si:ch211-216b21.2	88.306651	-0.919683492	0.28480884	-3.22912553	0.00124169	0.00534875
ENSDARG00000060510	cul4b	2792.21762	-0.283946353	0.08794271	-3.22876519	0.00124326	0.00535457
ENSDARG00000022437	cd81b	506.147362	-0.482310322	0.14939445	-3.22843539	0.00124469	0.00535831
ENSDARG00000062995	si:dkey-14o18.2	47.8435091	-1.514183998	0.46900544	-3.22849988	0.00124441	0.00535831
ENSDARG00000031438	kcnj11l	406.427946	-0.626329023	0.19400502	-3.22841657	0.00124478	0.00535831
ENSDARG00000061303	oca2	224.445895	0.626547753	0.19408507	3.22821207	0.00124567	0.00536122
ENSDARG00000043480	rbbp8	181.36486	-0.612432135	0.18972067	-3.22807284	0.00124627	0.0053629
ENSDARG00000079475	nckipsd	2116.39137	0.410390461	0.12713436	3.22800598	0.00124656	0.00536322
ENSDARG00000091667	si:dkey-234i14.3	50.9560293	-1.321116778	0.40931792	-3.22760555	0.00124831	0.00536981
ENSDARG00000097594	FP236741.1	10.6600205	-2.635728834	0.81670457	-3.22727329	0.00124976	0.00537512
ENSDARG00000055901	steap4	660.981662	0.79669029	0.24688349	3.2269889	0.001251	0.00537953
ENSDARG00000094673	rhoab	2667.35412	-0.364792198	0.11304648	-3.22692229	0.00125129	0.00537985
ENSDARG00000054574	znhit1	404.034608	-0.488569071	0.15141357	-3.22671921	0.00125218	0.00538274
ENSDARG00000014572	chpt1	2641.07115	0.450748354	0.13969589	3.22664005	0.00125253	0.0053833
ENSDARG00000103503	dgat1a	622.850836	-0.537329894	0.1665821	-3.22561607	0.00125702	0.00540166
ENSDARG00000036036	mdka	13123.6803	0.293445805	0.09099468	3.22486772	0.00126031	0.00541418
ENSDARG00000088823	crygm2d3	11915.1754	0.477584666	0.14809495	3.2248545	0.00126037	0.00541418
ENSDARG00000097442	si:dkey-77f5.15	21.7197868	-1.973391297	0.61203651	-3.22430321	0.0012628	0.00542367
ENSDARG00000101725	parietopsin	218.231595	0.740177063	0.22957323	3.22414364	0.0012635	0.00542576
ENSDARG00000098433	utp6	756.654431	-0.454148117	0.14088689	-3.22349458	0.00126637	0.00543713
ENSDARG00000078135	mrc2	1009.35313	0.625873917	0.19420759	3.22270578	0.00126986	0.00545067
ENSDARG00000070069	pitx3	537.11073	-0.42680045	0.13243637	-3.22268311	0.00126996	0.00545067
ENSDARG00000098240	meis2a	9532.73802	-0.418126699	0.12974845	-3.22259491	0.00127035	0.00545141
ENSDARG00000006491	agpat9l	524.318728	0.465466515	0.14446269	3.22205351	0.00127275	0.00546078
ENSDARG00000045150	csnk1e	1241.41324	-0.442587129	0.13738565	-3.22149452	0.00127524	0.0054705
ENSDARG00000102696	dnajc11b	562.57561	-0.485426534	0.15069092	-3.22133904	0.00127593	0.00547252
ENSDARG00000074849	rac1a	3438.55847	0.415434637	0.1289798	3.22092791	0.00127776	0.00547943
ENSDARG00000060438	tbl1x	713.932338	-0.458955072	0.14249993	-3.22073898	0.00127861	0.0054821
ENSDARG00000104108	slco1d1	1869.40987	0.491517658	0.1526647	3.21958938	0.00128374	0.00550238
ENSDARG00000013623	vdac2	37877.0337	0.35171686	0.10924304	3.21958156	0.00128378	0.00550238

Table S2. DEGs of WT vs. *terfa-/-*

ENSDARG00000093054	BX548073.3	35.6018121	3.61696225	1.123631	3.21899471	0.00128641	0.00551175
ENSDARG00000031119	baiap2l1b	291.781861	0.519537938	0.16139716	3.2190029	0.00128637	0.00551175
ENSDARG00000005834	gatad2b	1727.097	-0.465169188	0.14451053	-3.21892933	0.0012867	0.00551206
ENSDARG00000110634	CABZ01061508.1	28.0429075	1.557294493	0.48384214	3.21860039	0.00128818	0.00551744
ENSDARG00000100528	map1lc3c	44.7703051	-1.370171464	0.42571294	-3.21853374	0.00128848	0.00551777
ENSDARG00000078130	CR391986.1	88.305589	-1.015596608	0.31560448	-3.21794103	0.00129114	0.00552823
ENSDARG00000056628	tmem170a	273.344534	-0.559211172	0.17379745	-3.21760288	0.00129267	0.0055338
ENSDARG00000101794	atp6v0e1	577.214298	0.47103457	0.14641234	3.21717814	0.00129458	0.00554104
ENSDARG00000006353	itga5	1220.07812	0.411806576	0.12801552	3.21684887	0.00129607	0.00554645
ENSDARG00000088157	smoc2	432.651031	-0.523764061	0.16285225	-3.2161917	0.00129904	0.00555821
ENSDARG00000099124	f3a	363.824414	0.656966059	0.20430367	3.21563513	0.00130156	0.00556804
ENSDARG00000063895	mt-nd1	47735.592	-0.395359276	0.12295303	-3.21553095	0.00130204	0.00556911
ENSDARG00000008247	vezf1a	942.023123	-0.722807395	0.22479444	-3.21541487	0.00130256	0.0055704
ENSDARG00000070522	cacna1ia	140.348127	1.006497296	0.31303045	3.21533348	0.00130293	0.00557102
ENSDARG00000112959	SRL	3549.89342	0.428893928	0.13343367	3.21428554	0.0013077	0.00559043
ENSDARG00000004877	rock2b	1644.48354	0.35634334	0.11088453	3.21364329	0.00131062	0.00560139
ENSDARG00000078691	gigyf1b	2995.16612	-0.282155232	0.08779968	-3.21362474	0.00131071	0.00560139
ENSDARG00000078707	sema7a	174.205952	-0.780179483	0.2428058	-3.21318305	0.00131273	0.00560904
ENSDARG00000078536	si:ch211-158d24.2	733.524774	-0.870743634	0.27101405	-3.21290954	0.00131398	0.00561342
ENSDARG00000037042	ddhd1b	1319.6773	0.455844009	0.14189207	3.2126109	0.00131534	0.0056183
ENSDARG00000058647	hck	108.269063	0.807603643	0.2513991	3.21243649	0.00131614	0.00562074
ENSDARG00000102234	si:ch211-71n6.4	558.392184	0.593663408	0.1848047	3.21238268	0.00131639	0.00562083
ENSDARG00000071086	gpr155b	353.314901	-0.579023454	0.18025282	-3.2122851	0.00131684	0.00562177
ENSDARG00000099079	aclya	5255.83433	0.281864199	0.08775341	3.21200293	0.00131813	0.00562633
ENSDARG00000023302	gpbar1	24.3290854	1.531018789	0.47666307	3.21195176	0.00131837	0.00562637
ENSDARG00000016598	ckmt1	8342.34408	0.342755157	0.10673786	3.21118644	0.00132188	0.00564041
ENSDARG00000019746	rfng	647.204392	-0.442762754	0.13788493	-3.2111033	0.00132226	0.00564107
ENSDARG00000105665	FO704741.1	37.2466439	1.419787642	0.44217452	3.21092144	0.0013231	0.00564367
ENSDARG00000079549	cdc42ep1a	230.210206	-0.562555922	0.17520453	-3.2108527	0.00132342	0.00564405
ENSDARG00000086990	carmil3	846.968885	-0.598773273	0.18650796	-3.21044356	0.0013253	0.00565112
ENSDARG00000042904	foxo3b	6444.88888	0.371257091	0.11565147	3.21013727	0.00132672	0.00565618
ENSDARG00000117240	CABZ01083943.1	599.562545	0.511164816	0.15924369	3.20995343	0.00132757	0.00565883
ENSDARG00000080020	il13ra1	292.005145	0.585941042	0.18254398	3.20986233	0.00132799	0.00565965
ENSDARG00000042477	nr2c2	1155.76651	-0.34314826	0.10693093	-3.20906467	0.00133168	0.0056744
ENSDARG00000040443	polr2i	399.785389	-0.49135002	0.15312355	-3.20884691	0.00133268	0.00567773
ENSDARG00000071437	ptprc	104.714477	-0.928285822	0.28931622	-3.20855096	0.00133406	0.0056826
ENSDARG00000070922	cnbpb	7559.52366	-0.281034806	0.08759997	-3.20816104	0.00133587	0.00568934
ENSDARG00000045482	stk38l	670.786094	-0.395502838	0.12328243	-3.20810392	0.00133613	0.00568949
ENSDARG00000099226	CABZ01076667.1	2180.87334	0.315966935	0.09849786	3.20785597	0.00133729	0.00569342
ENSDARG00000106631	tmem63bb	263.12999	-0.645860223	0.20134328	-3.20775654	0.00133775	0.00569442
ENSDARG00000074506	tmem94	2167.95206	0.472566008	0.14732753	3.2075879	0.00133853	0.00569678
ENSDARG00000098403	CR925723.1	15.0129251	-3.39887534	1.06023741	-3.20576816	0.00134703	0.00573195
ENSDARG00000069505	zgc:136892	245.030087	0.510913215	0.15937661	3.20569768	0.00134736	0.00573237
ENSDARG00000018178	pgm2	1933.51631	-0.362206946	0.11299672	-3.20546439	0.00134845	0.00573604
ENSDARG00000079329	cop1	876.297033	-0.383040167	0.11950441	-3.20523872	0.00134951	0.00573955
ENSDARG00000015206	hnrnpua	3795.63841	-0.400837584	0.12507213	-3.20485134	0.00135132	0.0057463
ENSDARG00000067848	nmrk2	1160.79855	0.808420799	0.25226872	3.20460187	0.00135249	0.00575013
ENSDARG00000041381	arntl2	1125.97123	-0.450467983	0.14057089	-3.20456099	0.00135269	0.00575013
ENSDARG00000101272	si:dkey-29j8.1	20.6439448	2.062541033	0.64367223	3.20433437	0.00135375	0.00575325
ENSDARG00000042025	C9H2orf49	366.573326	-0.453889638	0.14164988	-3.20430661	0.00135388	0.00575325
ENSDARG00000054980	ebna1bp2	909.286533	-0.470068746	0.14670158	-3.20425149	0.00135414	0.00575337
ENSDARG00000059931	hyal2a	131.938141	0.714069212	0.22285588	3.20417491	0.0013545	0.00575391
ENSDARG00000056621	ctcf	6203.30389	-0.44597658	0.13919515	-3.20396642	0.00135548	0.0057571
ENSDARG00000079544	si:ch1073-464p5.5	468.386024	0.949690445	0.2964217	3.20384927	0.00135603	0.00575846
ENSDARG00000078717	itga8	1117.90582	0.391674515	0.12226358	3.2035257	0.00135756	0.00576395
ENSDARG00000037059	slc44a2	2096.74172	-0.397673661	0.12414808	-3.20322051	0.001359	0.00576907
ENSDARG00000028586	gpr135	150.552254	-0.787919782	0.24598721	-3.20309251	0.0013596	0.00577003
ENSDARG00000058880	cldnj	135.758101	0.639069874	0.19951765	3.20307435	0.00135969	0.00577003

Table S2. DEGs of WT vs. *terfa-/-*

ENSDARG00000068814	spata2l	152.97841	0.676380444	0.21117989	3.20286388	0.00136068	0.00577326
ENSDARG00000099958	BX957318.1	31.3913226	1.519367173	0.47471292	3.20060209	0.00137141	0.00581777
ENSDARG00000104307	hoxa1a	144.237019	0.858777738	0.26833914	3.20034464	0.00137263	0.00582198
ENSDARG00000006508	pip5k1bb	455.193454	0.41632325	0.13011346	3.19969391	0.00137574	0.00583414
ENSDARG00000070721	vdrb	762.214361	0.73683422	0.23033183	3.19901171	0.001379	0.00584697
ENSDARG00000061228	pfdn4	876.062677	0.391581907	0.12243015	3.19841076	0.00138187	0.00585817
ENSDARG00000010791	dla	1096.46926	-1.24538944	0.3893983	-3.1982406	0.00138269	0.00586063
ENSDARG00000037647	rnf175	1110.37675	0.3980452	0.12445974	3.1981844	0.00138296	0.00586077
ENSDARG00000105293	srsf4	4581.20511	-0.412317924	0.12893934	-3.19776671	0.00138496	0.00586812
ENSDARG00000007975	fth1b	731.35979	-0.591705872	0.18503965	-3.19772482	0.00138516	0.00586812
ENSDARG00000036100	eya3	1232.17995	-0.378027902	0.11824945	-3.19686824	0.00138928	0.00588357
ENSDARG00000117259	BX957317.3	11.3447162	-2.55555663	0.79938605	-3.19689922	0.00138914	0.00588357
ENSDARG00000103266	ogal	144.422905	0.761581653	0.23825793	3.19645879	0.00139126	0.00589092
ENSDARG00000063474	arfgef1	2543.73994	0.310839535	0.09725333	3.19618386	0.00139258	0.00589553
ENSDARG00000097451	chmp6a	18.8097364	-2.942000106	0.92056043	-3.19587938	0.00139405	0.00590075
ENSDARG00000018281	cfap161	30.6964967	1.495770911	0.46807223	3.19559846	0.00139541	0.00590549
ENSDARG00000016835	tcirg1a	582.106135	-0.440316001	0.13779592	-3.19542115	0.00139627	0.00590711
ENSDARG00000026967	ap2s1	4071.96112	-0.274799777	0.08599764	-3.19543399	0.00139621	0.00590711
ENSDARG00000056774	gpr37l1b	641.966109	-0.543888647	0.1702224	-3.19516505	0.00139751	0.00591034
ENSDARG00000088740	hmgcll1	30.0444202	1.696485824	0.53095285	3.19517225	0.00139747	0.00591034
ENSDARG00000053810	hnrnpc	2810.74407	-0.352235339	0.11026675	-3.19439313	0.00140125	0.00592516
ENSDARG00000062448	skor1b	426.856386	-0.472417042	0.1478984	-3.1941999	0.00140219	0.00592737
ENSDARG00000063310	oxr1b	1335.35374	-0.426318354	0.13346693	-3.19418726	0.00140225	0.00592737
ENSDARG00000039937	ccdc172	15.8715971	-2.011873458	0.63002978	-3.19329898	0.00140657	0.00594463
ENSDARG00000099656	CR381657.1	4.30456662	5.288149257	1.65606072	3.19320976	0.00140701	0.00594545
ENSDARG00000069135	ppp1r15a	1414.03921	-0.410462151	0.12854904	-3.19303953	0.00140784	0.00594795
ENSDARG00000103947	zgc:113295	147.354113	0.860918335	0.26964672	3.192764	0.00140918	0.00595262
ENSDARG00000101188	CABZ01048053.1	411.667781	0.579745609	0.1816315	3.19187805	0.00141351	0.00596989
ENSDARG00000052893	rx3	231.950106	-0.877683172	0.27498904	-3.19170235	0.00141437	0.00597251
ENSDARG00000067711	parvg	90.5114857	0.87055254	0.27278477	3.19135312	0.00141608	0.00597871
ENSDARG00000086699	smim15	893.785376	-0.395881092	0.12406334	-3.19095951	0.00141801	0.00598382
ENSDARG00000079076	hgfb	165.031767	0.634945159	0.19898184	3.19097034	0.00141796	0.00598382
ENSDARG00000011743	pigo	1102.22513	-0.358633372	0.11239017	-3.19096759	0.00141797	0.00598382
ENSDARG00000094894	tyrobp	10.1318285	2.614208325	0.81930068	3.19078011	0.00141889	0.00598652
ENSDARG00000070604	zgc:162509	67.21793	1.229967379	0.385497	3.19060166	0.00141977	0.0059892
ENSDARG00000100452	nop53	3368.87252	-0.406261041	0.12734301	-3.19028933	0.0014213	0.00599466
ENSDARG00000074323	efcab2	21.7772912	1.858910912	0.58290373	3.18905302	0.0014274	0.00601933
ENSDARG00000059070	gars1	6157.17918	0.373363992	0.11708343	3.18887121	0.0014283	0.0060221
ENSDARG00000006044	usta	329.911183	0.527617638	0.16546895	3.18862028	0.00142954	0.00602631
ENSDARG00000062226	ino80db	1227.60718	-0.508163227	0.15938756	-3.1882239	0.0014315	0.00603355
ENSDARG00000111882	si:ch211-194m7.4	3.82072743	5.230993335	1.64110262	3.18748704	0.00143515	0.00604792
ENSDARG00000078954	rxrba	2068.92318	-0.362168515	0.11362736	-3.18733536	0.0014359	0.00605007
ENSDARG00000097057	fxyd6l	3506.70902	-0.473972764	0.14871335	-3.18715683	0.00143679	0.00605278
ENSDARG00000038563	pitrm1	2099.65286	0.268591258	0.08428127	3.18684402	0.00143834	0.00605815
ENSDARG00000036051	irx4b	163.867072	-0.824514077	0.25872774	-3.18680195	0.00143855	0.00605815
ENSDARG00000101115	med30	379.557351	0.511301737	0.1604548	3.18657797	0.00143967	0.00606182
ENSDARG00000088915	si:ch211-241n15.3	46.3036212	1.459695211	0.45809335	3.18645798	0.00144026	0.00606331
ENSDARG00000011661	twf1b	1106.03446	-0.331720964	0.10410906	-3.18628334	0.00144113	0.00606594
ENSDARG00000061691	qpctla	677.38728	0.401032034	0.12588813	3.18562235	0.00144443	0.00607879
ENSDARG00000018553	ifih1	76.8767668	1.129229766	0.35451132	3.18531372	0.00144597	0.00608424
ENSDARG00000036700	si:ch211-114n24.6	1581.27869	1.081288857	0.33953414	3.18462483	0.00144942	0.00609668
ENSDARG00000019367	tgfb3	2377.94797	0.322482877	0.10126163	3.18465038	0.00144929	0.00609668
ENSDARG00000059503	ACSF3	328.622203	0.614229946	0.19287759	3.18455831	0.00144975	0.00609705
ENSDARG00000025032	nr3c1	2647.01687	-0.298662976	0.09378775	-3.18445628	0.00145026	0.00609817
ENSDARG00000058533	pole	1201.74386	-0.558512598	0.17539994	-3.18422342	0.00145143	0.00610204
ENSDARG00000040387	mmd	1074.83079	0.365549494	0.11481915	3.18369801	0.00145407	0.0061121
ENSDARG00000039499	soul2	1242.48071	0.440564978	0.13842824	3.18262366	0.00145947	0.00613274
ENSDARG00000041107	cftr	177.26716	0.81446124	0.25590836	3.18262845	0.00145945	0.00613274

Table S2. DEGs of WT vs. *terfa-/-*

ENSDARG00000042990	cyp2p10	106.595439	-1.212702892	0.38107045	-3.18235882	0.00146081	0.00613732
ENSDARG00000078226	cdh12a	249.252838	-0.753178214	0.23670091	-3.18198278	0.00146271	0.00614425
ENSDARG00000097448	CR925728.3	7.38898386	-3.987244487	1.2532876	-3.18142818	0.00146551	0.00615499
ENSDARG00000068483	neto1	103.842858	-0.960059712	0.30177982	-3.18132506	0.00146603	0.0061551
ENSDARG00000110266	si:ch211-200p22.4	909.61082	-0.540131892	0.16978175	-3.18133064	0.001466	0.0061551
ENSDARG00000054063	arpc4	2371.30504	0.327342204	0.10289963	3.18117959	0.00146677	0.00615715
ENSDARG00000077905	csmd3a	373.364855	0.753203817	0.23678317	3.1809855	0.00146775	0.00615816
ENSDARG00000007737	zyg11	2455.9564	0.413883427	0.13010892	3.18105343	0.00146741	0.00615816
ENSDARG00000060471	gcnt3	24.7289207	1.746908264	0.54916797	3.18100902	0.00146763	0.00615816
ENSDARG00000025428	socs3a	1439.91729	1.344480122	0.42281264	3.17984845	0.00147352	0.00618133
ENSDARG00000020232	eif6	1675.33796	0.402168695	0.1264868	3.179531	0.00147514	0.00618706
ENSDARG00000010192	pax3a	221.647369	-0.592290204	0.18630795	-3.17909246	0.00147737	0.00619434
ENSDARG00000094563	znf1144	135.236576	-0.715456492	0.2250494	-3.17910871	0.00147729	0.00619434
ENSDARG00000074153	slitrk5b	107.078248	-0.894605206	0.28140796	-3.17903301	0.00147767	0.00619456
ENSDARG00000035564	dgcr8	2419.89034	-0.342071266	0.10762452	-3.17837667	0.00148102	0.00620756
ENSDARG00000090366	znf1065	55.36225	1.289716759	0.40579371	3.17825713	0.00148163	0.00620907
ENSDARG00000075916	zgc:66472	420.448194	-0.459760013	0.14469369	-3.17747099	0.00148566	0.00622383
ENSDARG00000098057	dscaml1	2691.30239	-0.749883843	0.23599883	-3.17748964	0.00148556	0.00622383
ENSDARG00000092143	ank1a	749.474904	0.45420591	0.14297104	3.1769086	0.00148854	0.00623486
ENSDARG00000041502	tgfb1a	982.649503	0.428553459	0.13492597	3.17621178	0.00149212	0.0062488
ENSDARG00000113820	cyp46a1.4	118.118583	0.866934033	0.27299483	3.17564263	0.00149505	0.00626002
ENSDARG00000109996	CABZ01101996.1	108.483034	-1.0066097	0.3170134	-3.17529066	0.00149687	0.00626656
ENSDARG00000009779	mcl1a	2146.45094	0.410486832	0.12930542	3.17455247	0.00150068	0.00628041
ENSDARG00000057039	si:ch211-191j22.8	32.8340276	-1.569770742	0.49448047	-3.17458593	0.00150051	0.00628041
ENSDARG00000110978	ftr32	8.1273738	3.257333882	1.02610323	3.17446995	0.0015011	0.00628114
ENSDARG00000027777	tnfaip3	536.577848	0.540740361	0.17035043	3.1742824	0.00150208	0.00628414
ENSDARG00000053526	enpp7.1	16.0771875	2.877420984	0.90663867	3.17372407	0.00150497	0.00629518
ENSDARG00000010583	pard3ab	1901.61114	0.336746544	0.10612248	3.17318781	0.00150775	0.00630576
ENSDARG00000058159	ipo8	1302.12985	0.787173216	0.24812322	3.17250923	0.00151128	0.00631945
ENSDARG00000103727	tfr2	148.645753	0.867239498	0.27336765	3.17242919	0.00151169	0.00632013
ENSDARG00000086218	nkd3	4.65937258	-5.519355311	1.7398533	-3.17231074	0.00151231	0.00632164
ENSDARG00000078659	tmem176	685.886216	0.686451552	0.21639759	3.17217751	0.00151301	0.00632246
ENSDARG00000018121	cenpj	230.231089	-0.714422882	0.22521615	-3.17216536	0.00151307	0.00632246
ENSDARG00000117411	CT997819.5	94.4336984	-0.90046052	0.28386652	-3.17212659	0.00151327	0.00632246
ENSDARG00000000666	creg2	1248.94309	0.439614132	0.1385964	3.17190145	0.00151444	0.0063263
ENSDARG00000078076	nab2	136.582742	0.708634953	0.22341498	3.17183281	0.0015148	0.00632657
ENSDARG00000079765	si:ch211-112f3.4	292.190267	0.572313388	0.18043853	3.17179153	0.00151502	0.00632657
ENSDARG00000089861	usp44	948.322568	-0.373337136	0.11773065	-3.17111251	0.00151856	0.00633956
ENSDARG00000099844	GNAZ	936.135524	-0.465312689	0.14673549	-3.17109847	0.00151864	0.00633956
ENSDARG00000078701	prdm13	618.028511	-1.503338276	0.474098	-3.17094414	0.00151944	0.00634186
ENSDARG00000063155	dcaf12	1396.50405	-0.346114698	0.10917569	-3.17025442	0.00152306	0.00635587
ENSDARG00000090697	eif3ea	12824.2153	-0.320645402	0.1011592	-3.16971073	0.00152591	0.0063667
ENSDARG00000100475	fgf10b	30.0813065	-1.555558359	0.49077862	-3.1695724	0.00152663	0.00636866
ENSDARG00000006300	psmd9	483.69134	0.56335304	0.17775138	3.16933151	0.0015279	0.00637287
ENSDARG00000079347	zgc:194659	266.615735	-0.697165286	0.21998473	-3.16915303	0.00152884	0.00637506
ENSDARG00000071425	nat9	732.034263	-0.396534881	0.12512403	-3.16913449	0.00152894	0.00637506
ENSDARG00000076811	ganab	3976.03082	0.334020606	0.10540263	3.16899702	0.00152966	0.00637593
ENSDARG00000020761	arrdc2	2197.6765	0.639566022	0.20181884	3.16901045	0.00152959	0.00637593
ENSDARG00000025728	grin1b	6178.01623	-0.709885213	0.22402106	-3.16883243	0.00153053	0.00637751
ENSDARG00000021398	mul1a	614.349825	-0.543904347	0.17164212	-3.1688279	0.00153055	0.00637751
ENSDARG00000003446	ippk	777.161639	0.418633542	0.13212157	3.16854799	0.00153203	0.00638258
ENSDARG00000058822	wnt3a	110.128051	0.825850495	0.26066524	3.1682418	0.00153364	0.00638696
ENSDARG00000076780	acadsb	2010.85808	0.337134694	0.10641197	3.16820261	0.00153385	0.00638696
ENSDARG00000030402	twist1a	644.520641	0.402404157	0.12701207	3.16823549	0.00153367	0.00638696
ENSDARG00000062446	neurl2	166.113268	0.724132023	0.22858673	3.16786559	0.00153563	0.00639329
ENSDARG00000027418	trappc4	804.693034	-0.403023956	0.12722638	-3.16777025	0.00153613	0.00639432
ENSDARG00000062674	btbd10a	2241.72631	-0.484044835	0.15282072	-3.16740321	0.00153807	0.00640132
ENSDARG00000074169	gpam	2024.27439	-0.401974261	0.1269289	-3.16692472	0.0015406	0.00640994

Table S2. DEGs of WT vs. *terfa-/-*

ENSDARG00000069766	caln2	134.797121	-0.788622582	0.24901921	-3.16691465	0.00154066	0.00640994
ENSDARG00000112728	KLHL12	350.849401	0.499520765	0.15774779	3.16657851	0.00154244	0.00641628
ENSDARG00000035751	ipo7	9526.63869	0.224453092	0.07088498	3.16644099	0.00154317	0.00641824
ENSDARG00000059804	retreg3	963.727577	-0.378491333	0.11953637	-3.16632785	0.00154377	0.00641966
ENSDARG00000074340	serf2	4096.64058	0.331694802	0.10477393	3.16581438	0.00154649	0.00642993
ENSDARG00000102575	si:ch211-106j21.2	46.1393615	-3.526004655	1.11391821	-3.16540715	0.00154866	0.00643786
ENSDARG00000073909	ppip5k1b	1175.5662	0.414776233	0.13105899	3.16480578	0.00155187	0.0064501
ENSDARG00000104347	rtn4b	4631.67461	0.361956815	0.11438958	3.16424647	0.00155485	0.00646143
ENSDARG00000037009	banf1	2921.99169	-0.384089038	0.12139761	-3.16389297	0.00155674	0.0064682
ENSDARG00000024895	fam50a	845.423448	-0.437269006	0.13823175	-3.16330369	0.0015599	0.00648023
ENSDARG00000077352	b4galnt1b	578.861105	-0.504482424	0.15949522	-3.1629941	0.00156156	0.00648604
ENSDARG00000010267	dpydb	2633.29342	0.356370234	0.11268196	3.1626201	0.00156356	0.00649329
ENSDARG00000021336	pax4	29.5892319	-1.807753529	0.5716136	-3.16254467	0.00156397	0.00649389
ENSDARG00000037861	slc2a3b	108.563724	1.085548852	0.34326354	3.16243561	0.00156455	0.00649524
ENSDARG00000044492	ublcp1	496.60508	-0.483082651	0.15278272	-3.1618932	0.00156747	0.00650626
ENSDARG00000013860	ptpmt1	308.907233	0.499262661	0.15793452	3.16120032	0.0015712	0.00652067
ENSDARG00000062025	cpox	716.776489	0.446530203	0.14125692	3.16112093	0.00157163	0.00652136
ENSDARG00000004459	unc119.2	31.2566441	-1.492641845	0.47225694	-3.16065626	0.00157414	0.00652821
ENSDARG00000033446	tap2t	174.107746	0.675983036	0.21387514	3.16064329	0.00157421	0.00652821
ENSDARG00000003680	runx1t1	3827.32696	-0.49034772	0.15514286	-3.16062069	0.00157433	0.00652821
ENSDARG00000007302	sh3gl3b	431.665401	-0.519648354	0.16440752	-3.1607335	0.00157372	0.00652821
ENSDARG00000101687	CU929259.1	857.591006	0.60364753	0.19099919	3.16047161	0.00157514	0.00653046
ENSDARG00000095698	si:dkey-228a15.1	144.836437	1.899100144	0.60094672	3.16018056	0.00157671	0.0065359
ENSDARG00000039424	snrpb2	786.234946	-0.357799872	0.11323244	-3.15987075	0.00157839	0.00654176
ENSDARG00000059567	si:ch73-281f12.4	536.468669	0.431080424	0.13644921	3.15927389	0.00158163	0.00655408
ENSDARG00000077112	si:ch211-180f4.1	683.155408	-0.606698962	0.19206955	-3.15874626	0.00158449	0.00656486
ENSDARG00000039989	snrnp48	293.670751	-0.528625166	0.16735666	-3.15867431	0.00158489	0.00656539
ENSDARG00000076357	CABZ01102039.1	729.323952	-0.411618072	0.13037432	-3.15720208	0.00159291	0.00659753
ENSDARG00000099423	CR391930.1	2.85446445	5.249037129	1.66297539	3.15641299	0.00159723	0.0066143
ENSDARG00000045898	si:ch211-152c2.3	1475.05923	-0.5203184	0.1648892	-3.15556393	0.00160188	0.00663248
ENSDARG00000007823	atf3	639.784633	0.620513103	0.19665436	3.15534884	0.00160306	0.00663627
ENSDARG00000032221	phactr4b	1118.27939	-0.382256224	0.12115694	-3.15505008	0.00160471	0.00664196
ENSDARG00000027523	trpc7a	48.8243964	-1.404862957	0.44528664	-3.15496319	0.00160518	0.00664283
ENSDARG00000056559	ccz1	946.089461	-0.36728302	0.11643205	-3.1544839	0.00160782	0.00665264
ENSDARG00000079446	TAFA3	270.632588	-0.533522731	0.1691525	-3.15409309	0.00160998	0.00666045
ENSDARG00000098121	FP101882.1	221.299754	-0.821467601	0.26046322	-3.15387176	0.0016112	0.00666439
ENSDARG00000104559	tmem63ba	1710.4074	0.495029241	0.15696568	3.15374187	0.00161192	0.00666625
ENSDARG00000070746	sft2d2a	86.0792955	0.876914192	0.2781189	3.1530191	0.00161591	0.00668167
ENSDARG00000077072	si:ch73-280o22.2	282.462189	0.54555473	0.17304661	3.15264624	0.00161798	0.00668798
ENSDARG00000002168	tra2b	1382.67525	-0.3764409	0.11940408	-3.15266365	0.00161788	0.00668798
ENSDARG00000096932	si:ch211-37e10.2	753.198899	0.398626548	0.12644788	3.15249691	0.00161881	0.00669029
ENSDARG00000037145	slc8a4b	578.445068	-0.521657324	0.16547694	-3.15244726	0.00161908	0.00669032
ENSDARG00000102318	il6	3.01219017	5.683350277	1.8032021	3.15180993	0.00162262	0.00670382
ENSDARG00000053383	rprma	75.3287467	-0.967624256	0.30702776	-3.1515856	0.00162387	0.00670786
ENSDARG00000039959	gdnfa	127.51778	-0.676541286	0.21472308	-3.15076191	0.00162845	0.00672568
ENSDARG00000103254	si:dkeyp-80d11.4	12.056179	2.758467826	0.87557075	3.15047967	0.00163003	0.00673107
ENSDARG00000038000	crls1	559.792438	0.403544902	0.12810617	3.15008162	0.00163225	0.00673801
ENSDARG00000068926	si:ch211-137a8.2	197.59786	-0.762293701	0.24199003	-3.1501037	0.00163213	0.00673801
ENSDARG00000099116	nagk	485.270805	0.410839978	0.13042431	3.15002616	0.00163256	0.00673817
ENSDARG00000090286	serpina1	12604.5036	-0.347361603	0.11030466	-3.14911079	0.00163768	0.00675819
ENSDARG00000099702	ahrra	86.2213605	-0.927335061	0.29449464	-3.14890304	0.00163885	0.00676187
ENSDARG00000104528	cldn10b	84.0966789	-1.034318259	0.32848	-3.14880136	0.00163942	0.0067631
ENSDARG00000063544	pip4k2ab	637.857165	-0.589417897	0.18720075	-3.1485872	0.00164062	0.00676694
ENSDARG00000074640	FIBCD1	24.2518323	-1.706693649	0.54209322	-3.14833979	0.00164201	0.00677154
ENSDARG00000078807	paxx	111.949488	-0.768141069	0.24401364	-3.14794317	0.00164424	0.00677961
ENSDARG00000061466	cpne5a	233.964619	-0.764547442	0.24289158	-3.14769017	0.00164566	0.00678291
ENSDARG00000016238	arl6ip5b	883.373517	0.542606089	0.17238147	3.14770535	0.00164558	0.00678291
ENSDARG00000036785	si:ch211-226m16.[illegible]	1043.35971	0.441922569	0.14039737	3.14765558	0.00164586	0.00678291

Table S2. DEGs of WT vs. *terfa-/-*

ENSDARG00000052734	hmgcra	590.882832	-0.656484285	0.20861408	-3.14688385	0.00165021	0.00679858
ENSDARG00000060482	mid1	422.430337	-0.87870184	0.27922492	-3.14693203	0.00164993	0.00679858
ENSDARG00000017173	si:dkey-29p10.4	544.813568	-0.519587797	0.16515734	-3.14601691	0.0016551	0.00681764
ENSDARG00000004460	desi2	808.75697	-0.387093752	0.1230468	-3.14590669	0.00165573	0.00681867
ENSDARG00000009273	ppm1da	1106.55755	-0.501327617	0.15936028	-3.14587564	0.0016559	0.00681867
ENSDARG00000008678	snx3	1492.64846	0.393592178	0.12514986	3.14496709	0.00166106	0.00683762
ENSDARG00000102511	cript	583.896818	-0.434192549	0.13805931	-3.14497117	0.00166103	0.00683762
ENSDARG00000039521	ssx2ipa	766.775205	-0.50859414	0.16173759	-3.14456366	0.00166335	0.00684592
ENSDARG00000071881	arl9	665.863854	-0.497830094	0.1583186	-3.14448277	0.00166381	0.00684668
ENSDARG00000029058	rbbp4	4735.19813	-0.518201924	0.16482699	-3.14391424	0.00166704	0.00685886
ENSDARG00000077410	myo9b	1639.70041	0.428946257	0.1364586	3.14341683	0.00166988	0.00686939
ENSDARG00000099984	CABZ01023255.1	145.265236	0.802277036	0.25525709	3.14301574	0.00167217	0.00687767
ENSDARG00000018898	zfand5a	2538.23415	0.592275177	0.18847253	3.14250128	0.00167511	0.00688863
ENSDARG00000098573	sgcd	1486.30039	0.472683543	0.15042226	3.14237767	0.00167582	0.0068904
ENSDARG00000100334	lat	15.5072091	-1.961669368	0.62429593	-3.14221075	0.00167677	0.00689318
ENSDARG00000013122	maf1	1109.76809	-0.552620164	0.17592144	-3.14128951	0.00168206	0.00691376
ENSDARG00000078822	ccdc18	299.027737	-0.526455097	0.16762313	-3.1407067	0.00168541	0.00692639
ENSDARG00000099194	CABZ01058261.1	2378.53396	-0.581267787	0.18508369	-3.14056736	0.00168621	0.00692854
ENSDARG00000076001	pip5k1ca	497.969972	-0.486596568	0.15494725	-3.14040138	0.00168717	0.00693132
ENSDARG00000018060	pik3r2	2371.91286	0.370259753	0.11792962	3.13966719	0.0016914	0.00694757
ENSDARG00000104008	si:ch211-232d10.3	17.5760909	2.357936976	0.75104215	3.13955346	0.00169206	0.00694911
ENSDARG00000052450	hspb2	255.205229	0.702979729	0.22393073	3.13927322	0.00169368	0.00695461
ENSDARG00000029587	msra	290.693209	-0.555667813	0.17701201	-3.13915324	0.00169437	0.00695549
ENSDARG00000042899	ELAPOR1	275.697104	-0.637264921	0.20300624	-3.1391396	0.00169445	0.00695549
ENSDARG00000034808	kcnip1b	640.499644	-0.439573966	0.14004396	-3.13882847	0.00169625	0.00696173
ENSDARG00000013004	tdg.1	1588.7969	-0.533089884	0.16984338	-3.13871456	0.00169691	0.00696328
ENSDARG00000022525	mchr1b	123.823147	3.057854164	0.97426577	3.13862426	0.00169743	0.00696428
ENSDARG00000070391	tspan4b	599.55339	0.495498038	0.15788255	3.13839649	0.00169875	0.00696855
ENSDARG00000077009	wdfy4	422.124586	0.430768296	0.13727463	3.13800369	0.00170103	0.00697674
ENSDARG00000033437	rps6ka1	1294.29127	0.346465213	0.11043743	3.13720815	0.00170565	0.00699454
ENSDARG00000021380	ppm1db	526.496307	-0.578533902	0.18442663	-3.13693258	0.00170725	0.00699997
ENSDARG00000006921	lrp5	3862.5344	0.378824628	0.12076571	3.13685601	0.0017077	0.00700064
ENSDARG00000115869	ATP9A	335.657378	0.528347644	0.16847335	3.13609041	0.00171216	0.00701778
ENSDARG00000079440	coro2ba	986.861388	-0.450397599	0.1436554	-3.13526402	0.001717	0.00703642
ENSDARG00000001897	man2b1	1768.97747	0.450982283	0.14385445	3.13499018	0.0017186	0.00704184
ENSDARG00000038475	acy1	961.083348	0.372530774	0.11883488	3.13486044	0.00171936	0.00704379
ENSDARG00000104342	csnk1g1	1564.80403	0.285993196	0.0912394	3.13453626	0.00172126	0.00704987
ENSDARG00000092407	zmp:0000001316	61.8383112	0.92727317	0.29582709	3.13451067	0.00172141	0.00704987
ENSDARG00000060207	traf7	962.70145	0.400433843	0.12776846	3.13405874	0.00172406	0.00705841
ENSDARG00000005716	olfml2ba	458.839227	-0.524366748	0.16731022	-3.13409878	0.00172383	0.00705841
ENSDARG00000092920	si:ch211-106h4.12	248.784818	-0.607884418	0.19397426	-3.13384066	0.00172535	0.00706249
ENSDARG00000087625	si:ch211-173a9.6	194.24998	-1.094421987	0.34924607	-3.13367013	0.00172635	0.00706427
ENSDARG00000091092	zcchc10	274.98278	-0.56261951	0.1795374	-3.13371764	0.00172607	0.00706427
ENSDARG00000097959	si:dkey-248g15.3	2237.99257	0.999414275	0.31895627	3.1333897	0.001728	0.00706892
ENSDARG00000037432	haus7	302.017106	-0.555922768	0.17741949	-3.13338051	0.00172805	0.00706892
ENSDARG00000002670	ATG14	911.872002	-0.33709528	0.10759285	-3.13306391	0.00172992	0.00707538
ENSDARG00000010601	mtmr10	815.377712	-0.379011474	0.12097355	-3.13301122	0.00173023	0.00707549
ENSDARG00000116086	si:dkey-193i10.4	9.48002027	3.079592984	0.98305502	3.13267613	0.00173221	0.00708241
ENSDARG00000008153	serinc5	2064.70388	-0.273849266	0.08742985	-3.13221692	0.00173492	0.00709233
ENSDARG00000102379	si:zfos-80g12.1	768.844246	0.349010395	0.11144968	3.13155142	0.00173885	0.00710725
ENSDARG00000090232	clpb	646.348514	0.408573164	0.13047251	3.13148857	0.00173923	0.00710761
ENSDARG00000004227	pde3a	1211.29298	0.462214433	0.14761612	3.13119224	0.00174098	0.00711361
ENSDARG00000103979	calr3a	6278.12516	0.298298165	0.09530162	3.13004313	0.00174781	0.00714032
ENSDARG00000078864	lats2	1500.95082	0.426579106	0.13629856	3.12974049	0.00174961	0.00714651
ENSDARG00000043283	prrg1	767.583367	-0.419978868	0.13420111	-3.12947381	0.0017512	0.00715182
ENSDARG00000045827	lyrm5b	27.1095059	1.828399329	0.58435579	3.12891455	0.00175453	0.00716427
ENSDARG00000033411	cabp1b	105.916014	-1.319090813	0.42162169	-3.12861231	0.00175634	0.00717047
ENSDARG00000071001	nuggc.1	11.6314606	-3.347890013	1.07015164	-3.12842582	0.00175745	0.00717384

Table S2. DEGs of WT vs. *terfa-/-*

ENSDARG00000063233	baz1a	456.139638	-0.391361906	0.12511289	-3.12807031	0.00175958	0.00718134
ENSDARG00000086183	vkorc1	130.45697	0.785908799	0.25128643	3.12754174	0.00176275	0.00719309
ENSDARG00000077165	kcnj3a	264.707991	-0.597043222	0.19092833	-3.12705419	0.00176567	0.00720385
ENSDARG00000077590	arsj	369.697981	-0.614432931	0.19649523	-3.12696106	0.00176623	0.00720494
ENSDARG00000004869	penka	185.97686	-0.795433219	0.25440724	-3.12661395	0.00176832	0.00721227
ENSDARG00000070231	polh	510.132898	-0.474005549	0.1516445	-3.12576825	0.00177341	0.00723186
ENSDARG00000028259	aldh3a2a	675.332946	0.391975846	0.12541094	3.12553153	0.00177484	0.00723649
ENSDARG00000005210	coro2a	832.168037	0.381897756	0.12222939	3.12443472	0.00178147	0.00726233
ENSDARG00000009280	smyd1a	6158.81018	0.450401298	0.144173	3.12403358	0.0017839	0.00727105
ENSDARG00000095090	tspan37	132.729312	0.75783584	0.24262764	3.1234522	0.00178743	0.00728423
ENSDARG00000036245	wipf3	216.002392	0.679541009	0.21758161	3.12315459	0.00178924	0.00729041
ENSDARG00000008022	kif18a	419.050596	-0.805373264	0.25790253	-3.12278159	0.00179151	0.00729846
ENSDARG00000068883	tspan13a	467.101711	-0.727354296	0.23293121	-3.12261419	0.00179253	0.00730141
ENSDARG00000098258	SLC16A7	522.141402	-0.686172156	0.21976982	-3.12223099	0.00179486	0.00730973
ENSDARG00000069425	hsbp1a	977.226291	-0.47911291	0.15345489	-3.12217427	0.00179521	0.00730994
ENSDARG00000039302	terfa	1087.6095	-0.461677322	0.14787443	-3.12209032	0.00179572	0.00731068
ENSDARG00000069660	chrnb1	992.511188	0.506866268	0.16235058	3.12204777	0.00179598	0.00731068
ENSDARG00000087573	hdac11	81.191209	0.892637574	0.28593067	3.1218672	0.00179708	0.00731397
ENSDARG00000054879	six3b	1020.90425	-0.691874457	0.2216512	-3.12145591	0.00179959	0.007323
ENSDARG00000042659	thyn1	109.857579	1.099529785	0.35233477	3.12069625	0.00180424	0.00734071
ENSDARG00000089769	hapln1a	12732.3705	0.332444355	0.10654066	3.12035187	0.00180635	0.00734715
ENSDARG00000044976	krt93	4.96145014	4.051331421	1.29836146	3.12034171	0.00180641	0.00734715
ENSDARG00000017359	mettl8	67.934929	-0.937844826	0.30058544	-3.12006077	0.00180814	0.00735295
ENSDARG00000074262	nck1a	845.296344	0.38754403	0.12422494	3.11969599	0.00181038	0.00735966
ENSDARG00000062423	zgc:153901	169.690724	0.752885964	0.24133272	3.11970117	0.00181035	0.00735966
ENSDARG00000112019	CABZ01065423.1	419.16556	0.955540461	0.30630115	3.11961105	0.0018109	0.00736058
ENSDARG00000023820	faxdc2	2337.9407	0.687120571	0.22028649	3.11921343	0.00181335	0.00736931
ENSDARG00000039131	atp1a1a.3	2046.95014	0.503603936	0.16146055	3.11905257	0.00181434	0.00737213
ENSDARG00000113320	si:dkey-88n24.5	36.6802258	1.718145307	0.55094525	3.11854091	0.00181749	0.00738374
ENSDARG00000056557	copb1	6686.66776	0.280723662	0.09003601	3.11790418	0.00182142	0.0073985
ENSDARG00000036864	slc34a2b	1160.22248	0.481848036	0.15455675	3.1176124	0.00182322	0.00740461
ENSDARG00000002016	ppil2	778.644746	-0.383034912	0.1228704	-3.1173897	0.0018246	0.00740834
ENSDARG00000032277	rars2	259.353589	0.541765671	0.17378947	3.11736769	0.00182474	0.00740834
ENSDARG00000088711	lgals1l1	5969.05678	1.023814148	0.32846402	3.116975	0.00182717	0.00741598
ENSDARG00000056186	eif5a2	10831.5502	-0.342613588	0.10991889	-3.11696746	0.00182722	0.00741598
ENSDARG00000036086	itga11a	625.725765	0.43828442	0.14061543	3.11690133	0.00182763	0.00741644
ENSDARG00000069361	spa17	366.419618	-0.58559028	0.18789601	-3.11656586	0.00182971	0.00742367
ENSDARG00000041323	csdc2a	869.827234	-0.455108541	0.14603187	-3.11650143	0.00183011	0.00742408
ENSDARG00000079378	phldb1b	1942.63246	0.424222753	0.13613728	3.11613956	0.00183236	0.00743077
ENSDARG00000060498	tnfrsf9a	101.855235	0.86533581	0.27769313	3.11615856	0.00183224	0.00743077
ENSDARG00000110496	zgc:162989	272.603636	-0.671850203	0.21560916	-3.11605594	0.00183288	0.00743167
ENSDARG00000077237	rfx7a	1295.93061	-0.396341901	0.12720705	-3.11572285	0.00183495	0.00743885
ENSDARG00000054658	slc34a1b	22.367916	-2.052771564	0.6588692	-3.11559802	0.00183572	0.00744079
ENSDARG00000090753	si:dkey-13p1.3	39.7790391	1.316764606	0.42266325	3.11539887	0.00183696	0.00744263
ENSDARG00000001889	tuba1a	16875.2867	0.499490997	0.16032828	3.11542669	0.00183679	0.00744263
ENSDARG00000009299	dusp8a	2540.13636	-0.328803727	0.10554207	-3.11538078	0.00183708	0.00744263
ENSDARG00000088293	doc2b	506.067676	-0.592271393	0.19014319	-3.11487042	0.00184026	0.0074543
ENSDARG00000113977	fthl29	419.88221	-0.805990141	0.25882573	-3.11402636	0.00184553	0.00747444
ENSDARG00000011693	ttc36	399.19269	-0.632660737	0.20320224	-3.11345358	0.00184912	0.00748775
ENSDARG00000100376	amdhd2	1031.8009	0.636522074	0.20445128	3.11331906	0.00184996	0.00748994
ENSDARG00000040822	fundc1	1655.05902	-0.359298865	0.11541912	-3.1129925	0.00185201	0.0074963
ENSDARG00000074066	tas2r200.2	41.4285969	-1.340418508	0.43059119	-3.11297247	0.00185213	0.0074963
ENSDARG00000100655	CR356223.2	9.11455504	2.898617594	0.93121165	3.11273768	0.00185361	0.00750104
ENSDARG00000016687	acadvl	8294.32336	0.251612729	0.08086718	3.1114319	0.00186182	0.00753307
ENSDARG00000074768	zgc:113295	259.518653	-0.554663818	0.17830635	-3.11073513	0.00186622	0.00754964
ENSDARG00000059373	dnajc8	2579.32396	0.486851556	0.15654303	3.1100174	0.00187076	0.00756677
ENSDARG00000054867	hibch	1006.11868	0.490418543	0.15769425	3.10993292	0.0018713	0.00756771
ENSDARG00000117704	NA	8.37440739	-3.651525479	1.1743799	-3.10932219	0.00187517	0.0075809

Table S2. DEGs of WT vs. *terfa-/-*

ENSDARG00000042319	cdca4	2767.19208	-0.405763407	0.13049896	-3.10932285	0.00187517	0.0075809
ENSDARG00000004243	asic4b	76.6146327	-1.118158107	0.35969801	-3.10860241	0.00187975	0.00759816
ENSDARG00000077162	nwd2	1737.88686	-0.315161271	0.1013858	-3.10853477	0.00188018	0.00759866
ENSDARG00000087061	si:ch211-71k14.1	6.63284978	-5.0824693	1.63514315	-3.10827177	0.00188185	0.00760419
ENSDARG00000054026	mustn1a	36.5230958	1.560270765	0.50203565	3.10788839	0.00188429	0.00761282
ENSDARG00000063372	zpld1b	31.8967115	-1.446026127	0.46528461	-3.10783139	0.00188466	0.00761305
ENSDARG00000060554	mut	3014.14075	0.429118881	0.13809773	3.10735661	0.00188769	0.00762405
ENSDARG00000003750	pfas	2202.48816	0.500424741	0.16108099	3.10666547	0.0018921	0.00764051
ENSDARG00000101961	abhd18	586.503248	0.375033519	0.12072064	3.10662309	0.00189238	0.00764051
ENSDARG00000020345	clasp2	8070.86413	-0.341119396	0.10984087	-3.10557816	0.00189907	0.00766631
ENSDARG00000100652	znf1147	16.6440032	-2.426376418	0.78136902	-3.10528875	0.00190093	0.00767257
ENSDARG00000002790	ap2m1a	12116.607	0.279621718	0.0900639	3.1047036	0.0019047	0.00768527
ENSDARG00000095810	BX005108.2	327.12181	-0.645963764	0.20805687	-3.10474618	0.00190442	0.00768527
ENSDARG00000002917	gls2b	87.86075	-0.96560943	0.31104929	-3.10436149	0.0019069	0.00769292
ENSDARG00000071046	bbip1	308.363545	-0.552397436	0.17794699	-3.10428081	0.00190742	0.00769376
ENSDARG00000101020	KHDRBS3	527.102726	-0.454965976	0.14656876	-3.10411298	0.0019085	0.00769688
ENSDARG00000074466	gdpd3a	486.669282	0.412838206	0.13300941	3.10382716	0.00191035	0.00770307
ENSDARG00000103476	trpm1b	1043.25437	-0.608904648	0.19619077	-3.10363554	0.00191159	0.00770681
ENSDARG00000068755	exosc8	368.038896	-0.555519028	0.17905173	-3.1025616	0.00191854	0.00773357
ENSDARG00000021233	pgls	262.680142	-0.567673597	0.18300619	-3.10193664	0.00192259	0.00774741
ENSDARG00000098919	smarcc1b	975.931604	-0.466707089	0.15045583	-3.10195413	0.00192248	0.00774741
ENSDARG00000061830	prss12	1014.93467	0.394793772	0.12728001	3.10177356	0.00192365	0.00775042
ENSDARG00000105112	ntn1a	994.981875	0.381480735	0.12299853	3.10150659	0.00192539	0.00775506
ENSDARG00000076566	kank3	1474.85841	0.314236044	0.10131743	3.10150044	0.00192543	0.00775506
ENSDARG00000111014	LO018430.2	244.721279	0.537731981	0.17338128	3.10144191	0.00192581	0.00775533
ENSDARG00000102375	si:ch211-204c21.1	43.6944356	1.124918439	0.36273199	3.10123861	0.00192713	0.0077594
ENSDARG00000013729	srsf6a	5420.70146	0.404932969	0.13059069	3.10077973	0.00193012	0.00777018
ENSDARG00000069142	aars1	8987.06558	0.408802142	0.13184561	3.10061251	0.00193121	0.00777331
ENSDARG00000036105	si:dkeyp-92c9.2	101.432661	-0.797372295	0.2572086	-3.10009965	0.00193456	0.00778552
ENSDARG00000015538	znf207a	2420.43244	0.35183838	0.11350023	3.09989125	0.00193592	0.00778974
ENSDARG00000092532	si:dkey-21e2.8	22.3432289	-2.388395388	0.77079378	-3.09861789	0.00194426	0.00782203
ENSDARG00000087196	fibinb	778.30757	0.472046052	0.15237311	3.09796172	0.00194857	0.0078381
ENSDARG00000040245	kpnb3	8475.15307	0.382189007	0.1234177	3.09671158	0.0019568	0.00786915
ENSDARG00000099933	ppa1a	221.334214	-0.906797079	0.29282749	-3.09669384	0.00195692	0.00786915
ENSDARG00000003208	pomk	469.41575	0.419239137	0.13538997	3.09653028	0.001958	0.00787222
ENSDARG00000054890	rgra	380.867768	-0.492115768	0.15893862	-3.09626298	0.00195977	0.00787805
ENSDARG00000091509	atp6ap1la	552.513065	-0.644817189	0.2082836	-3.0958616	0.00196242	0.00788744
ENSDARG00000094263	CU539058.1	275.472979	-0.627805515	0.2028009	-3.09567418	0.00196366	0.00789115
ENSDARG00000104765	chek1	322.181694	-0.616550814	0.19917865	-3.09546645	0.00196504	0.0078954
ENSDARG00000095152	si:ch211-255i3.4	270.606945	-0.537642189	0.17369776	-3.0952741	0.00196631	0.00789797
ENSDARG00000100847	esrrb	477.902462	-0.462152405	0.14930688	-3.09531892	0.00196601	0.00789797
ENSDARG00000037199	C9H2orf76	145.166907	-0.717404508	0.23178748	-3.09509606	0.00196749	0.00790144
ENSDARG00000091535	ccser2b	137.885713	0.90761327	0.29326524	3.09485461	0.00196909	0.0079066
ENSDARG00000088881	si:dkeyp-69b9.3	933.411517	0.522448351	0.16882977	3.09452743	0.00197127	0.00791404
ENSDARG00000016573	mroh1	2091.85346	0.29635959	0.09577371	3.09437319	0.00197229	0.00791688
ENSDARG00000016721	sdha	12613.571	0.346699841	0.11204895	3.09418186	0.00197357	0.00791998
ENSDARG00000036868	tbpl1	284.66899	-0.589645225	0.19056706	-3.09416141	0.0019737	0.00791998
ENSDARG00000012140	ccnl1b	2096.45061	-0.299268707	0.09672238	-3.09410002	0.00197411	0.00792034
ENSDARG00000109381	CU457778.2	12.5076289	2.933267888	0.9484412	3.09272508	0.00198328	0.00795584
ENSDARG00000062662	lmod3	419.865379	0.553713364	0.17905169	3.09247771	0.00198493	0.00796119
ENSDARG00000103026	p3h2	630.843991	-0.394891727	0.12771596	-3.09195285	0.00198844	0.00797399
ENSDARG00000014179	pfkma	5212.35104	0.528405773	0.17091554	3.09161925	0.00199068	0.00798167
ENSDARG00000039363	dnajb12a	862.40266	-0.351517452	0.11370585	-3.09146321	0.00199173	0.00798458
ENSDARG00000091266	si:ch211-71m22.5	18.7711041	-1.758801667	0.56896757	-3.09121604	0.00199339	0.00798938
ENSDARG00000062900	si:ch211-81a5.1	207.964937	0.658093772	0.21289342	3.09118886	0.00199357	0.00798938
ENSDARG00000094124	CR589874.1	38.8979836	-1.558402399	0.50419729	-3.09085836	0.00199579	0.00799699
ENSDARG00000068833	dclre1b	743.270682	-0.554889272	0.17954582	-3.09051631	0.00199809	0.00800491
ENSDARG00000017665	snrkb	805.750373	0.488166391	0.15798456	3.08996269	0.00200182	0.00801856

Table S2. DEGs of WT vs. *terfa-/-*

ENSDARG00000032344	pde4a	1056.5479	-0.412696655	0.13359097	-3.08925572	0.00200659	0.00803378
ENSDARG00000019917	epb41l3b	5234.74997	0.265096209	0.0858109	3.08930701	0.00200624	0.00803378
ENSDARG00000103643	unc45a	948.313362	0.315151396	0.10201455	3.0892788	0.00200643	0.00803378
ENSDARG00000114730	CABZ01113192.1	1225.26319	-0.362102213	0.11721788	-3.08913809	0.00200738	0.00803567
ENSDARG00000092341	AL590150.2	23.3189275	-1.735375806	0.56184719	-3.08869711	0.00201036	0.00804631
ENSDARG00000099455	ogt.1	6143.57252	-0.316829813	0.10260179	-3.08795591	0.00201538	0.0080651
ENSDARG00000019364	mbip	351.423703	-0.508672937	0.16477777	-3.08702403	0.00202171	0.00808783
ENSDARG00000053152	pcmtd1	1020.16022	-0.432553731	0.14011894	-3.08704679	0.00202156	0.00808783
ENSDARG00000115956	CABZ01044281.1	7843.7361	-0.455269841	0.1475026	-3.0865209	0.00202514	0.00810022
ENSDARG00000071013	arl6ip6	143.211019	0.600183992	0.19446094	3.08639871	0.00202597	0.00810225
ENSDARG00000088507	znf982	161.564664	-0.777277501	0.25185536	-3.08620588	0.00202728	0.0081062
ENSDARG00000062978	ccdc151	93.4917395	0.797288943	0.25834857	3.08609771	0.00202802	0.00810654
ENSDARG00000042055	niban1a	625.256435	0.623662542	0.20208759	3.08610023	0.002028	0.00810654
ENSDARG00000089489	fam83e	509.905682	0.450819026	0.1461164	3.08534173	0.00203318	0.00812587
ENSDARG00000041239	wdr20b	321.59739	-0.561120129	0.18188422	-3.08504028	0.00203525	0.0081328
ENSDARG00000100372	alad	865.621549	0.332752872	0.10788081	3.08444911	0.0020393	0.00814636
ENSDARG00000110103	FO834799.1	324.929828	0.641299923	0.20791389	3.0844497	0.00203929	0.00814636
ENSDARG00000101948	si:ch211-223g7.6	55.5885001	-0.958632911	0.31091127	-3.08330067	0.00204718	0.00817656
ENSDARG00000093677	si:ch211-56a11.2	153.91664	-0.918133895	0.29779337	-3.08312405	0.0020484	0.0081801
ENSDARG00000063258	rfx5	684.141118	-0.396671376	0.12867676	-3.08269625	0.00205134	0.00819055
ENSDARG00000014967	g6pcb	12.9317299	2.342419613	0.76001374	3.08207536	0.00205563	0.00820502
ENSDARG00000061235	alg5	14.9866811	2.079515511	0.67470503	3.08211056	0.00205539	0.00820502
ENSDARG00000045863	asb15b	29.80429	-1.390849485	0.45135987	-3.08146465	0.00205985	0.00822055
ENSDARG00000024299	c1qtnf4	1341.72256	-0.369634374	0.11997757	-3.08086226	0.00206402	0.00823587
ENSDARG00000038847	gins3	171.061102	-0.689628175	0.22389323	-3.08016542	0.00206886	0.00825384
ENSDARG00000056279	ccdc157	59.7018302	0.928083326	0.30135631	3.07968772	0.00207218	0.00826576
ENSDARG00000100829	pcdh1g32	173.121344	-0.967451266	0.31417793	-3.07931011	0.00207481	0.00827492
ENSDARG00000030598	nampta	1206.09366	-0.407682708	0.13241053	-3.07892965	0.00207746	0.00828417
ENSDARG00000013117	hiat1a	1382.16061	-0.292728034	0.09508226	-3.07868201	0.00207919	0.00828436
ENSDARG00000104961	ghrhra	61.5808685	-1.107400008	0.35969919	-3.07868365	0.00207917	0.00828436
ENSDARG00000069828	si:ch211-117n7.7	291.745661	0.495136068	0.16082645	3.07869794	0.00207907	0.00828436
ENSDARG00000079472	F2RL2	185.335675	0.824291938	0.26772575	3.0788669	0.0020779	0.00828436
ENSDARG00000078966	rbm15b	1584.32285	-0.50062945	0.16260896	-3.07873235	0.00207883	0.00828436
ENSDARG00000018593	parp16	706.642973	0.347891729	0.1130019	3.07863606	0.00207951	0.00828436
ENSDARG00000092285	cenpv	94.4451993	0.901306441	0.29277146	3.07853251	0.00208023	0.00828458
ENSDARG00000077810	otud4	383.524898	0.549304445	0.17842856	3.07856797	0.00207998	0.00828458
ENSDARG00000044847	zgc:171599	268.521045	-0.571749069	0.18573453	-3.07831333	0.00208176	0.00828935
ENSDARG00000019753	kcnn3	678.703873	0.45828749	0.14888098	3.07821377	0.00208246	0.00829079
ENSDARG00000104049	crebl2	927.417584	-0.363916168	0.11823219	-3.0779788	0.0020841	0.00829467
ENSDARG00000108525	CR749167.2	48.1562969	4.545492495	1.47677255	3.07799091	0.00208401	0.00829467
ENSDARG00000099441	cbx4	608.121227	-0.422905011	0.13739923	-3.07792857	0.00208445	0.00829474
ENSDARG00000111833	CABZ01046996.1	8.10771295	2.50598511	0.81435428	3.07726646	0.00208909	0.00831186
ENSDARG00000103516	sec24c	3389.4365	0.300938002	0.0977957	3.07721089	0.00208947	0.00831208
ENSDARG00000075597	flrt1b	803.142897	-0.407387458	0.132403	-3.07687493	0.00209183	0.00832012
ENSDARG00000004648	kctd16a	117.578293	-0.785754247	0.25538851	-3.0767017	0.00209305	0.0083227
ENSDARG00000061974	grhl2b	588.253673	0.411694024	0.13381081	3.07668733	0.00209315	0.0083227
ENSDARG00000060350	apoda.2	1923.29638	-1.204740521	0.39162314	-3.07627516	0.00209604	0.00833288
ENSDARG00000088036	si:dkey-106g10.7	451.681738	-0.519622181	0.16891763	-3.07618686	0.00209666	0.00833401
ENSDARG00000030012	lrrfip1a	1540.19284	0.428616792	0.13933658	3.07612539	0.0020971	0.0083344
ENSDARG00000014676	bckdhb	2960.20275	0.338708869	0.11011693	3.07590188	0.00209867	0.00833932
ENSDARG00000089262	pbx3a	307.635616	-0.493370105	0.160407	-3.07573926	0.00209981	0.00834253
ENSDARG00000102976	uncx	486.420362	-0.524822088	0.17066164	-3.07521995	0.00210347	0.00835574
ENSDARG00000037860	cox6b2	6219.28761	0.394004828	0.12813027	3.0750331	0.00210479	0.00835964
ENSDARG00000019223	arglu1a	6304.41982	-0.319328765	0.10385121	-3.07486805	0.00210596	0.00836198
ENSDARG00000104139	atp1a3b	8036.21504	0.435056996	0.14148865	3.07485436	0.00210605	0.00836198
ENSDARG00000098348	znf326	63.1187014	1.134550162	0.36902831	3.07442579	0.00210908	0.00837266
ENSDARG00000075264	si:dkeyp-74a11.1	243.217067	-0.655426701	0.2132851	-3.07300747	0.00211913	0.00840958
ENSDARG00000104082	trps1	883.669761	0.459837123	0.14963678	3.07302211	0.00211903	0.00840958

Table S2. DEGs of WT vs. *terfa-/-*

ENSDARG00000069464	cox7a1	226.058631	-0.802750558	0.26122953	-3.07297024	0.0021194	0.00840958
ENSDARG00000061379	cmya5	1209.55302	0.464262117	0.1511032	3.0724836	0.00212286	0.00842196
ENSDARG00000057276	iqca1	82.5279706	1.052986789	0.34272763	3.07237207	0.00212365	0.00842376
ENSDARG00000074509	tacr2	37.3137276	-1.214274192	0.39525699	-3.07211311	0.00212549	0.00842973
ENSDARG00000006546	ak4	666.632112	-0.565682774	0.1841523	-3.07182026	0.00212758	0.00843665
ENSDARG00000074528	unm_sa821	218.76921	-0.641850492	0.20899736	-3.07109371	0.00213276	0.00845451
ENSDARG00000102733	EMC4	128.586322	0.978512997	0.31862002	3.07109704	0.00213274	0.00845451
ENSDARG00000099667	snrpg	1380.68084	-0.375910873	0.12240805	-3.07096542	0.00213368	0.0084568
ENSDARG00000067817	CABZ01083448.1	809.195289	0.341999818	0.11140743	3.06981155	0.00214194	0.00848818
ENSDARG00000017781	hsd17b10	1761.32798	0.384711844	0.12533761	3.06940463	0.00214486	0.0084984
ENSDARG00000063553	rlf	831.213414	-0.474391003	0.15458948	-3.06871466	0.00214982	0.00851669
ENSDARG00000014591	ilf2	6343.82734	-0.49957073	0.16286589	-3.06737491	0.00215948	0.0085536
ENSDARG00000099722	zgc:153993	718.333568	0.432320928	0.1409556	3.0670717	0.00216167	0.00856092
ENSDARG00000020735	efcab6	76.3670108	0.956463191	0.31193001	3.06627503	0.00216744	0.00858239
ENSDARG00000005606	paxip1	1374.1367	-0.415135297	0.13539475	-3.06611073	0.00216863	0.00858574
ENSDARG00000098349	hdac4	1074.76029	0.503486586	0.16421622	3.06599784	0.00216945	0.00858762
ENSDARG00000054807	sec13	3225.47205	0.4022081	0.13120515	3.06549015	0.00217314	0.00860084
ENSDARG00000035859	angptl4	3781.08941	-0.319056529	0.10409691	-3.06499515	0.00217674	0.00861235
ENSDARG00000063577	klhl35	90.4428482	1.035102894	0.33771625	3.06500767	0.00217664	0.00861235
ENSDARG00000009553	gng3	3579.62582	-0.401883249	0.13113878	-3.06456456	0.00217987	0.00862201
ENSDARG00000103824	hint1	1228.80145	0.439042651	0.1432625	3.06460275	0.00217959	0.00862201
ENSDARG00000091003	il34	119.968767	0.811313686	0.26476508	3.06427749	0.00218196	0.00862891
ENSDARG00000104853	il1rapl1b	338.510585	-0.740407118	0.24166209	-3.06381165	0.00218537	0.00864099
ENSDARG00000117405	BX571827.3	219.122899	-0.596865774	0.19484631	-3.06326443	0.00218937	0.00865543
ENSDARG00000005318	stam2	1589.03181	0.338104858	0.11037953	3.06311191	0.00219048	0.00865846
ENSDARG00000075962	vav3b	69.6273676	-0.919430837	0.3002053	-3.06267353	0.00219369	0.0086687
ENSDARG00000116283	CABZ01023247.1	4.48855682	4.264576977	1.39244079	3.06266307	0.00219377	0.0086687
ENSDARG00000006621	pspc1	1515.04184	-0.381986345	0.12475001	-3.06201456	0.00219853	0.00868612
ENSDARG00000103597	si:dkey-40j3.3	25.6621658	-1.793079338	0.58561616	-3.06186794	0.00219961	0.008689
ENSDARG00000068708	ifrd1	3891.1831	-0.296503569	0.09683964	-3.06179973	0.00220011	0.0086896
ENSDARG00000031774	pus7	998.96234	-0.353116776	0.11535878	-3.06103067	0.00220577	0.00871056
ENSDARG00000040133	ackr4b	219.316681	0.671718886	0.21945913	3.06079256	0.00220752	0.00871611
ENSDARG00000014106	cfl2	3531.07511	0.441217286	0.14417497	3.0602905	0.00221122	0.00872935
ENSDARG00000096365	si:ch211-195m9.3	165.937973	0.652125521	0.21310977	3.06004522	0.00221304	0.00873511
ENSDARG00000013252	tmc5	176.980536	0.817017778	0.26701881	3.05977609	0.00221503	0.00874019
ENSDARG00000060829	car15	32.020152	-1.736178681	0.56741498	-3.05980409	0.00221482	0.00874019
ENSDARG00000095347	si:dkeyp-72h1.1	247.760504	-0.626786075	0.20485645	-3.05963553	0.00221607	0.0087429
ENSDARG00000040284	si:dkey-79d12.5	183.976794	0.772428751	0.2524779	3.05939155	0.00221787	0.00874864
ENSDARG00000098288	tax1bp1a	804.983064	0.380288771	0.12430834	3.0592378	0.00221901	0.00875174
ENSDARG00000098118	trappc10	2895.33373	0.302951958	0.09903125	3.05915515	0.00221962	0.00875277
ENSDARG00000003867	cdk20	164.358883	-0.659579924	0.21563424	-3.05879035	0.00222233	0.00876204
ENSDARG00000021143	rtn1b	4031.5439	-0.466958967	0.15267063	-3.05860382	0.00222371	0.00876611
ENSDARG00000036104	myo1g	36.6353911	1.287444574	0.42100472	3.05802886	0.00222798	0.00878155
ENSDARG00000006584	phf8	2368.64823	-0.374193586	0.122372	-3.05783655	0.00222941	0.00878499
ENSDARG00000044251	rasgef1bb	822.817368	0.559623646	0.18301413	3.05781669	0.00222956	0.00878499
ENSDARG00000038290	thoc5	1716.65202	-0.379455618	0.12411922	-3.05718665	0.00223425	0.00880208
ENSDARG00000030311	tmc2b	51.0620271	-1.027500318	0.3361195	-3.05694946	0.00223602	0.00880765
ENSDARG00000103683	inpp5b	1434.77121	0.313978308	0.1027168	3.05673765	0.0022376	0.00881248
ENSDARG00000071733	si:ch211-207i20.3	120.811289	-0.696489779	0.22791705	-3.05589149	0.00224392	0.00883599
ENSDARG00000103714	rab6a	2024.93454	-0.282028009	0.09229453	-3.05573914	0.00224507	0.00883768
ENSDARG00000063522	bbs4	241.852582	-0.542517232	0.17753964	-3.05575264	0.00224496	0.00883768
ENSDARG00000079355	flrt2	734.781699	0.398360037	0.13037186	3.05556758	0.00224635	0.00884133
ENSDARG00000034497	fbxl14a	816.810731	-0.398429011	0.13041969	-3.05497594	0.00225079	0.00885739
ENSDARG00000039754	xpc	713.382744	0.432166509	0.1414866	3.05446961	0.00225459	0.00887095
ENSDARG00000057649	satb1a	591.913909	-0.529618058	0.17340626	-3.05420372	0.00225659	0.00887741
ENSDARG00000073866	tdp1	326.010029	-0.522610687	0.17112978	-3.05388511	0.00225899	0.00888544
ENSDARG00000101244	gli1	1060.29519	0.384756948	0.12599202	3.05382007	0.00225948	0.00888596
ENSDARG00000038814	myrip	1497.13228	-0.456956984	0.14964855	-3.05353432	0.00226163	0.00889302

Table S2. DEGs of WT vs. *terfa-/-*

ENSDARG00000078285	pik3ap1	75.2697062	0.938153184	0.30726184	3.05326945	0.00226363	0.00889947
ENSDARG00000101703	zgc:158260	12.6539314	2.158150951	0.70695786	3.05272925	0.0022677	0.00891409
ENSDARG00000102606	znf1158	109.973133	-0.778582196	0.25508905	-3.05219767	0.00227172	0.00892848
ENSDARG00000040430	nptxra	865.149636	-0.584863962	0.19164714	-3.05177505	0.00227493	0.0089383
ENSDARG00000038559	h1-0	19221.2489	-0.452613708	0.14831174	-3.05177274	0.00227494	0.0089383
ENSDARG00000110878	si:dkey-28k24.2	9.81656401	2.61380882	0.85652254	3.05165213	0.00227586	0.00893907
ENSDARG00000037560	mtm1	741.949839	-0.364866981	0.11956323	-3.05166555	0.00227576	0.00893907
ENSDARG00000039852	ftr93	77.8154739	1.030642971	0.3378039	3.05100973	0.00228073	0.0089568
ENSDARG00000079872	rapgef4	268.537066	-0.60187721	0.19728189	-3.05084879	0.00228196	0.00896019
ENSDARG00000059855	wdr66	55.6001059	1.229871477	0.4031645	3.05054511	0.00228426	0.00896784
ENSDARG00000030687	phka2	2537.73154	0.356827359	0.11698518	3.05019289	0.00228694	0.00897694
ENSDARG00000016404	ptch1	3720.17549	0.354107729	0.11610315	3.04994083	0.00228887	0.00898306
ENSDARG00000052408	mgat2	609.728245	-0.381508814	0.12514544	-3.04852342	0.00229969	0.00902412
ENSDARG00000070094	sytl1	127.945351	-0.657280767	0.21560988	-3.04847235	0.00230008	0.00902423
ENSDARG00000074695	mertka	509.643681	0.60334879	0.19792835	3.04831926	0.00230125	0.00902741
ENSDARG00000100823	selenok	1429.60984	0.363983874	0.1194195	3.04794331	0.00230413	0.00903728
ENSDARG00000093836	si:dkey-83f18.14	10.7331502	2.501268436	0.82075277	3.0475297	0.00230731	0.0090483
ENSDARG00000061174	znf740b	538.184877	-0.486713196	0.15972348	-3.04722379	0.00230966	0.00905609
ENSDARG00000054271	cdkn1bb	1701.61056	-0.348742275	0.11445628	-3.04694759	0.00231178	0.00906298
ENSDARG00000044457	gfi1ab	142.524059	-0.627402515	0.20592094	-3.04681268	0.00231282	0.00906562
ENSDARG00000077177	map2k4b	2042.80933	0.348074542	0.11425055	3.04658972	0.00231453	0.00907092
ENSDARG00000060688	trim69	184.498213	0.546523791	0.17942472	3.04597818	0.00231925	0.00908795
ENSDARG00000098788	pcdh2g28	8091.56584	-0.314027585	0.10312145	-3.04522068	0.0023251	0.00910944
ENSDARG00000101374	si:dkey-11n14.1	119.279017	-1.005865559	0.33035152	-3.04483409	0.00232809	0.00911969
ENSDARG00000069755	mtmr11	1070.57416	-0.382583539	0.12565196	-3.0447876	0.00232845	0.00911969
ENSDARG00000077202	mocs2	247.852874	0.524600002	0.17229857	3.04471481	0.00232901	0.00912046
ENSDARG00000095585	CT583727.1	7.06854939	3.247037313	1.0664723	3.04465229	0.0023295	0.00912092
ENSDARG00000061371	cdh18a	614.602129	-0.517727578	0.17010123	-3.04364395	0.00233732	0.0091501
ENSDARG00000051814	ptprz1a	2042.03656	-0.478576675	0.15726957	-3.04303426	0.00234206	0.00916722
ENSDARG00000095188	si:dkey-189h5.6	95.5319346	0.957880478	0.31480712	3.04275352	0.00234424	0.00917288
ENSDARG00000101790	si:ch73-299h12.3	156.845838	-0.663283629	0.21798478	-3.04279784	0.0023439	0.00917288
ENSDARG00000041340	mrpl51	1166.01513	-0.358288561	0.11775805	-3.04258229	0.00234558	0.00917666
ENSDARG00000001818	c3b.2	2029.43369	-0.474410105	0.15592802	-3.04249425	0.00234626	0.0091779
ENSDARG00000006110	chrd	181.548753	0.780645703	0.25658801	3.04240912	0.00234693	0.00917905
ENSDARG00000009534	wls	3578.0608	0.26802844	0.08810156	3.04226678	0.00234804	0.00918091
ENSDARG00000060494	eprs1	7802.80242	0.358652768	0.11789049	3.04225353	0.00234814	0.00918091
ENSDARG00000009822	her4.4	139.744179	-1.871089561	0.61517458	-3.04155862	0.00235357	0.00920068
ENSDARG00000105653	BX323797.4	31.5637219	1.168661046	0.38424766	3.04142661	0.0023546	0.00920327
ENSDARG00000045383	gpr22b	411.230578	-0.651308044	0.21415689	-3.04126589	0.00235586	0.00920673
ENSDARG00000114783	znf985	23.3857543	-1.844860699	0.6066192	-3.04121711	0.00235624	0.00920678
ENSDARG00000040439	rsl24d1	5739.83976	-0.350735774	0.11532959	-3.04116035	0.00235668	0.00920707
ENSDARG00000077360	fthl30	161.595262	1.065457015	0.35044309	3.04031391	0.00236332	0.00923153
ENSDARG00000079616	cramp1	1349.59732	-0.327590651	0.10779137	-3.03911769	0.00237272	0.00926681
ENSDARG00000104801	tubb6	664.817862	0.487862929	0.16053771	3.03893051	0.0023742	0.00927112
ENSDARG00000032206	cthl	109.326505	-0.871998899	0.28699485	-3.03837821	0.00237855	0.00928667
ENSDARG00000086896	fubp3	3707.59788	0.284788088	0.09374591	3.03787212	0.00238255	0.00930067
ENSDARG00000104172	diabloa	1346.85656	0.465876902	0.15335848	3.03782951	0.00238289	0.00930067
ENSDARG00000074320	KCNAB3	243.110758	-0.806316202	0.26549105	-3.03707491	0.00238886	0.00932252
ENSDARG00000092608	si:dkey-262g12.12	194.769439	-0.643196216	0.21178866	-3.03697195	0.00238968	0.00932424
ENSDARG00000052633	si:ch211-106n13.3	403.56009	0.469618823	0.15464376	3.0367784	0.00239121	0.00932877
ENSDARG00000000551	slc1a4	2559.95692	0.656868018	0.21634952	3.03614273	0.00239626	0.00934406
ENSDARG00000002298	ankrd22	109.489299	0.906942139	0.29870969	3.03619922	0.00239581	0.00934406
ENSDARG00000113932	LO018011.1	11.5932286	2.402897889	0.79142268	3.03617516	0.002396	0.00934406
ENSDARG00000059719	fam169aa	215.114467	0.981735754	0.32339101	3.03575466	0.00239935	0.00935462
ENSDARG00000020405	napab	1430.63435	0.304196859	0.10021832	3.03534182	0.00240263	0.00936597
ENSDARG00000102082	nr3c2	314.360854	-0.599720986	0.19760697	-3.03491824	0.00240601	0.00937766
ENSDARG00000111129	acy1	212.131822	0.626231022	0.2063776	3.0343944	0.00241019	0.00939249
ENSDARG00000088672	zmp:0000000936	157.375206	0.601520338	0.19825462	3.03407982	0.00241271	0.00940082

Table S2. DEGs of WT vs. *terfa-/-*

ENSDARG00000105784	LO018029.1	514.22686	-0.431853636	0.14233915	-3.03397656	0.00241353	0.00940089
ENSDARG00000104005	slc4a3	327.665794	-0.584053157	0.19250756	-3.03392323	0.00241396	0.00940089
ENSDARG00000097063	sparcl2	86.169163	0.903917102	0.29793105	3.03398089	0.0024135	0.00940089
ENSDARG00000018397	hpca	3198.759	-0.296304085	0.09766479	-3.03388849	0.00241424	0.00940089
ENSDARG00000092584	si:ch1073-385f13.:	69.2301517	0.932018808	0.30725189	3.03340297	0.00241813	0.00941455
ENSDARG00000044895	fmoda	4672.82409	-0.29612611	0.09764137	-3.03279335	0.00242302	0.00943211
ENSDARG00000092000	znf1020	57.9929396	1.065831429	0.35146841	3.03250986	0.00242529	0.0094395
ENSDARG00000110439	ap1s3a	214.818795	0.828711936	0.27330128	3.03222856	0.00242755	0.00944682
ENSDARG00000087762	rab27b	302.272517	-0.663811133	0.21897085	-3.03150452	0.00243338	0.00946802
ENSDARG00000054786	faah2b	43.5649775	1.53769577	0.50726185	3.03136492	0.00243451	0.00947092
ENSDARG00000011602	CRACD	1482.74717	-0.41318106	0.13630734	-3.03124582	0.00243547	0.00947317
ENSDARG00000029766	nr1i2	587.707853	0.538264447	0.17763193	3.03022353	0.00244373	0.00950381
ENSDARG00000101817	si:dkey-5i16.5	59.9839242	-0.925744818	0.30551823	-3.03008047	0.00244489	0.00950683
ENSDARG00000005057	dimt1l	418.089697	0.484085658	0.15980688	3.02919163	0.00245209	0.00953335
ENSDARG00000100019	arhgap11a	478.922651	-0.721325628	0.23825657	-3.02751624	0.00246573	0.00958486
ENSDARG00000003091	oclnb	1143.61217	0.398212966	0.13153545	3.02741929	0.00246652	0.00958644
ENSDARG00000112032	HEPACAM	69.3282918	-1.20524329	0.39811611	-3.02736628	0.00246695	0.00958662
ENSDARG00000006125	csnk1db	4459.11791	0.274198992	0.09059877	3.02652002	0.00247386	0.00961199
ENSDARG00000105418	CU457819.4	17.2294465	-1.946471414	0.64327357	-3.02588433	0.00247907	0.00963072
ENSDARG00000078006	wscd1b	564.0658	-0.454389579	0.15018787	-3.02547454	0.00248243	0.00964146
ENSDARG00000040803	lactb	429.513646	0.471687322	0.15590636	3.02545265	0.00248261	0.00964146
ENSDARG00000039150	lgmn	5043.17624	0.498887	0.16493611	3.0247288	0.00248856	0.00966305
ENSDARG00000095944	si:ch73-103b2.1	13.6834318	2.677499593	0.88523677	3.02461407	0.00248951	0.00966521
ENSDARG00000012535	epx	35.4614775	-1.432448592	0.47364299	-3.02432132	0.00249192	0.00967306
ENSDARG00000073841	herc2	7349.16848	0.385985577	0.12763459	3.02414548	0.00249337	0.00967717
ENSDARG00000097452	CU929070.5	129.852782	-0.608446362	0.20120448	-3.02402002	0.0024944	0.00967885
ENSDARG00000098296	gnsb	573.750898	0.5615669	0.18570344	3.02399838	0.00249458	0.00967885
ENSDARG00000056892	mpp6a	1082.53055	-0.360892784	0.11935947	-3.02357909	0.00249804	0.00968774
ENSDARG00000069739	fndc5a	106.566179	-0.876084172	0.28974787	-3.02360872	0.00249779	0.00968774
ENSDARG00000024219	cdk5rap2	1241.7986	-0.450576781	0.14902	-3.02359935	0.00249787	0.00968774
ENSDARG00000036816	pou2f2a	1479.25577	-0.388261662	0.12842402	-3.02327927	0.00250051	0.00969583
ENSDARG00000042908	hsh2d	32.07529	1.229616699	0.40674411	3.02307196	0.00250223	0.00970096
ENSDARG00000103230	CABZ01079302.1	18.7465385	1.740304903	0.57570791	3.0228956	0.00250369	0.0097051
ENSDARG00000102272	coro6	404.455193	0.508039663	0.16808731	3.02247481	0.00250717	0.00971709
ENSDARG00000003206	chchd6a	584.582739	-0.470368406	0.15562985	-3.02235348	0.00250818	0.00971947
ENSDARG00000020469	map3k7	1896.59463	-0.293896199	0.09725566	-3.02189302	0.00251199	0.00973275
ENSDARG00000103786	klhl13	96.2562808	-0.944867057	0.31272978	-3.02135304	0.00251648	0.00974861
ENSDARG00000096065	si:dkey-156k2.7	4.0857592	5.305347403	1.75602172	3.02123108	0.00251749	0.00975101
ENSDARG00000056784	aire	9.65341732	-2.775717114	0.91883324	-3.020915	0.00252012	0.00975968
ENSDARG00000038737	phf20b	1922.83712	-0.28900428	0.09567493	-3.02068978	0.002522	0.00976542
ENSDARG00000101464	CABZ01088864.1	264.09394	-0.571889403	0.18937428	-3.01988953	0.00252867	0.00978973
ENSDARG00000005232	ccdc85ca	613.662434	-0.408727875	0.13535349	-3.01970702	0.00253019	0.00979411
ENSDARG00000045586	zgc:172145	131.047491	0.817868625	0.27089976	3.01908216	0.00253542	0.0098128
ENSDARG00000060646	senp5	352.206315	-0.538823133	0.17848065	-3.01894426	0.00253657	0.00981574
ENSDARG00000101264	trim35-12	223.386359	0.795519539	0.26353202	3.01868272	0.00253876	0.00982269
ENSDARG00000087749	ccser2a	1214.46994	-0.425928832	0.14110781	-3.01846384	0.0025406	0.00982755
ENSDARG00000003776	pip4k2aa	1627.07019	-0.310778553	0.10296005	-3.01843832	0.00254081	0.00982755
ENSDARG00000045753	tmtc2a	553.267429	-0.488681075	0.16191966	-3.01804667	0.0025441	0.00983873
ENSDARG00000045834	si:dkey-14d8.7	37.2808265	-1.86483921	0.61797324	-3.01766984	0.00254726	0.00984944
ENSDARG00000052958	nppb	35.4030995	1.338613651	0.44362158	3.0174674	0.00254897	0.00985449
ENSDARG00000015917	rnft1	865.718437	0.327038257	0.10839762	3.01702428	0.00255269	0.00986737
ENSDARG00000074321	tead3a	474.048179	0.446954933	0.14814938	3.01692074	0.00255357	0.00986921
ENSDARG00000104637	fam219b	486.080791	-0.614890305	0.20381831	-3.01685506	0.00255412	0.00986981
ENSDARG00000042232	specc1lb	571.343939	0.482397319	0.15992397	3.01641668	0.00255782	0.00988256
ENSDARG00000100723	znf1097	31.3595115	-1.395881509	0.46280422	-3.01613822	0.00256017	0.0098901
ENSDARG00000089887	mmp20a	12.9156667	2.158825073	0.71583297	3.01582234	0.00256283	0.00989887
ENSDARG00000004939	mtdhb	895.499717	0.39090685	0.1296239	3.01570039	0.00256387	0.00990131
ENSDARG00000112349	CABZ01015105.1	126.788807	-0.980294488	0.32507443	-3.01560013	0.00256471	0.00990305

Table S2. DEGs of WT vs. *terfa-/-*

ENSDARG00000070526	zc2hc1c	29.0899892	1.286036146	0.42647269	3.01551815	0.00256541	0.00990419
ENSDARG00000078411	hspb15	86.9326701	-1.12004365	0.37146787	-3.01518315	0.00256824	0.0099136
ENSDARG00000096874	tmem176l.3b	754.405823	0.466663812	0.15479194	3.01478107	0.00257165	0.00992502
ENSDARG00000098363	BX901957.1	119.665198	0.781309603	0.25916322	3.0147395	0.002572	0.00992502
ENSDARG00000053774	alpi.2	68.787705	2.46939869	0.81922828	3.01429863	0.00257574	0.00993792
ENSDARG00000045517	itih5	299.634382	0.663373309	0.22009066	3.01409117	0.0025775	0.00994163
ENSDARG00000035909	mfsd2ab	2024.17705	-0.299035952	0.09921134	-3.0141309	0.00257717	0.00994163
ENSDARG00000019004	smarcd1	4252.37736	-0.383890218	0.12741401	-3.0129356	0.00258734	0.00997801
ENSDARG00000007413	arid2	3296.43653	-0.425723612	0.14132399	-3.01239455	0.00259196	0.00999427
ENSDARG00000058690	cgnb	659.115372	0.341985958	0.11353431	3.01218167	0.00259377	0.00999973
ENSDARG00000033978	nxn	1331.83359	0.317538466	0.10544863	3.01130952	0.00260124	0.01002694
ENSDARG00000101621	CABZ01040054.1	69.8678904	-1.533538416	0.50936277	-3.01069988	0.00260646	0.01004553
ENSDARG00000077880	si:ch211-255i20.3	71.785663	-1.231316382	0.40900836	-3.01049194	0.00260825	0.01005078
ENSDARG00000077011	uhrf1bp1	601.263158	0.589407934	0.1957875	3.01044716	0.00260863	0.01005078
ENSDARG00000096828	si:ch73-144d13.7	69.4169716	-1.060782475	0.3523741	-3.01038721	0.00260915	0.01005121
ENSDARG00000058601	gdap1	395.128751	-0.455650328	0.15139314	-3.0097159	0.00261492	0.01007189
ENSDARG00000102095	si:ch73-233f7.5	179.403071	-0.764420411	0.2539953	-3.00958483	0.00261605	0.01007467
ENSDARG00000019644	ldhba	23673.1703	0.328663617	0.10924286	3.00855939	0.0026249	0.01010717
ENSDARG00000020866	apoa4b.2	4001.69247	-0.5150069	0.17121651	-3.00792776	0.00263036	0.01012663
ENSDARG00000039272	pls1	414.318142	0.65478987	0.21770409	3.00770595	0.00263228	0.01013245
ENSDARG00000088870	si:ch211-22k7.9	19.4028112	1.745083632	0.58022011	3.00762351	0.00263299	0.01013363
ENSDARG00000014439	dgkza	1035.52067	-0.406612749	0.13519976	-3.00749604	0.0026341	0.01013631
ENSDARG00000099213	glyr1	2863.0116	-0.313436155	0.10422933	-3.00717801	0.00263685	0.01014378
ENSDARG00000052942	aspg	1115.89756	0.524528924	0.17442304	3.00722265	0.00263647	0.01014378
ENSDARG00000042660	fam32a	909.533878	-0.683879804	0.2274375	-3.00689119	0.00263934	0.01015179
ENSDARG00000042708	tuba8l	18701.0651	0.337641786	0.11230343	3.00651368	0.00264262	0.01016283
ENSDARG00000070151	nfyc	4054.87963	-0.383384829	0.12753557	-3.00610117	0.00264621	0.01017505
ENSDARG00000093111	si:ch211-209l18.2	81.3569684	1.079845852	0.35927215	3.00564862	0.00265015	0.01018863
ENSDARG00000099005	palm2akap2	277.853807	0.811888127	0.27019838	3.00478532	0.00265768	0.01021601
ENSDARG00000103591	dkk3a	543.017919	0.424586795	0.14131641	3.00451165	0.00266008	0.01022363
ENSDARG00000096948	im:7143333	57.8527071	-0.980634672	0.32644854	-3.00394873	0.002665	0.01024098
ENSDARG00000056204	zgc:113436	277.640676	-0.540836243	0.18005759	-3.00368486	0.00266732	0.01024828
ENSDARG00000012019	glra1	1972.4608	-0.388893397	0.12951017	-3.00280207	0.00267506	0.01027646
ENSDARG00000061459	RAPGEF2	2677.94027	-0.341714663	0.11380782	-3.00255876	0.0026772	0.01028309
ENSDARG00000012591	pdcd10b	715.32648	-0.321535538	0.1071227	-3.00156316	0.00268597	0.01031518
ENSDARG00000029856	cnn3b	1193.24677	-0.441560066	0.14716174	-3.00050864	0.00269529	0.01034937
ENSDARG00000068363	si:ch211-132b12.3	4.64879748	5.274711156	1.75836371	2.99978391	0.00270171	0.01037242
ENSDARG00000006747	tmem178b	1514.51823	-0.45696595	0.15237294	-2.99899666	0.0027087	0.01039765
ENSDARG00000008010	zgc:194189	34.39189	-1.500834713	0.50057642	-2.99821298	0.00271568	0.01042282
ENSDARG00000010940	BX957278.1	236.450672	0.514925488	0.17178112	2.99756738	0.00272144	0.01044331
ENSDARG00000036299	nucb2a	4166.19696	0.307678434	0.10264829	2.99740428	0.00272289	0.01044729
ENSDARG00000059305	tmem37	169.797302	0.825849845	0.27552923	2.9973221	0.00272363	0.01044849
ENSDARG00000055389	si:dkey-67c22.2	2669.81261	-0.307343461	0.10254705	-2.99709702	0.00272564	0.01045459
ENSDARG00000102995	rbm24a	1248.29483	-0.324078148	0.10817367	-2.99590599	0.00273631	0.0104939
ENSDARG00000016856	pon2	564.012332	0.482673479	0.16111405	2.99584972	0.00273681	0.01049421
ENSDARG00000058212	ndnl2	319.392086	-0.460839684	0.15384645	-2.99545219	0.00274038	0.01050628
ENSDARG00000094128	CR751602.1	190.047208	-0.628751918	0.20991196	-2.99531253	0.00274164	0.01050785
ENSDARG00000075977	prr5a	593.354869	-0.55267851	0.18451221	-2.9953493	0.00274131	0.01050785
ENSDARG00000004525	C2H1orf35	374.366623	-0.50575666	0.16886581	-2.99502115	0.00274426	0.01051627
ENSDARG00000094514	si:dkey-7l6.3	465.916386	-0.538575136	0.1798284	-2.99493931	0.002745	0.01051747
ENSDARG00000101322	tfr1a	1157.83948	0.551105398	0.18401657	2.9948684	0.00274563	0.0105183
ENSDARG00000093180	CR354547.1	14.0466582	1.820892533	0.60804916	2.9946469	0.00274763	0.01052431
ENSDARG00000057058	minpp1a	796.095043	0.391823633	0.13084386	2.99458938	0.00274815	0.01052468
ENSDARG00000037739	zgc:112980	586.787472	0.353265046	0.11797027	2.99452608	0.00274872	0.01052524
ENSDARG00000069515	bbs10	65.3946025	-1.038067126	0.34670083	-2.99412931	0.00275229	0.01053731
ENSDARG00000042115	tmem198a	1063.95755	-0.458682959	0.15322239	-2.99357665	0.00275728	0.01055317
ENSDARG00000012981	osbpl7	1582.83078	-0.420191859	0.14036361	-2.99359537	0.00275711	0.01055317
ENSDARG00000116197	si:dkeyp-4c4.2	55.1563245	1.207121733	0.40326663	2.99335885	0.00275925	0.01055907

Table S2. DEGs of WT vs. *terfa-/-*

ENSDARG00000061215	fam149b1	1094.158	-0.359312883	0.12004686	-2.99310531	0.00276154	0.01056622
ENSDARG00000016699	trpa1a	329.691718	-0.585489995	0.19565716	-2.99242819	0.00276768	0.01058806
ENSDARG00000003940	mtfr1l	1754.02145	-0.32931039	0.11005393	-2.99226376	0.00276917	0.01059051
ENSDARG00000005625	maco1a	830.096926	-0.328547082	0.10979867	-2.99226829	0.00276913	0.01059051
ENSDARG00000002210	stk25a	431.956502	0.514790432	0.17206035	2.99191777	0.00277231	0.01060088
ENSDARG00000058603	jph1a	832.155214	0.435086616	0.14543279	2.99166806	0.00277458	0.01060793
ENSDARG00000097054	CU927934.1	24.2399594	2.444949157	0.81748127	2.99083202	0.00278219	0.01063538
ENSDARG00000043608	eif4ebp1	2984.84673	0.384449146	0.12858982	2.98973244	0.00279222	0.01067209
ENSDARG00000038414	b3galt6	455.622945	-0.500795816	0.16751753	-2.98951291	0.00279423	0.01067812
ENSDARG00000053836	si:ch211-284o19.8	31.9170435	1.51528515	0.50689106	2.98937044	0.00279553	0.01068146
ENSDARG00000112027	si:ch211-76m11.3	8.85785143	2.585159253	0.86484852	2.98914687	0.00279758	0.01068764
ENSDARG00000054448	kif1c	206.40598	0.743036253	0.24860189	2.98886003	0.0028002	0.01069603
ENSDARG00000042126	elmo3	952.923973	0.413741987	0.13843601	2.98868761	0.00280178	0.01070042
ENSDARG00000027749	hirip3	690.637793	-0.48098496	0.16095721	-2.98827844	0.00280554	0.01071311
ENSDARG00000010658	insig1	801.122039	0.486348039	0.16277916	2.98777833	0.00281013	0.01072901
ENSDARG00000061373	znf142	422.079819	-0.415171757	0.1389601	-2.98770488	0.00281081	0.01072994
ENSDARG00000051785	dhx33	344.210927	0.611099719	0.20457685	2.9871401	0.00281601	0.01074813
ENSDARG00000099768	si:ch211-272n13.3	1362.4661	-0.353923839	0.11849175	-2.98690699	0.00281815	0.01075468
ENSDARG00000082396	dre-mir-206-1	8.05762859	2.629839076	0.88053305	2.98664436	0.00282058	0.01076062
ENSDARG00000041133	rapsn	884.162602	0.479564022	0.16056742	2.98668326	0.00282022	0.01076062
ENSDARG00000061903	BX908736.1	5.47041442	-5.17107571	1.73143884	-2.98657717	0.0028212	0.01076133
ENSDARG00000042128	pacsin1b	1292.50394	-0.38770742	0.12983208	-2.98622211	0.00282447	0.01077096
ENSDARG00000032435	itga9	827.424583	0.477193296	0.15979945	2.9862012	0.00282467	0.01077096
ENSDARG00000097078	CR848665.1	9.82040821	-2.822960883	0.94534723	-2.98616298	0.00282502	0.01077096
ENSDARG00000018856	dclk1a	1200.52561	-0.484448949	0.16225335	-2.98575628	0.00282878	0.01078364
ENSDARG00000063159	si:dkey-32e23.4	664.253124	0.605075188	0.20267373	2.98546438	0.00283148	0.01079228
ENSDARG00000101908	rab3ip	2030.37814	-0.3428995	0.11486745	-2.98517554	0.00283416	0.01079784
ENSDARG00000039208	nasp	2440.90897	-0.531136079	0.17792331	-2.98519668	0.00283396	0.01079784
ENSDARG00000028475	lin9	476.816751	-0.448072287	0.15009961	-2.9851663	0.00283424	0.01079784
ENSDARG00000033012	pcnt	3267.92471	-0.455905167	0.15273033	-2.98503363	0.00283547	0.01080087
ENSDARG00000041870	ift172	1132.22674	0.30278977	0.10144586	2.98474247	0.00283817	0.01080949
ENSDARG00000032469	ampd3b	779.21149	0.542178571	0.18165791	2.984613	0.00283937	0.01081241
ENSDARG00000097976	CR812464.1	53.7889616	-1.284065415	0.43028331	-2.98423243	0.00284291	0.01082256
ENSDARG00000020164	efnb2a	3435.45804	-0.273165267	0.09153562	-2.98425091	0.00284274	0.01082256
ENSDARG00000068940	atp5meb	5917.71563	0.310170677	0.10393944	2.98414805	0.00284369	0.01082388
ENSDARG00000044588	emp2	1536.88457	-0.49666402	0.16644298	-2.98398903	0.00284517	0.01082785
ENSDARG00000090617	ctif	343.268605	0.577948617	0.1937097	2.98358122	0.00284896	0.01084063
ENSDARG00000042823	spcs2	1464.94324	0.289153866	0.09694035	2.98280195	0.00285623	0.01086661
ENSDARG00000100061	si:dkey-16p6.1	31.9190104	-1.352384179	0.45342565	-2.98259304	0.00285818	0.01087236
ENSDARG00000062248	ptpn21	1064.49788	0.597161719	0.2002271	2.98242212	0.00285977	0.01087677
ENSDARG00000100464	si:dkey-193i10.1	14.7003682	1.977848933	0.66319339	2.98231097	0.00286081	0.01087905
ENSDARG00000055740	9-Mar	674.130214	-0.473232122	0.15869708	-2.98198377	0.00286387	0.01088902
ENSDARG00000068919	rad51c	70.667036	-0.8855384	0.29700184	-2.98159226	0.00286754	0.01090129
ENSDARG00000073985	pctp	302.586727	0.50323023	0.16880913	2.9810605	0.00287252	0.01091857
ENSDARG00000075677	mrpl17	550.761196	-0.494828448	0.16601999	-2.98053541	0.00287745	0.01093563
ENSDARG00000101609	si:dkey-109a10.2	4.95113401	4.701619991	1.57752439	2.98037864	0.00287892	0.01093956
ENSDARG00000039352	pald1b	447.686158	-0.471187303	0.15810748	-2.98017094	0.00288088	0.01094363
ENSDARG00000040627	grik1b	510.146788	-0.79329602	0.26618744	-2.98021579	0.00288045	0.01094363
ENSDARG00000011088	si:ch211-288d18.1	1027.19144	-0.55502425	0.18632311	-2.97882668	0.00289354	0.01099007
ENSDARG00000002401	gale	908.808881	0.379913718	0.12754541	2.97865452	0.00289517	0.01099457
ENSDARG00000117506	NA	5.97597746	-5.25696362	1.76491116	-2.97859958	0.00289569	0.01099486
ENSDARG00000086550	dbf4b	155.962611	-0.770922586	0.25882697	-2.97852493	0.0028964	0.01099586
ENSDARG00000093557	BX842699.1	8.1501889	2.762567035	0.92757684	2.97826219	0.00289888	0.01100193
ENSDARG00000071083	si:dkeyp-34c12.1	18.6007301	-2.032739349	0.68251784	-2.97829482	0.00289857	0.01100193
ENSDARG00000042856	itpka	737.68344	0.430823474	0.14470045	2.97734711	0.00290755	0.01103314
ENSDARG00000060783	acap2	1024.84815	0.333762061	0.11211118	2.97706328	0.00291024	0.01104167
ENSDARG00000102445	si:dkey-22o22.2	1780.69091	0.533805972	0.17932529	2.97674677	0.00291325	0.01105139
ENSDARG00000059961	chpf2	1290.35443	0.31833524	0.10695054	2.97647151	0.00291586	0.01105963

Table S2. DEGs of WT vs. *terfa-/-*

ENSDARG00000002758	dedd1	2101.44489	0.475813324	0.15986124	2.97641465	0.0029164	0.01105999
ENSDARG00000096401	CU570787.1	3.73765268	-5.588817768	1.87790129	-2.97609774	0.00291942	0.01106974
ENSDARG00000087981	slc6a11b	2929.94497	-0.460164911	0.15463164	-2.97587808	0.00292151	0.01107598
ENSDARG00000015765	iah1	198.390585	0.665461925	0.22364641	2.97550907	0.00292503	0.01108762
ENSDARG00000021973	st13	3729.61227	-0.252549826	0.0848795	-2.97539258	0.00292614	0.01109014
ENSDARG00000070916	dnajc27	604.117894	-0.374864192	0.12599035	-2.97534045	0.00292664	0.01109034
ENSDARG00000035625	snrnp27	1296.6656	-0.371035072	0.12475419	-2.9741291	0.00293821	0.01113252
ENSDARG00000052423	ppp1r12c	1080.92957	-0.282393953	0.0950044	-2.97243033	0.00295452	0.0111909
ENSDARG00000111669	si:ch73-267c23.10	17.4055342	-2.077579604	0.69894954	-2.97243146	0.00295451	0.0111909
ENSDARG00000035127	rxrab	219.604998	0.786064914	0.26450511	2.97183259	0.00296028	0.01120929
ENSDARG00000074435	ttc19	488.510091	-0.48647489	0.16369485	-2.97183997	0.00296021	0.01120929
ENSDARG00000059888	ago3a	823.940167	0.716665963	0.24120086	2.97124134	0.00296599	0.01122918
ENSDARG00000023659	gabpb1	384.55501	0.534277196	0.17984045	2.97084	0.00296987	0.01124215
ENSDARG00000042525	ebf2	590.721112	-0.804008663	0.27071383	-2.96995784	0.00297841	0.01127277
ENSDARG00000004635	epha7	1323.09324	0.312449535	0.10521982	2.96949313	0.00298292	0.01128812
ENSDARG00000016733	psat1	2576.75595	1.103874621	0.37175779	2.96933824	0.00298442	0.01129209
ENSDARG00000058346	sass6	162.046364	-0.675748725	0.22758431	-2.96922371	0.00298553	0.01129458
ENSDARG00000039309	hook2	127.611734	0.708090451	0.23848903	2.96906927	0.00298703	0.01129854
ENSDARG00000097269	si:ch211-222n22.1	36.5259157	1.26238601	0.42522428	2.96875333	0.00299011	0.01130672
ENSDARG00000096558	CR556696.1	95.6501658	-0.788715007	0.26567011	-2.96877587	0.00298989	0.01130672
ENSDARG00000012261	eftud2	3220.58769	-0.388132041	0.13075158	-2.96846931	0.00299287	0.01131545
ENSDARG00000077318	CR376737.1	544.38544	-0.514736169	0.17340449	-2.9684132	0.00299342	0.0113158
ENSDARG00000004634	osbp	1674.90402	0.34580998	0.11654567	2.96716293	0.00300562	0.01136019
ENSDARG00000097353	BX324164.3	107.097156	0.986769021	0.33260956	2.96674882	0.00300967	0.01137031
ENSDARG00000057013	cadm3	2264.73141	-0.564599335	0.190304	-2.96682856	0.00300889	0.01137031
ENSDARG00000056625	trpc6a	191.466939	0.552864643	0.18635157	2.96678291	0.00300933	0.01137031
ENSDARG00000039338	gadd45gip1	1014.93842	-0.328621195	0.11077685	-2.96651506	0.00301196	0.01137723
ENSDARG00000103823	rbms1b	259.523093	0.622112605	0.20973381	2.96620082	0.00301504	0.01138713
ENSDARG00000019274	rasd1	1148.71647	0.547749999	0.18471141	2.96543668	0.00302254	0.01141372
ENSDARG00000075730	dnph1	188.611359	-0.755900781	0.25493689	-2.96505058	0.00302633	0.01142632
ENSDARG00000015495	klf3	2183.02456	0.489834348	0.16520563	2.96499794	0.00302685	0.01142654
ENSDARG00000052578	c6ast4	190.815706	-2.824239055	0.95256306	-2.96488407	0.00302797	0.01142904
ENSDARG00000031108	lrba	3883.57884	0.355181855	0.11980909	2.96456514	0.00303111	0.01143915
ENSDARG00000040564	hnrnpul1	4976.21771	-0.325723157	0.1098991	-2.96383824	0.00303828	0.01146447
ENSDARG00000062970	niban2a	1348.54241	0.321618143	0.10853026	2.96339596	0.00304265	0.01147747
ENSDARG00000010516	rpl21	28210.2624	0.301372798	0.10169815	2.96340485	0.00304256	0.01147747
ENSDARG00000094736	znf1157	49.7274593	-0.973416112	0.32851155	-2.96311074	0.00304547	0.01148637
ENSDARG00000101069	CU651669.1	16.050773	2.084540323	0.70351404	2.9630401	0.00304617	0.01148726
ENSDARG00000044179	rbms2a	1832.24129	0.289326298	0.0976785	2.96202638	0.00305622	0.0115234
ENSDARG00000018065	ntm	158.65898	-0.629846343	0.21268272	-2.96143643	0.00306208	0.01154025
ENSDARG00000018846	dgat2	1088.85352	0.627063162	0.21173693	2.96151999	0.00306125	0.01154025
ENSDARG00000101012	CABZ01051600.2	13.6295362	2.360669587	0.7971327	2.96145121	0.00306193	0.01154025
ENSDARG00000075347	chfr	652.241953	0.44421327	0.1500057	2.9613092	0.00306334	0.01154326
ENSDARG00000016934	ttyh2	464.644574	-0.488048454	0.16481314	-2.9612229	0.0030642	0.01154475
ENSDARG00000056741	senp3b	947.495368	-0.350945418	0.11854189	-2.96051813	0.00307122	0.01156769
ENSDARG00000030307	hspa12b	623.539407	-0.403578433	0.13631811	-2.96056365	0.00307077	0.01156769
ENSDARG00000033599	pdia8	588.571093	-0.421306676	0.14231926	-2.96029283	0.00307347	0.0115744
ENSDARG00000069806	sstr2b	26.2405072	-1.959290519	0.66190261	-2.96008881	0.0030755	0.01158031
ENSDARG00000069189	si:dkey-242h9.3	88.3404579	3.549323498	1.1992092	2.95972003	0.00307919	0.01159243
ENSDARG00000093684	tmem238a	451.205518	0.497452466	0.16810448	2.95918618	0.00308453	0.01161077
ENSDARG00000077178	zgc:152977	128.391037	-0.701614132	0.23710935	-2.95903185	0.00308607	0.01161307
ENSDARG00000100791	tril	471.863103	-0.479148204	0.1619273	-2.95903287	0.00308606	0.01161307
ENSDARG00000117130	BX936439.1	143.064841	-0.618416607	0.20900306	-2.95888785	0.00308751	0.01161674
ENSDARG00000061948	amotl2b	1062.35049	-0.387704319	0.13104164	-2.95863451	0.00309005	0.01162453
ENSDARG00000103588	prrt2	872.321886	-0.4998702	0.16897526	-2.95824488	0.00309396	0.01163748
ENSDARG00000057743	si:dkey-10f21.4	178.984423	0.612184533	0.20700767	2.95730369	0.00310342	0.01167129
ENSDARG00000005359	sucla2	8128.33788	0.264747426	0.08955816	2.95615074	0.00311505	0.01171147
ENSDARG00000017742	grm6a	156.452458	-0.864166807	0.29232703	-2.95616455	0.00311491	0.01171147

Table S2. DEGs of WT vs. *terfa-/-*

ENSDARG00000005216	zgc:158328	619.308388	0.522530143	0.17679991	2.95548873	0.00312174	0.01173486
ENSDARG00000035152	ap2b1	7444.02689	0.304627944	0.10307463	2.9554115	0.00312252	0.01173602
ENSDARG00000093642	BX511034.3	8.60986127	2.382140933	0.80608338	2.95520412	0.00312462	0.01174214
ENSDARG00000110481	si:ch73-42p12.2	585.2793	0.375711003	0.12713773	2.95514962	0.00312518	0.01174244
ENSDARG00000105237	mcc	838.671493	0.433853473	0.14682023	2.95499785	0.00312671	0.01174644
ENSDARG00000022788	cops7a	1400.65003	-0.337324743	0.11418323	-2.95424066	0.0031344	0.01177352
ENSDARG00000002593	slc45a2	858.699932	0.474648928	0.1606871	2.95387086	0.00313815	0.01178586
ENSDARG00000061723	tmem64	272.779089	0.522241032	0.17680608	2.95375045	0.00313938	0.01178867
ENSDARG00000062084	clcn1a	200.535371	-0.654914309	0.22172909	-2.95366882	0.00314021	0.01179001
ENSDARG00000090192	si:dkey-16p6.1	41.8015027	0.955638201	0.32358299	2.95330171	0.00314395	0.01179921
ENSDARG00000099509	btr23	40.536465	1.228474832	0.4159624	2.95333143	0.00314364	0.01179921
ENSDARG00000105179	hdac7a	611.46221	0.358056182	0.12123984	2.95328814	0.00314408	0.01179921
ENSDARG00000037760	uncx4.1	513.849314	-0.505259727	0.17110397	-2.95293986	0.00314763	0.01181075
ENSDARG00000081311	dre-mir-124-2	14.655223	-2.600379546	0.88066264	-2.95275333	0.00314954	0.0118151
ENSDARG00000110056	CU929160.1	305.724859	0.615181634	0.20834642	2.95268632	0.00315022	0.0118151
ENSDARG00000073867	rex1bd	315.943175	-0.449097146	0.1520963	-2.95271583	0.00314992	0.0118151
ENSDARG00000062429	trim109	200.921386	0.630555843	0.21359642	2.95208989	0.00315631	0.01183616
ENSDARG00000100012	CR855860.1	183.370835	-0.762884627	0.25842683	-2.95203336	0.00315689	0.01183655
ENSDARG00000056722	cd99l2	9648.53228	-0.419277421	0.1420326	-2.95198029	0.00315743	0.01183679
ENSDARG00000010524	si:ch211-282j22.3	992.907615	0.313766875	0.10630072	2.95169089	0.00316039	0.01184611
ENSDARG00000088861	znf1021	42.2804793	1.303432292	0.4417724	2.95046115	0.003173	0.01188978
ENSDARG00000001769	ptpra	2633.58389	0.386355219	0.13094646	2.95048234	0.00317278	0.01188978
ENSDARG00000070849	rps15	27200.5417	0.298845463	0.10130048	2.95008922	0.00317682	0.01190231
ENSDARG00000088923	si:ch211-12h2.8	11.9350929	2.811963774	0.95328858	2.94975083	0.0031803	0.01191355
ENSDARG00000078815	grk5l	641.081778	-0.378639811	0.12837269	-2.94953552	0.00318252	0.01192004
ENSDARG00000068947	si:ch211-264e16.1	457.357946	0.783465279	0.26562742	2.94948953	0.00318299	0.01192004
ENSDARG00000077792	cdc42ep1b	162.076522	-1.046053851	0.35466668	-2.94939985	0.00318392	0.0119217
ENSDARG00000108937	FO704750.1	396.031263	0.445921886	0.15120379	2.94914492	0.00318655	0.01192795
ENSDARG00000045352	cst14a.2	1466.07552	0.476614271	0.16160882	2.94918481	0.00318613	0.01192795
ENSDARG00000041617	nme5	75.5911094	0.894999684	0.30349659	2.94896127	0.00318844	0.01193324
ENSDARG00000097856	CR377210.1	21.0944717	-1.690517798	0.57342733	-2.94809421	0.0031974	0.01196316
ENSDARG00000087401	slc25a34	381.644594	0.505767628	0.17155535	2.94813083	0.00319702	0.01196316
ENSDARG00000099954	plekhm1	998.358634	0.37689328	0.12785009	2.94793131	0.00319908	0.01196767
ENSDARG00000001803	kcnh6a	340.604029	0.501279752	0.17006982	2.94749395	0.00320361	0.0119828
ENSDARG00000079782	myh7l	2104.93637	0.357294655	0.12123675	2.94708215	0.00320788	0.01199516
ENSDARG00000094763	npy2r	21.5403991	1.480935769	0.5025073	2.94709307	0.00320777	0.01199516
ENSDARG00000060771	map7d3	2209.96375	-0.295962499	0.10042747	-2.94702739	0.00320845	0.01199548
ENSDARG00000006074	uck2a	1022.61354	-0.41670681	0.14141211	-2.94675475	0.00321128	0.01200426
ENSDARG00000103834	rbm14b	2641.92289	-0.412557086	0.14002242	-2.94636449	0.00321533	0.01201761
ENSDARG00000073742	prss59.2	5629.24259	-1.741799634	0.59121276	-2.94614689	0.0032176	0.01202426
ENSDARG00000059768	gpatch8	8113.61412	-0.26547792	0.09012556	-2.94564522	0.00322282	0.0120363
ENSDARG00000024006	rprmb	137.68957	-0.772682826	0.26230734	-2.94571559	0.00322209	0.0120363
ENSDARG00000027199	smad1	1842.99034	0.326723058	0.11091884	2.94560481	0.00322324	0.0120363
ENSDARG00000006456	pdgfrl	1415.27769	0.299457466	0.10166211	2.94561544	0.00322313	0.0120363
ENSDARG00000103359	slitrk5a	624.991525	-0.473416901	0.16071698	-2.94565573	0.00322271	0.0120363
ENSDARG00000093137	BX294189.1	15.4927262	1.935805796	0.65738112	2.94472377	0.00323243	0.01206881
ENSDARG00000091640	si:ch73-352p4.5	110.131894	-0.787420349	0.26744603	-2.94422151	0.00323768	0.0120866
ENSDARG00000079471	si:dkey-88j15.4	4.71413275	5.647236306	1.91824726	2.9439564	0.00324046	0.01209493
ENSDARG00000073810	thbs2b	2013.37012	0.399935225	0.13585147	2.94391541	0.00324089	0.01209493
ENSDARG00000004861	esrrga	1620.18801	-0.397042164	0.13488602	-2.94353834	0.00324484	0.01210785
ENSDARG00000036501	rab39bb	505.332837	-0.459913749	0.1563037	-2.9424367	0.0032564	0.01214919
ENSDARG00000104251	prdm10	1129.02772	-0.494173675	0.16795568	-2.94228611	0.00325799	0.01215328
ENSDARG00000061985	rbm47	3005.2017	0.347072544	0.11796318	2.94221079	0.00325878	0.01215441
ENSDARG00000033171	trappc6bl	384.948232	-0.477473776	0.16229484	-2.94201458	0.00326085	0.01216029
ENSDARG00000019709	rhoua	453.472669	0.585933453	0.1991651	2.94194851	0.00326154	0.01216106
ENSDARG00000105122	BX323031.2	49.8692413	-1.423002099	0.48371588	-2.94181392	0.00326296	0.01216452
ENSDARG00000040984	hspa13	468.285264	0.366651571	0.1246462	2.94153839	0.00326586	0.01217352
ENSDARG00000008184	mia3	2529.3292	0.271571634	0.09233784	2.94106556	0.00327085	0.01219029

Table S2. DEGs of WT vs. *terfa-/-*

ENSDARG00000057394	rab33a	394.809117	-0.521118932	0.17721593	-2.94058742	0.00327591	0.01220729
ENSDARG00000076373	vopp1	557.480007	-0.594480941	0.20217843	-2.94037768	0.00327812	0.01221373
ENSDARG00000095593	si:ch211-134c9.2	15.8609714	-1.834551874	0.62394883	-2.94022809	0.00327971	0.01221779
ENSDARG00000088741	BX322530.1	1232.38843	-0.500341331	0.17017927	-2.94008384	0.00328123	0.01222165
ENSDARG00000007136	gtf2h4	510.547317	-0.36171686	0.12303893	-2.93985693	0.00328364	0.01222877
ENSDARG00000062634	kat2b	428.918862	-0.512238752	0.17436274	-2.93777655	0.00330575	0.01230928
ENSDARG00000012927	tcea2	441.517727	-0.440078231	0.14980577	-2.93765874	0.00330701	0.01231212
ENSDARG00000034600	tmem165	2034.8493	0.290533018	0.09892278	2.93696789	0.00331438	0.01233773
ENSDARG00000098941	CU856520.1	512.645357	-0.666847518	0.22707444	-2.93669123	0.00331734	0.01234689
ENSDARG00000102772	klhdc3	811.591066	0.429948423	0.14641253	2.93655487	0.0033188	0.01235047
ENSDARG00000004246	slit2	3505.01897	0.352735325	0.12013496	2.93615891	0.00332304	0.01236255
ENSDARG00000053003	slc35f1	490.932651	0.545036375	0.18562879	2.93616298	0.003323	0.01236255
ENSDARG00000095912	si:dkey-229b18.3	665.237282	-0.341268029	0.1162325	-2.93608106	0.00332388	0.0123638
ENSDARG00000057021	vps36	686.394512	0.367542988	0.12519593	2.93574235	0.00332751	0.01237545
ENSDARG00000075849	prr12b	5037.76425	-0.417128689	0.14210485	-2.93535867	0.00333162	0.01238891
ENSDARG00000068122	CFAP77	61.1164448	0.84275333	0.28711261	2.93527104	0.00333257	0.01239056
ENSDARG00000030006	slc6a7	594.285549	0.449067176	0.15299429	2.93518918	0.00333344	0.01239198
ENSDARG00000000935	unk	523.182656	-0.474888637	0.16180551	-2.93493483	0.00333618	0.01240028
ENSDARG00000077339	ptrh2	360.930165	0.462758713	0.15767517	2.93488648	0.0033367	0.01240036
ENSDARG00000055648	cpda	2100.37461	0.361574327	0.1232029	2.93478739	0.00333776	0.01240247
ENSDARG00000029388	si:dkey-90m5.4	277.813896	0.686544667	0.23395613	2.93450173	0.00334084	0.01241203
ENSDARG00000093851	CU639468.1	278.87359	-0.614359791	0.20937553	-2.93424824	0.00334357	0.01242032
ENSDARG00000071026	mllt11	2227.05836	-0.454732937	0.1549781	-2.9341754	0.00334435	0.01242138
ENSDARG00000006385	triobpb	1100.90157	0.326855249	0.11139834	2.93411231	0.00334503	0.01242205
ENSDARG00000006868	trh	262.991804	0.687497865	0.23437465	2.93332859	0.00335349	0.01245159
ENSDARG00000016132	keap1a	600.864253	0.397848742	0.13563843	2.93315653	0.00335535	0.01245663
ENSDARG00000041609	adarb1a	944.804067	-0.48273777	0.1646009	-2.93277719	0.00335945	0.01246814
ENSDARG00000018944	hoga1	647.677154	0.485074229	0.16539688	2.93278957	0.00335932	0.01246814
ENSDARG00000079930	si:dkeyp-68b7.5	151.639826	-0.576837385	0.19669229	-2.93268929	0.0033604	0.0124698
ENSDARG00000087390	hbbe1.3	11687.9453	0.88392352	0.30148724	2.93187704	0.0033692	0.0125006
ENSDARG00000099843	nedd4a	1357.42014	0.369629108	0.1260891	2.93149131	0.00337339	0.01251426
ENSDARG00000076227	ror2	911.374876	0.369235992	0.125958	2.93142162	0.00337415	0.0125152
ENSDARG00000053542	kctd12.2	692.828569	0.407319197	0.13897523	2.93087615	0.00338008	0.01253533
ENSDARG00000090585	gpc1b	1036.0004	0.370033601	0.12626141	2.93069447	0.00338205	0.01254079
ENSDARG00000098375	polr1a	1591.12591	0.344644734	0.11761045	2.93039214	0.00338535	0.01254925
ENSDARG00000010270	fezf1	221.393481	-0.807654836	0.27561242	-2.93040077	0.00338525	0.01254925
ENSDARG00000017140	zgc:63587	1936.61835	0.385295988	0.13150131	2.92997839	0.00338986	0.0125641
ENSDARG00000102468	si:dkey-71l4.5	53.0338094	1.289037262	0.43997903	2.92976974	0.00339213	0.01256879
ENSDARG00000009982	fam199x	2167.34857	-0.29706281	0.1013934	-2.92980416	0.00339176	0.01256879
ENSDARG00000000151	thraa	328.463274	0.476291537	0.16258724	2.92945206	0.0033956	0.01257977
ENSDARG00000015793	creb3l1	781.833733	0.372184746	0.1270667	2.92905022	0.0034	0.01259342
ENSDARG00000055552	dvl1b	478.341816	-0.418181808	0.1427718	-2.92902251	0.0034003	0.01259342
ENSDARG00000104479	BX649416.2	86.1391665	-0.920771771	0.31441342	-2.92853836	0.0034056	0.01261117
ENSDARG00000043475	TAGAP	108.728026	-0.772964229	0.26396738	-2.92825659	0.00340869	0.01262072
ENSDARG00000035719	arl5c	422.894664	0.673076846	0.22986944	2.92808321	0.00341059	0.01262588
ENSDARG00000040380	arhgef1a	771.856921	0.349667722	0.11942545	2.92791623	0.00341242	0.01263078
ENSDARG00000037097	slc7a2	1236.55543	0.378659552	0.12933134	2.92782519	0.00341342	0.0126326
ENSDARG00000096797	si:ch73-127m5.2	634.922537	-0.442694404	0.15121841	-2.92751666	0.00341681	0.01264326
ENSDARG00000007960	hnrnpaba	31483.2875	-0.43558781	0.14879427	-2.92745014	0.00341754	0.01264408
ENSDARG00000103160	CR388178.2	33.3725353	-1.151738244	0.39345827	-2.9272183	0.00342009	0.01265163
ENSDARG00000030999	mybl1	418.783076	-0.608687514	0.20796183	-2.92691945	0.00342338	0.0126619
ENSDARG00000102900	tacr1b	100.413301	0.791407487	0.27041494	2.92664119	0.00342644	0.01267135
ENSDARG00000007566	appbp2	1916.71153	-0.298391329	0.10197522	-2.92611602	0.00343223	0.01269087
ENSDARG00000094202	CT583646.2	14.737222	-1.966325505	0.67200552	-2.92605558	0.0034329	0.01269145
ENSDARG00000101894	stk10	936.814436	0.369332051	0.12623632	2.92571939	0.00343661	0.01270328
ENSDARG00000030289	jag1a	454.060933	-0.507446681	0.17348425	-2.92503019	0.00344423	0.01272956
ENSDARG00000112767	LO017951.1	347.569298	-0.433510162	0.14823764	-2.92442705	0.00345091	0.01275236
ENSDARG00000101457	cdc42ep4a	518.18226	-0.418547471	0.14314543	-2.92393182	0.0034564	0.01277076

Table S2. DEGs of WT vs. *terfa-/-*

ENSDARG00000031346	rbm17	914.715692	-0.374073986	0.12794342	-2.92374547	0.00345847	0.01277651
ENSDARG00000090889	LECT2	24.0282528	1.663454682	0.56896496	2.9236505	0.00345953	0.01277851
ENSDARG00000063030	mesd	839.561026	0.462365124	0.15816225	2.92335951	0.00346277	0.01278856
ENSDARG00000101312	si:dkey-31n13.4	219.307883	0.724860138	0.24798369	2.92301533	0.00346659	0.012797
ENSDARG00000102443	shank2a	431.418984	-0.799225469	0.27342316	-2.92303499	0.00346638	0.012797
ENSDARG00000098861	si:dkey-71b5.6	6.44322162	3.33987296	1.14261047	2.92301973	0.00346655	0.012797
ENSDARG00000093444	si:dkey-106l3.7	73.4601376	-0.856136709	0.29291581	-2.92280814	0.0034689	0.01280362
ENSDARG00000021864	rplp1	50439.8061	0.292737371	0.10016781	2.92246954	0.00347268	0.01281564
ENSDARG00000102162	BX005043.1	8.2304978	3.475373549	1.18949673	2.92171761	0.00348107	0.01284471
ENSDARG00000097761	BX005234.1	38.150779	1.367665934	0.46814235	2.92147451	0.00348379	0.01285093
ENSDARG00000089562	BX957322.1	314.856742	-0.546651307	0.18711221	-2.92151597	0.00348332	0.01285093
ENSDARG00000094678	si:dkey-9i23.6	75.2376698	0.90158565	0.30865246	2.92103832	0.00348867	0.01286702
ENSDARG00000010565	aqp4	203.690858	-0.832314125	0.28495086	-2.9209041	0.00349017	0.01287066
ENSDARG00000075417	zgc:175107	128.130081	-0.616453188	0.21105402	-2.9208313	0.00349099	0.01287176
ENSDARG00000020311	cnih1	1215.42632	-0.324972182	0.11127324	-2.92048819	0.00349483	0.01288403
ENSDARG00000045401	mllt10	4361.7012	-0.282566373	0.09677259	-2.91990099	0.00350143	0.01290641
ENSDARG00000076401	cacng3b	995.380455	-0.653815755	0.22392425	-2.91980774	0.00350247	0.01290836
ENSDARG00000030644	gnai3	615.129324	0.359644699	0.12318436	2.91956463	0.00350521	0.01291652
ENSDARG00000070822	cnp	3598.49122	-0.340365603	0.11659416	-2.91923366	0.00350893	0.01292833
ENSDARG00000060383	mmaa	633.041603	0.357988642	0.12263563	2.91912436	0.00351016	0.01293094
ENSDARG00000105441	pcdh11	401.495014	-0.553107693	0.1894826	-2.91904211	0.00351109	0.01293244
ENSDARG00000006062	akap1b	3239.55204	0.231425569	0.07928581	2.91887749	0.00351294	0.01293735
ENSDARG00000037350	rpl9	103.401756	1.18010995	0.40431913	2.9187586	0.00351428	0.01293845
ENSDARG00000043713	asf1bb	959.228228	-0.452090441	0.15489048	-2.91877496	0.0035141	0.01293845
ENSDARG00000060994	fbxw7	2439.9046	0.276230431	0.09464162	2.91869939	0.00351495	0.01293899
ENSDARG00000039173	ctslb	21.2113142	-1.75968911	0.60293412	-2.91854295	0.00351671	0.01294357
ENSDARG00000090181	BX072576.2	103.524669	0.666268958	0.22832032	2.91813254	0.00352135	0.0129587
ENSDARG00000101478	plppr2a	554.073813	-0.606487698	0.20784917	-2.91792222	0.00352372	0.01296266
ENSDARG00000093153	CU137717.1	5.56049807	3.637897617	1.24675252	2.91789875	0.00352399	0.01296266
ENSDARG00000104007	pcdh1g1	102.192263	-1.102157533	0.37772162	-2.91790956	0.00352387	0.01296266
ENSDARG00000023176	tdo2b	28.8888965	-1.341369297	0.4597376	-2.91768458	0.00352641	0.01296965
ENSDARG00000038855	chmp5a	280.076422	-0.605312419	0.20746771	-2.91762227	0.00352711	0.01297032
ENSDARG00000028321	synj2bp	561.661859	0.401818153	0.13773295	2.9173714	0.00352995	0.01297884
ENSDARG00000090997	vegfba	159.775756	-0.615236647	0.21089697	-2.91723799	0.00353146	0.01298247
ENSDARG00000102612	slc15a4	250.64821	0.644429089	0.2209299	2.9168939	0.00353536	0.01299488
ENSDARG00000104985	zgc:110249	75.7875435	-0.807257218	0.27677312	-2.9166749	0.00353784	0.01300021
ENSDARG00000068551	elovl8a	95.4560934	-0.929182641	0.31857613	-2.9166738	0.00353786	0.01300021
ENSDARG00000110460	zgc:174357	5.68467668	4.310001364	1.47779904	2.91650031	0.00353982	0.01300552
ENSDARG00000095148	CT583625.3	7.25165731	2.740897258	0.93985096	2.91631055	0.00354198	0.01301049
ENSDARG00000043168	cela1.5	10.1806029	-2.705200443	0.92761746	-2.91628885	0.00354222	0.01301049
ENSDARG00000010029	spon1a	275.982009	-0.684707324	0.23480988	-2.91600732	0.00354542	0.01302031
ENSDARG00000042561	lpar2b	886.570772	0.351256692	0.12046046	2.9159501	0.00354607	0.01302077
ENSDARG00000067918	elp5	353.82557	-0.53501948	0.18349387	-2.91573494	0.00354852	0.01302783
ENSDARG00000102094	NA	5.37250587	-4.108101659	1.40896897	-2.9156793	0.00354915	0.01302823
ENSDARG00000103001	si:ch73-215d9.1	495.080774	0.538874557	0.1848395	2.91536471	0.00355273	0.01303945
ENSDARG00000008593	nbas	1053.66699	0.407912812	0.13997132	2.91425987	0.00356533	0.01308376
ENSDARG00000032238	dnm3a	283.334728	0.502651915	0.17248475	2.91418179	0.00356622	0.0130851
ENSDARG00000019360	sec23b	4588.94202	0.319235701	0.10957382	2.91343044	0.00357482	0.01311469
ENSDARG00000100781	col28a2b	549.779442	-0.515624969	0.1769888	-2.91331984	0.00357608	0.0131174
ENSDARG00000070432	ino80	1502.89091	-0.283776116	0.09743848	-2.9123619	0.00358707	0.01315576
ENSDARG00000063059	abcg8	71.0911323	0.82821431	0.28438816	2.91226721	0.00358816	0.01315586
ENSDARG00000103692	p4ha3	62.480209	0.874837865	0.30039678	2.91227445	0.00358807	0.01315586
ENSDARG00000006602	chrna2a	112.423292	-0.745611796	0.25603806	-2.91211308	0.00358993	0.01315848
ENSDARG00000092126	vtg5	6.60948014	2.613543256	0.89747308	2.91211325	0.00358992	0.01315848
ENSDARG00000097374	si:dkey-108k21.7	48.3410153	-1.315739821	0.45186386	-2.91180584	0.00359346	0.01316948
ENSDARG00000028845	calcr	230.963191	-0.661966067	0.2273487	-2.91167745	0.00359494	0.01317295
ENSDARG00000014817	ranbp1	4247.67896	-0.350388916	0.12034853	-2.91145151	0.00359754	0.01318054
ENSDARG00000092985	CU499330.1	169.755448	-0.736143109	0.25285341	-2.91134338	0.00359878	0.01318316

Table S2. DEGs of WT vs. *terfa-/-*

ENSDARG00000060176	trmt10b	96.4172016	-0.708966482	0.24355153	-2.91095067	0.00360331	0.0131978
ENSDARG00000114425	med8	499.600131	-0.463590653	0.15926907	-2.91073881	0.00360575	0.0132048
ENSDARG00000103858	arf4a	10.0531361	2.129114461	0.73150174	2.91060751	0.00360727	0.01320841
ENSDARG00000015349	mfge8a	1450.83892	-0.331676218	0.11397411	-2.91010151	0.00361311	0.01322743
ENSDARG00000056652	zmp:0000001102	58.9170552	-1.157493657	0.39775517	-2.91006567	0.00361353	0.01322743
ENSDARG00000069953	kcnq5b	180.760387	-0.581903937	0.20004405	-2.90887905	0.00362727	0.01327383
ENSDARG00000068006	gck	346.43204	-1.866815368	0.64175494	-2.90892249	0.00362677	0.01327383
ENSDARG00000034210	si:dkey-240h12.4	703.965739	0.422096602	0.14512996	2.90840428	0.00363278	0.01329204
ENSDARG00000063921	mt-nd5	38631.3631	-0.32437802	0.11153705	-2.90825366	0.00363453	0.01329649
ENSDARG00000063191	extl2	277.261439	0.438368554	0.15073634	2.90818089	0.00363538	0.01329763
ENSDARG00000000476	pms1	165.411082	-0.562536276	0.19346879	-2.90763313	0.00364175	0.01331898
ENSDARG00000008472	apex2	234.727917	-0.540576837	0.18593788	-2.907298	0.00364566	0.01333129
ENSDARG00000100160	si:dkey-205i10.2	2.78917343	5.325184279	1.8317539	2.90715051	0.00364738	0.01333562
ENSDARG00000020655	INSM2	237.622986	-0.559204913	0.1923748	-2.90685116	0.00365087	0.01334527
ENSDARG00000103687	sycn.2	188.470999	-2.058467616	0.70814809	-2.90683213	0.00365109	0.01334527
ENSDARG00000101762	fah	834.130153	-0.387731234	0.13340629	-2.90639403	0.00365621	0.01336201
ENSDARG00000041959	cxcr4b	208.783953	-0.695613663	0.23938649	-2.90581833	0.00366294	0.01338261
ENSDARG00000070959	si:ch211-288g17.3	11296.2798	-0.45052832	0.15504437	-2.90580259	0.00366313	0.01338261
ENSDARG00000100227	SLC7A1	783.506892	0.408190422	0.14047563	2.90577402	0.00366346	0.01338261
ENSDARG00000086075	ttc3	2989.04838	0.263105627	0.09055011	2.90563578	0.00366508	0.01338656
ENSDARG00000099563	inpp5ka	391.540484	-0.463371138	0.1594798	-2.90551615	0.00366648	0.01338971
ENSDARG00000099368	slc25a42	760.082088	0.392483981	0.13508913	2.90537051	0.00366819	0.01339397
ENSDARG00000023868	si:dkey-172h23.2	952.388705	-0.43111153	0.14839175	-2.90522565	0.00366988	0.0133982
ENSDARG00000027316	tcp11l2	2933.8873	0.301405637	0.1037518	2.90506407	0.00367178	0.01340119
ENSDARG00000057907	si:ch73-181d5.4	1175.44958	-0.415335889	0.14296737	-2.90510968	0.00367124	0.01340119
ENSDARG00000092274	si:dkey-3k20.1	8.8205928	-3.304752025	1.13778045	-2.9045604	0.00367769	0.0134208
ENSDARG00000055429	kat6b	2472.18392	-0.338262095	0.11647828	-2.90407865	0.00368336	0.01343949
ENSDARG00000092719	AL954655.2	142.102539	-0.610512586	0.21023019	-2.90401953	0.00368405	0.01344006
ENSDARG00000093612	si:dkey-92i17.2	110.461179	-0.798508138	0.27497516	-2.90392823	0.00368513	0.013442
ENSDARG00000033498	rorb	965.692786	-1.390682061	0.47903465	-2.90309284	0.00369497	0.01347396
ENSDARG00000039502	eef1a1a	1371.23192	-0.398623168	0.13730831	-2.90312497	0.00369459	0.01347396
ENSDARG00000052690	arrdc3a	3893.78792	0.389909154	0.13433826	2.90244316	0.00370264	0.01349996
ENSDARG00000113510	si:ch211-59d8.2	10.803143	1.991347777	0.68626151	2.90173315	0.00371105	0.01352861
ENSDARG00000006093	cdk15	138.952254	-0.65795937	0.22678759	-2.90121424	0.0037172	0.01354706
ENSDARG00000055976	pecr	519.730668	-0.38880468	0.13401288	-2.90124853	0.00371679	0.01354706
ENSDARG00000093157	si:ch73-236c18.8	396.178473	-0.46314482	0.15964624	-2.90106942	0.00371892	0.01355133
ENSDARG00000058004	six2a	764.417333	-0.327776492	0.11298774	-2.90099163	0.00371984	0.01355271
ENSDARG00000070029	ehhadh	579.84736	0.522569808	0.18015596	2.90065243	0.00372387	0.01356341
ENSDARG00000087324	crygm2d1	11021.7742	0.494369129	0.17043265	2.90067153	0.00372364	0.01356341
ENSDARG00000029841	tmprss9	540.084519	-0.635837125	0.21921224	-2.90055481	0.00372503	0.01356565
ENSDARG00000075834	si:dkey-182i3.8	190.393322	-0.765470975	0.26394115	-2.90015777	0.00372975	0.01358085
ENSDARG00000094770	CT583625.2	6.87211787	2.989264486	1.03081748	2.89989697	0.00373285	0.01359017
ENSDARG00000062134	kcnab2b	282.921587	-0.656410777	0.22636927	-2.89973446	0.00373479	0.01359522
ENSDARG00000073814	pax1b	153.810438	-0.675184556	0.23286806	-2.89942965	0.00373842	0.01360646
ENSDARG00000115564	si:ch211-271g18.4	8.68402333	2.58429787	0.89136373	2.89926299	0.00374041	0.0136117
ENSDARG00000073665	rasa1b	464.681896	0.452852251	0.15620608	2.89906928	0.00374272	0.01361812
ENSDARG00000110614	ugt2a7	23.4020051	1.434457655	0.49484775	2.89878584	0.00374611	0.01362844
ENSDARG00000096727	CT737227.1	6.21722693	-3.737597008	1.28942007	-2.89866514	0.00374755	0.01363169
ENSDARG00000099440	CR936249.1	10.0423465	-2.407153937	0.83053997	-2.89829993	0.00375192	0.01364558
ENSDARG00000097772	si:ch211-185a18.2	252.445783	0.525978333	0.18149569	2.89802109	0.00375525	0.01365572
ENSDARG00000098226	smpd3	509.366141	-0.465057155	0.16053084	-2.8969957	0.00376755	0.01369843
ENSDARG00000004598	or102-2	3.31320958	-5.172526259	1.78556228	-2.8968613	0.00376916	0.01370135
ENSDARG00000101256	CABZ01085052.1	2270.67606	0.353265004	0.12194853	2.896837	0.00376946	0.01370135
ENSDARG00000100961	zgc:174653	20.2484973	-1.631492565	0.56324327	-2.89660373	0.00377226	0.01370753
ENSDARG00000117735	CR926130.1	96.5604324	1.19838234	0.41371533	2.89663512	0.00377188	0.01370753
ENSDARG00000098212	CABZ01024848.1	3.59434439	5.275413617	1.82131632	2.89648403	0.0037737	0.01371076
ENSDARG00000077326	slc45a4b	1245.80569	0.355496926	0.12273943	2.89635472	0.00377525	0.0137144
ENSDARG00000071685	slco5a1a	577.169629	0.490254194	0.16931545	2.89550781	0.00378546	0.01374945

Table S2. DEGs of WT vs. *terfa-/-*

ENSDARG00000094559	zgc:174855	36.674411	-1.633850357	0.56428906	-2.89541384	0.00378659	0.01375156
ENSDARG00000041853	rbm39b	3225.03881	-0.262579248	0.09069621	-2.89515117	0.00378976	0.01376106
ENSDARG00000005468	irf2bp1	2373.12446	0.254621154	0.08797627	2.89420275	0.00380123	0.01380068
ENSDARG00000005085	ggctb	971.161156	0.474416214	0.16395817	2.89351991	0.0038095	0.01382871
ENSDARG00000078560	slc9a5	197.589022	-0.584386667	0.20199759	-2.89303783	0.00381535	0.01384793
ENSDARG00000003699	atp7a	1550.26337	0.369997171	0.12789489	2.89297857	0.00381607	0.01384852
ENSDARG00000054799	rfc1	1142.72263	-0.36659777	0.12672363	-2.892892	0.00381713	0.01385031
ENSDARG00000088030	rpl35a	21013.2177	0.284426575	0.09832471	2.89272734	0.00381913	0.01385555
ENSDARG00000058649	trim46b	548.68651	-0.504666987	0.17447596	-2.8924729	0.00382222	0.01386476
ENSDARG00000074544	znf1007	675.948662	-0.436645189	0.1509629	-2.89240059	0.0038231	0.01386593
ENSDARG00000062749	ino80b	869.702991	-0.328772711	0.11367086	-2.89232173	0.00382406	0.01386697
ENSDARG00000079915	pot1	173.72425	-0.790761018	0.27340353	-2.89228529	0.00382451	0.01386697
ENSDARG00000007175	recql	755.876636	0.391634776	0.13542462	2.89190229	0.00382917	0.01388064
ENSDARG00000076838	apom	4001.78197	0.462363944	0.15988329	2.89188412	0.00382939	0.01388064
ENSDARG00000095861	si:ch1073-441p17.	6.79117224	3.855812622	1.33360931	2.89126103	0.00383699	0.01390616
ENSDARG00000103725	znf341	411.527193	0.441989813	0.15289574	2.89079228	0.00384272	0.01392489
ENSDARG00000076530	jac6	3.4061225	4.481860906	1.55044957	2.89068473	0.00384404	0.01392559
ENSDARG00000012796	hnmt	246.341593	0.652161953	0.22560727	2.89069562	0.0038439	0.01392559
ENSDARG00000052997	sema4e	966.734527	0.395791481	0.13692704	2.89052823	0.00384595	0.01393043
ENSDARG00000093773	si:ch1073-296i8.2	100.366517	-0.862633259	0.29843904	-2.890484	0.00384649	0.01393043
ENSDARG00000098620	si:ch211-188p14.2	176.554768	0.798222558	0.27617062	2.89032399	0.00384845	0.01393549
ENSDARG00000052192	s1pr5b	74.1976536	0.972537018	0.3365227	2.88995968	0.00385291	0.01394962
ENSDARG00000017720	si:ch211-106h11.1	234.372746	0.71815716	0.2485112	2.88983827	0.0038544	0.01395298
ENSDARG00000020957	pth1r	102.774088	-0.878303101	0.30395677	-2.88956581	0.00385774	0.01396304
ENSDARG00000091885	grnas	3.93303818	-5.218277054	1.80609645	-2.88925714	0.00386153	0.01397268
ENSDARG00000101048	FQ790208.1	13.9673135	-1.981751235	0.68590117	-2.88926643	0.00386142	0.01397268
ENSDARG00000086310	cabcoco1	31.3279002	1.155469494	0.39999232	2.88872918	0.00386802	0.01399413
ENSDARG00000060103	cpeb3	416.753347	0.496674851	0.17194149	2.88862707	0.00386928	0.0139946
ENSDARG00000077664	zgc:110239	1430.94002	0.432456625	0.14970954	2.8886377	0.00386915	0.0139946
ENSDARG00000042534	rsrc2	2135.08861	-0.296999082	0.10282284	-2.88845445	0.0038714	0.01399954
ENSDARG00000079020	gcfc2	401.106955	-0.508308216	0.17598114	-2.88842441	0.00387177	0.01399954
ENSDARG00000079705	si:ch211-152p11.4	242.358035	0.573327736	0.19850944	2.88816358	0.00387498	0.01400912
ENSDARG00000030156	naglu	431.351152	0.465206142	0.16109842	2.88771387	0.00388053	0.01402712
ENSDARG00000040528	lgals3bpb	361.479457	-0.551699759	0.19105687	-2.8876206	0.00388168	0.01402924
ENSDARG00000057928	mzt2b	667.778011	-0.42826007	0.1483397	-2.88702271	0.00388906	0.01405389
ENSDARG00000059048	mpzl1l	1191.02743	0.36804327	0.12748423	2.88697104	0.0038897	0.01405415
ENSDARG00000110110	CU929146.3	10.2908476	3.106153245	1.07595644	2.8868764	0.00389087	0.01405634
ENSDARG00000092863	CU138515.1	3.78283881	-5.394567203	1.86875897	-2.88671108	0.00389292	0.01406168
ENSDARG00000053358	basp1	6927.12129	-0.421649859	0.14608297	-2.88637252	0.00389711	0.01407477
ENSDARG00000010738	zgc:101716	42.6166521	1.388513044	0.48114272	2.88586524	0.00390339	0.01409543
ENSDARG00000075584	itfg1	659.277651	0.310100254	0.107475	2.88532447	0.0039101	0.01411761
ENSDARG00000062916	mrpl37	1474.43149	0.267199099	0.09261196	2.88514676	0.00391231	0.01412353
ENSDARG00000101280	vat1l	95.4556668	0.780511265	0.27058507	2.88453192	0.00391996	0.01414909
ENSDARG00000100002	ctbs	201.669679	0.93996859	0.32605334	2.88286755	0.00394073	0.01422199
ENSDARG00000022085	si:dkey-34e4.1	501.000911	-0.550597644	0.19102508	-2.88233177	0.00394744	0.01424413
ENSDARG00000051798	brd1b	1564.43816	-0.347734455	0.12064943	-2.882189	0.00394923	0.01424852
ENSDARG00000030782	exoc3l2b	841.334548	0.517668677	0.1796486	2.88156248	0.00395709	0.01427481
ENSDARG00000098972	mrps16	532.356713	-0.385103651	0.133647	-2.88149865	0.00395789	0.01427563
ENSDARG00000010442	rnf11a	801.265154	-0.341054786	0.11838614	-2.88086762	0.00396582	0.01430216
ENSDARG00000036630	plp2	1834.47694	0.297193287	0.10316509	2.88075435	0.00396725	0.01430523
ENSDARG00000003281	pik3ip1	1048.14333	-0.467501697	0.16230708	-2.88035301	0.0039723	0.01432137
ENSDARG00000104569	CABZ01088484.1	195.187326	1.592046588	0.55276299	2.88016134	0.00397472	0.01432801
ENSDARG00000100401	TLX3	38.7148044	-1.255936623	0.43610602	-2.87988829	0.00397816	0.01433834
ENSDARG00000091879	si:dkey-29d8.3	119.990225	-0.689590913	0.23956034	-2.87856876	0.00399484	0.01439622
ENSDARG00000078094	lmf2a	269.250395	0.451157796	0.15673221	2.87852637	0.00399538	0.01439622
ENSDARG00000007024	uox	788.245496	-0.71917196	0.2499322	-2.87746825	0.0040088	0.0144425
ENSDARG00000102493	ticam1	203.768607	0.689318746	0.23958608	2.87712348	0.00401319	0.01445619
ENSDARG00000097208		361.095953	0.745120224	0.25899299	2.87698994	0.00401488	0.01446021

Table S2. DEGs of WT vs. *terfa-/-*

ENSDARG00000098793	CABZ01078120.1	3.7426762	4.534091349	1.57643377	2.8761699	0.00402533	0.01449574
ENSDARG00000058105	rpl36a	22062.579	0.293293746	0.10201992	2.8748675	0.00404197	0.01455018
ENSDARG00000056156	npdc1b	1313.89908	-0.366544453	0.12749731	-2.87491914	0.00404131	0.01455018
ENSDARG00000040031	tardbp	8031.83801	-0.392891332	0.13666503	-2.87484917	0.00404221	0.01455018
ENSDARG00000002877	reps1	1317.40374	0.304985855	0.1061082	2.87429107	0.00404936	0.01457381
ENSDARG00000056517	thoc3	761.464322	-0.369669054	0.12862051	-2.87410667	0.00405172	0.01457854
ENSDARG00000091511	gpx7	387.918148	0.461703252	0.16064288	2.87409716	0.00405184	0.01457854
ENSDARG00000055843	cdh10a	484.851182	-0.459370021	0.15986186	-2.87354356	0.00405895	0.014602
ENSDARG00000014031	abcc2	2155.63714	0.391640852	0.13630764	2.87321283	0.0040632	0.01461518
ENSDARG00000031325	tmed1a	322.230242	0.501155563	0.17444927	2.87278689	0.00406869	0.01463278
ENSDARG00000004074	bach2b	2078.33925	-0.558990575	0.1946152	-2.87228636	0.00407514	0.01465386
ENSDARG00000112670	smim20	280.200516	-0.4705967	0.16385282	-2.87206967	0.00407793	0.01466179
ENSDARG00000040001	fryl	4634.66191	0.329701406	0.11480279	2.87189368	0.0040802	0.01466784
ENSDARG00000016161	ccdc170	49.0972197	1.261017305	0.43914975	2.87149726	0.00408532	0.01468412
ENSDARG00000012387	pdha1a	9212.93639	0.240069794	0.08361476	2.87114143	0.00408993	0.01469854
ENSDARG00000063149	tmtc1	1402.75575	-0.335669058	0.11691989	-2.87093196	0.00409264	0.01470615
ENSDARG00000075618	slc36a1	556.101759	0.517303444	0.18019639	2.87077578	0.00409466	0.0147113
ENSDARG00000107947	CABZ01081385.1	442.218398	0.411255003	0.14326056	2.87067853	0.00409592	0.01471369
ENSDARG00000011618	slc26a2	1424.54797	0.403367607	0.1405179	2.87057806	0.00409722	0.01471624
ENSDARG00000100392	rps18	21084.7083	0.281360275	0.09803561	2.86998047	0.00410497	0.01474196
ENSDARG00000043705	mob3c	132.711846	0.693690469	0.24172623	2.86973605	0.00410815	0.01475122
ENSDARG00000058479	zgc:110425	18.3543047	-1.681909182	0.58616219	-2.86935804	0.00411306	0.01476515
ENSDARG00000099301	cdc37	4607.13798	0.334479546	0.11656995	2.86934628	0.00411321	0.01476515
ENSDARG00000020845	tns1b	2059.62805	0.535013177	0.186466	2.86922638	0.00411477	0.01476861
ENSDARG00000051761	trim44	149.813786	-0.930486992	0.32434011	-2.8688619	0.00411952	0.01478351
ENSDARG00000099845	zgc:174275	81.8940294	0.929911202	0.32415914	2.86868726	0.00412179	0.01478954
ENSDARG00000097637	si:ch211-246i5.5	490.726786	-0.464949496	0.16210256	-2.86824278	0.00412759	0.01480819
ENSDARG00000098823	PDZD4	734.662443	-0.429358911	0.14971906	-2.86776384	0.00413384	0.01482849
ENSDARG00000089717	qpct	281.962875	0.703711928	0.24539092	2.8677179	0.00413444	0.0148285
ENSDARG00000061850	si:dkey-19b23.13	207.074921	-0.675980115	0.23572662	-2.86764437	0.0041354	0.01482981
ENSDARG00000036227	npvf	76.9328824	1.260116508	0.43943283	2.86759758	0.00413601	0.01482986
ENSDARG00000017901	tln2a	3364.69332	0.329295709	0.11485164	2.86713976	0.004142	0.01484596
ENSDARG00000074726	nrde2	329.865496	-0.461917821	0.16110732	-2.8671436	0.00414195	0.01484596
ENSDARG00000045803	mfge8b	429.865303	-0.536871736	0.1872514	-2.86711735	0.00414229	0.01484596
ENSDARG00000115962	LO017799.1	63.795933	-1.062120692	0.37047125	-2.86694496	0.00414455	0.01485111
ENSDARG00000104786	nlgn3a	891.774106	-0.38037117	0.13267607	-2.86691624	0.00414493	0.01485111
ENSDARG00000031680	ercc1	260.393647	-0.508301155	0.17734492	-2.86617267	0.00415468	0.0148839
ENSDARG00000100883	CU896630.2	76.3242071	-0.859119547	0.29976333	-2.86599279	0.00415704	0.01489021
ENSDARG00000010487	sae1	1452.48657	-0.552922886	0.19295486	-2.86555569	0.00416278	0.01490863
ENSDARG00000105506	CR769769.3	4.31605247	4.833575629	1.68687821	2.86539692	0.00416487	0.01491396
ENSDARG00000029170	si:ch211-200p22.4	4031.12173	0.253400454	0.08843844	2.86527497	0.00416647	0.01491699
ENSDARG00000020964	thap12b	384.290539	-0.430561505	0.15027059	-2.86524132	0.00416692	0.01491699
ENSDARG00000004473	tbx21	136.902969	-0.606471812	0.21168251	-2.86500678	0.00417	0.0149259
ENSDARG00000077298	gas1a	2239.2218	-0.324925077	0.11342254	-2.86473122	0.00417363	0.01493506
ENSDARG00000014717	dync1h1	9272.31618	0.56804974	0.19829146	2.86472116	0.00417377	0.01493506
ENSDARG00000097551	si:dkey-7i4.15	139.008607	-0.581736024	0.20307306	-2.86466373	0.00417452	0.01493562
ENSDARG00000039871	rpz4	516.737662	0.430614225	0.15032963	2.86446675	0.00417712	0.01494061
ENSDARG00000070487	1-Mar	324.351507	0.462071328	0.16130949	2.86450192	0.00417666	0.01494061
ENSDARG00000079075	si:ch211-71m22.3	11.5986874	-2.44688594	0.85430581	-2.86418039	0.0041809	0.01494982
ENSDARG00000055523	slc22a6l	1069.60133	0.647244666	0.22597755	2.86419894	0.00418065	0.01494982
ENSDARG00000042656	nrn1b	24.3074478	-1.554011476	0.54258256	-2.86410141	0.00418194	0.0149514
ENSDARG00000063097	scube1	741.016451	0.442756477	0.1546205	2.86350436	0.00418983	0.01497417
ENSDARG00000033418	gpatch1	1141.40505	-0.293269004	0.10241391	-2.86356604	0.00418902	0.01497417
ENSDARG00000074635	abca1a	8668.55984	0.345566547	0.12068221	2.86344233	0.00419065	0.01497417
ENSDARG00000089086	mustn1b	201.738565	0.700744821	0.24472156	2.86343722	0.00419072	0.01497417
ENSDARG00000033950	lamb2l	2464.21892	0.379602228	0.13257361	2.8633318	0.00419211	0.014977
ENSDARG00000018008	mdh1b	44.683746	1.039407657	0.36303166	2.86313227	0.00419475	0.01498213
ENSDARG00000100890	si:zfos-1069f5.1	13.0952513	-1.896137609	0.66225592	-2.86314934	0.00419453	0.01498213

Table S2. DEGs of WT vs. *terfa-/-*

ENSDARG00000098387	tmem176l.3a	1227.95993	0.478611217	0.16718641	2.86274003	0.00419995	0.01499853
ENSDARG00000103537	ttll12	298.894612	0.666853579	0.23297755	2.86230829	0.00420568	0.01501633
ENSDARG00000092517	gpsm1a	1011.11033	-0.448044106	0.15653686	-2.86222757	0.00420675	0.01501633
ENSDARG00000091058	sting1	42.921047	0.993080701	0.34695649	2.8622629	0.00420628	0.01501633
ENSDARG00000097496	si:dkey-6e2.2	691.105326	-0.363994551	0.12717421	-2.86217276	0.00420748	0.01501677
ENSDARG00000058248	si:dkeyp-77h1.4	774.10965	0.609689678	0.21302999	2.86198988	0.0042099	0.01502328
ENSDARG00000110192	CABZ01081535.1	241.035533	0.544490343	0.19028929	2.86138202	0.00421799	0.01504996
ENSDARG00000096099	BX465862.3	31.0577137	1.458237172	0.50964399	2.8612859	0.00421926	0.01505025
ENSDARG00000045248	h3f3d	20173.0572	-0.425987343	0.14887974	-2.86128484	0.00421928	0.01505025
ENSDARG00000005332	lipib	69.6942054	0.816000052	0.2852108	2.8610419	0.00422251	0.01505963
ENSDARG00000093448	mpc1	2800.88098	0.285198121	0.09968964	2.86086024	0.00422493	0.0150661
ENSDARG00000031587	flvcr1	756.046301	-0.373472279	0.13054827	-2.86079839	0.00422576	0.01506688
ENSDARG00000008703	apeh	1246.02243	0.356334146	0.12458223	2.86023246	0.00423331	0.01509163
ENSDARG00000029259	zgc:136493	160.447999	0.567878723	0.19854615	2.86018508	0.00423394	0.01509172
ENSDARG00000051986	ndufs8a	7392.9927	0.256648187	0.08973331	2.86012178	0.00423478	0.01509257
ENSDARG00000032725	rps27a	37475.0909	0.283068615	0.09897548	2.85998724	0.00423658	0.0150968
ENSDARG00000068851	rnf183	783.525707	0.40765403	0.14254409	2.85984522	0.00423848	0.01509924
ENSDARG00000044942	mrpl45	699.091704	0.327921976	0.11466344	2.85986509	0.00423821	0.01509924
ENSDARG00000068621	si:ch211-181d7.3	410.688373	0.701709133	0.24537136	2.85978415	0.00423929	0.01509998
ENSDARG00000099247	si:dkey-68o6.5	59.0272864	-1.014538676	0.35478788	-2.85956412	0.00424224	0.0151083
ENSDARG00000077244	setd1bb	36.8176874	-1.237599964	0.43288725	-2.85894297	0.00425055	0.01513574
ENSDARG00000052045	ggt5a	708.21788	-0.346753011	0.12132674	-2.85800983	0.00426307	0.01517815
ENSDARG00000022952	zgc:66448	1632.36738	-0.29565302	0.10345543	-2.85778146	0.00426614	0.0151869
ENSDARG00000099156	katnal1	645.074632	-0.580155187	0.20303432	-2.85742424	0.00427095	0.01520183
ENSDARG00000054898	ms4a17a.16	14.8391501	-2.028310495	0.70990534	-2.85715629	0.00427455	0.01521249
ENSDARG00000057706	si:ch211-137i24.1(	622.589874	0.588867586	0.20612715	2.85681725	0.00427912	0.01522657
ENSDARG00000055295	cyb561d1	127.861507	0.689695499	0.24143168	2.85669015	0.00428084	0.01523048
ENSDARG00000116246	ccs	61.1824097	1.512930769	0.52963751	2.85654005	0.00428286	0.01523551
ENSDARG00000098852	smdt1b	1188.34666	0.406757935	0.142399	2.85646633	0.00428385	0.01523686
ENSDARG00000101844	mibp2	14834.554	0.345666749	0.12102165	2.8562389	0.00428692	0.01524342
ENSDARG00000016089	bicral	918.477975	-0.351649435	0.12311622	-2.85623978	0.00428691	0.01524342
ENSDARG00000069467	igsf9bb	307.347184	0.747548998	0.26173718	2.85610547	0.00428873	0.01524765
ENSDARG00000103827	znf1144	139.498192	0.791366236	0.27712182	2.85566194	0.00429472	0.01526678
ENSDARG00000056601	plekha3	380.328739	-0.411709422	0.14417835	-2.85555657	0.00429615	0.01526966
ENSDARG00000026359	pbld2	479.041465	0.433344138	0.15176627	2.85533898	0.00429909	0.01527794
ENSDARG00000103296	nup54	894.267189	-0.486964531	0.17056507	-2.85500742	0.00430358	0.01529171
ENSDARG00000060148	sh3pxd2aa	1090.86505	-0.422970677	0.14815841	-2.85485427	0.00430566	0.0152969
ENSDARG00000010301	b4galt6	1658.71367	-0.308292535	0.10799352	-2.85473183	0.00430732	0.01530061
ENSDARG00000033852	mad1l1	655.993062	-0.435001025	0.15241851	-2.85399084	0.00431738	0.01533416
ENSDARG00000116430	si:ch73-329n5.2	12.9833503	2.157747454	0.75621354	2.85335734	0.004326	0.01536257
ENSDARG00000089187	wfdc2	418.526278	0.583141318	0.20439298	2.85303986	0.00433032	0.01537573
ENSDARG00000042418	galnt17	499.493618	-0.594205975	0.20829163	-2.85275977	0.00433414	0.01538709
ENSDARG00000101510	cetn2	393.072561	-0.433531892	0.15200074	-2.85216961	0.00434219	0.01541348
ENSDARG00000061257	C3H17orf75	942.440477	-0.448238195	0.15721223	-2.85116612	0.00435592	0.01546
ENSDARG00000098650	iqgap3	2108.28227	-0.649540766	0.22782966	-2.85099296	0.00435829	0.01546401
ENSDARG00000007436	avpr2aa	134.040875	-0.690132067	0.24206536	-2.85101539	0.00435799	0.01546401
ENSDARG00000037914	znfl1	11.5130843	-2.590330636	0.90867206	-2.85067711	0.00436263	0.01547717
ENSDARG00000073850	hdac7b	53.5370651	-1.13862778	0.39946516	-2.85038068	0.00436669	0.01548878
ENSDARG00000039701	emx2	815.388806	-0.501190774	0.17583495	-2.85034787	0.00436714	0.01548878
ENSDARG00000074378	junba	993.646175	0.661710048	0.23221833	2.84951693	0.00437857	0.01552708
ENSDARG00000044339	rp2	538.116852	-0.362632159	0.12727482	-2.84920579	0.00438285	0.01553784
ENSDARG00000102717	tmem42a	143.032878	0.688121926	0.24151146	2.84923097	0.00438251	0.01553784
ENSDARG00000070743	tmem45a	431.085408	0.467793371	0.1641954	2.84900418	0.00438563	0.01554547
ENSDARG00000070954	hmx2	199.843856	-0.5150668	0.18080303	-2.84877303	0.00438882	0.01555455
ENSDARG00000075795	nol7	649.879649	-0.435166377	0.15278116	-2.84829867	0.00439537	0.01557554
ENSDARG00000069453	zgc:113314	150.740536	0.913610726	0.32080839	2.84783922	0.00440172	0.01559582
ENSDARG00000069497	sumf2	196.792141	0.531439715	0.18661614	2.84776935	0.00440268	0.01559702
ENSDARG00000071049	si:dkey-119m7.4	155.697626	0.598234152	0.21016165	2.84654295	0.00441968	0.01565276

Table S2. DEGs of WT vs. *terfa-/-*

ENSDARG00000098925	prdm1b	577.297104	-0.691457415	0.24290971	-2.84656145	0.00441942	0.01565276
ENSDARG00000052122	rag1	59.6513893	-1.060534803	0.37257605	-2.84649217	0.00442038	0.01565303
ENSDARG00000076568	sec61b	3426.8264	0.322241407	0.11321236	2.84634466	0.00442243	0.01565805
ENSDARG00000079133	si:dkey-10c21.1	28.5379375	1.397524949	0.49100542	2.84625159	0.00442372	0.0156604
ENSDARG00000005675	sec61a1l	1051.84674	0.341669162	0.1200479	2.84610687	0.00442573	0.01566528
ENSDARG00000096273	si:dkey-3n22.9	71.6835965	1.490608395	0.5238514	2.84547947	0.00443446	0.01569281
ENSDARG00000038954	ctnnbip1	2351.76714	-0.454139271	0.15960152	-2.84545706	0.00443477	0.01569281
ENSDARG00000030824	esyt2a	1289.19819	0.315747487	0.11097675	2.84516793	0.0044388	0.01570483
ENSDARG00000088390	nme4	135.366695	-0.632559205	0.22234503	-2.84494416	0.00444192	0.01571363
ENSDARG00000026017	xpnpep2	142.29008	0.968210533	0.34047656	2.84369219	0.00445941	0.01577325
ENSDARG00000003899	zdhhc23b	116.047202	0.613025229	0.2156731	2.8423815	0.00447779	0.015836
ENSDARG00000114870	CABZ01078594.1	4019.17725	0.391395704	0.13770256	2.84232693	0.00447855	0.01583646
ENSDARG00000081977	dre-mir-729	5.67133175	-3.708996479	1.30499737	-2.84214863	0.00448106	0.01584306
ENSDARG00000091637	pip4k2cb	14.6358282	-2.303194546	0.81047239	-2.84179275	0.00448606	0.0158585
ENSDARG00000079305	hbae3	45969.5921	0.474539751	0.16699451	2.84164876	0.00448809	0.01586275
ENSDARG00000020488	arhgap17a	721.95133	0.451910096	0.15903275	2.84161661	0.00448854	0.01586275
ENSDARG00000117696	CABZ01068362.1	39.8750783	1.103717319	0.38842759	2.84150083	0.00449017	0.01586626
ENSDARG00000089787	crebzf	1598.72921	-0.308019287	0.10842696	-2.84079994	0.00450005	0.01589891
ENSDARG00000056163	slc25a51a	144.593182	-0.607857335	0.21406888	-2.83954094	0.00451785	0.01595952
ENSDARG00000099652	xpot	3428.66822	0.342942934	0.12079907	2.83895336	0.00452618	0.01598212
ENSDARG00000079043	si:dkeyp-75b4.10	7.07481808	2.632902809	0.92741688	2.83896365	0.00452603	0.01598212
ENSDARG00000094938	FO834814.1	13.948713	-2.057944547	0.72488642	-2.83898897	0.00452567	0.01598212
ENSDARG00000006257	vldlr	2975.99516	-0.443511705	0.15623439	-2.83875855	0.00452894	0.01598961
ENSDARG00000069074	cry3a	11904.8559	0.290956021	0.10251151	2.83827662	0.00453579	0.0160115
ENSDARG00000056847	si:dkey-27c15.3	296.50896	-0.490347548	0.17276594	-2.83821888	0.00453661	0.01601212
ENSDARG00000078310	cecr2	1156.27507	-0.327481914	0.11539082	-2.83802399	0.00453938	0.01601963
ENSDARG00000090572	si:dkey-63j12.4	6.57245527	-4.784451143	1.68592039	-2.83788675	0.00454133	0.01602424
ENSDARG00000094336	si:dkeyp-82a1.6	334.774897	-0.423738269	0.14935381	-2.83714402	0.00455191	0.01605929
ENSDARG00000101088	si:ch73-359m17.5	73.802852	1.329220707	0.4686067	2.83653797	0.00456056	0.01608751
ENSDARG00000027153	gabrr3a	154.476797	-0.816814181	0.28796902	-2.83646542	0.00456159	0.01608878
ENSDARG00000045342	si:ch211-140b10.6	240.927369	-0.51875631	0.18289108	-2.83642217	0.00456221	0.01608878
ENSDARG00000091317	hunk	2627.4709	-0.385297841	0.1358429	-2.83634872	0.00456326	0.0160902
ENSDARG00000036158	nudcd1	614.636415	0.351636546	0.12399259	2.835948	0.00456899	0.01610811
ENSDARG00000101982	spryd3	627.672164	0.361958419	0.12763454	2.83589719	0.00456972	0.01610839
ENSDARG00000040725		468.628842	-0.493465523	0.17402717	-2.83556598	0.00457446	0.01612282
ENSDARG00000071678	atcaya	946.724015	-0.359842662	0.12691189	-2.83537397	0.00457721	0.01613023
ENSDARG00000019103	kdm4ab	1167.88013	-0.315266402	0.11121066	-2.83485772	0.00458461	0.01615173
ENSDARG00000009342	txndc5	5311.09009	0.273688251	0.09654295	2.83488593	0.00458421	0.01615173
ENSDARG00000059256	hoxd13a	50.4754682	1.01814515	0.35916316	2.83477059	0.00458586	0.01615385
ENSDARG00000088167	si:ch211-112g6.4	27.5412863	1.791051208	0.63185801	2.83457862	0.00458862	0.01616127
ENSDARG00000043342	gpx3	258.153043	0.732754675	0.25853859	2.83421781	0.0045938	0.01617724
ENSDARG00000043514	si:dkey-239i20.4	112.975598	1.10096348	0.38854409	2.83356125	0.00460325	0.01620821
ENSDARG00000100674	UBE2O	785.431365	-0.356291218	0.12574207	-2.83350838	0.00460401	0.01620859
ENSDARG00000076867	mrtfbb	869.982533	0.452324355	0.15965148	2.83319858	0.00460848	0.01622201
ENSDARG00000079589	si:dkeyp-73d8.6	12.659425	3.806160696	1.34350706	2.83300387	0.00461128	0.01622856
ENSDARG00000076791	cdk13	2975.11118	-0.318157501	0.11230493	-2.83297907	0.00461164	0.01622856
ENSDARG00000100185	elovl7b	1142.3283	0.441830489	0.15596559	2.8328716	0.00461319	0.01623172
ENSDARG00000002754	pdcd2l	231.635644	0.544252228	0.1921518	2.83240766	0.00461989	0.01625299
ENSDARG00000115129	AL935174.4	52.8072636	-1.033395897	0.3648786	-2.83216364	0.00462342	0.0162631
ENSDARG00000104598	pcdh1a6	73.2415272	-0.868727664	0.30676181	-2.831929	0.00462681	0.01627043
ENSDARG00000060847	setd1ba	1341.08617	-0.337147353	0.11905167	-2.83194135	0.00462663	0.01627043
ENSDARG00000111091	FO082779.1	281.096957	-0.419039214	0.14797445	-2.83183492	0.00462817	0.01627292
ENSDARG00000002060	brf2	89.4950345	-0.811985821	0.286754	-2.83164601	0.00463091	0.01628023
ENSDARG00000053728	ttll3	70.7694479	0.893732163	0.31564118	2.83148147	0.00463329	0.01628631
ENSDARG00000059209	abhd13	306.111819	0.483960465	0.17094042	2.8311646	0.00463789	0.01630015
ENSDARG00000079027	chsy1	1079.59451	0.313290118	0.11066334	2.83101998	0.00463998	0.01630291
ENSDARG00000074098	si:ch211-86h15.1	225.475854	-0.476356691	0.16826304	-2.83102397	0.00463992	0.01630291
ENSDARG00000018476	pak2b	3791.3144	-0.385436611	0.13615437	-2.83087952	0.00464202	0.01630776

Table S2. DEGs of WT vs. *terfa-/-*

ENSDARG00000112702	BX537263.3	5.07703041	4.038357986	1.42668478	2.83058882	0.00464624	0.01632028
ENSDARG00000075354	espl1	1219.52137	-0.564184084	0.19932242	-2.83050997	0.00464739	0.016322
ENSDARG00000102378	CABZ01003056.1	15.8282983	1.917522252	0.67755848	2.83004686	0.00465412	0.01634333
ENSDARG00000038585	eif4e2	585.341332	0.38107556	0.13468364	2.82941236	0.00466336	0.01637346
ENSDARG00000098066	CU302323.1	3.67523482	4.744079517	1.67678512	2.829271	0.00466542	0.01637838
ENSDARG00000075930	adamts2	539.139843	0.404996277	0.14314942	2.82918556	0.00466666	0.01638044
ENSDARG00000103634	CU914622.2	121.348102	1.223109345	0.43234098	2.82903867	0.00466881	0.01638565
ENSDARG00000061292	pi15b	19.5544884	-1.630650373	0.57642419	-2.82890689	0.00467073	0.01638777
ENSDARG00000104959	dnajc17	173.842781	-0.565219972	0.19980096	-2.82891519	0.00467061	0.01638777
ENSDARG00000079727	selenop2	1157.17874	0.425251004	0.15034058	2.82858428	0.00467544	0.01640198
ENSDARG00000114572	asb18	94.9019852	-0.7985958	0.28239239	-2.82796502	0.00468449	0.01643142
ENSDARG00000026925	nos2a	9.17913948	2.400882504	0.84901794	2.82783483	0.0046864	0.01643579
ENSDARG00000104791	CABZ01077956.1	25.7431398	1.592879364	0.56329609	2.82778348	0.00468715	0.0164361
ENSDARG00000086712	si:dkeyp-97b10.3	292.667842	-0.484802444	0.17146378	-2.82743362	0.00469227	0.01645175
ENSDARG00000100129	brd3b	3945.67012	-0.332962465	0.11776529	-2.82733958	0.00469365	0.01645426
ENSDARG00000070553	rnmt	1189.18031	-0.388632728	0.1374585	-2.82727309	0.00469463	0.01645536
ENSDARG00000077004	aldh1l1	4753.68994	0.355505027	0.12580754	2.82578479	0.00471649	0.01652968
ENSDARG00000024920	tlcd5a	636.634035	-0.455707584	0.16127719	-2.82561701	0.00471897	0.016536
ENSDARG00000058819	nog1	276.622924	-0.507990555	0.17979062	-2.82545642	0.00472133	0.01654196
ENSDARG00000076970	si:ch211-165b10.3	41.7926287	-0.988589483	0.3499318	-2.82509191	0.00472671	0.01655846
ENSDARG00000034240	capza1a	5469.89433	0.231425603	0.08192158	2.82496506	0.00472858	0.01656252
ENSDARG00000085246	AL732488.1	11.4458466	-2.218734941	0.78543941	-2.8248327	0.00473053	0.01656252
ENSDARG00000099500	clec3bb	54.2695757	0.915358795	0.32403117	2.82490971	0.00472939	0.01656252
ENSDARG00000075545	si:dkey-89b17.4	5537.347	-0.339146251	0.12005817	-2.82484949	0.00473028	0.01656252
ENSDARG00000059963	polk	214.806398	-0.579089578	0.20501166	-2.82466652	0.00473299	0.01656878
ENSDARG00000074908	col6a1	15300.111	0.302288914	0.10703823	2.82412113	0.00474105	0.01659466
ENSDARG00000003020	lrrc4.2	415.734651	-0.501387773	0.17755389	-2.82386251	0.00474487	0.01660572
ENSDARG00000060849	dlgap4a	895.592392	-0.364863829	0.12921649	-2.82366301	0.00474783	0.01661372
ENSDARG00000028213	ttn.2	38984.6964	0.595995264	0.21107877	2.82356807	0.00474923	0.0166163
ENSDARG00000007480	rpe65a	1895.16285	0.935093259	0.33127025	2.82275053	0.00476136	0.01665639
ENSDARG00000102937	hspbp1	479.962987	0.42526958	0.15066146	2.82268324	0.00476236	0.01665754
ENSDARG00000013667	tbck	888.856856	0.395795581	0.14024756	2.82212101	0.00477072	0.01668442
ENSDARG00000012222	nup35	632.149099	-0.343271844	0.12164533	-2.82190741	0.0047739	0.01669319
ENSDARG00000077938	cd248b	1510.30173	0.383454704	0.13590359	2.82152011	0.00477966	0.01671101
ENSDARG00000058820	bin1b	922.302692	0.334269497	0.11848464	2.82120541	0.00478436	0.01672506
ENSDARG00000053744	dcdc2b	133.46696	0.773586868	0.2742258	2.82098501	0.00478764	0.0167342
ENSDARG00000022845	lias	593.003884	0.409624845	0.14521335	2.82084846	0.00478968	0.01673897
ENSDARG00000002507	itga10	1009.58517	0.298598201	0.10586873	2.8204571	0.00479553	0.01675705
ENSDARG00000015290	sacm1la	1009.91241	0.286427332	0.10155952	2.82029032	0.00479802	0.0167634
ENSDARG00000115832	LO018490.1	175.320395	0.609904575	0.21626725	2.82014308	0.00480022	0.01676874
ENSDARG00000054827	ssuh2.4	86.9251651	0.930047181	0.32982539	2.8198168	0.00480511	0.01678344
ENSDARG00000052215	btr02	266.554742	0.500956664	0.17767862	2.81945387	0.00481055	0.01680007
ENSDARG00000015126	gorasp2	3018.37633	0.295960209	0.10497599	2.81931323	0.00481265	0.01680507
ENSDARG00000092115	eif4a1a	13930.2601	0.357113487	0.12668288	2.81895628	0.00481801	0.01682141
ENSDARG00000068702	lysmd4	319.961849	0.524836575	0.18619427	2.81875797	0.00482099	0.01682944
ENSDARG00000060521	rbm27	708.573303	-0.397550735	0.14104252	-2.81865868	0.00482248	0.01683228
ENSDARG00000030759	melk	391.577268	-0.467553892	0.16588215	-2.81859075	0.0048235	0.01683348
ENSDARG00000012450	vmp1	1341.31691	0.363202679	0.12888312	2.81807807	0.00483121	0.01685801
ENSDARG00000069262	erich3	112.496095	0.626063894	0.22216426	2.8180225	0.00483204	0.01685856
ENSDARG00000023152	wdr73	187.893247	-0.526022431	0.18667626	-2.81783255	0.0048349	0.01686617
ENSDARG00000107086	PIEZO2	151.920877	0.75386655	0.26761479	2.81698393	0.0048477	0.01690843
ENSDARG00000034768	phf3	1353.44514	-0.30758403	0.10920295	-2.8166276	0.00485308	0.01692482
ENSDARG00000025788	chp2	218.997914	-0.705089108	0.25035963	-2.81630511	0.00485795	0.01693945
ENSDARG00000115603	cacna1db	76.183367	-0.90946788	0.32299901	-2.81569871	0.00486713	0.01696907
ENSDARG00000028715	mindy3	732.882692	-0.393979959	0.13993079	-2.8155345	0.00486962	0.01697536
ENSDARG00000002219	si:dkey-103j14.5	72.6863843	0.916793967	0.32568885	2.81493818	0.00487866	0.01700451
ENSDARG00000076592	man1b1b	599.460075	0.412079083	0.14643058	2.81415996	0.00489049	0.01704334
ENSDARG00000074149	itpr1b	1031.36977	0.370945882	0.13181635	2.81411136	0.00489123	0.01704353

Table S2. DEGs of WT vs. *terfa-/-*

ENSDARG00000086172	ACVR1C	45.7723235	-1.357503771	0.48259863	-2.8129043	0.00490963	0.01710524
ENSDARG00000105607	si:dkey-183c6.9	34.8369033	1.209173566	0.42990344	2.81266315	0.00491331	0.01711381
ENSDARG00000020134	sipa1l1	1837.43838	0.295770205	0.10515871	2.81260787	0.00491415	0.01711381
ENSDARG00000057630	galm	646.255946	0.445403426	0.15835728	2.8126489	0.00491353	0.01711381
ENSDARG00000076176	ptcd1	500.806632	-0.358955597	0.12764094	-2.81222938	0.00491994	0.01713157
ENSDARG00000077537	nudt19	342.794904	0.497786021	0.1770233	2.81198018	0.00492375	0.01714244
ENSDARG00000045159	zdhhc4	447.105203	0.380117173	0.13524673	2.81054618	0.00494575	0.01721661
ENSDARG00000067658	BX470189.1	275.080309	-0.483159618	0.17197354	-2.80949975	0.00496186	0.0172685
ENSDARG00000100342	zgc:158464	639.578918	0.356122735	0.12675783	2.80947326	0.00496226	0.0172685
ENSDARG00000077862	si:dkey-169i5.4	8.5276399	2.383301553	0.84831838	2.80944233	0.00496274	0.0172685
ENSDARG00000094154	hist2h3c	60.0794483	-1.149699241	0.40931949	-2.80880648	0.00497255	0.01730022
ENSDARG00000009942	cdc6	584.277427	-0.605814778	0.21569694	-2.80863874	0.00497514	0.01730682
ENSDARG00000115299	si:ch211-227e10.3	35.3777942	-1.538724527	0.54791234	-2.8083407	0.00497975	0.017318
ENSDARG00000026166	emilin1b	2626.54928	0.274296054	0.09767113	2.80836373	0.0049794	0.017318
ENSDARG00000021232	nkx2.7	42.165339	-1.066955085	0.37998449	-2.80789113	0.00498671	0.01733976
ENSDARG00000029356	mboat1	426.613023	0.446397249	0.15898682	2.80776261	0.0049887	0.01734426
ENSDARG00000108060	LO017771.1	475.601012	0.653768158	0.23287705	2.80735333	0.00499504	0.01736388
ENSDARG00000014098	pkd2	1087.66107	0.338778977	0.12068275	2.80718643	0.00499763	0.01737045
ENSDARG00000023916	phactr1	725.816348	-0.455460471	0.1622657	-2.80688083	0.00500237	0.01738451
ENSDARG00000031020	pip4k2ca	2130.21837	-0.276534476	0.09852285	-2.80680564	0.00500354	0.01738614
ENSDARG00000060152	fam155b	384.868397	-0.577452755	0.20576506	-2.80636936	0.00501032	0.01740552
ENSDARG00000100396	pip5k1aa	662.19407	-0.421385507	0.15015394	-2.80635666	0.00501052	0.01740552
ENSDARG00000088590	pxna	2014.81741	0.300042959	0.10692195	2.80618669	0.00501316	0.01741227
ENSDARG00000103718	chmp5b	1700.13539	0.339105402	0.1208484	2.80603978	0.00501545	0.01741777
ENSDARG00000042690	s1pr1	2470.69856	-0.311094366	0.11087039	-2.80592828	0.00501719	0.01742137
ENSDARG00000101745	AL953886.1	298.000337	-0.56037393	0.19972701	-2.80569931	0.00502075	0.01743132
ENSDARG00000078604	tbc1d10b	1938.65004	-0.291758969	0.10399356	-2.80554855	0.0050231	0.01743704
ENSDARG00000039455	tspan15	454.237765	0.497518422	0.17735083	2.80527822	0.00502732	0.01744923
ENSDARG00000041022	pdcd4b	1683.88593	-0.3671092	0.1308928	-2.80465552	0.00503704	0.01748053
ENSDARG00000095774	emd	607.490102	0.386038415	0.13767041	2.80407685	0.00504609	0.01750949
ENSDARG00000087421	serbp1b	8501.8169	-0.237713324	0.0847811	-2.80384801	0.00504967	0.01751948
ENSDARG00000054355	dcaf7	6088.21748	-0.336700968	0.1201009	-2.80348404	0.00505537	0.01753437
ENSDARG00000096903	CU062633.1	5.42121027	5.22480928	1.86365997	2.80352069	0.0050548	0.01753437
ENSDARG00000017490	cel.1	899.234143	-1.676714538	0.59809546	-2.80342293	0.00505633	0.01753525
ENSDARG00000056680	stc2a	557.584452	-0.360050038	0.12845132	-2.80300761	0.00506285	0.0175554
ENSDARG00000098295	efna3b	474.248567	-0.464922209	0.16589453	-2.80251687	0.00507056	0.01757967
ENSDARG00000071863	itgb1a	6896.87791	0.258293397	0.09217084	2.80233299	0.00507345	0.01758725
ENSDARG00000092277	BX663611.2	10.3853422	2.668225431	0.95219658	2.80217919	0.00507587	0.01759318
ENSDARG00000062550	rc3h1a	1215.38794	0.48812123	0.17420014	2.80207138	0.00507756	0.01759661
ENSDARG00000056105	syt11b	1158.14591	-0.369540989	0.13188495	-2.80199505	0.00507877	0.01759832
ENSDARG00000098176	si:rp71-1h20.5	137.19277	-0.675461659	0.24106878	-2.80194587	0.00507954	0.01759854
ENSDARG00000098230	BX649416.1	131.265961	-0.659340586	0.23539902	-2.80094872	0.00509526	0.01765055
ENSDARG00000053709	si:ch73-89b15.3	199.493815	-0.639895799	0.22848233	-2.80063577	0.00510021	0.01766226
ENSDARG00000063354	abtb1	951.191815	-0.341886406	0.12207614	-2.80059984	0.00510077	0.01766226
ENSDARG00000055108	gde1	593.731574	0.389102903	0.13893477	2.80061567	0.00510052	0.01766226
ENSDARG00000054968	cd40	95.0699127	0.880263987	0.31433293	2.80041928	0.00510363	0.01766968
ENSDARG00000088813	ppp1r3ab	487.124585	0.732113447	0.26146737	2.80001839	0.00510997	0.01768918
ENSDARG00000003486	ppp1caa	5734.71154	-0.329307835	0.11762248	-2.7997016	0.00511499	0.01770408
ENSDARG00000087679	BX005012.1	10.0520784	-2.389008701	0.85336005	-2.79953192	0.00511768	0.01771092
ENSDARG00000042188	ttc38	179.345062	0.678018078	0.24222419	2.79913445	0.00512398	0.01773027
ENSDARG00000007526	ndufs2	7411.76696	0.273813935	0.09782615	2.79898507	0.00512635	0.017736
ENSDARG00000003693	hars	3791.11169	0.321144166	0.11475416	2.79854053	0.00513341	0.01775796
ENSDARG00000099364	BX511034.5	66.0629828	1.495601326	0.53444283	2.79843089	0.00513516	0.01776152
ENSDARG00000022718	casp8ap2	707.249638	-0.321115558	0.11475992	-2.79815068	0.00513961	0.01777447
ENSDARG00000092979	pias2	1380.91168	-0.316531786	0.11313128	-2.79791568	0.00514335	0.01778493
ENSDARG00000037895	ramp2	540.741643	0.583694479	0.20863765	2.79764688	0.00514764	0.01779726
ENSDARG00000020301	os9	1587.27777	0.288322515	0.10306184	2.79756809	0.00514889	0.01779912
ENSDARG00000101306	mrpl15	1112.76165	0.330858756	0.11828868	2.79704498	0.00515724	0.01782549

Table S2. DEGs of WT vs. *terfa-/-*

ENSDARG00000071868	znf711	847.112377	-0.325364992	0.11633922	-2.79669228	0.00516287	0.01784248
ENSDARG00000096497	si:dkey-22h13.3	50.364354	-0.882580383	0.31563386	-2.7962158	0.00517049	0.01786567
ENSDARG00000019459	elf2a	1142.70852	-0.299085809	0.10696219	-2.79618259	0.00517102	0.01786567
ENSDARG00000103197	si:ch211-227e10.1	9.42605203	2.499395552	0.8939108	2.79602343	0.00517357	0.01787199
ENSDARG00000045265	gsg1l2b	227.380345	-0.490390912	0.17542048	-2.79551692	0.00518168	0.01789753
ENSDARG00000098135	stk40	1150.87542	0.349263814	0.12495833	2.7950422	0.00518929	0.01792134
ENSDARG00000099624	chmp1b	2591.53356	0.303570947	0.10861821	2.7948439	0.00519248	0.01792985
ENSDARG00000016855	sf3b5	1091.42087	0.400287197	0.14333271	2.79271361	0.00522679	0.01804584
ENSDARG00000061956	sytl2a	194.042484	0.544453481	0.19497265	2.79246081	0.00523088	0.01805744
ENSDARG00000089529	desi1b	294.079052	0.530105835	0.18986006	2.79208716	0.00523693	0.01807579
ENSDARG00000098105	tmem106c	659.798452	-0.363404728	0.13018967	-2.79134832	0.0052489	0.01811459
ENSDARG00000077533	eif3f	8304.32529	0.330710131	0.11848956	2.79104872	0.00525376	0.01812885
ENSDARG00000011257	enpp2	480.841611	-0.464921188	0.16661946	-2.79031743	0.00526564	0.01816733
ENSDARG00000015854	chata	1131.80387	-0.378014152	0.13549701	-2.78983394	0.00527351	0.01819196
ENSDARG00000053828	ubxn11	34.8186222	1.240705376	0.44474543	2.78969789	0.00527573	0.01819203
ENSDARG00000041483	paqr6	1051.1423	0.343819714	0.12324552	2.78971363	0.00527547	0.01819203
ENSDARG00000061240	cbln4	74.4165807	0.981098609	0.35167879	2.78975769	0.00527475	0.01819203
ENSDARG00000012577	waca	2908.89913	-0.29306701	0.10505932	-2.78953839	0.00527832	0.01819847
ENSDARG00000040396	bcl7bb	392.465763	-0.433783016	0.15553605	-2.78895486	0.00528784	0.01822623
ENSDARG00000004577	zgc:194665	549.27236	-0.503909001	0.18067769	-2.78899407	0.0052872	0.01822623
ENSDARG00000030161	ppp1r14bb	1178.87449	-0.371402907	0.13318777	-2.78856623	0.00529419	0.01824558
ENSDARG00000071021	papss2a	1731.38153	0.53696254	0.19256898	2.7884166	0.00529664	0.0182511
ENSDARG00000057272	slc30a9	1699.79333	-0.274490619	0.09844095	-2.78837847	0.00529726	0.0182511
ENSDARG00000059498	arl16	259.491351	-0.467395876	0.1676312	-2.78823916	0.00529954	0.01825642
ENSDARG00000018693	cdh2	12071.3969	0.310527664	0.11137615	2.78809829	0.00530185	0.01826183
ENSDARG00000054754	hs6st1a	470.508972	-0.419553423	0.15049057	-2.787905	0.00530501	0.0182702
ENSDARG00000057054	lrit3b	15.0076548	-2.139363374	0.7675065	-2.78742053	0.00531295	0.018295
ENSDARG00000063914	mt-nd3	6158.02097	0.320426913	0.11497521	2.78692171	0.00532113	0.01832065
ENSDARG00000086523	ch25hl3	463.492871	-0.541047801	0.19414897	-2.78676629	0.00532369	0.0183269
ENSDARG00000054186	ncs1b	117.102723	-0.63769972	0.22885976	-2.78642133	0.00532936	0.01834388
ENSDARG00000117690	CR339059.4	21.1197089	1.650660852	0.59241688	2.78631636	0.00533108	0.01834728
ENSDARG00000078992	wnk1a	6086.36095	-0.335791078	0.12051862	-2.78621739	0.00533271	0.01835034
ENSDARG00000091085	lepa	10.506453	2.772180178	0.99499397	2.78612763	0.00533419	0.01835288
ENSDARG00000013704	cers2a	325.745386	0.541323939	0.19431	2.7858779	0.0053383	0.01836448
ENSDARG00000097635	wu:fb18f06	936.959979	0.567027143	0.20354773	2.78572083	0.00534089	0.01837084
ENSDARG00000069563	zgc:153151	36.9992371	1.199272528	0.43053752	2.78552383	0.00534413	0.01837946
ENSDARG00000024465	mrps18a	956.16854	0.34210493	0.12282718	2.78525441	0.00534857	0.0183922
ENSDARG00000077040	RIN2	189.66248	0.686687327	0.24658563	2.78478248	0.00535636	0.01841644
ENSDARG00000057209	plppr5a	172.815847	-0.78924887	0.28342885	-2.78464546	0.00535863	0.01842167
ENSDARG00000018146	gpx1a	1079.92419	0.392209415	0.14092118	2.78318288	0.00538285	0.01850237
ENSDARG00000041215	cetn4	326.20923	-0.501343375	0.18015336	-2.78286994	0.00538804	0.01851766
ENSDARG00000109591	CR855375.5	14.3887411	-2.17141337	0.78037551	-2.78252374	0.00539379	0.01853487
ENSDARG00000098045	CR751602.2	68.2446303	-0.772721598	0.277768	-2.7818957	0.00540424	0.01856564
ENSDARG00000059323	rpn1	8233.71156	0.30269456	0.10880829	2.78190707	0.00540405	0.01856564
ENSDARG00000102061	b3galt2	2092.94116	-0.42584528	0.15308647	-2.78173034	0.005407	0.01857253
ENSDARG00000006892	myo3b	265.655823	-0.740531857	0.26625732	-2.7812638	0.00541477	0.01859411
ENSDARG00000095455	si:dkey-20i20.10	80.6089191	-0.846863871	0.30448512	-2.78129808	0.0054142	0.01859411
ENSDARG00000058461	ice1	585.696023	-0.363078745	0.13056753	-2.78077354	0.00542296	0.01861449
ENSDARG00000095911	si:ch211-149b19.4	64.8955374	-1.422483433	0.5115291	-2.78084556	0.00542175	0.01861449
ENSDARG00000112606	NA	3.01345949	-5.546687439	1.9946381	-2.78079891	0.00542253	0.01861449
ENSDARG00000061377	efcab1	38.5140838	1.062485397	0.38209641	2.78067357	0.00542463	0.01861766
ENSDARG00000030463	tppp3	375.295528	0.747581646	0.26888251	2.78032828	0.0054304	0.01863465
ENSDARG00000020926	creb3l3l	3190.52165	0.250483012	0.09009248	2.78028764	0.00543108	0.01863465
ENSDARG00000013295	slc2a3a	2116.36659	0.325934876	0.11724072	2.78004848	0.00543508	0.01864581
ENSDARG00000037598	prf1.5	111.562673	-0.686321099	0.2468856	-2.77991553	0.0054373	0.01865087
ENSDARG00000021370	actr6	585.975003	-0.394561613	0.14194793	-2.77962224	0.00544222	0.01866514
ENSDARG00000029043	nup42	303.900894	-0.505944135	0.18202998	-2.77945498	0.00544502	0.01867218
ENSDARG00000116971	CU861448.1	102.554301	-0.74764059	0.26899956	-2.77933759	0.00544699	0.01867636

Table S2. DEGs of WT vs. *terfa-/-*

ENSDARG00000092507	znf1013	157.254499	-0.549312059	0.19764654	-2.77926473	0.00544821	0.01867797
ENSDARG00000098317	dync1li1	3095.18182	-0.263558463	0.09483432	-2.77914644	0.0054502	0.0186822
ENSDARG00000009470	hnf1a	215.868918	-0.656734363	0.23635766	-2.77856181	0.00546001	0.01871327
ENSDARG00000088246	thoc1	1542.81091	-0.359880788	0.12953038	-2.77835044	0.00546357	0.01872028
ENSDARG00000115455	CR352285.2	111.314102	-0.636707061	0.22916677	-2.77835687	0.00546346	0.01872028
ENSDARG00000060184	pkn1a	1740.97022	0.293818209	0.10578109	2.7776061	0.0054761	0.01876063
ENSDARG00000113137	BX276107.1	53.5397257	0.87451171	0.31488274	2.77726154	0.00548191	0.01877794
ENSDARG00000074222	rap1gap2b	135.462691	-0.741692929	0.26706958	-2.77715244	0.00548375	0.01878166
ENSDARG00000113391	si:dkey-265e15.2	5.0303015	3.038777116	1.09422927	2.77709361	0.00548474	0.01878247
ENSDARG00000093469	znf653	242.020892	-0.555354161	0.19999991	-2.77677205	0.00549017	0.01879847
ENSDARG00000033126	prkrip1	252.301531	-0.579903867	0.20885989	-2.77652103	0.00549441	0.0188104
ENSDARG00000004455	p2rx5	271.557506	0.584990974	0.21070685	2.77632628	0.0054977	0.01881908
ENSDARG00000069559	muc13a	1008.90696	-0.50896071	0.18333122	-2.77618134	0.00550015	0.01882488
ENSDARG00000077404	ncoa3	3136.92824	0.321793055	0.1159378	2.77556631	0.00551057	0.01885793
ENSDARG00000087839	si:dkey-33c14.6	94.6226939	0.690540561	0.24884702	2.77496019	0.00552085	0.01889051
ENSDARG00000078523	ddr1	2725.78566	0.312651049	0.1126791	2.77470316	0.00552521	0.01890284
ENSDARG00000097244	zgc:174263	749.206756	-0.310902354	0.11206481	-2.77430846	0.00553192	0.01892319
ENSDARG00000075435	akap11	1789.70844	-0.318082848	0.11466329	-2.77406009	0.00553615	0.01893503
ENSDARG00000008209	myt1la	2566.65682	-0.486482133	0.17537488	-2.77395564	0.00553792	0.01893851
ENSDARG00000055837	arhgef1b	2875.69367	0.285564828	0.10298104	2.7729844	0.00555448	0.01899251
ENSDARG00000008790	actr3b	89.4251735	-0.71887285	0.25930938	-2.77225935	0.00556687	0.01903225
ENSDARG00000073843	myo9ab	2701.85593	-0.431972043	0.15582367	-2.77218498	0.00556814	0.01903398
ENSDARG00000074662	cntf	92.4169452	0.70050858	0.25270163	2.77207777	0.00556997	0.01903763
ENSDARG00000069264	si:ch211-196h16.5	156.350621	-0.536933776	0.19369829	-2.7720109	0.00557112	0.01903893
ENSDARG00000024694	myo1b	3692.5819	-0.274824201	0.09915151	-2.77176024	0.00557541	0.01905097
ENSDARG00000025671	grm6b	711.569904	-0.682112587	0.24610077	-2.77168008	0.00557678	0.01905305
ENSDARG00000026165	col11a1a	65757.2587	0.279118442	0.10072498	2.77109453	0.00558682	0.01908472
ENSDARG00000095777	si:ch211-218h8.3	6.33252027	3.884449803	1.40204641	2.77055721	0.00559605	0.01911098
ENSDARG00000097901	si:ch73-90p23.1	10.0974609	2.090389867	0.75449949	2.77056498	0.00559591	0.01911098
ENSDARG00000054414	spata17	23.3017334	1.519921068	0.54862262	2.77043091	0.00559822	0.01911577
ENSDARG00000062268	jarid2b	184.596843	-0.556154319	0.20078063	-2.76996	0.00560632	0.0191408
ENSDARG00000026294	erbb2	1195.80758	0.426452046	0.15396092	2.76987206	0.00560783	0.01914334
ENSDARG00000014976	lims2	1422.66317	0.453244941	0.16365003	2.76959882	0.00561254	0.01915677
ENSDARG00000091734	traf3ip2l	441.244333	0.532859086	0.19242975	2.76910965	0.00562097	0.01918293
ENSDARG00000104651	msx2a	57.2124432	0.928853634	0.33544296	2.76903601	0.00562224	0.01918463
ENSDARG00000103734	FP085414.2	7.47177581	-3.129918475	1.13060079	-2.7683675	0.00563379	0.01922139
ENSDARG00000044437	cdca5	445.129857	-0.548048676	0.19801279	-2.76774385	0.00564458	0.01925556
ENSDARG00000097273	CR848759.1	4.59470829	-5.535895884	2.0001869	-2.7676893	0.00564553	0.01925614
ENSDARG00000067981	C5HXorf56	538.45248	-0.444933739	0.16081427	-2.76675527	0.00566172	0.01930842
ENSDARG00000017590	nit2	561.725186	-0.33813763	0.12221624	-2.76671604	0.00566241	0.01930842
ENSDARG00000090730	cfbl	63.1156072	0.845753558	0.30574912	2.76616841	0.00567192	0.01933823
ENSDARG00000087144	fam183a	24.0042043	1.604720648	0.58015815	2.76600551	0.00567476	0.01934523
ENSDARG00000023900	casd1	863.830005	-0.31418176	0.1136037	-2.76559451	0.00568191	0.01936697
ENSDARG00000089875	zgc:173705	37.3771882	-1.39448466	0.50425346	-2.76544393	0.00568454	0.01937326
ENSDARG00000058460	cacng3a	33.011721	-1.519657144	0.54954845	-2.76528329	0.00568734	0.01938015
ENSDARG00000114086	DOCK9	2563.77754	0.305617489	0.11057821	2.76381308	0.00571303	0.01946349
ENSDARG00000061631	fbxo11a	2329.98904	-0.347499296	0.12573271	-2.76379386	0.00571336	0.01946349
ENSDARG00000103148	nectin4b	290.033341	0.409442986	0.14817184	2.76329818	0.00572205	0.0194904
ENSDARG00000031683	fosab	594.732259	-0.664291285	0.24047575	-2.76240451	0.00573773	0.01954116
ENSDARG00000056840	otud5b	1597.91884	-0.300668672	0.10884877	-2.76226053	0.00574027	0.0195471
ENSDARG00000102867	srfb	387.95436	0.393382372	0.14241576	2.76221103	0.00574114	0.01954739
ENSDARG00000103364	cs	14242.1016	0.199578018	0.07225647	2.76207813	0.00574347	0.01955267
ENSDARG00000117647	LO017700.2	47.153844	-1.401266019	0.50734106	-2.76198032	0.00574519	0.01955585
ENSDARG00000025309	dpf3	708.971896	-0.510377999	0.18480998	-2.76163663	0.00575125	0.01957376
ENSDARG00000103146	nlrc3	28.5772511	1.299664047	0.47066705	2.76132363	0.00575676	0.01958985
ENSDARG00000026723	syncripl	2802.64842	-0.413461123	0.1497475	-2.76105536	0.00576149	0.01960327
ENSDARG00000056531	nutf2	2479.93668	0.268451635	0.09724429	2.76059016	0.0057697	0.01962852
ENSDARG00000102091	CABZ01052576.1	659.081567	0.582328008	0.21095136	2.76048467	0.00577157	0.01963217

Table S2. DEGs of WT vs. *terfa-/-*

ENSDARG00000098800	znf1156	323.890944	0.653954419	0.23695197	2.75986062	0.0057826	0.01966702
ENSDARG00000078072	si:ch211-232i5.1	512.523094	0.449199688	0.16277978	2.75955456	0.00578802	0.01968276
ENSDARG00000053475	ngb	518.650891	-0.478039602	0.17324448	-2.75933526	0.00579191	0.01969328
ENSDARG00000060788	lrrc14b	142.656985	0.634634628	0.23001427	2.7591098	0.00579591	0.01970418
ENSDARG00000007744	tsr1	2293.90623	0.30726301	0.11136882	2.75896794	0.00579842	0.01971004
ENSDARG00000076848	zgc:112492	109.564119	-0.769461753	0.2789065	-2.75885196	0.00580048	0.01971434
ENSDARG00000055206	dtnbp1b	231.189658	-0.660927902	0.23961123	-2.75833439	0.00580967	0.01974288
ENSDARG00000013505	ube2kb	1433.86949	-0.319818175	0.11595624	-2.75809367	0.00581395	0.01975473
ENSDARG00000032155	ppm1aa	1707.90695	0.364253132	0.13210256	2.75735109	0.00582718	0.01979522
ENSDARG00000078113	atp5mea	988.544376	-0.395971418	0.14360657	-2.75733489	0.00582746	0.01979522
ENSDARG00000032039	mxd1	626.467003	-0.512919936	0.18605627	-2.75680004	0.005837	0.01982491
ENSDARG00000117739	FQ312017.2	2.8829208	4.781687235	1.73465547	2.75656309	0.00584123	0.01983657
ENSDARG00000006219	gnl3	1921.14395	-0.32793036	0.11902665	-2.75510033	0.00586741	0.01992276
ENSDARG00000045175	pofut2	348.622532	0.40981782	0.14875622	2.7549626	0.00586989	0.01992842
ENSDARG00000117716	BX005389.5	22.4314683	-1.567566402	0.56905832	-2.75466738	0.00587518	0.01994369
ENSDARG00000103095	tbk1	2768.87485	0.26573325	0.09647237	2.75450122	0.00587817	0.01995109
ENSDARG00000044253	paqr3b	588.606809	0.319590295	0.11602695	2.75444885	0.00587911	0.01995156
ENSDARG00000052783	cdc42ep3	931.553841	-0.409853402	0.14879958	-2.75439891	0.00588001	0.01995188
ENSDARG00000012389	adar	2234.0057	-0.282101499	0.10242309	-2.75427644	0.00588221	0.01995663
ENSDARG00000094414	aknad1	38.5050525	-1.13951776	0.41376077	-2.75404982	0.00588628	0.01996772
ENSDARG00000071551	xrcc6	981.975552	-0.305674023	0.11100822	-2.75361615	0.00589409	0.01999147
ENSDARG00000045885	scaf11	3144.04955	0.232416678	0.08442618	2.75289831	0.00590702	0.02003262
ENSDARG00000103762	rln1	16.8194509	1.848734275	0.67165211	2.75251762	0.0059139	0.02005319
ENSDARG00000071036	si:dkey-286j15.3	143.819172	0.749304551	0.27223342	2.75243414	0.0059154	0.02005557
ENSDARG00000100053	CABZ01044099.1	368.627367	0.473354934	0.17198511	2.75230183	0.0059178	0.02006094
ENSDARG00000011510	rcc2	5537.39395	-0.371403646	0.13495321	-2.75209194	0.00592159	0.02007106
ENSDARG00000036445	serpinh2	739.867078	0.354836551	0.12899661	2.75074328	0.00594602	0.02015113
ENSDARG00000100072	mgrn1a	1021.10394	-0.319670374	0.11622697	-2.75039765	0.0059523	0.02016965
ENSDARG00000013533	hoxb4a	619.060753	-0.321460417	0.11689092	-2.75008896	0.00595791	0.02018591
ENSDARG00000005368	mcamb	2105.57456	0.319559291	0.11621517	2.7497211	0.0059646	0.02020583
ENSDARG00000040556	ift20	342.653213	-0.463326263	0.16851729	-2.74942865	0.00596993	0.02022111
ENSDARG00000013933	XRCC2	32.8589832	1.107641412	0.40287667	2.74933124	0.0059717	0.02022437
ENSDARG00000054103	ripply1	100.310782	-0.718715055	0.26142302	-2.74924167	0.00597333	0.02022714
ENSDARG00000060477	vps8	1224.60546	0.282266477	0.10267573	2.7491062	0.0059758	0.0202326
ENSDARG00000078415	lrrc3	23.1249819	-1.663283394	0.60503628	-2.74906392	0.00597657	0.0202326
ENSDARG00000004785	cops2	1848.12017	-0.296859026	0.10800393	-2.74859467	0.00598514	0.02025882
ENSDARG00000004470	prkcsh	3772.0818	0.302213702	0.10997182	2.74810123	0.00599415	0.02028657
ENSDARG00000069912	hmga2	2986.64572	0.367117267	0.13367232	2.74639708	0.00602538	0.02038949
ENSDARG00000005049	rab20	213.486541	0.558133095	0.20323363	2.7462635	0.00602783	0.02039327
ENSDARG00000063293	neto2b	158.979733	-0.919100086	0.33467497	-2.74624688	0.00602814	0.02039327
ENSDARG00000016889	eif3g	4682.91709	-0.285672075	0.10402495	-2.74618802	0.00602922	0.02039416
ENSDARG00000103956	si:dkey-17o15.2	911.516162	0.353101931	0.12858144	2.7461346	0.0060302	0.0203947
ENSDARG00000116300	zgc:165515	133.985566	-0.637995493	0.23237997	-2.74548408	0.00604217	0.0204324
ENSDARG00000077180	slc37a4b	1957.06332	0.353998449	0.12894322	2.74538253	0.00604404	0.02043594
ENSDARG00000078455	best4	17.8690425	-2.312357287	0.84238964	-2.74499731	0.00605114	0.02045716
ENSDARG00000019743	dctn1a	1540.55834	0.399390563	0.14550136	2.74492666	0.00605244	0.02045879
ENSDARG00000096566	zgc:165656	168.099202	0.56627102	0.20631012	2.74475644	0.00605558	0.02046662
ENSDARG00000068710	nid1a	5291.01054	-0.297073126	0.10823601	-2.74467932	0.00605701	0.02046864
ENSDARG00000017615	tlx3b	144.700868	-0.602438399	0.21950164	-2.74457356	0.00605896	0.02047246
ENSDARG00000025478	gipr	94.6658759	-0.837032434	0.30498669	-2.74448837	0.00606053	0.02047499
ENSDARG00000014945	znf598	1656.63878	0.224884588	0.081947	2.74426865	0.00606459	0.02048591
ENSDARG00000078227	cspg4	2481.72731	0.479746531	0.17482672	2.74412592	0.00606723	0.02049204
ENSDARG00000012627	cdc34b	1593.83287	-0.332457865	0.12116122	-2.74392967	0.00607085	0.02050151
ENSDARG00000057062	si:dkey-157g16.6	1334.89076	0.312145994	0.11377007	2.74365652	0.00607591	0.02051578
ENSDARG00000007181	nadk2	568.897468	0.348862089	0.12715909	2.74350893	0.00607864	0.02052222
ENSDARG00000030139	sdhdb	2640.66698	0.279764477	0.10199807	2.7428408	0.00609102	0.02056123
ENSDARG00000104042	b4galt4	1052.32558	0.392122083	0.14296689	2.74274752	0.00609275	0.02056428
ENSDARG00000092533	si:ch211-133l5.4	10.945673	-2.574852484	0.93897409	-2.74219759	0.00610296	0.0205938

Table S2. DEGs of WT vs. *terfa-/-*

ENSDARG00000058585	chst2b	344.977894	-0.476430983	0.17374123	-2.7421872	0.00610316	0.0205938
ENSDARG00000109769	LO017835.1	1812.69689	-0.451063923	0.16449344	-2.74213932	0.00610405	0.020594
ENSDARG00000019856	atp1a1b	10061.0779	-0.344632948	0.12570426	-2.74161705	0.00611376	0.02062397
ENSDARG00000079252	fam117ab	44.1168466	-0.999572963	0.36462445	-2.74137668	0.00611823	0.02063476
ENSDARG00000100186	CU655842.1	380.16283	-0.430024606	0.15686565	-2.74135609	0.00611862	0.02063476
ENSDARG00000097826	si:dkey-239b22.2	1784.7565	0.266068892	0.09706672	2.74109287	0.00612352	0.0206485
ENSDARG00000079501	si:ch211-149l1.2	178.704563	0.623507178	0.22748276	2.74089857	0.00612714	0.02065791
ENSDARG00000091970	ms4a17a.3	12.4309473	1.709117447	0.62364137	2.74054534	0.00613373	0.02067732
ENSDARG00000020239	lpin1	1701.87204	0.578863702	0.21125841	2.74007419	0.00614253	0.02070418
ENSDARG00000051953	ythdc1	2959.6239	-0.220260473	0.08039066	-2.73987643	0.00614623	0.02071383
ENSDARG00000028943	mtss1la	1660.92026	-0.397745739	0.14517783	-2.73971399	0.00614927	0.02072126
ENSDARG00000032650	fuk	591.844179	0.494647866	0.18057199	2.73933878	0.00615629	0.02074212
ENSDARG00000101624	lmnb2	7319.93388	-0.397345581	0.14505625	-2.73925173	0.00615792	0.0207448
ENSDARG00000008191	tmeff1a	191.784127	-0.59432604	0.21698629	-2.7390027	0.00616259	0.02075489
ENSDARG00000016531	klhl4	429.151025	-0.492971587	0.17997957	-2.73904186	0.00616185	0.02075489
ENSDARG00000034173	prkcq	233.348981	-0.530165351	0.19356619	-2.73893572	0.00616384	0.02075631
ENSDARG00000011301	nae1	1401.37421	-0.293245774	0.1070753	-2.73868742	0.0061685	0.02076917
ENSDARG00000095793	si:dkey-85n7.8	24.684939	-1.406030971	0.51347837	-2.73824774	0.00617675	0.02079415
ENSDARG00000096898	CR925728.1	11.8146513	2.688773792	0.98199063	2.73808498	0.00617981	0.02080162
ENSDARG00000030905	cited2	860.048054	0.315577099	0.11526625	2.73781001	0.00618498	0.0208162
ENSDARG00000061864	hdlbpb	1766.40911	-0.341021569	0.1245638	-2.73772607	0.00618656	0.0208187
ENSDARG00000103878	anpepb	604.300414	1.669227926	0.60978206	2.73741726	0.00619237	0.02083543
ENSDARG00000042995	sptlc1	968.760583	0.279829971	0.1022311	2.7372294	0.00619591	0.02084451
ENSDARG00000043928	gpn1	492.527555	-0.365623124	0.13362236	-2.73624213	0.00621453	0.02090433
ENSDARG00000056191	si:ch211-168k14.2	59.1965713	-0.788326932	0.28813304	-2.73598239	0.00621944	0.02091801
ENSDARG00000091059	onecut3b	84.7532117	0.908879932	0.33220706	2.73588383	0.0062213	0.02092144
ENSDARG00000073893	dnase2	317.257445	0.556581934	0.20346405	2.73552958	0.006228	0.02094114
ENSDARG00000097421	si:dkeyp-50b9.1	11.8723025	1.942146499	0.71003781	2.73527193	0.00623288	0.02095471
ENSDARG00000090371	si:dkey-46i9.6	343.209	0.409643788	0.14976913	2.73516844	0.00623484	0.02095563
ENSDARG00000012953	mob1bb	663.973578	0.383606621	0.14024802	2.73520164	0.00623421	0.02095563
ENSDARG00000102946	si:ch73-167f10.1	20.8555663	1.834107795	0.67069492	2.73463796	0.00624489	0.02098618
ENSDARG00000093425	BX539332.1	15.1795197	1.720052918	0.6289962	2.73459985	0.00624562	0.02098618
ENSDARG00000095696	trpm2	124.332476	-0.725339049	0.26529048	-2.73413145	0.00625451	0.02101322
ENSDARG00000055009	col4a1	4517.23241	0.355301511	0.12997536	2.73360674	0.00626448	0.02104388
ENSDARG00000103278	CR812832.1	173.436295	-0.599473111	0.21930666	-2.73349246	0.00626666	0.02104834
ENSDARG00000100850	rnf103	738.364241	-0.338491914	0.1238607	-2.73284347	0.00627902	0.02108701
ENSDARG00000036575	si:ch73-71d17.2	306.62728	-0.415818527	0.15217396	-2.73252091	0.00628517	0.02110433
ENSDARG00000098855	oxsm	412.475638	0.40170377	0.14701048	2.73248397	0.00628587	0.02110433
ENSDARG00000099733	CIR1	615.299109	-0.363830838	0.13315853	-2.73231334	0.00628913	0.02111242
ENSDARG00000063321	rabl2	532.023258	-0.404887454	0.14819291	-2.73216479	0.00629197	0.02111908
ENSDARG00000041723	TUBB4B	2433.59846	0.36997765	0.13544923	2.73148589	0.00630494	0.02115623
ENSDARG00000034577	si:dkey-23o4.6	29.5524969	-1.668843248	0.61097286	-2.73145235	0.00630559	0.02115623
ENSDARG00000057117	ghsrb	13.6745417	1.712647944	0.62700112	2.73149104	0.00630485	0.02115623
ENSDARG00000086317	si:ch211-67e16.4	854.356282	-0.336867664	0.12334692	-2.73105868	0.00631312	0.02117509
ENSDARG00000089402	si:ch73-281i18.3	39.9456683	-1.305241058	0.47793267	-2.73101449	0.00631397	0.02117509
ENSDARG00000060025	nsmfa	232.92615	-0.693701565	0.25400359	-2.73106991	0.00631291	0.02117509
ENSDARG00000078335	amot	1795.57018	0.37973917	0.13904864	2.73098074	0.00631462	0.02117509
ENSDARG00000092452	si:dkey-95p16.1	169.177261	0.95963644	0.35143752	2.73060325	0.00632185	0.02119364
ENSDARG00000029501	gsk3aa	756.532015	-0.486810356	0.17827859	-2.73061585	0.00632161	0.02119364
ENSDARG00000063594	hipk1a	309.436404	0.877523912	0.32137682	2.73051405	0.00632356	0.02119652
ENSDARG00000020893	slc25a55a	8322.86088	-0.276639286	0.10132125	-2.73031859	0.00632732	0.02120233
ENSDARG00000039666	tsta3	536.431965	0.405795835	0.14862736	2.7302903	0.00632786	0.02120233
ENSDARG00000003963	traf4a	1831.98224	0.281594659	0.10313668	2.73030557	0.00632756	0.02120233
ENSDARG00000116457	zmp:0000001088	33.3847731	-1.267978848	0.4644861	-2.72985315	0.00633625	0.02122761
ENSDARG00000031246	hbegfb	129.922993	0.608872144	0.22304665	2.72979734	0.00633733	0.02122834
ENSDARG00000060282	rnf121	1067.06481	0.396823521	0.14537506	2.72965334	0.0063401	0.02123475
ENSDARG00000061473	tbkbp1	520.373814	-0.455012712	0.16670117	-2.72951128	0.00634283	0.02124104
ENSDARG00000044968	vcla	1989.25322	0.33967132	0.12444658	2.72945493	0.00634391	0.02124181

Table S2. DEGs of WT vs. *terfa-/-*

ENSDARG00000086737	tmem68	489.890373	-0.367121465	0.13450688	-2.7293881	0.0063452	0.02124325
ENSDARG00000105487	CABZ01061349.1	10.5776385	-2.704529372	0.9909226	-2.72930436	0.00634681	0.02124579
ENSDARG00000086056	plekhg6	259.848619	0.472577586	0.17316843	2.72900543	0.00635257	0.02126219
ENSDARG00000095026	si:dkey-58f10.14	6.88638427	2.382190898	0.87302245	2.72867084	0.00635901	0.02128091
ENSDARG00000039041	sfrp5	527.057103	0.401944801	0.14732854	2.72822081	0.0063677	0.0213071
ENSDARG00000027079	znf804a	604.559958	-0.412666303	0.15126132	-2.72816801	0.00636872	0.02130764
ENSDARG00000014825	si:dkeyp-69b9.6	2630.36015	-0.325179671	0.11920428	-2.7279195	0.00637352	0.02132083
ENSDARG00000104815	lmx1ba	254.022435	0.477516951	0.17505696	2.72778047	0.0063762	0.02132407
ENSDARG00000102317	rpl26	16.3741759	-2.243725158	0.82253724	-2.72780984	0.00637564	0.02132407
ENSDARG00000015050	calm3a	18268.2958	-0.286934135	0.10522344	-2.72690322	0.00639318	0.02137797
ENSDARG00000045760	prmt8b	318.606458	-0.460777955	0.16898039	-2.7268132	0.00639492	0.02137805
ENSDARG00000079623	ubtd1b	277.728801	0.564508629	0.20702004	2.72683083	0.00639458	0.02137805
ENSDARG00000099814	FBXO46	1065.75728	0.335180765	0.12292746	2.72665499	0.00639799	0.02138543
ENSDARG00000103071	si:ch211-244c8.4	1419.16351	-0.483969264	0.17750529	-2.72650616	0.00640088	0.0213922
ENSDARG00000018328	rhocb	1645.50628	0.293047233	0.10749402	2.72617234	0.00640735	0.02141097
ENSDARG00000105116	p4hb	7207.25885	0.244094413	0.08954313	2.72599826	0.00641073	0.02141685
ENSDARG00000026499	ppm1e	790.779512	0.438608904	0.16089877	2.72599296	0.00641084	0.02141685
ENSDARG00000098420	SNTA1	554.5931	0.496769128	0.18229216	2.72512619	0.00642769	0.02147027
ENSDARG00000090629	tmtops3b	28.3905498	1.280200431	0.46985794	2.72465422	0.00643689	0.02149809
ENSDARG00000041589	adprhl1	145.985176	0.660507096	0.2424793	2.72397315	0.00645018	0.02153958
ENSDARG00000093619	sult3st2	66.1686398	0.971113874	0.35651874	2.72387893	0.00645202	0.02154283
ENSDARG00000020015	mrps33	446.741675	-0.396827025	0.1456926	-2.72372801	0.00645496	0.02154978
ENSDARG00000055653	nubp2	8.86464551	3.012697963	1.10621028	2.72344057	0.00646058	0.02156565
ENSDARG00000045493	slc35e3	433.852152	0.44311575	0.16271882	2.72319922	0.00646531	0.02157851
ENSDARG00000086048	si:ch211-229i14.2	7.76809763	3.121165903	1.14620724	2.72303803	0.00646846	0.02158614
ENSDARG00000037398	zgc:113229	123.481635	0.821505366	0.30176703	2.72231651	0.0064826	0.02163043
ENSDARG00000023185	si:rp71-1g18.13	141.363181	0.772975151	0.28399547	2.72178687	0.006493	0.02166026
ENSDARG00000103849	mdh1ab	3133.09274	0.459325466	0.16876065	2.72175688	0.00649359	0.02166026
ENSDARG00000055798	heyl	74.9568732	-0.988448449	0.36316943	-2.7217281	0.00649416	0.02166026
ENSDARG00000059483	tead1b	1847.63713	0.266825136	0.0980452	2.72145039	0.00649961	0.02167556
ENSDARG00000062947	amn	100.705632	0.796371594	0.29263898	2.72134489	0.00650169	0.02167957
ENSDARG00000090396	si:cabz01074946.1	20.5044521	1.358177815	0.49912319	2.72112745	0.00650597	0.02168801
ENSDARG00000109929	CABZ01058721.1	1206.97379	0.41114303	0.15109144	2.72115369	0.00650545	0.02168801
ENSDARG00000006900	impdh2	2400.25593	0.275487119	0.10124839	2.72090374	0.00651037	0.02169978
ENSDARG00000096856	znf1012	31.8862755	-1.622876256	0.5964594	-2.72084951	0.00651144	0.02170043
ENSDARG00000071662	si:rp71-36a1.3	41.0935339	-1.48392153	0.54540649	-2.7207625	0.00651315	0.02170324
ENSDARG00000076781	trim45	438.630439	0.373257709	0.1371949	2.72063836	0.0065156	0.02170848
ENSDARG00000018643	igf2a	655.332918	0.425417007	0.15639759	2.72009948	0.00652623	0.02174097
ENSDARG00000079742	mcf2l2	782.081709	-0.461011362	0.16948899	-2.72000774	0.00652804	0.02174409
ENSDARG00000071618	si:ch211-213a13.5	8.38787047	2.742539151	1.00843587	2.71959698	0.00653615	0.0217682
ENSDARG00000019294	cbln8	3.88218863	4.565915735	1.67896571	2.71948124	0.00653844	0.0217729
ENSDARG00000070800	zgc:109744	309.476443	-0.464485955	0.17081213	-2.71927963	0.00654243	0.02178326
ENSDARG00000024602	ACBD3	323.517183	0.45274248	0.16650367	2.71911419	0.0065457	0.02179124
ENSDARG00000096853	BX572077.1	18.5281302	1.629258117	0.59955598	2.7174412	0.00657888	0.02189878
ENSDARG00000059897	ntrk2a	1706.13372	0.376859865	0.13870845	2.7169207	0.00658924	0.02192444
ENSDARG00000015662	pla2g12b	7920.19694	0.268446627	0.09880419	2.71695592	0.00658854	0.02192444
ENSDARG00000079849	nrtn	56.1265421	-1.207821841	0.4445542	-2.71692822	0.00658909	0.02192444
ENSDARG00000098011	BX000438.2	850.097447	0.576166756	0.21207704	2.71678044	0.00659203	0.02193079
ENSDARG00000093496	si:zfos-905g2.1	201.713272	-0.596880647	0.2197625	-2.71602597	0.00660707	0.02197789
ENSDARG00000018967	gabbr1a	290.274045	-0.69955248	0.25758133	-2.71585087	0.00661057	0.02198658
ENSDARG00000074069	zgc:171452	25.5522638	1.836343766	0.67628226	2.71535107	0.00662056	0.02201583
ENSDARG00000056914	emg1	425.976461	-0.414395263	0.15261367	-2.71532206	0.00662114	0.02201583
ENSDARG00000087032	rbbp8l	310.125324	0.468074519	0.17241252	2.71485229	0.00663053	0.02204413
ENSDARG00000052982	gabrr2a	173.17662	-1.024263617	0.37729429	-2.71476048	0.00663237	0.02204729
ENSDARG00000020929	fam49ba	815.500297	0.373187679	0.13748196	2.71444843	0.00663862	0.02206512
ENSDARG00000078824	si:ch211-66e2.3	64.1025535	0.936712501	0.34522028	2.71337621	0.00666015	0.02213074
ENSDARG00000079068	adam12	293.189942	0.575603521	0.21213247	2.71341551	0.00665936	0.02213074
ENSDARG00000035137	ehd2a	7.78542434	-4.590860047	1.69202456	-2.71323488	0.00666299	0.02213722

Table S2. DEGs of WT vs. *terfa-/-*

ENSDARG00000070801	opa1	3546.74473	-0.23373093	0.08616801	-2.71250223	0.00667773	0.02218165
ENSDARG00000053840	ei24	723.567584	0.355203439	0.13095359	2.71243754	0.00667904	0.02218165
ENSDARG00000102412	eci2	2292.52025	0.343379415	0.12659266	2.71247484	0.00667829	0.02218165
ENSDARG00000041083	anpepa	140.387531	0.924926696	0.34100104	2.71238676	0.00668006	0.02218208
ENSDARG00000076103	grid2ipa	156.049546	-0.703005967	0.25919734	-2.71224225	0.00668297	0.02218879
ENSDARG00000096995	fbxl22	112.238131	0.74780743	0.27572223	2.71217683	0.00668429	0.02219021
ENSDARG00000053929	opn4b	118.665644	-0.84296243	0.31084787	-2.71181663	0.00669156	0.02221137
ENSDARG00000079730	fuz	147.127021	0.609269823	0.22470008	2.71148018	0.00669836	0.02223008
ENSDARG00000019728	bmpr1aa	2483.54533	-0.267365412	0.09860771	-2.7114047	0.00669988	0.02223008
ENSDARG00000099753	zgc:63863	482.80143	0.482350428	0.17789475	2.71143708	0.00669923	0.02223008
ENSDARG00000093452	BX511129.2	40.8920114	1.051597901	0.38785057	2.71134813	0.00670102	0.0222309
ENSDARG00000037960	lrrc17	2452.82145	0.309777195	0.11426712	2.71099166	0.00670823	0.02225185
ENSDARG00000086512	prodhb	96.8914673	0.729208082	0.26899609	2.71085016	0.0067111	0.02225837
ENSDARG00000031782	crybg1a	2912.99759	0.363299726	0.13403442	2.71049584	0.00671827	0.0222792
ENSDARG00000040930	deptor	934.922733	0.42352389	0.15625702	2.71043107	0.00671958	0.02228058
ENSDARG00000052827	top3a	299.030561	-0.411555976	0.15189249	-2.70952156	0.00673803	0.02232698
ENSDARG00000091655	gcsha	956.117093	0.29253299	0.10795959	2.70965256	0.00673537	0.02232698
ENSDARG00000068103	cfap45	207.803966	0.817963027	0.30188487	2.70951982	0.00673807	0.02232698
ENSDARG00000010673	trim16	486.986803	0.421975985	0.15573145	2.70963889	0.00673565	0.02232698
ENSDARG00000093881	NA	4.34015842	-4.849981758	1.78995169	-2.70956014	0.00673725	0.02232698
ENSDARG00000069669	adra2c	120.194531	-0.575389367	0.21237154	-2.70935247	0.00674147	0.02233527
ENSDARG00000104922	sstr1a	95.0066332	-0.673167565	0.24848272	-2.7091122	0.00674635	0.02234847
ENSDARG00000023323	ywhaqb	15140.3293	-0.260373686	0.09611498	-2.70898129	0.00674902	0.02235431
ENSDARG00000099445	cc2d1b	619.047362	0.485326868	0.17917993	2.70860061	0.00675676	0.02237699
ENSDARG00000069937	dnm2a	1615.42454	0.28232314	0.10425765	2.70793705	0.00677029	0.02241879
ENSDARG00000030350	mnx2b	84.298251	-0.951563404	0.3514076	-2.70786235	0.00677181	0.02242085
ENSDARG00000003820	nr1d2a	4794.04148	0.485926201	0.17947248	2.7075249	0.0067787	0.02244067
ENSDARG00000035470	sh3glb2b	1459.26469	-0.416109538	0.15369825	-2.70731471	0.00678299	0.02245189
ENSDARG00000105831	znf1009	167.272314	-0.599629251	0.22150346	-2.70708747	0.00678764	0.02246129
ENSDARG00000068894	nrip1b	1388.70238	-0.318674261	0.11771785	-2.70710238	0.00678733	0.02246129
ENSDARG00000115029	si:dkeyp-123a12.2	16.9277319	1.736383321	0.64144992	2.7069663	0.00679012	0.02246649
ENSDARG00000031496	ap1ar	585.097713	-0.496109593	0.18328037	-2.70683438	0.00679281	0.02247217
ENSDARG00000090685	tmem241	127.264598	0.660769825	0.2441153	2.70679403	0.00679364	0.02247217
ENSDARG00000089480	si:dkey-12j5.1	690.751494	0.309778257	0.1144497	2.70667613	0.00679605	0.02247716
ENSDARG00000003021	hdac8	536.545979	0.374923199	0.13855897	2.70587463	0.00681248	0.02252849
ENSDARG00000016304	tfdp1b	1075.92476	0.39875965	0.14737678	2.70571559	0.00681574	0.02253628
ENSDARG00000018077	rbp1.1	26.2812335	1.583966455	0.58544388	2.70558204	0.00681848	0.02254234
ENSDARG00000067630	zgc:154075	504.735357	0.393831869	0.14558714	2.70512806	0.00682781	0.02257017
ENSDARG00000100944	glrx2	228.102139	-0.560804956	0.20731967	-2.70502538	0.00682992	0.02257415
ENSDARG00000104018	mtpn	4391.67511	0.224240295	0.08290268	2.70486183	0.00683328	0.02258226
ENSDARG00000087555	si:ch73-308l14.2	394.809829	-0.480410915	0.1776195	-2.7047194	0.00683621	0.02258894
ENSDARG00000070414	si:dkey-61f9.1	3.6209416	-4.85989488	1.79689874	-2.70460142	0.00683864	0.02259396
ENSDARG00000061713	tpd52	859.38756	0.333642325	0.12339418	2.70387403	0.00685363	0.02264047
ENSDARG00000099258	vps52	996.725613	-0.282338253	0.10443902	-2.70337901	0.00686384	0.02267121
ENSDARG00000043972	ppp2r3c	420.19693	-0.37717389	0.13952389	-2.70329257	0.00686563	0.02267409
ENSDARG00000087018	FO704748.1	173.668505	0.603277228	0.22317641	2.70314067	0.00686877	0.02268144
ENSDARG00000102008	si:dkey-72l17.6	35.2604866	1.042610437	0.3857545	2.70278228	0.00687618	0.02270289
ENSDARG00000112489	CU855936.2	102.459628	-0.740601735	0.27406447	-2.70229017	0.00688636	0.02273351
ENSDARG00000097921	BX465857.1	16.7118953	5.379254107	1.99089301	2.70193029	0.00689382	0.0227551
ENSDARG00000037677	fgf24	221.020541	0.570447194	0.21113862	2.70176626	0.00689722	0.02276331
ENSDARG00000088342	tmem145	366.491965	-0.487486996	0.18044438	-2.70159147	0.00690085	0.02277225
ENSDARG00000100712	si:dkey-19b23.12	1170.73357	-0.368534615	0.13645275	-2.70082219	0.00691683	0.02282196
ENSDARG00000112493	BX088712.5	28.6532925	-1.290734834	0.47792603	-2.70070001	0.00691937	0.02282732
ENSDARG00000060767	smg7	4563.98973	-0.265814677	0.09846477	-2.69959171	0.00694246	0.02290045
ENSDARG00000070074	lrp8	2331.37069	-0.400331707	0.14829998	-2.69947241	0.00694495	0.02290562
ENSDARG00000078995	tex11	23.1993733	-1.442936687	0.53469606	-2.69861101	0.00696295	0.02296194
ENSDARG00000101680	arl1	1425.83029	-0.283455544	0.10505374	-2.69819566	0.00697165	0.02298756
ENSDARG00000096689	si:dkey-66i24.9	145.836184	-0.711381792	0.26369883	-2.69770556	0.00698192	0.02301838

Table S2. DEGs of WT vs. *terfa-/-*

ENSDARG00000101497	znf1047	15.7710477	-1.846551607	0.68450729	-2.69763616	0.00698337	0.02302012
ENSDARG00000004930	lmo7a	2606.20724	0.340306602	0.12615921	2.69743765	0.00698754	0.0230308
ENSDARG00000070955	hmx3a	453.23361	-0.497533829	0.18445396	-2.69733346	0.00698972	0.02303495
ENSDARG00000039482	calhm2.1	280.927694	0.562105611	0.20840263	2.69720976	0.00699232	0.02304045
ENSDARG00000009311	pvalb6	961.338722	-0.572298105	0.21219713	-2.6970115	0.00699649	0.02305112
ENSDARG00000056427	auts2a	4618.25264	-0.301152712	0.11166373	-2.69696086	0.00699755	0.02305157
ENSDARG00000061520	b4galnt1a	106.805901	0.842460307	0.31239939	2.69674117	0.00700217	0.02306373
ENSDARG00000100916	uvssa	357.114819	-0.47163277	0.17491453	-2.69636131	0.00701016	0.02308699
ENSDARG00000079555	ccdc85cb	447.689212	-0.430522864	0.15968321	-2.69610599	0.00701553	0.02310163
ENSDARG00000025507	wdr24	770.841462	0.424302372	0.15740164	2.69566676	0.00702479	0.02312905
ENSDARG00000003808	aqp3a	4802.82126	0.373960942	0.13875982	2.69502318	0.00703837	0.02316974
ENSDARG00000059351	hnrnpa3	491.120311	0.521232505	0.19340776	2.69499278	0.00703902	0.02316974
ENSDARG00000035399	ankra2	1019.99358	-0.343232474	0.12737934	-2.69456936	0.00704797	0.02319613
ENSDARG00000095858	CU683879.1	10.3623931	2.00909773	0.74563468	2.69448002	0.00704986	0.02319928
ENSDARG00000112002	NUP85	375.93802	-0.472857704	0.17550088	-2.69433242	0.00705298	0.02320648
ENSDARG00000004735	hnrnpub	10325.5809	-0.403182386	0.14967543	-2.69371119	0.00706614	0.0232467
ENSDARG00000008931	renbp	171.084356	0.709104038	0.26328146	2.6933307	0.00707421	0.02327017
ENSDARG00000100364	FO907110.1	10.7839178	-2.282433209	0.84754989	-2.69297801	0.00708169	0.02328864
ENSDARG00000100116	mrps24	991.609576	0.39480689	0.14660534	2.69299112	0.00708142	0.02328864
ENSDARG00000102210	KCNIP1	268.208597	-0.524022109	0.19459762	-2.69284956	0.00708442	0.02329453
ENSDARG00000098899	zmp:0000001082	214.849563	0.769409303	0.28574549	2.69263851	0.00708891	0.02330311
ENSDARG00000057899	zgc:114081	76.7573859	-0.839569297	0.31179844	-2.69266677	0.00708831	0.02330311
ENSDARG00000055570	gpr61	210.930835	-0.585975422	0.21764246	-2.69237644	0.00709448	0.02331835
ENSDARG00000008333	znfl2a	95.9366949	-0.858230084	0.31879404	-2.69211459	0.00710006	0.02333358
ENSDARG00000061496	si:ch211-250e5.2	172.797772	0.67883826	0.25216633	2.69202579	0.00710195	0.02333671
ENSDARG00000103763	l3hypdh	149.180004	0.724236014	0.26907608	2.69156595	0.00711174	0.02336582
ENSDARG00000076466	maml1	911.585395	-0.311527761	0.11574956	-2.69139467	0.0071154	0.02337473
ENSDARG00000002970	ss18	3400.95153	-0.383383838	0.14246758	-2.69102511	0.00712328	0.02339754
ENSDARG00000052615	tnmd	1398.88733	-0.521466256	0.19379038	-2.69087798	0.00712643	0.02340477
ENSDARG00000014746	rbfox1	5119.63828	-0.343771131	0.12776228	-2.69070914	0.00713003	0.02341352
ENSDARG00000079880	tas2r200.1	55.6239735	-0.939064604	0.34904159	-2.69040893	0.00713645	0.02343151
ENSDARG00000029177	lnx2a	1244.24143	-0.439691877	0.16343604	-2.69029944	0.00713879	0.0234361
ENSDARG00000037353	frs2b	904.302198	-0.36217424	0.13462608	-2.69022345	0.00714042	0.02343834
ENSDARG00000055123	kcnk2a	64.4748078	-0.900495724	0.33474406	-2.69010219	0.00714301	0.02344376
ENSDARG00000014017	rrm1	5282.18764	-0.512461571	0.19055562	-2.68930181	0.00716017	0.02349695
ENSDARG00000093931	rflnb	508.70143	0.382830233	0.14236195	2.68913309	0.00716379	0.02350573
ENSDARG00000012688	eif1	12451.3699	-0.297806339	0.11076075	-2.68873528	0.00717233	0.02353065
ENSDARG00000021582	dynlrb1	49.2439239	1.03535244	0.38509068	2.68859387	0.00717537	0.0235344
ENSDARG00000069106	fam89b	294.110718	-0.47356058	0.1761358	-2.68861069	0.007175	0.0235344
ENSDARG00000010956	sh3glb1b	1195.7882	-0.311455157	0.11585328	-2.68835863	0.00718042	0.02354789
ENSDARG00000092956	si:dkeyp-27c8.1	2.47183361	5.39287525	2.00622358	2.68807291	0.00718657	0.02356494
ENSDARG00000095458	si:dkey-240h12.3	43.3829209	1.253408748	0.46629293	2.68802866	0.00718752	0.02356495
ENSDARG00000025718	cxxc1b	670.971335	-0.407904328	0.15175167	-2.68797266	0.00718873	0.02356579
ENSDARG00000093945	vma21	1046.78491	-0.421666882	0.15689215	-2.68762258	0.00719627	0.0235874
ENSDARG00000102650	BX248331.2	187.859408	0.668076865	0.24860353	2.68731854	0.00720282	0.02360577
ENSDARG00000077124	si:ch211-255p10.4	369.05812	-0.440913997	0.16408534	-2.68710178	0.0072075	0.02361798
ENSDARG00000068064	sirt1	1311.73392	-0.306474686	0.11405771	-2.68701426	0.00720939	0.0236182
ENSDARG00000044754	gnrh2	31.9811038	1.580400213	0.58816299	2.68701067	0.00720947	0.0236182
ENSDARG00000007275	si:ch211-251b21.1	12851.1495	-0.306469482	0.11406042	-2.68690472	0.00721175	0.02362092
ENSDARG00000101676	KLHL28	747.042114	0.359027233	0.13362215	2.68688406	0.0072122	0.02362092
ENSDARG00000110464	CABZ01073097.1	125.414764	-0.598472475	0.22274782	-2.68677138	0.00721463	0.02362578
ENSDARG00000089511	si:ch73-78o10.1	171.848325	0.48594796	0.18088461	2.68650801	0.00722032	0.0236413
ENSDARG00000091359	CAMSAP3	1519.04746	-0.417164937	0.15529107	-2.68634203	0.00722391	0.02364682
ENSDARG00000099657	akt1	299.655249	0.502782704	0.18716125	2.68636117	0.0072235	0.02364682
ENSDARG00000022378	mtmr1b	144.055559	-0.577036837	0.21482756	-2.68604659	0.0072303	0.02366462
ENSDARG00000015199	cblb	901.26628	0.313752887	0.11683266	2.68548966	0.00724236	0.02370097
ENSDARG00000008542	yod1	1302.40815	-0.298461179	0.11114659	-2.68529329	0.00724662	0.02371178
ENSDARG00000062123	agap1	3315.44582	-0.323502002	0.12047819	-2.68515002	0.00724973	0.02371883

Table S2. DEGs of WT vs. *terfa-/-*

ENSDARG00000030614	syt1a	9088.77331	-0.443758978	0.16526926	-2.68506663	0.00725154	0.02372162
ENSDARG00000094910	si:dkey-22i16.7	3.39482058	5.253865528	1.95697264	2.68469034	0.0072597	0.02374522
ENSDARG00000040064	acp6	337.841743	0.466786028	0.17389082	2.68436263	0.00726682	0.02376538
ENSDARG00000005547	rint1	517.566861	0.347660653	0.12951795	2.68426609	0.00726892	0.02376912
ENSDARG00000061963	vrtn	17.3276131	1.378127214	0.51349799	2.68380257	0.00727901	0.02379897
ENSDARG00000079463	man2b2	640.776751	0.498442168	0.18575558	2.68332266	0.00728946	0.02382962
ENSDARG00000040777	inhbb	919.744729	0.326257749	0.12158897	2.68328421	0.0072903	0.02382962
ENSDARG00000006301	RAPH1	2345.03039	-0.367581601	0.13699795	-2.68311757	0.00729393	0.02383836
ENSDARG00000101986	irf6	749.742412	0.305113147	0.11373241	2.68272833	0.00730243	0.02386298
ENSDARG00000113804	si:dkey-205i10.1	13.0540817	1.606576573	0.59892052	2.6824537	0.00730843	0.02387033
ENSDARG00000103648	znf536	3018.78489	-0.359237393	0.1339214	-2.68244949	0.00730852	0.02387033
ENSDARG00000100113	AL935044.2	17.3133987	2.181080898	0.81306346	2.68254694	0.00730639	0.02387033
ENSDARG00000005462	kif4	1878.37555	-0.589302811	0.21968466	-2.6824941	0.00730754	0.02387033
ENSDARG00000020890	tmod4	7313.71451	0.294049924	0.10962223	2.68239318	0.00730975	0.02387122
ENSDARG00000084772	CR678095.1	43.5815528	-0.963151853	0.35911809	-2.68199199	0.00731852	0.02389672
ENSDARG00000092976	si:ch211-127i16.2	465.233498	0.587467731	0.21905081	2.68187889	0.007321	0.02390166
ENSDARG00000077653	rabl6b	1032.87957	-0.27776475	0.10358197	-2.68159365	0.00732724	0.02391891
ENSDARG00000104632	RERG	219.381244	0.493952437	0.18421197	2.68143506	0.00733071	0.0239271
ENSDARG00000096822	CR847898.1	3.72370498	4.825374094	1.79966733	2.68125892	0.00733457	0.02393656
ENSDARG00000014986	acvr1l	2394.58281	-0.283110433	0.10559183	-2.68117734	0.00733636	0.02393892
ENSDARG00000103244	CABZ01081752.3	103.582993	-0.676409615	0.25228451	-2.68113808	0.00733722	0.02393892
ENSDARG00000098198	NA	4.31455102	-4.696463343	1.75172966	-2.68104345	0.0073393	0.02394153
ENSDARG00000037706	gss	352.026155	0.466336708	0.17394044	2.68101371	0.00733995	0.02394153
ENSDARG00000057691	srsf1a	5409.59296	-0.332538117	0.12406108	-2.68043879	0.00735257	0.02397955
ENSDARG00000086126	trim33l	389.625603	0.660602376	0.24646107	2.68035185	0.00735448	0.02397957
ENSDARG00000025024	pnoca	174.121028	0.510102118	0.19031171	2.68035068	0.00735451	0.02397957
ENSDARG00000092731	mhc1uka	25.1231967	-2.071181869	0.77277382	-2.68019155	0.007358	0.02398783
ENSDARG00000037503	thoc2	5135.04408	-0.273122273	0.10191169	-2.67998977	0.00736244	0.02399914
ENSDARG00000060259	zgc:162160	146.314496	-0.558343174	0.2083463	-2.6798804	0.00736485	0.02400384
ENSDARG00000020334	ptpn11a	2112.96471	0.269160923	0.10044992	2.6795533	0.00737205	0.02402415
ENSDARG00000105098	dr1	1184.42197	-0.375100862	0.14000057	-2.679281	0.00737805	0.02404055
ENSDARG00000014462	rab3c	665.273401	-0.446903147	0.16682432	-2.67888494	0.00738678	0.02406355
ENSDARG00000053950	lin37	410.958009	-0.477146547	0.17811466	-2.67887296	0.00738704	0.02406355
ENSDARG00000096862	CR936975.1	3.59781143	4.244686618	1.58467075	2.67859215	0.00739324	0.02407743
ENSDARG00000095594	si:dkey-184a18.5	102.093188	-0.838717632	0.31311786	-2.67860045	0.00739306	0.02407743
ENSDARG00000062552	lpar6a	1022.50522	0.303539742	0.11332556	2.67847547	0.00739582	0.02408267
ENSDARG00000079497	tcima	1817.65314	-0.420038286	0.15682774	-2.67834184	0.00739877	0.02408912
ENSDARG00000030700	ctps1a	2233.94174	0.334268396	0.12481524	2.67810571	0.00740399	0.02410266
ENSDARG00000037607	nol4lb	950.820505	-0.418714395	0.15634955	-2.67806594	0.00740486	0.02410266
ENSDARG00000001431	actn3b	25851.4581	0.378546382	0.14135665	2.67795251	0.00740737	0.02410766
ENSDARG00000007220	ncam1b	1389.19256	-0.317757294	0.11870951	-2.6767637	0.0074337	0.0241902
ENSDARG00000104789	dele1	649.036558	0.352186041	0.13157827	2.6766277	0.00743672	0.02419685
ENSDARG00000062081	tbc1d1	1971.26778	0.259341087	0.09689332	2.67656324	0.00743815	0.02419834
ENSDARG00000020494	znf330	899.5157	0.281139494	0.10505217	2.67618924	0.00744646	0.02422219
ENSDARG00000052721	prim2	555.539618	-0.583231632	0.21794311	-2.67607282	0.00744905	0.02422743
ENSDARG00000100788	trip10b	324.015637	0.491399786	0.18368589	2.67521795	0.00746807	0.02428612
ENSDARG00000057940	dido1	4562.40095	-0.237582837	0.08881566	-2.67501057	0.00747269	0.02429797
ENSDARG00000069607	zgc:162331	149.343355	-1.095205955	0.40943261	-2.6749358	0.00747436	0.02430021
ENSDARG00000054127	slc26a6l	230.679286	0.860076728	0.32154041	2.6748636	0.00747597	0.02430227
ENSDARG00000110963	trib1	781.086261	0.34863643	0.13034766	2.67466582	0.00748038	0.02431343
ENSDARG00000052244	zgc:158564	4424.71137	-0.2339877	0.08748537	-2.6745925	0.00748201	0.02431557
ENSDARG00000101017	CT583642.1	23.9195791	1.273860917	0.47633501	2.67429625	0.00748863	0.02433388
ENSDARG00000007941	hmx4	670.378249	-0.349833301	0.13083709	-2.67380829	0.00749953	0.02436612
ENSDARG00000061242	tuft1a	950.915686	0.427441655	0.15989708	2.67322987	0.00751248	0.02440499
ENSDARG00000058679	zgc:114104	135.189495	0.635504511	0.23776197	2.6728602	0.00752076	0.02442871
ENSDARG00000054170	ltb4r2b	33.6073183	1.097537109	0.41064648	2.67270552	0.00752423	0.02443678
ENSDARG00000045145	shisa9a	349.428511	-0.544796298	0.20385838	-2.67242529	0.00753051	0.024454
ENSDARG00000052833	zfand3	4108.88894	-0.236629047	0.08855174	-2.6722124	0.00753529	0.02446633

Table S2. DEGs of WT vs. *terfa-/-*

ENSDARG00000097501	si:dkey-111k8.3	320.011703	0.510615858	0.19111561	2.67176427	0.00754536	0.02449582
ENSDARG00000087243	zgc:100918	2339.72794	0.345155827	0.12921315	2.67121277	0.00755777	0.0245329
ENSDARG00000029718	p2rx3b	231.274924	-0.670980277	0.25120359	-2.67106166	0.00756118	0.02453507
ENSDARG00000024112	gmppaa	503.187005	-0.360833557	0.13509037	-2.67105316	0.00756137	0.02453507
ENSDARG00000075919	ccser1	277.372711	0.508632328	0.190424	2.6710516	0.0075614	0.02453507
ENSDARG00000102828	mfsd11	187.081551	0.770623606	0.28851933	2.67096004	0.00756347	0.02453856
ENSDARG00000012366	fbp2	1875.98905	-1.018492586	0.38134387	-2.67079837	0.00756711	0.02454718
ENSDARG00000016771	tfa	66385.1245	-0.304233545	0.11392794	-2.67040332	0.00757602	0.02456856
ENSDARG00000043554	mrpl40	742.053404	-0.3096206	0.1159465	-2.67037462	0.00757667	0.02456856
ENSDARG00000100529	sh3bp5a	1638.11785	-0.32347024	0.12113206	-2.67039321	0.00757625	0.02456856
ENSDARG00000039980	ppt1	1353.69553	0.412983846	0.15466107	2.67025077	0.00757946	0.02457442
ENSDARG00000031678	col5a2a	21640.9328	0.245468325	0.09193279	2.67008445	0.00758322	0.02458339
ENSDARG00000040582	C18H3orf33	67.2855656	-0.979620492	0.36691692	-2.66987007	0.00758806	0.02459268
ENSDARG00000106630	LO018627.1	539.135088	-0.646182585	0.24202666	-2.66988184	0.00758779	0.02459268
ENSDARG00000025403	ftr83	930.33706	0.311503392	0.11667723	2.66978734	0.00758993	0.02459553
ENSDARG00000100599	c1s	12.9660784	1.953968741	0.73190742	2.66969387	0.00759204	0.02459878
ENSDARG00000045413	gcm2	481.482811	-0.463594201	0.1736532	-2.66965544	0.00759291	0.02459878
ENSDARG00000103494	usp43a	91.4494458	1.013998463	0.37983407	2.66958269	0.00759456	0.0246009
ENSDARG00000031337	hoxa10b	466.578404	-0.452025764	0.16933503	-2.66941681	0.00759831	0.02460985
ENSDARG00000003320	fam91a1	1534.9031	0.305082257	0.11431425	2.66880349	0.0076122	0.02465162
ENSDARG00000071212	p3h1	718.711004	0.371908097	0.13935955	2.66869475	0.00761466	0.02465639
ENSDARG00000105461	si:dkey-11o1.6	14.9367677	1.642424886	0.61551839	2.66836039	0.00762225	0.02467773
ENSDARG00000105011	pigb	488.79477	-0.394026459	0.14767084	-2.66827529	0.00762418	0.02468076
ENSDARG00000075188	adamts10	1067.28863	0.41824427	0.15675655	2.66811344	0.00762785	0.02468944
ENSDARG00000102020	pnpla3	570.09879	0.621678855	0.23307626	2.66727665	0.00764687	0.02474134
ENSDARG00000073896	cx47.1	34.6327116	-1.480797654	0.55516743	-2.66729923	0.00764636	0.02474134
ENSDARG00000114226	CABZ01102109.1	554.252994	-0.37862437	0.14195125	-2.66728453	0.00764669	0.02474134
ENSDARG00000105391	strc	89.8645347	0.773780662	0.29017345	2.66661427	0.00766196	0.02478692
ENSDARG00000070702	s100v2	1243.51227	0.507170763	0.19019936	2.66652195	0.00766406	0.0247905
ENSDARG00000094681	hbl2	49.4257508	-1.216798693	0.45634496	-2.66640108	0.00766682	0.02479619
ENSDARG00000006025	tp53i11b	823.413126	-0.336828726	0.12633894	-2.66607206	0.00767432	0.02481724
ENSDARG00000103665	brpf1	1477.78894	-0.40682853	0.15260005	-2.66597903	0.00767645	0.02482088
ENSDARG00000087516	CU693494.1	10.5028106	-2.293050357	0.86024598	-2.6655752	0.00768567	0.02484748
ENSDARG00000099885	trim105	348.166028	-0.398785866	0.14960841	-2.66553109	0.00768668	0.02484751
ENSDARG00000056886	tbccd1	253.767755	-0.462031672	0.17335893	-2.66517371	0.00769486	0.0248707
ENSDARG00000086877	mhc1zda	298.476019	-0.486495822	0.18255977	-2.66485776	0.00770209	0.02489084
ENSDARG00000103607	wtip	469.192501	0.369860455	0.1387949	2.66479867	0.00770344	0.02489197
ENSDARG00000014050	ngfb	205.218232	0.450233467	0.16897242	2.66453824	0.00770941	0.02490802
ENSDARG00000101044	si:ch73-138e16.6	628.517219	-0.333039554	0.12499445	-2.66443478	0.00771178	0.02491244
ENSDARG00000032126	scg5	336.073622	-0.477801703	0.17934054	-2.66421467	0.00771683	0.02492269
ENSDARG00000075299	prxl2c	151.412904	0.714743901	0.26827621	2.66420906	0.00771696	0.02492269
ENSDARG00000006290	ndufs5	3396.01767	0.285554229	0.10719376	2.66390729	0.00772389	0.02494182
ENSDARG00000101926	zgc:101130	243.813651	-0.484630656	0.1819791	-2.66311165	0.00774217	0.02499762
ENSDARG00000028848	lsm12a	736.100558	-0.440499223	0.16541062	-2.66306499	0.00774325	0.02499784
ENSDARG00000062465	pgap1	497.969753	0.401489215	0.15079977	2.66239934	0.00775858	0.02504408
ENSDARG00000061862	myo18ab	5462.10554	0.285665862	0.10732147	2.66177752	0.00777292	0.02508713
ENSDARG00000103330	si:ch73-236c18.5	144.741461	-1.965317301	0.73842637	-2.66149395	0.00777947	0.02510346
ENSDARG00000102145	ccdc77	394.595354	-0.629781036	0.23662892	-2.66147116	0.00778	0.02510346
ENSDARG00000091723	si:ch211-202f3.4	40.9138611	-1.156067082	0.4344473	-2.66100647	0.00779075	0.02513452
ENSDARG00000116862	pimr64	13.8808255	-1.736127714	0.65244232	-2.66096736	0.00779165	0.02513452
ENSDARG00000061147	zfp64	312.189442	-0.404391445	0.15198641	-2.66070798	0.00779766	0.02515063
ENSDARG00000020702	odf2b	271.927743	-0.64570739	0.24272776	-2.6602124	0.00780914	0.02518113
ENSDARG00000001259	gas2a	124.708007	0.643239686	0.24179874	2.6602276	0.00780879	0.02518113
ENSDARG00000101118	CABZ01066813.1	398.348047	-0.375545035	0.14119732	-2.65971794	0.00782061	0.02521361
ENSDARG00000075245	wdr11	1674.50989	0.282593099	0.10625057	2.6596856	0.00782136	0.02521361
ENSDARG00000078775	RASA2	341.503851	0.569044084	0.21395473	2.65964714	0.00782226	0.02521361
ENSDARG00000099207	si:dkey-11f4.7	69.2880157	1.113506885	0.41867605	2.65959059	0.00782357	0.02521458
ENSDARG00000099889	dusp27	1053.20324	0.373949583	0.14064639	2.65879266	0.00784212	0.02527109

Table S2. DEGs of WT vs. *terfa-/-*

ENSDARG00000043665	glrx5	1408.93973	0.310409261	0.11675426	2.65865463	0.00784533	0.02527817
ENSDARG00000002587	dpysl3	7735.93483	-0.380668074	0.14331322	-2.65619658	0.00790275	0.02545987
ENSDARG00000045799	acanb	1318.75469	0.505101855	0.19016819	2.65607964	0.00790549	0.0254654
ENSDARG00000099045	cacnb2a	848.543989	-0.307631193	0.11582864	-2.65591658	0.00790932	0.02547442
ENSDARG00000097714	FP017215.1	211.947838	0.688186527	0.25912217	2.65583811	0.00791116	0.02547705
ENSDARG00000092060	tbx5b	70.0073062	-1.034124584	0.38939972	-2.65568905	0.00791466	0.02548501
ENSDARG00000040254	hormad1	6.76137352	-4.317283375	1.62572059	-2.65561217	0.00791646	0.02548752
ENSDARG00000041619	rack1	63297.4006	0.298400952	0.11239371	2.65496125	0.00793175	0.02553344
ENSDARG00000104817	pyroxd1	456.673898	0.352813012	0.13289374	2.6548505	0.00793436	0.02553852
ENSDARG00000113657	LO018363.2	650.726064	-0.480191424	0.18088786	-2.65463603	0.0079394	0.02555145
ENSDARG00000093709	si:dkeyp-7a3.1	16.2532565	1.674227259	0.63074482	2.65436547	0.00794577	0.02556864
ENSDARG00000008107	src	958.735425	0.378098484	0.14244988	2.65425627	0.00794834	0.0255736
ENSDARG00000076280	ppp1r1b	603.243042	-0.361271474	0.13612747	-2.6539204	0.00795626	0.02559245
ENSDARG00000097648	si:dkey-35i13.1	1244.78669	-0.387713295	0.14608939	-2.65394559	0.00795567	0.02559245
ENSDARG00000091656	lsm8	536.810096	-0.449975542	0.16956188	-2.65375419	0.00796018	0.02560174
ENSDARG00000055288	prelid3a	331.230947	-0.428087799	0.16133765	-2.65336573	0.00796935	0.02562128
ENSDARG00000036194	pitx2	1135.5994	-0.415935769	0.15675455	-2.65342072	0.00796805	0.02562128
ENSDARG00000017369	sema3d	1757.99197	-0.280895187	0.10586302	-2.65338352	0.00796893	0.02562128
ENSDARG00000104118	antxr2a	741.539148	0.508579192	0.19168218	2.65324192	0.00797227	0.02562423
ENSDARG00000053992	sfi1	402.244404	-0.541021384	0.20391455	-2.65317697	0.00797381	0.02562423
ENSDARG00000098838	sdr42e1	129.038406	0.56146783	0.21162292	2.65315229	0.00797439	0.02562423
ENSDARG00000093873	BX324213.1	18.2046406	1.894204002	0.71394001	2.65316972	0.00797398	0.02562423
ENSDARG00000069698	lrguk	109.174194	0.745960869	0.28119266	2.65284614	0.00798162	0.02564417
ENSDARG00000029402	sort1a	1360.79088	-0.293221357	0.11055512	-2.6522639	0.0079954	0.02568179
ENSDARG00000114036	BX248510.6	45.5579683	-0.974938851	0.36758462	-2.65228409	0.00799492	0.02568179
ENSDARG00000114124	si:dkey-3p4.8	16.7827209	-1.888595405	0.71211062	-2.65210959	0.00799906	0.02569022
ENSDARG00000099923	zgc:154054	974.962516	0.330184223	0.12453158	2.65140957	0.00801566	0.0257402
ENSDARG00000098177	si:dkey-28i19.3	17.6695475	1.463897785	0.55218712	2.65109005	0.00802325	0.02576124
ENSDARG00000061378	smg8	768.093947	-0.324358045	0.12236388	-2.65076619	0.00803094	0.02578262
ENSDARG00000089318	BX088538.1	186.679633	0.565124917	0.21324625	2.65010482	0.00804668	0.02582981
ENSDARG00000009740	ppp1r7	1323.42074	-0.316659199	0.1194999	-2.64986991	0.00805228	0.02584444
ENSDARG00000092445	zgc:153352	71.0217328	0.819860688	0.30946737	2.64926377	0.00806674	0.0258875
ENSDARG00000040911	meox2a	81.7202476	-0.68428694	0.25829792	-2.64921586	0.00806788	0.02588783
ENSDARG00000101445	klhl7	594.706937	-0.422486465	0.15949252	-2.64894218	0.00807441	0.02590546
ENSDARG00000075829	kiss1	5.32723582	-3.47530929	1.31199268	-2.64887856	0.00807593	0.02590699
ENSDARG00000107511	lyn	465.634781	-0.370521585	0.13988331	-2.64879047	0.00807804	0.0259104
ENSDARG00000036094	pias1a	654.462605	-0.383585256	0.14483393	-2.64844888	0.00808621	0.02593325
ENSDARG00000090145	tmem240b	295.510073	0.60351642	0.22788193	2.64837327	0.00808802	0.02593571
ENSDARG00000075107	nkx2.4a	180.789569	-0.65956459	0.24905582	-2.64826006	0.00809073	0.02594022
ENSDARG00000100487	nploc4	1566.29485	-0.314953193	0.11892982	-2.64822725	0.00809151	0.02594022
ENSDARG00000045685	cntn1b	1326.81556	-0.415623673	0.15698377	-2.64755826	0.00810754	0.02598802
ENSDARG00000044574	rbms3	914.534255	0.424768208	0.16044018	2.64751768	0.00810851	0.02598802
ENSDARG00000042215	pias4b	22.9296093	1.628485971	0.61515535	2.64727596	0.00811431	0.02600325
ENSDARG00000045513	taf3	1849.74448	-0.310531589	0.11731344	-2.64702475	0.00812034	0.02601922
ENSDARG00000077471	hmces	50.0797438	-0.924382164	0.34927944	-2.64654042	0.00813198	0.02605315
ENSDARG00000002235	mmp14a	3469.73723	0.28367332	0.10720386	2.64611106	0.00814231	0.02608288
ENSDARG00000075887	herc3	704.888504	0.323439843	0.1222494	2.64573771	0.0081513	0.02610832
ENSDARG00000093082	LO018605.1	2.39017284	4.692833579	1.77386548	2.64554084	0.00815604	0.02612015
ENSDARG00000092823	si:dkeyp-67a8.4	39.8051171	1.240883219	0.4690553	2.64549451	0.00815716	0.02612036
ENSDARG00000019541	gpt2l	3419.22165	0.344433792	0.13020199	2.64538039	0.00815991	0.02612244
ENSDARG00000079238	trim59	226.076263	-0.561738505	0.21234564	-2.64539695	0.00815951	0.02612244
ENSDARG00000004452	tardbpl	7894.25951	-0.30171788	0.11405686	-2.64532856	0.00816116	0.02612308
ENSDARG00000104733	coq4	212.299902	0.50853303	0.19225133	2.64514707	0.00816554	0.02613037
ENSDARG00000012671	inhbaa	402.674899	0.40871044	0.15451162	2.64517614	0.00816484	0.02613037
ENSDARG00000016994	ssrp1b	680.682358	0.362297373	0.13698251	2.64484407	0.00817286	0.02615041
ENSDARG00000090524	f2rl2	75.437464	0.846231871	0.32000943	2.64439671	0.00818367	0.02618163
ENSDARG00000117760	CU929144.1	42.0142243	1.083537294	0.40977814	2.64420475	0.00818831	0.02619311
ENSDARG00000093799	setbp1	1213.00699	-0.431244399	0.16312367	-2.64366539	0.00820137	0.02622476

Table S2. DEGs of WT vs. *terfa-/-*

ENSDARG00000076638	FO082781.1	620.305123	-0.543757896	0.20568241	-2.64367723	0.00820108	0.02622476
ENSDARG00000053619	sra1	488.665231	0.353129396	0.13357348	2.64370898	0.00820031	0.02622476
ENSDARG00000043627	tbc1d9	729.256603	0.449948292	0.17020297	2.64359834	0.00820299	0.02622658
ENSDARG00000011373	mknk2a	385.560232	0.448141571	0.16956791	2.64284419	0.00822129	0.02628168
ENSDARG00000032963	her12	211.434579	-0.743077725	0.2812175	-2.64235944	0.00823306	0.02631594
ENSDARG00000035551	iqcd	51.3187721	0.952492649	0.3604793	2.642295	0.00823463	0.02631757
ENSDARG00000059914	sdr42e2	504.024244	0.543901874	0.20588255	2.64180654	0.00824651	0.02635216
ENSDARG00000008490	ltv1	1266.99496	0.327233804	0.12388241	2.64148725	0.00825429	0.02637362
ENSDARG00000103977	terf2ip	303.428227	-0.489725868	0.18544181	-2.64086012	0.00826959	0.02641909
ENSDARG00000075225	si:ch211-223a10.1	214.689401	-0.60460981	0.22896599	-2.64060972	0.0082757	0.02643523
ENSDARG00000011201	rplp2l	27421.7196	0.331946002	0.12572142	2.64032978	0.00828254	0.02645368
ENSDARG00000026109	slc48a1b	744.709831	0.31484881	0.11926855	2.63983089	0.00829474	0.02648564
ENSDARG00000100248	c1r	26.2437607	1.532313453	0.58046071	2.63982287	0.00829494	0.02648564
ENSDARG00000031100	ivns1abpa	7662.94372	-0.268039985	0.10153838	-2.63978993	0.00829574	0.02648564
ENSDARG00000029930	SPDEF	65.4340138	0.728113947	0.27583164	2.63970422	0.00829784	0.02648893
ENSDARG00000098087	si:dkey-30f3.2	21.9997117	1.665679111	0.63109113	2.63936383	0.00830618	0.02651214
ENSDARG00000056742	crmp1	2077.19515	-0.450494311	0.17068652	-2.63930807	0.00830755	0.0265131
ENSDARG00000114309	BX005421.3	5.30323974	2.652762053	1.0052097	2.63901359	0.00831477	0.02653274
ENSDARG00000041951	selenoo1	688.862792	0.286959334	0.10874488	2.63883073	0.00831925	0.02654364
ENSDARG00000058815	ihhb	54.5828447	-0.928428507	0.35189806	-2.63834503	0.00833118	0.02657828
ENSDARG00000103500		2.44443202	5.080168032	1.92567967	2.63811688	0.00833678	0.02659276
ENSDARG00000019986	grhprb	5872.74507	0.421050509	0.15961044	2.63798846	0.00833994	0.02659942
ENSDARG00000077986	TMEM151A	546.510943	-0.331145289	0.1255335	-2.63790381	0.00834202	0.02660264
ENSDARG00000091869	zgc:162958	202.538266	-0.504427405	0.19123476	-2.6377391	0.00834608	0.02660874
ENSDARG00000009026	ank2a	2955.92271	0.3358618	0.12732791	2.63777051	0.0083453	0.02660874
ENSDARG00000105177	ilf3b	9864.91785	-0.375377325	0.14232152	-2.63753032	0.00835122	0.02662171
ENSDARG00000036929	rnf139	1596.29941	0.263507993	0.09992087	2.63716672	0.00836017	0.02664343
ENSDARG00000059700	agmat	302.119157	0.574180963	0.21772592	2.63717325	0.00836001	0.02664343
ENSDARG00000111198	BX640461.1	4.96224922	-4.425933258	1.67865791	-2.63659036	0.00837439	0.02668531
ENSDARG00000036809	ANPEP	1458.23905	-0.400855677	0.1520438	-2.63644878	0.00837789	0.02669164
ENSDARG00000060102	kank1a	3489.63477	0.239311066	0.0907801	2.63616217	0.00838497	0.02669164
ENSDARG00000098655	KIF1C	261.590548	0.516970658	0.19610672	2.63617	0.00838477	0.02669164
ENSDARG00000096599	AL928650.3	25.3785675	-1.405716132	0.53320567	-2.63634882	0.00838035	0.02669164
ENSDARG00000095067	TC2N	81.9314484	0.924329968	0.35063422	2.63616586	0.00838487	0.02669164
ENSDARG00000090179	si:ch211-117c9.1	40.0975336	-1.11889827	0.42441157	-2.63635198	0.00838028	0.02669164
ENSDARG00000100670	pcdh1g31	452.459207	-0.53316686	0.20224274	-2.63627197	0.00838225	0.02669164
ENSDARG00000111747	bloc1s5	251.641396	-0.549394852	0.20840665	-2.63616762	0.00838483	0.02669164
ENSDARG00000103672	cirbpa	37437.0227	-0.394566518	0.1496916	-2.6358628	0.00839237	0.02671178
ENSDARG00000045527	nr2c1	1027.80689	-0.337679012	0.12811672	-2.63571379	0.00839605	0.02672009
ENSDARG00000089852	si:dkey-51d8.1	11.4213297	1.954255072	0.74146849	2.63565494	0.00839751	0.0267213
ENSDARG00000079926	syt15	35.9764258	-1.157713087	0.43928172	-2.63546837	0.00840213	0.02673257
ENSDARG00000110528	sypa	8595.02569	-0.349654225	0.13268098	-2.63530026	0.00840629	0.02674239
ENSDARG00000117309	BX927081.3	27.5088847	-1.448124313	0.54955377	-2.63509123	0.00841147	0.02675544
ENSDARG00000098992	zgc:114119	580.019377	-0.502990711	0.19088619	-2.63502931	0.008413	0.0267569
ENSDARG00000044039	stx11a	68.4917882	0.788780735	0.29935795	2.63490822	0.008416	0.02676302
ENSDARG00000081375	dre-mir-457b	6.79690664	-4.145234937	1.57324311	-2.63483432	0.00841784	0.02676542
ENSDARG00000045075	tmem106a	645.085258	0.389885299	0.14798039	2.63470915	0.00842094	0.02677187
ENSDARG00000003085	stambpl1	304.822152	-0.402900836	0.15292776	-2.63458277	0.00842408	0.02677841
ENSDARG00000077582	ank3b	1621.93308	0.476324369	0.18080941	2.63440027	0.00842861	0.02678939
ENSDARG00000057699	styx	372.849408	-0.470668982	0.17866669	-2.63434095	0.00843008	0.02679064
ENSDARG00000055754	smc1a	344.295741	-1.490078087	0.56566898	-2.63418737	0.00843389	0.02679934
ENSDARG00000075248	zgc:172133	9.14133047	2.363676972	0.89742408	2.63384615	0.00844237	0.02682285
ENSDARG00000021905	derl1	1479.40313	0.257007284	0.09759982	2.63327617	0.00845656	0.02686448
ENSDARG00000075850	gnal2	456.341439	-0.400585167	0.15213018	-2.63317363	0.00845911	0.02686916
ENSDARG00000012281	zgc:65851	2266.17859	0.37863883	0.14380219	2.63305331	0.00846211	0.02687524
ENSDARG00000100386	si:dkey-61p9.9	22.4549636	-1.696872768	0.64448977	-2.63289325	0.0084661	0.02688342
ENSDARG00000016128	ap3m2	1464.26661	-0.338938641	0.12873598	-2.63281984	0.00846793	0.02688342
ENSDARG00000089681	btf3l4	1457.99886	-0.283076589	0.10751713	-2.63285116	0.00846714	0.02688342

Table S2. DEGs of WT vs. *terfa-/-*

ENSDARG00000055527	cmn	3562.49757	-0.599500656	0.22772222	-2.63259619	0.0084735	0.02689769
ENSDARG00000038576	ube2d1b	1433.3004	-0.283437041	0.10768198	-2.63216776	0.0084842	0.02692819
ENSDARG00000102975	nsg2	4987.41685	-0.384058351	0.14592172	-2.6319479	0.00848969	0.02694219
ENSDARG00000027978	yy1b	1670.70535	-0.293868953	0.11166629	-2.63167104	0.00849661	0.02696071
ENSDARG00000055538	avl9	837.821027	-0.27937667	0.1061752	-2.63127989	0.0085064	0.02698831
ENSDARG00000090654	gak	3541.02968	-0.245529529	0.09331497	-2.63119123	0.00850861	0.02699191
ENSDARG00000036870	arih1l	801.616921	-0.313938716	0.11932727	-2.63090511	0.00851578	0.0270112
ENSDARG00000095908	sat1a.2	2211.93069	0.378488928	0.14387402	2.6306969	0.008521	0.02702085
ENSDARG00000100104	zgc:101785	86.1162406	0.902289515	0.34298229	2.63071753	0.00852048	0.02702085
ENSDARG00000092044	si:dkey-22f5.9	116.199335	-1.073835876	0.40820632	-2.6306204	0.00852292	0.02702349
ENSDARG00000006553	rras	1649.90308	0.357587267	0.13594485	2.63038479	0.00852883	0.02703623
ENSDARG00000016016	dbnlb	2024.37853	0.264977998	0.10073778	2.63037349	0.00852911	0.02703623
ENSDARG00000112930	CT737184.2	49.9677503	-1.237307204	0.47045841	-2.63000337	0.0085384	0.02706223
ENSDARG00000070833	lin52	209.636176	-0.558736296	0.21250376	-2.62930075	0.00855607	0.02711475
ENSDARG00000089627	si:ch211-160d20.5	5.58539206	-3.690112217	1.40348371	-2.62925191	0.00855729	0.02711519
ENSDARG00000100962	ttc39b	602.631324	0.432952567	0.16467715	2.6290993	0.00856114	0.0271239
ENSDARG00000088595	crispld2	227.095818	0.592161939	0.22524107	2.62901402	0.00856328	0.02712725
ENSDARG00000098623	si:dkey-23f9.4	150.0448	-0.610301108	0.23214455	-2.62897025	0.00856439	0.02712728
ENSDARG00000038248	ggact.2	318.699855	0.599450783	0.22803023	2.62882159	0.00856813	0.02713569
ENSDARG00000005518	slc5a9	513.731681	0.597604497	0.22737511	2.62827579	0.00858189	0.02717507
ENSDARG00000089355	si:dkeyp-121d4.3	2091.93221	-0.288218407	0.10966207	-2.62824162	0.00858275	0.02717507
ENSDARG00000097346	ten1	80.3678771	0.869146852	0.33073489	2.62792609	0.00859072	0.02719683
ENSDARG00000059950	plxdc2	2254.80926	0.329033863	0.12521003	2.62785546	0.0085925	0.02719901
ENSDARG00000102594	zgc:194627	224.254484	-0.860997385	0.32769521	-2.62743351	0.00860317	0.0272293
ENSDARG00000053131	irak3	16.782607	1.873396799	0.71309017	2.62715276	0.00861027	0.02724831
ENSDARG00000110991	ZNF276	1333.64237	-0.346201998	0.13178825	-2.62695649	0.00861524	0.02726056
ENSDARG00000105029	TPPP	154.145607	-0.711270773	0.27076951	-2.62684963	0.00861794	0.02726565
ENSDARG00000073901	CABZ01046997.1	20.246293	3.713735338	1.41420395	2.62602529	0.00863884	0.02732829
ENSDARG00000024818	rilpl2	324.298921	0.425035868	0.16186771	2.62582253	0.00864399	0.02734109
ENSDARG00000043732	cops4	2233.45	-0.253624041	0.09661067	-2.62521773	0.00865936	0.02738622
ENSDARG00000007825	map2k1	1517.47234	0.418934159	0.15960836	2.62476331	0.00867092	0.02741931
ENSDARG00000063646	lnpa	200.220894	0.516503908	0.1967936	2.62459713	0.00867516	0.02742572
ENSDARG00000033742	nt5c1bb	256.937982	0.448024478	0.17070031	2.62462606	0.00867442	0.02742572
ENSDARG00000022399	ntmt1	1126.06647	0.306253185	0.11673623	2.62346315	0.00870409	0.02751368
ENSDARG00000002682	tbcela	902.422775	-0.36813182	0.14032857	-2.62335614	0.00870682	0.02751882
ENSDARG00000078386	dync1i2a	3718.59839	0.243269014	0.09274097	2.62310192	0.00871332	0.02753236
ENSDARG00000031198	hmg20a	767.386931	-0.388255947	0.14801355	-2.62311091	0.00871309	0.02753236
ENSDARG00000068892	sostdc1a	650.045406	0.390030229	0.14872572	2.62248008	0.00872924	0.02757915
ENSDARG00000032087	sgsh	434.66798	0.358745883	0.13680547	2.62230665	0.00873368	0.02758969
ENSDARG00000077573	tmprss13b	93.1439021	0.829233851	0.31627697	2.62185971	0.00874514	0.02762238
ENSDARG00000018263	pdia2	943.763402	-1.194036224	0.45547112	-2.62154104	0.00875332	0.0276447
ENSDARG00000058154	znf385c	643.035512	-0.401344965	0.15310461	-2.62137744	0.00875752	0.02765446
ENSDARG00000088805	nes	802.891958	0.334843617	0.12774274	2.62123407	0.00876121	0.02766258
ENSDARG00000058459	plekhg2	1116.6019	0.368776635	0.14069933	2.62102619	0.00876655	0.02767594
ENSDARG00000087616	maptb	3612.74762	-0.279124115	0.10650259	-2.62081994	0.00877186	0.02768703
ENSDARG00000073792	man1b1a	541.092353	-0.343974769	0.13124785	-2.62080303	0.00877229	0.02768703
ENSDARG00000021945	ddx23	2901.24562	-0.307540838	0.1173553	-2.62059603	0.00877762	0.02770033
ENSDARG00000096121	CR381686.4	5.52627418	3.299352801	1.25924198	2.62011023	0.00879014	0.0277363
ENSDARG00000017084	bud31	1313.20982	-0.40161292	0.15328974	-2.61995954	0.00879402	0.0277438
ENSDARG00000058062	magt1	1450.97015	0.307481642	0.11736248	2.61993137	0.00879475	0.0277438
ENSDARG00000021913	ak9	161.584446	0.683696316	0.26096533	2.61987409	0.00879622	0.02774494
ENSDARG00000079840	kcnma1a	1623.09831	-0.390449896	0.14907727	-2.61911079	0.00881593	0.02780005
ENSDARG00000096725	si:dkey-21c1.1	509.200736	0.439915272	0.1679611	2.61914971	0.00881493	0.02780005
ENSDARG00000043740	efcab11	71.8626019	1.011208415	0.38615839	2.61863639	0.0088282	0.02782814
ENSDARG00000008434	bcl2l1	1858.64816	0.239772609	0.0915616	2.61870282	0.00882648	0.02782814
ENSDARG00000071541	si:dkey-42i9.8	3.77299694	-4.874272995	1.86134966	-2.6186767	0.00882716	0.02782814
ENSDARG00000017985	C1H4orf33	71.0334726	0.752528179	0.28740085	2.6183923	0.00883452	0.02784453
ENSDARG00000117431	CABZ01065684.1	199.061472	-0.487099533	0.18606828	-2.61785368	0.00884847	0.02788498

Table S2. DEGs of WT vs. *terfa-/-*

ENSDARG00000024904	stil	289.794594	-0.661101976	0.25256742	-2.61752679	0.00885695	0.02790816
ENSDARG00000070025	dchs1a	1097.37003	0.400375145	0.15296421	2.61744327	0.00885912	0.02791145
ENSDARG00000063407	LO018340.1	2545.5037	0.301312986	0.11512543	2.61725831	0.00886392	0.02792304
ENSDARG00000092381	zgc:153759	9.63518181	2.523546783	0.96422625	2.61717287	0.00886614	0.0279265
ENSDARG00000010603	kctd9b	448.767641	-0.383128566	0.1463983	-2.61702887	0.00886988	0.02793474
ENSDARG00000095295	si:rp71-36a1.1	249.883009	0.540341775	0.20649403	2.61674281	0.00887732	0.02795462
ENSDARG00000095434	si:ch211-217k17.8	617.342925	0.560480962	0.21421319	2.61646332	0.00888459	0.02797397
ENSDARG00000099541	CABZ01086734.1	304.825361	-0.766445918	0.29295039	-2.61629934	0.00888886	0.02798386
ENSDARG00000063255	btbd11a	948.436453	0.342518467	0.13092209	2.61620083	0.00889142	0.02798839
ENSDARG00000059701	flii	2752.55574	0.265068369	0.10133735	2.61570255	0.00890441	0.02801862
ENSDARG00000030448	sppl2	1149.78693	-0.372126603	0.14226458	-2.61573619	0.00890353	0.02801862
ENSDARG00000076339	cln5	103.123355	-0.886833907	0.33903318	-2.61577322	0.00890257	0.02801862
ENSDARG00000017522	ctdp1	635.739204	-0.443962526	0.16973322	-2.61564906	0.0089058	0.02801946
ENSDARG00000103946	si:dkey-124l13.1	59.4232084	-1.045883252	0.39986277	-2.6156055	0.00890694	0.02801949
ENSDARG00000101890	pigr	371.752386	0.451618785	0.17270398	2.61498771	0.00892307	0.02806667
ENSDARG00000094914	si:ch73-196i15.5	9.80962985	2.142583856	0.81982897	2.61345224	0.00896326	0.02818953
ENSDARG00000054433	yipf6	824.24919	0.316222282	0.12101775	2.61302394	0.0089745	0.02822131
ENSDARG00000060392	ash2l	2331.49143	-0.34674537	0.13272651	-2.61248012	0.00898879	0.02826267
ENSDARG00000033845	igsf9ba	3076.61473	-0.339514313	0.1299657	-2.61233775	0.00899254	0.02827087
ENSDARG00000031976	nos2b	88.9166472	0.754001442	0.28864476	2.61221248	0.00899583	0.02827435
ENSDARG00000079740	efna2b	106.528732	-0.691215769	0.26460965	-2.61220927	0.00899592	0.02827435
ENSDARG00000004587	kat5a	724.217667	-0.3559325	0.13627423	-2.61188406	0.00900448	0.0282953
ENSDARG00000076487	haao	511.934278	0.623577573	0.2387476	2.61186953	0.00900486	0.0282953
ENSDARG00000095843	col24a1	907.72442	0.30195196	0.11561123	2.61178749	0.00900702	0.02829851
ENSDARG00000069459	si:dkey-6n21.13	54.1689363	0.90853348	0.34788848	2.61156532	0.00901288	0.02831254
ENSDARG00000032801	grk5	148.860348	0.568953386	0.21786196	2.61153162	0.00901377	0.02831254
ENSDARG00000089291	cenpx	236.69094	0.537431948	0.2058196	2.61117959	0.00902305	0.02833812
ENSDARG00000067723	tank	184.354045	0.552494834	0.21159968	2.61103811	0.00902678	0.02834269
ENSDARG00000082187	dre-mir-21-1	2.45085822	5.079445128	1.94536667	2.61104768	0.00902653	0.02834269
ENSDARG00000038879	ift80	822.334625	0.350099132	0.13410303	2.61067284	0.00903643	0.02836939
ENSDARG00000058958	trim35-7	30.5661782	1.474606318	0.56495787	2.61011732	0.00905112	0.02841191
ENSDARG00000097145	FP565432.1	25.9010114	1.361067422	0.52158895	2.60946369	0.00906843	0.02846265
ENSDARG00000094052	taok3b	475.429381	-0.440048555	0.16864666	-2.60929303	0.00907295	0.02847326
ENSDARG00000011175	atp6v1d	4285.24485	-0.268115924	0.10276996	-2.60889404	0.00908354	0.02850288
ENSDARG00000032997	rnf185	831.214161	0.323268564	0.12394452	2.60817153	0.00910273	0.02855951
ENSDARG00000057395	si:ch211-130m23.[illegible]	92.3945855	0.657300069	0.25202842	2.60803946	0.00910625	0.02856692
ENSDARG00000097060	CR388369.1	6.89622527	2.582876132	0.99084233	2.60674788	0.00914066	0.02866917
ENSDARG00000039661	slc36a4	967.621858	0.315268323	0.12094399	2.60672988	0.00914114	0.02866917
ENSDARG00000043986	setd6	226.603774	0.458581918	0.17592783	2.60664793	0.00914333	0.02867241
ENSDARG00000094132	igf1	128.790531	-0.570954465	0.21906022	-2.60638136	0.00915045	0.02868388
ENSDARG00000021392	chrnb5b	59.9227056	-0.887930497	0.34066897	-2.60643196	0.0091491	0.02868388
ENSDARG00000092456	BX284635.1	136.808691	-0.646400093	0.24800487	-2.60640084	0.00914993	0.02868388
ENSDARG00000019715	metap1d	229.982581	-0.494291218	0.18965914	-2.6062083	0.00915508	0.02869476
ENSDARG00000099139	fbxw5	1493.03151	-0.237029971	0.09095456	-2.60602614	0.00915995	0.0287064
ENSDARG00000116491	h2az2b	12596.4224	-0.479446244	0.1839882	-2.60585321	0.00916457	0.02871728
ENSDARG00000011635	scxb	37.9098764	-1.008426519	0.38709176	-2.60513558	0.00918379	0.02877387
ENSDARG00000071493	olfm3a	317.505381	-0.409457109	0.1571997	-2.60469403	0.00919563	0.02880735
ENSDARG00000103337	rrp1	1279.48976	-0.320682613	0.12312264	-2.60457877	0.00919873	0.02881341
ENSDARG00000025948	mlh1	306.715332	-0.491316084	0.18864992	-2.60438003	0.00920406	0.02882649
ENSDARG00000104901	ostc	1926.52981	0.247510513	0.09504616	2.60410861	0.00921136	0.02884569
ENSDARG00000075858	fam155a	182.576955	-0.574892401	0.22079495	-2.60373889	0.0092213	0.02887319
ENSDARG00000097085	si:ch211-261a10.5	13.463802	2.099421422	0.80635063	2.60360859	0.0092248	0.02888053
ENSDARG00000098928	si:ch211-196h24.1	19.1799969	1.344136121	0.51628291	2.60348755	0.00922806	0.02888709
ENSDARG00000059936	herc4	779.772947	0.373659193	0.14353084	2.60333726	0.00923211	0.02889612
ENSDARG00000033587	CABZ01088134.1	17.0072193	1.461794964	0.56152194	2.60327309	0.00923384	0.02889789
ENSDARG00000024815	ogfrl2	102.176094	0.671094541	0.25780657	2.60309326	0.00923868	0.02890941
ENSDARG00000101508	nr6a1a	90.9628487	-0.867336669	0.33321413	-2.60294089	0.00924279	0.02891862
ENSDARG00000077415	zmp:0000001175	30.336616	1.811032206	0.69579992	2.602806	0.00924643	0.02892636

Table S2. DEGs of WT vs. *terfa-/-*

ENSDARG00000007630	mkrn2	699.083233	0.344152654	0.13223455	2.60259264	0.00925218	0.02894072
ENSDARG00000079620	amigo1	405.631921	-0.520657585	0.2000891	-2.60212872	0.00926471	0.02897062
ENSDARG00000100317	ahsa1b	1393.62258	0.303195094	0.11651768	2.60213807	0.00926445	0.02897062
ENSDARG00000075323	srfbp1	593.196722	-0.310528129	0.11933709	-2.60210907	0.00926524	0.02897062
ENSDARG00000090097	CR626886.1	28.7278332	-1.409005345	0.54153039	-2.60189526	0.00927102	0.02898505
ENSDARG00000001817	zmp:0000000521	114.597769	0.651222304	0.25031836	2.60157623	0.00927964	0.02900472
ENSDARG00000045565	noc4l	628.945623	-0.34542893	0.13277653	-2.60158132	0.00927951	0.02900472
ENSDARG00000096955	CR848791.2	4.94450134	3.096217536	1.19025644	2.6013029	0.00928704	0.02902419
ENSDARG00000017299	fabp11a	2456.07045	0.337079442	0.12960544	2.6008125	0.00930033	0.02906206
ENSDARG00000052497	zgc:110789	748.25191	-0.366611084	0.14097783	-2.60048757	0.00930914	0.02908594
ENSDARG00000077231	vwf	212.417588	0.579125519	0.22273274	2.60009156	0.00931989	0.02911587
ENSDARG00000103205	si:ch211-161f7.2	4.59654111	3.588585514	1.38049459	2.59949263	0.00933617	0.02916306
ENSDARG00000101776	syt12	633.647598	-0.350861851	0.13500477	-2.59888487	0.00935271	0.02921107
ENSDARG00000110206	CABZ01085887.1	90.7923518	-0.757925884	0.29164014	-2.59883941	0.00935395	0.02921127
ENSDARG00000088029	FP016180.1	3.05418748	-4.728299607	1.81951439	-2.59866019	0.00935884	0.02922285
ENSDARG00000007943	gabpb2a	723.639325	-0.366792223	0.14115431	-2.59851956	0.00936267	0.02922748
ENSDARG00000036102	ctdsp2	4633.53508	-0.311270694	0.11978578	-2.59856135	0.00936153	0.02922748
ENSDARG00000056267	dixdc1b	707.883309	-0.363005681	0.1397094	-2.59829107	0.0093689	0.02924326
ENSDARG00000045749	ppfibp1a	1293.46867	-0.27597577	0.10621808	-2.59819963	0.0093714	0.02924738
ENSDARG00000089844	scarb2c	720.435534	0.402344871	0.1548699	2.59795393	0.00937811	0.02926464
ENSDARG00000092938	znf1047	15.3532971	-1.630250468	0.62757822	-2.59768492	0.00938546	0.0292839
ENSDARG00000010144	plppr3a	2196.36415	-0.493025242	0.18983799	-2.59708419	0.00940189	0.0293278
ENSDARG00000079402	tapbp.1	63.7367216	0.847592184	0.32635857	2.59711946	0.00940092	0.0293278
ENSDARG00000068822	purba	3593.37584	-0.277866868	0.10702645	-2.59624488	0.00942489	0.02939585
ENSDARG00000077089	blm	437.233944	-0.51673925	0.19904834	-2.59604908	0.00943026	0.02940892
ENSDARG00000077029	bub1	1843.09543	-0.563992239	0.21728027	-2.59569013	0.00944012	0.02943597
ENSDARG00000100679	lrch3	858.437407	0.356456996	0.13735254	2.59519764	0.00945365	0.02947448
ENSDARG00000091783	si:dkeyp-71f10.5	88.4832523	0.648590179	0.249937	2.59501471	0.00945869	0.02948648
ENSDARG00000053668	stag2b	6718.92703	-0.264362475	0.10188299	-2.5947657	0.00946554	0.02950415
ENSDARG00000094578	epsti1	12.6839347	1.708440889	0.65845779	2.59460959	0.00946984	0.02951385
ENSDARG00000097162	CU861891.1	23.0232996	-1.47235921	0.56748149	-2.59455018	0.00947148	0.02951525
ENSDARG00000117288	LO017938.1	127.009853	-0.583591416	0.22494687	-2.59435227	0.00947693	0.02952854
ENSDARG00000045146	tomm22	1862.68704	-0.29587855	0.11408208	-2.59355852	0.00949884	0.02959308
ENSDARG00000056150	rbms2b	1036.01351	-0.328342807	0.12660878	-2.59336527	0.00950418	0.02960601
ENSDARG00000088198	CABZ01077220.1	5.34546951	-3.453655574	1.33193904	-2.59295318	0.00951557	0.02963779
ENSDARG00000014361	tmbim4	1679.18793	-0.315938872	0.12185109	-2.59282766	0.00951905	0.02964489
ENSDARG00000075989	arpc2	6724.55585	0.256756374	0.09903132	2.59267851	0.00952318	0.02965404
ENSDARG00000102855	neo1a	4421.87682	0.259345948	0.10004844	2.59220394	0.00953632	0.02968647
ENSDARG00000086842	dap1b	1774.50049	-0.324397467	0.12514706	-2.59213008	0.00953837	0.02968647
ENSDARG00000015228	ero1a	84.3817368	0.905294249	0.34924697	2.59213201	0.00953832	0.02968647
ENSDARG00000058939	b4galnt3b	679.252719	0.312045829	0.12037948	2.59218457	0.00953686	0.02968647
ENSDARG00000110283	BX296549.1	2.95892465	-5.193406131	2.00368963	-2.59192145	0.00954416	0.02970076
ENSDARG00000075454	hdc	287.353344	0.539853748	0.20832411	2.59141274	0.00955828	0.02974099
ENSDARG00000030278	idh3a	3272.13683	0.268482511	0.1036081	2.59132747	0.00956065	0.02974463
ENSDARG00000052905	zgc:165423	1282.12092	1.092104676	0.42146643	2.59120204	0.00956413	0.02975175
ENSDARG00000089230	si:ch211-253b8.2	506.152585	-0.335731277	0.12957972	-2.59092449	0.00957185	0.02977203
ENSDARG00000087187	grip2a	364.659854	-0.533834386	0.20604745	-2.59083231	0.00957441	0.02977628
ENSDARG00000045230	cox6b1	2242.12793	0.289866906	0.11188367	2.59078824	0.00957564	0.02977637
ENSDARG00000092963	BX321877.1	24.1322646	-1.498253257	0.57836195	-2.59051145	0.00958334	0.0297966
ENSDARG00000008832	neu1	865.815829	0.477725367	0.18441734	2.59045799	0.00958483	0.0297975
ENSDARG00000039915	bola1	433.492439	0.348095269	0.13437821	2.59041451	0.00958604	0.02979754
ENSDARG00000059680	fscn1a	8759.99324	-0.337725136	0.13039589	-2.58999826	0.00959764	0.02982986
ENSDARG00000101619	micos10	1403.19631	0.282493713	0.10907893	2.58981013	0.00960289	0.02984243
ENSDARG00000017571	mccc2	3614.95555	0.238469906	0.09208941	2.58954767	0.00961021	0.02986146
ENSDARG00000103194	BX936433.2	23.2659914	-1.224018275	0.47272265	-2.58929478	0.00961727	0.02987967
ENSDARG00000088835	efs	1296.03019	-0.291349123	0.11252762	-2.58913433	0.00962176	0.02988986
ENSDARG00000023228	vsnl1a	2535.21469	0.345801059	0.13357293	2.58885576	0.00962954	0.02991031
ENSDARG00000046142	asgrl1	52.1768902	-1.133565082	0.43787066	-2.58881263	0.00963075	0.02991031

Table S2. DEGs of WT vs. *terfa-/-*

ENSDARG00000111837	trhde.2	343.41914	-0.479706051	0.18531371	-2.58861604	0.00963625	0.02992365
ENSDARG00000061370	tsen34	375.78692	0.424715501	0.16407599	2.58852921	0.00963868	0.02992746
ENSDARG00000100242	dph5	656.376785	0.327885082	0.12669786	2.58792915	0.00965548	0.0299759
ENSDARG00000104278	slc2a8	230.853755	0.508232921	0.19639935	2.58775258	0.00966043	0.02998378
ENSDARG00000062712	tmem264	53.5866089	-1.072100235	0.41429526	-2.58776853	0.00965999	0.02998378
ENSDARG00000103594	npm3	1212.93904	-0.30328192	0.11720502	-2.5876188	0.00966419	0.02999168
ENSDARG00000035434	zmat5	238.880621	-0.488098598	0.18863289	-2.58755832	0.00966588	0.0299932
ENSDARG00000045391	cd226	30.4810046	-1.26166272	0.48760746	-2.58745575	0.00966876	0.02999839
ENSDARG00000100590	BX649421.1	12.6321584	1.749697092	0.67625037	2.58735102	0.0096717	0.03000376
ENSDARG00000023656	he1.1	974.144851	-1.548803932	0.59863009	-2.58724705	0.00967462	0.03000907
ENSDARG00000011837	C13H9orf72	540.546666	0.424757544	0.16420994	2.58667375	0.00969073	0.03005154
ENSDARG00000114210	CABZ01035356.1	152.71202	0.798613405	0.30873652	2.5867151	0.00968957	0.03005154
ENSDARG00000067626	ywhag1	1725.86464	-0.320573432	0.12394122	-2.58649565	0.00969574	0.03006332
ENSDARG00000019444	ssr4	5980.96239	0.293161656	0.11341059	2.58495848	0.00973907	0.03019392
ENSDARG00000042630	hebp2	1086.2886	0.251684061	0.09737615	2.58465821	0.00974756	0.03021646
ENSDARG00000113141	znf1018	21.0470614	-1.528448058	0.5913844	-2.58452552	0.00975131	0.03022388
ENSDARG00000035290	dmrt3a	149.433498	0.601662963	0.23279891	2.58447502	0.00975274	0.03022388
ENSDARG00000070868	cfap126	88.9890661	0.875177401	0.33863269	2.58444454	0.0097536	0.03022388
ENSDARG00000094603	si:dkey-21h14.12	3.35915615	4.188199584	1.62071979	2.5841602	0.00976164	0.03024503
ENSDARG00000053684	aldob	28778.068	0.382165231	0.14789294	2.58406673	0.00976429	0.03024946
ENSDARG00000063051	bap1	1142.62127	-0.314714987	0.12179411	-2.58399178	0.00976641	0.03025227
ENSDARG00000039052	klhl40a	276.508559	0.462590386	0.1790302	2.58386786	0.00976992	0.03025937
ENSDARG00000091967	ttf1	178.650138	-0.559697584	0.21663319	-2.58361876	0.00977698	0.03027745
ENSDARG00000053449	pyya	349.154355	0.387963384	0.15017958	2.58332976	0.00978518	0.03029528
ENSDARG00000105447	si:ch211-129p13.1	446.107365	-1.129862686	0.43736616	-2.58333359	0.00978507	0.03029528
ENSDARG00000111587	CABZ01103860.1	288.491581	0.760932193	0.29462769	2.58269067	0.00980332	0.03034767
ENSDARG00000040948	olig1	86.5564946	-0.948752104	0.36738348	-2.58245715	0.00980995	0.03036444
ENSDARG00000055338	CAPN1	120.458053	-0.590391734	0.2286259	-2.58234846	0.00981305	0.03037022
ENSDARG00000008255	cnot6a	3391.93299	-0.261555319	0.10128959	-2.58225277	0.00981577	0.03037486
ENSDARG00000060413	si:ch211-157b11.8	32.24358	-1.085342106	0.42031524	-2.58220976	0.00981699	0.03037486
ENSDARG00000075526	pwwp2a	1098.03721	-0.379785164	0.14708363	-2.58210365	0.00982001	0.03038042
ENSDARG00000079045	opn8b	120.453729	-0.611117177	0.23668895	-2.58194216	0.00982461	0.03039086
ENSDARG00000060638	CLSTN2	341.094975	-0.743551592	0.2879993	-2.58178266	0.00982915	0.03040112
ENSDARG00000014003	stard8	1084.36773	0.292710114	0.11339832	2.58125624	0.00984415	0.03044374
ENSDARG00000015445	lim2.4	2553.84692	-0.549860996	0.21302849	-2.58116175	0.00984684	0.03044828
ENSDARG00000039649	mtif3	339.061179	-0.415431892	0.16096127	-2.58094317	0.00985308	0.03046378
ENSDARG00000082753	NC_002333.17	1573.37836	0.633129067	0.24533307	2.58069192	0.00986025	0.03048216
ENSDARG00000055331	aldh9a1a.2	46.7527977	0.989019088	0.38333751	2.58002169	0.00987941	0.03053379
ENSDARG00000073765	gna13a	317.723717	-0.430420961	0.16682701	-2.58004357	0.00987879	0.03053379
ENSDARG00000018061	neil1	284.401171	-0.441262499	0.17103557	-2.57994583	0.00988158	0.0305367
ENSDARG00000003641	tfg	3982.68916	0.270856292	0.10500272	2.57951703	0.00989386	0.03057084
ENSDARG00000003144	pxmp2	137.302315	0.604348962	0.23430496	2.57932634	0.00989932	0.03058392
ENSDARG00000078986	mplkip	493.503159	-0.315724796	0.12241907	-2.57904912	0.00990727	0.03059707
ENSDARG00000020387	nipsnap3a	516.697338	0.461673437	0.17900335	2.57913297	0.00990487	0.03059707
ENSDARG00000063007	apc2	4340.8569	-0.455264624	0.17652254	-2.57907357	0.00990657	0.03059707
ENSDARG00000006292	maats1	79.9990228	0.835996106	0.32415885	2.57897048	0.00990953	0.03060023
ENSDARG00000017272	eapp	437.092537	-0.413809059	0.16052197	-2.57789667	0.00994037	0.03069144
ENSDARG00000071445	myoz1b	7141.31886	0.384718743	0.14923979	2.57785635	0.00994153	0.03069144
ENSDARG00000063437	wnt9a	233.627795	0.569627651	0.22097418	2.57780186	0.0099431	0.03069247
ENSDARG00000076104	sema4bb	806.856601	-0.34854033	0.13521798	-2.57761828	0.00994838	0.03070496
ENSDARG00000105525	BX571680.1	458.152984	-0.456086581	0.17696685	-2.57724301	0.00995919	0.0307345
ENSDARG00000069388	tmem88b	30.2249029	1.11454831	0.43247721	2.57712613	0.00996256	0.03074108
ENSDARG00000034605	zgc:153169	175.356003	0.533035723	0.20687504	2.57660722	0.00997753	0.03078344
ENSDARG00000070939	znf740a	1188.84549	-0.313187688	0.12155574	-2.57649443	0.00998078	0.03078966
ENSDARG00000098522	BX649431.1	17.5761686	-1.667002745	0.64708315	-2.5761801	0.00998986	0.03081385
ENSDARG00000099465	rnf213a	1423.22447	0.409255671	0.1588646	2.57612877	0.00999134	0.0308146
ENSDARG00000017853	prepl	215.260093	0.547261185	0.21249463	2.57541179	0.01001208	0.03087279
ENSDARG00000102600	CABZ01003344.1	12.2013293	2.040312406	0.79223417	2.57539058	0.0100127	0.03087279

Table S2. DEGs of WT vs. *terfa-/-*

ENSDARG00000000568	ell	1170.2455	0.291605378	0.113233	2.57526854	0.01001623	0.03087985
ENSDARG00000103443	vwde	1288.8981	-0.368973095	0.1432825	-2.57514421	0.01001983	0.03088712
ENSDARG00000091101	fabp10b	154.981239	0.634834709	0.24654272	2.57494813	0.01002551	0.03090081
ENSDARG00000060393	fibcd1	391.810347	-0.436728261	0.16961489	-2.57482261	0.01002915	0.03090819
ENSDARG00000098239	zgc:85932	2272.07209	0.40924324	0.15898213	2.57414616	0.01004878	0.03096485
ENSDARG00000028815	zgc:162945	206.622049	-0.502834868	0.19536369	-2.5738399	0.01005768	0.03098843
ENSDARG00000100313	pip5k1cb	1199.75383	-0.476505844	0.18514925	-2.57363099	0.01006375	0.0310033
ENSDARG00000016490	rab11fip3	1604.96854	-0.338905291	0.13169778	-2.57335611	0.01007175	0.03102409
ENSDARG00000014091	osr1	638.806549	-0.343479783	0.13348628	-2.57314681	0.01007785	0.03103901
ENSDARG00000098968	RSBN1	734.412853	-0.369511079	0.14360599	-2.57308954	0.01007951	0.0310403
ENSDARG00000117336	CU855948.1	14.8667258	-1.673273438	0.65040937	-2.57264658	0.01009242	0.0310762
ENSDARG00000077785	atf5b	1216.8828	0.295969636	0.11506809	2.57212616	0.01010761	0.0311191
ENSDARG00000104795	cxcl8a	77.6377031	1.192096936	0.46347643	2.57207674	0.01010905	0.03111969
ENSDARG00000012135	fbxl2	597.000572	-0.313039183	0.12171375	-2.57192946	0.01011335	0.03112908
ENSDARG00000097966	BX936420.1	12.5539964	-1.70446069	0.66273796	-2.57184709	0.01011576	0.03113181
ENSDARG00000090386	cd3eap	212.888179	-0.532520834	0.20706045	-2.57181333	0.01011674	0.03113181
ENSDARG00000098884	BX649355.1	10.2877394	-2.014794225	0.78348587	-2.57157699	0.01012365	0.03114921
ENSDARG00000040713	rbbp9	627.536656	0.310992181	0.1209431	2.57139262	0.01012904	0.03116194
ENSDARG00000075172	fbxo25	726.755247	-0.304592619	0.11845903	-2.57129082	0.01013202	0.03116725
ENSDARG00000053474	tdrkh	695.450631	-0.268182507	0.10430179	-2.57121671	0.01013419	0.03117006
ENSDARG00000027649	cpsf3	2225.24886	-0.290153832	0.11290179	-2.56996663	0.01017083	0.03127889
ENSDARG00000113771	GRIK2	649.62334	-0.437785311	0.17037641	-2.56951842	0.010184	0.03131551
ENSDARG00000020231	mapre3a	540.806387	-0.415067257	0.16154966	-2.56928582	0.01019084	0.03133266
ENSDARG00000052074	si:ch211-122f10.4	478.292595	0.387111608	0.1506803	2.5690924	0.01019653	0.03134628
ENSDARG00000099650	CABZ01079663.1	461.569873	-0.516598441	0.20113317	-2.56843984	0.01021574	0.03140148
ENSDARG00000099046	si:ch211-227p7.5	29.0201719	1.307419607	0.50905703	2.56831656	0.01021938	0.03140488
ENSDARG00000102257	soul3	1418.47301	0.261134677	0.10167427	2.56834565	0.01021852	0.03140488
ENSDARG00000043394	si:dkey-71h2.2	392.6517	-0.412928682	0.16078517	-2.56820124	0.01022278	0.03141144
ENSDARG00000100229	si:ch211-238e22.5	15.8372113	-1.560975009	0.60784567	-2.56804494	0.01022739	0.03142173
ENSDARG00000074552	ndufs7	3971.21179	0.30984197	0.12066169	2.56785703	0.01023294	0.03143488
ENSDARG00000071878	trmt112	543.865626	-0.326057003	0.12698015	-2.56777941	0.01023523	0.03143803
ENSDARG00000090982	slc44a1b	1550.45454	-0.293527532	0.1143533	-2.56684805	0.01026276	0.0315187
ENSDARG00000044016	myo6a	3440.03351	0.253022101	0.09857779	2.56672524	0.01026639	0.03152597
ENSDARG00000059581	mbd5	1497.8989	-0.355353349	0.13845532	-2.56655618	0.0102714	0.03153671
ENSDARG00000054778	pogza	647.375329	-0.346294634	0.13492763	-2.56652139	0.01027243	0.03153671
ENSDARG00000069329	drgx	172.855877	-0.533525971	0.20790669	-2.56617994	0.01028255	0.03156388
ENSDARG00000017165	slc3a1	285.703863	-0.560963312	0.21861688	-2.56596526	0.01028891	0.03157952
ENSDARG00000103025	hmgcs1	2056.86841	-0.426974348	0.16641896	-2.56565927	0.01029799	0.03160349
ENSDARG00000103473	BX537137.3	9.83284546	2.212600657	0.86248021	2.56539295	0.0103059	0.03162385
ENSDARG00000094908	lingo4a	58.2798121	-1.16588115	0.45455832	-2.56486593	0.01032157	0.03166801
ENSDARG00000101135	si:dkey-85k7.7	1041.08809	0.66914381	0.26090618	2.56469127	0.01032676	0.03168005
ENSDARG00000013168	jag1b	1791.46508	0.28670648	0.11179817	2.56450056	0.01033244	0.03169355
ENSDARG00000102674	ckap5	7815.16221	-0.330253419	0.12882453	-2.5635912	0.01035955	0.03177278
ENSDARG00000040255	ephx2	1079.90874	0.396321092	0.15460028	2.56352119	0.01036164	0.03177526
ENSDARG00000001898	manea	450.186935	-0.395241799	0.15419765	-2.56321554	0.01037076	0.03179933
ENSDARG00000031796	ache	4413.43656	0.298115453	0.11631813	2.56293196	0.01037924	0.03181982
ENSDARG00000002006	rxrbb	868.570904	-0.286964277	0.11196831	-2.56290622	0.01038001	0.03181982
ENSDARG00000006225	ddx39aa	5575.41766	-0.368570209	0.14381763	-2.56276091	0.01038435	0.03182921
ENSDARG00000074298	znf1015	533.483591	-0.308995216	0.12058345	-2.56250095	0.01039213	0.03184912
ENSDARG00000075382	slc9a6b	680.977499	-0.414014092	0.16157669	-2.56233797	0.01039701	0.03186015
ENSDARG00000100232	glis2b	541.802795	0.421890509	0.16465447	2.56227789	0.01039881	0.03186173
ENSDARG00000004078	acsl4a	2801.65349	-0.311388818	0.12156603	-2.56147899	0.01042276	0.03192723
ENSDARG00000102889	15-Sep	3906.64698	-0.290655948	0.1134717	-2.56148409	0.0104226	0.03192723
ENSDARG00000075334	arhgap32a	872.957802	-0.352881974	0.13779338	-2.5609502	0.01043863	0.03197193
ENSDARG00000104850	si:ch211-215p11.1	128.277107	-0.626951248	0.24482932	-2.56076868	0.01044409	0.0319847
ENSDARG00000079848	gmps	2987.32145	0.324415447	0.12669079	2.560687	0.01044654	0.03198827
ENSDARG00000101072	cnot6b	588.894997	0.370873874	0.14486107	2.56020391	0.01046108	0.03202883
ENSDARG00000022795	golt1ba	111.12194	0.806069321	0.31485464	2.56013164	0.01046325	0.03203155

Table S2. DEGs of WT vs. *terfa-/-*

ENSDARG00000109649	BX571720.2	7.08878179	-4.299986129	1.67964503	-2.56005647	0.01046552	0.03203453
ENSDARG00000040179	zgc:101562	81.1544195	0.815067595	0.3184083	2.55981896	0.01047267	0.03204854
ENSDARG00000058746	ubap1	1175.31971	0.296642787	0.1158825	2.55985833	0.01047148	0.03204854
ENSDARG00000078387	si:dkey-92i15.4	116.386097	0.730654162	0.28544863	2.55966954	0.01047717	0.03204968
ENSDARG00000074680	rims1a	1605.45948	-0.354481508	0.13848906	-2.55963546	0.0104782	0.03204968
ENSDARG00000044695	ccdc80l2	854.74436	0.289567647	0.11312455	2.55972419	0.01047553	0.03204968
ENSDARG00000100621	CABZ01080550.1	18.5446137	-1.865701393	0.72887675	-2.55969394	0.01047644	0.03204968
ENSDARG00000063684	ppm1h	836.170532	-0.450761919	0.17611135	-2.55952795	0.01048144	0.03205565
ENSDARG00000029747	mep1a.1	34.0494924	1.263584682	0.49374404	2.55918974	0.01049165	0.03208291
ENSDARG00000105241	btr26	10.827085	1.830764118	0.71550743	2.55869337	0.01050664	0.0321248
ENSDARG00000001734	fkbp8	835.690654	-0.329839339	0.12892118	-2.55845734	0.01051377	0.03214266
ENSDARG00000105389	mmel1	329.128053	0.740839033	0.2895761	2.55835696	0.01051681	0.03214798
ENSDARG00000109937	CR356242.1	4.2502838	3.824573137	1.49500995	2.55822588	0.01052077	0.03215615
ENSDARG00000056986	dact2	352.676061	0.463627062	0.18124195	2.55805605	0.01052591	0.03216395
ENSDARG00000098865	BX548044.7	5.84352507	-3.854187154	1.50666992	-2.5580833	0.01052509	0.03216395
ENSDARG00000010655	ppm1k	1284.63235	-0.290479617	0.11355766	-2.55799239	0.01052784	0.03216588
ENSDARG00000012269	clcn1b	250.074803	0.41877767	0.1637353	2.55765051	0.01053819	0.03219356
ENSDARG00000013649	casr	3.35056448	-4.807628303	1.87995967	-2.55730395	0.0105487	0.0322217
ENSDARG00000012860	tmprss4b	37.7626315	1.009757289	0.39488182	2.55711265	0.0105545	0.03223546
ENSDARG00000100257	zgc:174314	74.6310923	-0.657630331	0.25719906	-2.55689247	0.01056119	0.03225191
ENSDARG00000008697	epas1a	5384.44516	0.354084972	0.13849543	2.55665449	0.01056841	0.03227001
ENSDARG00000101629	CABZ01079192.1	654.207125	1.402327204	0.54855784	2.55638895	0.01057648	0.03229069
ENSDARG00000013379	ppp6r3	4135.47635	-0.236808504	0.09264655	-2.55604238	0.01058702	0.0323189
ENSDARG00000008131	sox1b	733.328421	-0.525738345	0.20572364	-2.5555563	0.01060182	0.0323601
ENSDARG00000100821	scrt1a	826.23095	-0.617017702	0.24147418	-2.55521194	0.01061232	0.03238815
ENSDARG00000094159	BX072561.2	12.1866753	2.713032275	1.06187241	2.55495129	0.01062027	0.03240844
ENSDARG00000014015	pdia7	3037.34689	0.264024829	0.10335355	2.55457922	0.01063162	0.03243911
ENSDARG00000070449	tspan5b	1024.53504	-0.363794641	0.14242257	-2.55433286	0.01063915	0.03245809
ENSDARG00000059123	plcd1a	1350.1355	0.375244075	0.14690778	2.55428321	0.01064067	0.03245874
ENSDARG00000010482	prg4a	74.4086527	0.890525278	0.34877266	2.55331157	0.0106704	0.03254544
ENSDARG00000020771	tnr	1769.33593	-0.433593693	0.16986304	-2.55260765	0.01069199	0.03260592
ENSDARG00000077745	si:ch73-380l3.2	33.1152097	1.155176049	0.45255245	2.5525794	0.01069285	0.03260592
ENSDARG00000078383	mcrip1	528.789231	-0.3593486	0.14078896	-2.55239193	0.01069861	0.03261947
ENSDARG00000005549	znf710b	1527.57679	-0.323622151	0.12680883	-2.5520474	0.0107092	0.03264774
ENSDARG00000073731	dok4	730.316636	-0.356821474	0.13982889	-2.55184378	0.01071546	0.03266268
ENSDARG00000009764	srek1	561.77724	-0.341500196	0.13382704	-2.55180258	0.01071672	0.03266268
ENSDARG00000077215	eif4g3a	2803.50717	0.331361852	0.12987544	2.55138203	0.01072966	0.03269811
ENSDARG00000101861	cyp2k19	281.755882	-0.701060231	0.27483817	-2.55081097	0.01074726	0.03274371
ENSDARG00000076997	stxbp4	98.4824213	-0.674206397	0.26430683	-2.55084746	0.01074614	0.03274371
ENSDARG00000068036	tmem119b	684.176271	-0.344428457	0.13503785	-2.55060688	0.01075355	0.03275887
ENSDARG00000036059	tmppe	367.382627	-0.366931939	0.14388225	-2.55022383	0.01076538	0.03279087
ENSDARG00000087709	fat3b	369.899222	-0.543244219	0.21306312	-2.5496868	0.01078197	0.03283739
ENSDARG00000103900	sept9b	753.304904	-0.325079301	0.12751424	-2.54935687	0.01079218	0.03286445
ENSDARG00000036412	si:ch211-81a5.5	42.3210496	1.087164962	0.42648708	2.54911584	0.01079964	0.03288314
ENSDARG00000023989	pef1	983.797041	0.363492195	0.14265639	2.54802602	0.01083344	0.03298201
ENSDARG00000070078	abcb11b	421.600037	-1.218950146	0.47846373	-2.54763333	0.01084564	0.03301511
ENSDARG00000103318	mrpl3	1136.74109	-0.361250584	0.1418225	-2.54720217	0.01085905	0.03305189
ENSDARG00000055313	slc12a10.1	42.5858382	-1.019894522	0.40043012	-2.54699754	0.01086542	0.03306722
ENSDARG00000098201	mtmr3	458.307518	0.385044118	0.15118972	2.54676133	0.01087278	0.03308556
ENSDARG00000055132	lrfn4a	513.828236	-0.382884782	0.1503527	-2.54657737	0.01087851	0.03309834
ENSDARG00000012986	adal	140.592167	-0.566600676	0.22249815	-2.54654111	0.01087964	0.03309834
ENSDARG00000035821	si:ch211-173p18.3	3185.20395	-0.221747523	0.08708742	-2.5462636	0.01088829	0.03312061
ENSDARG00000060434	map1b	2897.80057	-0.273295392	0.10733936	-2.54608751	0.01089379	0.03313327
ENSDARG00000101145	lipeb	530.530689	0.453567426	0.17815987	2.54584514	0.01090135	0.03315223
ENSDARG00000052387	pou2f3	303.90668	-0.38621785	0.15171658	-2.54565355	0.01090734	0.03316637
ENSDARG00000041450	rab11a	8409.78332	0.221893834	0.08717251	2.54545642	0.0109135	0.03318104
ENSDARG00000088981	tmem220	68.5681	0.790401105	0.31052143	2.54539955	0.01091528	0.03318239
ENSDARG00000055857	dop1b	2240.67118	0.260000359	0.10215299	2.54520548	0.01092135	0.03319678

Table S2. DEGs of WT vs. *terfa-/-*

ENSDARG00000056228	pld1a	714.532728	0.343299148	0.13489339	2.54496636	0.01092883	0.03320733
ENSDARG00000062310	wdr26a	1415.15139	-0.289144522	0.11361281	-2.54499937	0.01092779	0.03320733
ENSDARG00000103551	rnf213b	22.5548609	1.173079685	0.4609318	2.54501791	0.01092721	0.03320733
ENSDARG00000073917	b3glcta	367.983693	-0.382999781	0.15049965	-2.54485496	0.01093231	0.03321387
ENSDARG00000100862	gprc5c	419.597378	0.426686438	0.16767941	2.54465615	0.01093854	0.03322872
ENSDARG00000078284	phkb	3226.01636	0.250477169	0.09843799	2.5445173	0.01094289	0.03323787
ENSDARG00000101234	nsmfb	795.765955	-0.360154598	0.14155264	-2.54431566	0.01094921	0.03324894
ENSDARG00000089917	sh3tc2	1572.23471	-0.269999711	0.10611733	-2.54435072	0.01094811	0.03324894
ENSDARG00000097884	CU928013.1	281.786331	-0.439860784	0.17290428	-2.54395548	0.01096051	0.03327918
ENSDARG00000035569	cyp1d1	470.496042	0.460902421	0.18121926	2.54334129	0.01097979	0.03333367
ENSDARG00000101213	CU278559.1	7.1348812	2.395215404	0.94187334	2.54303344	0.01098947	0.03335898
ENSDARG00000010010	trim13	874.397569	-0.497841184	0.19579266	-2.54269586	0.01100009	0.03338714
ENSDARG00000054907	cox7a2l	1902.85837	0.299882551	0.11795146	2.5424235	0.01100867	0.0334091
ENSDARG00000093943	BX294181.2	3.55212658	-4.762481963	1.87341581	-2.54213824	0.01101766	0.03343131
ENSDARG00000086618	psma3	3786.66245	-0.299224755	0.11770786	-2.54209675	0.01101897	0.03343131
ENSDARG00000070141	si:ch211-191i18.2	164.872975	-0.572387325	0.22516645	-2.54206314	0.01102003	0.03343131
ENSDARG00000012505	u2af2a	2203.81435	-0.324833055	0.12778795	-2.54196931	0.01102299	0.03343621
ENSDARG00000076251	irf3	55.9441972	1.061968452	0.4178329	2.54161045	0.01103431	0.03346647
ENSDARG00000101528	si:ch211-194h1.2	21.9759454	-1.417089831	0.55769989	-2.5409541	0.01105504	0.03352527
ENSDARG00000060322	zgc:153654	15.115074	1.871000466	0.73654787	2.54022929	0.01107798	0.03359073
ENSDARG00000003008	prkcea	1465.94104	-0.263078259	0.10356768	-2.54015795	0.01108024	0.03359349
ENSDARG00000105718	CABZ01068273.1	30.6568567	1.274084023	0.50162203	2.53992838	0.01108752	0.03361145
ENSDARG00000055426	rwdd2b	269.99953	0.407651168	0.16050214	2.53984878	0.01109004	0.033615
ENSDARG00000111321	CR759968.2	7.00103862	-2.716046744	1.06939353	-2.53980098	0.01109156	0.03361549
ENSDARG00000015930	rwdd1	1316.76628	0.317737358	0.12510844	2.5396957	0.0110949	0.03362151
ENSDARG00000009018	rhbg	10492.435	0.443445646	0.17462102	2.53947462	0.01110191	0.03363867
ENSDARG00000060457	pmp22b	6442.22471	0.350954611	0.13823122	2.53889549	0.0111203	0.03368273
ENSDARG00000114589	jam3a	720.042798	-0.36422231	0.14345737	-2.53888879	0.01112052	0.03368273
ENSDARG00000026701	gtf2h1	582.093376	-0.320866087	0.12637795	-2.53894038	0.01111888	0.03368273
ENSDARG00000053708	ghrhrb	36.9425047	-1.075430265	0.42366003	-2.53842747	0.01113519	0.03372305
ENSDARG00000060766	pxnb	679.22226	0.521206072	0.20533756	2.53828902	0.0111396	0.03373229
ENSDARG00000055715	capn8	1040.15874	-0.269215651	0.10607913	-2.53787567	0.01115276	0.03376804
ENSDARG00000010059	itpkb	456.135611	0.433238087	0.17071647	2.53776383	0.01115632	0.03377472
ENSDARG00000026248	slc1a7b	128.894132	-1.077738396	0.42469544	-2.53767357	0.0111592	0.03377932
ENSDARG00000110917	si:ch211-223a21.6	18.9034309	-1.385091349	0.54586231	-2.53743723	0.01116674	0.03379802
ENSDARG00000004171	rabac1	1196.97789	0.324718403	0.12798536	2.53715276	0.01117582	0.03381739
ENSDARG00000074686	oga	7326.18622	-0.256225154	0.10098931	-2.53715134	0.01117586	0.03381739
ENSDARG00000090965	ripk3	68.1628124	0.757275468	0.2985314	2.53666937	0.01119126	0.03385986
ENSDARG00000102125	iqsec2a	1171.19904	-0.380418003	0.14997123	-2.53660661	0.01119327	0.03386181
ENSDARG00000095132	si:ch211-227e10.6	10.0673953	2.330378969	0.91875651	2.53644894	0.01119831	0.03386882
ENSDARG00000102199	SIKE1	817.141498	-0.30788283	0.12138204	-2.53647773	0.01119739	0.03386882
ENSDARG00000002479	ercc6l	1008.05406	-0.386823576	0.1525398	-2.53588626	0.01121632	0.03391915
ENSDARG00000035535	rasa1a	2174.74363	0.283640765	0.11185791	2.53572379	0.01122152	0.03393077
ENSDARG00000054814	ptp4a3b	1970.52252	-0.312950921	0.12342505	-2.53555438	0.01122695	0.03394305
ENSDARG00000103650	si:ch73-329n5.1	39.8059066	1.51573933	0.59781804	2.53545266	0.01123021	0.03394878
ENSDARG00000092546	pdap1a	3491.59744	-0.254986244	0.10059795	-2.53470613	0.01125417	0.03401707
ENSDARG00000077372	tfr1b	2010.25926	-0.362532586	0.14303174	-2.53463027	0.01125661	0.0340203
ENSDARG00000117262	CT030017.3	108.700592	0.666998774	0.26317695	2.53441182	0.01126363	0.03403738
ENSDARG00000102453	slc1a2b	22311.0238	-0.401920927	0.15860589	-2.53408572	0.01127412	0.03406493
ENSDARG00000003068	gorasp1a	1347.24945	-0.275268824	0.10863987	-2.53377343	0.01128417	0.03409116
ENSDARG00000033285	gsto2	741.622253	0.475246607	0.18757167	2.53368013	0.01128717	0.03409609
ENSDARG00000095007	sinhcafl	1390.00679	-0.386013286	0.15238294	-2.5331792	0.01130332	0.03414071
ENSDARG00000103031	znf1029	32.5062446	-0.998444289	0.39416641	-2.53305273	0.01130739	0.03414888
ENSDARG00000020847	atp6v0a1a	3039.56877	-0.243701723	0.09621053	-2.5330046	0.01130895	0.03414942
ENSDARG00000016360	kpna1	2525.7211	0.238955942	0.09433877	2.53295587	0.01131052	0.03415002
ENSDARG00000111979	CR394552.1	50.9709041	0.899763807	0.35527544	2.53258094	0.01132262	0.03418241
ENSDARG00000104243	eomesb	29.7516331	-1.390685643	0.54915136	-2.53242686	0.0113276	0.03419328
ENSDARG00000061732	raph1b	1827.03379	-0.280538345	0.11078338	-2.53231448	0.01133123	0.03420009

Table S2. DEGs of WT vs. *terfa-/-*

ENSDARG00000045842	zgc:113263	875.456977	0.25973348	0.10258998	2.53176277	0.01134908	0.03424865
ENSDARG00000019924	cmpk	3621.60004	0.249477502	0.09854026	2.53173179	0.01135008	0.03424865
ENSDARG00000060222	scn1ba	758.94359	-0.302851963	0.11962623	-2.53165175	0.01135267	0.03425232
ENSDARG00000044751	ddt	1253.28972	-0.321455775	0.12697883	-2.53156984	0.01135532	0.03425616
ENSDARG00000114184	si:busm1-105l16.2	12.6791712	2.309270645	0.91236505	2.53108187	0.01137113	0.03429969
ENSDARG00000033516	efr3ba	750.506487	-0.427161307	0.16878307	-2.53083037	0.01137929	0.03432013
ENSDARG00000092477	BX284666.1	12.3832327	1.936392553	0.76513785	2.53077605	0.01138105	0.03432128
ENSDARG00000026762	fam126a	1097.53665	-0.278192566	0.10993026	-2.53062776	0.01138586	0.03433162
ENSDARG00000054222	fbxl8	135.31993	0.676865698	0.26755485	2.52982034	0.01141209	0.03440655
ENSDARG00000019834	edrf1	491.678982	0.310900963	0.12291603	2.52937695	0.01142652	0.03444588
ENSDARG00000036987	has2	276.654448	0.535871389	0.21188737	2.5290388	0.01143754	0.03447072
ENSDARG00000105185	snx7	607.265979	0.335974125	0.13284445	2.52907915	0.01143622	0.03447072
ENSDARG00000102335	ano1	462.556412	-0.357930799	0.14153789	-2.52886919	0.01144307	0.0344832
ENSDARG00000040200	BX465228.1	4.8962771	4.970100808	1.96583653	2.52823708	0.01146369	0.03454116
ENSDARG00000076826	dpp6a	728.97572	-0.397242343	0.15713576	-2.52801997	0.01147078	0.03455834
ENSDARG00000102449	CABZ01048402.1	116.028207	-1.845398354	0.73006689	-2.52771133	0.01148087	0.03458454
ENSDARG00000091918	BX548073.1	13.1895421	2.041677958	0.80789457	2.52715891	0.01149895	0.03463479
ENSDARG00000091912	si:ch211-15j1.5	6.19255248	2.913382122	1.15289929	2.52700488	0.01150399	0.03464556
ENSDARG00000068428	si:ch211-153j24.3	156.274012	0.603908784	0.23898585	2.52696461	0.01150531	0.03464556
ENSDARG00000103887	carmil1	257.815657	0.467342818	0.18495436	2.52680079	0.01151068	0.03465753
ENSDARG00000076836	gpx4b	5454.07047	0.228371728	0.09040723	2.52603398	0.01153583	0.03472906
ENSDARG00000028306	prph	1212.9896	0.264565668	0.10474477	2.52581279	0.0115431	0.03474672
ENSDARG00000105495	si:dkey-31g16.2	8.39156618	-2.301541177	0.91123121	-2.52574885	0.0115452	0.03474884
ENSDARG00000037639	nkx3-2	513.288677	-0.347348546	0.13754374	-2.52536786	0.01155772	0.03478233
ENSDARG00000036222	npy	78.7821687	-0.825666184	0.32697533	-2.52516356	0.01156445	0.03479834
ENSDARG00000075132	fh	6564.01868	0.399324082	0.15814559	2.52504091	0.01156848	0.03480627
ENSDARG00000116043	CABZ01049625.1	29.3629241	-3.945333701	1.56254197	-2.52494574	0.01157162	0.03481149
ENSDARG00000114991	tmem14cb	110.679551	-0.744500659	0.29489576	-2.52462312	0.01158224	0.03483924
ENSDARG00000083622	dre-mir-124-6	42.7393861	-0.963029657	0.3814645	-2.52455909	0.01158435	0.03484137
ENSDARG00000070314	cald1a	2341.16376	0.31520977	0.12486631	2.52437799	0.01159032	0.03485511
ENSDARG00000058219	tubd1	119.851618	0.635646255	0.25181281	2.5242809	0.01159353	0.0348563
ENSDARG00000043006	gnav1	295.615319	0.450259907	0.17836915	2.52431494	0.0115924	0.0348563
ENSDARG00000008186	syk	305.292344	-0.392539507	0.15552493	-2.52396526	0.01160394	0.0348834
ENSDARG00000104973	wnt5a	75.2604075	0.768043211	0.30430757	2.52390436	0.01160595	0.03488522
ENSDARG00000117613	CABZ01030033.1	24.9437054	-1.328748611	0.52648738	-2.52379956	0.01160941	0.0348914
ENSDARG00000041182	rpl4	68670.6686	0.235756209	0.09341497	2.52375193	0.01161098	0.03489191
ENSDARG00000109211	znf979	32.6617497	-1.164200341	0.46137282	-2.52333965	0.01162461	0.03492454
ENSDARG00000002659	mapre1b	6367.67705	-0.2649832	0.10501295	-2.52333825	0.01162465	0.03492454
ENSDARG00000055043	depdc7a	226.368788	-0.456291584	0.18084836	-2.5230618	0.0116338	0.03494779
ENSDARG00000075727	map1lc3cl	144.774872	-0.656862522	0.26037706	-2.52273573	0.01164459	0.03497598
ENSDARG00000091937	BX927308.1	406.402152	-0.475510118	0.18850229	-2.52256949	0.01165009	0.03498829
ENSDARG00000043555	tmem30ab	1483.51385	-0.242621321	0.09618345	-2.52248519	0.01165289	0.03499245
ENSDARG00000075805	si:dkey-219c3.2	585.984173	-0.309167746	0.1225716	-2.52234406	0.01165756	0.03500226
ENSDARG00000100673	znf974	19.2058655	-1.432333479	0.56790576	-2.52213233	0.01166458	0.03501911
ENSDARG00000100190	si:ch211-188p14.4	675.618398	0.64881425	0.25726572	2.52196155	0.01167025	0.03503188
ENSDARG00000013721	g6pca.2	3979.63211	0.500228981	0.19837567	2.5216246	0.01168143	0.03506122
ENSDARG00000059824	soat2	359.711556	-0.421372749	0.16716586	-2.5206867	0.01171261	0.03514896
ENSDARG00000030641	letmd1	310.368627	0.428865642	0.1701402	2.52066022	0.01171349	0.03514896
ENSDARG00000103181	si:dkey-34d22.1	569.884005	0.415145343	0.16472769	2.52019166	0.0117291	0.03519153
ENSDARG00000116216	znf1046	23.0668555	-1.271902947	0.50470906	-2.52007158	0.0117331	0.03519799
ENSDARG00000104283	cdc42bpaa	965.529205	0.529605387	0.21015736	2.5200421	0.01173408	0.03519799
ENSDARG00000017799	tgm1	247.582197	0.519517677	0.20616331	2.51993271	0.01173773	0.03520468
ENSDARG00000100822	mapk15	16.7971955	1.478551286	0.58677902	2.51977533	0.01174298	0.03521618
ENSDARG00000096858	BX927361.2	5.06546775	-4.51758853	1.79298047	-2.51959717	0.01174892	0.03522975
ENSDARG00000038010	rac2	1121.52534	0.263721003	0.10467048	2.51953555	0.01175098	0.03523167
ENSDARG00000060723	stim1a	2092.48565	0.205331219	0.08150217	2.51933452	0.01175769	0.03524754
ENSDARG00000069327	nkx3.3	210.358236	-0.439747324	0.17459642	-2.51865032	0.01178056	0.03531184
ENSDARG00000041257	smtnl1	201.474522	0.491342471	0.19508731	2.51857732	0.011783	0.0353149

Table S2. DEGs of WT vs. *terfa-/-*

ENSDARG00000019990	cabp1a	201.363635	-0.615282684	0.24433292	-2.51821443	0.01179515	0.03534705
ENSDARG00000062460	SGK3	691.710974	0.355799613	0.14129389	2.51815285	0.01179721	0.03534897
ENSDARG00000071578	si:ch211-222k6.3	255.470756	-0.44513838	0.17679771	-2.51778365	0.01180958	0.03538177
ENSDARG00000096562	wu:fj29h11	1105.60354	-0.395138504	0.15696282	-2.51740192	0.01182239	0.03541587
ENSDARG00000088225	STRADB	609.085842	0.292593222	0.11624572	2.51702371	0.01183509	0.03544964
ENSDARG00000010432	eaf2	725.330626	0.407019507	0.16171241	2.51693424	0.01183809	0.03545071
ENSDARG00000100474	LO017721.1	7.7396666	3.027173433	1.20272541	2.51692814	0.0118383	0.03545071
ENSDARG00000077560	grin2cb	237.987604	-0.600914138	0.23876308	-2.51677998	0.01184328	0.03546135
ENSDARG00000094529	BX548157.1	21.9776851	-1.194099426	0.47451495	-2.51646322	0.01185393	0.03548896
ENSDARG00000095167	CR450836.1	100.096433	-0.838363199	0.33315896	-2.51640598	0.01185585	0.03549045
ENSDARG00000103825	galnt10	535.746232	0.413148051	0.16420877	2.51599262	0.01186977	0.03552782
ENSDARG00000035630	ypel1	1036.90963	0.318999311	0.12680551	2.51565821	0.01188103	0.03555727
ENSDARG00000117531	BX649471.3	2.50761734	-4.843227038	1.92558098	-2.51520299	0.01189639	0.03559893
ENSDARG00000099913	snrnp35	198.734646	0.475927564	0.18922484	2.51514312	0.01189841	0.03560069
ENSDARG00000044073	fuca2	2823.16049	0.288213648	0.11459377	2.51508999	0.0119002	0.03560177
ENSDARG00000100772	si:ch211-154c21.1	110.889685	0.656763722	0.26115952	2.51479912	0.01191002	0.03562388
ENSDARG00000060926	rnf19b	491.884949	0.439754152	0.17486741	2.51478622	0.01191046	0.03562388
ENSDARG00000043157	dnajb13	41.6945141	0.943455082	0.37519311	2.51458531	0.01191725	0.0356399
ENSDARG00000008235	cog5	772.518549	0.309166813	0.12296683	2.51422944	0.01192928	0.03567159
ENSDARG00000086391	cald1b	290.944006	-0.575400583	0.22890102	-2.51375277	0.01194541	0.03571554
ENSDARG00000022810	fzr1b	1216.84529	-0.313939384	0.12490724	-2.51338018	0.01195804	0.03574822
ENSDARG00000079306	rlim	1970.76102	-0.23495789	0.09348413	-2.51334534	0.01195922	0.03574822
ENSDARG00000027825	naa50	1736.77172	0.279910026	0.11139927	2.51267386	0.01198201	0.03581202
ENSDARG00000030106	stmn4	1804.41886	0.414753432	0.16510655	2.51203502	0.01200372	0.0358726
ENSDARG00000008403	phospho1	483.408949	0.453781684	0.18064642	2.51198822	0.01200531	0.03587304
ENSDARG00000057620	nitr3b	4.21845609	3.53694408	1.40807038	2.5119086	0.01200802	0.03587682
ENSDARG00000092243	BX005065.1	159.982616	-0.689274208	0.27445667	-2.51141355	0.01202487	0.03592286
ENSDARG00000067950	zcchc2	700.180798	-0.357982158	0.14254753	-2.51131782	0.01202814	0.03592829
ENSDARG00000062204	sigirr	224.526879	0.471450929	0.18773948	2.5111976	0.01203223	0.03593621
ENSDARG00000062806	ttll9	48.1905072	0.93627323	0.37285104	2.51111871	0.01203492	0.03593992
ENSDARG00000058551	popdc3	239.010498	0.45230805	0.18017417	2.51039348	0.01205967	0.0360095
ENSDARG00000097339	slc9a1b	292.473312	0.436554997	0.17392358	2.51003913	0.01207178	0.03604133
ENSDARG00000039436	il13ra2	236.270656	-0.506866964	0.20195504	-2.50980099	0.01207992	0.03606038
ENSDARG00000077317	snx29	659.4821	0.37479029	0.14933266	2.50976768	0.01208106	0.03606038
ENSDARG00000092324	NA	4.31061669	-4.648039799	1.85221233	-2.509453	0.01209183	0.03608819
ENSDARG00000102460	si:ch73-170d6.4	13.6887072	1.613137296	0.64291197	2.50911068	0.01210356	0.03611885
ENSDARG00000057782	baz2ba	6716.80601	-0.321564651	0.12818539	-2.50859054	0.01212139	0.03616685
ENSDARG00000002750	phf20a	644.994846	-0.43138915	0.17196708	-2.50855665	0.01212255	0.03616685
ENSDARG00000059150	tmem107	413.17983	0.400951049	0.15983749	2.50849188	0.01212478	0.03616914
ENSDARG00000111843	BX000451.4	121.237564	-0.663148702	0.26444525	-2.50769754	0.01215206	0.0362462
ENSDARG00000090153	cyldl	144.339834	0.518187424	0.20665151	2.50754235	0.0121574	0.03624907
ENSDARG00000056984	rad9a	103.602309	-0.598703701	0.23875945	-2.50756024	0.01215679	0.03624907
ENSDARG00000104676	prkd2	1113.03454	0.307770656	0.12273396	2.50762428	0.01215458	0.03624907
ENSDARG00000045411	acad11	725.637976	0.390337295	0.15568251	2.50726488	0.01216695	0.03627318
ENSDARG00000008029	mep1a.2	11.6980565	2.182415183	0.8705701	2.50688048	0.01218019	0.0363083
ENSDARG00000074764	zgc:171426	24.776167	-1.741804436	0.69497984	-2.50626613	0.01220137	0.03636273
ENSDARG00000002916	sec31b	1321.14038	0.331439173	0.13224214	2.50630524	0.01220003	0.03636273
ENSDARG00000008785	dldh	9372.85383	0.252197147	0.10063543	2.50604739	0.01220893	0.03638087
ENSDARG00000032618	mgst1.1	296.177585	0.382132484	0.15249236	2.50591232	0.01221359	0.03638237
ENSDARG00000035133	pho	371.225374	-0.497738668	0.19862626	-2.50590567	0.01221382	0.03638237
ENSDARG00000069834	pnpla4	81.6239969	-0.804774625	0.32114147	-2.50598166	0.0122112	0.03638237
ENSDARG00000068893	mettl5	435.596306	0.327683768	0.13078468	2.505521	0.01222711	0.03641761
ENSDARG00000075990	acap3a	566.597527	-0.376736011	0.15037219	-2.50535698	0.01223279	0.03643013
ENSDARG00000090785	diaph3	384.454765	-0.378786406	0.15122327	-2.50481568	0.01225152	0.03648156
ENSDARG00000038901	tmem256	176.585653	0.598017645	0.23875399	2.50474408	0.012254	0.03648458
ENSDARG00000036152	gas2b	117.393106	-0.753833631	0.30102182	-2.5042491	0.01227116	0.03653128
ENSDARG00000088378	znf1000	16.941077	-1.459710799	0.58291892	-2.50414037	0.01227493	0.03653376
ENSDARG00000037645	zgc:173570	108.781208	0.612024313	0.2444018	2.50417273	0.01227381	0.03653376

Table S2. DEGs of WT vs. *terfa-/-*

ENSDARG00000056605	wbp2	840.772091	-0.314483634	0.12559332	-2.5039837	0.01228037	0.03654557
ENSDARG00000117382	BX323560.1	13.70508	1.614853109	0.64495084	2.50383905	0.01228539	0.03655018
ENSDARG00000091459	gabra2a	146.223258	-0.699563543	0.27939771	-2.50382707	0.01228581	0.03655018
ENSDARG00000079031	si:ch211-22d5.2	87.8015741	-0.986407493	0.39396228	-2.50381201	0.01228633	0.03655018
ENSDARG00000089382	zgc:158463	577.186635	2.14504904	0.85673972	2.5037348	0.01228901	0.03655288
ENSDARG00000101859	mnta	1075.14806	0.31878179	0.12732422	2.50370112	0.01229018	0.03655288
ENSDARG00000025162	asic4a	269.367283	-0.50556734	0.20196092	-2.50329292	0.01230437	0.03659069
ENSDARG00000027710	btbd16	14.498957	1.750967459	0.69950115	2.50316596	0.01230878	0.03659944
ENSDARG00000059437	fktn	660.650088	0.303613063	0.12132776	2.50242038	0.01233474	0.03666785
ENSDARG00000091592	peli1a	150.321266	0.860184544	0.34374039	2.502425	0.01233458	0.03666785
ENSDARG00000104717	tbxa2r	31.3704254	1.006591222	0.40226507	2.50230833	0.01233864	0.03667507
ENSDARG00000075012	glra2	178.64187	-0.748149357	0.29908283	-2.50147879	0.01236759	0.03675671
ENSDARG00000073848	arhgef7b	1438.16509	-0.307229804	0.12284367	-2.50098188	0.01238495	0.03680392
ENSDARG00000074331	eif4g3b	2320.02183	0.311900988	0.12471639	2.5008822	0.01238844	0.03680988
ENSDARG00000077626	tafa4a	3.25170928	-4.498518066	1.79910125	-2.50042518	0.01240443	0.036853
ENSDARG00000002986	gda	68.3729882	1.311657002	0.52465615	2.50003168	0.01241822	0.03687648
ENSDARG00000105068		228.32128	0.509883988	0.2039455	2.50009927	0.01241585	0.03687648
ENSDARG00000019316	fer1l6	283.977572	0.762538405	0.30501167	2.50003025	0.01241827	0.03687648
ENSDARG00000030104	sh3bp4	321.455284	0.408400889	0.1633563	2.50006202	0.01241716	0.03687648
ENSDARG00000071360	morn2	62.7237339	0.815835616	0.32633826	2.49996924	0.01242041	0.03687842
ENSDARG00000000567	znf281a	1640.40248	-0.408028619	0.16324005	-2.49956192	0.0124347	0.03691203
ENSDARG00000097892	BX470264.1	127.072485	-0.698590525	0.27948122	-2.49959735	0.01243345	0.03691203
ENSDARG00000056835	laynb	101.004203	0.576713829	0.23073463	2.49946804	0.01243799	0.0369174
ENSDARG00000053520	LO017829.1	106.649055	0.569675245	0.22794137	2.49921828	0.01244676	0.03693902
ENSDARG00000057890	si:dkey-33c12.4	704.878095	0.366451678	0.14663453	2.49908171	0.01245156	0.03694884
ENSDARG00000074575	ppp1r9alb	991.128778	0.36990511	0.14803165	2.49882447	0.0124606	0.03697126
ENSDARG00000012248	rgma	4560.41019	-0.238455383	0.09543025	-2.49874009	0.01246357	0.03697565
ENSDARG00000091481	foxc1a	1518.84595	0.327032698	0.13091066	2.49813644	0.01248481	0.03703426
ENSDARG00000051783	rplp0	98205.942	0.252241634	0.10101143	2.4971593	0.01251927	0.03713204
ENSDARG00000027828	grin1a	6537.75663	-0.356218799	0.14270441	-2.49620033	0.01255317	0.03722609
ENSDARG00000038876	zgc:101569	639.89485	0.299738072	0.12007883	2.49617749	0.01255398	0.03722609
ENSDARG00000094177	si:ch211-208f21.2	11.4062556	2.48367247	0.99505392	2.49601797	0.01255962	0.03723839
ENSDARG00000044204	endou	95.7582365	-0.88718116	0.35548559	-2.49568813	0.01257131	0.03726859
ENSDARG00000058732	scgn	96.2895145	-0.68564441	0.27475515	-2.49547427	0.01257889	0.03728661
ENSDARG00000006691	rpl12	34034.5883	0.243699362	0.09766019	2.49538088	0.0125822	0.03728754
ENSDARG00000077085	zgc:110821	129.306359	-0.612092214	0.24528838	-2.49539837	0.01258158	0.03728754
ENSDARG00000007723	efnb1	803.623325	-0.362368413	0.1452205	-2.49529794	0.01258514	0.03729181
ENSDARG00000070618	tatdn2	10.502659	2.306303551	0.92432604	2.4951191	0.01259149	0.03730605
ENSDARG00000004037	cog2	1042.85442	-0.25102253	0.10060709	-2.4950779	0.01259295	0.03730605
ENSDARG00000010154	vstm4a	321.98526	-0.383277545	0.15364443	-2.49457491	0.01261081	0.03735451
ENSDARG00000073673	adgra3	1069.89038	0.295290545	0.11842641	2.49345174	0.01265078	0.03746815
ENSDARG00000093628	s100a11	1521.74771	-0.405253827	0.1625304	-2.49340322	0.01265251	0.03746815
ENSDARG00000068941	zgc:113983	25.4984534	-1.249658964	0.50119279	-2.49336982	0.0126537	0.03746815
ENSDARG00000096991	CR847968.1	590.405036	-0.3917531	0.1571839	-2.49232333	0.01269105	0.03757426
ENSDARG00000061385	haus3	312.12244	-0.55990276	0.22466879	-2.49212527	0.01269812	0.03759074
ENSDARG00000059060	lgalsla	200.864796	0.494738011	0.19852666	2.49204822	0.01270088	0.03759442
ENSDARG00000035692	rps3a	54020.2214	0.26809384	0.10758403	2.49194832	0.01270445	0.03760052
ENSDARG00000026178	ube3d	166.176508	-0.506735047	0.20335456	-2.49187948	0.01270691	0.03760333
ENSDARG00000097934	si:dkey-221l4.10	61.4479426	0.90635929	0.36374125	2.49176932	0.01271086	0.03761052
ENSDARG00000079900	shroom4	1538.22889	0.251448555	0.10092153	2.49152549	0.01271958	0.03763186
ENSDARG00000102360	si:ch211-11p18.6	5.75119185	4.372957345	1.75522237	2.49139792	0.01272415	0.0376409
ENSDARG00000110653	CABZ01088049.1	194.062222	0.543843762	0.2183255	2.49097679	0.01273924	0.03768106
ENSDARG00000063525	adpgk	979.953595	0.318063503	0.12769611	2.4907846	0.01274614	0.03769696
ENSDARG00000097215	si:dkey-172k15.3	152.504429	-0.640836036	0.25731581	-2.49046507	0.0127576	0.03772638
ENSDARG00000076228	kif2c	725.171872	-0.430196905	0.17277422	-2.48993691	0.01277658	0.037778
ENSDARG00000035357	gnb2	2506.70507	-0.279290673	0.11218141	-2.48963426	0.01278746	0.03780568
ENSDARG00000025013	gna14a	45.674978	-1.124836693	0.45181758	-2.48958153	0.01278936	0.03780679
ENSDARG00000018524	midn	4954.84425	0.232745049	0.09348931	2.48953657	0.01279098	0.03780708

Table S2. DEGs of WT vs. *terfa-/-*

ENSDARG00000069869	zgc:113030	136.322553	-0.637524545	0.25611462	-2.48921577	0.01280252	0.03783221
ENSDARG00000029866	slc6a14	107.693109	-0.765638735	0.30758219	-2.48921671	0.01280249	0.03783221
ENSDARG00000095623	dpm2	615.189963	0.324056245	0.13019671	2.48897418	0.01281123	0.03784987
ENSDARG00000101259	hgh1	316.489804	-0.466647192	0.18748642	-2.48896531	0.01281155	0.03784987
ENSDARG00000020197	rpl5a	30322.164	0.246858345	0.09918346	2.48890649	0.01281367	0.03785164
ENSDARG00000100947	mafk	189.24551	0.587571618	0.2360912	2.48874854	0.01281936	0.03786396
ENSDARG00000037266	myoz2b	569.815902	0.415554955	0.16699343	2.48845099	0.01283009	0.03789115
ENSDARG00000003822	ankrd16	173.406498	-0.474967255	0.19089098	-2.48815978	0.0128406	0.03790493
ENSDARG00000044332	zranb2	1734.60328	0.312759528	0.12569767	2.48818872	0.01283956	0.03790493
ENSDARG00000055192	zgc:136930	41336.9527	0.34566892	0.13892828	2.48811048	0.01284238	0.03790493
ENSDARG00000087107	si:ch73-222h13.1	50.4473289	0.994139471	0.3995436	2.48818772	0.01283959	0.03790493
ENSDARG00000097019	dhrs13b.1	11.0551098	1.769383047	0.7111335	2.48811658	0.01284216	0.03790493
ENSDARG00000090340	si:dkey-27o4.1	175.038525	-0.597757362	0.24026208	-2.4879388	0.01284858	0.03791873
ENSDARG00000068030	si:cabz01007802.1	599.856824	0.435185453	0.17492603	2.48782563	0.01285267	0.0379263
ENSDARG00000063299	pcloa	4788.19662	-0.394015596	0.15840716	-2.48735975	0.01286952	0.0379715
ENSDARG00000104367	casp20	442.787249	0.43377024	0.17442909	2.48679991	0.01288979	0.03802679
ENSDARG00000031693	kif9	47.3644728	0.907440456	0.36507941	2.48559749	0.01293342	0.03815097
ENSDARG00000031777	pparaa	342.255705	0.405017607	0.16299005	2.48492235	0.01295797	0.03821433
ENSDARG00000111552	FO904980.1	20.5340032	1.312923175	0.52835181	2.48494121	0.01295728	0.03821433
ENSDARG00000115108	C19H5orf49	4.06068861	-4.158704485	1.6737042	-2.48473087	0.01296494	0.03823035
ENSDARG00000060006	nedd4l	1949.8301	0.293783291	0.11824455	2.48453982	0.0129719	0.03824633
ENSDARG00000094503	AL954767.1	50.9968584	-0.874248916	0.35190101	-2.48436038	0.01297844	0.03826107
ENSDARG00000021805	tle5	743.673928	-0.308845528	0.12432167	-2.48424529	0.01298264	0.0382689
ENSDARG00000005544	btbd3a	232.543135	0.690661296	0.27802135	2.48420236	0.0129842	0.03826898
ENSDARG00000083894	CR925757.1	3.82830916	4.16733271	1.67783154	2.4837611	0.0130003	0.03831188
ENSDARG00000088227	tmem163b	283.372289	-0.442018506	0.17796646	-2.48371804	0.01300187	0.03831198
ENSDARG00000098270	znf1053	43.7748363	1.032325963	0.41564938	2.48364612	0.0130045	0.03831257
ENSDARG00000097718	stard10	780.190412	-0.292656804	0.11783439	-2.48362809	0.01300516	0.03831257
ENSDARG00000063062	ubn2a	1101.45483	-0.309193507	0.12451627	-2.48315757	0.01302235	0.03835867
ENSDARG00000060008	hhipl2	316.267239	0.539645174	0.21733327	2.48303072	0.01302699	0.03836778
ENSDARG00000067546	si:ch211-193c2.2	113.582509	-0.660366637	0.26601624	-2.48242978	0.01304898	0.03842801
ENSDARG00000099518	si:ch211-119e14.1	78.3573967	-0.844418382	0.34018572	-2.48222763	0.01305638	0.03844336
ENSDARG00000040303	nbn	528.675872	-0.330330839	0.1330797	-2.48220305	0.01305729	0.03844336
ENSDARG00000097467	CU693502.1	182.900284	-0.568211909	0.2289221	-2.48211911	0.01306036	0.03844786
ENSDARG00000079631	si:ch211-113e8.10	1341.80151	-0.330380094	0.13311659	-2.48188515	0.01306894	0.03846856
ENSDARG00000009250	etfb	5420.62973	0.27272582	0.1098957	2.48167863	0.01307652	0.0384863
ENSDARG00000053194	pdzd11	458.213556	0.361709496	0.14580996	2.48069129	0.01311279	0.03858849
ENSDARG00000011648	snrpd1	1526.8267	-0.37233333	0.15011777	-2.48027482	0.01312812	0.03862902
ENSDARG00000062986	pnpla7a	2368.11291	0.232908234	0.09391581	2.47996843	0.0131394	0.03865765
ENSDARG00000031214	arcn1b	3207.07172	0.198804841	0.0801809	2.47945391	0.01315837	0.03870431
ENSDARG00000079294	or115-15	6.69962458	-4.118750395	1.66114816	-2.47945999	0.01315815	0.03870431
ENSDARG00000078059	nudcd2	378.34589	-0.46556121	0.18777693	-2.47933122	0.0131629	0.03871304
ENSDARG00000074017	usp31	872.659426	-0.326128997	0.13154927	-2.47913963	0.01316997	0.03872926
ENSDARG00000056819	hoxa9b	446.388511	-0.363151198	0.14649358	-2.47895633	0.01317674	0.03874
ENSDARG00000031044	lipg	662.610756	0.302856079	0.12217001	2.4789723	0.01317615	0.03874
ENSDARG00000051873	ahcyl2a	748.852847	0.370024876	0.14927558	2.47880383	0.01318238	0.03875198
ENSDARG00000068714	ksr1a	495.916446	-0.402042783	0.16223668	-2.47812501	0.01320749	0.0388212
ENSDARG00000039726	zgc:123321	275.541356	0.519476643	0.20963428	2.47801378	0.0132116	0.03882871
ENSDARG00000059841	exosc1	849.819143	0.264754379	0.10685885	2.47760836	0.01322662	0.03886367
ENSDARG00000111958	BX248392.1	2.82070675	4.540386931	1.83255954	2.47762042	0.01322618	0.03886367
ENSDARG00000023852	lsm4	1391.14254	-0.353331004	0.14264263	-2.47703652	0.01324784	0.03892139
ENSDARG00000061916	tmem51b	332.290478	0.393021661	0.15867636	2.47687596	0.0132538	0.0389343
ENSDARG00000104491	ampd2a	810.245398	0.373787356	0.15091796	2.47675853	0.01325816	0.03894251
ENSDARG00000025081	grnb	1430.64143	0.286320801	0.11560516	2.47671303	0.01325985	0.03894287
ENSDARG00000078362	TNC	3974.07105	0.355902923	0.14373155	2.4761643	0.01328025	0.03899356
ENSDARG00000117353	BX005154.1	6.4619109	-3.443020009	1.39044738	-2.47619584	0.01327907	0.03899356
ENSDARG00000040282	zgc:92590	6.22167046	-3.71309036	1.49958284	-2.4760822	0.0132833	0.03899792
ENSDARG00000108250	BX511086.1	9.30846096	-2.180199104	0.88058841	-2.47584352	0.01329218	0.03901478

Table S2. DEGs of WT vs. *terfa-/-*

ENSDARG00000112337	mindy4b	153.191645	-0.515742475	0.20830973	-2.47584438	0.01329215	0.03901478
ENSDARG00000075949	prkd1	176.063881	-0.573594294	0.23170209	-2.47556806	0.01330244	0.03904028
ENSDARG00000052606	dpp9	2791.3592	0.204228828	0.08249956	2.47551418	0.01330445	0.03904157
ENSDARG00000007264	nipsnap2	1464.62256	0.373745826	0.15100944	2.47498323	0.01332424	0.03909504
ENSDARG00000013628	cd164	4235.65349	0.329456615	0.13312088	2.47486808	0.01332854	0.03910303
ENSDARG00000002791	atp1a1a.1	24108.5372	0.21307617	0.08609801	2.47480957	0.01333072	0.03910482
ENSDARG00000014871	syngr3a	2534.27032	-0.375022922	0.15156687	-2.47430669	0.0133495	0.03914977
ENSDARG00000017219	pabpc1a	43142.1435	0.185808386	0.07509616	2.47427274	0.01335077	0.03914977
ENSDARG00000013009	ubiad1	1209.15641	-0.286039886	0.11560552	-2.47427533	0.01335068	0.03914977
ENSDARG00000028699	crkl	2126.38492	-0.224132438	0.09058916	-2.47416393	0.01335484	0.03915708
ENSDARG00000026224	fanci	359.14297	-0.526976012	0.2129976	-2.47409368	0.01335747	0.03915762
ENSDARG00000034443	mpped2	499.741683	-0.485593841	0.19627291	-2.47407467	0.01335818	0.03915762
ENSDARG00000052558	porcn	317.978657	-0.404074933	0.1633645	-2.47345614	0.01338132	0.03922085
ENSDARG00000056381	cfap97	239.159286	0.440961069	0.17829383	2.47322672	0.01338992	0.03923216
ENSDARG00000013542	lpgat1	1293.92278	0.243352772	0.09839161	2.47330816	0.01338687	0.03923216
ENSDARG00000036156	fnbp1a	712.952731	-0.514254043	0.20792724	-2.4732404	0.0133894	0.03923216
ENSDARG00000097932	BX510649.1	13.607	1.494991942	0.6044962	2.47312051	0.0133939	0.03923457
ENSDARG00000041243	setdb1a	1527.52592	-0.240291005	0.09715942	-2.4731622	0.01339233	0.03923457
ENSDARG00000091902	b3gnt2b	1198.68101	0.392061906	0.15854297	2.47290632	0.01340193	0.03925346
ENSDARG00000097795	zgc:162952	216.18239	-0.546966095	0.22120019	-2.4727198	0.01340892	0.03926933
ENSDARG00000042559	tnni1c	770.692045	-0.341498418	0.13813809	-2.47215254	0.01343022	0.03932706
ENSDARG00000100167	CSDC2	93.6425897	0.590060166	0.23870725	2.47189881	0.01343976	0.03935035
ENSDARG00000097181	defbl3	12.2474427	-1.950604628	0.78921432	-2.47157786	0.01345183	0.03938105
ENSDARG00000020224	si:ch211-225b10.3	109.076108	0.564678786	0.22847746	2.47148576	0.01345529	0.03938656
ENSDARG00000032102	prdx3	2747.63557	0.244257838	0.09883865	2.47127867	0.01346309	0.03940473
ENSDARG00000038235	pkdccb	196.744098	-0.516593316	0.20907563	-2.47084424	0.01347945	0.03944799
ENSDARG00000036414	si:ch73-335l21.1	1719.49635	0.319189543	0.12918605	2.47077408	0.0134821	0.03945108
ENSDARG00000115426	CR925768.11	20.082512	1.356037993	0.54891096	2.47041523	0.01349563	0.03948139
ENSDARG00000070454	pla2g12a	260.194283	0.457055096	0.18500855	2.47045394	0.01349417	0.03948139
ENSDARG00000052290	rab33ba	496.840126	-0.31095943	0.12589255	-2.47003839	0.01350986	0.03951835
ENSDARG00000036542	pknox1.2	748.121715	-0.350442715	0.1418835	-2.46993289	0.01351384	0.03952536
ENSDARG00000076966	pagr1	634.543665	-0.374182826	0.15150376	-2.46979242	0.01351915	0.03953623
ENSDARG00000057017	slc6a4b	27.9807163	-1.341363534	0.54331177	-2.46886522	0.01355423	0.03963379
ENSDARG00000103843	spag8	28.2812061	1.282416305	0.5194437	2.46882637	0.0135557	0.03963379
ENSDARG00000062277	ppp2r2cb	497.281541	-0.428744078	0.17366742	-2.46876514	0.01355802	0.03963591
ENSDARG00000045481	gsap	206.153091	0.495747562	0.2008255	2.46854891	0.01356621	0.03964672
ENSDARG00000054438	fzd2	3525.50075	-0.238091185	0.09645015	-2.46854129	0.0135665	0.03964672
ENSDARG00000075492	ccdc160	132.107789	-0.551106768	0.22324719	-2.46859437	0.01356449	0.03964672
ENSDARG00000045847	si:ch211-214j24.1(	1776.20637	0.240786886	0.09756989	2.46784003	0.01359311	0.03971513
ENSDARG00000020187	GCA	2130.83772	0.290401551	0.11767265	2.46787636	0.01359173	0.03971513
ENSDARG00000044094	gfpt2	1024.65871	0.341352611	0.13833393	2.46759858	0.01360228	0.03973726
ENSDARG00000105306	si:dkeyp-44b5.5	34.8612112	0.960017292	0.38911776	2.4671639	0.0136188	0.03977302
ENSDARG00000086571	kcna7	394.278815	0.478625035	0.19399915	2.46715024	0.01361932	0.03977302
ENSDARG00000100274	WDR7	2096.54446	0.377680344	0.15308275	2.46716457	0.01361878	0.03977302
ENSDARG00000001882	kbtbd12	1023.24653	0.402710726	0.16323725	2.46702717	0.013624	0.03978202
ENSDARG00000008030	myl9b	4736.32226	0.360730485	0.14622735	2.46691538	0.01362826	0.0397851
ENSDARG00000022564		511.735965	-0.361426745	0.14650818	-2.46693909	0.01362735	0.0397851
ENSDARG00000035820	drd4b	33.5816104	-1.051104687	0.42609823	-2.46681306	0.01363215	0.03979179
ENSDARG00000101379	mfsd1	508.133949	0.496128527	0.20113015	2.46670393	0.01363631	0.03979925
ENSDARG00000015757	tmem50a	2007.82394	-0.287193258	0.11644265	-2.46639233	0.01364818	0.03982921
ENSDARG00000102340	ptn	1881.28907	-0.305126494	0.1237171	-2.46632435	0.01365077	0.0398321
ENSDARG00000096011	si:dkeyp-4f2.3	35.8344363	1.075305302	0.43600895	2.46624592	0.01365376	0.03983614
ENSDARG00000070178	zp3d.2	11.1837866	2.017030062	0.81796698	2.46590646	0.0136667	0.03986923
ENSDARG00000003054	tacr3l	35.5973665	-1.12491719	0.45629082	-2.46535134	0.0136879	0.03992638
ENSDARG00000078267	si:dkey-181m9.8	269.370189	0.419930507	0.17034029	2.4652447	0.01369197	0.03993358
ENSDARG00000027963	camkva	254.693565	-0.495934934	0.20117715	-2.46516538	0.013695	0.03993773
ENSDARG00000113433	FAM126B	78.8856382	0.923782472	0.37474799	2.46507655	0.0136984	0.03994295
ENSDARG00000096714	si:dkey-66i24.7	154.134239	-0.566370385	0.22977866	-2.46485199	0.01370699	0.0399633

Table S2. DEGs of WT vs. *terfa-/-*

ENSDARG00000043133	cldnf	608.643733	0.362990409	0.14729989	2.46429514	0.0137283	0.04002075
ENSDARG00000105532	znf16l	755.694218	-0.383599655	0.15566768	-2.46422157	0.01373112	0.04002233
ENSDARG00000042577	batf3	12.7644309	1.501493219	0.60932354	2.46419697	0.01373206	0.04002233
ENSDARG00000023318	fmn2a	361.556291	-0.44783104	0.18173863	-2.46414893	0.0137339	0.040023
ENSDARG00000117714	FQ311907.1	2.58590473	4.511695322	1.83125137	2.46372256	0.01375025	0.04006594
ENSDARG00000058699	tmeff2b	683.591705	-0.2824791	0.11466163	-2.46358871	0.01375539	0.04007621
ENSDARG00000042187	smek1	4442.26353	-0.273298864	0.11093972	-2.46348977	0.01375918	0.04007924
ENSDARG00000053097	hsf2	1017.05012	0.257269335	0.1044334	2.46347754	0.01375965	0.04007924
ENSDARG00000073911	tmem255a	1440.54706	0.325589148	0.1321689	2.46343232	0.01376139	0.0400796
ENSDARG00000078547	si:ch211-264f5.2	19.822438	1.320174531	0.53592093	2.46337556	0.01376357	0.04008125
ENSDARG00000059125	prkacbb	2748.04647	-0.239295502	0.09715164	-2.4631134	0.01377363	0.04010587
ENSDARG00000089434	etv7	12.3091156	1.796693108	0.72951836	2.46284836	0.01378382	0.04013082
ENSDARG00000114874	FP236318.4	465.824779	-0.504335002	0.20478976	-2.46269642	0.01378966	0.04014313
ENSDARG00000036056	vrk3	317.556266	-0.368727416	0.14973227	-2.46257817	0.01379421	0.04014666
ENSDARG00000017155	wnt10a	102.478522	0.577614285	0.23455661	2.46257948	0.01379416	0.04014666
ENSDARG00000001873	phgdh	2773.88585	0.526964415	0.21399232	2.46253893	0.01379572	0.04014666
ENSDARG00000091835	si:ch73-264p11.1	68.7603015	-0.751926299	0.30538325	-2.46223817	0.01380729	0.04017564
ENSDARG00000000460	nitr2b	5.65959004	3.123130134	1.26856611	2.46193723	0.01381889	0.04020466
ENSDARG00000014545	golim4b	337.737923	0.471697617	0.19160414	2.46183412	0.01382286	0.04021151
ENSDARG00000052121	rag2	44.353661	-0.976124267	0.39653492	-2.4616351	0.01383053	0.04022912
ENSDARG00000034080	plcd1b	1477.39522	0.261001754	0.10604823	2.46116092	0.01384882	0.04027762
ENSDARG00000002167	cacnb1	1319.31527	0.349478503	0.14200857	2.46096762	0.01385629	0.04029461
ENSDARG00000075263	ankrd1a	112.586751	-0.578774548	0.23523138	-2.46044788	0.01387637	0.0403483
ENSDARG00000075867	mrtfaa	163.338587	0.622973224	0.25324158	2.45999577	0.01389387	0.04039444
ENSDARG00000070352	hoxc12a	462.259918	-0.50628179	0.20585087	-2.4594591	0.01391466	0.04045015
ENSDARG00000034677	scel	2563.96069	0.341428618	0.13884858	2.45899964	0.01393248	0.04049722
ENSDARG00000075747	zfyve16	1119.68726	0.377677289	0.15361258	2.4586352	0.01394663	0.0405336
ENSDARG00000045832	cart2	53.1847758	0.811545869	0.33009391	2.45853025	0.0139507	0.04054071
ENSDARG00000060338	trmt12	130.961586	0.645100229	0.26244143	2.45807318	0.01396847	0.0405876
ENSDARG00000098810	VSTM2B	489.445691	-0.435266951	0.1771151	-2.45753726	0.01398933	0.04064346
ENSDARG00000006120	tbx2b	2546.37553	-0.309804829	0.12607914	-2.45722515	0.01400149	0.04067404
ENSDARG00000076309	mxra5b	674.861308	0.557132898	0.22677249	2.45679227	0.01401837	0.04071832
ENSDARG00000097740	elob	204.992698	-0.521451606	0.21230827	-2.45610595	0.01404517	0.0407914
ENSDARG00000103316	si:ch211-212k18.5	1263.76809	-0.313899825	0.12781079	-2.45597283	0.01405038	0.04080175
ENSDARG00000095192	BX957317.1	90.0688514	-0.641917422	0.26140516	-2.4556417	0.01406333	0.04083459
ENSDARG00000102165	sema6a	1604.80232	-0.426390925	0.17364452	-2.45553917	0.01406734	0.04084147
ENSDARG00000054264	cog4	1025.29182	0.304891134	0.12416901	2.45545265	0.01407073	0.04084653
ENSDARG00000056720	utp3	515.449813	-0.405217572	0.16503219	-2.45538499	0.01407338	0.04084945
ENSDARG00000101318	pcdh2ab9	239.086946	-0.54113547	0.22040061	-2.45523579	0.01407922	0.04085686
ENSDARG00000109626	si:ch211-226h7.3	324.715717	0.573006983	0.2333785	2.45526894	0.01407792	0.04085686
ENSDARG00000045514	atp5f1c	12944.3654	0.258991254	0.10550797	2.45470803	0.01409991	0.04091211
ENSDARG00000117530	CABZ01085662.1	187.941677	0.497361325	0.20262812	2.45455229	0.01410602	0.04092506
ENSDARG00000070407	abitram	166.320824	-0.578755612	0.23582591	-2.45416461	0.01412123	0.04096443
ENSDARG00000011727	b9d1	322.946111	-0.444196657	0.18104	-2.453583	0.01414409	0.04102595
ENSDARG00000035122	camsap1b	1269.29744	-0.263352048	0.10736137	-2.45294982	0.01416901	0.04108914
ENSDARG00000060854	kctd3	754.340739	0.387277134	0.15788519	2.45290348	0.01417084	0.04108914
ENSDARG00000089800	prr7	87.053225	-0.69247757	0.28230697	-2.45292414	0.01417002	0.04108914
ENSDARG00000003797	asb2a.1	589.635554	0.36858353	0.15026911	2.45282301	0.01417401	0.04109354
ENSDARG00000030621	th	256.870066	0.566158543	0.23093707	2.45157069	0.01422343	0.04123201
ENSDARG00000060315	rabgap1l2	249.247277	0.462150427	0.18852595	2.45138892	0.01423061	0.04124803
ENSDARG00000033899	ap2m1b	7046.05182	0.269757554	0.11005025	2.45122163	0.01423723	0.04126239
ENSDARG00000021389	jag2b	1894.78129	0.282223407	0.11515803	2.45074884	0.01425594	0.04131181
ENSDARG00000015531	slc4a8	678.669258	-0.507335867	0.20702468	-2.45060578	0.01426161	0.04132341
ENSDARG00000095457	si:ch211-147d7.5	35.8820744	1.095022516	0.446885	2.45034519	0.01427193	0.04133907
ENSDARG00000076126	actc1a	13.0047329	1.6781748	0.68486029	2.45039001	0.01427016	0.04133907
ENSDARG00000045275	klhl14	512.168617	-0.507785314	0.20723026	-2.45034345	0.014272	0.04133907
ENSDARG00000070866	tmem11	2038.1637	0.34978899	0.14276586	2.45008844	0.01428211	0.04136354
ENSDARG00000112449	BX248122.1	352.556138	-0.43388347	0.17711803	-2.44968548	0.01429811	0.0414024

Table S2. DEGs of WT vs. *terfa-/-*

ENSDARG00000036930	tbx18	370.670178	0.374858191	0.15302418	2.44966642	0.01429886	0.0414024
ENSDARG00000016664	rnf20	2361.52395	-0.342245194	0.13971461	-2.44960209	0.01430142	0.04140497
ENSDARG00000057886	BX901922.1	6.93063646	2.394902819	0.9777594	2.44937846	0.0143103	0.04142587
ENSDARG00000068701	gpr85	1289.99013	-0.412907654	0.16861584	-2.44880705	0.01433302	0.04148681
ENSDARG00000105255	zbtb4	847.812766	-0.332225982	0.13567602	-2.44867137	0.01433842	0.04149761
ENSDARG00000086452	dthd1	36.5180763	-1.031607997	0.4214868	-2.44754523	0.01438331	0.04162267
ENSDARG00000059743	tmem98	198.238415	0.408345417	0.16684994	2.44738127	0.01438985	0.04163676
ENSDARG00000005800	ampd3a	511.341605	-0.416315039	0.17016223	-2.44657732	0.01442199	0.04172488
ENSDARG00000011473	calcrla	363.465746	0.393152571	0.16070067	2.44648992	0.01442548	0.04173014
ENSDARG00000018923	fat2	2602.81297	0.374729471	0.15318646	2.44623102	0.01443585	0.04175526
ENSDARG00000091513	C1H3orf70	1044.82276	-0.273696587	0.11189294	-2.44605767	0.01444279	0.04176077
ENSDARG00000103433	rpl14	36378.7964	0.242501768	0.09913913	2.44607515	0.01444209	0.04176077
ENSDARG00000029353	serpine2	1306.76519	0.263128577	0.10757078	2.44609726	0.0144412	0.04176077
ENSDARG00000087805	FP236735.1	54.8728173	0.840245681	0.34355763	2.44571975	0.01445633	0.04179506
ENSDARG00000079011	col17a1b	1662.02477	0.264971869	0.10834715	2.44558233	0.01446184	0.04180613
ENSDARG00000079267	fermt3a	67.7468627	0.872083027	0.35661835	2.4454239	0.0144682	0.04181964
ENSDARG00000056679	morc3a	556.42691	-0.285408202	0.11672229	-2.44519013	0.01447758	0.0418394
ENSDARG00000077549	aifm2	1476.75128	0.250622703	0.1024986	2.44513293	0.01447988	0.0418394
ENSDARG00000043131	BX664625.1	16.4438037	-1.719983341	0.70343291	-2.44512777	0.01448008	0.0418394
ENSDARG00000037954	tnnt1	446.954179	-0.474921943	0.19424887	-2.44491479	0.01448864	0.04185924
ENSDARG00000055917	nipa1	124.394294	0.549597753	0.22479924	2.44483811	0.01449172	0.04186328
ENSDARG00000003846	mrpl21	689.956962	-0.375310187	0.15357685	-2.44379405	0.01453372	0.04197486
ENSDARG00000079188	nxph2b	89.3629758	-0.804018164	0.32900057	-2.44381995	0.01453268	0.04197486
ENSDARG00000103422	usp10	5324.06759	0.178729604	0.07313856	2.4437123	0.01453701	0.04197949
ENSDARG00000032619	tob1a	2795.43571	0.391342222	0.16015951	2.44345299	0.01454747	0.04200479
ENSDARG00000089390	pnpla2	258.425836	0.439369992	0.17984597	2.44303502	0.01456432	0.04204859
ENSDARG00000016505	apbb1ip	442.854677	0.346859916	0.14202062	2.44232079	0.01459317	0.04212698
ENSDARG00000076602	prss16	958.004871	0.479134863	0.19620019	2.4420713	0.01460326	0.04215121
ENSDARG00000099996	rhot1a	1927.91066	-0.23445787	0.09601826	-2.441805	0.01461404	0.04217742
ENSDARG00000069853	txndc9	2193.76371	-0.218158385	0.08936413	-2.4412298	0.01463734	0.04223976
ENSDARG00000032116	cnot7	135.834722	0.530532283	0.21733926	2.44103289	0.01464532	0.04225789
ENSDARG00000007693	nfkbiab	2703.43408	0.473734103	0.19410858	2.44056243	0.01466441	0.04230806
ENSDARG00000021383	naalad2	637.138562	-0.388669822	0.15926079	-2.44046152	0.01466851	0.04231497
ENSDARG00000086691	dnajc12	115.670919	0.615202619	0.2521869	2.43947094	0.01470879	0.0424202
ENSDARG00000017367	rhbdf1b	387.018337	-0.429419397	0.17603206	-2.43943852	0.01471011	0.0424202
ENSDARG00000087476	cbln20	393.628402	-1.256997127	0.51527301	-2.43947791	0.0147085	0.0424202
ENSDARG00000044090	zmpste24	1381.87998	-0.247619321	0.10152759	-2.43893617	0.01473057	0.04247428
ENSDARG00000021112	c1d	405.826057	-0.366432326	0.15024569	-2.43888749	0.01473256	0.04247508
ENSDARG00000077605	frrs1b	434.32538	0.364575346	0.14951095	2.43845247	0.0147503	0.0425213
ENSDARG00000068256	rwdd	503.130779	0.345312672	0.14162181	2.4382733	0.01475761	0.04253745
ENSDARG00000030116	susd6	2174.8023	0.20332082	0.08339464	2.43805616	0.01476648	0.04255808
ENSDARG00000100481	smg6	1400.12021	-0.255353063	0.10474666	-2.43781588	0.0147763	0.04258144
ENSDARG00000061204	ddx20	514.64736	-0.370384509	0.15194994	-2.43754291	0.01478746	0.04260866
ENSDARG00000112899	BX088542.1	10.8894004	1.960460617	0.80470444	2.43624927	0.01484045	0.0427564
ENSDARG00000030887	phf23a	521.729722	-0.333593122	0.13693159	-2.43620289	0.01484236	0.04275693
ENSDARG00000071025	dytn	64.1175422	-0.764214843	0.31378195	-2.43549654	0.01487137	0.04283553
ENSDARG00000044433	sned1	598.673222	0.334301289	0.13727265	2.43530883	0.01487909	0.0428528
ENSDARG00000088044	si:ch73-139j3.4	66.0094125	1.011094864	0.41525753	2.43486219	0.01489746	0.04290076
ENSDARG00000101137	znf999	17.9256292	-1.558493744	0.64012594	-2.43466738	0.01490548	0.04291889
ENSDARG00000099660	CU915256.1	16.2865052	1.946254281	0.79950068	2.43433725	0.01491909	0.0429481
ENSDARG00000077875	slc2a5	54.2039259	1.316752744	0.54090519	2.43435038	0.01491855	0.0429481
ENSDARG00000102068	shcbp1	390.322768	-0.4386936	0.18027451	-2.43347552	0.01495465	0.04304548
ENSDARG00000074254	abcb6b	164.504908	0.575322505	0.23643271	2.43334566	0.01496001	0.04305594
ENSDARG00000117370	CABZ01113830.1	23.0247441	1.483528432	0.60970425	2.43319351	0.0149663	0.04306905
ENSDARG00000045372	ngdn	424.395628	-0.377008095	0.15494816	-2.43312399	0.01496917	0.04307233
ENSDARG00000009549	ubr4	10941.8165	0.349615078	0.14369601	2.4330187	0.01497353	0.04307987
ENSDARG00000105464	si:ch211-285c6.6	8.06939184	-2.617415651	1.07581561	-2.43295935	0.01497598	0.04308195
ENSDARG00000030588	slc10a1	117.028454	-0.615590879	0.25303351	-2.43284334	0.01498078	0.04309077

Table S2. DEGs of WT vs. *terfa-/-*

ENSDARG00000018958	nrarpb	377.706725	-0.52598992	0.21621125	-2.43275931	0.01498426	0.04309578
ENSDARG00000068849	fam83ha	1439.84202	0.411965076	0.16934935	2.43263455	0.01498942	0.04310564
ENSDARG00000091271	cenpt	413.56974	-0.5275909	0.21688562	-2.43257668	0.01499182	0.04310754
ENSDARG00000099616	FP236734.1	291.479986	-0.472935092	0.1944249	-2.43248213	0.01499573	0.04311381
ENSDARG00000097863	si:dkeyp-84f3.9	800.954986	-0.363833074	0.14958279	-2.43231907	0.01500249	0.04312824
ENSDARG00000038898	zgc:113691	33.0150271	1.126938074	0.46333574	2.43222784	0.01500626	0.04313412
ENSDARG00000030871	siah1	1374.4295	-0.274301106	0.11279106	-2.43194019	0.01501819	0.0431634
ENSDARG00000036235	zbtb3	335.193508	-0.394282384	0.16216256	-2.43140212	0.01504051	0.04322256
ENSDARG00000101163	BX001023.2	26.0253373	-1.271498293	0.52295853	-2.43135588	0.01504243	0.04322308
ENSDARG00000117550	LO018168.1	610.898322	0.45038601	0.1852561	2.43115349	0.01505084	0.04324223
ENSDARG00000061690	nxpe3	454.763814	0.379244125	0.15600585	2.43096097	0.01505884	0.04325522
ENSDARG00000070358	smim12	595.3822	0.303782239	0.12496177	2.43100139	0.01505716	0.04325522
ENSDARG00000061061	dpagt1	605.91903	0.338124942	0.13911025	2.4306256	0.01507278	0.04328527
ENSDARG00000036966	hsd3b7	831.147945	-0.410514221	0.16889109	-2.43064459	0.01507199	0.04328527
ENSDARG00000038612	pih1d3	50.2364417	1.016868221	0.41838349	2.43046931	0.01507928	0.04329894
ENSDARG00000014081	fam184a	1050.8498	-0.365963151	0.15059114	-2.43017723	0.01509144	0.04332884
ENSDARG00000097938	asb5a	158.67269	0.551723531	0.22705933	2.42986509	0.01510444	0.04336116
ENSDARG00000055644	prss60.2	169.562832	0.639881633	0.26334585	2.42981475	0.01510654	0.04336218
ENSDARG00000098641	nbeal2	465.86763	-0.358094389	0.14738544	-2.42964561	0.01511359	0.04337741
ENSDARG00000097719	CU469514.1	12.7106369	2.035557723	0.83791257	2.42931995	0.01512718	0.04341138
ENSDARG00000089930	iqce	391.572571	-0.388688903	0.16002094	-2.4289877	0.01514105	0.04344617
ENSDARG00000104311	scmh1	854.232951	-0.344147446	0.14169394	-2.42880856	0.01514853	0.04346262
ENSDARG00000116471	BX276101.2	5.66836722	-2.996644306	1.23386289	-2.4286688	0.01515437	0.04347436
ENSDARG00000044615	zak	668.439079	0.431611461	0.17775509	2.42812441	0.01517714	0.04353392
ENSDARG00000089075	si:ch73-173p19.1	1228.6775	0.242530204	0.09988524	2.42808863	0.01517863	0.04353392
ENSDARG00000054560	her15.2	212.028837	-0.649038496	0.26737404	-2.42745518	0.01520517	0.04360498
ENSDARG00000031422	igfbp2b	395.941454	0.450380929	0.18556804	2.42703934	0.01522261	0.04364996
ENSDARG00000090428	ctrb1	3926.74939	-1.556662532	0.64141344	-2.42692534	0.01522739	0.04365864
ENSDARG00000102601	si:dkey-15h8.15	106.315761	-0.618352992	0.25479971	-2.42681988	0.01523182	0.0436663
ENSDARG00000075143	nanp	207.264086	0.509020302	0.20975918	2.42668902	0.01523731	0.04367701
ENSDARG00000088160	CABZ01056629.1	59.2029442	-0.847001106	0.3490848	-2.42634772	0.01525165	0.0437105
ENSDARG00000106560	MCMDC2	92.7025837	-0.743811027	0.30655842	-2.42632719	0.01525251	0.0437105
ENSDARG00000078389	ifi46	51.1829588	0.898982043	0.37059955	2.4257505	0.01527677	0.04377497
ENSDARG00000104833	CU693471.1	17.9188966	-1.383748936	0.57047726	-2.42559875	0.01528316	0.04378823
ENSDARG00000062823	pi4kab	569.88211	0.536911381	0.22137145	2.4253867	0.01529209	0.04380877
ENSDARG00000091625		216.419894	-0.435831539	0.17970855	-2.42521319	0.0152994	0.04381961
ENSDARG00000025326	myl12.2	1200.64298	0.292319051	0.12053221	2.42523596	0.01529844	0.04381961
ENSDARG00000035605	tchp	494.909886	-0.391437408	0.16142025	-2.42495851	0.01531014	0.0438385
ENSDARG00000004952	rsad2	7.06149259	-4.206929603	1.73486549	-2.42493129	0.01531128	0.0438385
ENSDARG00000102053	znf1164	79.1388794	0.774180566	0.319254	2.42496747	0.01530976	0.0438385
ENSDARG00000073723	zgc:194679	250.589453	0.506654472	0.20894729	2.42479556	0.01531701	0.04384984
ENSDARG00000024894	tbx5a	260.580921	0.426576365	0.17593667	2.42460178	0.01532519	0.04386661
ENSDARG00000076182	stat1b	20.1121998	1.436549416	0.59249583	2.42457304	0.0153264	0.04386661
ENSDARG00000021209	kctd9a	172.471721	-0.497355606	0.20515182	-2.42432949	0.01533668	0.04389099
ENSDARG00000101577	si:ch211-172l8.4	68.722646	-0.755249982	0.31154042	-2.42424397	0.0153403	0.04389628
ENSDARG00000037286	si:dkey-30k22.7	104.67242	-0.689662649	0.28451469	-2.42399662	0.01535075	0.04391607
ENSDARG00000055930	zc3h7bb	3875.0373	0.297906091	0.12289755	2.42401977	0.01534977	0.04391607
ENSDARG00000092792	rprm3	96.9326563	-0.905926516	0.37374845	-2.42389371	0.0153551	0.04392346
ENSDARG00000095946	si:ch73-341k19.1	151.82679	-0.491117549	0.20262015	-2.42383376	0.01535763	0.04392566
ENSDARG00000074866	ptpn5	1330.15895	-0.330235972	0.13626071	-2.42355967	0.01536923	0.04394871
ENSDARG00000061921	gltpa	392.144836	-0.376848722	0.15549302	-2.42357324	0.01536865	0.04394871
ENSDARG00000035338	tppp2	767.533406	0.395110709	0.16303296	2.42350211	0.01537166	0.04394872
ENSDARG00000101785	laptm5	106.072687	0.569597791	0.23503339	2.42347601	0.01537277	0.04394872
ENSDARG00000104312	CT573342.2	91.0468226	-0.768180776	0.31703552	-2.42301174	0.01539243	0.04399987
ENSDARG00000074556	ganc	216.844171	0.54691007	0.22574971	2.42263911	0.01540823	0.04403995
ENSDARG00000104441	hspb7	357.611566	0.451223632	0.18626343	2.42250258	0.01541402	0.04405144
ENSDARG00000029524	impdh1b	3829.18801	-0.303377176	0.12524109	-2.42234534	0.01542069	0.04406038
ENSDARG00000103713	cd248a	2713.87997	-0.269291942	0.11116925	-2.42236001	0.01542007	0.04406038

Table S2. DEGs of WT vs. *terfa-/-*

ENSDARG00000019742	tlr4ba	14.0074305	-1.659259983	0.68508805	-2.42196602	0.01543679	0.04410132
ENSDARG00000054746	uggt1	1797.52458	0.340749207	0.14069752	2.42185645	0.01544145	0.04410955
ENSDARG00000063580	ppp1r9ala	1540.45284	0.291692303	0.12044688	2.42175056	0.01544595	0.04411734
ENSDARG00000104276	EIF2AK4	959.552852	0.245042247	0.10118878	2.42163463	0.01545088	0.04412634
ENSDARG00000090699	nlrb5	18.182334	1.33924322	0.55306785	2.42148086	0.01545742	0.04413994
ENSDARG00000075446	naa25	2150.63793	-0.21434294	0.08851995	-2.4214084	0.0154605	0.04414367
ENSDARG00000103429	CR854926.1	5.88879719	-2.982796238	1.23193691	-2.42122483	0.01546831	0.04416037
ENSDARG00000068745	map4l	3341.11536	-0.317251358	0.13103323	-2.42115187	0.01547141	0.04416037
ENSDARG00000076381	slc25a1b	1406.40552	0.404531106	0.16708251	2.42114569	0.01547168	0.04416037
ENSDARG00000067606	rsph4a	139.418154	0.611826929	0.25276092	2.42057566	0.01549595	0.04420936
ENSDARG00000090844	CABZ01054965.1	80.1306108	0.793050442	0.32762648	2.42059327	0.0154952	0.04420936
ENSDARG00000036254	hoxa13b	155.080097	0.551471772	0.22782296	2.42061545	0.01549426	0.04420936
ENSDARG00000059811	smpd5	581.722215	-0.304616746	0.1258427	-2.4206152	0.01549427	0.04420936
ENSDARG00000016044	gins4	407.927636	-0.414465398	0.17123644	-2.42042761	0.01550227	0.04422229
ENSDARG00000013125	dlx1a	544.003877	-0.364328385	0.15053825	-2.42017155	0.01551319	0.04424837
ENSDARG00000075806	kirrel3a	520.46825	-0.440764436	0.18214351	-2.41987451	0.01552586	0.04427944
ENSDARG00000013292	tmem179aa	448.301553	-0.366108442	0.15129784	-2.41978627	0.01552963	0.0442814
ENSDARG00000061977	ppfibp2a	989.590928	-0.302339594	0.12494835	-2.41971658	0.01553261	0.0442814
ENSDARG00000102883	sptbn1	18914.4607	0.298195083	0.12323682	2.41969155	0.01553368	0.0442814
ENSDARG00000059333	si:ch211-285f17.1	1743.76709	0.331268323	0.1369033	2.41972489	0.01553225	0.0442814
ENSDARG00000035993	sumo3b	2212.74621	-0.342869696	0.14172545	-2.41925282	0.01555243	0.04432468
ENSDARG00000061187	cbx5	6553.15917	-0.280655849	0.11600741	-2.41929243	0.01555073	0.04432468
ENSDARG00000116023	CR318589.2	11.8321703	2.07727432	0.85876395	2.41891189	0.01556701	0.04435702
ENSDARG00000096532	si:dkey-202c14.3	24.6663121	-1.230498586	0.50870085	-2.41890412	0.01556734	0.04435702
ENSDARG00000035648	irx4a	454.089956	-0.553099087	0.22869332	-2.41851877	0.01558384	0.04439894
ENSDARG00000098428	asic1c	150.295145	0.571561884	0.23635881	2.41819578	0.01559768	0.04443328
ENSDARG00000074337	cbfa2t2	1136.10244	-0.356425619	0.14746111	-2.41708221	0.01564548	0.04456128
ENSDARG00000076533	nfe2l1b	1005.50046	0.261032532	0.10799564	2.41706553	0.0156462	0.04456128
ENSDARG00000098857	lifra	799.23259	0.449629449	0.18603237	2.41694195	0.01565151	0.04457131
ENSDARG00000098079	rab3gap1	1776.85743	0.276335944	0.11434522	2.41668118	0.01566273	0.04459814
ENSDARG00000073752	CACNA2D1	145.828204	-0.690403193	0.2857297	-2.41628083	0.01567996	0.04464209
ENSDARG00000101127	map1lc3b	3402.70548	-0.357730039	0.14806539	-2.41602748	0.01569088	0.04466805
ENSDARG00000086159	si:ch73-206d17.1	83.7879409	-0.759380643	0.31431574	-2.41598031	0.01569291	0.04466872
ENSDARG00000101930	CU914622.1	49.569524	-2.784052624	1.15237334	-2.41592939	0.01569511	0.04466985
ENSDARG00000070923	zgc:158296	806.112416	0.375124044	0.15531259	2.41528423	0.01572294	0.04474394
ENSDARG00000011360	nfatc2ip	458.371176	-0.420757302	0.17421299	-2.41518903	0.01572705	0.04475037
ENSDARG00000098949	mslna	79.3414509	1.851067492	0.76644045	2.41514849	0.0157288	0.04475037
ENSDARG00000091514	CT737190.1	69.9137965	-0.679742331	0.2814914	-2.41478902	0.01574433	0.04478537
ENSDARG00000069723	zbbx	33.2549424	1.172495579	0.48554958	2.41478034	0.0157447	0.04478537
ENSDARG00000070220	pou3f2a	802.063607	-0.479606476	0.19863799	-2.4144751	0.0157579	0.04481778
ENSDARG00000001776	stim2b	283.873302	0.430752805	0.17843065	2.41411896	0.01577331	0.04485648
ENSDARG00000070425	dll4	674.228102	-0.317340666	0.13146184	-2.41393746	0.01578117	0.04487299
ENSDARG00000069968	pcsk7	1369.16871	0.363511608	0.1505909	2.41390157	0.01578273	0.04487299
ENSDARG00000076473	trim37	1017.63926	-0.35190622	0.14579329	-2.41373392	0.01578999	0.0448885
ENSDARG00000009568	ca10b	166.863173	-0.577215119	0.23915471	-2.41356369	0.01579737	0.04490434
ENSDARG00000037410	med28	401.75711	-0.373755414	0.15486033	-2.41350008	0.01580012	0.04490704
ENSDARG00000045633	asb15a	713.723963	0.359211744	0.14884558	2.41331811	0.01580802	0.04492434
ENSDARG00000069373	crcp	122.335409	-0.635709265	0.26344192	-2.41309079	0.01581788	0.04494723
ENSDARG00000089858	cobll1a	217.162831	-0.52370507	0.21704211	-2.41291923	0.01582533	0.04496325
ENSDARG00000010553	mmgt1	1365.13211	-0.277671454	0.11508839	-2.41267994	0.01583572	0.04498763
ENSDARG00000105038	CABZ01038376.1	10.8073129	2.375219511	0.98451238	2.41258471	0.01583986	0.04499424
ENSDARG00000045308	pola1	1683.95982	-0.404782779	0.16778919	-2.41244856	0.01584577	0.04500591
ENSDARG00000055365	si:dkey-25e12.3	63.5526986	1.16000583	0.48088294	2.41224158	0.01585477	0.04502632
ENSDARG00000069823	proca1	449.808205	-0.322271409	0.13361649	-2.41191345	0.01586905	0.04506172
ENSDARG00000060944	rnf217	164.601957	0.632862787	0.2623984	2.41183938	0.01587227	0.04506572
ENSDARG00000027992	hao2	1095.48899	0.223248979	0.09259337	2.41106864	0.01590586	0.04515591
ENSDARG00000029133	tubgcp3	1467.29996	-0.325793885	0.13516578	-2.41032818	0.01593818	0.04524251
ENSDARG00000094737	si:ch211-12e13.12	67.4974837	-0.718614518	0.2981964	-2.40986991	0.01595821	0.0452942

Table S2. DEGs of WT vs. *terfa-/-*

ENSDARG00000074316	itga1	1447.87346	0.425944688	0.17675625	2.40978572	0.01596189	0.04529948
ENSDARG00000029838	si:dkey-23a13.11	61.6190936	1.180295389	0.48983092	2.40959757	0.01597013	0.04531767
ENSDARG00000101749	zgc:92161	71.7048125	-0.822826184	0.34149097	-2.4095108	0.01597392	0.04532327
ENSDARG00000060805	map1sa	1002.36616	0.267921177	0.11122027	2.40892391	0.01599963	0.04539104
ENSDARG00000099824	nots	26.9723419	1.157016695	0.4804386	2.40825092	0.01602916	0.04546443
ENSDARG00000056019	ttbk1a	1129.34384	-0.309970959	0.12871077	-2.40827523	0.01602809	0.04546443
ENSDARG00000102279	zmiz1b	415.110944	-0.408542023	0.1696561	-2.40805979	0.01603755	0.04548305
ENSDARG00000099891	si:ch211-57n23.1	98.9774928	0.613331306	0.2547562	2.40752255	0.01606117	0.04554483
ENSDARG00000053890	tmem62	712.542429	0.362678369	0.15065795	2.40729654	0.01607111	0.04556782
ENSDARG00000053761	si:dkey-187j14.4	65.1610793	0.82116818	0.34115949	2.40699204	0.01608452	0.04560063
ENSDARG00000054155	pcna	9894.11841	-0.437688161	0.18188941	-2.40634216	0.01611317	0.04567663
ENSDARG00000113372	BX248501.2	47.2534139	-0.899747272	0.37394436	-2.40609931	0.01612388	0.0457018
ENSDARG00000005789	enpp1	1622.96227	0.25749099	0.10702818	2.40582419	0.01613603	0.04573101
ENSDARG00000036179	myo1ea	1372.53442	0.230299322	0.09573035	2.40570859	0.01614113	0.04574027
ENSDARG00000103850	si:cabz01021426.2	201.841347	-0.476177363	0.19798384	-2.40513254	0.0161666	0.04580199
ENSDARG00000069230	zgc:153738	47.1050082	0.969473091	0.40308012	2.40516227	0.01616529	0.04580199
ENSDARG00000071714	si:dkey-20i20.8	217.033691	-0.56445188	0.23470275	-2.40496496	0.01617402	0.04581778
ENSDARG00000029252	ssb	2361.16981	-0.245966906	0.10227918	-2.40485814	0.01617875	0.04582595
ENSDARG00000013685	vsig8b	165.519148	0.634484441	0.26389693	2.40428884	0.01620396	0.04589215
ENSDARG00000036489	MAF1	2463.86506	-0.242936541	0.10105028	-2.40411556	0.01621165	0.04590868
ENSDARG00000036305	phf23b	487.448536	-0.328882877	0.13683179	-2.40355605	0.01623648	0.04597376
ENSDARG00000035871	rpl30	20300.8939	0.248511318	0.10341102	2.40314157	0.01625489	0.04602066
ENSDARG00000021488	cyth1b	583.831604	-0.301866388	0.12563449	-2.40273507	0.01627297	0.0460666
ENSDARG00000009681	med27	434.065172	-0.354254175	0.14745192	-2.40250629	0.01628316	0.04608849
ENSDARG00000026985	lims1	2201.90179	0.218326734	0.09087565	2.40247797	0.01628442	0.04608849
ENSDARG00000062192	tbc1d8b	1403.00982	-0.266280079	0.11084063	-2.40236882	0.01628928	0.046097
ENSDARG00000060452	sdk2b	1404.55096	0.349585681	0.14552494	2.40223897	0.01629506	0.04610812
ENSDARG00000034326	ing3	2018.12134	-0.274171726	0.11413914	-2.40208336	0.01630199	0.04612249
ENSDARG00000061699	sipa1l3	1808.18034	0.419583289	0.174681	2.40199735	0.01630583	0.04612808
ENSDARG00000117510	CU302321.1	9.31880396	6.286481684	2.61731751	2.40187965	0.01631107	0.04613242
ENSDARG00000010831	churc1	666.22173	0.347343453	0.14461214	2.40189689	0.01631031	0.04613242
ENSDARG00000069433	tnk1	944.753715	0.269860546	0.11235658	2.40182247	0.01631362	0.04613439
ENSDARG00000105431	si:dkey-23a13.9	10.4368519	-2.282073796	0.95027792	-2.40148039	0.01632888	0.04617212
ENSDARG00000002609	rnf145a	1177.73491	-0.318887431	0.1327909	-2.4014254	0.01633134	0.04617212
ENSDARG00000055698	pcnx2	1591.0162	-0.357577786	0.14890398	-2.40139851	0.01633254	0.04617212
ENSDARG00000031483	col9a1b	11922.7875	-0.743707186	0.30973193	-2.40113178	0.01634445	0.04620053
ENSDARG00000094683	CR392012.2	4.37702618	-3.449776982	1.43679273	-2.4010262	0.01634917	0.04620336
ENSDARG00000019962	dop1a	1853.69612	0.293973376	0.12243509	2.4010549	0.01634788	0.04620336
ENSDARG00000004988	ppp3ca	2574.8874	-0.274371712	0.11433199	-2.39978087	0.01640489	0.04635556
ENSDARG00000042873	slc25a27	51.8409009	0.732270216	0.30515094	2.39969839	0.01640859	0.04636074
ENSDARG00000043460	wu:fj39g12	78.042648	-0.845412933	0.35238641	-2.39910767	0.01643508	0.04643032
ENSDARG00000004392	hdr	200.582508	-0.484101382	0.20179367	-2.39899194	0.01644028	0.04643972
ENSDARG00000102024	chmp6b	2309.03666	0.230414008	0.09605053	2.39888319	0.01644516	0.04644823
ENSDARG00000002840	si:dkey-28b4.8	3716.18899	-0.311797579	0.12999185	-2.39859326	0.01645819	0.04647974
ENSDARG00000035579	si:dkey-174n20.1	249.992948	-0.532941532	0.22219577	-2.39852243	0.01646137	0.04648345
ENSDARG00000098805	vwa11	233.507325	1.32459379	0.55228566	2.39838526	0.01646754	0.04649558
ENSDARG00000007418	chmp4c	209.670044	-0.50144247	0.20914102	-2.39762845	0.01650159	0.04658645
ENSDARG00000015427	hdac1	12237.5193	-0.344226173	0.14357781	-2.39748866	0.01650789	0.04659894
ENSDARG00000024560	slc4a1b	607.111366	0.424711033	0.17717207	2.39716691	0.0165224	0.04663458
ENSDARG00000042214	tmem243a	62.8073849	0.860375686	0.35904273	2.39630445	0.01656133	0.04673916
ENSDARG00000019717	pbx2	1822.98452	0.221877109	0.09259593	2.39618633	0.01656667	0.04674892
ENSDARG00000052782	glrba	234.951872	-0.60931289	0.25434158	-2.39564802	0.01659101	0.04680742
ENSDARG00000074125	znf1161	83.8891217	-0.684482104	0.28571937	-2.39564474	0.01659116	0.04680742
ENSDARG00000117363	CABZ01071845.2	57.3543995	0.748581712	0.31251421	2.39535255	0.01660439	0.04683942
ENSDARG00000102267	znf750	251.990852	0.426960762	0.17824861	2.39531051	0.0166063	0.04683948
ENSDARG00000087260	mtss1lb	745.459668	-0.455706891	0.19032559	-2.39435429	0.01664966	0.04695646
ENSDARG00000039385	dlg4b	1293.67732	-0.363989183	0.1520623	-2.39368463	0.01668008	0.04703693
ENSDARG00000096867	clmn	506.548444	-0.325009673	0.1357929	-2.39342167	0.01669205	0.04706532

Table S2. DEGs of WT vs. *terfa-/-*

ENSDARG00000116324	BX005461.4	2.85724904	-4.614474965	1.9281	-2.39327574	0.01669869	0.0470787
ENSDARG00000104572	si:dkey-35h6.1	1481.02961	0.493538158	0.20627277	2.39264817	0.01672727	0.04715395
ENSDARG00000099917	znf1005	50.0699177	-0.969827698	0.40544246	-2.39202305	0.01675579	0.04722898
ENSDARG00000074589	rin2	960.446793	0.351995227	0.14715902	2.39193785	0.01675968	0.04723459
ENSDARG00000040971	zgc:92606	1552.19724	-0.406617012	0.1700008	-2.39185351	0.01676353	0.04724009
ENSDARG00000101631	etfa	4746.71591	0.24191922	0.10115004	2.39168678	0.01677115	0.04725619
ENSDARG00000039881	cemip	1704.61711	0.271724451	0.11361612	2.39160119	0.01677506	0.04726186
ENSDARG00000101260	GPATCH2	1445.62124	-0.3099744	0.12961568	-2.39148841	0.01678021	0.04727102
ENSDARG00000102776	cxcl19	5.06582094	3.961180954	1.65640711	2.39142957	0.0167829	0.04727324
ENSDARG00000087178	scpp9	39.0019814	-3.110439918	1.30080645	-2.39116275	0.01679511	0.04730225
ENSDARG00000117624	BX545917.5	3.31377808	4.006138903	1.67553284	2.39096412	0.0168042	0.04731471
ENSDARG00000100057	ppfibp1b	1945.02468	0.294430849	0.12314432	2.39094131	0.01680524	0.04731471
ENSDARG00000104274	tmem200b	193.239716	-0.456734338	0.19102244	-2.3909984	0.01680263	0.04731471
ENSDARG00000109737	zgc:171759	2.53489082	-4.585384123	1.91791949	-2.39081159	0.01681118	0.04732606
ENSDARG00000104716	zgc:91849	401.604907	0.448746058	0.18770824	2.39065721	0.01681825	0.04733524
ENSDARG00000104960	si:ch211-11n16.3	17.5629263	-1.94457356	0.8133925	-2.39069523	0.01681651	0.04733524
ENSDARG00000058736	gabra6b	75.0982685	-0.651415043	0.27249271	-2.3905779	0.01682188	0.04734011
ENSDARG00000037879	lfng	2395.87113	-0.294261592	0.12310981	-2.39023667	0.01683752	0.04737875
ENSDARG00000102296	zgc:175177	20.3189849	-1.385099209	0.57950517	-2.39014125	0.01684189	0.0473857
ENSDARG00000070430	chtopb	52.1571993	0.751275768	0.31435424	2.38990184	0.01685288	0.04741123
ENSDARG00000024101	adra1d	196.155506	-0.430084114	0.17996406	-2.38983332	0.01685602	0.04741471
ENSDARG00000040027	osbpl10b	733.188815	0.315999912	0.13223526	2.38967977	0.01686307	0.04742917
ENSDARG00000061051	brinp3a.2	314.656831	-0.394902372	0.16527938	-2.38930208	0.01688042	0.04746722
ENSDARG00000076559	rb1cc1	2695.50375	-0.210425229	0.08806972	-2.38930271	0.01688039	0.04746722
ENSDARG00000003779	ctnnd2b	2393.75138	-0.369793283	0.15478508	-2.38907579	0.01689082	0.0474911
ENSDARG00000114841	CNNM4	143.389006	-0.506000658	0.21180631	-2.38897824	0.0168953	0.04749833
ENSDARG00000034785	dachb	420.208238	-0.353493545	0.14801524	-2.38822395	0.01693002	0.04759056
ENSDARG00000115651	LO017917.1	548.498189	-0.350526595	0.14677577	-2.38817754	0.01693216	0.04759118
ENSDARG00000075209	p4htm	304.755304	-0.423587976	0.17738009	-2.38802437	0.01693922	0.04760564
ENSDARG00000055022	pds5a	4298.71431	-0.261205102	0.10940218	-2.38756769	0.01696028	0.04765944
ENSDARG00000070455	tmem245	593.781194	-0.390684391	0.16368171	-2.38685426	0.01699323	0.04774662
ENSDARG00000077847	olfm2a	2114.84323	-0.393885891	0.16503162	-2.38672989	0.01699898	0.04775738
ENSDARG00000092725	si:dkey-188i13.8	17.3756265	-1.589848016	0.66618955	-2.38647998	0.01701053	0.04778445
ENSDARG00000096269	CR388364.1	4.64099409	3.023092217	1.26684589	2.3863141	0.01701821	0.04780061
ENSDARG00000097040	CT025745.1	64.0167831	-1.101162071	0.46150873	-2.38600487	0.01703253	0.04783432
ENSDARG00000094433	caspbl	47.2063346	3.383945394	1.41826716	2.38597176	0.01703406	0.04783432
ENSDARG00000042707	cx30.3	853.458121	0.328415429	0.13769244	2.38513763	0.01707273	0.04793335
ENSDARG00000060018	si:ch1073-44g3.1	1013.67258	-0.348654488	0.14617853	-2.38512794	0.01707318	0.04793335
ENSDARG00000032623	rcan3	977.158274	-0.344189806	0.14433777	-2.38461356	0.01709707	0.047995
ENSDARG00000069274	ighmbp2	435.810199	-0.332962432	0.13965432	-2.38419008	0.01711676	0.04804484
ENSDARG00000045836	mapk11	582.689653	-0.493130181	0.20683845	-2.38413209	0.01711946	0.04804698
ENSDARG00000079716	sin3aa	2324.47503	-0.267806221	0.11233157	-2.38406899	0.01712239	0.0480498
ENSDARG00000091304	card19	468.702892	-0.362447788	0.15204555	-2.38381057	0.01713442	0.04807812
ENSDARG00000056976	ifit16	47.1388507	0.870048654	0.36499049	2.38375703	0.01713691	0.04807969
ENSDARG00000006758	fam234b	1500.10982	-0.339148601	0.14231022	-2.383164	0.01716455	0.04814091
ENSDARG00000090953	igsf5b	40.7166154	0.977691464	0.41024593	2.38318385	0.01716362	0.04814091
ENSDARG00000058828	ccdc51	426.612222	0.339102372	0.14228661	2.38323457	0.01716126	0.04814091
ENSDARG00000114893	znf1038	27.0926481	-1.118266547	0.46929425	-2.38286865	0.01717832	0.04817412
ENSDARG00000096850	BX957346.1	781.722138	0.274297084	0.11512368	2.38262967	0.01718948	0.04819996
ENSDARG00000086603	zgc:136963	47.4079713	-1.084295172	0.45529468	-2.3815239	0.01724117	0.04833946
ENSDARG00000011026	zgc:92907	197.817245	-0.482039502	0.20242407	-2.38133486	0.01725002	0.04835882
ENSDARG00000076090	jakmip1	459.705105	-0.389063404	0.16339061	-2.38118588	0.017257	0.04837293
ENSDARG00000096359	eppk1	1104.17817	0.532502026	0.2236523	2.38093699	0.01726867	0.04840017
ENSDARG00000079611	sema4c	1690.15876	-0.297286048	0.12488739	-2.3804328	0.01729232	0.04845433
ENSDARG00000004232	dlb	1311.56873	-0.975789088	0.40993362	-2.38035878	0.01729579	0.04845433
ENSDARG00000069471	mhc1zca	177.169054	-0.440102349	0.18488748	-2.38037945	0.01729482	0.04845433
ENSDARG00000117203	FO704764.1	2.4392675	-4.59342414	1.92969439	-2.38038944	0.01729435	0.04845433
ENSDARG00000005941	clul1	3157.97623	0.202976482	0.08527584	2.38023428	0.01730163	0.04846436

Table S2. DEGs of WT vs. *terfa-/-*

ENSDARG00000096769	CR932077.1	11.1004889	1.631726933	0.6855421	2.38019946	0.01730327	0.04846436
ENSDARG00000068868	nap1l4b	2147.04682	-0.25213975	0.10594104	-2.38000083	0.0173126	0.04848503
ENSDARG00000101524	znf1080	4.66215724	2.5337151	1.06467687	2.37979725	0.01732217	0.04850636
ENSDARG00000029234	stxbp5b	991.490986	0.300453437	0.12626046	2.37963207	0.01732993	0.04851717
ENSDARG00000038566	gnptg	361.953546	-0.389281926	0.16358659	-2.37966893	0.0173282	0.04851717
ENSDARG00000087303	cebpd	5253.44334	0.813253815	0.34177712	2.37948581	0.01733681	0.04853096
ENSDARG00000115752	CABZ01028768.1	2.94093981	4.186703379	1.75957627	2.37938158	0.01734171	0.04853922
ENSDARG00000033567	fkbp1ab	294.450857	0.546598873	0.22974877	2.37911561	0.01735423	0.04856879
ENSDARG00000102127	NHLRC3	1068.10916	0.324382028	0.13636053	2.3788558	0.01736647	0.04859756
ENSDARG00000012881	slc4a1a	1500.06576	0.998261699	0.41965923	2.37874356	0.01737176	0.04860689
ENSDARG00000040059	gbp	727.800641	-0.478866199	0.20132075	-2.37862313	0.01737743	0.0486173
ENSDARG00000075177	ribc2	56.151766	0.910777316	0.38292651	2.37846507	0.01738488	0.04863267
ENSDARG00000105746	LO018124.1	49.1409485	-0.988433026	0.41562192	-2.37820233	0.01739728	0.04866186
ENSDARG00000031983	six4b	514.769495	0.342482739	0.14402072	2.37801011	0.01740635	0.04868176
ENSDARG00000015627	rgs6	381.45202	-0.363063921	0.15271092	-2.37745884	0.01743239	0.0487491
ENSDARG00000014995	jagn1b	647.772651	0.362747558	0.15258096	2.37741033	0.01743468	0.04875003
ENSDARG00000104423	BX950868.2	10.7973296	-2.068300021	0.8700672	-2.37717273	0.01744592	0.04877596
ENSDARG00000058494	dnaja3a	1640.31935	0.229418977	0.09651502	2.37702874	0.01745273	0.04878403
ENSDARG00000078622	scpp5	628.556347	-0.618548295	0.26021717	-2.37704643	0.01745189	0.04878403
ENSDARG00000097388	BX571853.1	7.7206701	-2.474596363	1.04110263	-2.37689954	0.01745884	0.04878836
ENSDARG00000015890	mafa	673.731729	0.267392628	0.11249556	2.37691715	0.01745801	0.04878836
ENSDARG00000093124	scpp8	14.4060013	1.768470976	0.74403305	2.37687155	0.01746017	0.04878836
ENSDARG00000042742	stx6	1584.92044	-0.247799491	0.1042587	-2.37677529	0.01746472	0.04879561
ENSDARG00000059751	abtb2a	309.644701	0.614217252	0.25845513	2.37649477	0.01747801	0.04881078
ENSDARG00000117421	FP101883.1	2.88863025	4.559900508	1.91873523	2.37651368	0.01747711	0.04881078
ENSDARG00000075974	si:dkey-258f14.3	141.39308	0.542908201	0.22843824	2.3766082	0.01747264	0.04881078
ENSDARG00000026198	enoph1	1186.05153	-0.253043162	0.10647564	-2.37653584	0.01747606	0.04881078
ENSDARG00000115806	CABZ01043506.1	179.462138	0.653612994	0.27507338	2.37614048	0.0174948	0.04885218
ENSDARG00000105452	plxna1a	2050.08125	0.398657837	0.16778185	2.37604859	0.01749916	0.04885886
ENSDARG00000071774	eef1e1	994.672539	-0.320131427	0.13476229	-2.37552678	0.01752392	0.0489225
ENSDARG00000040747	tm4sf4	836.065522	0.550623454	0.23181224	2.37529932	0.01753473	0.04894717
ENSDARG00000014582	exoc3	1985.53508	0.247725849	0.10430627	2.37498512	0.01754966	0.04898089
ENSDARG00000093316	adgrf8	135.454196	0.676985947	0.28505125	2.37496222	0.01755075	0.04898089
ENSDARG00000059805	slc25a38a	85.0838326	1.208610124	0.50893848	2.37476662	0.01756005	0.04900135
ENSDARG00000025912	bscl2l	500.408832	-0.409778337	0.1725589	-2.37471572	0.01756247	0.04900261
ENSDARG00000040021	ebag9	852.313135	0.314533405	0.13246917	2.37438947	0.017578	0.04904043
ENSDARG00000058868	apc	9747.87336	-0.308958173	0.13012848	-2.37425476	0.01758441	0.04905282
ENSDARG00000097262	AL808129.1	179.102299	-0.577541319	0.24328036	-2.37397429	0.01759778	0.04908458
ENSDARG00000006636	slitrk2	379.292683	-0.445158123	0.18753126	-2.3737809	0.017607	0.04910479
ENSDARG00000062904	idua	430.580661	0.353416818	0.14889357	2.37362047	0.01761465	0.04911141
ENSDARG00000073737	suds3	903.937748	-0.304741066	0.12838349	-2.37367808	0.0176119	0.04911141
ENSDARG00000005783	ncanb	1390.16152	0.488791327	0.20592768	2.37360679	0.0176153	0.04911141
ENSDARG00000117235	CR293531.4	6.57178375	3.773456663	1.58981808	2.37351476	0.01761969	0.04911814
ENSDARG00000102556	nfat5b	235.555826	-0.527528224	0.22227086	-2.37335753	0.01762719	0.04913355
ENSDARG00000069241	ttc32	193.580623	-0.509476162	0.21467139	-2.37328389	0.01763071	0.04913784
ENSDARG00000096707	BX469896.1	23.5511421	-1.360917535	0.57365565	-2.37235969	0.01767488	0.04925541
ENSDARG00000013095	gclc	1638.37714	0.200144888	0.08437103	2.37219917	0.01768256	0.04927129
ENSDARG00000013266	colec11	122.64632	0.745488476	0.31434487	2.37156242	0.01771306	0.04935074
ENSDARG00000102138	foxa1	519.203423	0.356694034	0.15041284	2.37143339	0.01771924	0.04936244
ENSDARG00000076933	aldh1a3	393.967214	0.308181765	0.12996886	2.37119691	0.01773058	0.04938851
ENSDARG00000054122	tmem30b	709.556838	0.327119918	0.13796312	2.37106786	0.01773677	0.04940022
ENSDARG00000090834	epor	85.2469579	-0.770991811	0.3253144	-2.3699898	0.01778858	0.04953894
ENSDARG00000061915	git2a	973.794093	-0.244237825	0.10307356	-2.36954876	0.01780981	0.04959251
ENSDARG00000088750	otogl	298.862449	0.418697313	0.17671073	2.36939379	0.01781727	0.04960774
ENSDARG00000115349	nat14	748.86521	-0.266731659	0.1125786	-2.36929271	0.01782214	0.04961574
ENSDARG00000099842	CU138533.1	57.2205927	-0.792122506	0.33436766	-2.36901654	0.01783546	0.04964725
ENSDARG00000014057	gabrr2b	58.0982463	-0.765033519	0.32302625	-2.36833238	0.01786848	0.04973359
ENSDARG00000076980	ppp1r16a	211.707134	0.440464122	0.18600692	2.36799864	0.0178846	0.0497729

Table S2. DEGs of WT vs. *terfa-/-*

ENSDARG00000103308	mst1	1361.09508	0.428549456	0.18097894	2.36795204	0.01788685	0.0497736
ENSDARG00000000472	cntn2	1579.68835	-0.388871638	0.16422743	-2.36788478	0.01789011	0.04977707
ENSDARG00000068143	slc39a6	1439.08404	0.321388076	0.13574193	2.36764044	0.01790192	0.04980438
ENSDARG00000094534	CR786571.2	15.7871036	1.380189112	0.58299955	2.3673931	0.01791389	0.0498321
ENSDARG00000043865	timm9	677.93167	-0.310415597	0.13112621	-2.36730401	0.01791821	0.04983852
ENSDARG00000054343	slc7a8b	48.754769	-0.842726178	0.35607602	-2.36670302	0.01794733	0.04990277
ENSDARG00000055952	olfcx2	3.10840544	-4.568987955	1.9305093	-2.36672672	0.01794618	0.04990277
ENSDARG00000099397	cfap410	202.855922	-0.47341065	0.20002695	-2.36673431	0.01794581	0.04990277
ENSDARG00000099323	dlg2	707.95139	-0.45236074	0.19116156	-2.36637917	0.01796304	0.04994086
ENSDARG00000105714	CABZ01083442.1	84.5456045	0.639385003	0.27020667	2.36628133	0.01796779	0.04994847
ENSDARG00000069715	foxi2	87.0770222	-0.738983764	0.31232988	-2.36603605	0.01797969	0.04997599
ENSDARG00000062799	baiap2a	1330.89773	0.309092636	0.13064325	2.36592896	0.0179849	0.04998486
ENSDARG00000061398	epb41l4b	324.304167	0.369285718	0.15608795	2.36588227	0.01798716	0.04998557

Table S3. DEGs of WT vs. *terfa+/-* ∩ *terfa-/-*

GeneID	gene name	log2FC_homo_WT	padj_homo_WT	log2FC_hybrid_WT	padj_hybrid_WT
ENSDARG00000011373	mknk2a	0.448141571	0.02628168	-0.410767596	0.04865351
ENSDARG00000018342	ggt1b	0.87979633	0.0000342	-0.591510187	0.010822403
ENSDARG00000020890	tmod4	0.294049924	0.023871215	-0.318132356	0.01143236
ENSDARG00000033599	pdia8	-0.421306676	0.011574399	0.417524578	0.007648789
ENSDARG00000033655	stmn1b	-0.429961001	0.000850078	0.292682869	0.027449576
ENSDARG00000035852	cart3	1.29204077	0.000606181	-1.028814028	0.009322671
ENSDARG00000035909	mfsd2ab	-0.299035953	0.009941627	0.271394828	0.016756085
ENSDARG00000036094	pias1a	-0.383585256	0.025933251	0.334416019	0.049459463
ENSDARG00000040255	ephx2	0.396321092	0.031775262	-0.370106604	0.048090754
ENSDARG00000045633	asb15a	0.359211744	0.044924335	-0.504229873	0.002728191
ENSDARG00000045887	mmp30	0.712843896	0.00000141	-0.497958337	0.000849939
ENSDARG00000052207	c3a.3	1.405186509	1.29E-09	-0.880292331	0.000123541
ENSDARG00000054560	her15.2	-0.649038496	0.043604983	0.622713747	0.040991082
ENSDARG00000056742	crmp1	-0.450494311	0.026513101	0.469974911	0.016618317
ENSDARG00000068621	si:ch211-181d7.3	0.701709133	0.01509998	-0.610409131	0.040038597
ENSDARG00000074635	abca1a	0.345566547	0.014974168	-0.457594559	0.000457032
ENSDARG00000090371	si:dkey-46i9.6	0.409643788	0.020955626	-0.401781829	0.026844073
ENSDARG00000096037	si:ch211-214j24.14	0.473049792	0.004182943	-0.534898089	0.000963054
ENSDARG00000101318	pcdh2ab9	-0.54113547	0.040856863	0.563289641	0.022657708
ENSDARG00000102453	slc1a2b	-0.401920927	0.034064931	0.442430303	0.015650238
ENSDARG00000103044	ASS1	0.520321828	0.004320452	-0.463768775	0.011414641
ENSDARG00000105731	CR788316.1	2.101077235	1.05E-12	-0.785966065	0.017363712
ENSDARG00000105829	CR788316.4	2.039083946	0.00000038	-1.119287684	0.012360118
ENSDARG00000109626	si:ch211-226h7.3	0.573006983	0.040856863	-0.580141107	0.040453011
ENSDARG00000117646	CABZ01072309.2	0.444315165	0.001210406	-0.374708551	0.006127183
ENSDARG00000000380	pde6a	-2.41767745	5.38E-29	-1.110328872	1.13E-08
ENSDARG00000000460	nitr2b	3.123130134	0.040204657	3.405230212	0.027845749
ENSDARG00000000551	slc1a4	0.656868018	0.009344057	0.722040976	0.002671283
ENSDARG00000000606	dnah6	1.677255309	8.8E-15	0.685813405	0.003140681
ENSDARG00000000796	nr4a1	0.528224473	0.003382643	0.5497951	0.001335257
ENSDARG00000001014	myh9b	0.693650183	8.43E-10	0.307950682	0.009900866
ENSDARG00000001437	slc2a1a	-1.685404387	1.33E-08	-0.802649965	0.007049544
ENSDARG00000001442	inppl1b	-0.522796028	0.004077586	-0.661654474	0.0000707
ENSDARG00000001463	tdh2	-2.058345608	1.56E-19	-1.083855653	0.000000661
ENSDARG00000001686	uckl1a	-1.423272201	0.000000662	-0.701273728	0.013479237
ENSDARG00000001721	oc90	2.491490231	0.001860624	1.785847031	0.030862125
ENSDARG00000001818	c3b.2	-0.474410105	0.009177899	-0.607078917	0.000289605
ENSDARG00000001829	zgc:112982	-0.435909874	0.0000305	-0.246427199	0.024234648
ENSDARG00000001889	tuba1a	0.499490997	0.007442628	1.030287973	7.76E-11
ENSDARG00000001897	man2b1	0.450982283	0.007041835	0.389144068	0.020281477
ENSDARG00000001913	palmda	-1.170226211	3.8E-09	-0.967039333	0.000000154
ENSDARG00000001968	dock5	1.324783642	2.66E-12	0.483490931	0.020762042
ENSDARG00000001975	hsd11b2	1.146216474	7.93E-09	0.466656863	0.031406307
ENSDARG00000002172	aplnra	-0.920436881	6.54E-11	-0.597881254	0.00000875
ENSDARG00000002213	invs	1.139450238	0.00000389	0.768823787	0.002089128
ENSDARG00000002231	plpp2a	1.319403927	8.35E-10	0.581789069	0.011636475
ENSDARG00000002241	kcna2a	-1.05439618	0.00309004	-0.720297692	0.044069852
ENSDARG00000002293	si:ch211-197g15.6	1.035439282	0.000824653	1.037074704	0.00043227
ENSDARG00000002298	ankrd22	0.906942139	0.009344057	0.737906091	0.039599133
ENSDARG00000002385	atm	0.989382843	7.26E-09	0.61579607	0.000305964
ENSDARG00000002391	tlcd1	0.769701579	0.0000362	0.451932593	0.022281018
ENSDARG00000002396	cry-dash	0.751845261	0.00000108	0.683845726	0.00000235
ENSDARG00000002401	gale	0.379913718	0.010994569	0.352074543	0.01729968
ENSDARG00000002403	nusap1	-1.527204238	9.9E-12	-0.588092055	0.013490418

Table S3. DEGs of WT vs. *terfa+/-* ∩ *terfa-/-*

ENSDARG00000002634	b4galt1	1.300913735	5.08E-16	0.833325016	7.95E-08
ENSDARG00000002635	FAM184A	-0.769154658	0.00000275	-0.391390943	0.02337131
ENSDARG00000002710	ncl	-0.434960979	0.0000181	-0.295265904	0.003812993
ENSDARG00000002732	spon2b	-0.594317256	0.000339185	-0.348616965	0.045506295
ENSDARG00000002745	tdh	0.951684275	1.86E-11	0.634802865	0.00000334
ENSDARG00000002748	sema6d	-1.224527321	3.41E-11	-0.447958082	0.02410324
ENSDARG00000002758	dedd1	0.475813324	0.011059991	0.567750431	0.001171558
ENSDARG00000002790	ap2m1a	0.279621718	0.00768527	0.276229487	0.006708311
ENSDARG00000002792	arcn1a	0.370796284	0.000802754	0.349636808	0.001000704
ENSDARG00000002847	fndc1	-1.037963175	0.000628706	-0.717467345	0.019443184
ENSDARG00000002912	adipor1a	-0.431132603	0.0000469	-0.256059612	0.019590654
ENSDARG00000002917	gls2b	-0.96560943	0.007692915	-1.194618886	0.000378084
ENSDARG00000003021	hdac8	0.374923199	0.022528485	0.522895936	0.00044849
ENSDARG00000003061	cd276	0.618207053	0.000000491	0.399018444	0.001234762
ENSDARG00000003077	gpx9	-1.524235399	4.48E-10	-1.83018279	8.23E-17
ENSDARG00000003084	spire2	-0.640571156	0.002270051	-0.477532467	0.023440863
ENSDARG00000003203	rhcga	4.354994318	1.04E-30	3.788549837	1.95E-28
ENSDARG00000003208	pomk	0.419239137	0.007872219	0.439176206	0.003723543
ENSDARG00000003216	anxa2a	2.643242754	1.08E-28	1.064970394	0.00000701
ENSDARG00000003281	pik3ip1	-0.467501697	0.014321374	-0.490778894	0.007606379
ENSDARG00000003311	pank2	-0.698008688	6.4E-09	-0.296208378	0.020333337
ENSDARG00000003320	fam91a1	0.305082257	0.024651617	0.292007589	0.030360175
ENSDARG00000003420	chrna5	-0.762959724	0.0000257	-0.449283641	0.014070571
ENSDARG00000003429	hnrnpdl	-0.659128087	0.000000293	-0.322851978	0.017204485
ENSDARG00000003462	fech	1.429607076	0.00000102	0.931760133	0.001810587
ENSDARG00000003475	sult1st5	1.419711139	0.002453	1.337891494	0.003622259
ENSDARG00000003526	psma5	-0.342559684	0.002554624	-0.244684419	0.036311101
ENSDARG00000003564	dohh	0.846275166	2.84E-08	0.466549605	0.003336504
ENSDARG00000003570	hsp90b1	0.993314815	1.07E-23	0.919029228	8.7E-25
ENSDARG00000003599	rpl3	0.310183879	0.004814523	0.24189136	0.030954918
ENSDARG00000003631	clockb	-0.493712648	0.000592042	-0.33875043	0.021371298
ENSDARG00000003641	tfg	0.270856292	0.03057084	0.342419853	0.003494303
ENSDARG00000003811	plxnb2a	0.616980822	1.44E-08	0.304059546	0.007608662
ENSDARG00000003820	nr1d2a	0.485926201	0.02244067	0.715914003	0.000187675
ENSDARG00000003829	galnt2	0.37692864	0.000067	0.258659235	0.007020414
ENSDARG00000003952	pfn2	0.315576793	0.003153314	0.299816268	0.003853938
ENSDARG00000003961	parp3	1.71876914	3.61E-46	0.287975178	0.045970101
ENSDARG00000003963	traf4a	0.281594659	0.021202332	0.264684283	0.029426155
ENSDARG00000003973	chordc1a	0.873709962	0.0000226	1.022484536	4.72E-08
ENSDARG00000004015	adcyap1a	-0.860182526	0.002596089	-0.657605038	0.020302066
ENSDARG00000004034	arhgdig	0.706692282	4.74E-12	0.298135798	0.005778512
ENSDARG00000004161	ik	-0.359886717	0.00011778	-0.213623453	0.028933047
ENSDARG00000004169	stmn1a	-1.132833113	3.23E-09	-0.449571919	0.029426155
ENSDARG00000004173	copa	0.411576826	0.000147919	0.265040715	0.018086356
ENSDARG00000004246	slit2	0.352735325	0.012362546	0.354748783	0.009573233
ENSDARG00000004251	dhfr	-0.947778078	3.49E-08	-0.737785291	0.00000453
ENSDARG00000004392	hdr	-0.484101382	0.046439718	-0.51595658	0.029343173
ENSDARG00000004447	aplnr2	1.093681593	0.000623803	1.095856086	0.000314827
ENSDARG00000004470	prkcsh	0.302213702	0.020286574	0.269549587	0.039606104
ENSDARG00000004517	ppat	0.527867899	0.0000487	0.291600589	0.035704946
ENSDARG00000004561	prkcg	-1.259714412	0.001718787	-1.040596552	0.007666782
ENSDARG00000004592	gpr22a	-0.546639966	0.001192555	-0.383675327	0.024570427
ENSDARG00000004632	egln1b	0.574029696	0.0000761	0.423371475	0.003753377
ENSDARG00000004635	epha7	0.312449535	0.011288116	0.353596666	0.002418496
ENSDARG00000004643	cdhr1a	-2.514884729	2.79E-28	-0.784826919	0.000325025

Table S3. DEGs of WT vs. *terfa+/-* ∩ *terfa-/-*

ENSDARG00000004658	zgc:101810	1.495395259	3.3E-41	0.717198142	1.94E-11
ENSDARG00000004680	dnajb6a	-0.53005991	0.0000545	-0.325037149	0.016318513
ENSDARG00000004748	zgc:100868	0.639096289	1.01E-09	0.485853347	0.00000101
ENSDARG00000004754	hspa4a	0.648296591	2.08E-08	0.684269977	7.39E-11
ENSDARG00000004763	hhla2a.2	-1.674438793	0.000000164	-1.942489847	1.67E-11
ENSDARG00000004771	ankrd10b	-0.883362435	4.46E-10	-0.527791295	0.0001212
ENSDARG00000004782	fgfr3	1.005111254	1.37E-20	0.896694052	7.04E-20
ENSDARG00000004796	mgrn1b	-0.378121659	0.001469965	-0.323396538	0.005631574
ENSDARG00000004823	asz1	1.880403445	0.000693671	2.402130118	0.00000199
ENSDARG00000004836	dnajc5ab	-1.023123916	9.19E-23	-0.27701673	0.014445946
ENSDARG00000004840	rassf1	-0.727668056	0.000000114	-0.495130003	0.000190739
ENSDARG00000004932	anos1b	0.855016282	1.13E-13	0.471298863	0.0000409
ENSDARG00000004964	cyp4t8	-0.918520122	0.001800035	-0.620965746	0.039321449
ENSDARG00000005015	tspy	-0.834651636	1.47E-12	-0.274264947	0.032343407
ENSDARG00000005104	limk2	0.478819658	0.0000413	0.368018211	0.001389745
ENSDARG00000005115	calub	0.498277264	0.00026506	0.375518147	0.006142484
ENSDARG00000005122	atp2a2b	0.523550379	0.00000004	0.450984682	0.000000455
ENSDARG00000005176	zgc:101040	0.541436544	0.00098741	0.509509782	0.001319484
ENSDARG00000005220	pdcd6	0.436056991	0.000576465	0.284553744	0.031490754
ENSDARG00000005284	fbxl15	0.616767461	0.004944099	0.484316015	0.031174811
ENSDARG00000005315	celf1	-0.29929568	0.000533395	-0.203575809	0.021642137
ENSDARG00000005372	camk4	-0.666398538	0.000534861	-0.432507756	0.027845749
ENSDARG00000005392	slc5a5	1.809015734	0.002522953	1.366116542	0.0305126
ENSDARG00000005454	tacc3	-1.048570891	0.000000144	-0.433301909	0.045962991
ENSDARG00000005468	irf2bp1	0.254621154	0.013800684	0.209303861	0.047457067
ENSDARG00000005544	btbd3a	0.690661296	0.038268978	0.790603366	0.013376332
ENSDARG00000005619	nek2	-1.481757871	0.000000038	-0.814941034	0.00187029
ENSDARG00000005625	maco1a	-0.328547082	0.010590506	-0.284285839	0.026697851
ENSDARG00000005675	sec61a1l	0.341669162	0.015665283	0.325607434	0.019783224
ENSDARG00000005762	col14a1a	0.701106372	0.000000101	0.41251074	0.00205312
ENSDARG00000005774	ddx3xb	-0.500061131	0.000078	-0.417089418	0.000677738
ENSDARG00000005783	ncanb	0.488791327	0.049111413	0.808308743	0.000246719
ENSDARG00000005823	slc39a10	0.363593752	0.002475453	0.434876689	0.0000827
ENSDARG00000005840	got2b	0.461800329	0.000000351	0.232811753	0.01493606
ENSDARG00000005908	clk4b	-0.737819563	0.000000134	-0.397148931	0.005436617
ENSDARG00000005945	sart1	-0.558004511	0.00000207	-0.332272843	0.005559199
ENSDARG00000005972	ric8b	0.349626407	0.001069281	0.275690807	0.010046828
ENSDARG00000005993	prc1b	-1.067556083	9.55E-11	-0.568099592	0.000404398
ENSDARG00000006019	tktb	0.593589039	2.42E-12	0.35812076	0.0000147
ENSDARG00000006094	igf2r	0.76365118	0.0000983	0.582938678	0.00277967
ENSDARG00000006112	myof	0.989595269	2.29E-13	0.460247683	0.000921703
ENSDARG00000006125	csnk1db	0.274198992	0.00961199	0.291121633	0.004113904
ENSDARG00000006169	lrrk2	0.922274321	0.00000259	1.083059981	9.52E-10
ENSDARG00000006200	eif4g1a	0.330420489	0.001714455	0.251304789	0.018678719
ENSDARG00000006219	gnl3	-0.32793036	0.019922756	-0.389394287	0.003358462
ENSDARG00000006235	brms1la	-1.049987381	3.84E-10	-0.544619692	0.001158368
ENSDARG00000006242	ptp4a1	-0.685059763	0.000000051	-0.379777988	0.003069248
ENSDARG00000006288	prdm5	0.883874676	7.01E-08	0.672435499	0.000024
ENSDARG00000006300	psmd9	0.56335304	0.006372874	0.651098237	0.000746265
ENSDARG00000006314	itgav	0.929063419	1.98E-18	0.38968586	0.000314966
ENSDARG00000006413	rpl38	1.511494476	3.5E-27	1.177305004	4.09E-20
ENSDARG00000006422	esyt3	1.477383726	3.17E-16	0.804835453	0.00000744
ENSDARG00000006501	cyp2x10.2	-3.180865035	0.00000431	-3.548835429	2.63E-08
ENSDARG00000006508	pip5k1bb	0.41632325	0.005834145	0.398959608	0.006823068
ENSDARG00000006602	chrna2a	-0.745611796	0.013158476	-0.695251072	0.018034843

Table S3. DEGs of WT vs. *terfa+/-* ∩ *terfa-/-*

ENSDARG00000006604	nectin3b	0.523984117	0.000158214	0.421248826	0.002141811
ENSDARG00000006672	fam49a	-0.577590893	0.00000153	-0.299395691	0.016147424
ENSDARG00000006766	snd1	0.377352795	0.000100394	0.291573347	0.002411141
ENSDARG00000006791	arntl1a	-0.708035882	0.00000296	-0.465501601	0.002011693
ENSDARG00000006848	parp9	-0.948338658	0.000575449	-0.960999799	0.000196586
ENSDARG00000007024	uox	-0.71917196	0.014442497	-0.611361144	0.039551289
ENSDARG00000007025	ttc9c	0.932890338	0.0000231	0.855726812	0.0000477
ENSDARG00000007077	ankrd50l	1.431549523	2.47E-26	0.354213522	0.019752488
ENSDARG00000007080	rhcgl1	4.101602054	8.68E-32	4.09702604	1.33E-37
ENSDARG00000007169	itgb1bp1	0.659917055	3.64E-08	0.320067468	0.012168
ENSDARG00000007175	recql	0.391634776	0.013880637	0.328721111	0.042420879
ENSDARG00000007180	slc30a4	0.644773232	0.000933162	0.538222366	0.00524164
ENSDARG00000007207	cnpy2	0.793089075	0.00000116	0.938748499	1.63E-10
ENSDARG00000007219	actn1	1.253315704	8.52E-40	0.735554419	1.21E-16
ENSDARG00000007275	si:ch211-251b21.1	-0.306469482	0.023620923	-0.295426188	0.02756023
ENSDARG00000007276	ela3l	-3.673804671	0.001420194	-3.451471997	0.001896106
ENSDARG00000007302	sh3gl3b	-0.519648354	0.006528205	-0.388144267	0.047194522
ENSDARG00000007344	tcap	2.72678376	1.73E-31	1.195748883	0.000000229
ENSDARG00000007362	sft2d1	0.856135093	7.54E-09	0.479111935	0.001528087
ENSDARG00000007377	odc1	0.581854494	0.0000036	0.526654341	0.00000851
ENSDARG00000007461	srgap1a	0.755965218	0.00000168	0.42798855	0.009226816
ENSDARG00000007553	opn4.1	-2.0686911	1.16E-20	-0.632505342	0.00559798
ENSDARG00000007576	crybb1l1	-3.545227126	2.93E-24	-3.227072255	1.19E-24
ENSDARG00000007597	bloc1s4	-0.572121964	0.002891184	-0.400665725	0.041889829
ENSDARG00000007639	cnot4b	0.399041944	0.000873788	0.431755926	0.000113372
ENSDARG00000007655	crybb1l3	-2.531457979	5.58E-09	-1.392506609	0.000383646
ENSDARG00000007693	nfkbiab	0.473734103	0.04230806	0.50359519	0.027303842
ENSDARG00000007711	rab3il1	0.570705913	0.000905288	0.447684605	0.009559377
ENSDARG00000007715	lgsn	-1.609566151	1.53E-11	-2.047096723	5.62E-22
ENSDARG00000007720	sub1b	-0.599326858	0.0000386	-0.382208738	0.009341165
ENSDARG00000007739	atp1a1a.2	-0.697492158	0.0000408	-0.546039898	0.001024054
ENSDARG00000007786	tmx2b	-0.432770923	0.0000484	-0.345618712	0.00084728
ENSDARG00000007836	ctsla	1.176603708	2.8E-13	0.427966464	0.013768052
ENSDARG00000007867	dazap2	-0.457439649	0.0000431	-0.357887298	0.001059507
ENSDARG00000007869	ehd3	-0.587294924	0.003353868	-0.459261306	0.023070105
ENSDARG00000007886	slc35b2	0.564820329	0.00000426	0.368676428	0.003122729
ENSDARG00000007955	iars1	0.690146881	0.00000059	0.538104105	0.0000515
ENSDARG00000007971	cks1b	-0.861582358	0.0000231	-0.614245628	0.002089084
ENSDARG00000007976	si:ch211-220f16.2	-0.511425074	0.0000124	-0.307554164	0.009900866
ENSDARG00000007988	masp2	1.154515702	6.54E-08	0.768402582	0.00028812
ENSDARG00000008026	niban2b	0.838947702	4.58E-13	0.547509552	0.00000109
ENSDARG00000008049	si:dkey-42i9.4	-0.723916318	0.00000805	-0.40813071	0.014918918
ENSDARG00000008165	caspa	1.164822953	2.02E-12	0.469051102	0.009636279
ENSDARG00000008184	mia3	0.271571634	0.012190291	0.227044254	0.038883274
ENSDARG00000008191	tmeff1a	-0.59432604	0.020754891	-0.745336339	0.001872156
ENSDARG00000008263	mfsd4ab	-0.695286668	0.004018856	-1.012579156	0.00000337
ENSDARG00000008287	fam114a1	0.569117682	0.0000117	0.322457495	0.018465534
ENSDARG00000008447	fkbp4	0.302909592	0.002226856	0.341441427	0.000212612
ENSDARG00000008494	myl6	0.45416097	0.000246364	0.430410853	0.000268292
ENSDARG00000008502	pno1	-0.395917286	0.002197529	-0.439419392	0.000272916
ENSDARG00000008573	fam20b	0.433892893	0.001599588	0.332411162	0.017070887
ENSDARG00000008593	nbas	0.407912812	0.013083755	0.395548795	0.014169344
ENSDARG00000008678	snx3	0.393592178	0.006837616	0.316600077	0.032535927
ENSDARG00000008740	esf1	0.757276679	0.00000038	0.532164189	0.000294823
ENSDARG00000008818	hsf1	-0.452691454	0.002078988	-0.403028559	0.004986007

Table S3. DEGs of WT vs. *terfa+/-* ∩ *terfa-/-*

ENSDARG00000008832	neu1	0.477725367	0.029797498	0.487742387	0.023826773
ENSDARG00000008858	cyp7b1	1.057479703	1.24E-08	0.681820973	0.000236305
ENSDARG00000008866	cabp4	-2.19161959	0.000210208	-1.272092062	0.019657647
ENSDARG00000008867	rap1b	0.617516355	1.05E-08	0.235394104	0.049790839
ENSDARG00000008904	smarca2	0.511890959	0.0000876	0.489621932	0.0000724
ENSDARG00000008920	mrpl53	0.604998269	0.000111294	0.544113703	0.00030648
ENSDARG00000008969	fgb	1.283989307	2.4E-35	0.382425975	0.000385177
ENSDARG00000009001	pdia6	0.790039097	1.36E-08	0.648832023	0.000000766
ENSDARG00000009215	zgc:112437	-1.366460361	6.67E-09	-0.911021309	0.0000423
ENSDARG00000009336	aif1l	-0.929941295	4E-13	-0.302284276	0.029984471
ENSDARG00000009390	npl	0.748366626	0.000135249	0.783912595	0.0000176
ENSDARG00000009401	vcanb	1.12310929	2.21E-21	0.598846613	0.000000166
ENSDARG00000009418	mef2cb	-0.551205435	0.00000104	-0.277118084	0.019657662
ENSDARG00000009470	hnf1a	-0.656734363	0.018713266	-0.762757423	0.003887973
ENSDARG00000009505	prelid3b	0.728060054	6.81E-09	0.58467932	0.000000839
ENSDARG00000009534	wls	0.26802844	0.009180907	0.260929377	0.009334744
ENSDARG00000009629	unc119.1	-0.616003952	0.000199556	-0.498725924	0.002068867
ENSDARG00000009753	sf3b6	-0.888069998	0.000000144	-0.752363461	0.00000196
ENSDARG00000009839	fam117bb	0.570942024	0.000366045	0.387973881	0.019410876
ENSDARG00000009978	icn	1.449290763	3.82E-25	1.077117247	6.5E-17
ENSDARG00000010031	ssh1b	-0.881223449	9.51E-09	-0.349034248	0.035104525
ENSDARG00000010070	adam9	0.448020688	0.000178925	0.293854444	0.0174979
ENSDARG00000010085	p4ha2	1.162284223	1.77E-16	1.003538749	1.13E-14
ENSDARG00000010146	cpa2	2.657189681	1.07E-12	0.985148111	0.023344492
ENSDARG00000010160	rps15a	0.466310908	0.0000815	0.401217454	0.000439789
ENSDARG00000010183	si:ch73-265h17.1	1.59676372	0.000109726	0.957003966	0.031619669
ENSDARG00000010244	rpl22l1	0.462654495	0.000167469	0.520391361	0.00000423
ENSDARG00000010246	prmt1	-0.434980785	0.0000358	-0.311610082	0.002992843
ENSDARG00000010279	scamp2	-0.397622118	0.000538396	-0.514560607	0.000000702
ENSDARG00000010318	srpx	0.361548118	0.000792416	0.424395344	0.0000176
ENSDARG00000010385	sept4a	-2.186502497	2.84E-21	-0.537893258	0.026844343
ENSDARG00000010411	epn1	-0.374946086	0.0000441	-0.338988759	0.0000984
ENSDARG00000010415	sirt4	2.286617818	9.49E-27	1.798090834	5.73E-19
ENSDARG00000010434	clu	3.002780604	1.08E-46	0.687455094	0.002411141
ENSDARG00000010442	rnf11a	-0.341054786	0.014302162	-0.286727846	0.04125066
ENSDARG00000010478	hsp90aa1.1	1.756491963	1.69E-40	1.20736446	2.22E-23
ENSDARG00000010482	prg4a	0.890525278	0.032545439	1.24875149	0.000966641
ENSDARG00000010519	per3	0.471411198	0.004344717	0.538049007	0.000493643
ENSDARG00000010556	mmp25b	-1.181828219	0.00000419	-1.148140428	0.00000106
ENSDARG00000010572	slc25a25a	1.116208703	1.48E-09	0.624387316	0.000823397
ENSDARG00000010601	mtmr10	-0.379011474	0.007075489	-0.344201277	0.012853374
ENSDARG00000010641	slc20a1b	0.386032096	0.000130265	0.264869196	0.009840763
ENSDARG00000010655	ppm1k	-0.290479617	0.032165883	-0.350181348	0.006290549
ENSDARG00000010658	insig1	0.486348039	0.01072901	0.937716951	7.45E-09
ENSDARG00000010680	gngt2a	-4.629277104	1.61E-10	-1.75026119	0.02470655
ENSDARG00000010710	msi1	-1.186400228	1.44E-19	-0.469709917	0.000384639
ENSDARG00000010727	ttyh2l	-1.086383146	6.39E-13	-0.402206896	0.010964061
ENSDARG00000010738	zgc:101716	1.388513044	0.014095427	1.324073918	0.018930778
ENSDARG00000010785	thbs1b	0.371755593	0.001991104	0.283531245	0.02018244
ENSDARG00000010823	memo1	0.524145298	0.00004	0.402478308	0.00140896
ENSDARG00000010831	churc1	0.347343453	0.046132423	0.427859765	0.009284753
ENSDARG00000010933	cacna1fa	-3.015074184	4.29E-24	-0.677753153	0.026492413
ENSDARG00000010946	cbsb	0.992365149	4.1E-18	0.638411618	4.58E-09
ENSDARG00000010962	fkbp7	1.010517877	3.04E-12	0.620824674	0.0000127
ENSDARG00000011049	slc17a9b	0.927528827	6.65E-11	0.572598539	0.0000539

Table S3. DEGs of WT vs. *terfa+/-* ∩ *terfa-/-*

ENSDARG00000011170	ndrg2	-0.501627663	0.0000144	-0.343666868	0.003013444
ENSDARG00000011245	esrp1	0.428760834	0.001118109	0.340045461	0.009807679
ENSDARG00000011326	ankrd45	2.645115067	0.00000135	1.646876002	0.005989307
ENSDARG00000011400	tnnc1a	0.91678896	0.00000791	0.446493902	0.049288161
ENSDARG00000011407	col2a1b	0.427923514	0.0000848	0.254140038	0.026030437
ENSDARG00000011488	sirt2	0.586205506	0.000015	0.299315241	0.040320334
ENSDARG00000011555	spag7	-0.333656276	0.001853573	-0.229472482	0.038452253
ENSDARG00000011570	pou6f1	-0.916972081	3.66E-08	-0.374026648	0.037299079
ENSDARG00000011583	cry1b	0.559045533	0.000622874	0.701667825	0.00000208
ENSDARG00000011613	rbm39a	-0.499676794	1.92E-08	-0.201005988	0.037865453
ENSDARG00000011640	syt5b	-2.336080483	3.31E-34	-0.539559897	0.009341165
ENSDARG00000011652	ftr52p	1.507659392	0.00000121	0.716927969	0.04138024
ENSDARG00000011661	twf1b	-0.331720964	0.006065941	-0.282080849	0.019236681
ENSDARG00000011671	pde6b	-2.233322746	9.9E-18	-0.727353594	0.006428275
ENSDARG00000011672	sema3b	0.458430935	0.000000638	0.241877054	0.012410448
ENSDARG00000011693	ttc36	-0.632660738	0.007487745	-0.744936747	0.000735737
ENSDARG00000011743	pigo	-0.358633372	0.005983816	-0.388799877	0.001644253
ENSDARG00000011777	cttn	0.52212979	0.00000685	0.387139004	0.000679846
ENSDARG00000011821	plod2	0.609310512	0.0000108	0.32504777	0.027331324
ENSDARG00000011824	pbxip1b	-0.498295797	0.000430969	-0.411756052	0.003018965
ENSDARG00000011841	atp5l	0.832038827	2.31E-11	0.58594904	0.000000826
ENSDARG00000011862	inaa	-1.417282305	0.0000803	-0.861122196	0.01959323
ENSDARG00000011886	pdca	-2.83036981	2.76E-18	-0.735500835	0.017979673
ENSDARG00000011929	plp1b	-1.511821631	0.00000011	-1.008043684	0.000240404
ENSDARG00000011998	bfsp2	-1.443180009	3.79E-11	-1.743215577	3.27E-19
ENSDARG00000012002	slc38a7	0.596728871	0.000201392	0.553284013	0.000318058
ENSDARG00000012019	glra1	-0.388893397	0.010276461	-0.316685308	0.039641466
ENSDARG00000012125	cnga1b	-3.246505539	5.5E-23	-0.74299216	0.009900866
ENSDARG00000012126	zgc:109965	-2.559965889	1.38E-20	-0.613715928	0.04159915
ENSDARG00000012144	emc7	-0.543434872	0.0000204	-0.39381394	0.001763953
ENSDARG00000012192	cant1a	0.578060548	0.00000843	0.346456293	0.009900866
ENSDARG00000012199	gpt2	-0.638629296	0.000000266	-0.322013413	0.01217429
ENSDARG00000012325	zgc:110269	-0.71296148	0.0000732	-0.470219704	0.009034019
ENSDARG00000012341	capn9	0.64894493	9.98E-08	0.474122942	0.0000603
ENSDARG00000012395	mmp13a	3.119399745	8.52E-17	1.297311553	0.00127247
ENSDARG00000012405	col1a1a	0.764608092	0.000000223	0.554988867	0.000106971
ENSDARG00000012426	neflb	0.548154025	0.0000352	0.314104507	0.024580818
ENSDARG00000012458		0.331969201	0.000573896	0.221829174	0.026341191
ENSDARG00000012499	per1b	1.180700665	2.15E-20	1.355934086	7.74E-33
ENSDARG00000012513	sdcbp2	0.654400782	2.1E-10	0.266038421	0.01637352
ENSDARG00000012588	ptdss1a	0.680787737	4.75E-08	0.446778218	0.000297677
ENSDARG00000012591	pdcd10b	-0.321535538	0.010315183	-0.285993418	0.021259745
ENSDARG00000012632	zgc:92749	1.086665793	0.000623803	1.115982773	0.000205728
ENSDARG00000012694	c3a.1	1.698490113	2.73E-41	0.406880551	0.002726568
ENSDARG00000012848	arih2	-0.396778889	0.0000188	-0.289412064	0.001534184
ENSDARG00000012866	picalma	0.324392569	0.000560622	0.279111167	0.002411141
ENSDARG00000012874	snap23.1	0.585244668	0.0000041	0.299942378	0.027261433
ENSDARG00000013110	dmtn	-0.57251787	0.0000401	-0.35154369	0.012970722
ENSDARG00000013221	pde4ca	0.671524439	0.000341197	0.645818348	0.00030648
ENSDARG00000013251	hsf4	-2.493475024	4.81E-08	-2.116951955	0.000000281
ENSDARG00000013295	slc2a3a	0.325934876	0.018645807	0.355370536	0.007506536
ENSDARG00000013351	cirbpb	-1.100967136	5.67E-17	-0.70880294	1.35E-08
ENSDARG00000013371	isoc2	0.981642411	1.21E-12	0.626594762	0.00000345
ENSDARG00000013441	hey2	-0.9378319	0.0000147	-0.473405075	0.033000711
ENSDARG00000013475	cct4	0.479693895	0.000998613	0.407955361	0.004475945

Table S3. DEGs of WT vs. *terfa+/-* ∩ *terfa-/-*

ENSDARG00000013522	pck1	1.527654922	2.86E-12	1.064679011	0.000000321
ENSDARG00000013528	mcm9	-0.897376109	0.0000144	-0.567883666	0.005657893
ENSDARG00000013542	lpgat1	0.243352772	0.039232156	0.409376762	0.0000783
ENSDARG00000013575	rfx2	0.527752121	0.0000154	0.379051879	0.001863454
ENSDARG00000013576	gadd45bb	0.913594172	0.000301357	0.667170151	0.009475411
ENSDARG00000013659	brox	-0.399394265	0.003960147	-0.378484428	0.004838809
ENSDARG00000013667	tbck	0.395795581	0.016684421	0.448163879	0.004341155
ENSDARG00000013670	hyou1	0.809495055	1.6E-15	0.606347251	1.73E-10
ENSDARG00000013729	srsf6a	0.404932969	0.007770179	0.47285238	0.000897208
ENSDARG00000013741	lancl1	-1.416184974	2.4E-28	-1.271913334	4.89E-29
ENSDARG00000013763	rrbp1a	0.486554779	2.29E-09	0.230115374	0.007173347
ENSDARG00000013777	ppp1r13l	0.790161616	1.56E-12	0.398712492	0.000437752
ENSDARG00000013784	zgc:77158	0.804051988	0.0000412	0.548954111	0.006141011
ENSDARG00000013804	capns1b	0.644244835	0.000000574	0.309813108	0.024585645
ENSDARG00000013822	anapc16	0.615333408	0.001068144	0.493529172	0.008703245
ENSDARG00000013845	ttc14	-0.833573024	0.000000523	-0.762472695	0.000000821
ENSDARG00000013892	sorl1	0.537898569	0.000000204	0.426448718	0.0000163
ENSDARG00000013928	plekhb2	0.675467675	0.00000169	0.593270861	0.00000931
ENSDARG00000013963	mipb	-3.5706052	5.21E-48	-2.859029446	3.76E-39
ENSDARG00000013990	ube2q2	-0.35462271	0.001543745	-0.25511475	0.025875958
ENSDARG00000014024	ms4a17a.4	3.906656729	5.03E-11	1.71022611	0.011751863
ENSDARG00000014050	ngfb	0.450233467	0.024908019	0.52748006	0.005473959
ENSDARG00000014091	osr1	-0.343479783	0.031039013	-0.32329747	0.041490116
ENSDARG00000014103	dkk1a	0.853443685	0.000179154	0.516557648	0.034933034
ENSDARG00000014190	sst2	3.523898471	0.00152256	2.746684719	0.023842831
ENSDARG00000014233	sept8b	-2.830299508	6.4E-12	-1.1180061	0.009465654
ENSDARG00000014246	jag2a	1.323669358	1.02E-11	0.530452096	0.012045316
ENSDARG00000014340	rab11al	0.927565992	7.85E-09	0.704288287	0.00000503
ENSDARG00000014358	optc	1.120821744	0.003453334	1.105374866	0.003056518
ENSDARG00000014378	slc12a4	0.773956153	0.000000128	0.329475292	0.040661392
ENSDARG00000014386	galnt6	0.934234827	6.3E-18	0.738928481	2.24E-13
ENSDARG00000014465	arhgef25b	1.330309268	3.02E-12	0.577332695	0.004317242
ENSDARG00000014479	ptf1a	-1.823908353	0.000000351	-0.778467534	0.026988347
ENSDARG00000014496	trpv6	-0.703516373	0.002029496	-0.889614245	0.0000182
ENSDARG00000014532	aida	-0.450037925	0.001172679	-0.611564647	0.000000943
ENSDARG00000014545	golim4b	0.471697617	0.040211509	0.495457309	0.027930695
ENSDARG00000014624	si:dkeyp-94g1.1	5.621222991	1.6E-28	5.661540716	8.09E-32
ENSDARG00000014790	g3bp2	-0.82279902	1.48E-09	-0.409804299	0.003292879
ENSDARG00000014803	cryba1l2	-1.303013499	2.7E-09	-1.416729723	6.51E-13
ENSDARG00000014941	tprkb	1.7455982	0.00039195	1.155066012	0.031546708
ENSDARG00000014969	ankhb	-0.66861641	2.26E-08	-0.422599498	0.000292052
ENSDARG00000015076	gja8b	-1.461531948	8.47E-10	-1.559665758	3.1E-13
ENSDARG00000015126	gorasp2	0.295960209	0.01680507	0.326305158	0.005841034
ENSDARG00000015128	rpl27	0.70185428	8.17E-09	0.632822555	2.06E-08
ENSDARG00000015199	cblb	0.313752887	0.023700974	0.428026673	0.000721792
ENSDARG00000015201	pcmt	0.611807382	3.11E-09	0.45837039	0.00000361
ENSDARG00000015224	cd2ap	0.640391399	4.59E-08	0.307734832	0.013131213
ENSDARG00000015273	alpi.1	1.126696889	2.38E-20	0.551943423	0.00000634
ENSDARG00000015293	fam110a	0.651321584	0.001468413	0.526867695	0.010453116
ENSDARG00000015445	lim2.4	-0.549860996	0.030448283	-0.869653207	0.000125235
ENSDARG00000015495	klf3	0.489834348	0.011426541	0.422003391	0.030428323
ENSDARG00000015524	prps1a	0.776597205	7.28E-10	0.356431216	0.007155934
ENSDARG00000015538	znf207a	0.35183838	0.007789739	0.466511481	0.000106523
ENSDARG00000015540	psen2	0.732312565	8.35E-08	0.517413373	0.000106889
ENSDARG00000015607	si:ch211-220f21.2	7.482228096	9.02E-08	7.829934866	3.35E-08

Table S3. DEGs of WT vs. *terfa+/-* ∩ *terfa-/-*

ENSDARG00000015657	zgc:77112	-1.572367347	0.00000438	-0.753725069	0.023824284
ENSDARG00000015709	hsd17b12a	1.274563826	4.83E-29	1.080762878	2.29E-25
ENSDARG00000015732	cax2	0.720911487	0.0000165	0.517632569	0.002105378
ENSDARG00000015793	creb3l1	0.372184746	0.012593415	0.453205914	0.001085887
ENSDARG00000015829	mov10a	0.750013319	5.46E-10	0.320853375	0.013858961
ENSDARG00000015854	chata	-0.378014152	0.018191956	-0.41948786	0.006030664
ENSDARG00000015889	zc3h15	0.350129344	0.001940698	0.295640421	0.008310612
ENSDARG00000016038	hacd3	-0.540587519	0.000094	-0.326388806	0.022437729
ENSDARG00000016141	slc6a2	-0.696788558	0.00128137	-0.768952724	0.000131619
ENSDARG00000016143	xiap	0.819740727	1.78E-09	0.566469904	0.0000193
ENSDARG00000016177	eif4enif1	-0.554637622	0.00013547	-0.388268078	0.007795256
ENSDARG00000016188	SBNO2	2.042894604	1.65E-26	0.596468684	0.003945941
ENSDARG00000016260	fxr2	0.494326609	0.000211551	0.367165574	0.006125787
ENSDARG00000016319	c9	1.562717944	2.57E-33	0.662714927	0.000000199
ENSDARG00000016337	lctlb	-3.299933118	7.82E-57	-2.429005712	2.64E-48
ENSDARG00000016350	cap1	1.277563605	3.6E-26	0.79242067	6.2E-12
ENSDARG00000016360	kpna1	0.238955942	0.034150019	0.275672123	0.010451541
ENSDARG00000016404	ptch1	0.354107729	0.008983062	0.312534847	0.020990161
ENSDARG00000016447	ythdf1	0.318978726	0.000592052	0.31273796	0.000389657
ENSDARG00000016457	irf9	1.483141449	7.07E-13	0.614532444	0.005687253
ENSDARG00000016532	suco	0.388760462	0.000879618	0.319070025	0.005907883
ENSDARG00000016536	npas2	-0.759029111	0.000496084	-0.620864968	0.00359957
ENSDARG00000016538	zgc:55888	3.888044959	0.000000653	4.186031854	1.92E-08
ENSDARG00000016573	mroh1	0.29635959	0.007916881	0.368323326	0.000346057
ENSDARG00000016651	znf106a	0.847477513	6.38E-12	0.276319172	0.045509314
ENSDARG00000016706	atic	1.131230935	3.18E-08	0.678825776	0.000932261
ENSDARG00000016763	znf292a	-0.614182723	0.0000292	-0.324855095	0.037384374
ENSDARG00000016793	crybb1l2	-1.464822273	1.87E-10	-1.60890749	7.89E-15
ENSDARG00000016818	abcg2d	1.756455055	5.41E-08	1.038133829	0.002125398
ENSDARG00000016835	tcirg1a	-0.440316001	0.00590711	-0.337092256	0.038270898
ENSDARG00000016856	pon2	0.482673479	0.010494212	0.429005821	0.022940638
ENSDARG00000016864	farsb	0.575534623	0.000086	0.361910043	0.017360608
ENSDARG00000016999	lin28a	1.063834546	0.00000931	0.764638888	0.001503695
ENSDARG00000017128	myofl	1.116309108	1.61E-10	0.508553823	0.005683662
ENSDARG00000017165	slc3a1	-0.560963312	0.031579519	-0.839147323	0.000387779
ENSDARG00000017251	smtlb	-2.536064588	9.07E-08	-1.301743626	0.003703193
ENSDARG00000017354	epha2a	0.820411238	1.85E-13	0.372163499	0.001387235
ENSDARG00000017359	mettl8	-0.937844826	0.007352954	-0.81097286	0.017557451
ENSDARG00000017369	sema3d	-0.280895187	0.025621281	-0.253536327	0.044956724
ENSDARG00000017454	nup50	0.500271073	0.000327561	0.460378912	0.000571363
ENSDARG00000017474	zgc:110699	-1.123016152	0.001294537	-0.745341782	0.033508052
ENSDARG00000017490	cel.1	-1.676714538	0.017535248	-1.496959983	0.034565097
ENSDARG00000017590	nit2	-0.33813763	0.019308421	-0.473327219	0.0003192
ENSDARG00000017602	ccng2	-0.585728528	0.00000233	-0.301570549	0.020156994
ENSDARG00000017624	krt4	1.197008309	1.06E-33	0.663642349	1.04E-12
ENSDARG00000017658	gmppb	0.611372276	0.000000116	0.533252465	0.000000856
ENSDARG00000017708	lrrc40	-0.533393075	0.00026681	-0.388779765	0.007343755
ENSDARG00000017757	pik3cg	-0.530307275	0.002595756	-0.549572907	0.000943024
ENSDARG00000017780	rorcb	-1.46917058	5.84E-28	-1.378935501	1.11E-30
ENSDARG00000017843	srsf1b	0.387433003	0.000220935	0.508265142	6.79E-08
ENSDARG00000017853	prepl	0.547261185	0.030872786	0.562726785	0.023742496
ENSDARG00000017931	ints6l	-0.755620815	0.00000691	-0.439492149	0.010024536
ENSDARG00000018006	vrk1	-1.140825774	5.09E-14	-0.970236	1.34E-12
ENSDARG00000018010	crtap	0.568803691	0.000165909	0.455579476	0.002232672
ENSDARG00000018073	mrps22	-0.371046362	0.003182373	-0.325373977	0.00832606

Table S3. DEGs of WT vs. *terfa+/-* ∩ *terfa-/-*

ENSDARG00000018145	mid1ip1l	-0.445604111	0.001439921	-0.315276915	0.026936236
ENSDARG00000018303	etv4	0.599897818	0.000016	0.437363587	0.001555252
ENSDARG00000018319	arglu1b	-0.640966585	4.87E-09	-0.447196807	0.0000205
ENSDARG00000018329	gc2	-1.228247108	0.00000749	-0.648579461	0.016247038
ENSDARG00000018404	krt18a.1	0.994205538	3.76E-18	0.357106954	0.003149357
ENSDARG00000018485	cyp2v1	0.573818198	0.000631402	0.440117895	0.009226816
ENSDARG00000018491	pdia4	1.312680789	3.39E-34	0.962648762	1.93E-22
ENSDARG00000018593	parp16	0.347891729	0.008284358	0.301129024	0.022825918
ENSDARG00000018621	slc6a19a.1	1.87292398	9.55E-11	0.691970265	0.0322776
ENSDARG00000018637	sec61g	2.332814705	1E-13	1.270234598	0.0000518
ENSDARG00000018693	cdh2	0.310527664	0.018261831	0.313296499	0.01470388
ENSDARG00000018797	crygmxl1	-1.970841584	0.00000152	-1.233440638	0.001522272
ENSDARG00000018809	abhd3	0.561266552	0.00488536	0.50409548	0.0104732
ENSDARG00000018820	flncb	1.111483182	4.5E-18	0.682757831	2.51E-08
ENSDARG00000018891	rnaseh2a	-0.783809454	0.000000291	-0.366703059	0.022060361
ENSDARG00000018903	aimp2	0.670042238	0.00000115	0.361953469	0.011890242
ENSDARG00000018984	eya2	0.477419549	0.00032833	0.329348809	0.015644377
ENSDARG00000018997	cplx2l	-0.372089525	0.002517788	-0.278027922	0.026844343
ENSDARG00000019063	fat1b	-1.552166516	4.25E-17	-0.627716115	0.000759202
ENSDARG00000019081	DHDH	0.979093138	0.000000155	0.623003302	0.000844876
ENSDARG00000019135	ankef1a	0.917054641	0.004875394	0.784371261	0.017530901
ENSDARG00000019137	tram1	0.561023603	0.000576465	0.397979665	0.017000102
ENSDARG00000019236	gsr	1.002266517	2.11E-12	0.427335305	0.004672933
ENSDARG00000019360	sec23b	0.319235701	0.013114689	0.452725233	0.0000965
ENSDARG00000019362	ptbp1a	-0.345569041	0.001582111	-0.344855936	0.000924966
ENSDARG00000019367	tgfb3	0.322482877	0.00609668	0.296712487	0.010280108
ENSDARG00000019498	cry5	0.911564499	4.44E-09	1.076039172	1E-14
ENSDARG00000019601	col12a1b	1.295491978	1.85E-13	0.733903001	0.0000215
ENSDARG00000019636	yif1a	0.53548398	0.00016102	0.429446558	0.002198121
ENSDARG00000019713	oatx	1.190011911	3.24E-08	0.510320242	0.030097821
ENSDARG00000019728	bmpr1aa	-0.267365412	0.022230078	-0.345005268	0.001442414
ENSDARG00000019742	tlr4ba	-1.659259983	0.044101323	-1.596296249	0.046852602
ENSDARG00000019752	rom1a	-2.960849899	2.83E-27	-0.942113155	0.000640606
ENSDARG00000019782	impg2a	-3.921245809	1.36E-26	-1.011079776	0.001463455
ENSDARG00000019808	evpla	0.503854283	0.00000296	0.32180056	0.003215975
ENSDARG00000019815	fn1a	0.90539079	8.31E-14	0.421192698	0.000659027
ENSDARG00000019924	cmpk	0.249477503	0.034248655	0.235257282	0.046376485
ENSDARG00000019949	serpinh1b	2.81581519	7.19E-75	2.532681664	2.01E-74
ENSDARG00000019950	carf	0.975597986	3.14E-15	1.161521455	4.69E-26
ENSDARG00000019962	dop1a	0.293973376	0.046203357	0.335015313	0.018359676
ENSDARG00000019976	idi1	-0.467399395	0.000117364	-0.271772313	0.03267311
ENSDARG00000020007	col1a2	0.441799529	0.000211112	0.432153639	0.000125569
ENSDARG00000020028	cps1	-2.276897261	1.01E-11	-2.198466261	2.33E-13
ENSDARG00000020041	vps35l	0.362072405	0.001946135	0.242364923	0.048016755
ENSDARG00000020086	nuak1a	1.728834735	1.51E-15	0.630004048	0.006881255
ENSDARG00000020114	slc20a1a	1.355985261	2.42E-12	0.691948863	0.000414063
ENSDARG00000020133	jdp2b	-0.941410084	0.000343698	-1.344967595	1.11E-08
ENSDARG00000020252	btbd10b	0.355531565	0.001711684	0.336779009	0.002097174
ENSDARG00000020279	efcab7	-0.580252012	0.000365084	-0.378926721	0.022350461
ENSDARG00000020298	btg2	-0.627271196	0.004833812	-0.692523876	0.000958277
ENSDARG00000020326	tyk2	0.800464489	0.00000443	0.394925672	0.037501667
ENSDARG00000020465	ewsr1b	-0.745286394	0.000000984	-0.469386214	0.002199212
ENSDARG00000020469	map3k7	-0.293896199	0.00973275	-0.273467727	0.014465886
ENSDARG00000020473	hddc3	-0.924617047	0.000000251	-0.612545364	0.000437481
ENSDARG00000020527	nup62l	-0.588448605	0.0000022	-0.346495607	0.006160999

Table S3. DEGs of WT vs. *terfa+/-* ∩ *terfa-/-*

ENSDARG00000020573	ddx3xa	-0.442371853	0.0000145	-0.516450945	2.32E-08
ENSDARG00000020618	gatd3a	0.761624002	0.00000161	0.511960768	0.001301666
ENSDARG00000020625	jak1	1.151423058	8.91E-17	0.326814355	0.035778595
ENSDARG00000020699	slc9a8	0.601494472	0.0000454	0.327896133	0.039061406
ENSDARG00000020711	rrm2	6.254113226	3.26E-22	6.919188641	6.17E-32
ENSDARG00000020741	fga	1.464005097	1.54E-70	0.355288525	0.0000273
ENSDARG00000020759	elf1	0.518828595	0.00000723	0.288850738	0.017435773
ENSDARG00000020814	faimb	-3.175241382	1.35E-23	-0.767709763	0.009134715
ENSDARG00000020840	mcrs1	-0.577050719	0.0000154	-0.283041958	0.0479796
ENSDARG00000020850	eef1a1l1	0.437173291	0.00000903	0.376715661	0.0000593
ENSDARG00000020863	trim25l	0.776826666	0.0000178	0.42763318	0.028580647
ENSDARG00000020866	apoa4b.2	-0.5150069	0.010126628	-1.030504232	1.65E-09
ENSDARG00000020893	slc25a55a	-0.276639286	0.021202332	-0.765678273	7.6E-15
ENSDARG00000020924	myo1ca	0.592808068	0.0000234	0.447245596	0.001264784
ENSDARG00000020926	creb3l3l	0.250483012	0.018634651	0.261879854	0.01101225
ENSDARG00000020952	si:ch211-214j8.1	1.503425723	0.00000491	0.761882533	0.031546708
ENSDARG00000020957	pth1r	-0.878303101	0.013963039	-0.90954764	0.008058127
ENSDARG00000020984	slc16a10	0.608189526	0.00000122	0.348168414	0.007150691
ENSDARG00000021059	alas1	0.711571049	0.000000832	0.433786526	0.003103658
ENSDARG00000021082	sec31a	0.916456837	3.17E-16	0.639330206	1.58E-09
ENSDARG00000021140	pabpc1b	0.542437579	0.000000211	0.300667322	0.005277205
ENSDARG00000021209	kctd9a	-0.497355606	0.043890993	-0.67100582	0.003488702
ENSDARG00000021242	mvp	1.46842712	6.15E-30	0.439389973	0.001291588
ENSDARG00000021265	mybpc2b	0.703063187	0.00000014	0.452173693	0.000659467
ENSDARG00000021287	rab7b	1.370310571	1.66E-16	0.775096194	0.00000235
ENSDARG00000021339	cpa5	-1.988598141	0.004766908	-2.236206751	0.00071649
ENSDARG00000021345	prph2lb	-2.911619419	5.47E-20	-0.886818392	0.002299818
ENSDARG00000021346	pdhb	0.555308273	7.26E-08	0.395490516	0.0000777
ENSDARG00000021366	fbp1a	0.99857908	0.000000477	0.851741198	0.00000595
ENSDARG00000021378	phf21ab	-0.601376263	0.0000441	-0.327670232	0.034482716
ENSDARG00000021389	jag2b	0.282223407	0.041311806	0.337451536	0.010166662
ENSDARG00000021462	daw1	1.919423619	2.25E-15	0.72763342	0.006428275
ENSDARG00000021547	vrk2	1.840718862	2.27E-18	0.890720699	0.0000437
ENSDARG00000021787	abcb5	0.587949244	0.0000146	0.385477374	0.005058259
ENSDARG00000021838	rps23	0.762769222	1.18E-12	0.665578902	1.6E-11
ENSDARG00000021853	ntpcr	1.071772898	0.0000227	0.958743719	0.0000759
ENSDARG00000021889	gja3	-1.833560827	2.38E-12	-2.016553336	6.54E-18
ENSDARG00000021948	tnc	0.561182836	0.0000209	0.352425401	0.00914587
ENSDARG00000021987	plecb	0.757406194	0.000000566	0.405512368	0.010030054
ENSDARG00000022165	mgst1.2	0.686915663	0.00000906	0.471690435	0.00243833
ENSDARG00000022183	gsto1	1.467082645	0.000631402	1.434135892	0.000523755
ENSDARG00000022187	usp6nl	-0.401125514	0.002268656	-0.383081227	0.002434063
ENSDARG00000022218	uck1	-0.713071357	0.000101172	-0.69024745	0.000054
ENSDARG00000022303	higd1a	1.153018153	2.31E-14	0.849623486	2.55E-09
ENSDARG00000022309	dspa	0.640774748	0.000000174	0.272091046	0.042496285
ENSDARG00000022525	mchr1b	3.057854164	0.00696428	3.561619953	0.00080989
ENSDARG00000022531	ntn1b	0.838625093	0.000000913	0.545930147	0.001427425
ENSDARG00000022614	ddx43	-1.127166649	0.000683872	-0.840617303	0.011360275
ENSDARG00000022615	si:ch211-241e1.3	1.568099849	5.89E-15	0.716709347	0.00053732
ENSDARG00000022631	cyp2p9	-5.278181834	0.0000784	-6.044294831	0.00000125
ENSDARG00000022650	cyp2ad3	-0.772111146	0.0000229	-0.605961051	0.000624514
ENSDARG00000022689	itgb1b.2	-0.834255341	9.98E-08	-0.890731716	3.5E-10
ENSDARG00000022712	stat3	1.666260074	6.28E-24	0.562151379	0.001227096
ENSDARG00000022768	gid8a	-0.774567607	0.00000227	-0.367125849	0.032854194
ENSDARG00000022788	cops7a	-0.337324743	0.01177352	-0.324918207	0.013049165

Table S3. DEGs of WT vs. *terfa+/-* ∩ *terfa-/-*

ENSDARG00000022817	pvalb3	1.796303923	1.14E-14	1.099854308	0.00000161
ENSDARG00000022891	zgc:175214	-0.444486471	0.0000508	-0.238657279	0.040432285
ENSDARG00000023111	plg	1.043687968	1.22E-15	0.490392442	0.000196853
ENSDARG00000023151	ucp1	1.581084539	6.42E-19	0.923399928	7.12E-08
ENSDARG00000023217	crema	1.414717905	1.55E-12	0.468431566	0.03720392
ENSDARG00000023228	vsnl1a	0.345801059	0.029910306	0.519145135	0.000292138
ENSDARG00000023299	snu13b	-0.409214891	0.000770063	-0.270957034	0.030811993
ENSDARG00000023302	gpbar1	1.531018789	0.005626368	1.555023069	0.004126268
ENSDARG00000023472	ctnnb2	0.620851057	4.45E-09	0.287523628	0.009840763
ENSDARG00000023498	gmppab	0.52315883	0.001087319	0.49793161	0.00123332
ENSDARG00000023546	kcnn1b	-1.289439106	0.00000107	-0.717848287	0.006449923
ENSDARG00000023648	idh3g	0.565575608	2.04E-08	0.259291658	0.015944512
ENSDARG00000023814	dyrk1ab	-0.624160785	0.000000127	-0.271487174	0.032200385
ENSDARG00000024112	gmppaa	-0.360833557	0.024535072	-0.339345751	0.032689506
ENSDARG00000024160	hao1	1.014649454	6.15E-09	0.6079708	0.000491454
ENSDARG00000024278	adh8b	-0.537449576	0.000000152	-0.580372717	4.01E-10
ENSDARG00000024295	slc11a2	0.888440607	5.77E-11	0.674076382	0.000000161
ENSDARG00000024548	cryba4	-0.719260022	0.003128421	-1.123505344	0.000000163
ENSDARG00000024561	nolc1	0.397426388	0.00039854	0.383614245	0.000325564
ENSDARG00000024588	gpc5b	0.540075979	0.000515889	0.370941758	0.021295644
ENSDARG00000024651	snrpa1	-0.523253236	0.0000473	-0.294597936	0.028549227
ENSDARG00000024694	myo1b	-0.274824201	0.019050973	-0.250529474	0.032295815
ENSDARG00000024740	gng13a	-1.739441796	0.00000611	-0.752119471	0.049937624
ENSDARG00000024746	hsp90aa1.2	0.483127441	0.0000282	1.034636802	2.7E-26
ENSDARG00000024771	slc24a5	0.769195132	0.000185756	0.620721653	0.002439683
ENSDARG00000024964	patj	0.589697211	0.0000312	0.308433132	0.042977641
ENSDARG00000025013	gna14a	-1.124836693	0.037806791	-1.102799774	0.038156278
ENSDARG00000025032	nr3c1	-0.298662977	0.006098167	-0.282344184	0.007893921
ENSDARG00000025147	cd63	0.828233126	4.88E-08	0.358656571	0.029343173
ENSDARG00000025254	s100a10b	1.531815072	3.72E-31	0.612328141	0.00000326
ENSDARG00000025301	gfap	-1.417273232	2.14E-32	-0.652673027	0.000000014
ENSDARG00000025325	ccsapa	-0.996403991	5.01E-10	-0.355441973	0.036853414
ENSDARG00000025504	gucy2f	-1.995056049	1.16E-08	-1.033874176	0.002418473
ENSDARG00000025507	wdr24	0.424302372	0.023129046	0.5011759	0.004507788
ENSDARG00000025535	clint1a	0.507406495	4.82E-08	0.283755638	0.002889296
ENSDARG00000025581	rpl10	0.347115553	0.004412173	0.321308722	0.007096931
ENSDARG00000025641	gli2a	0.416474475	0.0000811	0.254795186	0.020941346
ENSDARG00000025850	rps21	0.55245498	0.00000304	0.600517329	2.75E-08
ENSDARG00000025858	zgc:56525	0.667072669	0.00000147	0.578405012	0.0000105
ENSDARG00000025903	lgals9l1	1.976664538	3.01E-15	0.727174628	0.008640864
ENSDARG00000025912	bscl2l	-0.409778337	0.049002605	-0.472393387	0.018190975
ENSDARG00000025983	l3mbtl3	1.480857369	8.59E-08	1.02668511	0.000164237
ENSDARG00000026052	ccdc3a	1.052279085	0.0000206	0.670249088	0.009644824
ENSDARG00000026090	adprm	1.271184338	2.42E-11	1.218954376	3.98E-12
ENSDARG00000026137	sfxn5b	0.712250863	1.49E-09	0.420182679	0.000388779
ENSDARG00000026149	slc46a1	0.625669412	0.001499199	0.61466068	0.001139398
ENSDARG00000026281	lyrm1	1.659811962	0.000000482	0.815153731	0.024250395
ENSDARG00000026376	aco1	1.083651519	1.27E-13	0.592963951	0.0000433
ENSDARG00000026453	zgc:66474	-0.509659773	0.0000353	-0.411373949	0.000523755
ENSDARG00000026489	khsrp	0.595162143	0.003975115	0.737463084	0.000100498
ENSDARG00000026499	ppm1e	0.438608904	0.021416847	0.426280644	0.023572436
ENSDARG00000026531	alcama	0.381104273	0.0000735	0.262451683	0.007089801
ENSDARG00000026611	socs3b	2.686561279	2.19E-39	0.863011361	0.0000377
ENSDARG00000026629	gmds	0.499592295	0.000978072	0.353375961	0.023371929
ENSDARG00000026704	ftr72	3.047458557	0.000304473	2.290285355	0.010393726

Table S3. DEGs of WT vs. *terfa+/-* ∩ *terfa-/-*

ENSDARG00000026726	anxa1a	2.093904115	5.48E-32	1.515212913	1.72E-20
ENSDARG00000026759	ldlrb	0.455605621	0.000372639	0.957230808	8.07E-19
ENSDARG00000026799	suv39h1a	-1.757460007	0.002375219	-1.262514033	0.021199651
ENSDARG00000026801	stau2	0.558507655	8.82E-08	0.385687995	0.000156633
ENSDARG00000026829	cotl1	0.890998408	3.22E-21	0.299234929	0.00271872
ENSDARG00000026842	acin1b	-0.294084629	0.000729312	-0.215131242	0.014675082
ENSDARG00000026926	rom1b	-2.679100611	7.96E-18	-0.882628217	0.005631574
ENSDARG00000026979	krt1-c5	-1.488789002	1.88E-16	-0.625788239	0.000472213
ENSDARG00000027016	gbf1	0.413790855	0.002626415	0.388557734	0.003658399
ENSDARG00000027316	tcp11l2	0.301405637	0.013401186	0.351590388	0.002197059
ENSDARG00000027491	uros	-0.580272383	0.000586498	-0.425647792	0.011558853
ENSDARG00000027495	elovl4b	-3.158677875	6.7E-21	-1.000231139	0.005039342
ENSDARG00000027529	hmox1a	1.580178086	2.29E-12	1.204068919	1.42E-08
ENSDARG00000027547	TPGS1	0.680485825	0.0000353	0.510667641	0.001816955
ENSDARG00000027584	ttpa	-0.842071664	0.001357643	-1.243023661	0.000000122
ENSDARG00000027600	pdlim5b	0.723610005	4.99E-12	0.355113748	0.000952063
ENSDARG00000027611	cavin2a	0.811856556	1.43E-15	0.602209624	2.62E-10
ENSDARG00000027612	gatad1	-0.848117994	2.81E-08	-0.369928424	0.019420775
ENSDARG00000027734	srsf5b	-0.633916675	2.03E-08	-0.3482178	0.002451468
ENSDARG00000027799	ucmaa	-1.742730798	0.0000307	-1.024809434	0.013343444
ENSDARG00000027930	naprt	0.654615449	9.21E-09	0.386856736	0.000751443
ENSDARG00000028017	tp53inp1	1.089322935	6.38E-17	0.656776908	0.000000212
ENSDARG00000028058	traf6	0.479536057	0.003942572	0.363333603	0.033346206
ENSDARG00000028071	bmp1a	1.011635984	5.12E-17	0.456516527	0.00018595
ENSDARG00000028173	slc4a2a	1.048515862	2.38E-17	0.401581522	0.002207521
ENSDARG00000028201	commd8	0.583782497	0.000689585	0.657464337	0.0000359
ENSDARG00000028336	dhdhl	0.810379753	1.76E-11	0.473522569	0.0000747
ENSDARG00000028396	fkbp5	2.064035041	2.19E-26	1.781628005	4.54E-24
ENSDARG00000028478	si:ch211-173n18.3	2.343421207	0.000000291	2.482659712	7.02E-09
ENSDARG00000028507	itgb4	1.75861341	9.28E-26	1.014762779	1.76E-10
ENSDARG00000028542	si:ch211-114c12.2	-0.389188213	0.0000404	-0.377241274	0.0000213
ENSDARG00000028618	krt18b	0.703725604	4.59E-08	0.36746655	0.005882011
ENSDARG00000028804	ankrd9	1.449799816	8.72E-09	1.172764472	0.000000891
ENSDARG00000028824	slc34a1a	1.178964347	0.000000022	0.599513604	0.007355541
ENSDARG00000028900	odf2a	-0.568698836	0.002113622	-0.406259176	0.030133367
ENSDARG00000029043	nup42	-0.505944135	0.018672182	-0.463021379	0.029911456
ENSDARG00000029071	creld2	0.795178246	0.00000414	0.925874823	3.01E-09
ENSDARG00000029100	chchd1	-0.428083212	0.001056029	-0.300564964	0.023842831
ENSDARG00000029146	lrp1ab	1.415642741	2.12E-19	0.818052248	5.99E-08
ENSDARG00000029170	si:ch211-200p22.4	0.253400454	0.014916994	0.250923866	0.013632532
ENSDARG00000029204	tyrp1a	0.721072914	0.00000497	0.443735129	0.006030664
ENSDARG00000029232	FO704779.1	1.554067615	0.000117545	1.153285039	0.00524164
ENSDARG00000029252	ssb	-0.245966906	0.045825949	-0.341899413	0.002612116
ENSDARG00000029290	stx11b.1	0.572692863	0.000194681	0.377906505	0.017530901
ENSDARG00000029406	h2ax	-1.193747996	1.58E-09	-0.551581359	0.006950812
ENSDARG00000029439	atp2a2a	0.542395808	0.000129182	0.463924294	0.000732753
ENSDARG00000029443	zgc:92242	0.526388243	0.003025461	0.58682958	0.000409414
ENSDARG00000029445	eif1b	-0.361292925	0.000656299	-0.401765108	0.0000424
ENSDARG00000029587	msra	-0.555667813	0.006955488	-0.65099992	0.000693112
ENSDARG00000029596	trim55a	0.889655024	3.33E-12	0.366686113	0.007884836
ENSDARG00000029615	zgc:77056	-1.12014334	7.74E-17	-0.749519591	1.09E-09
ENSDARG00000029668	crim1	0.6923767	0.000288544	0.455480676	0.021352417
ENSDARG00000029692	rufy3	-0.628156728	1.52E-09	-0.28545065	0.008085455
ENSDARG00000029710	lrrc30a	1.2546292	0.00000381	0.780067209	0.006331732
ENSDARG00000029832	slc26a1	-0.828837957	0.00000164	-0.681353304	0.000029

Table S3. DEGs of WT vs. *terfa+/-* ∩ *terfa-/-*

ENSDARG00000029841	tmprss9	-0.635837125	0.013565647	-0.583175072	0.022350461
ENSDARG00000029944	parpbp	-0.871418393	0.000713239	-0.530668579	0.046913217
ENSDARG00000030006	slc6a7	0.449067176	0.012391975	0.383577539	0.034721024
ENSDARG00000030092	tram2	0.500301473	0.00078062	0.453503214	0.001714254
ENSDARG00000030106	stmn4	0.414753432	0.035872599	0.472168844	0.01273402
ENSDARG00000030116	susd6	0.20332082	0.042558077	0.330202185	0.00020388
ENSDARG00000030156	naglu	0.465206142	0.014027123	0.39468834	0.040249678
ENSDARG00000030161	ppp1r14bb	-0.371402907	0.018245583	-0.441953904	0.002827976
ENSDARG00000030177	uchl3	-0.374041291	0.004050397	-0.311410515	0.016176651
ENSDARG00000030224	ppp2ca	-0.366466649	0.00098671	-0.280707811	0.011751863
ENSDARG00000030263	mfsd2b	-0.928168955	0.0000056	-0.49285454	0.015747979
ENSDARG00000030265	scdb	-0.995175272	3.57E-17	-0.461607308	0.0000744
ENSDARG00000030307	hspa12b	-0.403578433	0.01156769	-0.412883943	0.007330815
ENSDARG00000030311	tmc2b	-1.027500318	0.00880765	-1.210330875	0.0009816
ENSDARG00000030326	efna1a	-0.783536332	5.66E-12	-0.42205134	0.000149565
ENSDARG00000030349	cryba2a	-0.865079332	0.000762134	-1.408303935	3.52E-10
ENSDARG00000030357	zgc:66313	2.182247828	0.000000066	1.183601052	0.006425163
ENSDARG00000030364	angpt1	0.69609187	0.00000123	0.397884339	0.008065555
ENSDARG00000030367	metrn	-0.824652749	7.25E-08	-0.370485988	0.022005736
ENSDARG00000030408	rps26l	0.63696253	0.000000317	0.462916094	0.000131413
ENSDARG00000030440	rsrp1	0.63669398	0.000067	0.488815891	0.001976099
ENSDARG00000030448	sppl2	-0.372126603	0.028018621	-0.346945152	0.040219106
ENSDARG00000030465	tmem263	0.594127101	0.000105434	0.355251266	0.027670313
ENSDARG00000030514	acsl1a	0.457791451	0.000145754	0.465066519	0.0000382
ENSDARG00000030626	lrit2	-3.884130937	8.09E-15	-1.023219884	0.021826701
ENSDARG00000030694	atp6v1e1b	-0.872056331	1.74E-12	-0.427140153	0.000612711
ENSDARG00000030722	xirp1	-0.378000389	0.001334599	-0.464159965	0.0000156
ENSDARG00000030832	otofa	-0.622978531	0.000219731	-0.426639759	0.012577245
ENSDARG00000030887	phf23a	-0.333593122	0.042756929	-0.350014782	0.029576872
ENSDARG00000030913	cluha	0.487448023	0.00000955	0.260043827	0.025931915
ENSDARG00000030915	cpa1	-3.971203074	5.68E-17	-3.187440174	1.67E-15
ENSDARG00000031044	lipg	0.302856079	0.038740001	0.342326647	0.015009438
ENSDARG00000031203	commd1	0.750983557	0.002806357	0.821416143	0.000503296
ENSDARG00000031293	dnajc9	-0.700365834	0.000029	-0.360966238	0.041738551
ENSDARG00000031435	TXN	0.484591448	0.00014782	0.387719707	0.002059606
ENSDARG00000031483	col9a1b	-0.743707186	0.046200533	-1.010978601	0.00348389
ENSDARG00000031562	tada2a	-0.630630597	0.0000789	-0.355891743	0.032757688
ENSDARG00000031600	rd3	-1.358028151	1.08E-19	-0.667132023	0.00000132
ENSDARG00000031616	g6pca.1	1.698606159	6.85E-10	0.630458152	0.040846762
ENSDARG00000031632	chst10	-0.571846607	0.000170842	-0.353258706	0.023556824
ENSDARG00000031680	ercc1	-0.508301155	0.014883895	-0.492015777	0.015588867
ENSDARG00000031683	fosab	-0.664291285	0.01954116	-1.140504403	0.00000505
ENSDARG00000031751	npr1a	1.140317516	1.4E-10	0.396259885	0.048558062
ENSDARG00000031774	pus7	-0.353116776	0.008710564	-0.327417549	0.013179117
ENSDARG00000031814	dhrs13b.1	1.237892688	3.64E-10	0.748084003	0.000183055
ENSDARG00000031952	mb	-0.474951586	0.002430119	-0.698955809	0.000000545
ENSDARG00000031981	pcbd1	1.432953405	0.000000362	0.835922966	0.004609108
ENSDARG00000032056	arl6	-0.919921254	3.71E-09	-0.334316852	0.045716473
ENSDARG00000032098	c8g	-0.840485079	0.000685185	-0.53265677	0.038337023
ENSDARG00000032103	mapk6	-0.59212924	0.000253122	-0.365925194	0.030419417
ENSDARG00000032206	cthl	-0.871998899	0.009286666	-0.801033754	0.014520879
ENSDARG00000032242	tnnt2c	1.374562126	5.41E-13	0.574861161	0.004265417
ENSDARG00000032263	zgc:110224	-1.437070137	0.00000417	-0.721309475	0.023183858
ENSDARG00000032373	rnf145b	-0.37006261	0.00024889	-0.253922463	0.013179117
ENSDARG00000032405	ckap4	0.820332726	4.1E-12	0.334393667	0.007765894

Table S3. DEGs of WT vs. *terfa+/-* ∩ *terfa-/-*

ENSDARG00000032426	coq8ab	0.774499742	0.001981554	0.571079299	0.027930695
ENSDARG00000032496	pon1	0.632879436	0.000643843	0.605115225	0.000657084
ENSDARG00000032650	fuk	0.494647866	0.020742116	0.552818565	0.006821096
ENSDARG00000032653	sinhcaf	-1.326403629	7.04E-18	-0.460267295	0.003174233
ENSDARG00000032725	rps27a	0.283068615	0.015096804	0.265412788	0.021517089
ENSDARG00000032765	net1	0.459070784	0.0000318	0.311673618	0.005231573
ENSDARG00000032820	rxfp2a	-2.395383886	1.2E-10	-2.089998892	4.16E-10
ENSDARG00000032849	ndrg1a	-0.865911483	4.54E-10	-0.707512021	0.00000005
ENSDARG00000032929	cryba1l1	-1.66832157	6.57E-13	-1.970226629	1.46E-21
ENSDARG00000033160	nr1d1	2.026888811	1.05E-16	2.597153903	3.55E-33
ENSDARG00000033161	sst1.2	0.943132209	0.0000038	0.484171008	0.030102713
ENSDARG00000033172	nr2f5	0.446575831	0.001888663	0.30515326	0.04159915
ENSDARG00000033231	mcm6l	2.09915519	0.003181727	1.527382053	0.044941499
ENSDARG00000033251	osbpl3a	0.713832805	0.00000126	0.457886792	0.00225465
ENSDARG00000033273	primpol	-1.196505256	0.0000013	-0.663409552	0.008215711
ENSDARG00000033327	unc5b	0.558689305	0.00031016	0.381828332	0.016486513
ENSDARG00000033382	grifin	-1.628563568	0.00000038	-1.406298202	0.00000253
ENSDARG00000033443	mdm2	2.793154892	2.64E-72	1.119310487	4.71E-14
ENSDARG00000033444	map4k6	-1.169584105	4.66E-17	-0.804988792	2.11E-10
ENSDARG00000033537	p4ha1a	0.864964032	1.55E-11	0.545072053	0.000014
ENSDARG00000033594	ido1	-1.387442853	0.004774743	-1.689943477	0.000207159
ENSDARG00000033616	selenon	0.55444567	0.0000136	0.331067185	0.01239836
ENSDARG00000033899	ap2m1b	0.269757554	0.04126239	0.357462361	0.003658399
ENSDARG00000034007	prom1b	-2.372669594	4.7E-45	-0.531424716	0.002810348
ENSDARG00000034048	zgc:92275	-1.114121505	0.0000023	-1.059429463	0.00000101
ENSDARG00000034063	unm_sa911	0.959620974	0.002697242	0.992412687	0.001128686
ENSDARG00000034173	prkcq	-0.530165351	0.020756307	-1.77845812	5.86E-20
ENSDARG00000034187	calm1b	-0.559550218	0.0000368	-0.344780101	0.013498538
ENSDARG00000034211	capn2l	1.082971491	8.3E-09	0.861253939	0.00000135
ENSDARG00000034227	si:dkey-243i1.1	-1.500784713	1.18E-13	-0.942411023	0.000000562
ENSDARG00000034268	slit3	0.706433693	1.58E-12	0.311534361	0.002763539
ENSDARG00000034291	rpl37	0.653239907	5.46E-08	0.540603087	0.00000189
ENSDARG00000034293	hif1ab	0.490293814	1.89E-08	0.367982183	0.0000106
ENSDARG00000034424	atp1b2b	-1.873092766	4.74E-22	-0.578865241	0.004375153
ENSDARG00000034453	unc119a	-2.325006967	4.97E-22	-1.216228798	2.29E-08
ENSDARG00000034503	per2	1.024271118	7E-12	1.097818136	3.52E-16
ENSDARG00000034605	zgc:153169	0.533035723	0.03078344	0.507683039	0.039886584
ENSDARG00000034624	nuf2	-1.204907504	1.27E-08	-0.473702821	0.036779978
ENSDARG00000034757	zdhhc2	-1.42684384	1.18E-09	-0.55613783	0.016147424
ENSDARG00000034777	txn2	0.627659872	0.000000246	0.319930162	0.01246617
ENSDARG00000034823	copg2	0.415537305	0.000472473	0.336348174	0.004265222
ENSDARG00000034836	si:dkey-222n6.2	1.141915346	3.01E-08	0.810694162	0.0000667
ENSDARG00000034883	acbd5a	-0.642759601	0.000131961	-0.461297236	0.005952729
ENSDARG00000034940	slc1a7a	-2.175925333	4.64E-14	-0.625965124	0.040219106
ENSDARG00000035018	thy1	1.547427816	2.42E-11	1.106374756	0.000000602
ENSDARG00000035028	lpcat4	-1.041714837	6.8E-11	-1.278830412	2.88E-19
ENSDARG00000035066	ubtf	-0.843080389	2.98E-17	-0.436632065	0.0000074
ENSDARG00000035088	si:ch211-254c8.3	7.130359624	0.00000222	6.857360686	0.00000955
ENSDARG00000035131	surf4l	0.894562625	4.24E-10	0.77274292	7.14E-09
ENSDARG00000035133	pho	-0.497738668	0.036382371	-0.542953867	0.018295219
ENSDARG00000035136	selenow1	0.792094877	2.03E-09	0.29427949	0.044195412
ENSDARG00000035152	ap2b1	0.304627944	0.01173602	0.284016138	0.017456015
ENSDARG00000035308	grb10b	1.458809817	0.000000204	1.234431752	0.00000397
ENSDARG00000035329	capns1a	0.814938412	1.02E-11	0.482546168	0.0000424
ENSDARG00000035350	ins	1.004887442	1.53E-17	0.526912819	0.00000834

Table S3. DEGs of WT vs. *terfa+/-* ∩ *terfa-/-*

ENSDARG00000035398	enc1	0.395731461	0.000432503	0.280220178	0.014565932
ENSDARG00000035438	myhc4	1.821466076	3.06E-21	1.423730414	1E-15
ENSDARG00000035471	si:dkey-220k22.1	0.521232631	0.0000153	0.321077783	0.009992366
ENSDARG00000035544	etnppl	-0.707193824	0.001457404	-0.545392844	0.014615723
ENSDARG00000035559	tp53	2.584357944	1.47E-107	1.159210993	3.03E-26
ENSDARG00000035578	hs3st1l2	-1.245738467	9.89E-11	-0.401761733	0.047194522
ENSDARG00000035598	coro1ca	0.662544918	1.38E-10	0.316940684	0.003090113
ENSDARG00000035629	parvab	0.798447769	0.0000189	0.511016706	0.008310612
ENSDARG00000035732	arntl1b	-0.935949203	1.58E-11	-0.701569916	5.96E-08
ENSDARG00000035751	ipo7	0.224453092	0.00641824	0.199643721	0.014569318
ENSDARG00000035797	si:dkey-17m8.2	7.062045933	1.52E-13	7.027134227	3.29E-13
ENSDARG00000035798	gngt1	-3.468806249	6.65E-11	-1.346683007	0.017553193
ENSDARG00000035809	col1a1b	0.384303422	0.002234595	0.382738626	0.001454611
ENSDARG00000035835	eef2k	1.080589196	2.95E-17	0.57850276	0.00000393
ENSDARG00000035859	angptl4	-0.319056529	0.008612346	-0.334636893	0.00407922
ENSDARG00000035860	rps28	1.164427593	6.22E-15	0.811300437	8.8E-09
ENSDARG00000035861	si:rp71-39b20.4	-3.407828269	1.5E-17	-0.933973121	0.010080669
ENSDARG00000035868	spire1a	-0.483437567	0.000619429	-0.401182497	0.003731253
ENSDARG00000035872	hsd17b12b	-0.558324037	0.0000159	-0.3082108	0.022146059
ENSDARG00000035887	zgc:91944	-0.568569355	0.0000647	-0.460632995	0.000816406
ENSDARG00000035891	acana	0.568715741	0.000146964	0.692551217	0.000000355
ENSDARG00000035957	gmnn	-1.118345507	4.76E-08	-0.437206706	0.043617308
ENSDARG00000036044	rps20	0.709020246	7.3E-09	0.505792388	0.0000182
ENSDARG00000036139	lctla	-1.832161698	4.07E-15	-1.884896104	2.04E-19
ENSDARG00000036140	crybgx	-1.231029999	9.78E-17	-1.956976896	4.26E-50
ENSDARG00000036144	tssc4	-0.588176183	0.00000162	-0.300832	0.018607929
ENSDARG00000036152	gas2b	-0.753833631	0.036531285	-0.840856376	0.015272029
ENSDARG00000036168	nfatc1	0.599665085	0.0000132	0.534789969	0.0000445
ENSDARG00000036227	npvf	1.260116508	0.01482986	1.421474562	0.003941678
ENSDARG00000036237	slc27a2a	0.577697194	0.005295018	0.630659377	0.001289721
ENSDARG00000036252	rras2	0.552042326	0.0000303	0.382119786	0.004217108
ENSDARG00000036291	nucb2b	0.823572251	0.00000159	0.440706607	0.015376802
ENSDARG00000036305	phf23b	-0.328882877	0.045973759	-0.418315349	0.00688274
ENSDARG00000036316	rpl39	0.545542504	0.0000421	0.348735237	0.010595292
ENSDARG00000036329	ndufa1	0.775825067	3.65E-09	0.508389666	0.0000814
ENSDARG00000036371	acta1a	1.548153195	2.44E-23	1.20627733	3.05E-17
ENSDARG00000036414	si:ch73-335l21.1	0.319189543	0.039451083	0.379456314	0.009992366
ENSDARG00000036482	hexim1	-0.479258618	0.0000857	-0.313156999	0.011758623
ENSDARG00000036541	rhbdf1a	0.79358802	4.05E-17	0.269451141	0.008453453
ENSDARG00000036558	col18a1a	0.978212763	1.12E-20	0.430396423	0.0000415
ENSDARG00000036613	tab1	0.473223555	0.000288846	0.400695513	0.001714254
ENSDARG00000036629	rps14	0.376040134	0.004793863	0.307767956	0.022005736
ENSDARG00000036671	tnni4b.2	1.199131459	6.1E-09	0.507998741	0.026456221
ENSDARG00000036684	ndufa7	0.730399717	1.29E-09	0.399255142	0.00117223
ENSDARG00000036700	si:ch211-114n24.6	1.081288857	0.00609668	0.99182812	0.010593865
ENSDARG00000036754	hmgn3	-1.378284748	5.68E-17	-0.665349557	0.0000423
ENSDARG00000036764	hax1	0.720757989	0.0000159	0.488203148	0.003913775
ENSDARG00000036809	ANPEP	-0.400855677	0.026691637	-0.44223737	0.010895049
ENSDARG00000036830	krt91	1.001756488	1.73E-14	0.355568497	0.011470507
ENSDARG00000036832	cyt1l	1.592926524	5.46E-25	1.24188781	1.54E-18
ENSDARG00000036870	arih1l	-0.313938716	0.027011196	-0.343631456	0.011748713
ENSDARG00000036875	rps12	1.88538643	8.86E-08	1.006623357	0.005740295
ENSDARG00000036876	zgc:153284	1.480158976	3.84E-14	0.623551737	0.002774949
ENSDARG00000036897	sgms2b	0.943682951	0.000000826	0.553224145	0.005824183
ENSDARG00000036966	hsd3b7	-0.410514221	0.043285265	-0.712364837	0.0000694

Table S3. DEGs of WT vs. *terfa+/-* ∩ *terfa-/-*

ENSDARG00000037018	gab1	0.489536379	0.000100764	0.270245487	0.045716473
ENSDARG00000037042	ddhd1b	0.455844009	0.005618296	0.572072419	0.000153408
ENSDARG00000037064	galca	0.683142358	0.000755941	0.493155929	0.01738807
ENSDARG00000037071	rps26	0.548200929	0.0000721	0.49060443	0.00020236
ENSDARG00000037073	si:dkey-60a16.1	-0.577831628	0.002556312	-0.442624451	0.020762042
ENSDARG00000037121	mat2ab	0.705606024	0.0000142	0.659335194	0.0000168
ENSDARG00000037186	tpbgb	0.487023723	0.001822205	0.388993215	0.013413907
ENSDARG00000037238	smad5	0.397042992	0.00153783	0.26116701	0.047363131
ENSDARG00000037262	cdkn2a/b	3.490618839	8.43E-09	1.96979965	0.002727803
ENSDARG00000037281	fgg	1.244578191	1.27E-22	0.320281543	0.024359589
ENSDARG00000037285	mipa	-2.134731502	9.58E-10	-1.807061955	2.59E-08
ENSDARG00000037337	cnrip1b	-1.947604829	2.52E-20	-0.603782429	0.004834029
ENSDARG00000037402	lim2.3	-0.751128606	0.001398616	-0.981582871	0.00000402
ENSDARG00000037421	egr1	-1.454623576	4.4E-20	-1.353586694	2.72E-21
ENSDARG00000037432	haus7	-0.555922769	0.007068918	-0.566183827	0.004210377
ENSDARG00000037487	01-Mar	-2.04425541	1.12E-13	-0.813090107	0.002614703
ENSDARG00000037488	canx	0.323282904	0.000734996	0.473565954	2.32E-08
ENSDARG00000037517	slc35c2	0.730737965	1.34E-12	0.363689044	0.000507887
ENSDARG00000037539	tnnc1b	1.02602227	3.44E-19	0.460060529	0.000064
ENSDARG00000037574	rps6kal	-0.444682365	0.000157717	-0.314461349	0.007943452
ENSDARG00000037587	SYNPR	-2.061005514	4.76E-26	-0.867091696	0.00000175
ENSDARG00000037655	pls3	0.795283142	1.18E-12	0.251204502	0.045506295
ENSDARG00000037738	fbxl3l	-1.74003741	6.28E-08	-1.512072267	0.000000226
ENSDARG00000037746	actb1	1.023273776	8.55E-26	0.653655921	7.2E-13
ENSDARG00000037747	fscn1b	-0.663374763	0.00000055	-0.560553833	0.00000665
ENSDARG00000037804	phlda3	2.184071081	6.31E-48	1.025566129	2.02E-12
ENSDARG00000037837	ogfr	-0.587982258	0.00000166	-0.27840438	0.032873513
ENSDARG00000037838	unkl	-0.774362048	7.01E-15	-0.444219606	0.00000345
ENSDARG00000037859	il11a	5.613507662	1.87E-11	2.398335573	0.013627869
ENSDARG00000037870	actb2	0.929937279	1.53E-22	0.659296527	8.41E-14
ENSDARG00000037873	cyp3c3	2.907145662	1.42E-08	1.955900451	0.00017878
ENSDARG00000037874	cyp3c3	4.775121162	0.000000184	4.889922196	4.35E-08
ENSDARG00000037879	lfng	-0.294261592	0.047378754	-0.313872889	0.030526848
ENSDARG00000037914	znfl1	-2.590330636	0.015477168	-2.00821386	0.048918197
ENSDARG00000037916	cdk5r1a	-0.837495668	0.00000468	-0.387627436	0.043838941
ENSDARG00000037919	rbbp6	-0.399172178	0.0000885	-0.341321401	0.000505557
ENSDARG00000037943	cpt1cb	1.113993596	6.65E-11	0.555610322	0.00172617
ENSDARG00000037961	rcn3	1.192984978	2.25E-14	1.118253868	4.39E-15
ENSDARG00000038025	cbx7a	3.096485163	1.05E-29	1.247631817	0.00000595
ENSDARG00000038028	ndufa6	0.6804253	0.000000155	0.42909244	0.000934818
ENSDARG00000038030	rbx1	0.766358038	0.000000807	0.732730091	0.000000398
ENSDARG00000038052	si:ch211-149k23.9	-1.011606551	0.001478316	-0.685990619	0.030133367
ENSDARG00000038106	slc37a4a	0.709774621	0.000161928	0.418920738	0.035704946
ENSDARG00000038147	hbbe3	6.272414884	0.0000304	5.633152685	0.0000943
ENSDARG00000038151	zgc:92360	0.874976836	8.5E-15	0.319877967	0.008500535
ENSDARG00000038185	gh1	-1.051493714	0.000350617	-1.309203284	0.00000101
ENSDARG00000038205	her2	-2.410035874	9.46E-19	-1.076894171	0.00000507
ENSDARG00000038213	slc35b1	0.473182328	0.0000327	0.339802531	0.002909533
ENSDARG00000038248	ggact.2	0.599450783	0.027135686	0.813621301	0.001059507
ENSDARG00000038363	drd4a	-2.560410501	2.25E-11	-1.107737451	0.003182178
ENSDARG00000038378	sagb	-3.011325891	8.49E-18	-0.998819125	0.007605633
ENSDARG00000038392	si:dkeyp-52c3.7	7.120991475	0.0000283	7.2563045	0.0000217
ENSDARG00000038422	entpd4	0.499474327	0.0000825	0.294280484	0.028058671
ENSDARG00000038428	sulf1	0.544018199	0.000000391	0.255217834	0.027023258
ENSDARG00000038465	stmn3	-0.737197974	1.86E-09	-0.501107008	0.0000183

Table S3. DEGs of WT vs. ***terfa+/-*** ∩ ***terfa-/-***

ENSDARG00000038476	si:dkeyp-68b7.7	-0.827622375	0.0000104	-0.467588051	0.01311749
ENSDARG00000038608	sdhc	0.67400925	4.46E-09	0.417221091	0.000251451
ENSDARG00000038609	mpz	-1.633186829	1.67E-08	-1.182839097	0.0000133
ENSDARG00000038635	magoh	-0.695674396	0.00000194	-0.438629991	0.002752278
ENSDARG00000038639	elovl6l	1.112568728	6.28E-09	1.099042088	3.84E-10
ENSDARG00000038643	alas2	0.794232812	0.000251053	0.891556462	0.00000837
ENSDARG00000038667	fggy	1.354462212	4.26E-12	1.338736641	5.98E-14
ENSDARG00000038668	gbp1	0.964569169	0.003165724	0.876579038	0.00638549
ENSDARG00000038742	rbp1.2	-3.556819565	4.8E-23	-1.34618415	0.0000196
ENSDARG00000038743	copb2	0.397985571	0.000172623	0.341616542	0.000892496
ENSDARG00000038780	ubtfl	-0.824604677	2.81E-14	-0.545980361	0.000000101
ENSDARG00000038785	abcf2a	0.38655966	0.001611055	0.316681494	0.00950372
ENSDARG00000038858	setd7	0.570054214	0.0000841	0.53888899	0.0000873
ENSDARG00000038862	kcnb2	-2.147628078	1.58E-19	-0.593484355	0.01687606
ENSDARG00000038863	babam2	-0.559393378	0.0000196	-0.36786807	0.004990456
ENSDARG00000038879	ift80	0.350099132	0.028369389	0.377903629	0.014429204
ENSDARG00000038894	tmx3a	-3.953154683	5.21E-48	-1.050409223	0.0000151
ENSDARG00000038969	zgc:113142	-1.257596704	1.79E-08	-1.293543386	1.19E-10
ENSDARG00000039066	klhl31	0.730639391	7.18E-14	0.254156224	0.016962146
ENSDARG00000039077	tyr	1.346405014	7.28E-13	0.799021851	0.0000147
ENSDARG00000039099	aep1	0.781095234	0.000000038	0.429742794	0.003114464
ENSDARG00000039117	tefa	0.447126618	0.000873121	0.634396448	0.000000113
ENSDARG00000039133	lamb4	0.699496403	1.51E-08	0.36678903	0.004084982
ENSDARG00000039142	arpc5a	0.611342996	4.02E-08	0.255840236	0.035643091
ENSDARG00000039173	ctslb	-1.75968911	0.012943571	-1.472446983	0.031507294
ENSDARG00000039211	zgc:77439	1.306287863	3E-25	0.696189373	1.01E-08
ENSDARG00000039215	arrb2a	-0.653170072	0.00000233	-0.440035484	0.001196327
ENSDARG00000039225	vps13a	0.522864868	0.00000114	0.296246901	0.007670301
ENSDARG00000039268	emc4	-1.230000131	0.00000732	-1.490698822	1.81E-09
ENSDARG00000039328	ccndbp1	1.263622515	1.54E-12	0.68808385	0.000115402
ENSDARG00000039338	gadd45gip1	-0.328621195	0.011377229	-0.353518122	0.004325051
ENSDARG00000039351	ccl19b	2.20823963	0.000000068	1.064328387	0.018460712
ENSDARG00000039393	si:ch211-240l19.5	-2.480314281	0.00000554	-1.939793101	0.000207753
ENSDARG00000039436	il13ra2	-0.506866964	0.03606038	-0.892853233	0.0000303
ENSDARG00000039456	acbd6	-0.452946913	0.001695875	-0.299005657	0.046038493
ENSDARG00000039459	qsox1	0.925888881	1.71E-18	0.419553211	0.0000819
ENSDARG00000039563	loxl3b	0.534028628	0.000000415	0.312584545	0.003840053
ENSDARG00000039579	cfd	2.241647585	7.63E-59	1.477931891	4.27E-31
ENSDARG00000039613	poll	-0.901651618	1.03E-10	-0.81527745	1.19E-10
ENSDARG00000039626	nrgna	-1.570672947	3.03E-12	-0.813082398	0.000222576
ENSDARG00000039652	si:dkey-235d18.5	1.406834448	4.48E-25	0.589774535	0.0000196
ENSDARG00000039677	dsc2l	1.309364507	1.62E-28	0.764976255	8.91E-12
ENSDARG00000039682	si:ch211-121a2.2	-2.343444318	0.000995348	-4.997285196	9.26E-15
ENSDARG00000039752	si:dkey-167k11.5	0.846154227	0.001194282	0.729057719	0.004658982
ENSDARG00000039754	xpc	0.432166509	0.008870951	0.611921111	0.0000369
ENSDARG00000039820	mea1	-0.468309578	0.000041	-0.487485601	0.00000383
ENSDARG00000039832	gsta.2	4.042043001	7.08E-42	3.053765203	3.82E-27
ENSDARG00000039900	si:ch73-168d20.1	6.754305554	0.0000443	5.90096884	0.000711727
ENSDARG00000039931	slc25a33	0.859967135	0.000000222	0.614534156	0.000152235
ENSDARG00000039937	ccdc172	-2.011873458	0.005944628	-1.520464965	0.027758429
ENSDARG00000039964	fgfbp2a	0.967286549	0.000181388	0.75002211	0.004005311
ENSDARG00000040001	fryl	0.329701406	0.014667837	0.272346214	0.048205382
ENSDARG00000040072	scpep1	0.820076916	0.00000773	0.641745718	0.000322543
ENSDARG00000040087	epb41l4a	0.640453369	0.0000876	0.619455145	0.0000591
ENSDARG00000040157	glt8d1	0.618720718	0.00000087	0.373519609	0.003884722

Table S3. DEGs of WT vs. *terfa+/-* ∩ *terfa-/-*

ENSDARG00000040180	slc35a1	0.530975302	0.00090159	0.475267471	0.002289036
ENSDARG00000040245	kpnb3	0.382189007	0.00786915	0.32498054	0.024569148
ENSDARG00000040265	mpdu1b	0.527807984	0.001554944	0.82679417	1.45E-08
ENSDARG00000040274	scamp5b	-2.686944985	5.35E-24	-0.631107895	0.029984471
ENSDARG00000040334	mat2aa	1.037235374	2.69E-21	0.737256223	4.45E-13
ENSDARG00000040352	crot	-0.391841431	0.002348775	-0.276315111	0.037120832
ENSDARG00000040362	ehd2b	1.337454451	1.27E-20	0.615357111	0.0000183
ENSDARG00000040387	mmd	0.365549494	0.006112095	0.358603223	0.00559798
ENSDARG00000040396	bcl7bb	-0.433783016	0.018226231	-0.374607759	0.042210994
ENSDARG00000040401	slc25a20	0.542465207	0.000000932	0.295494005	0.010311799
ENSDARG00000040432	klf2b	-0.67384202	1.22E-09	-0.549614502	0.000000103
ENSDARG00000040505	yif1b	0.927150258	0.00000113	0.757146178	0.0000359
ENSDARG00000040528	lgals3bpb	-0.551699759	0.014029242	-0.896337029	0.00000705
ENSDARG00000040534	epcam	0.597669012	1.44E-08	0.419655349	0.0000398
ENSDARG00000040565	ckmb	0.901279123	4.73E-11	0.332086794	0.026584876
ENSDARG00000040607	rtf2	-0.563555046	0.0000618	-0.349762942	0.015251337
ENSDARG00000040623	fosl2	1.833184845	1.4E-15	0.593458712	0.019236681
ENSDARG00000040649	prickle1a	0.495229366	0.001577224	0.327715299	0.047247393
ENSDARG00000040668	lrrc51	2.303868838	0.00000156	1.161724002	0.037806595
ENSDARG00000040727	tfb1m	-0.632549646	0.00496956	-0.619747952	0.003974058
ENSDARG00000040764	id1	1.640952406	8.79E-09	1.062832708	0.000175184
ENSDARG00000040881	hnrnph1	0.414077031	0.004865321	0.828723059	2.18E-11
ENSDARG00000040912	kdelr3	0.675220279	0.000280085	0.77773558	0.00000521
ENSDARG00000040942	pnp6	-1.054928129	0.000000477	-0.554013401	0.00950709
ENSDARG00000040944	ntd5	1.251258638	5.14E-23	0.646385127	0.00000017
ENSDARG00000041051	mid1ip1a	0.479078083	0.000375782	0.360355156	0.007851893
ENSDARG00000041078	chka	-0.688462922	0.00022856	-0.58324665	0.001305051
ENSDARG00000041098	barx2	0.780477949	0.0000405	0.415292308	0.045199407
ENSDARG00000041108	ctsh	0.937896104	0.00032727	0.591224174	0.031277198
ENSDARG00000041140	ddb2	0.658100632	0.0000828	0.649179111	0.0000371
ENSDARG00000041141	cryba1a	-3.283887137	6.49E-10	-2.37949145	2.17E-08
ENSDARG00000041205	slc6a1l	-2.363676709	0.000000017	-1.052434347	0.010944943
ENSDARG00000041215	cetn4	-0.501343375	0.018517662	-0.516356722	0.012060521
ENSDARG00000041217	xpo6	0.48124092	0.00000596	0.276734815	0.012380681
ENSDARG00000041220	ftr53	1.464459581	0.000264251	1.081403238	0.010006112
ENSDARG00000041232	rps29	1.120264341	1.32E-15	1.048801448	1.92E-16
ENSDARG00000041239	wdr20b	-0.561120129	0.008132803	-0.687458045	0.000464433
ENSDARG00000041257	smtnl1	0.491342471	0.035314903	0.519599552	0.022662644
ENSDARG00000041294	noxo1a	1.258774604	0.0000031	1.53350344	2.54E-10
ENSDARG00000041295	lim2.5	-0.891707699	0.00204991	-0.856738771	0.002026015
ENSDARG00000041339	zgc:92380	1.7050266	4.27E-44	1.021029381	3.32E-19
ENSDARG00000041340	mrpl51	-0.358288561	0.009176658	-0.376719112	0.004227541
ENSDARG00000041363	dctn3	-0.41764102	0.002014164	-0.49008821	0.0000842
ENSDARG00000041400	ndufa3	0.848429954	1.37E-09	0.58707998	0.0000153
ENSDARG00000041414	bmf2	-1.432168831	2.07E-09	-0.994667524	0.00000561
ENSDARG00000041433	si:dkey-7c18.24	2.538619605	0.0000015	1.687829026	0.001930134
ENSDARG00000041435	uba52	0.428220108	0.001165532	0.288952588	0.034995166
ENSDARG00000041572	zfpm1	0.524783733	0.000973895	0.443997471	0.004735078
ENSDARG00000041589	adprhl1	0.660507096	0.021539581	0.627822296	0.028273817
ENSDARG00000041602	zgc:92335	0.521396035	0.000651056	0.323783069	0.045962991
ENSDARG00000041619	rack1	0.298400952	0.025533441	0.273683105	0.041213905
ENSDARG00000041691	bhlhe41	1.040719025	2.89E-08	1.029968098	2.33E-09
ENSDARG00000041703	rrbp1b	1.307061665	1.63E-25	0.440419951	0.000751322
ENSDARG00000041811	rps25	0.420651163	0.000199568	0.283950569	0.014228039
ENSDARG00000041830	tsn	-0.600281883	0.000291953	-0.420691658	0.011732123

Table S3. DEGs of WT vs. *terfa+/-* ∩ *terfa-/-*

ENSDARG00000041839	tsc22d2	0.384309265	0.00037531	0.285876333	0.008702118
ENSDARG00000041864	capn3a	-2.660809208	1.65E-35	-2.445820691	3.14E-37
ENSDARG00000041870	ift172	0.30278977	0.010809494	0.368470745	0.000833876
ENSDARG00000041925	cryba2b	-0.635020318	0.0000764	-0.928422937	5.55E-11
ENSDARG00000041947	styk1b	0.545168348	0.000763728	0.447678602	0.005413935
ENSDARG00000041952	prox2	-1.227869515	0.00000298	-1.641867212	4.78E-12
ENSDARG00000042107	cngb1b	-2.166823529	2.73E-21	-0.903197873	0.0000414
ENSDARG00000042130	zp3a.2	3.53102403	1.64E-12	1.221542287	0.045740467
ENSDARG00000042189	tspan33b	-1.399063142	0.004184284	-1.72760655	0.000141165
ENSDARG00000042221	mthfd1l	0.778886172	3.11E-15	0.32977521	0.001248926
ENSDARG00000042245	myl13	0.871632707	3.84E-14	0.320647208	0.009343963
ENSDARG00000042387	zgc:77752	-2.97117585	2.48E-15	-1.011260181	0.001943315
ENSDARG00000042440	p2rx7	2.234642032	0.000575968	1.565426139	0.022281018
ENSDARG00000042469	ndufa12	0.665917178	7.17E-10	0.365550242	0.000818498
ENSDARG00000042535	actc1a	0.966075388	3.49E-12	0.733356533	1.93E-08
ENSDARG00000042545	sema3ga	0.925815107	0.000000167	0.385172581	0.049776293
ENSDARG00000042563	mis18bp1	-0.799388464	0.001061767	-0.538819419	0.030681794
ENSDARG00000042613	crp3	2.558165979	0.000134779	2.247021024	0.000525699
ENSDARG00000042641	cyp51	0.808339551	0.000000044	1.324014018	1.86E-25
ENSDARG00000042658	acad8	1.042333619	1.61E-15	0.760713112	7.93E-10
ENSDARG00000042659	thyn1	1.099529785	0.00734071	0.882062628	0.036564804
ENSDARG00000042688	bora	-1.01034693	0.000195951	-0.673591752	0.012945124
ENSDARG00000042717	adipor1b	0.512550033	0.000026	0.367645439	0.002607688
ENSDARG00000042727	exo5	1.455161235	1.73E-16	0.618382187	0.001108036
ENSDARG00000042777	ndufa11	0.719272176	0.0000154	0.637456149	0.0000567
ENSDARG00000042780	apoba	-0.375405946	0.003851471	-0.422930334	0.00050361
ENSDARG00000042793	tpp1	0.814599971	0.00000414	0.719195586	0.0000176
ENSDARG00000042823	spcs2	0.289153867	0.010866606	0.426608817	0.0000249
ENSDARG00000042839	dph6	0.781462297	0.0000352	0.706552371	0.0000902
ENSDARG00000042846	disp2	-0.786609676	5.85E-08	-0.318281644	0.041999357
ENSDARG00000042861	ltk	-0.83503867	0.0000025	-0.602296417	0.000467946
ENSDARG00000042900	gtpbp1l	1.568593189	1.45E-26	0.441353448	0.006428275
ENSDARG00000042956	cyp2ad6	-0.803829537	0.001705304	-0.760482398	0.001832534
ENSDARG00000042969	znf1035	-0.540798788	0.0000356	-0.300865641	0.029132398
ENSDARG00000042970	gng8	-1.702724294	0.00000021	-1.076102639	0.000413106
ENSDARG00000042989	si:dkeyp-84f3.5	-0.59204285	0.0000735	-0.429207522	0.00375924
ENSDARG00000042990	cyp2p10	-1.212702892	0.006137316	-1.079644454	0.012853374
ENSDARG00000042993	prss1	-2.065226159	0.000492279	-1.651850197	0.004961043
ENSDARG00000043004	si:dkeyp-117h8.4	-0.787505273	0.001148155	-0.621314391	0.008678432
ENSDARG00000043035	capn3b	1.088215627	1.72E-21	0.332241256	0.007781833
ENSDARG00000043077	nisch	-0.494565476	0.0000169	-0.380328928	0.000650059
ENSDARG00000043079	MMP23B	-0.537497597	0.00494794	-1.009005994	1.97E-09
ENSDARG00000043081	ctsz	0.819154176	4.79E-08	0.516874241	0.000538247
ENSDARG00000043095	kctd6a	-0.540299343	0.000807441	-0.386950597	0.017540189
ENSDARG00000043126	blf	-0.892393927	0.004891683	-0.714324703	0.0224203
ENSDARG00000043130	notch2	0.553814516	0.0000331	0.404885818	0.002284129
ENSDARG00000043131	BX664625.1	-1.719983341	0.041839395	-3.115622712	0.000178673
ENSDARG00000043148	slc1a3b	1.531143154	1.61E-14	0.637029217	0.002200252
ENSDARG00000043154	ucp2	0.82844617	0.000000576	0.542352754	0.001020547
ENSDARG00000043168	cela1.5	-2.705200443	0.013010489	-2.650647297	0.010026983
ENSDARG00000043241	arrb1	-0.541570393	0.005300558	-0.415198997	0.034995166
ENSDARG00000043242	si:dkey-222f2.1	-0.604062953	0.00000386	-0.338497488	0.012055164
ENSDARG00000043243	prkchb	1.028881603	1.23E-08	0.795915545	0.000005
ENSDARG00000043323	lnx1	2.310278345	5.37E-57	1.060752527	1.65E-14
ENSDARG00000043361	nck2b	0.419979755	0.003045197	0.354876986	0.012045316

Table S3. DEGs of WT vs. *terfa+/-* ∩ *terfa-/-*

ENSDARG00000043442	zgc:153665	0.500264815	0.003652934	0.693425683	0.00000693
ENSDARG00000043446	efhd1	-1.280815847	1.68E-15	-0.448539103	0.007734095
ENSDARG00000043493	cltca	0.355279644	0.002039464	0.34752281	0.001671137
ENSDARG00000043555	tmem30ab	-0.242621321	0.034992446	-0.292926622	0.007111808
ENSDARG00000043569	dram2a	2.382695347	0.000016	2.432927984	0.00000427
ENSDARG00000043571	KIF2A	-0.723351111	1.19E-09	-0.345805855	0.004661598
ENSDARG00000043581	gadd45aa	0.957383694	3.07E-10	0.467704769	0.003515075
ENSDARG00000043589	ca4a	-0.773958236	0.001696091	-0.759198491	0.001258016
ENSDARG00000043680	ylpm1	0.514163771	0.0000182	0.765621448	3.39E-13
ENSDARG00000043701	gpd1a	0.828226041	0.0000323	0.519119001	0.011607251
ENSDARG00000043719	c3a.6	1.865726487	9.94E-28	0.491208326	0.008899278
ENSDARG00000043722	cpa4	-2.506137307	0.0000206	-2.382475922	0.0000156
ENSDARG00000043770	si:dkey-192d15.2	1.172380462	0.00000327	1.031688029	0.0000164
ENSDARG00000043781	psmb10	1.729122096	4.48E-25	0.652688393	0.000209072
ENSDARG00000043795	arhgdia	0.524689995	0.000000263	0.313506353	0.002418473
ENSDARG00000043816	st6galnac1.2	1.786369081	0.0000757	1.010854925	0.045037143
ENSDARG00000043818	CASKIN2	0.847045809	0.000246731	0.486269378	0.049776293
ENSDARG00000043843	akap7	0.696389248	0.0000369	0.401739811	0.023692971
ENSDARG00000043873	arid4a	-0.520423212	9.67E-08	-0.241378951	0.019443184
ENSDARG00000044010	loxl2a	1.268021675	1.29E-27	0.772377647	2.43E-12
ENSDARG00000044155	mafaa	0.353494807	0.004965336	0.340382628	0.005413096
ENSDARG00000044179	rbms2a	0.289326298	0.011523399	0.322984337	0.002956686
ENSDARG00000044182	stau1	0.38864247	0.00000394	0.318109629	0.0000821
ENSDARG00000044183	prkab1a	-0.499760623	0.0000781	-0.356849868	0.004424867
ENSDARG00000044199	gnat1	-3.301743833	9.25E-13	-1.275253984	0.009137534
ENSDARG00000044304	prrc1	0.297139037	0.00508316	0.302886659	0.002934909
ENSDARG00000044325	zgc:193690	-1.141763587	0.000604087	-1.330090306	0.0000139
ENSDARG00000044328	ankrd46a	-0.58001317	0.000000167	-0.395354262	0.00024081
ENSDARG00000044332	zranb2	0.312759528	0.037904932	0.327007218	0.026757162
ENSDARG00000044356	tp63	0.459432236	0.00000122	0.365661591	0.0000598
ENSDARG00000044365	angptl3	0.817630337	0.000012	0.61109187	0.000920835
ENSDARG00000044420	dnajc19	0.792531164	0.000114918	0.773537035	0.000069
ENSDARG00000044431	ppig	-0.727389635	0.0000014	-0.340480863	0.034676023
ENSDARG00000044457	gfi1ab	-0.627402515	0.009065619	-0.525484779	0.027425938
ENSDARG00000044485	sall4	0.711874948	0.0000008	0.369039034	0.015890173
ENSDARG00000044501	viml	-1.559838125	0.00000334	-1.078701578	0.000560476
ENSDARG00000044521	eef1b2	1.09668512	9.42E-19	0.78001658	1.67E-11
ENSDARG00000044569	cldn19	-0.731533674	0.000487262	-0.510395529	0.016408531
ENSDARG00000044605	stx12l	-0.49115343	0.0000495	-0.362506518	0.002374323
ENSDARG00000044632	myo7ab	-1.545830497	1.28E-23	-0.440273816	0.004061893
ENSDARG00000044751	ddt	-0.321455775	0.034256157	-0.35186495	0.016447335
ENSDARG00000044776	clic3	1.58907262	0.000000434	1.37444619	0.00000613
ENSDARG00000044808	slc4a4b	0.638817969	0.0000666	0.346079224	0.044118311
ENSDARG00000044847	zgc:171599	-0.571749069	0.00828935	-0.46554312	0.032257152
ENSDARG00000044852	wbp2nl	0.482632438	0.0000151	0.268406403	0.02244974
ENSDARG00000044862	opn1lw1	-4.373851909	3.01E-08	-2.815002615	0.000000839
ENSDARG00000044875	crygm2e	-1.721212077	0.00253698	-1.565200666	0.003076153
ENSDARG00000044894	zgc:113307	-0.522428289	0.003463983	-0.424109111	0.01726644
ENSDARG00000044925	cdk5rap1	0.672519837	0.0000662	0.466749467	0.006967009
ENSDARG00000044942	mrpl45	0.327921976	0.015099237	0.313648818	0.018549097
ENSDARG00000044982	dhrs3a	1.069176138	0.000627181	0.815106114	0.009594309
ENSDARG00000045019	aamp	0.469695315	0.0000146	0.341989932	0.001445582
ENSDARG00000045087	cdk5r1b	-1.055147694	2.68E-19	-0.317386271	0.011251318
ENSDARG00000045089	si:ch211-270n8.1	3.49288388	1.41E-13	2.412499457	9.17E-08
ENSDARG00000045129	fkbp10b	0.605275811	0.00000266	0.399339929	0.002087611

Table S3. DEGs of WT vs. *terfa+/-* ∩ *terfa-/-*

ENSDARG00000045131	id4	-1.114632439	6.66E-10	-0.396067159	0.043461557
ENSDARG00000045139	ca7	-1.800663878	6.85E-11	-0.545374053	0.041530715
ENSDARG00000045159	zdhhc4	0.380117173	0.01721661	0.351644971	0.027159275
ENSDARG00000045175	pofut2	0.40981782	0.019928422	0.422083484	0.013636525
ENSDARG00000045219	dkk1b	1.2329907	6.43E-09	0.638223291	0.004774884
ENSDARG00000045262	gsnb	0.576819444	0.00469138	0.503018277	0.012994545
ENSDARG00000045298	wipf2a	0.58524032	3.05E-09	0.307972609	0.002428866
ENSDARG00000045442	cpb1	-2.450763908	0.000221294	-2.003236234	0.002110365
ENSDARG00000045517	itih5	0.663373309	0.009941627	0.642803356	0.010761806
ENSDARG00000045522	acot15	1.658481024	8.09E-12	1.496933644	2.44E-11
ENSDARG00000045524	lamb1b	0.61463705	0.00000261	0.509066279	0.0000475
ENSDARG00000045543	atp6v1f	-0.548065433	0.00000129	-0.382096768	0.000556284
ENSDARG00000045548	lepb	7.504740626	2.3E-15	4.642844296	0.00000434
ENSDARG00000045580	lum	-0.643150545	3.3E-10	-0.542144582	1.16E-08
ENSDARG00000045601	cax1	1.150217819	0.000023	0.625204591	0.03177291
ENSDARG00000045636	rbl2	1.69274704	2.6E-46	0.630466251	0.000000086
ENSDARG00000045676	calua	0.54238471	0.0000319	0.376754104	0.004116616
ENSDARG00000045705	meig1	0.753118217	0.002819583	0.578506744	0.026476316
ENSDARG00000045773	PYURF	1.265072135	0.000303495	0.758348863	0.046239242
ENSDARG00000045776	cnbpa	-0.381813058	0.001130837	-0.372848784	0.000863652
ENSDARG00000045827	lyrm5b	1.82839933	0.007164272	1.440237606	0.04495069
ENSDARG00000045834	si:dkey-14d8.7	-1.86483921	0.009849442	-2.274771835	0.000799047
ENSDARG00000045835	si:dkey-14d8.6	-3.261581484	0.001152679	-3.064499236	0.001364661
ENSDARG00000045850	ccdc167	0.543083564	0.004149029	0.42491037	0.02825609
ENSDARG00000045863	asb15b	-1.390849485	0.008220545	-2.129876156	0.0000159
ENSDARG00000045885	scaf11	0.232416678	0.020032622	0.20039274	0.048020984
ENSDARG00000045898	si:ch211-152c2.3	-0.520318401	0.006632478	-0.500987238	0.007250562
ENSDARG00000045946	sec24d	0.87670502	1.64E-15	0.583850768	2.51E-08
ENSDARG00000045976	sidt2	0.876506507	6.85E-15	0.454734187	0.0000497
ENSDARG00000045979	zgc:153704	-1.03256023	6.77E-11	-0.390918265	0.020615801
ENSDARG00000045989	arf4a	0.528102413	0.0000389	0.290890924	0.033518886
ENSDARG00000046002	necap2	0.613484941	0.000000705	0.367804883	0.003628822
ENSDARG00000046030	zgc:110339	0.668577411	0.0000135	0.513340541	0.000637827
ENSDARG00000046053	slc27a6	-0.740367211	0.000024	-0.755293596	0.00000284
ENSDARG00000046133	b3galnt2	1.071558438	1.31E-09	0.503657963	0.007857865
ENSDARG00000046142	asgrl1	-1.133565082	0.029910315	-1.288071017	0.009807679
ENSDARG00000046157	RPS17	0.77485434	8.72E-09	0.606803825	0.00000201
ENSDARG00000051731	faap24	-2.023862011	0.0000304	-1.316835954	0.003076153
ENSDARG00000051748	ccnd2a	0.977678383	5.77E-13	0.322950305	0.033320526
ENSDARG00000051762	CABZ01080568.1	1.657818419	7.76E-38	1.034348075	1.42E-17
ENSDARG00000051853	galns	1.086850537	0.0000252	0.710728364	0.007165865
ENSDARG00000051857	tes	1.240648338	1.92E-16	0.583785862	0.000139611
ENSDARG00000051861	pkp3a	0.867084873	1.07E-15	0.465676751	0.0000122
ENSDARG00000051896	fbn2a	2.040868493	7.21E-43	1.590619535	4.27E-31
ENSDARG00000051914	slc14a2	2.779867952	5.23E-20	2.296027018	2.43E-15
ENSDARG00000051955	brms1	-0.588941765	0.000163564	-0.438883476	0.004341155
ENSDARG00000051981	STX3	-2.717398788	1.03E-44	-0.554200913	0.001282665
ENSDARG00000051989	tmem187	0.777226386	0.002653962	0.736538815	0.00353708
ENSDARG00000052011	rrad	2.523001313	4.65E-33	1.261687445	6.82E-10
ENSDARG00000052170	uap1	0.566295103	0.0000045	0.410897414	0.000761879
ENSDARG00000052244	zgc:158564	-0.2339877	0.024315565	-0.211564111	0.042670361
ENSDARG00000052279	osgn1	1.195972037	1.58E-20	0.452857492	0.000864808
ENSDARG00000052330	slc4a2b	0.810546074	8.67E-12	0.311129069	0.016213838
ENSDARG00000052351	si:dkey-156n14.3	-0.4661629	0.00058635	-0.28393994	0.046142995
ENSDARG00000052405	pak6b	1.124939804	0.000000123	0.807559596	0.000107629

Table S3. DEGs of WT vs. *terfa+/-* ∩ *terfa-/-*

ENSDARG00000052408	mgat2	-0.381508814	0.009024123	-0.440654076	0.001290251
ENSDARG00000052462	pisd	-0.614901178	0.00000466	-0.356509541	0.009379474
ENSDARG00000052578	c6ast4	-2.824239055	0.011429035	-2.703988365	0.013389014
ENSDARG00000052609	CU468164.1	-5.170418085	0.0000177	-6.387971334	0.00000421
ENSDARG00000052649	ube2ia	-0.523028157	0.000197407	-0.368891185	0.009226816
ENSDARG00000052652	fermt1	0.614204167	0.0000117	0.339620875	0.022005736
ENSDARG00000052690	arrdc3a	0.389909154	0.013499957	0.593109734	0.0000245
ENSDARG00000052712	suclg1	0.641435306	3.05E-09	0.24102788	0.043836383
ENSDARG00000052728	sltm	-0.860809004	7.2E-15	-0.588653883	0.000000017
ENSDARG00000052739	egf	-1.181912627	0.0000609	-0.71568533	0.017200735
ENSDARG00000052747	gpatch3	-0.57590078	0.000303551	-0.45046598	0.004179931
ENSDARG00000052779	zgc:153932	2.943318305	3.34E-15	1.064103593	0.009548001
ENSDARG00000052783	cdc42ep3	-0.409853402	0.019951881	-0.378241452	0.030799468
ENSDARG00000052842	ppifb	0.505150924	0.00021746	0.413745866	0.002089084
ENSDARG00000052846	fsta	0.642043679	8.66E-10	0.378130405	0.000314547
ENSDARG00000052855	ctso	0.663689993	0.002634966	0.646366505	0.002427356
ENSDARG00000052895	htra3a	2.994182332	6.41E-08	1.417381283	0.026492413
ENSDARG00000052905	zgc:165423	1.092104676	0.029751753	1.195857523	0.013636525
ENSDARG00000053097	hsf2	0.257269335	0.040079238	0.487357045	0.00000582
ENSDARG00000053155	alg3	0.633293248	0.0000051	0.525904522	0.0000834
ENSDARG00000053194	pdzd11	0.361709496	0.038588487	0.527522959	0.00084305
ENSDARG00000053269	slc2a15b	0.575958348	0.00263934	0.69471251	0.0000786
ENSDARG00000053279	apln	-0.518376969	3.29E-08	-0.232347693	0.019527005
ENSDARG00000053291	pnrc2	-0.589196018	0.00000233	-0.483174339	0.0000503
ENSDARG00000053323	zgc:112285	-1.197355679	0.000000004	-1.410062716	1.03E-14
ENSDARG00000053365	rpl31	0.630797722	0.0000158	0.473403303	0.000981134
ENSDARG00000053431	si:ch211-257p13.3	-0.574037416	0.0000028	-0.323872615	0.00950709
ENSDARG00000053450	ftr06	3.01353941	0.0000519	4.19142086	6.82E-10
ENSDARG00000053456	GK3P	-0.369893359	0.001366647	-0.45548955	0.0000155
ENSDARG00000053480	aqp9b	-2.882794079	9.13E-14	-1.003190004	0.01505119
ENSDARG00000053502	cryaa	-1.805388538	2.48E-16	-1.62745948	3.12E-16
ENSDARG00000053558	rtkn2a	-1.032504764	0.00000397	-0.818756762	0.000113372
ENSDARG00000053559	tspan3b	-1.098701565	3.26E-12	-0.455654747	0.00484031
ENSDARG00000053609	gatd1	-0.882052529	1.06E-08	-0.369818134	0.02204054
ENSDARG00000053624	csf1rb	-1.555743747	0.000000289	-0.968388471	0.000647049
ENSDARG00000053644	got1l1	2.061097076	0.00000383	1.117573812	0.022350461
ENSDARG00000053746	CABZ01084566.1	-1.949340127	2.95E-10	-0.684155784	0.03376057
ENSDARG00000053774	alpi.2	2.46939869	0.009937916	2.0703353	0.033889344
ENSDARG00000053792	si:rp71-1g18.1	-0.462576414	0.000923192	-0.329963191	0.019016717
ENSDARG00000053820	pcmtd2a	-1.182836547	0.000000613	-0.516117162	0.038035536
ENSDARG00000053831	vtnb	0.867285865	1.19E-15	0.693498254	4.97E-12
ENSDARG00000053853	slc13a2	1.730240218	2.21E-26	1.282112175	1.3E-17
ENSDARG00000053862	crygmx	-1.43695366	1.2E-15	-1.745096227	7.44E-28
ENSDARG00000053864	elmsan1b	0.797186265	2.77E-08	0.348150565	0.024634324
ENSDARG00000053875	cryba1b	-2.211570883	1.52E-10	-2.339791487	6.59E-14
ENSDARG00000053950	lin37	-0.477146547	0.024063554	-0.466758292	0.024657449
ENSDARG00000053953	myadml2	1.940484187	4.03E-12	0.886924775	0.003510583
ENSDARG00000053992	sfi1	-0.541021384	0.025624231	-0.751728951	0.00070465
ENSDARG00000054060	pof1b	0.851937875	3.11E-15	0.430038312	0.0000698
ENSDARG00000054076	tatdn3	1.31464922	0.001348404	0.986703816	0.021083855
ENSDARG00000054103	ripply1	-0.718715055	0.020227137	-0.686099532	0.023179096
ENSDARG00000054150	cx23	-1.400831238	0.0000608	-1.235771368	0.000187791
ENSDARG00000054191	pgk1	0.815259556	5.97E-12	0.277345862	0.034471559
ENSDARG00000054343	slc7a8b	-0.842726178	0.049902767	-1.16022404	0.003977968
ENSDARG00000054438	fzd2	-0.238091185	0.03964672	-0.286670338	0.009035346

Table S3. DEGs of WT vs. *terfa+/-* ∩ *terfa-/-*

ENSDARG00000054530	rars1	0.545556254	0.000000954	0.253539512	0.035920451
ENSDARG00000054537	nhsl1a	-0.924859574	1.02E-14	-0.473124929	0.000055
ENSDARG00000054543	samsn1a	-1.721588508	6.6E-25	-0.445941736	0.009984335
ENSDARG00000054578	arl6ip1	0.482959985	0.00000026	0.207598929	0.043211306
ENSDARG00000054584	FO681288.1	-0.805837315	0.002337109	-0.79955416	0.001400357
ENSDARG00000054616	cldni	1.055834259	1.97E-18	0.526351055	0.00000963
ENSDARG00000054641	tent5ab	-1.69891306	8.55E-09	-0.993784103	0.00058089
ENSDARG00000054666	pgpep1	0.87760442	4.96E-09	0.583905342	0.0000796
ENSDARG00000054744	si:ch211-244e12.7	-1.83557741	0.004052955	-1.979442753	0.000932261
ENSDARG00000054748	cuedc1b	-0.951855669	1.75E-12	-0.466171863	0.000479205
ENSDARG00000054804	anp32e	-1.507849912	8.16E-30	-0.453336509	0.001122311
ENSDARG00000054807	sec13	0.4022081	0.008600841	0.404036429	0.006428275
ENSDARG00000054814	ptp4a3b	-0.312950921	0.033943053	-0.381842997	0.006123318
ENSDARG00000054818	rpl32	0.437469512	0.000568232	0.329579044	0.009807679
ENSDARG00000054823	id3	-0.425603185	0.000656623	-0.311487003	0.013342057
ENSDARG00000054864	aplp2	0.681477486	0.001017019	0.511169945	0.014904722
ENSDARG00000054890	rgra	-0.492115768	0.007878045	-0.512280675	0.003812993
ENSDARG00000054898	ms4a17a.16	-2.028310495	0.015212488	-1.667839802	0.037659468
ENSDARG00000054934	zgc:101765	0.843371028	0.005119539	0.889551292	0.002059606
ENSDARG00000054973	itsn2b	0.323344753	0.000815402	0.209238931	0.038702769
ENSDARG00000054980	ebna1bp2	-0.470068746	0.005753365	-0.345798791	0.048581499
ENSDARG00000055014	si:dkey-33m11.8	-1.994997098	5.94E-15	-0.640143838	0.011022034
ENSDARG00000055045	casp3b	0.91813839	4.47E-11	0.569693569	0.000038
ENSDARG00000055092	pora	0.51466572	0.00000122	0.260869419	0.020743081
ENSDARG00000055100	cxcl12b	-0.496097149	0.0000214	-0.469359195	0.0000172
ENSDARG00000055118	mylipb	1.219600006	8.85E-12	0.780406758	0.00000812
ENSDARG00000055133	cenpf	-1.780438961	8.45E-20	-0.583880037	0.004557167
ENSDARG00000055177	pxdc1a	-1.489719403	0.000000573	-0.837420198	0.003477816
ENSDARG00000055190	slc17a5	-0.629231486	1.6E-09	-0.279887414	0.010314062
ENSDARG00000055270	si:ch1073-358c10.1	1.924143763	0.00000306	1.373335867	0.001051462
ENSDARG00000055276	rel	1.267360752	9.99E-10	0.494813911	0.03023931
ENSDARG00000055278	cfb	2.475932707	6.2E-56	0.807713044	0.000000243
ENSDARG00000055344	mipep	0.769517329	2.28E-09	0.444026543	0.000662053
ENSDARG00000055415	zp2l1	3.924661039	0.004665862	3.834953489	0.007355541
ENSDARG00000055439	adamtsl7	1.23132657	6.44E-13	0.760341561	0.00000624
ENSDARG00000055463	lrit3a	-2.681331759	5.86E-16	-0.846075496	0.012890939
ENSDARG00000055475	rps27.2	0.550701187	0.0000088	0.453451206	0.000140786
ENSDARG00000055477	pelo	0.400374668	0.001500993	0.262967579	0.047457067
ENSDARG00000055504	si:ch211-212k18.7	0.765705492	6.17E-14	0.396285963	0.000106889
ENSDARG00000055510	ypel3	-0.698091661	0.0000315	-0.407040389	0.018381631
ENSDARG00000055514	icn2	1.514008887	3.38E-39	1.090205978	8.58E-25
ENSDARG00000055524	rnf7	0.35589783	0.005183019	0.274135535	0.035659439
ENSDARG00000055527	cmn	-0.599500656	0.026897689	-1.075655182	0.00000521
ENSDARG00000055565	cacnb2b	-1.302353543	4.05E-12	-0.421658748	0.031770419
ENSDARG00000055592	capn2b	0.625669373	1.69E-08	0.248679797	0.042577132
ENSDARG00000055618	acta1b	1.144979659	1.99E-12	0.36541777	0.044713317
ENSDARG00000055620	acad9	0.447909477	0.0000595	0.290417625	0.011450092
ENSDARG00000055632	smtnl	1.152377342	1.25E-09	0.427344644	0.043409874
ENSDARG00000055679	mto1	0.99803525	7.37E-19	0.827391823	2.29E-15
ENSDARG00000055705	f5	1.373366484	0.000000055	0.613742667	0.02410324
ENSDARG00000055751	fosb	-1.135537043	0.0000188	-0.898069256	0.000358657
ENSDARG00000055786	prss23	0.509968431	0.000144492	0.310713957	0.027806493
ENSDARG00000055813	si:dkey-225f5.5	1.723559898	0.000000477	1.540479107	0.00000253
ENSDARG00000055965	klc3	-1.069251554	0.000292265	-0.651705402	0.02731965
ENSDARG00000055976	pecr	-0.38880468	0.013547056	-0.414888401	0.00588776

Table S3. DEGs of WT vs. *terfa+/-* ∩ *terfa-/-*

ENSDARG00000056090	capza1b	0.531988125	2.51E-08	0.212215588	0.043230584
ENSDARG00000056134	c1qtnf5	1.187441493	9.46E-12	0.621901172	0.000529458
ENSDARG00000056151	tyrp1b	0.712437573	0.000000186	0.390887034	0.005557063
ENSDARG00000056156	npdc1b	-0.366544453	0.014550184	-0.306840489	0.043520586
ENSDARG00000056160	hspd1	0.387345764	0.000934314	0.402059469	0.000266624
ENSDARG00000056186	eif5a2	-0.342613588	0.007415984	-0.375371235	0.002007263
ENSDARG00000056191	si:ch211-168k14.2	-0.788326932	0.020918006	-0.691442425	0.039473166
ENSDARG00000056239	tmem45b	1.072506393	2E-12	0.634843791	0.0000236
ENSDARG00000056307	znf706	0.63788887	0.000000625	0.33413071	0.012906742
ENSDARG00000056314	a2ml	-0.453065667	0.001995783	-0.318836433	0.03524865
ENSDARG00000056339	stk31	-1.401577263	0.002231203	-1.803131428	0.0000234
ENSDARG00000056347	rab3aa	-1.187698675	1.87E-17	-0.319426664	0.036573641
ENSDARG00000056369	ufsp2	0.499538952	0.001000037	0.451187467	0.002269296
ENSDARG00000056379	si:ch73-86n18.1	1.019496625	6.66E-08	0.453733459	0.027688081
ENSDARG00000056464	fitm1l	1.691071117	1.14E-18	0.932880315	0.000000696
ENSDARG00000056475	trnau1apb	-0.401160833	0.00011005	-0.358340196	0.000301138
ENSDARG00000056477	ccdc125	1.416829991	2.59E-08	0.664454942	0.015748819
ENSDARG00000056502	si:ch73-334d15.4	-0.841544318	0.004746519	-0.815009131	0.004686372
ENSDARG00000056525	bcar1	0.562365284	0.000000274	0.375444574	0.000533714
ENSDARG00000056541	nr0b1	-0.746976496	0.000430899	-0.538954259	0.009900866
ENSDARG00000056557	copb1	0.280723662	0.007398496	0.221047668	0.039242749
ENSDARG00000056590	calca	0.69925335	0.00000567	0.556985884	0.000212688
ENSDARG00000056600	papss2b	0.841598507	2.74E-17	0.703737457	1.55E-14
ENSDARG00000056640	rpia	0.67829265	6.49E-10	0.394394089	0.000329792
ENSDARG00000056680	stc2a	-0.360050038	0.017555395	-0.502727439	0.000270592
ENSDARG00000056691	cpeb4a	-0.452016381	0.0000437	-0.349635563	0.001197943
ENSDARG00000056723	fhad1	2.445608443	6.09E-11	2.00840599	2.02E-08
ENSDARG00000056725	hmgb3a	-1.736309337	1.11E-30	-1.099188748	4.5E-15
ENSDARG00000056744	ela2	-3.245613123	0.002785488	-2.579693071	0.018298874
ENSDARG00000056750	si:ch211-254p10.2	-1.766458454	0.0000716	-1.798274024	0.0000105
ENSDARG00000056767	itgb3a	1.316494448	5.27E-25	0.557654455	0.0000138
ENSDARG00000056784	aire	-2.775717114	0.009759677	-2.032990096	0.037171331
ENSDARG00000056829	zmp:0000000662	0.528008916	0.0000917	0.454189284	0.000513934
ENSDARG00000056855	gatc	-0.622709958	0.001052054	-0.492257147	0.008916167
ENSDARG00000056873	and3	0.687922646	2.03E-08	0.470786051	0.0000807
ENSDARG00000056885	per1a	2.352349042	8.03E-40	2.193592195	8.83E-42
ENSDARG00000056914	emg1	-0.414395263	0.022015827	-0.37296881	0.038823115
ENSDARG00000056921	ankk1	1.059895715	0.00000263	0.51115504	0.03971946
ENSDARG00000056922	ltbp1	0.508463981	0.00011886	0.311308782	0.02420914
ENSDARG00000056938	kera	-1.559233424	3.88E-13	-0.949963081	0.00000453
ENSDARG00000056986	dact2	0.463627062	0.032163949	0.429412704	0.048844531
ENSDARG00000057039	si:ch211-191j22.8	-1.569770742	0.006280408	-1.211823623	0.030453596
ENSDARG00000057121	c7b	2.272410461	2.27E-18	1.057937727	0.000072
ENSDARG00000057159	ankrd29	-0.876618481	0.000211138	-0.542405109	0.022655569
ENSDARG00000057169	abca4a	-1.706987746	2.67E-15	-0.80056733	0.000125647
ENSDARG00000057231	rorca	-1.090480281	6.71E-10	-1.003758073	5.43E-10
ENSDARG00000057245	im:7138535	-0.566810966	0.00470498	-0.647991573	0.000523041
ENSDARG00000057249	zmynd11	-0.560478681	1.05E-08	-0.378288388	0.000068
ENSDARG00000057303	galnt7	0.696422732	5.22E-14	0.596940747	2.72E-12
ENSDARG00000057321	tut1	-0.56363192	0.0000266	-0.387730041	0.003336836
ENSDARG00000057334	cmasa	0.557496498	0.000000826	0.433734465	0.0000728
ENSDARG00000057365	elovl8b	1.607364589	7.56E-08	1.403884447	0.000000759
ENSDARG00000057429	arg1	-1.540430019	0.001118855	-1.5511941	0.000426499
ENSDARG00000057465	gfpt1	0.743176041	0.000000022	0.358982722	0.010246028
ENSDARG00000057529	itpa	0.697231009	0.002041779	0.585956684	0.009343963

Table S3. DEGs of WT vs. *terfa+/-* ∩ *terfa-/-*

ENSDARG00000057556	rpl17	0.391051007	0.000093	0.32989778	0.000670104
ENSDARG00000057652	dbpb	1.363976688	1.27E-11	1.843784343	2.51E-25
ENSDARG00000057665	sprn2	-0.646888824	0.001477157	-0.963960789	0.000000116
ENSDARG00000057698	ctsd	0.722032696	0.0000156	0.473506379	0.005201629
ENSDARG00000057707	zgc:66443	-0.497666726	0.003822066	-0.346250402	0.049755759
ENSDARG00000057736	ano10a	0.787373705	2.94E-12	0.481711871	0.0000134
ENSDARG00000057867	lasp1	0.399607204	0.0000255	0.247490493	0.011360275
ENSDARG00000057879	p3h3	0.63388853	0.0000141	0.564829125	0.0000476
ENSDARG00000057881	si:dkey-33c12.3	0.831212487	0.000849059	0.603476208	0.017530901
ENSDARG00000057911	ACTC1	2.595076173	1.17E-37	2.37921311	8.57E-39
ENSDARG00000057912	eif1axb	-0.322413182	0.001431507	-0.314702998	0.001128686
ENSDARG00000057992	fstb	1.201362856	7.01E-14	0.374206083	0.038599911
ENSDARG00000058004	six2a	-0.327776492	0.013552709	-0.268785913	0.045705398
ENSDARG00000058082	birc7	-1.719640974	8.42E-11	-1.507163007	3.08E-10
ENSDARG00000058094	ciarta	0.660298717	0.00000055	0.894519846	1.69E-14
ENSDARG00000058102	sardh	0.979662758	2.13E-12	0.411356154	0.005151064
ENSDARG00000058158	trim55b	1.661460339	6E-21	0.850177536	0.000002
ENSDARG00000058159	ipo8	0.787173216	0.006319449	0.680257034	0.018268103
ENSDARG00000058206	si:ch211-153b23.5	1.199821092	7.59E-33	0.735081599	6.82E-15
ENSDARG00000058222	mpp4a	-1.957453969	1.12E-15	-0.749599995	0.001753747
ENSDARG00000058325	casp8	1.808809036	1.16E-17	0.751553244	0.000734402
ENSDARG00000058357	ankrd33ba	-2.200299756	3.52E-23	-0.986625546	0.00000122
ENSDARG00000058358	krt8	0.944543066	6.33E-15	0.467172573	0.000115399
ENSDARG00000058366	si:dkey-222f8.3	-0.475911739	0.003449704	-0.449147102	0.004350615
ENSDARG00000058371	krt5	0.863023168	5.28E-22	0.474013935	3.52E-08
ENSDARG00000058381	zgc:171775	-0.358591519	0.004106071	-0.37888671	0.001373857
ENSDARG00000058419	gcn1	0.394673486	0.000826615	0.324651347	0.005492568
ENSDARG00000058444	snx6	-1.09248503	0.0000845	-1.246137866	0.000000945
ENSDARG00000058448	zrsr2	-0.694267587	0.0000074	-0.533351259	0.000300872
ENSDARG00000058454	dynll1	0.423821863	0.000826615	0.578673692	0.000000348
ENSDARG00000058537	si:dkey-102c8.2	2.313450911	0.0000146	1.952144294	0.000211956
ENSDARG00000058543	lama5	0.864176153	1.75E-10	0.516605716	0.00011021
ENSDARG00000058553	sprb	2.330048005	6.31E-15	2.035735229	2.41E-13
ENSDARG00000058556	muc5.2	1.150840386	1.41E-08	0.897348117	0.00000337
ENSDARG00000058557	il11b	4.434153228	0.000251494	3.092402006	0.019443184
ENSDARG00000058574	abcg2c	0.619213813	0.00000924	0.536076727	0.0000591
ENSDARG00000058593	sri	-0.35119826	0.001930681	-0.559032862	1.92E-08
ENSDARG00000058601	gdap1	-0.455650328	0.010071885	-0.374302347	0.035642486
ENSDARG00000058732	scgn	-0.68564441	0.037286613	-0.659641611	0.042491201
ENSDARG00000058753	si:ch73-213k20.5	2.511850594	0.00000309	1.724607122	0.002012825
ENSDARG00000058794	si:dkey-19a16.2	-1.925700172	1.01E-08	-1.332469452	0.000021
ENSDARG00000058803	grk1a	-2.831266372	3.18E-24	-0.913586855	0.001108036
ENSDARG00000058815	ihhb	-0.928428507	0.026578283	-0.867779599	0.035104525
ENSDARG00000058819	nog1	-0.507990555	0.016541962	-0.433547464	0.041490116
ENSDARG00000058865	endog	0.562334314	0.003834138	0.410167228	0.041867421
ENSDARG00000058940	si:ch211-248e11.2	1.731594999	6.38E-08	1.128273767	0.000523755
ENSDARG00000058988	KCNJ15	0.631051095	0.00040668	0.506109553	0.004523162
ENSDARG00000059035	porb	1.432745616	3.07E-17	0.836332511	0.000000341
ENSDARG00000059097	nktr	-0.606978956	0.000000194	-0.484919928	0.0000119
ENSDARG00000059109	si:dkeyp-113d7.1	-0.45629891	0.00141161	-0.30814913	0.037631562
ENSDARG00000059150	tmem107	0.400951049	0.036169142	0.601944499	0.000454666
ENSDARG00000059177	tax1bp3	0.817003001	0.000000273	0.352617701	0.043461557
ENSDARG00000059280	hoxd3a	-0.834157601	2.78E-13	-0.353991738	0.002198121
ENSDARG00000059294	marco	2.828182734	2.37E-39	0.972548229	0.0000152
ENSDARG00000059323	rpn1	0.30269456	0.018565639	0.328030612	0.007912299

Table S3. DEGs of WT vs. *terfa+/-* ∩ *terfa-/-*

ENSDARG00000059349	homer2	0.600961634	0.001412641	0.448460799	0.019443184
ENSDARG00000059351	hnrnpa3	0.521232505	0.023169744	1.030171336	0.000000121
ENSDARG00000059357	sarnp	-0.656874537	0.000000198	-0.376585893	0.003122729
ENSDARG00000059373	dnajc8	0.486851556	0.007566773	0.503390895	0.004099198
ENSDARG00000059398	myef2	0.78248767	0.00000432	1.06578911	1.65E-12
ENSDARG00000059442	smtnb	-1.337165387	1.27E-13	-0.65117578	0.000299221
ENSDARG00000059461	mepce	-0.7134577	6.91E-10	-0.382101676	0.000781855
ENSDARG00000059466	wasf3a	-1.957777579	1.46E-18	-0.626461709	0.004123414
ENSDARG00000059556	recql5	0.825523879	0.0000134	0.528464846	0.006487091
ENSDARG00000059557	xylt2	0.855432049	0.000143235	0.735541629	0.000778984
ENSDARG00000059682	slc43a3a	-2.144287678	2.05E-25	-0.973303095	7.95E-08
ENSDARG00000059714	arsk	2.548389119	0.00000665	2.785238978	0.000000126
ENSDARG00000059746	plod1a	0.959647256	8.2E-10	0.563284307	0.000301842
ENSDARG00000059751	abtb2a	0.614217252	0.048810778	1.143296919	0.0000207
ENSDARG00000059792	trpm5	-1.826620234	2.83E-16	-0.48034043	0.035814255
ENSDARG00000059794	kdm6al	-0.518324607	0.0000183	-0.35874141	0.002971172
ENSDARG00000059824	soat2	-0.421372749	0.035148955	-0.655451298	0.000275396
ENSDARG00000059826	crtac1a	-2.120146887	1.33E-12	-2.057661552	2.74E-14
ENSDARG00000059835	zfyve27	0.431440456	0.002668342	0.330623863	0.02396977
ENSDARG00000059836	ddit3	-0.610805064	6.71E-08	-0.544231118	0.000000211
ENSDARG00000059897	ntrk2a	0.376859865	0.021924437	0.354661296	0.030215714
ENSDARG00000059903	hs3st3l	-2.479138693	1.65E-26	-0.589193204	0.007155934
ENSDARG00000059978	cplx4a	-3.103723047	6.93E-34	-0.991568015	0.0000987
ENSDARG00000059993	trpm4a	0.975683065	7.1E-13	0.645380275	0.000000826
ENSDARG00000060001	mettl9	0.733721185	0.0000114	0.61089637	0.000152281
ENSDARG00000060065	ubap2b	0.39533845	0.0000192	0.206410851	0.03712988
ENSDARG00000060094	ptgis	0.891688267	0.000000695	0.534933657	0.004087316
ENSDARG00000060106	crb2a	0.952323444	1.62E-10	0.338296149	0.045602388
ENSDARG00000060127	adamts3	1.190392309	6.47E-14	0.501309501	0.002982001
ENSDARG00000060153	h6pd	1.157456723	1.58E-14	0.430913661	0.007991159
ENSDARG00000060184	pkn1a	0.293818209	0.018760629	0.361337025	0.001975322
ENSDARG00000060196	lhpp	3.037637146	2.23E-50	2.636591822	3.11E-44
ENSDARG00000060238	uacab	-0.343741732	0.002981385	-0.480396277	0.00000371
ENSDARG00000060246	slc16a6b	0.603417351	0.000570823	0.453945855	0.01005864
ENSDARG00000060248	fgd4b	-1.843383104	3.49E-09	-0.87986673	0.003451784
ENSDARG00000060282	rnf121	0.396823521	0.021234749	0.377263672	0.02731965
ENSDARG00000060323	exoc5	0.811798099	5.09E-08	0.543944401	0.000222386
ENSDARG00000060338	trmt12	0.645100229	0.040587602	0.694073396	0.023912381
ENSDARG00000060345	apoda.1	-1.277846876	2.45E-13	-0.56361034	0.001212626
ENSDARG00000060350	apoda.2	-1.204740521	0.008332878	-1.047681035	0.021826701
ENSDARG00000060362	nadkb	0.463877595	0.001084352	0.33316285	0.022397751
ENSDARG00000060366	slc12a9	0.587161311	0.004724491	0.688597599	0.000362529
ENSDARG00000060383	mmaa	0.357988642	0.012930942	0.289985528	0.049707972
ENSDARG00000060410	thbs2a	0.910601754	0.00000903	0.607627386	0.003411761
ENSDARG00000060439	clcn2c	2.096088583	1.78E-21	1.494118741	4.41E-13
ENSDARG00000060452	sdk2b	0.349585681	0.046108118	0.406019012	0.015644377
ENSDARG00000060457	pmp22b	0.350954611	0.033682725	0.36569522	0.023746949
ENSDARG00000060518	pcsk5b	0.623957947	0.000000046	0.376549317	0.001061484
ENSDARG00000060526	bmp3	-0.571810846	0.00049275	-0.350749448	0.039911781
ENSDARG00000060622	si:ch73-14h1.2	1.340543542	0.00000655	1.104169617	0.000118284
ENSDARG00000060626	dgkaa	0.808433741	2.09E-17	0.296261092	0.003555013
ENSDARG00000060627	hip1ra	1.079264456	7.57E-08	0.626683648	0.002872324
ENSDARG00000060682	agr1	1.157507048	4.07E-15	0.605424434	0.0000355
ENSDARG00000060695	znf346	0.530608056	0.002096151	0.787143565	0.000000256
ENSDARG00000060797	pfkmb	0.893057619	1.62E-10	0.385910043	0.009322671

Table S3. DEGs of WT vs. *terfa+/-* ∩ *terfa-/-*

ENSDARG00000060841	pik3c2a	0.567500595	0.000000345	0.259899515	0.030867657
ENSDARG00000060854	kctd3	0.387277134	0.041089142	0.376063389	0.047153484
ENSDARG00000060885	znf592	-0.934841843	1.3E-09	-0.75528753	0.000000126
ENSDARG00000060900	znf362a	-0.734845185	1.43E-13	-0.448630222	0.00000223
ENSDARG00000060901	trim62.1	1.249679665	3.88E-08	1.304962938	3.5E-10
ENSDARG00000060951	polg	0.618691673	0.00135752	0.450693138	0.023135727
ENSDARG00000060954	tmcc1b	-0.409768671	0.0000992	-0.292789695	0.005386366
ENSDARG00000060983	smyd4	-0.666596755	0.00151824	-0.77477825	0.0000633
ENSDARG00000060994	fbxw7	0.276230431	0.012938993	0.258165955	0.01893085
ENSDARG00000061039	atp10a	0.925898351	0.00000371	0.659227602	0.000942074
ENSDARG00000061049	rnf182	-2.029608592	2.12E-09	-1.474515969	0.000000817
ENSDARG00000061101	snx19a	-3.0844315	3.27E-44	-0.77796666	0.00000519
ENSDARG00000061120	slc43a2b	1.082356289	7.3E-10	0.501102292	0.006452789
ENSDARG00000061124	srpra	0.354762889	0.000772587	0.243901722	0.025098002
ENSDARG00000061173	st14a	0.722108796	6.32E-12	0.447908083	0.0000123
ENSDARG00000061240	cbln4	0.981098609	0.018192027	1.0062131	0.013132231
ENSDARG00000061260	si:dkey-202e22.2	0.804400838	2.67E-08	0.407289885	0.007642965
ENSDARG00000061272	BX294434.1	0.484593779	0.0000906	0.35974177	0.003673945
ENSDARG00000061292	pi15b	-1.630650373	0.016387769	-1.595328589	0.013918855
ENSDARG00000061328	cdon	0.408824609	0.0000147	0.260069299	0.006915727
ENSDARG00000061335	galnt1	0.288576438	0.003370172	0.242854079	0.013376332
ENSDARG00000061357	chst6	0.721710027	0.0000425	0.542868324	0.002038311
ENSDARG00000061370	tsen34	0.424715502	0.02992746	0.474824431	0.011414641
ENSDARG00000061413	sec23ip	0.622163885	0.000000075	0.441742891	0.000089
ENSDARG00000061450	tesk2	-0.764750424	1.84E-09	-0.468668353	0.000140786
ENSDARG00000061451	n4bp2	-0.504398128	0.000438526	-0.533521753	0.0000639
ENSDARG00000061480	si:ch211-132b12.1	-2.073533515	0.002557189	-1.332969712	0.037296125
ENSDARG00000061585	cyp4v7	1.409430333	0.00000174	1.261252207	0.00000672
ENSDARG00000061587	ctdspl2a	-0.5426712	0.000000637	-0.291438427	0.009409673
ENSDARG00000061600	cemip2	0.651067423	0.00000182	0.363842731	0.010458417
ENSDARG00000061603	sorbs2b	-0.70768006	2.76E-11	-0.348327066	0.001174485
ENSDARG00000061763	spata5l1	0.982156809	0.0000949	0.540954984	0.048790076
ENSDARG00000061850	si:dkey-19b23.13	-0.675980115	0.014829806	-0.63590406	0.019643238
ENSDARG00000061858	zgc:153968	-2.033812425	0.000000937	-3.728819987	2.2E-22
ENSDARG00000061890	atp13a2	1.100186266	9.59E-17	0.93889168	2.11E-14
ENSDARG00000061896	slco2a1	1.122137151	5.73E-13	0.631912453	0.0000545
ENSDARG00000061921	gltpa	-0.376848722	0.043948708	-0.736771374	0.00000591
ENSDARG00000061923	amotl2a	-0.42979003	0.000118602	-0.293190046	0.009596335
ENSDARG00000061941	trpv4	0.733381956	0.000000597	0.391662349	0.011616742
ENSDARG00000061948	amotl2b	-0.387704319	0.011624532	-0.374688601	0.012492972
ENSDARG00000061977	ppfibp2a	-0.302339594	0.044281402	-0.396568696	0.004704872
ENSDARG00000062025	cpox	0.446530203	0.006521356	0.372281618	0.02469637
ENSDARG00000062030	gpr17	-1.446113286	1.41E-11	-0.598511828	0.003770575
ENSDARG00000062055	rnf38	-0.719949697	0.00000432	-0.478732589	0.002235875
ENSDARG00000062084	clcn1a	-0.654914309	0.011790011	-0.890788309	0.000177987
ENSDARG00000062122	klhl5	0.683994469	0.00126812	0.539753556	0.011514222
ENSDARG00000062132	cyp4v8	-0.959673646	0.0000115	-0.540496589	0.016632517
ENSDARG00000062146	larp4b	-0.376155527	0.000942067	-0.300820074	0.00761384
ENSDARG00000062147	otc	-1.413352412	0.000000276	-0.90642497	0.000534743
ENSDARG00000062156	abi3bpa	-1.061490672	0.000166714	-0.953229494	0.000338535
ENSDARG00000062190	pde3b	0.429584838	0.000539355	0.351949754	0.004210377
ENSDARG00000062304	pdpk1a	-0.623147318	0.000483945	-0.54297903	0.001572787
ENSDARG00000062307	ccdc61	-0.850576976	0.00000258	-0.507128145	0.005137128
ENSDARG00000062315	sik2b	0.823437799	2.29E-11	0.388369023	0.002411141
ENSDARG00000062361	paxbp1	-0.441585014	0.002926331	-0.305089964	0.047778058

Table S3. DEGs of WT vs. *terfa+/-* ∩ *terfa-/-*

ENSDARG00000062370	bcl2l13	0.715310025	4.04E-08	0.359796005	0.008461179
ENSDARG00000062423	zgc:153901	0.752885964	0.007359657	0.719926223	0.009065926
ENSDARG00000062460	SGK3	0.355799613	0.035348968	0.410251204	0.011044337
ENSDARG00000062562	egln2	0.918273141	0.00000788	0.559785853	0.008555966
ENSDARG00000062568	mrpl2	0.710436864	3.17E-10	0.547518213	0.000000305
ENSDARG00000062577	arhgap35a	0.442791466	0.0000487	0.33007961	0.002322407
ENSDARG00000062606	si:dkey-26i13.8	-1.819496184	9.31E-09	-0.857445969	0.007182845
ENSDARG00000062632	duox	1.314852685	0.005234942	1.546510733	0.000422587
ENSDARG00000062661	abca4b	-2.362200894	9.16E-25	-0.543229974	0.03124909
ENSDARG00000062749	ino80b	-0.328772711	0.013866969	-0.306252269	0.020155422
ENSDARG00000062750	si:ch73-74h11.1	1.022394314	1.76E-15	0.438157808	0.000973107
ENSDARG00000062788	irg1l	1.364816932	1.99E-12	0.962305159	0.000000193
ENSDARG00000062831	si:ch73-22o12.1	0.411519934	0.0000485	0.271254126	0.0088031
ENSDARG00000062865	mras	-0.672509567	0.000000169	-0.400456503	0.00181792
ENSDARG00000062892	foxe3	-1.620406344	0.0000786	-1.353238012	0.000318639
ENSDARG00000062902	si:dkey-220f10.4	-2.859244462	2.4E-35	-0.518809492	0.013636525
ENSDARG00000062906	kcnv2b	-2.906012514	1.26E-09	-1.310375267	0.000984572
ENSDARG00000062909	furina	0.807546146	2.07E-13	0.626166464	1.27E-09
ENSDARG00000062960	armc10	-0.502459173	0.001149101	-0.314615131	0.049707972
ENSDARG00000062970	niban2a	0.321618143	0.011477469	0.404135209	0.00057318
ENSDARG00000063126	gusb	0.820344056	9.9E-10	0.336527133	0.020707796
ENSDARG00000063159	si:dkey-32e23.4	0.605075188	0.010792281	0.724889028	0.001059507
ENSDARG00000063177	manf	0.559303725	0.000117439	0.566680158	0.0000311
ENSDARG00000063233	baz1a	-0.391361906	0.007181344	-0.30978309	0.034850708
ENSDARG00000063295	myh9a	1.078792266	4.76E-26	0.480537903	0.00000183
ENSDARG00000063297	abcb6a	0.976860235	5.42E-08	0.53708001	0.004030834
ENSDARG00000063309	tjp2a	0.448053529	0.000261976	0.357719665	0.003270012
ENSDARG00000063411	lrrc73	-0.834394758	0.000000176	-0.444383699	0.006023425
ENSDARG00000063412	cers1	-0.611112106	0.000047	-0.502881841	0.000490136
ENSDARG00000063435	trpm4b.2	1.268676075	1.52E-15	0.885360955	4.61E-09
ENSDARG00000063474	arfgef1	0.310839535	0.005895531	0.30628242	0.005136447
ENSDARG00000063475	abcg1	-1.37866387	5.92E-11	-1.000508673	0.000000187
ENSDARG00000063518	zgc:153913	0.676006677	0.0000111	0.329955999	0.048508366
ENSDARG00000063519	PLEKHB1	-2.117494873	8.79E-09	-0.737106111	0.045216577
ENSDARG00000063563	creb3l2	0.503017125	0.000181435	0.369392318	0.006331125
ENSDARG00000063570	dyrk1aa	-0.535441348	0.0000645	-0.460736467	0.000347955
ENSDARG00000063572	perp	0.810074438	4.24E-09	0.470565234	0.000659027
ENSDARG00000063594	hipk1a	0.877523912	0.021196518	1.130342121	0.001328755
ENSDARG00000063624	gfm1	0.429353104	0.000648546	0.272611692	0.039229564
ENSDARG00000063631	VIT	0.551086496	0.000000691	0.428835699	0.0000603
ENSDARG00000063634	hpse	-0.869435696	0.000011	-0.524191659	0.007678425
ENSDARG00000063661	nuak2	0.78835006	0.0000068	0.460979022	0.011540139
ENSDARG00000063665	mat2al	1.507357712	7.25E-18	0.532188382	0.004771301
ENSDARG00000063672	zmp:0000000711	0.534219651	0.0000969	0.346313132	0.014727649
ENSDARG00000063701	rreb1a	0.670766683	2.94E-09	0.524073873	0.00000102
ENSDARG00000063895	mt-nd1	-0.395359276	0.005569105	-0.543648535	0.0000242
ENSDARG00000063921	mt-nd5	-0.324378021	0.013296488	-0.5921942	0.000000174
ENSDARG00000067670	pomt1	0.807553713	0.0000361	0.583599352	0.002927812
ENSDARG00000067672	card9	-1.473365242	0.003941611	-1.744679517	0.000243321
ENSDARG00000067673	snapc4	-0.851001356	2.3E-09	-0.513321889	0.0001891
ENSDARG00000067711	parvg	0.87055254	0.005978714	0.79159258	0.012081716
ENSDARG00000067741	itpkcb	1.139609505	4.23E-13	0.587432373	0.000205774
ENSDARG00000067784	SLC9A1	0.778021433	0.000139385	0.522379226	0.014045935
ENSDARG00000067848	nmrk2	0.808420799	0.005750131	0.700758718	0.016408531
ENSDARG00000067859	scospondin	3.187708308	1.54E-28	1.486161187	0.000000162

Table S3. DEGs of WT vs. *terfa+/-* ∩ *terfa-/-*

ENSDARG00000067958	sh3gl1a	1.024840649	4.7E-11	0.617159658	0.0000635
ENSDARG00000067984	gas1b	-0.987980342	2.57E-10	-0.455274495	0.004106617
ENSDARG00000068050	agbl4	1.240759918	0.000502035	1.355720011	0.0000476
ENSDARG00000068096	atf5a	-0.392046042	0.000641012	-0.31571201	0.005363791
ENSDARG00000068217	stx2b	0.624339231	0.000178	0.357932751	0.045387664
ENSDARG00000068240	trim110	-1.103699409	0.00371979	-0.816600642	0.030892725
ENSDARG00000068242	cngb1a	-2.475212567	4.27E-17	-0.990772259	0.000695469
ENSDARG00000068288	lamc2	0.946110738	0.0000291	1.157131396	1.39E-08
ENSDARG00000068305	mrps14	0.489544826	0.002029644	0.527177587	0.00040397
ENSDARG00000068365	lmx1bb	0.435771426	0.002316087	0.470633891	0.000460253
ENSDARG00000068367	nfkbie	0.657210035	0.000130092	0.368230448	0.04636729
ENSDARG00000068374	si:ch211-132b12.7	2.811050006	1.36E-15	2.306193757	2.13E-12
ENSDARG00000068401	yap1	0.515196934	0.0000132	0.307597522	0.011858403
ENSDARG00000068428	si:ch211-153j24.3	0.603908784	0.034645562	0.71353587	0.008569072
ENSDARG00000068507	crybb1	-3.734810719	1.5E-20	-3.580817933	4.19E-23
ENSDARG00000068516	hapln1b	-0.802171152	0.000016	-0.446585797	0.02131872
ENSDARG00000068709	fam174b	-0.62203715	0.001535142	-0.447343315	0.024580818
ENSDARG00000068710	nid1a	-0.297073126	0.020468643	-0.506054608	0.00000669
ENSDARG00000068716	cuedc1a	-1.362358125	1.47E-19	-0.341123737	0.039019797
ENSDARG00000068732	spry4	0.502538839	0.000190167	0.303322351	0.032622475
ENSDARG00000068749	si:ch211-250k18.5	2.126539313	0.002907652	1.724164804	0.02183152
ENSDARG00000068755	exosc8	-0.555519028	0.007733574	-0.585855013	0.003238511
ENSDARG00000068812	tlr7	-3.334892773	0.000339943	-1.673882359	0.028751966
ENSDARG00000068830	zgc:172139	1.00234377	0.0000164	0.788981418	0.000571771
ENSDARG00000068840	zgc:66024	1.952994594	0.000320168	1.405900247	0.014873479
ENSDARG00000068893	mettl5	0.327683768	0.036417605	0.463977816	0.001108036
ENSDARG00000068910	nos1	1.254643331	0.00000007	0.842878129	0.000237988
ENSDARG00000068918	map2k2b	0.637269135	0.00064248	0.395533843	0.045618702
ENSDARG00000068919	rad51c	-0.8855384	0.010901287	-0.726222262	0.034609487
ENSDARG00000068965	nrip1a	-0.456070388	0.005126534	-0.358498595	0.03016775
ENSDARG00000068966	si:ch211-261n11.7	5.23412756	2.78E-30	5.184502031	9.19E-34
ENSDARG00000068969	zgc:153759	3.10420657	0.000000194	2.279656566	0.000144548
ENSDARG00000068992	hspa8	0.586192156	1.02E-10	0.340792195	0.000148068
ENSDARG00000069048	serpinf1	0.408276304	0.001739451	0.311620005	0.018460712
ENSDARG00000069074	cry3a	0.290956021	0.016011497	0.599021056	5.14E-09
ENSDARG00000069093	col2a1a	0.542322683	0.0000289	0.36492238	0.005401686
ENSDARG00000069111	ikzf2	1.01752277	0.000000114	0.52529641	0.010516589
ENSDARG00000069189	si:dkey-242h9.3	3.549323498	0.011592427	3.900373905	0.00376871
ENSDARG00000069261	metap2a	1.117696452	2.29E-09	0.792342679	0.0000145
ENSDARG00000069279	elovl7a	0.753214813	0.0000152	0.41599856	0.024142698
ENSDARG00000069280	tsen15	-1.025523654	0.000014	-0.690799476	0.003053829
ENSDARG00000069282	bbc3	1.738099667	3.06E-26	0.826035395	0.000000455
ENSDARG00000069283	ercc8	-0.580340959	0.001125135	-0.54696943	0.001328872
ENSDARG00000069296	moxd1l	-1.292243701	0.000000595	-0.535392523	0.047892653
ENSDARG00000069313	oxa1l	-0.676908443	0.00000917	-0.616673829	0.0000167
ENSDARG00000069361	spa17	-0.58559028	0.007423669	-0.484600854	0.027097523
ENSDARG00000069397	zswim8	0.338807296	0.003149693	0.310316272	0.005687253
ENSDARG00000069415	col17a1a	0.520284764	0.000373456	0.387136544	0.008510137
ENSDARG00000069451	gja8a	-2.318190869	4.14E-16	-2.099124505	2.2E-17
ENSDARG00000069463	alox12	0.574606762	9.27E-08	0.660342776	8.91E-12
ENSDARG00000069471	mhc1zca	-0.440102349	0.048454333	-0.794381859	0.0000591
ENSDARG00000069472	chsy3	0.591199199	0.0000376	0.576415746	0.0000186
ENSDARG00000069481	ghrh	3.687638832	0.00000734	2.189164336	0.013997858
ENSDARG00000069497	sumf2	0.531439715	0.015597016	0.511463438	0.018607929
ENSDARG00000069559	muc13a	-0.50896071	0.01882488	-0.445030264	0.041459819

Table S3. DEGs of WT vs. *terfa+/-* ∩ *terfa-/-*

ENSDARG00000069627	si:ch211-255g12.8	2.54507439	0.000998484	1.788208121	0.030133367
ENSDARG00000069669	adra2c	-0.575389367	0.022335268	-0.697457929	0.00319305
ENSDARG00000069673	chid1	0.636013478	0.000105707	0.615470801	0.0000724
ENSDARG00000069675	her8.2	-1.441602745	0.000000886	-0.731673216	0.010944943
ENSDARG00000069766	caln2	-0.788622582	0.006409941	-0.650187751	0.023824284
ENSDARG00000069815	dnali1	1.443216577	0.000016	1.115285945	0.000927514
ENSDARG00000069843	kctd12.1	-0.712058192	0.00062948	-0.55993653	0.006076706
ENSDARG00000069846	zgc:162944	0.534077614	0.0000236	0.402762177	0.001244212
ENSDARG00000069888	cldna	0.879331099	0.000124089	0.528108408	0.030487626
ENSDARG00000069909	HTRA2	0.733506628	0.00000316	0.507886288	0.001303706
ENSDARG00000069910	gtf2f2a	-0.548604889	0.00000335	-0.423009188	0.000196972
ENSDARG00000069934	parp4	1.542986557	1.26E-21	0.415654261	0.022005736
ENSDARG00000069946	itga6b	1.02573423	6.53E-09	0.538720484	0.003056518
ENSDARG00000069953	kcnq5b	-0.581903938	0.013273828	-0.484620923	0.039312028
ENSDARG00000069980	lman1	0.419637026	0.001139581	0.463544071	0.000112199
ENSDARG00000070000	txnipb	-0.782505151	0.000334025	-0.872702122	0.0000147
ENSDARG00000070006	rcn1	0.604167365	5.63E-08	0.390389481	0.00041739
ENSDARG00000070019	taf15	0.49156861	0.002596229	0.617373591	0.0000346
ENSDARG00000070025	dchs1a	0.400375145	0.027911454	0.374965873	0.039632241
ENSDARG00000070039	NA	-6.310895824	0.003568856	-6.321833047	0.001214507
ENSDARG00000070069	pitx3	-0.42680045	0.005450673	-0.385766709	0.010212617
ENSDARG00000070083	atp5f1b	0.533588646	0.00000354	0.309376872	0.009204282
ENSDARG00000070092	KCNV1	-1.553125301	0.0000805	-0.795586753	0.041530715
ENSDARG00000070108	dek	-1.092253863	6.22E-13	-0.437381338	0.005354617
ENSDARG00000070127	nfxl1	0.77804025	7.74E-08	0.688319503	0.000000398
ENSDARG00000070230	aldh1l2	0.734536243	0.002443926	0.65250799	0.006159665
ENSDARG00000070371	kat7a	-0.825203412	7.8E-09	-0.637545414	0.00000184
ENSDARG00000070387	nudt7	1.196185581	0.0000277	0.879980594	0.002273309
ENSDARG00000070394	uroc1	1.142833731	4.84E-10	0.503070595	0.010000255
ENSDARG00000070396	serpinb1l2	1.355283361	0.005092584	2.02880791	0.00000183
ENSDARG00000070427	s100v1	0.650307512	0.000000283	0.277026581	0.047153484
ENSDARG00000070437	rpl22	0.615168057	0.000000124	0.533855555	0.00000101
ENSDARG00000070465	si:dkey-13n23.3	-2.289112088	0.000251053	-2.278568217	0.0000812
ENSDARG00000070486	rbp7b	-1.244767574	0.005046168	-1.767676426	0.0000152
ENSDARG00000070494	pdgfra	-0.546980072	4.99E-08	-0.2564812	0.015349548
ENSDARG00000070513	brpf3a	-0.524675676	0.000107579	-0.329993219	0.01778103
ENSDARG00000070522	cacna1ia	1.006497296	0.00557102	1.041389445	0.002852067
ENSDARG00000070581	ggact.1	0.983406669	0.002105409	1.130603498	0.000140786
ENSDARG00000070651	prkcdb	1.060335272	5.47E-14	0.608810778	0.0000106
ENSDARG00000070673	ptgr2	0.672966838	0.00000351	0.508936955	0.000360926
ENSDARG00000070734	dyrk4	-0.557796465	0.002146525	-0.57984264	0.000732753
ENSDARG00000070826	bpgm	-0.666072616	0.00000371	-0.395593122	0.006536493
ENSDARG00000070834	taf13	-0.416077714	0.004226997	-0.336115459	0.021199651
ENSDARG00000070835	tnnc2	0.998451388	4.55E-18	0.4419197	0.000142476
ENSDARG00000070845	si:dkey-56d12.4	2.418404553	0.0000254	3.626765412	8.11E-13
ENSDARG00000070846	dazap1	-0.952996587	0.000000107	-0.656185595	0.000126931
ENSDARG00000070914	dusp6	0.512123862	0.0000121	0.269133473	0.030839613
ENSDARG00000070922	cnbpb	-0.281034806	0.005689336	-0.2960898	0.002264717
ENSDARG00000070957	im:7160594	0.658645366	0.004714702	0.64822634	0.004084982
ENSDARG00000071017	nt5e	-0.597456486	0.000499392	-0.405880485	0.019592434
ENSDARG00000071025	dytn	-0.764214843	0.042835534	-0.889702742	0.013511063
ENSDARG00000071062	hps5	0.882023796	0.000000113	0.440568433	0.012365358
ENSDARG00000071076	ldhbb	-1.594589446	1.04E-12	-0.949776808	0.0000092
ENSDARG00000071082	p4ha1b	0.815584685	0.002124472	0.679359375	0.01012263
ENSDARG00000071095	abi3bpb	-0.393082721	0.002556166	-0.330442748	0.010561044

Table S3. DEGs of WT vs. *terfa+/-* ∩ *terfa-/-*

ENSDARG00000071103	si:dkey-222p3.1	-3.300063979	1.83E-08	-3.501964874	3.06E-11
ENSDARG00000071143	si:dkey-208m12.2	1.366134483	0.00000312	1.294180321	0.00000283
ENSDARG00000071173	slc12a10.2	1.208326056	0.00000137	0.718495026	0.005151064
ENSDARG00000071212	p3h1	0.371908098	0.024656387	0.373316538	0.021708151
ENSDARG00000071213	rgl3a	0.877343693	0.000000395	0.633779517	0.000188489
ENSDARG00000071251	ppp1r18	-2.380357803	2.47E-27	-0.826853099	0.0000442
ENSDARG00000071304	tbc1d2	1.836156337	5E-12	0.669076484	0.024211365
ENSDARG00000071384	slc7a11	-2.381539245	1.09E-17	-1.760764006	2.07E-13
ENSDARG00000071410	cks2	-1.642363151	7.17E-09	-0.897623754	0.000796304
ENSDARG00000071437	ptprc	-0.928285822	0.005682601	-1.319898594	0.0000154
ENSDARG00000071454	crp1	-2.214783968	0.000000549	-1.314653985	0.000927514
ENSDARG00000071463	zgc:154125	-3.321526285	0.000406103	-1.936598548	0.016481138
ENSDARG00000071475	aox5	0.789870832	0.000000363	0.667806533	0.00000569
ENSDARG00000071549	ecm2	0.905081921	1.85E-13	0.508025925	0.0000336
ENSDARG00000071554	CU929149.1	4.213987597	0.001895467	3.793683603	0.007929346
ENSDARG00000071558	fblim1	1.35358042	7.71E-15	0.847185064	0.000000602
ENSDARG00000071573	lsm5	0.998164422	0.000301796	0.784719922	0.004735078
ENSDARG00000071578	si:ch211-222k6.3	-0.445138381	0.035381774	-0.578615187	0.003392486
ENSDARG00000071579	urad	-1.164251535	0.001930681	-1.028938451	0.004508388
ENSDARG00000071590	si:ch211-236g6.1	-1.867364018	7.38E-09	-2.146044061	1.65E-13
ENSDARG00000071618	si:ch211-213a13.5	2.742539151	0.021768199	2.917320778	0.015588867
ENSDARG00000071643	zgc:171490	1.096609556	0.004094516	0.826784156	0.036117531
ENSDARG00000071644	si:dkey-19a16.7	0.647306803	0.00113311	0.69782092	0.000178139
ENSDARG00000071648	zgc:113298	1.341873821	0.00000262	0.684882661	0.029941083
ENSDARG00000071662	si:rp71-36a1.3	-1.48392153	0.021703236	-1.684334752	0.006252003
ENSDARG00000071735	prlh2	1.09758456	0.004094516	1.078280437	0.003763972
ENSDARG00000071860	nrn1a	-0.752564189	3.24E-10	-0.332523256	0.00681127
ENSDARG00000071877	dhrs7cb	1.142175644	6.81E-14	0.382220318	0.023438204
ENSDARG00000071881	arl9	-0.497830094	0.006846676	-0.390825198	0.036778212
ENSDARG00000073711	mmrn2b	1.998276791	2.65E-45	1.027713659	7.88E-14
ENSDARG00000073720	RNF180	-1.967240737	3.1E-09	-2.351551305	4.21E-15
ENSDARG00000073742	prss59.2	-1.741799634	0.012024258	-1.680397876	0.013440525
ENSDARG00000073799	zgc:194210	-1.808267664	0.002385279	-1.52465875	0.007136859
ENSDARG00000073814	pax1b	-0.675184556	0.013606456	-0.630485798	0.018616493
ENSDARG00000073820	zgc:174917	2.130845903	2.32E-08	1.268939249	0.00132662
ENSDARG00000073821	znf1177	8.954797751	7.46E-12	8.529706103	2.1E-10
ENSDARG00000073841	herc2	0.385985577	0.009677169	0.313431666	0.039172477
ENSDARG00000073845	zgc:110843	1.537923424	7.66E-25	0.943313156	3.5E-11
ENSDARG00000073905	vwa5a	0.909508883	9.04E-11	0.361108279	0.017599224
ENSDARG00000073926	synrg	0.38806604	0.002492046	0.409963947	0.000717979
ENSDARG00000073961	si:ch211-160j14.2	-1.237578836	0.000536155	-1.282751439	0.000117134
ENSDARG00000073998	si:ch211-23l10.3	-0.801328045	0.001967137	-0.887540184	0.000223495
ENSDARG00000074002	slc6a11a	0.94187859	9.47E-09	0.609863541	0.000194329
ENSDARG00000074036	ky	1.978449282	0.00000127	1.548801902	0.000115399
ENSDARG00000074050	efnb2b	-0.65558937	0.0000798	-0.392313781	0.021451344
ENSDARG00000074069	zgc:171452	1.836343766	0.022015827	1.660634679	0.042677032
ENSDARG00000074085	si:ch1073-188e1.1	3.342783046	7.49E-10	2.208220354	0.000069
ENSDARG00000074094	tgm2b	1.890538148	1.99E-31	0.790865169	0.000000787
ENSDARG00000074100	zgc:163014	-2.8624298	0.001125135	-2.342764182	0.002039499
ENSDARG00000074137	c2cd3	-0.754886782	0.000000499	-0.33100519	0.036976336
ENSDARG00000074160	paqr4b	-2.326184671	0.00000248	-1.085208189	0.027097523
ENSDARG00000074170	fbxo4	0.974310147	0.0000268	0.529014008	0.035642486
ENSDARG00000074201	flna	1.57564367	3.7E-25	0.791432036	0.000000076
ENSDARG00000074210	zgc:110286	4.218867296	1.01E-12	3.03453333	0.000000579
ENSDARG00000074221	ABCA7	-1.329576116	2.05E-17	-0.841909235	9.02E-09

ENSDARG00000074225	si:ch211-269e2.1	-0.43668848	0.001797172	-0.31588935	0.026733126
ENSDARG00000074285	si:ch73-335m24.2	-2.152669559	3.44E-14	-1.202720827	0.00000326
ENSDARG00000074287	sptlc2b	0.603552623	0.000039	0.429867307	0.003522084
ENSDARG00000074298	znf1015	-0.308995216	0.031849124	-0.322274134	0.021371298
ENSDARG00000074331	eif4g3b	0.311900988	0.036809877	0.325484653	0.02621893
ENSDARG00000074340	serf2	0.331694802	0.00642993	0.268284449	0.029984471
ENSDARG00000074390	tmem176l.4	1.698307378	2.46E-35	0.722724301	7.51E-08
ENSDARG00000074471	vps39	0.435257764	0.000226164	0.423086259	0.000154875
ENSDARG00000074508	si:dkey-28e7.3	-0.855707274	8.79E-09	-0.486810109	0.000963798
ENSDARG00000074546	si:ch211-213a13.2	1.325967552	8.09E-08	1.149736961	0.000000919
ENSDARG00000074613	si:ch211-240l19.6	-2.788891551	2.55E-09	-2.705542405	1.64E-10
ENSDARG00000074626	pidd1	0.757513218	0.000620886	0.500261174	0.031468843
ENSDARG00000074653	si:ch211-233m11.1	7.2224283	0.00000219	6.10756067	0.00015811
ENSDARG00000074656	ctss2.1	1.664692457	1.45E-16	0.585202067	0.00919238
ENSDARG00000074663	zgc:162183	0.752274509	0.0000559	0.474011089	0.015377496
ENSDARG00000074712	si:ch211-186e20.2	-3.733041572	0.001095974	-2.890401047	0.003651706
ENSDARG00000074749	abca12	1.021671012	9.35E-20	0.596032099	3.27E-08
ENSDARG00000074758	csde1	0.590706555	9.82E-08	0.331441274	0.003481332
ENSDARG00000074764	zgc:171426	-1.741804436	0.036362729	-3.122607836	0.0000747
ENSDARG00000074768	zgc:113295	-0.554663818	0.007549636	-0.47691418	0.020471749
ENSDARG00000074779	alms1	-0.568461054	0.00000516	-0.316298414	0.013745649
ENSDARG00000074790	rab5if	0.463036874	0.000271029	0.435588921	0.000331358
ENSDARG00000074808	megf6b	0.718604022	0.00000465	0.418810759	0.010083717
ENSDARG00000074828	rhobtb2a	1.074858023	0.001344246	0.744654015	0.033408945
ENSDARG00000074892	pde6d	-1.684740414	1.99E-16	-0.551215539	0.007264458
ENSDARG00000074919	BFSP1	-3.468491267	5.31E-29	-3.287790995	5.04E-33
ENSDARG00000074967	zgc:162698	0.361695899	0.002907652	0.273849829	0.027445956
ENSDARG00000074979	larp4ab	0.521422766	0.000102089	0.58238093	0.00000235
ENSDARG00000074983	jac9	6.922893311	6.2E-09	4.044784265	0.000989494
ENSDARG00000074985	aurkaip1	0.50858567	0.0000846	0.274655982	0.048789596
ENSDARG00000074989	sparcl1	-1.003020756	5.22E-09	-0.806952266	0.000000493
ENSDARG00000074996	tbrg1	-0.580077705	0.000171345	-0.39051451	0.011669606
ENSDARG00000075014	sqstm1	0.643147382	0.0000209	0.534254244	0.000276092
ENSDARG00000075027	fgfr1bl	2.901020427	1.75E-34	0.988966696	0.0000859
ENSDARG00000075043	si:dkeyp-123h10.2	-0.866995489	0.000000213	-0.48958642	0.003643811
ENSDARG00000075046	rnf151	-2.819677687	0.0000162	-2.931163945	0.000000836
ENSDARG00000075067	aipl1	-2.041486001	5.82E-11	-0.802309735	0.008190535
ENSDARG00000075143	nanp	0.509020302	0.04367701	0.558000791	0.022793575
ENSDARG00000075161	defbl1	-1.181791037	1.17E-10	-0.448688631	0.022908556
ENSDARG00000075192	yme1l1a	0.692101726	3.15E-08	0.540000283	0.00000634
ENSDARG00000075245	wdr11	0.282593099	0.025213611	0.389167131	0.000734402
ENSDARG00000075249	fam171a2a	0.398182254	0.000197577	0.235559047	0.037808674
ENSDARG00000075263	ankrd1a	-0.578774548	0.040348297	-0.60842576	0.026705544
ENSDARG00000075295	tulp1a	-3.657143258	1.21E-15	-1.362396329	0.003839853
ENSDARG00000075323	srfbp1	-0.310528129	0.028970624	-0.293614721	0.037539682
ENSDARG00000075339	ipp	0.563009432	0.00320232	0.510361309	0.00659031
ENSDARG00000075444	cgref1	0.639337715	0.004973421	0.485362425	0.03851023
ENSDARG00000075446	naa25	-0.21434294	0.04414367	-0.282951434	0.004339196
ENSDARG00000075461	kmt5c	-0.393317048	0.000924912	-0.266805675	0.029305582
ENSDARG00000075487	si:ch211-267e7.3	0.65774791	0.000395404	0.75334175	0.0000104
ENSDARG00000075492	ccdc160	-0.551106768	0.03964672	-0.603335432	0.019592434
ENSDARG00000075500	smim1	1.957752634	0.000000361	0.95283786	0.028065776
ENSDARG00000075513	ccdc136b	-1.006522248	0.000623803	-0.619956261	0.043371852
ENSDARG00000075539	snphb	-0.699884658	0.000765506	-0.569814716	0.005382848
ENSDARG00000075540	FAM107A	-1.907921875	1.87E-09	-0.933154238	0.001707013

Table S3. DEGs of WT vs. *terfa+/-* ∩ *terfa-/-*

ENSDARG00000075551	aifm5	-1.619558494	0.00000541	-1.500463192	0.00000473
ENSDARG00000075584	itfg1	0.310100254	0.014117613	0.354589963	0.002991171
ENSDARG00000075600	si:dkeyp-41f9.3	-1.089912543	0.000105593	-1.714675452	5.89E-12
ENSDARG00000075618	slc36a1	0.517303444	0.014711295	0.502766804	0.015796826
ENSDARG00000075619	cenpo	-1.10462914	0.0000439	-0.929541196	0.000248597
ENSDARG00000075621	birc5a	-1.319567566	3.49E-10	-0.850276387	0.0000169
ENSDARG00000075650	vps26c	0.629003661	0.00000365	0.554609115	0.0000162
ENSDARG00000075677	mrpl17	-0.494828448	0.010935629	-0.405003536	0.039407635
ENSDARG00000075707	nid2a	0.757679772	1.7E-09	0.528096579	0.0000136
ENSDARG00000075718	rpz5	1.507807637	5.27E-25	0.539847179	0.000360264
ENSDARG00000075727	map1lc3cl	-0.656862522	0.034975979	-1.010484604	0.000346347
ENSDARG00000075730	dnph1	-0.755900781	0.011426321	-0.772622593	0.007182869
ENSDARG00000075768	sdhb	0.641799179	0.0000159	0.409495899	0.006945248
ENSDARG00000075833	lyve1a	1.622965006	7.81E-12	0.576259888	0.031570911
ENSDARG00000075842	pigt	-1.059979184	0.0000188	-0.597850443	0.01605088
ENSDARG00000075846	kcnip2	-2.26258648	0.0000127	-0.950086699	0.0479796
ENSDARG00000075864	igsf9a	-0.505987017	0.0000539	-0.35430349	0.004568778
ENSDARG00000075881	si:ch211-39k3.2	1.786057797	9.5E-10	1.239976591	0.000016
ENSDARG00000075887	herc3	0.323439843	0.026108318	0.340078892	0.016012119
ENSDARG00000075892	bag6	0.334211257	0.000249118	0.196648811	0.042304188
ENSDARG00000075917	cacna1db	1.395603555	1.63E-09	0.57514274	0.026427219
ENSDARG00000075948	slco4a1	-1.811955072	1.61E-15	-0.481286799	0.041776306
ENSDARG00000075954	serpinh1a	0.797344002	0.000000204	0.728817505	0.00000037
ENSDARG00000076005	piezo2a.2	0.87305563	6.43E-09	0.346224251	0.036132421
ENSDARG00000076043	si:dkeyp-73d8.9	5.591295454	2.95E-12	5.799277888	6.62E-14
ENSDARG00000076106	ftr43	3.44479304	1.55E-09	2.728747488	0.00000183
ENSDARG00000076121	smpd1	0.744111086	0.0000669	0.451863251	0.020707796
ENSDARG00000076135	mmrn2a	-0.421780897	0.000145754	-0.249303438	0.031025492
ENSDARG00000076146	zgc:172075	2.080506715	1.92E-16	1.537522758	2.4E-10
ENSDARG00000076176	ptcd1	-0.358955597	0.017131566	-0.336777328	0.023206722
ENSDARG00000076218	b3galt4	-1.120248158	0.000348978	-0.871920886	0.003945009
ENSDARG00000076221	fthl28	1.876337669	2.42E-20	1.327855224	3.58E-12
ENSDARG00000076225	tha1	0.993209455	4.39E-10	0.360983423	0.046038493
ENSDARG00000076229	mrtfab	0.535556127	0.000109624	0.323220342	0.026090233
ENSDARG00000076255	znf1084	1.451267484	0.002610448	1.02655878	0.044974437
ENSDARG00000076257	si:ch211-285c6.1	7.842631439	4.46E-09	7.343830542	0.00000012
ENSDARG00000076264	zgc:195170	-2.746897524	0.000000222	-3.32344029	9.98E-12
ENSDARG00000076313	NA	-8.044704169	0.00000307	-5.614915208	0.000000783
ENSDARG00000076317	plod3	0.709954324	9.65E-11	0.530123013	0.000000357
ENSDARG00000076320	ano9a	0.867217331	0.00000068	0.420101157	0.028074973
ENSDARG00000076321	col28a2a	2.719490266	1.04E-31	2.501035265	7.22E-33
ENSDARG00000076357	CABZ01102039.1	-0.411618072	0.006597527	-0.580208578	0.0000215
ENSDARG00000076371	SHROOM2	-1.269339448	2.18E-10	-0.451466259	0.03407853
ENSDARG00000076386	epdl1	0.687293902	0.0000935	0.794814724	0.00000078
ENSDARG00000076490	ankrd40	0.385563803	0.000834756	0.242714139	0.046964704
ENSDARG00000076554	cdkn1a	3.535902774	5.54E-70	2.085119699	4.89E-29
ENSDARG00000076557	bicc2	1.667744355	2.67E-15	0.503571094	0.038883274
ENSDARG00000076559	rb1cc1	-0.210425229	0.047467219	-0.289586049	0.003174233
ENSDARG00000076585	si:ch211-113e8.11	-0.994296103	1.05E-09	-0.353864522	0.045095283
ENSDARG00000076592	man1b1b	0.412079083	0.01704334	0.386459821	0.024285447
ENSDARG00000076611	fbxo21	-0.640865055	0.00000932	-0.509953351	0.000211956
ENSDARG00000076623	col14a1b	0.516917755	0.002130988	0.420393798	0.012459306
ENSDARG00000076667	ccng1	1.952177183	9.09E-79	0.743244265	5.61E-14
ENSDARG00000076710	si:dkey-42l23.7	-2.012826407	0.000000134	-1.560186133	0.00000887
ENSDARG00000076789	cx32.2	1.707473161	2.67E-08	0.979749445	0.002621019

Table S3. DEGs of WT vs. *terfa+/-* ∩ *terfa-/-*

ENSDARG00000076811	ganab	0.334020606	0.006375933	0.278611003	0.023931625
ENSDARG00000076830	si:dkey-65b12.6	0.978009259	2.61E-11	0.8360459	7.86E-10
ENSDARG00000076831	iqck	0.944192616	0.00016102	0.684627975	0.008021885
ENSDARG00000076839	ftr86	0.764427356	0.0000805	1.158854448	8.08E-12
ENSDARG00000076848	zgc:112492	-0.769461753	0.019714337	-0.790140999	0.0130515
ENSDARG00000076856	frem2a	0.562052878	0.00120079	0.425821041	0.015376802
ENSDARG00000076888	GANAB	0.715041463	3.04E-08	0.51887666	0.0000328
ENSDARG00000076945	dsg2.1	1.23744755	2.15E-20	0.76386847	1.88E-09
ENSDARG00000076958	jac8	5.655468517	0.000000362	2.719888645	0.035066519
ENSDARG00000076970	si:ch211-165b10.3	-0.988589483	0.016558459	-1.042603609	0.008060053
ENSDARG00000077011	uhrf1bp1	0.589407934	0.01005078	0.58407251	0.008819215
ENSDARG00000077013	znf280d	-0.734339592	3.73E-10	-0.320693212	0.008461179
ENSDARG00000077022	fam131a	-0.875150093	0.0000174	-0.542712357	0.007165865
ENSDARG00000077047	ptprnb	0.757077362	0.000124644	0.564776899	0.004343942
ENSDARG00000077057	esco1	-0.38538217	0.001502581	-0.327366829	0.005845673
ENSDARG00000077068	si:ch211-11p18.6	4.911098185	0.00000183	4.701655754	0.00000284
ENSDARG00000077072	si:ch73-280o22.2	0.545554731	0.006687982	0.953542087	3.21E-08
ENSDARG00000077081	slc38a10	0.515136808	0.000000198	0.224649639	0.037973549
ENSDARG00000077084	col28a1a	-0.417149948	0.001068619	-0.507672774	0.0000132
ENSDARG00000077095	si:ch211-160o17.4	0.498082971	0.00000634	0.571201659	1.15E-08
ENSDARG00000077114	arhgef16	0.744898462	0.0000172	0.460369083	0.010086434
ENSDARG00000077115	si:ch73-44m9.1	2.510364545	0.0000421	1.76685777	0.00632057
ENSDARG00000077167	nmbb	-1.944372935	0.0000534	-1.403552835	0.001526059
ENSDARG00000077169	si:ch211-153b23.4	0.83996868	3.06E-10	0.795381361	8.74E-11
ENSDARG00000077215	eif4g3a	0.331361852	0.032698112	0.35431127	0.018884973
ENSDARG00000077224	ypel2a	-0.764018233	0.0000828	-0.411986934	0.038741531
ENSDARG00000077236	hspb6	1.748540307	7.46E-12	0.933421708	0.000287941
ENSDARG00000077257	timd4	-1.392682868	0.000067	-0.9155268	0.006708311
ENSDARG00000077306	cutc	1.123334708	5.99E-14	0.615816083	0.0000412
ENSDARG00000077318	CR376737.1	-0.514736169	0.011315797	-0.505577344	0.010441367
ENSDARG00000077360	fthl30	1.065457015	0.009231532	0.903828406	0.029343173
ENSDARG00000077367	ntng2a	-1.18970992	2.33E-13	-0.413761775	0.014689796
ENSDARG00000077387	tcte1	2.039590267	0.000462496	1.758199066	0.002616387
ENSDARG00000077400	rbm10	-0.572751225	0.003109954	-0.603774195	0.000981134
ENSDARG00000077403	col8a1a	-0.905224604	0.001751238	-0.746470877	0.009322671
ENSDARG00000077404	ncoa3	0.321793055	0.018857927	0.437492156	0.000472213
ENSDARG00000077414	zgc:194312	-0.6529568	0.002332683	-0.795591785	0.0000527
ENSDARG00000077415	zmp:0000001175	1.811032206	0.028926358	2.212185047	0.004774884
ENSDARG00000077487	spsb3b	-2.235405486	0.000334545	-1.441156628	0.014359082
ENSDARG00000077533	eif3f	0.330710131	0.018128851	0.30990617	0.025819076
ENSDARG00000077569	lrrn3a	-0.758676974	0.000000614	-0.422969705	0.005719712
ENSDARG00000077572	si:ch211-193k19.2	5.642866142	0.00000548	5.825358202	0.00000471
ENSDARG00000077590	arsj	-0.614432931	0.007204944	-0.984310597	0.00000123
ENSDARG00000077717	rpl29	0.620560176	3.37E-08	0.407207333	0.000234583
ENSDARG00000077726	nocta	0.549464691	0.001506338	0.39961332	0.024569148
ENSDARG00000077737	spsb3a	-0.573514474	0.00000724	-0.426056088	0.000608127
ENSDARG00000077760	ascc1	2.207228463	7.36E-14	1.555948293	4.02E-08
ENSDARG00000077762	si:rp71-79p20.2	0.884656682	0.000187501	0.609478869	0.012928297
ENSDARG00000077764	faah	0.930011572	0.000000569	0.536118258	0.005862615
ENSDARG00000077777	tmsb4x	0.706069028	0.0000142	0.376155698	0.029383306
ENSDARG00000077788	arhgef10	0.758696979	0.000000534	0.358612565	0.028466606
ENSDARG00000077794	CU468012.1	2.716272757	0.00020493	2.063360589	0.006561847
ENSDARG00000077799	egr4	-1.558012577	1.24E-10	-1.117095297	0.000000815
ENSDARG00000077860	ankhd1	0.43072972	0.00340445	0.414808178	0.003603855
ENSDARG00000077872	CR626907.1	-0.686921526	0.000797669	-0.582769332	0.003723543

Table S3. DEGs of WT vs. *terfa+/-* ∩ *terfa-/-*

ENSDARG00000077875	slc2a5	1.316752744	0.042948103	2.217800224	0.0000913
ENSDARG00000077880	si:ch211-255i20.3	-1.231316382	0.01005078	-1.106632426	0.018200062
ENSDARG00000077882	ccn5	1.914643416	8.59E-09	0.91524527	0.01303186
ENSDARG00000077883	fam83d	-1.011132649	0.0000653	-0.724865	0.003774117
ENSDARG00000077905	csmd3a	0.753203817	0.006158157	0.907592543	0.000368982
ENSDARG00000077926	si:dkey-48p11.3	-0.730289034	0.005238657	-0.539329313	0.041193026
ENSDARG00000077960	si:ch211-186e20.7	-1.519062933	0.00078425	-1.43316212	0.000846939
ENSDARG00000078001	kbtbd7	-1.099435792	0.000000578	-0.572963618	0.008937898
ENSDARG00000078014	pacsin2	0.92145679	1.75E-17	0.390871567	0.000445261
ENSDARG00000078022	fyco1b	0.639532636	0.000102743	0.743731692	0.000000767
ENSDARG00000078041	xpo1a	0.404320721	0.001118855	0.47876463	0.0000264
ENSDARG00000078069	rrm2	-1.605812453	3.46E-12	-0.913944499	0.0000523
ENSDARG00000078073	nudt5	-0.535244837	0.002473268	-0.360040053	0.049484993
ENSDARG00000078092	limd2	0.909772616	9.48E-10	0.520240183	0.000517644
ENSDARG00000078095	cipcb	0.578931134	0.00000421	0.727620098	1.01E-10
ENSDARG00000078102	psda	-1.189525445	2.84E-17	-0.603023798	0.00000558
ENSDARG00000078105	depdc5	0.49624119	0.0000232	0.331643617	0.005339684
ENSDARG00000078113	atp5mea	-0.395971418	0.019795217	-0.365959701	0.030276591
ENSDARG00000078114	si:ch73-237c6.1	3.517817522	3.17E-60	2.977967215	1.34E-51
ENSDARG00000078134	crygm2f	2.308235146	0.004808731	2.599940966	0.001009344
ENSDARG00000078179	fndc3ba	0.468098199	0.001577224	0.323735746	0.035215542
ENSDARG00000078210	tulp1b	-3.116157385	1.78E-36	-1.075235257	0.000000428
ENSDARG00000078233	ctnnd1	0.356860109	0.0000328	0.277108018	0.001012141
ENSDARG00000078244	si:ch211-197l9.2	-1.298629016	6.67E-15	-0.610960352	0.000155815
ENSDARG00000078258	CABZ01049847.1	-0.879664655	0.00014563	-0.772812583	0.000472213
ENSDARG00000078261	nphp3	0.789534737	0.00000482	0.663674911	0.0000615
ENSDARG00000078322	col12a1a	1.340196281	8.66E-31	0.669821245	1.76E-09
ENSDARG00000078324	si:ch211-263k4.2	0.593335555	0.002573289	0.536735763	0.005400344
ENSDARG00000078419	filip1a	-1.053278286	0.000486622	-1.432636138	0.000000166
ENSDARG00000078468	fap	-0.430150149	0.0000991	-0.337292078	0.001865741
ENSDARG00000078508	si:dkey-266m15.6	0.779698664	0.00000499	0.484849139	0.005484144
ENSDARG00000078527	lingo4b	-0.639283549	0.0000616	-0.435556374	0.006066106
ENSDARG00000078551	zgc:171242	9.248854495	1.15E-11	8.842136673	1.85E-10
ENSDARG00000078585	mon1a	0.66183993	0.000918184	0.464102046	0.024942418
ENSDARG00000078592	nomo	-0.353145092	0.000774522	-0.244900536	0.023070105
ENSDARG00000078593	vwa8	0.591039369	0.00000524	0.37647526	0.004387516
ENSDARG00000078619	pnp5a	1.356809066	2.92E-48	1.160109282	3.33E-43
ENSDARG00000078671	cdk5r2b	-1.356724824	3.87E-12	-0.442675887	0.033469988
ENSDARG00000078683	RNF14	3.340990673	2.05E-74	1.545285335	4.95E-19
ENSDARG00000078694	phlpp1	-0.461028972	0.004232638	-0.37904609	0.01914779
ENSDARG00000078738	si:dkey-145c18.3	5.774265099	0.00000185	2.976762961	0.040542097
ENSDARG00000078785	tmem258	1.336178897	0.0000167	1.214261092	0.0000415
ENSDARG00000078797	dennd3a	0.815947558	3.91E-11	0.385441571	0.002813746
ENSDARG00000078805	ap5b1	-0.653417394	0.005158546	-0.478945037	0.044974437
ENSDARG00000078822	ccdc18	-0.526455097	0.006926389	-0.486679037	0.010411313
ENSDARG00000078882	slc22a31	1.445576806	1.99E-23	0.903629551	5.22E-11
ENSDARG00000078912	si:ch73-24k9.2	1.911789544	0.00000103	1.528818244	0.000067
ENSDARG00000078917	zgc:195245	-4.007992247	3.35E-11	-1.083491039	0.030764822
ENSDARG00000078953	epn3a	0.467254782	0.000196807	0.332322428	0.009136353
ENSDARG00000078959	tpcn3	0.452827638	0.003872586	0.350456061	0.029179105
ENSDARG00000078979	arhgef40	1.211077064	0.00000091	0.644758123	0.013130062
ENSDARG00000079011	col17a1b	0.264971869	0.041806129	0.333070811	0.006446642
ENSDARG00000079013	dpy19l3	-0.819768615	2.93E-08	-0.331917493	0.038368782
ENSDARG00000079049	cercam	0.669872075	7.82E-09	0.632917298	3.43E-09
ENSDARG00000079056	si:ch211-194c3.5	-0.760214515	9.82E-08	-0.563541772	0.0000393

Table S3. DEGs of WT vs. *terfa+/-* ∩ *terfa-/-*

ENSDARG00000079104	mfhas1	-0.449607398	0.000753611	-0.382600057	0.003278438
ENSDARG00000079105	mhc2dab	5.007072534	0.002525915	6.197941425	0.000115327
ENSDARG00000079119	si:ch211-229d2.5	-1.40568363	1.25E-18	-0.56538575	0.000425706
ENSDARG00000079163	best2	4.163235012	4.35E-09	2.684198871	0.000341524
ENSDARG00000079227	plekhs1	-1.687361788	0.000140225	-2.199287088	6.06E-08
ENSDARG00000079234	si:ch211-285c6.2	3.523878102	0.003859898	2.998465237	0.020333337
ENSDARG00000079238	trim59	-0.561738505	0.026122441	-0.62682939	0.009388207
ENSDARG00000079271	dcxr	1.075606568	3.45E-11	0.913210295	1.67E-09
ENSDARG00000079274	prss59.1	-3.301938589	0.00041074	-2.361157071	0.012698168
ENSDARG00000079305	hbae3	0.474539751	0.015862754	0.447879062	0.021494571
ENSDARG00000079327	hmcn2	1.081196333	5.76E-19	0.536321198	0.00000775
ENSDARG00000079350	znf654	-0.612331249	3.04E-08	-0.46074392	0.0000112
ENSDARG00000079355	flrt2	0.398360037	0.008841334	0.373856279	0.012539421
ENSDARG00000079388	agrn	0.472173329	0.0000483	0.430129826	0.000098
ENSDARG00000079397	cerkl	-1.629006644	1.32E-09	-0.807342271	0.00132471
ENSDARG00000079403	si:dkey-204l11.1	5.330030533	4.28E-50	3.707554377	5.75E-27
ENSDARG00000079440	coro2ba	-0.450397599	0.007036424	-0.371739766	0.027146441
ENSDARG00000079467	si:ch73-15b2.5	1.636702056	1.3E-12	0.916839591	0.000084
ENSDARG00000079468	rhobtb1	1.008695901	0.000393711	0.945456106	0.000556284
ENSDARG00000079504	mfn2	0.299930731	0.004313166	0.426243755	0.00000564
ENSDARG00000079525	slc39a5	0.667818586	0.004499309	0.943300287	0.00000724
ENSDARG00000079530	si:dkey-17m8.1	0.911173928	0.0000149	0.534924263	0.014866044
ENSDARG00000079571	mfsd4b	-0.609857747	0.001930681	-0.702982468	0.000108553
ENSDARG00000079572	plcd3b	-0.654860743	0.000119103	-0.42425671	0.013463255
ENSDARG00000079589	si:dkeyp-73d8.6	3.806160696	0.016228561	5.030888566	0.000874077
ENSDARG00000079591	si:ch211-203d1.3	1.101414121	8.76E-10	0.624905904	0.000606055
ENSDARG00000079645	sc:d217	2.312662528	1.52E-09	1.88911608	0.000000215
ENSDARG00000079651	si:ch1073-174d20.2	-0.353445296	0.000686358	-0.305098701	0.002638761
ENSDARG00000079672	efl1	1.029480809	2.09E-08	0.530319954	0.006443787
ENSDARG00000079730	fuz	0.609269823	0.022230078	0.536850668	0.047632342
ENSDARG00000079745	si:ch211-166a6.5	1.440798551	3.91E-48	0.834477588	1.03E-19
ENSDARG00000079777	map6d1	-1.238041177	0.00000459	-0.597336442	0.032122339
ENSDARG00000079781	slitrk4	-0.678514408	0.00000645	-0.550485373	0.000121454
ENSDARG00000079783	isg20	1.415313863	6.27E-37	0.445038038	0.000158137
ENSDARG00000079784	si:ch211-235o23.1	0.500839705	0.001164645	0.4200959	0.005992536
ENSDARG00000079795	CABZ01033206.1	1.021071462	0.000000484	0.570678731	0.007298926
ENSDARG00000079802	aanat2	-1.824257176	3.46E-12	-1.067159877	0.00000523
ENSDARG00000079848	gmps	0.324415447	0.031988273	0.467222768	0.000671042
ENSDARG00000079854	scyl1	0.340287331	0.001123915	0.262981128	0.012414812
ENSDARG00000079884	trim107	-0.969366654	0.000119859	-0.620374154	0.01299448
ENSDARG00000079897	si:dkey-7k24.5	-1.497897853	0.001549032	-2.311643797	0.000000267
ENSDARG00000079946	sqlea	1.462968199	0.000000979	2.067618517	3.4E-15
ENSDARG00000080010	adh5	0.473201741	0.0000573	0.39596185	0.000507887
ENSDARG00000081252	dre-mir-16b	4.657063493	0.003994741	5.537526938	0.000624514
ENSDARG00000083894	CR925757.1	4.16733271	0.038311884	4.666464811	0.022924582
ENSDARG00000086048	si:ch211-229i14.2	3.121165903	0.021586144	3.303374899	0.018052315
ENSDARG00000086103	slc37a1	0.820013519	0.000000635	0.587271089	0.000300987
ENSDARG00000086126	trim33l	0.660602376	0.023979571	1.361273674	3.54E-08
ENSDARG00000086184	FBXO48	-0.68340724	0.00000307	-0.513339482	0.000258157
ENSDARG00000086272	si:dkey-4p15.5	-2.278282911	2.17E-38	-0.555847788	0.00163839
ENSDARG00000086288	scg3	-1.198890583	2.05E-25	-0.452238772	0.0000847
ENSDARG00000086305	si:ch211-63b16.4	-1.086226339	0.00221781	-1.473572979	0.00000556
ENSDARG00000086309	erlec1	0.50669506	0.000052	0.358287163	0.004508388
ENSDARG00000086336	si:ch211-157b11.12	-0.613234381	0.003012709	-0.451817929	0.030133367
ENSDARG00000086374	isg15	4.633037169	1.74E-28	2.333403639	5.68E-08

ENSDARG00000086421	si:ch211-212k18.8	0.681891214	0.00263134	0.854188651	0.0000364
ENSDARG00000086425	prpf3	-0.532230843	0.00000291	-0.410264214	0.000175581
ENSDARG00000086445	adgrf7	4.735203647	0.0000856	3.699851775	0.004774884
ENSDARG00000086449	znf1055	2.021086891	0.001491351	1.384169832	0.046512995
ENSDARG00000086452	dthd1	-1.031607997	0.041622668	-1.594230775	0.000720396
ENSDARG00000086458	hdac10	-1.202373658	8.87E-11	-0.783887399	0.0000072
ENSDARG00000086615	CR846087.1	2.219170534	0.000000215	1.409245288	0.001930134
ENSDARG00000086618	psma3	-0.299224755	0.033431314	-0.361736342	0.006536493
ENSDARG00000086626	im:7147486	-0.653619567	0.00000108	-0.342430632	0.013636525
ENSDARG00000086645	hs3st3b1b	-0.604205373	8.09E-10	-0.233993427	0.026600914
ENSDARG00000086647	chrng	1.588757052	7.05E-15	0.530748312	0.020762042
ENSDARG00000086654	cbln11	2.64268192	0.000000136	1.720999405	0.000863122
ENSDARG00000086665	si:dkey-175g6.2	0.732923849	0.0000836	0.652445311	0.000272157
ENSDARG00000086678	ascc3	1.172720689	1.39E-20	0.818394519	4.45E-12
ENSDARG00000086699	smim15	-0.395881092	0.005983816	-0.397454372	0.004117518
ENSDARG00000086735	SAMD4B	-0.613757314	0.000000868	-0.288679725	0.030206077
ENSDARG00000086744	znf1152	1.051296155	0.00111168	0.695639511	0.044590385
ENSDARG00000086746	prodha	1.288059038	2.57E-16	0.548000769	0.00075737
ENSDARG00000086826	sult6b1	0.983058109	4.5E-12	0.653645247	0.00000187
ENSDARG00000086848	atad3	-0.525638781	0.002826533	-0.543072926	0.00115091
ENSDARG00000086856	stk35	0.743885679	0.0000186	0.631714021	0.000156633
ENSDARG00000086881	ier2b	-0.93094143	0.000000105	-0.475648957	0.007990567
ENSDARG00000086906	stambpb	-0.556627874	0.001609504	-0.400481468	0.025620185
ENSDARG00000087018	FO704748.1	0.603277228	0.022681441	0.557404097	0.035917154
ENSDARG00000087059	selenou1b	1.570928222	0.000109217	0.923955296	0.039544956
ENSDARG00000087061	si:ch211-71k14.1	-5.0824693	0.007604189	-3.11462279	0.010813776
ENSDARG00000087070	znf977	-1.632839465	2.02E-08	-1.24195507	0.00000337
ENSDARG00000087087	col28a1b	-1.598800277	0.0000811	-1.691387035	0.00000559
ENSDARG00000087093	si:ch211-157c3.4	0.710125946	0.00263134	0.610920043	0.009065926
ENSDARG00000087188	nfil3-6	1.042104923	0.000000288	1.269147779	2.91E-12
ENSDARG00000087349	reep6	-2.307836493	1.54E-16	-0.669564096	0.00919238
ENSDARG00000087359	c3a.2	3.596282377	1.09E-08	1.735526812	0.00848525
ENSDARG00000087369	cep162	-0.901527861	0.00000128	-0.674579899	0.00013767
ENSDARG00000087390	hbbe1.3	0.88392352	0.012500596	0.865277937	0.012365358
ENSDARG00000087440	ponzr4	1.907897784	4E-14	1.453154428	9.22E-10
ENSDARG00000087443	ptp4a2a	-0.699573178	0.000000034	-0.385297603	0.002721334
ENSDARG00000087476	cbln20	-1.256997127	0.042420196	-1.456758232	0.014074782
ENSDARG00000087492	FO704810.1	1.071437555	8.22E-08	0.672814041	0.000823389
ENSDARG00000087516	CU693494.1	-2.293050357	0.02484748	-2.855921703	0.004196223
ENSDARG00000087544	BX510934.1	2.757405158	0.0000226	2.873816326	0.00000451
ENSDARG00000087574	nox1	0.987146741	0.00000691	0.812225539	0.000139611
ENSDARG00000087625	si:ch211-173a9.6	-1.094421988	0.007064271	-1.825724103	0.000000423
ENSDARG00000087636	hmgn6	-1.145645583	2.65E-15	-0.331121823	0.041284274
ENSDARG00000087659	si:ch211-219a15.3	-1.299261971	0.0000765	-1.601676665	8.59E-08
ENSDARG00000087779	pum3	-0.623165731	0.000000626	-0.676750806	2.51E-09
ENSDARG00000087784	si:dkeyp-110a12.4	0.92467268	1.14E-08	0.36316302	0.045339025
ENSDARG00000087788	si:ch211-22k7.9	2.221804707	0.0000822	1.455587801	0.015628748
ENSDARG00000087798	pdyn	2.536374204	0.000792391	2.295433741	0.002739681
ENSDARG00000087822	agap3	0.703918759	0.000251957	0.448984304	0.025157962
ENSDARG00000087832	bcl3	2.564083975	8.75E-12	1.089971546	0.007805235
ENSDARG00000087835	si:ch211-113p18.3	-0.709940924	0.0000985	-0.797697592	0.00000173
ENSDARG00000087873	eevs	1.353261903	8.72E-19	0.848300012	5.95E-09
ENSDARG00000087911	psme4a	-0.480300634	0.001888563	-0.69847712	0.000000435
ENSDARG00000087993	bada	-0.874843038	8.08E-09	-0.461948075	0.002011528
ENSDARG00000088069	CR759879.1	1.99691936	0.000000133	1.609856411	0.0000113

Table S3. DEGs of WT vs. *terfa+/-* ∩ *terfa-/-*

ENSDARG00000088079	tmed6	-1.204860008	0.001164567	-1.316908766	0.000132814
ENSDARG00000088091	pfn1	1.011499517	9.19E-20	0.591151089	2.71E-08
ENSDARG00000088168	ablim3	-0.541314976	0.00000563	-0.277242122	0.026844343
ENSDARG00000088171	ciartb	0.762954526	0.000107535	0.933989375	0.000000163
ENSDARG00000088225	STRADB	0.292593222	0.035449636	0.282971666	0.041284274
ENSDARG00000088245	si:dkey-16p6.1	-1.601489785	0.0000074	-1.440936388	0.0000146
ENSDARG00000088330	hbae1.3	1.715520154	7.6E-11	1.463456779	2.82E-09
ENSDARG00000088343	prrt4	-1.247132636	7.82E-08	-0.82115326	0.000168315
ENSDARG00000088347	sp1	-0.736004677	0.0000133	-0.724998246	0.00000371
ENSDARG00000088411	notum2	-0.609634979	0.000143467	-0.554902247	0.000260472
ENSDARG00000088440	ssh2a	0.690571977	4.92E-10	0.362344422	0.001446837
ENSDARG00000088463	CLPB	0.672537204	0.002323346	0.649662377	0.002344246
ENSDARG00000088514	and1	1.017711913	0.000000136	0.420015317	0.047736365
ENSDARG00000088549	si:ch1073-390k14.1	0.994438563	1.36E-08	0.776736356	0.00000418
ENSDARG00000088581	f10	0.850519358	0.000275885	1.025884707	0.00000148
ENSDARG00000088589	ponzr3	2.461342034	2.23E-21	2.035326779	1.19E-17
ENSDARG00000088764	lyplal1	1.308828759	0.0000013	0.808833125	0.004081652
ENSDARG00000088805	nes	0.334843617	0.027662581	0.327936199	0.029383306
ENSDARG00000088816	trabd2b	0.593284867	0.000035	0.401067845	0.006142484
ENSDARG00000088820	zgc:174288	0.623066389	0.000700932	0.613512021	0.00045901
ENSDARG00000088861	znf1021	1.303432292	0.011889779	1.304492116	0.010458417
ENSDARG00000088862	stxbp6	0.543362957	0.0000553	0.287099317	0.049038555
ENSDARG00000088882	si:ch211-149b19.2	-2.10049599	5.04E-11	-0.725032697	0.018678719
ENSDARG00000088885	si:ch1073-340i21.3	3.456361417	0.000117287	2.340050891	0.010561044
ENSDARG00000088923	si:ch211-12h2.8	2.811963774	0.011913554	3.081815925	0.005199873
ENSDARG00000089123	negaly6	-1.09799458	0.0000358	-0.738947438	0.004333033
ENSDARG00000089124	hbae1.3	0.66227669	0.001805703	0.586140242	0.004880048
ENSDARG00000089262	pbx3a	-0.493370105	0.008342531	-0.420669214	0.023625371
ENSDARG00000089291	cenpx	0.537431948	0.028338122	0.503249417	0.040722182
ENSDARG00000089304	si:dkeyp-4f2.1	-1.082493614	0.0019827	-1.333280743	0.0000341
ENSDARG00000089340	rhobtb2b	1.630744029	9.29E-15	1.109398231	3.56E-08
ENSDARG00000089372	clk4a	-0.548691011	0.000000121	-0.243364874	0.028493825
ENSDARG00000089429	si:dkey-205h13.2	-1.612758038	1.44E-23	-0.616608813	0.000134975
ENSDARG00000089458	rp1l1a	-2.52680096	3.3E-18	-0.881250127	0.002514053
ENSDARG00000089517	zgc:92594	-1.069849191	0.0000112	-0.950307908	0.0000283
ENSDARG00000089586	ncam3	-1.549338319	5.06E-08	-1.159096391	0.0000096
ENSDARG00000089616	coro7	0.79845298	0.000000288	0.586878171	0.000109996
ENSDARG00000089626	ptges3b	-0.433308945	0.001621568	-0.304311433	0.03143101
ENSDARG00000089645	si:ch1073-406l10.2	0.675913749	0.004644898	0.498086382	0.04514045
ENSDARG00000089681	btf3l4	-0.283076589	0.026883418	-0.290639035	0.019946006
ENSDARG00000089724	cyldb	2.108117558	1.93E-18	1.450979493	1.96E-10
ENSDARG00000089742	fam161a	-2.54123345	1.15E-32	-0.844107284	0.0000215
ENSDARG00000089749	aqp8b	0.909577957	0.000000758	0.567560344	0.002525164
ENSDARG00000089767	rnf130	0.509682253	0.00000431	0.348274427	0.001707013
ENSDARG00000089791	slc25a32a	-0.484675407	0.001439395	-0.416545687	0.00524164
ENSDARG00000089831	si:dkey-207m2.4	-2.051917234	0.00000909	-1.573094718	0.000373794
ENSDARG00000089838	si:dkey-262k9.4	-1.45359714	0.00000157	-0.832124218	0.005151064
ENSDARG00000089844	scarb2c	0.402344871	0.029264641	0.469544308	0.007433719
ENSDARG00000089871	jcada	-0.380910342	0.002407056	-0.327764405	0.007912675
ENSDARG00000089885	slc16a12b	0.661419268	0.00000615	0.531860226	0.000168497
ENSDARG00000089892	cd44b	-1.76714801	0.000907967	-1.199703604	0.019807173
ENSDARG00000089893	tasora	-0.504034375	0.000984358	-0.376029144	0.014899264
ENSDARG00000089901	si:ch211-261n11.8	9.744489907	3.97E-14	9.544460627	3.8E-13
ENSDARG00000089917	sh3tc2	-0.269999711	0.033248942	-0.356093619	0.002477058
ENSDARG00000089936	selenow2b	0.702508122	0.000000796	0.565552321	0.0000344

Table S3. DEGs of WT vs. *terfa+/-* ∩ *terfa-/-*

ENSDARG00000089961	si:cabz01007807.1	1.257717751	1.67E-08	0.773716624	0.000597539
ENSDARG00000090097	CR626886.1	-1.409005345	0.028985045	-1.307859879	0.039263789
ENSDARG00000090116	znf1136	-1.27703579	0.0000591	-0.851559203	0.005891703
ENSDARG00000090119	tlr8a	5.114801853	0.003812819	6.142806959	0.000374714
ENSDARG00000090120	si:dkey-248g21.1	1.922793533	1.27E-11	1.055235578	0.000377225
ENSDARG00000090145	tmem240b	0.60351642	0.025935706	1.031339229	0.0000122
ENSDARG00000090167	FO904898.1	5.15672394	0.004728641	4.561328667	0.020532077
ENSDARG00000090179	si:ch211-117c9.1	-1.11889827	0.026691637	-1.272683242	0.008183527
ENSDARG00000090181	BX072576.2	0.666268958	0.012958701	0.594485631	0.028058671
ENSDARG00000090190	bcam	0.736788064	8.77E-09	0.522623454	0.0000245
ENSDARG00000090194	lamtor5	0.55373255	0.000253082	0.656691705	0.00000194
ENSDARG00000090228	gsta.1	-1.0828146	9.33E-16	-1.001411575	2.76E-16
ENSDARG00000090230	tmem108	-0.486845491	0.003001177	-0.345478018	0.040060383
ENSDARG00000090232	clpb	0.408573164	0.007107605	0.375504104	0.012186229
ENSDARG00000090255	phlpp2	-0.919845189	0.002553049	-0.86938423	0.003012049
ENSDARG00000090268	krtt1c19e	0.840329262	2.14E-11	0.353333686	0.00776139
ENSDARG00000090286	serpina1	-0.347361604	0.006758187	-0.283465508	0.029083901
ENSDARG00000090297	ldlrad2	2.285971979	1.37E-19	0.684718428	0.017413346
ENSDARG00000090340	si:dkey-27o4.1	-0.597757362	0.037918731	-0.803361952	0.002685514
ENSDARG00000090369	zgc:86896	0.850122476	1.4E-18	0.323159435	0.001309607
ENSDARG00000090392	si:dkey-71l4.2	-1.570650979	0.0000103	-0.878407864	0.011669606
ENSDARG00000090401	CABZ01020840.1	2.528380782	2.59E-15	1.386060128	0.0000174
ENSDARG00000090428	ctrb1	-1.556662532	0.04365864	-1.546920865	0.043681624
ENSDARG00000090447	mtbp	-0.995520811	0.0000115	-0.708796452	0.001167908
ENSDARG00000090468	ppp1r3aa	0.656310604	0.000831434	0.646764505	0.000559317
ENSDARG00000090629	tmtops3b	1.280200431	0.021498092	1.292317131	0.019233888
ENSDARG00000090708	si:dkey-28g23.6	-2.51997974	0.004210933	-1.903210505	0.026600914
ENSDARG00000090721	prdm2a	-0.693774397	0.0000212	-0.368904714	0.031126846
ENSDARG00000090722	C20H6orf58	1.003335222	7.26E-08	0.920300278	0.000000111
ENSDARG00000090753	si:dkey-13p1.3	1.316764606	0.007442628	1.085035992	0.032604904
ENSDARG00000090764	bcas3	0.595655971	0.000000263	0.573934639	8.81E-08
ENSDARG00000090814	si:dkey-18a10.3	1.444804577	0.0000109	1.583832764	0.00000016
ENSDARG00000090847	si:ch211-209l18.4	1.430782734	9.29E-15	1.011807612	9.04E-09
ENSDARG00000090871	si:dkey-210j14.4	-0.743989751	0.0000501	-0.420557764	0.025679211
ENSDARG00000090873	ccl34a.4	3.638387231	1.1E-26	2.009018594	5.43E-09
ENSDARG00000090912	npc2	0.659687048	0.00000894	0.65351273	0.00000223
ENSDARG00000090945	si:ch211-170d8.8	-3.697040418	0.000874542	-4.823122939	0.0000414
ENSDARG00000090969	cbln18	-2.458089163	5.14E-12	-1.632173275	0.00000043
ENSDARG00000091131	cry3b	-1.097274066	6.13E-17	-0.883522456	2.01E-13
ENSDARG00000091136	zgc:174259	-2.441134696	0.0000246	-2.618851426	0.000000766
ENSDARG00000091138	actr3	0.692261035	2.58E-09	0.337436498	0.005311961
ENSDARG00000091144	CR749162.1	5.115022467	0.000346183	5.157876086	0.000457032
ENSDARG00000091185	FP017295.1	2.352439778	0.001653337	3.21405135	0.00000217
ENSDARG00000091260	mylk4a	1.650858111	7.09E-17	0.60228651	0.004475945
ENSDARG00000091280	si:ch211-66k16.27	-1.28264754	3.27E-09	-1.240055625	2.84E-10
ENSDARG00000091298	pmela	0.745788755	0.000000012	0.450389778	0.000559317
ENSDARG00000091320	nlrc6	-1.760788018	0.00000694	-1.431697454	0.0000966
ENSDARG00000091326	cep120	-0.553150382	0.000173548	-0.450005702	0.00173584
ENSDARG00000091381	si:ch211-214c20.1	2.224737524	0.0000555	1.816582667	0.001159139
ENSDARG00000091465	si:dkey-66a8.7	0.893624479	0.000000064	0.445665605	0.012227818
ENSDARG00000091471	ccdc28a	2.025724613	3.39E-12	0.776444086	0.020839077
ENSDARG00000091514	CT737190.1	-0.679742331	0.04478537	-0.69155271	0.036855864
ENSDARG00000091555	ostf1	0.673604697	0.00000119	0.343890281	0.020299206
ENSDARG00000091595	LO018154.1	-0.519818167	0.002559138	-0.501412148	0.002324076
ENSDARG00000091633	si:dkey-177p2.18	-1.072989169	0.000564912	-1.030780176	0.000400244

Table S3. DEGs of WT vs. *terfa+/-* ∩ *terfa-/-*

ENSDARG00000091669	bicdl2l	1.27315727	3.79E-15	0.960513594	2.79E-10
ENSDARG00000091723	si:ch211-202f3.4	-1.156067082	0.025134521	-1.170443391	0.019360239
ENSDARG00000091754	CT573476.1	-0.725796184	0.004638531	-0.595186178	0.019443184
ENSDARG00000091760	cwf19l2	0.741270925	0.000118174	0.764140153	0.0000214
ENSDARG00000091800	zgc:174260	-3.250771449	2E-11	-4.829897319	3.04E-22
ENSDARG00000091801	serpinb14	1.246281478	1.04E-11	0.49504528	0.011937526
ENSDARG00000091836	casp7	-0.931342295	0.00000008	-1.128656684	3.79E-13
ENSDARG00000091879	si:dkey-29d8.3	-0.689590913	0.014396222	-0.591451994	0.034594557
ENSDARG00000091937	BX927308.1	-0.475510118	0.034988287	-0.567225951	0.007990567
ENSDARG00000091951	nuggc.2	-4.479497992	0.00000304	-1.459990612	0.021747188
ENSDARG00000091996	tcnbb	1.069001899	1.61E-11	0.503805673	0.002099611
ENSDARG00000092000	znf1020	1.065831429	0.009439497	0.91381159	0.029305582
ENSDARG00000092042	AL935153.1	-0.875773764	0.000278289	-0.82856156	0.000283647
ENSDARG00000092083	BX899181.1	2.76961686	0.000400884	1.816453178	0.037470624
ENSDARG00000092115	eif4a1a	0.357113487	0.016821406	0.349865194	0.017147206
ENSDARG00000092120	BX470185.1	2.928844696	0.0000361	1.635683735	0.036148737
ENSDARG00000092124	cox14	0.900886835	4.53E-09	0.498398469	0.001588953
ENSDARG00000092166	si:dkey-147f3.8	1.296593645	5.61E-09	0.703036873	0.00219727
ENSDARG00000092225	si:dkeyp-13a3.10	-0.743925288	0.00000655	-0.38478597	0.023344492
ENSDARG00000092257	BX510992.1	-1.595055107	0.00000905	-0.890005732	0.009807679
ENSDARG00000092277	BX663611.2	2.668225431	0.017593178	3.348512333	0.001724787
ENSDARG00000092290	si:dkeyp-26a9.2	-1.148154168	0.000000828	-0.497499589	0.044941499
ENSDARG00000092337	CU467861.6	1.664613571	1.58E-13	1.102210415	0.00000037
ENSDARG00000092354	si:ch211-284e13.6	-1.386228967	3.6E-24	-0.826679805	5.55E-11
ENSDARG00000092358	BX469930.1	-6.540677385	1.02E-11	-5.740788163	1.59E-11
ENSDARG00000092381	zgc:153759	2.523546783	0.027926495	3.148758569	0.004113904
ENSDARG00000092471	si:dkey-7j22.1	2.052200715	0.0000187	1.168730725	0.027693901
ENSDARG00000092510	CR405715.1	-4.410774703	0.002336189	-3.709823089	0.001229309
ENSDARG00000092521	jac1	3.204061332	0.000000409	2.682310586	0.0000149
ENSDARG00000092658	CR457445.1	3.260006131	0.003028142	2.699694946	0.019361877
ENSDARG00000092662	dnaaf3	7.058950784	0.00000617	6.49259087	0.0000604
ENSDARG00000092719	AL954655.2	-0.610512586	0.013440057	-0.537399655	0.027772034
ENSDARG00000092738	si:ch211-263k4.2	1.105217312	0.004435067	1.006182519	0.00914587
ENSDARG00000092770	si:ch211-253p18.2	3.44296227	0.00000286	3.827155057	5.19E-08
ENSDARG00000092774	tars3	-1.040851269	2.09E-09	-0.880367132	4.95E-08
ENSDARG00000092788	si:dkey-21e2.15	-3.31112536	0.000000929	-1.243279301	0.044359573
ENSDARG00000092798	ppib	0.489938711	0.0000677	0.312693215	0.013440492
ENSDARG00000092807	si:dkey-151g10.6	0.886861638	1.22E-14	0.668490717	4.82E-10
ENSDARG00000092813	fndc7rs4	-0.873535328	0.00000097	-0.467826579	0.010025742
ENSDARG00000092829	CR589943.1	-2.279217222	3.68E-09	-2.077784633	1.83E-09
ENSDARG00000092856	si:ch211-210c8.7	0.581298794	0.000984358	0.384921384	0.037808674
ENSDARG00000092878	si:dkeyp-85e10.3	-3.553773167	0.000365741	-1.649038977	0.043211535
ENSDARG00000092920	si:ch211-106h4.12	-0.607884418	0.007062493	-0.493800766	0.02897693
ENSDARG00000092968	znf1128	1.850800143	0.000146431	1.729445106	0.000282778
ENSDARG00000092985	CU499330.1	-0.736143109	0.013183161	-0.60401619	0.043409874
ENSDARG00000093024	si:ch211-213a13.2	1.560587858	0.00000543	1.349626237	0.0000487
ENSDARG00000093042	NA	-6.50350753	0.002322289	-6.514444983	0.000670104
ENSDARG00000093052	c6	2.964428993	8.65E-18	1.059673599	0.005212327
ENSDARG00000093065	ubac2	-0.60392623	0.000452485	-0.461406225	0.006446642
ENSDARG00000093068	c3b.1	0.68158451	0.0000114	0.453266883	0.003857646
ENSDARG00000093087	BX649388.3	5.528719393	0.000978931	5.381259864	0.002125398
ENSDARG00000093224	CR456628.1	-4.246498292	0.002912088	-2.646854562	0.007909539
ENSDARG00000093289	si:dkey-9i23.14	-2.154957601	0.001142358	-1.346799571	0.035215542
ENSDARG00000093320	BX323028.1	4.240485455	0.000146937	4.117839782	0.000230586
ENSDARG00000093392	CT583651.1	8.535059319	7.49E-10	8.442634709	1.99E-09

Table S3. DEGs of WT vs. *terfa+/-* ∩ *terfa-/-*

ENSDARG00000093443	BX323559.4	2.741770016	9.75E-08	2.210397053	0.0000163
ENSDARG00000093444	si:dkey-106l3.7	-0.856136709	0.012803617	-1.061511084	0.000925882
ENSDARG00000093452	BX511129.2	1.051597901	0.022230904	1.091898764	0.015406233
ENSDARG00000093471	CR361551.1	-1.977372563	0.00000432	-0.945540871	0.017091878
ENSDARG00000093484	si:dkey-95p16.2	-1.010402629	0.0000135	-0.912753985	0.0000246
ENSDARG00000093494	si:ch211-217k17.9	0.930094274	7.16E-12	0.755014141	2.66E-09
ENSDARG00000093535	BX005309.1	-0.762368567	0.000000064	-0.587170719	0.0000105
ENSDARG00000093546	si:ch73-56d11.5	4.514885356	2.65E-91	2.75784471	7.82E-39
ENSDARG00000093550	BX005438.1	-1.872058502	0.000272834	-1.077584276	0.033955184
ENSDARG00000093572	lamc3	0.690254397	4.84E-10	0.251992961	0.040202028
ENSDARG00000093576	BX000981.2	3.599175411	0.000000975	2.920641433	0.0000919
ENSDARG00000093584	zgc:193505	1.206627165	4.37E-12	0.863153636	0.000000191
ENSDARG00000093591	si:ch211-282k23.2	2.201815808	0.000077	2.210899301	0.0000347
ENSDARG00000093608	ccl34b.1	-1.688081747	1.55E-08	-0.80051477	0.007002038
ENSDARG00000093639	BX537282.1	8.279374549	0.000000408	5.273869179	0.004132345
ENSDARG00000093667	BX005283.1	-4.718358447	0.00000121	-2.643695847	0.0000283
ENSDARG00000093671	BX001030.1	0.891819793	0.002016359	1.10937982	0.0000257
ENSDARG00000093738	pth1a	6.310242101	7.32E-11	4.16453347	0.0000642
ENSDARG00000093753	BX004774.2	-3.542259874	2.31E-33	-1.401123269	3.46E-11
ENSDARG00000093761	si:ch211-250k18.7	2.585226542	0.002227253	2.373171068	0.005583045
ENSDARG00000093764	si:dkey-145c18.5	3.855407491	0.000333685	3.239521598	0.004232782
ENSDARG00000093822	si:dkey-259j3.5	1.316122855	0.004295969	1.018229335	0.034234794
ENSDARG00000093844	zgc:136461	-4.204895153	0.000753961	-3.113767707	0.013011452
ENSDARG00000093862	si:ch211-274k16.2	1.988100518	0.00000418	1.613169246	0.000172291
ENSDARG00000093881	NA	-4.849981758	0.02232698	-6.321998929	0.000751322
ENSDARG00000093921	BX276103.2	-4.288296491	1.58E-12	-3.606851545	1.17E-13
ENSDARG00000093924	BX890562.2	2.813025456	1.09E-08	2.373373315	0.00000112
ENSDARG00000093936	si:dkeyp-1h4.6	0.806044656	0.0000201	0.640979013	0.000535869
ENSDARG00000093945	vma21	-0.421666882	0.0235874	-0.396720156	0.03194819
ENSDARG00000093964	si:ch211-250k18.6	2.90840587	0.000385299	2.07904745	0.019684764
ENSDARG00000094088	si:ch211-229n2.7	0.958282833	0.000100249	0.725763447	0.003538251
ENSDARG00000094197	znfl1l	2.999749105	4.31E-08	4.236096794	3.78E-18
ENSDARG00000094217	si:dkey-17e16.15	-2.476794334	6.32E-22	-1.171259061	0.000000405
ENSDARG00000094249	wu:fc17b08	-0.560792863	0.002950415	-0.620282194	0.000417868
ENSDARG00000094310	si:ch211-255g12.6	-1.289917386	1.86E-17	-1.685805724	4.2E-36
ENSDARG00000094324	efemp2a	0.698448465	4.7E-09	0.330516256	0.008511285
ENSDARG00000094331	BX901918.2	10.63475503	1.08E-13	9.799563729	1.07E-11
ENSDARG00000094400	si:dkey-199f5.6	-2.9777595	0.000000682	-2.353263875	0.00000358
ENSDARG00000094422	si:dkey-12l12.1	-0.776611708	0.0000122	-0.819375717	0.000000479
ENSDARG00000094436	CR848723.1	4.972530074	0.00000126	5.107613972	0.000000562
ENSDARG00000094451	cfp	-1.19018888	9.08E-08	-1.379922588	5.59E-12
ENSDARG00000094508	CR925709.2	9.326934625	6.44E-12	7.294636381	0.000000366
ENSDARG00000094557	nupr1b	-0.748831634	0.0000521	-0.839795528	0.000000742
ENSDARG00000094560	BX005254.2	3.504456639	9.73E-10	3.568078212	1.14E-10
ENSDARG00000094563	znf1144	-0.715456492	0.006194335	-0.73108686	0.003354594
ENSDARG00000094584	BX470240.1	5.516280115	1.24E-23	3.614308982	3.36E-11
ENSDARG00000094653	si:dkey-149m13.5	1.716643143	0.000395241	1.173323633	0.023206722
ENSDARG00000094681	hbl2	-1.216798693	0.024796191	-1.195500783	0.023878067
ENSDARG00000094732	mical3b	-0.626850267	0.000297439	-0.508861002	0.002763272
ENSDARG00000094844	BX323457.3	6.982351582	0.0000539	4.799936653	0.01429196
ENSDARG00000094889	cplx4c	-1.561514893	0.000000131	-1.358279774	0.000000759
ENSDARG00000094899	si:ch1073-143l10.2	1.272610226	0.00015603	1.114187531	0.000721792
ENSDARG00000094901	abcc6b.2	3.272268222	5.12E-31	2.584216155	9.16E-23
ENSDARG00000094946	si:ch211-132b12.2	1.491210541	0.000000657	1.505659291	7.12E-08
ENSDARG00000094965	nfil3-5	-0.90535279	2.86E-15	-0.540397816	0.000000915

Table S3. DEGs of WT vs. *terfa+/-* ∩ *terfa-/-*

ENSDARG00000094977	si:ch211-156j22.4	-1.491785253	7.57E-08	-0.63557323	0.031252848
ENSDARG00000094978	si:ch73-18b11.2	1.084299227	0.002556166	0.771828506	0.042116347
ENSDARG00000094983	ccl34a.3	7.862746489	9.25E-08	5.870989186	0.000247562
ENSDARG00000094994	si:dkey-106c17.2	3.469243187	0.0000349	3.296786106	0.0000568
ENSDARG00000095082	ITLN1	2.352356619	4.63E-09	2.242365265	2.38E-09
ENSDARG00000095139	CR388373.2	-2.489785688	1.17E-12	-1.55204713	0.000000203
ENSDARG00000095157	zgc:163080	0.782794055	0.001221456	0.546695698	0.030315722
ENSDARG00000095185	si:ch211-274p24.2	-2.306042547	1.36E-08	-2.313414163	1.62E-10
ENSDARG00000095190	BX901918.3	6.14013257	0.000441476	5.696725574	0.001820579
ENSDARG00000095193	si:dkey-23c22.7	5.142428163	0.000000105	5.078714432	0.000000161
ENSDARG00000095252	si:dkey-40h20.1	4.070401853	1.82E-13	2.332773994	0.0000418
ENSDARG00000095268	si:dkey-261h17.1	-0.656428598	2.27E-09	-0.256647843	0.030930266
ENSDARG00000095280	CU682811.3	2.518652637	9.02E-16	1.80270062	3.91E-09
ENSDARG00000095283	si:dkeyp-2e4.3	1.37260992	0.00000202	0.828929649	0.00701686
ENSDARG00000095369	zgc:112966	6.219585973	0.00010985	6.656928977	0.0000395
ENSDARG00000095390	BX537288.2	-2.328409343	0.0000257	-2.035313118	0.0000478
ENSDARG00000095400	si:ch73-52f15.5	1.145227417	1.79E-19	0.630147145	0.000000505
ENSDARG00000095432	BX323074.2	-1.229655018	6.82E-08	-0.885898196	0.0000418
ENSDARG00000095448	ftr30	2.907236486	0.00000413	2.832440552	0.00000393
ENSDARG00000095451	si:ch211-196l7.4	-0.808926702	3.31E-08	-0.469786764	0.001351882
ENSDARG00000095509	CR759885.1	-1.276007421	0.000754256	-1.088280185	0.002522583
ENSDARG00000095527	CR392363.2	3.839807105	0.000983415	3.243608284	0.009007412
ENSDARG00000095592	si:dkey-11o15.5	1.655560013	0.003591202	2.184239148	0.0000228
ENSDARG00000095594	si:dkey-184a18.5	-0.838717632	0.02407743	-1.037451266	0.002967407
ENSDARG00000095615	si:dkeyp-86h10.3	0.518996381	0.003182373	0.37166314	0.041614923
ENSDARG00000095643	si:dkey-253d23.3	-0.925281328	0.0000939	-1.142377412	0.000000109
ENSDARG00000095688	zgc:162509	4.787481358	0.002153047	4.180110052	0.010292346
ENSDARG00000095696	trpm2	-0.725339049	0.021013216	-0.883823027	0.002738014
ENSDARG00000095767	dio3b	1.643264456	1.27E-13	0.532607474	0.031406307
ENSDARG00000095777	si:ch211-218h8.3	3.884449803	0.019110984	3.912150572	0.024597796
ENSDARG00000095802	pcare2	-2.783701802	5.46E-25	-0.651385786	0.024322024
ENSDARG00000095826	smdt1a	2.599344253	0.00000145	1.719813826	0.002798933
ENSDARG00000095861	si:ch1073-441p17.1	3.855812622	0.013906162	4.066058867	0.012365358
ENSDARG00000095865	oxr1a	-0.461480344	0.001543745	-0.391701196	0.006392902
ENSDARG00000095904	prpf31	-0.473659726	0.000095	-0.332376616	0.006331732
ENSDARG00000095907	AL928685.2	-0.758390104	0.00113266	-0.626806972	0.006066106
ENSDARG00000095912	si:dkey-229b18.3	-0.341268029	0.012363799	-0.396572651	0.001979683
ENSDARG00000095915	col6a3	1.520495893	1.11E-09	0.960139235	0.000109805
ENSDARG00000095922	CR855300.1	0.731461937	0.002102068	0.579841482	0.016318513
ENSDARG00000095944	si:ch73-103b2.1	2.677499593	0.009665209	3.14319972	0.001657544
ENSDARG00000095946	si:ch73-341k19.1	-0.491117549	0.04392566	-0.5074967	0.033077858
ENSDARG00000095949	si:dkey-22i16.9	2.83189532	0.0000051	1.568217154	0.02010318
ENSDARG00000095956	CU467655.1	-2.940428725	0.0000251	-1.589835036	0.013627869
ENSDARG00000096003	atp5md	1.371847873	4.44E-17	1.023489212	1.92E-11
ENSDARG00000096065	si:dkey-156k2.7	5.305347403	0.009751013	4.700891809	0.032981661
ENSDARG00000096099	BX465862.3	1.458237172	0.015050252	1.236948513	0.04620592
ENSDARG00000096118	ost4	1.38145207	3.65E-21	1.043544088	2.81E-14
ENSDARG00000096233	si:dkey-260c8.6	1.475132422	0.000392689	0.981340892	0.028243345
ENSDARG00000096273	si:dkey-3n22.9	1.490608395	0.015692811	1.573763736	0.008485976
ENSDARG00000096310	wu:fj64h06	1.785834806	2.52E-40	0.615787975	0.0000146
ENSDARG00000096508	BX322618.1	-1.585928817	0.000317062	-1.396985934	0.00104337
ENSDARG00000096519	irbpl	6.369553097	0.0000126	7.316276696	0.00000049
ENSDARG00000096527	NA	-7.122290033	0.000287222	-6.171441895	0.00050361
ENSDARG00000096544	CR774189.1	5.939029021	0.000633454	4.944365115	0.009007412
ENSDARG00000096599	AL928650.3	-1.405716132	0.026691637	-3.184010362	0.000000541

Table S3. DEGs of WT vs. *terfa+/-* ∩ *terfa-/-*

ENSDARG00000096603	bmb	4.017101422	8.67E-102	2.132414263	3.98E-33
ENSDARG00000096626	CU914144.1	-1.395788612	0.003578574	-1.19791485	0.008979531
ENSDARG00000096631	MED9	0.532369053	0.003788093	0.446986816	0.01555322
ENSDARG00000096637	si:dkey-11c5.11	1.865296403	0.000111889	1.251146798	0.01505119
ENSDARG00000096655	BX901920.1	2.649853735	9.51E-10	2.422817605	1.9E-09
ENSDARG00000096701	si:dkey-21e13.3	-2.495472641	2.21E-32	-1.038031404	2.71E-09
ENSDARG00000096716	hmx3b	1.764939368	0.003165724	1.588102934	0.008461179
ENSDARG00000096762	si:dkey-61p9.11	4.121420071	1.29E-53	1.705368173	5.47E-10
ENSDARG00000096777	si:ch73-81k8.2	1.817395422	0.002062528	1.280543791	0.045434954
ENSDARG00000096808	si:dkey-287g12.6	-0.793021777	0.001550835	-0.841785791	0.000325063
ENSDARG00000096822	CR847898.1	4.825374094	0.02393656	5.515680817	0.009334744
ENSDARG00000096829	blvrb	0.58259612	0.000117909	0.394364715	0.01089276
ENSDARG00000096849	si:dkey-16p21.8	0.818638297	8.66E-14	0.472201576	0.0000109
ENSDARG00000096856	znf1012	-1.622876256	0.021700433	-1.519620593	0.02788802
ENSDARG00000096863	si:ch73-380l3.4	3.481680192	0.000489192	3.006524997	0.003763972
ENSDARG00000096903	CU062633.1	5.22480928	0.017534372	5.774382533	0.008678432
ENSDARG00000096946	BX897682.1	6.042336617	1.03E-10	4.194948165	0.0000143
ENSDARG00000096950	BX927244.1	-4.33274071	0.000000345	-4.930356704	2.96E-09
ENSDARG00000096994	AL929222.1	-4.097446913	0.000311234	-2.073728224	0.006699886
ENSDARG00000097003	CR855375.2	-4.142250983	0.003991688	-2.562711084	0.012942382
ENSDARG00000097018	CR628341.1	6.508707183	3.18E-08	6.072483888	0.000000703
ENSDARG00000097057	fxyd6l	-0.473972764	0.006052776	-0.370342987	0.035642486
ENSDARG00000097080	si:ch73-181m17.1	3.757049587	1.29E-36	1.703490169	3.27E-08
ENSDARG00000097102	si:ch73-281n10.2	-1.138054472	1.13E-18	-0.365350114	0.00838212
ENSDARG00000097118	CU693494.2	1.035416023	0.00000131	1.022153599	0.000000249
ENSDARG00000097157	si:ch211-207n23.2	1.296976035	2.87E-09	0.612585155	0.00790967
ENSDARG00000097158	BX294100.1	-2.381810971	2.33E-08	-1.076121984	0.008448402
ENSDARG00000097162	CU861891.1	-1.47235921	0.029515246	-1.470663249	0.02495185
ENSDARG00000097247	BX322555.3	1.572004696	8.03E-08	1.295512101	0.00000363
ENSDARG00000097264	si:ch211-40k21.5	-0.636124458	0.001201587	-0.411524516	0.040847393
ENSDARG00000097265	CR847898.2	1.625697267	0.000529719	1.104031354	0.027277662
ENSDARG00000097269	si:ch211-222n22.1	1.26238601	0.011306719	1.090904712	0.033346664
ENSDARG00000097285	ANPEP	-0.761312227	0.0000651	-0.434899412	0.027455089
ENSDARG00000097292	slc26a6	1.489539625	0.000152409	0.949944294	0.022662644
ENSDARG00000097339	slc9a1b	0.436554997	0.036041328	0.633327033	0.000751322
ENSDARG00000097352	si:ch211-147g22.7	6.077902017	0.0000909	6.614017468	0.0000229
ENSDARG00000097353	BX324164.3	0.986769021	0.011370312	0.965506922	0.011669606
ENSDARG00000097416	si:ch73-306e8.2	-1.433055107	4.45E-08	-0.61636498	0.01851534
ENSDARG00000097452	CU929070.5	-0.608446362	0.009678853	-0.484157705	0.039351563
ENSDARG00000097491	ugt1b1	0.492957939	0.001062623	0.354471161	0.021833705
ENSDARG00000097504	CR626907.3	1.270160228	0.000000769	1.265137469	0.000000102
ENSDARG00000097533	BX571880.1	1.36680617	0.00000339	1.089725545	0.000129465
ENSDARG00000097573	si:ch211-107e6.5	5.706300922	0.000693109	4.439204147	0.019456
ENSDARG00000097574	CR847898.3	3.769728568	0.0000118	4.003566208	0.00000221
ENSDARG00000097576	zgc:92912	-1.156140201	5.94E-15	-0.937349749	2.19E-12
ENSDARG00000097591	proser3	-0.69213526	0.000970898	-0.484616727	0.021582925
ENSDARG00000097594	FP236741.1	-2.635728834	0.005375116	-1.721639279	0.035965874
ENSDARG00000097599	CR925804.1	5.225458615	0.004701432	5.136983818	0.007543533
ENSDARG00000097615	si:ch211-108d22.2	-0.802794637	0.0000446	-0.743110091	0.0000612
ENSDARG00000097639	si:ch73-265h17.4	1.979411886	0.00000332	1.604244674	0.000156633
ENSDARG00000097653	si:dkey-23k10.3	3.083344558	1.37E-12	2.361885614	4.35E-08
ENSDARG00000097660	trappc2	0.587903717	0.0000096	0.594794557	0.00000139
ENSDARG00000097663	BX324206.3	-1.060343045	0.00000008	-0.80076941	0.000016
ENSDARG00000097692	si:ch211-116o3.5	-0.620765309	0.000000509	-0.580377882	0.000000349
ENSDARG00000097714	FP017215.1	0.688186527	0.025477046	0.933374749	0.000927514

Table S3. DEGs of WT vs. *terfa+/-* ∩ *terfa-/-*

ENSDARG00000097718	stard10	-0.292656804	0.038312568	-0.352288823	0.008457906
ENSDARG00000097728	CR388132.1	7.293167332	0.00000295	6.260519939	0.0000842
ENSDARG00000097740	elob	-0.521451606	0.0407914	-0.546134986	0.028058671
ENSDARG00000097746	si:rp71-77l1.1	1.627062161	4.41E-18	0.851990738	0.00000475
ENSDARG00000097761	BX005234.1	1.367665934	0.012850927	1.7073861	0.000812589
ENSDARG00000097762	CU693494.3	1.802169449	0.00198653	2.014463593	0.000231921
ENSDARG00000097770	si:ch211-167j9.4	-1.285026086	0.00000432	-0.798336692	0.003691248
ENSDARG00000097772	si:ch211-185a18.2	0.525978333	0.013655716	0.449946209	0.037841689
ENSDARG00000097794	CR626944.2	0.772557491	0.000507105	0.549420876	0.015738088
ENSDARG00000097795	zgc:162952	-0.546966095	0.039269327	-0.821383767	0.000662051
ENSDARG00000097807	BX530017.2	2.317851245	0.000765684	2.371387875	0.000332953
ENSDARG00000097824	smco4	0.834257231	0.000000401	0.372769437	0.039460224
ENSDARG00000097826	si:dkey-239b22.2	0.266068892	0.020648498	0.250272566	0.028478676
ENSDARG00000097827	lrp1aa	0.519304038	0.000304223	0.372659596	0.010471396
ENSDARG00000097871	si:ch1073-145m9.1	1.079929992	0.000862562	1.337606287	0.00000566
ENSDARG00000097884	CU928013.1	-0.439860784	0.033279179	-0.524577227	0.007330815
ENSDARG00000097889	si:ch73-265h17.2	2.622248811	0.0000508	2.604824447	0.0000272
ENSDARG00000097921	BX465857.1	5.379254107	0.022755104	5.987961278	0.011398229
ENSDARG00000097929	BX005392.3	6.455747552	1.78E-11	5.019557133	5.69E-08
ENSDARG00000097964	arhgef33	0.545738262	0.003413089	0.462529988	0.013118126
ENSDARG00000097966	BX936420.1	-1.70446069	0.03113181	-2.38041261	0.001963534
ENSDARG00000097973	si:ch1073-190k2.1	12.71176784	0.000000109	11.58481372	0.000000599
ENSDARG00000097976	CR812464.1	-1.284065415	0.010822555	-1.23050741	0.011556557
ENSDARG00000098012	itgae.1	-1.57906167	2.21E-09	-1.454253248	9.85E-10
ENSDARG00000098051	opn6b	-3.560632973	1.79E-25	-1.649779827	2.23E-08
ENSDARG00000098082	CU459093.1	-1.267093547	0.000179659	-0.807625408	0.018849479
ENSDARG00000098087	si:dkey-30f3.2	1.665679111	0.026512144	1.627948651	0.030984792
ENSDARG00000098096	slc12a1	-1.076435373	0.003674808	-1.57295451	0.00000247
ENSDARG00000098108	dusp2	-0.503677676	0.002858003	-0.427261106	0.010143969
ENSDARG00000098118	trappc10	0.302951958	0.008752768	0.299517131	0.007682388
ENSDARG00000098121	FP101882.1	-0.821467601	0.006664394	-0.642839322	0.035917154
ENSDARG00000098164	aipl2	-2.816420494	6.53E-33	-0.633287167	0.005112949
ENSDARG00000098171		1.666150259	4.58E-42	1.199431836	2.58E-26
ENSDARG00000098176	si:rp71-1h20.5	-0.675461659	0.017598544	-0.577365036	0.042355173
ENSDARG00000098189	si:dkey-16p6.1	3.206771791	0.0000482	3.033023379	0.000127946
ENSDARG00000098198	NA	-4.696463343	0.023941532	-5.204470483	0.006282349
ENSDARG00000098201	mtmr3	0.385044118	0.033085562	0.386445252	0.030264903
ENSDARG00000098214	CABZ01072487.1	-0.907437397	0.001201587	-1.037857937	0.0000606
ENSDARG00000098223	AL773558.1	3.648478695	4.09E-15	3.065083176	3.8E-12
ENSDARG00000098228	thrap3b	-0.479767872	0.000000067	-0.426995605	0.000000242
ENSDARG00000098237	fbn2b	0.768092779	0.0000971	0.474939337	0.020537986
ENSDARG00000098258	SLC16A7	-0.686172156	0.007309726	-0.57872392	0.023893563
ENSDARG00000098286	CU695232.1	1.074511628	8.22E-10	0.715992597	0.0000337
ENSDARG00000098308	CR936540.1	0.732185631	0.0000805	0.562695582	0.002322407
ENSDARG00000098332	zgc:112970	3.372211674	8.89E-13	3.623053299	7.84E-16
ENSDARG00000098344	rab18b	0.724209207	0.000000134	0.326435421	0.0282042
ENSDARG00000098348	znf326	1.134550162	0.008372661	1.093909733	0.009993518
ENSDARG00000098350	mxd3	-1.261212631	1.1E-09	-0.54684991	0.010894652
ENSDARG00000098359	il17rd	0.897911702	8.11E-20	0.474142262	0.00000108
ENSDARG00000098360	cyp19a1b	-2.86665701	0.000000478	-2.396170332	0.00000117
ENSDARG00000098369	si:dkey-165o8.1	-2.715827774	4.14E-09	-1.360836536	0.000738228
ENSDARG00000098374	FLNB	1.050685731	5.86E-14	0.552070345	0.000073
ENSDARG00000098390	si:ch211-76m11.8	2.11479612	0.0000599	1.643005571	0.002207373
ENSDARG00000098397	FP016106.1	4.917026714	0.000880655	4.351407722	0.002929494
ENSDARG00000098452	si:dkey-151g22.1	2.421058157	0.001328661	1.748976496	0.031979334

Table S3. DEGs of WT vs. *terfa+/-* ∩ *terfa-/-*

ENSDARG00000098462	CU570782.1	2.501997243	0.003055858	2.32848581	0.007005119
ENSDARG00000098465	si:ch73-366l1.5	1.425082326	4.3E-22	0.733167001	0.000000323
ENSDARG00000098481	anxa1d	1.280988543	2.09E-10	0.83247342	0.0000276
ENSDARG00000098522	BX649431.1	-1.667002745	0.030813845	-1.510760129	0.045705398
ENSDARG00000098527	CABZ01060491.1	3.841134582	0.0000449	3.065642034	0.002164083
ENSDARG00000098540	zc3h7ba	0.695928337	0.00000111	0.340182215	0.028493825
ENSDARG00000098584	CABZ01102240.1	0.675700773	0.00000192	0.315010289	0.04103056
ENSDARG00000098592	zgc:92140	-0.535150126	0.0000156	-0.265809614	0.043488504
ENSDARG00000098621	CR759843.1	2.001732683	0.000891363	1.67436697	0.006397666
ENSDARG00000098639	pycr1b	0.81771956	2.76E-10	0.625035265	0.000000355
ENSDARG00000098641	nbeal2	-0.358094389	0.043377407	-0.503046672	0.00205312
ENSDARG00000098646	mthfd2	0.677663958	0.00000558	0.524871754	0.000304449
ENSDARG00000098660	CU469526.1	-0.552603251	0.000523329	-0.538531608	0.000348863
ENSDARG00000098733	pxdn	1.10712362	0.00000121	0.601147488	0.01153209
ENSDARG00000098746	dhrs13l1	1.105632932	1.17E-12	0.721437635	0.00000184
ENSDARG00000098753	elmo1	-0.746070537	3.81E-14	-0.243926761	0.023059792
ENSDARG00000098777	CU693379.1	0.634708505	2.04E-08	0.314187897	0.008085455
ENSDARG00000098785	glrx3	0.475535149	0.0000741	0.288094611	0.02157822
ENSDARG00000098790	sowahca	-1.23044089	0.000119586	-0.668064059	0.043373815
ENSDARG00000098837	tgm5l	1.694671425	0.0000172	1.22665869	0.002292299
ENSDARG00000098849	SLC25A29	-0.956717961	0.003086758	-1.100847615	0.00024919
ENSDARG00000098865	BX548044.7	-3.854187154	0.032163949	-3.92406981	0.02178688
ENSDARG00000098884	BX649355.1	-2.014794225	0.031149211	-1.77841497	0.048730184
ENSDARG00000098890	cyp2aa9	1.826182809	1.67E-24	1.110267149	9.59E-11
ENSDARG00000098892	si:ch211-223a21.1	1.375981722	0.0000695	1.101479162	0.001562907
ENSDARG00000098911	zgc:153293	-0.833181847	0.000252063	-0.692813226	0.001505677
ENSDARG00000098924	suz12b	-0.866512425	2.33E-15	-0.640985459	2.44E-10
ENSDARG00000098928	si:ch211-196h24.1	1.344136122	0.028887088	1.405089363	0.021017066
ENSDARG00000098941	CU856520.1	-0.666847518	0.012346892	-0.720447623	0.004662423
ENSDARG00000098972	mrps16	-0.385103651	0.014275626	-0.420458028	0.005009795
ENSDARG00000098988	si:ch211-193i15.1	-5.403635804	0.0000528	-3.390016203	0.0000278
ENSDARG00000098991	znf1095	2.320700024	0.000912966	1.645041509	0.029754036
ENSDARG00000099002	creb5a	1.175967537	0.00000807	0.593255271	0.04390204
ENSDARG00000099006	CABZ01029938.1	1.678521557	0.0000191	1.105508923	0.006284564
ENSDARG00000099023	CABZ01034698.1	3.463212872	0.00057298	2.520385763	0.01971246
ENSDARG00000099035	pcdh1g33	-1.370389689	3.38E-08	-0.698948419	0.005873705
ENSDARG00000099038	kcng1	0.933135484	0.0000462	0.711992679	0.001887741
ENSDARG00000099079	aclya	0.281864199	0.005626332	0.241413786	0.017696555
ENSDARG00000099116	nagk	0.410839978	0.006738167	0.368548251	0.01440694
ENSDARG00000099118	TRAPPC9	0.754460856	6.36E-08	0.890339372	1.07E-12
ENSDARG00000099149	hs3st3b1a	-1.814608257	3.15E-08	-0.918891517	0.00432267
ENSDARG00000099152	si:dkey-190j3.2	1.979645888	0.0000178	1.244774767	0.012127079
ENSDARG00000099154	CABZ01032488.1	1.467326127	1.39E-11	1.068116145	0.000000255
ENSDARG00000099161	dnaaf4	1.433903555	0.00253698	1.124719013	0.022281018
ENSDARG00000099175	hmgb1a	-1.763821851	1.35E-56	-1.013271324	3.63E-23
ENSDARG00000099183	fkbp10a	0.769612664	0.0000446	0.492323596	0.011918138
ENSDARG00000099186	slc1a5	0.544138409	0.000000398	0.522124007	0.000000157
ENSDARG00000099195	ier2a	-0.679555619	0.000197499	-0.601879343	0.0006001
ENSDARG00000099200	zgc:123103	0.419247583	0.002723112	0.291638386	0.045054803
ENSDARG00000099226	CABZ01076667.1	0.315966935	0.005693424	0.268629985	0.019055624
ENSDARG00000099227	galnt13	-1.636825378	2.37E-30	-0.373343429	0.010038624
ENSDARG00000099236	10-Sep	0.591310776	0.000000122	0.357466932	0.001515071
ENSDARG00000099257	si:ch73-119p20.1	-0.606213893	0.000262718	-0.42586584	0.011309385
ENSDARG00000099273	zgc:103508	-0.835558094	0.0000206	-0.481139504	0.016748259
ENSDARG00000099291	lsr	0.418415613	0.000400259	0.263751831	0.033492249

Table S3. DEGs of WT vs. *terfa+/-* ∩ *terfa-/-*

ENSDARG00000099294	si:ch73-199g24.2	0.432316432	0.003672491	0.452904082	0.001358942
ENSDARG00000099298	xrcc5	0.99159746	3.6E-11	0.516522196	0.000683687
ENSDARG00000099301	cdc37	0.334479546	0.014765149	0.416344311	0.001084989
ENSDARG00000099306	zgc:175088	1.012368073	3.3E-10	0.630141033	0.0000676
ENSDARG00000099309	si:zfos-169g10.3	1.154213165	0.00000719	0.689357535	0.01143236
ENSDARG00000099313	naga	0.900714948	0.0000102	1.138886441	4.45E-10
ENSDARG00000099346	znf1079	6.168380393	0.000160361	5.884125307	0.000529341
ENSDARG00000099362	CABZ01088346.1	0.748966392	0.0000283	0.557845585	0.001810587
ENSDARG00000099364	BX511034.5	1.495601326	0.017761522	2.241899084	0.0000685
ENSDARG00000099382	si:ch211-209n20.1	4.76536209	0.0000405	3.908805419	0.001723792
ENSDARG00000099384	si:dkey-238k10.1	-3.279151315	1.31E-20	-0.803436507	0.00688274
ENSDARG00000099416	ly6m3	6.346040767	0.00000244	5.706442016	0.0000451
ENSDARG00000099425	CABZ01073265.1	1.291472039	1.17E-22	0.429777875	0.002087611
ENSDARG00000099428	si:dkey-15h8.17	1.756737615	0.000000672	2.350493528	6.69E-14
ENSDARG00000099439	serp1	0.855134895	2.93E-09	0.903242266	4.72E-12
ENSDARG00000099445	cc2d1b	0.485326868	0.022376992	0.60656946	0.002200252
ENSDARG00000099446	slit1b	0.569211376	0.000314771	0.381930836	0.01914779
ENSDARG00000099447	pcdh1g18	-2.241991272	2.95E-12	-0.853140921	0.005415289
ENSDARG00000099470	muc5.3	1.92997532	1.25E-08	1.228492184	0.000275067
ENSDARG00000099509	btr23	1.228474832	0.011799214	1.009093419	0.046512995
ENSDARG00000099511	CABZ01034698.2	2.247647115	3.28E-13	1.948884731	1.83E-11
ENSDARG00000099519	cpdp	0.577984317	0.00000265	0.446838255	0.000190862
ENSDARG00000099534	CABZ01066926.1	-1.18417897	5.97E-09	-0.622823124	0.00205312
ENSDARG00000099572	hmgn2	-1.638474977	5.45E-24	-0.579447467	0.000533714
ENSDARG00000099583	CABZ01034082.1	7.979767763	0.000000017	6.778907735	0.00000523
ENSDARG00000099624	chmp1b	0.303570947	0.017929847	0.275729985	0.031570911
ENSDARG00000099645	zgc:153184	-0.817109818	0.00000077	-0.44038518	0.008296814
ENSDARG00000099657	akt1	0.502782704	0.023646817	0.494033138	0.02430403
ENSDARG00000099660	CU915256.1	1.946254281	0.042948103	2.605534029	0.003651706
ENSDARG00000099672	capgb	1.201969257	4.82E-24	0.530480389	0.00000784
ENSDARG00000099674	dicp3.1	1.096253131	0.002587496	0.77713656	0.04495069
ENSDARG00000099695	si:dkey-61n16.5	-2.316222605	1.2E-20	-0.513209845	0.046152264
ENSDARG00000099698	BX548059.1	6.210217823	5.47E-09	4.018740638	0.000665808
ENSDARG00000099719	cdkn1d	-0.783479443	9.62E-12	-0.318759228	0.008183527
ENSDARG00000099741	mvb12a	0.948263919	5.93E-09	0.585125101	0.000372664
ENSDARG00000099753	zgc:63863	0.482350428	0.022230078	0.658787315	0.000612705
ENSDARG00000099764	CR855389.1	2.371088245	0.000000142	1.762119176	0.00008
ENSDARG00000099774	cyb5b	0.466426514	0.001042407	0.309886677	0.03720392
ENSDARG00000099825	zgc:174944	1.979514342	0.0000183	1.053695036	0.040249678
ENSDARG00000099827	CABZ01000633.1	6.384199933	0.0000122	6.001519327	0.0000848
ENSDARG00000099829	BX927336.2	4.156394547	5.28E-10	2.882451207	0.000035
ENSDARG00000099839	si:ch211-9d9.1	-0.957969553	0.0000201	-0.611848341	0.005417608
ENSDARG00000099845	zgc:174275	0.929911202	0.014789537	1.208923584	0.000540483
ENSDARG00000099846	si:ch211-154o6.3	0.822413893	0.000000345	0.955466608	4.88E-11
ENSDARG00000099871	myo7aa	-0.864423249	8.34E-11	-0.485568177	0.000184161
ENSDARG00000099885	trim105	-0.398785866	0.024847508	-0.372370826	0.034565097
ENSDARG00000099918	arl4aa	-0.616868207	0.000203928	-0.683253504	0.00000756
ENSDARG00000099961	bnip3	-0.820385171	0.00000438	-0.454374558	0.011669606
ENSDARG00000099970	CR383676.2	0.671974165	0.000148286	0.761088491	0.00000292
ENSDARG00000100000	CR352226.5	0.90893517	8.72E-09	0.923759538	1.67E-10
ENSDARG00000100006	si:ch211-76m11.5	3.010525759	0.00000333	1.86969648	0.008920076
ENSDARG00000100047	ergic1	0.548515792	0.0000833	0.627205268	0.000000863
ENSDARG00000100057	ppfibp1b	0.294430849	0.047314705	0.476853879	0.000305024
ENSDARG00000100061	si:dkey-16p6.1	-1.352384179	0.010872361	-1.058266545	0.043461557
ENSDARG00000100072	mgrn1a	-0.319670374	0.020169649	-0.367576188	0.00482211

Table S3. DEGs of WT vs. *terfa+/-* ∩ *terfa-/-*

ENSDARG00000100075	abcg2a	0.664830033	0.000586327	0.519434832	0.007238251
ENSDARG00000100088	zmp:0000001114	0.870869671	2.75E-11	0.432084262	0.001269526
ENSDARG00000100113	AL935044.2	2.181080898	0.023870333	1.994598474	0.043488504
ENSDARG00000100157	tamm41	0.439488802	0.003365793	0.46220477	0.001154144
ENSDARG00000100160	si:dkey-205i10.2	5.325184279	0.01333562	4.71018982	0.04138024
ENSDARG00000100181	lamp1a	0.789091188	0.000304223	1.075264904	0.000000036
ENSDARG00000100184	ly97.2	7.904575993	5.74E-08	7.228363295	0.00000153
ENSDARG00000100185	elovl7b	0.441830489	0.01623172	0.637379527	0.000116768
ENSDARG00000100222	utrn	0.71093227	1.45E-10	0.456590192	0.0000234
ENSDARG00000100223	gpa33a	0.7210687	0.000000348	0.446890728	0.001728679
ENSDARG00000100240	CT737123.1	5.384699134	1.82E-08	4.500115274	0.00000577
ENSDARG00000100257	zgc:174314	-0.657630331	0.032251907	-0.629448341	0.036950803
ENSDARG00000100259	zgc:171759	3.353535772	0.005178046	3.335714537	0.00684139
ENSDARG00000100260	them6	1.175612345	3.24E-12	0.609915891	0.000427522
ENSDARG00000100274	WDR7	0.377680344	0.039773024	0.440653002	0.012020313
ENSDARG00000100293	arhgef15	-3.334073209	3.32E-11	-2.517648794	2.59E-09
ENSDARG00000100317	ahsa1b	0.303195095	0.028970624	0.378489308	0.003621739
ENSDARG00000100332	CABZ01084942.1	2.890865142	2.36E-08	1.589771612	0.004341155
ENSDARG00000100338	BX936384.1	4.574253037	0.001318435	4.610471015	0.001811382
ENSDARG00000100339	arf4b	3.451945155	5.26E-23	2.302877544	9.31E-12
ENSDARG00000100352	aglb	-0.566637597	0.000219301	-1.003011079	4.41E-14
ENSDARG00000100359	AREL1	-0.582157175	0.00000181	-0.369384483	0.002430198
ENSDARG00000100374	txnrd3	0.473075261	0.000000159	0.25576122	0.006180114
ENSDARG00000100376	amdhd2	0.636522074	0.007489939	0.761398319	0.000582838
ENSDARG00000100386	si:dkey-61p9.9	-1.696872768	0.026883418	-1.741478271	0.018849479
ENSDARG00000100396	pip5k1aa	-0.421385507	0.017405517	-0.605659138	0.000158137
ENSDARG00000100405	BX957252.3	-1.81975924	0.002873491	-2.894596758	0.000000452
ENSDARG00000100426	fam167b	-0.77220121	0.000199116	-1.168879843	2.38E-10
ENSDARG00000100442	cfh	1.175857932	7.01E-21	0.468745033	0.000260531
ENSDARG00000100449	BX322631.1	2.100185305	0.000000964	2.423325834	6.93E-10
ENSDARG00000100452	nop53	-0.406261041	0.005994658	-0.314959684	0.03720392
ENSDARG00000100464	si:dkey-193i10.1	1.977848933	0.010879054	1.695246208	0.036489613
ENSDARG00000100476	si:dkeyp-110c7.4	-1.914053219	4.29E-10	-0.85349521	0.00488362
ENSDARG00000100487	nploc4	-0.314953193	0.025940218	-0.430034209	0.000920835
ENSDARG00000100513	rps27l	2.573696072	4.16E-55	1.047636439	5.01E-11
ENSDARG00000100524	abca3b	0.403220891	0.00109137	0.315386286	0.010841822
ENSDARG00000100540	CABZ01114105.1	-0.931731865	0.0000317	-0.672369181	0.00205859
ENSDARG00000100552	taco1	0.981185872	0.000142814	0.882462315	0.000381093
ENSDARG00000100555	aqp11	1.15869862	1.18E-16	0.913417248	2.19E-12
ENSDARG00000100562	CABZ01080702.1	0.635823009	0.000288835	0.508759269	0.003416455
ENSDARG00000100578	si:dkeyp-90h9.1	3.235292266	0.000000598	2.270740267	0.00059948
ENSDARG00000100584	ccdc40	1.4367348	4.93E-10	0.804328661	0.000647603
ENSDARG00000100613	CABZ01089777.1	-2.78588746	2.12E-18	-1.178557994	0.0000823
ENSDARG00000100614	zgc:171497	-1.165590545	0.0000177	-1.034886383	0.0000418
ENSDARG00000100625	si:ch73-197b13.1	-1.860070772	4.14E-15	-1.742958908	1.57E-16
ENSDARG00000100651	PDCL3	0.593963125	0.00000559	0.361857698	0.007155655
ENSDARG00000100652	znf1147	-2.426376418	0.007672573	-1.789461153	0.040219106
ENSDARG00000100654	si:dkey-207m2.4	-2.657737083	3.16E-16	-1.370398619	0.000000915
ENSDARG00000100660	si:ch73-252i11.1	1.682468755	3.67E-13	0.570119548	0.033986403
ENSDARG00000100673	znf974	-1.432333479	0.035019106	-1.375272294	0.037658437
ENSDARG00000100690	si:ch211-256e16.11	2.937309717	2.91E-26	2.74881232	7.69E-26
ENSDARG00000100708	jakmip3	-1.152541879	4.18E-15	-1.251148046	1.2E-21
ENSDARG00000100709	agxt2	2.308921364	2.9E-18	1.997774077	2.31E-16
ENSDARG00000100712	si:dkey-19b23.12	-0.368534615	0.02282196	-0.438070698	0.004126231
ENSDARG00000100729	tln1	0.7118548	6.21E-10	0.298202931	0.01551096

Table S3. DEGs of WT vs. *terfa+/-* ∩ *terfa-/-*

ENSDARG00000100739	CABZ01052573.1	0.948276851	1.63E-11	0.807513654	6.82E-10
ENSDARG00000100743	si:dkey-190j3.4	4.329915665	0.000000333	3.373981301	0.000170033
ENSDARG00000100781	col28a2b	-0.51562497	0.0131174	-0.436623833	0.03710322
ENSDARG00000100797	CABZ01076996.1	1.021627886	1.01E-09	0.463406334	0.00950709
ENSDARG00000100826	hif1al	0.75463106	7.03E-16	0.405874857	0.00000995
ENSDARG00000100850	rnf103	-0.338491914	0.021087009	-0.363600738	0.009863584
ENSDARG00000100865	si:dkey-269o24.1	-1.50919272	0.000000806	-1.669767256	1.72E-09
ENSDARG00000100869	adcy9	0.800756681	1.79E-08	0.315467229	0.045899097
ENSDARG00000100887	si:dkey-11f4.20	3.047817795	4.83E-09	1.426396943	0.017479827
ENSDARG00000100954	wars1	0.69974301	0.000214742	0.597268573	0.001214183
ENSDARG00000100986	CABZ01117503.1	-2.552624167	7.9E-10	-1.690612655	0.00000643
ENSDARG00000101008	soul4	-0.459000714	0.005264262	-0.339756338	0.044956724
ENSDARG00000101029	si:ch73-347e22.4	-0.43776659	0.000965041	-0.318849271	0.017204485
ENSDARG00000101047	rtcb	0.606729149	0.00000625	0.406054064	0.002759938
ENSDARG00000101048	FQ790208.1	-1.981751235	0.013972684	-2.922931899	0.00022855
ENSDARG00000101060	gcna	-1.155612675	0.000000472	-0.784318642	0.000318442
ENSDARG00000101071	si:dkeyp-80d11.14	3.919856497	5.03E-22	2.668210048	1.03E-10
ENSDARG00000101072	cnot6b	0.370873874	0.032028831	0.405994019	0.015083115
ENSDARG00000101084	CABZ01069184.1	2.815084972	1.76E-11	1.738784064	0.0000455
ENSDARG00000101085	ss18l2	-0.984763918	0.000190223	-0.685240336	0.00919238
ENSDARG00000101088	si:ch73-359m17.5	1.329220707	0.016087512	1.268809036	0.020752634
ENSDARG00000101090	pappaa	1.943021117	2.48E-18	0.69460024	0.003763972
ENSDARG00000101094	ATP11B	0.546571378	0.000000589	0.272073659	0.019591937
ENSDARG00000101130	coq8b	-0.502481654	0.0000584	-0.504855501	0.0000138
ENSDARG00000101164	nansb	0.773043976	0.00000303	0.74435837	0.00000156
ENSDARG00000101181	s100w	0.936171393	8.09E-08	0.437453412	0.020707796
ENSDARG00000101199	rbp4	-0.792174856	8.05E-12	-0.682451805	1.79E-10
ENSDARG00000101201	si:dkey-207b20.2	7.766278966	0.000000202	6.606355982	0.0000249
ENSDARG00000101209	lamb1a	0.620879678	3.34E-10	0.218814869	0.046707885
ENSDARG00000101210	glg1b	0.728133936	0.0000663	0.654621601	0.000179414
ENSDARG00000101245	znf1063	2.596446001	0.000463922	2.051165007	0.008678432
ENSDARG00000101259	hgh1	-0.466647192	0.037849874	-0.474747555	0.031265798
ENSDARG00000101300	ssna1	-0.923861464	0.00024094	-0.822928929	0.000529182
ENSDARG00000101308	smug1	-0.781653237	0.000108275	-0.611255825	0.001872156
ENSDARG00000101316	ppp4r1	0.529125517	0.00000676	0.450686159	0.0000607
ENSDARG00000101334	ctsc	0.863612551	0.00000491	0.566799197	0.002992508
ENSDARG00000101369	CABZ01090890.1	0.512608898	0.000281641	0.727984859	6.04E-09
ENSDARG00000101374	si:dkey-11n14.1	-1.005865559	0.009119689	-1.041025983	0.004862189
ENSDARG00000101388	si:ch211-171l17.14	3.994692629	6.09E-13	2.709063894	0.00000233
ENSDARG00000101406	rplp2	0.752510695	5.92E-11	0.611952694	1.23E-08
ENSDARG00000101407	tgm1l4	2.626481096	8.87E-17	2.173771596	4.67E-13
ENSDARG00000101421	si:dkeyp-80d11.10	6.094696127	0.00000527	4.207512882	0.005041719
ENSDARG00000101438	lonp2	0.894188391	7.99E-13	0.664258019	1.92E-08
ENSDARG00000101459	si:dkey-279j5.1	2.24370815	0.000047	1.783151788	0.001307755
ENSDARG00000101482	hk2	0.954785174	2.63E-16	0.550960233	0.00000145
ENSDARG00000101495	ugt5b2	-0.88670525	0.00000398	-0.926901543	0.00000013
ENSDARG00000101514	ftr64	-2.344214556	0.00000282	-1.498048923	0.001016317
ENSDARG00000101528	si:ch211-194h1.2	-1.417089831	0.033525268	-1.734864183	0.006290989
ENSDARG00000101534	rab3db	0.853316606	0.002739302	0.751042428	0.008063016
ENSDARG00000101562	znf1014	-0.773487279	0.0000018	-0.43936634	0.007298926
ENSDARG00000101567	guca1e	-2.471981596	0.00000273	-1.250785509	0.011918138
ENSDARG00000101602	preb	0.539472373	0.000000964	0.413322902	0.000110227
ENSDARG00000101609	si:dkey-109a10.2	4.701619991	0.010939555	4.555651578	0.019807173
ENSDARG00000101618	ube2c	-0.628070817	0.001176363	-0.401409428	0.045209085
ENSDARG00000101621	CABZ01040054.1	-1.533538416	0.010045534	-1.88099798	0.000711126

Table S3. DEGs of WT vs. *terfa+/-* ∩ *terfa-/-*

ENSDARG00000101627	ssr1	0.455593549	0.00000186	0.27510295	0.004851603
ENSDARG00000101634	si:ch211-209j12.1	4.015775837	1.25E-13	2.915223056	9.02E-08
ENSDARG00000101645	myrfl	1.074614646	0.000000079	0.459351732	0.037850212
ENSDARG00000101687	CU929259.1	0.60364753	0.006530458	0.663641405	0.001593825
ENSDARG00000101725	parietopsin	0.740177063	0.005425759	0.634218846	0.017747482
ENSDARG00000101728	si:dkeyp-80d11.12	3.819985872	0.001550053	3.150576759	0.015229154
ENSDARG00000101736	CABZ01113816.1	5.895061815	2.04E-08	4.894245173	0.00000851
ENSDARG00000101748	NA	-6.846884777	0.000572335	-6.857821146	0.000089
ENSDARG00000101749	zgc:92161	-0.822826184	0.045323273	-1.233852807	0.001090176
ENSDARG00000101754	BX664622.1	3.042353348	6.57E-13	1.929181371	0.00000566
ENSDARG00000101756	im:7141269	1.044551616	0.000706827	1.030232172	0.000472213
ENSDARG00000101757	si:dkey-27n6.4	4.589119121	2.99E-23	2.828696078	4.52E-09
ENSDARG00000101762	fah	-0.387731234	0.01336201	-0.852437546	1.58E-10
ENSDARG00000101790	si:ch73-299h12.3	-0.663283629	0.009172883	-0.604651927	0.015124596
ENSDARG00000101794	atp6v0e1	0.47103457	0.005541042	0.412752328	0.014689796
ENSDARG00000101817	si:dkey-5i16.5	-0.925744818	0.009506827	-1.091834743	0.001081825
ENSDARG00000101826	zwi	-1.57697715	1.19E-12	-0.803316238	0.000220122
ENSDARG00000101828	si:dkey-16p6.1	4.891147168	5.76E-20	3.817706274	7.37E-13
ENSDARG00000101859	mnta	0.31878179	0.036552883	0.470011304	0.000647603
ENSDARG00000101892	ino80e	1.513653001	7.94E-30	0.817746051	1.85E-10
ENSDARG00000101900	xrn2	-0.542733272	0.00000069	-0.33782293	0.002085734
ENSDARG00000101913	CABZ01092156.1	0.798915518	0.0000996	0.485289554	0.024903773
ENSDARG00000101919	foxj1a	2.409015192	1.76E-29	0.840233579	0.000187751
ENSDARG00000101924	si:dkey-65b13.13	-2.180989379	1.51E-12	-0.817681859	0.003502335
ENSDARG00000101930	CU914622.1	-2.784052624	0.044669853	-4.24613997	0.001021065
ENSDARG00000101948	si:ch211-223g7.6	-0.958632911	0.008176556	-1.142004637	0.000735118
ENSDARG00000101954	hoxc6b	-1.22599566	0.000000258	-1.16208234	9.08E-08
ENSDARG00000101977	si:ch211-209n20.59	4.022601471	0.001834399	2.872593754	0.045716473
ENSDARG00000101992	si:dkey-238k10.3	-2.759913556	6.65E-11	-1.139573085	0.000935695
ENSDARG00000102020	pnpla3	0.621678855	0.024741337	0.750277117	0.004030834
ENSDARG00000102058	si:dkeyp-11e3.1	1.124062525	1.59E-12	0.487156789	0.004218904
ENSDARG00000102091	CABZ01052576.1	0.582328008	0.019632173	0.542698498	0.029136829
ENSDARG00000102094	NA	-4.108101659	0.013028231	-5.646687202	0.001951737
ENSDARG00000102095	si:ch73-233f7.5	-0.764420411	0.010074671	-0.609876096	0.042419355
ENSDARG00000102105	dad1	0.479165663	0.000162066	0.440828385	0.000287687
ENSDARG00000102114	ptdss1b	1.165208912	0.004608873	0.862725883	0.045434954
ENSDARG00000102116	CABZ01072487.2	2.153370101	0.000918501	2.407870293	0.0000969
ENSDARG00000102127	NHLRC3	0.324382028	0.048597564	0.390075809	0.012600252
ENSDARG00000102128	eps8a	-0.729334199	0.000000373	-0.438729499	0.002316071
ENSDARG00000102129	crygn1	-1.539219209	0.000333083	-1.584561385	0.0000744
ENSDARG00000102138	foxa1	0.356694034	0.049362443	0.419894586	0.015479401
ENSDARG00000102162	BX005043.1	3.475373549	0.012844714	3.304917234	0.024647099
ENSDARG00000102176	pla1a	1.354711436	0.000136894	0.848022408	0.024580818
ENSDARG00000102191	CPNE8	-0.497219214	0.003600977	-0.524846332	0.001170501
ENSDARG00000102200	man2a1	0.779033229	0.000137414	0.609407949	0.002759584
ENSDARG00000102207	zgc:86738	1.33945442	8.91E-15	0.593671443	0.000966934
ENSDARG00000102225	si:ch211-205a14.7	3.571505103	0.00000872	2.454692657	0.004873065
ENSDARG00000102227	CR450749.3	3.646420243	0.000000478	3.012670488	0.0000332
ENSDARG00000102249	pepd	0.897673943	2.27E-11	0.580488358	0.0000089
ENSDARG00000102252	USP34	0.539591248	0.000462552	0.587160536	0.0000415
ENSDARG00000102257	soul3	0.261134677	0.031404878	0.391421882	0.000329155
ENSDARG00000102261	RSPH1	0.95592191	0.0000101	0.579858943	0.010663729
ENSDARG00000102288	CABZ01056321.1	-3.149265413	1.67E-43	-0.859240354	0.0000127
ENSDARG00000102296	zgc:175177	-1.385099209	0.047385701	-2.271296878	0.000666849
ENSDARG00000102307	taf2	-0.497128379	0.000100304	-0.304406957	0.02131872

Table S3. DEGs of WT vs. *terfa+/-* ∩ *terfa-/-*

ENSDARG00000102318	il6	5.683350277	0.006703821	4.740348393	0.037621027
ENSDARG00000102321	si:ch73-299h12.1	-0.913389187	0.0000636	-0.547677346	0.016481138
ENSDARG00000102364	si:dkey-202l22.6	1.694982364	5.45E-24	1.093213335	3.91E-12
ENSDARG00000102395	HAPLN1	6.369879828	2.35E-38	5.892186294	7.4E-35
ENSDARG00000102403	cry2	0.738015239	4.62E-08	0.854812823	1.87E-12
ENSDARG00000102415	scinla	-0.82784884	0.000000176	-0.63011967	0.0000347
ENSDARG00000102421	acp7	0.916898484	0.000572335	0.915278041	0.000293929
ENSDARG00000102440	CABZ01070852.1	2.465302917	1.33E-20	1.554378225	1.07E-09
ENSDARG00000102441	ctnna1	0.656853902	1.16E-13	0.268865373	0.003896058
ENSDARG00000102445	si:dkey-22o22.2	0.533805972	0.011051391	0.568144235	0.00481132
ENSDARG00000102463	cox18	0.525036291	0.003341218	0.418153565	0.02131891
ENSDARG00000102473	zgc:171673	-2.245446569	4.3E-17	-1.191654422	0.000000698
ENSDARG00000102506	lrp2a	0.467905612	0.005213234	0.46332609	0.004226859
ENSDARG00000102528	AL935126.1	2.607451958	0.0000116	2.430417166	0.0000232
ENSDARG00000102550	CABZ01080371.1	-0.75819457	0.00000186	-0.36382141	0.030453596
ENSDARG00000102556	nfat5b	-0.527528224	0.049133549	-0.566238247	0.030309499
ENSDARG00000102599	si:ch1073-392o20.2	1.277649571	1.7E-11	0.621891861	0.001467356
ENSDARG00000102606	znf1158	-0.778582196	0.008928481	-0.652545112	0.02695425
ENSDARG00000102616	si:dkey-3h2.3	3.412617063	0.000169023	2.566646999	0.007554724
ENSDARG00000102618	utp18	0.875007714	0.001158454	0.984034712	0.0000831
ENSDARG00000102626	frem2b	1.107475974	1.45E-16	0.56615203	0.0000196
ENSDARG00000102640	pdia3	0.362456576	0.000317062	0.347052508	0.000292052
ENSDARG00000102673	si:dkey-26i24.1	2.017352116	0.001409125	2.046963275	0.000861384
ENSDARG00000102690	rims2b	-1.229184706	6.82E-12	-0.406145271	0.038611334
ENSDARG00000102713	si:ch211-205a14.1	5.998738564	8E-36	4.758246652	6.41E-23
ENSDARG00000102733	EMC4	0.978512997	0.008454514	1.288522623	0.000140515
ENSDARG00000102744	mgst3a	1.039314726	1.67E-11	0.659082769	0.0000122
ENSDARG00000102746	tmem39a	0.592527053	0.000402562	0.486116209	0.003291798
ENSDARG00000102753	si:dkey-109l4.3	-1.441403117	6.37E-08	-0.823009182	0.001328755
ENSDARG00000102759	TMEM132D	-1.614892096	1.63E-13	-0.483877579	0.03324906
ENSDARG00000102765	lonp1	0.574655018	1.32E-08	0.500480499	0.000000121
ENSDARG00000102776	cxcl19	3.961180954	0.04727324	4.651980465	0.020762042
ENSDARG00000102835	CABZ01021435.1	2.388885043	0.0000192	1.358745511	0.028794367
ENSDARG00000102855	neo1a	0.259345948	0.029686474	0.310440866	0.005939307
ENSDARG00000102859	CABZ01092170.1	0.912251941	0.0000427	0.744308341	0.000665941
ENSDARG00000102899	cremb	2.044701284	0.0000116	1.087984309	0.037695949
ENSDARG00000102904	si:ch211-246b8.2	2.469911499	0.000114421	1.845379527	0.006237235
ENSDARG00000102917	CR388095.1	-2.483352278	0.00000843	-2.089771861	0.0000379
ENSDARG00000102953	ints4	-0.48724723	0.000098	-0.355635442	0.004123102
ENSDARG00000102967	CT573282.1	-3.369618918	4.08E-20	-1.091419234	0.000518943
ENSDARG00000102995	rbm24a	-0.324078149	0.010493895	-0.287322586	0.022486212
ENSDARG00000103001	si:ch73-215d9.1	0.538874557	0.013039446	0.749292036	0.00013452
ENSDARG00000103002	nptna	-0.576830972	0.00000735	-0.332759606	0.012169461
ENSDARG00000103006	si:ch1073-303d10.1	-3.142095597	1.35E-10	-1.020194989	0.02731965
ENSDARG00000103019	gstp2	1.20273827	2.61E-14	0.969417463	4.99E-11
ENSDARG00000103026	p3h2	-0.394891727	0.007973989	-0.493210451	0.000325564
ENSDARG00000103038	pik3r3a	0.841593842	4.38E-12	0.503744116	0.0000249
ENSDARG00000103101	si:ch211-235e9.6	0.625118005	0.0000735	0.442807239	0.005477706
ENSDARG00000103103	BX957361.2	-1.789480609	0.0000911	-1.505070024	0.000356121
ENSDARG00000103106	trappc9	0.756625673	0.002596089	0.974191714	0.0000183
ENSDARG00000103119	si:dkeyp-33c10.7	3.108630391	0.000000386	1.73338016	0.009522893
ENSDARG00000103125	tsc2	0.551046646	0.00000713	0.437232169	0.000239456
ENSDARG00000103133	hoxc12b	-1.364101681	0.00000109	-0.792063658	0.003264717
ENSDARG00000103163	tomm20a	-0.655379676	0.000261927	-0.690423285	0.0000366
ENSDARG00000103167	si:dkey-245n4.2	-0.676017711	0.00054399	-0.639900394	0.000529182

Table S3. DEGs of WT vs. *terfa+/-* ∩ *terfa-/-*

ENSDARG00000103186	si:ch73-389k6.1	-2.820748057	9.64E-13	-1.546746503	0.0000349
ENSDARG00000103194	BX936433.2	-1.224018275	0.029879665	-1.83591994	0.000609429
ENSDARG00000103199	si:dkey-247k7.2	1.81756106	4.62E-26	0.698106505	0.0000797
ENSDARG00000103205	si:ch211-161f7.2	3.588585514	0.029163059	3.749502679	0.029083901
ENSDARG00000103220	slc10a3	0.745165271	2.68E-11	0.373593461	0.001145411
ENSDARG00000103230	CABZ01079302.1	1.740304904	0.009705102	1.613471406	0.018106743
ENSDARG00000103239	si:dkey-14o6.8	-1.736782215	0.003110394	-1.273827092	0.025448931
ENSDARG00000103254	si:dkeyp-80d11.4	2.758467826	0.006731068	3.038499079	0.002316071
ENSDARG00000103259	opn4xb	-1.66668449	0.00000111	-0.817771799	0.019384361
ENSDARG00000103277	cyp24a1	3.105154582	3.99E-11	1.845447447	0.0000707
ENSDARG00000103306	BX927130.4	3.889139503	3.59E-21	3.086433595	4.04E-15
ENSDARG00000103309	BX664625.5	5.118143268	0.0000174	4.3958847	0.000430159
ENSDARG00000103320	lclat1	0.506923694	0.001342782	0.367973545	0.023028719
ENSDARG00000103324	si:ch211-127b6.2	5.436527008	0.000000151	4.921086646	0.00000258
ENSDARG00000103351	FP102192.2	5.009783601	0.003672491	4.767002229	0.007781833
ENSDARG00000103369	taldo1	0.494535057	0.0000228	0.3643363	0.00163839
ENSDARG00000103413	zgc:109949	-2.07747735	7.06E-38	-0.677916528	0.00000809
ENSDARG00000103441	znf1060	2.091552359	0.00000636	2.143636698	0.000000885
ENSDARG00000103454	trpm6	1.123444341	2.13E-14	0.407931915	0.011203072
ENSDARG00000103456	cyp2aa11	2.323928019	0.0000029	1.985200881	0.0000465
ENSDARG00000103473	BX537137.3	2.212600657	0.031623851	2.228109522	0.034478001
ENSDARG00000103486	CT573799.1	-3.827983471	0.0000714	-7.392522909	0.00000147
ENSDARG00000103505	fbxl3a	-0.73926703	0.000000166	-0.471659105	0.000688156
ENSDARG00000103515	vcana	1.794151264	2.58E-13	0.837070563	0.001435028
ENSDARG00000103516	sec24c	0.300938002	0.008312081	0.44628119	0.0000115
ENSDARG00000103537	ttll12	0.666853579	0.01501633	0.733421019	0.005165632
ENSDARG00000103597	si:dkey-40j3.3	-1.793079338	0.008688998	-1.375196504	0.040883984
ENSDARG00000103606	ndst1b	-0.350937691	0.000826147	-0.236270669	0.028973884
ENSDARG00000103612	akt2l	0.785320506	0.00000148	0.35413488	0.047194522
ENSDARG00000103614	pemt	1.172896873	7.25E-08	0.931256585	0.0000103
ENSDARG00000103618	btr18	-1.979870856	0.0000807	-1.332638661	0.005583045
ENSDARG00000103650	si:ch73-329n5.1	1.51573933	0.033948782	2.267857725	0.000404398
ENSDARG00000103684	ap4e1	-0.642249768	0.000085	-0.377599722	0.025029991
ENSDARG00000103687	sycn.2	-2.058467616	0.013345274	-2.282959363	0.003980086
ENSDARG00000103725	znf341	0.441989813	0.013924888	0.609320609	0.000178139
ENSDARG00000103735	elovl1b	0.597885778	4.57E-10	0.321600996	0.000981134
ENSDARG00000103736	gramd2aa	-0.771115786	0.000683872	-0.482697906	0.038240278
ENSDARG00000103740	fundc2	-0.502089244	0.000437938	-0.427357708	0.002110365
ENSDARG00000103744	hacd1	0.995930114	3.61E-17	0.327416368	0.010999721
ENSDARG00000103754	aspm	-1.566535301	3.24E-12	-0.649583035	0.005882011
ENSDARG00000103760	cfhl2	2.288493126	6.37E-46	1.525251606	1.82E-24
ENSDARG00000103763	l3hypdh	0.724236014	0.023365815	0.738308404	0.018317671
ENSDARG00000103775	thbs1a	1.968198259	4.58E-23	0.472265339	0.037120832
ENSDARG00000103788	AL645792.2	5.77024014	9.22E-18	3.950172778	1.32E-08
ENSDARG00000103795	zgc:113348	-0.582902955	0.001682238	-0.403172072	0.033651582
ENSDARG00000103814	CR450808.4	2.431077122	0.001907385	2.894423455	0.0000799
ENSDARG00000103830	ddr2l	1.211674914	5.73E-11	0.528107611	0.007020414
ENSDARG00000103837	CU633479.2	1.990163477	1.18E-11	1.191324327	0.0000526
ENSDARG00000103844	si:ch211-266c8.1	-2.286330751	8.65E-28	-1.121140929	8.67E-10
ENSDARG00000103846	hspa5	1.004781671	1.1E-23	0.899882409	3.39E-23
ENSDARG00000103860	wu:fu71h07	-0.994306434	0.000657073	-0.668662034	0.020743647
ENSDARG00000103914	THEM6	-0.600669701	0.005282252	-0.468180085	0.030833766
ENSDARG00000103917	znf185	0.811865271	8.58E-12	0.431080945	0.000315961
ENSDARG00000103919	si:ch73-1a9.3	-1.485971626	2.36E-25	-0.45438736	0.002700569
ENSDARG00000103935	rab27a	0.878867275	0.000000626	0.452515878	0.015476579

Table S3. DEGs of WT vs. *terfa+/-* ∩ *terfa-/-*

ENSDARG00000103947	zgc:113295	0.860918335	0.005952615	1.225930379	0.0000113
ENSDARG00000103960	CU019624.1	-2.936268092	0.003882666	-2.764234776	0.003013444
ENSDARG00000103979	calr3a	0.298298165	0.007140324	0.266696177	0.015376802
ENSDARG00000103981	bhlha9	1.478460506	0.000000192	0.949989048	0.000882406
ENSDARG00000103996	spdl1	-1.018120543	2.97E-08	-0.475137247	0.011705505
ENSDARG00000104023	mag	-1.195981263	0.000000342	-0.545723171	0.02863247
ENSDARG00000104040	CABZ01046088.1	1.509099198	0.0000227	1.281420868	0.000273495
ENSDARG00000104047	si:ch211-262i1.3	-0.807516083	0.000069	-1.139684661	3.24E-10
ENSDARG00000104049	crebl2	-0.363916168	0.008294674	-0.429568168	0.000836877
ENSDARG00000104071	hikeshi	0.556663228	0.0000267	0.346554564	0.011669606
ENSDARG00000104082	trps1	0.459837123	0.008409577	0.439069727	0.010177339
ENSDARG00000104089	p3h4	0.753269553	0.000111334	0.431439263	0.0382048
ENSDARG00000104099	FP236513.1	0.972460962	0.00000766	0.791411297	0.000186435
ENSDARG00000104108	slco1d1	0.491517658	0.005502382	0.383046698	0.034402701
ENSDARG00000104125	ttc6	1.917430307	7.75E-14	1.489776165	9.81E-10
ENSDARG00000104129	si:dkey-23n7.10	1.468411188	0.000031	1.200911646	0.000533714
ENSDARG00000104139	atp1a3b	0.435056996	0.008361975	0.385003926	0.019322059
ENSDARG00000104166	si:ch211-232d10.1	-1.382862982	0.000156764	-1.123103038	0.001290251
ENSDARG00000104169	si:ch211-212k18.4	-0.293928997	0.001474091	-0.298659404	0.000652822
ENSDARG00000104197	ptpn22	1.027928637	0.00000117	0.545422193	0.015822846
ENSDARG00000104200	BX901923.2	1.170363747	6.48E-24	0.483338434	0.0000388
ENSDARG00000104204	slc1a8a	-3.859237522	2.34E-57	-0.612556533	0.002400476
ENSDARG00000104228	lypla1	1.734558421	8.85E-12	1.603355077	1.6E-11
ENSDARG00000104230	sec23a	0.651516788	2.31E-10	0.514481682	0.000000111
ENSDARG00000104231	opn9	-3.198637832	9.51E-20	-1.080969784	0.001156321
ENSDARG00000104244	twsg1a	1.152955287	6.57E-15	0.639918443	0.0000119
ENSDARG00000104246	exosc3	1.912990005	6.79E-08	1.898395808	7.02E-09
ENSDARG00000104266	si:dkey-29m1.2	3.020490028	3.03E-09	2.481348891	0.000000982
ENSDARG00000104280	bnip1b	-0.486032954	0.003057894	-0.340489337	0.04315243
ENSDARG00000104287	lto1	-0.716931829	0.004223655	-0.695729319	0.00368969
ENSDARG00000104301	zgc:193726	-1.675928277	0.000602666	-1.302642879	0.005823481
ENSDARG00000104307	hoxa1a	0.858777738	0.00582198	0.6963942	0.028621377
ENSDARG00000104329	si:ch73-281k2.5	1.767649087	1.87E-10	1.40761763	9.31E-08
ENSDARG00000104342	csnk1g1	0.285993197	0.007049867	0.224838918	0.038576995
ENSDARG00000104359	anxa1c	1.45592864	4.7E-16	0.997305903	4.36E-09
ENSDARG00000104375	sec24a	0.679462349	1.17E-14	0.246671634	0.009226816
ENSDARG00000104380	si:dkey-238k10.2	-2.661988311	8.09E-28	-0.614718558	0.01180492
ENSDARG00000104384	agbl1	0.881358321	0.00000766	0.432936048	0.043738518
ENSDARG00000104403	oscp1a	-3.080049286	4.22E-16	-1.169489624	0.0000639
ENSDARG00000104413	rogdi	-0.700333547	0.000000153	-0.329694661	0.019183947
ENSDARG00000104416	si:dkey-16p6.1	3.507311819	0.000000514	2.492334162	0.000760301
ENSDARG00000104418	BX248318.1	-1.266085202	0.00000177	-0.653476601	0.014971983
ENSDARG00000104435	si:ch73-217b7.1	-0.674716491	9.85E-08	-0.306969528	0.020457658
ENSDARG00000104457	si:dkeyp-72e1.7	-1.104482051	0.000000223	-0.498457945	0.023991923
ENSDARG00000104478	cap2	1.510469889	2.89E-17	0.817721054	0.00000308
ENSDARG00000104489	CR855389.2	3.894169893	0.001740931	3.002007465	0.019502393
ENSDARG00000104524	CR391991.4	5.021031629	1.61E-10	5.432053893	7.41E-13
ENSDARG00000104528	cldn10b	-1.034318259	0.006763101	-1.164257956	0.001171558
ENSDARG00000104537	cox7c	0.953958315	6.2E-15	0.703833523	8.49E-10
ENSDARG00000104540	cyp2aa8	0.823186157	0.00000186	0.504992817	0.004148992
ENSDARG00000104555	sh2d3cb	-1.391229806	2.76E-10	-0.769261609	0.000178139
ENSDARG00000104561	znf1081	1.795700975	0.0000195	1.128199412	0.011034077
ENSDARG00000104566	foxi1	1.85403157	9.06E-10	0.87117508	0.008628672
ENSDARG00000104574	pcsk6	0.998546805	1.77E-09	0.525670619	0.002273309
ENSDARG00000104613	znf1179	1.541574954	0.0000183	1.855482895	1.15E-08

Table S3. DEGs of WT vs. *terfa+/-* ∩ *terfa-/-*

ENSDARG00000104647	surf4	0.573848513	0.000000649	0.313480858	0.009148579
ENSDARG00000104672	CABZ01074397.1	1.915758409	7.27E-13	0.687449299	0.022333234
ENSDARG00000104678	CU467646.7	0.830322792	0.000000431	0.533709618	0.00132662
ENSDARG00000104687	slc16a9b	0.844140398	3.56E-12	0.764353731	7.99E-12
ENSDARG00000104696	apooa	0.650727011	8.06E-08	0.278477894	0.037482377
ENSDARG00000104697	si:dkey-57k17.1	1.958264298	0.0000352	1.495306792	0.001594424
ENSDARG00000104756	CDHR1	-1.626128558	1.38E-17	-0.518507068	0.007795256
ENSDARG00000104758	zgc:194981	0.89950062	0.000401692	0.594534633	0.027021128
ENSDARG00000104789	dele1	0.352186041	0.024196848	0.350612007	0.022560739
ENSDARG00000104791	CABZ01077956.1	1.592879364	0.016436104	1.99574949	0.001263072
ENSDARG00000104811	cyp2aa2	1.480910262	1.7E-10	1.218371604	4.17E-08
ENSDARG00000104832	CU914164.1	-3.242022483	0.0000138	-1.945822478	0.002074922
ENSDARG00000104834	ANO7	-0.88247397	0.003466851	-0.715500234	0.015899722
ENSDARG00000104850	si:ch211-215p11.1	-0.626951248	0.031984695	-0.849008463	0.001723792
ENSDARG00000104864	klhdc2	0.527896387	0.003120969	0.458940195	0.00944048
ENSDARG00000104874	fzd6	0.820440813	4.38E-11	0.42617767	0.000773739
ENSDARG00000104887	znf1105	-1.394542744	0.000139285	-0.96128277	0.006053722
ENSDARG00000104890	si:ch211-76m11.3	6.234076821	4.22E-57	5.603050487	4.17E-48
ENSDARG00000104894	si:ch73-138e16.2	-0.974767932	0.0000585	-0.578735911	0.01778103
ENSDARG00000104897	si:dkey-16p6.1	5.729980968	5.96E-17	4.660047336	2.2E-11
ENSDARG00000104906	mtr	0.776830362	8.32E-10	0.365112702	0.006054771
ENSDARG00000104919	si:ch211-153b23.3	1.49089271	5.61E-09	0.727977864	0.006823186
ENSDARG00000104937	PTPRD	-0.615555717	0.000616546	-0.455249588	0.012119971
ENSDARG00000104953	stt3a	0.479781927	0.0000071	0.404929306	0.000073
ENSDARG00000104956	si:dkey-165o8.2	-2.260226864	1.27E-11	-1.037618945	0.001326844
ENSDARG00000104960	si:ch211-11n16.3	-1.94457356	0.047335244	-2.686564638	0.004094161
ENSDARG00000104980	PPEF2	-3.832586998	1.99E-41	-1.058908547	0.00000179
ENSDARG00000105036	arl6ip4	-0.707540094	0.00000611	-0.425128227	0.00659031
ENSDARG00000105095	AL929028.8	4.978977325	1.71E-13	3.621741864	0.000000175
ENSDARG00000105122	BX323031.2	-1.423002099	0.01216452	-1.238579682	0.026694342
ENSDARG00000105137	zgc:173705	7.068969042	0.000000576	6.560203517	0.00000865
ENSDARG00000105159	hdac8	1.517748572	9.17E-21	1.167394164	8.95E-15
ENSDARG00000105197	CR318620.1	6.78312347	0.0000331	5.880082819	0.000661032
ENSDARG00000105214	agtpbp1	0.592023091	0.00000122	0.39188592	0.001389745
ENSDARG00000105255	zbtb4	-0.332225982	0.041497605	-0.624105841	0.0000108
ENSDARG00000105274	CABZ01074130.1	1.140664156	0.0000015	0.693764722	0.004067061
ENSDARG00000105279	si:ch211-108c17.2	1.352739667	2.47E-14	0.967387664	1.27E-08
ENSDARG00000105301	sox8a	-1.425772621	0.0000686	-0.806036568	0.025732559
ENSDARG00000105304	CR762475.4	-2.069302026	0.000404367	-1.67757937	0.002073543
ENSDARG00000105341	si:dkey-9l20.3	1.253340768	4.93E-13	1.1005781	5.15E-12
ENSDARG00000105392	si:ch73-22o18.1	-3.381128017	8.22E-20	-1.65114307	2.31E-08
ENSDARG00000105407	CU459186.6	5.144319507	0.00080792	6.772087409	0.00000655
ENSDARG00000105408	BX248082.2	1.140048991	0.000154512	0.745218356	0.018298874
ENSDARG00000105411	si:ch211-113d11.5	-2.680682201	0.000156921	-1.648417673	0.025519193
ENSDARG00000105424	si:rp71-46j2.7	0.899913086	0.0000564	0.679995459	0.002228212
ENSDARG00000105442	LOXL4	0.644063137	0.0000702	0.659800085	0.0000129
ENSDARG00000105445	CR769769.2	-0.916935363	5.27E-12	-0.900250835	5.89E-14
ENSDARG00000105450	si:ch211-63p21.1	-1.264552018	0.000000418	-1.035052769	0.0000112
ENSDARG00000105452	plxna1a	0.398657837	0.048858861	0.636340223	0.000434876
ENSDARG00000105456	FO834831.1	6.157238564	0.000134154	6.012815955	0.000305964
ENSDARG00000105470	si:dkey-23a13.2	2.274578903	0.0000744	1.43979508	0.019157662
ENSDARG00000105489	loc564660	4.906986183	0.000391468	3.282910952	0.03656457
ENSDARG00000105507	zgc:112234	-1.718512332	0.002371057	-1.421813528	0.008045521
ENSDARG00000105511	BX248521.2	1.480139041	0.004332754	1.566427035	0.001703681
ENSDARG00000105548	si:dkey-1h4.4	2.882680539	0.000295661	3.075547682	0.0000771

Table S3. DEGs of WT vs. *terfa+/-* ∩ *terfa-/-*

ENSDARG00000105551	si:cabz01101003.1	1.422116836	7.27E-12	0.490594831	0.039672221
ENSDARG00000105590	si:cabz01007794.1	1.435748993	4.31E-24	1.053132892	1.08E-15
ENSDARG00000105625	si:cabz01007812.1	1.204932646	0.000000136	0.533729808	0.035568728
ENSDARG00000105641	si:ch211-98n17.5	0.667679794	0.000013	0.526864623	0.000417868
ENSDARG00000105651	BX323060.3	0.730832404	0.00438744	0.894441344	0.00015177
ENSDARG00000105657	si:cabz01080528.1	1.187551265	5.87E-10	1.365171614	2.32E-15
ENSDARG00000105718	CABZ01068273.1	1.274084023	0.033611445	1.311667585	0.027277662
ENSDARG00000105781	zgc:171727	1.491387019	0.002871667	1.818682237	0.0000806
ENSDARG00000105806	CABZ01069020.1	-0.947119539	0.000877222	-0.629787644	0.027094731
ENSDARG00000106579	CABZ01060030.2	1.287278417	0.002427962	1.10559129	0.009226816
ENSDARG00000106630	LO018627.1	-0.646182585	0.024592677	-1.077031559	0.0000227
ENSDARG00000106808	CU856622.1	-1.259499554	0.000268701	-0.87970333	0.009152383
ENSDARG00000107511	lyn	-0.370521585	0.025910398	-0.34751518	0.035153544
ENSDARG00000108525	CR749167.2	4.545492495	0.008294674	4.995669821	0.002810348
ENSDARG00000109202	BX324216.3	-1.027414286	0.000907029	-0.696958794	0.025385969
ENSDARG00000109310	im:7150988	1.189834085	6.76E-12	0.793219041	0.00000216
ENSDARG00000109371	pdgfaa	1.821927075	1.39E-11	1.734494549	5.76E-12
ENSDARG00000109373	tmbim1b	1.2317997	0.0000227	0.64346968	0.044066369
ENSDARG00000109381	CU457778.2	2.933267888	0.007955837	2.506336223	0.032200385
ENSDARG00000109443	EML4	0.432824639	0.000902581	0.384928613	0.002451468
ENSDARG00000109510	lsm12b	0.520967378	0.0000018	0.442394187	0.0000202
ENSDARG00000109715	sncgb	-0.460682773	0.000300594	-0.328833835	0.010762027
ENSDARG00000109749	si:ch211-162i8.7	3.178783713	8.33E-11	2.082956697	0.0000337
ENSDARG00000109859	CABZ01072242.1	1.310861613	3.28E-09	0.598674875	0.012018654
ENSDARG00000109869	zgc:174719	2.538865573	0.003612957	2.020321431	0.029407747
ENSDARG00000110069	pdgfaa	-0.859748815	0.003913275	-0.685868576	0.021098416
ENSDARG00000110103	FO834799.1	0.641299923	0.008146364	0.836715844	0.000154096
ENSDARG00000110229	zgc:173702	2.544040725	0.00253698	1.882700184	0.037539682
ENSDARG00000110288	CT583625.5	3.608197616	2.42E-08	2.202979085	0.0015266
ENSDARG00000110357	txndc15	0.444740693	0.003182373	0.489647323	0.000535869
ENSDARG00000110510	ybey	0.715235356	0.003780266	0.719780467	0.002435973
ENSDARG00000110658	zgc:174696	-2.284103767	0.00000104	-1.129833163	0.010494988
ENSDARG00000110845	pimr107	6.148957432	0.000195959	6.278661943	0.000188705
ENSDARG00000110878	si:dkey-28k24.2	2.61380882	0.008939068	2.529236424	0.013184215
ENSDARG00000110967	FP102169.1	3.093311246	0.00000419	2.753362983	0.0000432
ENSDARG00000111014	LO018430.2	0.537731981	0.007755334	0.443065225	0.031265798
ENSDARG00000111240	dhrs13a.2	1.892767818	3.21E-29	1.563587527	2.64E-24
ENSDARG00000111249	CR394528.1	1.124509503	0.000093	1.238570809	0.00000327
ENSDARG00000111261	FO834850.1	0.858083804	9.84E-08	0.562960526	0.000492185
ENSDARG00000111309	AL935126.2	2.548829262	0.000344724	2.5764996	0.000168905
ENSDARG00000111326	BX323820.1	-2.2840848	5.83E-11	-1.009204396	0.002886384
ENSDARG00000111465	znf1104	2.726102064	2.08E-16	2.067633909	1.06E-10
ENSDARG00000111506	BX470259.1	-0.81265374	0.002078561	-0.59097198	0.026296041
ENSDARG00000111606	smyhc2	4.413462678	0.004358654	4.102524968	0.00848525
ENSDARG00000111788	BX511067.1	7.366434524	8.49E-08	8.094042979	5.04E-09
ENSDARG00000111843	BX000451.4	-0.663148702	0.036246195	-0.679634713	0.027970484
ENSDARG00000111882	si:ch211-194m7.4	5.230993335	0.006047919	5.220159679	0.008058127
ENSDARG00000111899	zgc:171435	1.979863465	9.81E-12	2.071629927	1.42E-14
ENSDARG00000112337	mindy4b	-0.515742475	0.039014784	-0.595492085	0.012481263
ENSDARG00000112439	CABZ01092982.2	0.801375073	0.001082881	0.644784356	0.008621605
ENSDARG00000112454	FO744833.2	1.3570823	0.0000183	0.894881981	0.006428275
ENSDARG00000112527	FP325130.1	1.039157671	1.71E-10	0.625951856	0.000112199
ENSDARG00000112575	CU467905.1	1.565916769	0.000184866	1.669312381	0.0000189
ENSDARG00000112632	si:ch211-106j21.6	-1.971265931	0.0000221	-3.320421597	6.51E-13
ENSDARG00000112670	smim20	-0.4705967	0.014661791	-0.50863267	0.00568685

Table S3. DEGs of WT vs. *terfa+/-* ∩ *terfa-/-*

ENSDARG00000112728	KLHL12	0.499520765	0.00641628	0.497185494	0.00515737
ENSDARG00000112928	CR450780.2	2.580291927	0.0000128	1.843866344	0.002959935
ENSDARG00000112931	ndufaf3	0.452388127	0.002133894	0.385040262	0.008357647
ENSDARG00000113016	CU207245.1	5.328921549	1.44E-10	4.84194852	4.97E-09
ENSDARG00000113045	CABZ01063543.1	-1.404453653	0.00000837	-0.900078564	0.002677801
ENSDARG00000113150	fthl31	5.719455118	0.000256737	5.933229224	0.000170391
ENSDARG00000113274	AL929536.3	-0.96734537	0.0000501	-0.775440673	0.000715714
ENSDARG00000113284	CABZ01090361.1	4.258754497	3.4E-20	5.32198265	4.84E-34
ENSDARG00000113393	golim4a	0.674499451	1.38E-09	0.500006185	0.00000275
ENSDARG00000113433	FAM126B	0.923782472	0.039942948	1.044193273	0.015982629
ENSDARG00000113660	si:ch211-196f19.1	3.088517598	2.31E-15	2.1469273	2.08E-08
ENSDARG00000113674	CABZ01058222.1	1.431684608	7.74E-12	0.946814674	0.00000424
ENSDARG00000113780	zgc:173615	2.013943554	0.000159449	1.742344571	0.001040677
ENSDARG00000113896	si:dkey-29p23.1	-2.80435194	8.14E-17	-3.277083293	9.71E-27
ENSDARG00000113899	zgc:77650	0.499647445	0.0000111	0.255175948	0.037470624
ENSDARG00000113932	LO018011.1	2.402897889	0.009344057	2.037394634	0.035778595
ENSDARG00000113941	CU182823.3	3.908967859	0.00070552	3.598793979	0.00271872
ENSDARG00000113971	NA	-9.085516217	3.29E-08	-9.096455369	3.26E-11
ENSDARG00000113977	fthl29	-0.805990141	0.007474442	-0.692105863	0.021371028
ENSDARG00000114022	WDR1	0.993726288	1.46E-17	0.64421047	5.38E-09
ENSDARG00000114031	smyhc3	2.081757765	1.51E-24	1.615208561	1.2E-17
ENSDARG00000114067	tspan17	0.697802438	0.001858669	0.48433576	0.038035536
ENSDARG00000114142	si:dkeyp-4f2.1	5.552547285	6.06E-77	4.327758396	6.78E-50
ENSDARG00000114184	si:busm1-105l16.2	2.309270645	0.03429969	2.385313708	0.030010305
ENSDARG00000114227	CABZ01075242.1	0.791327407	0.00081066	0.789427403	0.000452484
ENSDARG00000114472	ctxn3	1.641299673	0.000000333	1.717644162	7.62E-09
ENSDARG00000114508	tmem101	1.168376774	0.000000159	1.025416176	0.00000106
ENSDARG00000114516	CABZ01021592.1	0.819358145	4.96E-09	0.744967551	9.21E-09
ENSDARG00000114529	CU457778.9	1.748489843	4.55E-08	1.473608609	0.00000125
ENSDARG00000114730	CABZ01113192.1	-0.362102213	0.008035667	-0.284443164	0.041087794
ENSDARG00000114742	CABZ01072083.2	-0.799922978	0.000284032	-0.476890265	0.03516464
ENSDARG00000114818	CU633479.5	1.733493442	1.16E-14	1.637297325	2.34E-15
ENSDARG00000114883	CU655961.4	7.339391852	0.00000275	5.804241647	0.000571363
ENSDARG00000114991	tmem14cb	-0.744500659	0.03483924	-0.827111403	0.01470388
ENSDARG00000115199	dicp1.3-4	-3.217425396	0.0000174	-2.384818619	0.000292563
ENSDARG00000115405	hbbe1.2	0.837216506	0.0000204	0.845233708	0.00000371
ENSDARG00000115428	CABZ01101813.1	0.772511325	2.73E-08	0.773932086	1.31E-09
ENSDARG00000115504	CABZ01080056.1	1.254004314	0.000000224	1.13304151	0.00000076
ENSDARG00000115557	cox7b	0.903916183	2.04E-12	0.62676249	0.00000032
ENSDARG00000115869	ATP9A	0.528347644	0.007017784	0.498835151	0.009522893
ENSDARG00000116086	si:dkey-193i10.4	3.079592984	0.007082406	2.608861913	0.032115344
ENSDARG00000116367	si:dkey-51d8.1	3.794194551	0.000000867	2.321198636	0.007111808
ENSDARG00000116442	TENT5A	-0.687886595	0.001041221	-0.557395716	0.006440228
ENSDARG00000116524	zgc:103700	-2.406685455	0.004665862	-2.001484681	0.009564368
ENSDARG00000116617	spaca4l	0.670953304	8.63E-09	0.345167815	0.00419181
ENSDARG00000116625	znf1141	2.090108778	9.63E-11	1.530474435	0.00000102
ENSDARG00000116692	si:ch211-79g12.1	2.867213413	0.000673542	2.664548092	0.001650144
ENSDARG00000116713	si:ch73-265d7.2	2.378407772	2.18E-28	0.756056835	0.001047167
ENSDARG00000116727	CABZ01081294.2	-1.012322714	0.003263475	-0.754946338	0.030419417
ENSDARG00000116743	rnps1	-1.757807452	0.000000662	-0.940394795	0.006428275
ENSDARG00000116774	PCNP	6.968469797	0.00000108	8.144713664	9.72E-09
ENSDARG00000116806	CU693479.1	0.787082961	0.000000789	0.363250938	0.035704946
ENSDARG00000116835	park7	0.573196812	0.0000612	0.468290231	0.00078904
ENSDARG00000116978	CABZ01010830.1	-2.652083826	0.0000322	-1.901656231	0.000823389
ENSDARG00000117011	ewsr1b	-1.789340012	4.54E-10	-1.048625344	0.000178139

Table S3. DEGs of WT vs. *terfa+/-* ∩ *terfa-/-*

ENSDARG00000117109	CR626884.6	2.061743633	1.68E-10	0.894400422	0.009833673
ENSDARG00000117126	BX664603.1	-2.244023857	0.001052054	-1.367554246	0.030678315
ENSDARG00000117130	BX936439.1	-0.618416607	0.011616738	-0.836943058	0.000190739
ENSDARG00000117160	CABZ01034596.1	10.0147585	5.59E-15	9.497518337	4.45E-13
ENSDARG00000117211	CR936462.1	1.001589925	0.000280605	0.58940845	0.049181377
ENSDARG00000117216	LO017718.1	2.58119205	0.000016	1.579370715	0.010768078
ENSDARG00000117235	CR293531.4	3.773456663	0.049118144	6.551318156	0.000072
ENSDARG00000117241	CABZ01067657.1	2.521436424	0.000000151	1.582303838	0.001456629
ENSDARG00000117249	CABZ01079872.1	5.351490926	0.00149938	3.866087707	0.046865868
ENSDARG00000117259	BX957317.3	-2.55555663	0.005883566	-2.37485999	0.005491302
ENSDARG00000117260	CABZ01079870.1	4.552970639	1.23E-10	4.736715434	5.65E-12
ENSDARG00000117271	CR954298.1	7.028010995	0.0000101	7.377609357	0.00000392
ENSDARG00000117313	CABZ01005857.2	2.791605867	0.0000614	2.488820075	0.000344574
ENSDARG00000117321	CZQB01066256.1	-1.094034639	0.003067087	-0.94268638	0.009342671
ENSDARG00000117336	CU855948.1	-1.673273438	0.031076205	-1.873684358	0.01185086
ENSDARG00000117400	CU651668.1	0.581349292	0.001381153	0.396372759	0.037695949
ENSDARG00000117431	CABZ01065684.1	-0.487099533	0.027884977	-0.576035886	0.005988023
ENSDARG00000117445	CABZ01067151.2	-1.535235818	0.0000173	-0.772456985	0.033508052
ENSDARG00000117448	CABZ01072245.1	1.357009365	8.12E-13	0.820110512	0.0000102
ENSDARG00000117450	FO834828.2	1.663463249	9.67E-11	1.180116417	0.00000186
ENSDARG00000117474	CABZ01048956.1	3.146235677	0.00000979	2.652144216	0.000155437
ENSDARG00000117485	FO681323.1	1.386640642	6.57E-18	1.275505696	3.77E-18
ENSDARG00000117493	BX511218.1	-2.05862858	0.00000725	-2.066597522	0.000000679
ENSDARG00000117504	CABZ01072084.1	0.891739487	0.000000674	0.745132108	0.0000163
ENSDARG00000117506	NA	-5.25696362	0.01099486	-3.894376494	0.01153209
ENSDARG00000117527	CABZ01079873.1	7.301690219	0.000000237	7.538781034	0.000000156
ENSDARG00000117533	nadsyn1	0.852018701	0.001594305	0.775779984	0.003527508
ENSDARG00000117613	CABZ01030033.1	-1.328748611	0.034891402	-1.780109848	0.002822961
ENSDARG00000117614	CR550304.1	-2.281058856	3.4E-22	-0.781703046	0.000261797
ENSDARG00000117659	CABZ01079280.1	-1.682418824	0.00000149	-0.744788872	0.032645471
ENSDARG00000117704	NA	-3.651525479	0.007580901	-4.336947965	0.001526059
ENSDARG00000117707	CABZ01072532.1	1.546089197	0.001344246	1.186815424	0.015473433
ENSDARG00000117735	CR926130.1	1.19838234	0.013707529	1.093085764	0.024638489
ENSDARG00000117742	FO904898.6	6.154524871	0.002421202	4.694103032	0.036934808
ENSDARG00000117751	CABZ01112025.1	1.274974478	0.000164747	0.817360589	0.023380655
ENSDARG00000117755	CU467655.2	-3.355348982	4.37E-11	-1.742871753	0.000140515
ENSDARG00000117816	CABZ01078767.1	1.561689278	0.000792563	1.009069986	0.044345461
ENSDARG00000117818	CR926130.2	2.097526852	1.59E-12	1.471206378	0.000000241
